# Transcriptional Dynamics of Immortalized Human Mesenchymal Stem Cells during Transformation

**DOI:** 10.1371/journal.pone.0126562

**Published:** 2015-05-15

**Authors:** Masao Takeuchi, Atsunori Higashino, Kikuko Takeuchi, Yutaro Hori, Kazuko Koshiba-Takeuchi, Hatsune Makino, Yoko Monobe, Marina Kishida, Jun Adachi, Jun Takeuchi, Takeshi Tomonaga, Akihiro Umezawa, Yosuke Kameoka, Ken-ichi Akagi

**Affiliations:** 1 Section of Laboratory Equipment, National Institutes of Biomedical Innovation, Health and Nutrition, Ibaraki-shi, Osaka, Japan; 2 Center for Human Evolution Modeling Research, Primate Research Institute, Kyoto University, Kyoto, Japan; 3 Division of Cardiovascular Regeneration, Institute of Molecular and Cellular Biosciences, The University of Tokyo, Tokyo, Japan; 4 Department of Biological Science, Graduate School Science, The University of Tokyo, Tokyo, Japan; 5 Department of Reproductive Biology and Pathology National Research Institute for Child Health and Development, Tokyo, Japan; 6 Laboratory of Proteome Research, National Institutes of Biomedical Innovation, Health and Nutrition, Ibaraki-shi, Osaka, Japan; 7 PRESTO, Japan Science and Technology of Agency, Tokyo, Japan; 8 Central Institute, A-CLIP Institutes, Chiba-shi, Chiba, Japan; University of Southern California, UNITED STATES

## Abstract

Comprehensive analysis of alterations in gene expression along with neoplastic transformation in human cells provides valuable information about the molecular mechanisms underlying transformation. To further address these questions, we performed whole transcriptome analysis to the human mesenchymal stem cell line, UE6E7T-3, which was immortalized with *hTERT* and human papillomavirus type 16 E6/E7 genes, in association with progress of transformation in these cells. At early stages of culture, UE6E7T-3 cells preferentially lost one copy of chromosome 13, as previously described; in addition, tumor suppressor genes, DNA repair genes, and apoptosis-activating genes were overexpressed. After the loss of chromosome 13, additional aneuploidy and genetic alterations that drove progressive transformation, were observed. At this stage, the cell line expressed oncogenes as well as genes related to anti-apoptotic functions, cell-cycle progression, and chromosome instability (CIN); these pro-tumorigenic changes were concomitant with a decrease in tumor suppressor gene expression. At later stages after prolong culture, the cells exhibited chromosome translocations, acquired anchorage-independent growth and tumorigenicity in nude mice, (sarcoma) and exhibited increased expression of genes encoding growth factor and DNA repair genes, and decreased expression of adhesion genes. In particular, glypican-5 (GPC5), which encodes a cell-surface proteoglycan that might be a biomarker for sarcoma, was expressed at high levels in association with transformation. Patched (Ptc1), the cell surface receptor for hedgehog (Hh) signaling, was also significantly overexpressed and co-localized with GPC5. Knockdown of GPC5 expression decreased cell proliferation, suggesting that it plays a key role in growth in U3-DT cells (transformants derived from UE6E7T-3 cells) through the Hh signaling pathway. Thus, the UE6E7T-3 cell culture model is a useful tool for assessing the functional contribution of genes showed by expression profiling to the neoplastic transformation of human fibroblasts and human mesenchymal stem cells (hMSC).

## Introduction

Neoplastic transformation of human fibroblasts and epithelial cells is thought to result from the sequential acquisition of genetic and/or epigenetic alterations in specific genes [1]. Much progress has been made in identifying and characterizing the genetic elements required to transform normal human cells [2–10]. Collectively, the results of these studies suggest that the transformation of human cells *in vitro* depends upon functional alterations in four to six genes. These alterations include changes in genes involved in telomere maintenance (to extend replicative lifespan), disruption of tumor suppressor pathways, and activation of oncogenes [2–10]. For example, the transformation of normal human fibroblasts requires the co-expression of *MYC*, *RAS*, and h*TERT* together with the functional loss of the *RB*, *PTEN*, and *p53* tumor suppressor pathways. However, a review by Duesberg and colleagues suggests that aneuploidy, in which a cell contains an abnormal number of chromosomes, is the primary cause of, and driving force behind, tumorigenesis: they state that aneuploidy results in an imbalance of gene expression, leading to the initiating event that initiates the transformation of normal cells [11].

An alternative explanation for the role of aneuploidy in tumorigenesis comes from mouse models harboring modifications in mitotic checkpoint genes. Studies of these mice have indicated that those showing reduced expression of mitotic checkpoint components, such as Bub1, BubR1, CENP-E, and Mad2, display an increased aneuploidy. In some mice, (CENP-E heterozygous mice, for example), reduced levels of *CENP-E* are associated with an increase in spontaneous tumorigenesis [12]. However, mice deficient in several spindle checkpoint–proteins, including BubR1, Bub1, and Bub3, display significantly increased level of aneuploidy without any increase in spontaneous tumorigenesis [13–15]. This indicates that even though aneuploidy is common in most human tumors, it is a promoter, rather than an initiator of tumor formation [16].

In addition, it is important to consider that distinct cell types show considerable differences in their susceptibility to transformation. Chromosomal changes in, or transformation of, human fibroblasts and hMSCs during culture can be caused not only by the introduction of the six genetic elements mentioned above, but also by the introduction of hTERT alone [17,18]. In addition, some hMSCs transduced with the hTERT gene show transformed phenotypes [17], but some are resistant [2,7,8,19]. Similarly, human embryonic stem cells continue to accumulate genetic and chromosomal changes during culture; these changes are similar to those observed in tumors [20]. The connection between genetic alterations and aneuploidy, both of which may induce transformation, remains unknown. However, the similarity between the final phenotype shown by different cells (i.e., transformed) suggests that the similar transformation programs, or an overlapping set of cancer-related genes, are involved.

By contrast, it is less clear how such genetic elements or aneuploids alter subsequent gene expression patterns influencing the progress of neoplastic transformation. To identify these genes and/or programs, we undertook a comprehensive analysis of gene expression changes in an immortalized hMSC cell line, UE6E7T-3, and associated these changes with the phenotypic appearance, including chromosomal aberrations, during long-term culture.

Recently, we showed that UE6E7T-3 cells show the preferential loss of one copy of chromosome 13 upon prolonged culture, yielding cells with near-diploid aneuploidy [21,22]. However, at a population doubling level (PDL) of 110, near-diploid aneuploid cells do not display anchorage-independent growth. Here, we show that upon prolonged culture, these cells became near-triploid, began to proliferate at an increased rate, displayed anchorage-independent growth, and formed fibrosarcoma-like tumors in nude mice. Furthermore, we comprehensively examined the genetic and chromosomal alterations that might drive the transformation associated with these phenotypes and found an important factor, GPC5, which was overexpressed and contributed to cell proliferation at late stage.

## Materials and Methods

### Cell culture

The human mesenchymal stem cell line UE6E7T-3 (JCRB1136) was obtained from JCRB Cell Bank (Osaka, Japan), which entailed no ethical problems.

Imabayashi et al. isolated human mesenchymal cells (H4-3) from the bone marrow of a donor (91 years old, female) [23]. The UE6E7T-3 cell line, which was originally called ‘ThMSC3’, was immortalized from H4-3 cells using LXSN-E6E7 and LXSN-hTERT and deposited into JCRB Cell Bank (http://cellbank.nibio.go.jp/), which verified the quality of the cells. UE6E7T-3 cells were cultured in POWEREDBY10 medium (GP Biosciences Ltd., Yokohama, Japan, http://www.gpbio.jp/), as recommended by JCRB Cell Bank. POWEREDBY10 medium is widely available worldwide and the cells can also be cultured in DMEM containing 10% FBS. We confirmed that UE6E7T-3 cells also lost one of chromosome 13 in DMEM containing 10% FBS at near 100 PDL of culture period. Cells were seeded at a concentration of 5×10^3^ cells/ml and cultured for 6–10 days, as described previously [21]. The population doubling level (PDL) was calculated as previously reported using the following formula: PDL = log (cell output/input)/log2 [22]. At the start of cultivation of this study, the PDL of UE6E7T-3 was 60. Further cultures consist of Stage I (PDL 60–90), Stage II (PDL 91–150), Stage III (PDL 151–230), and Stage IV (PDL 231–295). These four samples were named U3-A, U3-B, U3-C, and U3-DT, respectively. U3-DT cells (JCRB1136.01) at Stage IV (PDL 260) were deposited into the JCRB bank; the quality and ethics of the cells were verified by the bank.

### Flow cytometry

Cells of Stage I (PDL 65 or 70), Stage II (PDL 92), Stage III (PDL 200) and Stage IV (PDL 252) were harvested with trypsin and suspended in 5% FBS-PBS at the concentrations of 1–2 x 10^6^ cells/ml. 100 μl of each cell suspension was mixed with 5 μl of antibody diluted to 20 fold with 5% FBS-PBS. After incubation at 4°C for 20 min, cell suspension was washed 2 times with 5% FBS-PBS and was suspended with 500 μl of 4% paraformaldehyde–PBS. Data acquisition of 10,000 cells was performed using FACSCanto (BD). Analysis was performed using FlowJo software (TOMY Digital Biology). Antibodies used in this test were as follows, APC anti-CD34 (555824, BD), FITC anti-CD44 (IM1219U, Immunotech), FITC anti-CD45 (F0861, DAKO), RPE anti-CD73 (550257, BD), FITC anti-CD90 (F7274, DAKO), and RPE anti-CD105 (A07414, Immunotech). APC IgG1 (X0968, DAKO), FITC IgG1 (X0927, DAKO), RPE IgG1 (X0928, DAKO), and RPE IgG3 (731609, Immunotech) were also used as negative control.

### Measurement of chromosome number and mFISH analysis

Measurement of chromosome number and mFISH analysis were performed as previously described [21]. Briefly, for mFISH analysis, metaphase chromosome spreads and the multicolor probe (mFISH-24Xcyte-kit, MetaSystems GmbH, Jena, Germany) were denatured with 0.07 N NaOH and hybridized at 37°C for 1–4 d. FISH images were captured and analyzed using a Zeiss Axio Imager microscope (Carl Zeiss Microimaging GmbH, Jena, Germany) and Isis mBAND/mFISH imaging software (MetaSystems GmbH).

### Anchorage-independent growth (agar colony assay)

Between 125 and 1×10^5^ cells were cultured in POWEREDBY10 medium containing 0.3% agarose (Invitrogen Co.) on 0.6% agarose for 21 days. Colonies of diameter >300 μm (including >100 cells) were counted [3].

### Tumorigenicity assay

Tumorigenicity experiments were performed by subcutaneous (s.c.) or intramuscular injection (i.m.) of 1×10^7^ U3-DT cells into BALB/cAJcl-nu/nu mice (Clea Japan, Inc., Tokyo, Japan) [24]. The protocols were approved by the Laboratory Animal Care and the Use Committee of the National Research Institute for Child and Health Development, Tokyo, Japan (approval numbers: 2003–002 and 2005–003). In the fourth week, the transplanted tissues were removed and embedded in paraffin blocks. Standard hematoxylin and eosin staining of paraffin-embedded tissue was performed for histological examination of tumorigenicity.

### cDNA synthesis and whole-transcriptome sequencing

After subconfluent culture, 5×10^6^–1×10^7^ UE6E7T-3 cells at PDL 80 (Stage I), PDL110 (Stage II), PDL219 (Stage III) and PDL270 (Stage IV) were harvested. Each cellular total RNA from the four samples was extracted using QIAGEN RNeasy Mini Kit (Qiagen K. K., Tokyo, Japan). RNA samples were stored at −80°C until use. Poly-A RNA was used to select mRNA using the SOLiD RiboMinus Kit (Life Technologies, Gaithersburg, MD, USA). Following rRNA depletion, poly-A RNA was used to synthesize cDNA using the SOLiD Whole-Transcriptome Analysis Kit (Life Technologies) [25]. DNA sequencing (longest 50bp-reads) was carried out using SOLiD 3 PLUS System (Life Technologies) as described previously [26].

### Mapping sequence data and RNA-Seq analysis

SOLiD sequence data were mapped on the NCBI/GenBank *Homo sapiens* genome sequence (hg19, http://hgdownload.cse.ucsc.edu/download.html#human). Quality control of these sequence data was performed using the CLC Genomics Workbench-v4.5 (CLC bio Japan, Inc. Tokyo, Japan). At least, 90% length and 80% similarity of the reference gene have been required in order to carry out the mapping of the sequenced reads to reference genome.

The Genomics Workbench yields gene expression values in units of “reads per kilobase of exon model per million mapped reads" (RPKM)

[27]. All sequence reads have been deposited to the DDBJ database of the National Institute of Genetics (http://www.ddbj.nig.ac.jp/), accession number DRA000533.

### Cluster analysis and pathway analysis

Alignment to the NCBI *H*. *sapiens* genome sequence reference assembly (hg19) detected 33,565 genes from the RefSeq transcripts (S1 Table). Of these, 18,123 genes were expressed in mRNA extracted from U3-A cells. The expression of these genes was normalized to the GAPDH expression in the same sample. We then filtered for fold changes of >2 or <0.5 in Relative Expression Value (REV; expression in U3-B,-C, or—DT divided by expression in U3-A), resulting in a set of 8,032 genes of U3-DT (S1A Fig). REV of GPC5 was the top value of all genes in U3DT cells. Of the top 10 REV, 6 genes (GPC5, CCDC3, COL4A5, ADCY8, CHRDL1, and PK1B) were also included in the top 10 REV of U3-C cells, but were not included in REV of U3-B cells. Genes with REV values between 0.5 and 2 were omitted from further analyses. Cluster and pathway analyses of the expression values of these 8,032 genes were performed using the Multi Experiment Viewer (MeV) software (http://www.tm4.org/mev) and IPA software (Ingenuity Systems, Inc., http//www.ingenuity.com/), respectively. “Diseases and Disorder” analysis was applied to REV of 8,032 genes in U3-DT cells by the IPA software and the most frequent result was “tumorigenesis”. Furthermore, even if 18,123 genes were analyzed without utilizing a cut-off (0.5 to 2.0 REV), the same results were obtained. A total of 1,732 genes were analyzed, including 162 tumor-related genes identified from the literature and 1,570 genes detected by the IPA software (S1B Fig and S2 Table). We mainly classified 1,732 genes to 12 groups in accordance with information in the Gene database of NCBI.

### qRT-PCR analysis using SYBR Premix EXTaq (Method A)

RNAs of U3-A (PDL 80, Stage I) or U3-DT cells (PDL 270, Stage IV) were extracted from 5 × 10^6^ cells by the standard Trizol method using Sepasol RNAI Super G (Nacalai Tesque). RNA was reverse transcribed by ReverTra Ace qPCR RT Master Mix (Toyobo CO. LTD) and the cDNA subjected to qRT-PCR in a real-time PCR instrument (Thermal Cycler Dice Real Time Single System; Takara) with SYBR Premix EXTaq (Takara). GAPDH was used as the internal control. Primers used in the experiments were shown in (S1 Method).

### siRNA treatment

U3-DT cells were seeded in 6-well tissue culture dishes (2 × 10^5^ cells per well) and cultured in DMEM containing 10% FBS. The following day, cells were transfected with 100 or 200 nM of Accell Human GPC5 siRNASMARTpool or a non-targeting control siRNA [Thermo Scientific] in Accell siRNA Delivery Media containing 0.1%-FBS [Thermo Scientific] [28].

### Cell proliferation assay

Cells were plated on 96-well tissue culture plates (2,000 cells per well) in 0.09 ml of DMEM containing 0.1% FBS. The cells were then used in CCK-8 assays (Dojindo Laboratories) according to the manufacturer’s instructions. Briefly, 10 μl of CCK-8 reagent was added to each well and incubated at 37°C for 60 min. Cell proliferation was calculated on the indicated days by measuring the absorbance at 450 nm. The assay was performed in quintuplicate wells and each assay was performed at least three times.

### Western blot analysis

siRNA-treated U3-DT cells were lysed in PTS buffer containing protease inhibitors and the protein concentration of each sample was adjusted to 10 μg/8 μl. Each sample was then analyzed by Western blotting with a rabbit MAb against anti-Glypican 5 (clone ab124886; Abcam) and HRP-linked anti-rabbit IgG-goat antibody (Cell Signaling Tech.) [29]. Colored marker (Protein MultiColor III) was purchased from BioDynamics Laboratory Inc.

### Cell-cycle analysis

siRNA-treated cells were suspended in 0.1% FBS containing DMEM and 5 × 10^3^ cells per well were cultured in a well of 98-wells plate for 5hr. Cells were treated with culture medium containing 20 μM 5-ethynyl-2’-deoxyuridine (EDU) for 30 min before they were harvested. The cells were then processed using the Click-iT plus EdU AlexaFluor 647 Imaging Kit (Invitrogen), stained with Hoechst 33342 and anti-Cyclin B1 rabbit IgG antibody and detected with Alexa 488-conjugated goat anti-rabbit IgG (Invitrogen; A-11008; 1:1000). Cell-cycle profiling of stained U3-DT cells was performed with an ImageXpress Micro (Molecular Devices).

### Immunocytochemistry

Cells cultured on coverslips were washed in phosphate-buffered saline (PBS), fixed in 4% paraformaldehyde in PBS and then blocked with 1% BSA in PBS. The cells were stained with an anti-GPC5 antibody (R&D Systems, Inc.), or anti-Ptc1 antibody (LifeSpan BioSciences, Inc.), and Alexa Fluor 488 or 594 labeled secondary antibody (Molecular Probes, Inc.). The cells were then counterstained with DAPI and visualized using a fluorescence microscope (model BZ-9000; Keyence).

## Results

### Phenotypic characteristics of UE6E7T-3 cell line

**Fig 1:**
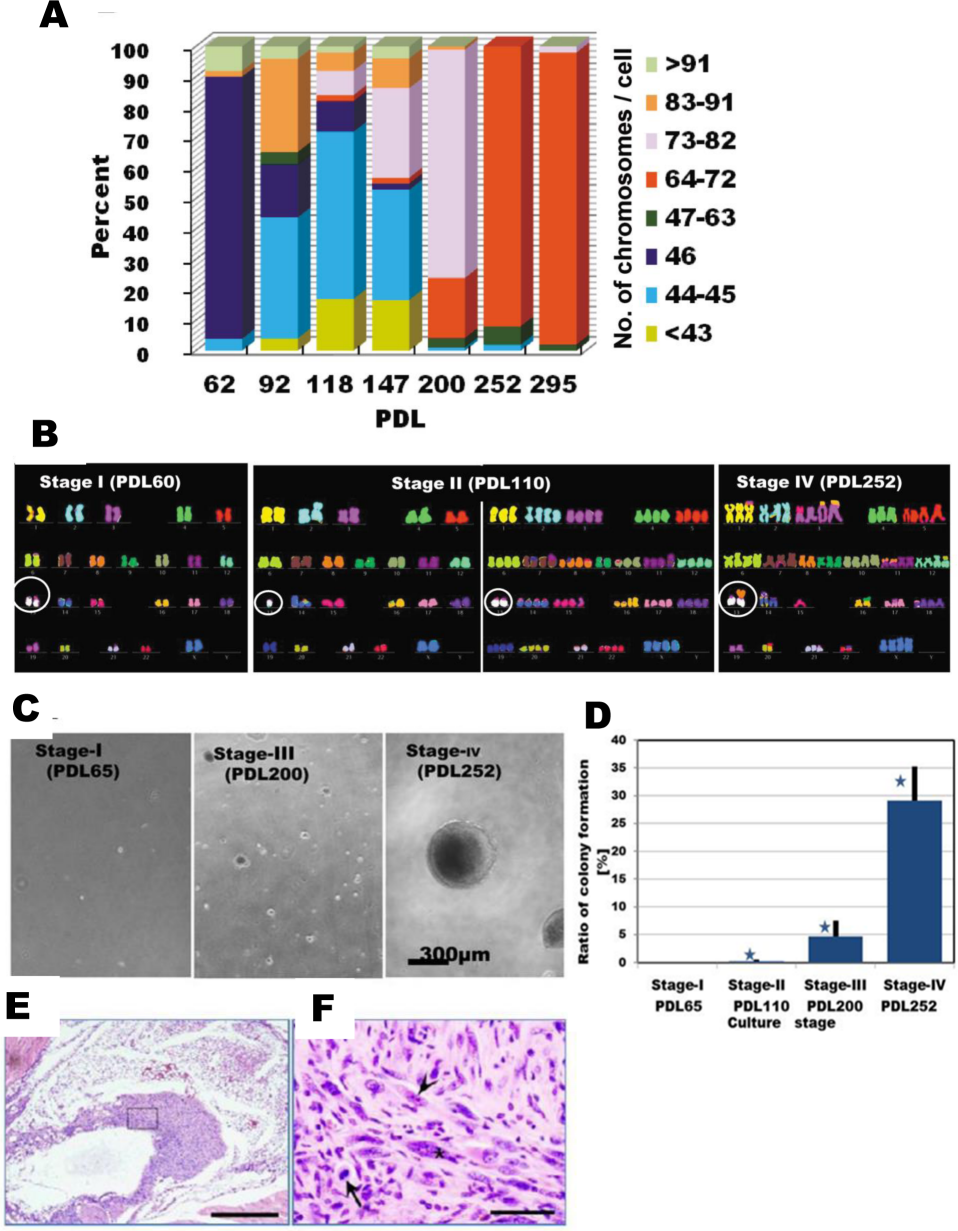
Phenotypic characteristics of UE6E7T-3 during long-term *in vitro* culture. (**A**) Growth curve. UE6E7T-3 cells were seeded at 5X10^3^ cells / ml in POWEREDBY 10. When cells were subconfluently grown, the cells were passaged with trypsin as shown in the text. After counting cell numbers, aliquot of the cultured cells was cultured continuously. (**B**) Phase-contrast images of UE6E7T-3 at four stages. Scale bar, 300 μm. (**C**) Flow cytometry. UE6E7T-3 cells (10,000 cells) were plotted. X-axis is fluorescence intensity. Y-axis is number of cell.

This laboratory has previously demonstrated that aneuploidy, specifically the loss of one copy of chromosome 13, arises in the immortalized hMSC line UE6E7T-3 after prolonged culture [21,22]. We wished to investigate whether continuing culture after the appearance of aneuploidy would induce transformation of UE6E7T-3. To this end, we analyzed the phenotypic characteristics of the cells at various PDL (Fig 1). We monitored the growth characteristics of UE6E7T-3 cell line during long-term culture (Fig 1A). Alterations were observed in population doubling time (DT). DT was 40 hours at initial stage (Stage 1, PDL 65) and it slightly became longer at next stage (DT = 44, Stage II, PDL 120), and decreased to 28–22 hours afterward more than 150 PDL (Stage III, PDL 220 and Stage IV, PDL 252). Over the course of culture until PDL 252, UE6E7T-3 cells exhibited typical fibroblastic morphology with a uniform bipolar spindle shape, and no obvious morphological changes were observed (Fig 1B). Typical markers associated with hMSCs showed similar patterns at Stage I–IV, including markers that are expressed in hMSCs (CD44, CD73, CD90, and CD105) and those that are not (CD34 and CD45), although expression of CD90 and CD105 was slightly reduced at later stages (Fig 1C). In additionally, UE6E7T-3 cells can differentiate into adipocytes and osteocytes, an important characteristic of MSCs [21].

### Changes in karyotype and neoplastic transformation during prolong cultivation

**Fig 2:**
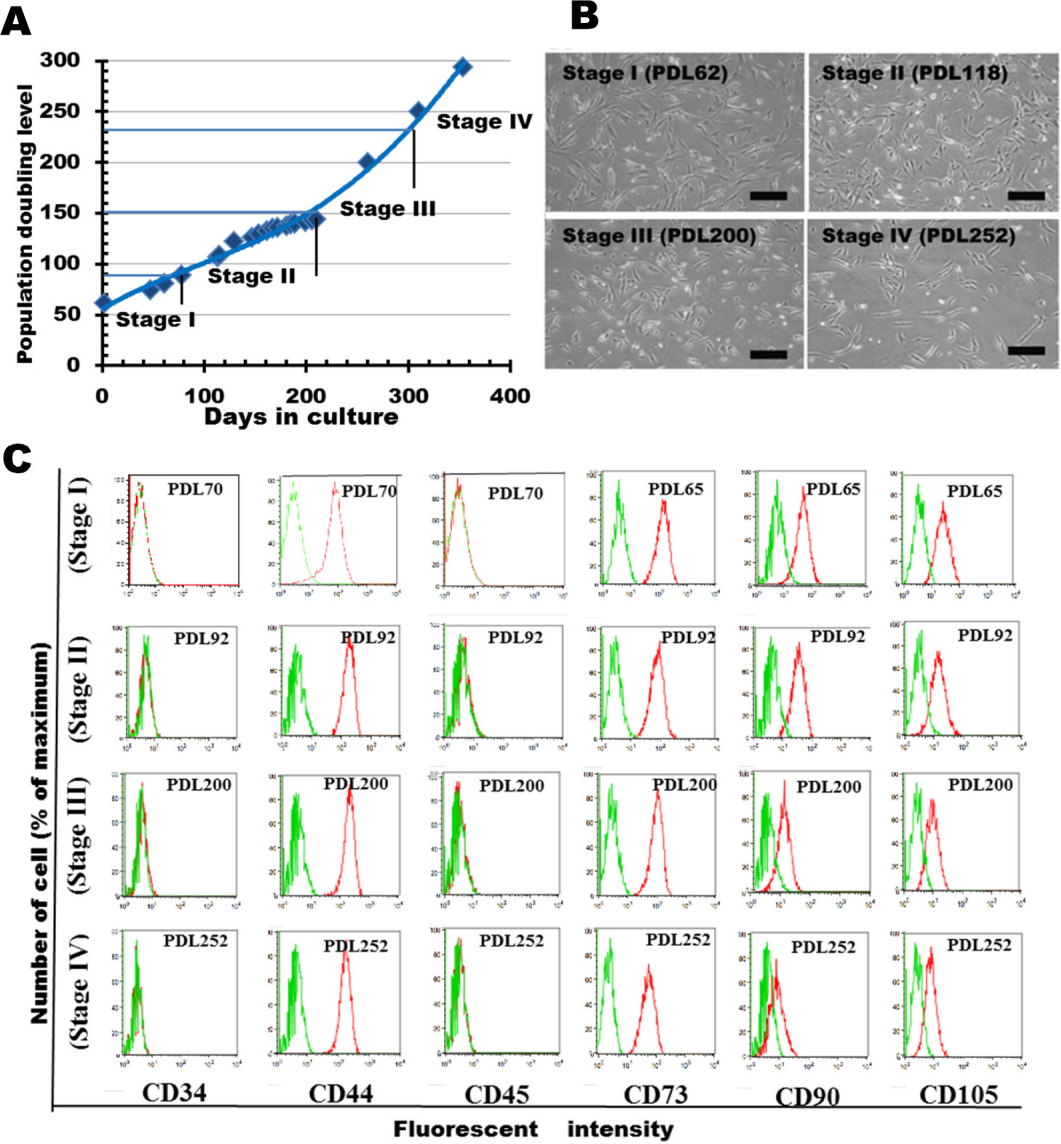
Phenotypic alterations of UE6E7T-3 during long-term culture. (**A**) Changes in chromosomal number at various culture stages. Chromosomes were counted by DAPI staining of 50–80 metaphase spreads at each PDL. Distribution pattern of cells at PDL 62, 92, 118, and 147 were rearranged from raw data from a previous report. (**B**) Multicolored fluorescence *in situ* hybridization (mFISH) karyotyping of UE6E7T-3 at three culture stages. Chromosome 13 is circled. (**C**) Colony formation in soft agar at three culture stages. (**D)** Graphical representation of the relative colony counts (expressed as percentages) at four culture stages. Colonies ≥300 μm in diameter were counted. Bars represent mean numbers of colonies of triplicates (percent + s.d.). ✰, p < 0.01. (**E**) Hematoxylin and eosin staining of invasive sarcoma following i.m. injection of U3-DT. Scale bar, 500μm. (Boxed area is magnified in D). (**F**) Magnification of transformed U3-DT in mouse muscle tissue. Dividing cells (arrow), cells containing two nuclear bodies (arrowhead), and cells containing increased chromatin (asterisk) indicate tumorigenesis. Scale bar, 50 μm.

The growth rate at PDL 252 (population doubling time (DT) of 22 hours) was 2-fold higher than the rate at PDL 65 (DT: 40 hours). Distinct alterations in numerical and structural karyotypes appeared as PDL increased (Fig 2A and 2B). As shown in Fig 2A, nearly 90% of the cell population contained 46 chromosomes at PDL 62 (Stage I). By PDL 92, this proportion had decreased markedly to 17%, and a new population that contained 44–45 chromosomes had appeared. Between PDL 92 and PDL 147 (Stage II), cells were unstable and comprised several populations differing in karyotype: near-diploid, with 44–45 chromosomes (37–55%); diploid (2–17%); near-tetraploid, with 83–91 chromosomes (6–30%); and several minor populations. These populations were gradually replaced by near-tetraploid cells with 73–82 chromosomes (72% at PDL 200, Stage III) and subsequently near-triploidy with 64–72 chromosomes. At further passages (PDL 252 and PDL 295, Stage IV); the population pattern remained stable with mostly near-triploid cells.

We next analyzed the structural karyotypes of UE6E7T-3 by mFISH. At PDL 60, cells exhibited a structurally normal diploid karyotype (Fig 2B, PDL60). By contrast, at PDL 92–147 there were several distinct populations (Fig 2B, PDL 110). Most of the near-diploid population, with 45 chromosomes, had lost one copy of chromosome 13 and one p-arm of chromosome 16 without translocations or insertions (n = 18 spreads; Fig 2B, PDL 110, left), whereas the population with 44 chromosomes had lost an additional chromosome (most often chromosome 21 or X) (n = 17 spreads). The near-tetraploid population consistently maintained two deletions of two copies of chromosome 13 and two p-arms of chromosome 16, with random loss of other chromosomes (Fig 2B, PDL 110, right), but exhibited few structural rearrangements. These results indicate that near-diploid aneuploidy arose through the loss of one or two chromosomes (one of which was chromosome 13) from a diploid cell; subsequently, near-diploid cells spontaneously became near-tetraploid via cleavage failure. After the formation of near-tetraploid, structural rearrangements such as translocations, insertions, and deletions occurred frequently, e.g., the fusion of chromosome 13 and chromosome 8, and the insertion of chromosome 13 into chromosome 14 (Fig 2B, PDL 252). This suggests that the deletion of chromosome 13 (formation of near-diploid aneuploidy) induces further chromosomal instability.

### Neoplastic transformation of UE6E7T-3 cell line

Transformed cells lose contact inhibition, distinguishing them from normal cells, which cannot grow past confluence. To test the contribution of chromosomal instability (CIN) to transformation *in vitro* and *in vivo*, we examined the ability of UE6E7T-3 cells to form colonies in soft agar and tumors in immunodeficient mice. When UE6E7T-3 at various stages were seeded in soft agar, no anchorage-independent growth could be detected at early PDL (PDL 65 and 200), even after 4 weeks in culture. By contrast, a considerable number of anchorage-independent colonies were detected when PDL 252 cells were seeded in soft agar (Fig 2C and 2D). To determine whether this colony formation indicated tumorigenicity [12], cells at PDL 262 were injected subcutaneously or intramuscularly into immunodeficient nude mice. Four of the six mice injected with the PDL 262 cells formed sarcomas at the injection site after 4 weeks. Of the three mice injected intramuscularly, all formed sarcomas, although only one of the three mice injected subcutaneously did. Histological examination of the injected quadriceps femoris revealed these tumors to be invasive sarcomas, exhibiting spindle-shaped cells characteristic of fibroblasts (Fig 2E and 2F, arrow head). This observation indicates that UE6E7T-3 cells had undergone neoplastic transformation during long-term propagation in culture.

### Gene expression profiling characterized at four stages of transformation *in vitro*

To date, the genes studied over the course of cellular transformation have been limited to a small number of genes encoding transcription factors (p53, E2F), cell-cycle regulators (p16, cyclins), oncogene products (RAS, MYC), and tumor suppressors (p53, RB). However, it is not obvious at which stage these factors act or how they play a causal role in advancing transformation. To obtain a comprehensive picture of changes in gene expression related to phenotypic alterations, we took representative samples at four stages during the progression of transformation (see Materials and Methods).

**Fig 3:**
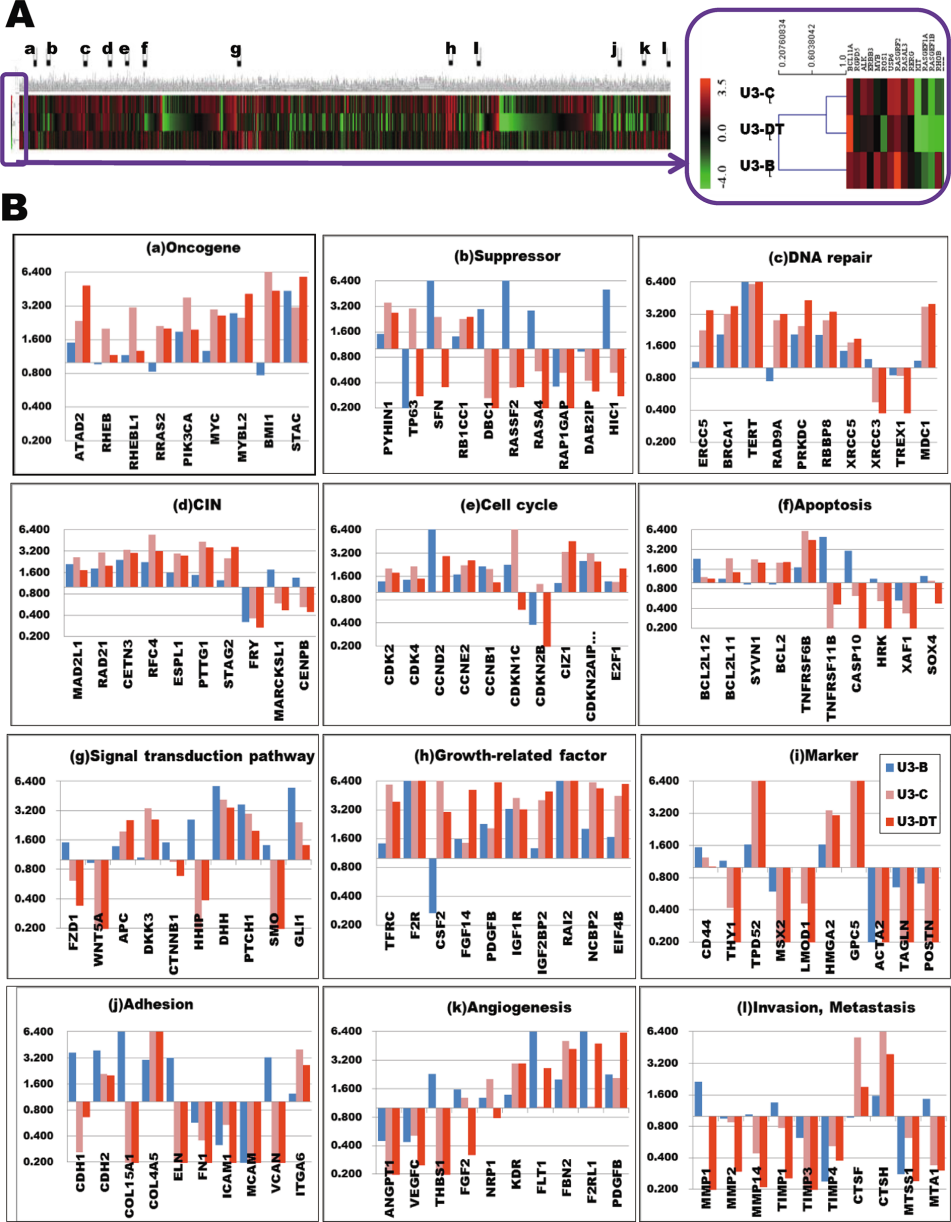
Alterations in expression of 1,732 selected genes. (**A**) Heat-map representation of expression of 1,732 genes from samples U3-B, U3-C, and U3-DT (taken from cultures at Stages II, III, and IV, respectively) compared with expression level in U3-A (from Stage I). Relative expression values (REV) from U3-A,-B,-C, and-DT are listed in S2 Table. (**B**) Functional classification of 1,732 genes and alterations in expression at three culture stages. All data described in this text is shown in S2 and S3 Tables. Blue, U3-B; orange, U3-C; red, U3-DT.

The expression profile from the Stage IV sample (U3-DT) was more similar to that of a Stage III sample (U3-C) than to that of the Stage II sample, U3-B (Fig 3A and S1A Fig). This observation suggests that important alterations in gene expression occur between Stages II and III.

Furthermore, to distinguish the genes that are important in the progression of phenotypic alterations from apparently random alterations, we filtered the 8,032 genes whose expression levels changed significantly (S1A Fig) and yield a subset of 1,570 genes by the IPA analysis (S1B and S1C Fig). The 1,732 genes (Fig 3A and S2 Table) were selected as described in Materials and Methods and were classified into 12 groups on the basis of pathways and cellular function (Fig 3A and 3B, and S2 and S3 Tables). REVs in each group are indicated relative to the control sample U3-A, which was derived from UE6E7T-3 (PDL 80) and exhibits a normal karyotype, typical fibroblastic morphology, and contact inhibition. Experimental results are also enumerated in S2 Table.

### Oncogene and tumor suppressor gene expression

Genetic alterations commonly occurring in human tumors are often found in oncogenes and tumor suppressor genes. Alterations in the expression of such genes occurred in UE6E7T-3, and expression levels were markedly dependent on culture stage (Fig 3Ba and 3Bb). For example, oncogenes, *BMI1* and *MYC*, had low level expression at the early stage (Stage II), but were strikingly enhanced after Stage III. A similar pattern was also exhibited by other oncogenes. Tumor suppressor gene expression displayed an even more marked dependence on culture stage. Expression levels of *SFN*, *DBC1*, *RASSF2*, *DIRAS3*, *RASA4*, and *HIC1* were high at Stage II, but then rapidly decreased (Fig 3Bb, and S2 and S3 Tables). The marked decrease in *DBC1* (tumor suppressor gene) expression at Stages III and IV is in agreement with previous observations [30,31]. Although expression of *PRDM2*, *PYHIN1*, *RBBP4*, and *RB1CC1* (genes encoding activators of p53 or RB) were increased at Stages III and IV, relative expression levels of both *TP53* and *RB1* in U3-C and U3-DT were similar to those in U3-A, as were those of genes such as *PTEN* (Fig 3B, and S2 and S3 Tables).

### DNA repair and chromosomal instability

When normal repair processes fail but apoptosis does not occur, irreparable DNA damage may occur during mitotic recombination events. This damage can take several forms, including double-strand breaks (DSBs) and DNA cross-linkages. In UE6E7T-3, the DNA repair processes appear to be constantly active at all stages, particularly at Stages III and IV, as evidenced by expression of genes encoding many kinds of DNA polymerases (*POLA1*, *POLE*), excision repair enzymes (*PRKDC*, *RBBP8*), nucleases, and checkpoint mediator proteins (*BRCA1*, *MDC1*) (Fig 3Bc, and S2 and S3 Tables).

A number of genes required for stepwise karyotypic alteration (a major source of chromosome instability) were also up-regulated during culture, as were genes associated with DNA repair, particularly at Stages III and IV. These included genes encoding components of the kinetochore and centromere (*NEK2*, *RAD21*, *SPC24*, and *STAG2*), participants in chromosomal segregation (*FOXMI*, *ESPL1*, and *PTTG1*), motor proteins involved in chromosome positioning (*KIF4A*, *KIF2C*, *CENPE*, and *CENPF)*, and regulators of cytokinesis (*PRC1)*. Overexpression of *MAD2L1* and *PTTG1* (securin) has been observed in several tumor types [32,33]. Recently, Schvartzman and colleagues demonstrated direct evidence for inhibition of the p53 or Rb pathways leading to up-regulation of *MAD2L1*, and that this up-regulation is required for generating chromosome instability [34]. Our observations of the inactivation of Rb and p53 by HPV-16 E6/E7, generation of chromosome instability, and *MAD2L1* overexpression strongly support their result. In addition, down-regulation of *FRY* or *CENPB* may also contribute to chromosome instability through de-regulation of chromosome alignment and formation of the spindle and kinetochore. All up-regulated chromosome instability genes in UE6E7T-3 are among the 70 genes best correlated with total functional aneuploidy in several cancer types [35].

### Cell cycle

**Fig 4:**
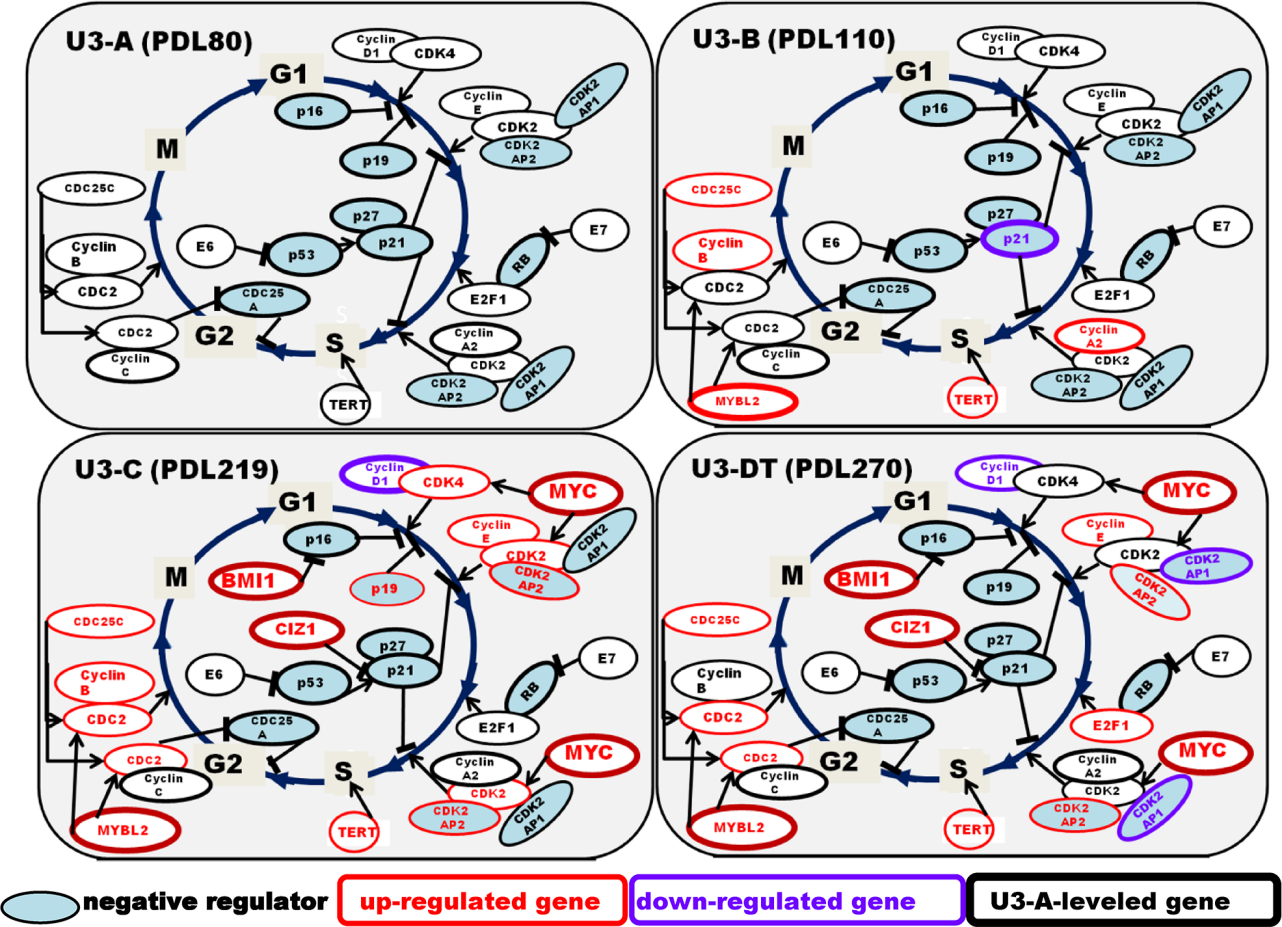
Gene expression in the cell-cycle pathway during long-term culture of UE6E7T-3. U3-A, U3-B, U3-C, and U3-DT are samples from culture Stages I, II, III, and IV, respectively. Changes in gene expression relative to the level in U3-A (Stage I) are shown. Red characters, up-regulated; purple characters, down-regulated; black characters, U3-A (control) level; Blue fill, negative regulator. Genes with REV >2 or <0.5 are indicated as up- or down-regulated, respectively.

Generally, one cell cycle is completed within 20–100 hours, most of which is spent in the G1 phase [36]. Mechanisms regulating the cell cycle are common to all cells and are driven by three types of factors: cyclin-dependent kinases (CDK), cyclins, and cyclin-dependent kinase inhibitors (CKI). As shown in Fig 3Be, and S2 and S3 Tables, CDK-related genes (*CDC25C*, *CDK2*, *CDK4*, *CDC2L6*, *CDC2*, and *CDK2AP2)* and cyclin genes (*CCND2*, *CCNE2*, and *CCNB1*) were up-regulated at all culture stages, whereas CKI-encoding genes such as *CDKNIC* (p57), *CDKN2B* (p15), *CDKNIA* (p21), *CDKN2A* (p16), and *CDK2AP1* were down-regulated at all stages. These alterations in cell-cycle gene expression resulted in an uncontrolled increase in proliferation. Gene expression profiles at four stages of culture (PDL 80, 110, 219, and 270) are shown in Fig 4. As a control, we used U3-A (PDL 80). Marked differences in gene expression patterns between U3-B and U3-C were observed. In U3-C (Stage III), we also observed high expression of *MYC*, *MYBL2* (CDK-activating gene), *BMI1* (p16-suppressing gene), *CIZ1* (p21-inhibiting gene, [37]), and *ATAD2* (cofactor gene of MYC) [38], in addition to increased gene-expression of CDKs and cyclins. These changes were concomitant with a strong depression of CKI-encoding gene expression. Together, these observations suggest that these alterations play a crucial role in rapid proliferation.

*E2F1*, which encodes a transcriptional activator, was also expressed at higher levels at late stages. The expression of *E2F1* is intimately regulated by RB. Functional inactivation of the RB pathway in this cell line leads to *E2F1* overexpression, which may be correlated with rapid cell proliferation. Another factor contributing to the higher rate of proliferation is the functional loss of p53 checkpoint pathways. Together, these alterations explain the decrease in U3-C doubling time, which dropped to 28 hours from 44 hours in U3-B.

### Apoptosis

Resistance to apoptosis is one of the hallmarks of cancer. At Stage II, apoptosis-inducing genes such as *TNFRSF11B* and *CASP10* were expressed at high levels, but as in the case of tumor suppressor genes, expression rapidly decreased. By contrast, expression of apoptosis-suppressing genes (*TNFRSF6B*, *BCL2*, etc.) was enhanced at later stages (Fig 3Bf, and S2 and S3 Tables).

### Signaling pathways and growth factors

Extracellular signaling molecules, such as growth factors, activate specific receptors on the target cell membrane, activating second messengers, and eventually elicit a physiological response. As shown in Fig 3Bg, and S2 and S3 Tables, striking alterations in gene expression were observed only in the Hedgehog (Hh) signaling pathway (*DHH*, *PTCH1*, *SMO*, and *GLI1*); however, some of these genes were down-regulated at later stages. By contrast, genes encoding Wnt signaling factors (*FZD1*, *WNT5A*, *APC*, and *CTNNB1*) are not likely to be implicated in the transformation of UE6E7T-3 cells.

Unregulated expression of growth factors, cytokines, receptors, and signaling components is linked to developmental abnormalities and a variety of chronic diseases, including cancer. In UE6E7T-3, such genes were considerably up-regulated, especially at later stages (Fig 3Bh, and S2 and S3 Tables). The expression levels of *IGF2BP2*, *RAI2*, *SLC25A27*, *NCBP2*, and *EIF4B* were greatly enhanced, as is the case in tumor development. *TFRC*, a marker of human pancreatic cancer [39], was also expressed at a high level, as was *F2R*, which encodes a G-protein-coupled receptor. By contrast, expression levels of genes encoding chemokines or interleukins and their receptors were undetectable.

### Lineage markers and adhesion-associated genes

Genes encoding markers of mesenchymal cells (CD44, CD73, CD90, and CD105) were expressed in U3-DT at levels similar to those in U3-A (Fig 1C). Tumor-marker genes (*HMGA2*, *TPD52*, and *GPC5*) were overexpressed in U3-C and U3-DT (Fig 3Bi, and S2 and S3 Tables). U3-A has the potential to differentiate into several types of cell, including adipocytes and osteoblasts [21]. Gene expression of markers characteristic of these cell types was down-regulated in U3-DT cells (*ACTA2*, *MSX2*, *CNN1*, *TAGLN*, *LMOD1*, and *POSTN*).

Expression of adhesion-related genes was strongly suppressed in U3-C and U3-DT, with the exception of *COL4A5*, a member of the collagen family (Fig 3Bj, and S2 and S3 Tables). E-cadherin (product of *CDH1*) is one of the most important molecules in cell-cell adhesion in epithelial tissue. *CDH1* expression was markedly reduced in U3-C (Fig 3Bj, and S2 and S3 Tables). Suppression of *CDH1* expression is one of the primary molecular events responsible for dysfunction in cell-cell adhesion, such as that which occurs in tumor progression. On the other hand, N-cadherin (*CDH2*) expression remained high. Other adhesion molecules were also expressed at low levels comparable to those in U3-A, as observed in tumor cells. Expression levels of integrins, receptors that mediate attachment of cells to extracellular components such as collagen and fibronectin, were normal; however, collagen expression reduced considerably with tumorigenic progression. These results suggest that reduced expression of these adhesion molecules plays an important part in promoting invasion into surrounding stroma.

### Angiogenesis, invasion, and metastasis

Oxygen and nutrients supplied by vasculature are crucial for cell function and survival. Beyond a certain size, tumors induce blood vessel growth (angiogenesis) by secreting various growth factors, thereby allowing the tumor to obtain oxygen and essential nutrients *in vivo*. In UE6E7T-3, genes encoding major contributors to angiogenesis (*TEK*, *VEGFC*, *FGF2*, and *ANGPT1*) were expressed at remarkably low levels, although the expression of genes encoding their receptors (*KDR*, *FLT1*) was high (Fig 3Bk, and S2 and S3 Tables). Another major contributor to angiogenesis is matrix metalloproteinase (MMP1, MMP2, MMP3, and MMP14). These proteases were also expressed at low levels, as were the MMP inhibitors (TIMP1, TIMP3, and TIMP4) (Fig 3Bl, and S2 and S3 Tables).

Similar low levels of expression were observed in invasion- and metastasis-related genes. Cell-cell and cell-matrix adhesion molecules play an important role in cell migration. E-cadherin, which is considered an important suppressor of invasion and metastasis, rapidly decreased in gene expression at Stage III (Fig 3Bj, and S2 and S3 Tables). This reduction is likely to represent a key step in the acquisition of invasive properties. Furthermore, integrin family genes (*ICAM1*, *ICAM2*, *ICAM5*, and *VCAM1*) were also expressed at low levels (Fig 3Bj, and S2 and S3 Tables). These results suggest that even U3-DT may still be in the earliest stages of tumorigenesis. Alternatively, in terms of angiogenesis-, invasion- and metastasis-related gene expression, tumorigenesis in fibroblastic cells may differ from that of epithelial cells.

### Validation of RNA-Seq

**Fig 5:**
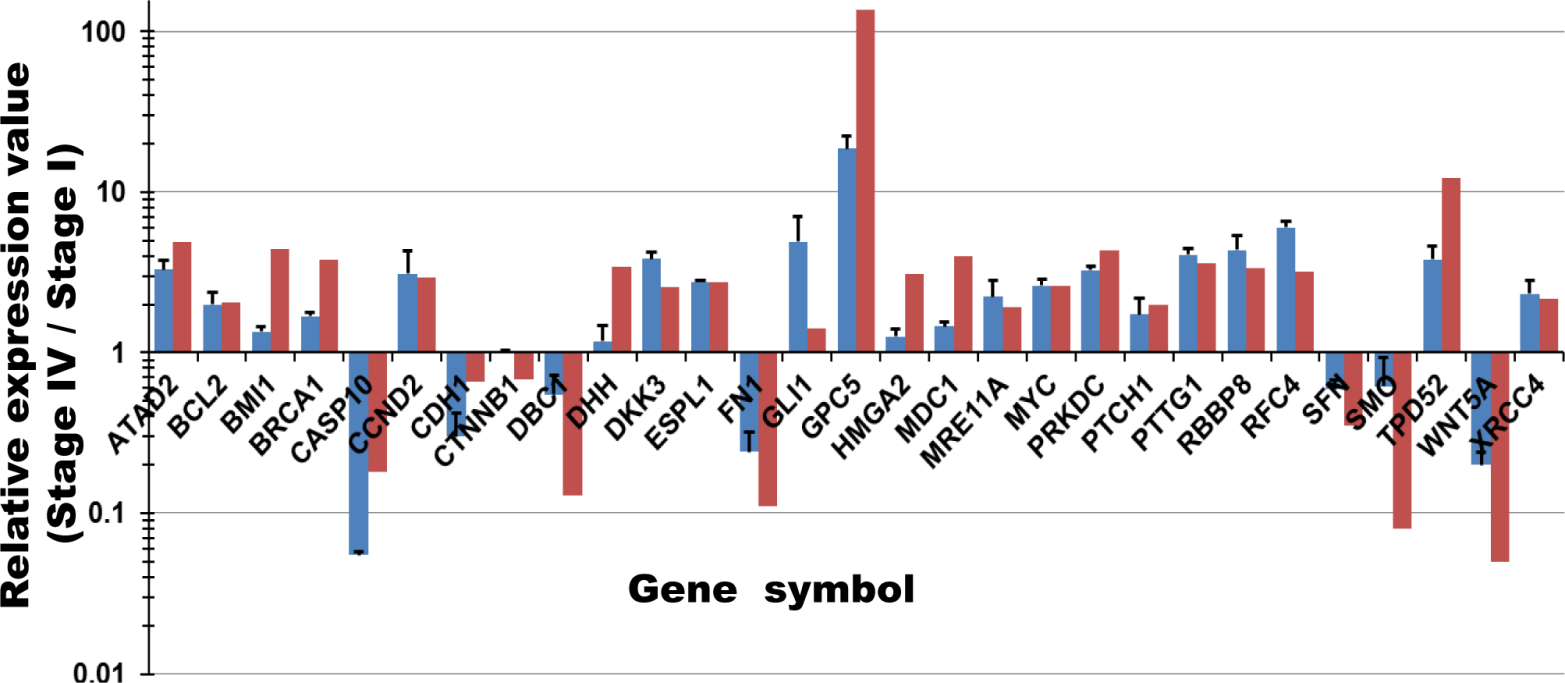
Comparison of RNA-Seq and qRT-PCR. Results of qRT-PCR analysis of remarkable genes at Stage IV are shown as the relative expression ratio (blue) compared to those of the RNA-Seq analysis with whole transcriptome sequencing (red). Bar, standard error.

To evaluate of RNA-Seq analysis, we chose 29 genes at Stage IV in each pathway identified by RNA-Seq for qRT-PCR analysis (Fig 5). The expression pattern of the genes determined by qRT-PCR analysis is similar to the results of RNA-Seq analysis, although slight differences in expression level were detected between two methods. In addition, to confirm the accuracy of RNA-Seq data we compared the expression levels of 41 samples according to RNA-Seq and qRT-PCR analyses, including 29 genes at Stage IV analyzed by Method A and 4 genes at Stage II-IV analyzed by Method B (S1 Method), and obtained a high concordance (Spearman’s rank correlation coefficient, R = 0.76) between these two sets of data (S1 Method and S1 Fig). A coefficient of 0.76 corresponds to a significance level of slight less than 0.1%. This indicated that our RNA-Seq could reliably measure gene expression differences.

### GPC5 expression affects UE6E7T-3 cell proliferation

**Fig 6:**
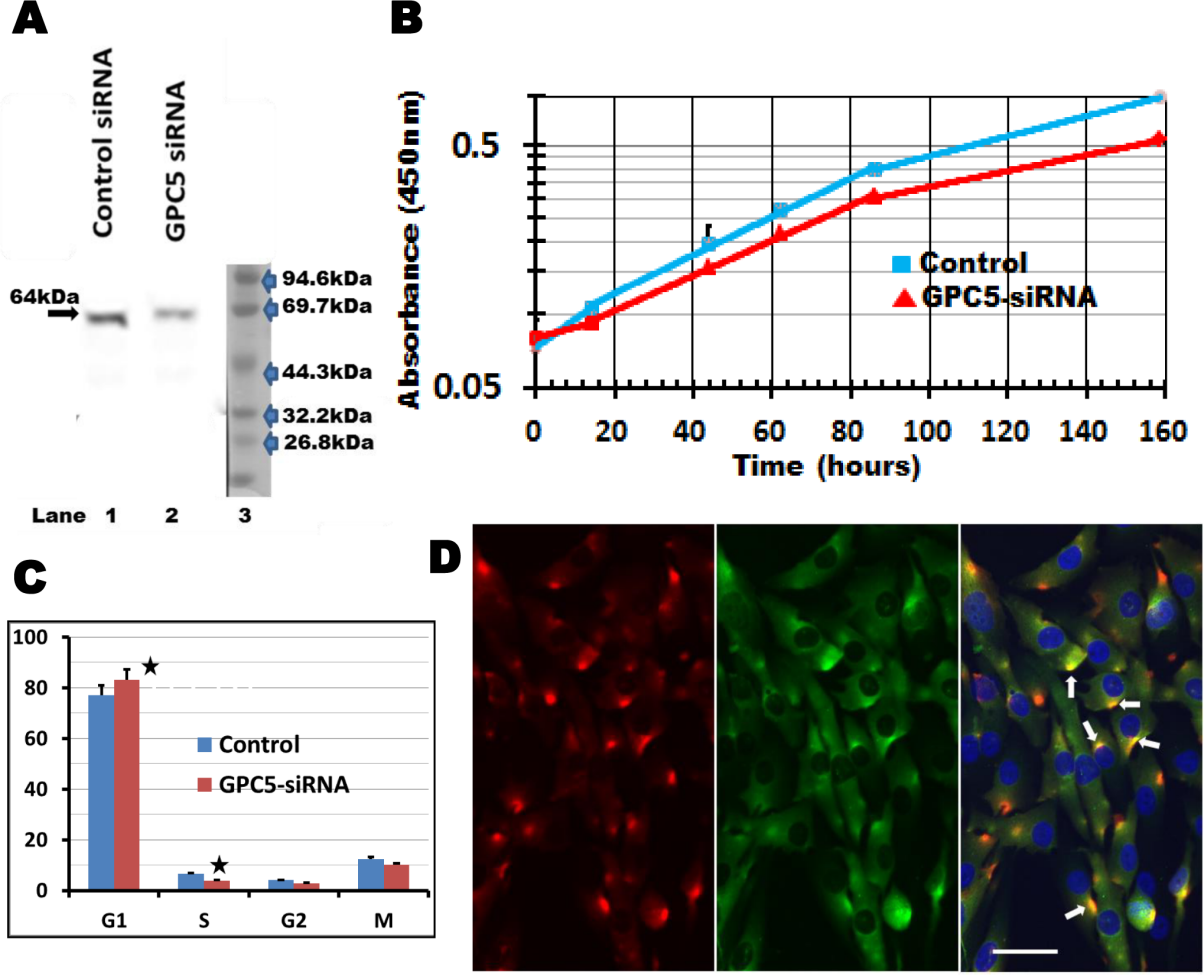
GPC5 expression affects proliferation in U3-DT cells. (**A**) Western blot analysis. Cells were treated with siRNA for 3 days. Cells were lysed and the levels of GPC5 were examined by Western blotting with anti-GPC5 Rabbit mAb. Lane 1, control siRNA-treated cells; Lane 2, GPC5 siRNA-treated cells; Lane 3, markers. (**B**) Growth curve. siRNA-treated cells (2,000 cells/well) were then seeded in 96-well culture plates and proliferation was assessed by CCK-8 assay at the indicated time points. An exponential approximation line of control (blue) or siRNA-treated (red) dots was drown between for 14 to 86hr. The results are expressed as the mean ± S.D. of quintuplicate wells. P < 0.05. (**C**) Cell-cycle distribution of U3-DT cells. Cell cycle distribution (%) of U3-DT cells incubated in medium containing siRNA (red) or not (blue) is shown. (**D**) Immunofluorescence profile of U3-DT cells. Double immunofluorescence labeling with anti-human GPC5 (red) and Ptc1 (green) antibodies in U3-DT cells. Nuclei are labeled with DAPI (merged image). Arrows: GPC5 and Ptc1 positive-merged spots (yellow) are clearly visible in U3-DT cells. Scale bar = 50 μm.

A unique pattern of gene expression was observed in UE6E7T-3 cells; GPC5 was overexpressed at the late stage of long-term culture. To assess whether this overexpression affected the proliferation in U3-DT cells, we examined the effect of GPC5 knockdown. Knockdown of GPC5 by GPC5 siRNA (SMARTpool siRNA) was confirmed by Western blot analysis (Fig 6A), and resulted in significant inhibition of proliferation of U3-DT cells compared with that in cells infected with control non-target siRNA (Fig 6B). These results show that overexpression of GPC5 stimulates the proliferation of U3-DT cells.

The proliferation rate of cells treated with target-specific siRNAs was comparable to the rate of cells treated with a non-targeting siRNA control, but the growth curve had lag phase at the initial stage (Fig 6B). To examine which phase in the cell cycle is affected by the target-specific siRNA, we used an ImageXpressMicro analyzer (Molecular Devices). As shown in Fig 6C, treatment with specific siRNAs resulted in an increase of cell numbers in G1 phase and slight decreases in S, G2 and M phases, indicating that the decreased proliferation induced by siRNA is due to the retention of cells in G1. Cell-cycle distribution analysis did not reveal any cells in the sub-G1 phase following siRNA treatment and we did not observe any cells with apoptotic morphology.

Li et al. [28] reported recently that GPC5 localizes to the primary cilium in rhabdomyosarcoma (RMS) cells and promotes cell proliferation by binding to Ptc1, the cell-surface receptor for Hh signaling. Our RNA-Seq and qRT-PCR analyses showed that Ptc1 is significantly upregulated in U3-DT cells. Therefore, to investigate the interaction between GPC5 and Ptc1, we examined the localization of endogenous GPC5 and Ptc1 in U3-DT cells with antibodies against GPC5 and Ptc1. Although both GPC5 and Ptc1 antibodies weakly and diffusely stained U3-A cells (data not shown), strong staining of GPC5 was detected at the same region of concentrated Ptc1 staining (Fig 6D). This fluorescence staining pattern is similar to the endogenous localization of GPC5 in the cilia of RMS cells [28] and of Ptc1 in the cilia of NIH 3T3 cells [40]. This finding suggests that GPC5 interacts with components of the Hh signaling pathway.

## Discussion

This study shows that the hMSC line, UE6E7T-3, immortalized with the *hTERT* gene in combination with the HPV-16 E6/E7 genes, gradually transformed during prolonged culture. The HPV-16 E6/E7 gene products, the E6 and E7 proteins, abrogate repression of the cell cycle through their associations with p53 and pRb, respectively [41]. In parallel with acquisition of increased tumorigenicity, numerous alterations in gene expression occurred spontaneously with culture passage. These alterations are similar to previous data, which MSCs derived from p21^-/-^p53^+/-^mice completely lost p53 expression after *in vitro* long-term culture and formed sarcomas *in vivo* [42] and muline MSCs derived osteosarcoma cells in consequence of aneuploidization and genomic loss of Cdkn2 [43]. Our comprehensive gene expression analysis provided several important pieces of evidence regarding transformation-related changes at different stages of prolonged culture. A summary of the alterations in cytological features and gene expression over the course of the transformation process is shown in Table 1.

**Table 1:** Summary of cellular and genetic characteristics of UE6E7T-3 cells at four stages of transformation.

Stage	I	II	III	IV
PDL	60–90	91–150	151–230	231–295
**Ploidy**	**diploid**	**aneuploid**	**mix polyploid**	**near-triploid**
**Number of chromosome (mode)**	**46**	**45**	**88**	**68**
**Cell shape**	**F**	**F**	**F**	**F**
**Doubling time (hr)**	**40**	**44**	**28**	**22**
**Growth in soft agar**	**negative**	**negative**	**slight**	**positive**
**Growth in mouse**	**NT**	**NT**	**NT**	**sarcoma**
**Oncogene**	**control**	**C**	**+++**	**+++**
**Tumor suppressor gene**	**control**	**+++**	**-**	**-**
**DNA repair gene (NHEJ)**	**control**	**+**	**+++**	**+++**
**CIN gene**	**control**	**++**	**+++**	**+++**
**Cell cycle activator**	**control**	**++**	**++**	**++**
**Cell cycle suppressor**	**control**	**+/-**	**-**	**-**
**Apoptosis activator**	**control**	**+++**	**-**	**-**
**Apoptosis inhibitor**	**control**	**C**	**++**	**+++**
**Growth factor, Signal transduction**	**control**	**+**	**+++**	**+++**
**Adhesion-related gene**	**control**	**++**	**-**	**-**
**Angiogenesis, Invasion, Metastasis**	**control**	**+/-**	**+/-**	**+/-**

Note: (+), (-) and (C) denote the up-regulated, down-regulated, or control-level expression of major genes shown in Fig 3B. F, fibroblastic-like; NT, not tested.

First, we demonstrated that only mitotically defective near-triploid cells (U3-DT), present in a mixed cell population exhibiting chromosome instability (CIN), contributed to the tumorigenicity of UE6E7T-3 cells; however, tumors formed at 66% of injection sites, so, therefore, additional genetic alterations may be necessary for U3-DT cell tumorigenesis. The contribution of polyploidy cells to tumorigenesis has been demonstrated in other studies. Tetraploidy can promote CIN and tumorigenesis in p53-null mouse mammary epithelial cells [44] and in Pim-1-expressing human prostate and mammary epithelial cells [45]. U3-A cells with microsatellite instability in chromosome 13 have a diploid karyotype during the first 90 PDL, and they preferentially lose one copy of chromosome 13 upon prolonged culture [22]. The loss of a whole chromosome 13, even a single copy, causes significant damage to near-diploid cells, but some survive (i.e., are selected), subsequently, acquire additional aneuploidy and tumor-related gene expression. Finally, unstable triploidy confers tumorigenicity and is a dominant feature of tumors. This karyotypic heterogeneity of genomes is consistent with the neoplastic progression theory [46], which holds that tumorigenesis occurs by a complex evolutionary process: according to this theory, progression develops as genetic instability is acquired, leading to the accumulation of genetic alterations and the continual selective outgrowth of variant subpopulations of tumor cells with a proliferative advantage.

By contrast, despite having a normal diploid karyotype with no detectable chromosome abnormalities, hTERT-transduced adult human MSCs (hTERT20) formed tumors after long-term culture [47]; however, these cells exhibit deletion of the Ink4a/ARF locus and epigenetic silencing of DBCCR1. A similar phenomenon was reported in hTERT-immortalized human fibroblast cell lines (WI-38, Leiden), which have a diploid karyotype and in which Ink4a is deleted [48,49]; however, these cell lines require active H-Ras or H-Ras and inactive p53 for tumor formation, resulting in the acquirement of chromosome abnormalities. Therefore, these reports suggest that h-TERT-mediated immortalization does not significantly affect genome integrity at the chromosome level, and that h-TERT-associated genetic and/or epigenetic alterations contribute to tumorigenicity rather than karyotype alterations. In addition, the genetic and/or epigenetic alterations may largely reflect differences in the pre-existing genetic makeup of each strain and the culture conditions.

Secondly, we showed that UE6E7T-3 acquired properties of transformed cells even without *RAS-* and *MYC*-mediated transduction. However, this transformation had a long latency, requiring continuous *in vitro* passages. Significant overexpression of *RAS* was not detected at any stage of prolonged culture, suggesting that, at least for UE6E7T-3, the RAS pathway is not critical in transformation. This result is consistent with those of two previous studies [18,50], in contrast to other reports that RAS activation is an essential requirement for transformation [2–10]. We did, however, observe the marked up-regulation of other oncogenes (*BMI1*, *STAC*, *MYC*, *PIK3CA*, *RRAS2*, and *ATAD2*) in Stages III and IV. From these results, we infer that alternative pathways or currently unidentified factors may exist, which influence transformation in long-term culture.

Thirdly, we clearly demonstrated that numerous alterations in gene expression, in multiple pathways related to the acquisition of increased tumorigenicity, occurred spontaneously over prolonged culture. Our analysis of gene expression identified a crucial stage (Stage III), involving drastic changes in multiple pathways that determines the fate of UE6E7T-3 (Table 1). These changes included high expression of oncogenes as well as genes related to DNA-damage repair, chromosome instability, cell-cycle activation, inhibition of apoptosis, and increase of growth factors, concomitant with a strong depression of genes involved in tumor suppression, apoptosis, and adhesion. In particular, the suddenly increased expression of *MYC*, *BMI1*, *GPC5*, and *CIZ1* at this stage suggests that these genes play a key role in converting UE6E7T-3 to a tumorigenic state. *BMI1* encodes a key regulator in several cellular processes, including normal stem cell renewal and cancer cell proliferation [51]. The high expression of *BMI1* in U3-C and U3-DT suggests that these cells are tumorigenic. The main target of BMI1 is the *INK4A* locus, which encodes two structurally distinct proteins, p16^INK4a^ and p14^ARF^, both of which play important roles in cell-cycle regulation. In UE6E7T-3, up-regulation of *BMI1* could result in low *CDKN2A* (p16^INK4a^-encoding gene) expression, allowing increased transcription of *E2F*, which would in turn lead to increased proliferation. *ATAD2* and *CIZ1*, which were also expressed at high levels in the late stages, are also highly expressed in a significant proportion of human tumors [38,52], suggesting that these genes contribute to proliferation, ultimately leading to transformation. Similar patterns were observed for apoptosis inhibitors and apoptosis accelerators (Fig 3Bf, and S2 and S3 Tables). When DNA damage is too extensive, checkpoint pathways trigger apoptosis. However, Stage III cells (U3-C) exhibited decreased expression of apoptosis-inducing genes and increased expression of anti-apoptotic genes. These cells failed to undergo apoptosis; the resulting spontaneous survivors (U3-DT) are distinct from U3-A and U3-B. Mutations arising from unrepaired DNA damage in normal human cells are prevented by the p53 and pRB checkpoint pathways, resulting in DNA repair, apoptosis, or senescence. Normal cells stop dividing until they have completed repair to their DNA. By contrast, genetically unstable UE6E7T-3 cells with defective DNA can easily avoid checkpoints due to breaks in their checkpoint pathways, and thereby maintain a higher rate of proliferation than normal cells. Such cells induce subsequent mutations by repetitive mitosis, over the course of which missegregation of whole chromosomes generates additional aneuploidy.

Similarly, expression of E-cadherin, a known tumor suppressor in many cancers [53,54], rapidly dropped at Stage III (Fig 3Bj, and S2 and S3 Tables). This shutdown was concomitant with the repression of expression of other adhesion genes, resulting in reduced cell-cell or cell-extracellular matrix interactions, suggestive of an invasive tendency. However, MMPs, which help to degrade extracellular matrix proteins, were expressed at lower levels in U3-C and U3-DT (Fig 3Bl, and S2 and S3 Tables). In addition, VEGFC, which along with MMPs is a major contributor to angiogenesis, was also expressed at low levels in U3-DT. VEGFC causes a signaling cascade resulting in maturation of endothelial cells into blood vessels. The low VEGFC expression suggests that cascade members downstream, including MMPs, VCAMs, and ICAMs, would not be stimulated, and U3-DT cells may be promoted with respect to malignancy in future. Together, these alterations in gene expression in different core pathways strongly suggest that the transition of UE6E7T-3 into a tumorigenic state occurred at Stage III, concomitant with drastic alterations in chromosomal numbers and chromosomal structures. At the same time, expression of genes encoding tumor markers rose dramatically at this stage, and the cells acquired defects in their ability to differentiate into several lineages.

Finally, from this comprehensive genetic analyses, we identified molecular signatures in U3-DT that are commonly expressed in various types of tumors, as mentioned above, suggesting that transformants of many cell types show common signature in gene expression.

In addition, one important finding in this study pertains to the glypican-5-gene (GPC5)-related signaling. GPC5, which is a sarcoma cell marker [55], was markedly overexpressed at later culture stage in association with transformation of UE6E7T-3 cells. Other studies recently showed that GPC5 stimulates the proliferation of rhabdomyosarcoma (RMS) cells [28] and lung metastatic cells in salivary adenoid cystic carcinoma [56], while GPC5 may be a tumor suppressor in non-small cell lung cancer [57]. We have shown here that GPC5 augmented cell proliferation in UE6E7T-3 cell line (Fig 6). GPC5 encodes one of the six members of the glypican family (GPC1 to GPC6), heparan sulfate proteoglycans, which serve as essential modulators of key regulatory proteins such as Wnt, FGF, and Hh [58–60]. Li, et al. demonstrated that GPC5 acts as an activator of Hh signaling in RMS cell proliferation [28], whereas these authors have previously reported that GPC3 acts as an inhibitor of the same signaling in mouse embryo [61]. This indicates that two members of glypican family can display opposite roles in the regulation of Hh signaling. In UE6E7T-3 cell line, GPC3 is little expressed (S1 Table). In addition of marked overexpression of GPC5, a high level of expression of Hh signaling components (DHH, PTCH, and GLI1) was also found in U3-DT cells. Furthermore, DKK3, an inhibitor of the Wnt signaling, was overexpressed in U3-DT.

Based on these results, we propose that GPC5 stimulates the proliferation of U3-DT through the Hh signaling pathway by stabilizing the interaction between ligands and receptors. In human cerebellar granule cells, *BMI1* expression is up-regulated in response to sonic Hh protein (product of *SHH*) and parallels expression of *GLI1* [62]. *GLI1* overexpression induces *BMI1* expression, implying that *BMI1* is a downstream target in the Hh signaling pathway, and that its activation promotes cell proliferation by suppressing p16^INK4A^. Therefore, *GPC5* may inhibit the Wnt signaling pathway and may also play an important role in the rapid proliferation of U3-DT at late stages, by regulating *BMI1* via the Hh signaling pathway. The possibility must be elucidated by future studies.

Expression of the *BCL11A*, *IL17REL*, and *CCDC3* genes was markedly elevated at Stage IV. Although BCL11A is an essential regulator of normal lymphocyte development in mouse embryos [63] and its expression is elevated in some lymphoma patients [64], the mechanism underlying this is unknown. The elevated expression of these genes suggests that they function in the tumorigenesis of U3-DT cells. In the future, we aim to clarify the functions of these genes in U3-DT cell tumorigenesis.

The results obtained in this study suggest that the UE6E7T-3 cell line can be used as a culture model for neoplastic transformation associated with dynamic alterations in gene expression and karyotype. Thus, this model may be a useful tool for assessing the functional contribution of genes showed by expression profiling to neoplastic transformation.

The cell lines generated in this study, each of which is characteristic of a particular stage in the transformation process, could have wider applications. U3-DT may prove useful for screening anticancer drugs, such as CtIP inhibitors, and U3-B might be useful for investigating the mechanisms of genetic aberration associated with neoplastic transformation or tumorigenesis. Furthermore, GPC5 might be a biomarker for sarcoma of human cells.

## Supporting Information

S1 Method Estimation of gene expression values by qRT-PCR. The qRT-PCR was performed by two methods.
(PDF)

S1 Fig Cluster and pathway analyses of 8,032 genes expressed in U3-B,-C, and-DT. Cluster analysis of 8,032 genes performed using MeV software (**A**) and ‘Diseases and Disorders’ analysis using IPA software (**B**). The ‘Diseases and Disorders’ analysis revealed that 1,570 genes had expression patterns in U3-B, U3-C, and U3-DT that were characteristic of ‘Cancer’ (p-value, 1.92E-21), within which the ‘Function’ characteristics pinpointed ‘Tumorigenesis’ (**C**).
(TIF)

S2 Fig Correlation between the qRT-PCR and the RNA-Seq data. (**A**) Relative expression values estimated by qRT-PCR or RNA-Seq. To validate the relative expression values generated by RNA-Seq, the relative expression values of 41 samples with 33 selected genes were calculated from qRT-PCR and RNA-Seq data. The correlation between two values was then verified statistically. (**B**) Correlation between the relative expression ratios calculated from the qRT-PCR and RNA-Seq data. The relative expression values of 41 samples calculated from qRT-PCR and RNA-Seq were plotted on the X-axis and Y-axis, respectively. Spearman’s rank correlation coefficient, (R) was calculated in accordance with a formula described in Method B (S1 Method).
(TIF)

S1 Table Gene expression value of 33,565 genes in U3-A, U3-B, U3-C, and U3-DT cells. (PDF)

S2 Table The relative expression value (REV) of 1,732 genes shown in Fig 3A. Expression value of U3-A,-B,-C, and-DT is the expression level of each gene divided by the level of GAPDH expression in the same sample. (*) Expression values for U3-A were obtained by dividing the value of each gene by that of GAPDH of U3-A, and then multiplying by 1,000. (**) REV of each gene in U3-A,-B,-C, and-DT is the expression level in U3-B,-C, or—DT divided by the expression level in U3-A.
(PDF)

S3 Table List of genes shown in Fig 3B. Of the 1,732 genes listed in S1 Table, gene symbols and function of 180 genes (Fig 3B) are shown, in general, according to the NCBI gene database.
(PDF)

## References

[1] Vogelstein B, Kinzler KW. Cancer genes and the pathways they control. Nat Med. 2004;10: 789–799. 15286780 10.1038/nm1087

[2] Hahn WC, Counter CM, Lundberg AS, Beijersbergen RL, Brooks MW, Weinberg RA. Creation of human tumour cells with defined genetic elements. Nature. 1999;400: 464–468. 10440377 10.1038/22780

[3] Akagi T, Sasai K, Hanafusa H. Refractory nature of normal human diploid fibroblasts with respect to oncogene-mediated transformation. Pros Natl Acad Sci U S A. 2003;100: 13567–13572. 14597713 10.1073/pnas.1834876100 PMC263854

[4] Drayton S, Rowe J, Jones R, Vatcheva R, Cuthbert-Heavens D, Marshall J, et al Tumor suppressor p16^INK4a^ determines sensitivity of human cells to transformation by cooperating cellular oncogenes. Cancer Cell. 2003;4: 301–310. 14585357 10.1016/s1535-6108(03)00242-3

[5] Boehm JS, Hession MT, Bulmer SE, Hahn WC. Transformation of human and murine fibroblasts without viral oncoproteins. Mol Cell Biol. 2005;25: 6464–6474. 16024784 10.1128/MCB.25.15.6464-6474.2005 PMC1190359

[6] Kendall SD, Linardic CM, Adam SJ, Counter CM. A Network of genetic events sufficient to convert normal human cells to a tumorigenic state. Cancer Res. 2005;65: 9824–9828. 16267004 10.1158/0008-5472.CAN-05-1543

[7] Funes JM, Quintero M, Henderson S, Martinez D, Qureshi U, Westwood C, et al Transformation of human mesenchymal stem cells increases their dependency on oxidative phosphorylation for energy production. Proc Natl Acad Sci U S A. 2007;104: 6223–6228. 17384149 10.1073/pnas.0700690104 PMC1851087

[8] Mahale AM, Khan ZA, Igarashi M, Nanjangud GJ, Qiao RF, Yao S, et al Clonal selection in malignant transformation of human fibroblasts transduced with defined cellular oncogenes. Cancer Res. 2008;68: 1417–1426. 10.1158/0008-5472.CAN-07-3021 18316605

[9] Narisawa-Saito M, Yoshimatsu Y, Ohno S, Yugawa T, Egawa N, Fujita M, et al An in vitro multistep carcinogenesis model for human cervical cancer. Cancer Res. 2008;68: 5699–5705. 10.1158/0008-5472.CAN-07-6862 18632622

[10] Sasai K, Sukezane T, Yanagida E, Nakagawa H, Hotta A, Itoh T, et al Oncogene‐mediated human lung epithelial cell transformation produces adenocarcinoma phenotypes in vivo. Cancer Res. 2011;71: 2541–2549. 10.1158/0008-5472.CAN-10-2221 21447735

[11] Li R, Sonik A, Stindl R, Rasnick D, Duesberg P. Aneuploidy vs. gene mutation hypothesis of cancer: Recent study claims mutation but is found to support aneuploidy. Proc Natl Acad Sci U S A. 2000;97: 3236–3241. 10725343 10.1073/pnas.040529797 PMC16222

[12] Weaver BA, Silk AD, Montagna C, Verdier-Pinard P, Cleveland DW. Aneuploidy acts both oncogenically and as a tumor suppressor. Cancer Cell. 2007;11: 25–36. 17189716 10.1016/j.ccr.2006.12.003

[13] Baker DJ, Jeganathan KB, Cameron JD, Thompson M, Juneja S, Kopecka A, et al BubR1 insufficiency causes early onset of aging-associated phenotypes and infertility in mice. Nat Genet. 2004;36: 744–749. 15208629 10.1038/ng1382

[14] Kalitsis P, Fowler KJ, Griffiths B, Earle E, Chow CW, Jamsen K, et al Increased chromosome instability but not cancer predisposition in haploinsufficient Bub3 mice. Genes Chromosomes Cancer. 2005;44: 29–36. 15898111 10.1002/gcc.20215

[15] Jeganathan K, Malureanu L, Baker DJ, Abraham SC, van Deursen JM. Bub1 mediates cell death in response to chromosome missegregation and acts to suppress spontaneous tumorigenesis. J Cell Biol. 2007;179: 255–267. 17938250 10.1083/jcb.200706015 PMC2064762

[16] Torres EM, Williams BR, Tang YC, Amon A. Thoughts on aneuploidy. Cold Spring Harb Symp Quant Biol. 2010;75: 445–451. 10.1101/sqb.2010.75.025 21289044 PMC3293208

[17] Burns JS, Abdallah BM, Guldberg P, Rygaard J, Schrøder HD, Kassem M. Tumorigenic heterogeneity in cancer stem cells evolved from long-term cultures of telomerase-immortalized human mesenchymal stem cells. Cancer Res. 2005;65: 3126–3135. 15833842 10.1158/0008-5472.CAN-04-2218

[18] Zongaro S, Stanchina E, Colombo T, D’Incalci M, Giulotto E, Mondello C, Stepwise neoplastic transformation of a telomerase immortalized fibroblast cell line. Cancer Res. 2005;65: 11411–11418. 16357149 10.1158/0008-5472.CAN-05-1140

[19] Milyavsky M, Tabach Y, Shats I, Erez N, Cohen Y, Tang X, et al Transcriptional programs following genetic alterations *in p53*, *INK4A*, and *H-Ras* genes along defined stages of malignant transformation. Cancer Res. 2005;65: 4530–4543. 15930270 10.1158/0008-5472.CAN-04-3880

[20] Närvä E, Autio R, Rahkonen N, Kong L, Harrison N, Kitsberg D, et al High-resolution DNA analysis of human embryonic stem cell lines reveals culture-induced copy number changes and loss of heterozygosity. Nat Biotechnol. 2010;28: 371–377. 10.1038/nbt.1615 20351689

[21] Takeuchi M, Takeuchi K, Kohara A, Satoh M, Shioda S, Ozawa Y, et al Chromosomal instability in human mesenchymal stem cells immortalized with human papilloma virus E6, E7, and hTERT genes. In Vitro Cell Dev Biol Anim. 2007;43: 129–138. 17514511 10.1007/s11626-007-9021-9

[22] Takeuchi M, Takeuchi K, Ozawa Y, Kohara A, Mizusawa H. Aneuploidy in immortalized human mesenchymal stem cells with non-random loss of chromosome 13 in culture. In Vitro Cell Dev Biol Anim. 2009;45: 290–299. 10.1007/s11626-008-9174-1 19184247

[23] Imabayashi H, Mori T, Gojo S, Kiyono T, Sugiyama T, Irie R, et al Redifferentiation of dedifferentiated chondrocytes and chondrogenesis of human bone marrow stromal cells via chondrosphere formation with expression profiling by large-scale cDNA analysis. Exp Cell Res. 2003;288: 35–50. 12878157 10.1016/s0014-4827(03)00130-7

[24] Morton CL, Houghton PJ. Establishment of human tumor xenografts in immunodeficient mice. Nature Protocol. 2007;2: 247–250. 17406581 10.1038/nprot.2007.25

[25] Tuch BB, Laborde RR, Xu X, Gu J, Chung CB, Monighetti CK, et al Tumor transcriptome sequencing reveals allelic expression imbalances associated with copy number alterations. PLoS ONE. 2010;5(2): e9317 10.1371/journal.pone.0009317 20174472 PMC2824832

[26] Higashino A, Sakate R, Kameoka Y, Takahashi I, Hirata M, Tanuma R, et al Whole-genome sequencing and analysis of the Malaysian cynomolgus macaque (Macaca fascicularis) genome. Genome Biol. 2012;13: R58 10.1186/gb-2012-13-7-r58 22747675 PMC3491380

[27] Mortazavi A, Williams BA, McCue K, Schaeffer L, Wold B. Mapping and quantifying mammalian transcriptomes by RNA-Seq. Nat Methods. 2008;5: 621–628. 10.1038/nmeth.1226 18516045 PMC13303166

[28] Li F, Shi W, Capurro M, Filmus J. Glypican-5 stimulates rhabdomyosarcoma cell proliferation by activating Hedgehog signaling. J Cell Biol. 2011;192: 691–704. 10.1083/jcb.201008087 21339334 PMC3044117

[29] Kume H, Muraoka S, Kuga T, Adachi J, Narumi R, Watanabe S. et al Discovery of colorectal cancer biomarker candidates by membrane proteomic analysis and subsequent verification using selected reaction monitoring (SRM) and tissue microarray (TMA) analysis. Mol Cell Proteomics. 2014;13: 1471–1484. 10.1074/mcp.M113.037093 24687888 PMC4047467

[30] Nishiyama H, Gill JH, Pitt E, Kennedy W, Knowles MA. Negative regulation of G1/S transition by the candidate bladder tumour suppressor gene DBCCR1. Oncogene. 2001;20: 2956–2964. 11420708 10.1038/sj.onc.1204432

[31] Serakinci N, Guldberg P, Burns JS, Abdallah B, Schrødder H, Jensen T, et al Adult human mesenchymal stem cell as a target for neoplastic transformation. Oncogene. 2004;23: 5095–5098. 15107831 10.1038/sj.onc.1207651

[32] Zou H, McGarry TJ, Bernal T, Kirschner MW. Identification of a vertebrate sister-chromatid separation inhibitor involved in transformation and tumorigenesis. Science. 1999;285: 418–422. 10411507 10.1126/science.285.5426.418

[33] Hernando E, Nahlé Z, Juan G, Diaz-Rodriguez E, Alaminos M, Hemann M, et al Rb inactivation promotes genomic instability by uncoupling cell cycle progression from mitotic control. Nature. 2004;430: 797–802. 15306814 10.1038/nature02820

[34] Schvartzman JM, Duijf PH, Sotillo R, Coker C, Benezra R. Mad2 is a critical mediator of the chromosome instability observed upon Rb and p53 pathway inhibition. Cancer Cell. 2011;19: 701–714. 10.1016/j.ccr.2011.04.017 21665145 PMC3120099

[35] Carter SL, Eklund AC, Kohane IS, Harris LN, Szallasi Z. A signature of chromosomal instability inferred from gene expression profiles predicts clinical outcome in multiple human cancers. Nat Genet. 2006;38: 1043–1048. 16921376 10.1038/ng1861

[36] Israels ED, Israels LG. The cell cycle. Oncologist. 2000;5: 510–513. 11110604 10.1634/theoncologist.5-6-510

[37] Lukasik A, Uniewicz KA, Kulis M, Kozlowski P. Ciz1, a p21 ^cip1/Waf1^-interacting zinc finger protein and DNA replication factor, is a novel molecular partner for human enhancer of rudimentary homolog. FEBS J. 2008;275: 332–340. 18081865 10.1111/j.1742-4658.2007.06203.x

[38] Ciró M, Prosperini E, Quarto M, Grazini U, Walfridsson J, McBlane F, et al ATAD2 is a novel cofactor for MYC, overexpressed and amplified in aggressive tumors. Cancer Res. 2009;69: 8491–8498. 10.1158/0008-5472.CAN-09-2131 19843847

[39] Ryschich E, Huszty G, Knaebel HP, Hartel M, Büchler MW, Schmidt J. Transferrin receptor is a marker of malignant phenotype in human pancreatic cancer and in neuroendocrine carcinoma of the pancreas. Eur J Cancer. 2004;40: 1418–1422. 15177502 10.1016/j.ejca.2004.01.036

[40] Rohatgi R, Milenkovic L, Scott MP. Patched1 regulates hedgehog signaling at the primary cilium. Science. 2007;317: 372–376. 17641202 10.1126/science.1139740

[41] Kiyono T, Foster SA, Koop JI, McDougall JK, Galloway DA, Klingelhutz AJ. Both Rb/p16^INK4a^ inactivation and telomerase activity are required to immortalize human epithelial cells. Nature. 1998;396: 84–88. 9817205 10.1038/23962

[42] Rodriguez R, Rubio R, Masip M, Catalina P, Nieto A, de la Cueva E, et al Loss of p53 induces tumorigenesis in p21-deficient mesenchymal stem cells. Neoplasia. 2009;11: 397–407. 19308294 10.1593/neo.81620 PMC2657886

[43] Mohseny AB, Szuhai K, Romeo S, Buddingh EP, Briaire-de Bruijin I, de Jong D, et al Osteosarcoma originates from mesenchymal stem cells in consequence of aneuploidization and genomic loss of Cdkn2. J Pathol. 2009;219: 294–305. 10.1002/path.2603 19718709

[44] Fujiwara T, Bandi M, Nitta M, Ivanova EV, Bronson RT, Pellman D. Cytokinesis failure generating tetraploids promotes tumorigenesis in p53-null cells. Nature. 2005;437: 1043–1047. 16222300 10.1038/nature04217

[45] Roh M, Franco OE, Hayward SW, van der Meer R, Abdulkadir SA. A role for polyploidy in the tumorigenicity of Pim-1-expressing human prostate and mammary epithelial cells. PLoS ONE. 2008;3(7): e2572 10.1371/journal.pone.0002572 18596907 PMC2440349

[46] Barrett MT, Sanchez CA, Prevo LJ, Wong DJ, Galipeau PC, Paulson TG, et al Evolution of neoplastic cell lineages in Barrett oesophagus. Nat Genet. 1999;22: 106–109. 10319873 10.1038/8816 PMC1559997

[47] Burns JS, Abdallah BM, Schrøder HD, Kassem M. The histopathology of a human mesenchymal stem cell experimental tumor model: support for an hMSC origin for Ewing’s sarcoma?. Histol And Histopathol. 2008;23: 1229–1240. 18712675 10.14670/HH-23.1229

[48] Milyavsky M, Shats I, Erez N, Tang X, Senderovich S, Meerson A, et al Prolonged culture of telomerase-immortalized human fibroblasts leads to a premalignant phenotype. Cancer Res. 2003;63: 7147–7157. 14612508

[49] Brookes S, Rowe J, Ruas M, Llanos S, Clark PA, Lomax M, et al INK4a-deficient human diploid fibroblasts are resistant to RAS-induced senescence. EMBO J. 2002;21: 2936–2945. 12065407 10.1093/emboj/cdf289 PMC126048

[50] Jin Y, Shima Y, Furu M, Aoyama T, Nakamata T, Nakayama T, et al Absence of oncogenic mutations of RAS family genes in soft tissue sarcomas of 100 Japanese patients. Anticancer Res. 2010;30: 245–252. 20150643

[51] Leung C, Lingbeek M, Shakhova O, Liu J, Tanger E, Saremaslani P, et al Bmi1 is essential for cerebellar development and is overexpressed in human medulloblastomas. Nature. 2004;428: 337–341. 15029199 10.1038/nature02385

[52] Rahman FA, Aziz N, Coverley D. Differential detection of alternatively spliced variants of Ciz1 in normal and cancer cells using a custom exon-junction microarray. BMC Cancer. 2010;10: 482–493. 10.1186/1471-2407-10-482 20831784 PMC2945943

[53] Derksen PW, Liu X, Saridin F, van der Gulden H, Zevenhoven J, Evers B, et al Somatic inactivation of E-cadherin and p53 in mice leads to metastatic lobular mammary carcinoma through induction of anoikis resistance and angiogenesis. Cancer Cell. 2006;10: 437–449. 17097565 10.1016/j.ccr.2006.09.013

[54] Derksen PW, Braumuller TM, van der Burg E, Hornsveld M, Mesman E, Wesseling J, et al Mammary-specific inactivation of E-cadherin and p53 impairs functional gland development and leads to pleomorphic invasive lobular carcinoma in mice. Dis Model Mech. 2011;4: 347–358. 10.1242/dmm.006395 21282721 PMC3097456

[55] Williamson D, Selfe J, Gordon T, Lu YJ, Pritchard-Jones K, Murai K, et al Role for amplification and expression of glypican-5 in rhabdomyosarcoma. Cancer Res. 2007;67: 57–65. 17210683 10.1158/0008-5472.CAN-06-1650

[56] Zhang Y, Wang J, Dong F, Li H, Hou Y. The role of GPC5 in lung metastasis of salivary adenoid cystic carcinoma. Arch Oral Biol. 2014;59: 1172–1182. 10.1016/j.archoralbio.2014.07.009 25093697

[57] Yang X, Zhang Z, Qiu M, Hu J, Fan X, Wang J, et al Glypican-5 is a novel metastasis suppressor gene in non-small cell lung cancer. Cancer Lett. 2013;341: 265–273. 10.1016/j.canlet.2013.08.020 23962560

[58] Filmus J, Capurro M, Rast J. Glypicans. Genome Biol. 2008;9: 2241–224.6. 10.1186/gb-2008-9-5-224 18505598 PMC2441458

[59] Li Y, Yang P. GPC5 gene and its related pathways in lung cancer. J Thorac Oncol. 2011;6: 2–5. 10.1097/JTO.0b013e3181fd6b04 21178712 PMC3020075

[60] Filmus J, Capurro M. The role of glypicans in Hedgehog signaling. Matrix Biology. 2014;35: 248–252. 10.1016/j.matbio.2013.12.007 24412155

[61] Capurro MI, Xu P, Shi W, Li F, Jia A, Filmus J. Glypican-3 inhibits hedgehog signaling during development by competing with patched for hedgehog binding. Develop Cell. 2008;14: 700–711. 10.1016/j.devcel.2008.03.006 18477453

[62] Leung C, Lingbeek M, Shakhova O, Liu J, Tanger E, Saremaslani P, et al Bmi1 is essential for cerebellar development and is overexpressed in human medulloblastomas. Nature. 2004;428: 337–341. 15029199 10.1038/nature02385

[63] Yu Y, Wang J, Khaled W, Burke S, Li P, Chen X, et al Bcl11a is essential for lymphoid development and negatively regulates P53. J Exp Med. 2012;209: 2467–2483. 10.1084/jem.20121846 23230003 PMC3526365

[64] Satterwhite E, Sonoki T, Willis TG, Harder L, Nowak R, Arriola EL, et al The BCL11 gene family involvement of *BCL11A* in lymphoid malignancies. Blood. 2001;98: 3413–3420. 11719382 10.1182/blood.v98.12.3413

