# Correction: Transcriptional Dynamics of Immortalized Human Mesenchymal Stem Cells during Transformation

**DOI:** 10.1371/journal.pone.0131383

**Published:** 2015-06-22

**Authors:** Masao Takeuchi, Atsunori Higashino, Kikuko Takeuchi, Yutaro Hori, Kazuko Koshiba-Takeuchi, Hatsune Makino, Yoko Monobe, Marina Kishida, Jun Adachi, Jun Takeuchi, Takeshi Tomonaga, Akihiro Umezawa, Yosuke Kameoka, Ken-ichi Akagi

The images for Figures 1 and 2 are incorrectly switched. The image that appears as Figure 1 should be Figure 2, and the image that appears as Figure 2 should be Figure 1. The figure legends appear in the correct order.

In the section entitled “Validation of RNA-Seq”, S2 Fig should be referenced instead of S1 Fig.

Additionally, the files linked to S1 Fig and the file linked to S2 Fig are incorrect. The file linked to S1 Fig should be the file linked to S2 Fig, and the file linked to S2 Fig should be the file linked to S1 Fig.
