# Supplementary material for: Transcriptional Dynamics of Immortalized Human Mesenchymal Stem Cells during Transformation
Source: PLoS One. 2015 May 15;10(5):e0126562. doi: 10.1371/journal.pone.0126562 (PMC4433180; doi:10.1371/journal.pone.0126562)
Supplement: S1 Method — The qRT-PCR was performed by two methods. (PDF) [file pone.0126562.s001.pdf]

Method A. qRT-PCR assay using SYBR® Premix EXTaq.

Method of this assay by SYBR® Premix EXTaq. was described in Method of the text. Thirty primer sets used in the experiments were purchased from the Takara-bio and shown in the following Table.

Gene symbol	Gene ID	Product size (bp)	Forward primer	Reverse primer
ATAD2	29028	84	GGCCAGACAGCAGGCTGATAA	TTTCTAGCCCTCAATGACCGAGTAA
BCL2	596	114	CTGAGTAAATCCATGCACCTAAACC	AATCCACTGTCACTCTTGCAAATTC
BMI1	648	172	CTGCAGCTCGCTTCAAGATG	TTAGCTCAGTGATCTTGATTCTCGT
BRCA1	4077	108	AGACTCCATGGTGCATTCAAGAGTA	GACATTGCTCAATAGGTGCTGGTAG
CASP10	843	167	AACCGTTTACTTCCAGAAGATTGGT	TTGAGGTTCTCCACATCTTGGAC
CCND2	894	70	TGGTCATTTCAGGCACAACGATA	GGTCTGATAGCCCAGCCAAGTTTA
CDH1	999	84	AAGTGCTGCAGCCAAAGACAGA	AAATTGCCAGGCTCAATGACAAG
CTNNB1	1499	144	CATCCTAGCTCGGGATGTTCAC	TCCTTGTCCTGAGCAAGTTCAC
DBC1	1620	111	GGCTCCTGCAACAAGGGCTA	CTGGAAGTCCAGGTCAGTCTCAAAG
DHH	50846	91	CCCGACATCATCTTCAAGGA	CAATGGCCAAAGCGTTCAC
DKK3	27122	87	AGGTGTTGTGCATTTGTTCAGCTC	GCAGTTTAACCCTGCCTGACTCTC
ESPL1	9700	159	GGAGCAATTTCGAGCCAATTTC	ACAGCCTGCCCATCAAGGA
FN1	2335	140	GCAGTGGCTGAAGACACAAGG	TGTAGGTGAATGGTAAGACACATGG
GAPDH	2597	138	GCACCGTCAAGGCTGAGAAC	TGGTGAAGACGCCAGTGGA
GLI1	2735	120	ACACATATGGACCTGGCTTTGGA	CTGCCCTATGTGAAGCCCTATTTG
GPC5	2262	170	GGGACTGTGATGATGAAGATGGTT	CACTGCACATCCTGCTCCTG
HMGA2	8091	177	CTGAGACTGGCAGATCGCTCAC	ATGAGCTGGCCAATGAGGTTTC
MDC1	9656	149	TTATTAGCTGCTGTGGAGGCACAT	CAGCAGGAACTCAGGCGAGA
MRE11A	4361	200	AGGTGGTGCTTCTGCCATTAG	CTGGTAAATTCCATAACAGGCTGA
MYC	4609	175	CCTGGTGCTCCATGAGGAGA	CAGTGGGCTGTGAGGAGGTTT
PRKDC	5591	90	GGTGCCAATCCAGCAGTCA	TCGTGCCACAGCCACATAGTC
PTCH1	5727	162	ATCCATAATGTCTGGAACTTTGCTG	CATGCTAGGTCGCCAATGGTA
PTTG1	9232	106	TTCCTGCCTCAGATGATGCCTAT	AGGTGCGCAATCTGGTGCT
RBBP8	5932	171	TGCTGGTTCTCATGAGCCAATAA	TCTGCTCCCGGATCTATACTCCAC
RFC4	5984	77	TTAACTGTGTCAGGAAGTCGCTCA	GAATCTGCTTCATCCAGAATCACAA
SFN	2810	81	GCCAAGACCACTTTCGACGAG	CATGATGAGGGTGCTGTCTTTGTAG
SMO	6608	122	TGCTGCACACACTCACCTCTAA	AGGCTTTGTCACTCACTGCTCCTA
TPD52	7163	127	GGGCAAGTCCATTATGCAAC	CACATCTTCAGCAAGCAGTCATTTA
WNT5A	7474	146	TTACCACTGCAACTATTGCACCTC	CACAATGAACCTTTAGTTTCCAACC
XRCC4	7518	146	AATGACTGCTGACCGAGATCCA	TTGGTGCAATATCAGTGACATCAAG

Method B. qRT-PCR assay by use of an Ambion® Cells-to-CT™ Kit.

U3-A (PDL 76, Stage I), U3-B (PDL110, Stage II), U3-C (PDL 200, Stage III), and U3-DT (PDL 250, Stage IV) cells were seeded in 96-well plates at a density $5x10^3$/well or $1.5x10^3$ cells/well. Two days later, RNA was extracted from the cells in each well and qRT-PCR was performed using an Ambion® Cells-to-CT™ Kit (Life Technologies). The cDNA was synthesized with a mixture containing a TaqMan Probe and gene-specific primers, and subjected to qPCR in a real-time PCR instrument (ABI-7900HT; Life Technologies), according to the manufacturer's protocol. GAPDH was used as the reference control gene. The gene expression value for each gene was calculated in accordance with the methods described in the manufacturer's protocol, using the software included with the ABI-7900HT PCR instrument.

The gene-specific primers used in the assay were as follows;

MYC: (CACCGCCCACCACCAGCAGCGACTC),

CDKN2A: (CCTAGAAGACCAGGTCATGATGATG),

HRAS: (TCGGCCAAGACCCGGCAGGGAGTGG),

DBC1: (GCCACATTGGGAGGGGAGGAGGCTT).

Means and standard deviations of these gene expression values were

calculated from 4 measurements.

To validate gene expression values, Spearman's Rank correlation Coefficient (R) was calculated as shown in Supporting Figure (S2 Fig), by following formula;

$$(R) = 1- (6\Sigma d^2)/(n^3-n)$$
