# Supplementary figures and images for: Transcriptional Dynamics of Immortalized Human Mesenchymal Stem Cells during Transformation
Source: PLoS One. 2015 May 15;10(5):e0126562. doi: 10.1371/journal.pone.0126562 (PMC4433180; doi:10.1371/journal.pone.0126562)

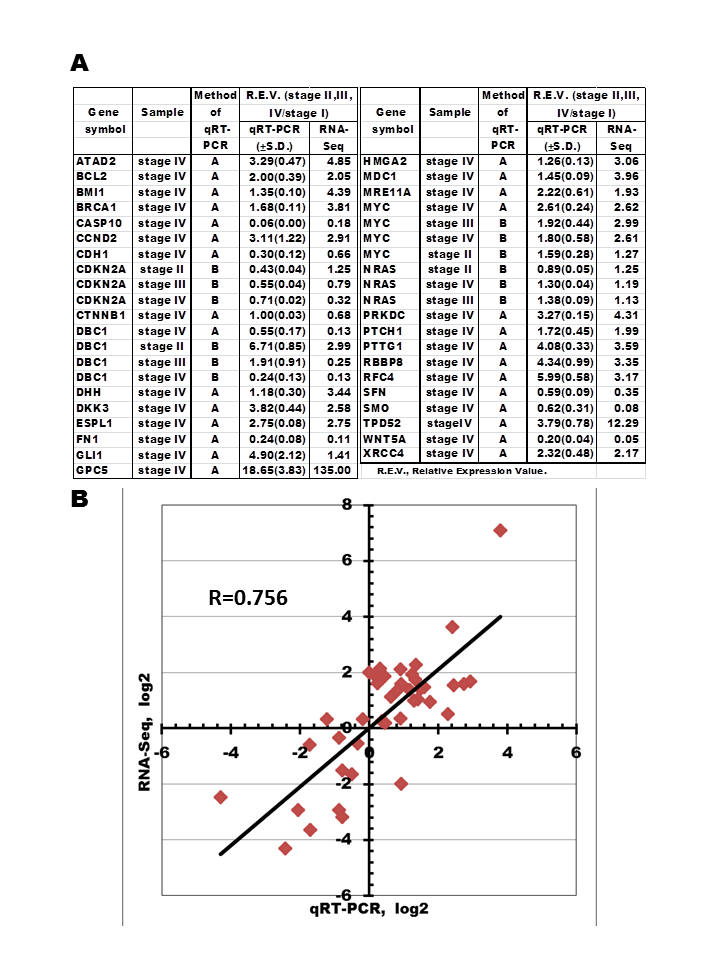

Supplement: S1 Fig — Cluster analysis of 8,032 genes performed using MeV software (A) and ‘Diseases and Disorders’ analysis using IPA software (B). The ‘Diseases and Disorders’ analysis revealed that 1,570 genes had expression patterns in U3-B, U3-C, and U3-DT that were characteristic of ‘Cancer’ (p-value, 1.92E-21), within which the ‘Function’ characteristics pinpointed ‘Tumorigenesis’ (C). (TIF) [file pone.0126562.s002.tif]

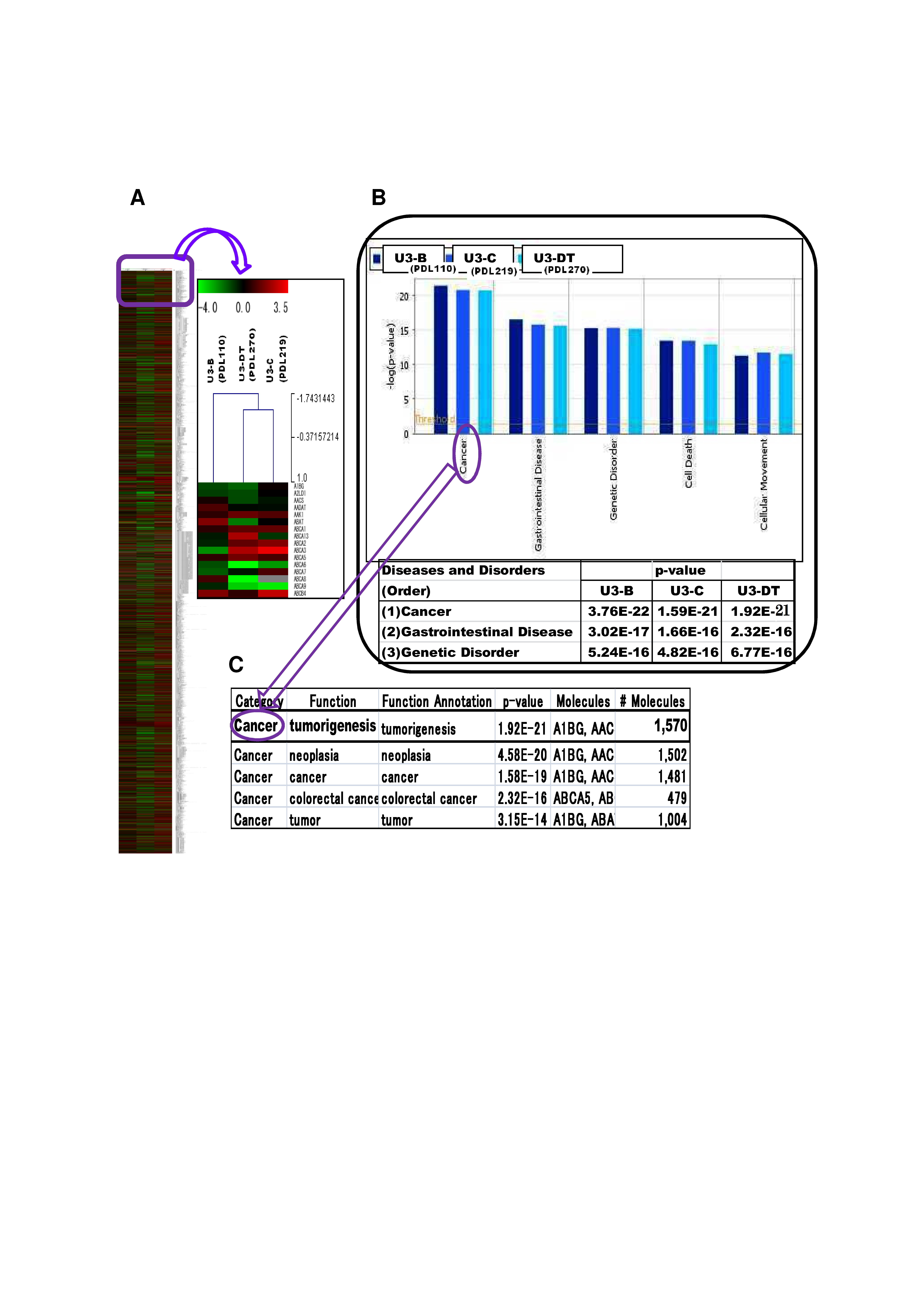

Supplement: S2 Fig — (A) Relative expression values estimated by qRT-PCR or RNA-Seq. To validate the relative expression values generated by RNA-Seq, the relative expression values of 41 samples with 33 selected genes were calculated from qRT-PCR and RNA-Seq data. The correlation between two values was then verified statistically. (B) Correlation between the relative expression ratios calculated from the qRT-PCR and RNA-Seq data. The relative expression values of 41 samples calculated from qRT-PCR and RNA-Seq were plotted on the X-axis and Y-axis, respectively. Spearman’s rank correlation coefficient, (R) was calculated in accordance with a formula described in Method B (S1 Method). (TIF) [file pone.0126562.s003.tif]
