# Supplementary material for: Transcriptional Dynamics of Immortalized Human Mesenchymal Stem Cells during Transformation
Source: PLoS One. 2015 May 15;10(5):e0126562. doi: 10.1371/journal.pone.0126562 (PMC4433180; doi:10.1371/journal.pone.0126562)
Supplement: S1 Table — (PDF) [file pone.0126562.s004.pdf]

Supporting Table S1. Gene expression values in U3-A, U3-B, U3-C and U3-DT cells

Feature ID	Chromosome	Chromosome region start	Chromosome region end	U3-A-Expression value	U3-B-Expression value	U3-C-Expression value	U3-DT-Expression value
LOC100287102	NC_000001	11874	14409	0	0	0	0.0205812
WASH5P	NC_000001	14362	29370	0	0	0	0
FAM138A	NC_000001	34611	36081	0	0	0	0
OR4G4P	NC_000001	52352	53495	0	0	0	0
OR4G11P	NC_000001	62915	63984	0	0	0	0
OR4F5	NC_000001	69091	70008	0	0	0	0.0333604
LOC100132632	NC_000001	87523	90233	0.0162111	0	0	0.022593
LOC100287060	NC_000001	128515	133564	0	0	0	0
LOC653340	NC_000001	133661	139667	0	0	0	0
LOC729737	NC_000001	137848	144331	0.2577609	0.1514316	0.9336243	0.4362153
RPL23AP21	NC_000001	227770	228778	0	0	0	0
LOC100132287	NC_000001	324467	327434	0	0	0.2623998	0.1370241
LOC100288609	NC_000001	327461	329281	0	0	0	0
LOC100288667	NC_000001	327953	333555	0	0	0	0
LOC100288646	NC_000001	329627	342806	0	0	0	0.0327889
OR4F29	NC_000001	367659	368597	0	0	0	0
RPL23AP24	NC_000001	471014	471360	0	0	0	0
LOC100131754	NC_000001	557143	566063	0	0	0	0
LOC100288578	NC_000001	566131	568843	0	0	0	0
LOC100288418	NC_000001	568871	570349	23.801578	19.06318	28.364901	34.496989
LOC100288005	NC_000001	610216	610497	0	0	0	0
OR4F16	NC_000001	621096	622034	0	0	0.0416375	0
LOC100287654	NC_000001	646888	745513	0.3874529	0.2276242	0.4924127	0.8485483
LOC100132630	NC_000001	656153	659632	0	0	0	0
LOC100133060	NC_000001	660025	662259	0	0	0	0
LOC100133331	NC_000001	662667	665155	0	0	0	0
LOC728534	NC_000001	674315	680197	0.4731269	0.3783299	0.9353485	0.989081
LOC100288069	NC_000001	697695	739562	0.1149474	0.0393927	0.170434	0.1601999
LOC100287934	NC_000001	720981	721912	0	0.04848	0	0
LOC100288100	NC_000001	753386	755217	0.5277626	0.5919223	0.6615854	0.8859817
NCRNA00115	NC_000001	761586	762902	0	0	0	0
LOC643837	NC_000001	763064	789740	0	0	0	0
FAM41C	NC_000001	803451	812182	0	0	0	0
LOC388579	NC_000001	808572	809801	0	0	0	0
FLJ39609	NC_000001	852953	854817	0	0	0	0
LOC100128838	NC_000001	855601	856492	0.3448852	0.7598106	0.9642897	1.6479755
SAMD11	NC_000001	861121	879961	0.3441522	3.1667302	0.8725768	1.127149
NOC2L	NC_000001	879583	894679	5.9644029	9.7305683	7.0654886	13.682758
KLHL17	NC_000001	895967	901095	2.1802443	1.6767278	2.229783	3.050525
PLEKHN1	NC_000001	901877	910488	0.3839072	0.3383117	0.1951626	0.3312175
C1orf170	NC_000001	910579	917467	0	0	0	0
HES4	NC_000001	934342	935552	3.0848278	1.2599218	3.7969751	3.0035938
RPL39P12	NC_000001	943454	943609	0	0	0	0
ISG15	NC_000001	948847	949920	344.07446	193.46896	197.30496	6.7034966
AGRN	NC_000001	955503	991492	4.9538586	3.2348822	15.176415	2.4771045
LOC100288175	NC_000001	993748	1004719	0	0	0	0
LOC401934	NC_000001	1006665	1009687	0.0277626	0.0285429	0.0740952	0.0580383
C1orf159	NC_000001	1017198	1051736	1.9634666	2.4696251	3.1404413	4.9197761
TTLL10	NC_000001	1109286	1133313	0.0280819	0.0288712	0.0124912	0.0293529
TNFRSF18	NC_000001	1138888	1142089	0.0366235	0.0753057	0.0325813	0
TNFRSF4	NC_000001	1146706	1149512	0	0.0846131	0.0366082	0
SDF4	NC_000001	1152288	1167447	3.0422537	3.9096957	4.2242926	2.3491488
B3GALT6	NC_000001	1167629	1170421	1.9511567	2.5883796	1.7498015	2.5109549
FAM132A	NC_000001	1177833	1182102	0	0.2180666	0.2264338	0.2069249
UBE2J2	NC_000001	1189292	1209234	9.1070402	12.774462	6.7221502	11.774933
SCNN1D	NC_000001	1217489	1227409	0.425728	0.2693496	0.3641726	0.4564065
ACAP3	NC_000001	1227764	1243269	3.016399	2.6083528	3.7131766	5.8007218
PUSL1	NC_000001	1243994	1247057	3.5442122	6.0123075	3.1214992	4.6925217
CPSF3L	NC_000001	1246965	1260046	9.695371	9.6504128	12.049742	13.913879
GLTPD1	NC_000001	1260143	1264276	1.6833796	2.5136229	3.2804159	3.5191379
TAS1R3	NC_000001	1266726	1269844	0.1030439	0.0882833	0.168063	0.3949281
DVL1	NC_000001	1270658	1284492	5.8617683	7.6953947	8.8651457	12.421719

MXRA8	NC_000001	1288071	1293915	54.041051	83.548543	65.604098	40.406516
LOC100288202	NC_000001	1288801	1290515	0.1131229	0	0.1006372	0.0788285
AURKAIP1	NC_000001	1309110	1310818	19.426115	34.823128	23.04268	29.850579
CCNL2	NC_000001	1321091	1334718	2.8609306	2.5554477	3.9253392	5.9692067
LOC148413	NC_000001	1334910	1337426	0	0	0	0
MRPL20	NC_000001	1337276	1342693	23.083314	41.978031	28.749779	31.920064
TMEM88B	NC_000001	1342863	1363167	0.0600386	0.061726	0.026706	0.0209186
LOC441869	NC_000001	1353800	1356650	0	0	0	0
LOC100288271	NC_000001	1365912	1369894	0	0	0	0
VWA1	NC_000001	1370909	1376146	0.1039375	0.0534293	0	0
ATAD3C	NC_000001	1385069	1405538	0.068331	0.0117086	0.0911837	0.0396798
ATAD3B	NC_000001	1407164	1431582	3.3959176	3.8627816	2.571151	3.8265364
ATAD3A	NC_000001	1447555	1470064	4.4941317	6.0913663	5.2259864	5.7942129
C1orf70	NC_000001	1470158	1475740	0.8655874	0.5663094	0.6650436	0.0822512
SSU72	NC_000001	1477053	1510262	10.017364	12.691577	18.783635	17.980071
LOC100288313	NC_000001	1510010	1510659	0.4185546	0.4303181	0	0
LOC643988	NC_000001	1533390	1535470	0	0	0	0
MIB2	NC_000001	1550884	1565985	1.7747282	1.6574676	1.8319451	2.3884384
MMP23B	NC_000001	1567560	1570030	0.0708842	0.2550676	0.0630606	0.1728824
CDC2L1	NC_000001	1571100	1590473	0.2633884	0.4061864	0.3514764	0.3277491
LOC728661	NC_000001	1592939	1624243	1.1860093	0.880636	2.1037004	3.3619515
LOC100288379	NC_000001	1602957	1606072	0	0	0	0
LOC100287685	NC_000001	1604131	1604782	0.0674053	0.0692997	0.0599656	0.0469707
MMP23A	NC_000001	1631378	1633247	0	0	0	0
CDC2L2	NC_000001	1634169	1655791	0.4013537	0.6017576	0.8926385	1.1653302
SLC35E2	NC_000001	1663681	1677431	0.3995294	0.6319357	0.7655467	0.599648
LOC100129381	NC_000001	1667005	1668928	0	0	0	0
NADK	NC_000001	1682671	1709909	3.3012118	2.9470472	3.7586841	5.689506
GNB1	NC_000001	1716729	1822495	64.306604	76.167544	77.620167	66.507288
CALML6	NC_000001	1846266	1848733	0.11975	0.1231155	0	0.0278155
TMEM52	NC_000001	1849029	1850740	0.045875	0	0.2040583	0.2237726
C1orf222	NC_000001	1853396	1859368	0.0318928	0	0	0.0444483
KIAA1751	NC_000001	1884752	1935276	0	0.0095931	0.008301	0.0325105
GABRD	NC_000001	1950768	1962192	0	0	0	0
PRKCZ	NC_000001	1981909	2116834	0.8036428	1.3770488	0.5467211	0.5051074
LOC100287750	NC_000001	2115806	2118698	0.365778	0.3196495	0.4067579	0.586244
LOC100128003	NC_000001	2120482	2125024	0	0	0	0
C1orf86	NC_000001	2120987	2126214	22.63635	20.239688	19.120316	31.757579
FLJ14100	NC_000001	2144366	2145019	0	0	0	0
SKI	NC_000001	2160134	2241652	4.9284858	6.6108534	3.3637471	3.9334214
MORN1	NC_000001	2252696	2322993	0.6963157	0.6057494	1.1436217	1.2690382
LOC100129534	NC_000001	2281853	2284100	0	0	0	0
RER1	NC_000001	2323214	2336874	13.100637	14.082379	24.864181	27.812895
PEX10	NC_000001	2336241	2344010	4.2592066	5.5771219	5.0709957	5.5963495
PLCH2	NC_000001	2407754	2436964	0.0363434	0.0093412	0.016166	0.0379883
PANK4	NC_000001	2439975	2458035	1.6135422	2.4626835	2.5453374	3.1065357
HES5	NC_000001	2460184	2461684	0	0	0.0598738	0
LOC115110	NC_000001	2481359	2484284	0	0	0	0
LOC100132814	NC_000001	2487208	2488448	0	0	0	0
TNFRSF14	NC_000001	2487805	2495268	7.6722748	2.3822532	4.1914789	4.2519603
LOC100131742	NC_000001	2495677	2496108	0	0	0	0
C1orf93	NC_000001	2518249	2522902	4.8344677	6.0711548	4.4163363	7.2125036
MMEL1	NC_000001	2522081	2564429	0.107943	0.0475615	0.0411553	0.0537279
LOC100287898	NC_000001	2572807	2706230	0.0495191	0	0.110134	0.1207742
ACTRT2	NC_000001	2938046	2939467	0	0	0	0.0646094
FLJ42875	NC_000001	2976181	2984289	0	0	0	0
PRDM16	NC_000001	2985744	3355185	0.0252055	0.1088382	0.0224235	0.0281027
LOC100288479	NC_000001	3369079	3380141	0.0545263	0.0560588	0	0
ARHGEF16	NC_000001	3371147	3397677	0.0605348	0.0933541	0.0942434	0.0527288
MEGF6	NC_000001	3404506	3528059	3.5683199	2.125849	2.7435044	4.5325973
TPRG1L	NC_000001	3541556	3546695	12.706614	15.315461	11.738931	13.231259
WDR8	NC_000001	3547331	3566671	2.9229842	3.5104232	4.3262757	5.9303031
TP73	NC_000001	3569129	3650467	0.042285	0.2173672	0.0250786	0.0491098
KIAA0495	NC_000001	3652548	3663886	1.5068755	1.6700377	1.6111296	1.6136103
CCDC27	NC_000001	3668965	3688209	0	0.0207835	0.0179842	0.0281738
LOC388588	NC_000001	3689352	3692520	0	0	0	0

LRRC47	NC_000001	3696784	3713068	4.5641102	6.3645804	5.8026221	5.6438597
KIAA0562	NC_000001	3731344	3773797	1.3562104	1.839069	1.1753192	2.3382123
DFFB	NC_000001	3773845	3801993	0.2467371	0.4028903	0.4390083	0.586606
C1orf174	NC_000001	3805697	3816857	10.103345	12.855302	21.590492	21.856531
LOC100133612	NC_000001	3816968	3832011	0	0	0	0
LOC728716	NC_000001	4000631	4005522	0	0	0	0.1107591
LOC644357	NC_000001	4235523	4235959	0	0	0	0
LOC284661	NC_000001	4472111	4484744	0	0	0	0
AJAP1	NC_000001	4715105	4843851	0.066247	0.0953524	0.0825092	0.2769811
LOC100287848	NC_000001	4715321	4772769	0	0	0	0.0665035
LOC100287877	NC_000001	5768213	5812682	0	0	0	0.0554296
NPHP4	NC_000001	5922870	6052531	1.2388446	1.1471995	2.7670008	4.2612785
KCNAB2	NC_000001	6086380	6160523	3.0906514	2.4935652	5.338772	4.1142221
CHD5	NC_000001	6161853	6240183	0.0182244	0.0093683	0.016213	0.0380985
LOC100287356	NC_000001	6237172	6238508	0	0	0	0
RPL22	NC_000001	6245080	6259679	6.7239741	8.4299009	15.414004	16.979549
RNF207	NC_000001	6266189	6281359	0.3984227	0.5916739	0.1772239	0.1619548
ICMT	NC_000001	6281253	6296044	11.308797	16.224736	13.852637	16.918417
C1orf211	NC_000001	6298694	6298984	0	0	0	0
HES3	NC_000001	6304262	6305638	0	0.0657691	0	0.0445777
GPR153	NC_000001	6308856	6321035	0.7013016	0.4806745	0.5496238	0.5585084
ACOT7	NC_000001	6324332	6453826	2.4466785	3.2341405	4.3205347	6.2172733
LOC100130071	NC_000001	6326515	6327225	0	0	0	0
HES2	NC_000001	6475292	6479979	0.0206233	0.0106015	0	0.0646701
ESPN	NC_000001	6484848	6521004	0.0248928	0.0127962	0.0110727	0.0173463
TNFRSF25	NC_000001	6521211	6526255	0.5275623	0.3301501	0.3673049	0.1918051
PLEKHG5	NC_000001	6526152	6580069	1.0588059	1.648854	0.7549397	0.5587887
NOL9	NC_000001	6585205	6614581	2.3391551	3.9019857	2.6322295	4.1452074
TAS1R1	NC_000001	6615434	6639817	0.1465484	0.0502224	0.0724297	0.045387
ZBTB48	NC_000001	6640063	6649340	1.0338549	1.0242602	1.1705858	1.3229738
KLHL21	NC_000001	6650784	6662929	14.417606	15.165189	10.708916	19.684009
PHF13	NC_000001	6673756	6684093	3.9756984	3.9150741	6.7967975	6.8509605
THAP3	NC_000001	6685252	6695645	2.4415684	2.7524002	3.1819169	4.7011875
DNAJC11	NC_000001	6694228	6761966	4.2707058	5.3786497	3.4668962	8.2955941
CAMTA1	NC_000001	6845384	7829764	0.1665889	0.2033842	0.4307123	0.3591404
RPL37P9	NC_000001	6894388	6894660	0	0	0	0
VAMP3	NC_000001	7831329	7841492	25.551761	29.876372	36.118703	40.541506
PER3	NC_000001	7844763	7905237	0.6943296	0.5463091	4.8785291	5.0457239
UTS2	NC_000001	7907672	7913551	0.0582096	0.0598456	0	0.1216883
TNFRSF9	NC_000001	7979907	8000887	0.1866167	0.0719481	0.4980582	0.0975315
PARK7	NC_000001	8021714	8045342	3.5213464	5.5009969	6.79427	12.747088
ERRFI1	NC_000001	8071779	8086393	18.497866	4.577545	16.606157	12.743238
RPL7AP18	NC_000001	8117241	8118547	0	0	0	0
LOC100129776	NC_000001	8242540	8258035	0	0	0	0
SLC45A1	NC_000001	8384390	8404227	0.4759075	0.2258229	0.8793288	0.739793
RERE	NC_000001	8412464	8877699	3.8502897	2.6987322	6.1176578	6.4665198
RPL7P11	NC_000001	8810469	8811150	0	0	0	0
RPL7P7	NC_000001	8846188	8847010	0	0	0	0
RPL27P3	NC_000001	8858497	8859616	0	0	0	0
RPL23AP19	NC_000001	8891066	8891437	0	0	0	0
ENO1	NC_000001	8921063	8938780	524.61383	521.92814	784.82106	1059.4484
LOC100113373	NC_000001	8953470	8954662	0	0	0	0
CA6	NC_000001	9005922	9035151	0	0	0	0.0686144
SLC2A7	NC_000001	9063359	9086404	0.0571127	0	0.0254045	0.0596976
SLC2A5	NC_000001	9097005	9148510	9.0821215	0.2057388	3.0949386	0.8045064
GPR157	NC_000001	9164476	9189229	0.6860956	0.9405045	0.4069131	0.8765143
LOC727721	NC_000001	9241898	9257040	0	0	0	0
H6PD	NC_000001	9294863	9331396	1.5859349	1.1894282	4.8888039	3.8092149
SPSB1	NC_000001	9352941	9429591	6.131328	6.0573002	5.7053856	4.4493489
SLC25A33	NC_000001	9599528	9642831	3.8503584	3.8039414	5.8873815	3.9198167
RPL9P11	NC_000001	9636463	9637139	0	0	0	0
TMEM201	NC_000001	9648977	9674935	2.0493639	2.3324557	2.9268296	2.5791382
PIK3CD	NC_000001	9711790	9789172	0.5847852	1.6951082	1.0188055	2.0544873
C1orf200	NC_000001	9712668	9714644	0	0	0	0
RPL26P7	NC_000001	9722346	9722686	0	0	0	0
CLSTN1	NC_000001	9789079	9884550	14.660697	18.440606	17.477937	23.104324

FLJ16126	NC_000001	9887909	9888292	0	0	0	0
CTNNBIP1	NC_000001	9908334	9970316	1.3793435	2.443977	2.480313	2.8119671
LZIC	NC_000001	9989776	10002826	4.7477834	6.3263185	5.2519118	5.5503511
NMNAT1	NC_000001	10003486	10045556	0.7322768	1.2308623	0.8169023	1.052958
RBP7	NC_000001	10057255	10076078	0.4696758	0.0689823	0.2387638	0.0935111
UBE4B	NC_000001	10093016	10241297	2.686759	4.3767834	4.1183211	10.237682
RPL21P21	NC_000001	10114498	10114840	0	0	0	0
KIF1B	NC_000001	10270764	10441661	2.4696184	3.5659228	3.9766443	4.527954
LOC100287506	NC_000001	10457024	10458956	0	0.0467495	0.0202264	0
PGD	NC_000001	10459085	10480201	47.396931	37.112438	75.914272	50.308912
APITD1	NC_000001	10490159	10512210	1.1842338	1.7755454	1.1413212	1.7650566
CORT	NC_000001	10509971	10511959	0	0	0	0
DFFA	NC_000001	10520588	10532613	4.1910726	6.7350846	4.462721	6.8923948
PEX14	NC_000001	10535003	10690815	3.3543043	4.3400464	5.7245657	5.4380625
CASZ1	NC_000001	10696661	10856707	0.1185561	0.0158985	0.0962994	0.474136
C1orf127	NC_000001	11006533	11024258	0	0	0.0172845	0.0270777
LOC390996	NC_000001	11025183	11051854	0	0	0	0
TARDBP	NC_000001	11072679	11085549	7.3055989	10.093138	12.293899	19.019814
MASP2	NC_000001	11086580	11107285	0.2535475	0.4170776	0.4511258	0.4947096
SRM	NC_000001	11114649	11120091	10.736764	22.325499	15.417893	40.223721
EXOSC10	NC_000001	11126675	11159938	9.0776262	11.939489	13.56162	11.298922
LOC100128221	NC_000001	11158998	11162108	0.2881851	0.3456654	0.0854592	0.535517
FRAP1	NC_000001	11166588	11322608	2.7351163	5.2977215	3.5490283	5.0368711
ANGPTL7	NC_000001	11249398	11256038	0	0	0	0.0273681
RPL39P6	NC_000001	11292967	11293367	0	0	0	0
UBIAD1	NC_000001	11333255	11348491	2.4535959	2.4607271	2.9531823	3.8637299
PTCHD2	NC_000001	11539295	11597640	0.07586	0.0173316	0.0299943	0.0293679
FBXO2	NC_000001	11708448	11714739	0.1430607	0.0882488	0.2545414	0.0398761
FBXO44	NC_000001	11714432	11723384	1.4828011	1.1401539	2.4720037	3.3603141
FBXO6	NC_000001	11724150	11734411	6.5454813	5.9428846	9.1757021	3.19873
MAD2L2	NC_000001	11734537	11751678	10.424468	19.155313	28.498828	21.677248
C1orf187	NC_000001	11751781	11780336	0.0247875	0.0254842	0.0220516	0.0345458
AGTRAP	NC_000001	11796142	11810828	20.429061	16.62608	30.117344	24.643457
LOC100288041	NC_000001	11821844	11838367	0.0107743	0.0110771	0.0287553	0.0300318
C1orf167	NC_000001	11838543	11849984	0.0343078	0	0.0610423	0.1075815
MTHFR	NC_000001	11845787	11866115	2.4680288	1.5262514	3.0540674	2.4741281
CLCN6	NC_000001	11866207	11903201	0.7384298	0.6153382	1.6319465	2.6324179
NPPA	NC_000001	11905776	11907835	0.156958	0.0537898	0.4654472	0.6927056
NPPB	NC_000001	11917521	11918992	0	0.1294653	0	0
LOC390997	NC_000001	11937776	11940470	0	0	0	0
RNU5E	NC_000001	11968211	11968329	0	0	0	0
KIAA2013	NC_000001	11980122	11986480	13.948542	17.139197	17.87031	15.946914
PLOD1	NC_000001	11994746	12035594	71.218373	49.409945	98.017233	68.916171
MFN2	NC_000001	12040238	12073572	19.601456	27.108109	20.397276	32.838094
MIIP	NC_000001	12079512	12092106	2.7951961	2.5607721	4.7764029	6.3062564
TNFRSF8	NC_000001	12123434	12204264	0.2823227	0.4595742	0.3662781	0.1065641
TNFRSF1B	NC_000001	12227060	12269277	9.12449	2.4097814	17.713591	12.791616
LOC390998	NC_000001	12280849	12290110	0.097016	0.0997426	0	0
VPS13D	NC_000001	12290113	12572099	2.0939264	2.645314	3.72563	5.9978174
LOC100287581	NC_000001	12556354	12558314	0	0	0	0
SNORA59A	NC_000001	12567300	12567451	0	0	0	0
DHRS3	NC_000001	12627939	12677820	3.2121631	3.5776448	3.8967673	6.2911572
AADACL4	NC_000001	12704566	12727097	0	0	0	0.0194444
AADACL3	NC_000001	12776118	12788726	0	0.0223183	0.0096561	0.0151271
C1orf158	NC_000001	12806163	12821102	0.0862576	0.0443409	0.0383686	0.0601077
PRAMEF12	NC_000001	12834984	12838049	0	0.0505973	0.0218911	0
PRAMEF1	NC_000001	12851546	12856223	0	0	0.0243447	0.019069
PRAMEF11	NC_000001	12884468	12893276	0.0234641	0	0.0417486	0.0327014
LOC441870	NC_000001	12897387	12901210	0	0	0	0
HNRNPCL1	NC_000001	12907261	12908578	0	0.0815585	0	0.1381989
PRAMEF2	NC_000001	12916941	12921764	0.0267651	0	0	0
PRAMEF4	NC_000001	12939033	12946025	0	0.0245562	0	0
PRAMEF10	NC_000001	12952727	12958094	0	0	0	0.0201745
PRAMEF7	NC_000001	12977513	12980233	0	0	0	0.0214911
LOC729356	NC_000001	12986916	12988081	0	0	0	0
PRAMEF6	NC_000001	12998302	13007406	0	0.0262694	0	0

LOC100129611	NC_000001	13012915	13015755	0	0	0	0
PRAMEF22	NC_000001	13035543	13038381	0.030393	0	0	0
PRAMEF23	NC_000001	13108514	13117745	0	0	0	0
LOC100132865	NC_000001	13123246	13126086	0	0	0	0
PRAMEF25	NC_000001	13140456	13145000	0	0	0.0274369	0
LOC649324	NC_000001	13162645	13165443	0	0	0	0
LOC440563	NC_000001	13182960	13183967	0	0.0448248	0.0775745	0.0911455
LOC645354	NC_000001	13196270	13199067	0	0	0	0
LOC645359	NC_000001	13216688	13219694	0	0	0	0
PRAMEF3	NC_000001	13328196	13331692	0	0	0	0.0145556
LOC100132443	NC_000001	13351482	13354323	0	0	0	0
PRAMEF5	NC_000001	13359819	13369057	0	0	0	0
LOC650236	NC_000001	13379146	13380311	0	0	0	0
PRAMEF8	NC_000001	13386646	13390765	0	0	0.0212026	0.0166079
LOC645382	NC_000001	13411551	13414482	0	0	0	0
PRAMEF9	NC_000001	13421176	13428191	0	0	0	0.0328769
PRAMEF13	NC_000001	13447414	13452656	0	0	0	0.014152
PRAMEF18	NC_000001	13474053	13477569	0	0	0.0184162	0.0288506
PRAMEF16	NC_000001	13495254	13498260	0	0	0	0
PRAMEF21	NC_000001	13521963	13526943	0	0	0	0
LOC729516	NC_000001	13607431	13611550	0	0	0	0.0166079
LOC645399	NC_000001	13632348	13635279	0	0	0	0
PRAMEF15	NC_000001	13641973	13648987	0	0.0242661	0	0.0164473
PRAMEF14	NC_000001	13668269	13673511	0	0	0	0
PRAMEF19	NC_000001	13694889	13698405	0	0	0	0
PRAMEF17	NC_000001	13716088	13719064	0	0	0	0
PRAMEF20	NC_000001	13736907	13747803	0	0	0.0489638	0
LRRC38	NC_000001	13801189	13843853	0.0186775	0.0768099	0	0.0650762
PDPN	NC_000001	13910252	13944452	0.4424319	0.0606489	0.01312	0.0102768
PRDM2	NC_000001	14026735	14151574	0.8071469	0.7921122	1.7339524	3.1286399
KIAA1026	NC_000001	14925213	15444544	1.0320891	0.4823164	0.4298729	1.0493794
TBCAP2	NC_000001	15018325	15018992	0	0	0	0
C1orf126	NC_000001	15438311	15478960	0	0	0	0
TMEM51	NC_000001	15479028	15546974	0.9229521	2.4832701	4.0879492	5.1725666
FHAD1	NC_000001	15573768	15724622	0.0777081	0	0.06145	0.0541501
EFHD2	NC_000001	15736441	15756835	18.842921	11.520335	19.904215	28.760192
CTRC	NC_000001	15764938	15773153	0	0	0.0869801	0
ELA2A	NC_000001	15783224	15798583	0	0.0493269	0.0426829	0
ELA2B	NC_000001	15802596	15817895	0	0	0.0847185	0
CASP9	NC_000001	15818791	15850790	1.6979497	2.0630655	1.9421269	3.4727659
DNAJC16	NC_000001	15853352	15898228	0.79508	1.0499048	1.1745493	1.4232309
AGMAT	NC_000001	15899152	15911605	1.0606814	0.5966843	0.053412	0.055783
LOC645317	NC_000001	15930788	15931813	4.1830245	8.492302	7.7253021	6.0511808
CD24L1	NC_000001	15936477	15941441	0	0	0	0
DDI2	NC_000001	15944070	15986748	2.0737369	1.464158	2.5338957	4.6311641
RSC1A1	NC_000001	15986364	15988217	1.5645002	2.4858182	2.2353516	3.9809038
PLEKHM2	NC_000001	16010827	16061264	19.735123	18.437283	22.043363	24.681185
LOC100288579	NC_000001	16060438	16061264	0	0.0546353	0.1418291	0.1481252
SLC25A34	NC_000001	16062809	16067884	0.1431538	0.0588709	0.2547073	0.1396574
TMEM82	NC_000001	16068917	16074477	0	0	0	0.0201479
FBLIM1	NC_000001	16085255	16113084	2.8770775	1.987926	4.3418712	1.8425251
RPL12P14	NC_000001	16119222	16119832	0	0	0	0
UQCRHL	NC_000001	16133657	16134194	0	0.167968	0	0.0569236
RPS16P1	NC_000001	16154707	16155228	0	0	0	0
LOC100288612	NC_000001	16160560	16162958	0.0366388	0.0941713	0.0814872	0.0510627
FLJ37453	NC_000001	16160710	16174642	0	0	0	0
SPEN	NC_000001	16174359	16266950	0.2444164	0.3843194	0.4668557	0.831558
ZBTB17	NC_000001	16268364	16302627	3.5944573	4.204635	4.3631235	6.1004847
LOC100129063	NC_000001	16316612	16320416	0	0	0	0
C1orf64	NC_000001	16330731	16333180	0	0.0492731	0.0426364	0
HSPB7	NC_000001	16340523	16345285	11.442952	23.59161	3.4744379	10.49421
CLCNKA	NC_000001	16348486	16360545	0.0346595	0.0178168	0.030834	0.0362282
LOC440568	NC_000001	16361005	16366526	0	0	0	0
CLCNKB	NC_000001	16370247	16383803	0.0678213	0.0174319	0	0.0236303
FAM131C	NC_000001	16384264	16400127	0.3617138	0.2656285	1.8847739	1.3683073
EPHA2	NC_000001	16450832	16482564	7.2838681	5.496207	12.097853	9.7400462

ARHGEF19	NC_000001	16524599	16539104	1.9152085	3.2220584	2.7880733	3.1039411
LOC645634	NC_000001	16542874	16552794	1.3317646	0.6389572	0.3949249	0.1237369
C1orf89	NC_000001	16558195	16563446	0.8236175	0.7790242	0.9378726	1.1249018
LOC100287973	NC_000001	16558195	16559516	0.1518948	0.1041092	0.0450433	0
LOC645652	NC_000001	16567708	16567893	0	0	0	0
FBXO42	NC_000001	16576559	16678948	1.6982684	1.9433721	3.2449928	4.4352562
C1orf144	NC_000001	16693583	16724640	16.398955	18.289704	28.037495	34.04637
RPL22P3	NC_000001	16695588	16696046	0	0	0	0
SPATA21	NC_000001	16725138	16763919	0.0436211	0.044847	0.0582098	0.0455954
NECAP2	NC_000001	16767167	16786585	14.902715	16.03772	19.623898	14.959415
CROCCL2	NC_000001	16793931	16819196	0	0	0	0
RNU1A3	NC_000001	16840618	16840780	0	0	0	0
LOC645685	NC_000001	16847223	16852283	0.0665882	0.0684597	0.0592387	0.3248093
LOC100288669	NC_000001	16860723	16861614	0	0	0	0
LOC100132147	NC_000001	16861669	16862667	0	0	0	0
LOC100133301	NC_000001	16865561	16866070	0.086173	0	0	0
TRNAV35P	NC_000001	16874160	16874232	0	0	0	0
NBPF1	NC_000001	16890412	16939982	4.0297842	4.7755672	2.9099681	4.3157785
CROCCL1	NC_000001	16944751	16957401	0	0	0	0
MSTP2	NC_000001	16972008	16976771	0	0	0	0
RNU1C2	NC_000001	16993280	16993443	0	0	0	0
LOC729574	NC_000001	16999498	17000007	0	0	0	0.0600488
EIF1AP1	NC_000001	17011046	17012718	0	0	0	0
ESPNP	NC_000001	17017713	17046652	0	0	0	0
TRNAV36P	NC_000001	17052061	17052133	0	0	0	0
LOC729587	NC_000001	17060447	17060956	0	0	0	0
RNU1A	NC_000001	17067011	17067174	0	0	0	0
MSTP9	NC_000001	17081401	17090975	0	0	0	0
TRNAQ49P	NC_000001	17180900	17180971	0	0	0	0
TRNAV34P	NC_000001	17186693	17186765	0	0	0	0
LOC100132070	NC_000001	17194855	17195363	0	0	0	0
LOC100288921	NC_000001	17196254	17198646	0.0367307	0	0.0326766	0.0383931
LOC440570	NC_000001	17198068	17199968	0	0	0	0
LOC100288891	NC_000001	17198766	17200539	0.1973301	0.0289823	0.1003145	0.0392878
LOC100129182	NC_000001	17215339	17216108	0	0	0	0
RNU1C1	NC_000001	17222475	17222638	0	0	0	0
CROCC	NC_000001	17248445	17299474	0.1914812	0.1221907	0.1468508	0.2530601
MFAP2	NC_000001	17300997	17308081	5.456158	15.752717	12.035135	10.416625
ATP13A2	NC_000001	17312453	17338423	2.562547	3.7087477	1.7415833	2.6823592
SDHB	NC_000001	17345217	17380665	22.67927	35.425668	38.114192	27.251626
PADI2	NC_000001	17393256	17445948	0.0503647	0.0517802	0.0448058	0.063173
LOC400743	NC_000001	17520821	17521393	0	0	0	0.0534466
PADI1	NC_000001	17531621	17572501	0.0913922	0.0117451	0.0406525	0.055725
PADI3	NC_000001	17575593	17610728	0.0413436	0.028337	0.0980811	0.0576197
PADI4	NC_000001	17634690	17690495	0.0582096	0.0797941	0.1035697	0.0540837
PADI6	NC_000001	17698741	17728195	0.0561998	0	0.0166656	0.0130541
RCC2	NC_000001	17733251	17766220	1.904317	4.1672419	3.4923751	5.0337188
ARHGEF10L	NC_000001	17866330	18024370	10.903934	3.8708522	9.6395422	10.344585
ACTL8	NC_000001	18149477	18153558	0	0.0810707	0.0233837	0.0732653
IGSF21	NC_000001	18434240	18704976	0	0.0465089	0	0.0157616
KLHDC7A	NC_000001	18807424	18812493	0.0346732	0.0267357	0.0385578	0.030202
LOC391012	NC_000001	18839519	18839889	0	0	0	0
PAX7	NC_000001	18957500	19075360	0.0417693	0.1359868	0.068125	0.0776173
TAS1R2	NC_000001	19166093	19186155	0.0697314	0.0537684	0.0155088	0.0242958
ALDH4A1	NC_000001	19197926	19229072	1.3490438	1.8445123	0.6928683	0.4458052
IFFO2	NC_000001	19230774	19282826	0.4519098	0.7820949	0.6030473	0.4933571
UBR4	NC_000001	19401000	19536746	8.3402576	10.953035	18.374526	27.47986
KIAA0090	NC_000001	19544584	19578046	5.5376016	8.0174418	9.077868	8.960559
MRTO4	NC_000001	19578075	19586622	6.7092109	10.555383	9.2712661	12.22031
AKR7L	NC_000001	19592476	19600568	0.3038771	0.195261	0.4561946	0.3838036
AKR7A3	NC_000001	19609057	19615280	0.2478949	0.1456355	0.283544	0.4441965
AKR7A2	NC_000001	19630459	19638640	11.680808	12.539887	12.343578	13.715988
PQLC2	NC_000001	19638740	19655794	6.0806423	7.8344613	5.2534645	6.6274681
CAPZB	NC_000001	19665273	19746244	58.812662	73.960068	75.525419	85.288429
LOC100288987	NC_000001	19677793	19680717	0	0	0	0
LOC644083	NC_000001	19726545	19727024	0	0	0	0

C1orf151	NC_000001	19923467	19955174	0.9505991	0.9773158	1.6611557	1.9162728
RPS14P3	NC_000001	19934573	19935110	0	0	0	0
NBL1	NC_000001	19969726	19984945	47.846703	16.527993	33.841651	49.33308
LOC100289050	NC_000001	19981813	19983834	0.073492	0.1511151	0.0653805	0
HTR6	NC_000001	19991780	20006055	0.066454	0.0227739	0.0394129	0.0308719
TMCO4	NC_000001	20008706	20126410	5.4233359	7.4140888	4.6538461	6.8272678
RNF186	NC_000001	20140522	20141771	0	0	0	0.0244999
OTUD3	NC_000001	20208888	20239438	0.4654533	0.9431992	0.7321417	1.1375626
PLA2G2E	NC_000001	20246800	20250110	0	0	0	0
PLA2G2A	NC_000001	20301931	20306909	0	0	0.0403068	0.0947161
PLA2G5	NC_000001	20396701	20418394	0.1160196	0.0238561	0.0206429	0.0161694
PLA2G2D	NC_000001	20438432	20446008	0.0167104	0	0	0.0116444
PLA2G2F	NC_000001	20465823	20476879	0.0323268	0.0166177	0	0.0112633
PLA2G2C	NC_000001	20490484	20501687	0	0	0	0
UBXN10	NC_000001	20512578	20519942	0	0.015203	0	0.0412179
VWA5B1	NC_000001	20617412	20681387	0.0196417	0.0807748	0.0349475	0.0547484
CAMK2N1	NC_000001	20808884	20812728	1.2337658	6.0731412	1.4967167	0.7946055
MUL1	NC_000001	20825941	20834674	9.3689477	13.235171	9.177907	12.097948
RPS4P4	NC_000001	20852445	20853303	0	0	0	0
FAM43B	NC_000001	20878932	20881513	0.034042	0.227492	0.1211389	0.0593046
CDA	NC_000001	20915444	20945401	1.0987058	7.577633	0.1629065	0.3190091
PINK1	NC_000001	20959948	20978004	4.3783013	6.8114828	4.1449299	12.83712
DDOST	NC_000001	20978260	20988037	71.627763	83.693432	120.40064	81.978449
KIF17	NC_000001	20990507	21044317	0.1861227	0.3229094	0.9934797	0.8592475
SH2D5	NC_000001	21046225	21059133	2.6364354	2.2862754	3.2530317	7.5483855
HP1BP3	NC_000001	21069171	21113181	18.482692	22.470705	24.100693	24.954472
EIF4G3	NC_000001	21132972	21503340	5.341499	5.2283755	8.4029078	9.3624203
RPS15AP6	NC_000001	21330004	21330457	0	0	0	0
ECE1	NC_000001	21543740	21672034	10.349322	18.445098	23.84563	18.651442
LOC100133291	NC_000001	21698905	21724860	0	0	0	0
NBPF2P	NC_000001	21749601	21754453	0	0	0	0
HS6ST1P	NC_000001	21754761	21758727	0	0	0	0
LOC100289081	NC_000001	21755957	21758730	0.0316858	0	0.0422829	0
NBPF3	NC_000001	21766631	21811393	2.8124068	3.0714155	2.1697799	3.4235459
LOC767853	NC_000001	21785917	21786695	0	0	0	0
ALPL	NC_000001	21835858	21904905	2.9990494	3.2000049	0.0432655	0.4066749
RAP1GAP	NC_000001	21922708	21995856	0.1955427	0.0628245	0.0869801	0.0255491
USP48	NC_000001	22004791	22109688	0.5843955	0.6704802	1.1528093	1.8295842
LDLRAD2	NC_000001	22138758	22151714	0.0438168	0.1013586	0.1949031	0.1373998
HSPG2	NC_000001	22148737	22222804	12.206121	10.338737	30.741171	13.177376
RPL21P29	NC_000001	22234551	22235107	0	0	0	0
CELA3B	NC_000001	22303418	22315847	0	0.0450483	0	0.0610666
CELA3A	NC_000001	22328149	22339035	0	0	0	0
HSPC157	NC_000001	22351707	22357716	0	0	0	0
CDC42	NC_000001	22379120	22419436	9.8761637	15.022944	17.188058	19.170883
LOC100289113	NC_000001	22426523	22446930	0	0.0602445	0	0.0816663
WNT4	NC_000001	22443798	22469519	0.0787804	0.0809946	0.0300365	0.1725345
LOC343384	NC_000001	22649358	22649824	0	0	0	0
ZBTB40	NC_000001	22778344	22857650	0.8699717	1.2461614	1.3348479	1.9821707
EPHA8	NC_000001	22890004	22930087	0.024366	0.0501017	0	0.0282987
C1QA	NC_000001	22963118	22966175	0	0.0411506	0.071216	0.055783
C1QC	NC_000001	22970118	22974603	0.1476011	0.0758747	0.0328275	0.0257136
C1QB	NC_000001	22979682	22988029	0	0	0.0374498	0
EPHB2	NC_000001	23037331	23241823	0.3520191	0.4268713	2.8104637	1.0755503
LOC646262	NC_000001	23279536	23289671	0	0	0.0503186	0
LOC729059	NC_000001	23337327	23340567	0	0	0	0
KDM1	NC_000001	23345946	23410184	3.8146485	7.6051271	7.6904783	9.9252906
LUZP1	NC_000001	23410516	23495351	2.9568093	3.4338747	3.1464166	4.5646838
LOC100127936	NC_000001	23466881	23467644	0	0	0	0
HTR1D	NC_000001	23518388	23521222	0.031004	0.382505	0	0.0648146
RPL29P6	NC_000001	23571222	23571838	0	0	0	0
HNRNPR	NC_000001	23636276	23670853	15.344202	26.127578	22.865158	36.55768
ZNF436	NC_000001	23685941	23696357	2.3005722	3.1442542	3.0862225	2.9327106
C1orf213	NC_000001	23695464	23698279	0.1404595	0.1283619	0.4165224	0.7503965
TCEA3	NC_000001	23707555	23751261	1.4963458	0.5128003	4.7331227	1.9502602
LOC100289176	NC_000001	23737325	23738645	0	0	0	0

ASAP3	NC_000001	23755056	23810750	0.9843896	0.8488211	0.8286575	0.8113527
E2F2	NC_000001	23832922	23857713	0.6263808	0.6091753	0.9187024	1.6633697
ID3	NC_000001	23884409	23886322	21.530539	40.938688	8.165563	36.545371
MDS2	NC_000001	23953824	23967056	0	0	0	0
LOC729856	NC_000001	23997268	23997735	0.0939065	0	0	0
RPL11	NC_000001	24018294	24022915	174.92695	237.63947	268.93219	359.70403
TCEB3	NC_000001	24069856	24088549	3.6364206	4.3144984	6.4147533	9.2872127
C1orf128	NC_000001	24104876	24114722	5.5544929	6.0727384	11.377343	13.273298
LYPLA2	NC_000001	24117646	24122029	4.0863196	3.7560094	5.8261151	10.315153
GALE	NC_000001	24122089	24127294	9.224871	7.5629914	12.688822	17.718915
HMGCL	NC_000001	24128367	24151949	9.2925366	6.6032957	8.8747584	8.3037605
FUCA1	NC_000001	24171567	24194821	4.2809458	1.5176766	5.6282484	3.8354574
LOC100289209	NC_000001	24191990	24194780	0	0.0961349	0.2495589	0
CNR2	NC_000001	24200460	24239817	0	0	0.0220268	0.0172535
PNRC2	NC_000001	24286301	24289952	2.4001517	3.3836143	6.745422	7.4503221
FUSIP1	NC_000001	24292939	24306821	5.5785681	5.6600308	6.3781454	10.02839
RPL36P5	NC_000001	24334371	24335009	0	0	0	0
MYOM3	NC_000001	24382531	24438665	0.1212365	0.2414975	0.0471867	0.0528015
IL22RA1	NC_000001	24446261	24469611	0.1099491	0.1130392	0.4750955	0.2189055
IL28RA	NC_000001	24480647	24513751	0.0772886	0.4271018	0.4125485	0.0673222
LOC284632	NC_000001	24526730	24538178	0	0	0	0
GRHL3	NC_000001	24645881	24681808	0.0305408	0.0156996	0.02717	0.0212821
C1orf201	NC_000001	24683489	24727949	2.530406	2.5653911	5.9873521	5.5592542
NIPAL3	NC_000001	24742245	24799473	3.2236263	6.7450342	4.4386473	9.86024
RCAN3	NC_000001	24829387	24862428	1.034076	3.3754659	1.5409041	1.1169073
RPL26P8	NC_000001	24890088	24890462	0	0	0	0
C1orf130	NC_000001	24921910	24935816	0.1803467	0.2085923	0.1002759	0.5733819
SRRM1	NC_000001	24969594	24999772	2.1225633	2.5152936	2.5044704	3.4875306
CLIC4	NC_000001	25071760	25170815	109.10738	77.190859	143.2872	183.00333
RUNX3	NC_000001	25226002	25291501	0.0368771	0.0094784	0.0246051	0.0385461
LOC391020	NC_000001	25451384	25451948	0	0	0	0
SYF2	NC_000001	25548764	25559013	0.8903412	0.9407911	1.2101104	1.6717013
C1orf63	NC_000001	25568740	25573985	2.1599131	3.1150332	4.6703577	5.6232712
RHD	NC_000001	25598981	25656936	0.0775648	0.1116427	0.3036168	0.3675418
SDHDP6	NC_000001	25619853	25621144	0	0	0	0
TMEM50A	NC_000001	25664811	25688852	37.972814	42.983684	58.423424	57.367919
RHCE	NC_000001	25688740	25747363	0.2172966	0.1396273	0.2899696	0.1514209
SDHDP7	NC_000001	25725202	25726491	0	0	0	0
LOC100288240	NC_000001	25735208	25747339	0	0	0	0
TMEM57	NC_000001	25757388	25826700	4.5844475	5.7339343	6.2119482	6.2882047
LDLRAP1	NC_000001	25870076	25895377	7.5318435	6.1270848	8.7519934	14.900281
MAN1C1	NC_000001	25943959	26111258	3.2950877	1.1998092	2.2229794	1.2246124
SEPN1	NC_000001	26126667	26144713	8.3696425	6.2685191	17.61737	22.205158
FAM54B	NC_000001	26146397	26159433	8.5409173	7.9912384	8.1188869	10.998448
LOC646471	NC_000001	26146445	26150097	0	0	0	0
C1orf135	NC_000001	26160497	26185848	1.6845756	2.5430736	2.4850951	2.8678319
LOC100289250	NC_000001	26186104	26190330	0.3119354	0.4988704	0.6166808	1.086845
PAQR7	NC_000001	26187975	26197744	4.012475	3.9458876	4.1257438	4.7208711
STMN1	NC_000001	26210677	26233368	11.322587	15.604706	23.912253	19.453004
LOC100288272	NC_000001	26213779	26225569	0	0	0	0
RPL39P7	NC_000001	26215478	26215600	0	0	0	0
PAFAH2	NC_000001	26286258	26324648	0.9695365	1.1229608	1.1573141	1.6847531
EXTL1	NC_000001	26348271	26362955	0.0985141	0.0337609	0.0097379	0.0381381
SLC30A2	NC_000001	26364514	26372604	0.0177712	0.0365414	0	0.0247674
TRIM63	NC_000001	26377798	26394121	0	0.0256142	0	0.017361
PDIK1L	NC_000001	26437656	26452034	0.4449917	0.6644618	0.6126668	0.9745622
GRRP1	NC_000001	26485511	26489119	0.0288374	0.0889437	0	0.0401901
ZNF593	NC_000001	26496388	26497364	2.9795411	6.126563	3.7952953	3.5862723
LOC100128346	NC_000001	26496438	26498152	0.1348105	0.2078991	0.2398624	0.2348534
CNKSR1	NC_000001	26503981	26516375	0.0346595	0	0.015417	0.0483042
CATSPER4	NC_000001	26517119	26529033	0.0309713	0	0	0.021582
LOC100133116	NC_000001	26536876	26554337	0	0	0	0
CCDC21	NC_000001	26560693	26605299	1.5971022	2.790233	4.3022226	5.0976608
MRPS6P1	NC_000001	26589526	26589889	0	0	0	0
SH3BGRL3	NC_000001	26606213	26608013	182.84829	231.42817	163.85912	242.22493
UBXN11	NC_000001	26608773	26644756	2.4392117	2.3769261	3.2644203	4.2863005

CD52	NC_000001	26644411	26647014	0	0.1789442	0	0
AIM1L	NC_000001	26648350	26670443	0.0367768	0.0378104	0.0654353	0.0384413
RPL34P4	NC_000001	26653178	26653530	0	0	0	0
ZNF683	NC_000001	26688125	26699266	0	0	0	0
LIN28	NC_000001	26737269	26756219	0.0547437	0.0225129	0.0389612	0.0534066
DHDDS	NC_000001	26758802	26797797	11.050298	9.745708	9.0963134	12.172021
RPL17P9	NC_000001	26781094	26781751	0	0	0	0
HMGN2	NC_000001	26798902	26803133	9.1735517	13.08001	11.556518	24.698988
LOC100128503	NC_000001	26845845	26846741	0	0	0	0
RPS6KA1	NC_000001	26856249	26901520	0.2078545	0.3606124	0.6240817	0.624628
LOC100289322	NC_000001	26966868	26967105	0	0	0	0
LOC100289354	NC_000001	26973937	26974174	0	0	0	0
ARID1A	NC_000001	27022522	27108601	1.2951546	2.0368057	2.8463574	3.7634533
PIGV	NC_000001	27114486	27124887	3.3634799	2.4836542	3.4716654	5.39982
ZDHHC18	NC_000001	27153201	27182208	7.1612074	9.437222	7.9541604	11.923781
SFN	NC_000001	27189633	27190947	0.1002621	0.75592	0.2081239	0.0465778
GPN2	NC_000001	27205873	27216869	5.8791674	10.682433	6.4731069	8.0517064
GPATCH3	NC_000001	27216979	27226962	0.9082772	0.9974729	0.7896643	1.3521553
NR0B2	NC_000001	27237975	27240567	0.0345777	0	0	0
NUDC	NC_000001	27248224	27272887	4.6697066	9.8742928	8.2202116	9.4851733
C1orf172	NC_000001	27276061	27286897	0.0492417	0.0253128	0.0219034	0
RPL12P13	NC_000001	27306571	27307515	0	0	0	0
RPL32P6	NC_000001	27310090	27310515	0	0	0	0
LOC646567	NC_000001	27312388	27312831	0	0.4070577	0.7044606	0.068975
TRNP1	NC_000001	27320195	27327377	2.3225592	8.2762824	2.8485656	1.9170011
FAM46B	NC_000001	27331511	27339333	1.8142067	0.7803368	1.0210822	1.4577132
SLC9A1	NC_000001	27425300	27481451	4.6332125	5.5145097	5.6440055	5.0304645
RPL18AP5	NC_000001	27503204	27503821	0	0	0	0
LOC646572	NC_000001	27526514	27528092	0	0	0	0
LOC440577	NC_000001	27533427	27534287	0.0510432	0.1049556	0.1362285	0.035569
WDTC1	NC_000001	27561007	27635110	5.8207154	5.2597921	9.5911979	15.931059
TMEM222	NC_000001	27648636	27662891	12.661665	12.093699	14.389067	18.386312
SYTL1	NC_000001	27668513	27680421	0.1389301	0.0714174	0.0411987	0.0645414
MAP3K6	NC_000001	27681670	27693337	4.9756547	5.4808886	4.5710185	5.2291555
FCN3	NC_000001	27695601	27701315	0.0425855	0.2626942	0.0378852	0.1187011
CD164L2	NC_000001	27705596	27709805	0.0285564	0	0.0508091	0.0397984
GPR3	NC_000001	27719152	27722318	1.6030383	1.1129971	1.3890656	0.5802914
WASF2	NC_000001	27732126	27816669	7.2972591	7.2589728	8.5886456	21.910774
AHDC1	NC_000001	27860756	27930143	0.4194566	0.4521122	1.9019136	1.3718965
FGR	NC_000001	27938800	27961727	0.0316858	0.0488645	0.0422829	0.0441599
LOC729973	NC_000001	27975896	27976459	0.2337672	0.0801124	0.1386438	0.2714971
IFI6	NC_000001	27992572	27998724	439.78893	150.99662	401.25013	7.6206092
LOC646597	NC_000001	28013320	28013951	0	0	0	0
FAM76A	NC_000001	28052490	28089426	3.1863103	3.9153728	5.5337398	3.2995608
STX12	NC_000001	28099694	28150963	10.291223	9.8026997	9.041074	7.9471509
PPP1R8	NC_000001	28157293	28178183	2.6891127	3.4880107	3.8110043	4.6629128
SCARNA1	NC_000001	28160912	28161077	0	0	0	0
C1orf38	NC_000001	28199055	28213196	5.8748279	2.1239352	7.3801501	1.3833493
RPA2	NC_000001	28218049	28241236	25.040272	27.057502	51.804274	64.935425
SMPDL3B	NC_000001	28261504	28285663	0.0387039	0.1193749	0.6197765	0.1213668
LOC100128789	NC_000001	28284985	28298139	0.0624265	0.064181	0.4998268	0.1305037
XKR8	NC_000001	28286504	28294604	0.1832873	0.0837505	0.14494	0.1561046
EYA3	NC_000001	28300819	28415131	3.4170019	5.1701301	5.2767375	10.737427
LOC653566	NC_000001	28421446	28423069	0	0	0	0
LOC100131223	NC_000001	28446888	28447832	0	0	0	0
RNU7-29P	NC_000001	28470667	28470725	0	0	0	0
PTAFR	NC_000001	28475838	28503191	0.0241209	0	0.0214586	0
DNAJC8	NC_000001	28526789	28559542	12.748722	15.89739	20.534529	26.321779
ATPIF1	NC_000001	28562611	28564611	4.0245634	4.7287705	7.979095	9.4871418
SESN2	NC_000001	28586006	28609002	10.138989	7.3641814	7.4717299	4.9034956
MED18	NC_000001	28655513	28662478	2.7511734	2.344992	4.0373623	3.4573833
PHACTR4	NC_000001	28696093	28826881	2.8161529	2.2689139	4.0832023	6.7552104
SNHG3-RCC1	NC_000001	28832496	28865607	0.9870553	2.7283056	4.088258	6.3820623
SNHG3	NC_000001	28832496	28836146	0	0	0	0
SNORA73A	NC_000001	28833877	28834083	0	0	0	0
RNU105A	NC_000001	28835070	28835227	0	0	0	0

SNORA73B	NC_000001	28835070	28835274	0	0	0	0
RCC1	NC_000001	28844745	28865607	0.0867856	0.1606045	0.0772069	0.181427
LOC100128092	NC_000001	28845050	28859254	0	0	0	0
TRNAU1AP	NC_000001	28879529	28905057	5.3905437	6.364882	7.1200709	8.792145
SNHG12	NC_000001	28905050	28908366	0	0	0	0
SNORD99	NC_000001	28905255	28905334	0	0	0	0
SNORA61	NC_000001	28906276	28906405	0	0	0	0
SNORA44	NC_000001	28906893	28907024	0	0	0	0
LOC100289582	NC_000001	28907076	28908385	4.3855904	3.0850012	11.129664	4.1176307
SNORA16A	NC_000001	28907432	28907565	0	0	0	0
LOC100132870	NC_000001	28907586	28909644	0.1494112	0.1097217	0.1898862	0.089242
RAB42	NC_000001	28918712	28921098	1.4877261	0.4960666	1.1088971	0.7004775
TAF12	NC_000001	28929608	28969604	5.0278473	8.0028493	6.7632591	4.5800128
RNU11	NC_000001	28975112	28975246	0	0	0	0
GMEB1	NC_000001	28995244	29041398	2.4725057	2.335888	2.6355683	3.9425844
YTHDF2	NC_000001	29063136	29096287	2.91552	3.1746508	4.3697279	5.074122
LOC100289620	NC_000001	29063691	29064838	0	0	0	0
OPRD1	NC_000001	29138654	29190208	0.0247735	0	0.0220392	0.0345264
EPB41	NC_000001	29213628	29446958	1.5387746	0.7945582	7.9435155	7.7463776
RPL27P4	NC_000001	29308756	29309156	0	0	0	0
TMEM200B	NC_000001	29445940	29450407	7.2681554	7.4907875	9.2461533	7.1429013
SFRS4	NC_000001	29474250	29508637	3.4046852	6.2863864	7.8195131	6.8996761
MECR	NC_000001	29519385	29557454	2.1667164	2.9887131	3.6141957	3.6739788
PTPRU	NC_000001	29563047	29653316	0.1409601	0.0805121	0.2159702	0.1200548
LOC100288450	NC_000001	29814633	29823398	0	0	0	0.0745131
MATN1	NC_000001	31184124	31196432	0.1376968	0.1179723	0.0714577	0.0959526
LOC100288680	NC_000001	31191614	31194454	0.3354827	0.0574852	0	0.2727406
LOC100129196	NC_000001	31192050	31192418	0.1191009	0.1224482	0.1059555	0.0829943
LAPTM5	NC_000001	31205315	31230683	0.2550567	0.1815405	0.191997	0.232421
SDC3	NC_000001	31342313	31381480	2.9007034	4.1998241	1.1375781	1.7522143
LOC100288670	NC_000001	31347183	31351518	0.047358	0.097378	0.042131	0.0330009
PUM1	NC_000001	31404353	31538564	2.1708876	2.2906349	6.7522258	4.9705002
SNORD103A	NC_000001	31408536	31408623	0	0	0	0
SNORD85	NC_000001	31441010	31441084	0	0	0	0
PRO0611	NC_000001	31460912	31461481	0	0	0	0
SEPW1P	NC_000001	31567418	31568485	0	0	0	0
RPL21P22	NC_000001	31580948	31581510	0	0	0	0
NKAIN1	NC_000001	31652593	31661000	0.0352573	0.1268688	0.0313659	0.0491374
SNRNP40	NC_000001	31732415	31769644	7.9290837	16.02564	17.333896	15.70845
ZCCHC17	NC_000001	31769842	31837780	6.9406384	8.292085	10.88484	13.687524
FABP3	NC_000001	31838100	31845923	12.218971	5.2308952	1.7107412	1.0887605
SERINC2	NC_000001	31885963	31907525	5.8098814	4.7740099	1.7816117	2.5763543
LOC441880	NC_000001	31960432	31961897	0	0	0	0
LOC149086	NC_000001	31971822	31974166	0	0	0	0
LOC284551	NC_000001	31984036	31989846	0	0	0	0
TINAGL1	NC_000001	32042086	32053287	0.0196461	0.0201982	0	0
HCRTR1	NC_000001	32083301	32092919	0	0.0451608	0.019539	0.0459144
PEF1	NC_000001	32095471	32110477	22.274383	28.176482	25.935528	27.828694
COL16A1	NC_000001	32117848	32169768	4.0375077	8.5204374	1.1412792	1.18463
BAI2	NC_000001	32192718	32229648	1.5339272	1.3016825	2.5487425	0.9218569
SPOCD1	NC_000001	32256025	32281580	13.575573	8.2045992	4.4547895	4.332223
PTP4A2	NC_000001	32373793	32403988	26.515569	25.439218	91.511494	59.504224
KHDRBS1	NC_000001	32479491	32509472	15.238661	19.369123	16.454469	19.150529
TMEM39B	NC_000001	32538503	32568467	2.9250473	3.901977	4.000081	7.2266627
KPNA6	NC_000001	32573644	32642169	6.8667249	10.295418	14.858454	22.674788
TXLNA	NC_000001	32645345	32663886	17.483992	18.079013	15.081421	28.17923
CCDC28B	NC_000001	32666202	32670991	5.712769	4.0701126	5.7955342	6.9840087
IQCC	NC_000001	32671272	32674288	0.2614669	0.4928284	0.5427525	0.5769685
DCDC2B	NC_000001	32674695	32681797	0.9121946	0.5693979	0.4057568	0.5675478
C1orf91	NC_000001	32681798	32687926	2.5589081	3.0901772	3.3966462	3.4813863
EIF3I	NC_000001	32687971	32697205	45.455373	74.65488	77.202943	101.26374
LOC339483	NC_000001	32697261	32707311	0	0	0	0
FAM167B	NC_000001	32712818	32714461	1.976751	0.2835778	0.7361466	1.3454444
LCK	NC_000001	32716840	32751766	0	0	0.017509	0.0137147
HDAC1	NC_000001	32757708	32799224	6.3894129	6.1584264	6.544308	10.603736
MARCKSL1	NC_000001	32799440	32801834	2.0183211	3.2148604	1.01158	1.2479737

LOC100286999	NC_000001	32822553	32827391	0.0823001	0.1692262	0.1464328	0.1146999
LOC100128071	NC_000001	32825189	32827734	0.0967596	0.0397916	0.034432	0.0539408
TSSK3	NC_000001	32827862	32829926	0.3670695	0.1715391	0.4749894	0.5348308
BSDC1	NC_000001	32830704	32860062	11.160737	14.222679	13.825222	16.533225
LOC343338	NC_000001	32867501	32868784	0	0	0	0
LOC100133117	NC_000001	32888812	32893180	0	0	0	0
ZBTB8B	NC_000001	32930658	32953460	0.0218702	0.0337273	0	0.01524
ZBTB8A	NC_000001	33004772	33071542	1.1087444	0.9981878	1.6155138	1.3322428
ZBTB8OS	NC_000001	33087305	33116185	5.5165145	5.0413838	6.598055	8.7133545
RBBP4	NC_000001	33116749	33151812	7.3228782	5.0210041	10.991009	21.034074
SYNC	NC_000001	33145507	33168361	8.291407	9.8438502	4.0519092	7.3208627
KIAA1522	NC_000001	33207512	33240571	1.5916211	3.6150061	2.0011134	4.6174983
YARS	NC_000001	33240840	33283633	6.6972763	8.5815123	9.4200423	13.460405
S100PBP	NC_000001	33283176	33324476	0.4466678	0.5390861	1.1229968	0.9946657
FNDC5	NC_000001	33327877	33336347	0.0330811	0.2550813	0.0882896	0.1383133
HPCA	NC_000001	33352098	33360247	0.0312576	0.1285445	0.0556153	0.0435631
LOC100129200	NC_000001	33353113	33359417	0	0	0	0
TMEM54	NC_000001	33360196	33366953	8.2762531	10.283433	10.394519	6.0756304
RNF19B	NC_000001	33402050	33430286	5.565202	4.6986577	12.122346	4.9709607
RPL18AP4	NC_000001	33445511	33446125	0	0	0	0
AK2	NC_000001	33473586	33502492	10.141017	12.596156	33.943749	37.82744
ADC	NC_000001	33546714	33585995	0.4028252	0.7661713	1.0750935	0.4771979
TRIM62	NC_000001	33611003	33647671	0.7322798	0.6940434	0.2951912	0.8292078
ZNF362	NC_000001	33722174	33766320	2.0658216	3.1276341	0.9189061	1.3409477
A3GALT2	NC_000001	33772367	33786699	0.0429601	0	0.0382185	0
PHC2	NC_000001	33789224	33841194	14.937136	12.22928	15.36745	17.824779
LOC100131451	NC_000001	33931850	33934576	0	0	0	0
ZSCAN20	NC_000001	33938232	33961995	0.3125763	0.2785131	0.9918064	1.0963387
CSMD2	NC_000001	33979609	34631443	0.0402334	0.3343599	0.0805336	0.3714797
LOC100287140	NC_000001	34304122	34382834	0	0	0	0
HMGB4	NC_000001	34326076	34330392	0	0	0	0
C1orf94	NC_000001	34632624	34684732	0	0	0.0247924	0.0388394
GJB5	NC_000001	35220721	35224112	0	0	0	0.047814
GJB4	NC_000001	35226778	35227928	0.0381827	0	0.0679367	0.0532144
GJB3	NC_000001	35246790	35251967	0	0.4610551	0	0
LOC100287174	NC_000001	35247872	35258631	0.1641391	0.5906327	0.1825283	0.0285947
GJA4	NC_000001	35258599	35261348	0	0	0	0
C1orf212	NC_000001	35319124	35325345	4.989376	6.023262	5.0727854	5.8511931
DLGAP3	NC_000001	35331037	35370984	0.0245042	0.0503857	0	0.0426887
LOC100130633	NC_000001	35447127	35450948	3.2783005	4.4450698	7.9885837	2.6155301
ZMYM6	NC_000001	35451766	35497569	1.3410831	1.5456324	1.8009957	2.2380863
ZMYM1	NC_000001	35544972	35581455	2.2132203	3.1855924	3.2065629	2.842165
SFPQ	NC_000001	35649201	35658743	6.8503752	12.336113	5.8907169	7.514207
ZMYM4	NC_000001	35734568	35887545	2.0924737	3.2043133	5.2715191	7.2161566
RPL5P4	NC_000001	35816249	35817267	0	0	0	0
KIAA0319L	NC_000001	35899091	36023004	2.3832443	2.1276643	5.0133342	4.0824762
NCDN	NC_000001	36023393	36032380	6.891747	6.0488441	4.3899021	7.331786
TFAP2E	NC_000001	36038971	36060929	0.0401537	0.0412822	0.1607483	0.1818746
PSMB2	NC_000001	36068739	36107143	37.395004	52.562466	62.371028	70.382856
C1orf216	NC_000001	36179477	36184790	1.1983158	1.3397941	0.6129816	1.0855444
LOC100289612	NC_000001	36200239	36201517	0.3779754	0.9891597	0.5196705	2.3226061
CLSPN	NC_000001	36201818	36235551	0.5118428	1.1453203	0.5982044	1.6714652
EIF2C4	NC_000001	36273828	36321188	0.4156516	0.4653188	1.3476252	1.0748959
EIF2C1	NC_000001	36348810	36389899	1.1460157	1.5407552	2.2377318	4.8447755
EIF2C3	NC_000001	36396772	36522063	2.0886533	3.1511484	3.0675537	3.5654386
LOC100128093	NC_000001	36545671	36546923	0	0	0	0
TEKT2	NC_000001	36549676	36553876	0.0873722	0.1197704	0.1036383	0.0202948
ADPRHL2	NC_000001	36554453	36559533	14.917969	13.547005	21.178811	23.090905
COL8A2	NC_000001	36560846	36565850	1.0682892	3.0915494	1.0647773	0.1240711
TRAPPC3	NC_000001	36602173	36615067	7.41757	10.490283	11.276952	15.603642
MAP7D1	NC_000001	36621803	36646441	4.2044337	5.7634664	5.598809	6.2005244
THRAP3	NC_000001	36690017	36770958	2.1319652	2.3040273	2.0025152	5.0304403
C1orf113	NC_000001	36772718	36790487	0.8148792	0.9229796	0.5529197	1.6169012
FAM176B	NC_000001	36787632	36789755	30.772102	38.667389	34.683324	14.469819
STK40	NC_000001	36805225	36851485	2.7767604	2.2556456	3.6596785	6.4180062
LOC100287265	NC_000001	36857590	36861824	0.0155459	0.0319656	0.1244705	0.064998

LSM10	NC_000001	36859031	36863493	7.1868285	9.6745637	9.0154387	7.3139415
C1orf102	NC_000001	36883507	36916052	1.269263	1.5767974	1.4820377	1.5846808
MRPS15	NC_000001	36921362	36930040	6.5580781	10.066768	15.760573	16.058226
CSF3R	NC_000001	36931644	36948509	0	0	0.0253551	0.039721
GRIK3	NC_000001	37261128	37499844	0.0190685	0.0098022	0.004241	0.0298974
LOC100287202	NC_000001	37356527	37503568	0.0586367	0.0904271	0.0521649	0.0408604
RPS29P6	NC_000001	37796453	37796580	0	0	0	0
LOC728431	NC_000001	37920480	37940195	0.1775686	0.3346919	0.7898498	0.5980616
ZC3H12A	NC_000001	37940119	37949978	5.873788	4.0924583	11.113152	2.8414836
C1orf149	NC_000001	37958176	37980364	5.7314881	7.4919847	8.9776897	9.4570537
SNIP1	NC_000001	38002142	38019903	1.0315651	1.0605574	1.0442895	0.8055917
FTHL1	NC_000001	38010164	38011059	0	0	0	0
RPS27P9	NC_000001	38021815	38022154	0	0	0	0
DNALI1	NC_000001	38022520	38032458	1.0286109	0.2899652	2.8633174	1.6185289
GNL2	NC_000001	38032413	38061586	4.8851698	8.4272123	6.7875457	9.804551
RSPO1	NC_000001	38076951	38100491	0.0294461	0.0302736	0.039294	0.0410384
C1orf109	NC_000001	38147242	38156192	1.8101819	2.9738545	1.6103881	3.1860277
CDCA8	NC_000001	38158159	38175391	10.972746	18.402966	12.970274	24.02916
LOC100287787	NC_000001	38181640	38183858	0.0198054	0	0	0
EPHA10	NC_000001	38181646	38230824	0.0595325	0.0136013	0.0058846	0.0276564
LOC391026	NC_000001	38232107	38245184	0	0	0	0
LOC100129497	NC_000001	38247885	38248557	0	0	0	0
MANEAL	NC_000001	38259774	38267278	1.6125196	4.7117548	4.1400383	6.8307179
YRDC	NC_000001	38268614	38273865	6.1757319	5.7586689	6.6440308	7.6061789
C1orf122	NC_000001	38273473	38275126	9.8033233	10.90161	8.2466792	9.387292
MTF1	NC_000001	38275239	38325292	1.3285905	1.8476905	3.4820963	3.4036179
RPS2P13	NC_000001	38306517	38307352	0	0	0	0
INPP5B	NC_000001	38326369	38412729	1.6576792	1.1564679	1.5186077	1.485176
SF3A3	NC_000001	38422647	38455761	4.052952	7.522615	7.0460942	10.348428
FHL3	NC_000001	38462442	38471234	5.0808695	6.8411489	4.5889162	7.0092151
LOC100287358	NC_000001	38464960	38471687	0	0.1943372	0.1681616	0.06586
UTP11L	NC_000001	38478384	38490497	6.2170669	9.4554534	14.799859	10.950261
POU3F1	NC_000001	38509523	38512450	0.0450289	0.0154315	0.066765	0.0104593
LOC400750	NC_000001	39174602	39176600	0	0	0	0
RRAGC	NC_000001	39305005	39325340	19.078775	19.552318	16.05115	16.076999
MYCBP	NC_000001	39328636	39339050	2.3960299	5.553388	3.30955	3.8519096
GJA9	NC_000001	39339739	39347289	0.2470069	0.1758109	0.1183238	0.4369308
RHBDL2	NC_000001	39351479	39407456	0.7894892	0.5411186	0.5533678	0.4001073
LOC100288425	NC_000001	39423947	39424597	0	0	0	0
AKIRIN1	NC_000001	39456916	39471737	5.6104126	6.6631429	7.8740145	7.392212
NDUFS5	NC_000001	39492006	39500287	33.971725	54.821349	59.832282	78.390099
MACF1	NC_000001	39547118	39952789	0.8751359	1.4414452	1.0786095	5.8594457
KIAA0754	NC_000001	39875176	39882154	0	0	0	0.0087763
LOC100288731	NC_000001	39949529	39966319	0.0279036	0.0573757	0.1241193	0.0972218
BMP8A	NC_000001	39957318	39995541	0.062493	0.1525924	0.0277978	0.0217738
TRNAK36P	NC_000001	39970195	39970267	0	0	0	0
OXCT2P	NC_000001	39980535	39982339	0	0	0	0
LOC728448	NC_000001	39987952	40025370	0	0	0	0
LOC100130494	NC_000001	39990431	39991357	0	0	0	0.0660731
PABPC4	NC_000001	40026485	40042521	11.741475	15.286769	9.3472957	20.55996
SNORA55	NC_000001	40033046	40033182	0	0	0	0
HEYL	NC_000001	40089103	40105348	0.1281912	0.0768799	0.0950354	0.0967728
NT5C1A	NC_000001	40124793	40137710	0	0	0	0.0553295
HPCAL4	NC_000001	40144645	40157089	0.0825513	0.0530446	0.01836	0.0359531
LOC100128091	NC_000001	40183700	40184268	0	0	0	0
PPIE	NC_000001	40204530	40229586	2.3039702	3.0201225	2.2546434	1.0636426
BMP8B	NC_000001	40223903	40254533	0.0116481	0.0239509	0	0
OXCT2	NC_000001	40235197	40237020	0.0240944	0.0743148	0	0.0335799
LOC100287437	NC_000001	40254654	40255843	0.1846564	0.0759385	0.0328551	0
TRIT1	NC_000001	40306703	40349177	1.9592126	2.0566823	2.4034637	2.1269273
MYCL1	NC_000001	40361098	40367687	0	0.0822015	0.0158066	0.0185718
LOC100289331	NC_000001	40363341	40366870	0.0780608	0.0802547	0.2083352	0
MFSD2	NC_000001	40420784	40435628	2.3960871	1.2111861	3.1796748	0.9183289
RPS2P12	NC_000001	40428196	40429113	0	0	0	0
CAP1	NC_000001	40506255	40538321	58.560662	77.608562	61.797559	63.856129
PPT1	NC_000001	40538382	40563142	15.866295	22.447345	23.342995	24.570839

LOC728602	NC_000001	40598434	40599121	0	0	0	0
RLF	NC_000001	40627041	40706593	1.3289945	1.3518874	1.7703378	2.1363915
TMCO2	NC_000001	40713573	40717365	0.2372374	0.0609763	0.211053	0
ZMPSTE24	NC_000001	40723733	40759856	8.4858824	10.357514	8.5657634	11.409078
COL9A2	NC_000001	40766163	40782939	0.5122895	0.2394034	0.1933472	0.14063
RPL21P20	NC_000001	40798714	40799270	0	0	0	0
LOC100287508	NC_000001	40819969	40830856	0	0	0	0
LOC100286988	NC_000001	40839343	40872543	4.7839149	9.7711566	5.9582647	6.1338653
SMAP2	NC_000001	40839728	40888994	17.435983	23.486248	28.655359	35.427848
ZNF643	NC_000001	40915779	40929352	0.6005072	1.8292875	0.455083	0.58894
ZNF642	NC_000001	40943302	40962015	0.9567239	1.3812434	0.5432733	0.7943461
DEM1	NC_000001	40974433	40982228	1.1299275	1.2635863	0.7054139	1.2294155
ZNF684	NC_000001	40997233	41013839	0.4405312	0.6038832	0.4292334	0.4385424
LOC728633	NC_000001	41059342	41060064	0	0	0	0
RIMS3	NC_000001	41086352	41131324	0.0303972	0.0500024	0.2271542	0.1101462
LOC100130557	NC_000001	41154752	41157933	0	0	0	0
NFYC	NC_000001	41157242	41237275	6.5275821	9.7221996	6.6525457	10.530355
KCNQ4	NC_000001	41249684	41304360	0.0564645	0.038701	0.0837207	0.0131156
CITED4	NC_000001	41326728	41328018	1.7021004	1.8899332	2.2107841	1.1623695
LOC100128362	NC_000001	41391281	41445040	0.1148973	0.1771898	0	0.0400325
CTPS	NC_000001	41445007	41478235	6.6217717	11.200512	5.0109415	8.778685
SLFNL1	NC_000001	41481269	41487387	0.270312	0.2547501	0.1603181	0.4238194
SCMH1	NC_000001	41492874	41651925	7.8998116	7.2771657	7.3877195	10.771995
RPL23AP17	NC_000001	41564310	41564805	0	0	0	0
RPL36AP9	NC_000001	41730173	41730551	0	0	0	0
FOXO6	NC_000001	41827603	41848353	0.0890843	0.1221173	0.2377555	0.2896948
EDN2	NC_000001	41944446	41950344	0	0.0363503	0	0
HIVEP3	NC_000001	41975684	42384496	0.2674937	0.3055685	0.9078109	0.8767718
LOC644449	NC_000001	42506216	42508516	0	0	0	0
GUCA2B	NC_000001	42619092	42621495	0	0	0	0.051298
GUCA2A	NC_000001	42628362	42630395	0	0	0.0679958	0
FOXJ3	NC_000001	42642210	42800903	6.2591154	8.3957688	11.009504	9.2910881
RIMKLA	NC_000001	42846468	42889900	0.0332658	0.0128253	0.0184963	0.0202833
RPS15AP8	NC_000001	42878023	42878495	0	0	0	0
ZMYND12	NC_000001	42896094	42921927	0.4210609	0.2164474	0.491646	0.1100295
PPCS	NC_000001	42922173	42926086	116.93949	63.086832	140.24184	252.07422
RPS3AP11	NC_000001	42957386	42958184	0	0	0	0
TMSL1	NC_000001	42965539	42966085	0	0	0	0
LOC728621	NC_000001	43000560	43120335	0.099302	0.1166776	0.0631013	0.0988537
PPIH	NC_000001	43124048	43142429	4.8892408	9.6579522	9.0413121	10.680426
YBX1	NC_000001	43148066	43168020	4.6904646	10.404457	8.3455347	13.688092
CLDN19	NC_000001	43198764	43205925	0.0244021	0.012544	0.0434176	0.0170044
LEPRE1	NC_000001	43212006	43232755	25.786453	35.17442	17.510935	12.370809
LOC100287607	NC_000001	43215846	43232900	0.1252086	0.0858184	0.0371297	0
C1orf50	NC_000001	43232916	43241418	2.1543251	2.4806574	1.3032522	0.8707073
LOC100129924	NC_000001	43250726	43263676	0.1288804	0	0	0
CCDC23	NC_000001	43272723	43283059	4.020327	4.7561476	4.0665388	3.0317862
ERMAP	NC_000001	43282776	43310660	1.142654	0.9165776	0.670244	1.0762457
LOC100287668	NC_000001	43312144	43314483	0.0187813	0	0	0
ZNF691	NC_000001	43312280	43318148	1.4770173	1.4325746	1.1652413	1.3982189
MKRNP4	NC_000001	43355302	43358111	0	0	0	0
LOC343515	NC_000001	43368903	43369583	0	0	0	0
SLC2A1	NC_000001	43391046	43424847	7.0652471	7.4115553	7.0631297	11.448865
FAM183A	NC_000001	43613594	43622067	0.1700125	0.0873954	0.0756239	0
EBNA1BP2	NC_000001	43629855	43637931	1.9281372	4.5798607	1.7448989	4.0308085
WDR65	NC_000001	43638026	43676549	0.2042746	0.6000452	0.9346032	2.0843626
LOC100131756	NC_000001	43705872	43707974	0	0	0	0
TMEM125	NC_000001	43736407	43739673	0.0268141	0.082703	0.0238545	0.0186851
LOC100288314	NC_000001	43737940	43739675	0	0.0599249	0.0259268	0
C1orf210	NC_000001	43747554	43751250	0.0297349	0.0305706	0	0.0207205
TIE1	NC_000001	43766664	43788779	0.0226421	0.1047529	0.040286	0.0552226
MPL	NC_000001	43803475	43820135	0.0241143	0.037188	0.0321791	0.0168038
CDC20	NC_000001	43824626	43828874	24.715961	63.931142	23.7153	32.690915
ELOVL1	NC_000001	43829072	43833699	31.175684	48.991848	30.054771	34.114834
MED8	NC_000001	43849588	43855483	9.509551	14.402678	9.0441519	8.3044772
C1orf84	NC_000001	43855556	43872565	0.8138561	1.140995	1.3493268	2.0622814

KIAA0467	NC_000001	43888797	43918304	1.1958117	1.1321279	2.0511202	2.7966146
HYI	NC_000001	43916829	43919918	3.860621	10.707405	1.6773215	1.9707531
PTPRF	NC_000001	43996547	44089343	1.5401488	2.96821	2.4066263	0.9940314
KDM4A	NC_000001	44115797	44171189	1.2963218	2.5753236	1.2572959	1.9289123
ST3GAL3	NC_000001	44173218	44396831	3.5517347	4.68432	4.3725441	3.6249856
SHMT1P	NC_000001	44315093	44317616	0	0	0	0
ARTN	NC_000001	44398992	44402912	0.0194289	0.0599249	0.1382761	0.0270777
IPO13	NC_000001	44412643	44433425	1.7613541	2.5150794	1.4254888	3.0684543
DPH2	NC_000001	44435653	44439043	6.6529562	9.6265798	5.4176042	9.6032358
ATP6V0B	NC_000001	44440602	44443972	38.271944	40.806536	40.053964	21.934844
B4GALT2	NC_000001	44444866	44456840	19.26654	29.965992	18.238769	19.185235
CCDC24	NC_000001	44457280	44462200	0.450597	0.4632611	0.8551757	0.5651891
SLC6A9	NC_000001	44462155	44497134	1.7955269	1.4247577	2.991012	3.065007
LOC644743	NC_000001	44568994	44570387	0	0	0	0
KLF17	NC_000001	44584522	44600812	0.1544013	0.1010169	0.0624362	0.1467177
LOC100130714	NC_000001	44653604	44674933	0	0	0	0
DMAP1	NC_000001	44679125	44686351	4.8776067	7.9058161	4.7377571	5.6879729
ERI3	NC_000001	44686742	44820939	8.9005885	14.438987	16.262613	17.991896
RNF220	NC_000001	44870960	45117396	8.4100271	10.777968	7.2609765	8.8720578
LOC100287761	NC_000001	44877945	45079962	0	0	0	0.0522609
TMEM53	NC_000001	45119501	45140099	1.7579293	1.1578247	1.9548783	1.5503845
C1orf228	NC_000001	45140394	45191263	0.1825743	0.0536302	0.1160165	0.0727
RNU5F	NC_000001	45187458	45187573	0	0	0	0
KIF2C	NC_000001	45205490	45233439	1.7390137	3.1523304	3.3519954	6.856316
RPS8	NC_000001	45241246	45244412	159.39805	315.64291	232.58892	281.74888
SNORD55	NC_000001	45241537	45241610	0	0	0	0
SNORD46	NC_000001	45242164	45242261	0	0	0	0
SNORD38A	NC_000001	45243514	45243584	0	0	0	0
SNORD38B	NC_000001	45244062	45244130	0	0	0	0
RPS15AP11	NC_000001	45245957	45246417	0	0	0	0
BEST4	NC_000001	45249257	45253426	0.0419353	0.021557	0.0186534	0.0438333
PLK3	NC_000001	45266036	45271667	6.6723779	4.4072021	3.0177086	1.3766909
TCTEX1D4	NC_000001	45271580	45272957	0.9343325	0.3913523	0.0923565	0.0723422
LOC149478	NC_000001	45274154	45279801	2.0688099	1.293418	0.6217806	0.8571849
PTCH2	NC_000001	45288087	45308616	0.024254	0.1745496	0.1941932	0.059154
EIF2B3	NC_000001	45316450	45452282	4.8743066	6.2641244	4.454153	5.2795149
LOC728887	NC_000001	45424229	45425067	0	0	0	0
LOC128192	NC_000001	45453922	45454397	0	0	0	0
MRPS17P1	NC_000001	45457349	45457925	0	0	0	0
HECTD3	NC_000001	45468220	45477027	8.8429463	8.5933151	7.7591641	8.8717605
UROD	NC_000001	45477830	45481341	2.0655351	2.1889284	2.5443101	5.2923687
ZSWIM5	NC_000001	45482074	45672250	0.6172927	0.2786232	0.3214599	0.529824
LOC100288481	NC_000001	45769582	45771291	0.0257007	0.0528461	0.0914563	0.1432743
LOC400752	NC_000001	45769582	45771291	0	0	0	0
HPDL	NC_000001	45792545	45794347	0.1950005	0.8019239	0.7372808	0.3227247
MUTYH	NC_000001	45794914	45806142	0.8050639	0.9125816	0.6978429	0.6760776
TOE1	NC_000001	45805342	45809650	2.2231338	3.5889826	2.1085226	3.0599272
TESK2	NC_000001	45809555	45956840	1.0572722	0.9107188	1.1312365	1.0055633
LOC126661	NC_000001	45960581	45965646	0.1844627	0.0948235	0.0820516	0.3534876
MMACHC	NC_000001	45965856	45976739	1.4957819	2.1878279	0.9740095	1.730037
PRDX1	NC_000001	45976707	45987609	69.768708	82.750801	116.1204	147.24263
LOC100128639	NC_000001	45996599	45997811	0	0	0	0
AKR1A1	NC_000001	46016498	46035721	18.753154	18.985859	19.943579	16.020702
NASP	NC_000001	46049717	46084567	1.8133979	2.440123	1.2217989	3.1684113
CCDC17	NC_000001	46085716	46089729	0.0408441	0.146972	0.0908401	0.0711545
GPBP1L1	NC_000001	46092976	46152302	4.0444601	3.9163783	5.7840968	7.2670591
RPS15AP10	NC_000001	46111444	46112357	0	0	0	0
RPL7AP16	NC_000001	46116650	46117522	0	0	0	0
TMEM69	NC_000001	46153847	46160108	7.2300966	7.5254476	10.604982	7.9737107
IPP	NC_000001	46159996	46216485	1.4475869	1.5796566	1.5476355	1.6281357
RPL6P1	NC_000001	46233964	46251532	0	0	0	0
MAST2	NC_000001	46269285	46501796	4.3197635	4.8506406	6.9977692	10.375525
LOC100101407	NC_000001	46311010	46313381	0	0	0	0
PIK3R3	NC_000001	46505812	46598708	1.052741	1.6311147	1.2729133	1.4413531
LOC100133124	NC_000001	46570290	46570847	0.1808569	0	0	0
TSPAN1	NC_000001	46646212	46651630	0.4126595	0.318193	0.3977061	0.2396313

POMGNT1	NC_000001	46654353	46685977	12.448786	14.086784	15.004548	17.573486
C1orf190	NC_000001	46669006	46686928	0.0237686	0.0977467	0.1480167	0.0993776
RAD54L	NC_000001	46713367	46744145	1.3969559	1.7952718	2.7216678	4.0106325
LRRC41	NC_000001	46744072	46769038	25.089849	28.216636	22.161476	26.18905
UQCRH	NC_000001	46769380	46782448	20.931701	30.436637	46.590647	54.97369
NSUN4	NC_000001	46806390	46830690	2.0606926	2.5158477	1.8773177	1.9330401
FAAH	NC_000001	46859939	46879520	0.2307544	0.0215673	0.055987	0.0438542
LOC729041	NC_000001	46903225	46911301	0.1827369	0.0469682	0.2438518	0.1273384
DMBX1	NC_000001	46972668	46979898	0	0	0	0.042285
KNCN	NC_000001	47011316	47016887	0.018373	0	0.0326903	0.0384091
MKNK1	NC_000001	47023090	47069966	3.7212329	3.1801108	2.2489132	3.3699402
MOBKL2C	NC_000001	47073387	47082563	3.0623894	2.0131968	3.5888553	3.2112519
ATPAF1	NC_000001	47098409	47134099	4.6278094	4.8563134	4.7322156	6.4941645
C1orf223	NC_000001	47137500	47139253	0	0.10594	0	0.0359026
KIAA0494	NC_000001	47140831	47184736	11.118514	10.882564	10.29811	11.698735
LOC100129898	NC_000001	47188130	47208804	0	0	0	0
CYP4B1	NC_000001	47264670	47285021	0.0203653	0.0628129	0.0181175	0.0141913
LOC100132680	NC_000001	47286166	47366124	0	0	0	0
CYP4Z2P	NC_000001	47323906	47366147	0	0	0	0
CYP4A11	NC_000001	47394846	47407156	0	0	0	0
CYP4X1	NC_000001	47489240	47516423	0.0186458	0.0383398	0.0331757	0.0129932
CYP4Z1	NC_000001	47533160	47583992	0.0230457	0	0	0
LOC100132432	NC_000001	47535126	47548225	0	0	0	0
CYP4A22	NC_000001	47603107	47614526	0.0263479	0	0	0
PDZK1IP1	NC_000001	47649261	47655771	0.0491591	0.0505407	0.6559994	0.034256
TAL1	NC_000001	47681963	47695443	0.0087861	0.0090331	0.0312655	0.0244901
STIL	NC_000001	47715811	47779819	3.0909994	4.8883417	5.8658033	5.8760227
CMPK1	NC_000001	47799469	47844511	16.169349	21.60476	21.483645	24.405833
FOXE3	NC_000001	47881744	47883724	0.0443697	0.0456168	0.0197363	0
MGC12982	NC_000001	47897805	47900313	0	0	0	0
FOXD2	NC_000001	47901689	47906363	0.1410104	0.1836331	0.1672623	0.2947849
RPL21P24	NC_000001	47963534	47964053	0	0	0	0
LOC100287946	NC_000001	47986374	48016425	0	0.0784434	0.0678777	0
LOC100287982	NC_000001	48226979	48228507	0	0	0	0
LOC388630	NC_000001	48231309	48462461	0.0483213	0.0496794	0	0
SKINTL	NC_000001	48567387	48648100	0	0	0	0
SLC5A9	NC_000001	48688357	48714316	0.0402702	0.0138007	0.0238837	0.009354
LOC100128922	NC_000001	48727949	48728197	0	0	0	0
SPATA6	NC_000001	48764278	48937845	1.0614294	1.4809971	0.8318631	0.8276994
PPP1R8P	NC_000001	48790646	48791818	0	0	0	0
LOC644462	NC_000001	48901376	48903575	0	0	0	0
RPL21P25	NC_000001	48962929	48963408	0	0	0	0
AGBL4	NC_000001	48998527	50489626	0.1323742	0.3629189	0.1046789	0.317728
BEND5	NC_000001	49193539	49242547	0	0	0	0
ELAVL4	NC_000001	50513686	50667540	0	0	0.015874	0
RPS2P11	NC_000001	50791784	50792721	0	0	0	0
LOC100129919	NC_000001	50864531	50865445	0	0	0	0
DMRTA2	NC_000001	50883225	50889141	0.0566526	0.0291224	0	0.0197389
FAF1	NC_000001	50906935	51425936	1.7354702	2.2915314	2.2250647	3.6280344
MRPS6P2	NC_000001	51312141	51312514	0	0	0	0
LOC100288672	NC_000001	51426417	51434095	0.0803441	0.1652044	0.1429527	0.055987
CDKN2C	NC_000001	51434367	51440309	10.924984	10.484446	9.2458327	10.962174
C1orf185	NC_000001	51567906	51613754	0	0.0495976	0	0
CFLP2	NC_000001	51623317	51624212	0	0	0	0
LOC100128235	NC_000001	51662965	51663125	0	0	0	0
RNF11	NC_000001	51701945	51739119	4.4462915	3.7775323	4.1463261	7.1232232
RPS2P8	NC_000001	51716234	51717166	0	0	0	0
TTC39A	NC_000001	51752930	51810785	0.291556	0.1262106	1.3514871	0.6201966
EPS15	NC_000001	51819935	51984995	12.637221	12.282614	10.201319	11.02965
LOC441884	NC_000001	52016895	52060671	0	0	0	0
OSBPL9	NC_000001	52082764	52254141	7.0949019	10.748778	10.313278	11.567118
NRD1	NC_000001	52254864	52344609	1.2771841	2.390478	1.8160071	5.659441
RAB3B	NC_000001	52384832	52456348	4.1343053	15.691916	4.4085528	14.503406
RPS26P14	NC_000001	52446145	52446492	0	0	0	0
TXNDC12	NC_000001	52485803	52521047	12.470311	19.965416	18.546908	14.049163
KTI12	NC_000001	52497777	52499472	0	0	0	0

LOC100128489	NC_000001	52498820	52499631	0	0	0.1093638	0
BTF3L4	NC_000001	52521857	52554090	9.1832126	10.346903	14.088462	11.166
ZFYVE9	NC_000001	52608046	52812358	0.7166411	1.8907789	1.4517564	2.3344701
LOC100190934	NC_000001	52625678	52626273	0	0	0	0
LOC724060	NC_000001	52626715	52629154	0	0	0	0
RPL9P12	NC_000001	52655577	52655981	0	0	0	0
ANAPC10P	NC_000001	52719239	52720067	0	0	0	0
CC2D1B	NC_000001	52816267	52831864	1.1144879	0.8974182	1.0608135	1.9551261
LOC729225	NC_000001	52834180	52835939	0.0998823	0.1026895	0	0.1566045
ORC1L	NC_000001	52838501	52870131	1.1832752	2.4330625	1.3746689	2.105036
PRPF38A	NC_000001	52870219	52883992	2.7269466	4.1727817	4.5839498	5.1483381
ZCCHC11	NC_000001	52888947	53018743	2.5169255	2.7503614	3.4190258	4.3374742
LOC645311	NC_000001	53020450	53039493	0	0	0	0
GPX7	NC_000001	53068044	53074722	3.2235867	7.2543848	4.7159248	2.6207109
FAM159A	NC_000001	53099066	53122737	0.0629631	0.0647327	0	0.0438752
C1orf163	NC_000001	53152514	53164038	4.2205551	5.8228919	3.1006743	4.9523502
LOC100288149	NC_000001	53174794	53175114	0	0	0	0
ZYG11B	NC_000001	53192131	53293014	3.4811015	4.5666142	3.9803367	4.926758
RNU2-1	NC_000001	53219999	53220049	0	0	0	0
RPS13P2	NC_000001	53237825	53238346	0	0	0	0
ZYG11A	NC_000001	53308408	53358629	1.4480472	0.7443724	0.901756	1.0847362
ECHDC2	NC_000001	53361904	53387446	2.2067623	1.7688821	3.1610798	3.5967942
LOC729255	NC_000001	53386028	53386438	0	0	0	0
LOC100128507	NC_000001	53390429	53392714	0	0	0	0
SCP2	NC_000001	53392948	53517282	12.632405	11.686217	8.7503054	11.104248
LOC100133211	NC_000001	53458282	53459425	0	0.039496	0	0
PODN	NC_000001	53527885	53551174	6.438927	2.8370973	0	0.0101744
SLC1A7	NC_000001	53552855	53608289	0.1970037	0.0843919	0	0.03432
CPT2	NC_000001	53662101	53679869	2.9402986	3.8261316	3.3486926	3.5237417
C1orf123	NC_000001	53679771	53686289	10.04531	11.327083	9.9455559	8.7781909
MAGOH	NC_000001	53692564	53704207	9.2284833	15.990014	14.869706	12.636697
LRP8	NC_000001	53711212	53793821	2.07006	2.2753526	4.9306893	4.6864639
FLJ40434	NC_000001	53904043	53905693	0	0	0	0
DMRTB1	NC_000001	53925072	53933158	2.3045743	1.3742199	0.4305448	0.754782
GLIS1	NC_000001	53971906	54199877	0.2809191	0.1925429	0.0694204	0.0978778
TMEM48	NC_000001	54233388	54304175	7.3217998	12.295047	10.034163	16.666931
RPL37P7	NC_000001	54307218	54307507	0	0	0	0
YIPF1	NC_000001	54317406	54355453	6.4801717	6.4118353	8.9508285	9.3368531
DIO1	NC_000001	54359861	54376759	0.0472562	0.1214608	0.1471414	0.0823249
HSPB11	NC_000001	54387234	54411288	11.700028	11.878498	15.678059	23.440007
LRRC42	NC_000001	54412037	54433839	12.051149	10.91361	11.291304	10.363144
LDLRAD1	NC_000001	54474506	54483803	0	0.1151169	0	0.0780252
TMEM59	NC_000001	54497349	54519111	34.541859	35.725791	35.639644	37.811613
C1orf83	NC_000001	54519274	54565416	0.5899811	1.1359264	0.753895	1.3155915
CDCP2	NC_000001	54604668	54619443	0.355072	0.1161527	0.0574331	0.0899739
CYB5RL	NC_000001	54638009	54665746	1.4108979	0.8779653	1.629524	2.0957041
MRPL37	NC_000001	54665840	54684056	30.523721	43.538153	35.248446	60.403081
SSBP3	NC_000001	54692195	54872092	0.9645702	1.2189395	1.2514081	1.8204089
TALDOP1	NC_000001	54990059	54990887	0	0	0	0
LOC645436	NC_000001	55002469	55003431	0	0	0	0
ACOT11	NC_000001	55013901	55100417	0.3471003	0.2333286	0.3444196	0.5023522
FAM151A	NC_000001	55074850	55089200	0.0221849	0	0.0394726	0
C1orf175	NC_000001	55107427	55175939	0.0100453	0.0103276	0.0089366	0.0139999
TTC4	NC_000001	55181529	55207981	6.2689575	9.5891216	4.7997509	4.9773038
PARS2	NC_000001	55222571	55230187	0.936264	0.9433262	1.2827067	1.1743669
TTC22	NC_000001	55246752	55266941	0	0	0	0.0797263
C1orf177	NC_000001	55271736	55307937	0.0245658	0	0.0218544	0.0342369
DHCR24	NC_000001	55315300	55352921	17.975093	16.203194	24.839634	36.912767
LOC100288209	NC_000001	55316929	55331353	0.0425855	0.1751295	0.1136557	0.2670774
TMEM61	NC_000001	55446465	55457966	0.0400622	0	0.0712809	0.0279169
BSND	NC_000001	55464617	55474465	0.032079	0	0	0.0670618
PCSK9	NC_000001	55505220	55530523	1.7042631	2.9202694	0	0.0336907
USP24	NC_000001	55532032	55680762	2.3525769	2.8988457	3.9450915	6.2198705
LOC645506	NC_000001	55688052	55689045	0	0	0	0
GOT2L1	NC_000001	55833138	55834447	0	0	0	0
LOC100288281	NC_000001	55838381	55849472	0	0	0	0

RPSAP20	NC_000001	56673139	56674201	0	0	0	0
LOC100288320	NC_000001	56714743	56719237	0	0	0	0.1890425
PPAP2B	NC_000001	56960433	57045257	86.081965	68.090591	25.364753	41.446784
LOC100288348	NC_000001	56977751	57045659	1.1475372	0.6275472	0.1520461	0.6295114
RPL21P23	NC_000001	57004084	57004629	0	0	0	0
PRKAA2	NC_000001	57110990	57181008	0.4887314	0.4348274	1.4423289	2.0270316
C1orf168	NC_000001	57184477	57285369	0	0	0.0225997	0.070809
C8A	NC_000001	57320443	57383894	0.018373	0	0.0163451	0
C8B	NC_000001	57394883	57431688	0	0	0.0383874	0.0601372
DAB1	NC_000001	57463579	58716211	0.7665389	1.313471	1.9245701	2.3028009
RPS20P5	NC_000001	58071485	58071998	0	0	0	0
LOC100288923	NC_000001	58343974	58346412	0	0	0	0
HNRNPA1L1	NC_000001	58513521	58515045	0	0	0	0
RPS26P15	NC_000001	58521716	58522174	0	0	0	0
VKORC1P2	NC_000001	58694077	58694673	0	0	0	0
OMA1	NC_000001	58946391	59012446	1.8647495	2.1065075	3.0516172	3.7378715
TACSTD2	NC_000001	59041095	59043166	0.1484738	0	0.0566084	0.044341
AK2P1	NC_000001	59095601	59097250	0	0	0	0
MYSM1	NC_000001	59125590	59165747	2.3255727	2.8970432	3.6394412	4.1755821
JUN	NC_000001	59246463	59249785	23.871971	24.474909	20.64888	16.7271
LOC100288387	NC_000001	59247439	59248986	0.4059883	0.3826154	0.1805892	0.1650301
LOC100131060	NC_000001	59250823	59365384	0	0	0	0
LOC391044	NC_000001	59465348	59466166	0	0	0	0
LOC729467	NC_000001	59597608	59612479	0	0	0	0
FGGY	NC_000001	59762625	60228402	1.7697275	1.2129772	1.180799	2.1287667
HOOK1	NC_000001	60280533	60342050	0.0075048	0.030863	0.013353	0.015689
CYP2J2	NC_000001	60358980	60392423	1.0176489	0.6569477	1.494845	0.3298318
C1orf87	NC_000001	60456066	60539426	0.0655292	0	0.0194322	0
NFIA	NC_000001	61542946	61928460	0.2280064	0.3967015	0.3510706	0.7608096
MGC34796	NC_000001	62119865	62121190	0	0	0	0
TM2D1	NC_000001	62146719	62191095	5.1331535	4.6629271	10.102811	5.1449796
INADL	NC_000001	62208149	62629592	0.227363	0.3400044	0.7631036	0.4392987
LOC100288454	NC_000001	62654878	62655682	0	0	0	0
RPS15AP7	NC_000001	62656164	62656643	0	0	0	0
L1TD1	NC_000001	62660496	62678000	0.2051525	0.0351531	0.0405576	0.0158843
KANK4	NC_000001	62701837	62785083	0	0.0164993	0.0071385	0.1565632
USP1	NC_000001	62901975	62917475	8.4797684	12.366777	10.77558	13.160381
DOCK7	NC_000001	62920397	63153969	6.1260794	7.6822035	5.4277534	7.3782853
ANGPTL3	NC_000001	63063187	63071180	0	0	0	0.0288099
RPL13AP9	NC_000001	63106788	63107364	0	0	0	0
ATG4C	NC_000001	63249803	63330050	1.3619817	2.7755161	1.9905789	2.0168016
FOXD3	NC_000001	63788730	63790797	0.0212516	0.0873954	0.018906	0.0296179
LOC441887	NC_000001	63825460	63833824	0	0	0	0
ALG6	NC_000001	63833261	63904233	0.7561547	1.4609881	1.6817404	1.5625865
ITGB3BP	NC_000001	63906462	63988835	5.0997948	4.7804964	10.141348	10.487017
EFCAB7	NC_000001	63989013	64038364	0.8562553	0.9951448	1.2751006	1.5046531
DLEU2L	NC_000001	64014651	64016307	0	0	0	0
PGM1	NC_000001	64058947	64125916	22.614139	18.278075	18.346754	26.548994
ROR1	NC_000001	64239690	64644707	3.0032717	3.4063362	1.5498305	1.7651012
RPL19P3	NC_000001	64254312	64255005	0	0	0	0
CFLP3	NC_000001	64308662	64309478	0	0	0	0
UBE2U	NC_000001	64669490	64710027	0	0.0795482	0.0344169	0
RNU7-62P	NC_000001	64849982	64850241	0	0	0	0
CACHD1	NC_000001	64936476	65158741	1.3082807	1.7819772	1.1638828	1.0103771
RAVER2	NC_000001	65210778	65298915	1.5818139	1.4294611	2.1959889	2.0571044
JAK1	NC_000001	65298906	65432187	18.473391	26.73627	13.726317	25.873264
LOC100130270	NC_000001	65384125	65384368	0	0	0	0
SLC2A3P2	NC_000001	65448479	65451791	0	0	0	0
LOC100288548	NC_000001	65514385	65533419	0.5539693	0.1898462	0.0547585	0.3002439
LOC100129205	NC_000001	65543083	65543814	0	0	0	0
MRPS21P1	NC_000001	65557885	65558368	0	0	0	0
AK3L1	NC_000001	65613232	65693177	4.9850692	4.0040431	3.4647311	5.6811019
RPS29P7	NC_000001	65620133	65620421	0	0	0	0
LOC645195	NC_000001	65713847	65721850	0	0	0	0
DNAJC6	NC_000001	65730430	65881552	0.6114537	0.3378933	2.4070501	2.4127078
LEPR	NC_000001	65886318	66102822	1.0149386	0.7361421	2.1954501	2.0926839

LEPROT	NC_000001	65886319	65898264	6.1688451	3.5059484	5.249368	4.5122968
PDE4B	NC_000001	66258193	66840262	1.2561455	0.9707541	0.607501	0.2232391
SGIP1	NC_000001	66999825	67210768	0.5617584	0.4909147	0.0416463	0.0782911
TCTEX1D1	NC_000001	67218142	67244470	0.5488041	0.0677074	0.3515266	0.1988628
INSL5	NC_000001	67263424	67266939	0	0.0631936	0	0.042832
WDR78	NC_000001	67278568	67390570	0.4555948	0.4683994	0.2965681	0.1780966
MIER1	NC_000001	67390642	67454302	1.1832216	1.0584923	1.2781896	1.4509339
SLC35D1	NC_000001	67469851	67519728	2.7985902	4.4046713	2.8892855	2.1427784
C1orf141	NC_000001	67557859	67594220	0.0615234	0	0.0729773	0.0857439
IL23R	NC_000001	67632169	67725662	0.5442987	0.3997116	0.4842232	0.5310046
LOC100130497	NC_000001	67743735	67744462	0	0	0	0
IL12RB2	NC_000001	67773047	67862583	0.0761479	0.022368	0.0290328	0.0606433
SERBP1	NC_000001	67873493	67896123	1.7218039	2.9993122	2.8207587	5.7093394
LOC100288621	NC_000001	68026806	68027130	0	0	0	0
LOC100130196	NC_000001	68108396	68126698	0	0	0	0
GADD45A	NC_000001	68150883	68154019	10.541089	10.203779	7.1270101	3.8874384
GNG12	NC_000001	68167149	68299142	35.830406	38.111932	29.687824	50.263534
RNU7-80P	NC_000001	68238277	68238335	0	0	0	0
LOC100133029	NC_000001	68509015	68509920	0	0	0	0
DIRAS3	NC_000001	68511645	68516460	0.4580748	1.8283903	0.5273737	0.1314372
GPR177	NC_000001	68564151	68698253	13.194553	13.284009	11.699796	12.697631
LOC100289178	NC_000001	68668176	68668658	0	0.0935474	0	0
RPS7P4	NC_000001	68708112	68708782	0	0	0	0
LOC100133210	NC_000001	68748051	68748306	0	0	0	0
LOC100130626	NC_000001	68841005	68841345	0	0	0	0
RPE65	NC_000001	68894507	68915642	0.0168513	0.0346498	0.0299828	0.0117427
DEPDC1	NC_000001	68939835	68962799	6.6008876	13.894765	7.3532455	9.2684331
AF357533	NC_000001	68979427	68980526	0	0	0	0
LOC100133218	NC_000001	70072651	70073984	0	0	0	0
LRRC7	NC_000001	70225858	70589171	0.0492614	0.0072351	0.0626062	0.049039
PIN1L	NC_000001	70385005	70386000	0	0	0	0
LRRC40	NC_000001	70610497	70671275	4.2913974	6.4593061	4.380795	4.8621163
SFRS11	NC_000001	70671365	70717684	2.8031845	2.1818291	3.4518571	4.4916488
ANKRD13C	NC_000001	70724685	70820417	1.4502063	2.4078679	1.759287	1.9941027
HHLA3	NC_000001	70820493	70833706	2.1974116	1.9077436	2.5196209	1.8715203
CTH	NC_000001	70876955	70905253	2.6052414	1.827362	2.8592126	1.0519349
LOC391048	NC_000001	70996092	70998157	0	0	0	0
LOC100131616	NC_000001	71122804	71132322	0	0	0	0
PTGER3	NC_000001	71318036	71513491	0.0256507	0.7340105	0.0114098	0.0178744
LOC100132618	NC_000001	71512189	71532867	0	0	0	0
ZRANB2	NC_000001	71528974	71546745	9.0065259	7.6957336	11.911493	13.937573
NEGR1	NC_000001	71868625	72748277	3.0931989	3.6424087	0.6620861	2.0960404
RPL31P12	NC_000001	72767128	72767554	0	0	0	0
LOC100288863	NC_000001	73444897	73445064	0	0	0	0
KRT8P21	NC_000001	73570462	73572167	0	0	0	0
LRRIQ3	NC_000001	74491699	74663871	0.1380573	0.0315416	0.1228196	0.2030969
FPGT	NC_000001	74663926	74673120	0.0256557	0.0131884	0.011412	0.1251454
TNNI3K	NC_000001	74663947	75010108	0.4185546	0.2017116	0.453811	0.5104146
C1orf173	NC_000001	75033795	75139422	0.0184166	0.0315571	0.0163839	0.0256669
CRYZ	NC_000001	75171170	75199092	1.277454	2.2150648	3.5789962	4.25161
TYW3	NC_000001	75198840	75231308	1.6619079	2.4490163	2.3162844	5.1341708
LHX8	NC_000001	75594119	75627218	0	1.3138031	0.016476	0.0129056
SLC44A5	NC_000001	75667816	76076799	0.0104044	0.0748778	0.0277682	0.0290008
RPL29P5	NC_000001	76047736	76048357	0	0	0	0
ACADM	NC_000001	76190043	76229355	6.655258	6.2202771	7.1018523	5.4340128
DLSTP	NC_000001	76207691	76210467	0	0	0	0
RABGGTB	NC_000001	76251886	76260764	6.9917641	9.1514511	14.975204	18.239817
SNORD45C	NC_000001	76252757	76252834	0	0	0	0
SNORD45A	NC_000001	76253574	76253657	0	0	0	0
SNORD45B	NC_000001	76255162	76255232	0	0	0	0
MSH4	NC_000001	76262630	76378923	0	0.0282132	0.0976524	0.1051744
ASB17	NC_000001	76384558	76398116	0	0.0408161	0.0353185	0.0553295
ST6GALNAC3	NC_000001	76540404	77096632	0.0685192	0.7185387	0.2438264	0.0381975
RCTPI1	NC_000001	77165449	77166665	0	0	0	0
ST6GALNAC5	NC_000001	77333186	77529737	0	0.2223593	0.0192409	0.0452139
LOC256483	NC_000001	77533611	77544260	0	0	0	0

PIGK	NC_000001	77554666	77685132	7.6952157	11.369105	7.4713031	7.3285215
RPL17P6	NC_000001	77594759	77595389	0	0	0	0
AK5	NC_000001	77747742	78025654	5.8150069	4.9484055	1.2220861	3.8434042
RNU7-8P	NC_000001	77886011	77886069	0	0	0	0
ZZZ3	NC_000001	78030190	78148343	7.7974309	7.4103706	9.7585643	11.575538
USP33	NC_000001	78161672	78225537	3.3909199	4.4191547	3.8154329	4.8922594
LOC100131291	NC_000001	78239550	78240546	0	0	0	0
FAM73A	NC_000001	78245309	78344077	2.0641212	2.7836893	2.4384867	2.5215007
C1orf118	NC_000001	78347033	78355218	0	0	0	0
NEXN	NC_000001	78354200	78409580	0.3110462	0.4263842	0.1729471	1.1650278
FUBP1	NC_000001	78413591	78444777	6.7202393	9.0241468	8.3916065	9.9605187
LOC100288957	NC_000001	78444781	78463008	0.9334138	0.9596475	0.0691992	0.8130499
DNAJB4	NC_000001	78470636	78482998	17.265377	7.1607624	2.4610521	3.7460789
LOC100131495	NC_000001	78509061	78511670	0	0	0	0
GIPC2	NC_000001	78511589	78603112	0.4359017	0.1440491	0.0138497	0.0542417
LOC100132264	NC_000001	78636093	78636880	0	0	0	0
MGC27382	NC_000001	78695306	78835149	0	0	0	0
PTGFR	NC_000001	78956728	79006386	0.3641448	0.6572434	1.4469915	2.1089495
IFI44L	NC_000001	79086088	79111830	18.592332	5.1537077	28.095135	1.8769078
IFI44	NC_000001	79115477	79129763	26.022649	7.9057864	34.272663	7.4166855
RPL23P3	NC_000001	79131956	79132382	0	0	0	0
LOC652549	NC_000001	79210408	79216344	0	0	0	0
ELTD1	NC_000001	79355449	79472495	4.1774501	7.7657021	1.4368544	0.0930847
LOC729779	NC_000001	79520630	79551669	0	0	0	0
ADH5P2	NC_000001	79986754	79988152	0	0	0	0
LOC553139	NC_000001	80555862	80558256	0	0	0	0
LOC646526	NC_000001	80749037	80749657	0	0	0	0
LOC100129325	NC_000001	80916767	80918530	0	0	0	0
COX6A1P	NC_000001	80961574	80962109	0	0	0	0
RPL7P10	NC_000001	81563880	81564711	0	0	0	0
RPL10AP4	NC_000001	81674220	81674934	0	0	0	0
LOC729817	NC_000001	81892142	81893297	0	0	0	0
LOC646555	NC_000001	81967270	81971426	0	0	0	0
LOC646556	NC_000001	82022802	82024806	0	0	0	0
RPS20P7	NC_000001	82061788	82062311	0	0	0	0
LOC729828	NC_000001	82155285	82188767	0	0	0	0
LPHN2	NC_000001	82266082	82458107	5.5378161	15.377877	3.1382203	3.037799
TTLL7	NC_000001	84335057	84464833	2.4455842	2.2542158	3.6546792	4.4493381
PRKACB	NC_000001	84543745	84704181	1.9353206	2.9805418	3.4573708	3.7018484
LOC100128775	NC_000001	84710017	84710495	0	0	0	0
SAMD13	NC_000001	84764049	84816481	0.1462504	0.0751804	0.3035862	0.2547827
UOX	NC_000001	84830641	84863576	0	0	0	0
DNASE2B	NC_000001	84864215	84880691	0.0980258	0	0	0.0910777
BXDC5	NC_000001	84944920	84964033	0.912343	1.0523729	0.752257	1.1474638
GNG5	NC_000001	84964006	84972262	25.245696	30.944464	28.910855	50.306872
SPATA1	NC_000001	84971974	85031875	0.2440336	0.3649341	0.7697148	0.355564
CTBS	NC_000001	85018804	85040163	4.9461248	3.3220947	4.8793157	2.8837357
LOC100289225	NC_000001	85101841	85102782	0	0	0	0
SSX2IP	NC_000001	85109596	85156180	3.0828541	3.5546523	3.9715517	5.0959884
LPAR3	NC_000001	85279086	85358896	0.0255365	0.0525083	0	0.0355896
MCOLN2	NC_000001	85391266	85462796	0.4186921	0.6234241	0.9633106	1.0463165
MCOLN3	NC_000001	85483765	85514169	2.9550313	3.7006434	4.4327354	6.7142526
WDR63	NC_000001	85527993	85598821	0.3946133	0.3005215	0.0520087	0.0916608
SYDE2	NC_000001	85623356	85666728	0.6436909	0.5370984	0.6141413	0.6565724
C1orf52	NC_000001	85715636	85725355	0.7136202	0.7059907	0.4911153	0.6474009
BCL10	NC_000001	85731459	85743583	2.2122499	3.0510584	2.2920918	2.85757
LOC646626	NC_000001	85742123	85743771	0	0	0.0522695	0
DDAH1	NC_000001	85784168	86044046	16.669343	17.490674	7.9992119	17.690274
LOC100288581	NC_000001	85824449	85930842	0	0.0894721	0.1548418	0.0606433
LOC100289301	NC_000001	86046382	86047260	0	0.167968	0.5813764	0.2846178
CYR61	NC_000001	86046444	86049650	90.711448	134.29019	53.356678	95.544283
LOC100288614	NC_000001	86047677	86048576	0.1831176	0.1255094	0.1629065	0.2126728
ZNHIT6	NC_000001	86118491	86174101	0.3142526	0.3230847	0.2655895	1.0730204
COL24A1	NC_000001	86194916	86622121	0.101419	0.1042694	0.0781951	0.0800958
ODF2L	NC_000001	86814420	86862003	1.4170672	1.4765818	1.5502738	2.0816911
CLCA2	NC_000001	86889769	86922241	0.2838892	0.0561284	0.0777094	0.0304347

CLCA1	NC_000001	86934526	86965977	0.0281449	0	0	0
CLCA4	NC_000001	87012759	87046437	0.0136783	0.0562507	0	0.0190631
LOC100129348	NC_000001	87016999	87019246	0	0	0	0
CLCA3P	NC_000001	87099959	87121059	0	0	0	0
SH3GLB1	NC_000001	87170257	87213867	3.682466	3.6867047	3.2576192	4.6372207
RPL17P5	NC_000001	87250561	87251191	0	0	0	0
SEP15	NC_000001	87328128	87380107	20.302172	25.882231	23.671026	25.298812
HS2ST1	NC_000001	87380335	87575681	2.9460136	3.7067182	3.6164576	4.7844786
LOC339524	NC_000001	87595448	87634887	0	0	0	0
LMO4	NC_000001	87794151	87814606	6.0088332	13.835066	6.4017291	5.983325
RPL36AP10	NC_000001	89043528	89043934	0	0	0	0
PKN2	NC_000001	89149922	89301938	3.0023612	3.3014085	4.4772605	4.6509276
GTF2B	NC_000001	89318321	89357301	5.5035476	7.3062522	7.2966277	6.6462502
LOC100130455	NC_000001	89357267	89389713	0	0	0	0
CCBL2	NC_000001	89401456	89458516	3.0941794	3.1012135	4.0667555	6.3221711
GBP3	NC_000001	89472360	89488549	1.3878389	1.3971183	0.8616898	0.8159918
GBP1	NC_000001	89517987	89531043	3.87609	2.5480476	5.3070138	1.3053227
LOC100288649	NC_000001	89524571	89525960	0.1417685	0.2915058	0.1261212	0.049395
LOC284672	NC_000001	89568802	89570637	0	0	0	0
GBP2	NC_000001	89573310	89591799	5.1992706	4.6141046	31.880712	6.5262265
GBP7	NC_000001	89597434	89641723	0	0.0368243	0.0159322	0.0249592
GBP4	NC_000001	89646831	89664633	0.0214556	0.0073529	0.12725	0.0149511
GBP5	NC_000001	89724633	89738544	0.542303	0.401432	3.9271247	0.0680217
LOC729930	NC_000001	89754941	89756020	0	0	0	0
GBP6	NC_000001	89829436	89853719	0.0180597	0.0185672	0.0240996	0.0251694
LOC391053	NC_000001	89859526	89860225	0	0	0	0
LOC400759	NC_000001	89873238	89890493	0	0	0	0
LRRC8B	NC_000001	89990397	90063420	0.3543773	0.4372045	0.819686	0.6914437
LRRC8C	NC_000001	90098644	90185094	1.0305607	2.2820533	1.1989124	1.8654532
LRRC8D	NC_000001	90286573	90401991	3.4690267	4.4247189	4.0987828	4.3361322
LOC492303	NC_000001	90458824	90460525	0	0	0	0
ZNF326	NC_000001	90460678	90494094	0.9419881	1.3258717	0.409033	1.1096537
BARHL2	NC_000001	91177579	91182794	0	0.0302028	0.0261347	0.0818847
PHKA1P1	NC_000001	91358412	91359583	0	0	0	0
ZNF644	NC_000001	91380855	91487671	3.7409169	5.086719	4.3814502	5.4764241
RPL5P6	NC_000001	91489432	91490424	0	0	0	0
HFM1	NC_000001	91726323	91870426	0.0089126	0.0274894	0.0396447	0.1117923
LOC645931	NC_000001	91793799	91795820	0	0	0	0
CDC7	NC_000001	91966404	91991321	2.6253117	4.1677223	3.8696859	4.8605224
WDR82P2	NC_000001	91998056	92001411	0	0	0	0
RPL39P13	NC_000001	92065669	92066061	0	0	0	0
HSP90B3P	NC_000001	92100568	92109335	0	0	0	0
TGFBR3	NC_000001	92145900	92351787	3.1567677	5.2519193	1.9493956	1.7273615
LOC100289437	NC_000001	92327029	92352647	0.7398692	1.4452603	0.5265665	0.3093422
BRDT	NC_000001	92414928	92479985	0.0254477	0	0.0113195	0.035466
EPHX4	NC_000001	92495533	92529093	0.3350177	0.2818092	1.354732	0.9974839
LOC100128094	NC_000001	92531788	92537630	0	0	0	0
LOC646817	NC_000001	92540011	92541029	0.0431288	0.0443409	0.1151057	0.0300539
BTBD8	NC_000001	92545862	92613397	0.5177546	0.2818092	0.4606089	0.1485614
LOC100240711	NC_000001	92580196	92581389	0	0	0	0
PRKAR1AP	NC_000001	92590712	92593107	0	0	0	0
KIAA1107	NC_000001	92632609	92650280	0.4630191	0.2975202	0.5835458	0.5041409
C1orf146	NC_000001	92683573	92711367	0	0	0.0520607	0.0407788
GLMN	NC_000001	92711955	92764566	1.3193121	1.6454585	0.7311553	2.2004096
RPAP2	NC_000001	92764607	92853729	1.2207842	2.1665322	1.3187671	2.4305459
GFI1	NC_000001	92940318	92952433	0.0453075	0.0155269	0.0268712	0.0420961
EVI5	NC_000001	92974253	93257961	0.5699082	1.2634019	0.6707268	0.7942694
LOC100289500	NC_000001	93176907	93198819	0	0	0	0
RPL5	NC_000001	93297594	93307481	70.419475	125.86492	89.913024	152.4412
SNORD21	NC_000001	93302846	93302940	0	0	0	0
SNORA66	NC_000001	93306276	93306408	0	0	0	0
FAM69A	NC_000001	93307721	93427079	3.58485	3.8594518	4.979336	5.7620491
LOC100127934	NC_000001	93396842	93397648	0	0	0	0
MTF2	NC_000001	93544792	93604638	1.0433447	1.2040151	2.0079176	1.6766527
TMED5	NC_000001	93617407	93646246	12.205991	8.9864385	15.097569	13.96965
CCDC18	NC_000001	93645920	93744270	0.764235	1.0767191	0.7134592	1.4727292

RPL36AP11	NC_000001	93656247	93656643	0	0	0	0
LOC100130531	NC_000001	93744518	93744686	0	0	0	0
LOC100131564	NC_000001	93810844	93812070	0	0	0	0
DR1	NC_000001	93811478	93828149	13.366617	17.163779	13.657953	17.569461
FNBP1L	NC_000001	93965025	94020218	1.097005	1.9846425	1.1801897	0.4325883
BCAR3	NC_000001	94027348	94147385	6.9990088	11.242417	5.9305989	12.757952
LOC100129046	NC_000001	94054668	94065587	0.0237686	0.0244367	0.0211452	0
LOC100132240	NC_000001	94218482	94219271	0	0	0	0
LOC100288818	NC_000001	94312732	94314495	0.0996559	0.1792992	0.288134	0.2256935
DNTTIP2	NC_000001	94335336	94344742	1.1818012	1.9933854	1.3634865	2.2646969
GCLM	NC_000001	94352590	94375012	5.1611294	4.1890922	2.988916	3.7758064
LOC100289636	NC_000001	94392186	94443360	0	0	0	0
ABCA4	NC_000001	94458393	94586705	0.0479915	0.0616754	0.0373578	0.0627045
ARHGAP29	NC_000001	94634463	94703307	4.9217006	9.2032003	13.641478	23.789118
LOC441893	NC_000001	94767526	94768783	0	0	0	0
RPL21P26	NC_000001	94871943	94872478	0	0	0	0
ABCD3	NC_000001	94883933	94984219	2.0834031	3.6651272	3.7069064	3.3472016
F3	NC_000001	94994732	95007371	27.311087	46.221217	3.3759276	8.6364502
LOC100130235	NC_000001	95116111	95137787	0	0	0	0
SLC44A3	NC_000001	95285901	95360802	0.4270135	0.1053635	0.4102737	0.3332672
CNN3	NC_000001	95362507	95392735	60.26396	63.444845	57.075633	48.57586
LOC729970	NC_000001	95409643	95428826	0.7433105	0.573151	2.0664675	0.7769525
ALG14	NC_000001	95448279	95538507	2.453118	3.8044681	2.8111779	5.8236524
TMEM56	NC_000001	95582894	95663163	0.727961	0.384145	3.5074333	3.4072534
RWDD3	NC_000001	95699711	95712781	1.2285848	2.2375167	1.7487729	1.9568612
LOC729977	NC_000001	95776484	95783819	0	0	0.1023496	0
LOC100132258	NC_000001	96853250	96884852	0	0	0	0
EEF1AL9	NC_000001	96912486	96914179	0	0	0	0
LOC100286918	NC_000001	97042713	97050290	0.0718108	0	0	0
RPL7P9	NC_000001	97144364	97145196	0	0	0	0
PTBP2	NC_000001	97187175	97280605	2.4205567	3.097772	3.7460089	4.0235784
DPYD	NC_000001	97543299	98386615	5.2860396	8.4718879	3.6741867	4.1448239
RPL26P9	NC_000001	98051422	98051856	0	0	0	0
FLJ35409	NC_000001	98508712	98511008	0	0	0	0
LOC100132699	NC_000001	98542358	98543074	0	0	0	0
SNX7	NC_000001	99127236	99226056	7.8305231	10.027524	8.8211693	11.526707
PAP2D	NC_000001	99355801	99470449	0.010946	0.0787755	0.0194757	0.0228828
LPPR4	NC_000001	99729900	99775140	1.6794377	1.417661	0.2594971	0.1478272
LOC100287018	NC_000001	99927438	99930334	0	0	0	0
PALMD	NC_000001	100111431	100160097	0.1534267	0.0350531	0.0606634	0.0831552
LOC100129320	NC_000001	100163786	100164776	0	0	0	0
FRRS1	NC_000001	100174259	100231349	1.444154	2.7164032	1.3723567	1.3379801
RPL39P9	NC_000001	100308166	100308318	0	0	0	0
AGL	NC_000001	100315640	100389579	0.9359791	0.9677837	0.7755754	0.8423053
LOC730081	NC_000001	100433823	100436074	0	0	0	0
SLC35A3	NC_000001	100435540	100489006	3.6197473	4.2681389	3.6204819	4.8452576
HIAT1	NC_000001	100503789	100548933	10.81649	11.377116	9.311764	7.3518261
SASS6	NC_000001	100549100	100598511	1.0144501	1.7266804	1.7648555	2.097164
CCDC76	NC_000001	100598719	100616053	0.7725001	1.2129772	0.8496754	1.311516
LRRC39	NC_000001	100614413	100643771	0.126288	0.1298374	0.0280873	0.1540044
DBT	NC_000001	100652478	100715409	0.5445273	0.6935224	0.4627359	0.5776676
BRI3P1	NC_000001	100678768	100679506	0	0	0	0
RTCD1	NC_000001	100731714	100758325	2.2619181	2.049876	2.0271708	3.6194099
LOC646970	NC_000001	100797341	100798172	0	0	0	0
CDC14A	NC_000001	100818023	100985833	0.5623939	0.5363015	0.2537861	0.2271875
GPR88	NC_000001	101003728	101007583	0.0380504	0.0260799	0	0.0088383
RPL7AP17	NC_000001	101052160	101053016	0	0	0	0
LOC100128787	NC_000001	101089213	101106298	0	0	0	0
RPL36AP12	NC_000001	101117441	101117873	0	0	0	0
VCAM1	NC_000001	101185297	101204601	6.1062824	0.3204497	0.3024957	0
EXTL2	NC_000001	101337941	101360418	4.3080532	4.6482405	3.4669126	3.6066708
SLC30A7	NC_000001	101361632	101447311	2.7815337	2.8927056	3.4262716	3.8281098
DPH5	NC_000001	101455179	101491362	3.0627639	5.547962	4.6925729	7.3005103
LOC100131646	NC_000001	101701239	101702820	0	0.1523892	0	0.051644
S1PR1	NC_000001	101702305	101707076	1.7867478	6.2812336	0.4614795	0.2309417
RPS20P6	NC_000001	101721832	101722179	0	0	0	0

PPIAP7	NC_000001	101736182	101737051	0	0	0	0
RPSAP19	NC_000001	102251822	102252854	0	0	0	0
OLFM3	NC_000001	102268127	102462790	0	0.052205	0.0112933	0.026538
HEJ1	NC_000001	102358660	102359604	0	0	0	0
COL11A1	NC_000001	103342023	103574052	0.1475962	0.0789071	0.0525223	0.0370263
RNPC3	NC_000001	104068578	104097861	0.81515	1.3169513	1.3882019	2.0935926
AMY2B	NC_000001	104097322	104122151	0.120903	0.1450178	0.1434115	0.1123333
LOC648740	NC_000001	104112026	104114013	0	0	0	0
AMY2A	NC_000001	104159999	104168400	0	0.0276859	0	0.0375305
AMY1A	NC_000001	104198141	104207173	0	0.0223349	0	0.0302767
AMY1B	NC_000001	104230041	104238889	0.0478218	0	0	0.0166621
AMYP1	NC_000001	104256342	104262511	0	0	0	0
AMY1C	NC_000001	104292441	104301310	0.0236281	0	0	0
LOC100131348	NC_000001	104460506	104470449	0	0	0	0
LOC100129138	NC_000001	104523665	104619709	0	0	0	0
LOC642337	NC_000001	104696151	104696731	0	0.0777683	0	0.1054213
CDK4PS	NC_000001	105976616	105977443	0	0	0	0
LOC401957	NC_000001	106433988	106607196	0	0	0	0
LOC126987	NC_000001	106623234	106626422	0	0	0	0.0371211
LOC100289022	NC_000001	107345372	107346048	0	0	0	0
LOC100287317	NC_000001	107346757	107346918	0	0	0	0
PRMT6	NC_000001	107599409	107601914	1.8764808	3.1732956	1.2481266	1.8819758
NTNG1	NC_000001	107682629	108024475	2.6543367	2.962846	3.0472937	1.9729574
NDUFA4P1	NC_000001	108047750	108048250	0	0	0	0
VAV3	NC_000001	108113782	108507545	0.1444774	0.0464181	0.369527	0.10697
SLC25A24	NC_000001	108677445	108742974	4.4845134	5.0716063	5.0919225	4.9424146
NBPF4	NC_000001	108765087	108786703	0.0390651	0.0267753	0	0.0181481
LOC727941	NC_000001	108815761	108880475	0	0	0	0
NBPF6	NC_000001	108992904	109013260	0.2373442	0.0203346	0	0
LOC642818	NC_000001	109044980	109046077	0	0	0	0
FAM102B	NC_000001	109102971	109181949	2.6171419	3.206837	5.0958625	3.7965673
C1orf59	NC_000001	109190910	109204148	0.990504	1.5717891	1.2068332	2.250726
PRPF38B	NC_000001	109234932	109244425	0.3794774	0.3657588	0.3586933	1.322175
FNDC7	NC_000001	109255556	109285371	0.0860605	0.0147465	0.0765618	0.0699655
STXBP3	NC_000001	109289285	109352148	2.9848979	3.5561846	2.8585116	4.0865798
LOC100271656	NC_000001	109305143	109310209	0	0	0	0
C1orf62	NC_000001	109358523	109399716	0.1008648	0.0148142	0.102551	0.0602457
LOC642864	NC_000001	109400007	109401146	0	0	0	0
GPSM2	NC_000001	109419603	109473044	6.3031589	7.4188375	8.4305334	9.1581815
CLCC1	NC_000001	109472130	109506111	0.9816772	0.9531969	1.4231999	2.2802391
WDR47	NC_000001	109512836	109584850	3.2740228	3.3144132	3.1092214	2.9603116
RPL17P7	NC_000001	109534876	109535499	0	0	0	0
RPS27P6	NC_000001	109572654	109572998	0	0	0	0
LOC100129428	NC_000001	109589448	109590105	0	0	0	0
TAF13	NC_000001	109606998	109618624	13.25302	10.023354	10.706092	9.8190684
TMEM167B	NC_000001	109633403	109639554	4.6025785	4.4690492	3.6538452	3.9979387
NDUFB3P1	NC_000001	109635089	109635662	0	0	0	0
SCARNA2	NC_000001	109642815	109643234	0	0	0	0
C1orf194	NC_000001	109648573	109656479	0	0	0	0.083789
KIAA1324	NC_000001	109656533	109745854	0.1422691	0.1994559	0.0460242	0.0721009
SARS	NC_000001	109756540	109780785	30.984083	37.243522	36.167827	33.58232
CELSR2	NC_000001	109792641	109818378	0.1167505	0.1028844	0.7567271	0.554967
PSRC1	NC_000001	109822178	109825771	4.683605	5.4888727	5.850602	5.7157421
MYBPHL	NC_000001	109834987	109849663	0	0	0.0284968	0
SORT1	NC_000001	109852192	109940563	3.1373702	1.100935	4.5403984	7.57549
PSMA5	NC_000001	109944472	109969037	40.022671	54.090192	52.54488	51.942812
SYPL2	NC_000001	110009100	110024764	1.1280705	0.4782578	0.2276122	0.4943418
ATXN7L2	NC_000001	110026561	110035426	0.9017853	1.0784983	0.9168608	0.9874856
CYB561D1	NC_000001	110036658	110043063	1.6362343	1.375566	0.6444237	0.9560996
AMIGO1	NC_000001	110049446	110052336	0.2667841	0.4937078	0.8385961	0.9295289
GPR61	NC_000001	110082494	110088455	0.0155679	0.0160055	0.0276993	0.0433934
GNAI3	NC_000001	110091186	110138452	10.620048	12.513796	10.357135	13.816806
RNU6V	NC_000001	110133927	110134262	0	0	0	0
GNAT2	NC_000001	110145889	110155705	0.193179	0.165507	0.1432145	0.3365371
AMPD2	NC_000001	110162459	110174677	5.8815477	4.703105	4.8562032	5.8396881
LOC100289114	NC_000001	110173686	110174678	0	0.0910038	0.1574927	0.0308408

RPL7P8	NC_000001	110193985	110194480	0	0	0	0
GSTM4	NC_000001	110198698	110208123	4.1265945	2.7341026	6.3836922	4.0098302
GSTM2	NC_000001	110210644	110226619	3.288495	3.1794069	4.9792242	2.9744679
GSTM1	NC_000001	110230418	110236367	7.5286844	8.1978324	21.874841	15.015236
GSTM5	NC_000001	110254864	110260890	0.11197	0	0.2241262	0.0585189
GSTM3	NC_000001	110276554	110283660	1.9061627	2.605682	2.44419	0.6010698
EPS8L3	NC_000001	110292702	110306564	0	0.0201622	0.0174465	0.0273314
LOC100287424	NC_000001	110353262	110358345	0	0	0	0
CSF1	NC_000001	110453233	110472355	20.230138	4.6617796	15.921505	14.75928
AHCYL1	NC_000001	110527308	110566364	13.91002	15.491773	21.862363	25.020724
FAM40A	NC_000001	110577241	110597263	5.2139686	5.7899025	2.960484	3.6614661
ALX3	NC_000001	110602997	110613322	0.1189397	0.0917119	0.0793591	0.2072049
UBL4B	NC_000001	110655062	110656569	0	0	0	0.0406165
SLC6A17	NC_000001	110693132	110744824	0.5216407	1.7006403	2.3142242	3.7545728
LOC100130800	NC_000001	110709429	110715118	0.054662	0	0	0
KCNC4	NC_000001	110754065	110776666	0.7922679	1.5243435	0.7450991	1.0568462
LOC100289210	NC_000001	110880020	110880943	0.3805042	0	0.3385071	0.3977257
RBM15	NC_000001	110881945	110889303	2.1775574	2.4231262	3.5667555	5.4001898
SLC16A4	NC_000001	110905505	110933636	6.0474435	0.1250628	5.503651	1.8285317
HBXIP	NC_000001	110943875	110950546	22.232635	20.996993	41.627408	37.038088
PROK1	NC_000001	110993788	110999976	0	0	0.0569935	0.0223213
CYMP	NC_000001	111023388	111033891	0	0	0	0
KCNA10	NC_000001	111059839	111061797	0.022434	0.0230645	0	0
KCNA2	NC_000001	111145776	111148345	0	0.0850511	0.0183989	0.0144117
KCNA3	NC_000001	111214310	111217655	0.0656728	0.0135037	0.0934789	0.0640688
LOC643232	NC_000001	111390295	111391694	0	0	0	0
OR11I1P	NC_000001	111396721	111397714	0	0	0	0
CD53	NC_000001	111413821	111442558	0	0	0	0.0376227
C1orf103	NC_000001	111489812	111506566	2.1216388	1.7748203	0.6213406	0.0918287
TMEM77	NC_000001	111659954	111682838	2.6564679	2.3957265	4.8923783	3.4424572
CEPT1	NC_000001	111682249	111727724	3.9680686	3.2521273	5.3118073	5.725861
DENND2D	NC_000001	111729801	111743281	1.3774176	1.2391139	0.1148802	0.1649724
LOC100287459	NC_000001	111733890	111743311	0.1298323	0.1334812	0.1155024	0
CHI3L2	NC_000001	111770281	111786062	0.1636806	0.140234	0.1698839	0.1520788
LOC149620	NC_000001	111823146	111828730	0	0	0	0
CHIA	NC_000001	111833484	111863185	0	0	0.1624231	0.054525
C1orf88	NC_000001	111889195	111895639	0.1685994	0.0577793	0.1999875	0.0913786
LOC100130383	NC_000001	111917924	111924135	0	0	0	0
LOC441897	NC_000001	111925635	111934625	0	0	0	0
OVGP1	NC_000001	111956937	111970399	0.078409	0.1813785	0.0697548	0.2731925
LOC100287536	NC_000001	111980808	111984695	0.3119662	0.641468	0.2081503	0.5434761
WDR77	NC_000001	111982512	111991830	5.238016	8.593853	4.9196688	8.5083237
ATP5F1	NC_000001	111991743	112004540	19.170235	29.382023	26.588562	29.278889
C1orf162	NC_000001	112016604	112021134	0.0958522	0.0492731	0.1279092	0.367365
ADORA3	NC_000001	112025970	112106597	0	0.013258	0.0229446	0.0179723
LOC643329	NC_000001	112163391	112192169	0	0	0	0
RAP1A	NC_000001	112233954	112256101	5.6323546	7.6393116	4.6578327	6.6335476
C1orf183	NC_000001	112268370	112298419	2.5786426	0.8582748	0.379588	2.8569431
DDX20	NC_000001	112298254	112310199	3.7750077	4.3662428	3.5171925	4.541298
KCND3	NC_000001	112318454	112531777	0	0.0167781	0.0290364	0.011372
LOC643355	NC_000001	112530792	112533648	0.6988235	0.6918542	0.6447184	0.5771473
AF357532	NC_000001	112905718	112906517	0	0	0	0
CTTNBP2NL	NC_000001	112938800	113003786	4.7151239	5.3071352	5.6923405	6.2235981
WNT2B	NC_000001	113033630	113063910	0.6391842	0.0888039	0.9221124	1.9983215
ST7L	NC_000001	113066140	113162040	1.4715019	1.3297738	1.2757363	1.0645884
CAPZA1	NC_000001	113162075	113214241	49.064833	49.404917	63.897408	74.349157
MRPL53P1	NC_000001	113168494	113168847	0	0	0	0
RNU7-70P	NC_000001	113177250	113177502	0	0	0	0
MOV10	NC_000001	113217048	113243368	17.393153	13.395959	11.634644	12.473995
LOC100289288	NC_000001	113217061	113217671	0	0	0.0791449	0.1239874
LOC100287627	NC_000001	113236310	113239478	0.0807872	0.0830577	0.1437411	0.1688872
RHOC	NC_000001	113243749	113250025	106.3596	115.31384	86.130418	75.268054
PPM1J	NC_000001	113252616	113257950	0.1282037	0.1054455	0.6158893	0.0893375
FAM19A3	NC_000001	113263189	113269857	0.1162652	0.1593771	0.0344776	0.0540121
LOC128322	NC_000001	113290717	113291097	0	0	0	0
RPL39P8	NC_000001	113433214	113433593	0	0	0	0

SLC16A1	NC_000001	113454469	113498975	6.5641349	11.035844	12.329232	11.678182
AFARP1	NC_000001	113465972	113467295	0	0	0	0
LOC100289355	NC_000001	113487621	113498603	0	0	0	0
LRIG2	NC_000001	113615831	113667342	2.05785	2.2057153	1.548322	1.350088
LOC100129578	NC_000001	113667786	113671213	0	0	0	0
RPS19P2	NC_000001	113711611	113712051	0	0	0	0
RPS15P1	NC_000001	113741429	113741913	0	0	0	0
MAGI3	NC_000001	113933475	114228545	0.2251883	0.2440317	0.2869645	0.4919659
PHTF1	NC_000001	114239824	114301777	1.7476331	2.3399542	2.6514995	3.408009
RPS2P14	NC_000001	114241506	114242228	0	0	0	0
LOC100289390	NC_000001	114301338	114302162	0.880319	0.4873402	0.6626706	1.1796948
RSBN1	NC_000001	114304454	114355070	1.3131809	2.0285586	2.1407889	1.7233171
PTPN22	NC_000001	114356437	114414375	0.6116744	0.6051348	1.3142039	0.4825348
BCL2L15	NC_000001	114419436	114430169	0.0353495	0.036343	0.007862	0.0246329
AP4B1	NC_000001	114437677	114447741	3.3438872	4.4928745	2.6914991	3.8219454
LOC100287722	NC_000001	114442537	114447508	0.0781997	0.0803975	0.1391372	0
LOC100289426	NC_000001	114447915	114450799	0.0532061	0.9299247	0.5680034	0.4819896
DCLRE1B	NC_000001	114448038	114456694	2.6722836	4.3190927	4.1549864	5.594849
HIPK1	NC_000001	114471996	114520422	5.4479125	6.6301148	7.4623942	7.1458049
OLFML3	NC_000001	114522030	114524875	29.969108	72.956	7.4358656	2.3804343
RPL13AP10	NC_000001	114544055	114545965	0	0	0	0
SYT6	NC_000001	114631914	114696472	0.0804544	0.0723762	0.0536809	0.0770878
MRP63P1	NC_000001	114821624	114821908	0	0	0	0
TRIM33	NC_000001	114935399	115053781	1.7865992	2.075218	3.0006526	2.7947634
RPL26P10	NC_000001	115002456	115002802	0	0	0	0
LOC347675	NC_000001	115010740	115012266	0	0	0	0
LOC643586	NC_000001	115062638	115080469	0	0	0	0
BCAS2	NC_000001	115110178	115124265	6.0664737	8.1773641	8.6350452	7.7736463
DENND2C	NC_000001	115127196	115212732	1.1722478	1.2961519	0.4525637	0.446966
AMPD1	NC_000001	115215722	115238176	0.0563198	0.1351063	0.0334024	0.0915737
NRAS	NC_000001	115247078	115259515	12.009167	16.519643	14.285818	17.265539
CSDE1	NC_000001	115259537	115300671	19.129207	32.456569	28.620144	32.685485
SIKE	NC_000001	115312100	115323308	3.8059743	4.0195837	3.2226389	3.6752078
NR1H5P	NC_000001	115377334	115397515	0	0	0	0
SYCP1	NC_000001	115397455	115537991	0	0.0129428	0.0111995	0.0263176
TSHB	NC_000001	115572415	115576941	0	0	0	0
TSPAN2	NC_000001	115590632	115632115	0.1096308	0.4085808	0.1828698	0.0763951
NGF	NC_000001	115828537	115880857	3.4773148	4.6087931	1.6026647	1.8392444
LOC100287778	NC_000001	116044458	116100090	0	0	0	0
LOC100132332	NC_000001	116107003	116108053	0	0	0	0
LOC100287840	NC_000001	116119850	116172220	0	0	0	0
VANGL1	NC_000001	116184574	116240845	2.1996871	3.0985281	2.1638395	2.3538625
CASQ2	NC_000001	116242624	116311426	0.0325301	0.0167222	0.0144699	0.0793391
NHLH2	NC_000001	116378998	116383747	0.0327972	0.0505784	0	0.057136
LOC400769	NC_000001	116399529	116400440	0	0	0	0
LOC100287899	NC_000001	116454062	116459320	0	0	0	0
SLC22A15	NC_000001	116519119	116612675	0.7262632	0.9859937	0.6626706	0.1297664
C1orf161	NC_000001	116654376	116677861	0.0816629	0.055972	0.0242165	0.0189686
ATP1A1	NC_000001	116915836	116947396	63.315853	77.987455	105.04896	94.009522
C1orf203	NC_000001	116947336	116961205	0	0	0	0
CD58	NC_000001	117057156	117113715	2.8707277	2.2001419	5.2238434	5.1102083
LOC728589	NC_000001	117074383	117076631	0	0	0	0
IGSF3	NC_000001	117117031	117210314	0.0484745	0.1931181	0.0215621	0.0548909
LOC100287935	NC_000001	117150517	117156708	0	0	0.1022159	0.0400325
GAPDHL9	NC_000001	117256454	117257456	0	0	0	0
LOC339396	NC_000001	117282603	117285231	0	0	0	0
CD2	NC_000001	117297086	117311851	0.0574111	0.0590247	0.2298355	0.0600096
PTGFRN	NC_000001	117452689	117532972	3.3317897	1.7237174	6.7341749	6.8756829
LOC100289477	NC_000001	117509546	117527544	0	0.2368724	0.4611771	0.0802749
IGSF2	NC_000001	117544382	117579167	0.0619513	0.0764309	0.0110227	0.1122423
TTF2	NC_000001	117602949	117645492	0.8237465	1.9264602	1.1837986	1.9680663
TRIM45	NC_000001	117653677	117664411	0.4540867	0.3659086	0.7861002	0.8979649
RPS15AP9	NC_000001	117680787	117681270	0	0	0	0
VTCN1	NC_000001	117686209	117753549	0.0168707	0	0.0300173	0
LOC100289512	NC_000001	117695845	117699534	0	0	0	0
MAN1A2	NC_000001	117910085	118068320	3.3197718	4.6541923	3.7878488	6.4284962

LOC441899	NC_000001	118092037	118092546	0	0	0	0
FAM46C	NC_000001	118148604	118171011	0.3611131	0.3870606	0.4374553	0.685312
LOC401959	NC_000001	118183434	118184294	0	0	0	0
LOC100192389	NC_000001	118242051	118242881	0	0	0	0
LOC100131261	NC_000001	118319133	118321663	0.1389118	0.053556	0.2626071	0.2298983
GDAP2	NC_000001	118406107	118472302	1.3097155	1.4867883	1.9500235	2.4939755
WDR3	NC_000001	118472372	118503049	1.3602754	1.8064037	1.401744	2.5119193
SPAG17	NC_000001	118496288	118727848	0.0246727	0.0126831	0.0274369	0.0644734
LOC644094	NC_000001	119156955	119234149	0	0	0	0
TBX15	NC_000001	119425666	119532179	7.9848719	8.4290638	9.1731055	8.8501079
LOC100289647	NC_000001	119572907	119576013	0.242139	0	0.1615602	0.3796473
WARS2	NC_000001	119573839	119683295	1.9687568	2.2312791	2.2203556	2.7762235
RPS3AP12	NC_000001	119669118	119669934	0	0	0	0
RPL6P2	NC_000001	119761916	119762740	0	0	0	0
HAO2	NC_000001	119911402	119936751	0.0259129	0	0.0691584	0.0361142
HSD3B2	NC_000001	119957773	119965651	0	0	0.0234257	0.0183492
LOC391073	NC_000001	119976790	119980943	0	0	0	0
LOC440606	NC_000001	119981437	119989222	0	0	0	0
LOC391075	NC_000001	120004458	120005715	0	0	0	0
LOC391076	NC_000001	120009844	120016426	0	0	0	0
GAPDHL1	NC_000001	120038412	120039539	0	0	0	0
HSD3B1	NC_000001	120049826	120057681	0.0261597	0.0268949	0.0232724	0.0364582
LOC644213	NC_000001	120076171	120077444	0	0	0	0
LOC441900	NC_000001	120081132	120088711	0	0	0	0
LOC401960	NC_000001	120101175	120102468	0	0	0	0
LOC128102	NC_000001	120110516	120110676	0	0	0	0
LOC644237	NC_000001	120138588	120139873	0	0	0	0
LOC391081	NC_000001	120143963	120152114	0	0	0	0
LOC100130667	NC_000001	120157784	120169154	0.3709916	0.7041569	0.4315965	0.5767023
ZNF697	NC_000001	120162000	120190390	0.7699592	0.7560215	0.7927262	0.5124241
PHGDH	NC_000001	120254419	120286849	25.343844	33.231663	27.436207	13.769796
HMGCS2	NC_000001	120291002	120311518	0.0859203	0.0883351	0.0382185	0.0449045
REG4	NC_000001	120336708	120354203	0	0.0354102	0	0
NBPF7	NC_000001	120377388	120387779	0.09316	0.0478891	0.1864749	0.0486882
LOC767850	NC_000001	120395828	120396611	0	0	0	0
ADAM30	NC_000001	120436156	120439113	0	0	0	0.0207065
NOTCH2	NC_000001	120454176	120612276	10.647589	12.505026	21.018578	17.97285
LOC100132913	NC_000001	120835885	120858711	3.9709957	4.8677166	6.8276374	4.1241148
FAM72B	NC_000001	120839005	120855681	0.3332216	0.3045217	0.2635051	0.3741034
LOC100132314	NC_000001	120876506	120905165	3.9583858	9.7045182	6.7720957	1.9096344
RPL22P6	NC_000001	120880883	120881272	0	0	0	0
HIST2H2BB	NC_000001	120905664	120906800	0	0	0	0
LOC728744	NC_000001	120924756	120926874	0	0	0	0.0193951
FCGR1B	NC_000001	120926908	120935944	0.0391695	0	0.0696926	0.0272949
SRGAP2P1	NC_000001	121090793	121134742	0	0	0	0
LOC100288423	NC_000001	121137901	121139473	0	0	0	0
MTIF2P1	NC_000001	121244145	121245730	0	0	0	0
LOC647121	NC_000001	121260910	121313686	0	0	0	0
LOC100289391	NC_000001	142690713	142690940	0	0	0	0.1343196
LOC375010	NC_000001	142697421	142713416	0	0	0	0
LOC727770	NC_000001	142826888	142842760	0	0	0	0
LOC100132329	NC_000001	142849225	142849452	0	0	0	0
LOC727788	NC_000001	142852669	142855999	0.1319371	0.2848548	0.3051746	1.3331154
LOC100289550	NC_000001	142873517	142891110	0.389612	0.8011241	0	0.2714971
LOC100130131	NC_000001	143187185	143187412	0	0	0	0
LOC100132733	NC_000001	143195831	143265530	0	0.160795	0.0695686	0.2361349
LOC100132482	NC_000001	143267328	143271286	0	0	0	0
LOC728783	NC_000001	143378617	143392505	0	0	0	0
LOC100131965	NC_000001	143400897	143401124	0	0	0	0
RNU1P10	NC_000001	143647014	143647177	0	0	0	0
TRNAN35P	NC_000001	143663558	143663631	0	0	0	0
RNU1P6	NC_000001	143673129	143673290	0	0	0	0
LOC100130000	NC_000001	143699874	143713693	0.0453542	0.0466289	0.1210451	0.0948139
LOC647481	NC_000001	143720364	143721293	0	0	0	0
LOC100287981	NC_000001	143737970	143744468	0.1284838	0.250285	0.2466534	0.5089224
PPIAL4G	NC_000001	143767144	143767881	0	0.0612241	0.0529777	0.4149713

FAM72D	NC_000001	143896452	143913143	0.3856719	0.6419706	1.3234028	0.5758962
LOC100132966	NC_000001	143914158	143918509	0	0	0	0
SRGAP2P2	NC_000001	143915685	144094477	0	0	0	0
NBPF8	NC_000001	144146648	144224213	0	0	0	0.002643
LOC100132395	NC_000001	144277346	144289974	0	0	0	0
LOC100132102	NC_000001	144294159	144301546	0	0	0	0
LOC728855	NC_000001	144300512	144340773	0	0	0	0
LOC730256	NC_000001	144334282	144340780	0.0202869	0.0486665	0.0240637	0.0518347
PPIAL4B	NC_000001	144360739	144364592	0	0.0117238	0.030434	0.031785
LOC100132926	NC_000001	144392143	144392429	0	0	0	0
LOC100133080	NC_000001	144457589	144470212	0	0	0	0
LOC100287384	NC_000001	144474398	144481775	0	0	0	0
LOC728875	NC_000001	144480744	144521969	0	0	0	0
LOC730257	NC_000001	144514531	144521058	0	0	0	0
C1orf152	NC_000001	144612022	144612727	0	0	0	0
NBPF20 (NC_000001 14	NC_000001	144614959	144621685	1.1692649	1.8239171	2.8336768	4.242529
LOC653513	NC_000001	144676434	144681668	2.8741412	0	1.3481919	1.2745193
NBPF20 (NC_000001 14	NC_000001	144811715	144828810	0.6248564	0.321209	1.2044274	0.7111939
PDE4DIP	NC_000001	144851427	145076079	16.385516	6.4645524	25.733488	33.443373
SEC22B	NC_000001	145096407	145116922	23.482498	11.909958	12.474292	24.890071
NUDT4P1	NC_000001	145136118	145139902	0	0	0	0
NOTCH2NL	NC_000001	145209111	145285912	8.9986706	6.9295786	12.331256	11.195794
NBPF10	NC_000001	145293371	145368684	1.227267	0.9266693	2.8780849	1.6963973
HFE2	NC_000001	145413191	145417545	0	0.0203988	0	0.0414784
TXNIP	NC_000001	145438462	145442635	5.7219579	3.9731826	29.543048	1.9936441
POLR3GL	NC_000001	145456236	145470387	0.4526856	0.7368967	0.5705224	0.3417368
ANKRD34A	NC_000001	145470508	145475647	0.3163494	0.4253144	0.1190679	0.0678292
LIX1L	NC_000001	145477085	145499091	8.0618812	10.900778	7.3395145	8.5688453
RBM8A	NC_000001	145507638	145511444	3.3903372	6.7929119	4.9661063	5.1096406
GNRHR2	NC_000001	145509820	145516076	0	0	0	0
PEX11B	NC_000001	145516383	145523731	4.4494173	7.6615334	8.0866394	5.934759
ITGA10	NC_000001	145524990	145543868	1.3941025	0.9875676	0.1285607	0.2014015
ANKRD35	NC_000001	145549209	145568526	0.1437498	0.0268709	0.0465032	0.0637449
PIAS3	NC_000001	145575988	145586546	3.0591119	4.655354	3.583719	5.2976185
NUDT17	NC_000001	145586491	145589435	1.286682	1.818911	0.4650214	0.4202865
POLR3C	NC_000001	145592605	145610884	9.0223274	12.180723	10.138753	8.6696036
RNF115	NC_000001	145611036	145688776	10.993617	12.057899	8.6831608	15.21188
CD160	NC_000001	145695798	145715565	0.02819	0.0289823	0.0752359	0.0589318
PDZK1	NC_000001	145727726	145764073	0.0625746	0.0214444	0.2597845	0.3197662
GPR89A	NC_000001	145764595	145827103	4.3151587	5.3237241	6.9493659	8.6199934
GPR89C	NC_000001	145883868	145924049	0.1680621	0.2015831	0.2741066	0.1951872
PDZK1P1	NC_000001	145924388	145942619	0	0	0	0
TRNAR33P	NC_000001	145941579	145941649	0	0	0	0
LOC728789	NC_000001	145943440	145945792	0.0373551	0.0192025	0.0498481	0.0260305
LOC100286941	NC_000001	145963450	145965653	0	0	0	0.0138951
RNU1D2	NC_000001	145969271	145969433	0	0	0	0
LOC200030	NC_000001	146032542	146068245	0.9984146	0.5548514	1.5363759	1.4604168
LOC767852	NC_000001	146059879	146060584	0	0	0	0
LOC100289252	NC_000001	146075482	146080247	0.1750926	0.0600045	0.2076896	0.1016762
LOC100132184	NC_000001	146391007	146393001	0.0440584	0	0.0195978	0
NBPF9	NC_000001	146401706	146420042	0.3418196	0.7530567	0.3475339	0.3743041
LOC728989	NC_000001	146490895	146514599	0	0	0	0
LOC644060	NC_000001	146522278	146524139	0	0	0	0
LOC100289144	NC_000001	146526493	146549732	0	0	0	0
TRNAE34P	NC_000001	146550178	146550249	0	0	0	0
LOC441904	NC_000001	146553892	146555128	0	0	0	0
NBPF13P	NC_000001	146556391	146596268	0	0	0	0
PRKAB2	NC_000001	146626685	146644129	0.6888438	1.049808	0.7642157	0.8019054
PDIA3P	NC_000001	146649430	146651528	0	0	0	0
FMO5	NC_000001	146655884	146697230	0.2489201	0.1137405	0.1230257	0.3276419
RPL7AP15	NC_000001	146695125	146695995	0	0	0	0
CHD1L	NC_000001	146714291	146767443	5.3584721	6.2296052	8.0663084	11.700535
LOC100289179	NC_000001	146747034	146751959	0.0856691	0	0	0
LOC100130018	NC_000001	146790820	146791946	0	0	0	0
OR13Z1P	NC_000001	146890632	146891724	0	0	0	0
OR13Z2P	NC_000001	146917211	146917966	0	0	0	0

OR13Z3P	NC_000001	146953887	146954477	0	0	0	0
LOC100289211	NC_000001	146972846	146973202	0	0	0	0
BCL9	NC_000001	147013182	147098017	0.7000355	0.6117536	1.8994517	2.3415007
ACP6	NC_000001	147119168	147142634	1.531382	0.9496511	1.6434817	1.3889602
GJA5	NC_000001	147228332	147245484	0.0133785	0.0137545	0	0.0093226
LOC391092	NC_000001	147313096	147314504	0	0	0	0
GJA8	NC_000001	147374936	147381393	0	0	0.0284553	0
GPR89B	NC_000001	147400506	147465753	2.0977181	2.5410325	2.7161352	1.3604625
PDZK1P2	NC_000001	147466333	147481824	0	0	0	0
TRNAR31P	NC_000001	147483291	147483361	0	0	0	0
LOC728905	NC_000001	147485151	147487503	0	0	0.016616	0.0260305
LOC100289324	NC_000001	147504907	147507387	0.6528812	0.5561624	0.3816825	0.2079788
LOC100132404	NC_000001	147520911	147528015	0	0	0	0
LOC100289427	NC_000001	147532525	147532980	0	0	0	0
LOC728920	NC_000001	147552768	147566156	0	0	0	0
NBPF11	NC_000001	147575800	147610081	0.1160264	0.0927791	0.0917514	0.0898354
LOC767851	NC_000001	147601716	147602421	0	0	0	0
FAM108A2	NC_000001	147617318	147622083	0	0	0	0
LOC100289456	NC_000001	147632468	147633807	0	0	0	0
LOC100289487	NC_000001	147718443	147718959	0	0	0	0
TRNAE32P	NC_000001	147781030	147781101	0	0	0	0
TRNAQ51P	NC_000001	147825689	147825760	0	0	0	0
TRNAN34P	NC_000001	147851060	147851133	0	0	0	0
LOC100132057	NC_000001	147887383	147900979	0	0.1007434	0.0871741	0.1024243
LOC100133183	NC_000001	147907215	147908803	0	0	0	0
LOC100132999	NC_000001	147930942	147931990	0.7960118	0.5599468	0.6336116	0.2919435
PPIAL4A	NC_000001	147954760	147955419	0.1997647	0.1369194	0.1184775	0.139204
LOC100132587	NC_000001	147983307	147983593	0	0	0	0
LOC100289551	NC_000001	147993781	148000730	0	0	0	0
NBPF14	NC_000001	148003642	148025848	1.9895242	1.9107104	3.8896196	3.7689604
LOC729086	NC_000001	148183855	148196113	0.3886578	0.4917921	0.2393729	0.2812489
PPIAL4D	NC_000001	148201873	148202559	0	0	0	0
LOC100129127	NC_000001	148229952	148230238	0	0	0	0
LOC100133078	NC_000001	148242700	148248044	0	0	0	0
LOC100288142	NC_000001	148250245	148354334	0.1056983	0.1487049	0.1880642	0.2616683
LOC200025	NC_000001	148349192	148349897	0	0	0	0
NBPF15	NC_000001	148560990	148596158	5.4334008	5.6310658	8.9477016	10.5054
LOC767854	NC_000001	148574410	148575115	0	0	0	0
LOC100132040	NC_000001	148616292	148616578	0	0	0	0
PPIAL4E	NC_000001	148644011	148644795	0	0.2877924	0.3486407	0.2340755
LOC645126	NC_000001	148736446	148737151	0	0	0	0
NBPF16	NC_000001	148739442	148758311	0.1491326	0.1179415	0.2245227	0.4156862
LOC100132583	NC_000001	148760500	148761483	0	0	0	0
LOC100132490	NC_000001	148778320	148778606	0	0	0	0
PPIAL4F	NC_000001	148805664	148806799	0	0	0	0
LOC645146	NC_000001	148845362	148854293	0	0.0910956	0	0
LOC100288681	NC_000001	148853582	148855710	0	0	0	0
LOC100133086	NC_000001	148864456	148866653	0.0999732	0.1438962	0.1245145	0
DRD5P2	NC_000001	148902094	148904339	0	0	0	0
LOC100288616	NC_000001	148932785	148951939	5.29055	12.494099	2.6096111	2.555115
NBPF17P	NC_000001	149025632	149090118	0	0	0	0
LOC100288708	NC_000001	149038717	149067585	0	0	0	0
TRNAQ37P	NC_000001	149079364	149079435	0	0	0	0
LOC647569	NC_000001	149114649	149115428	0	0	0	0
LOC100288733	NC_000001	149136787	149148384	0	0	0	0
LOC100288768	NC_000001	149148431	149148791	0	0	0	0
TRNAE30P	NC_000001	149161628	149161699	0	0	0	0
TRNAN36P	NC_000001	149211948	149212021	0	0	0	0
LOC100129567	NC_000001	149212092	149212584	0	0	0	0
RNU1G3	NC_000001	149224058	149224221	0	0	0	0
RNU1P9	NC_000001	149224058	149224221	0	0	0	0
LOC100288829	NC_000001	149230714	149239532	0	0	0	0
FAM91A3P	NC_000001	149239530	149263301	0	0	0	0
LOC100288865	NC_000001	149264234	149279311	0	0	0	0
TRNAE29P	NC_000001	149278785	149278857	0	0	0	0
LOC388692	NC_000001	149279476	149291742	0	0	0	0

LOC100133075	NC_000001	149285745	149289650	0	0	0	0
TRNAF17P	NC_000001	149287359	149287430	0	0	0	0
LOC645262	NC_000001	149305910	149336405	0	0	0	0
LOC100131962	NC_000001	149340658	149341066	0	0	0	0
TRNAR30P	NC_000001	149343081	149343151	0	0	0	0
FCGR1C	NC_000001	149369300	149378181	0	0	0	0
LOC729189	NC_000001	149378408	149380524	0.0278683	0.0286515	0	0.0194197
LOC653593	NC_000001	149380592	149399179	0	0	0	0
HIST2H3PS2	NC_000001	149398877	149400542	0	0	0	0
LOC100133008	NC_000001	149400063	149429348	0.088964	0.3658575	0.3165795	1.3018673
RPL22P5	NC_000001	149424565	149424954	0	0	0	0
LOC100132836	NC_000001	149524787	149525073	0	0	0	0
PPIAL4C	NC_000001	149552657	149556510	0.0114033	0.0234475	0.030434	0.0079463
LOC642441	NC_000001	149576434	149577482	0.7122211	0.5599468	0.5217978	0.1459718
LOC100288958	NC_000001	149615755	149623156	0	0	0	0
LOC100132827	NC_000001	149627341	149639967	0	0	0	0
LOC100132306	NC_000001	149640844	149649326	0	0	0	0
LOC729130	NC_000001	149649060	149719362	1.1920859	0.9804717	1.4140168	0.8306929
LOC100288037	NC_000001	149671871	149672242	0	0	0	0
TRNAF16P	NC_000001	149672905	149672976	0	0	0	0
LOC644634	NC_000001	149673344	149677285	0.0111487	0	0	0
TRNAG34P	NC_000001	149680210	149680280	0	0	0	0
LOC199882	NC_000001	149691432	149698599	0	0	0	0
LOC100129586	NC_000001	149725849	149726258	0	0	0	0
TRNAR32P	NC_000001	149728271	149728341	0	0	0	0
FCGR1A	NC_000001	149754250	149764074	0	0.0207644	0	0.0281479
LOC729127	NC_000001	149763336	149765367	0	0	0	0
HIST2H2BF	NC_000001	149782115	149783911	1.8097769	2.4892358	1.5447563	0.7328157
HIST2H3D	NC_000001	149784826	149785236	6.6296603	40.126379	39.763461	23.322596
LOC730631	NC_000001	149800254	149804109	0.2149058	0.0552364	0.1433896	0
HIST2H4A	NC_000001	149804221	149804616	18.533724	44.498805	34.358467	17.323163
HIST2H3C	NC_000001	149812259	149812765	29.038772	45.094283	50.587777	72.545323
HIST2H2AA3	NC_000001	149813785	149814318	24.360818	47.891022	36.534991	16.057989
H2BFO	NC_000001	149814374	149815203	0	0	0	0
HIST2H2BD	NC_000001	149814606	149815100	0	0	0	0
HIST2H2BC	NC_000001	149821759	149822340	0	0	0	0
HIST2H2AA4	NC_000001	149822628	149823161	23.867017	41.460425	35.217096	16.287388
HIST2H3A	NC_000001	149824181	149824687	27.825212	47.767857	57.451058	74.41785
HIST2H4B	NC_000001	149832330	149832725	19.643528	43.129611	31.593992	20.416586
LOC100132909	NC_000001	149832837	149836692	0.1074529	0.1104729	0.1911861	0.0748775
HIST2H2BE	NC_000001	149856010	149858232	3.6376404	5.6504659	6.0501856	4.3257813
HIST2H2AC	NC_000001	149858525	149858961	68.486833	146.71681	143.14898	83.044578
HIST2H2AB	NC_000001	149859019	149859466	6.7688125	17.448055	16.319743	13.124948
BOLA1	NC_000001	149871155	149872348	1.487163	2.7415833	3.604093	2.8945334
SV2A	NC_000001	149874870	149889434	1.532615	1.2585051	0.230194	0.2427244
SF3B4	NC_000001	149895211	149899702	16.285529	20.010209	13.907527	23.448099
MTMR11	NC_000001	149900544	149908243	3.2148018	3.4023646	4.5423161	3.1231051
OTUD7B	NC_000001	149912231	149982686	1.3980102	2.2404993	2.0545579	1.594996
LOC441907	NC_000001	150025846	150027021	0	0	0	0
VPS45	NC_000001	150039342	150117505	3.2294228	5.7581971	6.4815631	6.0771413
PLEKHO1	NC_000001	150122170	150131825	11.211561	13.177327	16.437924	15.606362
ANP32E	NC_000001	150190717	150208504	6.2217733	8.8891635	10.288833	9.2659004
CA14	NC_000001	150230218	150237478	0.0758164	0.0519648	0.0899311	0.2641594
APH1A	NC_000001	150237799	150241532	21.000389	29.103973	28.738229	21.796967
C1orf54	NC_000001	150245198	150253327	7.8478985	3.2273859	5.507792	1.032982
C1orf51	NC_000001	150255229	150259501	2.3210318	1.3746959	2.8728406	3.0413915
MRPS21	NC_000001	150266269	150280819	12.215643	9.2868973	18.61194	13.469728
PRPF3	NC_000001	150293928	150325704	0.6423615	0.275173	0.825446	1.0320199
RPRD2	NC_000001	150336990	150449042	1.7873817	2.3230244	3.0902028	5.4246651
TARS2	NC_000001	150459920	150479756	5.9644029	7.3850182	9.0680068	6.781223
ECM1	NC_000001	150480487	150486265	36.978829	33.002429	29.487296	18.112102
ADAMTSL4	NC_000001	150521898	150533413	1.4410932	1.17687	4.4371191	1.2962158
LOC100289061	NC_000001	150527093	150532712	0.1656861	0	0	0.0865925
C1orf138	NC_000001	150532554	150534591	0	0	0	0
MCL1	NC_000001	150547034	150552136	10.624485	12.233406	16.020228	16.690559
LOC100131311	NC_000001	150550796	150552139	0.088427	0.1818246	0.0393336	0.0308097

ENSA	NC_000001	150594599	150602098	6.9235694	8.8840706	13.903453	18.995552
GOLPH3L	NC_000001	150618701	150669672	2.7441658	3.0920209	3.1810697	2.8587083
HORMAD1	NC_000001	150670542	150693352	0	0.0240465	0.0624229	0.032597
CTSS	NC_000001	150702551	150738305	5.8633373	2.193048	6.8945219	1.7478589
LOC100132571	NC_000001	150751547	150753126	0	0	0	0
LOC100289153	NC_000001	150768563	150786424	1.6374942	2.7153487	3.2894586	5.9630172
CTSK	NC_000001	150768687	150780812	91.072511	71.916472	62.069108	16.967838
ARNT	NC_000001	150782186	150849186	2.3413548	2.9972406	5.162759	5.948489
RPS27AP6	NC_000001	150853708	150854110	0	0	0	0
CYCSP51	NC_000001	150876372	150876689	0	0	0	0
SETDB1	NC_000001	150898815	150937220	1.472849	1.9207519	3.4911681	3.3338824
LASS2	NC_000001	150937649	150947440	8.4913949	8.2610771	19.32247	10.477254
ANXA9	NC_000001	150954499	150968114	0.168661	0	0.4072663	0.1511096
FAM63A	NC_000001	150967549	150979333	0.9793765	0.4027608	1.3665347	1.5086147
PRUNE	NC_000001	150980973	151008189	0.9890936	2.0944943	2.1407132	1.5430741
BNIPL	NC_000001	151009034	151020075	0.0612092	0.083906	0.0181511	0.0284354
C1orf56	NC_000001	151020259	151023896	0.6636119	0.462178	0.4189705	0.2685084
CDC42SE1	NC_000001	151023447	151032125	1.2978743	1.5614748	2.112718	2.9633875
MLLT11	NC_000001	151032151	151040973	6.0484823	10.657253	9.8559202	3.675553
GABPB2	NC_000001	151043080	151091007	0.7876027	0.9485507	1.1010579	0.9408565
SEMA6C	NC_000001	151104161	151119104	0.3500502	0.3947163	0.3415512	0.4563831
TNFAIP8L2	NC_000001	151129105	151132225	0.074615	0.1534241	0	0.2339761
LYSMD1	NC_000001	151132224	151138424	0.5432198	0.9548327	1.5589141	0.9036049
SCNM1	NC_000001	151138517	151141614	9.0150219	11.26952	13.488204	9.8156661
TMOD4	NC_000001	151142463	151148547	0	0.0341781	0.2070219	0.1853245
VPS72	NC_000001	151148934	151162640	1.9916117	3.8562873	10.748878	6.2915158
PIP5K1A	NC_000001	151171021	151222007	10.966856	11.880374	15.065965	11.583878
PSMD4	NC_000001	151227197	151239955	27.847078	42.333998	32.816125	40.902146
ZNF687	NC_000001	151254791	151264381	3.031921	2.9467436	5.3685433	4.5991665
PI4KB	NC_000001	151264413	151298724	9.9266533	11.936958	12.897353	12.616991
RFX5	NC_000001	151313116	151319769	4.3365171	3.6216657	10.892854	11.579556
SELENBP1	NC_000001	151336780	151345164	10.980614	5.1676032	2.5453547	0.3053507
PSMB4	NC_000001	151372041	151374412	45.231045	70.876882	57.948932	54.661269
POGZ	NC_000001	151375200	151431932	2.0644847	2.4429998	6.5982375	7.0332295
CGN	NC_000001	151483862	151511168	0.0516835	0.0619921	0.1072846	0.0840353
TUFT1	NC_000001	151512781	151556059	1.3420386	1.4959468	1.3321575	1.2009756
RPS10P6	NC_000001	151529885	151530463	0	0	0	0
SNX27	NC_000001	151584662	151671559	2.3881537	3.4373823	4.1553235	3.8945681
TNRC4	NC_000001	151674830	151689290	0	0.0959308	0.0592926	0.0185774
C1orf230	NC_000001	151694013	151702082	0.0965895	0	0	0
RPS11P3	NC_000001	151730557	151730908	0	0	0	0
MRPL9	NC_000001	151732123	151736040	4.2519574	8.9527484	9.2296885	7.9406612
OAZ3	NC_000001	151735445	151743806	0.080936	0.1664214	0.7200288	0.1973979
TDRKH	NC_000001	151744040	151763010	0.6059039	0.5537182	2.50349	1.8014634
LOC100270670	NC_000001	151750382	151751106	0	0	0	0
LINGO4	NC_000001	151772765	151777882	0.0168642	0.0173382	0	0.0117517
RORC	NC_000001	151778547	151804348	0.027348	0.0140583	0.7055566	0.4097292
LOC100191040	NC_000001	151810339	151813033	0.1282786	0	0	0.1072675
LOC100132111	NC_000001	151810945	151816641	0	0	0	0
THEM5	NC_000001	151819577	151826173	0.2399358	0	0.7826628	0.7523855
THEM4	NC_000001	151846060	151882113	1.4343938	2.068687	3.3851473	3.5955773
KRT8P28	NC_000001	151921871	151923466	0	0	0	0
S100A10	NC_000001	151955386	151966714	64.31855	41.114754	46.84296	43.019971
NBPF18P	NC_000001	151986711	151996749	0	0	0	0
S100A11	NC_000001	152004982	152009511	149.3796	159.35897	268.08819	138.01818
LOC100131107	NC_000001	152052033	152053058	0	0	0	0
TCHHL1	NC_000001	152056620	152061540	0.0243954	0	0.0217028	0.0169997
TCHH	NC_000001	152078793	152086556	0.0063693	0.0065483	0.0226653	0.0221919
HDHD1CP	NC_000001	152095740	152097586	0	0	0	0
RPTN	NC_000001	152126071	152131704	0.0246278	0	0	0.0171616
HRNR	NC_000001	152184558	152196669	0.022835	0.079821	0.0325034	0.0318247
FLG	NC_000001	152274651	152297679	0.3309822	0.9818626	0.187099	0.2474592
FLG2	NC_000001	152321213	152332482	0.0048178	0.0346726	0	0.0201435
LOC100289191	NC_000001	152371944	152372349	0	0	0	0
CRNN	NC_000001	152381719	152386750	0.0689204	0.0708574	0.0408757	0.0160088
LCE5A	NC_000001	152483320	152484653	0	0	0	0

CRCT1	NC_000001	152486978	152488481	0	0	0	0
LCE3E	NC_000001	152538130	152539248	0	0.1478998	0	0
LCE3D	NC_000001	152551860	152552980	0	0.0740712	0.0640944	0
LCE3C	NC_000001	152573208	152573492	0	0	0	0
LCE3B	NC_000001	152586287	152586574	0	0	0	0
LCE3A	NC_000001	152595310	152595579	0	0	0	0
LCEP4	NC_000001	152616869	152617193	0	0	0	0
LCEP3	NC_000001	152628808	152628987	0	0	0	0
LCE2D	NC_000001	152635872	152637135	0	0	0	0.0502872
LCE2C	NC_000001	152647771	152649050	0	0	0.0636768	0
LCE2B	NC_000001	152658599	152659877	0	0	0	0
LCE2A	NC_000001	152670840	152671918	0	0.9922031	0	0.0517312
LCE4A	NC_000001	152681523	152681910	0	0	0	0
C1orf68	NC_000001	152691998	152692905	0.0484011	0	0	0.1011835
LCEP2	NC_000001	152709994	152710344	0	0	0	0
LCEP1	NC_000001	152716775	152717082	0	0	0	0
LOC100289227	NC_000001	152717404	152734528	0	0	0	0
KPRP	NC_000001	152730506	152734529	0	0.0179086	0	0.0121383
LCE1F	NC_000001	152748848	152749204	0	0	0.109517	0.085784
LCE1E	NC_000001	152758753	152760902	0	0	0	0.0259533
LOC100289267	NC_000001	152761954	152770749	0.0681368	0.0700518	0	0
LCE1D	NC_000001	152769227	152770657	0	0	0	0.0712206
LCE1C	NC_000001	152777311	152779107	0	0	0	0
LCE1B	NC_000001	152784447	152785585	0	0	0	0
LCE1A	NC_000001	152799949	152800281	0	0	0	0
LCE6A	NC_000001	152815330	152816459	0	0.0703791	0	0
SMCP	NC_000001	152850798	152857523	0	0	0	0
IVL	NC_000001	152881039	152884362	0.061754	0.0634896	0.0366254	0.0286884
SPRR4	NC_000001	152943128	152945069	0	0.0589862	0	0.0399803
SPRR1A	NC_000001	152956557	152958290	0	0	0	0
SPRR3	NC_000001	152974223	152976332	0	0	0	0
SPRR1B	NC_000001	153003679	153005376	0	0	0.1263249	0.0494748
SPRR2D	NC_000001	153012201	153013594	0	0.1325026	0	0
SPRR2A	NC_000001	153028596	153029988	0	0	0.0577512	0
SPRR2B	NC_000001	153042717	153044084	0	0	0	0
SPRR2E	NC_000001	153065611	153067001	0	0	0	0
SPRR2F	NC_000001	153084612	153085989	0.0668923	0	0	0
SPRR2C	NC_000001	153112594	153113969	0	0	0	0
SPRR2G	NC_000001	153122058	153123427	0	0	0	0
LELP1	NC_000001	153175919	153177596	0	0	0	0
RPLP0P4	NC_000001	153197483	153198548	0	0	0	0
LOR	NC_000001	153232179	153234600	0.0715187	0.0735287	0	0
PGLYRP3	NC_000001	153270338	153283194	0.0374665	0	0	0
PGLYRP4	NC_000001	153302596	153321022	0	0.0246365	0	0
S100A9	NC_000001	153330330	153333503	0	0	0	0.1061521
S100A12	NC_000001	153346184	153348075	0	0	0	0.1314372
LOC645900	NC_000001	153352293	153357407	0	0	0	0
S100A8	NC_000001	153362508	153363549	0	0	0	0
S100A7A	NC_000001	153389000	153395701	0.0102707	0	0	0.0357851
S100A7P1	NC_000001	153399484	153400877	0	0	0	0
S100A7L2	NC_000001	153409471	153412503	0	0	0.0814533	0
S100A7P2	NC_000001	153417385	153419139	0	0	0	0
S100A7	NC_000001	153430220	153433137	0	0	0.2671815	0
S100A6	NC_000001	153507076	153508717	507.23852	751.71156	954.4271	433.1425
S100A5	NC_000001	153509623	153514241	0.4951913	0.1909158	0.440536	0.0431336
S100A4	NC_000001	153516095	153518282	171.11758	43.501041	215.52186	132.70781
S100A3	NC_000001	153519809	153521734	3.5134765	3.6122232	3.2846193	2.9047988
S100A2	NC_000001	153533584	153538306	2.2362029	3.1905206	3.9787761	2.0989013
LOC100287038	NC_000001	153579359	153580655	1.0600579	3.7690677	4.9903425	0.2462302
S100A16	NC_000001	153579367	153585514	90.488915	133.6041	77.426359	104.60633
S100A14	NC_000001	153586731	153588790	0	0	0	0.0293623
S100A13	NC_000001	153591275	153606568	13.814091	28.855544	11.063246	8.3383489
S100A1	NC_000001	153600873	153604512	0	0.0761946	0.0659318	0.1549319
C1orf77	NC_000001	153606525	153617979	9.5170185	15.690137	15.028221	12.671547
SNAPIN	NC_000001	153631145	153634326	12.653694	10.826554	12.239238	10.563364
ILF2	NC_000001	153634514	153643479	35.169491	64.944133	60.950485	56.956194

NPR1	NC_000001	153651164	153666468	0.1890247	0.2807093	0.1121077	0.0804955
LOC100130379	NC_000001	153690612	153700173	0.1927554	0	0	0
INTS3	NC_000001	153700567	153746555	4.628194	5.1816753	5.2601143	12.695454
SLC27A3	NC_000001	153747768	153752633	0.2367704	0.3744998	0.5508981	0.4822815
LOC343052	NC_000001	153766423	153775062	0.0493431	0.0338199	0.0731616	0.0229228
GATAD2B	NC_000001	153777201	153895451	2.1333523	2.9908778	3.6336999	3.5506512
LOC645965	NC_000001	153824104	153825927	0	0	0	0
DENND4B	NC_000001	153901977	153919154	4.4581803	4.7264635	4.2685633	3.5319831
CRTC2	NC_000001	153920151	153931043	2.8211162	2.8654594	8.1037491	7.4016044
SLC39A1	NC_000001	153931588	153940188	22.7256	23.680794	25.372339	31.672206
CREB3L4	NC_000001	153940397	153946832	0.8050048	0.6207222	2.2156033	1.945828
JTB	NC_000001	153946745	153950451	18.930687	17.883901	28.739443	19.320524
RAB13	NC_000001	153954127	153958806	9.673181	11.628657	8.1989696	10.110987
RPS27	NC_000001	153963239	153964631	35.559253	53.808154	98.133776	67.357282
NUP210L	NC_000001	153965166	154127592	0.0448146	0.0230371	0.0531578	0.0572525
RPL34P5	NC_000001	153968108	153968439	0	0	0	0
RPS7P2	NC_000001	154051898	154052441	0	0	0	0
TPM3	NC_000001	154127780	154164609	0.7272943	1.067038	1.1051821	2.013533
C1orf189	NC_000001	154171848	154178809	0.1135613	0	0	0.0791341
C1orf43	NC_000001	154179183	154193273	51.705164	43.897889	75.846805	59.378057
UBAP2L	NC_000001	154192655	154243329	4.3090369	4.643033	7.6581052	10.856475
HAX1	NC_000001	154245039	154248351	21.992817	22.34175	29.081934	24.369584
AQP10	NC_000001	154293592	154297801	0.0245384	0.025228	0	0.0170993
ATP8B2	NC_000001	154298036	154323780	12.104415	12.079015	6.6558286	6.5399153
RNU7-57P	NC_000001	154311220	154311278	0	0	0	0
MRPS33P1	NC_000001	154350428	154350594	0	0	0	0
RPSAP17	NC_000001	154350602	154351645	0	0	0	0
IL6R	NC_000001	154377669	154440188	0.4735801	0.1514769	1.4511788	1.7747176
SHE	NC_000001	154451957	154474589	0	0.0144749	0.0062626	0.0098109
TDRD10	NC_000001	154474695	154520623	0.0173777	0.1965273	0.0154597	0.0121095
UBE2Q1	NC_000001	154521051	154531120	6.4770121	5.0608775	7.1571715	7.0599704
CHRNB2	NC_000001	154540257	154552354	1.4908125	1.3193964	0.8887364	0.5622683
ADAR	NC_000001	154554533	154600437	46.472793	39.610089	71.085955	27.392833
KCNN3	NC_000001	154679902	154842754	0.0136147	0.0699867	0	0.0094873
PMVK	NC_000001	154897208	154909484	13.160787	11.791511	8.9391663	12.730896
LOC100289369	NC_000001	154915184	154918584	0	0.4003593	0.1484718	0.1938283
PBXIP1	NC_000001	154916556	154928567	2.8437899	2.2457521	4.9009452	2.7571006
PYGO2	NC_000001	154929502	154934258	5.6248209	8.198372	8.0162304	9.4571165
SHC1	NC_000001	154934774	154946959	72.537638	51.273497	106.77412	100.42686
CKS1B	NC_000001	154947118	154951725	15.457283	19.878482	33.922888	14.824543
FLAD1	NC_000001	154955817	154965587	5.1326402	5.4830223	6.2070953	4.9737485
LENEP	NC_000001	154966062	154966791	0	0.2088601	0.0602428	0.1415634
ZBTB7B	NC_000001	154975112	154990999	5.2018372	2.7489902	9.67708	7.1057171
DCST2	NC_000001	154991003	155006257	0.0726717	0	0.1777897	0.0379804
DCST1	NC_000001	155006300	155023406	0.09642	0	0.1029335	0.1343786
ADAM15	NC_000001	155023762	155035252	27.550964	12.990038	42.681839	26.031663
EFNA4	NC_000001	155036213	155042029	1.2936234	1.3659262	2.2705826	2.0221678
EFNA3	NC_000001	155051348	155060014	0.0246623	0.1774881	0.1535819	0.3780849
EFNA1	NC_000001	155100349	155107386	0.028064	0.0577055	0.0748996	0.0391122
RAG1AP1	NC_000001	155108288	155111334	12.565987	9.6893669	13.362459	8.2548071
DPM3	NC_000001	155112367	155112996	7.4720028	7.5994021	10.221119	4.8148803
LOC648822	NC_000001	155120302	155121809	0.2482951	0	0.1472601	0.2306959
KRTCAP2	NC_000001	155141884	155145804	25.469085	22.054787	39.383471	31.240735
TRIM46	NC_000001	155146360	155157445	0.5594937	0.6615011	0.9083776	0.7017795
MUC1	NC_000001	155158300	155162700	2.1508107	1.5110273	5.9953853	6.769447
THBS3	NC_000001	155165379	155177690	11.660875	9.4616657	12.485877	4.719754
LOC100129268	NC_000001	155167951	155175279	0.1653432	0.033998	0.1470939	0.0460871
MTX1	NC_000001	155178490	155183625	12.545174	15.895317	13.992312	8.9097333
GBAP	NC_000001	155183616	155197325	0	0	0	0
MTX1P	NC_000001	155200767	155204227	0	0	0	0
GBA	NC_000001	155204243	155214488	71.334568	38.856011	88.670608	53.044381
C1orf2	NC_000001	155216996	155225274	5.9240563	6.2463574	9.2780117	11.654734
SCAMP3	NC_000001	155225770	155232195	24.833099	23.117089	38.040875	32.06854
CLK2	NC_000001	155232659	155243281	1.9948425	1.4588932	2.2137602	2.550879
LOC100287150	NC_000001	155247217	155252314	0.158372	0.0814115	0.0704461	0.05518
HCN3	NC_000001	155247374	155259638	0.4256487	0.4254558	0.2945203	0.4201961

PKLR	NC_000001	155259084	155271225	0.0138901	0.0285609	0	0.0290375
FDPS	NC_000001	155278539	155290457	35.489465	38.407263	64.308018	51.989775
C1orf104	NC_000001	155290251	155293938	0.067302	0	0.1197475	0
RUSC1	NC_000001	155290640	155300909	5.1641298	5.4185124	4.8588367	4.6648146
LOC100289439	NC_000001	155294310	155296469	0	0	0.0792251	0.0620565
ASH1L	NC_000001	155305052	155532324	1.0712708	1.4198963	1.48766	1.8441514
POU5F1P4	NC_000001	155402931	155404295	0	0	0	0
LOC645676	NC_000001	155531772	155533735	0	0	0	0
LOC100287181	NC_000001	155535838	155536097	0	0	0	0
LOC284620	NC_000001	155560315	155561053	0	0	0	0
MRPS29P1	NC_000001	155568604	155571988	0	0	0	0
MSTO1	NC_000001	155580007	155584758	7.2226397	7.6293285	5.8005405	6.4387551
YY1AP1	NC_000001	155629233	155658791	6.0769472	6.5536866	17.821022	17.015034
DAP3	NC_000001	155658874	155708320	8.2735473	11.65141	11.620402	11.530675
MSTO2P	NC_000001	155715609	155721369	0	0	0	0
GON4L	NC_000001	155719510	155826972	1.3711087	1.4441658	1.503561	1.8172919
LOC100132108	NC_000001	155815298	155815882	0	0	0	0
SYT11	NC_000001	155829290	155854990	7.1237991	5.5683164	5.466212	3.610819
RIT1	NC_000001	155867601	155881177	3.2335633	2.875907	4.235094	2.4052824
KIAA0907	NC_000001	155882836	155904188	3.6262887	3.0684823	9.532106	8.3815456
SNORA42	NC_000001	155889700	155889833	0	0	0	0
SCARNA4	NC_000001	155895749	155895877	0	0	0	0
RXFP4	NC_000001	155911480	155912625	0	0	0	0.0267233
LOC100289466	NC_000001	155916642	155921073	0.0489401	0.0503156	0	0
ARHGEF2	NC_000001	155916645	155947951	5.3789962	4.4662524	9.5706084	10.61392
SSR2	NC_000001	155978839	155990758	44.065531	64.840594	49.358937	35.420233
UBQLN4	NC_000001	156005092	156023516	2.2306203	3.0663387	3.6901324	5.7197876
ROBLD3	NC_000001	156024517	156028301	6.7761591	8.0945302	8.0950907	7.2852133
RAB25	NC_000001	156030966	156040295	0	0	0	0.0564514
MEX3A	NC_000001	156041804	156051789	0.0215292	0.3615262	0.1915296	0.1150183
LOC100287276	NC_000001	156076508	156085070	0.5208679	1.2718291	2.722349	0.8620336
LMNA	NC_000001	156084461	156109878	136.01899	141.24537	136.4094	127.1463
SEMA4A	NC_000001	156123388	156147535	0.1496689	0.1538754	0.61733	0.170665
SLC25A44	NC_000001	156163730	156182587	0.8349196	0.6344586	1.5070648	1.2057703
PMF1	NC_000001	156182784	156209833	15.312831	19.960874	15.031966	17.973011
BGLAP	NC_000001	156211951	156213112	1.0699008	1.0153573	0.5857313	0.8602494
PAQR6	NC_000001	156213207	156217843	0.2698758	0.1494019	0.1108103	0.1012632
LOC100289503	NC_000001	156215422	156218279	0.0847603	0.0871425	0.0754051	0.0295322
SMG5	NC_000001	156219015	156252620	16.007443	14.308572	17.470948	26.879824
TMEM79	NC_000001	156252704	156262234	0.7787283	0.4311002	0.7638325	0.7931023
LOC100289532	NC_000001	156252730	156255648	0	0.0824515	0	0
C1orf85	NC_000001	156262484	156265449	43.975959	34.60735	23.507874	13.872973
VHLL	NC_000001	156268415	156269090	0	0.1336787	0	0.0906061
CCT3	NC_000001	156278752	156308195	5.3886906	8.8599133	5.4654517	10.242385
C1orf182	NC_000001	156307105	156316786	0	0.3008216	0.312364	0.407788
RHBG	NC_000001	156339003	156355011	0	0	0	0.0513553
C1orf61	NC_000001	156374055	156399184	0	0.0514032	0	0.0348406
MEF2D	NC_000001	156433519	156470529	5.2821675	4.0075847	6.2832996	6.3137092
IQGAP3	NC_000001	156495197	156542396	1.263548	1.358787	1.3372763	3.4258167
TTC24	NC_000001	156552019	156556902	0	0	0.0729886	0.0190572
APOA1BP	NC_000001	156561558	156564091	17.485202	23.861351	19.356957	16.965254
GPATCH4	NC_000001	156564279	156571270	0.5585693	0.9877409	0.6161792	0.747328
HAPLN2	NC_000001	156589086	156595517	0	0	0.0441531	0.0172924
LOC100289565	NC_000001	156594933	156597096	0	0	0	0
BCAN	NC_000001	156611740	156629320	0.0325864	0.0335023	0.1642755	0.2497827
NES	NC_000001	156638555	156647189	1.1149156	2.2681125	2.2088225	3.8956079
CRABP2	NC_000001	156669403	156675375	15.964683	63.508559	2.8243855	2.7179975
ISG20L2	NC_000001	156692413	156697705	2.5713251	3.1062212	3.2215937	3.8075787
C1orf66	NC_000001	156698263	156706752	2.7697652	2.0909022	3.6874742	1.9435798
MRPL24	NC_000001	156707094	156710923	8.6758645	14.183786	14.382168	16.154871
HDGF	NC_000001	156711899	156722240	35.576392	50.803067	59.80614	93.450754
PRCC	NC_000001	156737274	156770609	13.000231	15.983389	14.953944	19.041375
SH2D2A	NC_000001	156776035	156786602	0.6567606	0.7033531	2.0692983	1.2966947
NTRK1	NC_000001	156785542	156851642	0.0615306	0.0632599	0.0410545	0.042877
INSRR	NC_000001	156810665	156828666	0.0635856	0	0.0188558	0.0073848
PEAR1	NC_000001	156863523	156886226	3.3778542	1.7271091	0.2330106	0.3398568

C1orf92	NC_000001	156890424	156902880	0.1114871	0.0687723	0.0595092	0.0932264
ARHGEF11	NC_000001	156904632	157015162	4.0892461	4.4534083	3.1441503	3.8319996
LOC246784	NC_000001	157028702	157030596	0	0	0	0
LOC149501	NC_000001	157040296	157044598	0	0	0	0
ETV3L	NC_000001	157061836	157069600	0	0.0914644	0	0.0309968
ETV3	NC_000001	157094459	157108383	2.5198986	2.6678254	2.5753687	1.7664179
CYCSP52	NC_000001	157098154	157098463	0	0	0	0
FCRL5	NC_000001	157483167	157522310	0.0163073	0.0083828	0.0145074	0.0170454
FCRL4	NC_000001	157543539	157567870	0.0381164	0.0130626	0	0.026561
FCRL3	NC_000001	157647978	157670647	0.0145572	0.0598654	0.025901	0.0507202
SONP1	NC_000001	157678786	157681275	0	0	0	0
LOC391106	NC_000001	157694039	157694811	0	0	0	0
FCRL2	NC_000001	157715526	157746909	0.0341611	0	0.0151953	0.0714144
FCRL1	NC_000001	157764651	157789891	0	0	0.0150839	0.0236303
CD5L	NC_000001	157800704	157811634	0.1768145	0.0403964	0.1223437	0.0958311
MRPS21P2	NC_000001	157830929	157831406	0	0	0	0
LOC729866	NC_000001	157893622	157895688	0	0	0	0
KIRREL	NC_000001	157963063	158065846	13.145556	24.367031	18.218124	18.202058
LOC646270	NC_000001	158144147	158147865	0	0	0	0
CD1D	NC_000001	158149737	158156217	0.0348427	0.0477626	0.0413294	0.0404663
RPS10P8	NC_000001	158165372	158165957	0	0	0	0
CD1A	NC_000001	158223927	158228059	0.0209677	0.0646709	0	0.0146111
CD1C	NC_000001	158259563	158264564	0.0339107	0.0348637	0.0150839	0.0236303
CD1B	NC_000001	158297740	158301321	0	0	0	0
CD1E	NC_000001	158323540	158327343	0	0	0	0.0296897
OR10T2	NC_000001	158368312	158369256	0	0.0478131	0.1241193	0.0648146
OR10K2	NC_000001	158389718	158390656	0	0	0	0
OR10T1P	NC_000001	158414858	158415804	0	0	0	0
LOC100129866	NC_000001	158416189	158425063	0	0	0	0
OR10K1	NC_000001	158435352	158436293	0	0	0	0
OR10R2	NC_000001	158449668	158450675	0	0	0	0.0303818
OR10R3P	NC_000001	158460909	158462047	0	0	0	0
OR10R1P	NC_000001	158484575	158485715	0	0	0	0
HSP90AA3P	NC_000001	158493403	158496268	0	0	0	0
OR6Y1	NC_000001	158516918	158517895	0	0	0	0
OR6P1	NC_000001	158532444	158533394	0	0	0	0
OR10X1	NC_000001	158548709	158549689	0	0.0460585	0	0
OR10Z1	NC_000001	158576229	158577170	0	0	0.0415048	0.0325105
SPTA1	NC_000001	158580496	158656506	0.0219275	0.0169078	0.0341378	0.0343799
OR6K1P	NC_000001	158664329	158665299	0	0	0	0
OR6K2	NC_000001	158669468	158670442	0.0450751	0	0	0
OR6K3	NC_000001	158686958	158687953	0	0	0	0.0307479
OR6K4P	NC_000001	158693945	158694826	0	0	0	0
OR6K5P	NC_000001	158712358	158713205	0	0	0	0
OR6K6	NC_000001	158724606	158725637	0	0	0	0.1187011
OR6N1	NC_000001	158735534	158736472	0	0.0481186	0	0
OR6N2	NC_000001	158746472	158747425	0	0	0	0
OR2AQ1P	NC_000001	158765687	158766127	0	0	0	0
OR10AA1P	NC_000001	158778192	158779125	0	0	0	0
MNDA	NC_000001	158801168	158819270	0	0	0	0.0183713
LOC646377	NC_000001	158845370	158851069	0.0743625	0.0764525	0.0661549	0.0518187
PYHIN1	NC_000001	158901342	158946844	0.1898867	0.260298	0.5818649	0.6763055
IFI16	NC_000001	158979682	159024945	61.79115	41.250099	115.13332	57.855705
AIM2	NC_000001	159032275	159046647	1.3918914	0.7916229	3.7147957	0.5984646
LOC100129690	NC_000001	159050922	159116840	0	0	0	0
CADM3	NC_000001	159141377	159172932	0	0.0122183	0.0211452	0.0248444
LOC100131825	NC_000001	159167352	159169634	0	0	0	0
LOC100286919	NC_000001	159170964	159174454	0.0307008	0	0.0409685	0.0320903
DARC	NC_000001	159174510	159176290	0.0325301	0	0	0.0453366
LOC649458	NC_000001	159236133	159246901	0	0	0	0
OR10J2P	NC_000001	159248734	159249891	0	0	0	0
FCER1A	NC_000001	159259504	159278014	0	0.038784	0	0.0262874
OR10J3	NC_000001	159283460	159284449	0.0443922	0	0	0.0928027
OR10J7P	NC_000001	159320841	159321813	0	0	0	0
OR10J8P	NC_000001	159335951	159336876	0	0	0	0
OR10J9P	NC_000001	159375113	159376299	0	0	0	0

OR10J4	NC_000001	159401894	159403025	0	0	0	0
OR10J1	NC_000001	159409549	159410511	0	0	0	0
OR10J5	NC_000001	159504868	159505797	0	0	0.0420404	0
LOC646430	NC_000001	159526585	159527986	0	0	0	0
OR10AE1P	NC_000001	159551413	159552010	0	0	0	0
APCS	NC_000001	159557616	159558661	0	0.0485321	0	0.0328946
OR10J6P	NC_000001	159567988	159569114	0	0	0	0
CRPP1	NC_000001	159674660	159675684	0	0	0	0
CRP	NC_000001	159682079	159684379	0.0218105	0	0	0
RPL27P2	NC_000001	159728934	159729410	0	0	0	0
DUSP23	NC_000001	159750759	159752333	5.3892722	6.9918841	7.7053596	6.7955934
FCRL6	NC_000001	159772173	159786047	0.0226188	0	0.0402445	0.0630466
SLAMF8	NC_000001	159796479	159807282	0.0133176	0.0547678	0.0118477	0
C1orf204	NC_000001	159804264	159825137	0.1581504	0.0722645	0.2501244	0.2571461
VSIG8	NC_000001	159824106	159832447	0.2659134	0.0745601	0.2795756	0.2358351
CCDC19	NC_000001	159842154	159869906	0.1224185	0.0503436	0.0653441	0.0853061
LOC100286989	NC_000001	159879800	159880159	0	0	0	0
TAGLN2	NC_000001	159887903	159895284	73.871807	120.8656	79.488801	99.891147
IGSF9	NC_000001	159896829	159915386	0.0108675	0.0782106	0	0.0378646
SLAMF9	NC_000001	159921284	159924010	0	0.0806846	0	0
RPL27AP2	NC_000001	159987799	159990574	0	0	0	0
PIGM	NC_000001	159997462	160001783	0.6812891	0.6168026	0.2261544	0.7369244
KCNJ10	NC_000001	160008033	160039961	0	0.0305293	0.0088058	0.0068975
LOC100287448	NC_000001	160011399	160040273	0.0632804	0.0325294	0	0.0220481
KCNJ9	NC_000001	160051360	160059212	0	0.0298339	0.0387232	0.0606633
IGSF8	NC_000001	160061130	160068408	3.7332785	1.9290449	5.2485729	1.1322578
ATP1A2	NC_000001	160085520	160113381	0.0563026	0.0330772	0.6225271	0.1008872
ATP1A4	NC_000001	160121352	160156767	0.0111121	0.0571219	0.049428	0.0154867
CASQ1	NC_000001	160160365	160171676	0.0936065	0.2646525	0.1040936	0.0652287
PEA15	NC_000001	160175125	160185162	108.86488	83.540798	89.343242	153.12439
WDR42A	NC_000001	160185505	160231979	8.1402552	9.0037684	12.947763	18.084931
LOC100287049	NC_000001	160222704	160232568	1.1693543	0.5009246	1.2136717	1.0864702
RPSAP18	NC_000001	160236059	160237093	0	0	0	0
PEX19	NC_000001	160246599	160254941	8.4446725	7.7707077	14.194047	10.475394
COPA	NC_000001	160258377	160313354	39.635627	52.511949	58.49454	45.526535
SUMO1P3	NC_000001	160287055	160288264	0	0	0	0
NCSTN	NC_000001	160313063	160328742	15.887248	10.760885	38.511435	15.985456
NHLH1	NC_000001	160336861	160342638	0.034348	0.0353133	0.168063	0.0718051
VANGL2	NC_000001	160370364	160398464	0.0246531	0	0.0146214	0.0343585
LOC100287088	NC_000001	160388844	160390246	0	0.0744372	0	0
SLAMF6	NC_000001	160454820	160493042	0.0160806	0	0.0143057	0.0336168
CD84	NC_000001	160515781	160549251	0.0134152	0	0.0119345	0.0654378
SLAMF1	NC_000001	160579891	160617081	0.0878525	0.0903216	0	0.0306096
LOC100129738	NC_000001	160639746	160640774	0	0	0	0
CD48	NC_000001	160648536	160681585	0.0410731	0	0	0.0286214
SLAMF7	NC_000001	160709077	160724601	0	0.0170054	0	0.0115261
LOC100128858	NC_000001	160743649	160751119	0	0	0	0
LY9	NC_000001	160765928	160798045	0.0391695	0.0268469	0.0232309	0.0272949
CD244	NC_000001	160799950	160832645	0.0178434	0	0.015874	0.0248679
ITLN1	NC_000001	160846330	160854960	0	0	0	0.0259094
LOC646347	NC_000001	160864679	160866356	0	0	0	0
LOC649671	NC_000001	160905327	160905929	0	0	0	0
ITLN2	NC_000001	160914816	160924589	0	0.0392899	0	0.0532607
F11R	NC_000001	160965001	160991133	0.0727921	0.0561284	0.0242842	0.0253622
KAT	NC_000001	161007421	161008774	0.065302	0	0	0.045505
USF1	NC_000001	161009041	161015757	7.7037802	12.597042	9.7907093	9.9867438
ARHGAP30	NC_000001	161016732	161039760	0.099318	0.0510547	0.0530136	0.0968923
PVRL4	NC_000001	161040781	161059385	0.3639345	0.1032174	0.0893148	0.0787047
KLHDC9	NC_000001	161068154	161070136	0.1934625	0.1325999	1.0900275	0.8088743
PFDN2	NC_000001	161070346	161087866	9.7206369	14.638859	16.260203	9.3496513
NIT1	NC_000001	161087891	161090857	5.2273227	5.0756685	4.7364892	3.5526658
DEDD	NC_000001	161090767	161102478	6.3092211	7.1907957	7.6495237	7.9011684
UFC1	NC_000001	161123534	161128646	13.435155	13.369777	15.576303	18.151109
USP21	NC_000001	161129254	161135516	1.9229685	2.7063975	4.4013827	6.2446651
PPOX	NC_000001	161136181	161141010	1.7648894	1.0478051	2.3440848	1.8014634
B4GALT3	NC_000001	161141101	161147314	4.4872746	7.7662594	5.9779757	5.326749

ADAMTS4	NC_000001	161159538	161168845	0.7710216	2.2946326	0.0902529	0.0636251
NDUFS2	NC_000001	161169105	161184185	13.817221	15.378288	19.108409	17.082143
FCER1G	NC_000001	161185087	161189038	0.0743625	0.6116197	0.2646197	0.207275
APOA2	NC_000001	161192083	161193418	0.0929138	0	0	0
TOMM40L	NC_000001	161195833	161200408	3.5270137	5.4307784	3.8090573	3.738087
NR1I3	NC_000001	161199456	161208000	0.0312132	0.064181	0.1388408	0.1522544
PCP4L1	NC_000001	161228517	161255240	0	0.0634598	0.0274562	0
MPZ	NC_000001	161274525	161279762	0.7418372	0.2773406	1.219924	0.4856119
SDHC	NC_000001	161284166	161334541	4.0595987	4.8218816	7.3872237	8.5616787
LOC642502	NC_000001	161284196	161332223	0.6462975	0.3322309	0.2874821	0.4503659
C1orf192	NC_000001	161334521	161337664	1.3627359	0.7588943	2.6772235	2.7696918
LOC148430	NC_000001	161375858	161376911	0	0	0	0
LOC100132578	NC_000001	161416517	161419988	0	0	0.0562555	0
LOC100133178	NC_000001	161423897	161427369	0	0	0	0.0440646
LOC729907	NC_000001	161431308	161434779	0	0	0.112511	0.0440646
LOC729912	NC_000001	161438688	161442160	0	0	0	0.0881291
FCGR2A	NC_000001	161475205	161489360	0.126652	0.3348297	1.0784425	0.5547528
TRNAD20P	NC_000001	161492935	161493006	0	0	0	0
HSPA6	NC_000001	161494036	161496687	0.1988608	0.2044498	0.132684	0.0692871
FCGR3A	NC_000001	161511549	161520413	0.0182889	0	0	0.0509777
FCGR2C	NC_000001	161551138	161570032	0.1171172	0.0602044	0.0520954	0.061209
HSPA7	NC_000001	161575849	161578341	0	0	0	0
FCGR3B	NC_000001	161592986	161601158	0.0191496	0	0	0.0133442
FCGR2B	NC_000001	161632940	161647951	0	0	0.0239422	0
RPL31P11	NC_000001	161653485	161655042	0	0	0	0
FCRLA	NC_000001	161676762	161684142	0.59972	0.9055948	0.2834365	0.1828351
FCRLB	NC_000001	161692457	161697933	0.372864	0.7411305	0.2211401	0.1905394
DUSP12	NC_000001	161719581	161726952	3.8001674	4.3410794	3.8815838	3.9476424
ATF6	NC_000001	161736084	161928852	6.4473893	8.6988945	15.698741	14.980095
OLFML2B	NC_000001	161952982	161993644	4.0749468	10.266355	4.9985495	1.7832208
NOS1AP	NC_000001	162039581	162339813	0.0829814	0.0590633	0.0681439	0.0533767
C1orf111	NC_000001	162343515	162346644	0	0	0.0666625	0.1044327
C1orf226	NC_000001	162348696	162356608	0.1447026	0.0850111	0.183902	0.0432148
LOC100287460	NC_000001	162351463	162354948	0	0.0984388	0.1277698	0.1000813
SH2D1B	NC_000001	162365056	162381928	0.0173366	0.1425906	0.0308462	0.0604041
UHMK1	NC_000001	162467655	162493844	9.1988196	9.9403484	12.511221	11.648014
LOC100130719	NC_000001	162511122	162511449	0	0	0	0
UAP1	NC_000001	162531296	162569633	4.2305131	5.4755992	4.8388908	10.739106
DDR2	NC_000001	162602228	162750255	31.866522	26.356984	28.842269	35.211076
HSD17B7	NC_000001	162760496	162782608	2.430317	2.7960762	2.6768577	2.0967659
LOC100287496	NC_000001	162792185	162799026	0	0	0	0
C1orf110	NC_000001	162824087	162838605	0.2548544	0.029113	0.1511504	0.3551854
LOC100287509	NC_000001	162949341	162949583	0	0	0	0
RGS4	NC_000001	163038396	163046592	30.856564	12.914411	15.813973	44.867217
RGS5	NC_000001	163112097	163172872	0.1809811	0.0620225	0.1274629	0.2207009
LOC100113374	NC_000001	163206996	163209777	0	0	0	0
NUF2	NC_000001	163291723	163325553	0.552391	0.2953164	1.1007862	2.3403627
LOC100287537	NC_000001	163738513	163740797	0	0	0	0
LOC100129160	NC_000001	164312242	164313108	0	0	0	0
LOC729952	NC_000001	164325998	164326621	0	0	0	0
PBX1	NC_000001	164528802	164821045	0.8680483	5.2329722	0.3978205	0.7881908
RPL35AP7	NC_000001	164890513	164890929	0	0	0	0
LMX1A	NC_000001	165171105	165325017	0	0.0491124	0	0.016644
RXRG	NC_000001	165370350	165414430	0.1038474	0.0213532	0	0.028946
LOC400794	NC_000001	165446079	165551341	0	0	0	0
LOC100129558	NC_000001	165466962	165467590	0	0	0	0
LRRC52	NC_000001	165513478	165533185	0	0	0	0.0272949
RPS2P10	NC_000001	165550795	165551752	0	0	0	0
TRNAK35P	NC_000001	165566150	165566222	0	0	0	0
MGST3	NC_000001	165600450	165624857	30.359412	27.87332	56.022153	39.678387
ALDH9A1	NC_000001	165631449	165667900	13.887641	13.12126	17.6721	12.335701
RPS13P1	NC_000001	165640493	165640968	0	0	0	0
RPL21P27	NC_000001	165645509	165646053	0	0	0	0
LOC440700	NC_000001	165677420	165678847	0	0	0	0
TMCO1	NC_000001	165693528	165738135	5.7727772	6.4634826	8.2750358	8.7893263
RPL26P12	NC_000001	165788579	165789068	0	0	0	0

UCK2	NC_000001	165796890	165877343	18.317623	30.290953	21.5193	27.56239
RPS3AP10	NC_000001	165991426	165992312	0	0	0	0
FAM78B	NC_000001	166039256	166136206	0	0.0848249	0	0.038329
LOC284685	NC_000001	166244866	166246864	0	0	0	0
FMO8P	NC_000001	166535415	166549948	0	0	0	0
FMO9P	NC_000001	166573153	166594475	0	0	0	0
FMO10P	NC_000001	166618387	166668597	0	0	0	0
RPL4P2	NC_000001	166716560	166717986	0	0	0	0
LOC646693	NC_000001	166765503	166766541	0	0	0	0
POGK	NC_000001	166808724	166823709	3.5140331	4.2344777	4.6182223	5.7322267
TADA1L	NC_000001	166825747	166845654	1.7000872	2.1295868	3.4247312	3.1047009
ILDR2	NC_000001	166887996	166944561	0.0170872	0.0351348	0.0152012	0.0357211
MAEL	NC_000001	166958519	166991447	0	0.0261025	0.0225867	0.017692
GPA33	NC_000001	167022082	167059868	0.0157351	0.0647095	0.0139984	0.0657892
LOC100287686	NC_000001	167042624	167059847	0	0	0	0.176005
DUSP27	NC_000001	167064087	167098402	0.0217889	0.0224013	0.04846	0.0303668
RPS17P6	NC_000001	167131617	167132099	0	0	0	0
POU2F1	NC_000001	167190143	167385342	1.0133099	0.7393342	3.8385115	3.2458499
CD247	NC_000001	167399877	167487847	0.0261441	0.0268789	0.1162926	0.0546548
AKR1D1P	NC_000001	167488787	167505324	0	0	0	0
CREG1	NC_000001	167510250	167523056	13.384037	8.8761076	21.034722	13.695034
RPS18P4	NC_000001	167560586	167561113	0	0	0	0
RCSD1	NC_000001	167599474	167675486	0.1910154	0.2114903	0.1045739	0.1023901
MPZL1	NC_000001	167691208	167760060	14.766064	17.676425	20.799142	17.196502
LOC100287751	NC_000001	167691216	167737357	0.2009828	0.0688771	0.0595999	0.0466843
ADCY10	NC_000001	167778838	167883453	0.0590702	0.0173515	0.0375361	0.0529232
BRP44	NC_000001	167887310	167906278	8.2865353	11.815637	14.348938	13.6798
IQWD1	NC_000001	167905908	168045081	3.1896834	4.3632924	4.6541894	7.5422947
GPR161	NC_000001	168053997	168105624	1.8229648	3.7146295	3.3311827	2.4834075
TIPRL	NC_000001	168148171	168171352	4.6950906	7.9562077	10.017233	7.8654579
RPL34P1	NC_000001	168179810	168180221	0	0	0	0
LOC100287608	NC_000001	168184643	168184772	0	0	0	0
SFT2D2	NC_000001	168195255	168212088	2.618411	3.9588261	5.3896177	6.2609272
ANKRD26L1	NC_000001	168214803	168216668	0	0	0	0
TBX19	NC_000001	168250278	168283664	0.2180281	0.1441001	0.3740731	0.1085219
LOC441914	NC_000001	168338292	168338839	0	0	0	0
LOC730016	NC_000001	168418914	168420554	0	0	0	0
XCL2	NC_000001	168510003	168513235	0	0	0	0
RPL7AP19	NC_000001	168511969	168512595	0	0	0	0
XCL1	NC_000001	168545856	168551315	0.1078925	0	0	0.0250613
RPL7AP21	NC_000001	168547839	168548701	0	0	0	0
DPT	NC_000001	168664706	168698428	0.0774191	0.1591899	0.0459161	0.0179829
MGC4473	NC_000001	168756179	168762126	0	0	0	0
SUMO1P2	NC_000001	168867347	168868281	0	0	0	0
RPL29P7	NC_000001	168907654	168908285	0	0	0	0
ATP1B1	NC_000001	169075947	169101960	3.8954115	7.290958	7.6595685	8.3383191
NME7	NC_000001	169101769	169337186	3.2494072	4.1759151	8.0459562	7.4345053
BLZF1	NC_000001	169337194	169365782	4.4175746	2.8848764	5.2287512	3.0651302
C1orf114	NC_000001	169364114	169396670	0.0942084	0.024214	0.0419052	0.0492361
SLC19A2	NC_000001	169433147	169455208	1.5704426	1.6766793	4.3632797	2.4749077
LOC100287813	NC_000001	169455384	169459388	0	0	0	0
F5	NC_000001	169481192	169555769	0.0095758	0	0.0127784	0.016682
SELP	NC_000001	169558087	169599377	0.0138551	0	0	0
SELL	NC_000001	169659808	169680837	0.0541679	0.0371269	0.176694	0.0125821
SELE	NC_000001	169691781	169703220	0.022683	0	0.0100897	0.1027415
C1orf156	NC_000001	169761670	169764031	1.003662	1.6885147	1.5693141	1.3987834
C1orf112	NC_000001	169764550	169822229	1.6988612	3.4126031	4.5689394	4.899252
SCYL3	NC_000001	169821804	169863076	0.8594995	0.7537064	0.8995701	1.1362121
KIFAP3	NC_000001	169890470	170043879	2.4782286	2.5026509	2.2960199	1.7371469
MRPS10P1	NC_000001	169959216	169959346	0	0	0	0
SIGLECP14	NC_000001	170084660	170085287	0	0	0	0
METTL11B	NC_000001	170115188	170136923	0.0468531	0.0963399	0.0416818	1.0121228
LOC284688	NC_000001	170240546	170253349	0	0	0	0
LOC100129402	NC_000001	170338364	170431445	0	0	0	0
GORAB	NC_000001	170501270	170522584	3.0357653	2.6250192	3.9169107	2.8159197
PRRX1	NC_000001	170633313	170708541	20.327811	44.661756	12.148961	13.954593

C1orf129	NC_000001	170904636	170967947	0.0389267	0.0200104	0	0.0271257
LOC646804	NC_000001	171046088	171053295	0	0	0	0
FMO3	NC_000001	171060036	171086959	0.0212413	0	0.0188968	0.0148018
FMO6P	NC_000001	171106879	171130702	0	0	0	0
FMO2	NC_000001	171154388	171181822	0.0169652	0	0.0075463	0.023644
FMO1	NC_000001	171217663	171255113	0.0620154	0	0	0.0144049
FMO4	NC_000001	171283486	171311223	6.1638474	4.5204526	3.0524981	1.4317381
TOP1P1	NC_000001	171308035	171310465	0	0	0	0
LOC100287900	NC_000001	171361381	171364078	0	0	0	0
CYCSP53	NC_000001	171413823	171414102	0	0	0	0
BAT2D1	NC_000001	171454666	171562650	2.2431205	3.0980915	5.9489747	6.5502557
MYOC	NC_000001	171604557	171621773	0.0214906	0	0.0191186	0
LOC730138	NC_000001	171639417	171640228	0	0	0	0
RPL4P3	NC_000001	171652206	171653632	0	0	0	0
VAMP4	NC_000001	171672160	171711214	1.9638203	2.2315415	3.4757478	2.9242005
LOC391136	NC_000001	171731134	171734629	0	0	0	0
METTL13	NC_000001	171750761	171766856	2.5540798	3.2624352	2.6967795	3.991032
RPS15P3	NC_000001	171771113	171783110	0	0	0	0
RPLP1P3	NC_000001	171793751	171794101	0	0	0	0
DNM3	NC_000001	171810621	172381857	0.0977394	0.1063973	0.1380997	0.152243
C1orf105	NC_000001	172389830	172437966	0.0896903	0	0	0.0312499
PIGC	NC_000001	172410597	172413230	0.2929882	0.3012227	0.2606504	0.0612498
C1orf9	NC_000001	172502260	172580973	2.2226692	2.5008673	3.6850579	2.5994592
LOC100287669	NC_000001	172502323	172525115	0.0810853	0	0.1442715	0.5085312
FASLG	NC_000001	172628185	172636013	0.0472562	0	0.0420404	0.03293
LOC441915	NC_000001	172717392	172719311	0	0	0	0
TNFSF18	NC_000001	173010360	173020103	0	0	0.0522695	0.0409424
GOT2L2	NC_000001	173110039	173111511	0	0	0	0
TNFSF4	NC_000001	173152870	173176471	0.0125818	0.3233854	0.0783518	0.017535
RPL26P11	NC_000001	173314999	173321149	0	0	0	0
LOC646870	NC_000001	173331503	173411085	0	0	0	0
PRDX6	NC_000001	173446486	173457946	25.658399	33.874023	43.475557	37.355016
SLC9A11	NC_000001	173469604	173572233	0.0099273	0.0102063	0.0353265	0.0069177
ANKRD45	NC_000001	173577475	173639001	0.4771616	0.4567397	0.5562364	0.0687942
LOC730159	NC_000001	173604736	173606273	0.0789017	0.2433576	0.1403862	0.1649455
KLHL20	NC_000001	173684080	173755840	1.1672163	1.4348078	0.9819539	0.6984311
RPS27P7	NC_000001	173710782	173711126	0	0	0	0
CENPL	NC_000001	173768688	173794026	2.3154388	3.1534088	4.0662538	4.6204486
DARS2	NC_000001	173793958	173827682	2.6614398	4.4800612	2.6130472	2.6425609
GAS5	NC_000001	173833039	173837125	0	0	0	0
SNORD81	NC_000001	173833313	173833355	0	0	0	0
SNORD47	NC_000001	173833507	173833583	0	0	0	0
SNORD80	NC_000001	173833971	173834041	0	0	0	0
SNORD79	NC_000001	173834488	173834568	0	0	0	0
SNORD78	NC_000001	173834771	173834824	0	0	0	0
SNORD44	NC_000001	173835106	173835166	0	0	0	0
SNORD77	NC_000001	173835449	173835509	0	0	0	0
SNORD76	NC_000001	173835773	173835853	0	0	0	0
SNORD75	NC_000001	173836017	173836076	0	0	0	0
SNORD74	NC_000001	173836812	173836883	0	0	0	0
ZBTB37	NC_000001	173837493	173855774	0.5176906	0.3611631	0.5427933	0.9791715
SERPINC1	NC_000001	173872939	173886473	0.05638	0	0.0250786	0.0392878
RC3H1	NC_000001	173900352	173962210	2.1862207	2.1942467	4.0925153	4.0467396
LOC100288007	NC_000001	173962230	173992193	0.405676	0.2085388	0.6616511	0.7538432
RPL30P1	NC_000001	174059272	174059610	0	0	0	0
RNU7-78P	NC_000001	174064280	174064535	0	0	0	0
RABGAP1L	NC_000001	174128634	174927327	0.4248772	0.6142759	0.6496574	0.8419528
GPR52	NC_000001	174417212	174418683	0	0	0	0
RPS26P12	NC_000001	174891238	174891571	0	0	0	0
CACYBP	NC_000001	174968571	174981163	6.575663	9.5682498	11.605431	15.03991
MRPS14	NC_000001	174983128	174992561	2.5612753	2.5078669	3.4359563	3.7679082
LOC646891	NC_000001	175013638	175014848	0	0	0	0
TNN	NC_000001	175036994	175117202	0.0087756	0	0.007807	0.0489215
KIAA0040	NC_000001	175126123	175161929	0	0	0	0
LOC100288070	NC_000001	175169921	175201060	0	0	0	0.075061
RPS29P4	NC_000001	175266216	175266377	0	0	0	0

TNR	NC_000001	175291935	175712752	0.0254036	0.0174117	0.0150665	0.0354045
RPS29P5	NC_000001	175891081	175891367	0	0	0	0
RFWD2	NC_000001	175913967	176176370	1.2991186	2.4269383	2.0436723	2.6827128
SCARNA3	NC_000001	175937533	175937676	0	0	0	0
RPL13P7	NC_000001	176200320	176200966	0	0	0	0
LOC100129734	NC_000001	176241736	176242546	0	0	0	0
LOC100130637	NC_000001	176336908	176343519	0	0	0	0
PAPPA2	NC_000001	176432307	176811972	0.4111047	0.2348105	2.031834	0.2188344
ASTN1	NC_000001	176830203	177134024	0.0419125	0.0369347	0.0319599	0.0667572
FAM5B	NC_000001	177140633	177251558	0.0370558	0.0126991	0.0329659	0.051644
SEC16B	NC_000001	177898242	177939050	1.0484668	0.5951094	1.2922406	0.875214
LOC100131700	NC_000001	177977216	178024960	0	0	0	0
RASAL2	NC_000001	178063630	178443558	3.3317236	4.4625582	4.7891501	7.0122937
RPS14P2	NC_000001	178380719	178381198	0	0	0	0
NCRNA00083	NC_000001	178463034	178464192	0	0	0	0
C1orf49	NC_000001	178482212	178491784	0.0859203	0	0	0.0299363
C1orf220	NC_000001	178511931	178518024	0.0511621	0.1753333	0.1365456	0.3089821
LOC646976	NC_000001	178693441	178695678	0	0	0	0
RALGPS2	NC_000001	178694300	178889238	3.6083653	2.7881427	7.4857698	6.3097538
LOC646981	NC_000001	178715473	178716820	0	0	0	0
ANGPTL1	NC_000001	178818670	178840215	0.1363312	0.0254842	0.0110258	0
FAM20B	NC_000001	178995074	179045702	9.3129452	9.0123637	14.978852	18.567556
RPS26P16	NC_000001	179004366	179004809	0	0	0	0
TOR3A	NC_000001	179051112	179065131	9.9111125	17.230958	17.082176	18.455671
ABL2	NC_000001	179068462	179198819	5.2274606	3.3469187	7.110336	10.741489
LOC100287822	NC_000001	179152976	179153830	0	0	0	0
LOC100130006	NC_000001	179170759	179191260	0	0	0	0
RPL39P11	NC_000001	179240251	179240401	0	0	0	0
SOAT1	NC_000001	179263017	179324453	16.087151	13.250069	14.565082	15.822402
C1orf125	NC_000001	179335115	179523870	0.0259895	0.0267199	0.0578024	0.0905526
MEF2AP	NC_000001	179416723	179417575	0	0	0	0
NPHS2	NC_000001	179519674	179545084	0.0234892	0	0.0208966	0.0327364
LOC126860	NC_000001	179555840	179558251	0	0	0	0
LOC100287850	NC_000001	179559507	179560440	0.2352689	0.1935049	0.2930224	0.2623116
TDRD5	NC_000001	179561025	179660398	0	0.0128508	0.0111199	0.0348406
FAM163A	NC_000001	179712298	179785333	0	0.0155323	0	0.0210553
LOC100288340	NC_000001	179785319	179787398	0	0	0	0.0595815
TOR1AIP2	NC_000001	179813953	179846934	3.2522267	3.4323999	4.4423233	4.9938407
IFRG15	NC_000001	179833916	179834311	0	0	0	0
LOC100287878	NC_000001	179850819	179851983	0.188619	0.0775681	0.1342406	0.1051498
TOR1AIP1	NC_000001	179851420	179889211	8.9120134	11.666741	6.4803162	6.3630887
CEP350	NC_000001	179923908	180084015	0.5911586	0.7555191	1.2755523	1.9982642
RPSAP16	NC_000001	179937730	179938716	0	0	0	0
RPS24P5	NC_000001	179969575	179969943	0	0	0	0
QSOX1	NC_000001	180123968	180167169	35.479317	18.190543	54.837991	25.83166
FLJ23867	NC_000001	180167144	180169860	0	0	0	0
LHX4	NC_000001	180199442	180244188	0.0940069	0.096649	0.1672623	0.5731929
ACBD6	NC_000001	180257365	180471479	1.741345	2.6428028	5.2375984	5.4315822
VDAC4P	NC_000001	180404917	180405078	0	0	0	0
XPR1	NC_000001	180601146	180859415	1.72772	2.0368695	2.7289144	2.458353
KIAA1614	NC_000001	180882313	180915239	0.9731016	0.8917061	1.4020547	1.6362751
LOC100287911	NC_000001	180922123	180923254	0.3494117	0	0.069077	0.0541076
LOC100129575	NC_000001	180933624	180935114	0	0	0	0
STX6	NC_000001	180942175	180992046	4.0014594	3.6158675	4.3527934	4.4762554
MR1	NC_000001	181003139	181024633	8.3512079	5.9743691	8.5748422	5.2132611
IER5	NC_000001	181057638	181059979	3.28392	3.2218737	3.3722068	1.9483804
LOC100287948	NC_000001	181205524	181206787	0	0	0	0
LOC100287984	NC_000001	181452624	181480517	0	0.0701606	0	0
CACNA1E	NC_000001	181452716	181770716	0.1175086	0.1022249	0.0683524	0.0440918
ZNF648	NC_000001	182023705	182030847	0.0120439	0	0	0.0167854
LOC100130996	NC_000001	182151816	182172511	0	0.0953236	0	0.0646094
RPL18P2	NC_000001	182297593	182298193	0	0	0	0
LOC730144	NC_000001	182302551	182305475	0	0.2198706	0	0
GLUL	NC_000001	182351669	182360950	11.753597	20.343527	16.482722	14.683047
TEDDM1	NC_000001	182367252	182369751	0.0351586	0.0180734	0.0312781	0.01225
C1orf120	NC_000001	182377042	182383950	0	0	0	0

LOC127671	NC_000001	182410712	182411497	0	0	0	0
RGSL1	NC_000001	182419256	182529732	0.0116884	0.0240337	0.0311949	0.0651593
RNASEL	NC_000001	182542772	182556108	0.4767769	0.4792839	0.3204718	0.6866234
RGS16	NC_000001	182567758	182573548	0.0903541	0.3344166	0.3376023	0.0503699
LOC284648	NC_000001	182575672	182585764	0.2313065	0.0713422	0.1646213	0.0322367
RGS8	NC_000001	182615792	182642067	0	0	0.0428701	0.0335799
NPL	NC_000001	182761394	182798588	0.2265373	0.0873391	0.2015338	0.0789301
LOC647070	NC_000001	182806325	182808967	0	0	0	0
DHX9	NC_000001	182808451	182856884	23.310691	33.27419	25.603393	45.441296
C1orf14	NC_000001	182869000	182922553	0.074331	0.0955252	0.0165317	0.064746
LOC100036575	NC_000001	182910441	182911657	0	0	0	0
LOC100271717	NC_000001	182924325	182925320	0	0	0	0
KRT18P28	NC_000001	182928154	182929547	0	0	0	0
LAMC1	NC_000001	182992595	183114727	15.035654	25.412442	15.224708	12.395137
LAMC2	NC_000001	183155174	183214262	0.4730703	0.4099371	0.5290767	0.4332598
NMNAT2	NC_000001	183217379	183387737	0.2624447	0.2466932	0.3802357	0.8882835
RPS3AP8	NC_000001	183235635	183236406	0	0	0	0
SMG7	NC_000001	183441634	183523326	3.4879549	4.8878525	10.867181	9.0088712
NCF2	NC_000001	183524697	183560046	1.5215571	0.2910364	3.4942269	2.5274155
ARPC5	NC_000001	183595332	183604985	23.238016	29.407966	70.363782	45.272903
RGL1	NC_000001	183605208	183897666	3.3350115	5.9296035	2.2175423	2.1143325
APOBEC4	NC_000001	183615410	183622448	0	0	0	0
GLT25D2	NC_000001	183904966	184006863	0.3561995	0.5754737	0.5507762	0.3605013
TSEN15	NC_000001	184020811	184043346	6.2110511	10.512057	10.392771	8.296842
C1orf21	NC_000001	184356150	184598155	1.2747886	1.7840167	2.3781697	2.3827272
LOC100129573	NC_000001	184535614	184536992	0	0	0	0
EDEM3	NC_000001	184659625	184724041	5.1423142	7.4056631	5.6417929	3.8142566
FAM129A	NC_000001	184760166	184943682	3.1790448	2.726922	6.8079215	11.894444
RNU7-13P	NC_000001	184790564	184790623	0	0	0	0
RNF2	NC_000001	185014551	185071740	5.0742819	8.0289289	7.6961978	5.0797109
C1orf25	NC_000001	185087217	185126116	1.1266221	1.6092999	2.1819422	1.7368919
C1orf26	NC_000001	185126192	185260913	0.5792923	0.5612134	0.6640144	0.6831405
RPL5P5	NC_000001	185195879	185196908	0	0	0	0
IVNS1ABP	NC_000001	185265520	185286461	3.7574221	3.594012	6.6388579	5.3752168
LOC100288079	NC_000001	185301732	185302412	0	0	0	0
HMCN1	NC_000001	185703683	186160085	0.0723944	0.1364533	0.0450829	0.0622183
PRG4	NC_000001	186265405	186283688	0.0261389	0.062705	0.0077513	0.0728586
TPR	NC_000001	186280784	186344457	2.9153957	5.7712627	4.8006076	7.4290882
C1orf27	NC_000001	186345001	186390500	1.1387961	1.2544308	1.9435067	1.9191159
OCLM	NC_000001	186369704	186370587	0	0	0	0
PDC	NC_000001	186412714	186430239	0	0.4836144	0.0597822	0.046827
LOC100131939	NC_000001	186549630	186580406	0	0	0	0
PTGS2	NC_000001	186640944	186649559	6.6416313	13.123601	1.505426	0.2726453
PLA2G4A	NC_000001	186798032	186958113	6.1839725	2.6387847	2.4970098	2.2382937
FDPSL1	NC_000001	187532163	187533307	0	0	0	0
LOC100129274	NC_000001	187683330	187684468	0	0	0	0
RPS3AP9	NC_000001	188663425	188664286	0	0	0	0
LOC100288819	NC_000001	189005110	189005503	0	0	0	0
LOC100131147	NC_000001	189101495	189101906	0	0	0	0
LOC647132	NC_000001	189958580	190003656	0	0	0	0
FAM5C	NC_000001	190066797	190446759	0	0	0	0.0424756
LOC100288924	NC_000001	190650161	190767086	0	0	0	0
LOC100288949	NC_000001	191148603	191148869	0	0	0	0
RGS18	NC_000001	192127592	192154945	0	0	0.0182273	0.0142773
LOC647150	NC_000001	192215343	192216801	0	0	0	0
RGS21	NC_000001	192286122	192336415	0	0	0.043587	0.0512122
RGS1	NC_000001	192544857	192549159	0.0313245	0.0322048	0.0278671	0
RGS13	NC_000001	192605282	192629390	0	0	0	0.0204438
RPS27AP5	NC_000001	192685267	192685813	0	0	0	0
LOC100130137	NC_000001	192769702	192770309	0	0	0	0
RGS2	NC_000001	192778171	192781403	1.8311763	1.5800743	0.9599849	0.4101546
LOC730190	NC_000001	192962579	192963807	0	0	0	0
UCHL5	NC_000001	192984899	193028521	2.6838615	3.6613679	5.4869749	6.1861175
TROVE2	NC_000001	193028753	193060907	5.6048938	6.5856229	9.1899846	5.5717318
GLRX2	NC_000001	193065600	193075244	1.1225168	1.2589802	0.8170544	1.9199808
CDC73	NC_000001	193091161	193221056	4.1441118	5.0035311	5.7990349	6.474387

B3GALT2	NC_000001	193147860	193155743	0	0	0	0.0086316
RPL23AP22	NC_000001	193725943	193726397	0	0	0	0
EEF1AL12	NC_000001	194158034	194159672	0	0	0	0
LOC100289115	NC_000001	194687922	194688366	0	0	0	0
KCNT2	NC_000001	196194913	196577499	0.2826253	0.1529308	0.7212108	0.1813963
CFH	NC_000001	196621008	196716634	6.2668411	1.579767	1.8405161	0.2799349
CFHR3	NC_000001	196743930	196762873	0	0	0	0
CFHR1	NC_000001	196788861	196801319	0.1016536	0.2090211	0.0602892	0
LOC100289145	NC_000001	196819413	196854207	0	0	0	0
CFHR4	NC_000001	196857212	196887763	0	0	0	0
CFHR2	NC_000001	196912934	196928356	0	0	0.037739	0
CFHR5	NC_000001	196946667	196978804	0.0621396	0.063886	0.0829217	0.3788868
F13B	NC_000001	197008321	197036397	0.0792932	0.0407608	0.0352707	0.0552546
ASPM	NC_000001	197053257	197115824	0.8355331	1.7553801	1.856488	4.4581587
ZBTB41	NC_000001	197122814	197169672	1.4365896	1.4129811	2.3530515	2.0057597
LOC127011	NC_000001	197191352	197192385	0	0	0	0
CRB1	NC_000001	197237408	197447585	0.0623758	0	0.0475639	0.1303979
MRPS21P3	NC_000001	197332717	197333342	0	0	0	0
DENND1B	NC_000001	197479507	197744623	0.8124673	1.2336765	2.5019773	1.4371743
LOC730232	NC_000001	197657687	197659778	0	0	0	0
RPL24P5	NC_000001	197704734	197705279	0	0	0	0
C1orf53	NC_000001	197871777	197876497	0.5979351	1.0757953	0.0664925	0.6770806
LHX9	NC_000001	197881635	197899273	0.393311	3.111853	0.0456392	0.0953304
NEK7	NC_000001	198126108	198291550	25.312225	57.643266	16.134603	30.81008
ATP6V1G3	NC_000001	198492352	198510075	0.0648204	0.1999266	0.2883301	0.6323721
PTPRC	NC_000001	198608137	198726545	0.0213929	0.0146628	0.0063439	0.0198766
LOC100129570	NC_000001	198648263	198685291	0	0	0	0
LOC100131234	NC_000001	198868124	198868372	0	0.3629189	0	0
RPS2P9	NC_000001	198918133	198918987	0	0	0	0
RPL23AP16	NC_000001	199341004	199341414	0	0	0	0
LOC100289212	NC_000001	199715844	199722018	0	0	0	0
LOC100289253	NC_000001	199846106	199847532	0	0	0	0
NR5A2	NC_000001	199996770	200146552	0.0435476	0.0268629	0.0464894	0.0242766
FAM58B	NC_000001	200182656	200183643	0.088964	0.0914644	0.0791449	0.0929905
LOC647209	NC_000001	200297147	200300579	0	0	0	0
ZNF281	NC_000001	200375420	200379166	9.3987556	7.0835666	12.203415	10.289472
KIF14	NC_000001	200520625	200589862	2.2828682	2.9989808	3.4922932	4.0443188
DDX59	NC_000001	200613165	200639126	1.5533427	1.7138532	2.9323174	2.9172837
CAMSAP1L1	NC_000001	200708686	200829832	2.3731567	3.0236988	3.695975	3.9114201
RPL34P6	NC_000001	200832936	200833271	0	0	0	0
GPR25	NC_000001	200842166	200843251	0.121404	0	0.0720029	0.0281997
C1orf106	NC_000001	200860639	200884864	0.0404494	0.2287241	0.0089962	0.0070467
C1orf81	NC_000001	200886588	200935792	0	0	0	0
KIF21B	NC_000001	200938520	200992828	0.0310902	0.0410966	0.0237075	0.0371398
CACNA1S	NC_000001	201008640	201081694	0.0071252	0.0366273	0.0126776	0.0347559
ASCL5	NC_000001	201083633	201099369	0	0	0	0.0257568
TMEM9	NC_000001	201103900	201123632	3.0269737	4.0808921	7.1894809	3.6216555
IGFN1	NC_000001	201181770	201198074	0.0223883	0	0.0298759	0.0858058
PKP1	NC_000001	201252580	201302117	0.0807427	0.3486502	0.0287324	0.0337588
TNNT2	NC_000001	201328142	201346805	0	0.0388842	0.0336468	0.1054213
LAD1	NC_000001	201349966	201368669	0.1393216	0.2068983	0.2616604	0.0323616
TNNI1	NC_000001	201372895	201390874	0	0.0073553	0.0127291	0.029912
LOC100289392	NC_000001	201390109	201398524	0	0	0	0
PHLDA3	NC_000001	201434622	201438299	7.4213373	6.2887189	7.5048757	7.2319963
CSRP1	NC_000001	201452658	201476387	8.4740254	11.125424	6.5713169	5.8865563
RPS10P7	NC_000001	201489032	201489730	0	0	0	0
LOC100289429	NC_000001	201592013	201598668	3.7348323	7.5218003	2.594367	5.5973293
NAV1	NC_000001	201617450	201796097	4.888392	11.010176	3.0024189	6.732812
IPO9	NC_000001	201798288	201853422	5.2432982	7.6822865	9.6339744	11.859897
SHISA4	NC_000001	201858032	201861430	9.9374617	6.4943715	9.8000909	9.4746556
LOC100128471	NC_000001	201864964	201869883	0.2661225	0.097715	0.0169107	0.0927224
LMOD1	NC_000001	201865584	201915716	0.5760797	0	0.2266811	0.0540394
LOC100288210	NC_000001	201869724	201915676	0.6914932	0.1579839	0.3417619	0.2677
TIMM17A	NC_000001	201924619	201939789	12.000331	16.341194	17.958014	19.88125
RPL10P4	NC_000001	201947670	201948239	0	0	0	0
RNPEP	NC_000001	201951766	201975275	12.494193	13.126218	11.714687	12.742386

LOC100289488	NC_000001	201977305	201984603	0	0.1063139	0.0306648	0
ELF3	NC_000001	201979690	201986316	0.0270617	0	0.1444491	0.0094288
GPR37L1	NC_000001	202092029	202098634	0.0714896	0.0183747	0.0476993	0.0249084
ARL8A	NC_000001	202101977	202113866	8.7334226	7.2062227	9.3534048	4.6622949
PTPN7	NC_000001	202116143	202129751	0.0131503	0.0405596	0	0.0458182
PTPRV	NC_000001	202137179	202158577	0	0	0	0
LGR6	NC_000001	202163118	202288889	0.0120737	0	0.0107411	0.0925477
UBE2T	NC_000001	202300785	202311094	16.020776	23.885499	50.637376	23.643216
PPP1R12B	NC_000001	202317836	202557697	0.1850372	0.1902377	0.5173591	0.5841831
CYCSP4	NC_000001	202338654	202338930	0	0	0	0
RPS27P8	NC_000001	202440934	202441278	0	0	0	0
SYT2	NC_000001	202559724	202679551	0.0338932	0.0464611	0.0502539	0.0826636
KDM5B	NC_000001	202696532	202777549	4.785214	7.4963532	8.0976002	5.6423088
LOC641515	NC_000001	202792409	202795439	0	0	0	0
LOC730222	NC_000001	202822119	202830578	0	0	0	0
LOC148709	NC_000001	202830882	202844369	0	0	0	0
RABIF	NC_000001	202848085	202858263	2.2787972	2.1383091	0.9171034	0.9830208
KLHL12	NC_000001	202860230	202896371	6.4381921	6.7138908	9.3820102	7.0919805
LOC730246	NC_000001	202880940	202881859	0	0	0	0
ADIPOR1	NC_000001	202909960	202927700	12.013663	12.802946	22.972581	19.112267
CYB5R1	NC_000001	202931001	202936404	12.464928	8.717674	10.208677	9.5248404
LOC401980	NC_000001	202955580	202956751	0	0	0.0333597	0.0261304
RPS20P8	NC_000001	202969183	202969467	0	0	0	0
TMEM183A	NC_000001	202976534	202993197	10.393163	16.200895	17.171228	18.595647
PPFIA4	NC_000001	203020311	203047864	0.6010759	0.0716486	0.7284779	1.9910724
MYOG	NC_000001	203052257	203055377	0	0.0259079	0.067255	0.0175601
ADORA1	NC_000001	203096836	203136533	3.598365	1.1764092	3.7369718	4.2805585
LOC100289552	NC_000001	203096911	203134586	0.1219655	0.0835956	0	0.1699808
MYBPH	NC_000001	203136939	203144942	1.0220519	0.100074	2.2298169	2.170534
CHI3L1	NC_000001	203148059	203155922	600.2271	1.0084483	1134.1274	316.08475
CHIT1	NC_000001	203185207	203198860	2.8003425	3.4861923	0.8643155	0.5708148
LOC100289584	NC_000001	203224862	203265237	0	0	0	0
LOC100289621	NC_000001	203274646	203288414	1.1913677	0.0680473	0.5299369	0.230609
BTG2	NC_000001	203274664	203278730	0.1134357	0.1166238	0.1441651	0.0338771
LOC100288349	NC_000001	203309749	203316915	0.0184734	0.094963	0.0328689	0.012873
FMOD	NC_000001	203309752	203320289	2.6492571	2.7842418	0.0261873	0.0307685
LOC100131747	NC_000001	203322683	203322861	0	0	0	0
PRELP	NC_000001	203444883	203460480	0.218986	0.0310539	0.033589	0.0631441
OPTC	NC_000001	203463271	203478077	0.0304351	0	0.0270759	0.1272502
ATP2B4	NC_000001	203595928	203713209	26.540647	22.12754	19.388565	46.097198
LOC100288427	NC_000001	203651155	203652330	0.1309932	0	0.1748028	0.2738439
SNORA77	NC_000001	203698709	203698833	0	0	0	0
LAX1	NC_000001	203734284	203745500	0.0266514	0.0274005	0.0355648	0.083573
ZC3H11A	NC_000001	203764751	203823256	5.2520804	6.8654562	12.208784	13.428028
RPL35AP5	NC_000001	203804668	203805111	0	0	0	0
SNRPE	NC_000001	203830740	203840280	2.4715554	3.0375396	5.8886443	8.3144466
KRT8P29	NC_000001	203841702	203843315	0	0	0	0
LOC100128723	NC_000001	203923526	203927157	0	0	0	0
C1orf157	NC_000001	204001575	204010388	0	0	0.0517849	0
SOX13	NC_000001	204042246	204096871	1.0898039	1.6085424	2.7453729	2.2481804
ETNK2	NC_000001	204100190	204121307	0.1347072	1.2983736	0.0149799	0
REN	NC_000001	204123944	204135465	0	0	0	0
KISS1	NC_000001	204159469	204165619	0	0	0.0547585	0
GOLT1A	NC_000001	204167288	204183220	0	0	0	0.1040483
PLEKHA6	NC_000001	204187981	204329044	0.0178193	0.0366401	0.0264208	0.0496687
RPL21P19	NC_000001	204315885	204316426	0	0	0	0
LOC127841	NC_000001	204337558	204338847	0	0	0	0
PPP1R15B	NC_000001	204372492	204380944	5.0655024	5.8160246	9.1758836	6.154004
LOC100288456	NC_000001	204379183	204381587	0.1096422	0.3193837	0.0650271	0.0127338
PIK3C2B	NC_000001	204391758	204459474	0.0924496	0.2792032	0.071965	0.0442905
LOC730254	NC_000001	204462058	204464522	0.0178289	0	0.0158611	0.0248478
MDM4	NC_000001	204485511	204527248	0.7024387	0.610041	1.1178496	0.7327108
LRRN2	NC_000001	204586301	204654597	0.026443	0.0679654	0	0.1013459
NFASC	NC_000001	204797823	204991949	2.4505314	2.4028478	4.309693	2.7923666
RPL13AP11	NC_000001	204915735	204916297	0	0	0	0
CNTN2	NC_000001	205012340	205047138	0.0460372	0.0236655	0.0358364	0.0320805

TMEM81	NC_000001	205052257	205053588	0.0989825	0.1696074	0.5870505	0.919666
RBBP5	NC_000001	205055979	205091131	3.9974634	4.8336303	6.6878812	7.6582984
LOC100286881	NC_000001	205103774	205107470	0	0	0	0
DSTYK	NC_000001	205111631	205180727	1.9559714	2.8278902	3.9596852	4.4374903
RPL17P8	NC_000001	205171285	205171899	0	0	0	0
TMCC2	NC_000001	205197091	205242470	0.4404095	1.17963	1.5878231	2.2128736
LOC100288519	NC_000001	205238097	205240287	0.1373382	0.1411981	0	0
NUAK2	NC_000001	205271191	205290883	2.3905977	1.8333753	2.8279921	2.1341065
KLHDC8A	NC_000001	205305648	205326039	0.0351586	0.0180734	0.015639	0.01225
LOC100130289	NC_000001	205320375	205320599	0	0	0	0
LEMD1	NC_000001	205350513	205391181	0	0	0	0.0393131
LOC284577	NC_000001	205425045	205426270	0	0	0	0
PCTK3	NC_000001	205473727	205501918	0.6943494	0.1119787	0.0847841	0.0569236
LOC284578	NC_000001	205523401	205525763	0	0	0	0
MFSD4	NC_000001	205538112	205572046	0.0537527	0	0.0765119	0.0449485
LOC100288622	NC_000001	205548694	205550003	0	0	0	0
ELK4	NC_000001	205585235	205602000	1.9808326	2.3554747	5.0424501	4.6980875
SLC45A3	NC_000001	205626981	205649630	0.7277058	1.148957	0.6242663	0.2444919
NUCKS1	NC_000001	205681947	205719361	6.4495545	11.742223	13.420286	15.979229
RAB7L1	NC_000001	205737114	205744610	6.7293372	4.4226455	9.959025	8.738696
SLC41A1	NC_000001	205758221	205782161	6.4102489	5.3989232	7.740577	6.1388559
LOC100131354	NC_000001	205781868	205784368	0.2175655	0.3168803	0.2419404	0.1768764
PM20D1	NC_000001	205797153	205819245	0.0818022	0.1682025	0.0727735	0.1710091
SLC26A9	NC_000001	205882176	205912588	0.0170408	0.0175197	0.0303199	0.0356241
FAM72A	NC_000001	206138911	206155074	0.2118554	0.2420107	0.4607105	0.2788553
RPL22P4	NC_000001	206180054	206180443	0	0	0	0
AVPR1B	NC_000001	206224283	206231482	0	0	0	0
C1orf186	NC_000001	206238872	206243605	0	0.0401274	0	0
CTSE	NC_000001	206317459	206332104	0	0	0.0350966	0.0412364
SRGAP2	NC_000001	206516196	206637783	2.3231457	2.22328	5.2108101	4.2193703
IKBKE	NC_000001	206643796	206670222	2.8418869	1.6755108	3.2112006	1.9146415
C1orf147	NC_000001	206665721	206671050	0	0	0	0
LOC100131192	NC_000001	206680864	206711721	0	0	0	0.0440013
RASSF5	NC_000001	206680879	206762616	0.3388779	0.3701773	0.565266	0.6124976
RPL7AP20	NC_000001	206702226	206703098	0	0	0	0
LGTN	NC_000001	206764974	206785904	9.3890357	10.126943	15.400376	18.225965
DYRK3	NC_000001	206808881	206822542	5.5980056	6.3548526	6.4672665	5.4450248
MAPKAPK2	NC_000001	206858289	206907626	15.722912	17.297853	21.820124	23.201183
RPS14P1	NC_000001	206869127	206869647	0	0	0	0
IL10	NC_000001	206940948	206945839	0	0.0277369	0	0
IL19	NC_000001	206972215	207016325	0.0446628	0	0.0397333	0.0933685
IL20	NC_000001	207039154	207042568	0.0356145	0	0.2217852	0
IL24	NC_000001	207070789	207077484	0.0890091	0	0.9898118	0.0310125
FAIM3	NC_000001	207076630	207096592	0.1930578	0.070887	1.0795688	0.4900749
RPL13AP8	NC_000001	207080943	207082080	0	0	0	0
PIGR	NC_000001	207101872	207119807	0.010302	0.021183	0.0274948	0.0287153
FCAMR	NC_000001	207131404	207143970	0.031974	0	0.0568899	0.0334211
TRNAP27P	NC_000001	207178154	207178230	0	0	0	0
C1orf116	NC_000001	207191866	207206101	0.0159754	0.0328487	0.0568485	0.0222645
YOD1	NC_000001	207217194	207224422	1.3749167	1.0962294	2.8145255	2.6983133
PFKFB2	NC_000001	207226620	207254368	0.201884	0.1087209	0.2223639	0.3717994
C4BPB	NC_000001	207262212	207273338	0.3674601	0.3400089	0.1634514	0.0512122
C4BPA	NC_000001	207277607	207318317	0	0	0.0348619	0.0136535
LOC100129101	NC_000001	207303594	207496039	0.7442214	0.8054082	0.3833095	0.9553215
C4BPAL1	NC_000001	207338840	207357406	0	0	0	0
C4BPAL2	NC_000001	207399143	207402547	0	0	0	0
CD55	NC_000001	207494817	207534311	9.4260964	9.8305686	14.101421	15.322949
CR2	NC_000001	207627670	207663240	0.0106027	0.0109007	0.0282974	0.036942
CR1	NC_000001	207669473	207815110	0.0176393	0	0.0078462	0.0153647
CR1L	NC_000001	207818458	207897036	0.0717717	0	0.0212834	0.0166711
CD46P	NC_000001	207825892	207826080	0	0	0	0
CD46	NC_000001	207925402	207968858	10.586166	9.1546317	41.220038	22.266858
LOC100188945	NC_000001	207935934	207937932	0	0	0	0
LOC100286909	NC_000001	207974863	207977480	0.0839347	0.0172587	0.1344072	0.3392366
C1orf132	NC_000001	207990582	208059030	0	0	0	0
LOC148696	NC_000001	207991724	207995941	0	0	0	0

CD34	NC_000001	208059883	208084683	0.5003	1.0769434	0.1808141	0.1089466
PLXNA2	NC_000001	208195587	208417665	0.2803409	1.0383812	0.1093256	0.2809867
RPS26P13	NC_000001	208870697	208871043	0	0	0	0
LOC391158	NC_000001	209405458	209407862	0	0	0	0
ATP5GP1	NC_000001	209440995	209441628	0	0	0	0
LOC642587	NC_000001	209602168	209605894	0	0.050654	0	0.1373313
CAMK1G	NC_000001	209757045	209787284	0.088964	0.0731715	0	0.0991899
LAMB3	NC_000001	209788215	209825820	1.7489403	2.7825261	6.7973091	1.3344767
G0S2	NC_000001	209848670	209849735	0	0.3284359	0	0
HSD11B1	NC_000001	209859550	209908295	0.5027725	0.4864969	2.2627124	0.2473072
ADORA2BP	NC_000001	209917720	209919021	0	0	0	0
TRAF3IP3	NC_000001	209929524	209955665	0.0854193	0.04391	0.1899785	0.1190471
C1orf74	NC_000001	209955662	209957890	0.7038474	1.3025324	1.7532541	1.4910255
IRF6	NC_000001	209961262	209979479	0.3848002	0.1873966	0.1981904	0.0141128
LOC100286943	NC_000001	209963888	209966188	0	0	0	0
C1orf107	NC_000001	210001333	210030908	2.7938259	3.6945426	2.6009606	3.2242428
SYT14	NC_000001	210111538	210337636	1.1099373	6.2309442	1.880821	2.173022
C1orf133	NC_000001	210404804	210407466	0	0	0	0
SERTAD4	NC_000001	210406195	210416460	0.0650122	0.5792744	0.6554819	0.7097482
LOC100131961	NC_000001	210438979	210476109	0	0	0	0
RNASEH1P3	NC_000001	210477021	210478163	0	0	0	0
HHAT	NC_000001	210501606	210849632	0.8099434	0.4947969	0.3550527	0.5235022
KCNH1	NC_000001	210851657	211307457	0.3632533	0.0891851	0.1784616	0.2833538
RPS25P2	NC_000001	211346760	211347240	0	0	0	0
LOC388734	NC_000001	211380329	211381719	0	0	0	0
RCOR3	NC_000001	211432708	211489727	1.6655737	1.7123848	3.4573945	3.1380214
TRAF5	NC_000001	211499957	211548288	0.9171434	0.8113496	0.8443784	1.1221443
C1orf97	NC_000001	211556161	211605877	0	0	0	0
LOC391160	NC_000001	211615616	211616148	0	0	0	0
RD3	NC_000001	211649864	211666259	0	0.0105915	0.0091649	0.0215365
SLC30A1	NC_000001	211748381	211752099	10.475272	11.84441	13.407094	5.7364737
LOC401981	NC_000001	211833475	211834624	0	0	0	0
NEK2	NC_000001	211836122	211848967	1.506207	2.503118	4.6072718	3.9395383
LPGAT1	NC_000001	211916799	212004114	3.365863	5.1674669	7.1894907	8.3704852
LOC100287655	NC_000001	212098512	212109978	0	0	0	0
INTS7	NC_000001	212114698	212208884	6.0933424	6.3690064	7.9392226	8.0587129
DTL	NC_000001	212208919	212278187	7.8293994	13.251886	16.24339	15.805269
RPL21P28	NC_000001	212224828	212225393	0	0	0	0
PPP2R5A	NC_000001	212458879	212535205	2.0091423	1.9356061	3.2997945	2.6140801
RPL23AP18	NC_000001	212482393	212482860	0	0	0	0
SNORA16B	NC_000001	212526160	212526292	0	0	0	0
TMEM206	NC_000001	212537816	212588267	1.0527864	0.9672288	3.1684572	1.6389461
NENF	NC_000001	212606229	212619721	20.79321	16.283165	24.925213	27.075103
LOC100287008	NC_000001	212617697	212619724	0.4139551	0.1418631	0.3068883	0.0480767
LOC100287039	NC_000001	212731175	212732177	0.0876336	0.0900965	0.311845	0.2137329
ATF3	NC_000001	212738697	212794117	0.653046	0.4355027	1.0677247	0.3689744
FAM71A	NC_000001	212797816	212800117	0	0.0196279	0.0509525	0.0133036
LOC100129948	NC_000001	212820605	212821100	0	0	0	0
BATF3	NC_000001	212859759	212873327	2.0607734	2.6253353	2.0325952	2.7784038
NSL1	NC_000001	212899495	212965139	0.8244449	1.0458488	1.2214292	1.5405101
TATDN3	NC_000001	212965185	212989968	0.5738817	0.723239	1.0540203	0.6450059
RPS5P4	NC_000001	212995656	212997939	0	0	0	0
C1orf227	NC_000001	213003483	213020991	0	0	0	0
RPS28P2	NC_000001	213028511	213028708	0	0	0	0
LQK1	NC_000001	213029946	213031480	0	0	0	0
FLVCR1	NC_000001	213031597	213070197	0.9094504	1.3169164	1.5953539	1.3656678
VASH2	NC_000001	213123887	213164927	0.1376763	0.0707728	0.0437431	0.0479692
ANGEL2	NC_000001	213165524	213189168	1.5219758	2.1576071	2.5650156	1.5678041
RPS6KC1	NC_000001	213224588	213446808	6.6693093	7.5575196	6.9967011	5.2027949
RPL31P13	NC_000001	213602060	213602407	0	0	0	0
PROX1	NC_000001	214161860	214209766	0.0714605	0.0293876	0.0254293	0.0398372
SMYD2	NC_000001	214454565	214510477	8.2158416	11.208701	6.9609909	8.978451
PTPN14	NC_000001	214531011	214724642	4.0519646	4.740445	3.7194798	5.1678078
LOC643454	NC_000001	214655562	214656832	0	0	0	0
KRT18P12	NC_000001	214705488	214706867	0	0	0	0
CENPF	NC_000001	214776532	214837914	1.0287028	1.7948729	1.9898139	5.0803508

LOC391162	NC_000001	215043995	215045264	0	0	0	0
KCNK2	NC_000001	215178885	215410436	2.0521472	5.8316458	0.3897449	0.5543328
LOC643536	NC_000001	215549848	215550723	0	0	0	0
KCTD3	NC_000001	215740735	215795149	6.3725495	8.5746166	11.358285	11.880677
USH2A	NC_000001	215796236	216596738	0.0370063	0.0313323	0.0542242	0.1137682
MRPS18BP1	NC_000001	216374977	216375168	0	0	0	0
ESRRG	NC_000001	216676588	217311097	0.0379715	0.0156155	0.0405366	0.015876
GPATCH2	NC_000001	217603834	217804409	1.486263	1.9729054	2.5607567	2.7662026
SPATA17	NC_000001	217804695	218040484	0.1779281	0.0731715	0.3482374	0.1983798
UBBP2	NC_000001	218023601	218024025	0	0	0	0
RRP15	NC_000001	218458629	218511325	0.6560282	0.6337654	0.6842451	0.8433566
RPS26P17	NC_000001	218511888	218512173	0	0	0	0
LOC728463	NC_000001	218517554	218519752	0	0	0	0
TGFB2	NC_000001	218519391	218617961	0.2181054	0.1811131	0.1194047	0.10522
LOC100130251	NC_000001	219054941	219065502	0	0	0	0
LYPLAL1	NC_000001	219347192	219386207	2.2763883	2.2687226	5.4141449	6.0051955
LOC728510	NC_000001	219608501	219643150	0	0	0.0756239	0.1184715
ZC3H11B	NC_000001	219781303	219785910	0	0	0	0
SLC30A10	NC_000001	220087606	220101993	0	0	0.0273984	0.021461
LOC728528	NC_000001	220109866	220132360	0	0	0	0.0949609
EPRS	NC_000001	220141942	220220000	3.2512052	4.0002852	3.9916159	5.2639372
BPNT1	NC_000001	220230824	220263191	3.0715546	4.7001019	3.8128467	4.3927599
IARS2	NC_000001	220267455	220321383	14.810393	18.065722	21.19575	22.901783
RPS15AP12	NC_000001	220317260	220317718	0	0	0	0
RAB3GAP2	NC_000001	220321610	220445843	4.7732013	5.8875902	8.5354216	6.4015982
SNORA36B	NC_000001	220373888	220374018	0	0	0	0
MORF4LP1	NC_000001	220427176	220427883	0	0	0	0
AURKAPS1	NC_000001	220439521	220441057	0	0	0	0
LOC127086	NC_000001	220487277	220489045	0	0	0	0
RPLP0P5	NC_000001	220489937	220491025	0	0	0	0
LOC100129664	NC_000001	220641296	220642187	0	0	0	0
MARK1	NC_000001	220701568	220837799	0.1249476	0.171279	0.7262247	0.0638502
C1orf115	NC_000001	220863628	220872499	0.0887246	0.015203	0.1052424	0.0515223
MOSC2	NC_000001	220921676	220957599	0.5577187	0.4300451	1.1659807	0.4080726
MOSC1	NC_000001	220960039	220987741	0.039187	0.3021627	0.2614639	0.0409606
LOC100129376	NC_000001	221006105	221055769	0.7568582	1.1931323	0.6284339	0.4219271
HLX	NC_000001	221052743	221058401	0.6363719	2.2205094	0.3602672	0.2284436
LOC100287901	NC_000001	221057440	221070396	0.3580304	1.7484412	0.4379564	0.9979594
LOC100132626	NC_000001	221307207	221307563	0	0	0	0
LOC100287182	NC_000001	221456609	221458526	0	0	0.0815382	0.0159671
LOC400804	NC_000001	221503270	221509638	0	0	0	0
LOC100132179	NC_000001	221705548	221743664	0	0	0	0
DUSP10	NC_000001	221874766	221915461	4.2902815	1.2283409	1.2199117	2.2233075
LOC100129950	NC_000001	222121576	222123751	0	0	0	0
LOC100287974	NC_000001	222376127	222376975	0	0	0	0
LOC728615	NC_000001	222641436	222643451	0	0	0	0
LOC100130483	NC_000001	222643929	222644334	0	0	0	0
LOC653056	NC_000001	222645263	222649181	0	0	0	0
LOC728417	NC_000001	222647578	222650466	0.046148	0.0948899	0.492654	0.1929464
HHIPL2	NC_000001	222695602	222721444	0.1365912	0.1930915	0.5771979	0.1903644
TAF1A	NC_000001	222731665	222763255	1.1226797	1.2023258	1.4773428	1.2875814
MIA3	NC_000001	222791444	222841351	1.6176931	1.3627888	2.0504202	2.4769845
AIDA	NC_000001	222841355	222885864	14.678651	22.306111	18.625321	13.142413
LOC100288071	NC_000001	222847489	222855691	0	0	0	0
C1orf58	NC_000001	222885906	222906109	7.7509649	8.0492996	12.0961	13.166515
FAM177B	NC_000001	222910558	222924002	9.0837237	13.941347	7.9357884	6.7397502
DISP1	NC_000001	223101783	223179335	1.3713553	1.1431601	1.8135072	1.8854026
TLR5	NC_000001	223283584	223316624	0.0384275	0.0395075	0.1253492	0.0446297
SUSD4	NC_000001	223394161	223537544	0.13657	0.3229392	0.0607482	0.0285502
C1orf65	NC_000001	223566715	223568812	0.0418954	0	0	0.0583887
CAPN8	NC_000001	223714972	223853436	0	0.021183	0	0.0861459
CAPN2	NC_000001	223900119	223963720	48.383374	47.463298	42.168877	42.837953
TP53BP2	NC_000001	223967595	224033674	3.2210961	3.0851895	4.1636581	5.8704611
LOC644214	NC_000001	224044281	224045089	0	0	0	0
ACTBP11	NC_000001	224051228	224052637	0	0	0	0
LOC100288102	NC_000001	224124531	224222721	0.088964	0.0914644	0.7914487	1.3018673

LOC730978	NC_000001	224133977	224137163	0	0	0	0
LOC100133049	NC_000001	224137784	224138178	0	0	0	0
LOC441124	NC_000001	224138275	224140026	0	0	0	0
LOC728531	NC_000001	224180449	224216684	0.0632349	0.1300242	0.1687665	0.308452
LOC100287497	NC_000001	224196244	224197161	0	0	0	0
FBXO28	NC_000001	224301791	224349749	2.7341623	3.8806812	5.9625761	4.9289811
LOC724084	NC_000001	224362849	224363612	0	0	0	0
DEGS1	NC_000001	224370928	224381143	17.721061	24.40257	25.281564	15.88123
NVL	NC_000001	224415036	224517872	0.9678895	1.6636697	1.8701176	2.0233918
CNIH4	NC_000001	224544595	224563691	13.631762	14.379855	28.612596	29.882757
WDR26	NC_000001	224572845	224621732	7.334689	5.8232735	8.2363643	7.9774028
AKR1B1P1	NC_000001	224762103	224763290	0	0	0	0
CNIH3	NC_000001	224804179	224928249	2.1862948	6.9718065	0.2307613	0.064555
LOC100288177	NC_000001	224848854	224931843	0	0	0	0
DNAH14	NC_000001	225117356	225586996	0.1975953	0.2500292	0.2974844	0.3601182
LBR	NC_000001	225589204	225616519	2.3189897	1.8170579	4.166134	4.3693794
ENAH	NC_000001	225674534	225840845	6.8731671	4.5656778	4.4286037	5.605571
SRP9	NC_000001	225965515	225978168	28.484965	30.261723	59.491049	63.518266
LEFTY3	NC_000001	225990758	225991254	0	0	0	0
EPHX1	NC_000001	225997797	226033264	11.81627	4.0731369	9.2621067	11.283695
TMEM63A	NC_000001	226033240	226070048	4.563108	2.30686	3.9922895	5.0289749
LEFTY1	NC_000001	226073982	226076836	0	0	0	0
LOC100288204	NC_000001	226074038	226075329	0	0	0	0.042125
PYCR2	NC_000001	226107580	226111965	8.0731837	8.2467048	15.472752	10.039461
LEFTY2	NC_000001	226124303	226128920	0.0217673	0	0	0.045505
LOC644482	NC_000001	226151881	226152134	0	0	0	0
C1orf55	NC_000001	226170403	226187066	2.4226888	2.1752049	3.3450898	2.3069876
H3F3A	NC_000001	226250421	226259703	13.909259	17.444497	22.362764	18.304861
LOC100133328	NC_000001	226314879	226315047	0	0	0	0
ACBD3	NC_000001	226332380	226374423	3.4394268	3.3206315	3.344953	3.1355065
RPL34P7	NC_000001	226376569	226376922	0	0	0	0
MIXL1	NC_000001	226411383	226413513	0	0.1292801	0.1118671	0.0438124
LIN9	NC_000001	226418861	226497198	1.7827558	2.8782696	4.0295868	2.8342736
LOC100128832	NC_000001	226497366	226513512	0	0	0	0
PARP1	NC_000001	226548392	226595801	4.8194171	6.5649159	8.0549815	12.488188
RPS3AP7	NC_000001	226626233	226627095	0	0	0	0
LOC391169	NC_000001	226680889	226681257	0	0	0	0
C1orf95	NC_000001	226736501	226793303	0.0107374	0.0441568	0.0191046	0.0598581
ITPKB	NC_000001	226819391	226926876	0.7916673	0.8139172	1.3324389	1.0635709
RPS27P5	NC_000001	226969202	226969446	0	0	0	0
PSEN2	NC_000001	227058273	227083804	2.3714451	2.7330256	3.8110769	3.345015
CABC1	NC_000001	227127938	227175246	1.9990133	1.931575	4.9473664	5.215865
CDC42BPA	NC_000001	227177566	227505826	2.1585082	2.1245574	3.5986432	4.1859247
LOC100126822	NC_000001	227451027	227452891	0	0	0	0
LOC100271842	NC_000001	227466151	227467218	0	0	0	0
LOC100130466	NC_000001	227597874	227611483	0	0	0	0
RPS18P3	NC_000001	227618183	227618720	0	0	0	0
LOC503543	NC_000001	227621767	227622307	0	0	0	0
LOC391171	NC_000001	227680721	227682843	0	0	0	0
LOC255208	NC_000001	227693883	227696002	0	0	0	0
LOC100288382	NC_000001	227729228	227729929	0.2504173	0	0.1113891	0.3490015
ZNF678	NC_000001	227751244	227844128	0.941001	0.9853636	0.6666119	0.8864457
MRP63P2	NC_000001	227759941	227760148	0	0	0	0
ZNF847P	NC_000001	227884733	227885408	0	0	0	0
LOC100130093	NC_000001	227916240	227922078	0	0	0	0
JMJD4	NC_000001	227918935	227923112	0.9823722	2.1085588	1.2419227	1.1649464
SNAP47	NC_000001	227922697	227968932	4.4375274	6.6811114	5.0709559	5.7575289
MPN2	NC_000001	228003418	228034171	0	0.0727006	0.0314542	0.0246379
WNT9A	NC_000001	228109165	228135676	0.0354421	0.0728765	0.0315303	0.0987899
LOC728728	NC_000001	228155043	228158163	0	0	0	0
LOC100132885	NC_000001	228156662	228159482	0	0	0	0
LOC729033	NC_000001	228159910	228161829	0	0	0	0
LOC100131767	NC_000001	228162980	228163386	0	0	0	0
LOC648262	NC_000001	228163703	228165815	0	0	0	0.0461914
WNT3A	NC_000001	228194752	228248961	0.0299783	0.0154104	0	0.010445
ARF1	NC_000001	228270361	228286913	111.6501	105.29089	118.59121	131.45847

C1orf35	NC_000001	228288428	228291022	0.6472995	0.6304661	0.8789375	0.8546478
MRPL55	NC_000001	228294380	228297013	9.7501044	11.86937	9.6233522	9.5998515
LOC391174	NC_000001	228302592	228303271	0	0	0	0
RPS28P3	NC_000001	228322483	228324494	0	0	0	0
GUK1	NC_000001	228327982	228336652	72.926847	59.339222	65.198217	62.225959
GJC2	NC_000001	228337553	228347527	0.4310571	0.6043255	0.0522928	0.3140313
C1orf69	NC_000001	228353429	228369958	0.2835729	0.4458888	0.1088242	0.3370906
OBSCN	NC_000001	228395861	228566575	0.0580806	0.0632255	0.1018205	0.1011822
TRIM11	NC_000001	228581377	228594517	3.0866651	3.2069973	3.0220341	2.0712478
TRIM17	NC_000001	228595636	228604583	0.2501797	0.2057688	0.1780534	0.3951597
HIST3H3	NC_000001	228612546	228613026	0.2741054	0.0939364	0	0.4456843
RPL23AP15	NC_000001	228636869	228637322	0	0	0	0
HIST3H2A	NC_000001	228645065	228645560	0.5316318	0.2732867	2.7589008	0.1234874
HIST3H2BB	NC_000001	228645808	228646259	7.2922951	3.1988249	12.542361	3.1844453
HIST3H2BA	NC_000001	228651917	228652295	0	0	0	0
RNF187	NC_000001	228675094	228683467	14.698515	15.380868	18.070793	14.223174
LOC100129094	NC_000001	228695568	228699658	0	0.0296674	0	0
RN5S1	NC_000001	228746013	228746133	0	0	0	0
RN5S2	NC_000001	228748254	228748374	0	0	0	0
RN5S3	NC_000001	228750495	228750615	0	0	0	0
RN5S4	NC_000001	228752736	228752856	0	0	0	0
RN5S5	NC_000001	228754977	228755097	0	0	0	0
RN5S6	NC_000001	228757192	228757312	0	0	0	0
RN5S7	NC_000001	228759412	228759532	0	0	0	0
RN5S8	NC_000001	228761654	228761774	0	0	0	0
RN5S9	NC_000001	228763893	228764013	0	0	0	0
RN5S10	NC_000001	228766135	228766255	0	0	0	0
RN5S11	NC_000001	228768376	228768496	0	0	0	0
RN5S12	NC_000001	228770616	228770736	0	0	0	0
RN5S13	NC_000001	228772841	228772961	0	0	0	0
RN5S14	NC_000001	228775082	228775202	0	0	0	0
RN5S15	NC_000001	228777313	228777433	0	0	0	0
RN5S16	NC_000001	228779554	228779674	0	0	0	0
DUSP5P	NC_000001	228780657	228788159	0	0	0	0
RN5S17	NC_000001	228781785	228781905	0	0	0	0
FTHL2	NC_000001	228823167	228823626	0	0	0	0
RHOU	NC_000001	228870869	228882411	0.7423001	0.4599884	0.5156319	0.5881224
LOC100288517	NC_000001	229168511	229178339	0	0	0	0
RAB4A	NC_000001	229406879	229440518	3.1953679	3.9471122	2.6517611	3.7886447
SPHAR	NC_000001	229440129	229441251	0.3130773	0.4828146	0.3481529	0.3545177
C1orf96	NC_000001	229456752	229478688	0.5149178	0.6705602	1.3055426	0.9329195
ACTA1	NC_000001	229566992	229569843	0.3532343	0.302635	0.1833108	0.3487093
LOC100288545	NC_000001	229566992	229568732	0.0591497	0.0608121	0.1052424	0.1236536
LOC731139	NC_000001	229576007	229576816	0.2712854	0.055782	0.1930744	0.3024679
NUP133	NC_000001	229577044	229644088	6.1759386	9.1233632	6.3099908	6.6757828
ABCB10	NC_000001	229652329	229694442	3.2246175	3.5612534	5.2103056	5.1054697
LOC100288582	NC_000001	229700407	229706550	0	0	0	0
TAF5L	NC_000001	229728858	229761794	2.0240734	2.4646834	3.3586999	3.3060664
URB2	NC_000001	229761981	229795946	2.8421892	3.8316942	2.834974	4.1793438
LOC100131918	NC_000001	229824744	229826597	0	0	0	0
LOC645201	NC_000001	229829397	229841610	0	0	0	0
LOC100129238	NC_000001	230118954	230141982	0.0488857	0	0	0.0510982
GALNT2	NC_000001	230202956	230417875	20.411928	20.063498	37.256236	40.878958
PGBD5	NC_000001	230457392	230513367	0.0127608	0.0262389	0.0227047	0.035569
RPS24P4	NC_000001	230747689	230748194	0	0	0	0
COG2	NC_000001	230778202	230829731	1.2323322	1.2059084	1.8095833	2.131326
AGT	NC_000001	230838269	230850336	0.6462975	0.856806	0.5749642	0.355552
CAPN9	NC_000001	230883130	230937749	0.0677952	0.0348503	0.0150781	0.0118106
C1orf198	NC_000001	230972865	231005335	15.762652	8.6495815	4.4850547	3.3204127
TTC13	NC_000001	231041989	231114607	1.2610299	1.5171472	3.6161668	2.0940088
ARV1	NC_000001	231114823	231136479	4.8358262	6.0974141	9.36177	8.0747949
FAM89A	NC_000001	231154704	231175995	1.4962349	2.051049	0.1826989	0.0817754
LOC644006	NC_000001	231157357	231158667	0	0	0.0298227	0
LOC100288703	NC_000001	231253577	231254071	0	0	0	0
TRIM67	NC_000001	231298674	231357314	0.0309422	0.0212079	0.027527	0.0251554
C1orf131	NC_000001	231359509	231376924	0.7962746	2.0151482	1.2260561	1.408531

GNPAT	NC_000001	231376919	231413719	2.3777961	2.2760296	3.1219698	3.0967694
EXOC8	NC_000001	231468482	231473578	1.1985098	1.0548999	1.388397	1.9287004
LOC100288727	NC_000001	231473636	231474338	0.1364852	0	0.0607105	0.0475542
C1orf124	NC_000001	231473682	231489989	0.9160653	1.0830831	1.1816878	1.3086191
EGLN1	NC_000001	231501431	231560790	1.9407703	2.4503879	2.2945114	2.4438214
LOC645339	NC_000001	231611508	231612274	0	0	0	0
TSNAX	NC_000001	231664399	231702270	2.6368939	3.2225143	5.4441516	4.8653109
LOC100287814	NC_000001	231727038	231747836	0	0	0	0
DISC1	NC_000001	231762561	232177018	0.2552511	0.1029117	0.1068604	0.0976536
DISC2	NC_000001	231950372	231954263	0	0	0	0
SIPA1L2	NC_000001	232533711	232651243	0.5215001	0.0835569	0.2530587	0.3775605
KIAA1383	NC_000001	232940638	232946092	0.3786557	0	0.2006841	0.0561409
LOC645395	NC_000001	233052925	233053770	0	0	0	0
C1orf57	NC_000001	233086370	233114219	4.4825248	4.758784	9.3192645	8.3182873
PCNXL2	NC_000001	233119882	233431459	1.5730334	2.1607436	1.8066207	2.196797
LOC100288792	NC_000001	233145533	233190435	0	0	0.0282497	0
RPS7P3	NC_000001	233424555	233425237	0	0	0	0
KIAA1804	NC_000001	233463514	233520894	0.1361798	0.3500177	1.292259	0.4164857
KCNK1	NC_000001	233749750	233808258	0.7117946	1.2963308	0.5608628	0.2692608
LOC100288820	NC_000001	233971823	233980827	0	0	0	0.151234
SLC35F3	NC_000001	234040679	234460262	0.0912104	0.031258	0.0270478	0.0423727
RPL9P10	NC_000001	234420697	234421402	0	0	0	0
RPS15P2	NC_000001	234492419	234492916	0	0	0	0
C1orf31	NC_000001	234509429	234519791	5.4849587	4.793247	6.7704053	10.606432
TARBP1	NC_000001	234527059	234614849	1.2336346	1.435652	1.8062618	3.3908247
IRF2BP2	NC_000001	234740015	234745271	9.4720079	12.848636	9.072178	7.4476972
PP2672	NC_000001	234895051	234895599	0	0	0	0
TOMM20	NC_000001	235272656	235292256	7.5517777	8.0308261	14.244581	13.54475
SNORA14B	NC_000001	235291118	235291252	0	0	0	0
RBM34	NC_000001	235294949	235324571	9.8428993	7.2512592	9.5931259	11.205822
ARID4B	NC_000001	235330210	235491532	0.7316254	0.6851612	0.8442032	0.9742214
RPL23AP23	NC_000001	235459182	235459652	0	0	0	0
GGPS1	NC_000001	235491753	235507847	1.8927906	1.5355059	3.6440194	2.5761169
TBCE	NC_000001	235530728	235612280	4.4217577	4.9614052	4.4928255	5.677646
RPS21P1	NC_000001	235596300	235596539	0	0	0	0
B3GALNT2	NC_000001	235613244	235667781	0.2406324	0.9446007	0.7784483	1.249995
LOC100288858	NC_000001	235701345	235703136	0	0	0	0
GNG4	NC_000001	235710985	235814054	0.0579137	0.1020709	0.0441614	0.0288261
LOC100131725	NC_000001	235777982	235779699	0	0	0	0
LYST	NC_000001	235824343	236030220	0.9586571	0.7140573	2.3728898	3.2151805
LOC100190799	NC_000001	235901195	235902909	0	0	0	0
NID1	NC_000001	236139130	236228481	5.3562608	8.5328505	6.0211447	3.0401035
LOC100288925	NC_000001	236208861	236266259	0.1523864	0.2193369	0.0813403	0.0637133
LOC343508	NC_000001	236273361	236275672	0	0	0	0
GPR137B	NC_000001	236305832	236372209	0.710231	0.9735895	1.1105087	1.6797191
ERO1LB	NC_000001	236380460	236445285	0.3115844	0.3966133	1.2011744	0.6100161
LOC100130485	NC_000001	236448774	236450303	0	0	0	0
EDARADD	NC_000001	236557680	236648008	0.2767704	0.1797152	0.1166318	0.13196
ENO1P	NC_000001	236646405	236648203	0	0	0	0
LGALS8	NC_000001	236681565	236712996	11.555469	7.4938996	11.12484	13.085006
LOC100287902	NC_000001	236686369	236687838	0	0	0	0.0208333
HEATR1	NC_000001	236713971	236767814	3.3781442	4.6108228	5.3312245	6.9042392
ACTN2	NC_000001	236849770	236927558	0.165	0.209552	0.0518077	0.0405807
MTR	NC_000001	236958581	237067281	0.8949488	1.0998422	1.2331397	1.8796099
RPSAP21	NC_000001	236982852	236983890	0	0	0	0
RPL35P1	NC_000001	237144609	237145043	0	0	0	0
MT1P2	NC_000001	237167347	237167743	0	0	0	0
RYR2	NC_000001	237205702	237997288	0.4457932	0.3561661	0.3559754	0.2264351
LOC100130331	NC_000001	238025475	238091621	0	0	0	0
ZP4	NC_000001	238045710	238053935	0.0268797	0.0552702	0.0717387	0.0936541
TRNAP23P	NC_000001	238105862	238105933	0	0	0	0
LOC100288950	NC_000001	238106118	238106953	0	0	0	0
TRNAL45P	NC_000001	238106956	238107030	0	0	0	0
LOC100288989	NC_000001	238111331	238112444	0	0	0	0
LOC100289023	NC_000001	238113056	238114836	0	0	0	0
LOC339535	NC_000001	238643684	238649317	0	0	0	0

KRT18P32	NC_000001	238654583	238656007	0	0	0	0
LOC100130099	NC_000001	238940440	239056037	0	0	0	0
RPL39P10	NC_000001	239215871	239216258	0	0	0	0
CHRM3	NC_000001	239792373	240072720	0.0319856	0.0328846	0.0142276	0.0445777
LOC128136	NC_000001	240134180	240161672	0	0	0	0
RPS7P5	NC_000001	240175911	240176577	0	0	0	0
FMN2	NC_000001	240255185	240638489	3.7187491	4.1465558	2.0068746	6.9786042
PSMD2P1	NC_000001	240305810	240308690	0	0	0	0
ADH5P3	NC_000001	240333443	240334830	0	0	0	0
GREM2	NC_000001	240652873	240775462	0.6513962	3.1000804	0.1588952	0.0439276
RPS11P2	NC_000001	240799839	240800373	0	0	0	0
LOC100128958	NC_000001	240817414	240817927	0	0	0	0
LOC645939	NC_000001	240932718	240934911	0	0	0	0
RGS7	NC_000001	240938817	241520478	1.1167174	0.8518182	0.2563775	0.9287873
LOC100287266	NC_000001	241082953	241083912	0	0	0	0
RPLP1P2	NC_000001	241161709	241162167	0	0	0	0
RPL36P6	NC_000001	241468903	241469189	0	0	0	0
FH	NC_000001	241660903	241683054	15.811543	23.020817	31.540159	32.284439
KMO	NC_000001	241695680	241758944	0.1752671	0.0360386	0.1403302	0.0793865
OPN3	NC_000001	241756452	241803701	0.2003719	0.5836762	0.5644785	0.4072457
CHML	NC_000001	241792166	241799232	0	0.0063936	0	0.0130005
WDR64	NC_000001	241846882	241964957	0.0175934	0	0.0313031	0.0122598
RPL6P3	NC_000001	241995211	241996129	0	0	0	0
EXO1	NC_000001	242011535	242053048	1.5253182	3.822457	3.6347288	3.2266683
RPL23AP20	NC_000001	242079366	242079889	0	0	0	0
BECN1L1	NC_000001	242121069	242122364	0.0339107	0.0348637	0.0301679	0.0236303
CFLP4	NC_000001	242156508	242157185	0	0	0	0
MAP1LC3C	NC_000001	242158792	242162385	0.2212159	0.1137166	0.0328	0.025692
LOC200149	NC_000001	242220387	242223577	0	0	0	0
PLD5	NC_000001	242252272	242687998	0.0805208	0.0165568	0.143267	0.1234422
RPL10AP5	NC_000001	242528462	242529150	0	0	0	0
LOC391183	NC_000001	242767884	242936446	0	0	0	0
LOC100129949	NC_000001	243045221	243049882	0	0	0	0
LOC100289254	NC_000001	243138218	243138690	0	0	0	0
LOC400723	NC_000001	243202679	243215538	0	0	0	0
LOC100287300	NC_000001	243215558	243215959	0	0	0	0
LOC729660	NC_000001	243217236	243217820	0.1808569	0.09297	0	0.1260283
LOC731275	NC_000001	243218160	243220543	0.0720463	0	0.2243303	0.351433
CEP170	NC_000001	243287730	243418708	4.2218082	8.0796491	6.9968058	6.9748648
LOC100287359	NC_000001	243319595	243343100	0.4367033	0.3265286	0.6004143	0.0829943
SDCCAG8	NC_000001	243419320	243663393	3.8142475	4.8584303	2.6125101	3.3047584
AKT3	NC_000001	243651535	244006553	4.5019584	5.9877382	9.0646327	8.5457389
LOC100289357	NC_000001	244197661	244211423	0	0.0735886	0.0636768	0.0498777
ZNF238	NC_000001	244214561	244220778	0.0902428	0.1133966	0.3478907	0.3074366
LOC440742	NC_000001	244227632	244228404	0	0	0	0
C1orf100	NC_000001	244515937	244552393	0	0	0	0
TGIF2P1	NC_000001	244558277	244570468	0	0	0	0
ADSS	NC_000001	244571796	244615413	5.1488135	6.7029421	10.848617	11.230233
C1orf101	NC_000001	244624673	244803662	0.0550385	0.0282927	0.0367228	0.076706
CYCSP5	NC_000001	244761692	244761989	0	0	0	0
PPPDE1	NC_000001	244816352	244872335	6.6035091	9.2177231	12.379781	10.003778
RPL37P8	NC_000001	244857732	244858022	0	0	0	0
FAM36A	NC_000001	244998639	245008359	6.7367129	4.2488979	5.0515968	8.4639859
C1orf199	NC_000001	245003940	245010243	0	0	0	0
HNRNPU	NC_000001	245013602	245027827	5.0457654	6.84417	6.7161462	13.581234
LOC100129656	NC_000001	245025965	245027510	0	0.0682529	0	0.0462611
EFCAB2	NC_000001	245133171	245288530	0.2801712	0.2085846	0.2750323	0.4510589
LOC100128825	NC_000001	245286765	245287761	0	0	0	0
KIF26B	NC_000001	245318287	245866428	0.0723726	0.4030357	0.0375577	0.0588374
SMYD3	NC_000001	245912644	246518396	5.3954302	11.497562	6.6617132	6.4257557
LOC100132379	NC_000001	246672137	246681066	0	0	0	0
TFB2M	NC_000001	246703863	246729565	3.4203831	2.656359	5.1444165	4.4068274
C1orf71	NC_000001	246729639	246831884	3.7281749	4.2552305	3.7453275	3.7923331
SCCPDH	NC_000001	246887378	246931439	1.4388871	1.954173	2.3863106	2.6614183
RPL35AP6	NC_000001	246918813	246919134	0	0	0	0
LOC100130097	NC_000001	246935180	246942272	0.0785491	0.0807568	0.1048192	0.3010488

LOC100131773	NC_000001	246943593	246984074	0.1426891	0.0488998	0.0846268	0.0331438
LOC149134	NC_000001	246952919	246954788	0	0	0	0
AHCTF1	NC_000001	247002400	247081679	2.4358344	4.7220793	5.4312377	4.7936781
ZNF695	NC_000001	247148625	247171355	0.1053599	0.1354013	0.246044	0.17437
ZNF670	NC_000001	247200087	247242069	1.7535454	1.4647986	1.170002	0.7942612
ZNF669	NC_000001	247263264	247267674	0.2219608	0.3194786	0.2961937	0.1237369
C1orf229	NC_000001	247273462	247275719	0.0194633	0.0400207	0.1038908	0
ZNF124	NC_000001	247319203	247335318	1.1551507	1.2146076	1.5181253	1.7928543
LOC100131465	NC_000001	247347115	247349984	0	0	0	0
LOC729806	NC_000001	247353153	247373952	1.4969062	0.841628	2.9546856	2.0536108
LOC343165	NC_000001	247393242	247395183	0	0	0	0
LOC441931	NC_000001	247400526	247401152	0	0	0	0
VN1R5	NC_000001	247419374	247420447	0	0	0	0
ZNF496	NC_000001	247463622	247495045	2.3892778	4.1126573	3.1561462	2.383897
LOC729810	NC_000001	247493000	247497878	0	0	0	0
NLRP3	NC_000001	247579458	247612410	0.0195369	0.020086	0.0086903	0.0408422
OR2B11	NC_000001	247614331	247615284	0	0.0473621	0	0
OR2W5	NC_000001	247654430	247655392	0	0	0	0
LOC644852	NC_000001	247681165	247683938	0	0	0	0
LOC148824	NC_000001	247687981	247694106	0	0	0	0
OR2C3	NC_000001	247693434	247697141	0.0480834	0.0164783	0.0570351	0.0446752
C1orf150	NC_000001	247712451	247739859	0.0167358	0.0688247	0	0.0116622
OR2G2	NC_000001	247751662	247752615	0	0	0	0.0321015
OR2G3	NC_000001	247768888	247769817	0.0472562	0	0	0
OR14L1P	NC_000001	247782983	247783861	0	0	0	0
OR3D1P	NC_000001	247830083	247831218	0	0	0	0
OR13G1	NC_000001	247835420	247836343	0.047563	0.0488998	0	0
OR6F1	NC_000001	247875131	247876057	0	0	0	0.1321462
OR14A2	NC_000001	247886201	247887545	0	0	0	0
OR14K1	NC_000001	247901917	247902861	0	0	0	0
OR1C1	NC_000001	247920764	247921708	0.0465061	0.0478131	0	0
OR9H1P	NC_000001	247938112	247939235	0	0	0	0
OR14A16	NC_000001	247978102	247979031	0.0472562	0	0	0.0987899
HSD17B7P1	NC_000001	247990766	247992040	0	0	0	0
OR6R1P	NC_000001	247996619	247997547	0	0	0	0
OR11L1	NC_000001	248004230	248005198	0	0	0.0403484	0
TRIM58	NC_000001	248020501	248043440	0.3322957	3.4075889	0.538179	1.6090233
OR2W3	NC_000001	248058889	248059833	0.1395182	1.9603381	0.2068654	0.6481456
OR2T8	NC_000001	248084320	248085258	0	0	0	0.0652287
OR2AJ1	NC_000001	248097179	248098121	0	0	0	0
LOC100128834	NC_000001	248099116	248100430	0	0	0	0
LOC100289622	NC_000001	248099312	248100816	0	0	0	0
OR2L13	NC_000001	248100493	248264224	0.0466542	0	0.0415048	0.0325105
OR2X1P	NC_000001	248102273	248103217	0	0	0	0
OR2L8	NC_000001	248112160	248113098	0	0	0	0
OR2AK2	NC_000001	248128634	248129641	0	0	0.0387873	0
OR2L9P	NC_000001	248137944	248139077	0	0	0	0
OR2L1P	NC_000001	248153569	248154493	0	0	0	0
OR2L6P	NC_000001	248166333	248167468	0	0	0	0
OR2L5	NC_000001	248185150	248186285	0	0	0	0
OR2L2	NC_000001	248201474	248202607	0	0	0	0
OR2L3	NC_000001	248223984	248224922	0	0	0	0
OR2T32P	NC_000001	248246938	248247898	0	0	0	0
OR2M1P	NC_000001	248285438	248286082	0	0	0	0
OR2M5	NC_000001	248308450	248309388	0	0	0	0
LOC100216488	NC_000001	248328901	248336001	0	0	0	0
OR2M2	NC_000001	248343288	248344331	0	0	0	0
OR2M3	NC_000001	248366370	248367308	0	0.0481186	0	0
OR2M4	NC_000001	248402231	248403166	0	0	0	0
OR2T33	NC_000001	248436154	248437116	0	0.0469194	0	0
OR2T12	NC_000001	248457918	248458880	0.0456368	0	0	0.0318015
OR2M7	NC_000001	248486932	248487870	0	0	0	0
OR14C36	NC_000001	248512077	248513015	0.0468032	0.0962373	0.0416375	0.1630718
OR2T4	NC_000001	248524883	248525929	0	0.0431551	0	0.0292501
OR2T6	NC_000001	248550910	248551836	0	0.0487415	0	0
OR2T1	NC_000001	248569296	248570405	0.039593	0	0.035223	0.0827699

OR2T7	NC_000001	248604408	248605531	0	0	0	0
OR2T2	NC_000001	248616099	248617073	0	0	0	0.0314101
OR2T3	NC_000001	248636652	248637608	0	0	0	0.0960028
OR2T5	NC_000001	248651890	248652837	0.0463589	0	0	0
OR2AS2P	NC_000001	248661511	248662060	0	0	0	0
OR2G6	NC_000001	248684948	248685898	0	0	0.0411121	0.0322028
OR2AS1P	NC_000001	248712723	248713204	0	0	0	0
OR2T29	NC_000001	248721845	248722774	0	0	0	0
OR2T34	NC_000001	248737102	248738058	0	0	0	0.0320009
OR2T10	NC_000001	248756131	248757069	0	0	0	0.0326144
OR2T11	NC_000001	248789479	248790429	0	0	0	0
OR2T35	NC_000001	248801588	248802559	0	0	0	0.0630142
OR2T27	NC_000001	248813232	248814185	0	0.0473621	0	0
OR14I1	NC_000001	248844670	248845605	0	0	0	0.0654378
LOC100131533	NC_000001	249065433	249068092	0	0	0	0
SH3BP5L	NC_000001	249104651	249120154	1.9198002	4.07531	2.6171532	4.6389665
ZNF672	NC_000001	249132530	249143714	1.751343	2.6315946	4.0349327	3.9532796
LOC100130262	NC_000001	249141783	249143716	0.0242273	0.0498163	0.1077662	0.0168825
ZNF692	NC_000001	249144205	249153271	0.8673993	1.0106814	2.3835797	2.9684641
PGBD2	NC_000001	249200442	249213345	0.1784535	0.4836909	0.2886494	0.5087189
RPL23AP25	NC_000001	249230903	249231268	0	0	0	0
FAM110C	NC_000002	41608	46385	0	0.0467737	0	0.0634055
LOC727818	NC_000002	194146	200893	0	0	0	0
SH3YL1	NC_000002	218155	256341	0.5175675	0.7634676	1.1210772	1.5210513
ACP1	NC_000002	264869	278283	2.5440028	3.6952968	4.111162	4.8628989
FAM150B	NC_000002	279561	288308	0.062873	0.03232	0.0279668	0.1752497
LOC727944	NC_000002	490940	492757	0	0	0	0.0562957
TMEM18	NC_000002	667973	677439	3.0623062	2.8718264	2.0248268	3.3739273
LOC100128185	NC_000002	687886	688026	0	0	0	0
LOC391343	NC_000002	902823	906011	0.0137812	0	0.0245203	0.0384131
SNTG2	NC_000002	946555	1371385	0	0	0.0414389	0.0162294
TPO	NC_000002	1417235	1546499	0	0.0287426	0.0248712	0.0389629
PXDN	NC_000002	1635659	1748291	7.0744991	7.7568925	10.254441	13.388563
LOC100288793	NC_000002	1652690	1657738	0.1192086	0	0.0353504	0.0276898
MYT1L	NC_000002	1792885	2335045	0.0492693	0.0443223	0.1534097	0.0386244
LOC730811	NC_000002	2323004	2330880	0	0	0	0
LOC729897	NC_000002	3135757	3149551	0	0	0	0
LOC100132738	NC_000002	3153392	3185045	0.0255662	0.0788541	0.0682331	0.1781552
TSSC1	NC_000002	3192741	3381653	8.043982	6.9836054	5.1115353	8.9507924
TTC15	NC_000002	3383446	3483342	2.6118204	2.8463395	1.6264835	2.814965
LOC100288893	NC_000002	3487251	3488865	0.3265503	0.1958414	0.2420902	0.2654788
ADI1	NC_000002	3501690	3523350	16.38881	22.05252	13.856774	31.977051
RNASEH1	NC_000002	3592678	3605940	4.1334805	4.5238236	3.5349134	3.5307809
RPS7	NC_000002	3622853	3628509	117.11479	217.47615	246.72282	191.32136
COLEC11	NC_000002	3642637	3692048	0	0.0653411	0	0.0885752
TMSL2	NC_000002	3664712	3665449	0	0	0	0
ALLC	NC_000002	3705786	3750261	0.060828	0	0	0.0635811
LOC100240713	NC_000002	3735375	3736649	0	0	0	0
LOC728597	NC_000002	3750316	3818443	0.3726856	0.0589477	0.0510079	0.3795647
LOC442006	NC_000002	4561786	4562646	0	0	0	0
SOX11	NC_000002	5832799	5841517	0.0302431	0.0051822	0.0089684	0.0140497
LOC150622	NC_000002	6072819	6120350	0	0	0	0
LOC400940	NC_000002	6122110	6128364	0	0	0	0
LOC391349	NC_000002	6636009	6651631	0	0	0	0
FLJ42418	NC_000002	6872257	6910442	0	0	0	0
CMPK2	NC_000002	6988451	7005936	25.583782	6.8780354	15.42189	1.3626995
RSAD2	NC_000002	7017796	7038363	11.224819	2.1485274	5.2991006	0.3052024
RNF144A	NC_000002	7057523	7184310	0.4057938	1.5822062	1.1034505	0.9176793
LOC644232	NC_000002	7464879	7490157	0	0	0	0
LOC100130731	NC_000002	7850216	7877226	0	0	0	0
LOC339788	NC_000002	8062556	8116945	0	0	0	0
LOC100288145	NC_000002	8743212	8743489	0	0	0	0
ID2	NC_000002	8822113	8824583	15.981175	44.520889	6.6786897	7.8582899
KIDINS220	NC_000002	8868987	8977755	1.779184	1.9151231	1.6624831	4.0522526
LOC100216337	NC_000002	8897038	8897979	0	0	0	0
MBOAT2	NC_000002	8996701	9143876	5.0140727	6.73475	4.7998326	7.0846196

LOC644651	NC_000002	9158417	9159028	0	0	0	0
RPL30P3	NC_000002	9221522	9221924	0	0	0	0
ASAP2	NC_000002	9346894	9545812	10.823703	11.436135	7.4338034	8.9512282
ITGB1BP1	NC_000002	9545813	9563643	4.8957875	6.4146384	6.158373	7.1246478
CPSF3	NC_000002	9563868	9613227	2.7126039	3.4340218	2.863433	4.2883293
IAH1	NC_000002	9614670	9628591	14.455379	17.2056	21.922255	18.557459
ADAM17	NC_000002	9629392	9695917	7.7512279	7.5769479	15.050155	12.748934
LOC100127913	NC_000002	9695937	9697482	0.2274165	0.175356	0.1264475	0.1584729
YWHAQ	NC_000002	9724106	9771106	109.40391	185.15599	127.96059	160.29282
TAF1B	NC_000002	9983571	10074545	4.6261297	5.1099106	4.7277613	4.3159898
GRHL1	NC_000002	10091822	10142405	0.3202506	0.1266351	0.7889643	0.6523236
UNQ5830	NC_000002	10143627	10143914	0	0	0	0
KLF11	NC_000002	10183682	10194963	4.0240441	5.000446	5.0739521	4.8483058
CYS1	NC_000002	10196926	10220538	0.4040845	0.382206	0.0431382	0.0563164
RRM2	NC_000002	10262735	10271546	12.987648	24.135978	45.490951	35.425242
C2orf48	NC_000002	10281509	10351856	0.0924253	0	0.1438922	0.1771155
HPCAL1	NC_000002	10443040	10567743	8.3112431	2.4592442	11.920429	13.53258
ODC1	NC_000002	10580508	10588453	18.414778	30.830769	29.370577	31.991265
NOL10	NC_000002	10710894	10830112	0.8047745	1.6677135	0.7271364	1.3581849
ATP6V1C2	NC_000002	10861775	10925236	0.1763355	0.1534004	0.1086043	0.3213722
PDIA6	NC_000002	10923517	10952960	46.477898	44.756411	66.590141	49.462862
KCNF1	NC_000002	11052063	11054351	0.0383995	0	0.0512419	0.0802749
RPL6P4	NC_000002	11242249	11243152	0	0	0	0
C2orf50	NC_000002	11273179	11286916	0.0416176	0.0855746	0.1110726	0.0580017
PQLC3	NC_000002	11295540	11319000	4.8175914	4.1443389	3.7391967	3.9394418
ROCK2	NC_000002	11321778	11484711	6.6804608	6.7340986	6.2546332	6.5259075
LOC646050	NC_000002	11448151	11449245	0	0	0	0
LOC650157	NC_000002	11491753	11512199	0.2146434	0.165507	0.2386909	0.2617511
E2F6	NC_000002	11584501	11606297	2.2245401	2.3428431	2.6909744	3.6579716
GREB1	NC_000002	11674242	11782912	0.1870138	0.1048744	0.1436854	0.1184715
TRNAG32P	NC_000002	11691827	11691898	0	0	0	0
NTSR2	NC_000002	11798304	11810329	0.081993	0	0.0243144	0.0190453
LPIN1	NC_000002	11886740	11967535	10.19553	7.0330267	15.170556	16.146936
LOC729992	NC_000002	12163621	12164575	0	0	0	0
LOC100130010	NC_000002	12166490	12166667	0	0	0	0
LOC100288243	NC_000002	12856559	12858261	0.0299579	0.0923996	0.0799541	0.0417517
TRIB2	NC_000002	12856998	12882860	2.981062	1.5272974	1.3836707	0.6669665
FAM84A	NC_000002	14772856	14776447	0.7307711	0	0.0907136	0.0236851
LOC100288316	NC_000002	14775432	14780912	0.5859764	0	0.3723578	0.0583331
NBAS	NC_000002	15307032	15701454	1.3036004	2.1158391	1.9060197	3.3097747
RPS26P18	NC_000002	15537559	15537906	0	0	0	0
LOC100288342	NC_000002	15731607	15782789	0.0978802	0.1006312	0.2176925	0.1364137
DDX1	NC_000002	15731770	15771225	6.3340024	9.3338957	6.4006732	8.1598438
RPLP1P5	NC_000002	16009934	16010404	0	0	0	0
MYCNOS	NC_000002	16080020	16081845	0	0	0	0
MYCN	NC_000002	16080683	16087129	0	0.0173449	0.0150087	0.0235124
FAM49A	NC_000002	16733901	16805287	0.0914953	0	0.7325706	0.4675554
LOC388925	NC_000002	17566577	17567684	0	0	0	0
RAD51AP2	NC_000002	17691986	17699706	0.0244905	0.0125894	0.0217874	0.017066
VSNL1	NC_000002	17721807	17837706	1.2274818	0.6084548	0.2145004	0.1985653
SMC6	NC_000002	17845079	17935096	0.8356766	1.1598706	0.6839658	1.6712949
GEN1	NC_000002	17935177	17966632	1.5732441	2.0253416	2.0324649	3.6273202
MSGN1	NC_000002	17997786	17998367	0	0	0	0
KCNS3	NC_000002	18059945	18114225	2.9424567	2.3270422	6.0743857	5.6255141
RDH14	NC_000002	18735989	18741959	3.6257291	3.8970684	4.2274243	4.115218
NT5C1B	NC_000002	18744137	18770838	0.138202	0.1105115	0.0546437	0.1284062
OSR1	NC_000002	19551246	19558372	2.5292277	1.8130615	0.3096428	1.1480287
CISD1B	NC_000002	20025973	20026668	0	0	0	0
TTC32	NC_000002	20096514	20101747	0.588115	0.4947088	1.3793545	1.8255706
WDR35	NC_000002	20110024	20189884	0.6197909	0.8582831	1.1871617	1.5821458
MATN3	NC_000002	20191813	20212455	1.191009	2.2915314	1.2563291	1.0907816
LAPTM4A	NC_000002	20232411	20251789	67.17979	54.168883	86.501979	60.538357
RPS16P2	NC_000002	20355334	20355874	0	0	0	0
SDC1	NC_000002	20400558	20425194	3.0097959	7.6622905	9.0412396	6.6915314
LOC100131373	NC_000002	20426249	20426554	0	0	0	0
LOC151457	NC_000002	20439503	20440807	0	0	0	0

PUM2	NC_000002	20448453	20527144	6.5012603	8.3919426	11.023581	12.423347
LOC100289450	NC_000002	20550577	20596442	0	0	0	0
RHOB	NC_000002	20646835	20649201	10.286151	30.809468	2.7915034	1.4620242
LOC100289378	NC_000002	20647433	20649457	0.1930376	0.5623117	0.1717316	0.1120969
LOC727919	NC_000002	20729156	20729860	0	0	0	0
RPS25P3	NC_000002	20806040	20806414	0	0	0	0
HS1BP3	NC_000002	20817564	20850864	5.3037816	4.2306556	5.5319069	5.3908962
GDF7	NC_000002	20866424	20871250	0	0.0226597	0	0.030717
C2orf43	NC_000002	20884818	21022827	2.3082055	2.2623342	3.4087163	5.0290854
APOB	NC_000002	21224301	21266945	0.0155613	0.0191984	0.0498376	0.0390374
LOC100129278	NC_000002	21346484	21366144	4.1885629	5.4715124	3.4945445	2.3350059
LOC100287183	NC_000002	21584930	21595426	0	0	0	0
LOC100130841	NC_000002	22410875	22413306	0	0	0	0
KLHL29	NC_000002	23608557	23931483	0.4006305	0.4789421	1.1521225	1.9347496
LOC100288602	NC_000002	23621423	23621989	0	0	0	0
ATAD2B	NC_000002	23971534	24149934	0.5129931	0.6564582	0.5437635	0.5742402
RPS13P4	NC_000002	24123563	24124019	0	0	0	0
UBXN2A	NC_000002	24163376	24223696	2.5497392	2.7405544	2.2064554	2.5843779
LOC388931	NC_000002	24232953	24247145	0.2622905	0	0	0.0609248
C2orf44	NC_000002	24252206	24270296	0.9706081	1.2763673	0.9538441	1.9818873
FKBP1B	NC_000002	24272584	24286551	1.0579314	1.4502195	0.0784304	0.3993214
SF3B14	NC_000002	24290454	24299314	22.857641	28.188348	71.653515	33.762836
TP53I3	NC_000002	24300305	24307728	6.4020111	17.695708	1.4238516	5.0279651
LOC100288638	NC_000002	24300309	24303899	0.6522929	0.7544538	0.3626861	0.2272718
PFN4	NC_000002	24338244	24346151	0.5103666	0.349807	0.2522424	0.1185479
LOC375190	NC_000002	24346350	24392507	0.2267042	0.3995585	0.4033647	0.3159531
C2orf84	NC_000002	24397972	24414567	0.0247735	0.0509396	0.0220392	0
ITSN2	NC_000002	24425735	24583397	0.1434938	0.1139979	0.3655605	0.9272002
LOC646049	NC_000002	24553271	24553543	0	0	0	0
RPL36AP13	NC_000002	24557381	24557652	0	0	0	0
NCOA1	NC_000002	24807346	24993571	1.4539835	1.4103565	2.2608201	2.8237266
C2orf79	NC_000002	25013136	25016251	7.2222619	9.4790354	8.817458	21.041219
CENPO	NC_000002	25016333	25042784	4.4083457	7.5352012	4.3308073	8.2922747
ADCY3	NC_000002	25042038	25142055	7.5862424	10.183478	9.6565741	11.311076
DNAJC27	NC_000002	25166505	25194824	0.3169561	0.4841411	0.4108749	0.4354248
RPS13P5	NC_000002	25191796	25192110	0	0	0	0
EFR3B	NC_000002	25264973	25382004	0.2710671	0.8481733	0.0209695	0.1108704
POMC	NC_000002	25383722	25391559	0	0	0.0301912	0.0236486
DNMT3A	NC_000002	25455845	25565459	0.1559831	0.184422	0.1803969	0.249999
LOC100288694	NC_000002	25468894	25470435	0	0	0	0.038377
LOC100131510	NC_000002	25592818	25593529	0	0	0	0
DTNB	NC_000002	25600112	25896503	1.2434827	0.730532	1.5803381	2.314815
LOC100289053	NC_000002	25610093	25611509	0	0	0	0
ASXL2	NC_000002	25962253	26101312	0.863772	1.0455039	1.4823722	2.9327142
LOC751599	NC_000002	26031557	26033596	0	0	0	0
LOC100288719	NC_000002	26045338	26045819	0	0	0	0
KIF3C	NC_000002	26149455	26205443	0.453158	0.7321192	0.4751315	0.5808069
LOC729769	NC_000002	26214890	26217950	0	0	0	0
LOC391358	NC_000002	26251074	26251481	0	0.1107436	0.0958274	0
RAB10	NC_000002	26256974	26360278	18.61041	16.444431	22.205627	26.050862
RPS2P15	NC_000002	26324186	26325005	0	0	0	0
LOC391359	NC_000002	26363124	26364133	0	0.1602248	0	0.1085989
LOC646196	NC_000002	26372073	26373600	0	0	0	0
PPIL1P1	NC_000002	26384702	26386728	0	0	0	0
FAM59B	NC_000002	26395015	26412665	0	0	0	0
HADHA	NC_000002	26413504	26467594	11.996406	14.163516	11.65074	15.075467
HADHB	NC_000002	26467616	26513333	18.659921	17.367112	21.514383	22.100861
GPR113	NC_000002	26531041	26569685	0.0895408	0.0418442	0.1158661	0.1247911
SELI	NC_000002	26568954	26618759	3.1248712	4.3743803	3.5110374	4.2646528
C2orf39	NC_000002	26624784	26679579	0.0529284	0.0181387	0	0.0245884
OTOF	NC_000002	26680071	26781566	0.0648987	0.0727884	0.0787305	0.045224
C2orf70	NC_000002	26785481	26802395	0	0	0	0.0440646
CIB4	NC_000002	26804073	26864211	0	0.1212977	0	0.0822144
RPL37P11	NC_000002	26901087	26901378	0	0	0	0
KCNK3	NC_000002	26915581	26954066	0.0773347	0.0227166	0.0196569	0.0615885
C2orf18	NC_000002	26987142	27004099	9.4479711	9.2284095	10.194151	13.183102

CENPA	NC_000002	27008882	27017457	4.8831369	8.4304461	6.8031404	9.5876628
LOC100128579	NC_000002	27050037	27070694	0	0	0	0
DPYSL5	NC_000002	27070969	27173219	0.016874	0	0.0150115	0.0293961
MAPRE3	NC_000002	27193525	27250087	4.0422973	3.5276881	6.5859536	5.2242439
TMEM214	NC_000002	27255774	27264565	28.522981	31.316259	27.535489	25.325968
AGBL5	NC_000002	27274491	27293490	2.4401186	3.4209523	6.547217	8.3745585
LOC100128731	NC_000002	27293340	27294567	67.321111	73.94491	67.918974	58.545712
EMILIN1	NC_000002	27301506	27309271	5.1369539	7.7350432	3.3870402	1.3067248
LOC100288662	NC_000002	27307803	27309442	0.0267977	0.1102034	0	0.0373474
KHK	NC_000002	27309611	27323619	0.4480996	0.1594708	0.7666189	0.4203415
CGREF1	NC_000002	27323469	27341971	6.8252936	2.2249402	6.241224	3.4800998
ABHD1	NC_000002	27346657	27353680	0.0607859	0.0312472	0.3514996	0.2117903
PREB	NC_000002	27353624	27357542	5.6432372	7.4505656	4.623084	8.6711549
C2orf53	NC_000002	27359715	27362332	0.0213031	0	0.0379036	0.0148448
TCF23	NC_000002	27372002	27375735	0	0.0700518	0.0606164	0.0474804
SLC5A6	NC_000002	27422458	27435071	3.1162021	3.8790202	2.001497	4.3137746
C2orf28	NC_000002	27434899	27440046	21.251076	20.620179	27.267616	13.209443
CAD	NC_000002	27440258	27466654	7.4442419	9.8719504	4.6424234	9.6639845
SLC30A3	NC_000002	27477440	27485960	0.0839909	0.1079393	0.0560405	0.0292641
DNAJC5G	NC_000002	27498289	27504296	0	0	0	0.0152666
TRIM54	NC_000002	27505600	27530307	0.0250132	0.0257162	0.0222525	0
UCN	NC_000002	27530265	27531130	1.1433686	0.5877516	0.3814397	1.0457276
MPV17	NC_000002	27532360	27545969	23.654361	13.595403	23.023691	25.272367
GTF3C2	NC_000002	27548721	27579868	4.7199504	5.2214493	8.4777885	10.906207
EIF2B4	NC_000002	27587219	27593324	5.2153756	7.6735524	4.0417315	5.3464318
SNX17	NC_000002	27593389	27599994	36.118004	44.033359	27.041048	35.196874
ZNF513	NC_000002	27600102	27603593	4.3824782	4.3152687	4.3746808	2.8244855
PPM1G	NC_000002	27604061	27632496	6.0080139	8.0620185	5.5184314	5.2196863
FTHL3P	NC_000002	27615490	27616443	0	0	0	0
NRBP1	NC_000002	27651473	27665126	9.9038269	15.150091	5.4889358	12.341784
KRTCAP3	NC_000002	27665263	27667164	0	0.1054455	0.1368643	0.035735
IFT172	NC_000002	27667240	27712571	0.4181641	0.5142141	0.226124	0.4513741
LOC100288811	NC_000002	27672438	27678729	0	0	0	0
FNDC4	NC_000002	27714750	27718126	3.3664714	2.2442982	0.6083403	0.5498183
GCKR	NC_000002	27719706	27746551	0.1606148	0.0412822	0.0893046	0.0419711
C2orf16	NC_000002	27799389	27805589	0.2409676	0.1311565	0.145016	0.1777932
ZNF512	NC_000002	27805893	27845963	4.42561	3.50811	5.6717718	6.7578541
CCDC121	NC_000002	27848510	27851879	0.0777295	0.3676046	0.0691503	0.1299959
GPN1	NC_000002	27851515	27873713	10.624357	14.365063	11.258669	13.049402
SUPT7L	NC_000002	27873679	27886449	2.6300866	3.0221238	2.6792981	3.2486376
SLC4A1AP	NC_000002	27886338	27917847	0.7398692	1.5517532	1.1189539	2.3303779
MRPL33	NC_000002	27994584	28002608	6.0237924	6.9781316	12.151946	10.937457
RBKS	NC_000002	28004266	28113223	2.0577767	2.1156108	1.5364428	2.4581842
BRE	NC_000002	28113482	28561768	7.3525559	4.8748252	13.620967	14.235328
RPL23AP34	NC_000002	28531028	28531437	0	0	0	0
FOSL2	NC_000002	28615779	28637516	11.870758	5.5361272	10.638927	15.52346
LOC100133088	NC_000002	28683001	28701042	0	0	0	0
PLB1	NC_000002	28718982	28866613	0.1215238	0.1784847	0.0540555	0.1875116
PPP1CB	NC_000002	28974614	29025806	13.831646	14.771844	26.221497	21.029328
SPDYA	NC_000002	29033700	29073476	0.0203747	0.1047367	0.3443921	0.1561769
TRMT61B	NC_000002	29072687	29093175	1.2631542	1.739708	1.6538016	1.6441773
WDR43	NC_000002	29117533	29171080	5.3475998	6.0786582	4.4223468	5.6333681
SNORD92	NC_000002	29136528	29136616	0	0	0	0
SNORD53	NC_000002	29149933	29150008	0	0	0	0
FAM179A	NC_000002	29204164	29275096	0.0371603	0.0764094	0.0440784	0.0776843
C2orf71	NC_000002	29284558	29297127	0.0187173	0.0064145	0.0166514	0.0217383
CLIP4	NC_000002	29338308	29406679	5.9084015	4.658845	7.0022762	8.0840513
ALK	NC_000002	29415640	30144432	0.077722	0.2469832	0.2262882	0.152632
YPEL5	NC_000002	30369750	30383399	9.152383	10.531859	16.97166	15.973618
LBH	NC_000002	30454397	30482899	4.0986438	3.7678467	0.0266151	0.0833897
LCLAT1	NC_000002	30670123	30867091	1.7457697	1.7055395	1.8621568	2.0699028
CAPN13	NC_000002	30945637	31030311	0.0163864	0	0.0437333	0.0456747
GALNT14	NC_000002	31133333	31361571	0.0810255	0.2832293	0.057666	0.1129236
LOC100129588	NC_000002	31394223	31394564	0	0	0.1143204	0
CAPN14	NC_000002	31395922	31440411	0.0804913	0.0591096	0	0.0160256
EHD3	NC_000002	31457203	31491260	2.9192518	7.5915177	1.0148126	0.9060109

RPL21P70	NC_000002	31514048	31514500	0	0	0	0
XDH	NC_000002	31557188	31637611	0.3228661	0.8930776	0.2940695	0.3696198
SRD5A2	NC_000002	31749656	31806040	0.0359495	0.0184799	0.0159908	0.0501021
AK2P2	NC_000002	32048479	32048899	0	0	0	0
MEMO1	NC_000002	32092892	32235698	5.1846315	6.700295	5.6469472	9.0321444
DPY30	NC_000002	32248972	32264844	10.108093	14.741085	22.334484	14.814785
SPAST	NC_000002	32288680	32382706	1.4500472	2.1148571	1.7175029	2.6318714
SLC30A6	NC_000002	32390933	32446809	5.5428586	6.8051736	6.0162376	6.3499748
DDX50P1	NC_000002	32426567	32429287	0	0	0	0
NLRC4	NC_000002	32449518	32490801	0.0653408	0.1612253	0.058129	0.1001706
LOC100288882	NC_000002	32465416	32475066	0	0	0	0.0745131
YIPF4	NC_000002	32502958	32531658	6.7991158	7.1281703	7.4613675	6.5301903
BIRC6	NC_000002	32582096	32843966	4.4947373	5.2771037	7.0337275	9.3729329
TTC27	NC_000002	32853129	33046118	2.4144022	2.3408647	1.984786	2.7047007
LOC285045	NC_000002	33050510	33162270	0	0	0	0
LOC100271832	NC_000002	33152194	33171203	0	0	0	0
LTBP1	NC_000002	33172392	33624576	13.060613	6.660188	2.921794	1.6000966
RASGRP3	NC_000002	33661416	33789798	0.2390347	0.1667608	0.2734095	0.0713867
FAM98A	NC_000002	33808729	33824362	3.4902421	3.9833819	3.0480434	3.3581378
LOC100133262	NC_000002	33827109	33827353	0	0	0	0
LOC100288283	NC_000002	33861569	33862525	0	0	0	0
MYADML	NC_000002	33951128	33953284	0	0	0	0
SLC25A5P2	NC_000002	34064589	34065809	0	0	0	0
LOC100130842	NC_000002	35444443	35444670	0	0	0	0
MRPL50P1	NC_000002	35949825	35950222	0	0	0	0
RPL21P36	NC_000002	36526502	36527027	0	0	0	0
LOC100288911	NC_000002	36565035	36582895	2.5484204	1.2853046	1.0694083	0.9716865
CRIM1	NC_000002	36583397	36778278	25.869957	26.969248	21.593283	34.727208
FEZ2	NC_000002	36779401	36825332	8.684758	9.1229494	5.4867228	8.37616
VIT	NC_000002	36923914	37041930	1.3366446	1.4901085	0.5444146	0.9202052
LOC100288939	NC_000002	37067065	37069988	0.0848968	0.1745656	0.3524571	0.6901937
STRN	NC_000002	37075472	37193615	4.4466162	7.1690825	7.6756647	7.3151118
HEATR5B	NC_000002	37208153	37311485	1.2035293	1.3371413	1.9168859	2.4122953
CCDC75	NC_000002	37311594	37323738	0.2447396	0.4313451	0.4043503	0.5359963
EIF2AK2	NC_000002	37333699	37384190	5.6547725	3.9169229	7.5659794	3.4596706
LOC100128455	NC_000002	37375515	37376447	0	0	0	0
SULT6B1	NC_000002	37394963	37415690	0.0470538	0.0967525	0.125581	0.0655779
RPL31P16	NC_000002	37421236	37421902	0	0	0	0
CEBPZ	NC_000002	37428772	37458740	2.6527311	3.1750499	3.6397133	4.8558366
C2orf56	NC_000002	37458774	37476303	1.2522563	1.1852724	1.6799044	2.4516524
PRKD3	NC_000002	37477645	37544222	5.4944596	5.4957958	6.9147651	6.422768
QPCT	NC_000002	37571753	37600465	1.9096707	2.6266335	2.6172181	0.2877264
CDC42EP3	NC_000002	37870743	37899326	13.446106	21.496449	3.0150597	3.9925425
LOC100129149	NC_000002	37898114	37899889	0	0	0	0
LOC344382	NC_000002	38044058	38045104	0	0	0.0373425	0.0877504
FAM82A1	NC_000002	38177606	38294280	0.2435418	0.1593369	0.1575721	0.4782727
CYP1B1	NC_000002	38294746	38303323	28.946303	4.9541281	13.672388	7.4467247
LOC100288457	NC_000002	38298085	38302793	0.0522261	0.0805408	0.0232309	0.0363932
C2orf58	NC_000002	38358247	38408997	0	0	0	0
RPL7P12	NC_000002	38458690	38459525	0	0	0	0
LOC391367	NC_000002	38512551	38513557	0	0	0	0
ATL2	NC_000002	38522027	38604432	3.5623121	5.6881717	7.2669466	6.6650938
RPLP0P6	NC_000002	38708916	38710017	0	0	0	0
LOC100288550	NC_000002	38708917	38710026	0.2915996	0.9422131	0.5558896	1.0740479
HNRPLL	NC_000002	38790328	38830178	4.112454	4.2875848	5.0498338	4.0261372
GALM	NC_000002	38893052	38961909	4.1948171	3.1480985	3.6688412	5.2788755
SFRS7	NC_000002	38970741	38978636	9.6583699	11.581764	10.634412	16.167573
LOC646640	NC_000002	38978532	38992372	0	0	0	0
LOC100289072	NC_000002	38996404	38997406	0	0	0	0
GEMIN6	NC_000002	39005327	39009107	4.8831369	8.3455634	5.6417844	5.7891184
DHX57	NC_000002	39024871	39103021	0.2729704	0.2993517	0.3804525	0.6213743
ASSP2	NC_000002	39037357	39038866	0	0	0	0
MORN2	NC_000002	39103103	39109850	4.7447484	4.5122426	5.3290879	6.0753807
LOC100271715	NC_000002	39146589	39202590	0.2198291	0.153685	0.2346793	0.4534296
SOS1	NC_000002	39208690	39347604	3.0221674	2.8898257	7.3090544	6.0196773
CDKL4	NC_000002	39405688	39456673	0	0.0288343	0.0998023	0.1368055

MAP4K3	NC_000002	39476422	39664219	2.2759478	1.5379714	1.5874771	2.1518575
LOC728730	NC_000002	39745780	39892483	0.0959568	0	0.4268293	0.0668665
TMEM178	NC_000002	39893090	39945103	0.0808367	0	0.0958861	0.0375535
THUMPD2	NC_000002	39963445	40006377	1.4271848	2.422203	1.8541114	2.0837546
SLC8A1	NC_000002	40339286	40739575	3.8289299	4.8048973	0.6637319	1.2850285
LOC729984	NC_000002	41383705	41384695	0	0	0	0
LDHAL3	NC_000002	42046788	42048789	0	0	0	0
RPS12P4	NC_000002	42077321	42077823	0	0	0	0
LOC400950	NC_000002	42142936	42180435	0.0936065	0	0.1665498	0.0652287
SGK493	NC_000002	42275161	42285668	2.0559549	1.8427457	1.4851134	0.5632725
EML4	NC_000002	42396490	42559688	6.4627423	7.3365012	7.1727017	9.89555
COX7A2L	NC_000002	42577642	42588356	19.790475	24.0277	33.147175	28.184758
KCNG3	NC_000002	42669157	42721237	0.0574637	0.0354472	0.2147094	0.2642837
RPS13P3	NC_000002	42696930	42697444	0	0	0	0
LOC100130921	NC_000002	42721784	42722521	0.6796118	0.3105388	0.8733133	1.631222
MTA3	NC_000002	42795671	42936353	4.5675868	4.5256396	6.1688674	5.8710052
OXER1	NC_000002	42989639	42991401	0.0498562	0.0512574	0.0887069	0.0173709
HAAO	NC_000002	42994229	43019751	0.5134139	0.7389809	0.0608996	0.0954046
LOC100128048	NC_000002	43051868	43054845	0	0	0	0
ZFP36L2	NC_000002	43449541	43453745	27.013942	15.611703	13.890064	9.1136586
LOC100129726	NC_000002	43454350	43455994	0	0	0	0
THADA	NC_000002	43457975	43823185	2.7957065	4.1242743	2.0899293	3.7670802
PLEKHH2	NC_000002	43864439	43995126	1.1502126	0.9421331	0.4610297	0.2466197
LOC728819	NC_000002	43902292	43903461	0	0	0	0
DYNC2LI1	NC_000002	44001182	44037149	1.5541228	1.1932949	1.8551217	1.6724419
ABCG5	NC_000002	44039611	44065958	0	0.0164903	0.0142692	0.0447079
ABCG8	NC_000002	44066103	44105605	0	0.0169544	0.0146708	0.0344745
LRPPRC	NC_000002	44113363	44223144	3.9506267	4.1777069	4.146622	9.6607865
PPM1B	NC_000002	44396000	44461742	0.9945966	1.2386986	1.1653736	1.1776632
RPL12_2_212	NC_000002	44497672	44498296	0	0	0	0
SLC3A1	NC_000002	44502597	44547963	0.2082855	0.2336066	0.5221993	0.4354248
PREPL	NC_000002	44545902	44586889	5.4980955	3.1737302	8.2225138	11.582976
C2orf34	NC_000002	44589043	44999729	0.7923699	0.3782255	0.6293877	1.2423486
LOC100130502	NC_000002	45148216	45150662	0	0	0	0
SIX3	NC_000002	45169037	45172390	0.0517952	0.0532509	0.1612746	0
LOC730043	NC_000002	45173413	45224012	0	0	0	0
SIX2	NC_000002	45232324	45236542	4.2642435	0.9019899	2.3778	3.1705422
LOC100289242	NC_000002	45396123	45398184	0.0332941	0	0	0.0232007
SRBD1	NC_000002	45615819	45838433	2.1058032	2.446313	1.8945491	2.8602011
PRKCE	NC_000002	45879043	46415129	0.7483938	0.5893487	0.8499471	0.737882
TRNAQ39P	NC_000002	45937441	45937513	0	0	0	0
RPL26P15	NC_000002	46231052	46231560	0	0	0	0
RPL36AP14	NC_000002	46483999	46484314	0	0	0	0
EPAS1	NC_000002	46524563	46613836	13.283691	7.95854	19.023728	27.45391
LOC388946	NC_000002	46706704	46711565	0	0	0	0
ATP6V1E2	NC_000002	46738986	46747096	0.3331094	0.5022915	0.8100051	0.5416224
RHOQ	NC_000002	46769867	46811827	3.028577	3.4718202	5.4919082	7.1808664
PIGF	NC_000002	46808413	46844251	4.4607649	3.5492699	5.1071842	5.9736082
CRIPT	NC_000002	46844325	46852881	1.2831601	1.4605688	2.6092223	2.5228002
LOC730078	NC_000002	46881304	46896806	0	0.0538539	0.0466002	0.0365016
SOCS5	NC_000002	46926099	46989927	2.4038608	2.190358	1.7443787	2.2071984
LOC388948	NC_000002	47043822	47049800	0.0427097	0	0.1519828	0.1488089
LOC100134259	NC_000002	47055003	47086145	0	0	0	0
MCFD2	NC_000002	47129013	47142949	18.819733	15.678334	13.784641	14.146555
TTC7A	NC_000002	47168313	47303275	2.7606101	2.3126053	1.8191965	2.5827495
C2orf61	NC_000002	47355587	47382426	0	0.0882488	0.0190906	0.0299071
CALM2	NC_000002	47387305	47403650	144.50708	129.702	152.43891	103.90196
BCYRN1	NC_000002	47562454	47562653	0	0	0	0
EPCAM	NC_000002	47596287	47614167	0.1279052	0	0.4096369	0.1782589
MSH2	NC_000002	47630263	47710360	7.9651803	9.5826166	8.7021609	11.938347
LOC100288705	NC_000002	47717436	47748478	0	0	0.1257156	0.0492361
KCNK12	NC_000002	47747914	47797470	0	0	0.0548738	0
LOC100131554	NC_000002	47799405	47800126	0	0	0	0
RPL18AP6	NC_000002	47917806	47918420	0	0	0	0
LOC100289315	NC_000002	47932607	47933178	0	0	0	0
LOC100289345	NC_000002	47958535	47997107	0	0	0	0

MSH6	NC_000002	48010221	48034092	5.4529114	6.0133178	6.8565278	10.95363
RPL36AP15	NC_000002	48024893	48025279	0	0	0	0
FBXO11	NC_000002	48034059	48132814	4.6625034	4.2431367	4.0353191	3.9069612
RPS27AP7	NC_000002	48110106	48111053	0	0	0	0
LOC100133087	NC_000002	48216764	48217665	0	0	0	0
FOXN2	NC_000002	48541795	48606434	3.6603415	3.8211114	5.1171077	4.0138043
KLRAQ1	NC_000002	48667908	48742525	1.0271478	0.9996951	0.8284931	1.2120161
FLJ46838	NC_000002	48756528	48759056	0	0	0	0
STON1-GTF2A1L	NC_000002	48796159	48906742	0.3447822	0.874365	0.633904	0.7848426
STON1	NC_000002	48807763	48826045	0.0150973	0.0465648	0.0201465	0.0157806
GTF2A1L	NC_000002	48844948	48960287	0.0456842	0.0234841	0.020321	0.0159173
LHCGR	NC_000002	48913913	48982880	0	0.0146083	0.037922	0
LOC646936	NC_000002	49142189	49143726	0	0	0	0
FSHR	NC_000002	49189652	49381630	0	0.0189687	0.0328275	0.064284
RPL7P13	NC_000002	50105750	50106572	0	0	0	0
NRXN1	NC_000002	50145643	51259674	0.0248155	0.0255129	0.0183971	0.0317028
LOC730100	NC_000002	51259745	51555575	0	0	0	0
LOC100128029	NC_000002	51737280	51738621	0	0	0	0
CRYGGP1	NC_000002	52002370	52002654	0	0	0	0
LOC100131852	NC_000002	52154020	52154539	0	0	0	0
LOC129656	NC_000002	52789481	52799665	0	0	0	0
ASB3	NC_000002	53897118	54014079	2.2232168	2.0612121	1.7835836	1.6045555
CHAC2	NC_000002	53994929	54002287	0.4825688	0.5315694	0.1839885	0.4323512
C2orf30	NC_000002	54014068	54046495	14.025133	14.144243	12.339347	10.725086
GPR75	NC_000002	54080050	54087126	0.3948068	0.2136331	0.3512311	0.7384722
PSME4	NC_000002	54091204	54197977	6.9646092	8.2741826	8.1180183	9.9695864
RPL21P30	NC_000002	54256624	54257009	0	0	0	0
ACYP2	NC_000002	54342410	54532437	0.7540138	0.6644618	0.5110793	0.7005691
TSPYL6	NC_000002	54480315	54483409	0	0	0	0
C2orf73	NC_000002	54558071	54588714	0.0915112	0	0.0407054	0.0637686
SPTBN1	NC_000002	54683454	54898583	2.4093241	2.1821529	1.0296733	3.2802292
RPL23AP32	NC_000002	54756359	54756978	0	0	0	0
EML6	NC_000002	54952149	55199157	0.18468	0.2071313	0.2489339	0.487471
RNU7-81P	NC_000002	55077427	55077486	0	0	0	0
RTN4	NC_000002	55199325	55277734	57.156348	43.273266	86.557082	62.438501
LOC100289544	NC_000002	55232261	55277698	0.1140525	0.156344	0.1691071	0.1059684
C2orf63	NC_000002	55399683	55459699	0.1215831	0.1083335	0.1730619	0.1807444
LOC100128027	NC_000002	55451417	55452263	0	0	0	0
RPS27A	NC_000002	55459065	55462749	4.8083405	6.4265232	5.6036992	6.7347927
MTIF2	NC_000002	55463756	55496384	1.9658752	2.6499212	2.3448176	2.7499477
LOC100289575	NC_000002	55509374	55511293	0	0.047066	0.0407266	0
NCRNA00117	NC_000002	55509455	55511608	0	0	0	0
CCDC88A	NC_000002	55514978	55647057	3.8369636	3.4246588	3.9830446	5.1665443
LOC652959	NC_000002	55662033	55662855	0	0	0	0
CCDC104	NC_000002	55746740	55772216	4.9395241	5.1479165	9.2100501	8.7937487
SMEK2	NC_000002	55775514	55844796	1.6561918	1.1554302	2.9906393	5.3377143
PNPT1	NC_000002	55861198	55921011	5.4611365	3.4832367	5.8829925	3.0430923
EFEMP1	NC_000002	56093102	56150932	69.673669	15.592409	26.438021	100.16017
LOC100129434	NC_000002	56400669	56412037	0.0286401	0.7214033	0	0.0099788
CCDC85A	NC_000002	56411258	56613309	0.1544753	5.2182689	0.0785289	0.2152891
LOC647016	NC_000002	57275484	57276507	0	0	0	0
LOC100131953	NC_000002	57992049	57993191	0	0	0	0
VRK2	NC_000002	58273729	58387055	3.4247946	4.2537527	3.5575262	4.0557271
FANCL	NC_000002	58386378	58468515	2.7404209	1.5913323	3.047443	4.9332223
LOC339799	NC_000002	58478564	58479822	0	0	0	0
LOC644456	NC_000002	58687052	58689167	0	0	0	0
LOC730134	NC_000002	59465850	59477046	0	0	0	0
LOC100286902	NC_000002	59733686	59742264	0	0	0	0
BCL11A	NC_000002	60678302	60780633	0.0059023	0.0182044	0.0210033	0.8513766
LOC100286932	NC_000002	60722805	60727099	0	0	0	0.0683591
LOC442017	NC_000002	60909819	60910426	0	0	0	0
RPL26P13	NC_000002	60938586	60939069	0	0	0	0
ATP1B3P1	NC_000002	60961525	60963058	0	0	0	0
PAPOLG	NC_000002	60983383	61026098	1.2630587	1.7988209	1.7775807	1.522226
RPL21P33	NC_000002	61079355	61079920	0	0	0	0
REL	NC_000002	61108752	61150178	0.7969008	0.923889	1.2217989	1.0042881

LOC100271700	NC_000002	61163065	61165003	0	0	0	0
RPS12P3	NC_000002	61165312	61165820	0	0	0	0
PUS10	NC_000002	61167548	61245365	0.3229791	0.4862256	0.2770694	0.5063956
PEX13	NC_000002	61244812	61279125	1.411405	1.8813908	2.5718664	2.7131675
KIAA1841	NC_000002	61293006	61365169	0.4776982	0.5893487	0.5382998	0.4604828
LOC339803	NC_000002	61370099	61372110	2.5960981	3.3935213	0.9898118	1.2663452
C2orf74	NC_000002	61372243	61391964	0.4953182	0.7407115	0.040059	0.4079133
LOC100286967	NC_000002	61404033	61412082	0.4994117	0.0855746	0.1110726	0.1450042
AHSA2	NC_000002	61404821	61414058	1.912581	1.1628859	1.7014851	3.4393874
USP34	NC_000002	61414590	61697849	5.067064	6.8234527	7.3459175	10.324727
LOC100130280	NC_000002	61476823	61477765	0	0	0	0
SNORA70B	NC_000002	61644379	61644512	0	0	0	0
XPO1	NC_000002	61705069	61765418	16.396421	20.655269	24.19516	29.990823
RPS29P10	NC_000002	61816628	61816798	0	0	0	0
LOC647077	NC_000002	61937206	61938113	0	0	0	0
RPS24P7	NC_000002	62030161	62030668	0	0	0	0
FAM161A	NC_000002	62051983	62081278	0.5237601	0.6241478	0.7201068	0.6055298
RPL31P30	NC_000002	62083830	62084197	0	0	0	0
LOC100127901	NC_000002	62085272	62093205	0	0	0	0
CCT4	NC_000002	62095262	62115791	29.78839	42.840989	42.816357	50.726555
COMMD1	NC_000002	62132803	62363205	9.1841326	13.371245	6.0318825	4.8106401
RPSAP26	NC_000002	62373432	62374461	0	0	0	0
B3GNT2	NC_000002	62423262	62451866	3.4052286	2.5804999	2.7307139	1.3814059
LOC100130512	NC_000002	62576080	62592334	0	0	0	0
TMEM17	NC_000002	62727356	62733604	0.3424538	0.445966	0.5890023	0.6681792
RPL37P13	NC_000002	62734677	62734958	0	0	0	0
RPL21P37	NC_000002	62759722	62760234	0	0	0	0
LOC100287103	NC_000002	62779475	62780048	0	0	0	0
LOC100129162	NC_000002	62780115	62788558	0.079186	0	0	0
EHBP1	NC_000002	62901013	63273622	4.8522252	7.7226518	4.1492483	4.1908929
RPS20P9	NC_000002	63167008	63167457	0	0	0	0
LOC100132215	NC_000002	63271057	63274846	0	0	0	0
OTX1	NC_000002	63277965	63284314	0.0201968	0	0.0359353	0.0422218
RPL27P5	NC_000002	63335231	63335564	0	0	0	0
ELP1P	NC_000002	63344986	63346677	0	0	0	0
C2orf86	NC_000002	63348518	63815851	0.3876645	0.4384158	0.4138525	0.7473875
MDH1	NC_000002	63816122	63834328	30.049777	31.89207	37.062519	36.083256
LOC388955	NC_000002	63849182	63850159	0	0	0	0
LOC100131592	NC_000002	63869613	63870966	0	0	0	0
RPS4P5	NC_000002	63870332	63871202	0	0	0	0
RPL27P6	NC_000002	63911405	63911880	0	0	0	0
LOC391378	NC_000002	63978825	64062075	0	0	0	0
FLJ36848	NC_000002	64066315	64069318	0	0	0	0
UGP2	NC_000002	64068098	64118696	16.482841	16.556741	13.219694	16.613085
VPS54	NC_000002	64119667	64246214	2.2117162	2.9865844	3.2401838	3.711177
PELI1	NC_000002	64319786	64371605	0.6183319	0.359836	1.0586546	0.4227485
RPL23AP37	NC_000002	64574389	64574879	0	0	0	0
HSPC159	NC_000002	64681327	64688517	1.2746939	0.9445185	2.0330325	0.7922297
AFTPH	NC_000002	64751465	64820139	2.5309377	2.2933498	4.3982376	3.7589834
SERTAD2	NC_000002	64858755	64881046	22.980141	13.9834	22.402877	26.518009
LOC730184	NC_000002	64868899	64873832	0	0	0	0
RPS10P9	NC_000002	64892705	64893257	0	0	0	0
RPL11P1	NC_000002	65187142	65187750	0	0	0	0
SLC1A4	NC_000002	65215579	65250999	4.8912484	2.7864349	7.679879	9.7483261
CEP68	NC_000002	65283495	65314142	1.2070395	2.2969385	1.1938697	1.4210111
LOC100287235	NC_000002	65298659	65302399	0.3587611	0.092211	0	0.1249995
RAB1A	NC_000002	65313988	65357435	15.630472	14.155073	14.423052	13.105084
LOC729317	NC_000002	65432108	65433811	0.1031649	0.2916769	0.0688337	0.3055299
LOC729324	NC_000002	65452469	65455079	0	0	0	0
ACTR2	NC_000002	65454829	65498387	23.337025	26.370384	18.395931	32.150673
SPRED2	NC_000002	65537985	65659656	5.5195199	4.3531536	4.5571786	5.4005162
LOC100289180	NC_000002	65571624	65593871	0	0.0809738	0.0700673	0
LOC100129140	NC_000002	65727847	65731767	0	0	0	0
RPS15AP15	NC_000002	65738902	65739280	0	0	0	0
LOC100131818	NC_000002	65836238	65861120	0	0	0.0757705	0.1187011
KRT18P33	NC_000002	65893562	65894928	0	0	0	0

LOC729348	NC_000002	66610438	66620174	0	0	0	0
LOC730198	NC_000002	66653867	66661509	0.0818403	0	0	0.0285148
MEIS1	NC_000002	66662532	66799891	2.459222	2.1448267	1.7330263	2.2624478
LOC100289292	NC_000002	66922008	66923951	0.0226071	0.0697275	0.0402238	0.1417818
DNMT3AP1	NC_000002	67047013	67049760	0	0	0	0
ETAA1	NC_000002	67624442	67637533	2.489909	2.0369002	2.941547	4.2164012
LOC402076	NC_000002	68250826	68258022	0	0	0	0
C1D	NC_000002	68269332	68290159	1.8569675	2.7701506	3.6279431	3.3238269
LOC100287361	NC_000002	68316700	68352879	0	0	0	0
WDR92	NC_000002	68357281	68384656	0.7050518	1.2081124	0.6795031	1.5558093
PNO1	NC_000002	68385005	68403091	2.7995335	3.8147766	3.8148787	5.5118588
PPP3R1	NC_000002	68405989	68479651	11.12207	13.310421	11.413736	9.3064642
CNRIP1	NC_000002	68511303	68547183	5.9117215	9.8105829	7.2885168	12.345323
PLEK	NC_000002	68592322	68624585	0.0154096	0.0316854	0.0137088	0.0536902
LOC391383	NC_000002	68675037	68676304	0	0	0	0
FBXO48	NC_000002	68689498	68694390	0.129307	0.2991178	0.7621078	0.4280049
APLF	NC_000002	68694691	68807294	0.9591196	0.9625978	0.9954693	1.1059647
PROKR1	NC_000002	68872954	68882708	0	0	0.0330775	0
ARHGAP25	NC_000002	68961968	69053965	0.0392278	0.0134434	0.0581636	0.0182237
BMP10	NC_000002	69092613	69098649	0	0	0	0
LOC100132413	NC_000002	69101750	69159634	0.0366235	0	0.0325813	0.1020829
GKN2	NC_000002	69172364	69180102	0	0.0564793	0	0
GKN1	NC_000002	69201705	69208112	0	0	0	0
ANTXR1	NC_000002	69240276	69476459	5.6500464	5.371276	4.214306	8.6953163
GFPT1	NC_000002	69546905	69614382	4.6994753	4.0681271	5.0811904	3.8808288
NFU1	NC_000002	69623252	69664753	6.6667233	7.0072564	5.7652343	6.695948
AAK1	NC_000002	69685127	69870977	0.9961461	1.3832304	1.8551349	3.4952542
SNORA36C	NC_000002	69747176	69747303	0	0	0	0
RPL36AP16	NC_000002	69821821	69822218	0	0	0	0
LOC100128857	NC_000002	69824463	69826086	0	0	0	0
ANXA4	NC_000002	69969127	70053596	1.5388036	1.2023594	1.6062492	1.7442741
LOC100287475	NC_000002	70056790	70072065	0.2079569	0.1425344	0	0
GMCL1	NC_000002	70056818	70106727	1.0677959	0.9052088	1.6665629	1.4359491
SNRNP27	NC_000002	70121090	70132349	4.6327196	4.5376493	4.5391048	6.1947759
MXD1	NC_000002	70142203	70170077	0.9990022	0.5661067	1.1196725	0.9811801
ASPRV1	NC_000002	70187221	70189397	0.040375	0.186794	0.0538781	0.0703373
PCBP1	NC_000002	70314585	70316335	38.501763	45.209206	42.13427	38.44288
MRPL36P1	NC_000002	70329621	70330218	0	0	0	0
LOC100289359	NC_000002	70351168	70352449	0.4640085	0.3407496	0	0.0461914
LOC100133985	NC_000002	70351168	70352448	0	0	0	0
C2orf42	NC_000002	70377017	70418151	2.7685297	3.2580741	2.1066834	1.8806879
LOC100287511	NC_000002	70409875	70418091	0	0	0	0.0496351
LOC100289431	NC_000002	70426950	70429671	0.0161456	0.0165993	0.0287271	0.0112509
TIA1	NC_000002	70436576	70475779	3.7119396	4.0007654	2.7140584	3.8371597
RPL39P15	NC_000002	70480810	70481187	0	0	0	0
PCYOX1	NC_000002	70485231	70508317	2.6563465	3.8150018	2.5740261	4.898158
SNRPG	NC_000002	70508510	70520869	9.7068268	34.496545	44.741214	46.070469
FAM136A	NC_000002	70523108	70529220	4.766936	6.8262694	4.8249901	8.0841544
LOC100128042	NC_000002	70569227	70583050	0	0	0	0
TGFA	NC_000002	70674416	70781105	0.0618409	0.0317894	0.082523	0.0646398
ADD2	NC_000002	70889262	70995329	0.0230015	0.5754341	0.0136419	0.0213712
FIGLA	NC_000002	71004442	71017775	0	0	0.0541518	0.1272502
LOC100287582	NC_000002	71028143	71030558	0	0.0760663	0	0.051557
LOC100289458	NC_000002	71034018	71036408	0	0	0	0
CLEC4F	NC_000002	71035775	71047732	0.0355137	0	0.015797	0.0247474
CD207	NC_000002	71057347	71062953	0	0.0483762	0	0.0327889
VAX2	NC_000002	71127720	71160576	0.6513687	1.5363145	1.3634722	0.8810994
ATP6V1B1	NC_000002	71162998	71192561	0.0226654	0.1864194	0.0806551	0.0789708
ANKRD53	NC_000002	71205575	71212629	0.4975272	0.1491905	0.1659802	0.3466967
TEX261	NC_000002	71213068	71222001	10.011373	11.540756	9.185083	27.841625
LOC100130138	NC_000002	71221646	71230064	0	0	0	0.1411285
OR7E91P	NC_000002	71251205	71257061	0	0	0	0
OR7E46P	NC_000002	71264751	71265895	0	0	0	0
TRNAE41P	NC_000002	71273488	71273560	0	0	0	0
OR7E62P	NC_000002	71282522	71283168	0	0	0	0
NAGK	NC_000002	71295408	71305771	26.1344	27.184677	10.631358	16.324189

MCEE	NC_000002	71336806	71357394	1.5154563	1.1945037	1.5728906	1.1616333
MPHOSPH10	NC_000002	71357444	71377230	0.4424913	0.4003362	0.3464142	0.8880351
LOC100128987	NC_000002	71383049	71399923	0	0	0	0
PAIP2B	NC_000002	71409868	71454233	0.0627832	0.0932356	0.0868835	0.0923607
ZNF638	NC_000002	71558889	71662189	0.6347778	0.7289885	0.4565788	1.7411194
DYSF	NC_000002	71680753	71913893	0.0301925	0.1241643	0.0483482	0.0462866
RPS20P10	NC_000002	72211307	72211564	0	0	0	0
CYP26B1	NC_000002	72356367	72374963	0.0679412	0.0199573	0.2245002	0.3516991
EXOC6B	NC_000002	72406444	73053177	2.0330716	3.5881961	1.5825923	2.5028813
RPS15AP13	NC_000002	72515861	72516244	0	0	0	0
LOC100289520	NC_000002	72920928	72922473	0	0	0	0
SPR	NC_000002	73114512	73119289	0.6626875	0.7742187	0.6967352	1.9940805
EMX1	NC_000002	73144604	73162020	0.4619787	0.3304088	0.1608218	0.3639154
SFXN5	NC_000002	73169165	73298965	2.0700254	2.4119642	0.8782786	2.2783726
LOC100289585	NC_000002	73280258	73280455	0	0	0	0
RAB11FIP5	NC_000002	73300510	73340146	16.690611	17.867319	12.093052	13.951177
NOTO	NC_000002	73429386	73438340	0.030351	0.031204	0	0.0422996
SMYD5	NC_000002	73441366	73454355	7.0193904	8.4135824	5.9247967	8.1006203
C2orf7	NC_000002	73455134	73460356	4.3420691	4.8395888	6.209401	8.4833458
CCT7	NC_000002	73461405	73480147	35.051735	54.759799	45.007663	74.101846
FBXO41	NC_000002	73481800	73498043	0.1666342	0.3997408	0.0658855	0.1290193
EGR4	NC_000002	73518057	73520673	0.0396466	0.0407608	0.052906	0.041441
RPSAP28	NC_000002	73597079	73598091	0	0	0	0
ALMS1	NC_000002	73612886	73837046	1.4794522	2.6889054	2.5687845	3.983936
GNG5P4	NC_000002	73645556	73646039	0	0	0	0
NAT8	NC_000002	73867850	73869537	0	0	0	0.0285414
ALMS1P	NC_000002	73867998	73912699	0	0	0	0
NAT8B	NC_000002	73927636	73928467	0.0528224	0	0	0
TPRKB	NC_000002	73956957	73964517	6.9545739	8.0512977	7.9546909	10.466215
DUSP11	NC_000002	73989325	74007284	7.5347487	9.3178706	7.9197021	7.4927249
C2orf78	NC_000002	74011316	74044274	0	0.0445157	0.0128399	0.0301723
STAMBP	NC_000002	74056086	74090011	5.9823883	5.4528526	8.134073	7.1429013
ACTG2	NC_000002	74120093	74146780	0.2311327	0.1018409	0	0.0460178
DGUOK	NC_000002	74153953	74186088	9.4504065	10.624419	19.856369	15.687219
LOC100287699	NC_000002	74212730	74226990	0.1470824	0.120973	0.1831881	0.3279773
TET3	NC_000002	74273450	74335303	0.2160798	0.3085455	0.5375337	0.5437359
LOC100131507	NC_000002	74347492	74362521	0	0	0	0
BOLA3	NC_000002	74362528	74375039	12.851288	14.354294	8.7510797	7.5733002
MOBKL1B	NC_000002	74383211	74405995	26.818673	30.972219	29.565879	25.532254
MTHFD2	NC_000002	74425690	74442425	13.517898	13.1544	15.832012	16.866078
SLC4A5	NC_000002	74443369	74570534	0.2061362	0.0989005	0.1589332	0.368686
LOC100131156	NC_000002	74571124	74577687	0	0	0	0
DCTN1	NC_000002	74588282	74607475	1.4478546	2.459338	1.1280452	4.4367534
LOC100287788	NC_000002	74592085	74607482	0.0298561	0.1534762	0.0796825	0.0624148
LOC100189589	NC_000002	74612845	74621008	0	0	0	0
C2orf81	NC_000002	74641303	74644844	0.6112136	0.4459555	0.2631061	0.4671359
LOC100130009	NC_000002	74645222	74645569	0.5580728	0	0.2482385	0.3888873
WDR54	NC_000002	74648885	74652882	8.3654811	10.861783	12.962642	9.7704035
RTKN	NC_000002	74652988	74669060	2.9463364	3.9172693	2.0447656	2.536845
INO80B	NC_000002	74682199	74685087	2.3206987	3.243363	1.8387469	2.5015371
WBP1	NC_000002	74685577	74688010	8.0351703	9.1142485	6.1079459	5.5992267
MOGS	NC_000002	74688184	74692537	2.1253031	2.4035385	7.4008518	5.7124125
MRPL53	NC_000002	74699109	74699927	7.2057977	9.6821576	5.8392468	7.755651
CCDC142	NC_000002	74699959	74710357	0.8305497	0.9371989	1.0812878	0.9175465
TTC31	NC_000002	74710216	74721683	4.9016272	4.6505464	3.5261832	6.483408
LBX2	NC_000002	74724644	74730443	0.484278	0.4356526	0.269267	0.738203
LOC151534	NC_000002	74729744	74732192	0	0	0	0
PCGF1	NC_000002	74732170	74734821	4.0261625	4.6879026	8.5013471	7.0646794
TLX2	NC_000002	74741611	74743890	0.4013537	0.5157923	1.0934821	0.7166781
DQX1	NC_000002	74745258	74753408	0.03337	0.0171539	0.0890605	0.1046408
AUP1	NC_000002	74753775	74756974	24.510118	30.018916	27.414637	25.543021
HTRA2	NC_000002	74756532	74760683	5.1186764	6.8926837	4.6457107	4.875961
LOXL3	NC_000002	74759946	74781062	10.392116	30.879909	3.2195048	1.3050653
DOK1	NC_000002	74781860	74784663	7.3712114	13.124702	3.7856373	4.8448882
C2orf65	NC_000002	74785010	74875164	0.0691282	0.0888388	0.1537458	0.0361284
SEMA4F	NC_000002	74881393	74909185	1.9130286	2.1148328	2.6351741	3.8200468

RPS28P5	NC_000002	74981797	74981946	0	0	0	0
HK2	NC_000002	75059782	75120481	7.5653167	5.510171	6.4381724	5.5956355
POLE4	NC_000002	75185775	75196859	20.14294	23.565481	19.380259	36.25704
TACR1	NC_000002	75273590	75426645	0.0968217	0.0723948	0.0704743	0.2453425
FAM176A	NC_000002	75719444	75796848	7.7149846	9.0495776	4.1850391	8.0423087
MRPL19	NC_000002	75873909	75889334	2.0438426	2.828653	2.8872356	3.7796898
LOC100287851	NC_000002	75874306	75878753	0	0	0	0
SUPT4H1P	NC_000002	75877435	75878774	0	0	0	0
C2orf3	NC_000002	75889294	75938322	1.1612165	1.8387074	2.1113439	2.3743879
LOC100129863	NC_000002	76333159	76337172	0	0	0	0
LOC647275	NC_000002	76484688	76486053	0	0	0	0
LRRTM4	NC_000002	76974849	77749502	0.0273707	0	0.0324663	0.0127153
TRNAP25P	NC_000002	77895593	77895663	0	0	0	0
RPL38P2	NC_000002	78019877	78020221	0	0	0	0
SNAR-H	NC_000002	78182033	78182152	0	0	0	0
CYCSP6	NC_000002	78639910	78640220	0	0	0	0
REG3G	NC_000002	79252826	79255631	0	0	0	0
REG1B	NC_000002	79312149	79315150	0	0	0	0.0379961
REG1A	NC_000002	79347584	79350545	0	0.0559201	0	0.0379021
REG1P	NC_000002	79362629	79365553	0	0	0	0
REG3A	NC_000002	79384133	79386879	0	0	0.0380327	0.0595815
CTNNA2	NC_000002	79740126	80875905	0.068687	0.0235392	0.0509215	0.0239319
LRRTM1	NC_000002	80529003	80531487	0	0	0.0176752	0
LOC100287912	NC_000002	81421301	81428307	0	0	0	0
LOC100130209	NC_000002	81645699	81651289	0	0	0	0
LOC100289658	NC_000002	81809822	81810843	0.0430022	0	0	0.0299656
LOC100287985	NC_000002	82836776	82837104	0	0	0	0
LOC100288014	NC_000002	83042857	83044658	0	0	0	0
LOC100286883	NC_000002	83045255	83046391	0	0	0	0
LOC1720	NC_000002	83083927	83084893	0	0	0	0
RPL37P10	NC_000002	83822107	83822370	0	0	0	0
LOC100124401	NC_000002	84258277	84260624	0	0	0	0
LOC388965	NC_000002	84517806	84519324	0	0	0	0
SUCLG1	NC_000002	84650652	84686586	5.9021386	7.530567	9.2897108	8.2678735
DNAH6	NC_000002	84743579	85046713	0.0240436	0.0282507	0.0091671	0.0335091
LOC100288044	NC_000002	84861661	85005347	0	0	0	0
DUXAP1	NC_000002	84977851	84978385	0	0	0	0
LOC100190800	NC_000002	85003113	85005443	0	0	0	0
LOC129293	NC_000002	85048794	85108252	2.7290642	2.9381123	5.2450747	3.749619
RPL12P18	NC_000002	85101651	85102252	0	0	0	0
TMSB10	NC_000002	85132763	85133799	382.22197	447.70939	509.40397	298.24311
RPS2P17	NC_000002	85142887	85143835	0	0	0	0
KCMF1	NC_000002	85198231	85286595	4.2767047	6.5564688	5.9477165	6.1630895
LOC647302	NC_000002	85329508	85329824	0	0	0	0
TCF7L1	NC_000002	85360734	85537505	1.3052354	1.8819599	0.6655507	0.3438505
LOC647305	NC_000002	85541819	85543660	0	0	0	0
TGOLN2	NC_000002	85545146	85555374	13.846894	14.600906	21.907265	26.45493
LOC647307	NC_000002	85568398	85568961	0	0	0	0
RETSAT	NC_000002	85569078	85581821	9.2648341	7.218973	10.296912	8.4354844
ELMOD3	NC_000002	85581843	85618875	1.2921774	1.0219186	1.793814	2.7409019
CAPG	NC_000002	85621871	85637676	166.34497	39.041969	407.65042	349.02167
SH2D6	NC_000002	85661918	85664152	0.0983182	0	0.0437333	0.068512
RNU7-64P	NC_000002	85668940	85669197	0	0	0	0
RPSAP22	NC_000002	85718048	85718903	0	0	0	0
LOC100286944	NC_000002	85764585	85766112	0.7765722	0.4435543	0.2558741	0.481019
MAT2A	NC_000002	85766288	85772403	45.304562	35.919122	63.88201	61.141119
GGCX	NC_000002	85776193	85788657	10.449041	8.0431187	7.0561052	7.752895
VAMP8	NC_000002	85804648	85809156	5.2121892	0.1217881	5.7434429	5.7370055
VAMP5	NC_000002	85811531	85820511	28.615916	12.705709	13.40488	7.2302344
RNF181	NC_000002	85822837	85824831	26.639012	31.300234	32.217268	26.946471
TMEM150	NC_000002	85825671	85829821	3.6812308	1.717668	0.7053685	1.203684
C2orf68	NC_000002	85832376	85839179	1.0304392	1.0911818	3.025134	3.683602
USP39	NC_000002	85843283	85876406	12.235474	14.68614	11.929271	11.631631
SFTPB	NC_000002	85884440	85895312	0.0373286	0	0.0110695	0.0867069
LOC100130649	NC_000002	85908623	85914312	0	0	0	0
GNLY	NC_000002	85921414	85925875	0.044482	0	0.0395724	0.0619937

ATOH8	NC_000002	85981022	86015189	8.5439842	20.39103	3.5835418	16.715922
ST3GAL5	NC_000002	86066271	86116157	0.6103921	1.01515	0.6388491	0.1376118
LOC90784	NC_000002	86247339	86250991	0	0	0	0
POLR1A	NC_000002	86253451	86333278	1.0131203	1.6542966	0.7245744	1.3935081
PTCD3	NC_000002	86333305	86369280	4.4901074	4.8444336	5.2544248	5.7802525
SNORD94	NC_000002	86362993	86363129	0	0	0	0
IMMT	NC_000002	86371055	86422893	19.740825	23.035856	34.355308	40.758335
MRPL35	NC_000002	86426556	86440477	3.5307248	5.5404594	4.2746357	6.4931032
REEP1	NC_000002	86441116	86564777	0.2620714	0.2460076	0.2128724	0.0397004
KDM3A	NC_000002	86668354	86719839	5.3118208	5.8088917	7.1574491	12.123808
VPS24	NC_000002	86730553	86790600	11.56897	10.39845	10.279751	18.315067
RNF103	NC_000002	86830516	86850978	3.3874392	3.2747246	4.4191381	4.4303462
RMND5A	NC_000002	86947414	87005164	1.4286907	1.7392285	3.7497746	3.5959343
CD8A	NC_000002	87011728	87018127	0.1796249	0.0205193	0.035511	0.0973543
CD8B	NC_000002	87042460	87089038	0	0	0.0203845	0.0638684
LOC400965	NC_000002	87052749	87055359	0	0	0	0
LOC100286979	NC_000002	87098454	87425407	0	0	0	0
RGPD1	NC_000002	87140906	87241099	0.038967	0.0400621	0.0288884	0.0135769
PLGLB1	NC_000002	87237587	87248969	0.0291918	0.0300122	0.0259698	0.030513
LOC285074	NC_000002	87257798	87303536	0	0	0	0
TRNAP28P	NC_000002	87339573	87339644	0	0	0	0
LOC100132498	NC_000002	87448252	87450337	0	0	0	0
LOC440871	NC_000002	87565634	87566264	0	0	0	0
LOC100131408	NC_000002	87579582	87585524	0	0	0	0
NCRNA00152	NC_000002	87754974	87821037	0	0	0	0
PAFAH1P1	NC_000002	87864782	87867029	0	0	0	0
RPS14P5	NC_000002	87954374	87954895	0	0	0	0
LOC730268	NC_000002	87991040	88038768	0.7573933	0.9384091	1.4512574	3.0448951
PLGLB2	NC_000002	88047606	88058995	0.0874302	0.0299625	0.0907437	0.2640076
RGPD2	NC_000002	88056717	88093226	0.088427	0.0779248	0.0786671	0.0352111
LOC100287117	NC_000002	88268128	88268493	0	0	0	0
LOC729859	NC_000002	88272037	88273230	0	0	0	0
KRCC1	NC_000002	88326724	88355248	2.3064674	2.9641136	4.3937367	2.9524269
SMYD1	NC_000002	88367382	88412903	0.01011	0.0103942	0	0.0634055
FABP1	NC_000002	88422510	88427578	0	0	0	0.0626276
THNSL2	NC_000002	88469835	88486146	1.1236764	1.1552574	0.0185121	0.0145004
MRPL45P1	NC_000002	88664211	88664959	0	0	0	0
FOXI3	NC_000002	88747726	88752053	0.0348243	0.071606	0.0619613	0.1213347
C2orf51	NC_000002	88824169	88829103	0	0	0	0
EIF2AK3	NC_000002	88856259	88926994	1.4011639	1.8180656	2.2695157	2.1143825
RPIA	NC_000002	88991176	89050452	3.3787899	3.5722973	4.1144112	6.5290771
FLJ40330	NC_000002	89065419	89106110	0	0	0	0
LOC100132330	NC_000002	89111147	89127933	0	0.5748524	0	0
IGK@ (NC_000002 891!	NC_000002	89156874	89630436	0	0	0	0
IGKC	NC_000002	89156874	89157196	0	0	0	0
IGKJ5	NC_000002	89160080	89160117	0	0	0	0
IGKJ4	NC_000002	89160398	89160435	0	0	0	0
IGKJ3	NC_000002	89160733	89160770	0	0	0	0
IGKJ2	NC_000002	89161037	89161075	0	0	0	0
IGKJ1	NC_000002	89161398	89161435	0	0	0	0
IGKV4-1	NC_000002	89185088	89185669	0	0	0	0
IGKV5-2	NC_000002	89196811	89197300	0	0	0	0
IGKV7-3	NC_000002	89214596	89215194	0	0	0	0
IGKV2-4	NC_000002	89231169	89231896	0	0	0	0
IGKV1-5	NC_000002	89246819	89247294	0	0	0	0
IGKV1-6	NC_000002	89265781	89266257	0	0	0	0
IGKV3-7	NC_000002	89277987	89278503	0	0	0	0
IGKV1-8	NC_000002	89291928	89292397	0	0	0	0
IGKV1-9	NC_000002	89309479	89309954	0	0	0	0
IGKV2-10	NC_000002	89319489	89320182	0	0	0	0
IGKV3-11	NC_000002	89326668	89327181	0	0	0	0
IGKV1-12	NC_000002	89339721	89340195	0	0	0	0
IGKV1-13	NC_000002	89345487	89345963	0	0	0	0
IGKV2-14	NC_000002	89377515	89378280	0	0	0	0
IGKV3-15	NC_000002	89384673	89385186	0	0	0	0
IGKV1-16	NC_000002	89399352	89399827	0	0	0	0

IGKV1-17	NC_000002	89416833	89417308	0	0	0	0
IGKV2-18	NC_000002	89428189	89428965	0	0	0	0
IGKV2-19	NC_000002	89434464	89434730	0	0	0	0
IGKV3-20	NC_000002	89442057	89442591	0	0	0	0
IGKV6-21	NC_000002	89459235	89459786	0	0	0	0
IGKV1-22	NC_000002	89470229	89470696	0	0	0	0
IGKV2-23	NC_000002	89471812	89472527	0	0	0	0
IGKV2-24	NC_000002	89475812	89476614	0	0	0	0
IGKV3-25	NC_000002	89491979	89493756	0	0	0	0
IGKV2-26	NC_000002	89495564	89496317	0	0	0	0
IGKV1-27	NC_000002	89512908	89513382	0	0	0	0
IGKV2-28	NC_000002	89521179	89521912	0	0	0	0
IGKV2-29	NC_000002	89533666	89534392	0	0	0	0
IGKV2-30	NC_000002	89544264	89545049	0	0	0	0
IGKV3-31	NC_000002	89551684	89552221	0	0	0	0
IGKV1-32	NC_000002	89553027	89553501	0	0	0	0
IGKV1-33	NC_000002	89567758	89568232	0	0	0	0
IGKV3-34	NC_000002	89575013	89575545	0	0	0	0
IGKV1-35	NC_000002	89586453	89586927	0	0	0	0
IGKV2-36	NC_000002	89594981	89595214	0	0	0	0
IGKV1-37	NC_000002	89597021	89597495	0	0	0	0
IGKV2-38	NC_000002	89609660	89609899	0	0	0	0
IGKV1-39	NC_000002	89619383	89619857	0	0	0	0
IGKV2-40	NC_000002	89629873	89630436	0	0	0	0
IGK@ (NC_000002 898	NC_000002	89890568	90274235	0	0	0	0
IGKV2D-40	NC_000002	89890568	89891301	0	0	0	0
IGKV1D-39	NC_000002	89901315	89901789	0	0	0	0
IGKV2D-38	NC_000002	89911273	89911512	0	0	0	0
IGKV1D-37	NC_000002	89923550	89924024	0	0	0	0
IGKV2D-36	NC_000002	89925831	89926064	0	0	0	0
IGKV1D-35	NC_000002	89934124	89934598	0	0	0	0
IGKV3D-34	NC_000002	89945569	89946101	0	0	0	0
IGKV1D-33	NC_000002	89952881	89953355	0	0	0	0
IGKV1D-32	NC_000002	89967232	89967706	0	0	0	0
IGKV3D-31	NC_000002	89968512	89969049	0	0	0	0
IGKV2D-30	NC_000002	89975704	89976489	0	0	0	0
IGKV2D-29	NC_000002	89986352	89987081	0	0	0	0
IGKV2D-28	NC_000002	89998825	89999557	0	0	0	0
IGKV1D-27	NC_000002	90007679	90008154	0	0	0	0
IGKV2D-26	NC_000002	90024762	90025514	0	0	0	0
IGKV3D-25	NC_000002	90027310	90029068	0	0	0	0
IGKV2D-24	NC_000002	90043637	90044439	0	0	0	0
IGKV2D-23	NC_000002	90047721	90048726	0	0	0	0
IGKV1D-22	NC_000002	90049551	90050018	0	0	0	0
IGKV6D-21	NC_000002	90060444	90060995	0	0	0	0
IGKV3D-20	NC_000002	90077777	90078311	0	0	0	0
IGKV2D-19	NC_000002	90085638	90085904	0	0	0	0
IGKV2D-18	NC_000002	90091428	90092204	0	0	0	0
IGKV6D-41	NC_000002	90108530	90109082	0	0	0	0
IGKV1D-17	NC_000002	90121658	90122133	0	0	0	0
IGKV1D-16	NC_000002	90139105	90139580	0	0	0	0
IGKV3D-15	NC_000002	90153745	90154258	0	0	0	0
IGKV2D-14	NC_000002	90160639	90161404	0	0	0	0
IGKV1D-13	NC_000002	90192948	90193424	0	0	0	0
IGKV1D-12	NC_000002	90198716	90199190	0	0	0	0
IGKV3D-11	NC_000002	90211740	90212253	0	0	0	0
IGKV2D-10	NC_000002	90218731	90219496	0	0	0	0
IGKV1D-42	NC_000002	90229065	90229531	0	0	0	0
IGKV1D-43	NC_000002	90248920	90249395	0	0	0	0
IGKV1D-8	NC_000002	90259774	90260248	0	0	0	0
IGKV3D-7	NC_000002	90273704	90274235	0	0	0	0
LOC100287657	NC_000002	90292233	90293473	0	0	0	0
LOC100287688	NC_000002	90411130	90414534	0.0433415	0	0	0
LOC100287723	NC_000002	90447482	90459040	0	0.040074	0.0173382	0
LOC100287753	NC_000002	90518597	90519837	0	0	0	0
LOC100289247	NC_000002	91634600	91636392	0	0	0	0

IGKV1OR15-118	NC_000002	91646155	91679742	0	0	0	0
LOC100132182	NC_000002	91741454	91747799	0	0	0	0
LOC388996	NC_000002	91777536	91809450	0	0	0	0
LOC654342	NC_000002	91824710	91847975	0	0	0	0
DRD5P1	NC_000002	91871911	91874157	0	0	0	0
FLJ37786	NC_000002	91909572	91911768	0	0	0	0
LOC100289350	NC_000002	91924722	91959554	0	0	0	0
GGT8P	NC_000002	91963368	91970153	0	0	0	0
LOC642838	NC_000002	91996926	92006299	0.0494913	0.1017644	0	0
LOC642762	NC_000002	92028072	92031839	0	0	0	0
LOC100128696	NC_000002	92066164	92068229	0	0	0	0
LOC389000	NC_000002	92070932	92119739	0	0	0	0
LOC440888	NC_000002	92128767	92130592	0	0	0	0
LOC391405	NC_000002	92222523	92231898	0	0	0	0
LOC100130488	NC_000002	95400395	95401687	0	0	0	0
LOC205272	NC_000002	95402887	95403963	0	0	0	0
ANKRD20B	NC_000002	95426673	95522820	0	0	0	0
LOC100132883	NC_000002	95454619	95454846	0	0	0	0
LOC647633	NC_000002	95524519	95524937	0	0	0	0
LOC100289554	NC_000002	95537178	95538599	0	0	0	0
TEKT4	NC_000002	95537232	95542568	0.0906149	0	0.0537424	0.2736243
RPS24P6	NC_000002	95578156	95578645	0	0	0	0
LOC442028	NC_000002	95582300	95614060	0	0	0	0
LOC100289586	NC_000002	95689407	95691314	0	0	0	0
LOC100131662	NC_000002	95691153	95692215	0	0	0	0
MAL	NC_000002	95691479	95719735	0.0832353	0	0.0370242	0.0580017
MRPS5	NC_000002	95752952	95787754	11.694834	9.3576702	10.38051	12.37164
ZNF514	NC_000002	95813400	95825263	1.1110508	0.916642	1.3056927	1.902107
ZNF2	NC_000002	95831183	95850062	0.4338094	0.7319002	0.5343631	0.5813379
LOC100287523	NC_000002	95851163	95858403	0	0	0	0
SLC2AXP1	NC_000002	95862243	95863132	0	0	0	0
LOC344065	NC_000002	95873564	95881351	0.4348619	0.3353128	0.5802978	1.3257523
PROM2	NC_000002	95940201	95957054	0.0344963	0.0354658	0.184133	0.2323709
KCNIP3	NC_000002	95963072	96051825	0.437006	1.2430303	0.9589724	1.9387974
FAHD2A	NC_000002	96068448	96078879	3.2842669	2.6107513	2.6506824	2.6189226
UBTFL5	NC_000002	96116053	96135411	0	0	0	0
LOC653192	NC_000002	96142715	96150479	0.0790436	0.0270884	0	0
LOC390233	NC_000002	96180575	96185860	0	0	0	0
LOC643085	NC_000002	96190721	96192450	0	0	0	0
OR7E102P	NC_000002	96212226	96213421	0	0	0	0
TRNAE31P	NC_000002	96217194	96217265	0	0	0	0
LOC643126	NC_000002	96240649	96246517	0	0	0	0
TRIM43	NC_000002	96257766	96265471	0	0	0.0240305	0.0188229
UBTFL3	NC_000002	96272821	96292179	0	0.0657691	0	0
LOC100287711	NC_000002	96325872	96327488	0.217431	0.0558855	0.314328	0.2840898
LOC643219	NC_000002	96455417	96465927	0	0	0	0
LOC400986	NC_000002	96502518	96657607	0.5529133	0.4909367	0.6856607	2.2066725
LOC729234	NC_000002	96676299	96688884	0	0	0	0
LOC150763	NC_000002	96687694	96700727	0.1089368	0.0319996	0.055379	0.065067
ADRA2B	NC_000002	96778623	96781888	0.094194	0	0	0.0375075
ASTL	NC_000002	96789589	96804175	0	0	0.0905036	0
LOC100288133	NC_000002	96808905	96810296	0.2733949	0.1054045	0.1216098	0.3572109
DUSP2	NC_000002	96808908	96811179	1.0432815	0.6703769	0.1856264	0.3635
STARD7	NC_000002	96850597	96874573	18.423203	21.009948	14.86516	17.406873
LOC285033	NC_000002	96905977	96907701	0.4092014	0.9255445	0.8008812	1.3687096
TMEM127	NC_000002	96916312	96931732	10.078432	9.0195004	7.2471723	11.870051
CIAO1	NC_000002	96931884	96939917	4.218076	4.5707364	6.5789637	6.1884469
SNRNP200	NC_000002	96940074	96971261	4.1111479	6.4628705	4.4095818	8.056482
ITPRIPL1	NC_000002	96991146	96994079	0.3508472	0.2088308	0.1478479	0.0772056
NCAPH	NC_000002	97001484	97041274	5.3676483	8.3430976	5.9059504	12.100063
NEURL3	NC_000002	97163383	97173846	0	0	0	0
LOC100287739	NC_000002	97166163	97171094	0.0725218	0.1491201	0.3225872	0.1010722
ARID5A	NC_000002	97202464	97218371	1.4343938	1.1265127	0.9570574	0.749657
KIAA1310	NC_000002	97258907	97304115	1.9209161	1.4362936	1.901244	2.7061151
FER1L5	NC_000002	97308574	97370624	0.0673123	0.083045	0.0359298	0.1219554
LMAN2L	NC_000002	97371666	97405813	6.4402239	10.357227	8.0339656	9.4895755

CNNM4	NC_000002	97426639	97477628	1.9410469	1.8073361	1.946733	1.569525
LOC100288569	NC_000002	97454322	97463384	0	0.1521327	0.0658208	0
CNNM3	NC_000002	97481991	97501121	1.0102014	1.01102	1.2327345	1.6259343
ANKRD23	NC_000002	97503651	97509758	0.119147	0.0524981	0.1665659	0.3439665
ANKRD39	NC_000002	97513722	97523756	1.1719528	1.5563172	1.7811113	1.8034651
SEMA4C	NC_000002	97525473	97535735	2.7220189	2.8112421	3.8084903	3.3970163
FAM178B	NC_000002	97541619	97652301	0.046003	0.0157653	0.0272837	0.0213712
LOC643445	NC_000002	97691512	97699368	0	0	0	0
LOC653924	NC_000002	97746819	97749209	0	0	0.0880576	0.1379499
FAHD2B	NC_000002	97749323	97760582	0.6948337	1.2144155	1.0508437	1.1620507
ANKRD36	NC_000002	97779326	97915981	0.3926933	0.30924	0.4088148	1.2401329
IGKV2OR2-7	NC_000002	98000700	98001604	0	0	0	0
UBE3AP1	NC_000002	98021380	98021916	0	0	0	0
ANKRD36B	NC_000002	98121261	98206329	0.1510508	0.279533	0.2754769	0.7631221
UBTFL6	NC_000002	98253048	98255087	0	0	0	0
COX5B	NC_000002	98262521	98264657	33.505088	56.993717	65.343258	44.633486
ACTR1B	NC_000002	98272402	98280561	9.7511356	9.1647467	6.8048465	7.256116
LOC728537	NC_000002	98280404	98319529	0	0	0	0
RNU4P2	NC_000002	98280755	98281433	0	0	0	0
LOC100287998	NC_000002	98321579	98329836	0	0	0	0
ZAP70	NC_000002	98330031	98356323	0.017282	0	0	0
TMEM131	NC_000002	98372799	98612354	3.3933538	4.5632244	4.1898656	5.5819879
LOC728851	NC_000002	98443673	98444813	0	0	0	0
VWA3B	NC_000002	98703595	98929410	0.0370324	0.0190366	0.0164725	0.0645142
CNGA3	NC_000002	98962618	99015064	0.1142106	0.1878728	0.0711234	0.0716278
INPP4A	NC_000002	99061321	99207496	1.7647118	1.9434416	1.9330891	3.2034025
C2orf64	NC_000002	99215865	99224955	2.1154384	1.1418188	3.6698076	3.2062147
UNC50	NC_000002	99225042	99234978	4.1525888	3.9925841	4.2415557	2.7865156
MGAT4A	NC_000002	99235569	99347589	0.0104863	0.0323432	0.0093289	0.0146146
LOC100130391	NC_000002	99310566	99311956	0	0	0	0
C2orf55	NC_000002	99410309	99552684	0	0.0233566	0.030316	0.0237464
RNU7-46P	NC_000002	99457042	99457297	0	0	0	0
TSGA10	NC_000002	99613724	99771187	0.1720312	0.0471643	0.0714204	0.0559431
LOC550644	NC_000002	99718442	99719564	0	0	0	0
C2orf15	NC_000002	99758185	99767950	0.2151312	1.7062264	0.027341	0.021416
LIPT1	NC_000002	99771418	99779620	1.2948353	1.1485093	1.2422681	0.6015285
MITD1	NC_000002	99785726	99797492	4.4431179	4.3693839	7.089119	5.855746
MRPL30	NC_000002	99797578	99814089	4.9781765	6.2788924	6.1484689	8.0466301
LYG2	NC_000002	99858709	99871570	0	0	0	0.035201
LYG1	NC_000002	99900701	99917639	0.1775686	0.228199	0.1184775	0.1237369
TXNDC9	NC_000002	99935487	99952860	3.9352338	6.1425801	6.1329515	5.304309
EIF5B	NC_000002	99953834	100016728	0.6548268	0.644378	0.6075186	2.1511728
REV1	NC_000002	100016938	100106480	1.7174531	1.9184334	1.9243204	2.9887397
AFF3	NC_000002	100163715	100759037	0.9685413	0.6656753	1.5947503	2.692215
LOC150577	NC_000002	100824716	100867946	0	0	0	0
LONRF2	NC_000002	100889753	100939195	0.0189541	0.0422214	0.008431	0.0264159
CYCSP7	NC_000002	100986291	100987013	0	0	0	0
CHST10	NC_000002	101008322	101034091	1.7953985	3.4991054	1.222233	2.0561641
LOC100288057	NC_000002	101010785	101035069	0	0	0.0270946	0
LOC100289659	NC_000002	101015582	101017024	0	0	0	0
LOC129522	NC_000002	101041150	101043126	0	0	0	0
NMS	NC_000002	101086944	101099742	0	0	0	0
LOC643896	NC_000002	101125739	101126694	0	0	0	0.0320344
PDCL3	NC_000002	101179418	101193201	1.062934	1.8073361	0.6910267	1.3959247
LOC100131946	NC_000002	101207515	101207786	0	0	0	0
LOC100131574	NC_000002	101247126	101280630	0	0	0	0
NPAS2	NC_000002	101436613	101613287	1.3829863	2.0763601	3.6910289	2.692297
RPL31	NC_000002	101618691	101636155	61.040859	115.65853	100.13306	67.74281
TBC1D8	NC_000002	101623690	101767846	1.6744202	1.1658124	1.7064527	3.1754757
C2orf29	NC_000002	101869345	101886778	7.7494102	9.6001271	11.133024	9.6812562
SNORD89	NC_000002	101889398	101889511	0	0	0	0
LOC100288163	NC_000002	101890563	101891122	1.3208485	1.2345192	1.3887113	0.5857217
RNF149	NC_000002	101892553	101925152	8.0655401	9.2135811	6.6650826	4.1465985
CREG2	NC_000002	101964816	102003965	0.0125998	0.2461252	0	0.0263402
LOC731220	NC_000002	102013823	102091165	2.5418295	2.116747	2.4647974	1.4878483
RPS6P3	NC_000002	102125983	102126805	0	0	0	0

MAP4K4	NC_000002	102314488	102511152	4.0582959	5.9958731	2.3146093	3.5155379
IL1R2	NC_000002	102608306	102644885	0.0836048	0.0859545	0.0495847	0.0194197
LOC100131131	NC_000002	102737460	102739005	0	0	0	0
IL1R1	NC_000002	102770402	102796334	7.3351542	7.0072486	3.2587944	4.8118568
IL1RL2	NC_000002	102803433	102855811	0.6265532	0.5061277	1.1944266	0.9199938
LOC100129822	NC_000002	102866227	102866715	0	0	0	0
IL1RL1	NC_000002	102927962	102968497	0.1402468	0.1572964	0.5671245	0.159921
IL18R1	NC_000002	102979097	103015218	0.0873474	0.141118	0.7659659	0.4260701
IL18RAP	NC_000002	103035254	103069025	0	0.0169353	0.0439628	0.0229572
SLC9A4	NC_000002	103089762	103150431	0	0.0436766	0.0094484	0.0222027
SLC9A2	NC_000002	103236166	103327809	0.0242273	0.0415136	0.0215532	0.02251
MFSD9	NC_000002	103333666	103353337	0.6821233	0.5343196	1.0980839	0.9506614
TMEM182	NC_000002	103378490	103434138	0.4164548	0.6296461	0.3704898	0.307273
LOC728815	NC_000002	104110699	104112825	0	0	0	0
LOC644265	NC_000002	104240034	104240321	0.4577941	1.5688681	0.4072663	0.8506911
LOC100287010	NC_000002	104995539	104996105	0	0	0	0.0540121
LOC402093	NC_000002	105011458	105012738	0	0	0	0
LOC150568	NC_000002	105050805	105129215	0	0	0	0
RPL23AP27	NC_000002	105294690	105295133	0	0	0	0
LOC100287073	NC_000002	105363096	105365828	0	0	0	0.0541076
LOC644442	NC_000002	105387741	105388347	0	0	0	0
POU3F3	NC_000002	105471969	105473471	0.1462017	0.0300621	0.052026	0.0203758
LOC100128131	NC_000002	105486304	105489129	0	0.0850912	0	0
MRPS9	NC_000002	105654483	105716418	0.8138561	0.7127697	1.4748739	1.2602831
GPR45	NC_000002	105858200	105859924	0	0	0	0.1775355
LOC100288263	NC_000002	105880817	105883455	1.0945418	1.5411772	1.5452747	1.9731243
TGFBRAP1	NC_000002	105883529	105946148	3.984561	3.7172376	3.6498063	4.4322775
LOC100287152	NC_000002	105912844	105924776	0	0.0437824	0.0378852	0.0296753
C2orf49	NC_000002	105954013	105961984	3.5673875	4.3808033	4.1433722	3.7633729
FHL2	NC_000002	105977283	106055230	3.162867	3.5127034	1.5284699	5.4556091
NCK2	NC_000002	106432885	106510728	1.1295931	1.3790918	1.88422	2.1400517
C2orf40	NC_000002	106682113	106694611	0	0.0576319	0	0.0781247
UXS1	NC_000002	106709759	106810795	5.5251616	5.8105335	7.8795478	5.8780956
RPL22P10	NC_000002	106863827	106864199	0	0	0	0
LOC100131114	NC_000002	106869675	106870172	0	0	0	0
RPS21P2	NC_000002	106905200	106905556	0	0	0	0
RPL27AP4	NC_000002	106921211	106921660	0	0	0	0
LOC402096	NC_000002	106961022	106998052	0	0	0	0
PLGLA	NC_000002	107002769	107007851	0	0	0	0
RGPD3	NC_000002	107021136	107084801	0.0299324	0.1923353	0.0798861	0.1095049
CD8BP	NC_000002	107103833	107124137	0	0	0	0
LOC644604	NC_000002	107313494	107315132	0	0	0	0
ST6GAL2	NC_000002	107418056	107503563	0.0310896	0.0127853	0.0165949	0.0519947
LOC649489	NC_000002	107557336	107610393	0	0	0	0
LOC729121	NC_000002	108439520	108443225	0	0	0	0
RGPD4	NC_000002	108443388	108509000	0.0551498	0.1259994	0.0708684	0.1836126
RPL22P8	NC_000002	108531366	108531751	0	0	0	0
LOC100128964	NC_000002	108537063	108537704	0	0	0	0
SLC5A7	NC_000002	108602995	108630439	0.0341943	0.0263665	0.0076051	0.011914
SULT1C3	NC_000002	108863651	108881807	0	0.0493808	0.0427296	0.0334698
LOC100131624	NC_000002	108893269	108894435	0	0	0	0
SULT1C2	NC_000002	108905095	108926371	0.30414	0.0469032	0.3111571	0.0317905
SULT1C4	NC_000002	108994421	109004270	0.0264112	0	0.0234961	0.0184044
LOC100288503	NC_000002	109035637	109040345	0	0	0	0
GCC2	NC_000002	109065636	109125854	2.846026	3.4419851	2.6505866	2.9527022
LIMS1	NC_000002	109204767	109300889	2.5835015	5.9024692	1.2768637	2.2603601
LOC100288532	NC_000002	109311203	109320902	0	0	0	0
RPL10P5	NC_000002	109328692	109329412	0	0	0	0
RANBP2	NC_000002	109335937	109402267	1.1033029	2.0101232	1.5824649	4.9084533
CCDC138	NC_000002	109403219	109492847	0.5403471	1.2756698	1.0326315	1.255118
RPL39P16	NC_000002	109494764	109494919	0	0	0	0
EDAR	NC_000002	109510927	109605828	0.0208582	0.0321666	0	0.0290697
LOC100287216	NC_000002	109743783	109745862	0	0	0	0
SH3RF3 (NC_000002 10	NC_000002	109745997	110107395	0.761085	0.6550956	0.0472383	0.0370015
SH3RF3 (NC_000002 11	NC_000002	110259066	110262207	0.5035443	0.1006632	0.0124435	0.0487347
SEPT10	NC_000002	110300376	110371783	6.6854255	7.7342209	7.0409261	7.6985711

RPL37P12	NC_000002	110310114	110310423	0	0	0	0
ANKRD57	NC_000002	110371911	110376564	2.5401965	2.2912082	2.0078036	1.1318176
LOC100131577	NC_000002	110422786	110436450	0	0	0	0
LOC100286911	NC_000002	110489878	110494567	0	0	0	0
RPL22P11	NC_000002	110494260	110495353	0	0	0	0
RPL22P15	NC_000002	110494261	110526876	0	0	0	0
RGPD5	NC_000002	110550335	110615263	0.0846243	0.2030063	0.0501894	0.0786261
LIMS3	NC_000002	110656009	110664033	0.1084249	0.557361	0.0643052	0.0503699
LIMS3-LOC440895	NC_000002	110656009	110726134	0	0	0	0
LOC440895	NC_000002	110705267	110726151	0	0	0	0
GPAA1P1	NC_000002	110741545	110742764	0	0	0	0
LOC645129	NC_000002	110744436	110752010	0	0	0	0
LOC645135	NC_000002	110765228	110768849	0	0	0	0
LOC100287011	NC_000002	110779927	110805172	0	0	0	0
MALL	NC_000002	110841447	110874143	1.6941821	17.196558	0.0894097	0.4902382
LOC100287041	NC_000002	110841447	110873639	1.4520341	0.5971375	0.172236	0.3102961
NPHP1	NC_000002	110880913	110962639	0.4153595	0.3613358	0.2984547	0.6011426
LOC100287074	NC_000002	110941576	110942758	0	0	0	0
NCRNA00116	NC_000002	110969106	110980517	0	0	0	0
LOC100287119	NC_000002	111001078	111045083	2.3708685	6.7210534	0.3411926	1.3484179
LOC442041	NC_000002	111117696	111121317	0	0	0	0
LOC151009	NC_000002	111132686	111142113	0	0	0	0
LOC729248	NC_000002	111134613	111142223	0	0	0	0
LOC100288570	NC_000002	111143920	111191647	0.595385	0.3400657	0.3334965	0.2612258
LOC100288695	NC_000002	111206682	111230619	0.3121816	0.2496321	0.0617168	0.0725135
RGPD6	NC_000002	111271171	111335608	0.0475287	0.1194466	0.0704715	0.0367999
RGPD7	NC_000002	111296065	111336309	0.1197137	0.3145334	0.2011679	0.0926903
RPL22P12	NC_000002	111391269	111392074	0	0	0	0
BUB1	NC_000002	111395409	111435684	8.2199006	16.321246	12.593391	16.309366
ACOXL	NC_000002	111490150	111875799	0.0264669	0.0272107	0.0470913	0.0184432
RPL5P9	NC_000002	111725353	111726357	0	0	0	0
BCL2L11	NC_000002	111878491	111926022	0.2644	0.271831	0.5292395	0.4951569
LOC100128130	NC_000002	111995301	111996145	0	0	0	0
RPS14P4	NC_000002	112053133	112053658	0	0	0	0
LOC541471	NC_000002	112124579	112252692	0	0	0	0
PAFAH1P2	NC_000002	112140746	112143106	0	0	0	0
RPL34P8	NC_000002	112432603	112432920	0	0	0	0
LOC100287218	NC_000002	112458191	112495215	0	0	0.0266514	0.0417517
LOC100133059	NC_000002	112500163	112502242	0	0	0	0
ANAPC1	NC_000002	112526638	112641741	4.6301607	7.2012836	6.5162609	6.3413682
LOC100130388	NC_000002	112645463	112647062	0	0	0	0
MERTK	NC_000002	112656191	112786945	0.1696843	0.1619923	0.0323477	0.092905
TMEM87B	NC_000002	112812800	112876895	3.2126394	3.6583645	1.8978672	1.8215104
FBLN7	NC_000002	112895962	112945791	5.8304527	4.1272355	8.1800474	5.3417034
ZC3H8	NC_000002	112973439	113012664	0.4109872	0.9859221	0.7312512	0.9737337
ZC3H6	NC_000002	113033178	113097640	0.4570403	0.2075327	0.9012872	0.6210435
RGPD8	NC_000002	113125736	113191222	0.3804282	0.6378267	0.4113341	0.6566261
TTL	NC_000002	113239743	113290223	10.076434	14.069502	11.016065	14.001808
POLR1B	NC_000002	113299492	113334727	0.4468761	0.3675485	0.4120098	1.1720003
CHCHD5	NC_000002	113342036	113346617	1.8937574	3.6254141	1.2780779	1.7746958
RPS20P11	NC_000002	113379342	113379858	0	0	0	0
LOC100131455	NC_000002	113402455	113404355	0.9172261	0.4580309	0.3730239	0.7669916
SLC20A1	NC_000002	113403527	113421402	19.727219	27.07429	13.195131	13.746712
NT5DC4	NC_000002	113465074	113500456	0.0339894	0.0349446	0.0302379	0.0236851
CKAP2L	NC_000002	113495444	113522254	6.3646385	9.083617	10.655371	10.086654
IL1A	NC_000002	113531492	113542971	0.1200771	0.3549243	0.5741787	0.4079133
IL1B	NC_000002	113587337	113594356	2.4937248	1.1160119	14.120015	1.2061868
LOC100128413	NC_000002	113610742	113611699	0	0	0	0
IL1F7	NC_000002	113670548	113676459	0.0523817	0.0538539	0.0932004	0.4380197
LOC100287419	NC_000002	113678640	113696545	0	0	0	0
IL1F9	NC_000002	113735606	113743227	0	0.1148731	0.0331335	0.0519066
LOC442042	NC_000002	113753094	113753516	0	0	0	0
IL1F6	NC_000002	113763449	113765621	0.0921347	0	0	0
IL1F8	NC_000002	113779668	113810440	0.2779934	0.0779472	0.6969664	0.0704425
IL1F5	NC_000002	113816215	113822321	0.0941076	0	0.0837207	0.0327889
IL1F10	NC_000002	113825547	113833427	0	0	0	0.0433473

IL1RN	NC_000002	113875470	113891593	0.5615723	5.7094029	2.0538712	0.5362615
PSD4	NC_000002	113931560	113960677	1.2384697	1.9099156	1.2802338	0.8751702
PAX8	NC_000002	113973574	114036498	0.1842466	0.0334279	0.4242399	0.7023708
LOC654433	NC_000002	113993846	114024580	0	0	0	0
LOC100288059	NC_000002	114059873	114061461	0	0	0.0984206	0.0385461
LOC100130100	NC_000002	114163973	114164672	0.228897	0	0	0.2126728
CBWD2	NC_000002	114195383	114253746	0.8910196	1.5357507	0.8859317	2.0270492
FOXD4L1	NC_000002	114256661	114258727	0.0212618	0.0437188	0.0189151	0
LOC729468	NC_000002	114288678	114328603	0	0	0.1002502	0.0785253
FAM138B	NC_000002	114334959	114336429	0	0	0	0
LOC100287524	NC_000002	114341020	114345878	0.03292	0	0	0.1376399
WASH2P	NC_000002	114341230	114356614	0	0	0	0
MGC13005	NC_000002	114356605	114361294	0	0	0	0
RPL23AP7	NC_000002	114368816	114384715	0	0	0	0
RABL2A	NC_000002	114384817	114400975	1.8834956	1.2284888	1.8377658	1.9052344
bA395L14.12	NC_000002	114415450	114416469	0	0	0	0
LOC645529	NC_000002	114424816	114427448	0.0335996	0	0	0.0234135
SLC35F5	NC_000002	114471933	114514400	26.757807	15.880823	10.447067	14.10809
ACTR3	NC_000002	114647537	114716167	23.902523	27.273315	22.878996	29.947337
LOC440900	NC_000002	114737020	114764887	0	0	0	0
LOC391428	NC_000002	115017623	115028754	0	0	0	0
DPP10	NC_000002	115199899	116601936	1.2528908	1.986582	1.7033069	1.6784966
RPSAP23	NC_000002	116225561	116226529	0	0	0	0
LOC100287649	NC_000002	117508173	117509071	0	0	0	0
LOC100130697	NC_000002	117575842	117577236	0	0	0	0
TRNAL46P	NC_000002	117781417	117781491	0	0	0	0
LOC100287713	NC_000002	117781494	117782417	0	0	0	0
DDX18	NC_000002	118572255	118589953	5.8831099	9.5047156	7.7883597	9.0532509
LOC100287740	NC_000002	118593580	118595780	0	0.2223957	0.0641469	0.2763525
HTR5B	NC_000002	118617003	118661256	0	0	0	0
CCDC93	NC_000002	118673147	118771697	1.1578942	1.1039807	1.1394345	1.7985467
LOC100288058	NC_000002	118771750	118773249	0.6445741	0.451834	0.1303252	0.244999
INSIG2	NC_000002	118846050	118867597	2.0927729	1.8017384	1.407694	2.5609654
EN1	NC_000002	119599747	119605759	2.2107652	1.4644182	0.4487904	1.0752827
MARCO	NC_000002	119699745	119752236	0.02395	0.0984924	0.0426132	0.1168251
C1QL2	NC_000002	119913819	119916471	0.6005615	0.4630803	0.2098942	0.3138714
STEAP3	NC_000002	119981384	120023228	3.9475669	6.5600528	3.7364088	7.358976
C2orf76	NC_000002	120060020	120124258	1.0177485	1.1414754	1.6873686	1.0960483
DBI	NC_000002	120124504	120130122	47.067648	45.426978	68.570034	83.702498
TMEM37	NC_000002	120189446	120196096	0.4689201	0.0535666	0.1854064	0.0907673
LOC100288409	NC_000002	120194281	120208127	0.0423802	0.1742851	0.7163488	0.0295322
SCTR	NC_000002	120197419	120282028	0	0	0.0214468	0.0335983
LOC100287807	NC_000002	120230779	120282028	0.0704863	0	0	0.0245588
PCDP1	NC_000002	120302047	120414237	0.1044255	0.0920232	0.066357	0.0519771
TMEM177	NC_000002	120436743	120439694	1.1126135	1.8198148	1.867145	2.2730778
RPL17P15	NC_000002	120456165	120457766	0	0	0	0
LOC645955	NC_000002	120480778	120517562	0.0980987	0	0.1745427	0.0683591
PTPN4	NC_000002	120517207	120735037	1.1591722	1.0886186	1.2097142	1.2349368
RPL27P7	NC_000002	120750744	120751215	0	0	0	0
EPB41L5	NC_000002	120770669	120936695	0.3158359	0.4697968	0.1554337	0.3090584
LOC729669	NC_000002	120932471	120975897	0.1296408	0.1332844	0.1153321	0.1806777
TMEM185B	NC_000002	120978854	120980984	0	0	0	0
RALB	NC_000002	121010414	121052286	9.8936975	14.72807	9.4588096	15.87455
INHBB	NC_000002	121103719	121109383	0.2195353	0.0282132	0.0244131	0.4589429
LOC84931	NC_000002	121221911	121223925	0	0	0	0
GLI2	NC_000002	121554867	121750229	0.4934356	1.8623385	0.5775974	0.7148369
TFCP2L1	NC_000002	121974163	122042778	0.0236612	0	0.0084198	0.0197856
RPS17P7	NC_000002	122067572	122067992	0	0	0	0
CLASP1	NC_000002	122095352	122407052	2.2783973	3.0806865	2.7810185	4.0971121
RNU4ATAC	NC_000002	122288456	122288585	0	0	0	0
RPL12P15	NC_000002	122416001	122416636	0	0	0	0
LOC440902	NC_000002	122466085	122470204	0	0	0	0
MKI67IP	NC_000002	122484521	122494503	5.588354	8.8131031	7.2661184	9.0042076
TSN	NC_000002	122513121	122525429	3.6236658	5.8467958	4.6692222	7.0900907
LOC100131284	NC_000002	124484968	124520651	0	0	0	0
LOC100288443	NC_000002	124769622	124771990	0	0.0381455	0	0.0258547

LOC100287999	NC_000002	124773760	124782750	0	0.0759385	0.0657102	0.0514704
CNTNAP5	NC_000002	124782864	125672864	0.0249517	0.017102	0.0147985	0.0347747
LOC150554	NC_000002	127312878	127315841	0	0	0	0
GYPC	NC_000002	127413684	127454246	28.970543	11.306397	21.173285	32.197584
LOC339760	NC_000002	127646773	127659013	0.097016	0.0997426	0.0863081	0.2704184
BIN1	NC_000002	127805607	127864864	7.596601	8.9113121	6.9194042	7.3949837
CYP27C1	NC_000002	127941412	127963343	0.3377025	0.0192885	0.0083453	0.0130736
LOC100130102	NC_000002	128004834	128007663	0	0	0	0
ERCC3	NC_000002	128014866	128051752	8.1663805	10.482549	7.4356461	13.731082
MAP3K2	NC_000002	128062139	128100770	4.2427255	6.3554876	5.8950995	5.1568303
PROC	NC_000002	128176017	128186818	0.1001669	0.0514911	0.0668334	0
IWS1	NC_000002	128238383	128284087	0.2929882	0.451834	0.2606504	0.663539
MYO7B	NC_000002	128293378	128395303	0.0981718	0.0471011	0.0232897	0.054728
LIMS2	NC_000002	128396001	128431883	2.2270522	4.5792875	0.1995501	0.770367
GPR17	NC_000002	128403747	128410213	0	0	0	0.014852
LOC100288504	NC_000002	128410216	128411929	0	0	0	0
SFT2D3	NC_000002	128458597	128461407	0.4690313	0.5304348	0.5980773	0.6863633
WDR33	NC_000002	128461808	128568745	1.4728086	2.1693857	1.4572347	1.8519663
LOC100288089	NC_000002	128476895	128477811	0	0	0.1181195	0
RNY4P7	NC_000002	128556480	128556572	0	0	0	0
LOC100131832	NC_000002	128598180	128599339	0	0	0	0
POLR2D	NC_000002	128603840	128615729	4.1585424	4.1393826	3.9349808	5.0975604
RPS26P19	NC_000002	128604305	128604654	0	0	0	0
AMMECR1L	NC_000002	128619204	128642434	0.2552931	0.4180048	0.3112328	0.5139287
LOC646509	NC_000002	128642710	128647040	0.2313065	0.226998	0.1590092	0.0879183
LOC100288164	NC_000002	128643553	128651226	1.3689567	0.7279818	0.3779571	0.6578921
LOC100288192	NC_000002	128666816	128689158	0	0	0	0
SAP130	NC_000002	128698796	128784867	4.3288088	4.2390732	2.8112507	10.23343
UGCGL1	NC_000002	128848785	128948995	5.4449719	8.2447017	7.1517794	7.120571
LOC100288533	NC_000002	128960935	128961309	0	0	0	0
RPL21P34	NC_000002	128975127	128975683	0	0	0	0
HS6ST1	NC_000002	129023054	129076171	0.2001691	0.4458888	0.7024107	0.88341
LOC100130549	NC_000002	129274850	129284042	0	0	0	0
LOC151121	NC_000002	129999746	130000359	0	0	0	0
LOC100287217	NC_000002	130248768	130254295	0	0	0	0
LOC389033	NC_000002	130680435	130691890	0	0	0	0
RPL22P7	NC_000002	130697421	130697790	0	0	0	0
RAB6C	NC_000002	130737235	130740311	0.0428485	0.0146842	0	0.0199057
LOC646674	NC_000002	130750462	130751613	0	0	0	0
LOC100130693	NC_000002	130763773	130766771	0	0	0	0
LOC440905	NC_000002	130783571	130808704	0	0	0	0
LOC100132088	NC_000002	130794531	130796293	0	0	0	0
POTEF	NC_000002	130831108	130878140	0.0991562	0.090616	0.029404	0.0614187
LOC100132461	NC_000002	130893110	130896405	0	0	0	0
CCDC74B	NC_000002	130896862	130902631	1.6133273	2.457289	3.4286784	2.9146723
SMPD4	NC_000002	130908980	130939694	16.636295	20.617772	15.074864	30.725311
FAM128B	NC_000002	130939703	130948300	24.04828	25.898557	25.031	44.491957
TUBA3E	NC_000002	130949318	130956034	0.0856134	0.0586797	0.1269401	0.0795451
LOC100287310	NC_000002	130960354	130961948	0	0.3502589	0.151541	0.1187011
LOC284988	NC_000002	130968650	130971376	0.0322319	0.0497067	0.0430116	0.0561512
LOC643276	NC_000002	130986893	130989659	0	0	0	0
LOC391436	NC_000002	131006233	131010167	0	0	0	0
LOC100287418	NC_000002	131010366	131033359	0.0675088	0	0	0.0705642
TRNAQ34P	NC_000002	131029911	131029982	0	0	0	0
LOC100288720	NC_000002	131034080	131034757	0	0	0	0
LOC100287451	NC_000002	131037884	131044512	0	0	0	0
RPL19P4	NC_000002	131051362	131052052	0	0	0	0
CCDC115	NC_000002	131095816	131099922	5.3999652	5.4417966	3.6386421	4.1913611
IMP4	NC_000002	131100489	131104197	5.3207106	10.595917	7.1188132	7.2693944
PTPN18	NC_000002	131113580	131132982	2.311804	3.4974342	3.1542326	6.368706
LOC100216479	NC_000002	131174323	131183103	0	0	0	0
LOC732043	NC_000002	131183804	131187096	0	0	0	0
LOC646802	NC_000002	131198341	131199038	0	0.0647327	0	0
LOC653269	NC_000002	131219680	131266860	0	0.022654	0.0196027	0.023032
CFC1B	NC_000002	131278667	131285565	0	0.0389512	0	0.0264008
LOC646743	NC_000002	131297394	131306853	0	0	0	0

LOC150527	NC_000002	131328419	131341734	0	0	0	0
CFC1	NC_000002	131350335	131357082	0	0	0	0.0607034
LOC653781	NC_000002	131369106	131415610	0	0.0275761	0.0238618	0.0560724
C2orf14	NC_000002	131437623	131443436	0	0	0	0
LOC100132887	NC_000002	131449566	131452842	0	0	0	0
LOC100288897	NC_000002	131453549	131461323	0	0	0	0
GPR148	NC_000002	131486723	131487909	0	0	0	0
FAM123C	NC_000002	131513077	131525707	0	0.0069792	0	0.0094609
CYCSP8	NC_000002	131534219	131534530	0	0	0	0
ARHGEF4	NC_000002	131674224	131804836	0.5368353	0.4988536	0.0275529	0.1079101
FAM168B	NC_000002	131805449	131851004	9.8611182	12.511941	11.8392	16.858979
PLEKHB2	NC_000002	131862420	131907425	7.6103484	6.5236945	6.5797899	7.2154716
NF1L2	NC_000002	131946021	131955578	0	0	0	0
LOC100287589	NC_000002	131958712	131975100	0	0	0	0
POTEE	NC_000002	131975924	132022416	0	0	0.0227311	0.0890258
LOC100128270	NC_000002	132044682	132045038	0	0	0	0
LOC100287617	NC_000002	132056255	132058026	0	0	0	0
LOC150519	NC_000002	132057771	132068935	0	0	0	0
LOC100287648	NC_000002	132085674	132088667	0	0	0	0
LOC401002	NC_000002	132109656	132110812	0	0	0	0
LOC150786	NC_000002	132118065	132121731	0	0.0369649	0.010662	0
LOC100130819	NC_000002	132120967	132121632	0	0.081854	0	0.0554799
LOC100287681	NC_000002	132127861	132129651	0	0	0	0
LOC100289214	NC_000002	132138486	132139163	0	0	0	0
LOC100289181	NC_000002	132139878	132140195	0	0	0	0
TRNAS31P	NC_000002	132140142	132140211	0	0	0	0
TRNAQ36P	NC_000002	132143133	132143204	0	0	0	0
LOC100287712	NC_000002	132152785	132156142	0.0823001	0	0	0
LOC389043	NC_000002	132160474	132163200	0	0	0	0
GNAQP	NC_000002	132180315	132182481	0	0	0	0
LOC401010	NC_000002	132199734	132202467	0	0	0	0
LOC730041	NC_000002	132203046	132221203	0	0	0	0
LOC646836	NC_000002	132227710	132229299	0.6813679	0.1751295	0	0.3561032
TUBA3D	NC_000002	132233666	132240507	0.0284271	0.029226	0.050579	0.2377093
FAM128A	NC_000002	132241533	132250064	16.805601	22.493901	19.294903	16.262562
LOC150776	NC_000002	132250386	132279149	0	0	0	0
CCDC74A	NC_000002	132285492	132291239	2.0812821	3.6283171	3.4884582	4.2458651
LOC100127989	NC_000002	132291694	132294998	0	0	0	0.034256
ACTBL3	NC_000002	132349268	132384999	0.0149841	0.0154052	0.0666512	0.0730904
LOC100287806	NC_000002	132414630	132415649	0	0	0	0
C2orf27A	NC_000002	132480064	132524977	0.7931592	0.9637149	0.5452491	0.6029508
LOC100128267	NC_000002	132528454	132535326	0	0	0	0.1017438
LOC100287862	NC_000002	132546214	132547294	0	0	0	0
C2orf27B	NC_000002	132552534	132559234	0.066337	0	0.0295076	0.0462262
LOC100289256	NC_000002	132578635	132581149	0.0174744	0.0179656	0	0.0121769
LOC647996	NC_000002	132580236	132587709	0	0	0	0
LOC100289293	NC_000002	132722304	132737446	0	0.0204542	0.0176992	0.0138637
LOC100287925	NC_000002	132795056	132796401	0	0	0	0
LOC730076	NC_000002	132842995	132862654	0	0	0	0
NCRNA00164	NC_000002	132905164	133015542	0	0	0	0
LOC100133239	NC_000002	133009893	133017101	0	0	0	0
LOC647696	NC_000002	133018761	133020670	0	0	0	0
LOC100132958	NC_000002	133023011	133028148	0	0	0	0
ZNF806	NC_000002	133066882	133076309	0.0744885	0.0255273	0	0.0346044
AZFP	NC_000002	133089314	133090325	0	0	0	0
LOC339742	NC_000002	133106835	133112412	0.1363642	0.0801124	0.034661	0.095024
GPR39	NC_000002	133174147	133404169	0.9035794	2.4345541	0.443503	1.802102
LYPD1	NC_000002	133402337	133429070	0.1317569	1.3244968	0.0260477	0.0816119
NAP5	NC_000002	133429372	134326031	0.0983829	0.1249475	0.0257424	0.0443605
MGAT5	NC_000002	135011830	135206468	5.100972	8.5290436	5.4261801	8.5258852
FAM12CP	NC_000002	135076967	135077773	0	0	0	0
LOC151162	NC_000002	135206960	135212192	0	0	0	0
TMEM163	NC_000002	135213330	135476571	0.0464324	0.1193434	0.0413075	0.0485339
LOC100288088	NC_000002	135530571	135574162	0	0	0	0
ACMSD	NC_000002	135596186	135659604	0.1755121	0.1082669	0.0624562	0.2201469
CCNT2	NC_000002	135676393	135714584	3.924804	3.5528661	3.9940663	4.7027966

YSK4	NC_000002	135722273	135782248	0.0316554	0.0108484	0.0657102	0.0073529
RAB3GAP1	NC_000002	135809853	135927559	3.7438551	3.6112123	4.116518	5.6134618
ZRANB3	NC_000002	135957571	136288806	0.915093	0.8308468	0.7718016	0.9523691
R3HDM1	NC_000002	136289083	136482839	0.7376721	1.2251148	2.1286172	4.8438316
UBXN4	NC_000002	136499189	136542633	11.507123	9.0839575	13.441614	21.309239
LCT	NC_000002	136545415	136594750	0.0280193	0	0.018695	0.0341687
MCM6	NC_000002	136597196	136634011	10.181555	11.925238	14.738458	15.341015
LOC391448	NC_000002	136655519	136664130	0	0	0	0
DARS	NC_000002	136664254	136743222	16.220342	12.414733	18.151238	21.036469
LOC100131316	NC_000002	136738592	136743709	0.2143816	0	0.04768	0.1120422
CXCR4	NC_000002	136871919	136875725	0	0.0225354	0.0390001	0.0305485
LOC389053	NC_000002	136956684	136958073	0	0	0	0
UBBP1	NC_000002	137086870	137087572	0	0	0	0
THSD7B	NC_000002	137748462	138435287	0.0601413	0.0463737	0.0267517	0.0628632
HNMT	NC_000002	138721808	138773934	0.8625551	1.1139039	2.5360077	1.2827558
RPL15P5	NC_000002	139036108	139037021	0	0	0	0
LOC440917	NC_000002	139044689	139046450	0	0	0	0
LOC647002	NC_000002	139064889	139065546	0	0	0	0
LOC100129375	NC_000002	139169636	139171099	0	0	0	0
SPOPL	NC_000002	139259350	139330805	1.2263197	1.8517788	2.6524159	3.092397
NXPH2	NC_000002	139426727	139537811	0.1888401	0.0705991	0.5039921	0.5622536
LOC647012	NC_000002	139654896	139656744	0	0	0	0
LOC129560	NC_000002	139659003	139660317	0	0	0	0
MRPS18BP2	NC_000002	140426104	140426879	0	0	0	0
RPL9P13	NC_000002	140490277	140490857	0	0	0	0
LOC100288227	NC_000002	140978246	140980784	0	0	0	0
LRP1B	NC_000002	140988996	142889270	0.114317	0.0163995	0.0709532	0.1352378
RPS16P3	NC_000002	141965651	141966004	0	0	0	0
LOC100289623	NC_000002	143300976	143301544	0	0	0	0
LOC100129955	NC_000002	143611592	143613688	0	0	0	0
KYNU	NC_000002	143635195	143799885	4.3537927	0.3280952	15.128	0.5718338
LOC100288300	NC_000002	143852307	143854121	0	0	0	0
LOC100288333	NC_000002	143857499	143858181	0	0	0	0
ARHGAP15	NC_000002	143886899	144525921	0.0499696	0.025687	0	0
GTDC1	NC_000002	144703581	145061211	2.1195347	2.2946631	2.3141416	2.4168702
ZEB2	NC_000002	145145583	145277916	2.6005339	1.0726995	2.2009961	3.690408
LOC100289660	NC_000002	145161562	145162940	0.0644402	0	0	0
LOC100131409	NC_000002	145425384	145568842	0.1259262	0	0.0560137	0.0877504
SGCEP	NC_000002	145512076	145513709	0	0	0	0
RPL6P5	NC_000002	146094801	146095631	0	0	0	0
LOC727713	NC_000002	146583104	146585410	0	0	0	0
RNU7-2P	NC_000002	146902625	146902885	0	0	0	0
RPL17P12	NC_000002	146951864	146952389	0	0	0	0
PABPCP2	NC_000002	147344625	147348558	0	0	0	0
RPL26P14	NC_000002	148559416	148568133	0	0	0	0
ACVR2A	NC_000002	148602570	148688393	0.8967317	0.8960858	1.1705412	0.759198
ORC4L	NC_000002	148691732	148779136	2.9917108	2.9988984	4.205184	5.4932985
LOC100130682	NC_000002	149053125	149055399	0	0	0	0
LOC641381	NC_000002	149088855	149089707	0	0	0	0
MBD5	NC_000002	149216038	149271046	1.0757428	1.0719466	1.6416413	2.150834
RPS29P8	NC_000002	149352697	149352984	0	0	0	0
EPC2	NC_000002	149402560	149545136	2.5709897	2.3578265	2.1046753	3.7934139
UBBP3	NC_000002	149620995	149621370	0	0	0	0
RPS20P13	NC_000002	149627550	149628075	0	0	0	0
KIF5C (NC_000002 1496	NC_000002	149632819	149686923	0.0666893	0.0685636	0	0.0464717
KIF5C (NC_000002 1497	NC_000002	149793795	149883269	0.2382778	0.1513078	0.0124693	0.0244179
LYPD6B	NC_000002	149894981	150071772	0.2793912	0.0287243	0	0.0584073
LOC151276	NC_000002	149924982	149925441	0	0	0	0
FAM8A3P	NC_000002	150174907	150181365	0	0	0	0
LYPD6	NC_000002	150187113	150330138	1.6393888	1.9966267	0.4150961	0.2724164
RPL17P13	NC_000002	150296411	150296941	0	0	0	0
MMADHC	NC_000002	150426147	150444330	24.650156	29.587739	25.820174	31.008488
LOC642340	NC_000002	150443872	150704748	0.204173	0.1574335	0.090819	0.2489828
LOC100289521	NC_000002	150714722	150718540	0	0	0	0
TRNAE38P	NC_000002	151219277	151219349	0	0	0	0
RND3	NC_000002	151324709	151344180	43.473204	29.639503	73.546557	37.907276

FABP5L10	NC_000002	152042675	152043305	0	0	0	0
RBM43	NC_000002	152104728	152118389	2.1558663	1.1890369	1.9878425	1.2049646
NMI	NC_000002	152126982	152146430	7.636711	4.4297453	7.6661893	3.9756434
TNFAIP6	NC_000002	152214105	152236560	1.9457053	1.4288497	1.5386252	0.1506494
RIF1	NC_000002	152266428	152332419	4.1416084	5.0503218	5.6666518	7.2771988
NEB	NC_000002	152341853	152590989	0.0426227	0.0241013	0.0379183	0.0950437
ARL5A	NC_000002	152657480	152685009	3.3006245	4.1775755	5.3174663	7.1029617
CACNB4	NC_000002	152694136	152955544	2.1356576	0.2513128	0.1716813	0.1972328
LOC100127920	NC_000002	152954848	152965816	0.1462504	0	0	0.1019131
STAM2	NC_000002	152973315	153032506	5.3653691	5.2546212	4.4028481	6.4945585
RPL30P2	NC_000002	152988078	152988398	0	0	0	0
FMNL2	NC_000002	153191751	153506348	1.3243593	2.2044637	1.7813061	2.4170308
NUDCP1	NC_000002	153246099	153247363	0	0	0	0
PRPF40A	NC_000002	153508107	153573975	3.9464671	5.5939068	7.1775818	8.6325706
ARL6IP6	NC_000002	153574423	153617767	3.925625	3.5053315	4.9254584	4.5555869
LOC100131393	NC_000002	153727254	153735177	0	0	0	0
RPL23AP29	NC_000002	154227140	154227578	0	0	0	0
LOC642635	NC_000002	154276978	154300006	0	0	0	0
RPRM	NC_000002	154333852	154335322	0	0.0921483	0.0265789	0.0208191
LOC100131207	NC_000002	154617910	154619159	0	0	0	0
GALNT13	NC_000002	154728426	155310489	0.0387961	0.0239319	0.0276113	0.0162208
KCNJ3	NC_000002	155555093	155713014	0	0	0	0.0423874
LOC642721	NC_000002	155797020	155797579	0	0	0	0
ATP5AP2	NC_000002	156126143	156126549	0	0	0	0
LOC100286884	NC_000002	156168139	156169945	0	0	0	0
LOC100128759	NC_000002	156889758	156890601	0	0	0	0
NR4A2	NC_000002	157180944	157189287	0.4854095	0.2303317	0.5314877	0.5030425
GPD2	NC_000002	157291965	157442915	9.4536895	7.1500623	9.8776141	9.2844879
RPLP0P7	NC_000002	157634204	157634890	0	0	0	0
CDK7PS	NC_000002	158066842	158067986	0	0	0	0
GALNT5	NC_000002	158114340	158167913	22.753141	25.513975	7.2800641	9.8300871
ERMN	NC_000002	158175125	158184146	0.0223257	0.0459064	0.049654	0
LOC728066	NC_000002	158234999	158237021	0	0	0	0
CYTIP	NC_000002	158271131	158300604	0.2784051	0.2248948	0.0707648	0.235576
ACVR1C	NC_000002	158383279	158485399	0.1065177	0.1095114	0.1033757	0.1180865
ACVR1	NC_000002	158592958	158732374	7.6259785	9.0163537	5.879711	6.9181521
LOC100130766	NC_000002	158818129	158819400	0	0	0	0
UPP2	NC_000002	158851691	158992666	0.0170408	0.0175197	0.0303199	0.0356241
CCDC148	NC_000002	159028478	159313214	0.1809397	0.0413389	0.286167	0.1961337
LOC100128061	NC_000002	159225572	159281818	0	0	0	0
PKP4	NC_000002	159313476	159537941	5.2745536	7.4994015	7.7922615	13.548057
RPL7AP22	NC_000002	159404916	159405786	0	0	0	0
DAPL1	NC_000002	159651829	159672497	0	0	0	0.0562957
OR7E89P	NC_000002	159710016	159711184	0	0	0	0
OR7E28P	NC_000002	159718757	159719903	0	0	0	0
OR7E90P	NC_000002	159731105	159732278	0	0	0	0
TRNAE36P	NC_000002	159738171	159738242	0	0	0	0
TANC1	NC_000002	159825146	160089170	0.9308337	1.4625771	1.1666251	1.5461341
BTF3L4P	NC_000002	159860162	159861230	0	0	0	0
WDSUB1	NC_000002	160092304	160143236	1.9617842	1.381152	2.6747971	3.4622012
BAZ2B	NC_000002	160175490	160473059	0.6259048	0.5880222	0.4656186	0.8159114
LOC643072	NC_000002	160465681	160473759	0	0	0	0
RPS3AP13	NC_000002	160546182	160546855	0	0	0	0
MARCH7	NC_000002	160569010	160625084	8.8580779	9.1591498	8.8273915	8.4509828
CD302	NC_000002	160625139	160654766	1.3143603	1.6465427	2.4073646	1.9626398
LY75	NC_000002	160659866	160761262	0.5202476	0.0130456	0.6152208	0.0132633
PLA2R1	NC_000002	160798012	160919121	0.5098055	0.5626729	0.6069394	0.188075
ITGB6	NC_000002	160958233	161056590	0.0366694	0	0	0.0255527
RBMS1	NC_000002	161128662	161350318	18.559566	19.935094	11.019566	11.296764
LOC100131736	NC_000002	161905117	161947218	0	0	0	0
TANK	NC_000002	161993466	162092682	5.1965162	3.5550896	5.0354227	5.9365149
PSMD14	NC_000002	162164786	162268226	27.393971	37.2796	37.38776	51.089083
TBR1	NC_000002	162272620	162281573	0.0275883	0.0425456	0.0245434	0
LOC100130897	NC_000002	162279170	162283846	0	0.0728177	0.0315049	0.1233879
AHCTF1P	NC_000002	162356835	162364196	0	0	0	0
LOC100132762	NC_000002	162424991	162426695	0	0	0	0

KRT18P46	NC_000002	162436539	162437918	0	0	0	0
SLC4A10	NC_000002	162480845	162841786	0.1418956	0.0648372	0.0631171	0.0933853
DPP4	NC_000002	162848755	162931052	11.494153	4.2402885	11.86962	8.95974
TIMM8AP	NC_000002	162933024	162934196	0	0	0	0
LOC100132597	NC_000002	162944769	162946786	0	0	0	0
GCG	NC_000002	162999388	163008757	0.0389612	0	0.1386438	0
FAP	NC_000002	163027200	163100045	12.362416	10.743248	6.9897446	8.460398
IFIH1	NC_000002	163123589	163175039	5.1129483	1.0037821	2.1943094	0.367033
GCA	NC_000002	163200583	163219149	1.117097	0.2100902	1.660374	0.3227669
KCNH7	NC_000002	163227917	163695240	0.0203747	0.0209473	0.0090629	0.0354948
FIGN	NC_000002	164464118	164592513	0.8624901	0.378604	1.879442	3.3292315
LOC100129745	NC_000002	165070040	165071010	0	0	0	0
GRB14	NC_000002	165349323	165478360	0.1288804	0.1703605	0.0491381	0.089809
COBLL1	NC_000002	165541256	165697928	1.6693722	2.3220395	1.6677816	2.5256349
SLC38A11	NC_000002	165754812	165812035	0	0	0.0241194	0.0188926
SCN3A	NC_000002	165944030	166060577	0.4052945	0.7059141	0.0593866	0.0631304
SCN2A	NC_000002	166095912	166248820	0.4160044	0.3981999	0.0978396	0.0333205
CSRNP3	NC_000002	166428890	166537212	0.0448757	0.0461369	0.0133075	0.0312711
GALNT3	NC_000002	166604313	166650803	0.403565	0.2074536	0.0359023	0.056244
TTC21B	NC_000002	166730453	166810348	1.8581054	2.5563322	2.172087	3.0212043
SCN1A	NC_000002	166845670	166930149	0.0868113	0.0334692	0.1834207	0.049151
SCN9A	NC_000002	167051695	167168314	0.1763684	0.9209417	0.0784511	0.0582161
SCN7A	NC_000002	167261539	167343478	0.0153477	0.0157791	0	0.0267373
XIRP2	NC_000002	167759975	168116263	0.0034868	0.0286788	0.01551	0.0291573
LOC643496	NC_000002	168149687	168414843	0	0	0	0
LOC401018	NC_000002	168570250	168586777	0	0	0	0
B3GALT1	NC_000002	168675182	168727366	0.0202713	0.2500926	0.522984	0.7910485
STK39	NC_000002	168810530	169104105	6.2284605	7.4661638	7.786076	8.4279216
PHF5GP	NC_000002	169087966	169088734	0	0	0	0
LASS6	NC_000002	169312835	169631152	0.926852	0.7218949	1.5241742	2.4366815
NOSTRIN	NC_000002	169659107	169721565	0.0796164	0.2728466	0.0944386	0.1479463
SPC25	NC_000002	169727401	169746944	1.1427846	2.3162368	2.0333058	3.5038865
G6PC2	NC_000002	169757750	169766510	0	0	0.0126899	0.0298197
ABCB11	NC_000002	169779449	169887833	0.0092038	0.018925	0	0.0128272
DHRS9	NC_000002	169921299	169952677	7.6970108	9.1614964	5.6378258	4.0353776
LOC100287279	NC_000002	169967484	169972190	0	0	0	0
LRP2	NC_000002	169983619	170219122	0.0223442	0.0114861	0.0248475	0.0233555
BBS5	NC_000002	170336006	170363165	0.598598	0.6726702	0.9164459	1.0961708
KBTBD10	NC_000002	170366212	170382772	0.4280672	0.1833742	0.3173504	0.1615761
FASTKD1	NC_000002	170386259	170430424	1.168836	1.6823607	1.7677091	2.4332932
PPIG	NC_000002	170440850	170494254	0.4547489	0.651202	0.6646297	1.0185658
C2orf77	NC_000002	170501935	170550931	0	0.0208314	0.0180256	0.0423581
PHOSPHO2	NC_000002	170550975	170558218	0	0	0.0695068	0.0816663
KLHL23	NC_000002	170551001	170606438	0.5565583	0.7969935	1.8036869	4.3908125
AF357534	NC_000002	170622281	170623130	0	0	0	0
LOC100128621	NC_000002	170646567	170646885	0	0	0	0
SSB	NC_000002	170655389	170668575	1.9794091	2.7225543	2.0940875	3.9888764
METTL5	NC_000002	170668267	170681353	12.356052	12.510846	24.691008	19.372926
UBR3	NC_000002	170684018	170940641	2.02753	2.1356328	1.9413625	3.5802812
MYO3B	NC_000002	171034655	171510991	0.0158974	0.0081721	0.0353568	0.0443116
HMGB1L4	NC_000002	171458173	171458738	0	0	0	0
LOC440925	NC_000002	171568949	171571071	0	0	0	0
SP5	NC_000002	171571857	171574498	0.0225723	0.0464134	0.1004046	0.1730219
LOC728350	NC_000002	171608219	171609588	0	0	0	0
LOC285141	NC_000002	171627192	171655481	0.0414215	0.0425857	0.1105492	0.2886416
LOC100289451	NC_000002	171668301	171670646	0	0.0192598	0	0
GAD1	NC_000002	171673200	171717661	0.0478869	0.0246164	0.0106504	0.0250271
GORASP2	NC_000002	171785711	171823640	7.2631532	8.0277833	25.001164	26.139602
TLK1	NC_000002	171847333	172017410	4.0034725	3.1987567	2.9346518	4.0018179
METTL8	NC_000002	172173912	172291312	0.6212719	0.699829	0.8170358	0.9750269
RPS26P20	NC_000002	172231354	172232013	0	0	0	0
C2orf37	NC_000002	172290859	172341562	2.2089164	1.5534259	1.7058275	1.9507994
MRPS29P2	NC_000002	172347927	172348441	0	0	0	0
RPS15P4	NC_000002	172373749	172374247	0	0	0	0
CYBRD1	NC_000002	172378866	172414643	39.056129	37.053086	37.015712	29.91496
RPL21P38	NC_000002	172443560	172444116	0	0	0	0

DYNC1I2	NC_000002	172543982	172604919	18.308948	22.539606	23.245171	26.434849
SLC25A12	NC_000002	172640880	172750733	4.6331865	6.4175747	5.3013648	4.1213819
HAT1	NC_000002	172778958	172848600	7.0712091	14.320422	11.180907	13.815595
MAP1D	NC_000002	172864804	172945587	0.6875864	0.3829102	0.6626706	1.497305
DLX1	NC_000002	172950208	172954402	0.2581936	0.9669969	0.1148481	0.1799196
DLX2	NC_000002	172964166	172967478	0.1716728	0.529493	0.0848471	0.0398761
LOC100287337	NC_000002	172964722	172967628	0.1842141	0.1893915	0.0468234	0.1100295
LOC100287375	NC_000002	173292283	173337493	0.1655301	0.0850912	0.0736301	0.1153479
ITGA6	NC_000002	173292314	173371181	3.789684	4.2072669	13.108788	13.23564
LOC100129169	NC_000002	173417343	173520045	0.2999879	0.3598223	0.3113572	0.9755365
PDK1	NC_000002	173420779	173463862	1.9784388	0.8590376	2.477774	1.7935899
LOC100287452	NC_000002	173538937	173550677	0	0	0	0
LOC91149	NC_000002	173587917	173600934	0	0	0	0
RAPGEF4	NC_000002	173600525	173917621	0.0092737	0.0095344	0.0082502	0.0193869
ZAK	NC_000002	173940565	174132737	5.8573868	7.2181619	7.4396017	9.3654537
RPS2P18	NC_000002	174162338	174163282	0	0	0	0
LOC100289479	NC_000002	174202947	174203189	0	0	0	0
CDCA7	NC_000002	174219561	174233718	4.2384238	4.0681141	4.8419761	5.8525123
LOC100130171	NC_000002	174296077	174296997	0	0	0	0
LOC100131562	NC_000002	174342724	174343128	0	0	0	0
LOC643997	NC_000002	174350593	174351090	0.6177462	1.0887567	0.471055	0.245983
LOC100129456	NC_000002	174439829	174441069	0	0	0	0
RPL5P7	NC_000002	174736300	174737295	0	0	0	0
SP3	NC_000002	174773258	174830063	10.716059	13.352295	16.026604	15.985079
LOC100128905	NC_000002	174890008	174891891	0	0.0239827	0.0207524	0.0162552
RPSAP24	NC_000002	174907661	174908682	0	0	0	0
LOC100129313	NC_000002	174932215	175112989	0	0	0	0
OLA1	NC_000002	174937175	175113365	7.5466661	8.4848537	10.671948	15.818635
LOC644158	NC_000002	175164052	175165684	0	0	0	0
LOC402112	NC_000002	175174743	175175702	0	0	0	0
SP9	NC_000002	175199821	175202268	0.0604099	0.3105388	0	0.0420961
CIR	NC_000002	175212878	175260443	4.4610029	3.4775848	5.1243324	4.1675796
SCRN3	NC_000002	175260467	175293728	2.1012526	1.5744621	3.1049931	3.5985473
GPR155	NC_000002	175296375	175351811	0.2293565	0.1995253	0.099405	0.2049035
LOC442058	NC_000002	175366680	175367043	0	0	0	0
WIPF1	NC_000002	175424302	175547627	3.003891	3.4431843	3.1122027	2.5417804
RNU7-44P	NC_000002	175430844	175431105	0	0	0	0
LOC100130325	NC_000002	175431852	175452481	0.2205683	0	0.0490559	0.0384252
LOC100133109	NC_000002	175584462	175589200	0.4747494	1.8826417	2.41343	1.7959034
LOC440926	NC_000002	175584528	175585586	0	0	0	0
CHRNA1	NC_000002	175612320	175629200	0.0640334	0.0219443	0.0189886	0.0148737
CHN1	NC_000002	175664042	175870170	1.6853304	3.1155222	1.052405	0.9372658
RPL21P31	NC_000002	175898464	175898962	0	0	0	0
ATF2	NC_000002	175939006	176032897	7.140807	8.7969579	11.260692	12.157105
ATP5G3	NC_000002	176040986	176046490	11.111381	12.542069	26.751694	30.960582
RPS15AP14	NC_000002	176048937	176049383	0	0	0	0
LOC100287682	NC_000002	176707626	176708030	0	0	0	0
KIAA1715	NC_000002	176790410	176867018	5.1380934	5.4235757	6.6259014	8.8607627
EVX2	NC_000002	176944835	176948690	0.0815366	0.0558855	0	0.0189393
HOXD13	NC_000002	176957532	176960666	0.0377724	0.3495063	0.0336034	0.0131607
HOXD12	NC_000002	176964530	176965488	0	0.0555761	0	0.1130069
HOXD11	NC_000002	176972084	176974316	0	0	0.0267242	0.0209329
HOXD10	NC_000002	176981492	176984670	0	0	0.1300362	0.0509283
HOXD9	NC_000002	176987413	176989645	0.3497207	0.1677898	0.7259495	1.0722769
LOC100129455	NC_000002	176993381	176994359	0	0	0	0
HOXD8	NC_000002	176994477	176996728	1.8711328	2.2603724	3.3500309	2.8685357
HOXD4	NC_000002	177016113	177017951	0.4740179	1.5316407	0.1204856	0.8257864
HOXD3	NC_000002	177028805	177037826	0.1529299	0.4127235	0.1700634	0.3729868
HOXD1	NC_000002	177053307	177055635	0.3782886	0.3889204	0.4355172	0.1705689
RPLP1P4	NC_000002	177065579	177066113	0	0	0	0
RPSAP25	NC_000002	177106763	177107798	0	0	0	0
MTX2	NC_000002	177134137	177202753	6.322084	6.7861003	10.207983	9.9365892
LOC375295	NC_000002	177494309	177503962	3.2699577	3.41565	3.4908541	4.3385244
FUCA1P	NC_000002	177669183	177670667	0	0	0	0
RPL29P8	NC_000002	177671762	177674476	0	0	0	0
LOC391465	NC_000002	178042409	178042989	0	0	0	0

HNRNPA3	NC_000002	178077422	178088685	2.5878608	3.3374104	4.3351984	8.7951613
NFE2L2	NC_000002	178095033	178129859	9.7238752	9.3478236	10.067038	10.663693
LOC100286920	NC_000002	178096518	178129859	0.0576748	0.296479	0.1539274	0.1607605
LOC100130691	NC_000002	178148236	178257419	0	0	0	0
AGPS	NC_000002	178257471	178408564	1.5824804	2.4758028	2.4993878	4.0353721
TTC30B	NC_000002	178414880	178417524	1.1132444	1.554514	1.3599153	1.1925756
LOC100128904	NC_000002	178467817	178483699	0.8443037	1.3248925	0.4348566	0.7122065
TTC30A	NC_000002	178479026	178483694	0.0188255	0	0.0251216	0.0065592
PDE11A	NC_000002	178487977	178973066	0.0404018	0.0323068	0.0399362	0.0312818
LOC728664	NC_000002	178859544	178863604	0	0	0	0
LOC100286990	NC_000002	178882720	178883192	0	0	0	0
HCP9	NC_000002	178957227	178969197	0	0	0	0
RBM45	NC_000002	178977182	178994383	1.4526306	1.189703	1.1608801	1.9730314
OSBPL6	NC_000002	179059374	179260877	1.3925712	1.6459024	2.0387703	0.8175803
PRKRA	NC_000002	179296141	179315958	2.589951	3.5988621	7.74031	8.6432092
DFNB59	NC_000002	179316163	179326113	0.3165884	0.1775379	0.1280208	0.3008338
NUDCP2	NC_000002	179319446	179320385	0	0	0	0
FKBP7	NC_000002	179328391	179343355	1.5133689	1.3069579	1.1713114	0.9596639
PLEKHA3	NC_000002	179345199	179369783	3.1671195	3.2378389	3.8939276	4.6247286
TTN	NC_000002	179390716	179672150	0.1165704	0.0879683	0.1033551	0.3022228
CCDC141	NC_000002	179700492	179749678	0	0.0119343	0.0103269	0.0242669
RPS6P2	NC_000002	179866845	179867499	0	0	0	0
SESTD1	NC_000002	179966419	180129350	1.9265209	1.6130751	3.0647881	3.5115433
LOC644776	NC_000002	180264455	180265602	0	0	0	0
ZNF385B	NC_000002	180306709	180726232	0.011541	0.0355962	0.2053444	0.096507
LOC100131482	NC_000002	180678978	180679451	0	0	0	0
LOC100287810	NC_000002	180712376	180726305	0	0	0	0
CWC22	NC_000002	180809604	180871780	0.6299381	0.6338629	0.9896609	1.3449169
LOC729009	NC_000002	181737420	181770051	0	0	0	0
RPL27AP3	NC_000002	181803594	181804025	0	0	0	0
UBE2E3	NC_000002	181845112	181928150	13.815984	15.502809	11.167457	26.06258
ITGA4	NC_000002	182321619	182402474	3.7502684	5.9135133	4.5577399	4.818811
CERKL	NC_000002	182401405	182521751	0.3295088	0.3387696	0.4519244	0.6984118
NEUROD1	NC_000002	182541194	182545381	0	0	0.029732	0.0232889
LOC442060	NC_000002	182559151	182559887	0	0	0	0
SSFA2	NC_000002	182756472	182795465	9.5900638	15.143653	12.114114	17.91321
KRT18P29	NC_000002	182825826	182827205	0	0	0	0
PPP1R1C	NC_000002	182850551	182982419	0	0	0.2313465	0.3322225
PDE1A	NC_000002	183007183	183387465	1.6992286	2.3822532	0.8520383	0.6889252
DNAJC10	NC_000002	183580999	183643255	16.675802	19.375091	16.429743	16.798765
RPL31P15	NC_000002	183652828	183653162	0	0	0	0
FRZB	NC_000002	183698737	183731498	0.4319237	0.2220315	0.1344879	0
LOC100287864	NC_000002	183723501	183732250	0	0	0.02579	0.0202011
NCKAP1	NC_000002	183789605	183903229	13.017481	18.62157	15.075184	18.73063
KRT8P10	NC_000002	183935571	183937290	0	0	0	0
DUSP19	NC_000002	183943287	183964736	0.3104727	0.1511993	0.2253253	0.2960576
NUP35	NC_000002	183989083	184026408	3.6601099	5.8381493	4.5011282	4.7259457
LOC129402	NC_000002	184124678	184125994	0	0	0	0
LOC644877	NC_000002	184471961	184472916	0	0	0	0
ZNF804A	NC_000002	185463093	185804214	0.2626575	0.8101186	0.5841685	0.568701
RPL23AP33	NC_000002	185766835	185767289	0	0	0	0
ELF2P4	NC_000002	186410450	186412545	0	0	0	0
FLJ44048	NC_000002	186669761	186698017	0.0260511	0	0.0077253	0.0363069
RPL21P32	NC_000002	186821314	186821779	0	0	0	0
RPL23AP35	NC_000002	187126144	187126642	0	0	0	0
LOC100131051	NC_000002	187229325	187229860	0	0	0	0
ZC3H15	NC_000002	187350885	187374088	3.0479561	4.8274675	4.9284185	6.6731811
DPRXP1	NC_000002	187353283	187353896	0	0	0	0
ITGAV	NC_000002	187454790	187545628	14.71647	15.712968	21.87815	14.910989
FAM171B	NC_000002	187558789	187628514	7.33912	8.5488978	2.9237988	4.5459476
ZSWIM2	NC_000002	187692207	187713897	0.0179674	0.0184724	0	0.0125204
IMPDH1P7	NC_000002	187865866	187868107	0	0	0	0
CALCRL	NC_000002	188207846	188313021	0.3076992	0.0903849	0.203348	0.1286502
GAPDHL3	NC_000002	188280157	188281186	0	0	0	0
TFPI	NC_000002	188328957	188419219	7.2787269	1.1235853	4.6913303	4.9759882
LOC344328	NC_000002	188690068	188691419	0	0	0	0

LOC729141	NC_000002	189084787	189093480	0	0	0	0
GULP1	NC_000002	189157390	189460653	2.4196361	1.7020696	1.3481919	1.7304698
DIRC1	NC_000002	189598465	189654831	0.0302257	0.0310752	0.1882276	0.0631875
COL3A1	NC_000002	189839099	189877472	9.1178572	10.394651	2.3501269	1.8408397
COL5A2	NC_000002	189896641	190044605	6.7285821	10.014676	4.2877561	5.1085656
KRT18P19	NC_000002	190175931	190177311	0	0	0	0
WDR75	NC_000002	190306159	190340264	1.1997834	1.4869631	0.8626613	1.3628873
SLC40A1	NC_000002	190425316	190445537	0.8514561	0.6464391	0.3029916	0.1916907
ASNSD1	NC_000002	190526125	190535557	5.2823249	6.1439187	4.0031097	6.8289389
ANKAR	NC_000002	190541430	190611370	0.2994769	0.2749051	0.2473927	0.2086874
OSGEPL1	NC_000002	190611386	190627924	1.5750014	1.7753409	1.603743	2.684305
ORMDL1	NC_000002	190634993	190649097	4.9144677	5.3839067	6.5401519	8.5755273
PMS1	NC_000002	190648811	190742355	1.3480287	1.3474176	2.254134	2.6789158
LOC100287965	NC_000002	190781881	190782469	0	0	0	0
LOC653447	NC_000002	190787896	190789604	0	0	0	0
RNF11P1	NC_000002	190793087	190795768	0	0	0	0
MSTN	NC_000002	190920426	190927455	0	0.0160282	0	0
C2orf88	NC_000002	191002486	191068210	0.2057501	0.8567078	0.0457603	0.1362062
LOC100288000	NC_000002	191044950	191068494	0	0.102057	0	0.0188654
HIBCH	NC_000002	191069360	191184559	1.043357	1.4389618	0.7244482	1.4363724
INPP1	NC_000002	191208196	191236391	7.0097734	8.1885777	5.3684591	4.1626048
MFSD6	NC_000002	191273081	191367041	2.5560885	1.2339015	4.8413496	6.677845
TMEM194B	NC_000002	191371619	191399468	1.377536	1.0311307	1.483493	1.4062014
NAB1	NC_000002	191513848	191557492	6.3921125	3.9834997	8.7836106	6.1469044
GLS	NC_000002	191745547	191829776	5.5416512	6.6556463	3.4879571	5.4218318
STAT1	NC_000002	191833762	191878976	199.45957	123.34487	180.80598	39.853104
STAT4	NC_000002	191894306	192015925	2.6900584	0.7364191	1.1611736	0.2994827
MYO1B	NC_000002	192110107	192290115	29.896874	39.587577	17.680234	20.556837
OBFC2A	NC_000002	192542798	192553248	6.7366736	7.6655655	3.7076153	7.7656226
SDPR	NC_000002	192699036	192711981	0.4502613	0.1543053	2.014963	1.25504
DNAJB1P	NC_000002	192744718	192746971	0	0	0	0
TMEFF2	NC_000002	192814743	193059644	0.8969027	2.1682052	1.2292119	2.4830982
PCGEM1	NC_000002	193614571	193641625	0	0	0	0
RPS17P8	NC_000002	193638808	193639150	0	0	0	0
LOC645314	NC_000002	194141388	194142327	0	0	0	0
LOC343981	NC_000002	194915095	194994990	0	0	0	0
LOC100287191	NC_000002	195052103	195053036	0	0	0	0
LOC391470	NC_000002	196447514	196448755	0	0	0	0
SLC39A10	NC_000002	196521532	196602426	4.9237928	6.6895969	12.100785	16.362831
DNAH7	NC_000002	196602427	196933536	0.0319134	0.0473926	0.0473183	0.0617736
STK17B	NC_000002	196998307	197036336	5.9955756	3.7290993	6.5346789	6.8151463
HECW2	NC_000002	197063977	197457335	1.7513301	1.5396019	0.5701493	0.6853676
CCDC150	NC_000002	197504356	197597530	0.3220088	0.5640262	0.3819572	0.5152625
LOC100130452	NC_000002	197565358	197577685	0	0	0	0
GTF3C3	NC_000002	197629100	197664425	3.6798043	4.8597532	5.4560932	5.5558408
C2orf66	NC_000002	197670009	197675000	0	0	0.0846268	0.0441917
PGAP1	NC_000002	197697728	197791454	0.4626962	0.8090972	1.0695276	1.0361697
ANKRD44	NC_000002	197859250	198062762	0.6080737	1.1767788	0.7000649	0.9720865
LOC729342	NC_000002	198244101	198245616	0	0	0	0
SF3B1	NC_000002	198256698	198299771	12.765539	18.863664	12.921183	18.624167
COQ10B	NC_000002	198318231	198339851	5.8107844	6.2716576	6.8727737	4.4525532
HSPD1	NC_000002	198351308	198364998	9.7783012	17.528044	20.158679	34.119836
HSPE1	NC_000002	198364721	198368187	9.453712	16.831173	19.692373	24.519184
MOBKL3	NC_000002	198380320	198417515	3.4390385	4.7754815	3.8541299	3.724367
RFTN2	NC_000002	198435527	198540584	0.3247059	0.3918894	0.2763079	0.0885396
MARS2	NC_000002	198570087	198573114	0.8563229	1.1788272	0.9167527	1.3350343
BOLL	NC_000002	198591603	198650938	0.0858923	0.2207658	0.0764122	0.0897798
PLCL1	NC_000002	198669533	199013120	0.2882138	2.0831775	0.108777	0.3043013
SATB2	NC_000002	200134224	200325255	0.8177224	1.5383106	1.5710225	2.2307908
LOC100287287	NC_000002	200213394	200233407	0.2979541	0.8424024	0.8614718	0.363346
LOC100288092	NC_000002	200324263	200326146	0.3279719	0.2075013	0.5835458	4.1489502
FLJ32063	NC_000002	200332821	200337481	0	0	0	0
LOC729399	NC_000002	200339261	200526115	0	0	0	0
LOC348751	NC_000002	200625259	200716735	0.6895263	0.2984866	0.8071339	0.2528892
C2orf69	NC_000002	200775979	200792996	2.8057079	3.236043	1.9087331	3.5241612
C2orf60	NC_000002	200793634	200820459	0.875691	0.9339586	1.2741292	0.6330282

C2orf47	NC_000002	200820040	200828840	6.340935	10.184291	10.112336	5.0702092
LOC26010	NC_000002	201170604	201346986	5.3763401	5.9142981	11.347429	8.4972816
KCTD18	NC_000002	201353681	201374792	1.5097862	1.4446394	1.4894311	1.8749925
SGOL2	NC_000002	201390942	201448505	2.8579052	4.6032223	2.5895546	5.4950709
AOX1	NC_000002	201450731	201536218	4.7475492	2.4084383	5.0635072	10.092431
AOX2P	NC_000002	201635395	201658946	0	0	0	0
BZW1	NC_000002	201676647	201688560	74.341308	59.426347	41.039868	47.447765
LOC391472	NC_000002	201692936	201710322	0	0	0	0
CLK1	NC_000002	201717733	201729422	6.9404203	5.6269737	9.8210101	8.9099407
PPIL3	NC_000002	201735679	201753999	3.3865538	4.193906	5.112583	4.0225322
NIF3L1	NC_000002	201754050	201768655	3.0814277	6.9852501	6.3140322	7.1282044
ORC2L	NC_000002	201774894	201828410	1.9432413	3.1560338	2.4678694	3.3362572
FAM126B	NC_000002	201838441	201936392	0.7345895	0.6584102	1.0095911	0.8367453
LOC645691	NC_000002	201927757	201929488	0	0	0	0
RPL23AP30	NC_000002	201931414	201931914	0	0	0	0
NDUFB3	NC_000002	201936462	201950473	7.3344069	5.8050198	12.842644	11.276445
RPL17P10	NC_000002	201967221	201967680	0	0	0	0
CFLAR	NC_000002	201980816	202029016	5.9682784	3.1172282	7.7797625	6.0048781
LOC729172	NC_000002	201982235	201983904	0	0	0	0
RNU7-45P	NC_000002	202006527	202006787	0	0	0	0
LOC100130026	NC_000002	202027179	202027479	0	0	0	0
CASP10	NC_000002	202047621	202094110	0.1036352	0.2841277	0.055318	0.0240724
CASP8	NC_000002	202098166	202152434	0.4012392	0.3222782	0.5688947	0.5941489
ALS2CR12	NC_000002	202153147	202222101	0.1309932	0.0224458	0.0388451	0.0152136
TRAK2	NC_000002	202241930	202316319	11.062808	10.529179	6.2117628	11.795763
STRADB	NC_000002	202316392	202345574	12.851702	8.8479247	9.0172801	7.3830211
ALS2CR11	NC_000002	202352148	202483901	0.1668656	1.3724432	0.1855603	0.4360448
LOC100288230	NC_000002	202415222	202417593	0	0	0	0
ALS2CR4	NC_000002	202484907	202508224	2.1821685	3.5467073	1.9770036	3.9301368
MPP4	NC_000002	202509597	202563417	0.5721495	0.6801407	0.0318125	0.7973931
ALS2	NC_000002	202564986	202645895	0.9799054	0.953282	0.646783	0.9691882
RPS2P16	NC_000002	202627437	202628333	0	0	0	0
PFTK2	NC_000002	202671198	202758266	0.1718966	1.2370944	0	0.0998203
FZD7	NC_000002	202899310	202903160	3.4464726	5.690457	0.3451875	0.3101455
FLJ39061	NC_000002	202937978	202974436	0	0	0	0
LOC339809	NC_000002	202974476	203061773	0	0	0	0
LOC100288301	NC_000002	203043383	203044180	0.1165736	0.3595496	0.3111212	0.4061655
LOC645805	NC_000002	203064186	203066744	0	0	0	0
SUMO1	NC_000002	203070903	203103322	7.5981226	10.770634	20.355314	15.984301
RPL39P14	NC_000002	203096371	203096763	0	0	0	0
LOC100287425	NC_000002	203104292	203104435	0	0	1.0860435	0
NOP58	NC_000002	203130515	203168384	5.9939573	8.944704	8.1577618	10.44207
SNORD70	NC_000002	203141154	203141241	0	0	0	0
SNORD11B	NC_000002	203156040	203156151	0	0	0	0
SNORD11	NC_000002	203157774	203157857	0	0	0	0
LOC442063	NC_000002	203200659	203201450	0	0	0	0
BMPR2	NC_000002	203241050	203432474	2.5633434	4.147739	1.7556653	2.3672752
RPL13AP12	NC_000002	203385116	203385715	0	0	0	0
LOC100288334	NC_000002	203479576	203479770	0	0	0	0
LOC100288371	NC_000002	203480150	203481166	0	0	0	0
FAM117B	NC_000002	203500211	203634480	0.3861459	0.2729365	0.3292125	0.6783105
ICA1L	NC_000002	203637873	203736371	0.2660119	0.4260104	0.2867124	0.3279582
LOC442064	NC_000002	203637877	203639167	0	0	0	0
WDR12	NC_000002	203745323	203776949	3.1969354	4.9697782	4.4031877	7.3408451
ALS2CR8	NC_000002	203776978	203851060	0.3516445	0.2711456	0.3389021	0.3828748
NBEAL1	NC_000002	203879602	204082717	2.1348225	3.1475741	2.2056586	2.924544
RPL7P14	NC_000002	203904828	203905442	0	0	0	0
RPL23AP36	NC_000002	203939408	203939854	0	0	0	0
RPL12P16	NC_000002	204055415	204056042	0	0	0	0
CYP20A1	NC_000002	204103164	204170563	0.7951887	0.9826967	0.3894393	0.8199844
MRPL50P2	NC_000002	204180113	204180594	0	0	0	0
LOC100288412	NC_000002	204192914	204193949	0	0	0	0.0440646
ABI2	NC_000002	204193003	204296892	3.7166097	3.2841639	3.3908691	4.229825
TRNAE40P	NC_000002	204229131	204229201	0	0	0	0
RAPH1	NC_000002	204298538	204400058	3.4504109	1.5209305	3.4881605	3.0601248
LOC729532	NC_000002	204499300	204510972	0	0	0	0

LOC100287498	NC_000002	204536898	204537146	0	0	0	0
CD28	NC_000002	204571198	204602557	0	0	0	0.0080444
KRT18P39	NC_000002	204628956	204630364	0	0	0	0
LOC100131478	NC_000002	204637346	204638225	0	0	0	0
CTLA4	NC_000002	204732509	204738683	0	0	0	0.0309812
ICOS	NC_000002	204801503	204826300	0.0168191	0.0172918	0.0299254	0.0234404
LOC100132132	NC_000002	205171565	205172087	0	0	0	0
LOC100132669	NC_000002	205243818	205244593	0	0	0	0
PARD3B	NC_000002	205410516	206480537	1.3098297	0.4488809	1.4105788	2.1137171
NRP2	NC_000002	206547224	206662857	1.1785573	1.7677662	5.9717619	2.4598296
LOC100287570	NC_000002	206813815	206814874	0	0	0	0
INO80D	NC_000002	206858445	206950906	0.9575591	0.7607279	1.4520531	1.9909637
RPL27P8	NC_000002	206905113	206905601	0	0	0	0
LOC729570	NC_000002	206948291	206952228	0.3897436	0.1717275	0.1981296	0.1810592
NDUFS1	NC_000002	206987803	207024187	5.1961035	5.3553637	5.5837319	6.5784784
EEF1B2	NC_000002	207024318	207027653	44.027418	66.187578	46.459179	62.105046
SNORD51	NC_000002	207026605	207026674	0	0	0	0
SNORA41	NC_000002	207026952	207027083	0	0	0	0
GPR1	NC_000002	207040040	207082771	6.958154	5.3214274	2.0408997	1.7439534
LOC100132849	NC_000002	207100960	207126891	0	0	0	0
ZDBF2	NC_000002	207139523	207179148	1.7066433	1.0509839	3.3602481	2.6562086
LOC645978	NC_000002	207272967	207275791	0	0	0	0
LOC729366	NC_000002	207284001	207284962	0	0	0	0
ADAM23	NC_000002	207308368	207482685	1.3086679	2.8091775	1.1386398	0.1603397
LOC200726	NC_000002	207507142	207514173	0	0.0328368	0	0.0445129
DYTN	NC_000002	207516345	207583120	0	0.0220299	0	0.0298634
MDH1B	NC_000002	207602487	207630050	0.1319201	0.1550031	0.1005941	0.1181921
FASTKD2	NC_000002	207630112	207660913	2.0059884	2.3486261	2.9780579	3.263126
CPO	NC_000002	207804278	207834198	0.0727018	0.0373725	0.0323388	0
KLF7	NC_000002	207945529	208030614	9.7066869	11.192472	11.497881	11.846353
CREB1	NC_000002	208394616	208470284	1.3931846	1.8204582	2.2229519	2.7652897
FAM119A	NC_000002	208473839	208489973	0.4718915	0.549231	0.8475364	1.3339443
RPS29P9	NC_000002	208519475	208519773	0	0	0	0
LOC729406	NC_000002	208546160	208546757	0	0	0	0
CCNYL1	NC_000002	208576264	208620898	4.4251478	4.5495171	2.3280687	2.692297
FZD5	NC_000002	208627310	208634143	0.1406022	0.0550681	0.2561236	0.4852205
PLEKHM3	NC_000002	208686012	208890284	0.4033181	0.5943366	1.1441829	0.9649319
RPL9P14	NC_000002	208896015	208896716	0	0	0	0
RPL12P17	NC_000002	208921926	208922557	0	0	0	0
CRYGEP1	NC_000002	208972730	208977666	0	0	0	0
CRYGD	NC_000002	208986331	208989313	0	0	0.0553007	0.0433167
CRYGC	NC_000002	208992861	208994554	0.06987	0	0	0.0973764
LOC646091	NC_000002	209000097	209002421	0	0	0	0
CRYGB	NC_000002	209007297	209010877	0	0	0	0
CRYGA	NC_000002	209025464	209028297	0	0.0745601	0	0
C2orf80	NC_000002	209030071	209054773	0	0	0	0
RPSAP27	NC_000002	209072783	209073790	0	0	0	0
LOC100128483	NC_000002	209079448	209087349	0	0	0	0
IDH1	NC_000002	209100953	209119806	4.0759202	5.4902974	4.6500754	2.7087698
PIKFYVE	NC_000002	209130991	209223475	1.958027	2.0736658	2.1202666	2.0891936
LOC100288663	NC_000002	209225152	209226193	0	0	0	0
PTH2R	NC_000002	209271556	209359231	0.0323983	0	0	0.0225764
LOC130195	NC_000002	209937321	209940279	0	0	0	0
CRYGFP	NC_000002	210009751	210012629	0	0	0	0
LOC402116	NC_000002	210043492	210045408	0	0	0	0
MAP2	NC_000002	210288771	210598834	0.5763558	0.5111347	1.3425233	1.71075
C2orf21	NC_000002	210636717	210864024	0.0200873	0.0147514	0.0178703	0.029995
SNAI1L1	NC_000002	210673458	210675157	0	0	0	0
RPE	NC_000002	210867352	210886291	7.5113403	8.1829603	7.280025	10.504417
C2orf67	NC_000002	210885435	211036051	1.4541097	1.3529965	1.8211805	1.9020257
RPL6P6	NC_000002	210952950	210953788	0	0	0	0
ACADL	NC_000002	211052714	211090215	0.0529072	0.145051	0.0470677	0.0614464
MYL1	NC_000002	211154868	211179895	0.0381827	0.0785116	0.0339683	0.0266072
LANCL1	NC_000002	211295973	211341499	4.4710891	7.8997445	8.0230579	7.2144507
CPS1	NC_000002	211342409	211543831	0.207303	1.2420291	0.2988916	0.2739701
LOC29034	NC_000002	211482295	211484600	0	0	0	0

RPS27P10	NC_000002	212163603	212163946	0	0	0	0
ERBB4	NC_000002	212240442	213403352	0.040546	0.0265272	0.0229542	0.0333912
LOC646249	NC_000002	213696876	213698090	0	0	0	0
IKZF2	NC_000002	213864408	214016333	0.8052626	0.2946744	0.9187523	1.1286187
SPAG16	NC_000002	214149116	215275225	1.0216864	0.9696009	2.3352266	2.5520732
RPL5P8	NC_000002	215145313	215146248	0	0	0	0
VWC2L	NC_000002	215276461	215440653	0.0484011	0	0	0
LOC100128203	NC_000002	215494151	215562788	0.7990588	0.912796	0.9478198	0.7424213
BARD1	NC_000002	215593262	215674428	3.7592849	4.8181763	4.9790532	5.4624351
RPL10P6	NC_000002	215711843	215712226	0	0	0	0
ABCA12	NC_000002	215796266	216003151	0.0189228	0.0243183	0.02946	0.1219721
ATIC	NC_000002	216176692	216214479	4.7072108	8.667118	7.6710414	13.061048
FN1	NC_000002	216225177	216300791	588.39814	303.35845	180.83223	83.728015
LOC646324	NC_000002	216669428	216669704	0	0	0	0
MREG	NC_000002	216807314	216878346	0.6565562	0.9843878	0.6449334	0.8197135
LOC100288812	NC_000002	216844845	216878360	0	0.1369194	0.0592387	0.0464013
PECR	NC_000002	216903111	216946532	1.4123695	1.35526	2.8480283	2.1488265
TMEM169	NC_000002	216946589	216967506	0.0888869	0.1436051	0.1355593	0.0530914
XRCC5	NC_000002	216974020	217071016	7.8158663	10.248597	11.603884	19.258852
MARCH4	NC_000002	217122585	217236750	3.1899503	7.6050265	0.3777967	1.376399
SMARCAL1	NC_000002	217277137	217347776	6.883458	8.6828345	6.8538504	9.8885149
RPL37A	NC_000002	217363520	217366190	171.71202	254.74832	227.13824	211.82207
PSMB3P2	NC_000002	217474994	217475760	0	0	0	0
IGFBP2	NC_000002	217498127	217529159	3.196235	0.3159678	0.027341	0.192744
IGFBP5	NC_000002	217536828	217560272	3.0853223	7.3652059	0.087733	0.0098172
RPL31P14	NC_000002	217650458	217650902	0	0	0	0
TNP1	NC_000002	217724181	217724782	0	0	0	0
DIRC3	NC_000002	218148746	218621316	0	0	0	0
LOC100288848	NC_000002	218559102	218560030	0	0	0	0
LOC100128899	NC_000002	218625261	218626019	0	0	0	0
TNS1	NC_000002	218664512	218808796	5.9147922	10.179017	4.6798371	5.3972027
IL8RBP	NC_000002	218923878	218926013	0	0	0	0
RUFY4	NC_000002	218938040	218954863	0.0327483	0	0.0582676	0.068461
IL8RB	NC_000002	218990746	219001975	0.0153719	0	0	0
IL8RA	NC_000002	219027568	219031716	0.0530349	0.0363503	0	0.0246379
HMGB1L9	NC_000002	219064999	219066573	0	0	0	0
ARPC2	NC_000002	219081874	219119071	83.832558	84.696496	85.245089	95.009943
GPBAR1	NC_000002	219125738	219128582	0	0.0902315	0.0585585	0.1070265
AAMP	NC_000002	219128852	219134893	19.118584	22.478301	18.796487	26.166934
PNKD	NC_000002	219135115	219211516	4.3511933	4.2067723	4.3744794	4.8233978
LOC100288953	NC_000002	219136103	219137670	1.5154563	1.9680611	0.5676598	5.335732
TMBIM1	NC_000002	219138917	219157280	70.047756	37.373925	50.41572	54.978762
RPL19P5	NC_000002	219218765	219219348	0	0	0	0
C2orf62	NC_000002	219221579	219232817	0.0664372	0.0341522	0.0886566	0
SLC11A1	NC_000002	219246752	219261617	0.057046	0.0234597	0.0101499	0.0159008
CTDSP1	NC_000002	219264478	219270664	11.062027	13.068588	15.60642	25.075715
VIL1	NC_000002	219283838	219314248	0.0160512	0	0.0285592	0.0111851
USP37	NC_000002	219314974	219433084	2.5388421	3.3189999	5.1743915	4.9681544
RQCD1	NC_000002	219433678	219458999	21.095151	30.272879	29.757592	30.148491
PLCD4	NC_000002	219472632	219501904	1.195483	0.8193881	1.9751361	1.4578553
TRNAQ44P	NC_000002	219491132	219491207	0	0	0	0
ZNF142	NC_000002	219502639	219524355	1.2125595	1.8482142	1.5051998	3.2668502
BCS1L	NC_000002	219524379	219528166	4.7568742	6.8739631	3.8086624	5.2852316
RNF25	NC_000002	219528587	219536781	8.5306305	11.578066	7.7894573	5.7286768
STK36	NC_000002	219536762	219567440	1.4107722	1.6064802	1.08825	3.1172418
TTLL4	NC_000002	219575568	219620139	1.8909085	2.2695685	2.9575505	4.290057
CYP27A1	NC_000002	219646472	219680016	0.9391467	0.4138035	7.8092826	1.5626301
PRKAG3	NC_000002	219687106	219696512	0.0573487	0	0.051019	0.1065676
RPL23AP31	NC_000002	219706328	219706788	0	0	0	0
WNT6	NC_000002	219724546	219738954	0.0517038	0.0265785	0.0229986	0.0360293
WNT10A	NC_000002	219745255	219758651	0	0	0	0.0128947
LOC100288883	NC_000002	219748572	219757638	0.049659	0	0.044178	0.0346044
KRT8P30	NC_000002	219818123	219818705	0	0	0	0
CDK5R2	NC_000002	219824398	219826877	0.0177211	0.0364382	0.0157651	0.0370462
LOC151300	NC_000002	219841006	219842644	0	0	0	0
FEV	NC_000002	219845809	219850379	0.0233892	0.0961861	0	0.032597

CRYBA2	NC_000002	219854912	219858127	0.099318	0.1531641	0	0.1038131
CCDC108	NC_000002	219867576	219906245	0.0170496	0.0175288	0.0101119	0.0237617
IHH	NC_000002	219919146	219925189	0	0	0.0193648	0.0151683
LOC100288913	NC_000002	219919146	219925237	0	0	0	0
NHEJ1	NC_000002	219940046	220025587	1.170281	1.8906987	1.5430803	2.6794948
SLC23A3	NC_000002	220026181	220034817	0.0908772	0.1868627	0.3072183	0.4939496
C2orf24	NC_000002	220036619	220041702	7.4377191	8.300326	7.3708525	7.7817314
FAM134A	NC_000002	220042939	220050197	14.411286	14.796732	16.676205	25.135764
ZFAND2B	NC_000002	220071538	220074370	8.4279606	11.19061	10.715404	14.337579
ABCB6	NC_000002	220074494	220083672	4.8946382	4.1352239	4.920084	5.0285803
ATG9A	NC_000002	220084102	220094361	7.9905876	12.454476	14.072222	21.103812
ANKZF1	NC_000002	220094479	220101391	1.0999557	0.7881822	1.8236635	2.9382231
GLB1L	NC_000002	220101493	220110131	1.0526523	0.4328949	1.9041529	1.1614225
STK16	NC_000002	220110192	220115059	1.0166783	0.9819036	1.4115139	1.4705953
TUBA4A	NC_000002	220115001	220118638	19.803466	16.233008	24.733575	60.089409
TUBA4B	NC_000002	220117965	220136910	0	0	0	0
DNAJB2	NC_000002	220144040	220151622	2.7001794	2.0675925	2.3020647	6.1249757
PTPRN	NC_000002	220154345	220174143	0.8228193	11.432694	0.043059	0.0505918
RESP18	NC_000002	220192131	220197899	0.1658424	0.0568345	0.0983587	0.0385219
DNPEP	NC_000002	220238180	220252662	12.059626	12.102035	12.13981	11.380697
LOC100288941	NC_000002	220283036	220290904	0.0461157	0	0.0410258	0.0321352
DES	NC_000002	220283099	220291461	0.0781997	0.0602981	0.0173922	0.0408695
SPEG	NC_000002	220299700	220358354	0.784053	0.3563761	1.4941511	2.8669487
GMPPA	NC_000002	220363613	220371711	19.320006	20.551319	19.506239	22.293845
ACCN4	NC_000002	220379052	220403494	0.0801682	0.0164843	0	0.0558644
CHPF	NC_000002	220403669	220408487	18.455025	13.656234	8.6796334	6.1024498
TMEM198	NC_000002	220408745	220415317	0.8916458	1.3037591	0.4054301	0.7456012
OBSL1	NC_000002	220415464	220436011	4.4768358	3.9760775	3.1393183	3.2192562
INHA	NC_000002	220436954	220440427	0.2161895	0.0317522	0	0.0430427
STK11IP	NC_000002	220462596	220481173	2.9534774	3.3752666	2.149769	3.0786465
SLC4A3	NC_000002	220492292	220506702	0.2247991	0.4832449	0.5272399	0.7191613
LOC100129746	NC_000002	220548787	220556575	0	0	0	0
RPL23P4	NC_000002	220562535	220562936	0	0	0	0
EPHA4	NC_000002	222282747	222437010	1.8969464	0.434182	0.7452435	0.8442586
LOC729770	NC_000002	222437226	222439174	0	0	0	0
RPL23P5	NC_000002	222448232	222457651	0	0	0	0
LOC646644	NC_000002	222808942	222809530	0	0	0	0.0519947
HSPA9P	NC_000002	222826389	222829176	0	0	0	0
RPL23AP28	NC_000002	222909244	222909713	0	0	0	0
PAX3	NC_000002	223064607	223163700	0.0320868	0.0109962	0.0190302	0
CCDC140	NC_000002	223162866	223169936	0	0	0.0690363	0.0360505
LOC440934	NC_000002	223181395	223186441	0	0	0	0
SGPP2	NC_000002	223289322	223423617	0.2929882	0.0753057	0.0325813	0.0765622
NANOGP2	NC_000002	223316894	223318205	0	0	0	0
FARSB	NC_000002	223436162	223520827	8.6597501	9.7529779	12.098709	14.194693
MOGAT1	NC_000002	223536457	223574649	0	0.0824515	0	0.0558848
RPL31P17	NC_000002	223591149	223591594	0	0	0	0
ACSL3	NC_000002	223725732	223808119	13.705479	12.826657	17.150572	11.973267
KCNE4	NC_000002	223916862	223920355	4.3784966	3.4333892	3.1822065	2.6477436
TRNAK39P	NC_000002	224186315	224186387	0	0	0	0
LOC100289013	NC_000002	224262980	224414221	0	0	0	0
SCG2	NC_000002	224461658	224467121	1.1359801	0.5292079	0.7579496	0.2226364
LOC646696	NC_000002	224569507	224570057	0	0	0	0
AP1S3	NC_000002	224620047	224702319	1.0212857	0.496769	0.79133	2.9308667
LOC100132512	NC_000002	224701924	224785530	0	0	0	0
WDFY1	NC_000002	224740065	224810052	26.39565	19.428765	22.115966	22.441847
MRPL44	NC_000002	224822121	224832431	6.8637685	5.9968817	7.3587523	3.5565505
SERPINE2	NC_000002	224839765	224904036	111.4345	82.408953	25.28711	11.068749
LOC100289117	NC_000002	225159647	225160470	0	0	0	0
FAM124B	NC_000002	225243415	225266711	0.0338845	0.0348369	0.0301446	0.011806
LOC100289075	NC_000002	225266131	225266760	0	0	0	0
CUL3	NC_000002	225334869	225450110	2.701417	4.2093028	4.0054257	6.2929069
DOCK10	NC_000002	225629807	225907330	0.9462679	1.8058768	1.2732621	3.1857127
KIAA1486	NC_000002	226265602	226518734	0.1436217	0.0689071	0.0936979	0.0200163
IRS1	NC_000002	227596033	227663506	5.7601563	4.4117951	2.573386	2.6958026
RHBDD1	NC_000002	227700773	227861592	1.7231044	1.7342371	3.9210518	3.8676074

COL4A4	NC_000002	227867427	228029275	0.1869957	0.1179723	0.5898095	0.4057256
COL4A3	NC_000002	228029281	228179507	0.0325703	0.0502286	0.1690236	0.1248297
MFF	NC_000002	228192228	228222549	5.3105024	4.6958384	7.6406481	12.228631
TM4SF20	NC_000002	228226872	228244022	0.0765649	0.0590375	0	0.0533534
AGFG1	NC_000002	228336888	228425938	3.4150663	3.6558326	8.416402	7.5423138
C2orf83	NC_000002	228474806	228497888	0.0238331	0.0490059	0.0212026	0.0166079
SLC19A3	NC_000002	228549926	228582745	0.2099995	0.0479781	0.2906111	0.1300765
LOC100289106	NC_000002	228643206	228643451	0	0	0	0
LOC100129886	NC_000002	228666410	228669393	0	0	0	0
CCL20	NC_000002	228678558	228682280	0.1043901	0	7.1972953	0.2182295
TDGF2	NC_000002	228732690	228735526	0	0	0	0
LOC100132934	NC_000002	228734830	228736380	0	0.0768425	0	0.0520831
WDR69	NC_000002	228736327	228789026	0.1316604	0.189505	0.1874059	0.1284447
SPHKAP	NC_000002	228844670	229046361	0.019072	0.006536	0.0056557	0.0398704
LOC646794	NC_000002	229216833	229217930	0	0	0	0
RPL17P14	NC_000002	229868602	229869098	0	0	0	0
PID1	NC_000002	229888689	230136057	0.6852311	1.4730237	0.7065825	0.6728357
LOC100289215	NC_000002	230125534	230126312	0	0	0	0
DNER	NC_000002	230222345	230579286	2.175921	0.4004641	0.7647446	1.1418812
RNU7-9P	NC_000002	230470955	230471015	0	0	0	0
LOC100130031	NC_000002	230566510	230566868	0	0	0	0
TRIP12	NC_000002	230631930	230786655	14.624342	20.8555	17.47469	20.048147
FBXO36	NC_000002	230787207	230877825	0.8118323	1.0914641	1.0972319	0.6745089
SLC16A14	NC_000002	230899698	230933619	0.0818784	0.2735837	0.6009407	0.1569044
RNY4P19	NC_000002	230922947	230923040	0	0	0	0
SP110	NC_000002	231033634	231084827	8.2789803	5.0592682	4.7495282	2.5772345
SP140	NC_000002	231090445	231177930	0.0985386	0.1392986	0.0109578	0.2231634
SP140L	NC_000002	231191894	231268445	2.1743503	1.8459486	1.9490166	2.3875467
LOC729879	NC_000002	231275415	231277244	0	0	0	0
SP100	NC_000002	231280871	231410317	8.3966134	6.3498161	11.613476	4.5617197
HMGB1L3	NC_000002	231379576	231380916	0	0	0	0
LOC646839	NC_000002	231436610	231438671	0	0	0	0
CAB39	NC_000002	231577557	231685790	6.1061714	9.1199678	6.7204571	8.5312161
ITM2C	NC_000002	231729621	231743963	7.7169109	4.0758785	0.5092302	0.2806911
GPR55	NC_000002	231772043	231789941	0.0229615	0.0118034	0.0102136	0.0240007
LOC100289170	NC_000002	231812752	231822669	0	0	0	0
SPATA3	NC_000002	231860839	231871999	0	0	0	0
C2orf72	NC_000002	231902281	231914429	0.0859083	0.1009403	0	0.0427602
PSMD1	NC_000002	231921609	232037473	17.636492	21.786605	19.009631	28.147936
HTR2B	NC_000002	231972947	231989824	0.0196636	0	0	0.0274048
ARMC9	NC_000002	232063342	232209914	8.0842554	5.4594913	4.2834574	10.825025
RPS28P4	NC_000002	232120779	232121170	0	0	0	0
LOC100289203	NC_000002	232258390	232259722	0.0329694	0.033896	0.0879915	0.0229744
B3GNT7	NC_000002	232260335	232263684	0	0	0.0272647	0
LOC729898	NC_000002	232298674	232299137	0.42772	1.4291587	0.2853837	0
LOC100289360	NC_000002	232301814	232302132	0	0	0	0
LOC100289394	NC_000002	232319142	232322149	0	0	0.0222145	0.139204
NCL	NC_000002	232319459	232329205	0.9219909	1.8791772	0.8202286	2.885524
SNORA75	NC_000002	232320511	232320647	0	0	0	0
SNORD20	NC_000002	232321155	232321234	0	0	0	0
SNORD82	NC_000002	232325079	232325153	0	0	0	0
C2orf52	NC_000002	232373137	232379050	0	0	0	0
NMUR1	NC_000002	232387871	232395182	0	0.0138007	0	0
RPL21P35	NC_000002	232424559	232425108	0	0	0	0
RPL23AP26	NC_000002	232450575	232455638	0	0	0	0
C2orf57	NC_000002	232457612	232458994	0.0317775	0.0326706	0.0565402	0.0221438
PTMA	NC_000002	232573235	232578251	22.665355	34.897445	61.332688	67.384873
PDE6D	NC_000002	232597147	232645974	8.1371055	11.437301	9.6519929	9.1217213
COPS7B	NC_000002	232651162	232673434	9.2253496	9.1646657	10.79781	9.9604435
RPL28P2	NC_000002	232654196	232654596	0	0	0	0
GAMTP	NC_000002	232674683	232675209	0	0	0	0
NPPC	NC_000002	232790135	232790959	0	0	0	0
DIS3L2	NC_000002	232826293	233201908	0.8031659	0.6967172	0.5247246	0.9706915
LOC730069	NC_000002	233207804	233209423	0	0	0	0
ECEL1P3	NC_000002	233215835	233216072	0	0	0	0
ALPP	NC_000002	233243348	233247599	0	0	0	0

ECEL1P2	NC_000002	233250460	233251754	0	0	0	0
ALPPL2	NC_000002	233271552	233275424	0	0	0.0313533	0
LOC100289433	NC_000002	233281147	233282347	0	0	0	0
LOC100131546	NC_000002	233282644	233284785	0	0	0	0
LOC100289284	NC_000002	233284843	233286264	0.042096	0	0	0
DIS3L2P	NC_000002	233306749	233312676	0	0	0	0
ALPI	NC_000002	233320833	233324742	0.0344692	0.017719	0.0153324	0.0360293
ECEL1	NC_000002	233344537	233352532	0.0153719	0.0158039	0.0273505	0.0107117
LOC646960	NC_000002	233385309	233390423	0	0	0	0.0167075
CHRND	NC_000002	233390922	233400205	0	0	0	0
CHRNG	NC_000002	233404437	233411038	0.0401904	0.12396	0.1430181	0.196044
TIGD1	NC_000002	233412779	233415226	0.5744867	0.8674918	1.1179859	0.7380996
EIF4E2	NC_000002	233415357	233433920	8.493464	12.225043	7.8660117	15.813242
EFHD1	NC_000002	233498324	233547486	0.1176345	0.3628217	0.0418603	0.0163945
GIGYF2	NC_000002	233562015	233725287	0.4219469	0.6760609	0.580126	1.4930583
KCNJ13	NC_000002	233630860	233641275	0.0134769	0	0.0239789	0.0093913
C2orf82	NC_000002	233734994	233741107	0	0	0.0840808	0
LOC100289490	NC_000002	233741854	233743418	0.1123277	0	0.0249825	0.1174117
NGEF	NC_000002	233743396	233877951	1.3019807	4.9737175	3.5997468	3.228821
NEU2	NC_000002	233897382	233899767	0	0	0	0.0535868
INPP5D (NC_000002 23[illegible]	NC_000002	233925036	233995874	0	0	0	0
INPP5D (NC_000002 23[illegible]	NC_000002	234054795	234116549	0.0982693	0.0449028	0.0777094	0.0304347
ATG16L1	NC_000002	234160217	234204320	3.8979048	6.8073672	4.1566281	4.9017793
SCARNA5	NC_000002	234184372	234184649	0	0	0	0
SCARNA6	NC_000002	234197322	234197587	0	0	0	0
SAG	NC_000002	234216309	234255701	0.0251276	0	0.0223542	0.0350199
DGKD	NC_000002	234263153	234380743	0.3503767	0.4591091	0.3850472	0.9957753
LOC100289669	NC_000002	234378039	234380744	0	0	0	0.0339522
USP40	NC_000002	234384165	234474236	0.7745905	1.2307389	1.7958291	1.7774097
LOC100286893	NC_000002	234384166	234385996	0	0.0246769	0	0
UGT1A12P	NC_000002	234494085	234494937	0	0	0	0
UGT1A11P	NC_000002	234512199	234513044	0	0	0	0
UGT1A8	NC_000002	234526291	234681945	0	0	0	0.0127817
UGT1A10	NC_000002	234545123	234681951	0	0	0	0
UGT1A13P	NC_000002	234556575	234557672	0	0	0	0
UGT1A9	NC_000002	234580544	234681951	0	0	0	0.0128893
UGT1A7	NC_000002	234590584	234681945	0	0	0	0
UGT1A6	NC_000002	234600321	234681951	0	0	0	0
UGT1A5	NC_000002	234621638	234681945	0	0	0	0
UGT1A4	NC_000002	234627438	234681945	0	0	0	0
RPL17P11	NC_000002	234630187	234630711	0	0	0	0
UGT1A3	NC_000002	234637773	234681945	0	0	0	0
DNAJB3	NC_000002	234651396	234652661	0	0.0356899	0	0
UGT1A2P	NC_000002	234655754	234657186	0	0	0	0
LOC100286922	NC_000002	234662962	234663915	0	0	0	0
UGT1A1	NC_000002	234668919	234681945	0	0	0	0
HEATR7B1	NC_000002	234687052	234742067	0.0497997	0.0255997	0.0221516	0.0404861
HJURP	NC_000002	234745486	234763212	2.4207551	3.4072729	3.1021675	5.7434198
MSL3L2	NC_000002	234774083	234777055	0	0	0	0
TRPM8	NC_000002	234826043	234928166	0.0156372	0.0080383	0.0208669	0.0272415
LOC100130859	NC_000002	234854790	234864073	0.1494838	0.0768425	0.1329849	0.83333
SPP2	NC_000002	234959346	234985776	0	0.0443409	0	0
RPS20P12	NC_000002	235238046	235238517	0	0	0	0
LOC100287159	NC_000002	235358924	235364427	0	0	0.0740484	0.2320067
ARL4C	NC_000002	235401685	235405693	3.8039502	3.6741804	1.1117791	1.8715628
SH3BP4	NC_000002	235860628	235964358	14.118041	17.470102	6.140347	15.498564
LOC642692	NC_000002	236055667	236056144	0	0	0	0
AGAP1	NC_000002	236402736	237034127	1.8724081	1.7853927	2.1318099	2.7988299
GBX2	NC_000002	237074307	237076652	0.03292	0.0676905	0	0
LOC100128572	NC_000002	237075923	237203570	0	0.062842	0	0
ASB18	NC_000002	237103515	237172988	0	0	0	0
IQCA1	NC_000002	237232794	237416092	0.0137855	0.1417296	0.012264	0.0384252
RPL3P5	NC_000002	237316433	237317746	0	0	0	0
CXCR7	NC_000002	237478380	237490997	1.6300343	2.4922847	1.0225231	1.3543099
LOC93463	NC_000002	237964070	237964827	0	0	0	0
COPS8	NC_000002	237994084	238007489	19.580895	18.263975	15.315878	15.121281

COL6A3	NC_000002	238232655	238322850	26.935477	31.838904	44.237412	47.898224
LOC100286991	NC_000002	238249351	238253413	0	0.0466289	0.0806967	0
MLPH	NC_000002	238395878	238463961	4.0601801	0.8901948	2.9770777	4.2887875
LOC100287288	NC_000002	238429322	238438955	0	0	0	0
PRLH	NC_000002	238475217	238475818	0	0	0	0
RAB17	NC_000002	238482965	238499736	0.0223541	0	0	0.0311545
LRRFIP1	NC_000002	238536224	238690290	2.1598128	3.5632864	3.1135203	3.8611065
RBM44	NC_000002	238707388	238751451	0.0513054	0.0105495	0.0182571	0.0357517
RAMP1	NC_000002	238768187	238820756	1.8352009	0.1489563	0.3437149	1.7163393
UBE2F	NC_000002	238875700	238950684	9.7698802	12.280275	9.0091879	10.426934
SCLY	NC_000002	238969632	239008054	1.9472997	2.0571305	2.0343449	3.2367758
ESPNL	NC_000002	239008951	239041928	0.3472314	0.2508581	0.050093	0.0326979
KLHL30	NC_000002	239049417	239061547	0.0617771	0.0127027	0.0219834	0.034439
FAM132B	NC_000002	239069674	239075695	0.5175064	1.4324448	2.4790123	1.137337
LOC100287347	NC_000002	239075749	239077544	0.4744411	0.8471888	3.0433901	1.8270524
ILKAP	NC_000002	239079043	239112324	6.1216872	9.1431416	6.2046726	6.2183571
LOC151174	NC_000002	239133754	239140318	0	0	0	0
LOC643387	NC_000002	239140327	239142986	0	0	0	0
HES6	NC_000002	239146908	239148681	0.898212	1.1213399	0.7419976	0.9835727
PER2	NC_000002	239152679	239197207	0.1316645	0.0783692	0.2589243	0.3766541
TRAF3IP1	NC_000002	239229185	239309541	0.168384	0.2921341	0.3932226	0.4400126
LOC100287019	NC_000002	239334020	239335530	0.4071974	0.5382536	0.1293765	0.2432154
ASB1	NC_000002	239335626	239360891	2.3926156	3.934454	1.8252778	3.5227798
LOC100287050	NC_000002	239419331	239420067	0	0	0	0.0831069
LOC100287387	NC_000002	239755218	239756320	0.2943901	1.1241803	0.1122418	0.1465305
TWIST2	NC_000002	239756726	239757391	0.7258717	3.4599902	0.1761152	0.2299165
LOC100287089	NC_000002	239756805	239775646	3.4695972	37.832992	2.3383712	2.4177535
FLJ43879	NC_000002	239835312	239847374	0	0	0	0
HDAC4	NC_000002	239969864	240322643	0.4210726	0.3120957	0.8363116	0.9757927
LOC100288992	NC_000002	240115027	240117153	0	0	0	0.0431945
MGC16025	NC_000002	240115027	240117153	0	0	0	0
FLJ45964	NC_000002	240499995	240504804	0	0	0	0
LOC100288898	NC_000002	240882747	240885078	0.0188457	0.0968769	0.0335314	0.0131325
NDUFA10	NC_000002	240900156	240964798	12.37573	14.942775	16.796335	14.660633
OR6B2	NC_000002	240968908	240969846	0.0468032	0.0481186	0	0
PRR21	NC_000002	240981230	240982399	0	0	0.0334167	0
OR6B3	NC_000002	240984494	240985489	0	0.0453649	0	0
OR9S24P	NC_000002	241018850	241019777	0	0	0	0
OR5S1P	NC_000002	241048519	241049466	0	0	0	0
MYEOV2	NC_000002	241065980	241075764	3.2425573	4.7624139	3.2967644	3.0665227
OTOS	NC_000002	241078446	241080073	0.1552941	0.0798293	0.069077	0
GPC1	NC_000002	241375115	241407495	13.747236	8.8748733	10.066085	18.48626
PP14571	NC_000002	241388836	241396117	0	0	0	0
LOC100288928	NC_000002	241405476	241407497	0.021735	0.0670377	0.0386722	0.0151458
ANKMY1	NC_000002	241418839	241497405	0.3355812	0.2919339	0.4478135	0.8274563
DUSP28	NC_000002	241499471	241503431	0.1695752	0.3196254	0.1508588	0.4135836
RNPEPL1	NC_000002	241508104	241518144	7.9960738	4.4361321	5.1942183	7.9059075
CAPN10	NC_000002	241526145	241557122	2.0711236	1.0386989	1.1347282	2.3936686
GPR35	NC_000002	241568795	241570669	0.1640734	0.0240978	0.2710765	0.2613323
AQP12B	NC_000002	241615835	241622317	0	0	0	0.0274909
LOC100289063	NC_000002	241624478	241627223	0	0	0	0
LOC100289094	NC_000002	241627222	241629111	0	0	0	0
AQP12A	NC_000002	241631262	241637900	0	0.0416821	0	0.0282517
KIF1A	NC_000002	241653184	241759624	0.0299103	0.030751	0.0044348	0.0625281
AGXT	NC_000002	241808162	241818536	0.027502	0	0	0
C2orf54	NC_000002	241825465	241835573	0.016704	0	0.0297207	0.01164
LOC728763	NC_000002	241847085	241930699	0	0	0	0
SNED1	NC_000002	241938255	242033643	0.7331136	1.9900796	0.308936	0.3629814
MTERFD2	NC_000002	242026509	242041724	1.1802079	1.1139205	0.6626706	0.9235325
PASK	NC_000002	242045515	242088878	1.6201666	2.5833336	2.2440104	3.4545944
LOC100289155	NC_000002	242089430	242092991	0.4120147	0.7059907	0.2443598	0.3349596
PPP1R7	NC_000002	242089902	242122439	14.785368	20.966776	12.155624	16.74539
ANO7	NC_000002	242127924	242164791	0.3062939	0.1523721	0.1318488	0.2960589
HDLBP	NC_000002	242166679	242255254	52.245594	66.428172	45.613827	73.486798
SEPT2	NC_000002	242254723	242293442	34.520011	39.557777	32.860892	32.164587
FARP2	NC_000002	242295711	242434255	0.7696713	0.8365203	0.7825382	1.0190415

STK25	NC_000002	242434432	242448034	20.875923	24.628225	17.037846	21.770698
LOC100289192	NC_000002	242434792	242448987	0.411735	0.0552139	0.207034	0.3617603
BOK	NC_000002	242498192	242513553	6.2179736	8.7031466	2.465206	3.956155
THAP4	NC_000002	242523820	242576655	0.936802	0.900318	1.1776375	2.2564206
ATG4B	NC_000002	242577027	242613272	4.7921467	4.5838234	4.1418056	6.2454462
DTYMK	NC_000002	242615157	242626227	15.484135	21.154242	16.89401	22.568761
ING5	NC_000002	242641456	242668896	0.6848416	0.6171646	0.3685611	0.7541332
D2HGDH	NC_000002	242674030	242708231	0.3059042	0.1747231	0.1511893	0.1539534
LOC728846	NC_000002	242674130	242715546	0.227711	0.1560739	0.2701041	0.1586781
GAL3ST2	NC_000002	242716240	242743702	0.1516502	0.0623649	0	0.0422704
NEU4	NC_000002	242752073	242758734	0.0763653	0	0.0509525	0.0133036
PDCD1	NC_000002	242792033	242801058	0.0208088	0.0213937	0.0185121	0.0435012
C2orf85	NC_000002	242811886	242815482	0.0244428	0	0.021745	0
LOC285095	NC_000002	242836134	242842599	0	0	0	0
FLJ38379	NC_000002	242945848	242948164	0	0	0	0
LOC100289268	NC_000002	242962494	242968396	0	0	0	0.0261751
LOC728323	NC_000002	243030844	243102469	0	0	0	0
LOC100289034	NC_000002	243159830	243160835	0	0	0	0
LOC642891	NC_000003	237441	239090	0	0	0	0.0385219
CHL1	NC_000003	238650	451098	0.0344692	0.0118127	0.0153324	0.0240195
RPS8P6	NC_000003	350400	351002	0	0	0	0
RPSAP32	NC_000003	659472	660040	0	0	0	0
CNTN6	NC_000003	1134629	1445278	0.0248998	0	0.0221516	0.0173512
RPL23AP38	NC_000003	1637421	1637929	0	0	0	0
RPL23AP39	NC_000003	1771738	1772158	0	0	0	0
RPL21P17	NC_000003	1947332	1947853	0	0	0	0
LOC100288445	NC_000003	2140345	2141457	0	0.0552364	0.0477965	0
CNTN4	NC_000003	2142247	3099645	0.0080198	0.0164903	0.0285384	0.0335309
IL5RA	NC_000003	3111401	3152058	0.1056085	0.0775548	0.3087003	0.0735922
TRNT1	NC_000003	3168600	3190707	3.1474349	2.5013658	2.2503432	4.4941605
CRBN	NC_000003	3191695	3221390	2.5746239	1.9493293	3.5510959	5.0346076
LOC100130207	NC_000003	3782694	3832392	0	0	0	0
LRRN1	NC_000003	3841121	3889387	0.045983	0.0236377	0	0.0240321
LOC100288506	NC_000003	4023656	4027546	0	0	0	0
SETMAR	NC_000003	4344988	4358949	1.4574145	1.3084404	1.2052496	2.088385
LOC100288015	NC_000003	4345311	4355191	0	0.0420702	0	0.0570296
SUMF1	NC_000003	4402830	4508954	9.6571534	7.5726372	13.778798	20.131438
MRPS10P2	NC_000003	4456244	4456647	0	0	0	0
ITPR1	NC_000003	4535034	4889286	0.4056897	0.1329193	0.6623345	0.8201428
EGO	NC_000003	4790876	4793274	0	0	0	0
LOC100288535	NC_000003	5018303	5021029	0.2121395	0.1999266	0.1415439	0.1355083
BHLHE40	NC_000003	5021097	5026866	19.983051	12.252369	7.7422198	11.301438
LOC643182	NC_000003	5121263	5126193	0	0	0	0
ARL8B	NC_000003	5163930	5222601	18.418505	16.605089	28.409588	32.399787
EDEM1	NC_000003	5229359	5261650	6.806869	7.6737616	14.519411	14.623093
MRPS35P1	NC_000003	5488662	5488789	0	0	0	0
MRPS36P1	NC_000003	6814724	6815033	0	0	0	0
GRM7	NC_000003	6902927	7783217	0	0	0	0.0297835
LOC100288428	NC_000003	8540171	8543323	0.1652189	0.0849312	0.0734917	0
LMCD1	NC_000003	8543511	8609806	3.6243351	4.4818599	0.9469999	2.6845337
LOH3CR2A	NC_000003	8613468	8615580	0	0	0	0
C3orf32	NC_000003	8661317	8693737	0.0568909	0	0	0.0594658
OR7E122P	NC_000003	8729920	8730840	0	0	0	0
CAV3	NC_000003	8775496	8788451	0	0.0317969	0.0275141	0.0431033
OXTR	NC_000003	8792094	8811300	0.090698	1.8856636	0.0627569	0.126404
RAD18	NC_000003	8921560	9005146	1.7025251	3.8419242	3.427134	4.3132215
SRGAP3	NC_000003	9022278	9291311	0.0590438	0.1163478	0.0262635	0.0651447
LOC100288831	NC_000003	9259518	9260213	0	0	0	0
LOC100288572	NC_000003	9379286	9406498	0.2781534	0.5719418	0.0618632	0.0969142
LOC391508	NC_000003	9390127	9390876	0	0	0	0
THUMPD3	NC_000003	9404717	9428475	3.3568499	5.199497	4.5875787	4.462922
LOC440944	NC_000003	9430537	9439174	0	0	0	0
SETD5	NC_000003	9439403	9519838	4.0555714	4.8909527	5.9387982	6.4775632
LHFPL4	NC_000003	9540045	9595486	0.0810521	0.0462945	0.0480708	0.0941338
LOC643459	NC_000003	9642395	9644412	0	0	0	0
MTMR14	NC_000003	9691144	9744077	1.4608343	2.2799794	1.7223597	3.556754

CPNE9	NC_000003	9745510	9771592	0.0428972	0.0220514	0.0381626	0.0149463
BRPF1	NC_000003	9773434	9789699	1.8125857	1.9113114	1.6621427	1.1659218
OGG1	NC_000003	9791628	9808353	1.1439216	1.7482146	1.0589211	1.1741512
CAMK1	NC_000003	9799031	9811661	6.223283	7.251281	11.257357	10.986133
TADA3L	NC_000003	9821651	9834420	14.836206	16.247012	12.650312	23.166349
ARPC4	NC_000003	9834227	9848789	42.102228	48.316057	32.508755	40.853836
TTLL3	NC_000003	9851904	9878040	1.3435086	0.4515684	0.9538795	1.1702628
RPUSD3	NC_000003	9879533	9885702	5.4442797	6.4442505	4.4291456	6.1649103
CIDEC	NC_000003	9908398	9920740	0.2064913	0.3892071	0.2143171	0.0959276
JAGN1	NC_000003	9932271	9936033	8.4719483	8.818929	10.692949	9.6302328
IL17RE	NC_000003	9944303	9958086	1.5029093	1.5776782	0.4362939	0.154337
IL17RC	NC_000003	9958764	9975314	3.0526518	3.8036612	4.3835323	5.6764119
LOC100288640	NC_000003	9974091	9995712	0	0	0	0.0250818
CRELD1	NC_000003	9975506	9987090	0.6665709	0.5607041	2.0485453	1.6785094
LOC100289441	NC_000003	9985228	9995549	0.0289896	0	0.1289498	0.0606033
PRRT3	NC_000003	9987226	9994078	0.7011151	0.1561777	1.746448	1.2132696
TMEM111	NC_000003	10005636	10028522	35.280664	31.419199	48.980561	52.40257
LOC442075	NC_000003	10028595	10046944	0	0	0	0
CYCSP10	NC_000003	10042324	10042631	0	0	0	0
LOC401052	NC_000003	10048102	10052779	0.7876858	0.3096385	1.1129513	0.8717678
CIDECP	NC_000003	10059237	10067820	0	0	0	0
FANCD2	NC_000003	10068113	10143614	1.1380575	1.709478	1.7684959	1.622135
CYCSP11	NC_000003	10099246	10100140	0	0	0	0
C3orf24	NC_000003	10123004	10149915	0.0433415	0.0891191	0.154231	0.030202
C3orf10	NC_000003	10157333	10168874	28.289806	32.235437	34.276862	25.182192
VHL	NC_000003	10183319	10193762	1.0661296	1.2787755	1.159227	1.7231653
IRAK2	NC_000003	10206563	10285427	1.3935732	1.3806401	4.6434699	2.8691512
TATDN2	NC_000003	10290596	10322902	4.1948803	6.126563	5.5629453	11.057466
C3orf42	NC_000003	10326103	10327430	0	0	0	0
GHRL	NC_000003	10327434	10334631	0.069319	0	0.4316766	0.3381296
GHRLOS	NC_000003	10327438	10335133	0	0	0	0
SEC13	NC_000003	10342615	10362858	44.750308	57.211457	47.02042	35.173318
ATP2B2	NC_000003	10365707	10547268	0.0294231	0.0050417	0.0043626	0.0615095
LOC285370	NC_000003	10801169	10805877	0	0	0	0
SLC6A11	NC_000003	10857917	10980146	0.0662203	0.0226938	0.0589114	0.046145
SLC6A1	NC_000003	11034420	11080935	0.0097793	0.0402166	0	0.0272585
HRH1	NC_000003	11178779	11304939	1.3682513	1.4442183	1.6635535	2.0785254
ATG7	NC_000003	11314010	11599139	7.1695393	6.8700956	8.7081293	9.8283365
VGLL4	NC_000003	11597544	11762220	7.3977935	2.8031351	5.2497599	6.8357917
C3orf31	NC_000003	11831919	11888352	2.06939	3.3535965	2.2466279	2.9818317
LOC100129929	NC_000003	11888201	11943081	0	0	0	0
CYCSP12	NC_000003	11920291	11920594	0	0	0	0
LOC100128204	NC_000003	11946990	11955089	0	0	0	0
SYN2	NC_000003	12045862	12233532	0.0077455	0.0238897	0.0068907	0.026987
TIMP4	NC_000003	12194568	12200647	0.7381089	0.1580945	0.3283211	0.3643268
GSTM1L	NC_000003	12299296	12299952	0	0	0	0
PPARG	NC_000003	12329349	12475855	3.7869665	5.0314703	8.7075444	7.9843916
TSEN2	NC_000003	12525931	12574820	0.4024117	0.6644618	0.824477	1.2916153
LOC100129480	NC_000003	12578980	12583432	0.058075	0.1194143	0.0129163	0.1011724
MKRN2	NC_000003	12598594	12625212	5.4478654	7.0706227	6.1606573	6.7624867
RAF1	NC_000003	12625100	12705700	18.733353	19.494396	25.320897	25.650089
LOC100288753	NC_000003	12713780	12717377	0	0	0	0
TMEM40	NC_000003	12775392	12800808	0	0	0.0685922	0.0179093
KRT18P17	NC_000003	12828834	12830219	0	0	0	0
CAND2	NC_000003	12838171	12876301	2.2031273	1.3996277	1.0262071	0.4127732
RPL32	NC_000003	12876444	12883081	34.373711	59.815144	53.714461	40.986927
SNORA7A	NC_000003	12881811	12881949	0	0	0	0
LOC100288782	NC_000003	12923914	12926576	0	0.1083535	0	0
IQSEC1	NC_000003	12938719	13114617	0.7600161	0.7999806	1.0195632	1.2021295
RPL39P17	NC_000003	13094274	13094682	0	0	0	0
NUP210	NC_000003	13357737	13461809	0.1650121	0.1256665	2.2835458	0.0468466
LOC100128772	NC_000003	13501791	13514100	0	0	0	0
HDAC11	NC_000003	13521839	13546834	1.5912291	1.7657881	1.3706618	2.0768596
FBLN2	NC_000003	13590625	13679922	0.7419567	1.7450574	0.5696454	1.246526
LOC285375	NC_000003	13692221	13788132	0	0	0	0
TRNASUP4P	NC_000003	13833886	13833953	0	0	0	0

WNT7A	NC_000003	13860082	13921618	0	0.0263614	0	0
LOC100132526	NC_000003	13974553	13978444	0.039042	0	0	0.0090687
TPRXL	NC_000003	13978807	14107484	0	0	0	0
LOC100128368	NC_000003	14130897	14131460	0	0	0	0
CHCHD4	NC_000003	14153577	14166371	1.5891505	4.0281961	3.3150056	4.9068539
TMEM43	NC_000003	14166440	14185180	9.0473918	14.696098	12.248699	13.378912
XPC	NC_000003	14186647	14220172	0.5432998	0.5099981	0.4097837	0.3621324
LSM3	NC_000003	14220329	14239838	28.853311	31.927449	45.811996	69.193598
SLC6A6	NC_000003	14444106	14530857	3.4534408	0.8942	3.402262	3.3824625
GRIP2	NC_000003	14535173	14583588	0.9877448	0.171449	0.9700213	0.1787792
LOC131973	NC_000003	14614293	14617361	0	0	0	0
C3orf19	NC_000003	14693253	14714166	0.7572162	1.1906437	0.7396837	1.1898179
C3orf20	NC_000003	14716654	14814541	0.0402211	0.082703	0.0238545	0.0373702
FGD5	NC_000003	14860469	14976072	0.5067797	0.0306484	0.0198902	0.0415464
NR2C2	NC_000003	14989236	15090780	1.2873192	1.6272534	1.5864318	3.6323385
MRPS25	NC_000003	15090019	15106816	0.8373842	0.9301883	0.8905271	1.8712968
ZFYVE20	NC_000003	15111580	15140655	2.1849076	2.496657	2.9566144	2.9716863
RPS24P10	NC_000003	15173343	15173710	0	0	0	0
LOC100287512	NC_000003	15182887	15183732	0	0	0	0
LOC344875	NC_000003	15186187	15222468	0	0	0	0
CAPN7	NC_000003	15247733	15294423	3.3303262	4.4583439	3.7504304	3.8421322
SH3BP5	NC_000003	15296357	15382901	2.3763633	1.5195209	2.1398602	2.6858614
HMGN2L7	NC_000003	15415760	15417267	0	0	0	0
METTL6	NC_000003	15451377	15469042	0.7913847	1.0125132	0.7666189	1.2254853
EAF1	NC_000003	15469064	15484120	3.6035983	3.0102133	5.3924673	3.6848116
COLQ	NC_000003	15491640	15563258	0.1044521	0.1342347	0.0813081	0.0363932
HACL1	NC_000003	15602239	15643130	3.9401863	6.6550922	5.4505473	7.3771259
BTD	NC_000003	15643255	15687325	4.7885483	4.6824446	4.7901618	6.2436193
ANKRD28	NC_000003	15708743	15901053	13.290544	10.506442	12.125858	16.986445
LOC100288942	NC_000003	15773428	15776042	0	0	0	0
RN7SLP1	NC_000003	15779959	15780272	0	0	0	0
IMPDH1P8	NC_000003	15919551	15921802	0	0	0	0
GALNTL2	NC_000003	16216184	16271253	1.789747	0.4770495	1.7101499	4.8236988
DPH3	NC_000003	16298568	16306496	5.9570666	5.4242313	5.4919336	5.5448734
OXNAD1	NC_000003	16306714	16347594	0.5940465	0.6453125	1.0270465	1.1559505
RFTN1	NC_000003	16357352	16555222	4.9628416	7.074017	4.3890338	3.4072983
DAZL	NC_000003	16628299	16647006	0.0432278	0	0.0640944	0.0100409
LOC100132260	NC_000003	16734252	16735073	0	0	0	0
PLCL2	NC_000003	16926452	17132098	0.5744208	1.0464397	0.8965275	1.313197
LOC100287913	NC_000003	17198648	17202644	0	0.0113043	0.0293452	0.0229859
TBC1D5	NC_000003	17198654	17784240	5.0553948	4.1366588	5.1063007	7.5369863
RPL31P19	NC_000003	17506623	17507040	0	0	0	0
RNU7-10P	NC_000003	17706459	17706516	0	0	0	0
TRNAC31P	NC_000003	17741392	17741464	0	0	0	0
PP1P	NC_000003	17912909	17914193	0	0	0	0
LOC100132683	NC_000003	17918943	17919661	0	0	0	0
SATB1	NC_000003	18389266	18480252	0.5944235	0.6915417	1.7186508	1.2808056
LOC100132345	NC_000003	18485534	18568000	0	0.0411882	0.0712809	0.0837508
LOC131185	NC_000003	18580165	18581391	0	0	0	0
KCNH8	NC_000003	19190017	19577135	0.0347211	0.0356969	0.0231666	0.0302438
EFHB	NC_000003	19920964	19975706	0.0769133	0.015815	0.1368483	0.0964732
LOC402125	NC_000003	19981845	19984820	0	0	0	0
LOC100130288	NC_000003	19987411	19988929	0.1252086	0	0	0.2181259
RAB5A	NC_000003	19988572	20026667	6.8132762	6.7374067	7.0792042	6.0645716
C3orf48	NC_000003	20041460	20054201	0	0	0	0
RPL39P18	NC_000003	20053895	20054293	0	0	0	0
KAT2B	NC_000003	20081524	20195896	2.6875473	1.3300255	5.5517895	5.9802312
SGOL1	NC_000003	20202085	20227683	0.579837	0.8065334	0.9558194	1.6043301
VENTXP7	NC_000003	21447218	21448200	0	0	0	0
ZNF385D	NC_000003	21462647	21792816	0.5842061	0.3003126	1.2048192	1.1287719
HMGB1L5	NC_000003	22423161	22424357	0	0	0	0
LOC100129341	NC_000003	22945623	22946634	0	0	0	0
LOC100130785	NC_000003	23031314	23033073	0	0	0	0
RPL24P7	NC_000003	23175810	23176358	0	0	0	0
UBE2E2	NC_000003	23244784	23632296	6.0179113	6.4694416	10.018751	8.1086325
UBE2E1	NC_000003	23847439	23932807	26.710692	29.156547	44.271459	31.08446

NKIRAS1	NC_000003	23933572	23958537	1.167547	2.1515906	1.9793755	2.7170945
RPL15	NC_000003	23958639	23962334	84.875436	100.75014	102.3442	203.86365
NR1D2	NC_000003	23986751	24022109	3.9293355	4.1487264	7.9413531	7.3111145
LOC152024	NC_000003	24141465	24144738	0	0	0	0
THRB	NC_000003	24158644	24536313	0.2973726	0.770205	0.9971533	0.9643748
RPL31P20	NC_000003	24472257	24472599	0	0	0	0
LOC644990	NC_000003	24535392	24541502	0	0	0	0
LOC100129045	NC_000003	24596140	24604696	0	0	0	0
LOC100130354	NC_000003	25469117	25469438	0	0	0	0
RARB	NC_000003	25469754	25639423	0.5893441	0.0577055	0.0998661	0.1466709
TOP2B	NC_000003	25639475	25705788	1.5922659	2.7167511	2.1624593	3.6355608
LOC641456	NC_000003	25728339	25728745	0	0	0	0
NGLY1	NC_000003	25760435	25831530	3.4574658	3.2860656	3.7593813	4.2617838
RPL32P11	NC_000003	25790949	25791387	0	0	0	0
LOC246135	NC_000003	25796384	25799034	0	0	0	0
LOC100131542	NC_000003	25821067	25831477	0	0	0.0621583	0
OXSM	NC_000003	25831563	25836025	1.3239153	1.7753793	1.9459168	3.2490048
LOC645065	NC_000003	26057160	26057864	0	0	0	0
LOC645101	NC_000003	26257813	26258088	0	0	0	0
VENTXP4	NC_000003	26387655	26389317	0	0	0	0
LRRC3B	NC_000003	26664300	26752267	0.0259588	0.0266884	0	0
NEK10	NC_000003	27257097	27410912	1.182389	0.376671	0.2666753	1.5202194
SLC4A7	NC_000003	27414214	27498245	11.217932	3.8735287	13.361821	17.154196
RPS20P15	NC_000003	27503997	27504310	0	0	0	0
UBA52P4	NC_000003	27527741	27528108	0	0	0	0
RPS27P11	NC_000003	27665636	27665979	0	0	0	0
LOC643634	NC_000003	27674467	27676842	0	0	0	0
LOC100289346	NC_000003	27744616	27756157	0	0	0	0
EOMES	NC_000003	27757868	27763785	0.1276637	0.0164065	0.0709832	0.1112014
CMC1	NC_000003	28283124	28361264	2.5975792	2.1653387	4.3719323	5.0878392
AZI2	NC_000003	28363845	28390618	7.5905132	5.6093991	8.6409354	7.6541306
ZCWPW2	NC_000003	28431987	28566633	0.5824464	0.1633135	0.6123715	0.6272565
RPL34P11	NC_000003	28489216	28489568	0	0	0	0
C3orf53	NC_000003	28616647	28617818	0	0	0	0
LOC131572	NC_000003	29128828	29129879	0	0	0	0
RBMS3	NC_000003	29322943	30046620	5.4483767	6.1895071	6.6412303	9.1979515
RPS12P5	NC_000003	29432306	29432732	0	0	0	0
TGFBR2	NC_000003	30647994	30735634	12.575323	27.346332	10.505808	10.071575
GADL1	NC_000003	30767692	30936153	0.0120241	0.0247242	0.021394	0.0502734
LOC339897	NC_000003	31222972	31273781	0	0	0	0
LOC391524	NC_000003	31408165	31497386	0	0	0	0
STT3B	NC_000003	31574491	31677556	14.73798	24.513043	21.684099	22.823619
OSBPL10	NC_000003	31702317	32023238	2.1285149	2.5684167	1.6444299	2.2014315
ZNF860	NC_000003	32023266	32033120	0.099302	0.0583388	0.2019242	0.1977074
RPL21P40	NC_000003	32071646	32072203	0	0	0	0
GPD1L	NC_000003	32148144	32210196	0.3596786	0.3813433	0.529967	0.7440827
RPSAP11	NC_000003	32232162	32233194	0	0	0	0
LOC100129194	NC_000003	32278177	32280071	0.0463834	0.0238435	0	0
CMTM8	NC_000003	32280171	32411813	0.4131885	0.926839	0.0668334	0.0523502
KRT18P15	NC_000003	32300421	32301781	0	0	0	0
CMTM7	NC_000003	32433163	32496333	1.9192788	3.076886	4.0515612	6.2564518
CMTM6	NC_000003	32522804	32544403	9.2962392	12.497251	7.5151886	10.135506
RPL31P18	NC_000003	32549423	32549854	0	0	0	0
DYNC1LI1	NC_000003	32567466	32612350	6.066094	8.8730383	5.7741676	7.6531386
LOC100289480	NC_000003	32587372	32612380	0	0	0	0.0967611
LOC391526	NC_000003	32662389	32663392	0	0	0	0
RPL30P4	NC_000003	32676657	32677114	0	0	0	0
CNOT10	NC_000003	32726698	32815354	2.2928118	3.4287295	3.9653861	5.4970595
SUGT1P2	NC_000003	32794322	32795412	0	0	0	0
RPL23AP43	NC_000003	32823051	32827672	0	0	0	0
LOC389102	NC_000003	32857841	32858858	0	0	0	0
TRIM71	NC_000003	32859510	32933771	0.0280103	0.0143988	0.0124594	0.0585562
CCR4	NC_000003	32993066	32996403	0.3978416	0.2454138	0.0235954	0.0184821
GLB1	NC_000003	33038100	33138694	12.782862	12.638596	18.314276	7.9520022
LOC100131671	NC_000003	33075355	33076302	0	0	0	0
LOC100289514	NC_000003	33096347	33118983	0.0887843	0.0912796	0	0

TMPPE	NC_000003	33131908	33138293	0	0	0	0
LOC100132168	NC_000003	33155449	33159180	0.5144442	0.0755575	0.1307611	0.0512122
CRTAP	NC_000003	33155450	33189265	23.001623	23.532597	18.53475	24.091018
SUSD5	NC_000003	33191537	33260707	3.8723617	3.0964849	1.8357498	5.2866923
FBXL2	NC_000003	33318934	33428125	2.115751	1.1448497	3.0049649	4.5652796
UBP1	NC_000003	33429828	33481897	7.8212063	8.8310553	7.0890676	8.8610426
CLASP2	NC_000003	33537737	33759848	1.8097399	3.21094	2.0730702	3.5850636
PDCD6IP	NC_000003	33839557	33911194	4.7097128	6.1384398	6.7244417	11.113867
FECHP	NC_000003	34914216	34916710	0	0	0	0
KRT8P18	NC_000003	35257005	35272126	0	0	0	0
LOC100130503	NC_000003	35594617	35683573	0	0.01336	0.0115605	0.0271658
ARPP-21	NC_000003	35683849	35835988	0.0324341	0.0250093	0.0360679	0.3729229
RPL36AP17	NC_000003	35913350	35913708	0	0	0	0
LOC100131711	NC_000003	36211238	36211940	0	0	0	0
LOC100289649	NC_000003	36339751	36342517	0	0	0	0
STAC	NC_000003	36422097	36589496	0.7564495	2.9735955	2.0188753	5.7776939
NBPF21P	NC_000003	36657402	36679390	0	0	0	0
DCLK3	NC_000003	36753913	36781352	0.0411192	0.0338199	0.0146323	0.0286535
HSPD1P6	NC_000003	36808291	36810378	0	0	0	0
LBA1	NC_000003	36868311	36902411	0.4105462	0.5797308	0.7304666	0.8341272
RNU6ATAC4P	NC_000003	37009683	37009807	0	0	0	0
LOC645571	NC_000003	37017787	37018334	0	0	0	0
EPM2AIP1	NC_000003	37027357	37034795	1.6837271	1.6338668	1.5346806	2.2436562
MLH1	NC_000003	37034979	37092337	11.579071	13.963175	10.424985	15.10617
RPL29P11	NC_000003	37057889	37058525	0	0	0	0
LRRFIP2	NC_000003	37094117	37217851	4.6898087	4.9282368	3.3726007	4.946231
LOC100130139	NC_000003	37176149	37176596	0	0	0	0
RPS16P4	NC_000003	37237725	37238160	0	0	0	0
LOC100288390	NC_000003	37258270	37259478	0	0	0.0323388	0.0253308
GOLGA4	NC_000003	37284738	37408242	0.6561993	0.7450392	0.8528163	3.1610982
TCEA1P2	NC_000003	37317028	37319650	0	0	0	0
C3orf35	NC_000003	37440968	37476988	0.0181155	0.0186247	0.0966964	0.2398486
ITGA9	NC_000003	37493813	37861281	0.0665546	0.1596587	0.0986814	0.1159448
RNU7-73P	NC_000003	37614549	37614803	0	0	0	0
CTDSPL	NC_000003	37903669	38025960	4.6562436	5.5274869	5.3007698	5.8706276
VILL	NC_000003	38035078	38048676	1.0565237	1.0700052	0.6172561	0.9230319
PLCD1	NC_000003	38048987	38071154	2.9974298	2.2569999	2.7041544	1.7945414
DLEC1	NC_000003	38080696	38164228	0.04697	0.0321934	0.0626787	0.0545509
ACAA1	NC_000003	38164201	38178733	7.1998752	8.0480606	4.4922437	5.8421291
MYD88	NC_000003	38179969	38184513	19.044234	12.524522	7.6823287	7.6830835
OXSR1	NC_000003	38207026	38296979	4.4502079	3.5363529	4.1578441	7.1500932
SLC22A13	NC_000003	38307298	38319806	0	0.0352995	0.0763624	0.0358885
SLC22A14	NC_000003	38347445	38359859	0	0.0228545	0	0.0929435
XYLB	NC_000003	38388240	38456467	1.1897193	1.7368823	0.9525666	2.404227
ACVR2B	NC_000003	38495790	38534633	0.1275206	0.1430232	0.333462	0.3958373
EXOG	NC_000003	38537763	38567796	2.1792512	2.3266723	1.441614	3.1637271
RPL18AP7	NC_000003	38568238	38568851	0	0	0	0
SCN5A	NC_000003	38589553	38691163	0.0306758	0.0210253	0.0227417	0.0285015
SCN10A	NC_000003	38738837	38835501	0.0149636	0.0461526	0.006656	0.0469227
SCN11A	NC_000003	38887260	38992052	0	0.0139026	0.01203	0.0141346
WDR48	NC_000003	39093507	39137882	5.3804444	5.5806152	9.9332385	9.008293
GORASP1	NC_000003	39138091	39149130	4.9310577	4.700138	4.7704913	8.1546127
TTC21A	NC_000003	39149152	39180394	0.15482	0.0848913	0.2295536	0.2229618
CSRNP1	NC_000003	39183342	39195102	3.6945189	3.2881271	1.20187	1.4217321
XIRP1	NC_000003	39224707	39234077	0.0613136	0.0420246	0.0242428	0.0094946
LOC729454	NC_000003	39238020	39256163	0	0	0.1030239	0
CX3CR1	NC_000003	39304985	39321527	0.0282807	0.0145378	0.037739	0.0394143
CCR8	NC_000003	39371197	39375171	0.029555	0.0303856	0	0.0205951
hnRNPA1p	NC_000003	39376376	39377467	0	0	0	0
LOC645715	NC_000003	39400036	39401707	0	0	0	0
SLC25A38	NC_000003	39424815	39438819	2.6898635	3.2334638	4.8595844	7.699475
RPSA	NC_000003	39448204	39454033	190.39125	243.84068	192.42002	251.88762
SNORA6	NC_000003	39449882	39450030	0	0	0	0
SNORA62	NC_000003	39452545	39452698	0	0	0	0
MOBP	NC_000003	39509070	39567859	0.0142828	0.0146842	0	0.059717
LOC100288488	NC_000003	39543588	39544513	0	0	0	0

LOC100132681	NC_000003	39685129	39686196	0	0	0	0
MYRIP	NC_000003	39851303	40301812	0.2075584	0.0927791	0.128452	0.3270008
EIF1B	NC_000003	40351173	40353915	6.7746286	7.4115066	8.6926406	7.14177
LOC100129750	NC_000003	40364281	40369039	0	0	0	0
ENTPD3	NC_000003	40428673	40470110	0.1101459	0.0323547	0.0279968	0.0548243
RPL14	NC_000003	40498801	40503859	26.433979	47.476006	52.074982	71.414885
ZNF619	NC_000003	40518633	40530117	0.6630488	0.6292466	0.4991179	0.4738859
RNU5B-2P	NC_000003	40540408	40540494	0	0	0	0
ZNF620	NC_000003	40547530	40559200	0.6599999	0.2594453	0.5353465	0.4328605
ZNF621	NC_000003	40566376	40581043	1.4517754	1.5255023	1.5906831	1.989836
RPL5P10	NC_000003	40627960	40628958	0	0	0	0
LOC651628	NC_000003	40637698	40638985	0	0	0	0
LOC729505	NC_000003	40739739	40752050	0.1062407	0.0364089	0.0945147	0.6909723
RPS27P4	NC_000003	40803003	40803347	0	0	0	0
MRPS31P1	NC_000003	41230521	41230733	0	0	0	0
CTNNB1	NC_000003	41240942	41281939	32.902104	44.782581	27.084523	29.579352
ULK4	NC_000003	41288090	42003660	1.5627382	1.5237348	1.291592	2.4027778
LOC100288588	NC_000003	41996136	42003922	0	0	0	0.0528927
LOC729032	NC_000003	41996937	41997290	0	0	0	0
LOC645874	NC_000003	42015958	42019655	0	0	0	0
LOC100287063	NC_000003	42051943	42128865	8.1678368	7.4763902	1.9689422	5.8018355
TRAK1	NC_000003	42132746	42267268	6.5292334	5.2827063	1.8973747	4.561233
CCK	NC_000003	42299318	42306395	0	0	0	0
LOC100287105	NC_000003	42305981	42307604	0.0512815	0	0	0
LOC391530	NC_000003	42309597	42364187	0	0	0	0
LOC100130064	NC_000003	42382041	42383859	0	0	0	0
LYZL4	NC_000003	42438575	42452065	0	0	0	0
RPL35AP8	NC_000003	42511132	42511374	0	0	0	0
VIPR1	NC_000003	42544104	42579065	0	0.0161948	0	0.0219533
SEC22C	NC_000003	42589472	42623428	2.3085647	3.2305253	3.0426121	3.8727959
SS18L2	NC_000003	42632298	42636490	0.8627874	0.9979156	1.8709265	1.1648727
NKTR	NC_000003	42642147	42690233	1.1435448	1.0086133	1.3011104	2.9819623
ZBTB47	NC_000003	42700874	42709072	2.5950384	1.9794633	1.163618	2.0635335
KBTBD5	NC_000003	42727011	42733938	0.1090076	0.0933927	0	0.0253203
LOC100287203	NC_000003	42727032	42733367	0.3193332	0.4103851	0.2485767	0.2225241
HHATL	NC_000003	42734155	42743013	0	0.0774573	0	0
CCDC13	NC_000003	42749874	42814745	0.0155294	0.0638635	0.0967078	0.0757506
HIGD1A	NC_000003	42824400	42846027	11.43661	14.627294	13.130639	9.6135052
CCBP2	NC_000003	42850964	42908775	0	0	0.0132086	0.0103462
CYP8B1	NC_000003	42913684	42917633	0.0222523	0	0	0.0155063
LOC729102	NC_000003	42930853	42931798	0	0	0	0
ZNF662	NC_000003	42947402	42960825	0.1642925	0.1228436	0.0132872	0.0208156
LOC729085	NC_000003	43020759	43099207	0.0128017	0.1316149	0.0341662	0.0267622
C3orf39	NC_000003	43120729	43147565	4.2770738	6.5336125	5.9924953	8.8327072
SNRK	NC_000003	43328004	43392635	1.1795921	1.3166942	1.694028	1.6381022
ANO10	NC_000003	43407818	43663560	7.0085695	7.9988173	15.258633	14.808372
RPL18AP9	NC_000003	43526412	43527407	0	0	0	0
ABHD5	NC_000003	43732375	43764217	1.1001425	1.4180481	1.9282192	3.3868724
LOC375337	NC_000003	44283378	44373590	0.0082393	0	0.0073299	0.0229658
C3orf23	NC_000003	44379611	44450940	2.7552995	1.9407968	3.4016526	3.9071769
ZNF445	NC_000003	44481262	44519162	0.7332567	1.0360132	0.7019175	1.2340789
ZNF852	NC_000003	44540462	44552132	0.3120057	0.2978622	0.337048	0.7299033
LOC100131881	NC_000003	44566071	44567246	0	0	0	0
LOC100287268	NC_000003	44595763	44597062	0	0.0347565	0	0.0235576
ZNF167	NC_000003	44596713	44624975	0.4200284	0.5227456	0.0590004	0.0077024
ZNF660	NC_000003	44626456	44637557	0.1993117	0.4098268	0.0177313	0.0555553
ZNF197	NC_000003	44666511	44689963	0.7153394	0.8782488	1.143023	1.2921685
ZNF35	NC_000003	44690233	44702283	1.3604209	1.8080183	1.564493	1.6300898
LOC100127945	NC_000003	44750437	44752378	0	0	0	0
ZNF502	NC_000003	44754135	44765323	1.3427507	1.2976596	0.8122928	0.7204753
ZNF501	NC_000003	44771124	44778575	0.5165316	0.6933137	0.5233432	0.3699381
KIAA1143	NC_000003	44790236	44803173	3.2997505	3.4803788	3.1180708	5.194494
KIF15	NC_000003	44803209	44894748	0.2998329	0.7753199	0.8568001	1.6461584
TMEM42	NC_000003	44903408	44907156	3.4327499	2.8791068	5.5050015	3.7769634
PPIAP18	NC_000003	44914039	44916199	0	0	0	0
TGM4	NC_000003	44916098	44956088	0.0293086	0.0753308	0.0391106	0.0919053

ZDHHC3	NC_000003	44956753	45017674	1.7945124	2.1761831	1.8953967	3.1745006
EXOSC7	NC_000003	45017741	45054158	2.4180012	3.3146125	3.2832898	3.7837687
LOC100288769	NC_000003	45048927	45052963	0.1147473	0	0.2041648	0
CLEC3B	NC_000003	45067759	45077563	25.842798	2.0682543	0.4130025	0.8267279
RNU5B-3P	NC_000003	45076010	45076081	0	0	0	0
LOC100288800	NC_000003	45118041	45120487	0	0.0184648	0.0159778	0.0750917
CDCP1	NC_000003	45123769	45187914	3.4927613	2.323966	20.297406	14.034153
RPS24P8	NC_000003	45201190	45201706	0	0	0	0
TMEM158	NC_000003	45265956	45267814	4.3971873	10.086666	26.562789	10.032572
LARS2	NC_000003	45430075	45590328	2.1749303	2.4618128	1.3395312	2.120352
LOC100130135	NC_000003	45635870	45646265	1.7260635	1.2285517	2.8348688	3.4695859
LIMD1	NC_000003	45636323	45722755	3.4129117	2.2289711	5.5622571	9.6153541
SACM1L	NC_000003	45730754	45786900	4.1968368	4.2294687	4.7144893	5.1468301
SLC6A20	NC_000003	45796941	45838035	0.0242719	0.008318	0.0143953	0.0902058
LZTFL1	NC_000003	45865446	45883621	1.0346847	0.9041999	1.541811	1.7844985
SDHDP4	NC_000003	45924319	45925617	0	0	0	0
CCR9	NC_000003	45928019	45944667	0.069101	0.0177608	0	0.1083427
FYCO1	NC_000003	45959395	46037307	1.5036961	1.1900155	2.1100274	4.1373292
CXCR6	NC_000003	45984973	45989845	0	0	0	0.0156809
LOC100288867	NC_000003	46058503	46062157	0.0601207	0.0494483	0.1069701	0.0335156
XCR1	NC_000003	46062291	46068979	0.0640178	0.0329085	0.1139041	0.0223051
LOC100114903	NC_000003	46064604	46066403	0	0	0	0
LOC391533	NC_000003	46183849	46185282	0	0	0	0
CCR1	NC_000003	46243200	46249832	0.8375784	0.050654	5.1136577	0.3204397
CCR3	NC_000003	46283688	46308162	0.1129775	0	0.0603047	0.0157454
LOC100131327	NC_000003	46352145	46367474	0	0	0	0
CCR2	NC_000003	46395235	46402413	0.0245452	0.0378526	0	0.008552
CCR5	NC_000003	46411633	46417697	0.0480177	0.0246836	0.0533974	0.0167303
CCRL2	NC_000003	46448721	46451014	0.5181464	0.024214	0.6285782	0.5580096
LTF	NC_000003	46477496	46506395	0.1116855	0	0.4305535	0.090798
RTP3	NC_000003	46539485	46542439	0	0.055304	0.1435651	0.1124537
LRRC2	NC_000003	46556876	46621572	0.5771656	0.4385903	0.7069411	1.6962009
LUZPP1	NC_000003	46599202	46599917	0	0	0	0
TDGF1	NC_000003	46619180	46623953	0	0.0219337	0	0
LOC100132146	NC_000003	46666613	46667698	0	0	0	0
ALS2CL	NC_000003	46710679	46735171	0.7811441	0.6570804	1.0265974	0.8350553
TMIE	NC_000003	46742823	46752413	0.0944615	0.0242791	0.0630267	0.0164561
TSP50	NC_000003	46753606	46759373	0	0	0	0.0227356
LOC100287362	NC_000003	46761239	46777913	0	0	0	0
TESSP5	NC_000003	46783581	46786245	0	0	0	0.032373
LOC100288960	NC_000003	46795220	46798357	0.0356433	0.0366451	0.0317093	0.0496754
LOC729756	NC_000003	46851046	46853976	0.0488314	0	0	0.0340276
TESSP2	NC_000003	46871894	46875585	0.0498279	0	0	0.1041663
MYL3	NC_000003	46899357	46904973	0	0.0486366	0	0.0329654
PTH1R	NC_000003	46919236	46945289	0.227176	0.1910952	0.1102375	0.0287828
CCDC12	NC_000003	46963221	47018238	1.1990621	2.036737	1.159477	1.9254075
NBEAL2	NC_000003	47021173	47051193	0.8513819	0.3685516	1.8603124	1.4779878
LOC100129354	NC_000003	47053032	47054957	0	0	0	0
SETD2	NC_000003	47057898	47205467	3.6115409	5.020378	5.5496011	6.8926858
MRP63P3	NC_000003	47070334	47070529	0	0	0	0
KIF9	NC_000003	47269516	47324337	0.1219768	0.2508099	0.1302167	0.2209955
RNU13P3	NC_000003	47292015	47292116	0	0	0	0
KLHL18	NC_000003	47324330	47388306	1.8784475	3.0498318	2.4322227	2.6503545
LOC100287401	NC_000003	47422354	47453176	0.3972722	0.3573828	0.2208902	0.6574833
PTPN23	NC_000003	47422491	47454931	2.1126549	2.8244992	4.122962	4.2885304
SCAP	NC_000003	47455184	47517445	8.61406	10.926844	8.3800188	10.400223
C3orf75	NC_000003	47537130	47555199	6.7111458	10.751573	4.3184116	12.054715
CSPG5	NC_000003	47603728	47620359	0.4073052	0.3140644	0.7065825	0.4115484
SMARCC1	NC_000003	47627378	47823405	2.1832889	3.9006689	2.9881759	6.3941469
LOC100288999	NC_000003	47823483	47825474	0.0441247	0.0680473	0	0.1383654
DHX30	NC_000003	47844651	47891683	5.4295018	7.7470635	4.9112456	4.965339
LOC100289035	NC_000003	47886992	47887985	0	0.0655782	0.1134908	0
MAP4	NC_000003	47892180	48130769	16.618676	16.690699	15.25446	24.022197
RPL17P16	NC_000003	48162119	48162777	0	0	0	0
CDC25A	NC_000003	48198668	48229801	1.0317043	1.6337226	0.8334343	1.3552294
NDUFB1P	NC_000003	48222961	48223281	0	0	0	0

CAMP	NC_000003	48264862	48266975	0	0	0	0
ZNF589	NC_000003	48282596	48312480	0.9534089	0.496816	1.2432213	1.6108777
MRPS18AP1	NC_000003	48297530	48298439	0	0	0	0
LOC100289064	NC_000003	48331571	48333003	0	0	0	0
NME6	NC_000003	48335576	48342848	5.465643	6.4220064	6.2847187	5.9591557
SPINK8	NC_000003	48348336	48369831	0	0	0	0
FBXW12	NC_000003	48413709	48436190	0.0533676	0	0	0
PLXNB1	NC_000003	48445261	48471460	1.147411	0.5959736	0.7762095	0.4705754
LOC100287439	NC_000003	48454094	48455061	0.05069	0.156344	0	0.1059684
CCDC51	NC_000003	48473580	48481529	1.4882867	2.0701559	1.5836331	1.3217909
CCDC72	NC_000003	48481686	48485537	1.61365	1.9517668	0.6755519	1.5874667
ATRIP	NC_000003	48488218	48507054	2.6799838	4.1419619	2.2127757	3.9913652
TREX1	NC_000003	48507229	48509044	11.834078	9.1063464	8.5256806	5.8180523
SHISA5	NC_000003	48509197	48541661	48.919292	31.165283	47.8882	42.784341
PFKFB4	NC_000003	48555117	48594227	3.2866729	2.2526968	2.8679017	5.4054226
UCN2	NC_000003	48599151	48601201	0.2367582	0.2129857	0	0.1031141
COL7A1	NC_000003	48601506	48632593	0.4841064	1.1876104	0.247318	0.2104229
UQCRC1	NC_000003	48636432	48647098	23.781511	28.417761	39.097566	45.227093
TMEM89	NC_000003	48658275	48659189	0	0.0941321	0	0
SLC26A6	NC_000003	48663156	48672926	2.6612251	2.3138905	3.0033424	4.0268006
CELSR3	NC_000003	48673896	48700348	0.8854309	1.0085244	0.6863809	0.8397392
NCKIPSD	NC_000003	48711277	48723334	8.9290704	6.984137	10.133962	13.426162
IP6K2	NC_000003	48725436	48754706	5.0037547	5.737298	4.0440554	6.1817103
PRKAR2A	NC_000003	48788093	48885270	7.586192	12.961051	9.5075559	14.186997
SLC25A20	NC_000003	48894356	48936402	0.9410756	0.7498316	0.8999975	0.9016961
C3orf71	NC_000003	48955221	48956818	0.1375101	0.1413748	0.0978662	0.076658
ARIH2	NC_000003	48956281	49022974	5.7408391	6.6357911	5.7708584	7.5789982
P4HTM	NC_000003	49027341	49044582	4.9665544	2.5332788	7.8777399	4.8828102
WDR6	NC_000003	49044637	49053386	5.3864703	7.6278154	5.728312	11.167043
LOC100287476	NC_000003	49044819	49052379	1.4189564	0.4862788	0.3506508	0.5218589
DALRD3	NC_000003	49052435	49058467	9.5429411	10.59306	8.3607897	8.1136369
NDUFAF3	NC_000003	49057908	49060928	9.4088399	7.4352434	5.0351295	4.2810782
IMPDH2	NC_000003	49061758	49066875	7.6488073	12.7953	11.44094	17.507674
QRICH1	NC_000003	49067142	49131504	22.033774	24.983763	18.705502	22.449006
QARS	NC_000003	49133365	49142171	12.895098	16.920533	9.7019885	20.845082
USP19	NC_000003	49146108	49158213	5.7219579	6.8067702	6.8316355	7.5640179
LAMB2	NC_000003	49158547	49170599	45.4162	22.350536	48.84503	20.785687
LAMB2L	NC_000003	49190292	49191834	0	0	0	0
CCDC71	NC_000003	49199968	49203785	6.3545457	6.5580765	5.610026	5.4928728
KLHDC8B	NC_000003	49209068	49213919	4.7695755	6.117856	4.7280777	5.1120598
LOC646498	NC_000003	49215069	49227421	0	0	0.1907198	0
CCDC36	NC_000003	49235861	49295537	1.1746268	0.8541843	0.420541	0.8484728
STGC3	NC_000003	49298046	49298811	0	0	0	0
C3orf62	NC_000003	49306030	49314508	0.159144	0.1006872	0.1415789	0.238857
USP4	NC_000003	49314577	49377536	3.5414595	3.608092	3.5206788	4.9579597
LOC100289130	NC_000003	49385350	49395460	0.8597468	0.9359048	1.3497433	1.6915928
GPX1	NC_000003	49394609	49395791	77.048717	64.203972	30.405546	27.388944
RHOA	NC_000003	49396578	49449526	195.046	203.3018	161.98435	268.91575
TCTA	NC_000003	49449639	49453909	7.3281169	5.7873477	3.5692235	5.9195736
AMT	NC_000003	49454211	49460012	0.501549	0.1503965	0.5019659	0.5679364
LOC100289156	NC_000003	49457273	49459445	0.0428345	0.0880768	0	0.0298488
NICN1	NC_000003	49459766	49466757	2.342453	0.8961071	2.0112166	2.0214128
DAG1	NC_000003	49507565	49573048	14.222291	14.736564	25.885675	37.280049
LOC100132677	NC_000003	49591002	49591768	0	0	0	0
BSN	NC_000003	49591922	49708982	0.0220306	0.0283122	0.0293985	0.0383794
APEH	NC_000003	49711435	49720934	12.413224	12.663803	8.3213455	13.724565
MST1	NC_000003	49721380	49726196	0.5467605	0.581511	0.3354574	0.8408375
RNF123	NC_000003	49726990	49758962	3.1605544	3.2069066	2.5360583	5.9162515
AMIGO3	NC_000003	49754267	49757238	0	0	0	0.0206089
GMPPB	NC_000003	49758932	49761384	6.5574402	8.4478102	4.6669445	5.7258451
IP6K1	NC_000003	49761728	49823973	2.7443054	4.20687	5.0840836	6.2373857
LOC100287546	NC_000003	49764502	49765829	0	0	0.109517	0.128676
PHF5EP	NC_000003	49812689	49813509	0	0	0	0
CDH29	NC_000003	49828165	49837254	0.1242478	0.1094913	0.2526499	0.2968486
C3orf54	NC_000003	49840687	49842463	1.9016062	3.0411905	1.9172845	1.1484329
UBA7	NC_000003	49842638	49851391	11.514357	5.2370741	7.4937984	4.5387308

TRAIP	NC_000003	49866028	49893992	2.3450638	4.464763	2.6464261	3.3590529
CAMKV	NC_000003	49895422	49907369	0.0290279	0.0149219	0	0.0101139
LOC389120	NC_000003	49910777	49914738	0	0	0	0
MST1R	NC_000003	49924435	49941306	0.0184192	0.0378738	0.0573518	0.0128352
MON1A	NC_000003	49946302	49967445	2.60142	3.788922	2.3844255	2.7740921
RBM6	NC_000003	49977592	50114682	1.0073745	0.9483398	1.176922	1.3616695
RBM5	NC_000003	50126368	50156393	2.9204309	2.9587841	2.3584661	4.0207501
LOC100129060	NC_000003	50188168	50190541	0	0	0	0
SEMA3F	NC_000003	50192848	50226508	6.9747194	2.7115181	13.777555	10.06893
GNAT1	NC_000003	50229043	50235129	0.0244225	0.0125544	0	0.0085093
SLC38A3	NC_000003	50242692	50258406	0.0895806	0.1657769	0.047816	0.0249693
GNAI2	NC_000003	50273647	50296786	41.670914	38.32374	46.38393	33.965774
LOC100287609	NC_000003	50297429	50298839	0	0.4920153	0.1419149	0.2223222
SEMA3B	NC_000003	50305040	50314573	5.7506569	0.4167926	8.268327	2.0507264
C3orf45	NC_000003	50316518	50325545	0.306473	0.0945259	0.1635881	0.2135626
IFRD2	NC_000003	50325163	50330026	10.815141	14.182746	8.1198358	15.798894
HYAL3	NC_000003	50330262	50336899	3.2135164	2.070712	1.4898146	2.4285434
NAT6	NC_000003	50333833	50336712	0.0367768	0.2646726	0.0327176	0.1537651
HYAL1	NC_000003	50337320	50349812	0.1353642	0.1855581	0.1873258	0.0314424
HYAL2	NC_000003	50355227	50360281	19.152773	26.344855	13.206449	12.275319
TUSC2	NC_000003	50362341	50365668	3.9715406	3.7339434	4.8581398	5.043455
RASSF1	NC_000003	50367217	50378367	11.812025	9.6573739	8.6735214	9.4461549
ZMYND10	NC_000003	50378537	50383156	0.2230875	0.2293574	0.3969296	0.2418208
TUSC4	NC_000003	50384919	50388486	3.5919228	4.1001765	4.7462189	5.1716291
CYB561D2	NC_000003	50388297	50391500	5.3292706	5.1788285	3.7668751	5.7993956
TMEM115	NC_000003	50392180	50396939	3.1676291	4.7016619	2.4820889	2.8212895
CACNA2D2	NC_000003	50400230	50540892	0.0493431	0.008455	0.0804778	0.0859605
C3orf18	NC_000003	50595462	50605182	2.3008679	1.4805861	1.6051355	1.8917062
HEMK1	NC_000003	50606909	50622422	1.3269937	1.2332556	1.0671461	1.7292451
CISH	NC_000003	50643885	50649262	0.2712854	0.8367297	0.3275369	0.3240728
MAPKAPK3	NC_000003	50654601	50686719	21.341261	17.097399	17.50007	32.73187
DOCK3	NC_000003	50712672	51421629	0.4266819	0.2012739	1.7461049	2.4311011
ARMET	NC_000003	51422703	51426775	24.410104	30.25997	23.145759	23.729906
RBM15B	NC_000003	51428731	51435339	5.133611	6.3307354	4.5906659	4.5550394
VPRBP	NC_000003	51433298	51534001	3.6516642	3.9404581	4.4030907	5.2364599
RAD54L2	NC_000003	51575596	51697634	1.171457	1.3859939	1.6955788	2.3323368
TEX264	NC_000003	51705222	51738339	10.196734	10.706363	8.740429	9.9779223
GRM2	NC_000003	51741081	51752629	0	0.0134514	0.0116396	0.0182345
LOC100289270	NC_000003	51742875	51746701	0	0	0.0495533	0
IQCF6	NC_000003	51812577	51813009	0	0	0.0902946	0.0707272
LOC100287638	NC_000003	51823154	51853638	0	0	0	0
IQCF3	NC_000003	51862569	51864876	0	0.0522351	0	0.0354045
IQCF2	NC_000003	51895645	51897440	0	0	0	0
LOC100129058	NC_000003	51907612	51908285	0	0	0	0
IQCF5	NC_000003	51907737	51909600	0	0.0882488	0	0
IQCF1	NC_000003	51928892	51937351	0	0	0	0.0440646
RRP9	NC_000003	51967446	51975922	7.1490643	11.918111	5.3464591	7.6214862
PARP3	NC_000003	51976361	51982883	1.7318583	1.3784767	1.1762403	1.673987
GPR62	NC_000003	51989330	51991520	0.0200585	0.0206223	0.0535339	0.0838655
PCBP4	NC_000003	51991474	52001469	4.4573829	5.2419178	5.5098349	7.1712348
LOC100287670	NC_000003	52002527	52008147	5.0709498	2.8740921	5.6679903	2.1745476
ABHD14B	NC_000003	52002531	52008030	9.8980324	6.7426773	18.536896	11.349418
LOC100289334	NC_000003	52008747	52012089	0.0645348	0.1990458	0.057412	0.0899409
ABHD14A	NC_000003	52009084	52015212	8.9800059	6.126563	4.1600988	3.950667
ACY1	NC_000003	52017563	52023218	9.3176463	8.0467967	6.6866508	5.6704722
RPL29	NC_000003	52027644	52029958	57.101084	75.349011	95.8031	62.263242
RPL29P10	NC_000003	52027765	52028142	0	0	0	0
DUSP7	NC_000003	52084759	52090289	3.6889557	5.1054691	3.8813564	8.4286388
C3orf74	NC_000003	52097076	52097567	0	0	0	0
WDR51A	NC_000003	52109270	52188420	7.0261334	10.261344	8.6929474	13.89387
ALDOAP1	NC_000003	52227063	52229012	0	0	0	0
ALAS1	NC_000003	52232116	52248343	1.6063214	1.7628021	2.5850957	2.2890053
TLR9	NC_000003	52255098	52260179	0	0.0467253	0.0202159	0.0237525
TWF2	NC_000003	52262626	52273183	19.391312	22.772435	20.451034	14.963787
PPM1M	NC_000003	52280225	52284615	5.7753172	5.4299545	5.5007811	2.9921718
WDR82	NC_000003	52288438	52312659	4.4542127	6.1585019	4.791547	6.321911

GLYCTK	NC_000003	52321836	52329272	0.7521419	0.4758652	0.5147126	1.1288791
DNAH1	NC_000003	52350335	52434513	0.1810225	0.1447523	0.2415639	0.2919992
PPP2R5CP	NC_000003	52407670	52409060	0	0	0	0
BAP1	NC_000003	52435024	52444009	0.7968299	0.9704664	0.8179407	2.033116
LOC100287700	NC_000003	52436887	52438519	0	0.047066	0	0.0638018
PHF7	NC_000003	52444527	52457657	0.7723767	0.6948238	0.7730187	0.7535119
SEMA3G	NC_000003	52467268	52479043	0.0093507	0.0288405	0.0415932	0.0260637
TNNC1	NC_000003	52485107	52488057	0	0	0.0561747	0.0880025
NISCH	NC_000003	52489524	52527088	5.0835328	4.0017367	4.4179727	4.7524386
STAB1	NC_000003	52529356	52558511	0.060993	0.0456053	0.0295969	0.0502301
NT5DC2	NC_000003	52558385	52569093	17.20685	28.239626	13.849816	40.78559
LOC440957	NC_000003	52570621	52574586	3.8173297	3.823985	3.1347714	1.0231028
PBRM1	NC_000003	52579368	52719866	2.2627938	2.4549843	3.4393719	4.5957127
GNL3	NC_000003	52719936	52728513	14.875102	18.07758	19.6583	19.85899
SNORD19	NC_000003	52723256	52723331	0	0	0	0
SNORD19B	NC_000003	52724754	52724846	0	0	0	0
SNORD69	NC_000003	52726752	52726828	0	0	0	0
GLT8D1	NC_000003	52728504	52740048	10.48456	11.859014	13.757446	10.927542
SPCS1	NC_000003	52739857	52742198	16.526155	23.528684	20.864047	21.225722
NEK4	NC_000003	52744800	52804951	0.7585455	1.2185383	1.0965876	1.4288306
LOC100130124	NC_000003	52787542	52788710	0	0	0	0
ITIH1	NC_000003	52811608	52826077	0.0151025	0.0310539	0.0134356	0.0736681
ITIH3	NC_000003	52828784	52843025	0.0868256	0.0148776	0.0257475	0.0201678
ITIH4	NC_000003	52847006	52864694	0.0539242	0.0415798	0.0119931	0.0563648
MUSTN1	NC_000003	52867137	52869220	0.0620738	0.1276367	0.4417804	0.4325548
TMEM110	NC_000003	52873898	52931547	4.2889909	4.6099669	3.3448488	4.8324428
SFMBT1	NC_000003	52938627	53080070	1.4623364	1.2624268	1.5293434	1.524632
LOC401068	NC_000003	53044412	53082201	0	0	0	0
LOC553148	NC_000003	53092184	53099107	0	0	0	0
LOC100287762	NC_000003	53117572	53124780	0.027571	0	0.2207516	0.0384252
RFT1	NC_000003	53122499	53164470	2.8178572	3.6058127	3.2504643	3.3146927
PRKCD	NC_000003	53195223	53226733	1.410684	1.0997018	1.3928939	1.6311664
TKT	NC_000003	53258723	53290068	22.975931	25.410965	37.406157	28.158582
DCP1A	NC_000003	53317449	53381637	1.1296992	1.2368683	2.3167478	1.8811476
RPS25P4	NC_000003	53508684	53509175	0	0	0	0
CACNA1D	NC_000003	53529031	53846492	0.0055829	0.0286988	0.0198666	0.0233421
CHDH	NC_000003	53850387	53880419	0.20514	0.3225614	0.4938188	0.6222518
IL17RB	NC_000003	53880577	53899827	0.430865	1.0409901	0.7857844	1.306061
ACTR8	NC_000003	53901155	53916187	1.6955914	2.6276872	1.2311574	1.3118742
SELK	NC_000003	53919226	53925989	8.4350518	12.53867	15.151502	16.136091
CABYRP	NC_000003	54081685	54082888	0	0	0	0
CACNA2D3	NC_000003	54156693	55108584	0.0119587	0.0122948	0.0212776	0.0416665
RPS15P5	NC_000003	54660180	54660707	0	0	0	0
HESRG	NC_000003	54666151	54673884	0	0	0	0
LRTM1	NC_000003	54952360	54962072	0	0	0	0
WNT5A	NC_000003	55499743	55521331	11.547891	9.7285948	1.305931	0.77113
LOC100289467	NC_000003	55521727	55522538	0.9390648	3.8618292	0.3341672	0
ERC2	NC_000003	55542336	56502391	0.0214801	0	0.0191093	0.0199576
C3orf51	NC_000003	55691234	55693497	0	0	0	0
CCDC66	NC_000003	56591184	56655864	0.404346	0.6737373	0.7194349	1.0396136
C3orf63	NC_000003	56654160	56717135	2.8814405	3.7197738	4.0702483	5.2065785
ARHGEF3	NC_000003	56761446	57113336	1.1505271	0.7234037	1.4211145	1.6498474
SPATA12	NC_000003	57094469	57109460	0.0182131	0.018725	0	0
IL17RD	NC_000003	57124010	57199403	0.1411182	0.1658106	0.2735036	0.5654364
RPL19P2	NC_000003	57168131	57168830	0	0	0	0
HESX1	NC_000003	57231944	57234280	0.0374665	0.2311171	0.3666438	0.156649
APPL1	NC_000003	57261765	57307499	4.6406316	5.9638214	6.5990447	7.5539008
ASB14	NC_000003	57302379	57326686	0.0828691	0.1277972	0.1105839	0.1732394
DNAH12	NC_000003	57327727	57530071	0.0479737	0.0358705	0.0155195	0.0577427
RPS8P5	NC_000003	57516020	57516700	0	0	0	0
PDE12	NC_000003	57541981	57547768	4.5676224	5.7483517	4.2495225	5.0773026
ARF4	NC_000003	57557090	57583215	50.995103	49.857547	57.657674	53.804743
FAM116A	NC_000003	57611181	57678816	1.3209001	1.1640207	1.854994	1.5779242
LOC100287789	NC_000003	57741948	57742942	1.0386584	2.4026624	1.2705265	1.5832655
SLMAP	NC_000003	57743174	57914894	2.823823	4.4570053	4.8296993	5.2311726
LOC100132534	NC_000003	57785781	57787176	0	0	0	0

PPIAP16	NC_000003	57927409	57928275	0	0	0	0
FLNB	NC_000003	57994127	58157978	10.333384	21.586617	10.90749	24.330322
DNASE1L3	NC_000003	58178354	58200398	0.2703582	0	0.0801727	0.0627988
ABHD6	NC_000003	58223259	58280461	2.2589354	1.6804525	3.2349845	3.0202554
RPP14	NC_000003	58291972	58305920	1.004531	1.7169693	2.0219027	2.1262415
PXK	NC_000003	58318617	58410878	6.7429206	7.0438851	16.317978	16.422912
PDHB	NC_000003	58413354	58419565	6.0588262	5.6926318	7.8401451	10.100554
KCTD6	NC_000003	58477823	58488087	2.6502604	1.6585412	3.3418475	2.1358725
ACOX2	NC_000003	58490863	58522916	2.9860297	0.9249216	1.3111988	0.6935948
RPL27P9	NC_000003	58497037	58497414	0	0	0	0
FAM107A	NC_000003	58549844	58563491	0.1648851	0.4042382	0.0677014	0.1148985
FAM3D	NC_000003	58619670	58652561	0	0	0.0298911	0
C3orf67	NC_000003	58727737	59035715	1.447139	1.4523869	2.4522189	3.4574539
LOC339902	NC_000003	59623022	59644223	0	0	0	0
FHIT	NC_000003	59735036	61237133	0.7257506	0.2072633	1.0043411	1.2924261
LOC100132946	NC_000003	60599883	60603834	0	0	0	0
PTPRG	NC_000003	61547243	62280573	4.1251048	3.0244606	4.9754122	5.7122216
RPL10AP6	NC_000003	61728098	61728811	0	0	0	0
ID2B	NC_000003	62109158	62110392	0	0	0	0
C3orf14	NC_000003	62305396	62319321	5.8940988	4.7654369	8.7562256	10.168417
FEZF2	NC_000003	62355347	62359190	0	0.0649809	0.0562285	0.0146811
CADPS	NC_000003	62384021	62861064	0.0079172	0	0.0070433	0.033102
RPL14P2	NC_000003	62764847	62765463	0	0	0	0
RPS10P10	NC_000003	62912018	62912603	0	0	0	0
LOC285401	NC_000003	63088364	63110738	0	0	0	0
LOC100129031	NC_000003	63188894	63189124	0	0	0	0
SYNPR	NC_000003	63263914	63602597	0.0294263	0.0756334	0.0392677	0.071769
SNTN	NC_000003	63638344	63650893	0.0277101	0	0	0.0193095
LOC100130345	NC_000003	63738271	63798811	0.0292403	0.0601243	0	0.0407517
C3orf49	NC_000003	63805041	63834312	0	0	0	0
THOC7	NC_000003	63819546	63849597	14.823808	17.302374	18.191228	14.856712
ATXN7	NC_000003	63850233	63989138	3.4283718	3.1654399	3.9933763	5.6261773
PSMD6	NC_000003	63996231	64009120	12.39824	18.480979	14.736315	16.506528
LOC100287879	NC_000003	64069323	64072924	0	0	0	0.0699198
PRICKLE2	NC_000003	64079526	64211131	0.70765	1.2541896	1.6960131	2.252154
ADAMTS9	NC_000003	64501330	64673365	0.0360576	0.037071	0.0267316	0.0376896
MAGI1	NC_000003	65339906	66024509	0.5560034	0.4336503	0.5415412	0.8583917
RPL17P17	NC_000003	65829537	65830149	0	0	0	0
SLC25A26	NC_000003	66271405	66429351	1.2154533	1.4030751	2.5040653	2.942128
LRIG1	NC_000003	66429221	66550845	8.592147	10.124042	7.159403	7.2221206
LOC100289036	NC_000003	66673116	66673527	0	0	0	0
RPL21P41	NC_000003	66687573	66687966	0	0	0	0
KBTBD8	NC_000003	67049403	67061634	1.0565852	2.0061939	0.2371089	0.1193952
SUCLG2	NC_000003	67425143	67705038	5.9793513	7.0695119	2.4768441	2.8124888
LOC100288654	NC_000003	67564364	67564747	0	0	0	0
FAM19A1	NC_000003	68053454	68594772	0	0.0707834	0.040833	0.0479763
LOC375350	NC_000003	68194142	68194923	0	0	0	0
RPLP0P8	NC_000003	68682646	68683550	0	0	0	0
PSMC1P	NC_000003	68684836	68686393	0	0	0	0
FAM19A4	NC_000003	68780927	68981711	0	0	0	0.0137208
C3orf64	NC_000003	69024368	69062774	4.1632931	2.41313	1.4550568	1.3543627
TMF1	NC_000003	69068978	69101484	2.587445	2.8900562	1.0173665	0.9927821
UBA3	NC_000003	69103881	69129524	14.995079	10.725461	5.295423	5.7087432
ARL6IP5	NC_000003	69134090	69155239	33.89053	18.599934	10.840021	9.3256208
LMOD3	NC_000003	69156039	69171746	0.0964953	0.1322764	0.1430748	0.0597704
FRMD4B	NC_000003	69219146	69435430	0.0751823	0.0085884	0.0074316	0.1338856
LOC100131568	NC_000003	69519576	69521682	0	0	0	0
LOC642487	NC_000003	69584810	69591851	0	0	0	0
MITF	NC_000003	69788633	70017487	1.6876939	0.8500368	0.8037901	1.2057506
LOC654340	NC_000003	70174216	70175762	0	0	0	0
LOC100128448	NC_000003	70189368	70189639	0	0	0	0
LOC100128160	NC_000003	70246077	70264477	0	0	0.0757705	0
LOC100289131	NC_000003	70800274	70800501	0	0	0	0
FOXP1	NC_000003	71004736	71633140	12.247081	12.196487	3.1844171	4.6828492
EIF4E3	NC_000003	71728440	71803924	0.1807192	0.3303081	0.0297727	0.0326491
GPR27	NC_000003	71803201	71804328	0.0389612	0.1201686	0.0693219	0.0271497

PROK2	NC_000003	71820806	71834357	0	0	0.024239	0
CCDC137P	NC_000003	72136041	72136811	0	0	0	0
RYBP	NC_000003	72423748	72495774	6.5848194	6.3836167	1.8471291	1.749993
SHQ1	NC_000003	72798428	72897598	2.5307268	3.3633712	0.7275881	1.2688679
PSMD12P	NC_000003	72925820	72927337	0	0	0	0
LOC100289157	NC_000003	72933373	72933833	0	0	0	0
GLT8D4	NC_000003	72937385	73024525	6.3635449	6.6300144	2.1987642	1.9004449
PPP4R2	NC_000003	73046119	73115011	4.3888111	7.1081959	1.8184914	1.5919909
RNU7-19P	NC_000003	73076962	73077021	0	0	0	0
FLJ10213	NC_000003	73110810	73112488	0	0	0	0
LOC344709	NC_000003	73231624	73232442	0	0	0	0
PDZRN3	NC_000003	73431652	73674072	0.6442783	0.2714696	0.1033581	0.1177597
LOC100289228	NC_000003	73442425	73483146	0.1440926	0.0740712	0.0640944	0
LOC100129282	NC_000003	73673149	73674444	0.0978802	0.1509468	0.0435385	0
LOC100289304	NC_000003	73677479	73697876	0	0	0	0
HSP90AB5P	NC_000003	74150457	74152906	0	0	0	0
AKR1B1P2	NC_000003	74185083	74186739	0	0	0	0
CNTN3	NC_000003	74311722	74570343	0.4837208	0.5696527	0.1643084	0.3493332
LOC100130204	NC_000003	75132811	75134282	0	0	0	0
LOC653115	NC_000003	75263610	75264799	0	0	0	0
MYLKP	NC_000003	75377699	75388223	0	0	0	0
OR7E66P	NC_000003	75397293	75398014	0	0	0	0
OR7E22P	NC_000003	75405636	75406661	0	0	0	0
OR7E55P	NC_000003	75419464	75420614	0	0	0	0
LOC100131901	NC_000003	75428323	75428649	0	0	0	0
LOC339879	NC_000003	75464218	75472067	0	0	0	0
FAM86D	NC_000003	75470703	75484266	0	0	0	0
LOC100128878	NC_000003	75514081	75514254	0	0	0	0
RPS3AP15	NC_000003	75591256	75592104	0	0	0	0
LOC100128045	NC_000003	75624698	75629733	0	0	0	0
OR7E121P	NC_000003	75647702	75648924	0	0	0	0
UNC93B3	NC_000003	75668191	75673001	0	0	0	0
RPL23AP49	NC_000003	75673837	75674304	0	0	0	0
LOC391552	NC_000003	75702583	75705163	0	0	0	0
LOC643255	NC_000003	75707585	75708266	0	0	0	0
LOC100288801	NC_000003	75713531	75718813	0.0766985	0.1577082	0	0
ZNF717	NC_000003	75786029	75834255	0.4727157	0.6038199	0.267617	0.299461
LOC401076	NC_000003	76483169	76484579	0	0	0	0
ROBO2	NC_000003	77089294	77696663	0.0070441	0.0289684	0	0.0294517
MRPS17P3	NC_000003	78574253	78574641	0	0	0	0
ROBO1	NC_000003	78646390	79639061	3.045974	2.0562082	2.96031	3.4000944
RPS12P6	NC_000003	78808128	78808519	0	0	0	0
LOC100130821	NC_000003	80209647	80213945	0	0	0	0
LOC100129557	NC_000003	80265390	80266504	0	0	0	0
LOC728290	NC_000003	81042218	81144576	0	0	0.087077	0
LOC100127895	NC_000003	81416130	81420368	0	0	0	0
GBE1	NC_000003	81538850	81810950	26.731984	24.66935	14.647394	20.643876
RPL7AP23	NC_000003	82368367	82368965	0	0	0	0
CYP51P1	NC_000003	82854162	82857302	0	0	0	0
LOC100130326	NC_000003	84740835	84741742	0	0	0	0
LOC100289598	NC_000003	85006155	85009464	0	0	0.0861813	0
CADM2	NC_000003	85775632	86117951	0.0131897	0.0135604	0.011734	0.0091911
VGLL3	NC_000003	86987123	87040257	30.010436	9.9398269	8.8868361	16.844272
LOC285232	NC_000003	87098348	87102020	0	0	0	0
LOC100289640	NC_000003	87100342	87101966	0	0	0	0
CHMP2B	NC_000003	87276413	87304698	4.8664138	5.5533633	5.4748494	5.0808398
POU1F1	NC_000003	87308783	87325737	0	0.0674379	0.0291773	0.0457088
KRT8P25	NC_000003	87372346	87374056	0	0	0	0
LOC100129005	NC_000003	87590779	87644526	0	0	0	0
LOC643766	NC_000003	87679451	87680769	0	0	0	0
HTR1F	NC_000003	88031726	88042919	0.0138289	0.2274809	0	0.0385461
CGGBP1	NC_000003	88101100	88108146	12.63548	14.463865	18.321331	12.886873
LOC100151661	NC_000003	88144432	88145154	0	0	0	0
ZNF654	NC_000003	88188262	88193814	1.7731786	2.3334579	3.9988836	4.1269757
C3orf38	NC_000003	88198893	88207115	10.84502	9.6895204	14.137561	10.223819
LOC344653	NC_000003	88366306	88368172	0	0	0	0

LOC100130327	NC_000003	88650211	88650552	0	0	0	0
LOC728432	NC_000003	88849024	89000078	0	0	0	0
EPHA3	NC_000003	89156674	89531284	2.6735065	0.0270803	0.1347384	0.1376605
LOC100289000	NC_000003	89636112	89636792	0	0	0	0
LOC100286924	NC_000003	89637025	89637719	0	0	0	0
PROSP	NC_000003	90250256	90291222	0	0	0	0
PROS1	NC_000003	93591881	93692934	3.9897138	2.2213069	9.730707	11.616923
ARL13B	NC_000003	93698982	93774123	1.7388267	3.1977108	3.3879473	2.3636365
STX19	NC_000003	93733213	93747454	4.6680764	1.3656469	0.7427862	0.370249
DHFRL1	NC_000003	93776770	93781660	0.2342656	0.1445098	0.1875683	0.5305483
NSUN3	NC_000003	93781855	93845630	2.0213124	2.5819087	2.642832	3.0944999
LOC389137	NC_000003	94224688	94226464	1.0139997	0.8899376	1.4521324	1.034042
WDR82P1	NC_000003	94654443	94657128	0	0	0	0
LOC255025	NC_000003	94657107	94710172	0	0	0	0
RPS18P6	NC_000003	94890940	94891221	0	0	0	0
LOC100287639	NC_000003	95373867	95385973	0	0	0	0
LOC285216	NC_000003	95393655	95425292	0	0	0	0
LOC644063	NC_000003	96068212	96071099	0	0	0	0
LOC391556	NC_000003	96285201	96285512	0	0	0	0
RPL18AP8	NC_000003	96338113	96338718	0	0	0	0
LOC100131442	NC_000003	96472019	96473366	0	0	0	0
LOC100129736	NC_000003	96495779	96496603	0	0	0	0
EPHA6	NC_000003	96533425	97467786	0.0729345	0.0856964	0.0463461	0.2323367
ARL6	NC_000003	97483595	97517373	0.8771498	1.1505753	1.453041	1.2646199
CRYBG3	NC_000003	97595819	97663810	0.1193996	0.1982971	0.351347	0.8960257
MINA	NC_000003	97660661	97691295	0.872054	1.4886332	2.1298	3.1358683
GABRR3	NC_000003	97705527	97753831	0.0626043	0.1930915	0.0556945	0.0872504
OR5BM1P	NC_000003	97771737	97772662	0	0	0	0
OR5AC1	NC_000003	97783216	97784337	0	0	0	0
OR5AC2	NC_000003	97806017	97806946	0	0	0.0420404	0
OR5AC4P	NC_000003	97823598	97824513	0	0	0	0
POU5F1P7	NC_000003	97838117	97838819	0	0	0	0
OR5H1	NC_000003	97851542	97852483	0.0466542	0	0	0.065021
OR5H14	NC_000003	97868230	97869162	0	0	0.0838104	0
OR5H15	NC_000003	97887544	97888485	0.0466542	0	0	0
OR5H5P	NC_000003	97916055	97916979	0	0	0	0
OR5H3P	NC_000003	97926267	97927182	0	0	0	0
OR5H4P	NC_000003	97940883	97941810	0	0	0	0
OR5H7P	NC_000003	97957196	97958122	0	0	0	0
OR5H6	NC_000003	97983129	97984106	0	0	0	0.0313138
OR5H2	NC_000003	98001732	98002676	0	0	0	0.0324073
OR5H8P	NC_000003	98030757	98031684	0	0	0	0
OR5K4	NC_000003	98072698	98073663	0	0	0	0
OR5K3	NC_000003	98109510	98110475	0.0454951	0	0.0404737	0.0317028
LOC100130484	NC_000003	98152914	98153679	0	0	0	0
OR5K1	NC_000003	98188421	98189372	0	0	0	0
OR5K2	NC_000003	98216525	98217475	0	0.0475115	0	0.1932169
CLDND1	NC_000003	98234317	98241910	2.6451926	2.3640411	8.2593992	6.0719664
LOC100287763	NC_000003	98235925	98244221	0	0.0846131	0.3660821	0
LOC100130963	NC_000003	98243877	98244156	0	0	0	0
GPR15	NC_000003	98250878	98251960	0.0811602	0	0	0.1979447
CPOX	NC_000003	98298290	98312441	2.1036445	3.0681123	3.9460252	4.0681656
RPL19P7	NC_000003	98338920	98339540	0	0	0	0
LOC100287790	NC_000003	98344026	98347278	0	0	0	0
LOC339843	NC_000003	98376646	98379414	0	0	0	0
ST3GAL6	NC_000003	98451572	98513237	1.9719016	1.3587371	3.7511268	5.7010513
DCBLD2	NC_000003	98514814	98620533	62.990302	85.850688	60.818435	76.197793
COL8A1	NC_000003	99357454	99515158	16.583926	4.1817595	7.3988245	5.5419738
C3orf26	NC_000003	99536681	99897447	0.880432	2.5269515	2.1213203	3.6555573
FILIP1L	NC_000003	99551988	99833349	0.0985386	0.1924853	0.4470798	1.1604494
TMEM30C	NC_000003	99904531	99909039	0	0	0	0.068512
LOC391559	NC_000003	99944218	99944917	0	0	0	0
TBC1D23	NC_000003	99979686	100044082	7.5713024	10.018308	14.695008	13.387309
NIT2	NC_000003	100053562	100074478	7.1921575	10.807045	19.71797	20.288078
TOMM70A	NC_000003	100082307	100120225	7.1466276	10.28026	13.074369	13.352728
LNP1	NC_000003	100120037	100175170	0.277526	0.2649455	0.7935906	1.0360243

TMEM45A	NC_000003	100211463	100296285	8.2613684	5.6623701	7.2745471	11.454958
GPR128	NC_000003	100328445	100414265	0	0.0147177	0.0127354	0.0199511
TFG	NC_000003	100428160	100467811	23.152804	29.970791	40.333408	38.838447
ABI3BP	NC_000003	100468179	100712334	54.284369	9.8690328	11.966145	26.023958
LOC100128020	NC_000003	100881938	100882863	0	0	0	0
IMPG2	NC_000003	100945288	101039419	0.0197343	0	0.0351123	0.0687581
SENP7	NC_000003	101043117	101232085	0.4453611	0.5128234	0.855804	0.9620706
ZNF108P	NC_000003	101076137	101077477	0	0	0	0
LOC100287852	NC_000003	101129780	101133477	0	0	0.0958274	0
LOC643412	NC_000003	101175829	101176690	0	0	0	0
FAM172B	NC_000003	101237716	101242724	0	0	0	0
RG9MTD1	NC_000003	101280712	101285089	6.4886347	5.2414989	8.7799744	9.6510162
PCNP	NC_000003	101293042	101313281	2.23765	3.4508092	4.5575992	7.7964183
RPS18P5	NC_000003	101295295	101295834	0	0	0	0
RPL32P7	NC_000003	101318083	101318488	0	0	0	0
ZBTB11	NC_000003	101368283	101395988	3.9492453	5.2694845	5.7053881	5.2766444
LOC100009676	NC_000003	101395274	101398061	0	0	0	0
RPL24	NC_000003	101399934	101405563	70.927129	109.26022	138.37199	136.77659
LOC285359	NC_000003	101431278	101432260	0	0	0	0
CEP97	NC_000003	101443494	101486181	2.5207215	3.841255	4.6800079	5.026618
FAM55C	NC_000003	101498029	101547075	1.23791	0.9233325	2.6819384	2.7265715
NFKBIZ	NC_000003	101546857	101579866	5.2788454	3.8131281	7.320834	3.509636
LOC152225	NC_000003	101659703	101716770	0	0	0	0
ZPLD1	NC_000003	102153859	102198685	0.0121438	0.0124851	0.0432137	0.0253867
LOC100287880	NC_000003	102775948	102960081	0	0.1491201	0	0.2021444
LOC644681	NC_000003	103257478	103384467	0	0	0	0
LOC100128179	NC_000003	103723221	103782333	0	0	0	0
TRNAE27P	NC_000003	103879524	103879595	0	0	0	0
LOC391562	NC_000003	104221545	104222960	0	0	0	0
ALCAM	NC_000003	105085713	105295744	41.463919	41.752475	26.620321	30.934938
LOC100287987	NC_000003	105085753	105097166	0.0743625	0.2293574	0.4630845	0.2590937
CBLB	NC_000003	105377109	105587887	9.2406242	5.7161094	3.4121869	5.5996696
LOC100128733	NC_000003	106567512	106568119	0	0	0	0
LOC100288046	NC_000003	106616518	106617572	0	0	0	0
LOC100288080	NC_000003	106617589	106619438	0	0	0	0
LOC100288113	NC_000003	106619732	106620692	0	0	0	0
TRNAQ42P	NC_000003	106620878	106620949	0	0	0	0
LOC344593	NC_000003	106823758	106825847	0	0	0	0
LOC344595	NC_000003	106851621	106967195	0.7700899	0.6142759	0.767777	2.0354904
LOC649024	NC_000003	107046677	107077474	0	0	0	0
CCDC54	NC_000003	107096188	107097481	0.1698154	0.1396705	0.241716	0.284002
LOC100288696	NC_000003	107142520	107149606	0.1327741	0.0455019	0	0.0925223
BBX	NC_000003	107241783	107530050	3.7088574	3.4881444	6.664907	5.7471117
LOC151658	NC_000003	107560509	107596915	0	0	0	0
LOC285205	NC_000003	107642475	107647754	0	0	0	0
CD47	NC_000003	107761941	107809935	11.599505	9.8801715	26.613551	19.041731
IFT57	NC_000003	107879659	107941417	0.4665126	0.9874612	1.0497629	1.6063002
HHLA2	NC_000003	108021332	108097126	0	0.0509587	0.0146983	0.0805918
MYH15	NC_000003	108099216	108248169	0.0434885	0.1149705	0.0552694	0.1039012
RPL13P8	NC_000003	108262221	108262722	0	0	0	0
KIAA1524	NC_000003	108268716	108308491	1.2771457	1.8530807	3.5368316	5.4833389
DZIP3	NC_000003	108308337	108413693	0.5788923	0.7908318	0.6843132	1.3704384
RETNLB	NC_000003	108474486	108476130	0.0766985	0	0.0682331	0
TRAT1	NC_000003	108541631	108573714	0	0	0.0230664	0.1445422
GUCA1C	NC_000003	108626642	108672677	0.0475116	0	0	0.033108
MORC1	NC_000003	108677086	108836993	0.0233271	0	0.0207524	0.0325105
LOC100288721	NC_000003	108895291	108896213	0	0	0	0
C3orf66	NC_000003	108897012	108904108	0	0	0	0
DPPA2	NC_000003	109012641	109035364	0.0318696	0	0	0
DPPA4	NC_000003	109044988	109056419	0.0936065	0.3047514	0.0971541	0.1087145
LOC100288251	NC_000003	109082857	109086298	0	0	0	0
LOC100129989	NC_000003	109106659	109116717	0	0	0	0
LOC644950	NC_000003	109128525	109130216	0.1031649	0	0	0
FLJ25363	NC_000003	109128837	109214014	0	0.060527	0.1309363	0.0410246
PPIAP15	NC_000003	109189927	109190726	0	0	0	0
LOC440973	NC_000003	109634828	109635787	0	0	0	0

RPSAP29	NC_000003	110401063	110402084	0	0	0	0
LOC151760	NC_000003	110610877	110612317	0	0	0	0
PVRL3	NC_000003	110790865	110853062	3.4093174	6.84597	7.2271258	6.4033836
CD96	NC_000003	111260926	111371206	0.0098077	0	0.0174504	0.0273375
ZBED2	NC_000003	111311747	111314166	0	0	0	0.0140031
PLCXD2	NC_000003	111393507	111565294	0.0841819	0.1406403	0.4212595	0.5682816
PHLDB2	NC_000003	111451327	111695364	7.5834585	6.5892461	8.4640563	15.714668
LOC100128035	NC_000003	111576840	111578440	0	0	0	0
ABHD10	NC_000003	111697828	111712210	3.8230308	2.9905807	4.8354402	4.9690139
TAGLN3	NC_000003	111717586	111732735	0.0557719	0.2006877	0.1736567	0
LOC100132753	NC_000003	111749778	111750544	0	0	0	0
TMPRSS7	NC_000003	111758465	111800116	0.0175162	0.0360171	0.0467488	0.06103
C3orf52	NC_000003	111805182	111837044	2.0257087	1.8049559	1.2185808	2.379545
GCET2	NC_000003	111839688	111852117	0.0380175	0.0912006	0.1127381	0.4415352
SLC9A10	NC_000003	111859752	112013074	0.0213186	0.0109589	0.1137935	0.0891338
CD200	NC_000003	112051916	112081659	0.095915	0	0.0170657	0
BTLA	NC_000003	112182813	112218408	0.0410092	0	0.0486439	0.076205
OR7E100P	NC_000003	112243033	112244047	0	0	0	0
ATG3	NC_000003	112251359	112280485	24.167989	27.211904	40.545624	42.756473
SLC35A5	NC_000003	112280895	112303003	6.7118218	6.5623512	9.3754367	10.520792
CCDC80	NC_000003	112323407	112359977	2.5640515	3.8343484	1.5207009	8.2974808
LOC100129297	NC_000003	112521325	112538791	0	0	0	0
CD200R1L	NC_000003	112534556	112564797	0.0339107	0	0	0.0236303
CD200R1	NC_000003	112641532	112693937	0.1650121	0.0377	0.0326221	0.1405397
GTPBP8	NC_000003	112709800	112720221	1.6517022	2.3973507	3.3421648	4.784432
C3orf17	NC_000003	112721291	112738555	1.880525	1.9705577	3.5067967	3.8052719
BOC	NC_000003	112931375	113006306	1.9915807	3.9156837	2.2923357	2.1818706
LOC100288391	NC_000003	112942741	112992157	0.0908021	0.1555902	0.0807801	0.0843661
PHF5DP	NC_000003	113080809	113081655	0	0	0	0
WDR52	NC_000003	113081712	113160340	0.0269621	0.0970196	0.1559105	0.5448598
CCDC52	NC_000003	113161565	113234034	0.2742731	0.5888439	0.8540034	1.2085809
SIDT1	NC_000003	113251218	113348422	0.0263584	0.0270992	0.0390819	0.0428577
LOC100288429	NC_000003	113251402	113286626	0	0.0459648	0	0
KIAA2018	NC_000003	113367232	113415493	0.3270149	0.4219051	1.7626419	1.8364203
NAT13	NC_000003	113437841	113465096	2.6177219	4.0432574	4.3514628	9.1377923
ATP6V1A	NC_000003	113465908	113530903	17.235358	14.948564	25.053727	25.919055
GRAMD1C	NC_000003	113557681	113666021	0.1986493	0.2763143	0.6133359	0.5129932
RPS10P4	NC_000003	113569035	113569617	0	0	0	0
ZDHHC23	NC_000003	113666748	113681829	0.4885722	0.4305459	1.510917	1.718495
KIAA1407	NC_000003	113682984	113775460	0.0773736	0.0454561	0.2458348	0.2541803
LOC100127892	NC_000003	113693367	113703499	0	0	0	0
QTRTD1	NC_000003	113775611	113807269	2.0236423	3.6974405	2.1231661	3.1115367
LOC391566	NC_000003	113822096	113822475	0	0	0	0
DRD3	NC_000003	113847557	113897899	0	0	0.02384	0.0186737
LOC645180	NC_000003	113950245	113951868	0	0	0	0
ZNF80	NC_000003	113953478	113956425	0.0343346	0.0529493	0	0.1435541
TIGIT	NC_000003	114012875	114028348	0	0.0211236	0.0182784	0.0286348
ZBTB20	NC_000003	114057517	114866127	2.7124878	2.2705993	5.8415587	4.4000668
LOC100129154	NC_000003	114567107	114650299	0	0	0	0
LOC645207	NC_000003	114993887	114998707	0	0	0	0
GAP43	NC_000003	115342151	115440334	0.2924312	0.279175	0.3344849	0.0727778
LOC100288551	NC_000003	115342151	115395455	0.0430022	0.2210538	0.0765119	0
LSAMP	NC_000003	115529142	116164378	0.3141199	0.2055125	0.0508091	0.4377825
BZW1L1	NC_000003	116363067	116365571	0	0	0	0
LOC285194	NC_000003	116428635	116435887	0	0	0	0
LOC728873	NC_000003	116745545	116745886	0	0	0	0
IGSF11	NC_000003	118619477	118864898	0.0455068	0.0233929	0.2530259	0.031711
C3orf30	NC_000003	118864997	118870302	0	0.0249082	0.0215532	0
UPK1B	NC_000003	118892425	118924000	0.0213548	0.04391	0.0189978	0.0297618
B4GALT4	NC_000003	118930589	118959752	6.637233	13.300575	7.8562088	5.8924147
CDGAP	NC_000003	119013220	119138323	2.2357623	2.0748907	1.2340487	1.360853
RPS26P21	NC_000003	119017512	119017859	0	0	0	0
TMEM39A	NC_000003	119149701	119182471	7.0596585	10.100179	9.810752	6.8458047
KTELC1	NC_000003	119187785	119213555	1.8907816	2.1869125	1.5603614	2.7044897
C3orf1	NC_000003	119217368	119243128	16.659574	20.751525	22.21898	20.74279
CD80	NC_000003	119243140	119278481	0.0318812	0.0327772	0.0283624	0.0444322

CSRP2P	NC_000003	119249679	119250377	0	0	0	0
LOC100288489	NC_000003	119298375	119305386	0.3788641	0.2337073	0.0674096	0.105603
ADPRH	NC_000003	119298523	119308792	1.7379935	0.3153247	1.0800432	0.4541637
PLA1A	NC_000003	119316722	119348658	0.1253515	0.0515498	0.0892129	0.1222899
RPL10P7	NC_000003	119354373	119354997	0	0	0	0
POPDC2	NC_000003	119360908	119379404	0.4009875	0.1099353	0.1902558	0.1676544
COX17	NC_000003	119388372	119396243	4.5714473	7.4771587	10.536933	13.393859
C3orf15	NC_000003	119421869	119485949	0.0892249	0.0509626	0.2557702	0.2210684
NR1I2	NC_000003	119499331	119537332	0.0481889	0.0198173	0.0171481	0.0470119
GSK3B	NC_000003	119545543	119812513	13.865586	14.505373	25.584139	28.082222
LOC100129707	NC_000003	119746763	119748715	0	0	0	0
GPR156	NC_000003	119885879	119962945	0.5392421	0.683757	1.7909723	1.3402298
LOC442087	NC_000003	120025260	120026862	0	0	0	0
LRRC58	NC_000003	120043576	120068186	4.5704331	7.1571261	8.8291392	12.344819
LOC100130701	NC_000003	120076665	120106964	0	0	0	0
FSTL1	NC_000003	120113061	120169918	71.667663	111.0288	67.921839	94.227327
LOC100288625	NC_000003	120203018	120203374	0	0	0	0
RPL34P9	NC_000003	120231701	120232036	0	0	0	0
NDUFB4	NC_000003	120315128	120321175	20.681521	33.180221	60.909111	53.462786
HGD	NC_000003	120347015	120401328	0.0915588	0.7530567	0.0203633	0.0319009
RABL3	NC_000003	120405528	120461384	1.7995799	2.1526988	2.9099141	4.3614622
GTF2E1	NC_000003	120461558	120501916	1.1881817	2.2196923	2.1398602	2.080028
LOC100130335	NC_000003	120518627	120525592	0	0	0	0
STXBP5L	NC_000003	120627050	121143608	0.0281569	0.0434224	0.0250492	0.1340758
POLQ	NC_000003	121150273	121264853	0.3353681	0.6329793	0.7347492	1.2940581
RPL7AP11	NC_000003	121212934	121213810	0	0	0	0
ARGFX	NC_000003	121286778	121309469	0.0173537	0.0089207	0	0.0362782
FBXO40	NC_000003	121312170	121349139	0.0460834	0.0236893	0.0341642	0.0267606
HCLS1	NC_000003	121350246	121379791	0	0	0.0192315	0.1054472
GOLGB1	NC_000003	121382048	121468602	0.8251344	0.5211139	1.3527723	4.7806024
IQCB1	NC_000003	121488610	121553926	2.7277044	2.9785512	5.4561753	5.6669011
EAF2	NC_000003	121554034	121605373	0.0430865	0.2214873	0.2299857	0.2401951
SLC15A2	NC_000003	121613287	121660458	0.0155239	0.0798011	0.0552421	0.1514476
ILDR1	NC_000003	121706170	121741030	0.0826405	0	0	0.0230349
CD86	NC_000003	121774221	121839983	0.015415	0.0316965	0	0.0214836
CASR	NC_000003	121972795	122005344	0	0.0094427	0.0081709	0.0192006
LOC100130002	NC_000003	122036456	122037622	0	0	0	0
CSTA	NC_000003	122044011	122060816	0.0530776	0.163708	0.1416578	0.0369866
CCDC58	NC_000003	122078436	122102074	4.9579482	9.628218	9.3115372	16.634683
FAM162A	NC_000003	122103023	122128961	5.3493075	8.5190662	18.988913	20.721129
WDR5B	NC_000003	122130700	122134882	0.8300049	0.9829523	1.3178955	1.3471139
KPNA1	NC_000003	122140748	122233786	7.176914	9.5036652	11.946636	13.24307
PARP9	NC_000003	122246771	122283424	38.157342	20.208846	39.230592	15.127724
DTX3L	NC_000003	122283185	122294050	11.604223	7.3556197	15.739346	10.364036
PARP15	NC_000003	122296449	122355536	0.2989676	0.0808869	0.0979889	0.0877189
LOC339881	NC_000003	122379460	122381294	0	0	0	0
PARP14	NC_000003	122399672	122449687	11.802392	7.4387308	20.948748	5.6815745
HSPBAP1	NC_000003	122458846	122512650	0.9080213	1.0735726	2.1002825	2.2620649
DIRC2	NC_000003	122513901	122599986	1.8569675	3.3579514	5.6941615	4.6529258
LOC100129550	NC_000003	122605360	122611263	0	0	0	0
SEMA5B	NC_000003	122628043	122746576	0.1213782	0.3167734	0.1162876	0.2602497
PDIA5	NC_000003	122785958	122880875	3.6645339	3.5791498	15.904489	5.8003045
SEC22A	NC_000003	122920774	122992983	1.9035231	3.3983419	4.656924	3.9883146
ADCY5	NC_000003	123003399	123167392	0.0228778	0.0352812	0.0203527	0.1673926
PTPLB	NC_000003	123213363	123303924	3.1841179	9.2498455	10.77077	10.836098
MYLK	NC_000003	123331143	123603149	2.9807245	2.9264577	6.6365048	10.684838
LOC100288589	NC_000003	123418926	123453036	0.2998417	0.1321152	0.1905339	0.0596976
CCDC14	NC_000003	123632269	123680255	3.8101763	2.7194214	5.4533027	11.351758
ROPN1	NC_000003	123687878	123710199	0.0398082	0.040927	0	0.0277399
KALRN	NC_000003	123813585	124440036	0.1693438	0.1849846	0.0282474	0.0368767
RPL7P15	NC_000003	123870742	123871476	0	0	0	0
LOC100288624	NC_000003	124223606	124282493	0.0439482	0	0.0390976	0.0306249
UMPS	NC_000003	124449213	124464040	0.9259221	2.226872	1.5150674	2.4541381
RPS26P22	NC_000003	124452265	124452601	0	0	0	0
ITGB5	NC_000003	124481795	124606144	31.266847	50.020652	29.763775	38.744286
MUC13	NC_000003	124624289	124653580	0.1536114	0.363236	0.1639884	0.2890149

HEG1	NC_000003	124684554	124774802	10.641462	9.0159541	11.106681	30.190056
SLC12A8	NC_000003	124801480	124931609	2.2662763	1.4674951	0.9468071	1.5007063
ZNF148	NC_000003	124944513	125094198	3.3434367	4.3038575	5.4162194	6.8290622
SNX4	NC_000003	125165494	125239058	1.9327471	1.9512642	3.4698315	3.7371088
OSBPL11	NC_000003	125247702	125314381	2.8806086	4.2962226	3.8777232	5.414489
OR7E130P	NC_000003	125422128	125423290	0	0	0	0
OR7E29P	NC_000003	125430878	125432022	0	0	0	0
OR7E93P	NC_000003	125443277	125444447	0	0	0	0
OR7E53P	NC_000003	125453336	125453991	0	0	0	0
OR7E97P	NC_000003	125465972	125466942	0	0	0	0
LOC651856	NC_000003	125475035	125488202	0.1775686	0.3194786	0.078985	0.1546711
RPS24P9	NC_000003	125506491	125507093	0	0	0	0
RPS3AP14	NC_000003	125513925	125514772	0	0	0	0
LOC100131679	NC_000003	125593456	125593629	0	0	0	0
LOC100125556	NC_000003	125635444	125648867	0	0	0	0
ALG1L	NC_000003	125648118	125655882	0.4913467	0.3367707	0.0971368	0.2663033
ROPN1B	NC_000003	125688028	125702297	0	0	0	0
SLC41A3	NC_000003	125725200	125803134	4.0660453	3.7394286	4.874832	5.733172
ALDH1L1	NC_000003	125822408	125899485	0.0281269	0.0289174	0	0.0391998
LOC644662	NC_000003	125985645	126010671	0.042096	0.0432791	0	0.0586683
KLF15	NC_000003	126061478	126076236	0.1044314	0.1431553	0.1083893	0.0242573
CCDC37	NC_000003	126113782	126155398	0.0842727	0.1083015	0.112457	0.0146811
ZXDC	NC_000003	126156444	126194762	0.6333612	0.7564002	1.50824	1.6316625
UROC1	NC_000003	126200124	126236594	0.0140544	0.0144494	0	0
CHST13	NC_000003	126243176	126262134	0	0	0.0224828	0.0704425
C3orf22	NC_000003	126268519	126277758	0	0	0	0
TR2IT1	NC_000003	126290622	126327398	0.5038157	1.035951	1.3446253	1.3503033
C3orf46	NC_000003	126379485	126382232	0	0	0	0
LOC645852	NC_000003	126390949	126392066	0	0	0	0
CHCHD6	NC_000003	126423118	126679244	1.0685088	1.9105033	1.9011501	2.6545878
PLXNA1	NC_000003	126707502	126756232	10.921121	11.750296	14.690917	13.494959
C3orf56	NC_000003	126911974	126917025	0	0	0	0
LOC100128149	NC_000003	126940032	127057202	0	0	0	0
GPR175	NC_000003	127291908	127309568	5.4507876	6.6792855	8.0879175	7.6947635
MCM2	NC_000003	127317253	127341279	19.460047	28.517501	32.439028	43.543495
LOC100288735	NC_000003	127336554	127338075	0.0757728	0.0779024	0.0674096	0.0528015
PODXL2	NC_000003	127348039	127391652	2.2065211	2.2060988	22.475247	20.087437
ABTB1	NC_000003	127391781	127399763	2.397969	3.8997579	4.8484084	5.3016282
MGLL	NC_000003	127407909	127542051	23.193612	30.49093	15.139066	23.186762
KLHDC6	NC_000003	127641902	127706514	0.0083425	0	0.0074217	0
SEC61A1	NC_000003	127771212	127790526	79.242191	100.66852	105.55052	124.30197
RUVBL1	NC_000003	127799800	127842671	1.416883	1.840048	3.3392152	4.5729456
EEFSEC	NC_000003	127872313	128127489	1.4080887	3.180781	1.8525471	3.1371152
DNAJB8	NC_000003	128181282	128186091	0	0.0183077	0.0316836	0.0372264
GATA2	NC_000003	128198265	128212030	0.186815	4.1534158	1.39189	1.5621617
LOC90246	NC_000003	128226678	128229438	0	0	0	0
LOC100129727	NC_000003	128256860	128257474	0	0	0	0
C3orf27	NC_000003	128290843	128294929	0.0325905	0.0167532	0.0144967	0.0227103
RPN1	NC_000003	128338813	128369719	29.680544	37.656052	62.546084	58.437134
POU5F1P6	NC_000003	128393489	128395453	0	0	0	0
RAB7A	NC_000003	128444979	128533641	64.288072	59.27285	92.488865	110.71333
FTHL4	NC_000003	128483149	128484060	0	0	0	0
RPS15AP16	NC_000003	128517683	128518044	0	0	0	0
LOC100128615	NC_000003	128562961	128571454	0.0492417	0	0.0876136	0.0343136
ERV3 (NC_000003 1285	NC_000003	128573609	128574320	0	0.0634598	0	0.0860249
LOC653712	NC_000003	128580156	128584285	0.1140032	0.4102254	0.0507102	0.2383259
ACAD9	NC_000003	128598333	128631957	2.4064857	2.8601532	2.8393184	3.722558
KIAA1257	NC_000003	128689779	128712986	0	0.026346	0	0.0178571
CCDC48	NC_000003	128749292	128759583	0	0	0	0.0468032
LOC100130760	NC_000003	128759837	128766263	0	0	0	0
GP9	NC_000003	128779645	128781253	0	0	0	0
LOC100131426	NC_000003	128797099	128810190	2.7715101	1.2720552	2.2454683	2.2416859
RAB43	NC_000003	128806418	128840619	2.6090248	2.7229934	3.323337	3.3124728
ISY1	NC_000003	128848235	128880029	1.3749447	1.9019179	1.6457451	2.4040007
CNBP	NC_000003	128886658	128902810	29.25986	38.734353	50.293056	35.077432
RPS27P12	NC_000003	128936879	128937215	0	0	0	0

COPG	NC_000003	128968453	128996616	18.600081	21.004255	22.461386	36.831547
C3orf37	NC_000003	128997684	129024136	2.1040305	2.8194054	3.2809146	6.3589043
H1FX	NC_000003	129033615	129035117	15.292698	16.68449	33.452741	11.00295
C3orf47	NC_000003	129035114	129043412	0	0	0	0
RPL32P3	NC_000003	129101677	129118282	0	0	0	0
SNORA7B	NC_000003	129116053	129116191	0	0	0	0
C3orf25	NC_000003	129120164	129147494	0.0816123	0.041953	0.0181511	0.0568707
LOC100288961	NC_000003	129129975	129147490	0	0	0	0
MBD4	NC_000003	129149793	129158852	5.604734	7.920815	8.6426198	10.328147
IFT122	NC_000003	129158968	129239191	1.4751265	1.7765711	1.1623347	2.0044574
RHO	NC_000003	129247482	129254187	0	0	0.0141248	0.0331917
H1FOO	NC_000003	129262057	129270204	0	0	0.0366425	0
PLXND1	NC_000003	129274056	129325582	3.5778409	3.1085041	6.28744	5.657943
TMCC1	NC_000003	129366635	129612403	0.9077202	1.3419464	2.1750342	1.2096665
TRH	NC_000003	129693114	129696778	0.1093784	0.0224905	0	0.0152438
RPS17P9	NC_000003	129703935	129717779	0	0	0	0
LOC100130333	NC_000003	129719098	129722233	0	0	0	0
OR7E129P	NC_000003	129740299	129741523	0	0	0	0
OR7E21P	NC_000003	129753232	129754485	0	0	0	0
ARVP6125	NC_000003	129762156	129763668	0	0	0	0
LOC644974	NC_000003	129800674	129817233	0.1360626	0.1865156	0.1210451	0.1264185
LOC729375	NC_000003	129816625	129830276	0	0	0	0
LOC100133174	NC_000003	129851556	129851728	0	0	0	0
LOC646300	NC_000003	129854005	130012605	0	0	0	0
COL29A1	NC_000003	130064359	130203688	0.0594029	0.0763405	0.0572503	0.0620914
COL6A6	NC_000003	130279178	130395888	0.0311322	0.5014451	0.032312	0.0976236
PIK3R4	NC_000003	130397778	130465696	1.5001183	2.1071839	2.5061547	1.9022796
GSTO3P1	NC_000003	130546435	130547224	0	0	0	0
ATP2C1	NC_000003	130613434	130735556	7.4863711	12.65749	10.864123	14.136781
ASTE1	NC_000003	130732721	130745646	1.0896256	1.8670827	1.0427976	2.0938121
NEK11	NC_000003	130745727	131069303	1.032341	1.4335185	0.9780355	1.2051889
LOC100132405	NC_000003	131080706	131082633	2.944315	15.313389	1.8104291	6.5775601
NUDT16P	NC_000003	131080738	131083966	0	0	0	0
LOC100133263	NC_000003	131100609	131102610	0.3632085	0.0622361	0.2154136	0.6749284
NUDT16	NC_000003	131100707	131104929	0.4698192	0.9660471	0.6568009	0.6080077
MRPL3	NC_000003	131181045	131221829	24.269162	65.545916	41.240825	46.900419
SNORA58	NC_000003	131197941	131198077	0	0	0	0
LOC100289095	NC_000003	131245303	131245991	0	0.0687723	0	0.0932264
CPNE4	NC_000003	131253572	131753844	0.0341213	0.245562	0.0758881	0.0237771
LOC729674	NC_000003	131894246	131895557	0	0	0	0
RPL7P16	NC_000003	131962304	131963130	0	0	0	0
ACPP	NC_000003	132036211	132087146	0.0345324	0.0828402	0.1024033	0.216572
LOC100130550	NC_000003	132105605	132106794	0	0	0	0
LOC646392	NC_000003	132120097	132120639	0	0	0	0
DNAJC13	NC_000003	132136553	132257876	7.7624212	10.872275	10.56831	18.520199
ACAD11	NC_000003	132276982	132378975	1.6808098	2.6174862	2.2319476	1.7224341
CCRL1	NC_000003	132316094	132321382	0.0177497	0.0364971	0	0.0123687
UBA5	NC_000003	132373290	132396944	1.3767566	2.4839769	1.5850364	2.3702301
NPHP3	NC_000003	132399454	132441276	1.751382	2.3475175	2.3589593	2.3211034
NCRNA00119	NC_000003	132441186	132593050	0	0	0	0
TMEM108	NC_000003	132757171	133116619	0.0347783	0.0119186	0.041253	0.0403916
BFSP2	NC_000003	133118790	133194056	0	0.0277881	0	0.0565035
LOC391578	NC_000003	133209371	133210407	0	0	0	0
CDV3	NC_000003	133292434	133309118	13.783902	16.611391	22.224164	20.503755
TOPBP1	NC_000003	133319449	133380737	3.8062161	6.7445482	8.4470049	13.015287
LOC100129696	NC_000003	133406887	133465097	0	0	0	0
TF	NC_000003	133465235	133497635	0.0189595	0.0389848	0	0.0528471
SRPRB	NC_000003	133524677	133539523	9.7466852	17.684965	10.060061	11.635699
RAB6B	NC_000003	133543079	133614691	0.4662733	1.1943823	1.0475701	0.5011444
C3orf36	NC_000003	133646989	133648656	0	0.0270884	0	0.0550807
SLCO2A1	NC_000003	133651540	133748920	0.0312206	0.2246866	0.037033	0.0435115
RYK	NC_000003	133875978	133969586	5.8298675	10.235424	10.05366	8.1562176
RPL39P5	NC_000003	134070581	134070985	0	0	0	0
AMOTL2	NC_000003	134074190	134093406	29.363486	21.884577	11.178578	23.085407
LOC100289132	NC_000003	134077140	134078363	0.2635576	0.1354825	0.058617	0.0459144
LOC100289158	NC_000003	134156448	134157054	0	0	0	0

ANAPC13	NC_000003	134196546	134204865	21.355639	20.871141	28.158015	26.644658
CEP63	NC_000003	134204575	134293855	0.1890106	0.2613307	0.2725175	0.7221349
KY	NC_000003	134318765	134369864	0.1772106	0.8713489	0.2604677	0.5207947
EPHB1	NC_000003	134514260	134979309	0.1459751	0.9304819	0.0173151	0.0271257
PPP2R3A	NC_000003	135684567	135866733	1.3297314	4.0448708	2.3605054	3.3919019
MSL2	NC_000003	135867760	135914688	4.1921269	5.34244	8.3031098	6.6129192
LOC100289083	NC_000003	135870789	135914586	0.0839775	0.1726754	0.0996116	0.1170378
RPL31P23	NC_000003	135924331	135924775	0	0	0	0
PCCB	NC_000003	135969167	136049011	7.1228115	7.8211622	11.272341	12.138527
STAG1	NC_000003	136055999	136471245	7.4523134	15.122358	8.0374786	14.47064
LOC100131035	NC_000003	136527357	136538363	0	0	0	0
TMEM22	NC_000003	136537861	136574734	3.7974686	6.9091124	1.9358989	2.0515695
NCK1	NC_000003	136581073	136667968	5.005598	4.6505382	10.091012	7.5522166
IL20RB	NC_000003	136676707	136729920	0.6247543	0.7752054	0.8432808	0.4203415
LOC100289118	NC_000003	137254414	137254869	0	0	0	0
NPM1P17	NC_000003	137442497	137443410	0	0	0	0
SOX14	NC_000003	137483579	137484396	0	0.0552364	0.0955931	0
LOC402143	NC_000003	137599137	137601079	0	0	0	0
CLDN18	NC_000003	137717658	137752494	0.0121003	0.0995229	0.1076475	0.1686392
DZIP1L	NC_000003	137780832	137834451	0.6290901	0.9701561	0.6492009	1.8148725
A4GNT	NC_000003	137842560	137851229	0	0	0.0662296	0.1383394
DBR1	NC_000003	137879852	137893773	3.0680841	4.160283	2.9360059	3.8252206
ARMC8	NC_000003	137906148	138016219	2.6809208	4.0091176	3.0984296	3.0460523
TXNDC6	NC_000003	137980279	138048728	0.020604	0.021183	0	0.0861459
LOC100289271	NC_000003	138043721	138048665	0	0	0	0
MRAS	NC_000003	138066625	138124377	6.2796969	7.9437895	6.2731169	5.8816437
FAM62C	NC_000003	138153415	138197256	0.2174187	0.528342	0.1142947	0.1377328
CEP70	NC_000003	138213186	138313129	0.6931377	0.9331908	2.2756751	3.0015371
FAIM	NC_000003	138327542	138352218	1.9050408	2.5026327	2.3303185	4.2775266
LOC256374	NC_000003	138353767	138363524	0.1743977	0.0896496	0.2327236	0.4861092
PIK3CB	NC_000003	138374231	138478185	1.6277123	1.687522	4.806579	7.5013318
LOC100128589	NC_000003	138496602	138497853	0	0	0	0
RPL23AP40	NC_000003	138515693	138516081	0	0	0	0
LOC646612	NC_000003	138542716	138545171	0	0	0	0
LOC646619	NC_000003	138608094	138608799	0	0	0	0
FOXL2	NC_000003	138663066	138665801	1.0119659	0.0660576	0.5144417	0.6380183
C3orf72	NC_000003	138666076	138672830	0.614736	0.0554398	0.3837798	0.6463164
LOC729627	NC_000003	138722804	138725110	0.0190499	0	0.0169474	0.0663738
LOC389151	NC_000003	138737873	138739768	0	0	0.0412422	0.0161524
FLJ46210	NC_000003	138760944	138763734	0	0	0	0.0329182
LOC646641	NC_000003	138800496	138801219	0	0	0	0
BPESC1	NC_000003	138823027	138844009	0	0	0	0
PISRT1	NC_000003	138951834	138952364	0	0	0	0
MRPS22	NC_000003	139062861	139075888	9.3140509	19.916103	14.135006	14.317062
COPB2	NC_000003	139076433	139108522	28.552982	45.27021	37.8832	47.099357
RBP2	NC_000003	139171726	139195352	0	0	0.1136557	0
ACTGP1	NC_000003	139212583	139214526	0	0	0	0
RBP1	NC_000003	139236276	139258671	0	0.0231235	0.040018	0
LOC100289182	NC_000003	139257398	139258671	0	0	0	0
NMNAT3	NC_000003	139279033	139396840	0	0.024253	0	0.0328769
CLSTN2	NC_000003	139654027	140286919	0.3060713	0.2128673	0.1041107	0.2634668
TRIM42	NC_000003	140396881	140419991	0	0.0356055	0.0308097	0.0241331
LOC100289216	NC_000003	140583917	140586625	0.016223	0	0	0.0113049
RPL23AP41	NC_000003	140621036	140621493	0	0	0	0
SLC25A36	NC_000003	140660662	140698785	2.7061022	3.3250176	5.8717648	4.8818461
SPSB4	NC_000003	140770743	140867453	0.1094601	0.1125365	0	0
ACPL2	NC_000003	140950682	141013486	0.4006598	0.9658822	0.8972406	1.1071553
RPL31P21	NC_000003	140977260	140977621	0	0	0	0
LOC100289442	NC_000003	141026447	141030023	0	0	0	0
LOC100289257	NC_000003	141037425	141040566	0.0279747	0.0287609	0.0124435	0.1072163
ZBTB38	NC_000003	141043055	141168634	2.4945574	7.9687874	12.821167	18.255875
KRT18P35	NC_000003	141189390	141190792	0	0	0	0
RASA2	NC_000003	141205926	141331197	3.3741119	3.957034	4.82686	3.6981431
LOC646730	NC_000003	141381934	141426234	0	0	0.0452518	0.0708909
RNF7	NC_000003	141457051	141465243	4.9472681	10.031337	6.2106202	6.971517
GRK7	NC_000003	141497043	141535892	0.0251708	0	0.0447853	0.01754

LOC729687	NC_000003	141583849	141584121	0.3219651	0	0	0.2243581
ATP1B3	NC_000003	141595470	141645382	16.104074	25.603115	27.767078	34.839311
LOC100289326	NC_000003	141655549	141657389	0.0238719	0	0	0
TFDP2	NC_000003	141671328	141724377	2.8497682	4.6242388	8.8274972	6.2206784
RPL19P6	NC_000003	141762386	141763067	0	0	0	0
GK5	NC_000003	141876369	141944428	1.4819544	1.4499564	1.8959292	2.2588032
XRN1	NC_000003	142025449	142166853	1.4719087	1.6207284	1.9060644	2.7887696
LOC731755	NC_000003	142166878	142171023	0	0	0	0
ATR	NC_000003	142168077	142297668	2.4767819	3.5211402	4.2267639	6.2318714
RPL6P9	NC_000003	142300131	142300820	0	0	0	0
PLS1	NC_000003	142315229	142432506	0.354329	0.1645169	2.0641888	1.1947287
TRPC1	NC_000003	142443266	142526730	1.5121043	2.1653387	2.3733347	2.2202849
RNU7-47P	NC_000003	142520052	142520109	0	0	0	0
PCOLCE2	NC_000003	142536711	142607934	10.285267	12.656084	3.7459971	5.4836169
RPL8P3	NC_000003	142545896	142546570	0	0	0	0
PAQR9	NC_000003	142680073	142682178	0.0208681	0.107273	0.0556945	0.2035842
LOC100289361	NC_000003	142719707	142720309	0	0	0.1945153	0.0507875
SR140	NC_000003	142720372	142779567	4.2805955	6.4167211	8.3653286	7.3977298
LOC100289534	NC_000003	142838106	142840009	1.1958703	0.2195501	0.5699354	0.3869032
CHST2	NC_000003	142838668	142841812	2.4407661	3.4745041	0.603873	0.1710887
LOC100289567	NC_000003	142840517	142872817	2.1331048	3.1605806	1.1720898	0.655779
PBX2P1	NC_000003	142894904	142898094	0	0	0	0
SLC9A9	NC_000003	142984064	143567346	1.6846822	0.8283624	3.1278053	2.4414834
LOC100240712	NC_000003	143221916	143223186	0	0	0	0
LOC100128739	NC_000003	143316743	143346551	0	0	0	0
RPS17P10	NC_000003	143574531	143575007	0	0	0	0
C3orf58	NC_000003	143690640	143711210	1.2343396	1.4756173	2.5707522	4.2806819
GM2AP	NC_000003	145402276	145406285	0	0	0	0
RPL21P39	NC_000003	145542030	145542565	0	0	0	0
PLOD2	NC_000003	145787227	145879282	16.918987	11.229077	20.810305	25.322098
PLSCR4	NC_000003	145910122	145968966	2.0172959	1.7900528	3.2260833	2.0081887
LOC440981	NC_000003	146109150	146134390	0.0623379	0	0	0
PLSCR2	NC_000003	146151082	146213722	0.1042251	0.0357181	0.2163502	0.0726282
PLSCR1	NC_000003	146232967	146262628	39.49375	25.413108	50.349173	11.335362
PLSCR5	NC_000003	146303625	146324003	0	0.0229474	0.0198566	0
RPL21P71	NC_000003	146994514	146995074	0	0	0	0
ZIC4	NC_000003	147103837	147124407	0.4623866	0.4311604	0.8609691	1.0790268
ZIC1	NC_000003	147127181	147134506	1.7643144	1.5374968	2.2347873	1.8968573
FLJ30375	NC_000003	147137531	147139986	0.0178942	0.0551915	0	0.0498777
LOC646849	NC_000003	147308776	147309622	0	0	0	0
LOC344741	NC_000003	147884120	147885083	0	0	0	0
RPL38P1	NC_000003	148413742	148414035	0	0	0	0
AGTR1	NC_000003	148415658	148460790	0.6324716	1.1071779	0.9124286	0.4049964
CPB1	NC_000003	148545588	148577974	0.0300604	0	0	0.0209472
CPA3	NC_000003	148583043	148614874	1.0165961	0.1875942	3.3392939	2.0888855
UBQLN4P	NC_000003	148701962	148705477	0	0	0	0
GYG1	NC_000003	148709375	148745419	16.581023	15.595158	36.931496	40.126226
HLTF	NC_000003	148747904	148804341	5.7409482	9.3896361	12.158167	7.2913652
HPS3	NC_000003	148847371	148891305	3.4459521	2.3347972	12.850986	9.701433
CP	NC_000003	148890286	148939832	0.0282565	0	0.0502755	0.4397486
LOC389160	NC_000003	148947218	148994451	0	0	0	0
TM4SF18	NC_000003	149038855	149051419	0.2286985	0.0391877	0.4069131	0.0531221
RPS25P5	NC_000003	149074909	149075391	0	0	0	0
TM4SF1	NC_000003	149086805	149095568	104.81562	12.985886	284.87351	254.83304
LOC100289491	NC_000003	149089485	149095652	0.5218128	0.2384348	0.515799	0.7272399
RPL32P8	NC_000003	149150830	149151227	0	0	0	0
FKBP1P4	NC_000003	149180386	149182004	0	0	0	0
TM4SF4	NC_000003	149192434	149221068	0.1231043	0.0632821	0.0821377	0.10723
WWTR1	NC_000003	149238032	149375782	35.101367	37.241802	37.903298	35.976948
LOC100128025	NC_000003	149272910	149376098	1.0028936	0.9574315	0.9877951	0.8985294
COMMD2	NC_000003	149458531	149470278	5.555254	10.153573	17.35229	12.043491
C3orf16	NC_000003	149478887	149510610	0	0	0.0157906	0.0247374
LOC100289522	NC_000003	149530271	149563924	0	0	0.1167092	0.0304725
RNF13	NC_000003	149530475	149679926	6.625585	7.0403817	16.707122	11.940087
PFN2	NC_000003	149682691	149688741	60.454904	78.738407	136.45162	109.13567
TMEM183B	NC_000003	149699448	149701153	0.3091318	0.2913349	0.1833415	0.2154153

LOC391587	NC_000003	149702101	149702584	0	0	0	0
LOC646908	NC_000003	149717882	149718346	0	0	0	0
LOC730021	NC_000003	149768513	149769274	0	0	0	0
RPL32P9	NC_000003	149868325	149868734	0	0	0	0
TSC22D2	NC_000003	150126788	150177631	6.3201636	6.1618995	12.578171	12.68644
SERP1	NC_000003	150259780	150264428	9.8093783	15.911053	41.72297	39.092097
LOC100129720	NC_000003	150264499	150320988	0.7284237	1.8722404	1.5120605	0.9305902
EIF2A	NC_000003	150264574	150303803	5.8121791	6.5462933	21.741028	25.295723
SELT	NC_000003	150321066	150348234	10.225205	14.202347	30.773929	29.922875
LOC677762	NC_000003	150328840	150329877	0	0	0	0
C3orf44	NC_000003	150377675	150421742	0.0428137	0.0220085	0.1904411	0.2834256
LOC100128469	NC_000003	150437779	150439022	0	0	0	0
SIAH2	NC_000003	150458910	150481263	5.2167558	8.6055411	22.667634	17.883994
LOC100129582	NC_000003	150496789	150497524	0	0	0	0
C3orf76	NC_000003	150588832	150621044	0	0	0.3971129	0.311056
CLRN1	NC_000003	150643950	150690786	0.0829212	0	0.0122948	0
CLRN1OS	NC_000003	150690465	150797617	0	0	0	0
MED12L	NC_000003	150804676	151151810	0.0456961	0.0939608	0.116876	0.266684
GPR171	NC_000003	150915619	150920988	0	0	0	0
P2RY14	NC_000003	150929905	150996230	0.0155075	0.0159433	0	0.0216125
GPR87	NC_000003	151011874	151034636	0.0577886	0	0.1285259	0.0201347
P2RY13	NC_000003	151044096	151047337	0	0	0	0.0221198
P2RY12	NC_000003	151055376	151102544	0	0	0	0.0198992
IGSF10	NC_000003	151154477	151176497	0.0167486	0.0344386	0.0794666	0.2061888
AADACL2	NC_000003	151451704	151475556	0	0.0301223	0	0
LOC100128024	NC_000003	151477512	151479795	0	0	0	0
LOC201651	NC_000003	151488244	151502682	0	0	0	0
AADAC	NC_000003	151531861	151546276	0	0.0281341	0	0
SUCNR1	NC_000003	151591437	151599650	0.0619862	0.0955925	0.0275723	0.1079862
LOC401093	NC_000003	151980405	151987415	0	0	0	0
MBNL1	NC_000003	151985829	152183569	10.860731	12.017968	22.278959	35.197718
TMEM14E	NC_000003	152057487	152058779	0	0	0	0.0236851
LOC100287091	NC_000003	152518223	152544334	0	0	0	0
P2RY1	NC_000003	152552736	152555845	0.1554439	0.4213243	1.1440124	0.6400698
LOC100289682	NC_000003	152785067	152785340	0	0	0	0
LOC100287133	NC_000003	152871523	152878806	0.1687535	0.0991408	0.2359151	0.0839958
RAP2B	NC_000003	152880029	152886265	3.2836101	5.476776	7.5976029	6.4716353
LOC152118	NC_000003	153202284	153220486	0	0.0779024	0	0.0528015
RPL21P42	NC_000003	153741585	153742126	0	0	0	0
SGEF	NC_000003	153839149	153975616	0.2884076	0.44477	0.6716239	0.9694036
DDX50P2	NC_000003	153893783	153899497	0	0	0	0
DHX36	NC_000003	153993457	154042286	2.1176938	2.9279745	4.8506534	4.825687
GPR149	NC_000003	154055461	154147504	0.075675	0.1361532	0.0336613	0.0263667
LOC100130011	NC_000003	154369444	154370158	0	0	0	0
LOC100130896	NC_000003	154375106	154375354	0	0	0	0
RPL9P15	NC_000003	154394377	154394921	0	0	0	0
MME	NC_000003	154797436	154901518	63.547161	76.232009	555.44859	115.16823
PLCH1	NC_000003	155197671	155421997	0.0806883	0.048391	0.1375832	0.1077681
RPL6P7	NC_000003	155375532	155376433	0	0	0	0
RPL7AP24	NC_000003	155478351	155479012	0	0	0	0
C3orf33	NC_000003	155480401	155524055	0.2503524	0.0935959	0.2429678	0.1268768
SLC33A1	NC_000003	155545371	155572167	3.9171609	4.4860542	13.67459	11.102814
GMPS	NC_000003	155588325	155655521	7.584066	10.224653	24.887502	38.203196
LOC389168	NC_000003	155705042	155706847	0	0	0	0
VN2R1P	NC_000003	155751371	155755436	0	0	0	0
LOC730086	NC_000003	155761918	155762732	0	0	0	0
MRE11B	NC_000003	155830423	155832072	0	0	0	0
KCNAB1	NC_000003	155838337	156256927	0.2149462	0.3229814	0.8531448	1.7167445
LOC751837	NC_000003	155892970	155894712	0	0	0	0
SSR3	NC_000003	156258543	156272935	64.559723	106.49952	130.28268	121.66992
LOC100287227	NC_000003	156390960	156392824	0.1326139	0.0272682	0.2595493	0.147857
TIPARP	NC_000003	156392379	156424534	7.7104171	17.338365	8.347473	10.985358
METT5D2	NC_000003	156432013	156432821	0	0	0	0
PA2G4P4	NC_000003	156527060	156529810	0	0	0	0
LEKR1	NC_000003	156544096	156763918	0.0762725	0.1411491	0.081425	0.0744096
LOC100124394	NC_000003	156694667	156695909	0	0	0	0

CCNL1	NC_000003	156865585	156878482	6.4289985	4.0277775	7.3428952	6.206642
KRT18P34	NC_000003	156880398	156881768	0	0	0	0
VEPH1	NC_000003	156978698	157217343	5.0387427	7.5404626	7.552355	7.7467662
PTX3	NC_000003	157154580	157161417	141.09695	27.161333	14.747327	1.4064057
C3orf55	NC_000003	157261159	157319022	1.0997433	1.2799831	0.6645479	0.2313494
SHOX2	NC_000003	157813800	157823936	1.4979312	1.4987799	1.9513088	1.3606915
RSRC1	NC_000003	157827892	158262582	3.1737401	3.3735465	10.982731	11.751407
RPL15P6	NC_000003	157993598	157994210	0	0	0	0
RPS12P7	NC_000003	158214355	158214718	0	0	0	0
MLF1	NC_000003	158288953	158324252	1.4762972	3.0939539	11.29822	6.9374186
LOC100129559	NC_000003	158326995	158328605	0	0	0	0
GFM1	NC_000003	158362317	158410361	7.7048803	12.051513	17.181283	16.363293
LXN	NC_000003	158384203	158390482	1.732726	1.7814244	2.5924909	2.414865
RARRES1	NC_000003	158414897	158450275	0.8441437	0.5424178	1.5019473	0.3676456
LOC100287290	NC_000003	158446138	158461125	0.0967313	0.1988998	0.4015891	0.1797498
RPL35AP9	NC_000003	158481155	158481594	0	0	0	0
MFSD1	NC_000003	158519912	158547504	10.12782	14.134188	19.612106	30.256416
GPR79	NC_000003	158558856	158559873	0	0	0	0
IQCJ	NC_000003	158787117	158983492	0.0225491	0	0	0.0157131
SCHIP1	NC_000003	158991544	159615139	3.2136884	3.0465542	3.4530614	5.5258565
RPS2P19	NC_000003	159104545	159105222	0	0	0	0
IL12A	NC_000003	159706629	159713806	1.7432493	0.5345288	2.6391537	2.4082194
LOC730109	NC_000003	159733817	159757067	0	0.0449586	0	0.060945
BRD7P2	NC_000003	159818494	159820699	0	0	0	0
LOC401097	NC_000003	159943083	159946000	0.1636196	0.3364364	0.0436682	0.06841
IFT80	NC_000003	159974788	160117320	1.402821	1.1580089	4.5364836	5.5798357
RPL35AP10	NC_000003	160060475	160060803	0	0	0	0
SMC4	NC_000003	160117430	160152741	7.162901	12.707045	21.0838	28.623772
TRIM59	NC_000003	160153291	160167626	5.7032922	13.056091	4.2668396	4.5589667
LOC401098	NC_000003	160167359	160169957	0	0	0	0
B3GAT3P	NC_000003	160170164	160171597	0	0	0	0
KPNA4	NC_000003	160217962	160283376	14.587761	27.820711	39.303234	41.60376
SCARNA7	NC_000003	160232695	160233024	0	0	0	0
LOC100287001	NC_000003	160281997	160283359	0	0	0	0
KRT8P12	NC_000003	160285567	160287260	0	0	0	0
RPL6P8	NC_000003	160297055	160303575	0	0	0	0
ARL14	NC_000003	160394948	160396236	0.0681897	0.0350531	0.0303317	0
LOC100287426	NC_000003	160471216	160473193	0.0243211	0.0250046	0.1298204	0.186427
PPM1L	NC_000003	160473996	160788817	0.3882861	0.2661326	1.7655314	2.0743946
LOC100287464	NC_000003	160474022	160680208	0	0.1593771	0.8274617	1.0262305
B3GALNT1	NC_000003	160801671	160823160	4.1159975	2.9410783	3.1462749	3.7934139
NMD3	NC_000003	160939099	160969795	2.5453888	3.809451	6.4350465	5.5681597
LOC100129403	NC_000003	161042702	161044007	0	0	0	0
LOC646085	NC_000003	161046712	161048313	0	0	0	0
C3orf57	NC_000003	161062580	161089871	0.0190582	0.0979692	0.0169547	0.026561
RPL23AP42	NC_000003	161145486	161147405	0	0	0	0
OTOL1	NC_000003	161214596	161221730	0	0.0315086	0	0
LOC100132484	NC_000003	162036750	162071898	0	0	0	0
LOC100287573	NC_000003	162204307	162204737	0	0	0	0
RPS6P4	NC_000003	162819827	162833942	0	0	0	0
RNU7-82P	NC_000003	163187994	163188053	0	0	0	0
LOC730129	NC_000003	163719385	163720253	0	0.0814115	0.0704461	0
SI	NC_000003	164696686	164796282	0.0073125	0	0.0195163	0.0356696
SLITRK3	NC_000003	164904508	164914469	0.0104093	0	0.0092604	0.0435219
BCHE	NC_000003	165490692	165555253	0.01796	0.2769722	0.0798888	0.2002444
LOC100287601	NC_000003	166011544	166034681	0	0	0	0
LOC100287630	NC_000003	166285819	166409538	0	0	0	0
LOC100133259	NC_000003	166528617	166530530	0	0	0	0
LOC131055	NC_000003	166783590	166784336	0.3529978	1.3911891	0.2616972	1.5578921
ZBBX	NC_000003	166958075	167098071	0.0135978	0.01398	0.024194	0.0284266
SERPINI2	NC_000003	167159723	167191818	0.0614661	0	0	0.064248
WDR49	NC_000003	167196472	167371289	0.0169423	0	0.0904338	0.0236121
PDCD10	NC_000003	167401694	167452651	10.055714	15.205122	30.201561	28.419262
SERPINI1	NC_000003	167453432	167543357	2.4747119	2.0444978	2.2408861	1.1701814
LOC100287660	NC_000003	167674087	167674402	0	0	0	0
GOLIM4	NC_000003	167727654	167813417	2.2763883	4.7762581	4.8079811	5.0609824

LOC389174	NC_000003	167967313	168031910	0.0636931	0.0654832	0.0566631	0.3106872
RPSAP33	NC_000003	168384347	168384959	0	0	0	0
C3orf50	NC_000003	168527584	168548387	0	0	0	0
RPL21P43	NC_000003	168576410	168576960	0	0	0	0
EVI1	NC_000003	168801287	168865522	1.2774346	2.165854	1.3092335	2.1187023
MDS1	NC_000003	168867270	169381556	0.3785015	0.4756148	0.598623	1.5532235
RPL22P1	NC_000003	169201406	169201808	0	0	0	0
SDHDP3	NC_000003	169427060	169428366	0	0	0	0
LOC100287726	NC_000003	169471279	169482915	9.1558816	11.802716	31.390834	16.195849
TERC	NC_000003	169482398	169482848	0	0	0	0
ARPM1	NC_000003	169484709	169487683	1.1398045	1.4197278	1.2675021	1.6190709
MYNN	NC_000003	169490853	169505338	2.6082869	4.137727	9.4562715	8.8239518
LRRC34	NC_000003	169511261	169530312	0.3058332	1.0061718	2.1222061	2.5360894
LRRIQ4	NC_000003	169539710	169555561	0.1204061	0.0742741	0.1499633	0.1678076
LRRC31	NC_000003	169557029	169587759	0.0177425	0	0.0157842	0.0370911
KRT18P43	NC_000003	169620701	169622086	0	0	0	0
SAMD7	NC_000003	169629482	169656951	0.0194032	0.0199485	0	0
LOC100128164	NC_000003	169672018	169684522	0	0	0	0
SEC62	NC_000003	169684580	169716161	0.7726719	1.0085272	2.1936717	2.7717364
GPR160	NC_000003	169755735	169803184	0.1963923	0.2019119	1.6112701	1.1100378
PHC3	NC_000003	169805368	169899537	2.7294238	3.5192564	14.587539	13.316215
RNU7-32P	NC_000003	169878724	169878782	0	0	0	0
PRKCI	NC_000003	169940220	170023770	3.7703336	6.7719595	14.353386	13.581795
SKIL	NC_000003	170075473	170110950	6.8715045	21.685011	8.9680314	14.356479
CLDN11	NC_000003	170136653	170152479	61.139137	30.618669	0.8637999	0.1552873
SLC7A14	NC_000003	170177342	170303863	1.5049873	4.4902483	0.0154338	0.0181337
KRT8P13	NC_000003	170213154	170214885	0	0	0	0
RPL28P1	NC_000003	170371596	170372090	0	0	0	0
RPL22L1	NC_000003	170582664	170588045	2.3283831	2.1866649	8.2258251	4.9455356
EIF5A2	NC_000003	170606204	170626426	1.6509359	2.5868048	5.8819347	4.7068397
KLF7P	NC_000003	170670648	170712671	0	0	0	0
SLC2A2	NC_000003	170714137	170744768	0	0.0525541	0.0227378	0.0178103
TNIK	NC_000003	170781670	171177852	0.1291645	0.7303709	0.1340597	0.2550198
LOC100287142	NC_000003	171178401	171180045	0.1602975	0.247204	0.1663726	0.1303186
PLD1	NC_000003	171318616	171528273	0.4086015	0.3312211	3.6490129	3.5810246
LOC100287176	NC_000003	171394306	171395611	0	0	0	0
FLJ23172	NC_000003	171561139	171577108	0	0	0	0.0325623
FNDC3B	NC_000003	171757418	172118493	11.163818	15.35445	29.800445	26.367821
RPS27AP8	NC_000003	171943218	171943659	0	0	0	0
BZW1P1	NC_000003	172141976	172144954	0	0	0	0
GHSR	NC_000003	172162951	172166203	0.0748055	0.038454	0	0.1042549
TNFSF10	NC_000003	172223464	172241269	3.5978086	0.8733574	10.980215	0.7660572
SLC31A1P	NC_000003	172320217	172321904	0	0	0	0
AADACL1	NC_000003	172348435	172429006	11.804235	12.367758	17.456712	21.549518
ECT2	NC_000003	172472299	172539263	5.23782	8.3086614	21.218679	28.980064
RNU4P4	NC_000003	172514198	172514400	0	0	0	0
LOC100287755	NC_000003	172546521	172546962	0	0	0	0
SPATA16	NC_000003	172607147	172859032	0.0211188	0	0	0.0588657
NLGN1	NC_000003	173116244	174001116	2.1536242	1.3689156	1.8761744	3.4560406
LOC100287781	NC_000003	174020727	174021073	0	0	0	0
RPL8P4	NC_000003	174094999	174095830	0	0	0	0
NAALADL2	NC_000003	174577111	175523428	0.4115673	0.1839715	0.6367682	0.7606342
UBE2V1P2	NC_000003	175435790	175436470	0	0	0	0
LOC442097	NC_000003	175695281	175698355	0	0	0	0
LOC100129423	NC_000003	175785809	175786245	0	0	0	0
LOC100131216	NC_000003	175831485	175832323	0	0	0	0
TBL1XR1	NC_000003	176738542	176915048	9.4203538	12.244357	30.782618	30.704363
ASSP7	NC_000003	177014950	177018114	0	0	0	0
LOC100287937	NC_000003	178192927	178211443	0	0	0	0
KCNMB2	NC_000003	178254224	178562217	0.0633488	0	0.0140892	0
ZMAT3	NC_000003	178741502	178789584	2.1134168	1.3371167	12.470083	9.3020078
PIK3CA	NC_000003	178866311	178952500	4.8660354	8.2406151	15.967648	12.54861
KCNMB3	NC_000003	178960552	178984838	0.3186754	0.2829547	0.4510266	0.484507
ZNF639	NC_000003	179041551	179053320	3.9528612	6.8082524	13.417485	10.540325
MFN1	NC_000003	179065480	179111014	5.8197999	9.308885	14.699085	19.642043
GNB4	NC_000003	179113876	179169371	14.152589	21.68494	37.084296	40.382151

LOC100287236	NC_000003	179178778	179178969	0	0	0	0
LOC442098	NC_000003	179182134	179183137	0	0	0	0
ACTL6A	NC_000003	179280708	179306193	3.3664438	5.2864106	15.159025	14.605464
MRPL47	NC_000003	179306253	179322434	12.53008	24.350107	46.87077	50.652823
NDUFB5	NC_000003	179322575	179342288	7.9679514	11.221185	39.134485	30.653797
USP13	NC_000003	179370933	179507189	1.4514606	3.0300731	4.4553384	5.5435932
PEX5L	NC_000003	179518050	179754517	0.0122863	0.2021064	0.1202329	0.0599313
LOC647249	NC_000003	179930299	179930920	0	0	0	0
LOC131054	NC_000003	179997050	180042852	0	0	0	0
TTC14	NC_000003	180319918	180328918	1.2577622	1.7839279	2.3195548	3.4226676
CCDC39	NC_000003	180331796	180397283	0.0684197	0.0468951	0.3043402	0.3973129
LOC647253	NC_000003	180488507	180491845	0	0	0	0
LOC100132223	NC_000003	180538332	180539408	0	0	0	0
LOC100130898	NC_000003	180545792	180547634	0	0	0	0
RPL32P10	NC_000003	180605458	180605863	0	0	0	0
FXR1	NC_000003	180630452	180694950	0.3825281	0.6991629	1.9158059	5.7063764
DNAJC19	NC_000003	180701503	180707530	3.010578	3.51001	11.762403	9.9271322
FAUP2	NC_000003	180960393	180960689	0	0	0	0
RPL7AP25	NC_000003	181222797	181223691	0	0	0	0
SOX2OT	NC_000003	181328151	181459009	0	0	0	0
SOX2	NC_000003	181429722	181432224	0.0175582	0.0180517	0	0
LOC100132918	NC_000003	182178205	182178860	0	0	0	0
LOC402152	NC_000003	182328457	182329210	0	0	0	0
ATP11B	NC_000003	182511291	182639423	5.8461592	7.7074664	18.737143	18.804897
DCUN1D1	NC_000003	182660559	182698326	4.7684947	5.1175363	13.259929	11.017961
MCCC1	NC_000003	182733006	182817365	2.6698334	3.5647654	9.0842076	9.0847765
LAMP3	NC_000003	182840003	182880667	0.0394626	0.4598131	0.1521306	0.0091664
MCF2L2	NC_000003	182895831	183145855	0.0276288	0.0568106	0.0901243	0.0256705
B3GNT5	NC_000003	182971032	182991179	0.0106593	0.0219177	0.2275871	0.0371391
KLHL6	NC_000003	183205319	183273499	0	0.0071493	0.0123726	0.0629942
LOC100131977	NC_000003	183331351	183333054	0	0	0	0
KLHL24	NC_000003	183353411	183402307	0.59349	0.9060101	3.8878905	2.6317928
YEATS2	NC_000003	183415606	183530408	3.731645	3.767021	13.760226	13.199648
MAP6D1	NC_000003	183533664	183543393	0.4522269	0.4649368	0.2743047	0.544315
LOC100288104	NC_000003	183535502	183545942	0.0536608	0.055169	0.0954764	0.112179
PARL	NC_000003	183547173	183602693	5.1667185	10.911856	19.659541	22.448123
RPSAP31	NC_000003	183605310	183606325	0	0	0	0
LOC647265	NC_000003	183613688	183628724	0	0	0	0
ABCC5	NC_000003	183637722	183735727	0.8214623	1.6123219	2.7792379	5.5464768
EEF1AL6	NC_000003	183744156	183745543	0	0	0	0
HTR3D	NC_000003	183749332	183757157	4.0360621	1.4343937	1.0417152	1.0590236
HTR3C	NC_000003	183770835	183778461	0	0	0.0460242	0.0540757
5-HT3c2	NC_000003	183794434	183802119	0	0	0	0
HTR3E	NC_000003	183817967	183824783	0	0	0	0.0480516
HSP90AA5P	NC_000003	183833630	183835791	0	0	0	0
EIF2B5	NC_000003	183852810	183863099	3.8367504	7.8735742	8.8637338	14.033761
RPS3P3	NC_000003	183867462	183868308	0	0	0	0
DVL3	NC_000003	183873284	183891314	4.219447	6.6409425	11.546989	17.484373
AP2M1	NC_000003	183892634	183901879	109.32689	203.44176	207.35817	366.93024
ABCF3	NC_000003	183903863	183911795	7.7746678	13.737722	15.718073	20.753951
VWA5B2	NC_000003	183948317	183960117	0.054662	0.033719	0.1556122	0.1142719
ALG3	NC_000003	183960117	183967313	7.8881442	11.930587	30.885584	36.923612
ECE2	NC_000003	183967445	184010819	0.2317307	0.725089	1.2548508	1.2848126
CAMK2N2	NC_000003	183977003	183979251	0.0323149	0.0332231	0.0287482	0.5629573
PSMD2	NC_000003	184017022	184026840	31.464648	89.346234	68.066159	179.79801
EIF4G1	NC_000003	184032356	184053146	16.637704	28.131619	34.558569	46.738053
SNORD66	NC_000003	184043484	184043559	0	0	0	0
FAM131A	NC_000003	184058258	184064063	1.6972121	3.8338921	3.2537	2.7068495
CLCN2	NC_000003	184064340	184079391	0.535022	0.5814907	1.1967255	0.9799961
POLR2H	NC_000003	184081194	184086363	10.920145	23.059495	55.860468	40.397982
THPO	NC_000003	184089773	184095932	0.0486961	0.1501941	0.0216607	0
CHRD	NC_000003	184097861	184107617	0.0624087	0.6416274	0.0111041	0.0260933
LOC391600	NC_000003	184217272	184218158	0	0	0	0
EPHB3	NC_000003	184279587	184300196	0.3332567	2.1735144	0.6578026	0.4644531
MAGEF1	NC_000003	184428155	184429836	5.7221538	7.6559272	15.109047	11.998689
VPS8	NC_000003	184529931	184770402	2.5339678	2.9543338	5.8642475	8.4951119

C3orf70	NC_000003	184795838	184870802	0.1936374	0.3598746	0.079507	0.0311387
EHHADH	NC_000003	184908412	184971837	1.6302817	1.8900713	1.3783409	0.418967
EIF2S2P2	NC_000003	184962954	184964355	0	0	0	0
MAP3K13	NC_000003	185080970	185200628	0.1976867	0.114324	0.2088428	0.2496827
RPL4P4	NC_000003	185135196	185136622	0	0	0	0
TMEM41A	NC_000003	185207389	185216845	1.9587822	4.2514273	8.3256931	7.9622517
LIPH	NC_000003	185225570	185270369	0.2486965	0.1643697	1.1062367	0.8417509
SENP2	NC_000003	185304031	185348889	1.7178014	2.062782	4.5723857	3.4953348
RPL34P10	NC_000003	185324363	185324662	0	0	0	0
IGF2BP2	NC_000003	185361527	185542827	2.957018	3.4216796	10.277622	19.562882
C3orf65	NC_000003	185431040	185435955	0	0	0	0
TRA2B	NC_000003	185632356	185655924	3.0220812	4.4446144	4.9031446	6.760317
LOC344887	NC_000003	185677764	185698101	0	0	0	0
ETV5	NC_000003	185764106	185826901	14.635125	13.680591	36.095193	29.990282
DGKG	NC_000003	185864990	186080023	0.6135482	0.2959271	1.1994772	1.1190062
CRYGS	NC_000003	186256232	186262167	0.052195	0.160986	0.0464342	0.4728307
TBCCD1	NC_000003	186263856	186288332	0.7533983	2.1659345	2.6313282	3.2472093
DNAJB11	NC_000003	186288467	186303589	0.9367296	1.7656036	3.0092857	5.4214557
LOC100287402	NC_000003	186314765	186315089	0.2704507	0.5561034	0.3609006	0
AHSG	NC_000003	186330850	186339107	0	0	0	0.0389382
FETUB	NC_000003	186358149	186370797	0	0	0	0.0188229
HRG	NC_000003	186383798	186396023	0.0900579	0.0231472	0.1602359	0.1255118
LOC647280	NC_000003	186404333	186410331	0	0	0	0
KNG1	NC_000003	186435120	186461743	0.055004	0.01885	0.016311	0.0127763
PSMD10P2	NC_000003	186478900	186481614	0	0	0	0
LOC100132058	NC_000003	186482899	186491265	0	0	0	0
EIF4A2	NC_000003	186501361	186507686	10.179442	15.387684	46.664836	41.572746
SNORD2	NC_000003	186502585	186502654	0	0	0	0
SNORA81	NC_000003	186504464	186504641	0	0	0	0
SNORA63	NC_000003	186505088	186505222	0	0	0	0
SNORA4	NC_000003	186505402	186505538	0	0	0	0
RFC4	NC_000003	186507681	186524484	7.2600484	14.842719	33.846758	30.315926
ADIPOQ	NC_000003	186560463	186576252	0.4991935	0.246742	0.5978221	0.2742726
RPS20P14	NC_000003	186617862	186618386	0	0	0	0
RPL29P9	NC_000003	186639941	186640589	0	0	0	0
ST6GAL1	NC_000003	186648516	186796341	0.2618853	0.2393294	1.1821599	0.202769
RPL39L	NC_000003	186838741	186857263	22.31795	36.763735	85.92566	63.776418
RPL39P19	NC_000003	186838742	186839137	0	0	0	0
RTP1	NC_000003	186915274	186919253	0	0.0407608	0.0176353	0.0276273
MASP1	NC_000003	186935938	187009752	0.3323566	5.0949548	0.2956737	0.1282068
RTP4	NC_000003	187086168	187089372	6.8479493	5.3025886	7.8272247	0.7852533
SST	NC_000003	187386694	187388201	0.0695383	0	0	0
RTP2	NC_000003	187416047	187420345	0.032651	0	0.0290472	0.0227525
LOC100131635	NC_000003	187420101	187451637	0.2615966	0.336186	0.9308944	0.2734364
BCL6	NC_000003	187439165	187463475	1.585128	0.6541666	5.3824944	3.3215197
LOC100287440	NC_000003	187455564	187457792	0.0197166	0	0	0.0137393
LOC339929	NC_000003	187868994	187871845	0.4776982	0.2534833	0.4661	0.6764963
FLJ42393	NC_000003	187896331	187898596	0	0	0	0
LPP	NC_000003	187930721	188597441	1.761804	2.5022355	4.7505239	7.7669653
LOC647299	NC_000003	188707171	188707924	0	0	0	0
LOC100132319	NC_000003	188868602	188868763	0	0	0	0
TPRG1	NC_000003	188889763	189041271	0.0240351	0.0123553	0.0534558	0.025123
TP63	NC_000003	189349216	189615068	0.1549371	0.0207772	0.4015231	0.0563302
LEPREL1	NC_000003	189674517	189840226	7.5965424	4.5252314	13.873971	45.283405
LOC391603	NC_000003	189884553	189885424	0	0	0	0
CLDN1	NC_000003	190023493	190040215	0.487032	0.2108295	3.0671464	0.8841828
LOC100288275	NC_000003	190030520	190040232	0.187813	0.1287276	0	0.0436252
CLDN16	NC_000003	190105841	190128333	0.058833	0.0302432	0.0261697	0.1434901
TMEM207	NC_000003	190146444	190167665	0	0	0	0.0211498
IL1RAP	NC_000003	190231891	190369301	1.9849646	2.2046681	2.8367543	2.8441752
LOC100130481	NC_000003	190432600	190434188	0	0	0	0
LOC100131685	NC_000003	190522927	190549983	0	0	0	0
LOC647309	NC_000003	190572902	190573894	0.0885161	0.2275096	0.1181195	0.0308408
SNAR-I	NC_000003	190595719	190595839	0	0	0	0
OSTN	NC_000003	190930322	190967910	0.3279719	0	0.0972576	0
UTS2D	NC_000003	190984944	191048325	0.1095056	0.0750555	0.1786018	0.1271797

CCDC50	NC_000003	191046874	191116459	3.8305532	4.539041	10.38495	11.193874
PYDC2	NC_000003	191178952	191179245	0	0	0	0
LOC100129725	NC_000003	191668900	191669536	0	0	0	0
LOC100288383	NC_000003	191670070	191670563	0	0	0	0
FGF12	NC_000003	191859684	192445388	0.1900585	0.1264354	0.5967576	0.3583679
LOC100132663	NC_000003	192232633	192234362	0	0	0	0
C3orf59	NC_000003	192514604	192635950	1.1874374	2.9111634	1.1492455	1.1366122
VEZF1L1	NC_000003	192871330	192874996	0	0	0	0
HRASLS	NC_000003	192958918	192988644	0	0.0425456	1.141266	1.2111534
MGC2889	NC_000003	192959568	192961761	0	0	0	0
ATP13A5	NC_000003	192992831	193096514	0.0120176	0.0247106	0.0320735	0.0334973
ATP13A4	NC_000003	193119866	193272696	0	0.0740018	0.118921	0.0214962
OPA1	NC_000003	193311112	193415315	2.944947	5.201075	10.20726	8.6556299
LOC100128023	NC_000003	193677565	193721823	0.0927178	0	0	0
HES1	NC_000003	193853934	193856396	0.4234516	0.5597393	0.322898	0.063231
LOC100131551	NC_000003	194018988	194030593	0	0	0	0
CPN2	NC_000003	194060494	194072057	0.0145139	0.0596875	0.038736	0.0505695
LRRC15	NC_000003	194075976	194090472	0.8878431	28.889993	0.0526567	0.0154671
GP5	NC_000003	194115550	194119995	0.0125818	0.0905479	0.0111931	0.03507
ATP13A3	NC_000003	194123403	194188968	20.366839	34.140352	51.379579	55.670579
FLJ34208	NC_000003	194206901	194208740	0.4666318	0.7850398	1.6227754	0.6503353
LOC100288802	NC_000003	194283707	194284730	0	0	0.2917729	0
TMEM44	NC_000003	194308402	194354113	3.0173413	3.892131	4.6516933	4.1790879
LOC100132805	NC_000003	194324923	194366132	0.0723428	0.2107319	0.0429054	0.0840189
LSG1	NC_000003	194361517	194393206	1.0950147	2.0238926	1.8388553	2.8464333
FAM43A	NC_000003	194406622	194409766	2.4314761	6.5368674	0.1740432	0.107114
LOC285303	NC_000003	194476318	194478151	0	0	0	0
LOC100288491	NC_000003	194711695	194712282	0	0	0	0
C3orf21	NC_000003	194789013	194991895	3.004179	6.7418086	5.1009319	6.2915498
ACAP2	NC_000003	194995474	195163817	4.3451122	5.8805936	14.845173	8.9124768
RPL31P22	NC_000003	195070404	195070759	0	0	0	0
PPP1R2	NC_000003	195241221	195270224	5.3239854	7.9237117	12.100285	7.4464299
RPL24P6	NC_000003	195281388	195281945	0	0	0	0
APOD	NC_000003	195295573	195311076	0.1166767	0.1999266	0.0691992	0.0271017
LOC651714	NC_000003	195342087	195355148	0	0	0	0
LOC100288553	NC_000003	195373624	195375996	0	0	0	0
LOC100288016	NC_000003	195376772	195378509	0	0	0	0
SDHALP2	NC_000003	195384910	195415742	0	0	0	0
LOC440993	NC_000003	195428420	195435418	0	0	0	0
MUC20	NC_000003	195447753	195464540	0.1190113	0.1971293	0.1117577	0.0783245
MUC4	NC_000003	195473636	195539148	0.0052551	0.0162083	0.0233753	0.0201407
TNK2	NC_000003	195590236	195635880	0.7359534	1.1956741	1.5923549	1.4118974
LOC100133326	NC_000003	195664114	195665682	0	0	0	0
LOC727978	NC_000003	195666196	195668729	0.0325301	0	0.1446986	0.3173563
SDHALP1	NC_000003	195686792	195717150	0	0	0	0
LOC100131360	NC_000003	195719846	195725757	0	0	0	0
LOC100288115	NC_000003	195726134	195727926	0	0	0	0
TFRC	NC_000003	195776155	195809032	19.073284	24.686607	96.979684	97.467507
RNU7-18P	NC_000003	195799691	195799749	0	0	0	0
FLJ25996	NC_000003	195869708	195870981	0	0	0	0
ZDHHC19	NC_000003	195924323	195938300	0	0	0	0
OSTalpha	NC_000003	195943383	195960301	0.0604099	0.0621078	0.1074847	0.1052401
PCYT1A	NC_000003	195965253	196014584	5.7468629	7.9634676	11.73174	22.402785
TCTEX1D2	NC_000003	196018098	196045145	2.2277906	3.1232766	13.87333	9.1733506
TM4SF19	NC_000003	196050419	196065258	0.1287546	0.2206221	0.3054497	0.7177706
UBXN7	NC_000003	196080361	196159345	2.4013907	4.5564696	11.193122	8.0374153
LOC727900	NC_000003	196195654	196197937	0.269385	0.4549992	1.2153797	1.246985
RNF168	NC_000003	196198628	196230582	1.1006034	1.5802485	1.9751361	2.6578586
C3orf43	NC_000003	196233750	196242237	0.0971232	0.0249632	0.0432017	0.0169198
RPS29P3	NC_000003	196263373	196263663	0	0	0	0
WDR53	NC_000003	196281059	196295413	1.510633	1.9557424	4.8032019	3.6843425
FBXO45	NC_000003	196295725	196315930	0.1533167	0.3388952	0.5933173	0.8226463
LRRC33	NC_000003	196366656	196388875	1.7650608	1.3564186	2.6170784	2.8574937
C3orf34	NC_000003	196433148	196439123	0.8138561	1.129585	3.2038283	2.2543313
PIGX	NC_000003	196439246	196462878	1.4312089	2.3334849	5.6588582	6.5984524
PAK2	NC_000003	196466728	196559518	4.9610889	7.2275779	14.488836	20.153854

SENP5	NC_000003	196594727	196661585	1.8741399	2.8866378	9.6008448	7.758173
NCBP2	NC_000003	196662273	196669464	3.0784015	5.6218979	16.467823	21.931364
LOC152217	NC_000003	196669494	196670884	0	0	0	0
PIGZ	NC_000003	196673214	196695704	0.2928797	0.334568	3.6911808	2.2223162
RPSAP30	NC_000003	196717463	196727294	0	0	0	0
LOC440995	NC_000003	196728611	196729547	1.6885126	2.1699606	4.1309061	0.4575756
MFI2	NC_000003	196730156	196756686	2.0857244	2.9467436	5.4827572	1.2283708
DLG1	NC_000003	196769431	197025447	2.6188567	1.9435639	5.5776519	10.568846
LOC100129516	NC_000003	197233149	197233618	0	0	0	0
BDH1	NC_000003	197236654	197300194	1.0602853	1.0655885	1.748739	1.8927819
RPL36P7	NC_000003	197307221	197307500	0	0	0	0
LOC220729	NC_000003	197340898	197354752	0	0	0	0
LOC100131868	NC_000003	197361186	197362930	0	0	0	0
LOC728210	NC_000003	197362937	197366122	0	0	0	0
KIAA0226	NC_000003	197398259	197476568	1.7747621	1.8998849	8.0083273	5.4823887
FYTTD1	NC_000003	197476683	197511187	6.5975842	8.005174	22.81132	17.776843
LRCH3	NC_000003	197518145	197598456	1.9852611	2.9015028	6.1815017	7.0391106
RPL17P18	NC_000003	197577225	197577861	0	0	0	0
IQCG	NC_000003	197615946	197686886	0.374825	0.4624314	0.8336368	0.7182807
RPL35A	NC_000003	197677052	197682722	72.369299	168.22415	459.23484	256.95791
LMLN	NC_000003	197687071	197770591	0.3194448	0.4357918	2.1204718	3.2448501
LOC348840	NC_000003	197784402	197807542	0	0	0	0
LOC100129091	NC_000003	197807547	197808805	0	0	0	0
LOC100128827	NC_000003	197836989	197837809	0	0	0	0
LOC728247	NC_000003	197844118	197847649	0	0	0	0
LOC100288736	NC_000003	197872324	197878700	0	0	0	0
FAM157A	NC_000003	197879237	197907728	0.172346	0	0.3373123	0.3602927
LOC100133150	NC_000003	197945374	197950628	0	0	0	0
LOC100288770	NC_000003	197948675	197950628	0	0	0	0
LOC100288351	NC_000003	197951445	197952832	0	0	0	0
ZNF595	NC_000004	53227	88099	0.9834569	0.9321051	0.7518763	1.1564639
LOC100129037	NC_000004	105771	107949	0	0	0	0
LOC100287931	NC_000004	123966	125449	0	0	0	0
ZNF718	NC_000004	124420	156491	0.7171563	0.898599	0.6978148	1.3586764
LOC100288172	NC_000004	195200	199049	0	0	0	0
ZNF732	NC_000004	206394	249445	0	0	0	0
LOC100288199	NC_000004	206686	207175	0	0	0	0
LOC654254	NC_000004	264464	299110	0.601207	0.9477595	0.0713134	0.1256835
ZNF141	NC_000004	331596	367691	1.0438374	1.293313	1.4524674	1.4920769
LOC100288237	NC_000004	367742	369262	0	0	0	0
ABCA11P	NC_000004	419224	467998	0	0	0	0
ZNF721	NC_000004	433777	493442	0.7990588	0.5505908	1.1646083	1.3387278
PIGG	NC_000004	492989	533320	5.4766258	6.7288512	5.0044884	5.3240173
PDE6B	NC_000004	619363	664681	0.29745	0.4714568	0.0220516	0.0259094
ATP5I	NC_000004	666225	668122	29.665056	49.199704	58.211931	49.765427
MYL5	NC_000004	671711	675817	3.1913996	1.9823278	2.0702191	2.6408745
MFSD7	NC_000004	675618	682973	2.3552107	2.1066216	0.1257156	0.0328241
PCGF3	NC_000004	699573	764428	3.2499663	3.9154695	3.0706701	4.2699707
LOC100128084	NC_000004	729829	731544	0	0	0	0
LOC100129917	NC_000004	774588	775631	0.3367681	0.1731165	0.2246987	0.3520101
CPLX1	NC_000004	778745	819945	0.0205366	0.0422275	0.0548097	0.0572428
GAK	NC_000004	843065	926174	4.112569	3.9987959	3.2272102	4.3595335
TMEM175	NC_000004	926262	952444	3.594187	2.1151846	2.9108171	3.3163997
DGKQ	NC_000004	952675	967344	2.6638165	1.8030477	2.1589682	1.5589886
SLC26A1	NC_000004	972861	987224	0.0214801	0.099377	0.0286639	0.0598727
IDUA	NC_000004	980785	998317	2.4326331	0.7027611	1.2877515	0.3362292
FGFRL1	NC_000004	1005610	1020686	11.798274	12.953413	14.010867	18.041904
RNF212	NC_000004	1065266	1107582	0.0670965	1.6038383	0	0.0233778
FLJ35816	NC_000004	1145226	1147489	0	0	0	0
SPON2	NC_000004	1160720	1166980	0.9407878	1.3356969	0.1195644	0.0780451
LOC100130872	NC_000004	1189571	1202750	0	0	0	0
CTBP1	NC_000004	1205228	1242908	7.2745563	9.717252	9.006769	8.8803513
C4orf42	NC_000004	1244177	1246616	1.5489951	1.5925297	1.7625952	2.5102359
MAEA	NC_000004	1283672	1333925	8.1512024	7.8849061	6.0548537	11.360165
KIAA1530	NC_000004	1341104	1381837	0.9703468	0.9007624	0.9219147	1.0438061
CRIPAK	NC_000004	1385340	1389782	0.5044699	0.2237306	0.5367885	0.8202477

NKX1-1	NC_000004	1396720	1400119	0.0355568	0.0365562	0.0632647	0.0247774
LOC100289589	NC_000004	1604677	1659196	0.2111446	0.124045	0.0536686	0.1681531
FAM53A	NC_000004	1641608	1685718	0.4183952	0.2580925	0.2382182	0.2215814
SLBP	NC_000004	1694527	1714030	42.088394	42.956973	56.345825	46.336772
TMEM129	NC_000004	1717679	1723084	5.0655875	3.4878974	4.3135488	3.8969267
TACC3	NC_000004	1723266	1746898	5.6416824	7.5549378	3.5849979	9.195172
FGFR3	NC_000004	1795039	1810598	0.0615234	0.0843367	0.0364886	0.0500173
LETM1	NC_000004	1813206	1857974	3.1701947	4.2354269	3.1424078	6.6778158
WHSC1	NC_000004	1873123	1983934	1.7937529	2.6485088	3.0541754	4.338091
SCARNA22	NC_000004	1976363	1976487	0	0	0	0
LOC100289148	NC_000004	1976514	1977373	0.0765649	0.2361502	0	0.0533534
WHSC2	NC_000004	1984443	2010959	2.2304218	2.439867	2.8890609	4.3643249
C4orf48	NC_000004	2043689	2045697	2.6520485	3.3108527	1.9380259	1.3860397
NAT8L	NC_000004	2061587	2067635	0.1501221	0.1157559	0	0
LOC100289625	NC_000004	2069193	2070816	0.0811852	0	0.0481497	0.0377154
POLN	NC_000004	2073645	2230958	0.2122371	0.654606	0.2157851	0.2429707
HAUS3	NC_000004	2230096	2243860	1.0751621	1.1134481	1.0961282	1.6406185
LOC100289662	NC_000004	2235978	2243636	0	0	0	0
MXD4	NC_000004	2249160	2263739	7.5479603	11.759899	8.922079	20.405763
ZFYVE28	NC_000004	2271325	2420369	0.951677	0.6925923	0.3900244	0.2831497
LOC441005	NC_000004	2427636	2452087	0	0	0	0
LOC402160	NC_000004	2451703	2464653	0.0318465	0	0.0283316	0
RNF4	NC_000004	2470807	2517584	8.455598	8.7392396	15.64168	20.659063
C4orf8	NC_000004	2627159	2734302	2.4513698	2.8162061	3.1390397	3.9599462
LOC100289183	NC_000004	2656498	2661397	0	0	0.0709575	0.0555805
TNIP2	NC_000004	2743387	2758061	2.7297038	4.4201154	4.1890249	4.2640229
SH3BP2	NC_000004	2794750	2842823	1.6589574	0.8721729	2.2892516	2.5288103
ADD1	NC_000004	2845584	2931789	2.3443378	2.6990085	2.9025233	6.5721531
MFSD10	NC_000004	2932295	2935769	8.5545221	3.7802846	8.0331367	4.4621337
C4orf10	NC_000004	2937273	2952794	0	0	0	0
NOP14	NC_000004	2939663	2965118	1.129581	1.0839061	1.1254954	1.7946724
GRK4	NC_000004	2965343	3042474	0.1709309	0.3319437	0.1351688	0.1191115
HTT	NC_000004	3076408	3245687	3.0266389	3.3900126	4.3377262	8.3454214
RNU7-33P	NC_000004	3080211	3080269	0	0	0	0
C4orf44	NC_000004	3250767	3265840	0	0	0.041177	0.0483805
LOC100286945	NC_000004	3314239	3314581	0	0	0	0
RGS12	NC_000004	3315874	3441640	0.6069161	0.5146173	0.6234236	0.893807
RPL7AP29	NC_000004	3325653	3326509	0	0	0	0
HGFAC	NC_000004	3443726	3451213	0.0215644	0.0443409	0	0
DOK7	NC_000004	3465033	3496209	0.0342543	0.0176085	0.0304736	0.0477395
LOC100289219	NC_000004	3510306	3512946	0.2163298	0.307952	0.2516693	0.7885239
LRPAP1	NC_000004	3514290	3534224	21.582221	17.037308	27.542616	37.081461
FLJ35424	NC_000004	3578596	3591045	0	0	0	0
LOC100129786	NC_000004	3643676	3644308	0	0	0	0
ADRA2C	NC_000004	3768296	3770253	0.7855915	0.6922891	0.0399362	0.0938454
LOC100131415	NC_000004	3891243	3891892	0	0	0	0
OR7E162P	NC_000004	3903233	3904267	0	0	0	0
LOC100131503	NC_000004	3912010	3912336	0	0	0	0
LOC728263	NC_000004	3937173	3945154	0	0	0	0
LOC348926	NC_000004	3943669	3957148	0	0	0	0
LOC100130441	NC_000004	3996883	3997056	0	0	0	0
RPS3AP16	NC_000004	4078394	4079242	0	0	0	0
LOC100130459	NC_000004	4106475	4109606	0	0	0	0
OR4D12P	NC_000004	4128333	4129357	0	0	0	0
UNC93B4	NC_000004	4147309	4149522	0	0	0	0
OR7E99P	NC_000004	4158206	4159230	0	0	0	0
OR7E43P	NC_000004	4176126	4176778	0	0	0	0
OTOP1	NC_000004	4190530	4228621	0.1175715	0.0241752	0.020919	0.0491571
TMEM128	NC_000004	4237269	4249934	2.5881753	3.1044023	2.9120011	1.6267256
LYAR	NC_000004	4269428	4291896	0.1642925	1.0979144	0.6577161	0.6296704
ZNF509	NC_000004	4291924	4323513	0.3882616	0.4605851	0.2789836	0.2601502
D4S234E	NC_000004	4387983	4420785	0.0703172	0.0867521	0.0625561	0.244999
STX18	NC_000004	4420695	4543775	3.054789	3.6012721	5.7976001	5.818443
LOC728015	NC_000004	4497925	4509510	0	0	0	0
LOC100289434	NC_000004	4853626	4855584	0.022434	0.2306452	0.0399158	0.0312658
MSX1	NC_000004	4861392	4865663	0.3171522	1.4905865	0.2821474	0.0631441

LDHAL1	NC_000004	4895351	4896995	0	0	0	0
CYTL1	NC_000004	5016313	5021197	0.131713	0.4062444	0	0.9790171
STK32B	NC_000004	5053527	5502725	0.8860531	0.9109557	0.0485081	0.0854913
C4orf6	NC_000004	5526883	5529528	0	0	0	0.1403202
EVC2	NC_000004	5564150	5710294	0.4787824	0.2973942	0.5412963	1.2094255
EVC	NC_000004	5712924	5816031	0.6151385	0.5410765	5.2961088	11.273575
CRMP1	NC_000004	5822491	5894785	0.1879736	0.4509322	0.8807265	2.8555276
C4orf50	NC_000004	5958845	5990166	0	0.0284351	0	0.0385461
JAKMIP1	NC_000004	6027926	6202318	0.0930543	0.0410013	0.0118262	0.0277903
LOC285484	NC_000004	6202466	6235083	0	0	0	0
WFS1	NC_000004	6271577	6304992	4.1050546	4.7293616	2.1911822	3.7523889
LOC100129623	NC_000004	6303624	6304993	0	0.0357747	0.0309561	0
PPP2R2C	NC_000004	6322305	6474326	0.0290919	0.0598192	0.008627	0.0270299
MAN2B2	NC_000004	6576902	6624188	3.5328494	2.2900593	4.0277313	3.1043606
MRFAP1	NC_000004	6642445	6644449	2.6357479	4.0352843	2.8545811	6.7079264
LOC93622	NC_000004	6675677	6677774	0	0	0	0
S100P	NC_000004	6695566	6698897	0	0.1771898	0.2299857	0.0600488
MRFAP1L1	NC_000004	6709428	6711606	0.1104227	0.2270523	0.2455877	0.288551
CNO	NC_000004	6717842	6719387	1.2507906	1.2567182	0.8345535	0.8121734
KIAA0232	NC_000004	6784459	6885899	1.0513128	1.0521138	2.7411577	2.7939989
TBC1D14	NC_000004	6911171	7034845	4.2535938	4.0037013	4.8844078	8.1992448
CCDC96	NC_000004	7042576	7044728	0.1224753	0.1049313	0.0544787	0.0142243
TADA2B	NC_000004	7045156	7059679	1.7224608	1.9699628	1.0880584	1.1576659
GRPEL1	NC_000004	7061780	7069800	6.8084694	6.9096958	5.797046	7.2896984
SORCS2	NC_000004	7194374	7744564	0.0580846	0.4180192	0.0258368	0.0404756
TRNASUP5P	NC_000004	7325838	7325909	0	0	0	0
PSAPL1	NC_000004	7432021	7436700	0	0	0	0.0065438
LOC84740	NC_000004	7755817	7780655	0	0	0	0
AFAP1	NC_000004	7760440	7941653	6.6176874	6.0244536	1.9523624	3.4448061
LOC389199	NC_000004	7940728	7942023	0	0	0	0
ABLIM2	NC_000004	7967037	8160559	0.036381	0.0374035	0.0242742	0.0697172
SH3TC1	NC_000004	8201060	8242830	3.0433635	0.9757918	0.5873813	0.3019354
HTRA3	NC_000004	8271492	8308834	1.5070935	3.7578628	6.1643777	3.9835277
ACOX3	NC_000004	8368009	8442452	1.575041	2.1066722	1.5100312	2.7705179
C4orf23	NC_000004	8456143	8478281	1.3533491	1.8063597	2.154485	2.4652117
LOC100287044	NC_000004	8475034	8477686	0.0875463	0.0900068	0.0778836	0
GPR78	NC_000004	8582291	8589520	0	0	0.020143	0.0315558
CPZ	NC_000004	8594435	8621488	1.6048512	0.1269197	0.7060154	0.2457855
LOC100287077	NC_000004	8620970	8638654	0.4242107	0.0872266	0.3019117	0.1182428
HMX1	NC_000004	8868773	8873543	0.0231794	0.0238309	0.0618632	0.0646094
LOC650293	NC_000004	8951477	8952127	0	0	0	0
LOC100288392	NC_000004	8957227	8998383	0	0	0	0
LOC100288430	NC_000004	9002184	9005344	0	0	0	0
LOC100286946	NC_000004	9032000	9033017	0	0	0	0
LOC100288460	NC_000004	9035293	9042843	0	0	0	0
LOC100286982	NC_000004	9114948	9115121	0	0	0	0
LOC100287013	NC_000004	9155022	9167181	0	0	0	0
LOC100288492	NC_000004	9167906	9174517	0	0	0	0
LOC100287045	NC_000004	9172114	9178456	0	0	0	0.0121769
LOC100287106	NC_000004	9192736	9199930	0	0	0.1480968	0.1160033
LOC100287144	NC_000004	9212383	9213975	0	0	0	0
LOC100287178	NC_000004	9217131	9218723	0	0	0	0
LOC100287205	NC_000004	9221878	9223470	0	0	0	0
LOC100287238	NC_000004	9226622	9228214	0	0	0	0
LOC100287270	NC_000004	9231367	9232959	0	0	0	0
LOC100288520	NC_000004	9236111	9238060	0.078479	0	0	0.0546873
LOC100287302	NC_000004	9240856	9242448	0	0	0	0
LOC100287327	NC_000004	9245605	9247197	0	0	0	0
LOC100287364	NC_000004	9250356	9251948	0	0	0	0
LOC100287404	NC_000004	9255104	9256696	0	0	0	0
LOC100287441	NC_000004	9259850	9261442	0	0	0	0
LOC100287478	NC_000004	9264598	9266190	0	0.0283637	0	0
LOC100287513	NC_000004	9269345	9270937	0	0	0	0
LOC728369	NC_000004	9326891	9328483	0	0	0	0
LOC728373	NC_000004	9331637	9333229	0	0	0	0
LOC728379	NC_000004	9336384	9337976	0	0	0	0

USP17L5	NC_000004	9341129	9342721	0	0	0	0
LOC728393	NC_000004	9345874	9347466	0	0	0	0
LOC728400	NC_000004	9350619	9352211	0	0	0	0
LOC728405	NC_000004	9355364	9356956	0	0	0	0
USP17	NC_000004	9360109	9361701	0	0	0	0
LOC728419	NC_000004	9364855	9366447	0	0	0	0
DUB4	NC_000004	9369600	9370796	0	0	0	0
LOC100287791	NC_000004	9382671	9383119	0	0	0	0
LOC728429	NC_000004	9385743	9390709	0	0	0	0
LOC100133128	NC_000004	9400930	9405291	0	0	0	0
LOC100288554	NC_000004	9423738	9431428	0	0	0	0
DEFB131	NC_000004	9446260	9452240	0	0	0	0
OR7E86P	NC_000004	9460848	9462070	0	0	0	0
OR7E84P	NC_000004	9470652	9471875	0	0	0	0
OR7E85P	NC_000004	9485250	9486477	0	0	0	0
OR7E83P	NC_000004	9514472	9515636	0	0	0	0
LOC100132613	NC_000004	9533665	9534392	0	0	0	0
RPS24P11	NC_000004	9554655	9555747	0	0	0	0
RPS3AP19	NC_000004	9564767	9565614	0	0	0	0
LOC100128096	NC_000004	9651129	9651688	0	0	0	0
LOC644517	NC_000004	9694721	9705218	0	0	0	0
LOC100132066	NC_000004	9704468	9712436	0	0	0	0
LOC100129927	NC_000004	9748380	9748705	0	0	0	0
OR7E35P	NC_000004	9756416	9757529	0	0	0	0
DRD5	NC_000004	9783258	9785633	0	0.0190166	0	0.0128893
SLC2A9	NC_000004	9827848	10041872	0.169113	0.086933	0.0376119	0.1178446
WDR1	NC_000004	10075963	10118573	69.131422	66.416582	52.159113	65.020621
LOC100130161	NC_000004	10168599	10169365	0	0	0	0
LOC100129344	NC_000004	10197169	10239955	0	0	0	0
RAF1P1	NC_000004	10256550	10258283	0	0	0	0
LOC100287951	NC_000004	10412622	10413274	0	0	0	0.0872504
ZNF518B	NC_000004	10441504	10459032	1.7424688	2.8597914	2.1307497	2.1017073
LOC100130072	NC_000004	10480154	10480556	0	0	0	0
CLNK	NC_000004	10491838	10686386	0.0262065	0.053886	0	0.0182617
LOC643446	NC_000004	11370224	11375375	0.9030459	0.3920021	1.0711662	0.9369255
HS3ST1	NC_000004	11399988	11430537	0.0223655	0.1379646	0	0.0155852
HSP90AB2P	NC_000004	13335037	13339925	0	0	0	0
RAB28	NC_000004	13369382	13485956	1.4372055	2.1775133	4.822706	3.3910508
NKX3-2	NC_000004	13542454	13546114	0.039222	0.0201622	0.1221254	0.1093258
LOC285548	NC_000004	13547700	13549448	0	0	0	0
BOD1L	NC_000004	13570366	13629328	0.2042746	0.231446	0.3337868	1.0487176
LOC100288683	NC_000004	13633438	13634756	0	0	0	0
RPL32P12	NC_000004	13649318	13650024	0	0	0	0
LOC391636	NC_000004	13978790	13979828	0	0	0	0
LOC441009	NC_000004	15003373	15004054	0.3866413	0	0.1146556	0.2245226
CPEB2	NC_000004	15005522	15071774	7.2479046	1.8548722	5.2111559	4.6261145
C1QTNF7	NC_000004	15341560	15447791	0.0363284	0	0.0080797	0.0189863
LOC100288116	NC_000004	15437358	15444083	0	0.0829053	0	0
CC2D2A	NC_000004	15471489	15603180	1.4767	1.0964798	0.8174216	0.954705
FBXL5	NC_000004	15606154	15657013	6.6270139	8.6726127	7.8645175	7.0767704
LOC285550	NC_000004	15683352	15692070	0.9621506	1.0201042	1.1056096	1.2082335
BST1	NC_000004	15704573	15733796	8.7896464	4.7931041	0.1585036	0.4345422
RPL10AP7	NC_000004	15732553	15733284	0	0	0	0
LOC100288771	NC_000004	15744849	15745301	0	0	0	0
CD38	NC_000004	15779931	15850706	0.1768541	0	0	0.0616195
LOC100130067	NC_000004	15866533	15912551	0	0	0	0
FGFBP1	NC_000004	15937192	15939971	0.0371812	0.1529049	0	0.0518187
FGFBP2	NC_000004	15961863	15964859	0	0.0385853	0	0
PROM1	NC_000004	15969857	16077566	0.1158361	0.1190917	0.103051	0.0807192
TAPT1	NC_000004	16162128	16228161	1.3314743	1.5363145	2.7780746	2.1560235
FLJ39653	NC_000004	16228370	16230374	0	0	0	0
RPS21P4	NC_000004	16257860	16258224	0	0	0	0
LDB2	NC_000004	16503164	16900424	2.8993056	4.0249098	2.0546988	4.0749465
RPS7P6	NC_000004	17429288	17429972	0	0	0	0
QDPR	NC_000004	17488016	17513857	6.5138033	4.0181247	3.9026608	3.4089399
LOC100288832	NC_000004	17490713	17513857	0	0.1152638	0	0

CLRN2	NC_000004	17516788	17528727	0	0	0	0.0372113
LOC645108	NC_000004	17553737	17554374	0	0	0	0
LAP3	NC_000004	17578927	17609590	46.668837	30.079236	46.11651	11.943703
MED28	NC_000004	17616273	17626160	9.1258623	7.6187469	12.055846	11.642713
FAM184B	NC_000004	17633696	17783135	0.1026029	0.0937658	0.1318465	0.0953304
C4orf30	NC_000004	17802278	17812381	3.6623526	3.1592616	6.4045045	5.4812337
NCAPG	NC_000004	17812525	17846488	4.0141395	6.5101299	8.8438962	10.702948
LCORL	NC_000004	17844843	18023385	1.1401963	1.1461918	2.4006288	3.531263
LOC100288868	NC_000004	17907939	17914601	0	0	0	0
RPL21P46	NC_000004	19815356	19815892	0	0	0	0
SLIT2	NC_000004	20255235	20620788	2.3882978	10.195931	2.7565758	3.0315536
PACRGL	NC_000004	20702036	20729980	1.3568195	1.5039338	3.1685437	2.8807773
LOC100288212	NC_000004	20702036	20706443	0.1443292	0.0741928	0.1283992	0.0502872
KCNIP4	NC_000004	20730239	21950374	0.2381211	0.0765042	0.4236783	0.6948415
RPL31P25	NC_000004	21658146	21658471	0	0	0	0
NCRNA00099	NC_000004	21844964	21854811	0	0	0	0
LOC100131836	NC_000004	21857816	21859321	0	0	0	0.0406705
GPR125	NC_000004	22388999	22517672	1.802263	3.0815873	7.4079598	9.6844462
RPS27P13	NC_000004	22595784	22596130	0	0	0	0
GBA3	NC_000004	22694548	22821192	0.0204696	0.0420898	0.0364206	0.0285281
PPARGC1A	NC_000004	23793644	23891700	0.4104061	0.0500607	0.4331797	0.4556408
LOC729175	NC_000004	24473456	24484502	0.0968023	0.0497615	0	0.0337278
DHX15	NC_000004	24529087	24586184	27.540503	33.063584	32.084375	46.120092
ATP5LP3	NC_000004	24659311	24660020	0	0	0	0
LOC391640	NC_000004	24769632	24773919	0	0	0	0
SOD3	NC_000004	24797085	24802467	3.2479727	4.6099481	1.8922301	0.5007338
LOC100288285	NC_000004	24807739	24810165	0.2897288	0.2978716	0.3221884	0.8706702
CCDC149	NC_000004	24809392	24981770	0.1906648	0.1764211	0.1413506	0.3985884
LGI2	NC_000004	25000471	25032414	0.1247938	0.2209632	0.0678456	0.0193247
SEPSECS	NC_000004	25123722	25161985	1.3457579	1.6006129	2.2535973	1.4250544
PI4K2B	NC_000004	25235653	25280831	6.8344931	4.6174651	8.9356558	8.4109982
ZCCHC4	NC_000004	25314396	25372005	0.9741832	1.8415831	1.56558	1.6095306
ANAPC4	NC_000004	25378848	25420120	2.9605845	2.5534033	2.5752888	4.229259
LOC645433	NC_000004	25506619	25508297	0	0	0	0
LOC100288962	NC_000004	25623088	25625223	0	0	0	0.0502872
SLC34A2	NC_000004	25657435	25680370	0.0211646	0.0108797	0.0094143	0.0221225
RPS29P11	NC_000004	25680442	25680731	0	0	0	0
KIAA0746	NC_000004	25749049	25864610	2.7823897	3.6750624	5.6553524	2.6524955
LOC389203	NC_000004	25915814	25931501	2.210414	2.0051806	4.0871222	2.6577804
LOC645481	NC_000004	26113487	26115461	0	0	0	0
RBPJ	NC_000004	26321332	26433278	15.949716	8.1666492	28.392613	18.776098
CCKAR	NC_000004	26483018	26492042	0.1045142	0.0537258	0.0232447	0.0546223
TBC1D19	NC_000004	26585546	26756917	2.0385264	2.9382567	1.7601905	2.1447164
STIM2	NC_000004	26862364	27025809	1.6302107	1.9303219	1.5603019	2.4130116
MRPL51P1	NC_000004	27424555	27424817	0	0	0	0
LOC391642	NC_000004	27586769	27587773	0	0	0	0
LOC645641	NC_000004	28824866	28825805	0	0	0	0
LOC100131674	NC_000004	29455415	29472050	0	0	0	0
RPS3AP17	NC_000004	29964661	29965378	0	0	0	0
LOC100130674	NC_000004	30008566	30009986	0	0	0	0
PCDH7	NC_000004	30722037	31148421	0.0266218	0.9077735	0.0671033	0.0371023
LOC642305	NC_000004	33012567	33013367	0	0	0	0
RPL31P31	NC_000004	33969769	33970189	0	0	0	0
LOC651644	NC_000004	36066402	36067321	0	0	0	0
ARAP2	NC_000004	36067622	36245979	0.0179137	0.0429733	0.0265608	0.0332879
FLJ16686	NC_000004	36283244	36345958	0	0	0.0141966	0.0111201
LOC100130532	NC_000004	36498556	36511507	0	0	0	0
KIAA1239	NC_000004	37246690	37451087	0.2116004	0.0483439	0.0993518	0.2293692
C4orf19	NC_000004	37455552	37595132	0.0463345	0.0357275	0.0206102	0.0403596
RELL1	NC_000004	37592422	37687999	1.165129	1.3546064	2.3830528	3.9684865
RPL21P45	NC_000004	37822943	37823500	0	0	0	0
PGM2	NC_000004	37828282	37864559	5.4697768	5.8188631	4.383081	6.8948537
GAFA3	NC_000004	37868283	37868438	0	0.2896372	0.2506254	0
TBC1D1	NC_000004	37892720	38140794	6.5288776	8.6665069	6.1519552	9.8744773
PTTG2	NC_000004	37962056	37962631	0	0	0	0
PSME2P4	NC_000004	37997072	37997890	0	0	0	0

MRPS33P2	NC_000004	38008405	38008726	0	0	0	0
FLJ13197	NC_000004	38614322	38666249	0	0	0	0
KLF3	NC_000004	38665790	38703129	2.5672342	3.3555616	3.6486419	3.9106033
TLR10	NC_000004	38774234	38784589	0	0	0.0108786	0.0170422
TLR1	NC_000004	38797876	38806412	0.4007906	0.2535722	0.9873815	0.3544795
TLR6	NC_000004	38828408	38831160	0.5268041	0.4103106	0.724292	0.6452027
FAM114A1	NC_000004	38869437	38945428	35.150285	32.490237	32.83974	33.2131
TMEM156	NC_000004	38968422	39034041	0.0936065	0.072178	0.0416375	0
KLHL5	NC_000004	39046659	39123829	10.478628	11.206606	21.534551	14.742111
WDR19	NC_000004	39184024	39287430	1.7501508	1.2995226	1.3753347	2.3984971
RFC1	NC_000004	39289076	39367995	2.9880838	3.3126475	4.0915126	5.7010064
KLB	NC_000004	39408473	39453153	0.0361476	0.0520289	0.045021	0.0654916
RPL9	NC_000004	39455744	39460568	224.8856	211.35959	92.88396	401.09372
LIAS	NC_000004	39460665	39479273	0.6858973	0.9402326	0.9717892	0.7788986
LOC401127	NC_000004	39481875	39483525	0	0	0	0
UGDH	NC_000004	39500375	39529211	13.712289	11.122286	6.1055465	10.045037
C4orf34	NC_000004	39552550	39640481	18.201051	11.500733	8.8643211	7.5914148
RNU7-11P	NC_000004	39622632	39622692	0	0	0	0
UBE2K	NC_000004	39699664	39784412	3.2427008	3.618118	4.6141836	5.6286716
PDS5A	NC_000004	39824483	39979576	7.7028044	9.8292046	11.383292	11.989434
PABPCP1	NC_000004	39975056	39975997	0	0	0	0
KRT18P25	NC_000004	40021812	40023198	0	0	0	0
LOC344967	NC_000004	40044537	40058819	0	0	0	0
N4BP2	NC_000004	40058524	40157028	0.8439194	1.1921742	0.6762698	0.8529356
RHOH	NC_000004	40198527	40246281	0.0219851	0.022603	0	0.0459603
CHRNA9	NC_000004	40337469	40356973	0.0696854	0.0477626	0.0413294	0.0161865
RNU7-74P	NC_000004	40379470	40379528	0	0	0	0
RBM47	NC_000004	40425272	40631883	0.4857084	0.1635832	0.9461492	0.612731
RPL37P14	NC_000004	40493752	40494042	0	0	0	0
LOC100289402	NC_000004	40751749	40752863	0	0	0	0
NSUN7	NC_000004	40751914	40812002	0.2495708	0.0488733	0.1268715	0.0414074
ARL4P2	NC_000004	40787905	40789053	0	0	0	0
APBB2	NC_000004	40812044	41216635	2.7091376	2.4555094	1.7779603	2.971019
UCHL1	NC_000004	41258929	41270446	344.75036	190.32423	289.52946	254.52552
LIMCH1	NC_000004	41362804	41702061	0.1519744	0.6320847	0	0.033696
RPL12P20	NC_000004	41391132	41391610	0	0	0	0
OR5M14P	NC_000004	41725227	41725580	0	0	0	0
PHOX2B	NC_000004	41746099	41750987	0.0580175	0.0596481	0.0129035	0
LOC100133310	NC_000004	41841904	41845967	0	0	0	0
TMEM33	NC_000004	41937137	41962824	3.6561831	4.1395186	5.1322838	5.2900042
WDR21B	NC_000004	41983713	41988484	0.0184192	0.0568106	0.1065106	0.0385057
SLC30A9	NC_000004	41992523	42089551	5.4581521	6.3043385	7.1457066	11.45733
ATP1BL1	NC_000004	42031102	42033282	0	0	0	0
BEND4	NC_000004	42112957	42154895	0.025076	0.0051562	0.0178467	0.0034948
SHISA3	NC_000004	42399856	42404504	0.0668923	2.5445751	0	0.0155377
ATP8A1	NC_000004	42410390	42659122	0.0210682	0.0324905	0.0281143	0.0587246
RPS7P7	NC_000004	42473792	42474410	0	0	0	0
GRXCR1	NC_000004	42895284	43032675	0	0	0	0
RPS2P21	NC_000004	43412021	43412977	0	0	0	0
PGBD3P4	NC_000004	43588337	43590259	0	0	0	0
LOC402175	NC_000004	43900979	43901420	0	0	0	0
KCTD8	NC_000004	44175920	44450824	0.1016928	0.3310778	0.1055468	0.2125907
LOC100289468	NC_000004	44423724	44450510	0	0.0494348	0	0.0335064
YIPF7	NC_000004	44624354	44653658	0.0469031	0.0482213	0.0417263	0.0653679
GUF1	NC_000004	44680433	44702697	0.8443564	0.7180474	1.0479186	2.4988041
GNPDA2	NC_000004	44704168	44728612	3.9869616	6.9259229	8.1945888	7.5684006
LOC100131309	NC_000004	45325262	45331748	0	0	0	0
GABRG1	NC_000004	46037786	46126082	0.0129851	0.006675	0.0173279	0.0090486
GABRA2	NC_000004	46251575	46392056	0.0997528	0.1172073	0.1774857	0.1092327
RAC1P2	NC_000004	46725687	46726624	0	0	0	0
COX7B2	NC_000004	46736844	46847031	0.1870138	0	0.0831863	0
GABRA4	NC_000004	46920917	46995580	0.0355408	0.0202998	0.0140525	0.0412771
GABRB1	NC_000004	47033295	47428462	0.0197431	0.020298	0	0.0412734
COMMD8	NC_000004	47452815	47465676	5.9034938	7.8998698	5.9466091	9.5553103
ATP10D	NC_000004	47487410	47595503	3.254182	2.4833625	1.0863751	2.7024702
RPL21P52	NC_000004	47492963	47493520	0	0	0	0

CORIN	NC_000004	47596018	47840059	0.0177856	0.0639992	0.0158226	0.0309843
RPL15P7	NC_000004	47708339	47709304	0	0	0	0
NFXL1	NC_000004	47849257	47916633	1.4971635	1.8543617	1.7619075	1.3800911
CNGA1	NC_000004	47937994	48014961	0.0145476	0.0149564	0.0129419	0.0202747
NIPAL1	NC_000004	48018791	48039084	0.0200311	0.0823763	0.0178202	0.0418754
TXK	NC_000004	48068410	48136273	0.1055723	0.0155056	0.0670857	0.1996818
TEC	NC_000004	48137800	48271814	0.1335444	0.0249632	0.2268091	0.3722361
SLAIN2	NC_000004	48343613	48428215	7.0261605	6.9079265	9.3272183	10.77042
SLC10A4	NC_000004	48485449	48491164	0.1064122	0.1094029	0.0236668	0
ZAR1	NC_000004	48492309	48496424	0.0916862	0	0	0.0638906
FRYL	NC_000004	48499380	48782316	2.4365627	3.3117511	3.5704167	6.6712318
OCIAD1	NC_000004	48833015	48863832	16.151914	16.451392	20.718368	21.974005
OCIAD2	NC_000004	48887405	48908815	7.6506739	3.6075753	2.8657902	0.481019
FLJ21511	NC_000004	48988265	49064096	0.0711424	0	0	0.0247874
LOC100132617	NC_000004	49235409	49267929	0	0	0	0
LOC643579	NC_000004	49267988	49273705	0	0	0	0
LOC100133097	NC_000004	49518063	49531357	0	0	0	0
LOC728384	NC_000004	49552046	49559491	0	0	0	0
LOC100288338	NC_000004	49561447	49561674	0	0	0	0
DCUN1D4	NC_000004	52709276	52783003	5.5437629	5.349969	6.4719534	9.4639131
RPL37AP2	NC_000004	52844248	52844538	0	0	0	0
LRRC66	NC_000004	52859864	52886477	0.0526799	0.0135401	0.0351491	0.0458868
SGCB	NC_000004	52886861	52904485	5.1558804	4.5088368	3.0883331	3.4282114
LOC100288475	NC_000004	52917495	52961116	0.4420081	0.0649187	0.0561747	0.0440013
SPATA18	NC_000004	52917593	52963458	1.5330779	0.0735544	0.218219	0.0427324
USP46	NC_000004	53457126	53525502	1.1860651	1.6349797	1.7033915	2.6648055
LOC643783	NC_000004	53525633	53527655	0.435131	0.5592005	0.3871046	0.4169228
KIAA0114	NC_000004	53578621	53580305	0	0	0	0
SNORA26	NC_000004	53579416	53579537	0	0	0	0
LOC100288413	NC_000004	53609684	53617566	0.0160161	0.0329325	0.0997387	0.0223213
RASL11B	NC_000004	53728495	53733002	0.1571986	0.2770571	0.0599349	0.1095422
SCFD2	NC_000004	53739149	54232242	4.8154864	3.4001364	1.5141689	2.5552874
LOC100130982	NC_000004	54241848	54244507	0	0	0	0.1881713
FIP1L1	NC_000004	54243820	54326103	7.9407469	8.1639225	5.2549646	6.556312
LNX1	NC_000004	54326437	54457724	0.5436634	0.2079788	0.2699487	0.3964671
LOC441016	NC_000004	54440648	54442554	0.10693	0	0.0475639	0.0372565
LOC100129728	NC_000004	54774211	54794317	0	0	0	0
RPL21P44	NC_000004	54851653	54853449	0	0	0	0
CHIC2	NC_000004	54875956	54930788	8.242735	5.8236385	5.9080766	3.6749854
MORF4LP4	NC_000004	54953093	54953986	0	0	0	0
GSX2	NC_000004	54966248	54968122	0.0362609	0	0.0322587	0.0252681
RPL22P13	NC_000004	55087290	55087639	0	0	0	0
PDGFRA	NC_000004	55095264	55164412	2.7275447	4.824878	0.0535257	0.1211206
KIT	NC_000004	55524095	55606881	0.8236048	0.4975761	0.0075536	0.0473337
RPL38P3	NC_000004	55842282	55842564	0	0	0	0
KDR	NC_000004	55944426	55991762	0.1016144	0.1268568	0.2582828	0.3945071
LOC100128865	NC_000004	56042272	56086126	0	0	0	0
SRD5A3	NC_000004	56212409	56237866	7.2696323	5.2997073	6.5995515	5.2730054
TMEM165	NC_000004	56262090	56292342	19.748747	16.923656	20.533683	18.593119
CLOCK	NC_000004	56298660	56412997	2.8485667	3.5517379	3.2351028	4.9519283
PDCL2	NC_000004	56422692	56458379	0	0	0	0
NMU	NC_000004	56461398	56502465	1.2387369	2.1595008	3.4976989	4.7288415
LOC644145	NC_000004	56686237	56703430	0	0	0	0
EXOC1	NC_000004	56719816	56771244	3.5703337	4.5536594	4.1695338	5.7881191
LOC644173	NC_000004	56814122	56815581	0	0	0	0
CEP135	NC_000004	56815037	56899527	0.2867874	0.382505	0.2758206	0.6211395
KIAA1211	NC_000004	57036361	57196890	0.0313334	0.1159705	0.1951255	0.1441068
MRPL22P1	NC_000004	57186813	57187500	0	0	0	0
AASDH	NC_000004	57204456	57253638	1.2831601	1.6997686	2.041591	1.9516697
RPL7AP31	NC_000004	57222279	57223155	0	0	0	0
PPAT	NC_000004	57259528	57301845	2.6983698	3.2084316	4.0078658	5.0196677
PAICS	NC_000004	57301915	57327534	21.430851	21.493895	27.798269	44.21853
SRP72	NC_000004	57333762	57369847	17.416018	23.68748	20.809669	20.045231
ARL9	NC_000004	57371375	57390058	0.7359752	0.5404713	1.5900924	1.4286726
GLDCP	NC_000004	57458631	57462431	0	0	0	0
HOPX	NC_000004	57514154	57547872	0.3013328	0.26214	0.7217378	0.581485

RPL17P20	NC_000004	57576187	57576812	0	0	0	0
LOC285453	NC_000004	57627089	57627774	0	0	0	0
SPINK2	NC_000004	57676034	57687893	0.0743625	0	0	0
REST	NC_000004	57774042	57798340	2.8554953	3.6635191	3.2981566	3.9378427
C4orf14	NC_000004	57829516	57843826	5.9384274	4.8325558	5.5927416	5.2838698
LOC100288697	NC_000004	57842980	57843791	0.0715769	0.0735886	0	0.0498777
POLR2B	NC_000004	57845109	57897334	8.1376929	12.947432	9.7118019	14.127645
IGFBP7	NC_000004	57897244	57976539	114.10703	34.314962	27.967087	18.154207
LOC100129983	NC_000004	57938766	57939298	0	0	0	0
RPS26P24	NC_000004	58219165	58219495	0	0	0	0
SRIL	NC_000004	58969212	58969894	0	0	0	0
RPL17P19	NC_000004	62077370	62077918	0	0	0	0
LPHN3	NC_000004	62362839	62938168	0.0215257	0.0368844	0.0127666	0.0899996
RPS12P9	NC_000004	62641145	62641984	0	0	0	0
RPS15AP17	NC_000004	62971338	62971797	0	0	0	0
RPL21P47	NC_000004	63114012	63114488	0	0	0	0
LOC100289193	NC_000004	63157253	63158055	0	0	0	0
LOC100131441	NC_000004	63355164	63355935	0	0	0	0
LOC644534	NC_000004	63376187	63376489	0	0	0	0
LOC644548	NC_000004	63682544	63684512	0	0	0	0
LOC644578	NC_000004	64214366	64216615	0	0	0	0.0646094
SRD5A2L2	NC_000004	65144177	65275178	0	0.0321133	0.0277879	0.010883
LOC391657	NC_000004	65296627	65298955	0	0	0	0
LOC100289159	NC_000004	65473421	65474549	0	0	0	0
RPS6P5	NC_000004	65632824	65633656	0	0	0	0
LOC100133173	NC_000004	65733985	65737783	0	0	0	0
LOC100131356	NC_000004	65900352	65904950	0	0	0	0
LOC644682	NC_000004	66082352	66083112	0	0	0	0
EPHA5	NC_000004	66185281	66535653	0.7270642	0.7302484	0.4278939	0.7872519
RPL6P10	NC_000004	66439158	66440089	0	0	0	0
LOC728048	NC_000004	66959788	66960404	0	0	0	0
RPS23P3	NC_000004	67296810	67297239	0	0	0	0
CENPC1	NC_000004	68337989	68411256	1.1548057	0.7285469	2.7318096	2.4781553
STAP1	NC_000004	68424446	68472616	0.0872566	0	0.0517506	0.0810718
UBA6	NC_000004	68481479	68566889	9.5981248	8.585337	11.486085	10.583563
LOC550112	NC_000004	68566996	68588223	0	0	0	0
GNRHR	NC_000004	68603093	68621804	0.0376076	0.0386646	0.0066914	0.0576542
TMPRSS11D	NC_000004	68686594	68749716	0	0	0	0.0440171
TMPRSS11A	NC_000004	68776019	68829232	0.0362012	0.0558279	0.0161028	0.0126132
LOC644759	NC_000004	68857471	68880961	0	0	0	0
GRINL1B	NC_000004	68904134	68908238	0	0	0	0
TMPRSS11F	NC_000004	68918916	68995587	0	0.0435083	0.1694165	0.1032134
SYT14L	NC_000004	68926328	68929015	0	0	0	0
LOC100130017	NC_000004	69048010	69078188	0	0	0	0
FLJ41562	NC_000004	69054242	69083798	0.0137252	0	0.0244207	0.0095643
TMPRSS11B	NC_000004	69092371	69111412	0	0.0170826	0.0443451	0.0347352
LOC100128725	NC_000004	69170061	69170657	0	0	0	0
YTHDC1	NC_000004	69176105	69215824	0.3516421	0.3904471	0.3503702	0.8037254
MT2P1	NC_000004	69241969	69242383	0	0	0	0
TMPRSS11E	NC_000004	69313167	69363322	0	0	0.0183041	0
TMPRSS11E2	NC_000004	69313167	69363322	0	0	0	0.028675
UGT2B29P	NC_000004	69382636	69383656	0	0	0	0
UGT2B17	NC_000004	69402902	69434245	0	0.0217542	0	0.0442343
LOC100132651	NC_000004	69480319	69481855	0	0	0	0
LOC728807	NC_000004	69481907	69494617	0	0	0	0
UGT2B15	NC_000004	69512315	69536374	0	0	0.018287	0.0143241
LOC728811	NC_000004	69570313	69580803	0	0	0	0
UGT2B10	NC_000004	69681713	69697741	0.0159464	0.0163946	0	0.0111121
LOC100289505	NC_000004	69699859	69776500	0	0	0	0
LOC100174950	NC_000004	69748019	69750165	0	0	0	0
LOC100289535	NC_000004	69780640	69790456	0	0	0	0
UGT2A3	NC_000004	69794177	69817509	0	0	0.0790116	0.0412595
LOC100289568	NC_000004	69838710	69842660	0	0	0	0
LOC642381	NC_000004	69861057	69863429	0	0	0	0
UGT2B27P	NC_000004	69885382	69886389	0	0	0	0
UGT2B26P	NC_000004	69893385	69894272	0	0	0	0

UGT2B7	NC_000004	69962193	69978705	0	0	0	0
LOC100127903	NC_000004	69990932	69993790	0	0	0	0
LOC100289599	NC_000004	70010457	70013360	0	0	0	0
LOC642474	NC_000004	70047378	70116536	0	0	0	0
UGT2B11	NC_000004	70066035	70080449	0	0	0	0.0177845
UGT2B28	NC_000004	70146217	70160768	0	0.02465	0	0
LOC642496	NC_000004	70212328	70223897	0	0	0	0
UGT2B25P	NC_000004	70255030	70255928	0	0	0	0
UGT2B24P	NC_000004	70274253	70275275	0	0	0	0
LOC100131521	NC_000004	70324292	70339744	0	0	0	0
UGT2B4	NC_000004	70345883	70361626	0	0	0	0.0582499
UGT2A1	NC_000004	70454135	70518914	0.0168902	0	0.015026	0
UGT2A2	NC_000004	70455090	70505334	0	0	0	0
SULT1B1	NC_000004	70592686	70626430	0	0	0	0
SULT1D1P	NC_000004	70657490	70679689	0	0	0	0
SULT1E1	NC_000004	70706930	70725870	0.02469	0	0.0219649	0.03441
CSN1S1	NC_000004	70796799	70812288	0	0.092117	0	0.031218
CSN2	NC_000004	70820974	70826726	0.0411886	0	0	0.0861056
STATH	NC_000004	70861648	70868173	0	0	0	0
HTN3	NC_000004	70894176	70902247	0	0	0	0
HTN1	NC_000004	70916159	70924559	0	0	0	0.0570296
CSN1S2A	NC_000004	70933103	70950990	0	0	0	0
CSN1S2B	NC_000004	70999321	71012421	0	0	0	0
C4orf40	NC_000004	71019904	71032326	0.0274848	0	0.0163008	0.0319209
ODAM	NC_000004	71062244	71070293	0	0	0.0303553	0.0475542
C4orf7	NC_000004	71091815	71100969	0	0	0.1450745	0
CSN3	NC_000004	71108333	71117145	0	0	0	0
C4orf35	NC_000004	71200671	71202833	0	0.0292639	0	0.0595043
SMR3A	NC_000004	71226493	71232823	0	0	0	0.1024243
SMR3B	NC_000004	71248795	71255961	0	0	0	0
PROL1	NC_000004	71263599	71275914	0.0421364	0	0	0.0587246
MUC7	NC_000004	71296209	71348714	0.0158429	0.0162882	0.0281886	0.0662398
LOC100129410	NC_000004	71346722	71381822	0	0	0	0.0389629
AMTN	NC_000004	71384298	71398459	0	0	0.0392546	0.0922436
AMBN	NC_000004	71458001	71473005	0.0222298	0	0.0197762	0.0154906
ENAM	NC_000004	71494461	71512536	0.0232366	0.0238897	0.0551252	0.0107948
IGJ	NC_000004	71521258	71532348	0.5926305	1.0261667	0.1387422	0.4781741
UTP3	NC_000004	71554196	71556268	2.014029	2.4193717	1.5465511	1.5216413
RUFY3	NC_000004	71570654	71674336	0.5572071	0.4448865	0.5589887	1.0285399
GRSF1	NC_000004	71681499	71705627	4.6863622	5.3307738	5.4545484	6.0144873
MOBKL1A	NC_000004	71768064	71853891	1.5032594	2.0325388	0.9102911	1.2411923
DCK	NC_000004	71859265	71896631	6.962263	8.6484532	10.138072	9.8793719
SLC4A4	NC_000004	72053003	72437804	0.6090204	0.7051093	0.1952438	0.5467363
GC	NC_000004	72607410	72649888	0.0246762	0	0.0219526	0.0687813
NPFFR2	NC_000004	72897521	73013918	2.8533152	3.4501005	2.5862824	1.0504246
ADAMTS3	NC_000004	73146687	73434516	0.9588431	0.3027234	0.5641978	0.462977
LOC728019	NC_000004	73672984	73673990	0	0	0	0
RNU6ATAC5P	NC_000004	73892465	73892589	0	0	0	0
COX18	NC_000004	73920416	73935472	1.3603255	1.6083413	1.7201892	1.8281488
ANKRD17	NC_000004	73940502	74124502	4.6268698	4.3809826	5.5174485	6.3438415
HMGA1L2	NC_000004	73964458	73966223	0	0	0	0
ALB	NC_000004	74269972	74287129	0.0195586	0.0402166	0.0347998	0.0136292
AFP	NC_000004	74301933	74321492	0.1946526	0.2445952	0.1924093	0.3617112
AFM	NC_000004	74347462	74369718	0.0440143	0	0.0391563	0.0766772
RASSF6	NC_000004	74438862	74486340	0	0.0304402	0.0263402	0.0206321
IL8	NC_000004	74606275	74609433	2.79163	25.03811	8.1837615	1.1857182
CXCL6	NC_000004	74702273	74704477	0.4238527	11.738424	0.2121025	0
PPBPL1	NC_000004	74713583	74714555	0	0	0	0
PF4V1	NC_000004	74719013	74720198	0.0593094	0.1219525	0.211053	0
CXCL1	NC_000004	74735109	74736953	11.47515	20.277229	23.075716	4.1092312
LOC642958	NC_000004	74804321	74808029	0	0	0	0
CXCL1P	NC_000004	74809712	74810210	0	0	0	0
PF4	NC_000004	74846796	74847715	0	0.1898462	0	0
PPBP	NC_000004	74852755	74853900	0	0	0.055774	0.0436874
CXCL5	NC_000004	74861359	74864416	0.0893984	0.3492614	0.6362501	0.0498371
CXCL3	NC_000004	74902306	74904490	2.0097121	8.8885381	22.905304	0.9248238

PPBPL2	NC_000004	74919755	74921116	0	0	0	0
LOC643014	NC_000004	74951712	74960331	0	0	0	0
CXCL2	NC_000004	74962752	74964997	3.245969	4.8370613	14.633197	1.6773792
LOC100287052	NC_000004	75023748	75026406	0.0492693	0	0	0
MTHFD2L	NC_000004	75023829	75168816	0.4281276	0.3253358	1.5731761	0.8431246
EPGN	NC_000004	75174204	75179307	0.2594347	0.5334522	0.1846402	0.0723138
EREG	NC_000004	75230860	75254477	2.6779173	0.2245502	23.485573	2.8057365
AREG	NC_000004	75310853	75320726	0.0709988	0.1824855	0.3789748	0.2226364
AREGB	NC_000004	75480633	75490486	0.3914417	0.0731715	0.4748692	0.1487848
BTC	NC_000004	75671448	75719882	1.9266799	0.2049134	4.9056658	2.0138809
DKFZP564O0823	NC_000004	75858298	75975323	0.0348865	0.0717339	3.8717375	0.2370253
RCHY1	NC_000004	76404347	76439628	1.0480824	0.6276925	1.0500851	1.0281564
THAP6	NC_000004	76439654	76455236	1.9529818	1.5027459	1.442392	2.0798897
C4orf26	NC_000004	76481258	76489927	0.0622496	0	0	0
CDKL2	NC_000004	76501704	76555721	0.316912	0.3545675	0.0082922	0.0064952
G3BP2	NC_000004	76567953	76598667	13.915492	14.951923	16.7696	17.475897
USO1	NC_000004	76649829	76735366	9.5678133	8.7489476	8.4105817	10.979927
PPEF2	NC_000004	76781025	76823681	0.0257459	0.0132347	0.0229043	0.0358815
NAAA	NC_000004	76831808	76862166	0.8748477	0.9170714	0.6867254	0.2988376
LOC100288094	NC_000004	76861123	76862164	0.283537	0.0971686	0	0
SDAD1	NC_000004	76871068	76912113	3.5385753	3.129003	3.2257435	3.7240989
CXCL9	NC_000004	76922623	76928641	0.1554161	0.0177538	0.030725	0
ART3	NC_000004	76932337	77033955	0.0771473	0.0264385	0.0457549	0.1075186
CXCL10	NC_000004	76942271	76944650	1.8327348	0.2691777	4.3256881	0
CXCL11	NC_000004	76954835	76957233	0.0590305	0.2124136	0.262576	0
RPL36P8	NC_000004	76957903	76958217	0	0	0	0
NUP54	NC_000004	77035817	77069655	5.56354	7.658529	7.8228349	8.3002007
SCARB2	NC_000004	77079894	77135035	47.511852	31.403797	56.05247	34.06921
LOC100289107	NC_000004	77082388	77084610	0	0.1195328	0.1034327	0.1620364
FAM47E	NC_000004	77172853	77204932	0.1701222	0.0874517	0.1765697	0.019758
STBD1	NC_000004	77227677	77232283	4.5506934	4.1996802	3.6021402	4.3821167
CCDC158	NC_000004	77234192	77328458	0.2424061	0.1744533	0.3019117	0.1520264
LOC100131940	NC_000004	77265701	77266887	0	0	0	0
RPL36AP18	NC_000004	77322377	77322772	0	0	0	0
SHROOM3	NC_000004	77356253	77704406	0.4825532	0.96763	0.1525586	0.5252361
RPL26P17	NC_000004	77807198	77812600	0	0	0	0
ANKRD56	NC_000004	77816082	77819002	0.0300912	0.0464054	0.013385	0.0524219
SEPT11	NC_000004	77870895	77959768	16.155228	17.520261	13.246016	19.76421
AF357530	NC_000004	77879718	77880517	0	0	0	0
CCNI	NC_000004	77969165	77997125	42.083055	47.987889	42.789537	83.588859
RPL7P17	NC_000004	78003552	78004266	0	0	0	0
LOC339966	NC_000004	78033645	78034596	0	0	0	0
CCNG2	NC_000004	78078357	78091213	0.6422833	0.6355721	1.2142101	0.9902454
CXCL13	NC_000004	78432907	78532988	0	0	0	0
CNOT6L	NC_000004	78634541	78740544	2.1789207	2.389161	4.5037337	4.0118103
MRPL1	NC_000004	78783805	78873944	4.6891049	4.3221797	4.114019	6.557645
LOC100128297	NC_000004	78879154	78881281	0	0	0	0
LOC100130279	NC_000004	78885094	78885687	0	0	0	0
LOC391670	NC_000004	78909014	78909967	0	0	0	0
FRAS1	NC_000004	78978724	79465423	0.0168804	0.1012367	0.022526	0.0254864
LOC442112	NC_000004	79101380	79103299	0	0	0	0
ANXA3	NC_000004	79472742	79531605	0.0814862	0.418882	0.1933131	0.2649866
LOC100287192	NC_000004	79540817	79570396	0	0	0	0
RPS21P3	NC_000004	79689603	79689966	0	0	0	0
BMP2K	NC_000004	79697532	79833341	4.5714234	5.3224076	5.7636365	6.4273568
PAQR3	NC_000004	79839094	79860582	2.8092076	2.863992	5.406109	5.061828
ARD1B	NC_000004	80238272	80247171	0.0289896	0.0298044	0.0773699	0.1414077
GK2	NC_000004	80327507	80329372	0	0	0	0.0328241
OR7E94P	NC_000004	80508908	80509237	0	0	0	0
GDEP	NC_000004	80748625	80784401	0	0	0	0
ANTXR2	NC_000004	80822771	80994477	4.2784326	4.1881623	5.9585917	4.1153588
LOC100287256	NC_000004	80977823	80994393	0.1813545	0.124301	0	0.042125
LOC643513	NC_000004	81000686	81002276	0	0	0	0
RPSAP39	NC_000004	81082207	81083235	0	0	0	0
PRDM8	NC_000004	81106424	81125483	5.3112792	2.0985656	3.3075429	3.7011109
FGF5	NC_000004	81187742	81212171	17.558089	10.195401	3.8306763	12.371564

MRPS25P1	NC_000004	81242694	81243213	0	0	0	0
C4orf22	NC_000004	81256874	81884904	0.4943558	0.3557748	0.1759171	0.103346
LOC100132983	NC_000004	81257024	81312246	0	0	0	0
BMP3	NC_000004	81952119	81978685	0.0076645	0.0157598	0.0204556	0.0320456
PRKG2	NC_000004	82009837	82126215	0.066028	0.1629209	0.1174807	0.0368087
RASGEF1B	NC_000004	82348219	82393061	0.3292728	0.0597401	0.0344624	0.0404912
COX5BL1	NC_000004	82841066	82841566	0	0	0	0
LOC441026	NC_000004	83049681	83052784	0	0	0	0
LOC345258	NC_000004	83196054	83197756	0	0	0	0
HNRNPD	NC_000004	83274467	83295149	5.6858147	6.6664036	6.7212474	11.058607
LOC391672	NC_000004	83322728	83323751	0	0	0	0
HNRPDL	NC_000004	83344347	83351378	9.3030018	12.06178	10.871574	15.57419
ENOPH1	NC_000004	83351726	83382244	4.4952015	4.0382385	5.0861779	5.6870429
FLJ12993	NC_000004	83405604	83483126	0.1163883	0.0717957	0.0207085	0
LOC100287465	NC_000004	83405604	83411957	0	0	0	0
RPL7AP26	NC_000004	83411916	83412808	0	0	0	0
RPS6P6	NC_000004	83415959	83417087	0	0	0	0
LOC100131937	NC_000004	83498640	83498791	0	0	0	0
LOC442113	NC_000004	83501270	83502537	0	0	0	0
C4orf11	NC_000004	83534266	83542590	0	0	0	0
SCD5	NC_000004	83550690	83720010	1.7501508	0.9049307	1.6025085	1.5405155
LOC100127906	NC_000004	83601844	83613925	0	0	0	0
SEC31A	NC_000004	83739814	83812400	57.993009	47.399946	55.53883	56.605465
THAP9	NC_000004	83821837	83841284	0.4897648	0.2573596	0.6971334	0.7735853
LIN54	NC_000004	83845756	83934040	3.7217931	4.29108	6.0502902	5.6151352
COPS4	NC_000004	83956239	83996971	16.669152	15.302388	10.646706	16.205957
PLAC8	NC_000004	84011201	84035911	0.0853778	0.1316661	0.0569659	0.267726
LOC391674	NC_000004	84168332	84169359	0	0	0	0
COQ2	NC_000004	84184977	84205964	2.7431926	3.7897652	5.4146824	5.1572454
LOC100287938	NC_000004	84213614	84214787	0.9358652	0.7697343	2.4644121	2.0607882
HPSE	NC_000004	84216468	84256306	2.3316586	1.3075583	3.5409907	3.2331731
LOC100287427	NC_000004	84298522	84303878	0	0	0	0
HELQ	NC_000004	84328499	84377025	0.8442171	0.7924706	0.6204235	0.8440598
MRPS18C	NC_000004	84377118	84382929	2.7443142	2.2168484	3.5923722	3.0324367
FAM175A	NC_000004	84382092	84406290	2.0379534	2.83096	2.9755669	2.9811828
LOC100288343	NC_000004	84398704	84399257	0	0	0	0
RPL30P5	NC_000004	84423852	84424187	0	0	0	0
AGPAT9	NC_000004	84457653	84527026	0.8324843	2.9064304	1.6663524	1.3898425
LOC152845	NC_000004	85164991	85166737	0	0	0	0
NKX6-1	NC_000004	85414436	85419387	0.0787603	0.0404869	0	0.0274416
CDS1	NC_000004	85504057	85572493	0.1549519	0.338527	0.0603092	0.0539883
WDFY3	NC_000004	85590690	85887544	2.6946118	3.055359	3.7832376	4.9782572
C4orf12	NC_000004	85887971	85928168	0	0	0	0
ARHGAP24	NC_000004	86396284	86923823	2.7225937	2.6559761	2.0711664	2.1828715
MAPK10	NC_000004	86937632	87281216	0.4527734	0.0150161	0.2858579	0.1526664
PTPN13	NC_000004	87515468	87736329	0.9478227	1.601277	1.1987246	1.5387413
SLC10A6	NC_000004	87744841	87770268	0.0387551	0	0	0.0270061
RPL6P13	NC_000004	87791318	87792586	0	0	0	0
C4orf36	NC_000004	87797358	87813575	0.0968023	0.3483302	0.3444719	0.0674557
LOC260422	NC_000004	87834099	87836072	0	0	0	0
LOC728530	NC_000004	87856603	87864398	0.5639951	0	1.1192788	1.4813613
LOC442777	NC_000004	87870690	87871257	0	0	0	0
AFF1	NC_000004	87928153	88062191	4.5644677	3.7676925	16.551219	11.8313
KLHL8	NC_000004	88082214	88141674	0.537828	0.5825657	0.5553631	0.682635
GAPDHL4	NC_000004	88128243	88129441	0	0	0	0
HSD17B13	NC_000004	88224941	88244056	0	0.0377314	0	0.025574
HSD17B11	NC_000004	88257691	88312455	5.8660213	11.338067	4.9472093	4.7417057
LOC100129693	NC_000004	88331761	88332540	0	0	0	0
NUDT9	NC_000004	88343735	88379499	0.4389659	0.3716614	0.4364593	0.539804
SPARCL1	NC_000004	88394487	88450655	0.0584612	0.0450782	0.0130022	0.0101845
DSPP	NC_000004	88529681	88538025	0.0405895	0.0208651	0.0090274	0.0494976
DMP1	NC_000004	88571454	88585513	0	0	0.0145506	0
LOC100128026	NC_000004	88707048	88707523	0	0	0	0
IBSP	NC_000004	88720702	88733601	0.027623	0	0	0
MEPE	NC_000004	88754140	88767944	0	0	0.0398345	0.0156011
HSP90AB3P	NC_000004	88812811	88815556	0	0	0	0

SPP1	NC_000004	88896802	88904563	9.033052	4.5322428	0.2646604	0.2073069
PKD2	NC_000004	88928820	88998929	6.3975274	4.5219148	3.3870122	5.802736
ABCG2	NC_000004	89011416	89080011	1.1505292	0.9381343	0.2647093	1.0367257
RPL31P24	NC_000004	89084691	89085162	0	0	0	0
LOC100287631	NC_000004	89141721	89144973	0	0	0	0
PPM1K	NC_000004	89181531	89205888	3.7164277	1.7345574	4.0514524	1.1263378
HERC6	NC_000004	89299891	89364249	21.572943	11.525311	11.792606	1.1103389
HERC5	NC_000004	89378268	89427314	3.4903378	1.2090065	3.8952883	0.8281706
LOC728333	NC_000004	89428154	89431638	0	0	0	0
PIGY	NC_000004	89442135	89444955	17.572796	17.298597	23.724391	23.857074
LOC100129137	NC_000004	89448311	89449321	0	0	0	0
HERC3	NC_000004	89513647	89629686	3.3854686	7.0720241	2.1569969	3.1100459
NAP1L5	NC_000004	89617066	89619023	0	0	0	0.0312818
FAM13AOS	NC_000004	89630940	89651254	0	0	0	0
FAM13A	NC_000004	89647106	89978323	0.5946696	0.5598204	1.134556	1.1433155
LOC731282	NC_000004	90031864	90033519	0.3103141	0.5981916	0.8627	0.810897
TIGD2	NC_000004	90033968	90036052	1.0539144	1.6253022	1.7064165	1.5128837
GPRIN3	NC_000004	90165429	90229161	0.0484316	0.0426795	0.0369309	0.0192852
SNCA	NC_000004	90646705	90758133	0.2848233	0.0585657	0.7601601	1.1313144
LOC644248	NC_000004	90757063	90761746	0	0	0	0
MMRN1	NC_000004	90816052	90875780	0.0088445	0.0181861	0.0157366	0.0308159
KIAA1680	NC_000004	91048684	92523370	0.0108769	0.0559131	0.0193528	0.0303179
TMSL3	NC_000004	91759636	91760269	0.4159139	0.356336	0.1850043	0.1449127
LOC133083	NC_000004	93103623	93105202	0	0	0	0
GRID2	NC_000004	93225550	94693649	0.0401721	0.0413011	0.0357382	0.1399675
ATOH1	NC_000004	94750078	94751142	0	0	0	0
LOC644429	NC_000004	95117010	95118806	0	0	0	0
SMARCAD1	NC_000004	95128759	95212443	3.883719	3.8286624	3.6868974	5.7582738
PGDS	NC_000004	95219707	95264027	0	0.0559547	0	0.0189628
RPL35AP11	NC_000004	95290922	95291351	0	0	0	0
PDLIM5	NC_000004	95373038	95589377	9.1147037	3.2684823	8.4423625	9.5832388
BMPR1B	NC_000004	95679128	96079601	0.7667227	1.3164948	0.773513	1.1236466
UNC5C	NC_000004	96089689	96470162	0.1808073	0.0495704	0.0321702	0.0755962
RPL30P6	NC_000004	96566103	96566441	0	0	0	0
PDHA2	NC_000004	96761239	96762625	0	0.0325764	0.0563772	0.0220799
COX7AP2	NC_000004	97823918	97824340	0	0	0	0
C4orf37	NC_000004	98480029	99064391	0	0.1095355	0.0236955	0.0928027
RPL5P12	NC_000004	98946491	98947475	0	0	0	0
RAP1GDS1	NC_000004	99182527	99365012	7.9109154	9.1424007	9.1896432	11.269564
RPL21P48	NC_000004	99330401	99330947	0	0	0	0
TSPAN5	NC_000004	99393398	99579727	18.414064	22.023753	17.25535	25.534989
LOC100287756	NC_000004	99634955	99635122	0	0	0	0
BTF3L3	NC_000004	99661776	99662646	0	0	0	0
EIF4E	NC_000004	99799607	99851786	4.0634675	4.6079898	4.7165317	5.748241
TBCAP3	NC_000004	99830533	99831054	0	0	0	0
LOC100287782	NC_000004	99877151	99878014	0	0	0	0
LOC100287817	NC_000004	99897935	99898268	0	0	0	0
METAP1	NC_000004	99916788	99983960	5.679952	8.2700306	7.664252	8.103984
ADH5	NC_000004	99992129	100009931	41.155757	35.955324	42.306405	51.56655
ADH4	NC_000004	100044832	100065449	0.1109804	0.1140995	0	0.0154671
PCNAP1	NC_000004	100081399	100083146	0	0	0	0
PCNAP2	NC_000004	100083151	100084122	0	0	0	0
ADH6	NC_000004	100123795	100140403	0.0438605	0.0450932	0.026013	0
ADH1A	NC_000004	100197524	100212142	0	0.0310326	0.0268527	0
ADH1B	NC_000004	100227527	100242572	0.0163864	0	0.0291555	0
ADH1C	NC_000004	100257649	100273917	0.0298359	0	0	0.0207908
ADH7	NC_000004	100333418	100356525	0	0.0210449	0	0.0427921
LOC100131750	NC_000004	100390703	100431185	0	0	0	0
C4orf17	NC_000004	100432200	100463460	0	0	0	0.0575295
RG9MTD2	NC_000004	100467864	100485189	0.5829284	0.9753504	0.7117892	1.0513612
MTTP	NC_000004	100485240	100545154	0.0322359	0.0441891	0.1051524	0.0374387
LOC285556	NC_000004	100557686	100576743	0.01726	0.0088725	0.015355	0.021048
DAPP1	NC_000004	100737981	100791347	0.0299171	0.0461369	0.0798453	0
MAPKSP1	NC_000004	100799495	100815703	4.1894576	3.5787788	3.5900335	4.5651104
DNAJB14	NC_000004	100820710	100867879	2.9870816	2.7279928	2.5725802	2.4690339
H2AFZ	NC_000004	100869244	100871512	36.755015	46.614766	69.318704	71.501168

LOC100131076	NC_000004	100871636	100873620	0.0221402	0	0.039393	0.1234252
LOC256880	NC_000004	100871636	100873620	0	0	0	0
LOC644721	NC_000004	100911885	100912638	0	0	0	0
LOC100288885	NC_000004	100962998	100963261	0	0	0	0
DDIT4L	NC_000004	101107027	101111613	1.1190522	1.0459121	1.6290652	0.5198668
EMCN	NC_000004	101318981	101439179	0.1185387	0.8226243	0	0.0413013
PPP3CA	NC_000004	101944587	102268628	3.0075779	2.8312097	7.1489347	7.9574908
FLJ20021	NC_000004	102268382	102270040	0.4650607	0.4781312	1.1032823	0.6211395
BANK1	NC_000004	102711764	102995969	1.2228429	0.0888937	0.7801931	0.3615078
SLC39A8	NC_000004	103172198	103266655	2.9488599	3.1668154	5.8636608	3.6977722
LOC100287975	NC_000004	103330369	103373414	0	0	0	0.0305638
LOC100288009	NC_000004	103421998	103423048	0.199992	0.5140319	0.1334388	0.2438841
NFKB1	NC_000004	103422486	103538459	13.017714	20.927049	8.3937736	8.6964159
MANBA	NC_000004	103552643	103682151	1.685722	1.0780697	0.5904193	0.6659593
RPL21P49	NC_000004	103583738	103584273	0	0	0	0
LOC100288914	NC_000004	103655515	103655772	0	0	0	0
UBE2D3	NC_000004	103717133	103790032	32.206338	34.86259	36.549222	36.514278
LOC100288943	NC_000004	103747345	103748708	0.047358	0	0	0.0660019
CISD2	NC_000004	103790135	103809912	6.018997	5.4269195	8.3294814	7.9225229
NHEDC1	NC_000004	103806208	103940876	0.3084808	0.2114338	0.4025018	0.8455161
LOC100128183	NC_000004	103816564	103819398	0	0	0	0
LOC777774	NC_000004	103883293	103884925	0	0	0	0
NHEDC2	NC_000004	103946652	103998170	0.8760879	1.0620318	1.3261299	2.6424322
BDH2	NC_000004	103998782	104021024	4.5001785	3.0947536	3.4143422	3.5239586
CENPE	NC_000004	104026963	104119566	0.9951121	2.0671459	1.2121517	3.6520843
LOC650560	NC_000004	104493246	104495255	0	0	0	0
TACR3	NC_000004	104510625	104640973	0	0.0515498	0.0223032	0.01747
CXXC4	NC_000004	105393330	105412467	0	0	0.0513766	0.1609718
LOC728847	NC_000004	105412934	105471213	0	0.0760663	0.1974625	0.1031141
RPL6P14	NC_000004	105807267	105808112	0	0	0	0
LOC100288146	NC_000004	106024022	106025157	0	0	0	0
TET2	NC_000004	106067943	106200958	0.4598076	0.4907966	0.7347403	1.151035
PPA2	NC_000004	106290234	106395227	7.7493893	9.6416464	11.895195	11.093052
EEF1AL7	NC_000004	106405863	106407507	0	0	0	0
LOC402182	NC_000004	106447523	106448554	0	0	0	0
ATP5EP1	NC_000004	106453545	106453963	0	0	0	0
FLJ20184	NC_000004	106473777	106552837	0.0305833	0.0314429	0	0.0426234
LOC100288245	NC_000004	106586640	106604478	0.3099311	0	0.4411573	0.9502783
LOC644892	NC_000004	106600207	106601900	0	0	0	0
INTS12	NC_000004	106603784	106629881	2.2312565	2.087767	4.1930076	4.2277356
GSTCD	NC_000004	106629941	106768882	0.7770411	1.2035074	1.1401586	1.5189377
NPNT	NC_000004	106816605	106892828	0.0766985	0.0197135	0.0426457	0.0267233
TBCKL	NC_000004	106967237	107237385	4.1691072	2.2113931	1.6418615	3.9877107
SCYE1	NC_000004	107236767	107270382	5.0538445	3.4639223	5.035565	4.8271044
LOC100288276	NC_000004	107279340	107423428	0	0.0274838	0	0.0372565
DKK2	NC_000004	107842959	107957453	0.1208365	0.0869628	0	0.0505222
RAC1P5	NC_000004	108123810	108125914	0	0	0	0
LOC100288317	NC_000004	108228811	108238849	0	0	0	0
PAPSS1	NC_000004	108534822	108641419	8.2121991	7.038799	6.7982392	4.6503552
SGMS2	NC_000004	108745721	108836203	5.2332119	3.6259991	4.2910505	6.1792784
CYP2U1	NC_000004	108852717	108874613	0.860098	0.7796799	1.0449055	1.4307077
HADH	NC_000004	108910940	108956331	7.2404252	7.2312348	16.27702	12.685619
LEF1	NC_000004	108968701	109089578	0.1575719	0.1914551	0.5352339	0.4591735
RPSAP34	NC_000004	109328995	109329717	0	0	0	0
LOC391681	NC_000004	109338861	109340919	0	0	0	0
LOC285456	NC_000004	109459346	109541613	0	0	0	0
RPL34	NC_000004	109541722	109551640	49.014186	53.062675	88.094712	80.016923
OSTC	NC_000004	109571741	109588978	22.096421	34.369564	43.014576	25.937397
AGXT2L1	NC_000004	109663196	109684209	0	0.0645785	0	0.0145902
LOC100129714	NC_000004	109709901	109710964	0	0	0	0
COL25A1	NC_000004	109734972	110223799	0.0756424	0.093322	0.0269174	0.0210843
RBMXP4	NC_000004	110267437	110278316	0	0	0	0
SEC24B	NC_000004	110354971	110461615	3.736451	4.9112393	6.4882099	5.1217242
LOC389217	NC_000004	110474399	110476215	0.0986492	0	0	0.0343714
LOC100131440	NC_000004	110476326	110476888	0	0	0	0
CCDC109B	NC_000004	110481355	110608872	12.246171	10.852528	20.131871	16.322051

CASP6	NC_000004	110609785	110624629	2.7517255	3.2915061	3.3189384	3.5031468
PLA2G12A	NC_000004	110631145	110651242	1.2844469	0.9753055	1.4264823	1.9714583
CFI	NC_000004	110661848	110723335	3.2742551	0.5854397	3.5822851	1.0061853
GAR1	NC_000004	110736666	110745893	1.6649685	2.1397033	2.0057946	1.7161534
RRH	NC_000004	110749150	110765863	0.0564162	0.0580018	0	0.1179392
LRIT3	NC_000004	110772486	110793471	0.012127	0	0.021577	0.0084506
KRT19P3	NC_000004	110800226	110801059	0	0	0	0
EGF	NC_000004	110834040	110933422	0.1528791	0.1571757	0.200008	0.1315986
ELOVL6	NC_000004	110970229	111119820	5.0011844	4.1426999	8.1722211	11.556558
LOC100288483	NC_000004	111173004	111173270	0	0.1692262	0	0
ZBED1P	NC_000004	111198525	111214716	0	0	0	0
LOC100133103	NC_000004	111320920	111321672	0	0	0	0
LOC100130995	NC_000004	111337054	111338020	0	0	0	0
ENPEP	NC_000004	111397229	111484493	0.1847673	0.0271372	0.0547914	0.0306555
PITX2	NC_000004	111538579	111558508	3.6659187	2.5957358	0.9771219	0.5566352
LOC729065	NC_000004	111714723	111718500	0.0692098	0.071155	0	0.0964563
LOC391686	NC_000004	111866265	111866955	0	0	0	0
LOC100288547	NC_000004	111962271	111985353	0	0	0	0
RPL36AP23	NC_000004	112472993	112473384	0	0	0	0
LOC100288584	NC_000004	112980714	112989696	0	0	0	0
LOC132719	NC_000004	112998641	112999556	0	0	0	0
RPS12P8	NC_000004	113037646	113038140	0	0	0	0
C4orf32	NC_000004	113066553	113110237	1.0801656	0.6298493	2.0653153	0.9998585
RPL36AP19	NC_000004	113092336	113092734	0	0	0	0
C4orf16	NC_000004	113152895	113191211	2.8563328	2.3927936	5.8350562	4.4757817
TIFA	NC_000004	113196782	113207059	1.4444424	1.2791917	2.7227071	2.2622347
ALPK1	NC_000004	113218499	113363774	0.9969813	0.4250006	1.5359243	1.2369694
LOC285412	NC_000004	113377080	113378945	0	0	0	0
NEUROG2	NC_000004	113434672	113437328	0.461197	0.7507518	0.0854778	0.093736
LOC91431	NC_000004	113460500	113508807	0.8470588	1.0160097	1.4443367	1.6232268
LOC728914	NC_000004	113486144	113486542	0	0	0	0
C4orf21	NC_000004	113510786	113558056	1.2906583	1.7331362	2.2378289	2.367761
LARP7	NC_000004	113558120	113578742	0.3069515	0.297015	0.3051987	0.7045987
LOC645264	NC_000004	113603958	113606659	0	0	0	0
LOC256085	NC_000004	113614594	113614947	0	0	0	0
RPL32P13	NC_000004	113659831	113660262	0	0	0	0
LOC100289140	NC_000004	113679882	113680092	0	0	0	0
RPL7AP30	NC_000004	113709180	113710056	0	0	0	0
LOC100131158	NC_000004	113718200	113719855	0	0	0	0
ANK2	NC_000004	113739239	114304896	0.7536058	0.3920604	0.5331119	0.5293605
RPS26P25	NC_000004	114135094	114135583	0	0	0	0
CAMK2D	NC_000004	114372188	114683083	13.356109	6.361562	9.6043741	13.252019
ARSJ	NC_000004	114821440	114900878	9.1128228	4.2691457	5.1901642	8.628386
UGT8	NC_000004	115519611	115598202	0.0257158	0.0925348	1.0180466	0.1702378
LOC100131828	NC_000004	115634732	115636075	0	0	0	0
NDST4	NC_000004	115748931	116035032	0.0263242	0.027064	0.0234187	0.0183437
MRPS33P3	NC_000004	116064138	116064459	0	0	0	0
LOC100128462	NC_000004	116549965	116550807	0	0	0	0
LOC100131611	NC_000004	116685255	116706518	0	0	0	0
KRT18P21	NC_000004	116841813	116843189	0	0	0	0
LOC645368	NC_000004	116964563	116965201	0	0	0	0
RLFP	NC_000004	117016288	117018293	0	0	0	0
LOC100288825	NC_000004	117115244	117158121	0	0	0	0
LOC100288861	NC_000004	117300075	117300476	0	0	0	0
LOC100129008	NC_000004	117347795	117415608	0	0	0	0
LOC344978	NC_000004	117465210	117520767	0	0	0	0
TRAM1L1	NC_000004	118004710	118006736	0.2818584	0.5349786	0.3086142	0.1057593
RPSAP35	NC_000004	118334618	118335437	0	0	0	0
NT5C3P1	NC_000004	118495739	118497341	0	0	0	0
LOC100288955	NC_000004	118954275	118975494	0.0318696	0.0655307	0.0850563	0.0666241
NDST3	NC_000004	118955500	119179789	0.01478	0	0.019723	0.0411971
LOC100132656	NC_000004	119113882	119116050	0	0	0	0
SNHG8	NC_000004	119199914	119200974	0	0	0	0
SNORA24	NC_000004	119200345	119200475	0	0	0	0
PRSS12	NC_000004	119202417	119273922	4.8423104	3.9530417	2.4166008	6.3188387
LOC100128177	NC_000004	119321669	119321989	0	0	0	0

LOC100288991	NC_000004	119375708	119381545	0	0	0	0
CEP170L	NC_000004	119437495	119475359	0	0	0	0
LOC729218	NC_000004	119551594	119554281	0.2942962	0.3529953	0.8145326	0.432941
LOC100289025	NC_000004	119554367	119556254	0	0	0	0
LOC100132769	NC_000004	119557056	119557462	0	0	0	0
LOC729227	NC_000004	119557485	119562441	0	0	0	0
METTL14	NC_000004	119606574	119632090	2.6327478	2.4296735	2.9507597	3.0769335
SEC24D	NC_000004	119643978	119757326	12.504099	12.332856	12.451944	17.999559
SYNPO2	NC_000004	119809996	119982402	0.2519163	0.5474988	0.1049637	0.1866558
MYOZ2	NC_000004	120056939	120108937	0	0.0697813	0	0.0472971
LOC100289084	NC_000004	120114851	120133814	0	0	0	0
MRPL42P1	NC_000004	120116421	120116846	0	0	0	0
USP53	NC_000004	120133782	120216673	3.1120279	4.830068	4.7071216	3.8820922
C4orf3	NC_000004	120217574	120221968	3.8236045	1.9734913	2.6579183	3.2469491
FABP2	NC_000004	120238405	120243316	0	0	0.0173844	0.0817027
GK7P	NC_000004	120313701	120316225	0	0	0	0
GK6P	NC_000004	120314018	120316225	0	0	0	0
LOC100128874	NC_000004	120324234	120325181	0	0	0	0
LOC100131884	NC_000004	120325496	120325901	0	0	0	0
LOC100128460	NC_000004	120326802	120329031	0	0	0	0
FLJ14186	NC_000004	120328576	120331294	0	0	0	0
LOC645513	NC_000004	120365410	120383006	0	0	0	0
PDE5A	NC_000004	120415550	120549981	2.0199048	0.4216278	0.2940485	1.4416725
LOC730456	NC_000004	120548945	120551908	0.133446	0.0914644	0.0131908	0.0206646
MAD2L1	NC_000004	120980577	120988013	8.7407471	16.558868	19.657538	19.869966
LOC100289220	NC_000004	121012186	121013510	0	0	0	0
SAR1P3	NC_000004	121263135	121266015	0	0	0	0
PRDM5	NC_000004	121615929	121844013	1.7721061	1.767254	1.5134542	1.1360842
LOC100129988	NC_000004	121843097	121844760	0.0264112	0.054307	0.0469923	0.1840437
C4orf31	NC_000004	121956782	121993673	0.168384	0.3304951	0.2859801	0.1280037
TNIP3	NC_000004	122052563	122137655	0.1856492	0.0867577	0.6456203	0.0823249
QRFPR	NC_000004	122249797	122302181	0.131077	0.2502702	1.8490966	0.8351053
ANXA5	NC_000004	122589152	122618147	321.57243	251.29205	147.22104	244.02225
TMEM155	NC_000004	122680085	122686340	0.9298201	0.5072403	0.3207486	0.370249
LOC100192379	NC_000004	122685740	122687963	0	0	0	0
EXOSC9	NC_000004	122722472	122738176	1.8712751	1.9788351	2.4971073	4.3962721
CCNA2	NC_000004	122737599	122745088	9.7069361	17.214262	14.406573	18.289554
BBS7	NC_000004	122745635	122791642	1.1307069	1.1734524	1.3760066	2.2448333
TRPC3	NC_000004	122800183	122872909	0.437175	0.5118871	0.3781196	0.3554144
KIAA1109	NC_000004	123091758	123283907	3.1815042	2.9806882	5.6858228	5.9428159
ADAD1	NC_000004	123300168	123350939	0.0224111	0.046082	0.0398751	0.0468509
IL2	NC_000004	123372625	123377650	0.055004	0	0	0
IL21	NC_000004	123533783	123542211	0	0.0733497	0	0.0497157
LOC729338	NC_000004	123651344	123653613	0	0	0	0
BBS12	NC_000004	123653857	123666098	0.7044723	0.5292754	0.8195544	0.8118803
LOC727709	NC_000004	123660428	123661974	0	0	0	0
RPL34P12	NC_000004	123736937	123737272	0	0	0	0
FGF2	NC_000004	123747863	123819390	15.356579	21.744596	16.882253	6.4152203
RPS26P23	NC_000004	123761504	123761845	0	0	0	0
NUDT6	NC_000004	123813799	123844123	3.2920024	2.1322504	3.8658267	3.6703974
SPATA5	NC_000004	123844225	124240605	1.3064086	1.7038821	2.415683	2.937972
COILP2	NC_000004	123944897	123947445	0	0	0	0
SPRY1	NC_000004	124317956	124324910	0.335739	0.1898462	0.5077606	0.7486601
LOC285419	NC_000004	124573940	124851518	0	0	0	0
RPL21P50	NC_000004	124666868	124667422	0	0	0	0
LOC100289258	NC_000004	124983617	124984142	0	0.111564	0.0965372	0.151234
LOC645773	NC_000004	125356902	125358275	0	0	0	0
LOC391696	NC_000004	125381213	125385004	0	0	0	0
ANKRD50	NC_000004	125585466	125631932	1.9021778	2.8461527	1.6066113	2.2683634
FAT4	NC_000004	126237567	126414087	0.6465382	0.9255445	0.946496	2.6860927
LOC100289295	NC_000004	126602324	126605217	0	0	0	0
LOC645841	NC_000004	126644422	126649942	0	0	0	0
LOC132817	NC_000004	127479776	127485931	0	0	0	0
LOC100129299	NC_000004	128419483	128419976	0	0	0	0
LOC729424	NC_000004	128543142	128544912	0.1024434	0	0.0455683	0.0713867
INTU	NC_000004	128554120	128637930	0.6514871	0.2372198	1.0263413	0.9079643

SLC25A31	NC_000004	128651555	128695447	0	0.0249357	0	0.0169011
HSPA4L	NC_000004	128703453	128754522	2.0318993	1.7154751	3.8307474	5.4948496
RPL21P53	NC_000004	128733829	128734395	0	0	0	0
PLK4	NC_000004	128802045	128820353	3.6402729	5.8744344	5.2574184	5.8119035
MFSD8	NC_000004	128838960	128887139	1.2570451	1.2426678	1.2645417	0.7951014
C4orf29	NC_000004	128886461	128952468	0.3396	0.4107582	0.7464081	0.626418
LARP2	NC_000004	128982503	129132289	0.4233934	0.4352929	0.8081122	0.901205
RPL15P8	NC_000004	128990175	128990767	0	0	0	0
PGRMC2	NC_000004	129191295	129208948	21.796044	18.40534	13.792928	20.184955
PHF17	NC_000004	129730779	129796379	0.8114677	0.7403492	0.7936165	1.5615767
SCLT1	NC_000004	129805152	130014764	1.4251146	1.2579722	1.4471094	2.0664019
C4orf33	NC_000004	130014829	130033843	6.5786705	3.4863736	5.7520081	3.9541381
LOC100127912	NC_000004	130054307	130055225	0	0	0	0
LOC391697	NC_000004	130057081	130063810	0	0	0	0
LOC100128983	NC_000004	130817009	130820068	0	0	0	0
LOC391698	NC_000004	130821615	130825318	0	0	0	0
CYCSP14	NC_000004	130943742	130944038	0	0	0	0
LOC100132483	NC_000004	132644262	132646176	0.0229495	0.0471889	0.0204165	0.1759131
LOC100132213	NC_000004	132646767	132648682	0	0.0235822	0	0.0319675
LOC100131968	NC_000004	132649244	132651166	0.205686	0.1644742	0.0203315	0
LOC100133083	NC_000004	132651748	132653660	0.0229735	0.0236191	0	0.0480265
LOC100132829	NC_000004	132654233	132656148	0.0229375	0.0471643	0	0.0319675
LOC100128869	NC_000004	132656720	132658636	0	0	0	0
LOC100132315	NC_000004	132659214	132661131	0	0.0235576	0	0.0798354
LOC100131850	NC_000004	132661701	132663614	0.0229615	0.0472136	0.0204272	0
LOC100131722	NC_000004	132664195	132666111	0	0.0471397	0	0.0159754
LOC100131591	NC_000004	132666667	132668584	0.0229136	0	0.0203845	0
LOC100129002	NC_000004	132669136	132671052	0.0229255	0.0235698	0.0407904	0
LOC100132410	NC_000004	132671629	132673547	0	0	0.0203739	0
LOC100132117	NC_000004	132674103	132676019	0	0.0471397	0	0
LOC100133270	NC_000004	132676590	132678502	0	0	0	0
LOC100133002	NC_000004	132679058	132680974	0	0	0	0.0159754
LOC100132752	NC_000004	132681545	132683457	0	0	0	0
LOC100128747	NC_000004	132684023	132685933	0	0.0472877	0	0
RPL7AP28	NC_000004	132778495	132779062	0	0	0	0
PCDH10	NC_000004	134070470	134112732	0.9701098	1.0901539	0.1003531	0.1257695
PABPC4L	NC_000004	135117488	135122903	0.9600342	1.4713851	0.9331539	1.0530399
LOC345016	NC_000004	135243340	135249953	0	0	0	0
LOC646272	NC_000004	135865430	135865678	0	0	0	0
RPS2P27	NC_000004	135873291	135874224	0	0	0	0
LOC389223	NC_000004	135966619	135968005	0	0	0	0
LOC100132574	NC_000004	136486272	136488082	0	0	0	0
LOC100289626	NC_000004	137018954	137020903	0	0	0.0259957	0
LOC646316	NC_000004	137274667	137277492	0	0	0	0
LOC100131921	NC_000004	138222155	138222341	0	0	0	0
LOC100289663	NC_000004	138346580	138347096	0	0	0	0
RPS23P2	NC_000004	138369207	138369704	0	0	0	0
PCDH18	NC_000004	138440072	138453629	12.918072	10.611138	8.6390659	6.8239654
SLC7A11	NC_000004	139085248	139163503	3.9037764	1.9341568	3.728209	2.7901397
LOC100131429	NC_000004	139402407	139403134	0	0	0	0
LOC152594	NC_000004	139481842	139483541	0	0	0	0
CCRN4L	NC_000004	139936943	139967093	6.4100543	4.5322143	3.9817889	3.5734249
ELF2	NC_000004	139978871	140060606	4.9722896	4.7870658	6.0890538	6.6841021
LOC100129986	NC_000004	140098819	140100951	0	0.0926839	0	0.0314101
C4orf49	NC_000004	140187317	140201492	2.4944478	0.4049297	1.109565	1.1435728
NDUFC1	NC_000004	140211091	140216957	18.133979	12.06353	18.410019	27.577257
NARG1	NC_000004	140222676	140311935	2.5242142	3.5144454	4.0899686	6.5341161
LOC729350	NC_000004	140334995	140375447	1.2148292	0.8081584	0.7628793	0.7469483
RAB33B	NC_000004	140374961	140397070	1.9878368	1.6699991	2.5667567	1.6780755
SETD7	NC_000004	140427192	140477577	4.1804721	4.4203956	5.3639273	10.298554
MGST2	NC_000004	140586922	140625407	0.932734	0.5076787	1.0738408	0.5352663
MAML3	NC_000004	140637545	141075233	0.2666912	0.2104223	0.3972657	0.4062572
SCOC	NC_000004	141178440	141303704	12.479682	13.638095	20.207731	21.209058
RPL14P3	NC_000004	141287356	141288046	0	0	0	0
CLGN	NC_000004	141309607	141348815	7.2260735	6.6125957	9.4349547	5.7082516
LOC646484	NC_000004	141418880	141422484	0	0	0	0

ELMOD2	NC_000004	141445352	141471847	3.197472	3.6020824	3.4800465	3.6266303
UCP1	NC_000004	141481050	141489959	0.0439482	0	0	0.1224995
LOC100287548	NC_000004	141540937	141543974	0.3515859	0.0602445	0.1042602	0.1224995
TBC1D9	NC_000004	141541936	141677471	7.6504851	8.31849	10.013139	9.5011926
LOC644962	NC_000004	141556581	141563708	0	0	0	0
RNF150	NC_000004	141786725	142054616	0.4243017	0.407145	0.0503294	0.1576909
ZNF330	NC_000004	142142049	142155851	3.9276844	2.7239068	4.9207936	4.0001824
LOC100286983	NC_000004	142557752	142577450	0	0.1206499	0	0
IL15	NC_000004	142557754	142654612	0.9264152	0.5343024	1.3669072	0.6140721
INPP4B	NC_000004	142949181	143767604	0.8205659	2.0816795	0.4266223	1.7302611
RPL5P13	NC_000004	143208070	143208778	0	0	0	0
LOC100130178	NC_000004	143238281	143239198	0	0	0	0
FLJ44477	NC_000004	143766690	143769122	0	0	0	0
LOC100287014	NC_000004	143966249	143967031	0	0	0	0
USP38	NC_000004	144106070	144143141	2.6901901	3.1397898	3.0103997	3.2599726
GAB1	NC_000004	144257983	144395718	0.8667414	0.8111306	1.0379885	1.3124948
RPS2P20	NC_000004	144270382	144271293	0	0	0	0
RPSAP36	NC_000004	144346129	144347136	0	0	0	0
LOC100128055	NC_000004	144434207	144435788	0.0644875	0	0	0.0224687
SMARCA5	NC_000004	144434616	144474567	2.2824322	1.419681	1.1675461	3.5388395
LOC100129551	NC_000004	144476210	144482613	0.3113216	0.1600357	0.2769603	0.4579872
LOC441046	NC_000004	144480625	144482613	0	0	0	0
FREM3	NC_000004	144498455	144621828	0	0.0134295	0.0116206	0.0591653
GYPE	NC_000004	144792019	144826716	0.2099438	0.3597405	0.0622573	0.0487657
GYPB	NC_000004	144917257	144940496	0	0	0	0.1162234
GYPA	NC_000004	145030456	145061904	0	0	0	0.0230262
KRT18P51	NC_000004	145493390	145494144	0	0	0	0
LOC646576	NC_000004	145564068	145567590	0	0	0	0
HHIP	NC_000004	145567173	145659881	0.6349461	1.4847803	0.0775306	0.3209973
HSPD1P5	NC_000004	145766717	145768812	0	0	0	0
ANAPC10	NC_000004	145916310	146019368	3.2797188	2.9568931	5.2967999	3.6918625
ABCE1	NC_000004	146019156	146050676	9.430951	13.057292	11.270614	16.765551
OTUD4	NC_000004	146054802	146100832	1.170534	1.0757953	3.9444679	3.1844271
LOC100287121	NC_000004	146101313	146117369	0.6658823	0.2818929	0.4530021	0.3821286
LOC100131639	NC_000004	146117881	146125960	0	0	0	0
RPS23P4	NC_000004	146190962	146191283	0	0	0	0
LOC152905	NC_000004	146296532	146299129	0	0	0	0
LOC100132841	NC_000004	146377157	146377996	0	0	0	0
SMAD1	NC_000004	146402951	146480328	3.5496649	3.7073561	3.4085057	4.6918884
MMAA	NC_000004	146540540	146581187	0.86521	0.919938	1.282858	1.3243469
LOC729497	NC_000004	146543749	146547284	0	0	0	0
LOC646603	NC_000004	146601356	146653949	0.0519483	0	0.1386438	0
ZNF827	NC_000004	146681874	146859607	1.3612748	1.5933151	1.7979105	2.4152573
LSM6	NC_000004	147096835	147111213	2.5687658	3.3299076	4.1233773	6.1483237
LOC345051	NC_000004	147138105	147152030	0	0	0	0
SLC10A7	NC_000004	147175137	147443123	0.8160149	1.0141143	1.0290932	1.0685277
POU4F2	NC_000004	147560045	147563623	0.0279836	0.0719252	0.0622374	0.0390002
TTC29	NC_000004	147628179	147867034	0.344499	0.3035839	0.1094557	0.0857359
LOC100287219	NC_000004	147957190	148017266	0	0.0745601	0	0
RPL31P26	NC_000004	148347082	148347515	0	0	0	0
EDNRA	NC_000004	148401907	148466106	0.529988	0.9221102	0.2901489	0.34801
GTF2F2L	NC_000004	148427241	148428362	0	0	0	0
TMEM184C	NC_000004	148538539	148556674	4.1215317	4.1006785	3.035811	5.229391
LOC90826	NC_000004	148559533	148605280	0.5435294	0.957952	0.980893	1.0713297
ARHGAP10	NC_000004	148653453	148993927	4.2711469	4.7903869	2.4563916	5.4415278
NR3C2	NC_000004	148999915	149363643	0.112323	0.3772341	0.4130259	0.6366051
ASSP8	NC_000004	149447412	149448306	0	0	0	0
LOC100130396	NC_000004	149624678	149624984	0	0	0	0
LOC100287246	NC_000004	150273103	150474418	0	0	0	0.0570296
LOC285423	NC_000004	150477957	150736980	0	0	0	0
LOC100216487	NC_000004	150711110	150712118	0	0	0	0
DCLK2	NC_000004	151000080	151178609	0.4259729	1.1386568	0.5197129	1.7725283
LRBA	NC_000004	151185594	151936879	3.5427049	2.4369165	4.8031129	5.9082986
LOC729558	NC_000004	151500241	151502697	0	0	0	0
MAB21L2	NC_000004	151503077	151505845	0.0476146	0.0326352	0.0282395	0
LOC649288	NC_000004	151948206	151948862	0	0	0	0

RPS3A	NC_000004	152020754	152025804	44.919463	57.814784	42.035283	68.017685
SNORD73B	NC_000004	152023109	152023383	0	0	0	0
SNORD73A	NC_000004	152024979	152025043	0	0	0	0
SH3D19	NC_000004	152041433	152147660	5.2501579	6.6551849	5.9882489	11.648122
ESSPL	NC_000004	152198325	152212605	0.0445271	0.091557	0	0.2792542
FAM160A1	NC_000004	152330398	152584784	0.2304977	0.6220617	1.5037525	2.9112373
PET112L	NC_000004	152592001	152682146	4.0866829	4.2464759	3.8106031	3.9899145
FBXW7	NC_000004	153242410	153456172	0.914616	0.7388239	0.589494	0.9299974
LOC100287853	NC_000004	153258874	153259203	0	0	0	0
DKFZP434I0714	NC_000004	153457273	153460415	0.2622905	0.2996247	0.4148283	0.9341806
RPS3AP18	NC_000004	153472386	153473535	0	0	0	0
RPS14P6	NC_000004	153534976	153535522	0	0	0	0
TMEM154	NC_000004	153547271	153601191	0.0997205	0.1318154	0.0887141	0.0694892
TIGD4	NC_000004	153690506	153700877	0.0541902	0.1114264	0.0803485	0.0377619
ARFIP1	NC_000004	153701112	153833064	6.8247012	4.9407926	5.8817108	7.0146923
LOC152667	NC_000004	153848582	153850136	0	0	0	0
LOC729870	NC_000004	153855668	153857989	0.1172996	0.1205963	0	0.0272463
FHDC1	NC_000004	153864135	153900848	0.0746035	0.4392831	0.0120671	0.0330824
LOC100287377	NC_000004	153892523	153899077	0.01646	0	0.0292866	0.01147
TRIM2	NC_000004	154074270	154260474	3.9750571	3.8448394	3.9380471	5.1632392
ANXA2P1	NC_000004	154228621	154229963	0	0	0	0
MND1	NC_000004	154265801	154336243	1.5412239	2.2567693	3.4901121	4.0355844
KIAA0922	NC_000004	154387498	154557863	0.4202221	0.3150237	0.4205714	0.7320688
WDR45p	NC_000004	154579618	154581164	0	0	0	0
TLR2	NC_000004	154605441	154627243	0.3745221	0.0796651	0.4595659	0.1799875
RNF175	NC_000004	154631312	154681387	0	0.0287976	0.0249188	0.0390374
SFRP2	NC_000004	154701742	154710228	0.0220845	0.3632836	0.0785881	0.0461682
DCHS2	NC_000004	155155527	155412930	0.0361982	0.1116467	0.0483044	0.0706282
LOC100128050	NC_000004	155437381	155445961	0	0	0	0
PLRG1	NC_000004	155457662	155471523	23.194215	28.331488	26.313561	32.771883
FGB	NC_000004	155484132	155492238	0	0.0231591	0	0.015697
FGA	NC_000004	155504280	155511897	0.0112227	0.0115382	0.0299522	0.0234613
LOC100129857	NC_000004	155506923	155507766	0	0	0	0
FGG	NC_000004	155525286	155533902	0.0221737	0.0227969	0.059179	0.0154515
LRAT	NC_000004	155665163	155674270	0	0	0.0239961	0.0250613
RBM46	NC_000004	155702427	155749965	0.0348657	0	0.0155088	0.0485916
NPY2R	NC_000004	156129781	156138228	0.0121203	0	0.0215651	0.0253377
MAP9	NC_000004	156263810	156298122	0.7551449	0.8194999	0.8157545	0.5972123
LOC100287527	NC_000004	156374200	156375947	0	0	0	0
LOC100287939	NC_000004	156379112	156379792	0	0	0	0
LOC100287564	NC_000004	156380521	156380715	0	0	0	0
TRNAQ54P	NC_000004	156383888	156383959	0	0	0	0
LOC100287591	NC_000004	156384025	156384975	0	0	0	0
GUCY1A3	NC_000004	156587862	156658214	0.0782848	0.0536567	0.0386913	0.054552
GUCY1B3	NC_000004	156680126	156728783	1.9748975	2.1138434	1.7930247	0.7352233
ACCN5	NC_000004	156750881	156787425	0.6493533	0.3738579	0.2772877	0.289597
TDO2	NC_000004	156824847	156841550	0.1038352	0.0533767	0.4849669	0.1266238
CTSO	NC_000004	156845270	156875048	2.8020182	1.7253805	5.4520642	2.8714086
FTHP2	NC_000004	156927434	156928332	0	0	0	0
PDGFC	NC_000004	157682764	157892546	22.098346	19.864469	12.872162	10.877077
LOC100287976	NC_000004	157771388	157892548	0.6658823	0.7416467	1.6784308	1.198701
GLRB	NC_000004	157997277	158093242	1.178468	0.6648964	1.1890365	0.9814382
LOC391707	NC_000004	158125614	158126007	0	0	0	0
GRIA2	NC_000004	158141736	158287227	0.0149738	0.0307894	0.0066606	0.0104344
LOC340017	NC_000004	158493642	158497306	0	0	0	0
LOC100287620	NC_000004	158736272	158736530	0	0	0	0
RPL6P11	NC_000004	158889544	158890714	0	0	0	0
C4orf18	NC_000004	159045732	159094202	3.6287531	0.8819715	0.3815886	1.8770665
TMEM144	NC_000004	159131401	159176439	0.8609509	0.4630005	0.3652877	0.2861276
LOC646890	NC_000004	159198031	159201728	0	0	0	0
LOC100131038	NC_000004	159390739	159391855	0	0	0	0
RXFP1	NC_000004	159443047	159573263	0.2733099	0.0374655	0.3566113	0.3809064
C4orf46	NC_000004	159587831	159593202	1.9823458	1.3074346	2.2626676	4.2918269
ETFDH	NC_000004	159593277	159629842	1.5154563	1.1156396	2.1138318	4.0415974
PPID	NC_000004	159630279	159644552	0.7907806	0.9361883	1.3430461	1.8368248
FNIP2	NC_000004	159690182	159827958	3.1575932	2.0913906	2.9036189	4.5170373

C4orf45	NC_000004	159814684	159956333	0	0	0	0.0749691
PSME2P3	NC_000004	159928241	159929029	0	0	0	0
LOC100240706	NC_000004	159947250	159947874	0	0	0	0
FABP5L12	NC_000004	159947269	159947868	0	0	0	0
RAPGEF2	NC_000004	160188998	160281302	5.1866774	3.6565369	4.132729	3.4885692
RPS14P7	NC_000004	161739574	161740006	0	0	0	0
FSTL5	NC_000004	162305049	163085186	0.0727921	0.037419	0.0242842	0.0253622
LOC100131135	NC_000004	163244043	163245059	0	0	0	0
LOC100128187	NC_000004	163903281	163906711	0	0	0	0
NAF1	NC_000004	164047860	164088073	1.7085706	2.4397085	3.0568723	4.8153156
LOC100287743	NC_000004	164085491	164088042	0.0742369	0.1526466	0	0.0517312
LOC133332	NC_000004	164186579	164187721	0	0	0	0
NPY1R	NC_000004	164245117	164253748	0.015929	0	0.0566837	0.0222
LOC100287774	NC_000004	164252957	164254404	0	0	0	0
NPY5R	NC_000004	164265091	164273086	0	0.0490324	0.0424282	0.0166169
RPL35AP12	NC_000004	164303331	164303668	0	0	0	0
TKTL2	NC_000004	164392247	164395047	0.0156902	0.0322623	0.0139584	0.0328007
C4orf43	NC_000004	164415673	164441691	2.841283	3.9105551	3.7303725	4.7102915
MARCH1	NC_000004	164449049	164534776	0.4664896	0.3029055	0.5460554	1.2318387
LOC646954	NC_000004	164837557	164854596	0	0	0	0
LOC100288073	NC_000004	165109536	165111488	0	0	0	0
ANP32C	NC_000004	165118159	165118863	0.0623379	0	0	0.1737582
LOC100133261	NC_000004	165646685	165648811	0	0	0	0
LOC653794	NC_000004	165823609	165838135	0	0	0	0
RPL26P16	NC_000004	165841729	165842289	0	0	0	0
LOC100131276	NC_000004	165850646	165851778	0	0	0	0
LOC391710	NC_000004	165859459	165861142	0	0	0	0
NACA3P	NC_000004	165864395	165865192	0	0	0	0
LOC391711	NC_000004	165869231	165875273	0	0	0	0
TRIM61	NC_000004	165875598	165898818	0.3894147	0.5147476	0.3216888	0.3682739
C4orf39	NC_000004	165878100	165880273	0	0	0.0179842	0
LOC391713	NC_000004	165931611	165933003	0	0	0	0
RPL21P51	NC_000004	165933478	165934037	0	0	0	0
TRIM60	NC_000004	165953151	165962896	0.023859	0.0245295	0	0
TRIM75	NC_000004	165980300	165981706	0	0.0321133	0.0555758	0.0435322
TMEM192	NC_000004	165997230	166034024	1.2025879	0.8443617	0.9393853	1.4103114
KLHL2	NC_000004	166128788	166244299	0.8620071	0.8862339	1.2948711	1.1816673
GK3P	NC_000004	166198944	166201175	0	0	0	0
SC4MOL	NC_000004	166248818	166264225	21.799474	15.948326	38.147087	14.76122
CPE	NC_000004	166300097	166419482	5.556881	5.6758392	2.6730626	0.4540756
HADHAP	NC_000004	166324840	166327536	0	0	0	0
LOC100132326	NC_000004	166438380	166438652	0	0	0	0
LOC402191	NC_000004	166468448	166472384	0	0	0	0
LOC646995	NC_000004	166585360	166586823	0	0	0	0
TLL1	NC_000004	166794410	167024993	0.092467	0.0746945	0.0646338	0.105857
LOC100128186	NC_000004	167017541	167020701	0	0	0	0
SPOCK3	NC_000004	167654535	168155741	0.04411	0.0151166	0.026161	0.0307376
LOC100288029	NC_000004	168567071	168569950	0	0	0	0
ANXA10	NC_000004	169013707	169108893	0.1846564	3.2590269	0.109517	0.2144599
DDX60	NC_000004	169137442	169239958	4.9860582	3.7646635	7.5581331	1.3060832
DDX60L	NC_000004	169277886	169401638	3.4552134	1.5052214	1.7366405	0.9340724
PALLD	NC_000004	169418217	169849608	10.788462	11.822822	5.2228802	17.170809
RPL9P16	NC_000004	169676949	169677632	0	0	0	0
CBR4	NC_000004	169908741	169931422	1.0300367	0.8759514	1.2783637	2.3128163
SH3RF1	NC_000004	170015407	170192249	12.036724	5.9386352	3.4010798	8.0154002
RPL6P12	NC_000004	170087405	170088297	0	0	0	0
NEK1	NC_000004	170314429	170533614	1.0945115	1.2895463	0.9808151	1.6868457
CLCN3	NC_000004	170541722	170642157	7.8002416	8.2983093	9.3231914	11.294389
LOC100288206	NC_000004	170625148	170628417	0.148725	0.2293574	0	0.0518187
C4orf27	NC_000004	170650619	170679093	5.0598293	5.164879	5.4980952	6.0947537
LOC441050	NC_000004	170712300	170712987	0	0	0	0
LOC402192	NC_000004	170753715	170754552	0	0	0	0
MFAP3L	NC_000004	170907748	170947429	0.2858762	2.1290608	0.7815791	0.7579694
AADAT	NC_000004	170981373	171011372	1.0968777	2.0674602	0.8457044	1.3503482
HSP90AA6P	NC_000004	171502620	171526461	0	0	0	0
LOC100132889	NC_000004	172090285	172113700	0	0	0	0

LOC441052	NC_000004	172733289	172734758	0	0	0	0
GALNTL6	NC_000004	172734575	173961559	0	0.0475615	0	0.0429823
GALNT7	NC_000004	174089904	174245118	3.3060662	1.8253801	6.5268516	7.0749371
HMGB2	NC_000004	174252527	174255595	13.266889	20.172626	54.093244	32.255241
SAP30	NC_000004	174292093	174298683	6.1028203	4.1557322	5.8170409	11.902004
SCRG1	NC_000004	174309299	174320617	0.1946766	0	0	0.0339146
RPL5P11	NC_000004	174340338	174341361	0	0	0	0
LOC100288167	NC_000004	174356964	174357186	0	0	0	0
HAND2	NC_000004	174447652	174451378	1.354823	0.0954041	1.7996768	1.577802
NBLA00301	NC_000004	174451609	174462981	0	0	0	0
MORF4	NC_000004	174537087	174537794	0.1241475	0.1276367	0.2208902	0.2595329
LOC100128266	NC_000004	174554879	174555522	0	0	0	0
FBXO8	NC_000004	175157809	175205402	2.348248	2.7138237	5.6267557	5.3748811
KIAA1712	NC_000004	175204828	175254531	0.9390648	0.8827038	1.6708361	1.7294269
HPGD	NC_000004	175411380	175443611	0	0	0	0.0482852
GLRA3	NC_000004	175563198	175750465	1.1962813	1.1802098	0.8707459	0.5557443
ADAM29	NC_000004	175839509	175899331	0.026435	0	0.0117587	0.0460524
LOC100131553	NC_000004	175938227	175940169	0	0	0	0
LOC100129957	NC_000004	176195531	176211942	0	0	0	0
LOC391718	NC_000004	176415736	176417964	0	0	0	0
LOC100288265	NC_000004	176495711	176497337	0	0	0	0
GPM6A	NC_000004	176554088	176923648	0.0231428	0.0237933	0.082354	0.0483805
WDR17	NC_000004	176987132	177103966	0.2567279	0.3621547	0.4992761	0.6032614
SPATA4	NC_000004	177105725	177116822	0.10693	0.0732902	0.1268372	0.1987016
ASB5	NC_000004	177134828	177190275	0.0600181	0.138836	0.0133484	0.0731902
LOC100132013	NC_000004	177239505	177241615	0.4788296	0.1926341	0.2407714	0.2901457
SPCS3	NC_000004	177241090	177253396	14.056549	8.9953901	10.488968	18.124384
VEGFC	NC_000004	177604694	177713895	32.304914	12.775846	14.237842	10.562338
NEIL3	NC_000004	178230991	178284092	2.9823321	3.1225831	6.3480644	4.041668
AGA	NC_000004	178351924	178363591	6.1368183	2.7008207	4.7507086	2.7909002
LOC285501	NC_000004	178649907	178911669	0	0.0559201	0	0.0379021
RPL19P8	NC_000004	179207825	179208518	0	0	0	0
LOC100288304	NC_000004	179990579	179990770	0	0	0	0
LOC100288337	NC_000004	181494617	181494906	0	0	0	0
LOC132386	NC_000004	182443813	182444574	0	0	0	0
LOC100288373	NC_000004	182525584	182526434	0	0.0530945	0	0
LOC100128118	NC_000004	182826942	182827602	0	0	0	0
MGC45800	NC_000004	183060159	183065668	0	0	0	0
ODZ3	NC_000004	183245137	183724177	0.6057619	2.3615747	2.7885498	4.3146799
DCTD	NC_000004	183811244	183838630	12.934813	15.577431	12.866584	17.157131
LOC100132090	NC_000004	183949753	183950224	0	0	0	0
FAM92A3	NC_000004	183958818	183961272	0	0	0	0
LOC100128823	NC_000004	184018170	184020352	0.1006602	0.0620935	0.05373	0.0982016
C4orf38	NC_000004	184018174	184020352	0	0	0	0
WWC2	NC_000004	184020463	184241930	1.2023526	0.8565817	1.1007148	2.4613933
CLDN22	NC_000004	184239220	184241927	0	0	0	0
CLDN24	NC_000004	184242917	184243579	0	0.0681499	0	0.0923827
LOC100129666	NC_000004	184272251	184302858	0	0	0	0
LOC100127981	NC_000004	184340035	184340958	0	0	0	0
LOC100131811	NC_000004	184362513	184366612	0.1468613	0.1132416	0.0979889	0.0767541
CDKN2AIP	NC_000004	184365789	184369049	4.8872507	4.2633033	5.5336066	4.4247402
LOC389246	NC_000004	184404506	184405569	0	0	0	0
ING2	NC_000004	184426220	184432249	3.3473116	2.996032	2.6275246	2.1130068
RWDD4A	NC_000004	184560789	184580331	3.6100939	3.4501788	3.1664052	4.8895872
C4orf41	NC_000004	184580446	184634745	4.1074021	4.1329935	4.4228797	4.3778825
STOX2	NC_000004	184826509	184938875	0.0755449	0.0873766	0.058806	0.2566331
ENPP6	NC_000004	185009859	185139114	0.1004914	0.0688771	0.0099333	0.0389036
RPL6P16	NC_000004	185175688	185176474	0	0	0	0
LOC391722	NC_000004	185220282	185238554	0.2325303	0.0796885	0.0689551	0.1080243
LOC728175	NC_000004	185262843	185269065	0.1558448	0	0.2079658	0.0542994
IRF2	NC_000004	185308876	185395726	6.8633065	4.1704715	11.031465	7.2476199
LOC100129245	NC_000004	185480329	185480937	0	0	0	0
CASP3	NC_000004	185548850	185570629	3.1896584	1.7072666	1.7844755	2.1310241
CCDC111	NC_000004	185570767	185616112	1.8766565	2.0869022	2.163569	2.2151328
MLF1IP	NC_000004	185615236	185655286	3.5221046	3.3713632	6.544559	12.380527
ACSL1	NC_000004	185676749	185747215	4.100295	4.8923946	5.1273811	6.0686355

SLED1	NC_000004	185719450	185720200	0	0	0	0
HELT	NC_000004	185940083	185941926	0.0446628	0.0459181	0	0
SLC25A4	NC_000004	186064398	186068425	0.430865	0.6133493	0.4717655	0.5542964
KIAA1430	NC_000004	186080819	186125182	4.1525179	3.7228382	5.136614	7.2623456
SNX25	NC_000004	186131284	186285120	3.0776073	7.5657237	3.2368355	5.7951839
LRP2BP	NC_000004	186285032	186300152	0.1192856	0.1226382	0.1440197	0.3621787
ANKRD37	NC_000004	186317840	186321390	1.2704521	2.080178	1.4232519	2.7542717
UFSP2	NC_000004	186321443	186347068	7.2730236	7.1876098	7.3981923	11.609559
LOC100288644	NC_000004	186346203	186348049	0.1961975	0.0806846	0.0349085	0.0273436
C4orf47	NC_000004	186350545	186370821	0.0451214	0.1391686	0.2408474	0.5030781
CCDC110	NC_000004	186366336	186392913	0.2274753	0.0779562	0.1484035	0.2430546
LOC100131727	NC_000004	186392591	186393417	0	0.2865755	0.0826587	0
LOC100131683	NC_000004	186421814	186422626	0	0	0.0480905	0.075338
PDLIM3	NC_000004	186422851	186456712	0.8333497	0.4734788	0.8779393	0.0458456
SORBS2	NC_000004	186506598	186877870	1.1050238	0.1834031	0.3350338	0.555937
TLR3	NC_000004	186990309	187006252	2.3556599	0.6388972	1.5171038	0.7150169
FAM149A	NC_000004	187065995	187093817	1.1539661	0.8354919	0.5205297	0.2038638
FLJ38576	NC_000004	187110186	187113275	0	0	0	0
CYP4V2	NC_000004	187112674	187134617	2.3076559	1.8442205	1.3963416	1.4192652
KLKB1	NC_000004	187148672	187179625	0.0586759	0.1407583	0.0347998	0.0408877
F11	NC_000004	187187118	187210835	0	0.0275677	0.047709	0.0373702
SLC25A5P6	NC_000004	187249609	187250834	0	0	0	0
MTNR1A	NC_000004	187454809	187476537	0.0397722	0	0	0.0277148
FAT1	NC_000004	187508937	187644987	12.451602	7.3081796	9.7134835	15.621509
LOC100286957	NC_000004	187543069	187544347	0	0	0	0.0239444
MRPS36P2	NC_000004	187821893	187821995	0	0	0	0
LOC644325	NC_000004	188667288	188669359	0	0	0	0
LOC389249	NC_000004	188891427	188892438	0	0	0	0
ZFP42	NC_000004	188916925	188926199	1.9432892	0.0170761	0.1920893	0.1388883
TRIML2	NC_000004	189012427	189026408	0.1690317	0.1737823	0.0300751	0.2826912
FAUP3	NC_000004	189027712	189028068	0	0	0	0
TRIML1	NC_000004	189060598	189068649	0.0267163	0.0549342	0.0237675	0
RPL7AP27	NC_000004	189270472	189271201	0	0	0	0
LOC644491	NC_000004	189308625	189315164	0	0	0	0
LOC285442	NC_000004	189659525	189662227	0	0	0	0
HSP90AA4P	NC_000004	190394060	190396545	0	0	0	0
FRG1	NC_000004	190861974	190884359	5.4355915	8.4908406	10.271077	10.511703
TUBB4Q	NC_000004	190903678	190906026	0	0.0346233	0.0299598	0
DUX4C	NC_000004	190940255	190945506	0	0	0	0
FRG2	NC_000004	190945522	190948412	0	0	0	0.0146952
LOC644881	NC_000004	190959056	190959857	0	0	0	0
LOC100132380	NC_000004	190962073	190964569	0	0	0	0
LOC100287020	NC_000004	190987701	190990406	0	0	0	0
LOC653543	NC_000004	190992093	190993703	0	0.0280468	0	0
LOC653544	NC_000004	190995386	190996996	0	0	0	0
LOC653545	NC_000004	190998679	191000289	0	0	0	0
LOC441056	NC_000004	191001979	191003583	0	0	0	0
DUX4	NC_000004	191005267	191006883	0	0	0	0
LOC653548	NC_000004	191008566	191010176	0	0	0	0
LOC728410	NC_000004	191011866	191013476	0	0	0	0
LOC100128803	NC_000005	58313	59042	0	0	0	0
PLEKHG4B	NC_000005	140373	190087	0.0305329	0.094173	0.0237675	0.0505317
LOC389257	NC_000005	191626	195468	0	0.0287243	0.0248554	0
LOC100288685	NC_000005	195827	196463	0	0	0	0.0961535
CCDC127	NC_000005	204875	218297	1.3927257	1.2727719	5.0386299	4.1623954
SDHA	NC_000005	218356	256815	6.5830406	7.4675497	11.189429	14.12076
PDCD6	NC_000005	271736	315089	15.836852	13.773971	30.968028	26.928307
AHRR	NC_000005	304291	438406	4.0291702	5.0443226	2.8454685	4.5929203
C5orf55	NC_000005	441613	443258	1.2282009	1.2627196	0.5225677	0.8558593
EXOC3	NC_000005	443334	467411	2.226032	1.9779999	1.8813228	3.7560904
LOC25845	NC_000005	470625	473080	0	0	0	0
SLC9A3	NC_000005	473334	524549	0.3323417	0.2440587	0.2675022	0.2867291
LOC100288152	NC_000005	477929	481007	0.0570942	0.0586988	0.0126981	0
CEP72	NC_000005	612405	653668	1.1278106	1.0472974	1.5859112	1.8253239
TPPP	NC_000005	659977	693510	0.0510856	0.0300122	0.1233567	0.2186765
LOC100132605	NC_000005	663645	675900	0	0.0510547	0.044178	0.0173022

ZDHHC11B	NC_000005	732182	767034	0.1084472	0.0836213	0.0723582	0.0566778
ZDHHC11	NC_000005	795720	851101	0	0.0866911	0.0450087	0.1645235
LOC100288739	NC_000005	806009	817116	0	0	0	0
LOC100129204	NC_000005	850931	853557	0	0	0	0
LOC100132536	NC_000005	853865	863735	0	0	0	0
BRD9	NC_000005	863856	892915	0.9580838	1.4775163	1.3472438	2.2720989
TRIP13	NC_000005	893004	918160	5.065917	10.358934	10.909402	11.189609
NKD2	NC_000005	1009168	1038925	0.0488857	0	0.021745	0.0510982
SLC12A7	NC_000005	1050489	1112172	2.7343357	1.5844858	5.1009644	2.7772561
SLC6A19	NC_000005	1201710	1225232	0.0084941	0	0	0.023676
SLC6A18	NC_000005	1225470	1246304	0	0	0	0.0144389
TERT	NC_000005	1253282	1295162	32.025989	343.15995	167.81399	268.38308
CLPTM1L	NC_000005	1317999	1345002	15.038152	18.216399	22.260858	23.282321
SLC6A3	NC_000005	1392909	1445538	0	0.0115117	0.0398447	0.015605
LPCAT1	NC_000005	1461542	1524076	0.8018937	1.1335927	1.3475086	0.9546021
SDHAP3	NC_000005	1572072	1594646	0	0	0	0
LOC728613	NC_000005	1597672	1634120	0	0	0	0
MRPL36	NC_000005	1798499	1799956	3.5788462	5.9606768	6.622389	7.2322595
NDUFS6	NC_000005	1801509	1816165	5.6893583	6.1883459	15.184233	15.743371
IRX4	NC_000005	1877541	1882880	0.0197876	0.0406874	0.0176036	0.0413663
IRX2	NC_000005	2746279	2751769	0.5606742	0.4729699	0.8057398	1.0518849
C5orf38	NC_000005	2752262	2755511	0.4938004	0.6599823	0.5271582	1.445219
LOC100288833	NC_000005	2752437	2755508	0.3413455	0.0877348	0.379588	0.1189316
LOC100130748	NC_000005	2787558	2788745	0	0	0	0
IRX1	NC_000005	3596168	3601517	0.0946141	0.972732	0	0.0329654
LOC340094	NC_000005	5034472	5070117	0	0	0	0
ADAMTS16	NC_000005	5140443	5320412	0.4064372	0.2543497	0.3144155	0.6649551
LOC442131	NC_000005	5375745	5376957	0	0	0	0
KIAA0947	NC_000005	5422807	5490338	2.7030549	3.5550972	5.3575219	7.3293943
FLJ33360	NC_000005	6310554	6337405	0	0.0186785	0.0161627	0.0126601
MED10	NC_000005	6372039	6378639	7.0539477	8.7905451	9.3179996	12.035458
FLJ25076	NC_000005	6448736	6492706	0.0878525	0.1806433	0.0781561	0.0306096
LOC255167	NC_000005	6582287	6588613	0	0	0	0
NSUN2	NC_000005	6599352	6633157	2.1571449	2.2621271	2.3668356	4.4293836
SRD5A1	NC_000005	6633500	6669675	3.6105879	2.4747093	2.7285404	3.9769056
POLS	NC_000005	6714718	6757161	3.5172245	3.5107538	4.9314982	6.7579374
LOC100130063	NC_000005	7288037	7288590	0	0	0	0
LOC442132	NC_000005	7299605	7306705	0	0	0	0
ADCY2	NC_000005	7396343	7830194	0.0134132	0	0.0059664	0.0373873
C5orf49	NC_000005	7831511	7851264	0.0428345	0	0.0762136	0.2089417
LOC100288963	NC_000005	7849642	7861795	0	0	0.0507761	0.0198863
FASTKD3	NC_000005	7859272	7869115	1.1526805	1.1286445	1.5788775	1.5809679
MTRR	NC_000005	7869217	7901237	6.8719053	6.613207	9.2056995	11.080638
LOC729506	NC_000005	8457640	8461768	2.2458409	0.5362041	0.587079	0.8084553
LOC100289001	NC_000005	8619290	8620426	0	0	0	0
LOC100128382	NC_000005	8839871	8842572	0.0162651	0.0334444	0.0434096	0.0453366
SEMA5A	NC_000005	9035138	9546233	2.2629171	4.3698717	0.9966436	1.2890044
SNORD123	NC_000005	9548939	9549026	0	0	0	0
TAS2R1	NC_000005	9629109	9630463	0	0	0	0.0226014
LOC285692	NC_000005	9641427	9790037	0	0	0	0
FAM173B	NC_000005	10226438	10250014	1.4284366	1.2727719	0.7201068	3.2847919
CCT5	NC_000005	10250282	10266501	28.011671	38.438422	38.546087	59.296892
CMBL	NC_000005	10277707	10308168	1.7049351	1.2281132	1.7872621	1.5739992
MARCH6	NC_000005	10353828	10435491	9.6074209	9.3776872	16.237657	16.179181
ROPN1L	NC_000005	10442009	10465138	0.2528182	0.1299618	0.0374857	0.1761738
RPL30P7	NC_000005	10488864	10489302	0	0	0	0
ANKRD33B	NC_000005	10564580	10650225	1.9532547	0.7910899	0.9478198	2.2478867
LOC645763	NC_000005	10664224	10665930	0	0	0	0
DAP	NC_000005	10679342	10761387	7.8814079	6.4437473	8.5807638	11.847199
CTNND2	NC_000005	10971952	11904110	0.0808466	0.0332475	0.0575387	0.0338023
RPS23P5	NC_000005	13250347	13250774	0	0	0	0
RPL29P13	NC_000005	13637900	13638496	0	0	0	0
DNAH5	NC_000005	13690437	13944589	0.1298156	0.0841404	0.4468867	0.3756085
TRIO	NC_000005	14143829	14509458	2.9393361	4.904036	5.8873815	11.280328
LOC100289578	NC_000005	14293175	14297622	0.085171	0.0875647	0	0
FAM105A	NC_000005	14581891	14616289	0.1059745	0.1730428	0.0332745	0.0304077

LOC391739	NC_000005	14639532	14641127	0	0	0	0
EEF1AL11	NC_000005	14651752	14653336	0	0	0	0
LOC100287064	NC_000005	14652539	14653498	5.5899067	9.6113815	13.075392	12.021608
LOC728178	NC_000005	14661917	14664713	0	0	0	0
FAM105B	NC_000005	14664783	14699842	0.7680922	0.8812366	1.3270197	1.8810874
ANKH	NC_000005	14704909	14871887	4.2839753	4.299773	2.7202035	2.6344784
LOC100288785	NC_000005	14874495	14874820	0	0	0	0
LOC402198	NC_000005	14904903	15004884	0.0288943	0.0594128	0	0.0201347
LOC100270647	NC_000005	15324369	15325545	0	0	0	0
LOC391741	NC_000005	15450812	15451943	0	0	0	0
FBXL7	NC_000005	15500305	15939900	2.1482805	1.2182285	3.9680344	4.9475088
LOC100287480	NC_000005	15616137	15928602	1.0064481	0.3449115	0.7162913	0.9351108
MARCH11	NC_000005	16067474	16179897	1.0354295	0.3548435	0.4093986	0.260552
LOC100131397	NC_000005	16179833	16184128	0	0	0	0
LOC100132778	NC_000005	16192040	16192816	0	0	0	0
ZNF622	NC_000005	16451628	16465894	4.9610593	3.9789166	4.7369982	4.8869487
FAM134B	NC_000005	16473147	16617118	1.2521563	0.2271791	1.1576374	1.4114818
LOC100288944	NC_000005	16615929	16617227	0.0613802	0.1262106	0	0.0427722
MYO10	NC_000005	16662016	16936385	0.9497124	1.1859161	0.7559547	1.4254099
RPS26P28	NC_000005	16902336	16902775	0	0	0	0
LOC285696	NC_000005	17130137	17217531	0	0	0	0
LOC100288979	NC_000005	17202341	17216477	0	0	0	0
BASP1	NC_000005	17217750	17276943	12.057027	15.388003	17.710305	23.586439
LOC646012	NC_000005	17292568	17307073	0	0	0	0
FTHL10	NC_000005	17353822	17354740	0	0	0	0
LOC285697	NC_000005	17486342	17486938	0	0	0	0
LOC340096	NC_000005	17491434	17491877	0.0989825	0.3052933	0	0.068975
LOC391742	NC_000005	17498340	17498936	0	0	0	0
LOC391744	NC_000005	17511905	17512347	0	0	0	0
LOC646103	NC_000005	17518476	17519072	0	0	0	0
LOC391746	NC_000005	17521910	17522506	0	0	0	0
LOC646066	NC_000005	17525344	17525940	0	0	0	0
LOC391747	NC_000005	17528778	17529374	0	0	0	0
LOC729724	NC_000005	17585271	17585867	0	0	0	0
LOC729706	NC_000005	17590473	17591069	0	0	0	0
LOC729711	NC_000005	17593907	17594503	0.0736151	0	0	0
LOC402207	NC_000005	17597341	17597937	0	0	0	0
LOC391764	NC_000005	17604890	17605486	0	0	0	0
LOC100288018	NC_000005	17611099	17611692	0	0	0	0
LOC100289108	NC_000005	17614851	17616213	0	0	0	0
LOC100289141	NC_000005	17620493	17621048	0	0	0	0
LOC391765	NC_000005	17624768	17626242	0	0	0	0
LOC391766	NC_000005	17632197	17632793	0	0	0	0
LOC391767	NC_000005	17634569	17635162	0	0	0	0
LOC391768	NC_000005	17649155	17649795	0	0	0	0
LOC391769	NC_000005	17655237	17655647	0	0.2198706	0	0
LOC100133112	NC_000005	17670277	17694147	0	0	0	0
RPL36AP21	NC_000005	18049653	18050024	0	0	0	0
RPL32P14	NC_000005	19041145	19041477	0	0	0	0
LOC646273	NC_000005	19233475	19234648	0	0	0	0
CDH18	NC_000005	19473141	19839353	1.5556896	2.3351422	0.0276797	0.1409286
NUP50P3	NC_000005	20302313	20305934	0	0	0	0
LOC728411	NC_000005	21459589	21589481	0	0	0	0
LOC100288118	NC_000005	21473484	21474815	0.2969475	0.3392147	0.1174101	0.0459833
GUSBP1	NC_000005	21491430	21500267	0	0	0	0
CDH12	NC_000005	21750973	22853731	0.0202293	0.0103989	0.0449914	0.1268695
HSPD1P1	NC_000005	21882626	21884875	0	0	0	0
PMCHL1	NC_000005	22142461	22152381	0	0	0	0
LOC100128381	NC_000005	23301031	23301181	0	0	0	0
LOC391771	NC_000005	23303671	23305251	0	0	0	0
PRDM9	NC_000005	23507724	23528706	0.0238137	0	0	0.0082972
LOC503540	NC_000005	24170299	24172338	0	0	0	0
LOC100130746	NC_000005	24170320	24172359	0	0	0	0
CDH10	NC_000005	24487210	24644911	0.0134769	0.0692784	0.0479578	0
LOC100288253	NC_000005	25188941	25191118	0.0201783	0.0207454	0.0179511	0.014061
MSNL1	NC_000005	25909519	25913382	0	0	0	0

LOC100131678	NC_000005	26739859	26740567	0	0	0	0
CDH9	NC_000005	26880709	27038689	0.0571684	0.0146938	0.038144	0.0099593
LOC100128659	NC_000005	28213701	28217212	0	0	0	0
LOC100289481	NC_000005	28808645	28809318	0	0	0	0
LOC729862	NC_000005	28925705	28927696	0	0	0	0
LOC100130528	NC_000005	28998904	29003498	0	0	0	0
LOC100131259	NC_000005	29105671	29151330	0	0	0	0
LOC100130803	NC_000005	29600714	29600974	0	0	0	0.1173367
PGBD3P2	NC_000005	29881806	29883060	0	0	0	0
HPRTP2	NC_000005	30248481	30249588	0	0	0	0
RPL19P11	NC_000005	31053630	31054355	0	0	0	0
CDH6	NC_000005	31193796	31325237	0.4763246	0.0199882	0.008648	0.0067739
RNASEN	NC_000005	31400601	31532282	2.4779832	2.7037721	4.856973	9.0738317
C5orf22	NC_000005	31532373	31555165	4.063873	3.1773489	6.9817081	5.4856856
PDZD2	NC_000005	31799031	32111038	0.0868244	0.0465728	0.1477661	0.1315276
RPL9P17	NC_000005	31822567	31823271	0	0	0	0
RPL21P56	NC_000005	31840563	31841107	0	0	0	0
RPL5P14	NC_000005	31847336	31848323	0	0	0	0
GOLPH3	NC_000005	32124824	32174425	11.200558	8.6449558	14.391294	14.248122
RPL27P10	NC_000005	32222003	32222494	0	0	0	0
MTMR12	NC_000005	32227111	32313114	3.0840864	3.3798265	4.6582409	7.8230121
ZFR	NC_000005	32354456	32444844	8.9387814	11.087422	12.927488	14.510282
LOC646616	NC_000005	32522852	32524192	0	0	0	0
SUB1	NC_000005	32585605	32604185	8.2332445	11.273304	16.96792	14.941308
NPR3	NC_000005	32711665	32787256	2.7903639	1.3490131	0.3398503	1.66666
LOC100288353	NC_000005	32712080	32713555	0.0297752	0	0	0.0207486
C5orf23	NC_000005	32788945	32791819	1.5133478	0.7700823	0.3263797	1.4699942
RPS8P8	NC_000005	33162262	33162965	0	0	0	0
TARS	NC_000005	33440898	33468196	21.40356	26.253935	33.59647	43.788203
LOC646639	NC_000005	33502177	33503238	0	0	0	0
ADAMTS12	NC_000005	33527286	33892124	0.5055599	1.9422936	0.14992	0.2039598
RXFP3	NC_000005	33936491	33938342	0.0474603	0	0	0.0165361
SLC45A2	NC_000005	33944721	33984780	0.0437441	0.0149912	0.0778319	0.0406435
PSMC6P3	NC_000005	33970959	33973033	0	0	0	0
AMACR	NC_000005	33987895	34008206	2.7576041	6.5974186	1.8515027	2.8159418
C1QTNF3	NC_000005	34017963	34043317	0.0116512	0	0.0103652	0.048714
LOC643373	NC_000005	34174641	34183012	0	0	0	0
LOC729915	NC_000005	34188958	34195614	0.015025	0.0077237	0.0267334	0.0314101
LOC100132524	NC_000005	34373134	34373996	0	0	0	0
LOC401180	NC_000005	34656411	34666943	0.0457794	0	0	0
RAI14	NC_000005	34656433	34832717	13.763842	13.595142	13.039198	11.4791
TTC23L	NC_000005	34839269	34899567	0.0323387	0.0332475	0.0575387	0.0225349
RPL21P54	NC_000005	34883182	34883624	0	0	0	0
RAD1	NC_000005	34905365	34915780	2.7684664	2.585768	4.1827633	5.2251287
BXDC2	NC_000005	34915820	34925787	14.741473	12.351786	17.352808	20.352433
DNAJC21	NC_000005	34929698	34959069	0.3468852	0.4512518	0.6046015	1.317146
AGXT2	NC_000005	34998206	35048076	0.0588067	0.0604595	0.1046322	0.0546385
PRLR	NC_000005	35055802	35230794	0.0372822	0.072827	0.1824199	0.1039188
SPEF2	NC_000005	35617989	35814713	0.1950458	0.1617158	0.2910628	0.5480472
IL7R	NC_000005	35856991	35876924	6.3791893	2.0025885	3.0324982	19.732262
CAPSL	NC_000005	35904397	35938881	0.0399893	0.0822264	0.1067267	0.640921
UGT3A1	NC_000005	35953210	35991499	0.0311358	0	0	0
UGT3A2	NC_000005	36035124	36066984	0.0187733	0	0.1169086	0.0261639
LMBRD2	NC_000005	36103414	36152015	2.6882144	3.2383532	4.5414534	5.5724601
SKP2	NC_000005	36152189	36184147	5.957427	7.6560764	11.077644	15.193342
C5orf33	NC_000005	36192694	36242258	1.5162251	3.1520638	3.9276093	3.4804529
RANBP3L	NC_000005	36249104	36302004	0.069319	0.0356336	0.0616681	0.0483042
RPL39P22	NC_000005	36588608	36588763	0	0	0	0
SLC1A3	NC_000005	36606457	36688436	0.8852881	0.5309321	2.5127452	1.1456789
LOC646719	NC_000005	36864527	36881303	0	0	0	0
NIPBL	NC_000005	36876861	37065921	3.3672916	4.5492192	5.9047146	7.9591078
KRT18P31	NC_000005	36885261	36886658	0	0	0	0
RPS4P6	NC_000005	37085107	37086020	0	0	0	0
C5orf42	NC_000005	37106330	37213855	0.9007279	1.1401569	1.6535755	2.7718762
OFD1P1	NC_000005	37209101	37212577	0	0	0	0
LOC100287003	NC_000005	37226743	37228197	1.2007714	1.4320422	1.623724	4.2506662

NUP155	NC_000005	37291941	37371197	6.9727921	10.692564	18.801779	24.30144
RNU7-75P	NC_000005	37327237	37327297	0	0	0	0
WDR70	NC_000005	37379412	37752774	2.4852911	3.9604678	2.339958	4.3151926
LOC100287032	NC_000005	37813681	37814281	1.8281294	3.5334774	0.5204335	0.2038261
GDNF	NC_000005	37815753	37839782	1.2247096	3.7773905	0.5447678	0.2625927
EGFLAM	NC_000005	38258533	38465581	1.4248169	1.8352863	0.8304681	1.0784613
LIFR	NC_000005	38475065	38595507	1.0519187	0.8643177	2.1610209	2.1607864
LOC100132789	NC_000005	38763247	38764389	0	0	0	0
LOC729989	NC_000005	38843622	38846353	0.2469002	0.3046072	0.1757194	0.4473297
OSMR	NC_000005	38846136	38934402	14.737384	9.5185788	29.095426	37.135332
RICTOR	NC_000005	38938022	39074501	4.2735771	5.1994327	8.8669817	5.9142349
FYB	NC_000005	39105357	39219667	0.0455139	0.0374345	0.0161962	0.0507454
C9	NC_000005	39284377	39364655	0.1468749	0.0167781	0.1597004	0.0568602
DAB2	NC_000005	39371780	39425335	58.919502	25.024351	35.984729	52.109593
LOC100129040	NC_000005	39571576	39625336	0	0	0	0
LOC285634	NC_000005	39719083	39757690	0	0	0	0
LOC100287271	NC_000005	39888676	39899862	0	0	0	0
LOC100127944	NC_000005	40680028	40692786	0.6543811	0.1614654	0.3260071	0.3100792
PTGER4	NC_000005	40680032	40693837	1.3380024	0.4761717	1.8884948	0.3944656
TTC33	NC_000005	40711678	40756072	0.3192752	0.3200423	0.7455947	1.09017
PRKAA1	NC_000005	40759481	40798297	7.9158219	7.3191826	10.517474	10.244111
RPL37	NC_000005	40831430	40835387	42.716562	45.902883	87.508819	73.967859
SNORD72	NC_000005	40832758	40832837	0	0	0	0
CARD6	NC_000005	40841410	40855456	5.7496344	4.23491	4.7142293	11.010604
C7	NC_000005	40909599	40983041	0.0109542	0.0225241	0	0.0305333
HEATR7B2	NC_000005	40998122	41071444	0.0083235	0	0.0296194	0.034801
C6	NC_000005	41142336	41261540	0.0228243	0.0234658	0.0203051	0.0397622
PLCXD3	NC_000005	41307048	41510730	0	0.0350304	0	0.0118716
TCP1P2	NC_000005	41585869	41588083	0	0	0	0
OXCT1	NC_000005	41730167	41870791	0.6917091	0.5841587	0.7801931	2.5994135
LOC100288462	NC_000005	41870222	41871103	0	0	0.0498693	0
RPS2P22	NC_000005	41895041	41895770	0	0	0	0
C5orf51	NC_000005	41904470	41921738	4.5025477	4.2656869	11.200681	11.88752
FBXO4	NC_000005	41925356	41941672	2.8068425	5.0599085	5.2335324	5.6802049
RPSAP38	NC_000005	41951415	41951989	0	0	0	0
LOC100130042	NC_000005	41967239	41968467	0	0	0	0
LOC100129630	NC_000005	42423504	42424692	0.112256	0.115411	0	0.1173367
GHR	NC_000005	42424026	42721926	0.231837	0.3212581	0.4842359	0.2247697
CCDC152	NC_000005	42756920	42802539	0.3021362	0.1164854	0.335986	0.0789527
SEPP1	NC_000005	42799982	42812024	2.5734552	0.0615298	5.750164	0.8757909
LOC402213	NC_000005	42892418	42892918	0	0	0	0
LOC100130593	NC_000005	42908307	42911424	0	0	0	0
LOC100132940	NC_000005	42915488	42918093	0	0	0	0
LOC100129186	NC_000005	42950264	42955114	0	0	0	0
LOC730974	NC_000005	42971043	42973474	0	0	0	0
FLJ32255	NC_000005	42991482	42995503	0	0	0	0
LOC100288522	NC_000005	42996388	43001008	0	0.1092706	0.2363819	0.1851565
LOC648987	NC_000005	43016151	43019744	0	0	0	0
C5orf39	NC_000005	43039182	43040447	0.5207136	0.2855191	0.9264826	0.4112345
LOC153684	NC_000005	43042236	43045370	0	0	0	0
LOC100132356	NC_000005	43065221	43067528	0	0	0	0
ZNF131	NC_000005	43121642	43175836	3.0754663	4.3218906	5.7472612	5.4021524
MGC42105	NC_000005	43192327	43280952	0.1710048	0.1758109	0.169034	0.1324033
HMGCS1	NC_000005	43289493	43313595	10.1419	8.5475153	14.358052	5.008171
LOC100288555	NC_000005	43348431	43348773	0	0	0	0
CCL28	NC_000005	43381600	43412488	1.7773182	1.3842954	1.1499284	1.8014634
C5orf28	NC_000005	43444354	43483992	1.416658	1.2928246	2.4073112	2.0741949
C5orf34	NC_000005	43486810	43515187	0.3422198	0.6296048	1.6824772	2.0583935
PAIP1	NC_000005	43526369	43557521	9.5353604	10.620299	17.536808	20.327849
LOC728931	NC_000005	43587032	43603770	0.3310601	0.297819	0.4049654	0.4037178
NNT	NC_000005	43602791	43705668	5.3960375	4.0279054	9.7864934	14.067474
RPL29P12	NC_000005	43666736	43667374	0	0	0	0
LOC100129791	NC_000005	43715450	43719976	0.009708	0.0499044	0.0777288	0.0811793
FGF10	NC_000005	44305097	44388784	0	0	0.0623566	0.0488435
MRPS30	NC_000005	44809027	44815616	3.9737623	5.4382418	6.5786922	4.8046216
LOC100287671	NC_000005	44839189	44841137	0	0	0.0401206	0.0157131

HCN1	NC_000005	45259352	45696220	0.0409278	0.0673249	0.0436926	0.0399281
EMB	NC_000005	49692031	49737234	0.8567304	0.0104858	2.0687503	2.1747999
LOC100128182	NC_000005	49899062	49899348	0	0	0	0
LOC202319	NC_000005	49928964	49929441	0	0	0	0
PARP8	NC_000005	49962813	50138176	2.0517104	0.0917119	5.6344936	2.8387066
LOC100287592	NC_000005	50265051	50266004	0	0	0	0
RPS10P12	NC_000005	50571051	50571614	0	0	0	0
LOC642366	NC_000005	50672932	50679488	0	0	0	0
ISL1	NC_000005	50678958	50690564	0.0323387	0.0166238	0.0287694	0.0225349
LOC100288136	NC_000005	50723830	50740958	0	0	0	0
LOC100128982	NC_000005	51226642	51227894	0	0	0	0
RPS17P11	NC_000005	51578345	51578696	0	0	0	0
LOC100128154	NC_000005	51875386	51876964	0	0	0	0
LOC100287776	NC_000005	52083767	52097842	0.1324312	0.1750541	0.5385803	0.2109333
PELO	NC_000005	52083774	52098451	0.2107107	0.4332655	0.5088039	0.5139106
ITGA1	NC_000005	52084136	52249485	3.9219857	3.1466339	9.5624363	6.5068288
RPL17P21	NC_000005	52199738	52200308	0	0	0	0
ITGA2	NC_000005	52285156	52390609	1.1951864	8.0272456	2.7923284	2.07435
MOCS2	NC_000005	52393892	52405598	7.9374183	10.279367	12.809532	24.026362
LOC257396	NC_000005	52405672	52408736	0.6316994	0.6958429	0.4014124	0.5030781
RPL13AP13	NC_000005	52502393	52503008	0	0	0	0
RPS19P4	NC_000005	52709307	52710090	0	0	0	0
FST	NC_000005	52776595	52781923	39.921229	11.181751	12.976104	26.426663
NDUFS4	NC_000005	52856465	52979171	14.367691	17.244553	39.328912	21.51896
ASSP9	NC_000005	53154894	53156461	0	0	0	0
ARL15	NC_000005	53180614	53606403	0.4596356	0.4362034	0.157271	0.4434817
HSPB3	NC_000005	53751445	53752207	0.2879963	0	0	0
SNX18	NC_000005	53813589	53842416	3.0315958	3.8501636	3.1964346	2.3932878
LOC100288234	NC_000005	54104067	54130037	0	0	0	0
LOC100130990	NC_000005	54152991	54154322	0	0	0	0
LOC727930	NC_000005	54157442	54173519	0	0	0	0
LOC100130574	NC_000005	54173939	54174419	0	0	0	0
ESM1	NC_000005	54273695	54281414	0.3997206	0.108146	0.0374318	0
GZMK	NC_000005	54320107	54329960	0.0420557	0	0	0.0293061
GZMA	NC_000005	54398474	54406080	0	0	0	0
CDC20B	NC_000005	54408823	54469003	0.0297148	0.015275	0.0528703	0.0310597
GPX8	NC_000005	54455984	54463129	6.7738875	10.434354	5.369399	6.3291415
LOC345643	NC_000005	54516194	54522966	0	0	0	0
CCNO	NC_000005	54526980	54529508	1.1654102	0.1576532	1.0913487	0.9189601
LOC100288375	NC_000005	54527751	54529436	0.0604515	0.0621505	0	0
DHX29	NC_000005	54552073	54603521	3.8809639	5.6062571	4.1256541	7.1067793
SKIV2L2	NC_000005	54603576	54721409	4.913337	8.2434989	11.950612	16.929643
PPAP2A	NC_000005	54720682	54830873	6.6985213	6.4293592	6.5968896	3.5482142
LOC100288887	NC_000005	54786914	54830906	0.2450645	0.4199201	0.2906882	0.2276943
RNF138P1	NC_000005	54824670	54830370	0	0	0	0
LOC100132104	NC_000005	54847713	54860858	0	0	0	0
SLC38A9	NC_000005	54921676	55008163	0.9195643	0.8205434	1.7750573	1.4750237
DDX4	NC_000005	55033845	55112974	0.0305196	0	0.0135755	0.0531682
LOC402216	NC_000005	55134577	55135853	0	0	0	0
IL31RA	NC_000005	55147334	55212981	0.110192	0.0944074	0.0326766	0.0767862
IL6ST	NC_000005	55236694	55290763	92.539074	70.210639	116.36007	71.83754
ANKRD55	NC_000005	55395507	55529186	0	0.0507869	0.0292975	0.0458972
RPL17P22	NC_000005	55432771	55433388	0	0	0	0
LOC100128603	NC_000005	55488375	55489465	0	0	0	0
LOC345645	NC_000005	55571126	55572511	0	0.0362046	0	0
RNU6ATAC2P	NC_000005	55593392	55593516	0	0	0	0
LOC100289077	NC_000005	55760576	55762100	0.5475517	0.5036838	0.2563775	0.1405732
RPL26P19	NC_000005	55800419	55800941	0	0	0	0
MAP3K1	NC_000005	56110900	56191979	0.1462601	0.2345784	0.3851464	0.3669115
C5orf35	NC_000005	56205100	56213012	1.0450415	1.2776797	1.4071103	0.8463173
MIER3	NC_000005	56215429	56247954	1.3942431	1.8243635	2.2702297	2.0726701
LOC100130001	NC_000005	56271420	56272051	0	0	0	0
LOC100129574	NC_000005	56410024	56418452	0	0	0	0
GPBP1	NC_000005	56469775	56559412	7.7305061	6.6210581	17.014481	14.794955
LOC100288510	NC_000005	56613811	56615877	0	0	0	0
ACTBL2	NC_000005	56775843	56778636	0.0157295	0.1617158	0	0.0328828

LOC100130514	NC_000005	57457028	57498769	0	0	0	0
PLK2	NC_000005	57749809	57755913	15.616924	16.94783	9.8094754	10.014093
LOC100289379	NC_000005	57756088	57756766	0.160395	0	0.3567296	0.1676544
GAPT	NC_000005	57787330	57792185	0.0198322	0	0	0
RAB3C	NC_000005	57878939	58147406	0	0.0447804	0.0387488	0.0607034
RPL5P15	NC_000005	58118330	58119067	0	0	0	0
PDE4D	NC_000005	58264865	59189621	0.235493	0.3066747	0.9869831	0.5178296
LOC642781	NC_000005	58335035	58336614	0	0	0	0
LOC100132036	NC_000005	58824688	58825080	0	0	0	0
LOC653198	NC_000005	59283260	59818157	0	0	0	0
RPL31P8	NC_000005	59725690	59726129	0	0	0	0
LOC643106	NC_000005	59755673	59756566	0	0	0	0
PART1	NC_000005	59783540	59843484	0	0	0	0
DEPDC1B	NC_000005	59892739	59995993	1.5971602	2.3999171	6.3705299	6.2986471
LOC728167	NC_000005	60039388	60040795	0	0	0	0
ELOVL7	NC_000005	60047616	60140101	0.2491268	0.0349266	4.2814907	2.2015823
ERCC8	NC_000005	60169659	60240905	2.107107	3.6252827	3.1178587	4.5847421
GNL3LP	NC_000005	60187503	60189489	0	0	0	0
NDUFAF2	NC_000005	60240956	60448864	6.356907	9.1120908	18.216529	21.041293
C5orf43	NC_000005	60453536	60458302	3.6800942	4.5183402	4.8296993	7.2164506
ZSWIM6	NC_000005	60628100	60841999	5.2150092	3.4320665	5.5843516	4.9362651
RPL3P6	NC_000005	60686443	60687724	0	0	0	0
FLJ37543	NC_000005	60933636	61002362	0.0147974	0.0456398	0	0
LOC643307	NC_000005	61127771	61138634	0	0	0	0
KIF2A	NC_000005	61601989	61682210	5.7420458	7.651594	11.135824	16.588476
DIMT1L	NC_000005	61684351	61699728	4.8831369	8.4446821	7.9601338	6.1367772
IPO11	NC_000005	61708573	61924416	2.4473654	3.9985311	4.9789691	4.9906719
CKS1BP3	NC_000005	61807588	61808309	0	0	0	0
RPL35AP14	NC_000005	61871802	61872098	0	0	0	0
LRRC70	NC_000005	61874562	61877275	0	0	0	0
ISCA1L	NC_000005	62071197	62073170	0	0.0240209	0	0.0325623
HTR1A	NC_000005	63256278	63257546	0.0346322	0	0	0
RNF180	NC_000005	63461671	63668696	0.1910793	0.2053791	0.4713343	0.2541986
RGS7BP	NC_000005	63802452	63908126	0.0115018	0.0709501	0.0204646	0.0080149
MRPL49P1	NC_000005	63970681	63971217	0	0	0	0
FAM159B	NC_000005	63986135	64020577	0.1401299	0.2401137	0.2649098	0.2644635
SFRS12IP1	NC_000005	64013975	64064496	1.243576	1.2917075	2.3095849	2.6265211
SDCCAG10	NC_000005	64064755	64314590	2.2819274	2.2808929	3.1202865	3.7839393
ADAMTS6	NC_000005	64444563	64777704	0.4656045	1.0008981	0.4680088	1.2430296
RPS2P23	NC_000005	64711160	64712084	0	0	0	0
CENPK	NC_000005	64813593	64858995	2.7927819	4.2808076	6.4146208	9.9429125
PPWD1	NC_000005	64859131	64883373	1.571076	3.101244	2.9817019	4.4959307
TRIM23	NC_000005	64885507	64920187	1.7579293	2.7573461	3.7694063	3.9419715
C5orf44	NC_000005	64920558	64961954	3.0592388	2.6064547	4.8257711	3.1286131
SGTB	NC_000005	64961755	65017941	4.1183837	5.7090343	4.0151544	4.1615688
NLN	NC_000005	65018085	65119393	4.972969	5.6302816	7.2739657	8.7590766
ERBB2IP	NC_000005	65222384	65376850	11.374687	10.564135	10.107931	16.295482
SFRS12	NC_000005	65440085	65476716	0.7638062	0.583921	0.9234274	2.3814801
FLJ46010	NC_000005	65498722	65645893	0	0	0	0
LOC202227	NC_000005	65867887	65868380	0	0	0	0
MAST4	NC_000005	65892176	66463092	0.9844486	1.2271258	1.1389843	0.9845742
LOC100129571	NC_000005	65892308	65893621	0	0.0434038	0	0.0588374
CD180	NC_000005	66478103	66492617	0.0485437	0.033272	0	0.0112757
RPL21P55	NC_000005	66904441	66904988	0	0	0	0
LOC359819	NC_000005	66985788	66986267	0	0	0	0
EEF1B3	NC_000005	67455026	67455886	0	0	0	0
PIK3R1	NC_000005	67522462	67597649	1.7559008	1.6683904	1.3759997	0.8922869
LOC100288757	NC_000005	68364749	68365033	0	0	0	0
LOC100130639	NC_000005	68370543	68371507	0	0	0	0
SLC30A5	NC_000005	68389818	68425880	5.8684936	7.6121549	8.3553573	9.1658967
CCNB1	NC_000005	68462913	68474072	35.843111	70.160562	61.084109	63.517724
CENPH	NC_000005	68485375	68506184	4.9042303	7.2540667	10.583646	7.871189
MRPS36	NC_000005	68513573	68525985	3.4722079	4.0117693	3.5302542	4.8391456
CDK7	NC_000005	68530622	68573257	9.3574679	12.241515	11.623601	15.302345
CCDC125	NC_000005	68576519	68616410	0.8872292	0.7997062	0.9839266	1.0417201
CFLP5	NC_000005	68608686	68609780	0	0	0	0

LOC728324	NC_000005	68629703	68630454	0	0	0	0
TAF9	NC_000005	68647553	68665840	13.137009	21.957377	19.743004	18.189723
RAD17	NC_000005	68665124	68710628	1.420474	1.7143787	3.4724089	4.1315181
MARVELD2	NC_000005	68710943	68739836	0.4969021	0.0283815	0.8718364	0.7021407
RPS27P14	NC_000005	68765679	68766014	0	0	0	0
OCLN	NC_000005	68788119	68850133	1.0820587	0.6332522	3.7912791	2.3896685
GTF2H2C	NC_000005	68856051	68888730	1.168614	0.7938205	0.7982884	0.6689195
GTF2H2D	NC_000005	68856074	68888352	7.3368928	4.5860025	2.1034187	3.1762908
LOC643784	NC_000005	68905253	68919473	0	0	0	0
LOC728488	NC_000005	68927847	68928572	0	0	0	0
LOC653188	NC_000005	68935290	68942015	0	0	0	0
LOC100286984	NC_000005	68945521	68946687	0	0	0	0
LOC728499	NC_000005	68949135	68949824	0	0	0	0
LOC100287046	NC_000005	69140636	69212152	0.7027393	1.3894035	2.2121624	3.5785528
LOC728506	NC_000005	69198283	69198972	0	0	0	0
LOC100287154	NC_000005	69201418	69202582	0	0	0	0
LOC100288852	NC_000005	69216385	69218610	0.0789726	0.0608941	0.0526921	0.0275156
CDH12P	NC_000005	69281026	69281327	0	0	0	0
SERF1B	NC_000005	69321096	69338940	0.1163267	0.4066267	0.8899922	1.0862186
SMN2	NC_000005	69345350	69373422	1.3497614	1.2766809	1.6090521	2.4830982
LOC100287312	NC_000005	69386778	69388151	0.0959568	0.0657691	0.0284553	0.1783108
LOC728519	NC_000005	69388865	69424264	0	0	0	0
LOC100093624	NC_000005	69428502	69428803	0	0	0	0
LOC100288916	NC_000005	69491166	69493394	0.062873	0.0215467	0.037289	0.0146041
LOC100287078	NC_000005	69497625	69503697	0.0683487	0.6324271	0.3648295	0.428653
LOC100287280	NC_000005	69507212	69508378	0	0	0	0
LOC643367	NC_000005	69509583	69513906	0	0	0	0
SMA4	NC_000005	69515011	69521622	0	0	0	0
LOC100287248	NC_000005	69525262	69526428	0	0	0	0
LOC728526	NC_000005	69528876	69529565	0	0	0	0
GTF2H2B	NC_000005	69711216	69743885	2.8414805	2.4885494	7.2090818	1.9800568
LOC728535	NC_000005	69754439	69774408	0	0	0	0
SMA5	NC_000005	69776843	69852701	0	0	0	0
LOC441081	NC_000005	69783486	69784211	0	0	0	0
LOC100287220	NC_000005	69800353	69801519	0	0	0	0
LOC340089	NC_000005	69803963	69804652	0	0	0	0
LOC100287185	NC_000005	69821638	69822804	0	0	0	0
LOC728540	NC_000005	69825252	69825941	0	0	0	0
LOC100133280	NC_000005	70068521	70075280	0	0	0	0
LOC100287339	NC_000005	70076484	70077648	0	0	0	0.0262874
LOC653080	NC_000005	70081224	70094525	0	0	0	0
LOC100093625	NC_000005	70156009	70156510	0	0	0	0
SERF1A	NC_000005	70196490	70214357	1.5306306	3.3046633	1.3616914	3.0798201
SMN1	NC_000005	70220768	70248839	0.5949914	1.3624534	1.780443	1.5265324
LOC100287378	NC_000005	70262225	70263598	0	0.0657691	0.0284553	0.0891554
NAIP	NC_000005	70264310	70320941	0.1677157	0.1586351	0.3282501	0.7199254
GTF2H2	NC_000005	70330951	70363497	1.7345022	2.9875238	1.7835384	4.2068003
LOC647859	NC_000005	70370030	70388897	0	0	0	0
LOC100287529	NC_000005	70395003	70396371	0	0.099014	0	0.089481
LOC653406	NC_000005	70397083	70424583	0	0	0	0
LOC100170939	NC_000005	70423617	70503957	0	0	0	0
LOC100093626	NC_000005	70428816	70429117	0	0	0	0
LOC728565	NC_000005	70435842	70528771	0	0	0	0
LOC100287490	NC_000005	70507576	70508742	0.0376592	0	0	0.0787272
LOC728452	NC_000005	70511190	70511879	0	0	0	0
LOC100287454	NC_000005	70525601	70527286	0.0260666	0	0.0231895	0.0726569
LOC728575	NC_000005	70529211	70529900	0	0	0	0
PMCHL2	NC_000005	70671612	70681820	0	0	0	0
BDP1	NC_000005	70751442	70863649	0.3769826	0.399817	0.4342213	1.2858301
MCCC2	NC_000005	70883115	70954531	2.4282774	2.8039781	5.4485448	6.3016897
CARTPT	NC_000005	71014994	71016872	0	0	0	0
MAP1B	NC_000005	71403118	71505397	5.2756956	7.9058695	5.9630397	16.699366
MRPS27	NC_000005	71515236	71616084	11.062122	13.095225	19.921341	24.887531
PTCD2	NC_000005	71616200	71655180	2.2360727	2.5352551	2.8258821	2.6066825
RPL10AP8	NC_000005	71669046	71678220	0	0	0	0
LOC100128169	NC_000005	71711217	71714164	0	0	0	0

ZNF366	NC_000005	71739234	71803249	0.0158658	0	0.0564586	0.0110559
LOC389300	NC_000005	71869941	71956496	0.1464941	0.225917	0.0651626	0.1020829
RPL7P22	NC_000005	72021244	72021981	0	0	0	0
TNPO1	NC_000005	72112418	72210215	15.710938	18.025625	21.699418	22.328589
RPL35AP13	NC_000005	72174651	72175044	0	0	0	0
FCHO2	NC_000005	72251899	72386349	2.8759579	3.4557448	3.3180961	4.8974754
TMEM171	NC_000005	72416402	72427641	4.5039021	1.7726074	4.2572209	3.1630179
LOC100130129	NC_000005	72448237	72449563	0	0	0	0
TMEM174	NC_000005	72469023	72470970	0.024999	0	0	0.0348406
LOC100287621	NC_000005	72469092	72470943	0	0	0	0
FOXD1	NC_000005	72742083	72744352	7.7248214	9.3352491	5.132632	6.7860413
BTF3	NC_000005	72794250	72801448	79.064593	96.408139	135.76152	185.90699
FUNDC2P	NC_000005	72804434	72805540	0	0	0	0
ANKRA2	NC_000005	72848163	72861498	2.2113668	2.4501014	3.8581867	2.6031895
UTP15	NC_000005	72861598	72877794	2.1282728	3.3624857	2.588676	3.1420874
RGNEF	NC_000005	72921983	73207528	3.4475764	2.6239409	6.1787851	15.055871
LOC100289045	NC_000005	73240861	73249005	0	0	0	0
ENC1	NC_000005	73923234	73937249	1.625057	2.2194493	1.2756139	2.775501
HEXB	NC_000005	73980969	74017113	39.994972	25.859832	79.223473	32.735272
GFM2	NC_000005	74017031	74063042	4.8588728	5.3883312	7.2245502	9.215996
TINP1	NC_000005	74063103	74072734	0.9553963	0.9413209	2.1602822	2.4133736
FAM169A	NC_000005	74073399	74162615	0.3180367	0.7908236	0.1908161	0.1855429
LOC260421	NC_000005	74094719	74096231	0	0	0	0
LOC441086	NC_000005	74148026	74163100	0	0	0.022002	0
LOC100131411	NC_000005	74225705	74226357	0	0	0	0
RPL39P21	NC_000005	74227433	74227835	0	0	0	0
RPL27AP5	NC_000005	74286014	74286462	0	0	0	0
GCNT4	NC_000005	74323289	74326724	0.7034787	0.56545	0.5689401	0.2852142
LOC100130128	NC_000005	74343544	74349672	5.0432397	6.501801	1.4955353	1.5619246
ANKRD31	NC_000005	74364217	74532703	0.1405515	0.0684482	0.0460668	0.0876322
HMGCR	NC_000005	74632993	74657926	6.4263019	7.1394114	7.8331657	7.3788445
COL4A3BP	NC_000005	74666928	74807806	1.6193101	1.5361392	1.642404	2.0932633
RPS26P26	NC_000005	74670288	74670634	0	0	0	0
POLK	NC_000005	74807657	74895646	1.3636419	1.2317284	2.1663102	1.8869052
ANKDD1B	NC_000005	74907327	74965449	0.0673708	0.0692643	0.0599349	0.0312978
C5orf37	NC_000005	74970023	75013313	1.1957369	2.0757763	2.1100827	2.2401784
SLC25A5P9	NC_000005	75048115	75049364	0	0	0	0
LOC391798	NC_000005	75127071	75162009	0	0	0	0
LOC644601	NC_000005	75202523	75207696	0	0	0	0
LOC100288946	NC_000005	75216491	75240123	0	0	0	0
LOC100132039	NC_000005	75374464	75375593	0	0	0	0
SV2C	NC_000005	75379305	75621416	0	0.0382586	0.0165527	0
RAP1BL	NC_000005	75465911	75470171	0.0453542	0.0466289	0.0201742	0.0158023
HMGN2L4	NC_000005	75537026	75538230	0	0	0	0
LOC100129566	NC_000005	75672500	75672967	0	0	0.1670836	0
IQGAP2	NC_000005	75699149	76003957	0.030472	0.1331458	0.1965383	0.4565331
F2RL2	NC_000005	75911307	75919240	0.0386641	0.1457529	0	0.0269427
F2R	NC_000005	76011868	76031595	2.8722073	30.916832	21.036748	34.450979
LOC100287744	NC_000005	76114802	76131140	0.1505592	4.040037	0.1339416	0.1993397
F2RL1	NC_000005	76114833	76131140	0.0152439	2.7896793	0.0135614	0.0956032
S100Z	NC_000005	76145826	76217056	0.0383158	0	0.0681736	0
CRHBP	NC_000005	76248680	76265299	0	0.3441608	0.0425436	0
AGGF1	NC_000005	76326232	76361038	0.7380469	1.1837121	1.5670542	1.9611991
ZBED3	NC_000005	76372532	76383030	0.4955403	1.1781437	0.8541399	1.3380849
SNORA47	NC_000005	76376259	76376396	0	0	0	0
LOC728723	NC_000005	76382623	76444176	0	0	0	0
PDE8B	NC_000005	76506706	76723243	0.0616039	0.0506682	0.0657655	0.0600993
ALDH7A1P1	NC_000005	76586009	76587819	0	0	0	0
WDR41	NC_000005	76728069	76788332	4.930861	7.9132776	7.3670941	5.3515718
RPS2P24	NC_000005	76841196	76842138	0	0	0	0
RPL7P23	NC_000005	76878179	76879025	0	0	0	0
OTP	NC_000005	76924537	76934522	0	0.0337441	0	0
TBCA	NC_000005	76986995	77072185	0.1941748	0.2661762	1.151622	1.3530874
ACTBP2	NC_000005	77080627	77082421	0	0	0	0
AP3B1	NC_000005	77298150	77590528	5.3915417	6.6879596	6.385478	11.947237
TRNAQ33P	NC_000005	77318776	77318848	0	0	0	0

LOC728769	NC_000005	77654588	77656363	0	0	0	0
SCAMP1	NC_000005	77656339	77776562	4.068238	4.5516271	8.0587071	6.2632879
LHFPL2	NC_000005	77781038	77944648	53.739671	14.69692	9.8422019	20.614883
ARSB	NC_000005	78073032	78282357	4.3487696	2.6514811	2.2065542	4.0756762
DMGDH	NC_000005	78293429	78365449	0.3706306	0.3370802	0.1268166	0.0397339
BHMT2	NC_000005	78365583	78385289	2.0364652	1.665955	0.4480538	0.1983674
BHMT	NC_000005	78407604	78428113	0.2461101	0.0722934	0.2658634	0.01225
LOC100289109	NC_000005	78523369	78523905	0	0	0.0728074	0.0570296
JMY	NC_000005	78531954	78623036	0.5651729	0.4370344	1.740439	1.8345305
RPS3AP20	NC_000005	78579911	78580769	0	0	0	0
HOMER1	NC_000005	78669786	78809700	1.2827794	1.3617209	0.7700755	0.7776132
RPL29P15	NC_000005	78806205	78806840	0	0	0	0
RPL7AP32	NC_000005	78825104	78825974	0	0	0	0
PAPD4	NC_000005	78908243	78982471	3.4929052	4.2323368	6.4367267	4.3377276
CMYA5	NC_000005	78985659	79096049	0.0784059	0.1121524	0.0818829	0.0641384
LOC100132835	NC_000005	79145372	79145765	0	0	0	0
MTX3	NC_000005	79272539	79287088	0.5760797	0.8371515	1.5621286	2.0959552
THBS4	NC_000005	79330991	79379110	0.2488872	0.2424147	0.1398423	0.1186657
SERINC5	NC_000005	79407474	79551870	5.426806	7.3781264	5.03889	11.28285
LOC100128156	NC_000005	79424184	79439706	0	0.0680473	0	0.0461218
KRT18P45	NC_000005	79584219	79585660	0	0	0	0
LOC644936	NC_000005	79594904	79596297	0	0	0	0
RPL39P20	NC_000005	79611441	79611837	0	0	0	0
SPZ1	NC_000005	79615843	79617661	0	0	0	0.0168361
LOC391804	NC_000005	79627302	79628360	0	0	0	0
LOC441089	NC_000005	79646424	79647785	0	0	0	0
LOC644037	NC_000005	79654183	79655914	0	0	0	0
ZFYVE16	NC_000005	79703838	79775498	3.8752801	4.121797	3.8480577	3.2346044
FAM151B	NC_000005	79783800	79838206	0.4024037	0.2955095	0.8438324	0.5007338
RPS27AP9	NC_000005	79794349	79794791	0	0	0	0
RPL7P24	NC_000005	79796131	79797287	0	0	0	0
ANKRD34B	NC_000005	79852574	79866098	0.0353566	0.0121168	0.0104847	0.0246379
DBIL1	NC_000005	79898882	79899551	0	0	0	0
DHFR	NC_000005	79922045	79950800	7.090958	10.773882	18.485752	35.550503
MSH3	NC_000005	79950294	80172634	2.5493779	3.0806865	3.2242798	5.5946747
RPS26P27	NC_000005	80151324	80151658	0	0	0	0
RASGRF2	NC_000005	80256558	80521953	0.0107769	0.1107979	0.0479372	0.0450587
LOC100129411	NC_000005	80409204	80493506	0	0.0357464	0	0.0242285
LOC100287929	NC_000005	80527915	80529859	0	0.1161527	0.2412189	0.3306542
CKMT2	NC_000005	80529139	80562217	0.0267	0.0823513	0.1662715	0.1860564
ZCCHC9	NC_000005	80597402	80608966	0.8097664	0.5802447	2.0301919	1.5902366
ACOT12	NC_000005	80625947	80689988	0.0220956	0.1135832	0	0
SSBP2	NC_000005	80715672	81047072	4.647503	2.4586451	10.71771	9.6999743
SHFM1P	NC_000005	81188269	81188710	0	0	0	0
ATG10	NC_000005	81267844	81551213	0.8524832	1.6782939	2.6947146	2.6289619
PPIAP11	NC_000005	81305169	81306048	0	0	0	0
RPS23	NC_000005	81569139	81574235	42.993412	82.090906	97.448972	95.66347
ATP6AP1L	NC_000005	81601166	81614147	0.289896	0.1192174	0.8768583	0.4444244
RPL5P16	NC_000005	82073616	82074412	0	0	0	0
LOC100127911	NC_000005	82264709	82272972	0	0	0	0
TMEM167A	NC_000005	82351986	82373179	11.389823	14.483341	14.732416	13.785111
SCARNA18	NC_000005	82360023	82360156	0	0	0	0
XRCC4	NC_000005	82373317	82649579	1.2637696	2.1478026	3.0286847	3.6124416
RPL13P9	NC_000005	82497048	82497580	0	0	0	0
VCAN	NC_000005	82767530	82877800	13.796472	40.431594	2.2180256	2.6949486
HAPLN1	NC_000005	82934017	83016896	5.7213495	14.632932	0.0083578	0.0392795
RPL13AP14	NC_000005	83042207	83042808	0	0	0	0
EDIL3	NC_000005	83238126	83680611	2.5565044	2.9474042	0.4995654	1.6476061
LOC100289244	NC_000005	84295667	84300695	0	0	0	0
LOC391807	NC_000005	84505856	84512641	0	0	0	0
RPL5P17	NC_000005	84760177	84761190	0	0	0	0
LOC645181	NC_000005	84845228	84845910	0	0	0	0
RPS2P25	NC_000005	85058317	85059084	0	0	0	0
LOC100129564	NC_000005	85372529	85383840	0	0	0	0
NBPF22P	NC_000005	85578262	85593362	0	0	0	0
COX7C	NC_000005	85913784	85916583	17.816851	22.203146	77.81088	62.453683

RPL10AP9	NC_000005	86180027	86180732	0	0	0	0
RASA1	NC_000005	86564151	86687733	5.8967238	7.0158631	7.5146239	10.03035
CCNH	NC_000005	86690082	86708836	7.7180494	8.9842178	16.626396	23.713168
LOC100289347	NC_000005	87434931	87441180	0	0.0482557	0	0.0327072
TMEM161B	NC_000005	87491023	87564665	2.7954735	2.1947219	4.9587892	4.4272665
RPS3AP22	NC_000005	87677726	87678588	0	0	0	0
LOC645323	NC_000005	87836597	87980620	0	0	0	0
MEF2C	NC_000005	88014058	88199869	0.5855475	0.9261605	0.457951	0.4080328
LOC100288032	NC_000005	88119548	88179555	0.2671625	0.4120067	0.2376752	0.0310282
CETN3	NC_000005	89689528	89705603	1.1635988	2.5306386	3.3842088	4.6467483
LOC731157	NC_000005	89705803	89706483	0	0	0	0
MBLAC2	NC_000005	89754020	89770585	0.5852919	1.0134595	1.3245671	1.7029722
POLR3G	NC_000005	89770681	89810369	0.9097351	1.4029549	2.1304305	2.628985
LYSMD3	NC_000005	89811444	89825401	3.321129	4.0825181	6.1201775	7.5034905
GPR98	NC_000005	89854617	90460033	0.020459	0.0233711	0.0182009	0.0491063
LOC729040	NC_000005	90575914	90576959	0	0	0	0
RPS18P7	NC_000005	90650257	90650788	0	0	0	0
ARRDC3	NC_000005	90664541	90679149	8.4178661	7.3296795	29.156392	10.142053
LOC100129716	NC_000005	90676196	90734901	0	0	0	0
LOC133789	NC_000005	90765676	90772882	0	0	0	0
LOC100131236	NC_000005	91139501	91171691	0	0	0	0
LOC100129283	NC_000005	92176492	92177356	0	0	0	0
LOC100288772	NC_000005	92223401	92226547	0	0	0	0
CCT7P2	NC_000005	92250408	92305070	0	0	0	0
LOC391811	NC_000005	92602987	92604293	0	0	0	0
FLJ42709	NC_000005	92745065	92917003	0	0	0	0
NR2F1	NC_000005	92919043	92929788	1.9782886	0.6355902	1.14885	0.7275682
LOC100129010	NC_000005	92923700	92929650	0.5242334	0.538967	0.2331863	0.0608844
FAM172A	NC_000005	92953775	93447389	0.8969027	1.0049966	1.6854718	3.3637507
POU5F2	NC_000005	93076015	93077309	0	0	0	0
C5orf36	NC_000005	93486556	93954309	0.2071026	0.3494125	0.1842442	0.2775333
LOC100288834	NC_000005	93900198	93904645	0	0	0	0
LOC100288871	NC_000005	93905245	93906432	0	0	0	0
LOC100131573	NC_000005	93917897	93929330	0	0	0	0.0336907
ANKRD32	NC_000005	93954391	94031573	1.406159	2.3344165	2.5736924	3.3090611
MCTP1	NC_000005	94042284	94620279	1.6633995	0.9802077	2.3550576	3.0957066
RPL7P18	NC_000005	94161595	94162421	0	0	0	0
FAM81B	NC_000005	94727048	94786144	0	0.0289081	0	0.0195937
TTC37	NC_000005	94800112	94890682	14.008286	14.99697	24.273198	31.360255
ARSK	NC_000005	94890825	94940806	1.1036327	1.5668982	2.4779324	2.1149019
GPR150	NC_000005	94955980	94957284	0.2357376	0.2077398	0.2995982	0.070402
RFESD	NC_000005	94982481	94992849	0.0928157	0.333985	0.137619	0.1077961
SPATA9	NC_000005	94994019	95018714	0	0.1102931	0.063625	0.0249185
RHOBTB3	NC_000005	95066850	95132071	6.9246495	7.1842096	6.0409462	6.7566206
GLRX	NC_000005	95149553	95158577	31.140004	19.834608	47.050015	29.955482
LOC727938	NC_000005	95169746	95170750	0	0	0	0
C5orf27	NC_000005	95187936	95195837	0	0	0	0
ELL2	NC_000005	95220802	95297775	22.148571	15.200635	29.966609	36.591816
LOC100288964	NC_000005	95308745	95309153	0	0	0	0
PCSK1	NC_000005	95726119	95768952	1.2869684	1.689684	0.1315114	0.1211907
CAST	NC_000005	95997941	96110387	8.5061094	11.587356	12.107633	23.567573
ERAP1	NC_000005	96096514	96143892	6.8171298	4.0884235	17.402168	9.1539981
ERAP2	NC_000005	96211644	96255407	13.444715	8.3602394	14.440886	28.219445
LOC100289002	NC_000005	96270571	96271779	0.5156898	0.4123648	0.4077973	0.4791376
LNPEP	NC_000005	96271346	96365115	4.4360676	4.1932459	8.4636649	7.900516
RPS20P16	NC_000005	96278598	96278893	0	0	0	0
LOC642737	NC_000005	96392102	96393062	0	0	0	0
LIX1	NC_000005	96427574	96478520	0.0221569	0.0113898	0.0098557	0.084919
RIOK2	NC_000005	96498641	96518944	6.8609139	8.1142681	7.2134155	7.589353
YTHDF1P	NC_000005	96704291	96706323	0	0	0	0
LOC100289037	NC_000005	96783756	96785445	0	0	0	0
LOC100289066	NC_000005	97013251	97014385	0	0	0	0
LOC391813	NC_000005	97073598	97074616	0	0	0	0
PSME2P1	NC_000005	97549090	97549869	0	0	0	0
LOC402221	NC_000005	97674347	97675651	0	0	0	0
LOC100289133	NC_000005	97727774	97742089	0	0	0	0

MRPS35P2	NC_000005	97738247	97738425	0	0	0	0
LOC100289160	NC_000005	97745610	97745804	0	0	0.2005003	0
LOC642909	NC_000005	97904212	97913487	0	0	0	0
DDX18P4	NC_000005	98015006	98017575	0	0	0	0
RGMB	NC_000005	98104999	98132198	47.680953	16.435709	38.150005	66.024028
CHD1	NC_000005	98190908	98262238	0.5649223	0.9516715	1.089912	1.9208728
LOC100289230	NC_000005	98262616	98266713	0.1960383	0.1465804	0.269529	0.5588481
RPS9P3	NC_000005	98290073	98290713	0	0	0	0
LOC728093	NC_000005	98858006	98861727	0	0	0	0
LOC441066	NC_000005	98861792	98870230	0	0	0	0
LOC100287683	NC_000005	98863560	98864725	0.0376915	0.1550031	0.1005941	0.0262649
LOC285706	NC_000005	99283430	99285204	0	0	0	0
LOC643031	NC_000005	99382678	99384483	0	0	0	0
LOC100287717	NC_000005	99385414	99385662	0	0	0	0
LOC100289371	NC_000005	99387159	99387577	0	0	0	0
LOC100287745	NC_000005	99387621	99388301	0	0	0	0
LOC100289404	NC_000005	99389049	99389243	0.6761266	0	0	0.471152
LOC100133050	NC_000005	99715209	99723952	0	0	0	0
LOC100287777	NC_000005	99721018	99722184	0	0	0	0
LOC441098	NC_000005	99724017	99728594	0	0	0	0
FAM174A	NC_000005	99871124	99922440	2.2756366	2.7051551	2.8785425	3.0476214
ST8SIA4	NC_000005	100142639	100238987	1.7694065	3.5337944	2.6323851	33.765678
OR7H2P	NC_000005	101151587	101152484	0	0	0	0
SLCO4C1	NC_000005	101569690	101632253	0.0247178	0.0423541	0.0952884	0.0172243
SLCO6A1	NC_000005	101707651	101834720	0.0174121	0.0179015	0.0154903	0.0121335
PAM	NC_000005	102201527	102365417	21.907291	24.332167	37.03667	48.358747
LOC134505	NC_000005	102368080	102368735	0	0	0.0708289	0.6102784
GIN1	NC_000005	102421704	102455842	0.4210312	0.4328644	1.2668977	1.3116319
HISPPD1	NC_000005	102465257	102538910	4.0611129	4.9452767	3.383372	6.8718956
C5orf30	NC_000005	102594442	102614361	6.0885092	6.815038	3.0005109	5.2601535
LOC100129962	NC_000005	102766223	102873671	0	0	0	0
NUDT12	NC_000005	102884556	102898490	1.2599837	1.1140403	1.3338906	2.3969587
RAB9P1	NC_000005	104435175	104435799	0	0	0	0
LOC345571	NC_000005	105751274	105857631	0	0	0	0
LOC100289569	NC_000005	105878764	105881599	0	0	0	0
LOC100287833	NC_000005	106530890	106531069	1.2207842	2.2591701	1.0860435	0.8506911
EFNA5	NC_000005	106712590	107006596	0.8484851	0.8808011	1.1725605	2.7151954
LOC345576	NC_000005	107060498	107085121	0	0	0	0
FBXL17	NC_000005	107195449	107717111	2.9314775	2.2142696	5.0774677	3.4185135
RPS20P3	NC_000005	107929185	107929701	0	0	0	0
LOC100289641	NC_000005	108063252	108083684	0.1768541	0.2272807	0.3146685	0.1848584
FER	NC_000005	108083523	108523373	1.7132362	3.6299886	3.5916747	5.7823923
GJA1P1	NC_000005	108385283	108388357	0	0	0	0
PJA2	NC_000005	108670410	108745675	13.268413	12.945337	21.126657	20.819131
LOC100287867	NC_000005	108784047	108784220	0	0	0	0
KRT18P42	NC_000005	108923990	108925361	0	0	0	0
MAN2A1	NC_000005	109025156	109203429	6.1020166	7.2691705	7.8073142	5.8705646
LOC100289673	NC_000005	109218883	109221262	0	0	0	0
FLJ43080	NC_000005	109755812	110062381	0.2332502	0.0799353	0.1902137	0.1219035
SLC25A46	NC_000005	110074754	110098484	9.3526977	10.994757	14.338015	18.195865
LOC728366	NC_000005	110281972	110286725	0	0	0	0
LOC100131280	NC_000005	110319229	110328856	0	0	0	0
TSLP	NC_000005	110407390	110413722	1.2503053	0.3374294	0.8203259	0.6098838
WDR36	NC_000005	110427870	110466200	3.7334663	4.3661752	6.8800328	7.4192856
LOC100286926	NC_000005	110427911	110430907	0.0622496	0.0639992	0	0.043378
LOC100129099	NC_000005	110490942	110491665	0	0	0	0
RPS3AP21	NC_000005	110527883	110528765	0	0	0	0
CAMK4	NC_000005	110560082	110820749	0.417164	0.7934437	1.3731466	1.3953433
STARD4	NC_000005	110834022	110848157	6.2117642	4.3506986	10.9487	6.87166
C5orf13	NC_000005	111065000	111312628	11.618943	34.285293	2.8910172	6.1105978
C5orf26	NC_000005	111496548	111498004	0	0	0	0
SNORA13	NC_000005	111497182	111497314	0	0	0	0
EPB41L4A	NC_000005	111498315	111755010	0.7749529	1.0847088	0.5980507	0.214706
FLJ11235	NC_000005	111755286	111756941	0	0	0	0
LOC100286958	NC_000005	111882670	112022655	0	0	0	0
APC	NC_000005	112043218	112181936	2.6918292	3.3806867	4.4892383	9.0343391

SRP19	NC_000005	112196993	112203604	3.8194287	5.0997068	6.7957394	5.3230601
REEP5	NC_000005	112212081	112258031	13.229603	14.094437	25.797367	23.145568
XBPP1	NC_000005	112220737	112222477	0	0	0	0
ZRSR1	NC_000005	112227370	112228794	0	0	0	0
DCP2	NC_000005	112312433	112356667	0.849787	1.1059757	1.8441011	2.3823533
LOC100287967	NC_000005	112312512	112321678	0.2738208	0.9853078	0.6089963	0.3816184
MCC	NC_000005	112357796	112824527	0.8205314	0.814335	0.9578186	0.7205076
TSSK1B	NC_000005	112768675	112770666	0	0	0	0
YTHDC2	NC_000005	112849410	112930981	2.5099292	4.0468112	4.4284866	6.2652869
KCNN2	NC_000005	113698016	113832197	0.0452297	0.0310006	0.0402376	0.0315179
TRIM36	NC_000005	114460459	114516243	0.2874782	0.3786835	0.67134	0.1878059
LOC728254	NC_000005	114500897	114504749	0	0	0	0
PGGT1B	NC_000005	114546527	114598569	0.9830738	1.0107032	0.6595116	1.0107221
RPS2P26	NC_000005	114600028	114600734	0	0	0	0
CCDC112	NC_000005	114602885	114632458	0.1680329	0.2983958	0.1494867	0.4151443
CTNNAP1	NC_000005	114724725	114728138	0	0	0	0
LOC100129959	NC_000005	114731775	114738664	0	0	0	0
LOC402226	NC_000005	114847774	114849394	0	0	0	0
FEM1C	NC_000005	114856608	114880591	7.0991553	6.0161626	10.499105	11.57455
TICAM2	NC_000005	114914339	114961870	6.5782772	7.7364566	8.9402884	7.4342082
TMED7	NC_000005	114949203	114961870	1.3679019	1.7909888	1.7993825	0.5540015
CDO1	NC_000005	115140430	115152405	1.3614694	0	0.1211201	0.0758981
ATG12	NC_000005	115165815	115177499	8.0481713	6.4739253	15.629082	13.384591
LOC100287022	NC_000005	115173348	115177562	0.0737386	0.3790554	0.6559994	0.3083041
AP3S1	NC_000005	115177619	115249778	23.457241	16.439888	55.840666	38.845365
LOC100288062	NC_000005	115290594	115293236	0.0166282	0.034191	0.0295857	0.0231743
LVRN	NC_000005	115298151	115363299	0.0665738	0.0293335	0.0592259	0.1126646
LOC644100	NC_000005	115387163	115394644	0.0957478	0	1.0221586	0.2001626
RPS25P6	NC_000005	115387553	115388039	0	0	0	0
COMMD10	NC_000005	115420727	115628978	3.2714274	5.9969543	5.1617573	6.2798205
SEMA6A	NC_000005	115779251	115910551	0.0064064	0.013173	0.0113987	0.1116067
LOC100128691	NC_000005	115783249	115785557	0	0	0	0
RPS14P8	NC_000005	115898217	115898760	0	0	0	0
RPS17P2	NC_000005	116051914	116052392	0	0	0	0
LOC100287135	NC_000005	116355238	116355375	0	0	0.2833157	0
RPL35AP15	NC_000005	116636803	116637138	0	0	0	0
LOC100129526	NC_000005	117054971	117072662	0	0	0	0
LOC100130968	NC_000005	117114521	117115285	0	0	0	0
RNU7-34P	NC_000005	118093907	118093968	0	0	0	0
DTWD2	NC_000005	118172569	118324240	0.1855474	0.1907623	0.2672532	0.2955356
PTMAP2	NC_000005	118309257	118310515	0	0	0	0
LOC100128407	NC_000005	118345509	118348451	0	0	0	0
LOC100127986	NC_000005	118347037	118347833	0	0	0	0
DMXL1	NC_000005	118407084	118584822	2.3797261	2.7296873	4.5980669	4.1964279
TNFAIP8	NC_000005	118604418	118730294	3.0302205	3.197912	6.0342362	2.8107765
HSD17B4	NC_000005	118788148	118878027	6.9573735	6.1552239	11.749755	12.286628
RPL21P58	NC_000005	118882794	118883340	0	0	0	0
FABP5L6	NC_000005	118890943	118891353	0	0	0	0
FAM170A	NC_000005	118965293	118971517	0.0328954	0	0	0.0229228
LOC348958	NC_000005	119015867	119017703	0	0	0	0
PRR16	NC_000005	119800019	120022965	6.6022004	3.8165118	3.7014135	10.190932
LOC100287194	NC_000005	119801325	120022120	4.3709815	2.696297	4.2420505	3.2673921
LOC728682	NC_000005	120315174	120379784	0	0	0	0
RPL23AP44	NC_000005	120911543	120911752	0	0	0	0
RPL18P3	NC_000005	120951910	120952559	0	0	0	0
LOC100130699	NC_000005	121007068	121007567	0	0	0	0
FTMT	NC_000005	121187650	121188528	0	0	0.0444796	0.0696812
SRFBP1	NC_000005	121297656	121364295	0.9142968	0.7647402	1.668126	2.1921193
LOX	NC_000005	121400161	121414055	126.56776	143.11548	39.167633	42.573756
LOC100288137	NC_000005	121413320	121414057	0.2136867	0.366154	0.1267344	0
ZNF474	NC_000005	121465215	121489266	0.0449139	0.0923524	0.0599349	0.0156489
LOC728460	NC_000005	121492359	121518358	0	0.0660576	0	0.1343196
SNCAIP	NC_000005	121647820	121799794	0.1108249	0.1898994	0.0438191	0.042904
ARGFXP1	NC_000005	122011295	122016359	0	0	0	0
LOC100286947	NC_000005	122110339	122131077	0.1121128	0	0	0.1562494
SNX2	NC_000005	122110750	122165803	15.739797	13.517895	28.00512	36.308761

SNX24	NC_000005	122181160	122344902	1.0282693	1.4588932	3.091946	2.221271
LOC100130882	NC_000005	122346937	122390362	3.0105572	2.9434453	3.5972911	1.8510672
PPIC	NC_000005	122359078	122372425	4.9423982	5.337198	2.9418071	1.7344187
PRDM6	NC_000005	122424841	122523745	0.0725937	0.0447804	0.0774977	0.040469
CEP120	NC_000005	122680579	122759252	0.5450069	0.4830383	1.487998	1.833433
CSNK1G3	NC_000005	122847793	122952465	5.2497552	5.9846236	7.5969497	7.3507944
KRT18P16	NC_000005	122971757	122973161	0	0	0	0
ZNF608	NC_000005	123972610	124080805	0.2958428	0.0960497	0.2216337	0.2549813
RPL28P3	NC_000005	124144519	124144924	0	0	0	0
LOC644659	NC_000005	124588096	124684296	0	0	0	0
LOC100130551	NC_000005	125221943	125231988	0	0	0	0
RPSAP37	NC_000005	125302431	125303195	0	0	0	0
GRAMD3	NC_000005	125759101	125830056	10.983276	8.0412457	11.238027	12.798142
ALDH7A1	NC_000005	125878918	125931082	3.787147	5.1787228	3.6444084	4.8554792
PHAX	NC_000005	125936633	125961080	0.5019787	1.0063693	0.8261622	1.0668861
RPLP1P7	NC_000005	125963671	125964140	0	0	0	0
C5orf48	NC_000005	125967414	125971974	0.0957478	0	0.0851799	0
LOC644754	NC_000005	125998827	125999374	0	0	0	0
LOC100288235	NC_000005	126072791	126073723	0	0	0	0
LMNB1	NC_000005	126112833	126172709	3.8428017	7.070685	6.3896438	8.3947446
LOC100131792	NC_000005	126187126	126189758	0	0	0	0
MARCH3	NC_000005	126203406	126366440	0.3187734	0.2294128	0.2930427	0.4294591
LOC100288306	NC_000005	126372994	126373107	0	0	0	0
FLJ44606	NC_000005	126383213	126407315	0.9155882	0.2689488	0.3490854	0.1822909
MRPS5P3	NC_000005	126478171	126479651	0	0	0	0
LOC100130316	NC_000005	126506173	126520938	0	0	0	0
LOC100128956	NC_000005	126565206	126567880	0.2939681	0.0755575	0.0653805	0
MEGF10	NC_000005	126626456	126796914	0.0971674	0.111651	0.0661033	0.0079659
LOC389322	NC_000005	126847151	126850297	0	0	0	0
PRRC1	NC_000005	126853309	126890781	6.2582432	6.8194092	13.201992	11.235433
CTXN3	NC_000005	126984713	126994322	0	0	0.0420404	0.016465
LOC728586	NC_000005	127039084	127277209	0	0	0	0
LOC100288463	NC_000005	127120306	127122733	0	0	0	0
FLJ33630	NC_000005	127357243	127418766	0	0	0	0
SLC12A2	NC_000005	127419483	127525380	2.575392	3.4579134	1.6585119	3.2142729
LOC100144494	NC_000005	127444900	127446890	0	0	0	0
FBN2	NC_000005	127593601	127873735	1.2868095	2.3299535	5.6181787	7.1079189
SLC27A6	NC_000005	128301210	128369335	0	0.0318081	0.0412857	0.0215592
ISOC1	NC_000005	128430442	128449721	2.1951486	4.792884	6.2612476	4.178987
ADAMTS19	NC_000005	128796103	129074376	0.0086359	0.0266359	0.0230483	0.0300893
KIAA1024L	NC_000005	129083884	129100756	0.153397	0	0.0682331	0.1068931
CHSY3	NC_000005	129240523	129522327	1.4347361	2.0185025	1.8921789	0.762991
RNU7-53P	NC_000005	129722313	129722372	0	0	0	0
LOC100130265	NC_000005	129865052	129865581	0	0	0	0
RPL11P2	NC_000005	130325572	130326164	0	0	0	0
LOC402229	NC_000005	130329947	130331857	0	0	0	0
HINT1	NC_000005	130494875	130501034	36.361261	54.312529	82.494864	70.37456
LYRM7	NC_000005	130506641	130541119	0.8134632	1.2217627	1.4221873	2.3659974
CDC42SE2	NC_000005	130599702	130730383	1.3820199	1.3343745	4.5758157	2.956134
RAPGEF6	NC_000005	130761601	130970929	1.6420218	2.0570152	4.0447874	5.2163254
FNIP1	NC_000005	130977407	131132756	7.0301224	6.9544456	11.20789	12.404604
ACTBP4	NC_000005	130994133	130995892	0	0	0	0
LOC100287186	NC_000005	131132982	131134021	0	0.1369194	0.1184775	0.139204
LOC728637	NC_000005	131252575	131281391	0.0526326	0	0.0468234	0
ACSL6	NC_000005	131289152	131347349	0.0140634	0.0144587	0.0375337	0.0391998
IL3	NC_000005	131396347	131398896	0.095126	0	0	0.0331438
CSF2	NC_000005	131409485	131411859	0.9566196	0.2314131	24.93033	3.8428144
P4HA2	NC_000005	131528303	131563556	17.848894	24.445122	63.746659	15.040822
LOC100132383	NC_000005	131534765	131546359	0	0	0	0
PDLIM4	NC_000005	131593351	131609147	8.1194119	14.490189	22.7771	7.4460488
SLC22A4	NC_000005	131630145	131679899	1.4235057	2.1849638	5.1012335	2.3611334
SLC22A5	NC_000005	131705401	131731306	0.8307288	0.7851994	1.6807185	1.5312439
C5orf56	NC_000005	131746673	131798059	0.9115189	0.4685686	1.0232943	1.104008
IRF1	NC_000005	131817301	131826465	8.2480221	3.5470369	14.411314	5.601061
IL5	NC_000005	131877136	131879214	0	0	0	0.075061
RAD50	NC_000005	131892630	131979599	5.0470452	6.2051153	8.0673287	9.5595122

IL13	NC_000005	131993865	131996801	0	0.0704889	0	0
IL4	NC_000005	132009373	132018368	0	0	0.0424512	0.0665035
KIF3A	NC_000005	132028323	132073265	0.189644	0.1949739	0.2936848	0.5335004
CCNI2	NC_000005	132083137	132089856	0	0.0191862	0	0.0390126
SEPT8	NC_000005	132086509	132113561	2.4270919	2.8091324	3.0997223	1.3955772
ANKRD43	NC_000005	132149033	132152489	0.0254256	0	0.0678581	0.0088588
SHROOM1	NC_000005	132158012	132162002	0.3030913	0.3541019	0.6128145	0.489614
LOC100287281	NC_000005	132158569	132166334	0.832783	0.4670119	1.3470307	0.8968526
RNU7-15P	NC_000005	132176941	132176999	0	0	0	0
GDF9	NC_000005	132196878	132200477	0.0217566	0	0.2129075	0.0606433
UQCRQ	NC_000005	132202319	132204536	7.0982633	15.165659	18.895101	15.109561
LEAP2	NC_000005	132209358	132210582	0	0	0	0.4331666
AFF4	NC_000005	132211071	132299354	11.523795	10.494057	17.814287	19.864613
LOC402230	NC_000005	132323344	132326234	0	0	0	0
ZCCHC10	NC_000005	132332677	132362240	1.2426456	1.5969628	2.3768077	2.0926519
HSPA4	NC_000005	132387662	132440709	6.2968663	8.5161035	7.5192069	9.9610018
RPL6P15	NC_000005	132507910	132508734	0	0	0	0
FSTL4	NC_000005	132532152	132948223	0.0163316	0.0335811	0.0217935	0.022761
LOC100287340	NC_000005	133056186	133056625	0	0	0	0
C5orf15	NC_000005	133291198	133304406	11.463918	9.0723359	19.094559	13.882562
VDAC1	NC_000005	133307607	133340433	40.930745	47.034771	54.922771	60.113618
LOC100288556	NC_000005	133417600	133417731	0	0.3422985	0	0
TCF7	NC_000005	133450402	133483920	0.7069129	1.3864741	0.744992	2.3493473
SKP1	NC_000005	133492082	133512724	24.457297	28.2752	48.122309	37.387541
PPP2CA	NC_000005	133532148	133561950	13.069735	18.668284	37.88455	42.501723
LOC100288591	NC_000005	133562095	133563518	0.2469002	0.3172992	0.1647369	0.2795811
RPS13P6	NC_000005	133603077	133603588	0	0	0	0
CDKL3	NC_000005	133634115	133702765	0.3497207	0.3595496	0.5531044	0.717559
LOC100287379	NC_000005	133706843	133710137	0.079186	0	0.1408921	0.05518
UBE2B	NC_000005	133706870	133727799	7.1493133	5.9248475	11.115537	16.17962
CDKN2AIPNL	NC_000005	133736482	133747589	2.8646125	6.5612861	7.8066096	9.4249832
RPS10P11	NC_000005	133758516	133758781	0	0	0	0
PHF15	NC_000005	133861798	133918918	1.3395949	1.2164493	1.9781686	2.9473425
SAR1B	NC_000005	133942119	133968527	12.556638	18.933997	16.584242	21.852477
SEC24A	NC_000005	133984479	134063601	6.7295085	6.0485061	15.138293	10.121985
CAMLG	NC_000005	134074206	134087847	4.2890921	6.1940024	10.488725	7.0480315
DDX46	NC_000005	134094461	134166811	0.3327653	0.5251109	0.6402665	2.410516
RPL34P13	NC_000005	134140467	134140822	0	0	0	0
C5orf24	NC_000005	134181370	134195425	5.7275463	5.4227113	7.1259325	8.7210313
TXNDC15	NC_000005	134209460	134237323	1.9537842	2.302651	2.8403761	4.084424
PCBD2	NC_000005	134240810	134298336	0.4065858	0.3800118	0.674096	1.262085
CATSPER3	NC_000005	134303614	134347386	0.0656924	0.1013081	0.1753254	0.2288855
PITX1	NC_000005	134363424	134369964	7.3031892	2.1994438	12.20671	10.820876
LOC100288655	NC_000005	134364245	134375222	0.1232769	0	0.1096706	0.0859043
LOC100288626	NC_000005	134370211	134371584	0	0	0	0
LOC389328	NC_000005	134370962	134375737	0.1840378	0.1892102	0.1964702	0.2051918
H2AFY	NC_000005	134670071	134735577	9.8954932	15.480664	25.393451	28.335832
LOC100287455	NC_000005	134733909	134737616	0.6399631	0.3542804	0.1423322	0.1715199
C5orf20	NC_000005	134779904	134783038	0.0140186	0.0144126	0.0249426	0
TIFAB	NC_000005	134784558	134788089	0	0.0354936	0	0
NEUROG1	NC_000005	134869972	134871639	0.0263479	0	0	0
CXCL14	NC_000005	134906369	134914969	0.0222523	0	0	0.0310125
LOC340074	NC_000005	134984372	134989624	0	0	0	0
LOC100130472	NC_000005	135146687	135160791	0.0457318	0	0	0
LOC153328	NC_000005	135170365	135224326	0.0366541	0.0376842	0.0326085	0
IL9	NC_000005	135227935	135231516	0	0.0764525	0.0661549	0
FBXL21	NC_000005	135272263	135277367	0	0.6101285	0.0293305	0.0229744
LECT2	NC_000005	135282600	135290723	0	0	0.0363023	0.0284354
TGFBI	NC_000005	135364584	135399507	216.52926	897.0798	91.910641	168.42045
SMAD5OS	NC_000005	135465202	135470579	0	0	0	0
SMAD5	NC_000005	135468536	135518422	5.413778	8.9347869	11.30771	10.471593
LOC389332	NC_000005	135527156	135528851	0	0	0	0
TRPC7	NC_000005	135549123	135693075	0.2885747	0.0349041	0.1057099	0.153775
LOC100131130	NC_000005	135759527	135765657	0	0	0	0
LOC391834	NC_000005	136193317	136194034	0	0	0	0
SPOCK1	NC_000005	136310987	136835018	66.937518	67.424082	43.552436	57.931586

KLHL3	NC_000005	136953189	137071779	0.1808038	0.1261364	0.0746795	0.0359975
HNRNPA0	NC_000005	137087073	137090039	14.70866	16.766743	16.53773	14.92537
NPY6R	NC_000005	137143462	137145414	0	0	0	0
MYOT	NC_000005	137203545	137223540	0.1547473	0.1590965	0.0344169	0.0134793
PKD2L2	NC_000005	137225125	137276156	0.0198144	0.0407425	0.0528822	0.1242669
FAM13B	NC_000005	137273638	137368802	4.000977	3.165409	1.9187283	5.6958789
LOC100130172	NC_000005	137368092	137373515	0.1076108	0	0.0382934	0.0149975
WNT8A	NC_000005	137419774	137427199	0.0293379	0	0	0.0408877
NME5	NC_000005	137450861	137475132	0.2150811	0.0737086	0.1275614	0.099918
BRD8	NC_000005	137475459	137514358	0.2917334	0.2999326	0.5860449	1.5017339
KIF20A	NC_000005	137514417	137523404	6.5713432	12.548775	10.103865	14.549244
CDC23	NC_000005	137523337	137549032	4.0953344	7.9196264	8.9224239	8.5516697
GFRA3	NC_000005	137588069	137610253	0.0227593	0	0.0202473	0
CDC25C	NC_000005	137620959	137667516	2.1194892	4.742655	7.5791971	7.2254441
FAM53C	NC_000005	137673224	137685418	6.5941001	7.6999728	9.3180319	13.908582
KDM3B	NC_000005	137688285	137772716	1.9416436	2.2018038	2.9152449	4.0860141
REEP2	NC_000005	137774776	137782658	0.9631342	1.0332555	2.4028518	1.8821388
LOC100131361	NC_000005	137789445	137789913	0	0	0	0
EGR1	NC_000005	137801181	137805004	11.463538	5.6911492	2.2815225	1.015621
RPL7P19	NC_000005	137809419	137810106	0	0	0	0
ETF1	NC_000005	137841784	137878932	18.70391	20.858589	21.832742	28.200789
HSPA9	NC_000005	137891024	137911115	21.835332	24.557575	32.224625	33.515437
SNORD63	NC_000005	137896732	137896799	0	0	0	0
RPL10AP10	NC_000005	137946344	137956653	0	0	0	0
CTNNA1	NC_000005	138089107	138270723	2.7343489	2.6910614	3.4409184	10.292434
LRRTM2	NC_000005	138205079	138211057	0	0	0.006947	0
SIL1	NC_000005	138282409	138534065	2.9166247	2.4306807	5.9167256	5.1734334
RPL12P21	NC_000005	138370796	138371334	0	0	0	0
SNHG4	NC_000005	138609778	138615286	0	0	0	0
MATR3	NC_000005	138609792	138666130	7.3476115	10.217079	9.527504	21.062431
SNORA74A	NC_000005	138614469	138614668	0	0	0	0
LOC100128695	NC_000005	138677460	138678840	0	0	0	0
PAIP2	NC_000005	138677519	138705409	6.2669382	6.9071907	10.276399	9.5483004
SLC23A1	NC_000005	138702885	138719039	0	0.0191374	0.0496792	0.0129711
MGC29506	NC_000005	138723257	138725605	0	0	0	0.0740626
LOC389333	NC_000005	138727635	138730770	0.6446488	0.0576319	0.074804	0.0292968
LOC202051	NC_000005	138732456	138739776	0.324102	0.8663484	1.0379885	0.9033887
DNAJC18	NC_000005	138747380	138775139	0.1862739	0.319182	0.209905	0.4845982
RNU5B-4P	NC_000005	138783596	138783707	0	0	0	0
ECSCR (NC_000005 13ε	NC_000005	138784245	138784863	0	0	0.0868835	0.1361106
ECSCR (NC_000005 13ε	NC_000005	138837129	138842320	0.1552941	0.478976	0.8289237	0.2164302
TMEM173	NC_000005	138855549	138862292	23.666435	18.523053	23.138903	16.020004
LOC100130601	NC_000005	138862434	138872726	0	0	0	0
LOC642262	NC_000005	138873426	138877559	0	0	0	0
UBE2D2	NC_000005	138940751	139008019	7.78713	10.637878	19.555601	16.727785
CXXC5	NC_000005	139028301	139062680	17.372763	19.016373	17.508792	13.883843
PSD2	NC_000005	139175406	139224048	0.0487231	0.0100185	0.0346763	0.0203713
NRG2	NC_000005	139227260	139422879	0.1557946	0.072806	0.2015988	0.0493472
PURA	NC_000005	139493708	139496321	3.513841	4.2694339	3.1708814	2.2377015
C5orf53	NC_000005	139505521	139508391	0.933766	0.5980388	1.5252272	2.0480587
C5orf32	NC_000005	139554653	139623374	43.706758	20.754574	92.416334	86.018889
PFDN1	NC_000005	139624635	139682689	16.961893	19.520322	34.815733	35.644213
HBEGF	NC_000005	139712428	139726188	2.1806375	1.1113814	1.0445914	1.0130367
SLC4A9	NC_000005	139739787	139754722	0.0134357	0	0.0478111	0.0468127
LOC100288074	NC_000005	139781399	139833509	0.2768393	0.7827046	0.4925678	0.8198786
ANKHD1	NC_000005	139781429	139919441	1.8976461	1.7312264	3.3949488	1.8345864
ANKHD1-EIF4EBP3	NC_000005	139781429	139929163	1.5795794	2.1073639	3.1676595	2.9303285
EIF4EBP3	NC_000005	139927251	139929163	0.5088073	0.1961653	1.5276907	1.3295895
SRA1	NC_000005	139929653	139937678	4.128607	5.5435488	5.479279	7.0588143
APBB3	NC_000005	139937853	139944189	1.9346997	1.4865716	2.3009225	3.278196
SLC35A4	NC_000005	139944420	139948683	7.0131917	7.3803524	7.5193286	16.966436
LOC728921	NC_000005	139961078	139962126	0	0	0	0
RPL36P11	NC_000005	139988670	139988988	0	0	0	0
LOC100288344	NC_000005	140004847	140012529	0.7857252	0	0.3262014	0.1825082
CD14	NC_000005	140011317	140013255	1.1578572	0.0258782	1.1420251	0.3683405
TMCO6	NC_000005	140019012	140024989	1.2097978	1.2198801	2.2560268	2.2697104

NDUFA2	NC_000005	140024958	140027240	14.17443	18.350938	32.358907	36.63488
IK	NC_000005	140027384	140042065	1.3854481	0.863972	0.9900676	3.2761498
WDR55	NC_000005	140044384	140050553	5.5701829	5.2889098	4.7280777	8.0716733
DND1	NC_000005	140050381	140053171	2.4948778	2.3395024	2.8780491	3.7445266
HARS	NC_000005	140053490	140070971	6.8920852	10.42163	6.3304612	12.490085
HARS2	NC_000005	140071018	140078890	2.0297376	4.3913186	3.0775594	5.8789927
ZMAT2	NC_000005	140080032	140086239	1.5695797	1.4343937	1.3963416	3.909128
VTRNA1-1	NC_000005	140090861	140090958	0	0	0	0
VTRNA1-2	NC_000005	140098511	140098598	0	0	0	0
VTRNA1-3	NC_000005	140105744	140105831	0	0	0	0
PCDHA@	NC_000005	140165876	140391929	0	0	0	0
PCDHA1	NC_000005	140165876	140391929	0	0	0.014382	0.0056327
PCDHA2	NC_000005	140174444	140391929	0.0080124	0.0164753	0	0.0055834
PCDHA3	NC_000005	140180783	140391929	0	0	0	0.0057339
PCDHA4	NC_000005	140186672	140391929	0	0.0168469	0	0
PCDHA5	NC_000005	140201361	140391929	0	0	0	0
PCDHA6	NC_000005	140207650	140391929	0	0	0	0
PCDHA7	NC_000005	140213969	140391929	0	0	0	0
PCDHA8	NC_000005	140220907	140391929	0	0.0085075	0.0147232	0.0115326
PCDHA9	NC_000005	140227357	140391929	0	0	0	0
PCDHA10	NC_000005	140235634	140391929	0	0	0	0
LOC100288105	NC_000005	140240341	140243224	0	0	0	0
PCDHA14	NC_000005	140240773	140243135	0	0	0	0
PCDHA11	NC_000005	140247831	140391929	0	0	0	0
PCDHA12	NC_000005	140254931	140391929	0	0	0	0
PCDHA13	NC_000005	140261854	140391929	0	0	0	0
PCDHAC1	NC_000005	140306302	140391929	0	0	0	0
PCDHAC2	NC_000005	140346352	140391929	0	0	0	0
PCDHACT	NC_000005	140358534	140391929	0	0	0	0
PCDHB1	NC_000005	140430979	140433512	0.0173434	0	0	0.0241712
PCDHB2	NC_000005	140474237	140476964	0.0161101	0.2650053	0	0.0224523
PCDHB3	NC_000005	140480234	140483406	0.0138507	0.01424	0.0246439	0
PCDHB4	NC_000005	140501581	140505201	0	0.0249563	0	0
PCDHB5	NC_000005	140514800	140517704	0.0605139	0.0155537	0.0269174	0.0421685
PCDHB6	NC_000005	140529839	140532868	0.0145044	0.0596481	0	0.0101072
PCDHB17	NC_000005	140535580	140537990	0	0	0	0
PCDHB7	NC_000005	140552243	140555957	0.0236599	0.0608121	0.0105242	0.11541
PCDHB8	NC_000005	140557430	140560021	0.0169553	0.1045912	0.0150839	0.0118152
PCDHB16	NC_000005	140560980	140565796	0.0182471	0.0468999	0.0162332	0.038146
PCDHB9	NC_000005	140566893	140571111	0.0104167	0	0.009267	0.0145176
PCDHB10	NC_000005	140571952	140575213	0.0134728	0.0554058	0	0.0281651
PCDHB11	NC_000005	140579348	140582618	0	0.08288	0.0119528	0.0093625
PCDHB12	NC_000005	140588291	140591698	0	0.1325804	0.0344169	0.0089862
PCDHB13	NC_000005	140593509	140596993	0.0252214	0.3111626	0.0336564	0.0439381
PCDHB14	NC_000005	140603078	140605860	0.0789584	0.259768	0.1123897	0.1430555
PCDHB18	NC_000005	140613936	140617101	0	0	0	0
PCDHB19P	NC_000005	140619689	140624320	0	0	0	0
PCDHB15	NC_000005	140625147	140627802	0.0992806	0	0.0883228	0.0345914
SLC25A2	NC_000005	140682196	140683612	0.031015	0	0	0.0216125
TAF7	NC_000005	140698057	140700351	17.789938	18.289926	29.182627	31.932607
PCDHGA1	NC_000005	140710252	140892546	0.0472257	0.0097106	0.0084027	0.052654
PCDHGA2	NC_000005	140718354	140892546	0.0181679	0.0466964	0	0.0063301
PCDHGA3	NC_000005	140723601	140892546	0	0	0.0083703	0
PCDHGB1	NC_000005	140729828	140892546	0	0.0097927	0	0
PCDHGA4	NC_000005	140734768	140892546	0	0.0287792	0.0249029	0.0325105
PCDHGB2	NC_000005	140739703	140892546	0	0	0.0084682	0.0066331
PCDHGA5	NC_000005	140743898	140892546	0	0.0097736	0	0.0066245
PCDHGB3	NC_000005	140749962	140892546	0	0	0	0.0198605
PCDHGA6	NC_000005	140753651	140892546	0.0379027	0.068194	0	0.0132061
PCDHGA7	NC_000005	140762467	140892546	0	0.1659121	0.0079758	0.0062474
PCDHGB4	NC_000005	140767452	140892546	0	0	0	0
PCDHGA8	NC_000005	140771483	140892546	0	0	0	0
PCDHGB5	NC_000005	140777695	140892546	0.0473784	0.09742	0.0421492	0.0792364
PCDHGA9	NC_000005	140782520	140892546	0	0	0	0
PCDHGB6	NC_000005	140787770	140892546	0	0	0	0.0065945
PCDHGA10	NC_000005	140792743	140892546	0.0092835	0	0.0082589	0

PCDHGB7	NC_000005	140797282	140892546	0	0	0	0
PCDHGA11	NC_000005	140800537	140892546	0	0	0	0.0404632
PCDHGB8P	NC_000005	140805853	140807825	0	0	0	0
PCDHGA12	NC_000005	140810158	140892546	0.0091312	0	0.0162467	0.0127259
PCDHGB9P	NC_000005	140819690	140821993	0	0	0	0
PCDHGC3	NC_000005	140855569	140892546	0.0618367	0.0272463	0.039294	0.0738691
PCDHGC4	NC_000005	140864741	140892546	0	0	0	0
PCDHGC5	NC_000005	140868808	140892546	0	0	0	0.0063563
DIAPH1	NC_000005	140894588	140998622	18.163751	19.06443	19.589298	52.596509
RPS27AP10	NC_000005	140968505	140968963	0	0	0	0
HDAC3	NC_000005	141000443	141016423	12.38233	19.815651	19.357818	25.350156
RELL2	NC_000005	141016517	141020631	2.5351477	2.3265166	1.0746911	1.2804827
FCHSD1	NC_000005	141018869	141030986	0.5900457	0.6275472	0.4615685	0.6096619
ARAP3	NC_000005	141032968	141061800	0.8435521	1.1248623	1.5454758	1.8216623
PCDH1	NC_000005	141232672	141257944	0.10687	0.4310426	0.0804477	0.0229142
LOC729080	NC_000005	141275189	141276254	0	0	0	0
KIAA0141	NC_000005	141303385	141321612	3.3772239	3.0255929	3.0439008	5.9853786
PCDH12	NC_000005	141324530	141338627	0.2120235	0.1656667	0.0301795	0.0177296
RNF14	NC_000005	141346437	141368754	3.1030322	3.7383468	5.9223995	8.1348821
GNPDA1	NC_000005	141380234	141392620	19.24768	16.090563	20.289203	36.47787
MRPL11P2	NC_000005	141416272	141416690	0	0	0	0
NDFIP1	NC_000005	141488324	141534008	4.7528113	4.8110211	6.8803889	7.6626051
SPRY4	NC_000005	141689992	141704620	2.7555682	2.7153487	2.3339492	1.263767
RPS12P10	NC_000005	141946265	141946647	0	0	0	0
FGF1	NC_000005	141971743	142077635	1.9090724	0.6101742	1.4255696	1.6198169
LOC100288384	NC_000005	141993483	142066052	0.1134148	0.1749035	0.3026908	0.5927396
ARHGAP26	NC_000005	142150292	142608572	0.4326283	0.3098519	0.8865171	1.1584679
NR3C1	NC_000005	142657496	142815077	7.9824681	13.211253	10.582613	10.417049
RPL7P21	NC_000005	142699160	142713355	0	0	0	0
HMHB1	NC_000005	143191726	143200284	0	0	0.1018166	0
YIPF5	NC_000005	143537726	143550278	12.598493	20.522432	13.440496	17.283083
KCTD16	NC_000005	143550437	143856944	0.3306928	0.2353756	0.1056079	0.1831702
LOC100128121	NC_000005	143890901	143902551	0	0	0	0
LOC100132712	NC_000005	144381414	144389267	0	0	0	0
ASSP10	NC_000005	144608274	144609848	0	0	0	0
LOC650866	NC_000005	145108568	145108873	0	0	0	0
PRELID2	NC_000005	145138582	145214899	0.7956943	1.0273279	1.3498949	1.5215729
GRXCR2	NC_000005	145239296	145252531	0.1764989	0	0	0
SH3RF2	NC_000005	145316126	145442879	0.2888481	0.1484831	1.1820493	1.8014634
PLAC8L1	NC_000005	145463876	145483946	0	0.0623219	0	0
LARS	NC_000005	145492589	145562294	5.0664181	5.3299455	8.6677424	12.227215
RPL35AP16	NC_000005	145517697	145517944	0	0	0	0
RPL35AP17	NC_000005	145518324	145518571	0	0	0	0
RBM27	NC_000005	145583163	145668786	1.1353181	1.484933	2.384581	2.1346598
POU4F3	NC_000005	145718676	145720007	0.0432136	0	0.076888	0
TCERG1	NC_000005	145826873	145891069	3.2953328	4.3451784	8.0968536	10.081934
GPR151	NC_000005	145894417	145895676	0	0	0.0620596	0.2187491
PPP2R2B	NC_000005	145969067	146461033	0.0254404	0.2615537	0.1810596	1.1523109
STK32A	NC_000005	146614579	146763768	0.0480571	0.0247039	0.1710118	0
DPYSL3	NC_000005	146770371	146833260	13.697924	40.557695	21.577211	30.15186
JAKMIP2	NC_000005	146970706	147162252	0.0394981	0.0270721	0.2225446	0.0183492
SPINK1	NC_000005	147204143	147211260	0.3986234	1.2294803	1.8617888	0.1388883
LOC100288484	NC_000005	147228871	147232229	0	0	0	0
SCGB3A2	NC_000005	147258274	147261753	0	0	0	0
C5orf46	NC_000005	147272271	147286101	0	0	0	0.1695104
LOC100130260	NC_000005	147301823	147303051	0	0	0	0
SPINK5	NC_000005	147443535	147516925	0.0114687	0	0.0612175	0.0239756
SPINK5L2	NC_000005	147549296	147554961	0	0	0.1329849	0
SPINK6	NC_000005	147582404	147594700	0.3843869	0.2634601	0	0
SPINK5L3	NC_000005	147648423	147665773	1.6405037	0.1775379	0.6145	0.1805003
SPINK7	NC_000005	147691990	147695482	0	0	0	0
SPINK9	NC_000005	147715122	147719412	0	0	0	0.0676046
FBXO38	NC_000005	147763546	147822399	3.1735926	3.8306769	5.0837557	4.9618461
HTR4	NC_000005	147830595	148033741	0.0355472	0.0487284	0.0105413	0.0577984
ADRB2	NC_000005	148206156	148208188	0.1729394	0.1111249	0.3653978	0.1958305
SH3TC2	NC_000005	148361713	148442737	0.0545633	0.0934946	0.1515067	0.2200659

ABLIM3	NC_000005	148521054	148639999	4.7141702	9.3180995	7.4858084	10.462617
AFAP1L1	NC_000005	148651458	148721365	0.74076	1.4470004	0.5836863	0.3392113
GRPEL2	NC_000005	148724977	148734146	5.3316003	4.6889474	7.5718793	6.5278364
PCYOX1L	NC_000005	148737609	148749215	1.5783483	1.3522566	3.2763323	3.0307142
IL17B	NC_000005	148753830	148758838	0.1264697	0.1950363	0.112511	0
LOC728264	NC_000005	148786440	148812399	0	0	0	0
CSNK1A1	NC_000005	148874845	148931007	47.967632	61.626774	76.221008	62.027778
RPL29P14	NC_000005	148924833	148925407	0	0	0	0
FLJ41603	NC_000005	148961135	149014527	0.0542013	0.0185749	0.096438	0.0692443
PPARGC1B	NC_000005	149109864	149227281	0.1208107	0.082804	0.3104877	0.0841857
PDE6A	NC_000005	149237519	149324356	0.0233684	0.040042	0.0138595	0.0379961
SLC26A2	NC_000005	149340300	149366963	1.3116438	2.7361837	3.4183135	4.1642249
TIGD6	NC_000005	149372681	149380217	0.6039265	0.5950291	0.4365317	0.754005
HMGXB3	NC_000005	149380296	149432692	6.6026688	6.7968194	6.9506783	10.947013
RPS20P4	NC_000005	149401090	149401616	0	0	0	0
CSF1R	NC_000005	149432854	149492935	0.2203471	0.045308	0.1666229	0.207288
RPL7P	NC_000005	149473848	149474677	0	0	0	0
PDGFRB	NC_000005	149493402	149535422	19.220622	9.4494514	5.706575	4.0250759
CDX1	NC_000005	149546344	149564121	0	0.0254985	0.0220641	0.0345653
SLC6A7	NC_000005	149569520	149590635	0	0	0.0313701	0.0327626
CAMK2A	NC_000005	149599054	149669403	0.0448086	0.175058	0.0478355	0.0936732
ARSI	NC_000005	149675909	149682525	7.9931107	10.643138	3.0122532	15.220574
LOC100287940	NC_000005	149676951	149718870	0.2069126	0.297819	0.4417804	0.6344137
TCOF1	NC_000005	149737202	149779871	0.3812023	0.4522109	0.6260828	1.7572908
CD74	NC_000005	149781200	149792332	3.8026541	0.1193749	43.900833	9.7295683
RPS14	NC_000005	149823792	149829319	180.26603	390.95715	635.90748	428.78726
NDST1	NC_000005	149887674	149937773	17.037465	23.9759	11.422527	24.393116
SYNPO	NC_000005	150020220	150038792	1.1730588	1.6324011	0.9697967	1.2178942
LOC100288180	NC_000005	150029880	150033791	0	0.0115499	0	0.0234853
MYOZ3	NC_000005	150040404	150058925	0.1032457	0.0117942	0.0102056	0.0239819
RBM22	NC_000005	150070356	150080624	3.3673874	5.2913953	4.7829412	6.5596169
DCTN4	NC_000005	150088309	150138657	10.108195	12.592267	17.368638	19.711259
MST150	NC_000005	150157867	150176298	37.584083	30.038383	47.095245	41.107115
IRGM	NC_000005	150226085	150228231	0.1589448	0.0272353	0.1178347	0.0369197
ZNF300	NC_000005	150273964	150284391	1.8406154	1.7322245	2.7962821	3.9267724
LOC134466	NC_000005	150309998	150326146	0	0	0	0
GPX3	NC_000005	150399999	150408554	6.0644068	2.9763059	5.2174491	5.2171854
TNIP1	NC_000005	150409506	150460997	1.6944545	2.3918998	3.0268311	5.0416721
ANXA6	NC_000005	150480268	150537367	46.586633	79.191081	43.60418	61.712019
CCDC69	NC_000005	150560613	150603654	1.2024671	1.2229694	0.6786574	0.1801993
GM2A	NC_000005	150632613	150649955	3.1561738	4.4816057	7.9360641	10.581767
SLC36A3	NC_000005	150655926	150683334	0	0.015147	0	0.0102665
SLC36A2	NC_000005	150694539	150727151	0.0128466	0	0.0228574	0.0268561
SLC36A1	NC_000005	150827163	150871940	1.8042146	2.2227761	1.0497354	2.8646171
FAT2	NC_000005	150883653	150948505	0.0211638	0.0341922	0.045725	0.0758459
SPARC	NC_000005	151041008	151066517	175.84889	386.64103	173.64248	123.54277
ATOX1	NC_000005	151122383	151138210	18.873474	26.795883	23.986237	20.29133
RPLP1P6	NC_000005	151145421	151145929	0	0	0	0
LOC729421	NC_000005	151149706	151151069	0.8010006	0.7137111	1.4726908	1.0791269
G3BP1	NC_000005	151151476	151184915	24.260664	36.258285	32.353753	41.31928
GLRA1	NC_000005	151202168	151304403	0.0257158	0.0264385	0.0686324	0.1075186
GLULL1	NC_000005	151228368	151230472	0	0	0	0
TRNAQ53P	NC_000005	151248074	151248145	0	0	0	0
NMUR2	NC_000005	151771102	151784840	0.0638782	0	0	0
RPL36AP20	NC_000005	151875279	151875578	0	0	0	0
TRNAC32P	NC_000005	151988596	151988771	0	0	0	0
GRIA1	NC_000005	152870084	153193429	0.0375947	0.115954	0.0066891	0.0576345
FAM114A2	NC_000005	153371263	153418497	2.4669486	2.3484097	3.5493978	5.2845424
MFAP3	NC_000005	153418519	153437014	2.3066511	3.4453574	3.3994516	3.1176842
GALNT10	NC_000005	153570295	153799810	7.824987	20.836966	9.7867217	20.381632
SAP30L	NC_000005	153825517	153840613	2.0403275	2.7654419	4.2457758	4.7868284
HAND1	NC_000005	153854532	153857824	0.0505733	0.0779921	0.0224957	0.0352415
LOC100128833	NC_000005	153872838	153874311	0	0	0	0
LOC100131740	NC_000005	153923690	153940000	0	0	0	0
LARP1	NC_000005	154092462	154197165	26.564579	27.632274	33.210389	51.131803
RPL21P57	NC_000005	154108749	154109271	0	0	0	0

C5orf4	NC_000005	154198052	154230213	0.7807487	0.3373632	1.1072946	1.395624
CNOT8	NC_000005	154238198	154256352	7.239157	8.6618201	12.344942	13.406864
GEMIN5	NC_000005	154266976	154317776	1.8298209	3.0852471	2.2428285	2.8562063
MRPL22	NC_000005	154320633	154348971	2.659332	4.8131074	6.1609779	5.0092379
LOC100288010	NC_000005	154373158	154374004	0	0	0	0
KIF4B	NC_000005	154393260	154397685	0	0	0.0265008	0.0138386
LOC100131520	NC_000005	154490744	154491884	0	0	0	0
LOC100130088	NC_000005	154769471	154778368	0	0	0	0
LOC100131033	NC_000005	154870896	154873161	0	0	0	0
SGCD	NC_000005	155753767	156194799	0.3812168	0.5433588	0.0192694	0.0090561
PPP1R2P3	NC_000005	156277549	156279539	0	0	0	0
TIMD4	NC_000005	156346370	156390266	0.0680313	0.0349717	0.0302613	0
LOC100286948	NC_000005	156350731	156351724	0	0	0	0
HAVCR1	NC_000005	156456531	156485487	0	0.0490857	0.0424743	0.0499047
HAVCR2	NC_000005	156512843	156536138	0.0757728	0.0389512	0.0505572	0.0924026
MED7	NC_000005	156565451	156569921	1.8771109	2.1749299	2.5976688	2.9275307
FAM71B	NC_000005	156589343	156593279	0	0.1555749	0.0192315	0.0150639
ITK	NC_000005	156607907	156682109	0.020132	0	0.01791	0.070144
CYFIP2	NC_000005	156693091	156822606	0.1296408	0.253875	0.1482841	0.0989426
C5orf40	NC_000005	156768607	156772729	0	0	0	0
RPL26P18	NC_000005	156849412	156849932	0	0	0	0
NIPAL4	NC_000005	156887027	156901730	0.214447	0.6614222	0.1073126	0.0373588
ADAM19	NC_000005	156904312	157002768	0.5536177	7.0154396	0.0801766	0.0314009
SOX30	NC_000005	157052687	157079428	0.0403812	0.0138387	0.0119748	0.0656582
LOC100289096	NC_000005	157097251	157098521	0	0	0.0307613	0
C5orf52	NC_000005	157098561	157107162	0	0	0	0
LOC442142	NC_000005	157117240	157118907	0.0263479	0	0	0.0550807
THG1L	NC_000005	157158323	157166772	1.8859011	3.3930831	2.1571071	2.6048747
LSM11	NC_000005	157170755	157183746	1.613094	3.0698602	3.297569	2.7862541
CLINT1	NC_000005	157213297	157286168	7.5963075	7.6383036	10.136829	14.324396
RPLP2P2	NC_000005	157358187	157358628	0	0	0	0
LOC345471	NC_000005	157413533	157414664	0	0	0	0
LOC100128898	NC_000005	157505104	157603553	0	0	0	0
LOC100130177	NC_000005	157615985	157616955	0	0	0	0
EBF1	NC_000005	158122923	158526788	3.5998552	1.5420956	5.0261944	6.5674511
RNF145	NC_000005	158584419	158634834	6.7190337	9.6092006	10.129084	12.151573
UBLCP1	NC_000005	158690089	158713048	4.5013866	5.9690447	6.8813023	6.7087944
IL12B	NC_000005	158741791	158757481	0.0374506	0.0192516	0.0166585	0.0130485
LOC285627	NC_000005	158875564	158893284	0	0	0	0
ADRA1B	NC_000005	159343740	159400017	0.2924683	0.2405505	0.6417968	0.6114106
LOC100131743	NC_000005	159377299	159378576	0	0	0	0
TTC1	NC_000005	159436180	159492550	0.5310021	0.9312855	0.3334547	1.9154153
PWWP2A	NC_000005	159502889	159546452	1.5752873	1.7760402	2.3898599	2.7734738
FABP6	NC_000005	159614374	159665729	0	0	0	0.0353228
LOC100287079	NC_000005	159674524	159675346	0	0	0	0
CCNJL	NC_000005	159678671	159739573	0.0536936	0.2346114	0.0358255	0.0561238
LOC727947	NC_000005	159685260	159685595	0.2615966	0.4034232	0.2327236	0
SNRPEP1	NC_000005	159753700	159753980	0	0	0	0
C1QTNF2	NC_000005	159774761	159797648	1.2030623	0.8433236	0.2432449	0.3048515
C5orf54	NC_000005	159820155	159827060	0.454043	0.4668039	1.1142876	1.2764912
SLU7	NC_000005	159828648	159846168	3.1973573	3.1222238	6.1941087	6.374448
PTTG1	NC_000005	159848865	159855746	2.8393521	3.80759	10.707901	13.41989
ATP10B	NC_000005	159990127	160279219	0.0348519	0.0119438	0.0206701	0.5545345
LOC285629	NC_000005	160358785	160365633	0	0	0	0
GABRB2	NC_000005	160715436	160975130	0.0059317	0.0060984	0.0105541	0.0124004
GABRA6	NC_000005	161112658	161129598	0	0	0	0.0243151
GLRXL	NC_000005	161177462	161178843	0	0	0	0
GABRA1	NC_000005	161274197	161326965	0	0.0084282	0.0145859	0.0285627
LOC100287123	NC_000005	161274789	161379341	0.1551114	0	0.32198	0.0360293
GABRG2	NC_000005	161494648	161582545	0.0107796	0.0110825	0	0.0225349
LOC100129748	NC_000005	162188839	162189325	0	0	0	0
MRP63P6	NC_000005	162466287	162466612	0	0	0	0
CCNG1	NC_000005	162864577	162872022	16.720984	13.712467	22.130996	16.863537
NUDCD2	NC_000005	162880586	162887143	6.5366041	8.8650978	16.166926	14.246383
HMMR	NC_000005	162887517	162918951	2.8543264	3.3660987	2.6513153	4.4070185
MAT2B	NC_000005	162930231	162946328	2.3792397	2.8637367	8.0879647	6.6991921

LOC391844	NC_000005	163136211	163195064	0	0	0	0
RPS15P6	NC_000005	164027977	164028476	0	0	0	0
LOC574080	NC_000005	165209348	165210276	0	0	0	0
RPL21P59	NC_000005	165332089	165332642	0	0	0	0
RPL7P20	NC_000005	165455489	165456336	0	0	0	0
RPLP0P9	NC_000005	165809310	165809691	0	0	0	0
ODZ2	NC_000005	166711843	167691162	0.1458106	0.1077468	0.0040537	0.0349273
LOC100289231	NC_000005	167718656	167798490	0	0.0657691	0.1138212	0.0891554
WWC1	NC_000005	167719092	167896764	0.0741295	0.0653254	0.4239495	0.9224361
RARS	NC_000005	167913463	167946311	12.075495	17.967792	16.461171	23.455222
FBLL1	NC_000005	167956582	167957639	0	0	0	0
SLC2A3P1	NC_000005	167979499	167980658	0	0	0	0
PANK3	NC_000005	167982630	168006588	3.3363498	5.0173888	4.7955335	5.1694867
RPL10P9	NC_000005	168043301	168044025	0	0	0	0
SLIT3	NC_000005	168093071	168728133	3.692305	4.8794715	4.280384	5.8688196
LOC728095	NC_000005	168440259	168463475	0	0	0	0
CCDC99	NC_000005	169010638	169031782	5.7488504	17.782073	7.1219704	11.799991
DOCK2	NC_000005	169064251	169510386	0.8217317	0.3853595	1.1478537	2.8078247
LOC100131897	NC_000005	169290719	169407744	0.049547	0.1273489	0	0.0517895
FOXI1	NC_000005	169532917	169536729	0.0191412	0.0196792	0.0170286	0.0133384
KRT18P41	NC_000005	169567608	169568995	0	0	0	0
LOC133874	NC_000005	169659950	169673235	0	0	0	0
LCP2	NC_000005	169675088	169724822	0	0.0186554	0.0161427	0.0126445
LOC257358	NC_000005	169758435	169762104	0	0	0	0
KCNIP1	NC_000005	169780881	170163636	0.0413241	0	0	0.0287963
KCNMB1	NC_000005	169805167	169816638	0	0	0	0
GABRP	NC_000005	170210723	170241051	0	0	0.0236239	0.0185045
RANBP17	NC_000005	170289022	170727019	0.039593	0.0203529	0.0176115	0.0482825
LOC100240749	NC_000005	170323870	170332088	0	0	0	0
USP12P1	NC_000005	170678647	170680956	0	0	0	0
LOC728145	NC_000005	170732985	170735809	0	0	0	0
TLX3	NC_000005	170736288	170739138	0	0	0	0
RPL19P10	NC_000005	170771971	170772666	0	0	0	0
RPL10P8	NC_000005	170793303	170793679	0	0	0	0
NPM1	NC_000005	170814798	170837888	29.515528	59.273137	62.116547	66.812974
FGF18	NC_000005	170846667	170884176	0.1150477	0.1478514	0.0255874	0.0801698
C5orf50	NC_000005	171129776	171233616	0.0393801	0	0.0350337	0.0548833
FBXW11	NC_000005	171288556	171433877	6.4150394	8.4268311	13.167399	11.918479
STK10	NC_000005	171469074	171615346	0.6717457	1.0134174	0.7470045	0.7581171
UBTD2	NC_000005	171636650	171710795	2.871576	4.2710678	3.2289532	3.1386691
LOC651815	NC_000005	171680784	171682744	0	0	0	0
LOC100288254	NC_000005	171723519	171724157	0	0	0	0
SH3PXD2B	NC_000005	171760503	171881527	1.5653414	1.6209553	2.6393496	2.1894602
NEURL1B	NC_000005	172068276	172118543	0.9451233	0.3872662	1.4744602	1.6560438
LOC100130394	NC_000005	172083362	172083737	0	0	0	0
LOC401218	NC_000005	172189302	172190261	0	0	0	0
DUSP1	NC_000005	172195102	172198203	21.287084	21.50416	17.094271	20.274733
LOC100288288	NC_000005	172211110	172222613	0	0	0	0
ERGIC1	NC_000005	172261223	172379688	20.088054	25.777315	51.854878	88.995729
LOC100268168	NC_000005	172381785	172386371	0	0	0	0
RPL26L1	NC_000005	172386439	172396774	9.2848377	12.854996	13.987016	15.010504
LOC285591	NC_000005	172402455	172403696	0	0	0	0
ATP6V0E1	NC_000005	172410763	172461900	99.556199	102.86652	117.07106	144.51331
SNORA74B	NC_000005	172447729	172447932	0	0	0	0
C5orf41	NC_000005	172483370	172563970	2.4693645	1.4602054	4.5084964	2.288712
BNIP1	NC_000005	172571445	172591390	2.5049858	3.1295863	5.0211881	3.2480932
LOC100289336	NC_000005	172639451	172642279	0.0155349	0.0159715	0.0138203	0.0216507
RPL7AP33	NC_000005	172652839	172653427	0	0	0	0
NKX2-5	NC_000005	172659138	172662262	1.0259209	1.0547545	0.6413481	0.6762591
STC2	NC_000005	172741726	172756506	42.878932	41.986822	17.576708	56.692995
LOC285593	NC_000005	173006646	173012075	0	0	0	0
BOD1	NC_000005	173034540	173043656	16.437557	40.022113	30.930972	25.927198
CPEB4	NC_000005	173315331	173387313	1.7196888	1.0410386	2.4910922	1.8369408
C5orf47	NC_000005	173416162	173433143	0.0348519	0	0	0.0121431
RPL12P22	NC_000005	173466754	173467225	0	0	0	0
HMP19	NC_000005	173472724	173536182	0.037643	0	0.0167441	0.0262312

LOC724105	NC_000005	173906840	173907628	0	0	0	0
GAPDHL16	NC_000005	173940236	173941512	0	0	0	0
LOC100127922	NC_000005	173986788	173989159	0.0856691	0	0.7621358	0.4178833
LOC100288354	NC_000005	174044649	174060212	0	0	0	0
MSX2	NC_000005	174151575	174157902	0.9952045	0.5320509	0.0708289	0.1248297
LOC645398	NC_000005	174350049	174351002	0	0	0	0
LOC100288396	NC_000005	174670648	174718311	0	0	0	0
DRD1	NC_000005	174867675	174871163	0	0.0267912	0.0231827	0.0090794
LOC100128769	NC_000005	174905175	174911196	0	0	0	0
SFXN1	NC_000005	174905514	174955621	6.1712663	9.1091914	10.091381	10.208293
HRH2	NC_000005	175085040	175113245	0.0759974	0.2902096	0.0096585	0.0226963
CPLX2	NC_000005	175223610	175311023	0	0	0.0158482	0.0124138
THOC3	NC_000005	175386534	175395545	5.0727992	7.1186104	7.8708447	10.47076
OR1X1P	NC_000005	175433148	175434392	0	0	0	0
LOC100288464	NC_000005	175487726	175489328	0	0	0	0
FAM153B	NC_000005	175511909	175543458	0	0	0.0186534	0
LOC643201	NC_000005	175603523	175612177	0.2497059	0.1711492	0	0.1160033
C5orf25	NC_000005	175665370	175772992	2.1974116	2.3907722	3.3783333	4.5639989
KIAA1191	NC_000005	175773065	175788809	16.628221	21.649049	30.982194	30.679024
ARL10	NC_000005	175792502	175800503	2.2111169	3.5507954	1.9345573	3.7946835
NOP16	NC_000005	175811232	175815540	4.7467364	11.106535	6.6982794	11.976209
HIGD2A	NC_000005	175815784	175816751	23.880218	25.512723	35.130219	29.421937
CLTB	NC_000005	175819456	175843540	25.537486	25.186694	28.959937	33.211439
FAF2	NC_000005	175875356	175937075	7.0375574	9.3268949	10.89363	14.12882
RNF44	NC_000005	175953700	175964421	1.2910388	1.2185266	4.7259759	4.5793521
LOC100289372	NC_000005	175956079	175956809	0.0737386	0.1516222	0.0655999	0.1541521
PCDH24	NC_000005	175976365	176022769	0.0536608	0.0331014	0.0381905	0.0897432
GPRIN1	NC_000005	176022803	176037131	1.1175049	1.3851376	2.601549	4.0173319
LOC729370	NC_000005	176035003	176037187	0	0	0	0
SNCB	NC_000005	176047210	176057557	0	0.3739316	0	0.0211206
EIF4E1B	NC_000005	176057683	176073642	0.0222635	0	0.0198063	0
TSPAN17	NC_000005	176074388	176086059	8.7794574	12.954437	12.176444	15.513607
LOC729378	NC_000005	176169905	176170870	0	0	0	0
UNC5A	NC_000005	176237560	176307899	0.0117729	0.0363113	0.0104735	0.0820382
HK3	NC_000005	176307870	176326333	0.0429742	0	0.0254873	0.0399281
UIMC1	NC_000005	176332006	176433443	2.7341066	2.7415428	4.0088518	6.1743706
ZNF346	NC_000005	176449697	176493758	1.822869	2.79651	2.1284482	3.8206669
FGFR4	NC_000005	176513921	176525127	0.0279569	0.0862279	0.0746137	0.0487037
LOC100289405	NC_000005	176523606	176525127	0	0.0530322	0	0
NSD1	NC_000005	176560080	176727214	1.0499922	1.5396748	1.7394659	3.4848433
RPL21P60	NC_000005	176691727	176692275	0	0	0	0
RAB24	NC_000005	176728199	176730744	4.3893705	4.0362344	4.8265605	4.7685139
PRELID1	NC_000005	176730835	176733950	39.815401	52.088002	53.672981	82.103473
MXD3	NC_000005	176732501	176739292	2.9057831	4.2739124	4.3909003	5.7258156
LMAN2	NC_000005	176758563	176778885	24.109156	22.22175	41.253054	25.058238
RPS20P17	NC_000005	176773079	176773426	0	0	0	0
RGS14	NC_000005	176784844	176799599	0.5845151	0.3568099	0.6337511	0.0890998
SLC34A1	NC_000005	176811435	176825849	0.0510432	0.0174926	0.0151365	0.0118563
PFN3	NC_000005	176827108	176827637	0	0	0	0
F12	NC_000005	176829139	176836577	0.0214277	0.1101497	0.1334388	0.0895901
GRK6	NC_000005	176853687	176869850	3.9770206	8.3399615	6.9994988	9.8747974
PRR7	NC_000005	176873796	176883283	2.0247153	1.4387669	3.946841	7.4279854
DBN1	NC_000005	176883614	176900694	6.4146394	14.511557	9.9394595	13.363751
PDLIM7	NC_000005	176910395	176924602	18.477752	29.105605	15.891633	15.430568
DOK3	NC_000005	176928905	176937427	0.1298323	0.0934369	0.0577512	0.3076059
DDX41	NC_000005	176938578	176943967	9.6920055	9.5993254	11.335321	14.701106
FLJ10404	NC_000005	176946790	176981538	2.2312335	1.6192535	2.4160912	3.8272304
TMED9	NC_000005	177019213	177023108	26.049202	37.609865	32.516235	47.466377
B4GALT7	NC_000005	177027172	177037331	6.384282	9.6685399	7.140785	7.8269731
LOC202181	NC_000005	177045501	177099278	0	0	0	0
FAM153A	NC_000005	177150365	177207505	0	0	0	0
LOC100288592	NC_000005	177208895	177210365	0	0	0	0
OR1X5P	NC_000005	177263314	177264765	0	0	0	0
LOC728554	NC_000005	177302262	177311269	0	0	0	0
LOC100128340	NC_000005	177378191	177380275	0	0	0	0
LOC285647	NC_000005	177398179	177400822	0	0	0	0

PROP1	NC_000005	177419236	177423243	0.0300193	0	0	0.0209186
LOC100288656	NC_000005	177433440	177434948	0	0	0	0
FAM153C	NC_000005	177435689	177474656	0	0	0	0.0169479
RPL19P9	NC_000005	177482573	177483269	0	0	0	0
N4BP3	NC_000005	177540556	177553107	0.0440437	0.1433915	0.0195912	0.0409218
RMND5B	NC_000005	177558028	177575479	6.0526049	5.2730629	8.2174087	10.061492
NHP2	NC_000005	177576464	177580961	10.640098	18.179053	19.297278	30.230874
LOC645853	NC_000005	177592666	177593599	0	0	0	0
LOC100288686	NC_000005	177611504	177631396	0.0286868	0.1032258	0.0510412	0.0799605
GMCL1L	NC_000005	177611511	177614433	0	0	0	0
HNRNPAB	NC_000005	177631508	177638184	3.0492716	5.8718518	6.3942605	7.8248588
AGXT2L2	NC_000005	177635540	177659803	2.8381869	1.144736	2.1170565	2.2211785
COL23A1	NC_000005	177664617	178017556	0.0573924	0.0295027	0.0127645	0.029995
MRPL50P3	NC_000005	177771844	177772320	0	0	0	0
CLK4	NC_000005	178029665	178054054	0.7537989	0.6668472	1.1384612	2.12554
VN2R2P	NC_000005	178089169	178089854	0	0	0	0
ZNF354A	NC_000005	178138522	178157703	0.2924148	0.7604252	0.4437688	0.6232852
LOC100132848	NC_000005	178157032	178157731	0	0	0	0
AACSL	NC_000005	178191862	178203277	0	0	0	0
LOC100129457	NC_000005	178266435	178268140	0	0	0	0
ZNF354B	NC_000005	178286954	178311424	0.6401199	0.8020722	1.4414669	1.8539412
ZFP2	NC_000005	178322916	178360210	0.0913685	0.0563618	0.2601085	0.1273384
PIGFP1	NC_000005	178349021	178349915	0	0	0	0
ZNF454	NC_000005	178368224	178393434	0.2292951	0.3536092	0.1699894	0.1997275
LOC100130798	NC_000005	178396472	178417116	0	0	0	0
GRM6	NC_000005	178405328	178422124	0.0071542	0	0.0127291	0.0099707
LOC100288803	NC_000005	178424960	178425847	0	0	0	0
DKFZp686E2433	NC_000005	178450776	178461388	1.0463865	0.9196315	1.3062551	1.2583955
ZNF354C	NC_000005	178487607	178507695	1.3866712	2.1859871	2.2040697	2.6411864
LOC645944	NC_000005	178507697	178510978	0	0	0	0
ADAMTS2	NC_000005	178540850	178772329	10.497565	11.156396	7.1038787	11.375424
LOC100288835	NC_000005	178547438	178549768	0	0	0.0406843	0
LOC100128622	NC_000005	178933542	178951801	0	0	0	0
LOC100289470	NC_000005	178949458	179080240	0	0.0374035	0	0
LOC100288901	NC_000005	178977564	179029659	0.0935069	0.0961349	0.1663726	0.0325797
RUFY1	NC_000005	178977571	179037019	1.5803303	1.3152703	1.1381141	1.6361236
HNRNPH1	NC_000005	179041179	179050722	18.486006	34.093478	20.809153	28.323587
LOC285679	NC_000005	179068558	179072047	0	0	0	0
CBY3	NC_000005	179105584	179107969	0	0.2320072	0	0.1965653
LOC729595	NC_000005	179106033	179121810	0.0507485	0.313049	0.0902946	0
CANX	NC_000005	179125930	179158642	17.311327	24.803891	31.203852	31.416315
MAML1	NC_000005	179159851	179204287	3.1177673	5.0133602	5.6019577	6.7853129
LOC100289556	NC_000005	179219178	179220712	0.1145231	0.0588709	0.0254707	0.1596085
LTC4S	NC_000005	179220986	179223513	0.1317788	0	0.058617	0.0918287
MGAT4B	NC_000005	179224105	179233941	16.321767	15.619367	29.542959	44.049275
SQSTM1	NC_000005	179233388	179265078	227.30073	200.46157	457.29038	409.77868
C5orf45	NC_000005	179264266	179285840	0.5854885	0.8276726	1.0091795	1.2239752
TBC1D9B	NC_000005	179289071	179334856	12.108099	13.358191	19.469301	24.962389
LOC100129309	NC_000005	179335679	179367131	0	0	0	0
RPS15AP18	NC_000005	179360775	179361226	0	0	0	0
RNF130	NC_000005	179382474	179499109	11.730633	13.919521	32.514272	29.364732
LOC646058	NC_000005	179512607	179514055	0	0	0	0
RASGEF1C	NC_000005	179527795	179636130	0.0594967	0.0407792	0	0.0552796
RPS8P7	NC_000005	179653333	179654000	0	0	0	0
MAPK9	NC_000005	179660594	179719071	2.9731462	4.965209	8.2840652	7.1724475
GFPT2	NC_000005	179727700	179780315	12.304337	11.044498	14.672949	20.413195
CNOT6	NC_000005	179921417	180005353	2.0422834	2.53864	3.2918935	2.9454627
SCGB3A1	NC_000005	180017105	180018487	0	0	0	0
FLT4	NC_000005	180028506	180076624	0.0275149	0.0777927	0.0183585	0.3259496
OR2AI1P	NC_000005	180119835	180120763	0	0	0	0
OR2Y1	NC_000005	180166123	180167058	0	0	0	0
LOC100289003	NC_000005	180167571	180229804	6.3070745	5.4725952	4.974245	5.8600276
MGAT1	NC_000005	180217541	180237137	12.142122	13.814326	12.946477	15.102467
LOC100130663	NC_000005	180237157	180238384	0.6084039	0.4047373	0.2547073	0.6733483
LOC729678	NC_000005	180249059	180262726	0	0	0	0
ZFP62	NC_000005	180274611	180287684	0.554792	0.8611688	1.8484245	2.6379846

BTNL8	NC_000005	180326129	180377906	0	0	0	0
RPS29P12	NC_000005	180372726	180372993	0	0	0	0
LOC100128762	NC_000005	180394189	180397807	0	0	0	0
LOC646227	NC_000005	180409218	180413252	0.0108918	0.0223957	0.0193792	0.0455389
BTNL3	NC_000005	180415901	180433727	0.0184347	0	0	0.012846
BTNL9	NC_000005	180467225	180488523	0	0.0129095	0	0.0349999
RPL13P10	NC_000005	180492155	180492680	0	0	0	0
FOXO1B	NC_000005	180525777	180527878	0	0	0	0
LOC729707	NC_000005	180529064	180542669	0	0	0	0
OR2V1	NC_000005	180551360	180552304	0	0	0	0
OR2V2	NC_000005	180581943	180582890	0	0	0.0824843	0.0323047
TRNAL43P	NC_000005	180591564	180591645	0	0	0	0
TRIM7	NC_000005	180620924	180632177	0.1490718	0.3352595	0.5553396	0.4804412
TRIM41	NC_000005	180650306	180662808	0.6191602	0.6490433	0.5940238	1.235147
GNB2L1	NC_000005	180663928	180670906	274.38914	422.49944	452.49527	538.71066
SNORD96A	NC_000005	180668818	180668889	0	0	0	0
LOC100289627	NC_000005	180669038	180670914	0	0.413011	0.7862399	1.1757266
SNORD95	NC_000005	180670314	180670376	0	0	0	0
TRIM52	NC_000005	180683373	180688119	0.6863847	0.4838919	3.5939752	2.5008267
LOC100132062	NC_000005	180751082	180754049	0	0	0	0.051384
LOC100286887	NC_000005	180754076	180755896	0	0	0	0
LOC100286914	NC_000005	180756242	180769421	0	0	0	0.0327889
LOC100289067	NC_000005	180757121	180758807	0.0416176	0	0	0
OR4F3	NC_000005	180794288	180795226	0	0	0	0
RPL23AP45	NC_000005	180898962	180901521	0	0	0	0
LOC100288138	NC_000006	95039	95320	0	0	0	0
OR4F1P	NC_000006	105922	106856	0	0	0	0
LOC100288169	NC_000006	122141	140474	0.1277565	0.0656735	0.1136557	0.0445129
LOC646070	NC_000006	141879	144430	0	0	0	0
LOC100132266	NC_000006	144746	145149	0	0	0	0
LOC100288308	NC_000006	146069	146569	0	0	0	0
FLJ43763	NC_000006	203313	203839	0	0	0	0
DUSP22	NC_000006	292101	351355	0.890241	0.9152613	3.0623346	2.2746365
IRF4	NC_000006	391752	411443	0.0165405	0.6632114	0.0220724	0.0172892
EXOC2	NC_000006	485138	693109	0.4452207	0.87478	1.1354313	2.0131617
HUS1B	NC_000006	655939	656964	0	0	0	0
LOC100288082	NC_000006	709100	711405	0.1143493	0.1567507	0.1695471	0.0531221
LOC285768	NC_000006	961241	1101567	0	0	0	0
FOXQ1	NC_000006	1312675	1314993	0	0.4481321	1.8714229	2.0073228
LOC100288267	NC_000006	1384027	1385295	0	0.2492386	0.0308097	0.2654639
FOXF2	NC_000006	1390069	1395832	0.2612378	5.1236779	0.3754224	0.672151
ELF2P2	NC_000006	1512576	1515467	0	0	0	0
FOXC1	NC_000006	1610681	1614132	3.80664	1.0733021	4.0773823	2.5018006
FLJ46552	NC_000006	1617524	1620998	0.0379409	0	0.0225022	0.2732004
GMDS	NC_000006	1624041	2245846	11.355046	5.2425718	6.1031487	11.118003
LOC100128372	NC_000006	2341684	2341950	0	0.1692262	0.4392985	0.1146999
C6orf195	NC_000006	2622972	2635298	0	0.0218277	0.0944386	0.2219194
MYLK4	NC_000006	2663863	2751154	0.0381495	0.0078443	0.0407266	0.0850691
WRNIP1	NC_000006	2765666	2785979	6.3966883	9.864702	9.7449061	11.998208
LOC100288968	NC_000006	2766286	2769117	0	0.0759385	0.0657102	0
SERPINB1	NC_000006	2833733	2842081	6.3560088	5.2067493	11.006585	8.1003158
MGC39372	NC_000006	2854891	2877086	0.0642988	0.033053	0	0
SERPINB9	NC_000006	2887504	2903545	0.2650677	0.0654042	0.2169467	0.0221652
LOC100288376	NC_000006	2930013	2948934	0.5613825	0.7420632	1.0701888	1.0618115
SERPINB6	NC_000006	2948393	2972090	18.850214	27.203373	28.004878	31.666794
DKFZP686I15217	NC_000006	2988201	2991405	0	0	0	0
NQO2	NC_000006	3000067	3019994	1.3504724	1.9041293	1.8536159	1.5325883
LOC401233	NC_000006	3022832	3025005	0	0	0	0
FAM136B	NC_000006	3045618	3046034	0	0.1083535	0	0.073441
SERPINBP1	NC_000006	3048971	3049567	0	0	0	0
RIPK1	NC_000006	3077058	3115421	3.7874641	3.8822178	2.8635122	4.0738063
LOC100288414	NC_000006	3118625	3124377	0.054662	0	0	0.0761813
BPHL	NC_000006	3118926	3153432	1.1923939	2.4518125	1.2123276	1.2067943
TUBB2A	NC_000006	3153900	3157783	3.9312114	5.2123974	1.9054335	1.0013069
LOC100132153	NC_000006	3163113	3180036	0	0	0	0
LOC100131504	NC_000006	3224432	3225452	0.0458271	0.0942302	0	0.0319342

TUBB2B	NC_000006	3224495	3227968	0	0.0895164	0.0580945	0.1213467
LOC100289364	NC_000006	3228159	3229942	0.0492693	0.1519621	0.1095784	0.1029985
PSMG4	NC_000006	3259162	3268300	4.2598574	2.731818	5.1779885	4.3497908
SLC22A23	NC_000006	3269207	3456793	1.4014618	1.8131271	2.7560383	3.2942538
LOC643327	NC_000006	3456035	3469245	0	0	0	0
LOC100129626	NC_000006	3694154	3694309	0	0	0	0
C6orf145	NC_000006	3722836	3752246	7.0309158	6.4253516	5.3995271	11.697196
LOC100289494	NC_000006	3722836	3724425	0	0.0284172	0.0245897	0.0577828
FAM50B	NC_000006	3849632	3851551	0.8148621	0.8377639	1.1357142	1.2113672
LOC100289591	NC_000006	3850559	3866460	0.211969	0.2179264	0.5657204	0.7385421
RPS25P7	NC_000006	3914109	3914555	0	0	0	0
TDGF4	NC_000006	3941393	3942457	0	0	0	0
LOC728344	NC_000006	3943757	3979333	0	0	0	0
PRPF4B	NC_000006	4021569	4065217	0.4346658	0.5374663	0.731577	2.1898302
C6orf146	NC_000006	4068601	4079391	0	0.1028766	0.017804	0.1255118
C6orf201	NC_000006	4079440	4130999	0.0532383	0.0547346	0.2131303	0.0927465
PECI	NC_000006	4115927	4135831	29.689072	29.844501	31.032357	48.112855
LOC100129052	NC_000006	4186916	4190045	0	0	0	0
KU-MEL-3	NC_000006	4610896	4611326	0	0	0	0
LOC442153	NC_000006	4689098	4704013	0	0	0	0
CDYL	NC_000006	4706393	4955778	1.8701375	4.4150842	3.009085	3.6843169
LOC100288606	NC_000006	4775351	4893421	0.203464	0.09297	0.1810072	0.0945212
RPS18P8	NC_000006	4979263	4979766	0	0	0	0
RPP40	NC_000006	4995280	5004271	2.1993075	4.3662994	1.0120164	2.0610358
LOC100129461	NC_000006	5030265	5043174	0.3662353	0.3765283	0.7819513	0.9697878
LOC100129033	NC_000006	5045036	5045380	0	0.7857983	0.1133263	0.5326066
LOC442155	NC_000006	5066029	5067216	0	0	0	0
PPP1R3G	NC_000006	5085720	5087455	0.7594741	0.6246553	0.7657357	0.6350781
LYRM4	NC_000006	5108653	5261168	3.0840864	5.1829813	7.3604729	7.5838936
FARS2	NC_000006	5261584	5771816	2.3633215	3.288743	3.9925814	5.1401887
LOC100287565	NC_000006	5368782	5404934	0	0.4507073	0	0
RPL34P16	NC_000006	5788543	5788965	0	0	0	0
LOC100128914	NC_000006	5817654	5932095	0	0	0	0
LOC442156	NC_000006	5958721	5974245	0	0	0	0
LOC100287622	NC_000006	5980771	5999440	0	1.061751	0	0
NRN1	NC_000006	5998232	6007633	0.0213652	1.1202496	0	0.0148881
F13A1	NC_000006	6144311	6320924	0.0113767	0.0233929	0.010121	0.0554942
LOC285780	NC_000006	6346698	6623059	0	0	0	0
LOC643875	NC_000006	6534930	6535864	0	0	0	0
LY86	NC_000006	6588934	6655216	0.049998	0	0	0.0348406
LOC652960	NC_000006	6794889	6795726	0	0	0	0
RREB1	NC_000006	7108188	7251694	0.7909815	0.7797924	1.0217806	1.2496098
LOC100288758	NC_000006	7229009	7247440	0	0	0	0.0144253
LOC100288790	NC_000006	7268542	7290188	0.1399625	0.0479654	0.5810679	0.2925944
SSR1	NC_000006	7281283	7313541	8.9429282	10.229599	10.366028	9.5945325
CAGE1	NC_000006	7326887	7389942	0.015415	0	0.0137136	0.0429672
RPS3P4	NC_000006	7338994	7339831	0	0	0	0
RIOK1	NC_000006	7390062	7418270	0.1534267	0.1752653	0.1516585	0.677121
LOC442157	NC_000006	7481327	7487756	0	0	0	0
RPS26P29	NC_000006	7506436	7506782	0	0	0	0
LOC644051	NC_000006	7516727	7518072	0	0	0	0
LOC644058	NC_000006	7538987	7541185	1.0987058	0.4841079	0.1396342	0.382811
DSP	NC_000006	7541870	7586946	0.9304559	1.546359	1.2697668	0.7050332
SNRNP48	NC_000006	7590432	7612200	1.0636883	1.6024787	1.4334837	2.8547993
RPL29P1	NC_000006	7620555	7621217	0	0	0	0
BMP6	NC_000006	7727011	7881961	0.3255425	0.8149019	0.1259181	0.3057556
TXNDC5	NC_000006	7881750	7911041	49.485131	72.144813	84.394153	86.243284
MGC26597	NC_000006	7986238	7990677	0	0	0	0
MUTED	NC_000006	8014214	8064647	2.4974986	2.3885499	4.3231229	4.6274596
EEF1E1	NC_000006	8073593	8102828	6.2480619	7.7664643	16.204292	20.096808
SCARNA27	NC_000006	8086641	8086766	0	0	0	0
SLC35B3	NC_000006	8413301	8435794	4.3055378	4.3857478	4.5716792	6.1388018
HULC	NC_000006	8652442	8654079	0	0	0	0
OFCC1	NC_000006	9896944	10060922	0.09175	0.0943286	0	0.0319675
RPL7AP36	NC_000006	10118667	10119544	0	0	0	0
RPL21P62	NC_000006	10214083	10214635	0	0	0	0

LOC442161	NC_000006	10365744	10366820	0	0	0	0
TFAP2A	NC_000006	10396916	10419797	0.1550384	0.3081653	0.1930971	0.1296444
C6orf218	NC_000006	10429677	10434774	0.0407683	0	0.0362686	0
MRPL48P1	NC_000006	10459910	10460822	0	0	0	0
GCNT2	NC_000006	10521568	10629601	0.3069095	0.0433088	0.2409134	0.062902
RPL21P63	NC_000006	10574945	10575472	0	0	0	0
GCNT6	NC_000006	10633993	10647501	0.2242257	0.1921063	0.1329849	0.598956
C6orf52	NC_000006	10671651	10695030	0.707701	0.2910364	2.0146894	0.9369931
PAK1IP1	NC_000006	10695188	10709972	2.0843851	3.2597244	3.2124252	3.7641801
TMEM14C	NC_000006	10723338	10731362	25.975506	37.231491	81.257708	51.685324
TMEM14B	NC_000006	10747995	10757214	12.388699	18.779932	43.119949	29.96323
MAK	NC_000006	10762956	10831110	0.1262221	0.1179723	0.2143731	0.2638697
GCM2	NC_000006	10873456	10882098	0	0.0382101	0	0.0517968
LOC100288888	NC_000006	10875288	10882071	0	0	0	0
SYCP2L	NC_000006	10887064	10974542	0.0280819	0.0144356	0.0374737	0.0391372
ELOVL2	NC_000006	10980992	11044624	1.5749735	1.8410517	3.9059178	4.3824998
LOC221710	NC_000006	11094266	11138971	1.4234245	2.2721677	2.9658499	2.8517093
HERV-FRD	NC_000006	11102722	11111959	0.0275365	0	0	0.0095943
NEDD9	NC_000006	11183531	11382581	1.8574109	1.8286979	0.1610394	0.3071263
LOC100129074	NC_000006	11514461	11516608	0	0	0	0
TMEM170B	NC_000006	11538511	11583757	0.4309681	0.2431539	1.8608982	1.2891601
C6orf105	NC_000006	11713888	11779280	0.3074957	0.2675013	0.1683426	0.3791024
LOC389369	NC_000006	11810835	11811481	0.3966447	0	0	0
LOC100129761	NC_000006	11861850	11935836	0	0	0	0
HIVEP1	NC_000006	12012724	12165232	1.2034506	1.2830986	1.7799658	1.259644
EDN1	NC_000006	12290529	12297427	1.4440133	1.8718838	0.2978862	0.1458328
RPL15P3	NC_000006	12514306	12515007	0	0	0	0
LOC100289173	NC_000006	12514307	12514962	0.8789646	0.7530567	1.49874	1.8374927
PHACTR1	NC_000006	12717833	13287528	0.1635283	0.0630466	0.5455474	0.1281972
RNU1P5	NC_000006	13214272	13214487	0	0	0	0
LOC100130357	NC_000006	13295024	13295543	0	0	0	0.2126728
TBC1D7	NC_000006	13305183	13328770	2.9116132	3.7682182	3.2606699	6.922225
GFOD1	NC_000006	13363819	13487787	1.0862288	0.9658443	0.3395246	0.2557188
LOC100128648	NC_000006	13365054	13486988	0.3279719	0.5057843	0.1458864	0.1142719
C6orf114	NC_000006	13469509	13486415	0	0	0	0
RPS4P7	NC_000006	13521445	13522348	0	0	0	0
SIRT5	NC_000006	13574792	13614790	0.9122752	0.6195401	2.040132	1.1022857
NOL7	NC_000006	13615559	13621127	2.5142009	2.7916518	2.6840434	2.2075141
RANBP9	NC_000006	13621730	13711796	6.4235289	5.0945628	7.6110263	8.6080207
CCDC90A	NC_000006	13791020	13814789	9.6550224	7.9411024	12.120563	17.717078
MRPL35P1	NC_000006	13949326	13949697	0	0	0	0
RNF182	NC_000006	13974437	13980236	1.6872018	1.5819741	3.2301121	6.3864534
CD83	NC_000006	14117865	14137149	0.4965902	0.3464425	0.8362272	0.7044464
RPL6P17	NC_000006	15103177	15104442	0	0	0	0
JARID2	NC_000006	15246527	15522253	9.6508973	4.599978	2.8256753	5.6929499
DTNBP1	NC_000006	15523038	15663271	0.894667	0.7133233	0.9096235	1.7685326
LOC441131	NC_000006	15935013	15935548	0	0	0	0
LOC644906	NC_000006	16107853	16108832	0	0	0	0
MYLIP	NC_000006	16129317	16148479	1.0157306	0.5589093	2.5072332	1.7944265
LOC100131159	NC_000006	16161165	16162898	0	0	0	0
MRPL42P2	NC_000006	16171837	16172265	0	0	0	0
GMPR	NC_000006	16238811	16295780	7.2811922	3.9069476	7.0453039	4.3258904
ATXN1	NC_000006	16299343	16761721	0.9710262	1.5505774	1.2608561	1.7362544
LOC100130360	NC_000006	16760665	16762883	0.1793805	0.2305276	0.3191638	0.3749985
FLJ23152	NC_000006	17128169	17131603	0	0	0	0.0293764
RBM24	NC_000006	17281809	17294102	0.2417394	0.347947	0.6308371	0.6288937
CAP2	NC_000006	17393736	17558023	3.4119044	2.5077012	3.4744781	4.9574464
RPL7P26	NC_000006	17531059	17531588	0	0	0	0
LOC100289078	NC_000006	17582265	17582536	0	0	0	0
FAM8A1	NC_000006	17600518	17611950	2.102476	1.9235084	2.8756692	2.8204577
NUP153	NC_000006	17615269	17706818	5.1853813	6.514927	10.209229	13.14495
KIF13A	NC_000006	17760585	17987799	2.1008669	2.6422225	4.2460537	6.7276187
RPS12P12	NC_000006	17953800	17954173	0	0	0	0
NHLRC1	NC_000006	18120718	18122851	0.8031776	0.8045779	0.3114614	0.1291583
TPMT	NC_000006	18128542	18155374	2.4746235	2.5580757	2.8270547	3.345179
AOF1	NC_000006	18155619	18224084	1.4857338	1.2126642	1.1401355	1.7940251

DEK	NC_000006	18224400	18264799	21.338849	27.053385	33.160074	45.921317
DDX18P3	NC_000006	18363327	18365692	0	0	0	0
IMPDH1P9	NC_000006	18366247	18368877	0	0	0	0
RNF144B	NC_000006	18387594	18468850	0.8479944	0.3221971	1.5251987	0.8028754
RPL21P61	NC_000006	19143884	19144426	0	0	0	0
RPL5P20	NC_000006	19348341	19349350	0	0	0	0
KRT18P38	NC_000006	19612919	19614321	0	0	0	0
LOC100130994	NC_000006	19638271	19644239	0	0	0	0
ID4	NC_000006	19837617	19840915	0.9374623	0.5782859	0.6004746	0.7839133
LOC100287917	NC_000006	19838755	19839452	0	0	0	0.0877504
RPL29P17	NC_000006	20042573	20043216	0	0	0	0
MBOAT1	NC_000006	20100935	20212670	0.3086441	0.3725044	0.4536511	0.5330131
E2F3	NC_000006	20402137	20493945	1.5880675	1.6597616	2.5679974	3.099783
CDKAL1	NC_000006	20534688	21231764	2.6098825	3.4150246	3.6694559	4.8201117
RPL36AP25	NC_000006	20722559	20722800	0	0	0	0
SOX4	NC_000006	21593972	21598850	0.2792366	0.3148669	0.25643	0.1757525
FLJ22536	NC_000006	21666675	22194629	0	0	0	0
LOC100133113	NC_000006	22213592	22224199	0	0	0	0
PRL	NC_000006	22287480	22297730	0	0	0	0.0226181
HDGFL1	NC_000006	22569678	22570750	0.1228748	0.0421094	0	0
LOC389370	NC_000006	22643793	22718153	0	0	0	0
RPL6P18	NC_000006	23102887	23103805	0	0	0	0
LOC100129616	NC_000006	23854367	23857874	0	0	0	0
LOC100128365	NC_000006	23941932	23973169	0	0	0	0
LOC100289286	NC_000006	23981619	24002657	0	0	0	0
NRSN1	NC_000006	24126414	24147757	0.0182509	0	0.0162365	0
DCDC2	NC_000006	24171983	24358280	0.0093348	0	0.0249135	0.0065049
KAAG1	NC_000006	24357131	24358512	0	0	0.0565811	0.0221598
MRS2	NC_000006	24403153	24425816	2.8747499	3.0635102	6.6797514	5.8267764
GPLD1	NC_000006	24428405	24489850	0.0327239	0.0224291	0.0679283	0.0456067
ALDH5A1	NC_000006	24495197	24537435	0.1785131	0.2534465	0.6503657	0.4146502
KIAA0319	NC_000006	24544332	24646383	0.1937468	0.1394345	1.6948981	1.6561286
LOC100289319	NC_000006	24570034	24600017	0	0	0	0
TTRAP	NC_000006	24650205	24667115	6.8947356	6.2435246	12.247185	15.924937
ACOT13	NC_000006	24667275	24701942	6.7795649	7.7038006	13.808477	14.545781
C6orf62	NC_000006	24705089	24719403	35.169248	27.49738	52.014149	34.55051
LOC100289381	NC_000006	24750900	24751846	0	0	0	0
GMNN	NC_000006	24775164	24786278	12.406785	15.581766	20.755498	26.743206
FAM65B	NC_000006	24804513	24911195	0.1580613	0.7829721	0.051133	0.1101434
LOC100289422	NC_000006	24948066	24948648	0	0	0	0
LOC134997	NC_000006	24976605	24977415	0	0	0	0
LOC442167	NC_000006	25023476	25024712	0	0	0	0
CMAH	NC_000006	25081295	25138051	0	0	0	0
NUP50P2	NC_000006	25139971	25141866	0	0	0	0
LOC100132239	NC_000006	25152775	25153753	0	0	0	0
LOC100129757	NC_000006	25218913	25221323	0	0	0	0
RPL21P68	NC_000006	25261427	25261986	0	0	0	0
LOC100128495	NC_000006	25272429	25273979	0	0	0	0
LRRC16A	NC_000006	25279648	25620758	0.6711551	0.5622369	0.3022248	0.6640009
SCGN	NC_000006	25652429	25702011	0.0296547	0.0304881	0	0.0413291
HIST1H2AA	NC_000006	25726291	25726790	0.0878965	0	0	0
HIST1H2BA	NC_000006	25727137	25727573	0	0	0	0.0700798
LOC100288742	NC_000006	25732011	25732390	0	0	0	0
LOC100129474	NC_000006	25732462	25733054	0	0	0	0
SLC17A4	NC_000006	25754927	25781403	0	0.0125509	0	0.0170138
SLC17A1	NC_000006	25783125	25832287	0.0238331	0.0490059	0.0212026	0.0166079
SLC17A3	NC_000006	25845328	25874471	0.6076913	0.7247338	0.086499	0.1016312
HIST1H2APS2	NC_000006	25882154	25882644	0	0	0	0
SLC17A2	NC_000006	25912982	25930839	0.0387209	0	0.0172236	0.0539645
TRIM38	NC_000006	25963071	25985358	3.367218	1.4206015	3.5803632	2.0379193
HIST1H1PS2	NC_000006	26016335	26017069	0	0	0	0
HIST1H1A	NC_000006	26017260	26018040	1.7444241	7.5209248	5.3565167	11.920567
HIST1H3A	NC_000006	26020718	26021186	4.1230751	8.574249	9.0032774	12.602562
HIST1H4A	NC_000006	26021907	26022278	12.995445	17.733271	6.306059	15.312439
HIST1H4B	NC_000006	26027124	26027480	5.6627974	17.212725	8.9803932	29.166551
HIST1H3B	NC_000006	26031817	26032288	44.320674	116.78761	82.585324	107.70614

HIST1H2AB	NC_000006	26033320	26033796	12.806717	30.501164	23.360181	53.994806
HIST1H2BB	NC_000006	26043455	26043885	0.8157444	2.0966776	7.8013704	7.5318726
HIST1H2APS5	NC_000006	26044128	26044778	0	0	0	0
HIST1H3C	NC_000006	26045639	26046097	37.341635	90.268365	81.602327	63.251383
HIST1H1C	NC_000006	26055915	26056699	7.6248981	29.566734	31.406241	19.035956
HFE	NC_000006	26087448	26097059	4.1337446	4.0465783	4.6452553	3.7902077
HIST1H4C	NC_000006	26104161	26104565	36.510839	98.940065	79.297883	243.89967
HIST1H1T	NC_000006	26107640	26108364	0	0	0.161783	0.1267236
HIST1H2BC	NC_000006	26123695	26124132	27.492729	71.076173	50.434074	62.508313
HIST1H2AC	NC_000006	26124373	26124918	51.916867	81.594935	121.51753	121.4899
HIST1H1E	NC_000006	26156559	26157343	13.380417	35.053111	48.112418	28.050048
HIST1H2BD	NC_000006	26158349	26171577	1.8597247	4.2768253	3.5266178	6.616065
HIST1H2BE	NC_000006	26184024	26184458	10.608194	29.810658	12.403366	16.40367
HIST1H4D	NC_000006	26188938	26189304	5.8677476	14.773864	8.0964986	11.682515
HIST1H3D	NC_000006	26197012	26199464	17.599639	47.641295	16.109645	20.38114
HIST1H2AD	NC_000006	26199012	26199471	0	0.2946744	0.2549841	0.0665758
HIST1H2BF	NC_000006	26199787	26200216	11.242571	18.598749	10.729099	15.38366
RPS10P1	NC_000006	26202351	26202943	0	0	0	0
HIST1H4E	NC_000006	26204873	26205249	1.6320298	3.2359466	2.2815556	1.8683613
HIST1H2BG	NC_000006	26216428	26216872	29.628021	56.961547	48.322834	62.970255
HIST1H2AE	NC_000006	26217148	26217711	18.54553	40.056207	19.479461	18.733303
HIST1H3E	NC_000006	26225383	26225844	5.0416803	0.4889979	4.9929792	8.5511024
HIST1H1D	NC_000006	26234440	26235216	27.488855	33.495032	82.371576	58.766658
HIST1H4F	NC_000006	26240654	26241021	4.5381326	0.7366859	8.7119576	19.889527
HIST1H4G	NC_000006	26246839	26247205	0.11975	0	0.1065329	0
HIST1H3F	NC_000006	26250370	26250835	72.995561	15.222734	101.01603	113.89037
HIST1H2BH	NC_000006	26251879	26252303	8.5828312	0.5315694	23.366545	30.55282
HIST1H3G	NC_000006	26271146	26271612	20.327234	23.607602	34.576648	32.920105
HIST1H2APS4	NC_000006	26272421	26272768	0	0	0	0
HIST1H2BI	NC_000006	26273204	26273640	5.8329461	15.715966	16.372665	16.468756
HIST1H4H	NC_000006	26285354	26285727	26.674462	42.404744	42.338273	76.80785
LOC100289545	NC_000006	26322104	26343616	0	0	0	0
BTN3A2	NC_000006	26365398	26378548	6.260202	2.8091464	16.266721	5.663835
BTN2A2	NC_000006	26383354	26395102	2.058725	1.3181133	4.5732636	2.7489372
BTN3A1	NC_000006	26402465	26415444	4.3832882	2.9647902	10.028605	4.267461
BTN2A3	NC_000006	26422347	26431843	0.923387	0.3592093	0.8214707	1.1303902
BTN3A3	NC_000006	26440763	26453643	7.1627094	3.5667061	13.795228	4.2826897
BTN2A1	NC_000006	26458189	26469865	0.917647	0.8418366	1.6955257	2.7742421
BTN1A1	NC_000006	26501495	26510653	0.0462289	0.0475281	0.0411265	0.010738
HCG11	NC_000006	26521934	26527621	0	0	0	0
HMGN4	NC_000006	26538572	26547165	7.7450668	9.7985336	19.22115	13.935953
ABT1	NC_000006	26597180	26600278	1.7345422	2.664919	2.912812	2.5803667
ZNF322A	NC_000006	26634611	26659963	1.3459146	1.986187	2.0281862	2.5393128
POM121L6P	NC_000006	26838446	26865940	0	0	0	0
GUSBL1	NC_000006	26839266	26924333	0	0	0	0
LOC729400	NC_000006	26866104	26869446	0	0	0	0
C6orf41	NC_000006	26924772	26991753	0	0	0	0
LOC100270746	NC_000006	26987145	26988085	0	0	0	0
HIST1H2BJ	NC_000006	27100095	27100575	2.4669486	4.3210738	7.9658242	4.8388581
HIST1H2AG	NC_000006	27100821	27103071	6.5990681	11.581885	18.063735	31.522809
HIST1H2BK	NC_000006	27106073	27114619	34.837013	48.048691	32.565411	39.140088
HIST1H4I	NC_000006	27107088	27107457	0	0	0	0
HIST1H2AH	NC_000006	27114908	27115394	5.4059328	20.275923	18.969889	30.415597
RPL10P2	NC_000006	27179023	27179663	0	0	0	0
PRSS16	NC_000006	27215508	27224250	0	0	0.0432814	0.0113007
LOC100288875	NC_000006	27219367	27222904	0	0	0	0
LOC442172	NC_000006	27228786	27237011	0	0	0	0
TRNAI28P	NC_000006	27251864	27251937	0	0	0	0
TRNAS32P	NC_000006	27261671	27261744	0	0	0	0
POM121L2	NC_000006	27276845	27279610	0	0	0	0
FKSG83	NC_000006	27292578	27293742	0.0754476	0	0	0.0262874
ZNF204	NC_000006	27325602	27343153	0	0	0	0
LOC100128090	NC_000006	27356046	27357004	0.0916543	0.1413454	0.0407691	0
ZNF391	NC_000006	27356524	27369227	0.2202919	0.2264832	0.3674583	0.4029589
MCFD2L	NC_000006	27375491	27375775	0	0	0	0
ZNF184	NC_000006	27418521	27440897	1.7142927	1.9663956	3.705572	2.9321692

HNRNPA1P1	NC_000006	27491226	27492316	0	0	0	0
LOC100128240	NC_000006	27528602	27531117	0	0	0	0
RPL8P1	NC_000006	27620370	27620512	0	0	0	0
LOC100289652	NC_000006	27647787	27670181	1.478207	1.0521361	2.0737389	1.9016742
GPR89P	NC_000006	27704377	27706273	0	0	0	0
LOC100131289	NC_000006	27729523	27730966	0	0.0545693	0.4721928	0.0739731
RSL24D1P	NC_000006	27748462	27748890	0	0	0	0
HIST1H4PS1	NC_000006	27774945	27775115	0	0	0	0
HIST1H2BL	NC_000006	27775257	27775709	8.4403889	27.828188	23.303185	26.298185
HIST1H2AI	NC_000006	27775977	27776445	3.5608375	6.8401312	5.0018208	8.4887722
HIST1H3H	NC_000006	27777842	27778314	34.378109	132.39788	86.626319	69.472504
HIST1H2AJ	NC_000006	27782080	27782518	11.412525	35.508596	18.791768	32.020089
HIST1H2BM	NC_000006	27782822	27783267	4.7298545	14.892287	26.737124	28.564909
HIST1H4J	NC_000006	27791903	27792258	0.3703503	0.2538393	1.4277201	0.6881995
HIST1H4K	NC_000006	27798952	27799305	0.2482951	3.5738284	1.104451	1.8167301
HIST1H2AK	NC_000006	27805626	27806124	8.7896464	19.546733	16.403979	10.851859
HIST1H2BN	NC_000006	27806364	27806894	21.533655	41.761942	35.875717	39.832804
HIST1H2BPS2	NC_000006	27831840	27832179	0	0	0	0
HIST1H2AL	NC_000006	27833107	27833576	21.506582	50.663091	48.24806	37.987881
HIST1H1B	NC_000006	27834570	27835425	17.134247	49.186994	47.263513	90.789196
HIST1H3I	NC_000006	27839623	27840099	18.058393	23.870476	37.54022	48.858558
HIST1H4L	NC_000006	27840926	27841289	3.8635808	3.7239068	7.9484062	14.386962
LOC100288931	NC_000006	27846372	27846596	0	0	0	0
HIST1H3J	NC_000006	27858093	27858570	14.250996	29.964725	21.348252	27.485508
HIST1H2AM	NC_000006	27860477	27860963	25.267977	54.368529	49.534287	35.52989
HIST1H2BO	NC_000006	27861203	27861669	15.43364	40.636036	44.288249	24.001511
RNU7-26P	NC_000006	27865284	27865343	0	0	0	0
OR2B2	NC_000006	27879024	27880097	0.3273611	0.5469127	0.4368443	0.2566331
OR2W6P	NC_000006	27905081	27906278	0	0	0	0
OR2B6	NC_000006	27925019	27925960	0	0.0479654	0.1660194	0.0975315
RPLP2P1	NC_000006	27932953	27933234	0	0	0	0
OR2W4P	NC_000006	27944828	27945950	0	0	0	0
LOC340192	NC_000006	27978275	27980579	0	0	0	0
OR2W2P	NC_000006	28001592	28002739	0	0	0	0
OR2B7P	NC_000006	28014112	28015246	0	0	0	0
OR2B8P	NC_000006	28020906	28022043	0	0	0	0
OR1F12	NC_000006	28040894	28042307	0	0	0	0
ZNF165	NC_000006	28046572	28057341	0.1334292	0.2057688	0.1928912	0.2440692
ZSCAN12L1	NC_000006	28058929	28063493	0	0	0	0
LOC100129195	NC_000006	28090630	28107454	0.1763472	0.1813034	0.3249726	0.4652102
ZSCAN16	NC_000006	28092387	28097857	0.2410953	0.2832815	0.2451258	0.1200034
ZNF192	NC_000006	28109716	28125236	1.4999117	1.7358786	3.419761	4.1236781
ZNF389	NC_000006	28129551	28137376	0.1339885	0.0275509	0.04768	0.1680634
LOC222701	NC_000006	28155716	28157189	0	0	0	0
LOC222699	NC_000006	28183116	28186707	0	0	0	0
ZNF193	NC_000006	28193070	28201260	0.8322512	1.0764525	0.573208	0.6734854
ZKSCAN4	NC_000006	28212490	28220002	0.5190736	0.810376	1.2656255	0.7234223
NKAPL	NC_000006	28227098	28228736	0	0	0	0
ZNF187	NC_000006	28234788	28245981	1.4665012	1.5718755	1.8736853	2.5221767
PGBD1	NC_000006	28249364	28270326	0.5161946	0.9876959	0.6250508	0.7993443
ZNF323	NC_000006	28292514	28324048	0.1207645	0.1241586	0.1074355	0.1332431
LOC100289039	NC_000006	28317690	28327768	0	0	0	0
ZKSCAN3	NC_000006	28317691	28334524	0.4595894	0.4134429	0.6644031	0.7072412
ZSCAN12	NC_000006	28346770	28367510	0.4886678	0.3167316	0.7466057	0.6070196
ZSCAN23	NC_000006	28400432	28411279	0.0556131	0.0714701	0.2102685	0.2325204
COX11P	NC_000006	28414703	28415884	0	0	0	0
OR2E1P	NC_000006	28423206	28424033	0	0	0	0
TRLP1	NC_000006	28446350	28446431	0	0	0	0
TRMEP1	NC_000006	28448503	28448575	0	0	0	0
GPX6	NC_000006	28471073	28483570	0.0256707	0	0	0.0536651
GPX5	NC_000006	28493789	28502728	0	0	0	0.0210915
SCAND3	NC_000006	28539407	28555112	0	0.0555875	0.0080167	0.0188384
LOC646160	NC_000006	28601157	28601372	0	0	0	0
TRNAA46P	NC_000006	28601859	28601930	0	0	0	0
TRNAK43P	NC_000006	28660987	28661060	0	0	0	0
TRNAF15P	NC_000006	28694855	28694927	0	0	0	0

RPSAP2	NC_000006	28699714	28700751	0	0	0	0
TRNAA44P	NC_000006	28746544	28746615	0	0	0	0
NOL5BP	NC_000006	28751410	28751781	0	0	0	0
RPL13P	NC_000006	28829177	28829793	0	0	0	0
TRIM27	NC_000006	28870779	28891768	6.2614018	11.06377	12.615516	11.738333
C6orf100	NC_000006	28911203	28912313	0	0	0	0
KRT18P1	NC_000006	28936847	28938244	0	0	0	0
ZNF311	NC_000006	28962594	28973037	0.1466027	0.535904	0.1883871	0.2156682
OR2AD1P	NC_000006	28994457	28995384	0	0	0	0
LOC100129636	NC_000006	29003798	29044517	0	0.0621505	0.0537793	0
OR2W1	NC_000006	29011990	29012952	0	0	0	0
OR2P1P	NC_000006	29039609	29040343	0	0	0	0
SAR1P1	NC_000006	29042193	29045018	0	0	0	0
OR2B3	NC_000006	29054084	29055025	0	0	0	0
OR2J1	NC_000006	29068720	29069655	0	0	0	0
OR2J3	NC_000006	29079668	29080603	0	0	0	0.0327189
OR2N1P	NC_000006	29105547	29106693	0	0	0	0
OR2J2	NC_000006	29141311	29142351	0	0	0	0
OR2J4P	NC_000006	29149287	29150219	0	0	0	0
OR2H4P	NC_000006	29183013	29183953	0	0	0	0
OR2G1P	NC_000006	29197007	29197997	0	0	0	0
OR2U1P	NC_000006	29230436	29231856	0	0	0	0
OR2U2P	NC_000006	29236242	29237198	0	0	0	0
OR2B4P	NC_000006	29258373	29259527	0	0	0	0
OR14J1	NC_000006	29274467	29275432	0	0	0	0
LOC442192	NC_000006	29297403	29298838	0	0	0	0
OR5V1	NC_000006	29323007	29324054	0	0	0.0373068	0
OR12D3	NC_000006	29341200	29343068	0	0	0	0
OR12D2	NC_000006	29364416	29365448	0	0	0	0
OR12D1P	NC_000006	29384942	29386073	0	0	0	0
OR11A1	NC_000006	29393281	29395509	0	0	0	0.0137393
OR10C1	NC_000006	29407793	29408731	0	0	0	0.0978431
OR2H1	NC_000006	29426230	29432099	0.0440069	0	0.0130499	0.0408877
LOC100286971	NC_000006	29432452	29432737	0	0	0	0
MAS1LP	NC_000006	29442802	29443848	0	0	0	0
MAS1L	NC_000006	29454543	29455679	0	0	0	0
RPS17P1	NC_000006	29456978	29457470	0	0	0	0
GPR53P	NC_000006	29505279	29506536	0	0	0	0
OR2I1P	NC_000006	29520882	29521940	0	0	0	0
UBD	NC_000006	29523389	29527702	0.0443922	0.0456398	0.1184775	0
OR2H5P	NC_000006	29541850	29542428	0	0	0	0
SNORD32B	NC_000006	29550029	29550105	0	0	0	0
RPL13AP	NC_000006	29550285	29550802	0	0	0	0
OR2H2	NC_000006	29555683	29556745	0	0.0850111	0	0
GABBR1	NC_000006	29570005	29600962	0.5666308	0.4766368	0.8248754	0.9931099
SUMO2P	NC_000006	29603231	29604281	0	0	0	0
MOG	NC_000006	29624809	29640149	0.0382991	0.0393755	0	0.0133442
ZFP57	NC_000006	29640260	29648887	0.0221961	0.0456398	0.0592387	0.0154671
HCP5P15	NC_000006	29680477	29682694	0	0	0	0
HCG4P11	NC_000006	29688955	29689944	0	0	0	0
HLA-F	NC_000006	29691117	29695073	32.948233	9.6061296	48.399931	8.8916755
LOC285830	NC_000006	29694378	29716826	0	0	0	0
RPL23AP1	NC_000006	29694409	29694931	0	0	0	0
MICE	NC_000006	29709234	29716880	0	0	0	0
HCG9P5	NC_000006	29716031	29716425	0	0	0	0
IFITM4P	NC_000006	29718584	29718925	0	0	0	0
3.8-1.5	NC_000006	29732894	29734070	0	0	0	0
HCP5P14	NC_000006	29738324	29740663	0	0	0	0
HCG4P10	NC_000006	29758257	29759346	0	0	0	0
HCG4	NC_000006	29758808	29760850	0	0	0	0
HLA-V	NC_000006	29759530	29760527	0	0	0	0
HCP5P13	NC_000006	29760911	29762023	0	0	0	0
HCG4P9	NC_000006	29766201	29767903	0	0	0	0
HLA-P	NC_000006	29767821	29770856	0	0	0	0
RPL7AP7	NC_000006	29770910	29771797	0	0	0	0
HCG2P8	NC_000006	29772896	29776439	0	0	0	0

MICG	NC_000006	29780167	29780469	0	0	0	0
HCP5P12	NC_000006	29784119	29786368	0	0	0	0
HLA-G	NC_000006	29794756	29798899	4.8181522	1.1739667	4.1129252	1.3197032
LOC100133214	NC_000006	29800562	29801089	0.0832353	0.0855746	0	0
HCG4P7	NC_000006	29853887	29854784	0	0	0	0
P5-09	NC_000006	29855512	29855661	0	0	0	0
HLA-H	NC_000006	29857607	29860349	0	0	0	0
P5-07	NC_000006	29860757	29861343	0	0	0	0
HLA-T	NC_000006	29864220	29866724	0	0	0	0
HCG2P7	NC_000006	29866808	29870438	0	0	0	0
LOC100287004	NC_000006	29874054	29876924	0	0	0	0
3.8-1.3	NC_000006	29878012	29879177	0	0	0	0
HCP5P6	NC_000006	29883529	29885891	0	0	0	0
HCG4P6	NC_000006	29892369	29893428	0	0	0	0
P5-05	NC_000006	29894219	29894350	0	0	0	0
HLA-K	NC_000006	29894235	29897486	0	0	0	0
HLA-U	NC_000006	29901540	29902656	0	0	0	0
P5.8	NC_000006	29903014	29903868	0	0	0	0
HCG4P5	NC_000006	29908689	29909578	0	0	0	0
P5-04	NC_000006	29910314	29910426	0	0	0	0
HLA-A	NC_000006	29910331	29913654	403.39851	114.77179	629.29075	137.97314
HCP5P3	NC_000006	29916002	29917301	0	0	0	0
HCG4P4	NC_000006	29922982	29923410	0	0	0	0
HLA-W	NC_000006	29923610	29926834	0	0	0	0
HCG2P6	NC_000006	29928106	29935197	0	0	0	0
MICD	NC_000006	29938148	29943521	0	0	0	0
HCG9	NC_000006	29942892	29946180	0.067302	0	0.1796213	0.0937975
3.8-1.2	NC_000006	29963733	29964188	0	0	0	0
HCP5P2	NC_000006	29968781	29971048	0	0	0	0
NCRNA00171	NC_000006	29968788	30028961	0	0	0	0
HCG4P3	NC_000006	29972622	29973605	0	0	0	0
HLA-J	NC_000006	29973748	29977733	0	0	0	0
ETF1P1	NC_000006	29999390	30001754	0	0	0	0
ZNRD1	NC_000006	30029036	30032686	2.6092664	3.8924002	4.5970362	3.9573475
PPP1R11	NC_000006	30034932	30038110	10.247544	9.205032	14.027192	17.815434
RNF39	NC_000006	30038043	30043628	0.0607579	0.0624655	0.1981904	0.1411285
TRIM31	NC_000006	30070674	30080867	0	0	0.0192884	0.0151085
TRIM40	NC_000006	30104510	30116512	0	0.0707095	0.0407904	0.0479263
TRIM10	NC_000006	30119722	30128711	0.0247875	0.0382262	0.0110258	0.0431823
TRIM15	NC_000006	30130983	30140473	0.0592827	0.0203163	0	0.0137702
TRIM26	NC_000006	30152232	30181153	1.9655746	1.5415209	3.2618669	4.3022335
HLA-L	NC_000006	30226648	30231555	0	0	0	0
FLJ45422	NC_000006	30227374	30234728	4.1102663	0.8963788	9.2651088	1.1884082
HCG18	NC_000006	30255174	30294927	0	0	0	0
TRIM39	NC_000006	30294621	30311506	2.2407246	1.994957	1.8495563	2.599694
RPP21	NC_000006	30312937	30314632	8.2508175	12.204712	15.279508	23.878018
HLA-N	NC_000006	30318851	30319815	0	0	0	0
UBQLN1P	NC_000006	30326371	30331888	0	0	0	0
LOC100129192	NC_000006	30364741	30387141	0	0	0	0
MICC	NC_000006	30382387	30387208	0	0	0	0
LOC100129772	NC_000006	30433220	30435814	0	0	0	0
LOC646520	NC_000006	30436659	30438028	0	0	0	0
RANP1	NC_000006	30453662	30454724	0	0	0	0
HLA-E	NC_000006	30457271	30461098	159.92996	67.053238	219.91802	78.138743
GNL1	NC_000006	30513694	30525008	5.3199722	6.7316808	7.0601856	9.9604692
PRR3	NC_000006	30524756	30532180	2.2585153	2.4651275	2.7799043	3.029071
ABCF1	NC_000006	30539170	30559309	3.793453	4.943565	4.7178933	10.11399
PPP1R10	NC_000006	30568182	30585020	6.9146255	8.0988173	6.3158272	5.1165707
LOC100287108	NC_000006	30568847	30570768	0	0.030223	0.0784567	0.0204849
MRPS18B	NC_000006	30585486	30594174	21.544374	21.973154	25.640255	23.896988
C6orf134	NC_000006	30594613	30612456	0.7091693	0.6874377	0.8832553	2.1743805
PTMAP1	NC_000006	30601227	30603024	0	0	0	0
C6orf136	NC_000006	30614801	30620984	0.7269985	0.9885376	1.543874	3.6115785
DHX16	NC_000006	30620907	30640757	2.4075875	3.3850757	1.8987269	3.237513
KIAA1949	NC_000006	30644166	30655672	13.254229	12.948842	13.423583	12.871905
NRM	NC_000006	30655826	30658769	6.3491103	6.8778117	6.3371671	13.877235

RPL7P4	NC_000006	30664523	30665313	0	0	0	0
MDC1	NC_000006	30667584	30685458	1.3659263	1.4349777	4.4202324	7.1158783
TUBB	NC_000006	30688157	30693199	196.98068	299.04254	300.26679	467.73437
LOC100287146	NC_000006	30691146	30693972	9.1695268	11.784047	6.7590426	17.252167
FLOT1	NC_000006	30695511	30710453	45.605296	50.911571	72.768475	56.430104
IER3	NC_000006	30710976	30712327	41.360957	75.900828	36.417491	36.873341
DDR1	NC_000006	30851861	30867933	3.8800741	3.3604655	4.24307	4.6638444
GTF2H4	NC_000006	30875977	30881880	1.9464252	3.4756463	4.2834163	3.2302465
VARS2	NC_000006	30881982	30894233	0.6586836	0.6216881	0.6244083	1.602727
SFTA2	NC_000006	30899127	30899952	0	0	0	0
DPCR1	NC_000006	30919559	30921998	0.0223201	0.0229474	0.0198566	0.0155535
MUC21	NC_000006	30951485	30957675	0.0120209	0.0123587	0	0.0167532
LOC100287207	NC_000006	30951497	30955400	0.0267488	0	0	0
LOC729792	NC_000006	30993965	31013075	0.0090821	0	0.024239	0.0126575
HCG22	NC_000006	31021984	31027653	0	0	0	0
C6orf15	NC_000006	31079000	31080332	0	0	0	0.0269585
PSORS1C1	NC_000006	31082623	31107837	1.8375567	0.4198225	1.8163794	0.7113793
CDSN	NC_000006	31082870	31088223	0	0	0	0.0120145
PSORS1C2	NC_000006	31105311	31107127	0	0	0	0
CCHCR1	NC_000006	31110216	31126015	3.3650131	3.8622116	5.3807541	8.4698508
TCF19	NC_000006	31126303	31131992	19.448355	28.618888	33.456447	47.549153
POU5F1	NC_000006	31132114	31138451	0.1095966	0.0563384	0.3168755	0.1527425
PSORS1C3	NC_000006	31141512	31145676	0	0	0	0
HCG27	NC_000006	31165537	31171745	0	0	0	0
HLA-C	NC_000006	31236529	31239855	346.77316	106.12914	468.94005	114.46676
LOC100287272	NC_000006	31243352	31246531	0	0	0	0
RPL3P2	NC_000006	31248108	31249348	0	0	0	0
WASF5P	NC_000006	31255162	31256941	0	0	0	0
HLA-B	NC_000006	31321649	31324989	627.38058	167.54074	787.52247	154.21535
DHFRP2	NC_000006	31331244	31334737	0	0	0	0
HLA-S	NC_000006	31349346	31350264	0	0	0	0
MICA	NC_000006	31371371	31383090	19.639869	27.970677	28.356476	23.692214
HLA-X	NC_000006	31429623	31430267	0	0	0	0
HCP5	NC_000006	31430959	31433482	3.9739462	0.2228528	9.0793771	0.4405552
HCG26	NC_000006	31439006	31440185	0	0	0	0
MICB	NC_000006	31465855	31478901	9.3100161	8.4434549	16.10826	13.123186
LOC100287303	NC_000006	31483885	31484339	0	0	0	0
PPIAP9	NC_000006	31486654	31488179	0	0	0	0
RPL15P4	NC_000006	31495853	31496498	0	0	0	0
MCCD1	NC_000006	31496739	31498008	0.1239724	0.1274567	0	0.3023613
BAT1	NC_000006	31497996	31510225	29.401841	31.986375	29.639364	45.901423
SNORD117	NC_000006	31504151	31504226	0	0	0	0
SNORD84	NC_000006	31508878	31508955	0	0	0	0
ATP6V1G2	NC_000006	31512239	31514627	0.1654262	0.0850378	0.2698075	0.1729134
NFKBIL1	NC_000006	31514628	31526606	7.1970018	6.5552507	9.5187722	10.259144
LOC100289233	NC_000006	31525942	31551339	0.8208721	0.9088615	0.4493973	1.7600505
LOC100287329	NC_000006	31527070	31529090	0	0.03099	0	0
LTA	NC_000006	31540093	31542098	0	0	0.0282089	0
TNF	NC_000006	31543350	31546112	0	0	0	0
LTB	NC_000006	31548335	31550202	0	0	0	0
LST1	NC_000006	31553971	31556686	0	0.053662	0.0928683	0.0363716
NCR3	NC_000006	31556660	31560762	0	0.0738291	0	0
LOC100130756	NC_000006	31578860	31579133	0	0	0	0
AIF1	NC_000006	31582994	31584798	0	0.029113	0.0251917	0
BAT2	NC_000006	31588450	31605554	10.901246	14.462087	19.509199	18.421879
SNORA38	NC_000006	31590856	31590987	0	0	0	0
BAT3	NC_000006	31606805	31620477	27.80172	28.047229	40.942442	44.132899
APOM	NC_000006	31623671	31625987	0.2887532	0.5343635	0.5651422	0.8048588
C6orf47	NC_000006	31626075	31628549	2.7345566	3.2678097	2.9856323	5.1474543
BAT4	NC_000006	31629862	31633163	4.1932683	4.477849	6.1830626	8.0234921
CSNK2B	NC_000006	31633657	31637843	24.545555	31.484179	39.062905	30.217633
LY6G5B	NC_000006	31638728	31640227	1.4195165	1.2842827	0.9597594	3.007094
LY6G5C	NC_000006	31644461	31648141	0.0612946	0.4411211	0.3817057	0.3417002
BAT5	NC_000006	31654736	31671088	8.655111	6.0398221	6.8221262	6.3437248
LY6G6F	NC_000006	31674684	31678372	0	0.1006312	0	0
LY6G6E	NC_000006	31679753	31681842	0	0	0	0

LY6G6D	NC_000006	31683133	31685581	0	0	0	0
LY6G6C	NC_000006	31686425	31689510	0	0	0.0444291	0.034801
C6orf25	NC_000006	31691161	31692851	0	0.0519349	0	0
DDAH2	NC_000006	31694817	31698039	7.1651137	12.696163	7.5198924	8.0301146
CLIC1	NC_000006	31698358	31704341	103.0377	153.82583	169.42278	146.36782
MSH5	NC_000006	31707774	31730453	2.1368912	1.7360505	3.974897	3.4363175
C6orf26	NC_000006	31730773	31732627	0.7684937	0.4444269	1.7519128	2.4098265
C6orf27	NC_000006	31733371	31745108	0.2111446	0.124045	0.456183	0.3573253
VARS	NC_000006	31745295	31763712	9.6002425	11.837757	9.9974679	12.252804
LSM2	NC_000006	31765173	31774743	7.6320356	14.692505	21.052535	20.630746
HSPA1L	NC_000006	31777396	31782835	0.398427	0.2137173	0.4469174	0.6156361
HSPA1A	NC_000006	31783291	31785719	3.3110442	5.5804943	9.5772135	5.6357845
LOC100287365	NC_000006	31786223	31797340	0	0	0	0
HSPA1B	NC_000006	31795512	31798031	2.6682855	3.4604748	12.411926	3.7551934
C6orf48	NC_000006	31802693	31807541	8.5077931	8.9078916	20.291729	19.822516
SNORD48	NC_000006	31803040	31803102	0	0	0	0
SNORD52	NC_000006	31804853	31804916	0	0	0	0
NEU1	NC_000006	31826829	31830709	23.024544	20.704514	31.716036	25.952034
LOC100287406	NC_000006	31829802	31830652	0.207303	0	0	0
SLC44A4	NC_000006	31830969	31846823	0.0667399	0.0686156	0	0.0348803
EHMT2	NC_000006	31847536	31865464	3.8875578	3.9284964	3.5570114	7.1236801
ZBTB12	NC_000006	31867394	31869769	0.6833773	0.944854	1.1739752	2.0526058
C2	NC_000006	31895266	31913449	2.2066763	1.1430067	2.6824317	1.2090312
CFB	NC_000006	31913721	31919861	21.647892	3.6291198	140.6292	8.2303632
RDBP	NC_000006	31919864	31926864	20.608652	28.221535	22.717163	26.720746
SKIV2L	NC_000006	31926581	31937532	7.2087747	8.4840776	10.14474	12.088381
DOM3Z	NC_000006	31937588	31940032	2.4047146	2.3586304	3.6146806	5.7782789
STK19	NC_000006	31938952	31949223	2.531332	2.8239626	3.6174831	3.0775
C4A	NC_000006	31949834	31970457	1.020545	0.1998529	3.2641351	1.0949551
CYP21A1P	NC_000006	31972719	31976761	0	0	0	0
TNXA	NC_000006	31976197	31980800	0	0	0	0
STK19P	NC_000006	31981047	31981961	0	0	0	0
C4B	NC_000006	31982572	32003195	0.4615901	0.4495861	3.6165428	0.6997392
CYP21A2	NC_000006	32006082	32009419	0	0	0	0.0144867
TNXB	NC_000006	32008932	32077153	2.1584398	2.5338003	0.6176107	0.514556
ATF6B	NC_000006	32083045	32096017	11.457452	9.931367	39.022838	41.488749
FKBPL	NC_000006	32096484	32098067	3.1391594	3.3282417	3.3454018	3.0761596
PRRT1	NC_000006	32116140	32119720	0.208615	0.0953236	0.3918005	0.4199614
PPT2	NC_000006	32121301	32131452	2.0123164	3.1423446	2.2799876	4.1141845
EGFL8	NC_000006	32132405	32136062	0.8658044	0.7121104	1.0167216	1.8823802
AGPAT1	NC_000006	32135989	32145842	14.050435	14.386364	31.206626	36.08647
RNF5	NC_000006	32146162	32148570	12.214284	14.624003	36.037158	31.675336
AGER	NC_000006	32148746	32152023	0.6400228	0.7520122	0.8405163	0.9557001
PBX2	NC_000006	32152510	32157963	7.6187878	8.7751145	14.492748	10.303608
GPSM3	NC_000006	32158543	32163300	1.1168163	1.4895627	1.2083726	0.4206714
NOTCH4	NC_000006	32162620	32191844	0.0521254	0.0803856	0.0753548	0.059025
C6orf10	NC_000006	32260475	32339656	0.0204601	0	0.0364037	0.0142574
HNRNPA1P2	NC_000006	32293175	32294298	0	0	0	0
BTNL2	NC_000006	32362513	32374900	0	0	0	0
HLA-DRA	NC_000006	32407647	32412823	0.3510242	0.036089	3.9971952	0.6604407
LOC100289398	NC_000006	32409847	32411243	0	0	0	0
HLA-DRB9	NC_000006	32427597	32427866	0	0	0	0
HLA-DRB5	NC_000006	32485151	32498006	0.2251831	0	2.4707258	0.0523055
HLA-DRB6	NC_000006	32520490	32527779	0	0	0	0
HLA-DRB1	NC_000006	32546546	32557562	0.9422863	0.0387508	14.150234	0.6566226
HLA-DQA1	NC_000006	32605183	32611429	0.0285008	0	0.1267755	0.0595815
HLA-DQB1	NC_000006	32627657	32634466	0.0359054	0.0369145	0.0638849	0.0250203
LOC100287443	NC_000006	32699198	32703009	0	0	0	0
HLA-DQA2	NC_000006	32709163	32715221	0	0	0.0686324	0.0179198
HLA-DQB2	NC_000006	32723875	32731330	0	0	0	0
HLA-DOB	NC_000006	32780540	32784825	0.3203224	0.065865	0.5414386	0.6026762
TAP2	NC_000006	32789610	32806547	5.5033309	3.7891028	7.6165736	4.0634469
PSMB8	NC_000006	32808494	32812712	4.8335619	2.3955103	7.321146	3.3854913
TAP1	NC_000006	32812986	32821748	39.883914	18.780499	43.521206	8.5276592
LOC100289461	NC_000006	32820165	32821879	0.2596098	0.1525178	0.1649686	0.0775313
PSMB9	NC_000006	32821938	32827628	12.916083	7.2431408	16.825384	2.9514434

PPP1R2P1	NC_000006	32844255	32847851	0	0	0	0
HLA-Z	NC_000006	32864179	32864266	0	0	0	0
HLA-DMB	NC_000006	32902415	32908817	0.6472494	0.1330881	1.2667842	0.3608233
HLA-DMA	NC_000006	32916391	32920899	8.0689267	1.5302756	18.050391	7.7244568
BRD2	NC_000006	32936437	32949282	8.2799789	9.0523479	15.598355	20.772529
LOC100287516	NC_000006	32938023	32942504	0	0.1152638	0.0498693	0
HLA-DOA	NC_000006	32971959	32977389	0.2522137	0.2204068	0.0448753	0.0175753
HLA-DPA1	NC_000006	33032794	33041378	3.5705564	2.9289154	9.7321512	0.6087919
HLA-DPB1	NC_000006	33043760	33054976	3.1328853	2.5285848	5.105345	0.0816119
RPL32P1	NC_000006	33047076	33047788	0	0	0	0
HLA-DPA2	NC_000006	33059259	33061091	0	0	0	0
COL11A2P	NC_000006	33071570	33074821	0	0	0	0
HLA-DPB2	NC_000006	33080293	33096890	0	0	0	0
HLA-DPA3	NC_000006	33098974	33099120	0	0	0	0
COL11A2	NC_000006	33130469	33160245	0.0068402	0.112519	0.0182557	0.0857973
RXRB	NC_000006	33161365	33168432	6.9552854	9.5867392	11.504884	13.112774
SLC39A7	NC_000006	33168603	33172214	12.142177	16.257923	13.669809	21.839257
HSD17B8	NC_000006	33172419	33174607	0.5332445	0.1827438	0.6325188	0.2167585
LOC100287550	NC_000006	33175142	33176977	0.0532061	0.1094029	0.0946672	0.296609
RING1	NC_000006	33176286	33180499	7.2825608	9.2030635	5.9838622	6.9778204
LOC100289525	NC_000006	33179155	33180499	0.1249713	0.0856557	0.0741186	0.087085
VPS52	NC_000006	33218049	33239662	2.7402489	3.031621	3.351977	5.7285438
RPS18	NC_000006	33239852	33244281	458.21302	700.00927	1060.3952	640.24077
B3GALT4	NC_000006	33244917	33246602	0.4431316	0.1875942	0.0927582	0.2179707
WDR46	NC_000006	33246885	33256991	8.4887783	11.776407	8.1445296	7.0684316
PFDN6	NC_000006	33257378	33258711	6.5108492	8.0630313	14.612221	14.848426
RGL2	NC_000006	33259436	33266738	4.4552331	3.1519693	8.545035	10.418773
TAPBP	NC_000006	33267471	33281989	7.9950576	4.3070327	19.34319	11.59483
LOC100289558	NC_000006	33280790	33281732	0.0601207	0.1236208	0	0
ZBTB22	NC_000006	33282182	33285719	2.6732893	2.2351628	3.3237945	3.7257089
DAXX	NC_000006	33286335	33290793	0.97476	1.1749412	1.4353217	2.7872738
MYL8P	NC_000006	33306755	33307272	0	0	0	0
LYPLA2P1	NC_000006	33332501	33334139	0	0	0	0
RPL35AP4	NC_000006	33357133	33357256	0	0	0	0
KIFC1	NC_000006	33359313	33377701	0.7470875	0.9517568	1.3003625	2.7161755
RPL12P1	NC_000006	33367792	33368421	0	0	0	0
PHF1	NC_000006	33378794	33384216	13.009454	9.5364968	19.081688	19.5674
CUTA	NC_000006	33384319	33386065	37.423807	40.887399	59.966992	48.734168
SYNGAP1	NC_000006	33387847	33421466	2.3400057	1.623896	4.7359447	3.9389403
ZBTB9	NC_000006	33422356	33425321	1.3288182	1.8326601	1.6578982	1.9535782
BAK1	NC_000006	33540323	33548070	5.0300986	6.8609543	7.0243688	5.348531
GGNBP1	NC_000006	33551515	33556803	0	0	0	0
C6orf227	NC_000006	33553883	33561115	0.0208582	0	0	0.0290697
ITPR3	NC_000006	33589161	33664351	16.047992	17.08452	20.308737	26.897252
C6orf125	NC_000006	33665346	33679504	8.7722755	19.109186	22.021356	16.038721
IP6K3	NC_000006	33689443	33714762	0.0155679	0.0320109	0.0553986	0.032545
LEMD2	NC_000006	33738990	33756906	8.3993725	10.205517	10.340476	10.306828
MLN	NC_000006	33762449	33771793	0	0	0	0.05354
GRM4	NC_000006	33989628	34101443	0.0113298	0.0116482	0	0.0394752
KRT18P9	NC_000006	34157512	34158918	0	0	0	0
CYCSL1	NC_000006	34187149	34188714	0	0	0	0
HMGA1	NC_000006	34204577	34214008	86.596328	62.16483	79.824196	158.77723
C6orf1	NC_000006	34214157	34216904	11.916312	13.26279	10.957693	10.716168
RPL35P2	NC_000006	34231047	34231500	0	0	0	0
NUDT3	NC_000006	34255997	34360441	1.4606133	1.601775	1.8769142	2.7367875
RPS10	NC_000006	34385231	34393876	81.922918	150.83068	189.41442	138.20839
PACSIN1	NC_000006	34433905	34503000	0.0415193	0.0320147	0	0.0216993
SPDEF	NC_000006	34505580	34524091	0.0464078	0	0.0619286	0.0161694
LOC100101247	NC_000006	34543878	34544534	0	0	0	0
C6orf106	NC_000006	34555065	34664625	3.7792098	3.5377816	5.5828386	10.333038
RPL7P25	NC_000006	34584299	34585101	0	0	0	0
LOC100131607	NC_000006	34664763	34683717	0	0	0	0
RPS10P13	NC_000006	34711917	34712495	0	0	0	0
SNRPC	NC_000006	34725312	34741571	30.757598	44.929919	42.442519	66.91944
UHRF1BP1	NC_000006	34759794	34845291	1.0310703	0.7739293	3.697486	3.9580581
TAF11	NC_000006	34845555	34855819	2.6087991	4.5979196	5.5598626	6.2328519

ANKS1A	NC_000006	34857038	35059190	1.1833015	0.9675551	1.1388836	1.5961006
TCP11	NC_000006	35085849	35109187	0.0823386	0.0211632	0.1098761	0.1147536
SCUBE3	NC_000006	35182190	35218609	6.4697983	34.804028	0.2522424	0.5089178
ZNF76	NC_000006	35227510	35263760	4.9199491	3.9341748	5.4232583	9.9620316
DEF6	NC_000006	35265595	35289548	0.4593892	0.1771126	0.6981708	0.3734741
PPARD	NC_000006	35310335	35395955	6.7674823	5.4830697	7.1168173	9.9877505
MKRNP2	NC_000006	35413888	35417743	0	0	0	0
FANCE	NC_000006	35420138	35434881	2.442524	2.0867481	2.12703	2.2773882
RPL10A	NC_000006	35436178	35438558	87.040726	115.37792	216.80389	178.97877
TEAD3	NC_000006	35441374	35464861	9.9181065	10.272388	8.4051269	7.4744771
TULP1	NC_000006	35465651	35480647	0.1259262	0.1078878	0.0933562	0.0877504
RPS15AP19	NC_000006	35523623	35524081	0	0	0	0
FKBP5	NC_000006	35541362	35696360	0.7650003	0.646206	1.3537734	2.7086362
RPL36P9	NC_000006	35575370	35575757	0	0	0	0
LOC285847	NC_000006	35694539	35704724	0	0	0	0
C6orf81	NC_000006	35704859	35716685	0.3007578	0	0.2006719	0.20958
C6orf126	NC_000006	35744392	35747329	0	0	0	0
C6orf127	NC_000006	35748831	35755841	0	0	0	0.0642031
CLPS	NC_000006	35762760	35765102	0.1630732	0	0.1450745	0.056818
LHFPL5	NC_000006	35773071	35791852	0	0	0	0.0570561
SRPK1	NC_000006	35800811	35888956	10.782987	12.754132	21.086328	25.990065
SLC26A8	NC_000006	35911293	35992377	0.0740701	0.0380759	0.0439298	0.051615
DPRXP2	NC_000006	35957267	35958269	0	0	0	0
MAPK14	NC_000006	35995454	36079013	6.8022032	7.3017651	14.676651	14.617952
LOC100287611	NC_000006	35995454	36009375	0	0	0	0.0458456
MAPK13	NC_000006	36098262	36107842	9.6369534	14.598451	12.238698	11.224796
BRPF3	NC_000006	36164550	36200567	4.6407231	5.5213322	4.9983605	7.4083426
LOC100287642	NC_000006	36178104	36179407	0.0759037	0	0.135052	0
PNPLA1	NC_000006	36210945	36276372	0.0676387	0.0347698	0.0451299	0.0471333
C6orf222	NC_000006	36283534	36304662	0	0.0120941	0.0209302	0.0163945
ETV7	NC_000006	36333971	36355467	3.9191228	0.2500926	2.2842981	0.3013518
PXT1	NC_000006	36358328	36368311	0.0312799	0	0	0.0435941
KCTD20	NC_000006	36410544	36458319	5.8153898	4.1419619	5.072243	6.4020521
STK38	NC_000006	36461669	36515247	9.0575634	10.790443	6.450661	12.332166
SFRS3	NC_000006	36562090	36572244	10.721467	15.434788	13.965193	21.117919
LOC389386	NC_000006	36641528	36643048	0	0	0	0
CDKN1A	NC_000006	36646459	36655109	63.350704	26.971926	64.434154	43.797588
GPR166P	NC_000006	36704782	36705483	0	0	0	0
CPNE5	NC_000006	36708555	36807220	0.0394863	0	0.1053843	0.0091719
LOC100127961	NC_000006	36808556	36812209	0	0	0	0
PPIL1	NC_000006	36822605	36842800	4.3265099	6.5551049	6.820128	7.9691681
C6orf89	NC_000006	36853640	36896740	4.7522159	7.7436226	7.88206	12.261746
LOC100287733	NC_000006	36922149	36931054	0.1118276	0.0383235	0	0.1558518
PI16	NC_000006	36922209	36932613	0.0802707	12.977333	0.0357055	0.0559359
LOC100287080	NC_000006	36935686	36937792	0.126288	0.1817723	0.2471685	0.2464071
MTCH1	NC_000006	36935917	36953949	61.775186	118.35121	67.19959	104.16692
FGD2	NC_000006	36973423	36996845	0.0577317	0.0296771	0	0.0201149
COX6A1P2	NC_000006	37012610	37013157	0	0	0	0
RPL12P2	NC_000006	37059003	37059621	0	0	0	0
PIM1	NC_000006	37137922	37143204	11.323686	7.2720425	17.896265	14.006727
LOC100287767	NC_000006	37137928	37140896	0	0.0683561	0.1774473	0.1853245
TMEM217	NC_000006	37179957	37225372	1.0518011	0.4091641	0.6828165	1.1885464
TBC1D22B	NC_000006	37225548	37300746	1.7518359	1.3051243	3.0379102	3.7772452
RNF8	NC_000006	37321748	37362514	0.280969	0.2808416	0.4999156	0.7233367
FTSJD2	NC_000006	37400907	37449284	17.146631	13.015975	13.168409	13.931201
C6orf129	NC_000006	37450696	37467700	2.830804	1.8917366	6.2329452	10.356239
LOC100287124	NC_000006	37517779	37518636	0	0	0	0
MDGA1	NC_000006	37600284	37665766	0.1430896	0.1623295	0.0877906	0.2269274
LOC100287155	NC_000006	37617647	37619926	0.128316	0	0.0570767	0.0447079
ZFAND3	NC_000006	37787307	38122400	8.5286461	9.3239894	12.770621	10.756368
BTBD9	NC_000006	38136227	38607924	0.7509269	0.8062357	1.0570338	1.5466441
LOC100128379	NC_000006	38555099	38556305	0	0	0	0
GLO1	NC_000006	38643701	38670952	25.140879	33.197771	65.741974	67.488435
DNAH8	NC_000006	38690552	38998567	0.0151839	0.0405876	0.0567336	0.0952266
ZRF1PS	NC_000006	38730531	38734758	0	0	0	0
LOC100128655	NC_000006	39007444	39010968	0	0	0	0

GLP1R	NC_000006	39016557	39055520	0.0140724	0.0578718	0.0375577	0.0196125
C6orf64	NC_000006	39071840	39082865	1.9456248	1.2237171	2.260328	4.2906731
LOC100131031	NC_000006	39077166	39081406	0.0299681	0.0154052	0	0.0313245
KCNK5	NC_000006	39156747	39197251	0.0116173	0.0238876	0.0103351	0.0323816
KCNK17	NC_000006	39266777	39282237	0.0255959	0	0.0455417	0.1070176
KCNK16	NC_000006	39282474	39290330	0.0261441	0.0268789	0	0.0364365
KIF6	NC_000006	39302876	39693181	0.2987418	0.0341264	0.1378061	0.1233631
LOC100124373	NC_000006	39321621	39322274	0	0	0	0
LOC100131899	NC_000006	39521289	39522719	0	0	0	0
LOC100287824	NC_000006	39747043	39769324	0.0492693	0	0	0.1716641
DAAM2	NC_000006	39760793	39872641	1.4566912	0.1817514	0.9121717	1.5078379
MOCS1	NC_000006	39872046	39902254	1.1962122	0.9714638	0.6528185	1.1487832
RPL23P6	NC_000006	39926090	39926598	0	0	0	0
LOC442210	NC_000006	39960554	39968071	0	0	0	0
TDRG1	NC_000006	40346163	40347631	0	0	0	0
LRFN2	NC_000006	40359373	40555126	0.0139784	0.0287426	0.0248712	0.0292222
UNC5CL	NC_000006	40994771	41006928	0.0735658	0.1210135	0.0654462	0.071769
BZRPL1	NC_000006	41010293	41012076	0	0	0	0.063935
APOBEC2	NC_000006	41021013	41032255	0.0370558	0.0761946	0	0.2323979
C6orf130	NC_000006	41034531	41040188	0.7861268	1.1930881	2.4644121	2.3999053
NFYA	NC_000006	41040707	41070146	2.3885677	4.7079666	5.1111627	7.6525263
LOC221442	NC_000006	41068773	41108573	0	0	0	0
TREML1	NC_000006	41117342	41122070	0.0448451	0	0	0
TREM2	NC_000006	41126246	41130922	0.0418156	0	0	0
TREML2	NC_000006	41157552	41168925	0	0.0120233	0	0.0081492
TREML3	NC_000006	41176292	41185685	0	0	0	0
TREML4	NC_000006	41196062	41206120	0	0	0	0.0295893
TREML2P	NC_000006	41217115	41217327	0	0	0	0
TREM1	NC_000006	41243712	41254457	0.0477698	0	0.7649524	0.0332879
RPL32P15	NC_000006	41275696	41278043	0	0	0	0
NCR2	NC_000006	41303528	41318625	0	0	0	0.032545
FOXP4	NC_000006	41514164	41570122	0.6722624	0.7519174	1.537877	1.6730687
MDFI	NC_000006	41606195	41621982	0.0271789	0.0558855	0	0.056818
LOC100130606	NC_000006	41634644	41645461	0	0	0	0
TFEB	NC_000006	41651716	41702798	0.8027927	1.1516585	3.0394454	1.6782537
PGC	NC_000006	41704452	41715121	0	0	0	0.0452362
FRS3	NC_000006	41737914	41747630	0.6695714	1.0430148	1.0108327	0.8766124
PRICKLE4	NC_000006	41748500	41755110	0.4539003	0.7137111	0.3562962	0.5023522
TOMM6	NC_000006	41755181	41757634	27.48051	38.675722	43.217918	33.744743
USP49	NC_000006	41765383	41863099	0.0829995	0.1564424	0.0861451	0.2602681
MED20	NC_000006	41873092	41888877	4.5757239	5.9442248	5.7431452	7.1556919
BYSL	NC_000006	41888965	41900784	3.9531324	4.0188252	2.200466	6.7713299
CCND3	NC_000006	41902671	42016610	15.94325	14.097669	20.073203	20.703075
LOC100287341	NC_000006	41903634	42016700	0.5219505	0.643944	0.2321708	0.7274318
TAF8	NC_000006	42018251	42048644	0.6709654	0.6793711	0.8230114	1.3459928
C6orf132	NC_000006	42069985	42110182	1.5655639	1.3605709	1.0541953	2.3747691
GUCA1A	NC_000006	42123144	42147794	0	0.0711923	0	0
GUCA1B	NC_000006	42151022	42162694	0.1161627	0.0199046	0.086118	0.0269823
MRPS10	NC_000006	42174539	42185633	4.682335	7.8965385	12.386986	12.750825
TRERF1	NC_000006	42192669	42419783	2.0698574	1.8538309	2.2901476	4.0442616
HCRP1	NC_000006	42326762	42326920	0	0	0	0
RPL36AP5	NC_000006	42467416	42467817	0	0	0	0
UBR2	NC_000006	42532058	42658911	2.1523621	2.4015476	3.525312	5.1041464
PRPH2	NC_000006	42664333	42690358	0.0871125	0.0447804	0.0387488	0.040469
LOC442211	NC_000006	42694972	42695438	0	0	0	0
TBCC	NC_000006	42712234	42713884	1.4906729	2.2714854	2.4391576	1.9847741
FLJ38717	NC_000006	42750454	42751297	0	0	0	0
KIAA0240	NC_000006	42788794	42836296	1.5784937	1.1651284	3.4926759	3.6618235
RPL7L1	NC_000006	42847671	42854731	11.23696	17.825295	18.460184	19.311688
C6orf226	NC_000006	42858003	42858554	1.1146291	1.0641019	1.3457495	0.4993187
PTCRA	NC_000006	42883727	42893576	0.0811602	0	0.0361012	0
LOC100287530	NC_000006	42891913	42893583	0.0639712	0	0.0569106	0
CNPY3	NC_000006	42896860	42907008	9.1631441	9.4974743	14.99663	17.490015
RPL24P4	NC_000006	42924074	42924504	0	0	0	0
GNMT	NC_000006	42928500	42931618	0	0.4230656	0.4026903	0.1433749
PEX6	NC_000006	42931611	42946981	2.814295	2.5833849	4.1131801	2.3638414

PPP2R5D	NC_000006	42952330	42980080	8.5930633	13.075779	20.270849	29.348409
MEA1	NC_000006	42979965	42981618	42.479915	54.397068	51.124121	67.388845
KLHDC3	NC_000006	42981977	42989032	19.413003	23.132748	18.331536	23.209062
C6orf153	NC_000006	42989385	42997337	27.187992	38.20512	35.251903	41.115022
CUL7	NC_000006	43005355	43021654	4.5707446	4.4844795	5.0810399	5.60887
MRPL2	NC_000006	43021767	43027242	4.8527597	6.5702147	7.4182006	4.1912742
KLC4	NC_000006	43027372	43042833	1.7529603	2.7671996	2.1120544	2.0583304
PTK7	NC_000006	43044029	43129457	17.58343	12.856374	18.670031	9.7518146
SRF	NC_000006	43138920	43149244	2.0864723	2.2288246	1.8742927	3.8795295
CUL9	NC_000006	43149922	43192325	1.1537047	1.1396148	1.4691145	2.2778509
C6orf108	NC_000006	43193367	43197211	3.8599451	5.4991091	9.2225123	9.4141721
TTBK1	NC_000006	43211222	43255997	0.0678213	0	0.0164552	0.0300749
SLC22A7	NC_000006	43265998	43273276	0.017174	0	0.0152785	0.0119675
CRIP3	NC_000006	43273211	43276530	0.2298548	0.0945259	0.0817941	0
RPL34P14	NC_000006	43295590	43296214	0	0	0	0
ZNF318	NC_000006	43303808	43337181	2.3604471	3.3410659	5.0056214	6.3154727
RPS2P28	NC_000006	43331153	43331957	0	0	0	0
ABCC10	NC_000006	43399489	43418163	3.4896002	2.1668141	1.2986416	2.8710823
DLK2	NC_000006	43418090	43423786	0	0.1122569	0.0485684	0.0380433
TJAP1	NC_000006	43445311	43474291	4.572555	4.5515633	4.9734403	12.148619
C6orf154	NC_000006	43474707	43478424	0.39369	0.170423	0.2765033	0.0577555
YIPF3	NC_000006	43479565	43484702	30.933884	25.098651	30.218635	25.527318
POLR1C	NC_000006	43484791	43497114	2.1073162	3.1085176	2.7169955	3.9371803
XPO5	NC_000006	43490068	43543812	4.0638186	5.2899285	4.8106624	6.7826912
RPS2P29	NC_000006	43506522	43507463	0	0	0	0
POLH	NC_000006	43543878	43588260	1.4889736	1.6865892	1.9520896	1.9695743
GTPBP2	NC_000006	43588218	43596936	12.834831	8.1448428	26.117541	15.420382
MAD2L1BP	NC_000006	43597279	43608689	4.5565958	5.239057	6.788105	6.8577181
RSPH9	NC_000006	43612810	43638744	0.1921234	0.1481423	0.1709183	0.3346981
MRPS18A	NC_000006	43639042	43655528	16.833002	20.03415	21.835622	19.530583
LOC100132242	NC_000006	43673593	43674494	0	0	0	0
VEGFA	NC_000006	43737953	43754224	17.314762	15.971807	15.521359	16.682389
LOC100132354	NC_000006	43858765	43905944	0	0	0	0
C6orf223	NC_000006	43968339	43972887	0.1110204	0.0652232	0.0282191	0.0773635
RPL29P16	NC_000006	44056862	44057500	0	0	0	0
LOC652990	NC_000006	44059750	44061875	0	0	0	0
MRPL14	NC_000006	44081372	44095191	15.764656	13.117762	19.826249	21.536205
TMEM63B	NC_000006	44095376	44123256	9.6991096	6.2005356	10.486312	13.392999
CAPN11	NC_000006	44126548	44152139	0.0161101	0.0331257	0.1003237	0.0112261
SLC29A1	NC_000006	44187242	44201888	8.246117	8.1425919	15.901519	13.407853
HSP90AB1	NC_000006	44214849	44221614	70.580375	112.58465	141.43213	158.494
SLC35B2	NC_000006	44221838	44225283	31.668579	30.520945	33.424586	38.7765
NFKBIE	NC_000006	44225903	44233525	2.247643	1.7156038	5.1049514	2.1120606
TMEM151B	NC_000006	44238480	44247182	0.0475733	0.0586924	0.016929	0.0198906
TCTE1	NC_000006	44247902	44265425	0	0.1922698	0.0237675	0.0186169
AARS2	NC_000006	44266463	44281063	1.4153132	1.6440636	1.5616133	1.5562203
SPATS1	NC_000006	44310486	44344904	0.0973742	0	0.0866268	0.0678542
CDC5L	NC_000006	44355302	44414780	6.387143	7.5606893	7.1939521	11.218914
LOC100128935	NC_000006	44440641	44445639	0	0	0	0
SUPT3H	NC_000006	44796469	45345670	0.478298	0.9645682	0.8019174	1.5511136
RUNX2	NC_000006	45296054	45518819	0.3386863	0.7948161	1.1659184	1.8521664
CLIC5	NC_000006	45866188	46048085	0.0207401	0.0071077	0.018451	0.0144525
ENPP4	NC_000006	46097701	46114436	0.8639396	0.2606735	2.7067545	1.7079259
ENPP5	NC_000006	46127762	46138717	0.1642645	0.0460585	0.3586933	0.010406
ACTGP9	NC_000006	46172649	46174293	0	0	0	0
RCAN2	NC_000006	46188469	46293531	1.5364662	1.3072955	0.5420398	0.5353354
RPL36P10	NC_000006	46406047	46406350	0	0	0	0
CYP39A1	NC_000006	46517445	46620523	0.1354039	0	0.1376675	0.0808756
SLC25A27	NC_000006	46620679	46645927	0.0297048	0.0458095	0.1849834	0.4450388
LOC100131283	NC_000006	46638184	46656900	0	0	0.0307129	0.0481145
TDRD6	NC_000006	46655866	46670150	0.0128937	0.0132561	0.0229412	0.0359394
PLA2G7	NC_000006	46672053	46703151	0.0477958	0	0	0.066612
LOC100287718	NC_000006	46714660	46726942	0	0	0	0.0589507
MEP1A	NC_000006	46761094	46807519	0.030009	0.0308524	0.0133484	0.083646
GPR116	NC_000006	46820242	46922675	0.0218938	0.0225092	0.0129849	0.0457695
GPR110	NC_000006	46967813	47010082	0.0238202	0.0612241	0.2754842	0.1327908

TNFRSF21	NC_000006	47199268	47277680	0.2055916	0.5595083	1.5815471	1.3905077
CD2AP	NC_000006	47445525	47594999	1.0637876	0.717992	1.6543501	3.2084823
RPS12P14	NC_000006	47530271	47530779	0	0	0	0
GPR111	NC_000006	47624326	47658337	0	0.0403243	0.0697859	0.0409972
GPR115	NC_000006	47666289	47689757	0.0416966	0.2143425	0.0123648	0.0387411
RPL27AP7	NC_000006	47720567	47720970	0	0	0	0
OPN5	NC_000006	47749798	47794117	0.0118875	0.0122216	0.0423019	0.0331348
C6orf138	NC_000006	47846039	48036425	0.5632201	0.0701878	0.7136255	0.0832521
LOC389395	NC_000006	48116234	48117781	0	0	0	0
RBMXP1	NC_000006	48180653	48182705	0	0	0	0
LOC100287991	NC_000006	49045220	49083546	0	0	0	0
RNU7-65P	NC_000006	49312460	49312521	0	0	0	0
LOC442215	NC_000006	49325859	49328146	0	0	0	0
MUT	NC_000006	49398991	49431031	7.1612737	5.1782195	10.547619	11.015845
CENPQ	NC_000006	49431096	49460820	2.4924344	4.2882395	7.4891749	7.2131513
C6orf140	NC_000006	49467671	49495777	0	0.0212129	0	0.0143779
C6orf141	NC_000006	49518113	49519808	0.0518257	0.4262585	0.484109	0.6681135
RHAG	NC_000006	49572888	49604587	0.0227711	0.0234111	0	0.0158678
CRISP2	NC_000006	49660072	49681299	0	0	0	0.0217506
CRISP3	NC_000006	49695089	49712056	0	0	0.0371121	0.0581393
PGK2	NC_000006	49753364	49755007	0	0.0274838	0	0.0558848
CRISP1	NC_000006	49801970	49834218	0.069833	0.0239319	0.0207085	0
DEFB133	NC_000006	49913814	49917157	0	0	0	0
DEFB114	NC_000006	49928005	49931818	0.2092773	0	0.1861789	0
DEFB113	NC_000006	49936390	49937338	0	0	0	0
DEFB110	NC_000006	49976851	49989648	0	0	0	0
DEFB111	NC_000006	49986556	49989694	0	0	0	0
DEFB112	NC_000006	50011288	50016364	0	0	0	0
TFAP2D	NC_000006	50681257	50740746	0.086173	0.0664462	0.0574964	0.0600488
TFAP2B	NC_000006	50786439	50815326	0.0304667	0.031323	0.013552	0.0318456
RPS17P5	NC_000006	50824922	50825400	0	0	0	0
FTHP1	NC_000006	50880263	50881168	0	0	0	0
LOC646517	NC_000006	51274381	51275452	0	0	0	0
RPS15AP20	NC_000006	51401929	51402373	0	0	0	0
PKHD1	NC_000006	51480145	51952423	0.0454454	0.0389355	0.0224608	0.0633363
IL17A	NC_000006	52051185	52055436	0.0472816	0.0486104	0	0.0329477
IL17F	NC_000006	52101484	52109298	0.0543914	0	0	0
SLC25A20P	NC_000006	52111169	52112902	0	0	0	0
MCM3	NC_000006	52128812	52149582	5.5659284	7.9735415	7.7792971	15.317361
LOC647163	NC_000006	52226888	52268491	0.2053656	0.2815165	0.2435985	0.2862138
PAQR8	NC_000006	52226926	52272575	1.1223588	1.9168138	2.5415893	2.2493579
EFHC1	NC_000006	52284994	52360583	0.9841507	0.5747083	2.1923214	2.8638815
TRAM2	NC_000006	52362200	52441862	6.4580298	5.5830986	4.8975837	13.396486
LOC724104	NC_000006	52522014	52522794	0	0	0	0
LOC730101	NC_000006	52529199	52533951	0	0	0	0
TMEM14A	NC_000006	52535884	52551386	6.6680076	4.0954414	7.4728352	8.0861748
LOC100129499	NC_000006	52553459	52553744	0	0	0	0
LOC730152	NC_000006	52604261	52609957	0	0	0	0
GSTA2	NC_000006	52614885	52628361	0.1007217	0.0345175	0.0597365	0
LOC647169	NC_000006	52630072	52646188	0.0675088	0.0694061	0	0
GSTA1	NC_000006	52656178	52668664	0	0	0	0
GSTAP1	NC_000006	52670411	52678514	0	0	0	0
GSTA5	NC_000006	52696540	52710893	0	0	0.0925386	0.0724849
LOC647175	NC_000006	52712708	52735127	0	0	0	0
LOC647177	NC_000006	52737812	52755596	0	0	0	0
LOC100287869	NC_000006	52746874	52748971	0.0418954	0.0430728	0	0.0145972
GSTA3	NC_000006	52761437	52774496	0	0	0.0854592	0
GSTA4P	NC_000006	52804604	52822419	0	0	0	0
GSTA4	NC_000006	52842746	52860178	2.8512276	3.6052379	3.7027523	3.653975
RN7SK	NC_000006	52860418	52860749	0	0	0	0
ICK	NC_000006	52866098	52926600	1.2610987	1.2892987	0.8774702	3.0536509
FBXO9	NC_000006	52929796	52965671	3.3514606	3.1736285	2.3773956	4.6278413
GCM1	NC_000006	52991759	53013624	0	0.0326824	0.0424205	0.0332277
LOC647188	NC_000006	53041160	53041339	0	0	0	0
LOC100287893	NC_000006	53061518	53061957	0	0	0	0
LOC100130545	NC_000006	53071390	53073618	0	0	0	0

LOC100128227	NC_000006	53100419	53101041	0	0	0	0
ELOVL5	NC_000006	53132196	53213942	17.979496	27.247191	34.139493	39.104819
RPS16P5	NC_000006	53199263	53202814	0	0	0	0
RPL31P28	NC_000006	53219459	53219904	0	0	0	0
RPL31P33	NC_000006	53233468	53233824	0	0	0	0
LOC100130403	NC_000006	53243301	53243706	0	0	0	0
NANOGP3	NC_000006	53283266	53283954	0	0	0	0
GCLC	NC_000006	53362139	53409831	2.1873616	2.2853052	3.1660929	2.8672202
KLHL31	NC_000006	53512699	53530506	0.0765249	0.1573512	0.0612708	0.0533256
LRRC1	NC_000006	53659778	53788919	0.276404	0.4546757	0.9712917	0.9341551
C6orf142	NC_000006	53883714	54131078	0.1210695	0	0.1077068	0.0843661
TINAG	NC_000006	54173203	54254950	0	0	0.0216727	0.0339522
CLNS1B	NC_000006	54349297	54350651	0	0	0	0
RPL10P10	NC_000006	54467285	54467945	0	0	0	0
RPSAP44	NC_000006	54489325	54490321	0	0	0	0
KRAS1P	NC_000006	54635272	54640504	0	0	0	0
LOC100288434	NC_000006	54695035	54711414	0.1836277	0	0.0544534	0.042653
FAM83B	NC_000006	54711569	54806820	0.0555077	0.0570678	0.6049197	0.2127399
HCRTR2	NC_000006	55039071	55147418	0.0484011	0	0	0
GFRAL	NC_000006	55192267	55267291	0.045995	0.0472877	0.0613777	0.0641023
HMGCLL1	NC_000006	55299171	55444012	0	0	0	0
LOC100288467	NC_000006	55545270	55545822	0	0	0	0
BMP5	NC_000006	55620236	55740375	0.020234	0	0.0360014	0.0281997
LOC652423	NC_000006	55804585	55805422	0	0	0	0
COL21A1	NC_000006	55921388	56112378	0.2632892	0.1082756	0.1311684	0.1394375
LOC100288495	NC_000006	56141324	56141594	0	0	0	0
LOC442221	NC_000006	56296644	56313490	0	0	0	0
DST	NC_000006	56322785	56819413	1.8630876	2.0021083	2.6012467	11.891053
RPL17P26	NC_000006	56735947	56736573	0	0	0	0
LOC100288034	NC_000006	56758472	56759545	0	0	0	0
BEND6	NC_000006	56819773	56892144	0.6185307	1.0877486	0.6805873	1.3384206
FTHL15	NC_000006	56869325	56869600	0	0	0	0
MRPL30P1	NC_000006	56894294	56894664	0	0	0	0
KIAA1586	NC_000006	56911384	56920023	1.4871985	1.3417723	2.2275892	2.1995769
ZNF451	NC_000006	56954828	57035098	2.7504331	3.0260206	5.4382991	4.2188823
BAG2	NC_000006	57037104	57050013	20.336493	19.037332	21.596025	21.793934
RAB23	NC_000006	57053581	57087078	34.748792	14.803189	23.820623	13.90892
PRIM2	NC_000006	57182422	57513376	21.082107	18.006306	17.867809	17.484653
LOC100131935	NC_000006	57686969	57687941	0	0	0	0
LOC730180	NC_000006	57841107	57842390	0	0	0	0
GUSBL2	NC_000006	58246159	58287724	0	0	0	0
LOC727805	NC_000006	58254228	58287494	0	0	0	0
LOC100288096	NC_000006	58260183	58261352	0	0	0.0334167	0
LOC100288064	NC_000006	58272112	58275382	0.2284072	0.5801599	0.0836695	0.2996014
GAPDHP15	NC_000006	58293909	58294971	0	0	0	0
LOC100288628	NC_000006	58397997	58399034	0	0	0	0
LOC727842	NC_000006	58446019	58447307	0	0	0	0
KHDRBS2	NC_000006	62389865	62996100	0.0573238	0.019645	0.0679958	0.079891
LOC100131203	NC_000006	62664439	62668231	0	0	0	0
LOC100132056	NC_000006	63170845	63171408	0	0	0	0
LOC100288607	NC_000006	63257330	63258186	0	0	0	0
LOC442225	NC_000006	63902963	63903536	0	0	0	0
FKBP1C	NC_000006	63921352	63922932	0	0	0	0
LOC100128610	NC_000006	63938991	63940016	0	0	0	0
LGSN	NC_000006	63985856	64029882	0.0234016	0.0080198	0.0138792	0.0271786
LOC100133007	NC_000006	64105454	64110355	0	0	0	0
LOC642554	NC_000006	64150671	64153487	0	0	0	0
LOC442227	NC_000006	64190039	64191816	0	0	0	0
RPL7AP34	NC_000006	64258555	64259412	0	0	0	0
LOC727916	NC_000006	64280910	64283473	0	0	0	0
PTP4A1	NC_000006	64281920	64293489	9.1666914	10.428991	10.501412	14.378386
RPL9P18	NC_000006	64325641	64326298	0	0	0	0
PHF3	NC_000006	64356431	64424405	1.5499232	2.0097411	8.4307116	9.9584857
EYS	NC_000006	64429876	66417118	0.0190982	0.0425425	0.070793	0.2062804
LOC441155	NC_000006	66012315	66014950	0	0	0	0
LOC100289348	NC_000006	66497772	66499379	0	0	0	0

MCART3P	NC_000006	66497772	66499376	0	0	0	0
ADH5P4	NC_000006	66545470	66547961	0	0	0	0
NUFIP1P	NC_000006	66801368	66804842	0	0	0	0
RNU7-66P	NC_000006	67438634	67438897	0	0	0	0
LOC100289683	NC_000006	68591214	68599231	0	0.0561284	0	0.0887678
LOC100128757	NC_000006	68598930	68638449	0	0	0	0
LOC100128293	NC_000006	68643413	68644574	0	0	0	0
BAI3	NC_000006	69345632	70099403	0.0155928	0.032062	0.0346794	0.0217313
LMBRD1	NC_000006	70385749	70506888	1.9613973	2.1494801	5.733287	3.5145765
LOC100240707	NC_000006	70455692	70456947	0	0	0	0
COL19A1	NC_000006	70576448	70922157	0.0151111	0.0207145	0.0268866	0.0140401
RPL37P15	NC_000006	70808281	70808574	0	0	0	0
COL9A1	NC_000006	70925743	71012786	0.0547983	0.033803	0	0.1298312
FAM135A	NC_000006	71123107	71270875	0.5449066	0.9337021	1.0974519	1.1074952
RNU7-48P	NC_000006	71222998	71223056	0	0	0	0
C6orf57	NC_000006	71276625	71298606	1.6742184	1.377018	3.9469923	3.9666509
LOC642590	NC_000006	71317937	71319037	0	0	0	0
LOC100128312	NC_000006	71373157	71374009	0	0	0	0
SMAP1	NC_000006	71377479	71571718	2.5712287	3.9449055	4.6804467	7.7366179
B3GAT2	NC_000006	71571069	71666788	0.0228303	0.0704157	0.0609313	0.0636361
LOC157713	NC_000006	71874644	71877263	0	0	0	0
LOC100132834	NC_000006	71961243	71962568	0	0	0	0
OGFRL1	NC_000006	71998477	72011973	6.64275	6.0763885	18.178121	14.608419
C6orf155	NC_000006	72124149	72130448	0	0	0	0
LOC100131890	NC_000006	72260659	72279008	0	0	0	0
KRT19P1	NC_000006	72294363	72295736	0	0	0	0
RIMS1	NC_000006	72596650	73112508	1.1945091	0.4675435	0.3883864	0.3549241
LOC643067	NC_000006	73308179	73309362	0	0	0	0
KCNQ5	NC_000006	73331835	73905580	3.6876862	4.9192155	1.5286266	2.2934119
LOC653194	NC_000006	73918806	73919790	0	0	0	0
LOC100129128	NC_000006	73933267	73935175	0	0	0	0
KHDC1	NC_000006	73951037	73972907	2.7903639	2.5500333	2.1031318	1.0802426
C6orf147	NC_000006	73983862	74020088	0	0	0	0
RPSAP41	NC_000006	74000126	74000991	0	0	0	0
EIF3EP	NC_000006	74001659	74003138	0	0	0	0
LOC643095	NC_000006	74025142	74033173	0	0	0	0
LOC780810	NC_000006	74036678	74040783	0	0	0	0
DPPA5	NC_000006	74062787	74063953	0.0718108	0	0	0.0500407
C6orf221	NC_000006	74072400	74073898	0	0.0442108	0	0.0299656
OOEP	NC_000006	74078278	74079515	0	0.066544	0.0575811	0
LOC100128859	NC_000006	74079347	74097440	0	0	0	0
RPL39P3	NC_000006	74082653	74083036	0	0	0	0
RPS6P8	NC_000006	74100761	74101490	0	0	0	0
DDX43	NC_000006	74104285	74127289	0.0326025	0.117316	0.0435062	0.0340781
C6orf150	NC_000006	74134856	74162043	2.8778531	3.2596239	2.647005	2.7191901
MTO1	NC_000006	74171454	74211179	2.8040071	2.7524355	3.8357984	5.2432462
LOC100129409	NC_000006	74211426	74231001	0	0	0	0
EEF1A1	NC_000006	74225473	74230755	127.80276	184.13008	252.43089	220.34739
SLC17A5	NC_000006	74303102	74363737	7.6650809	5.5218098	8.6949022	5.0733428
RPS27P15	NC_000006	74328035	74328377	0	0	0	0
CD109	NC_000006	74405514	74538041	13.142136	14.39632	17.278748	22.290321
LOC100128592	NC_000006	74991982	74992393	0	0	0	0
LOC100127900	NC_000006	75558790	75560590	0	0	0	0
COL12A1	NC_000006	75794042	75915623	15.698237	14.137617	3.7682773	8.6487047
COX7A2	NC_000006	75947503	75953525	85.278271	84.502575	144.32825	102.95172
TMEM30A	NC_000006	75962638	75994632	11.702764	17.092929	21.433107	27.207886
FILIP1	NC_000006	76017800	76203496	0.0285255	0	0.0338361	0.0397554
LOC100288170	NC_000006	76174845	76175289	0	0	0	0
RPL26P20	NC_000006	76209421	76209858	0	0	0	0
SENP6	NC_000006	76311622	76427997	3.5118903	4.2225572	5.4895453	6.0189753
MYO6	NC_000006	76458909	76629254	1.1517258	0.975444	1.4488939	2.3758853
IMPG1	NC_000006	76631062	76782335	0.0268961	0.013826	0.0119638	0.0468557
LOC100131680	NC_000006	77370164	77371973	0	0	0	0
HTR1B	NC_000006	78171948	78173120	0	0.0385195	0	0
RPS6P7	NC_000006	78201312	78206460	0	0	0	0
IRAK1BP1	NC_000006	79577189	79608320	0.4938004	0.5467309	0.9461814	1.8263756

PHIP	NC_000006	79644136	79788011	2.2036553	3.6929105	5.3846555	7.0458207
TRNAF13P	NC_000006	79668010	79668082	0	0	0	0
HMGN3	NC_000006	79910962	79944455	1.7178462	1.422713	1.0612803	1.4963296
LOC100288198	NC_000006	79943391	79946517	0.0734184	0.0452891	0.143693	0.2148755
LOC100131959	NC_000006	80036248	80045765	0	0	0	0
DBIL2	NC_000006	80146408	80146941	0	0	0	0
LCA5	NC_000006	80194708	80247147	0.1490086	0.2393696	0.2651244	0.298526
LOC100129436	NC_000006	80247397	80251792	0.1997647	0.1026895	0.1777162	0.139204
LOC100130012	NC_000006	80256938	80262967	0	0	0	0
SH3BGRL2	NC_000006	80341000	80413369	0.1608093	0.0778018	0.2272136	0.1450166
ELOVL4	NC_000006	80624529	80657315	1.3931302	3.2189482	1.7376696	1.3210732
LOC100289245	NC_000006	80657163	80659377	0.0777845	0	0.0691992	0
GAPDHL8	NC_000006	80662668	80663727	0	0	0	0
RPL35AP18	NC_000006	80673932	80674239	0	0	0	0
TTK	NC_000006	80714359	80752239	6.2931262	11.81345	13.976589	17.324518
LOC643562	NC_000006	80773213	80780300	0	0	0	0
BCKDHB	NC_000006	80816344	81055987	1.7640858	1.9232159	2.2329429	2.128561
RPL17P25	NC_000006	81083696	81084321	0	0	0	0
LOC648934	NC_000006	81265939	81266708	0	0	0	0
FAM46A	NC_000006	82455447	82462428	12.443646	10.917444	15.257247	5.4570346
IBTK	NC_000006	82879956	82957448	5.2509576	6.0635734	9.2683234	9.6673858
LOC100132659	NC_000006	82973727	82974691	0	0	0	0
TPBG	NC_000006	83073539	83076628	17.738365	36.074459	15.122333	8.6358032
UBE2CBP	NC_000006	83602186	83775545	0.4075941	0.8134491	0.8745227	1.6874592
DOPEY1	NC_000006	83777385	83878127	1.0515528	1.3556737	2.1481635	2.0509635
PGM3	NC_000006	83878628	83902935	19.703972	26.013313	24.038925	18.276632
RWDD2A	NC_000006	83903032	83906256	1.0249411	1.0856789	1.5196934	1.3851535
ME1	NC_000006	83920108	84140779	1.857336	2.8105152	3.037043	6.0885175
GAPDHL5	NC_000006	84101825	84103340	0	0	0	0
PRSS35	NC_000006	84222271	84235423	0.0543465	0.5214902	0.0644642	0.0883653
SNAP91	NC_000006	84262604	84419127	0.0592294	0.0608941	0.2634607	0.0825468
RIPPLY2	NC_000006	84562985	84567234	0.2647484	0.1360946	1.7075744	0.8301925
CYB5R4	NC_000006	84569370	84670146	2.3861523	1.8298574	6.0377638	7.7414023
LOC100132652	NC_000006	84672790	84676105	0	0	0	0
MRAP2	NC_000006	84743420	84800606	0.5756543	0.4081608	0.1589332	0.0553295
KIAA1009	NC_000006	84833960	84937335	0.5474283	0.1934673	1.0729383	1.353026
LOC442233	NC_000006	85139299	85140519	0	0	0	0
TBX18	NC_000006	85444157	85473899	1.0906072	1.2322746	2.1710196	2.7690308
RPL31P32	NC_000006	85967006	85967459	0	0	0	0
KRT18P30	NC_000006	85987143	85995378	0	0	0	0
LOC100289382	NC_000006	85997444	85998731	0	0	0	0
LOC100289423	NC_000006	86001386	86001511	0	0	0	0
LOC643870	NC_000006	86136351	86137681	0	0	0	0
NT5E	NC_000006	86159302	86205498	118.8888	73.494027	212.95019	88.885369
SNX14	NC_000006	86215214	86303629	3.4983043	3.235673	6.0235907	5.4521895
SYNCRIP	NC_000006	86323939	86353027	2.3233206	3.6522736	2.8536422	3.3946403
LOC100289483	NC_000006	86351089	86353054	0.2092773	0.0717197	0	0.1458328
LOC100127917	NC_000006	86369696	86441776	0	0	0	0
SNHG5	NC_000006	86386725	86388451	0	0	0	0
SNORD50A	NC_000006	86387012	86387086	0	0	0	0
SNORD50B	NC_000006	86387307	86387377	0	0	0	0
LOC100127905	NC_000006	86445462	86445638	0	0	0	0
LOC100133102	NC_000006	86578662	86579193	0	0	0	0
RPL7P27	NC_000006	86796067	86796893	0	0	0	0
LOC643916	NC_000006	86880165	86883630	0	0	0	0
LOC100289579	NC_000006	86923950	86924303	0	0	0	0
LOC643926	NC_000006	87141965	87142427	0	0	0	0
LOC100289653	NC_000006	87606836	87607450	0.0714605	0.0734689	0.0635733	0.0995931
HTR1E	NC_000006	87647024	87726397	0.0214591	0	0	0.0448607
RPL7P29	NC_000006	87680152	87680976	0	0	0	0
CGA	NC_000006	87795222	87804824	0	0	0	0
LOC442234	NC_000006	87831411	87832406	0	0	0	0
LOC100286905	NC_000006	87865140	87961289	0.5294968	0.1814594	0.3140367	0.245983
ZNF292	NC_000006	87865269	87973406	1.2705825	1.4405005	3.0542554	4.1328425
GJB7	NC_000006	87992697	88038996	0.0385511	0.0792691	0.0171481	0.0402959
LOC644016	NC_000006	88008490	88010180	0	0	0	0

C6orf162	NC_000006	88032306	88052043	0.7402948	1.1138061	0.8995331	1.3337046
C6orf163	NC_000006	88057220	88075181	0.062456	0.0642114	0.1296461	0.0580291
C6orf164	NC_000006	88106842	88109459	0	0	0	0
C6orf165	NC_000006	88117721	88174183	0.0552808	0.0994603	0.2090121	0.41411
TAF13P	NC_000006	88135358	88135779	0	0	0	0
SLC35A1	NC_000006	88182643	88222057	3.2972703	2.2046466	4.9846319	3.7276872
RARS2	NC_000006	88224096	88299735	7.100806	7.473019	12.95427	14.76079
ORC3L	NC_000006	88299843	88377169	5.6882419	7.9714246	14.714138	10.903481
AKIRIN2	NC_000006	88384578	88411985	4.4812638	4.5838234	6.1519979	9.970522
LOC100286972	NC_000006	88407973	88411987	0	0.1864515	0.1075586	0
NCRNA00120	NC_000006	88410020	88410933	0	0	0	0
SPACA1	NC_000006	88757507	88776550	0.0293184	0	0.0260824	0
CNR1	NC_000006	88849583	88855056	0.0240856	0.0165084	0.0142848	0.0111892
RPS14P9	NC_000006	88974892	88975304	0	0	0	0
ACTBP8	NC_000006	88985179	88986792	0	0	0	0
RNGTT	NC_000006	89319989	89673348	1.930059	2.1665355	2.1024906	2.820261
LOC100130179	NC_000006	89378294	89380624	0	0	0	0
CYCSP16	NC_000006	89712449	89712619	0	0	0	0
LOC100131124	NC_000006	89769353	89769783	0	0	0	0
PNRC1	NC_000006	89790429	89794879	8.323997	8.4732118	14.645548	20.014571
LOC100287034	NC_000006	89790470	89792407	0	0.0427468	0.0369892	0.0869202
SRrp35	NC_000006	89805678	89827800	0.0488449	0	0.0325904	0.0340371
PM20D2	NC_000006	89855769	89875284	0.5787575	0.9501183	2.1342554	4.0915566
GABRR1	NC_000006	89887223	89927496	0	0.014294	0.0123687	0.0290651
GABRR2	NC_000006	89967239	90024967	0	0	0.1198577	0.0563302
LOC100288513	NC_000006	90011502	90012833	0	0	0	0
UBE2J1	NC_000006	90036344	90062619	7.3078359	8.9804873	11.498754	11.546045
RRAGD	NC_000006	90074335	90121995	2.0519881	2.2013838	3.6986329	1.3677372
LOC100128021	NC_000006	90131818	90182639	0	0	0	0
ANKRD6	NC_000006	90142897	90343403	0.3641183	0.2872933	0.2485972	0.094412
LYRM2	NC_000006	90341943	90348474	0.3036573	0.194065	0.2555396	0.7377422
MDN1	NC_000006	90353231	90529442	0.6415432	0.8828941	1.3010052	2.9551247
LOC100132736	NC_000006	90524395	90525182	0	0	0	0
CASP8AP2	NC_000006	90539619	90584155	1.9362172	2.2377942	3.682015	4.4234929
RPL22P14	NC_000006	90586230	90586597	0	0	0	0
LOC644269	NC_000006	90595334	90597574	0	0	0	0
GJA10	NC_000006	90604188	90605819	0	0	0.0239568	0
BACH2	NC_000006	90636247	91006562	0.023846	0.0294195	0.1442558	0.2326361
LOC100129711	NC_000006	90659817	90661708	0	0	0	0
MAP3K7	NC_000006	91225353	91296907	8.0026015	10.205139	18.353518	16.340772
LOC100287208	NC_000006	92432427	92440682	0	0	0	0
LOC100129847	NC_000006	92525558	92526131	0	0	0	0
RPL5P19	NC_000006	92723552	92724356	0	0	0	0
LOC100128159	NC_000006	93596985	93597776	0	0	0	0
COPS5P	NC_000006	93801543	93802701	0	0	0	0
EPHA7	NC_000006	93949738	94129300	3.1684532	0.0272025	0.0529618	0.0599222
TSG1	NC_000006	94416801	94486199	0	0	0	0
LOC100132830	NC_000006	95151747	95156547	0	0	0	0
CYCSP17	NC_000006	95952058	95952372	0	0	0	0
MANEA	NC_000006	96025413	96057326	2.2963768	2.084324	5.547512	4.4189811
KRT18P50	NC_000006	96438948	96440277	0	0	0	0
FUT9	NC_000006	96463845	96663488	0.0343399	0.021183	0.0305497	0.04068
KIAA0776	NC_000006	96969702	97003152	1.7345237	2.1485213	2.93743	3.2547125
FHL5	NC_000006	97010435	97064512	0.0413825	0	0.0552226	0.0144185
RPS7P8	NC_000006	97096331	97096983	0	0	0	0
LOC100286973	NC_000006	97120029	97120906	0	0	0	0
GPR63	NC_000006	97245888	97285353	0.0399893	0	0.1600901	0.0696653
NDUFAF4	NC_000006	97338140	97345767	1.9894261	2.8510789	2.9229319	2.2265001
KLHL32	NC_000006	97372496	97588630	0	0.0356055	0.0308097	0.0723992
C6orf167	NC_000006	97590037	97731052	1.5254506	2.8909431	4.4874216	5.0208785
LOC100190924	NC_000006	98626536	98627716	0	0	0	0
POU3F2	NC_000006	99282580	99286666	0.0107532	0.0442216	0.028699	0.0224797
FBXL4	NC_000006	99321601	99395849	4.621132	5.0438799	7.2507189	8.8003791
BDH2P1	NC_000006	99622547	99625446	0	0	0	0
C6orf168	NC_000006	99720793	99797531	0.0675434	0.3240613	0.1001475	0.1035472
COQ3	NC_000006	99817348	99842082	1.0769922	2.2859587	2.9361808	2.7356611

SFRS18	NC_000006	99847840	99873207	0.859323	0.9676148	1.2134564	2.8609834
USP45	NC_000006	99880182	99963252	0.4196685	0.9336583	0.9058296	1.1170314
LOC100130890	NC_000006	99973955	99979790	0.9005785	0.0925889	0.8011796	0.3765354
CCNC	NC_000006	99990263	100016690	6.9913312	9.1659437	10.676838	15.000482
LOC100287005	NC_000006	100010322	100016972	0	0	0	0
RPS3P5	NC_000006	100023558	100024408	0	0	0	0
PRDM13	NC_000006	100054650	100063454	0.0137167	0.0282044	0	0.0095583
MCHR2	NC_000006	100367786	100442114	0.0184424	0	0	0.0128514
LOC100287035	NC_000006	100441649	100448805	0.1268347	0.0651997	0.1128357	0.0441917
LOC100128985	NC_000006	100498248	100499141	0	0	0	0
LOC442239	NC_000006	100583940	100584539	0	0	0	0
LOC100129854	NC_000006	100610754	100626052	0	0	0	0
SIM1	NC_000006	100836750	100911551	0.0660049	0.6899093	0.2153057	0.4446165
ASCC3	NC_000006	100956608	101329224	3.7740523	5.0816255	5.0045778	7.7379482
LOC153893	NC_000006	100978037	100979105	0	0	0	0
LOC728098	NC_000006	101337479	101338216	0	0	0	0
GRIK2	NC_000006	101846905	102517958	0	0.7116264	0.0337412	0.0660731
LOC100132919	NC_000006	102901203	102942944	0	0.028471	0.0246361	0.0192973
LOC100130877	NC_000006	104204219	104212075	0	0	0	0
FLJ10088	NC_000006	104240543	104242231	0	0	0	0.0725278
LOC100132478	NC_000006	104251841	104255659	0	0	0	0
LOC100131717	NC_000006	104262418	104291337	0	0	0	0
LOC100129694	NC_000006	104465502	104469868	0	0	0	0
NPM1P10	NC_000006	104472802	104474734	0	0	0	0
HACE1	NC_000006	105175968	105307794	1.210113	1.7082012	1.9907633	2.4293774
LOC100129852	NC_000006	105384169	105401270	0	0.0270236	0	0.0366326
LIN28B	NC_000006	105404923	105531206	0.0079848	0.0164184	0.0568279	0.0278206
BVES	NC_000006	105544697	105584543	2.8181026	1.8371184	2.563086	2.9346785
POPDC3	NC_000006	105605775	105627858	6.0642852	3.202936	6.4739476	7.3413534
PREP	NC_000006	105725506	105850969	3.085169	5.1701611	2.1545517	4.8802017
RPL7AP35	NC_000006	105746016	105746821	0	0	0	0
RPL35P3	NC_000006	105750295	105750734	0	0	0	0
LOC100130683	NC_000006	106114201	106115873	0	0	0	0
PRDM1	NC_000006	106534195	106557814	0.4460911	1.7495829	0.8598525	0.2878278
LOC100287304	NC_000006	106553569	106595107	0.4107311	0.6861965	0.8221451	0.2504371
ATG5	NC_000006	106632352	106773695	4.8229256	6.0588101	8.4968507	7.6467791
LOC100287330	NC_000006	106957400	106967355	0	0.0734391	0.0158869	0.0124441
AIM1	NC_000006	106959730	107018335	2.1936295	3.0269828	4.8761958	8.2877335
RTN4IP1	NC_000006	107018903	107077373	0.4001356	0.4285224	0.5487898	1.1617936
QRSL1	NC_000006	107077441	107116292	1.9316731	2.5455438	2.9812131	2.8334334
RPL21P65	NC_000006	107090295	107090834	0	0	0	0
LOC553137	NC_000006	107218001	107222877	0	0	0	0
C6orf203	NC_000006	107349407	107372546	1.5623466	1.1898197	1.1067777	1.3104787
BEND3	NC_000006	107386385	107420489	0.7605018	1.4735352	1.1319428	1.2178612
PDSS2	NC_000006	107473761	107780779	3.4767605	3.2819025	3.7534544	7.1733949
RPS24P12	NC_000006	107550963	107551356	0	0	0	0
SOBP	NC_000006	107811317	107982513	1.1424284	0.6235193	0.1254736	0.0687979
SCML4	NC_000006	108023364	108145521	0.0096888	0.0099611	0.0258582	0.0202545
SEC63	NC_000006	108188960	108279482	2.8735382	3.5382079	3.7653963	5.4983435
RPL23AP50	NC_000006	108253004	108253458	0	0	0	0
RPL3P7	NC_000006	108325506	108326798	0	0	0	0
OSTM1	NC_000006	108362613	108395941	6.2965902	4.8349376	7.7022292	6.9175066
LOC100287366	NC_000006	108477081	108480596	0.0209777	0	0.0186623	0.0292362
NR2E1	NC_000006	108487215	108510013	0.0134852	0	0.0359904	0.009397
SNX3	NC_000006	108532717	108582464	32.781058	37.591109	62.198245	72.232039
LACE1	NC_000006	108616098	108844258	1.0880199	0.958799	1.4000455	2.3692987
RPL36AP24	NC_000006	108639256	108639658	0	0	0	0
FOXO3	NC_000006	108881026	109005971	4.9933218	7.6645059	7.1718656	8.9671465
ZNF259P	NC_000006	109106692	109108262	0	0	0	0
RPL37AP3	NC_000006	109138883	109139129	0	0	0	0
ARMC2	NC_000006	109169619	109295352	0.1673973	0.3309654	0.4353084	0.7088677
RPS12P13	NC_000006	109177556	109177940	0	0	0	0
ATP5J2P2	NC_000006	109228680	109229098	0	0	0	0
SESN1	NC_000006	109307645	109415277	0.6371618	0.4749252	0.8644261	0.7991994
LOC100287407	NC_000006	109323458	109331491	0.0759037	0	0.067526	0
C6orf182	NC_000006	109416356	109485115	1.5924303	2.6750445	4.8824524	6.0140088

FLJ37396	NC_000006	109487012	109591574	0.050866	0.0261478	0.011313	0.0620296
C6orf185	NC_000006	109611726	109676266	0	0	0	0
RPL7P28	NC_000006	109648378	109649096	0	0	0	0
CD164	NC_000006	109687717	109703762	54.370402	42.715157	72.466182	57.037852
PPIL6	NC_000006	109711418	109761847	0.1098401	0.0250949	0.1954335	0.0255136
SMPD2	NC_000006	109761931	109765122	1.621899	2.7163831	2.3505084	2.8255096
MICAL1	NC_000006	109765265	109777190	7.5236479	6.9045692	4.6230592	3.0330812
LOC100287481	NC_000006	109775161	109787166	0.1502504	0.0772366	0.4010007	0.4188018
ZBTB24	NC_000006	109783719	109804440	0.2120068	0.3713483	0.7893559	1.1709351
C6orf224	NC_000006	109814059	109827615	0.1217403	0.0312904	0.0541518	0.1060418
AKD2	NC_000006	109870887	110012415	0.4795378	0.1256706	0.6524626	0.6093525
FIG4	NC_000006	110012424	110146634	4.0763577	2.6920867	8.8016742	7.5058075
GPR6	NC_000006	110300298	110301923	0.0270284	0	0	0
WASF1	NC_000006	110421022	110501207	2.5659216	3.3536749	6.1317611	6.5529631
CDC40	NC_000006	110501624	110553423	0.9809364	1.1492275	1.532243	1.5022325
C6orf186	NC_000006	110567149	110679475	17.683076	13.912056	4.6519356	1.307329
DDO	NC_000006	110713383	110736753	1.3372195	0.2908235	2.2419903	1.1647847
SLC22A16	NC_000006	110745906	110797844	0.0222974	0.0229241	0.0396728	0.0310755
RPS19P5	NC_000006	110883378	110890566	0	0	0	0
CDC2L6	NC_000006	110931181	111136412	2.0402746	2.127057	4.6810084	6.7694999
LOC100129155	NC_000006	110966965	110967761	0	0	0	0
RPS20P18	NC_000006	111021643	111022126	0	0	0	0
LOC441166	NC_000006	111179442	111180350	0	0	0	0
AMD1	NC_000006	111195987	111216913	9.7634189	8.4925248	16.125889	19.497511
LOC442244	NC_000006	111244769	111245314	0	0	0	0
GTF3C6	NC_000006	111279763	111289091	0.9714872	1.1414754	1.2758153	1.7407826
BXDC1	NC_000006	111303291	111346794	3.195393	3.7137043	7.2097724	8.6162724
LOC442245	NC_000006	111368071	111368724	0	0	0	0
SLC16A10	NC_000006	111408781	111544608	0.0151912	0.0624727	0.0135145	0.0211717
KIAA1919	NC_000006	111580482	111590263	0.3363385	0.2305276	0.5784844	0.7734344
REV3L	NC_000006	111620234	111804414	5.9262965	4.6699227	10.23749	10.359972
LOC100287229	NC_000006	111751021	111752987	0	0	0	0
LOC643749	NC_000006	111804708	111898404	0	0	0	0
TRAF3IP2	NC_000006	111880143	111927321	7.5172404	6.558048	16.502896	17.336598
FYN	NC_000006	111982485	112194627	9.2709327	5.3604738	9.2769233	12.11451
WISP3	NC_000006	112375278	112390889	0	0	0.0833045	0.0217506
TUBE1	NC_000006	112391860	112408751	1.3967004	1.6720022	2.5872137	2.0932111
C6orf225	NC_000006	112408674	112423993	2.073185	2.7911873	2.4152371	2.2243453
LAMA4	NC_000006	112429134	112575828	20.910094	6.7146548	32.115857	30.993557
LOC100128588	NC_000006	112619624	112647951	0	0	0.0269267	0.0210915
RFPL4B	NC_000006	112668532	112672498	0	0	0.0411553	0.0322367
RPSAP45	NC_000006	112676954	112677995	0	0	0	0
LOC442249	NC_000006	112682639	112684118	0	0	0	0
LOC643859	NC_000006	112686922	112688646	0	0	0	0
PA2G4P5	NC_000006	112937031	112939232	0	0	0	0
LOC100287612	NC_000006	113007091	113011966	0.0116081	0.0119343	0.0206538	0.008089
LOC643884	NC_000006	113543368	113545274	0	0	0	0
RPS27AP11	NC_000006	113902665	113903201	0	0	0	0
RPL30P8	NC_000006	114151025	114155968	0	0	0	0
MARCKS	NC_000006	114178527	114184652	4.7010577	9.7506013	3.4168229	2.2767038
FLJ34503	NC_000006	114225551	114242806	0	0	0	0
HDAC2	NC_000006	114257326	114292354	1.0978791	1.7678983	1.6003819	1.8573102
HS3ST5	NC_000006	114376750	114384041	0.0320322	0	0.0284968	0.133928
RPSAP43	NC_000006	114405363	114405956	0	0	0	0
LOC441167	NC_000006	114551305	114560363	0	0	0	0
LOC728614	NC_000006	114670647	114677328	0	0	0	0
FRK	NC_000006	116262693	116381921	0.0771292	0.0475782	0.0274465	0.032248
LOC728402	NC_000006	116359919	116360993	0	0	0	0
LOC100287673	NC_000006	116421408	116422403	0	0	0.0684721	0
NT5DC1	NC_000006	116421999	116566853	1.9804093	2.7580792	2.4990454	4.3651952
COL10A1	NC_000006	116440085	116447296	0	0	0	0
TSPYL4	NC_000006	116571127	116575261	3.5498693	3.6605658	2.0612501	2.7477218
LOC100287430	NC_000006	116579604	116580397	0	0	0	0
RPS5P1	NC_000006	116579608	116580350	0	0	0	0
TSPYL1	NC_000006	116597744	116601280	9.1450095	11.675892	10.014813	17.005163
DSE	NC_000006	116601283	116759442	7.5279746	13.111834	10.560084	8.4914435

LOC100287467	NC_000006	116691329	116692877	0	0.0291694	0.1009621	0.0197707
LOC644101	NC_000006	116773956	116774728	0	0	0	0
KRT18P22	NC_000006	116778486	116779768	0	0	0	0
FAM26F	NC_000006	116782556	116784934	2.2588361	3.0964279	0.2467836	0.0828446
BET3L	NC_000006	116817752	116866773	0	0.0448693	0	0.060824
FAM26E	NC_000006	116832808	116839709	0.0124183	0.1021382	0.0220953	0
FAM26D	NC_000006	116850195	116880031	0.100568	0.0344648	0.0298227	0
LOC100287703	NC_000006	116892098	116892553	0	0.0990864	0.0857403	0
RWDD1	NC_000006	116892583	116914438	7.2744213	10.787494	15.776211	16.001557
RSPH4A	NC_000006	116937650	116954141	0	0.0639765	0.0968789	0.065044
ZUFSP	NC_000006	116956781	116989957	0.6206812	0.7822183	1.0865383	1.409163
KPNA5	NC_000006	117002367	117063030	1.3237419	0.565316	1.630575	1.1211146
FAM162B	NC_000006	117073360	117086886	0.1272632	2.1806661	0.1509559	0.1478035
GPRC6A	NC_000006	117113248	117150198	0	0.0157984	0.0136705	0.021416
RFX6	NC_000006	117198376	117253314	0	0.025856	0.0335601	0.0087625
RPS29P13	NC_000006	117369833	117370004	0	0	0	0
VGLL2	NC_000006	117586721	117594728	0	0	0	0.0549079
ROS1	NC_000006	117609530	117747018	0.0417532	0.079721	0.0318384	0.0124694
LOC100132917	NC_000006	117752754	117753198	0	0	0	0
DCBLD1	NC_000006	117803820	117891021	7.5504794	14.886093	3.0819708	5.1686252
GOPC	NC_000006	117881432	117923681	0.906838	1.1580455	0.6114302	0.8514302
LOC442253	NC_000006	117924091	117966250	0	0	0	0
NUS1	NC_000006	117996617	118031890	3.4347402	4.7897599	5.981253	8.5235319
SLC35F1	NC_000006	118228689	118638839	0.0181155	0	0.008058	0.0378708
RPL29P4	NC_000006	118320091	118320749	0	0	0	0
LOC644303	NC_000006	118773597	118776154	0	0	0	0
C6orf204	NC_000006	118786239	118973020	1.285394	0.6292953	1.3940079	1.5440398
BRD7P3	NC_000006	118822536	118825009	0	0	0	0
PLN	NC_000006	118869442	118881587	0	0	0	0.0178675
SSXP10	NC_000006	118909953	118911472	0	0	0	0
LOC100287603	NC_000006	118910233	118911457	0	0	0	0
LOC100287632	NC_000006	119104011	119104285	0	0	0	0
ASF1A	NC_000006	119215241	119230336	3.4506982	2.9533989	6.1864223	5.7368245
MCM9	NC_000006	119231762	119256303	0.3386117	0.2931608	0.586622	0.6954555
FAM184A	NC_000006	119280995	119470358	0.1162093	0.0977526	0.4135319	0.6993662
MAN1A1	NC_000006	119499266	119670926	7.5592665	6.8180679	16.485097	13.982538
RPL13AP15	NC_000006	119590298	119590876	0	0	0	0
LOC100287768	NC_000006	121102334	121102782	0	0	0	0
C6orf170	NC_000006	121400627	121655644	0.693649	0.7835781	1.3255995	1.2531614
RPS15AP21	NC_000006	121702585	121703042	0	0	0	0
GJA1	NC_000006	121756745	121770873	72.226743	16.182298	14.814605	24.783648
LOC260339	NC_000006	121799313	121800434	0	0	0	0
SLC25A5P7	NC_000006	121974885	121976097	0	0	0	0
RPL23AP48	NC_000006	122001118	122001605	0	0	0	0
LOC100129422	NC_000006	122004071	122005388	0	0	0	0
LOC644502	NC_000006	122179325	122179914	0	0	0	0
HSF2	NC_000006	122720696	122754264	2.4116938	2.5967472	7.1468668	6.9607158
SERINC1	NC_000006	122764493	122792952	25.767601	26.564027	35.510289	36.195708
PKIB	NC_000006	122793062	123047518	0.0201597	0.1450843	0.8070598	0.7445498
FABP7	NC_000006	123100646	123105219	0.0439482	0	0	0.0612498
SMPDL3A	NC_000006	123109971	123130865	1.8420216	3.8493377	2.4580702	2.0370078
ATP5LP2	NC_000006	123180748	123181158	0	0	0	0
RLBP1L2	NC_000006	123317582	123385063	0	0	0.0389031	0.0457088
TRDN	NC_000006	123537483	123957942	0.0491042	0.0302905	0.0174738	0.0410613
NKAIN2	NC_000006	124125069	125146786	0.0662869	0.02726	0.0589707	0.0369531
STL	NC_000006	125229389	125284173	0	0	0	0
RNF217	NC_000006	125304514	125404661	1.5766182	1.6462562	2.9805319	2.8324579
TPD52L1	NC_000006	125474879	125584644	1.5108048	0.3328427	3.6241451	2.7447712
HDDC2	NC_000006	125596496	125623282	4.2451543	4.8960343	6.7058983	7.3575559
HEY2	NC_000006	126070732	126082415	0	0	0.0146323	0.0114614
LOC100129733	NC_000006	126080436	126092814	0.0661871	0	0.0588819	0.0922436
NCOA7	NC_000006	126112001	126252266	3.7785006	1.5986062	5.3324636	2.6330097
HINT3	NC_000006	126277861	126301390	1.911358	1.5640408	6.7206171	4.657156
TRMT11	NC_000006	126307576	126360422	0.9451233	0.5321138	1.6015388	1.4740085
LOC442256	NC_000006	126579067	126661321	0	0	0	0
C6orf173	NC_000006	126661253	126669754	12.545223	13.637172	18.340313	41.816879

LOC100130535	NC_000006	126923453	126946434	0	0	0	0
LOC728666	NC_000006	126964786	126965445	0	0	0	0
RPS4P9	NC_000006	127004158	127005054	0	0	0	0
RSPO3	NC_000006	127440048	127518184	2.1963852	0.4431814	2.1913629	2.3029451
RNF146	NC_000006	127588020	127609507	4.2206693	3.665227	6.0150101	9.7656955
ECHDC1	NC_000006	127609857	127664754	4.576792	5.6502874	5.7231903	13.397584
YWHAZP4	NC_000006	127676817	127680000	0	0	0	0
RPL5P18	NC_000006	127683631	127684602	0	0	0	0
LOC100287856	NC_000006	127737680	127738082	0	0	0	0
RPL17P23	NC_000006	127756759	127757360	0	0	0	0
KIAA0408	NC_000006	127761735	127796859	0.3339221	0.238213	0.1333767	0.0807292
C6orf174	NC_000006	127771330	127837847	0.9516349	0.4574248	0.241886	0.1205704
C6orf58	NC_000006	127898319	127912962	0.0732471	0	0.1629065	0
LOC100287884	NC_000006	127976005	127976307	0.1450437	0	0	0.505361
LOC100287918	NC_000006	127980683	127981495	0	0	0.0732164	4.7600466
TSEPA	NC_000006	128029345	128239742	0.0436211	0.0112118	0.0679114	0.0303969
MRPS17P5	NC_000006	128230976	128231161	0	0	0	0
PTPRK	NC_000006	128289924	128841870	8.3670123	9.9379997	9.7680403	12.098717
LOC100287955	NC_000006	128799795	128842138	0.0352998	0.0725838	0.0314037	0.0245983
EEF1DP5	NC_000006	128901393	128902087	0	0	0	0
RPL21P64	NC_000006	128960333	128960798	0	0	0	0
LAMA2	NC_000006	129204286	129837711	0.6030243	2.2328329	0.2258809	0.195888
MESTP1	NC_000006	129248899	129251350	0	0	0	0
ARHGAP18	NC_000006	129898240	130031370	6.217312	8.8068248	4.1343525	5.6978553
RPL5P21	NC_000006	130077478	130078257	0	0	0	0
LOC100130402	NC_000006	130106353	130123359	0	0	0	0
C6orf191	NC_000006	130152389	130182416	0	0	0	0.0561924
L3MBTL3	NC_000006	130339734	130462585	0.230205	0.5163817	0.2420325	0.4010401
SAMD3	NC_000006	130465460	130544099	0.3731957	0.2009775	0.0474293	0.0743022
TMEM200A	NC_000006	130758262	130764212	6.7107891	6.6565235	0.1712416	0.262168
LOC285733	NC_000006	131148324	131156432	0	0	0	0
EPB41L2	NC_000006	131160487	131384462	2.3989639	1.7487088	3.026344	6.1112733
LOC100288020	NC_000006	131276703	131388460	0.0704299	0	0.1253127	0.0245392
AKAP7	NC_000006	131466461	131604673	0.9152948	0.9699737	1.3278891	1.6092535
RPL21P67	NC_000006	131790159	131790708	0	0	0	0
ARG1	NC_000006	131894365	131905468	0	0	0	0
MED23	NC_000006	131895106	131949363	3.5631571	4.214416	4.8109292	5.3669069
ENPP3	NC_000006	131958442	132068550	0.0833142	0.0713798	0.0370593	0.0193522
OR2A4	NC_000006	132021609	132022541	0	0	0.0419052	0
LOC643854	NC_000006	132029581	132032157	0	0.0175333	0	0
RPL15P10	NC_000006	132101810	132102488	0	0	0	0
RPL15P9	NC_000006	132101861	132102452	0	0	0	0
ENPP1	NC_000006	132129156	132216295	3.1003523	3.6489149	4.3289968	5.3867194
LOC100131774	NC_000006	132236146	132398533	4.5678924	14.176329	0.7319751	1.6212008
CTGF	NC_000006	132269316	132272518	17.624291	25.752997	0.1667985	3.2271096
MOXD1	NC_000006	132617194	132722664	13.174347	10.838664	17.239466	12.324523
STX7	NC_000006	132778663	132834337	6.1342263	4.8886959	10.05366	8.5563185
RPL21P66	NC_000006	132839950	132840470	0	0	0	0
TAAR9	NC_000006	132859427	132860475	0	0	0.0372713	0
TAAR8	NC_000006	132873832	132874860	0	0	0	0.0297618
TAAR7P	NC_000006	132880163	132880372	0	0	0	0
TAAR6	NC_000006	132891461	132892498	0	0	0	0.0295037
TAAR5	NC_000006	132909731	132910877	0.0766316	0	0.0340868	0.0267
TAAR4P	NC_000006	132915430	132916678	0	0	0	0
LOC100287784	NC_000006	132921025	132935928	0.0985386	0	0	0.1373313
TAAR3	NC_000006	132929341	132930454	0	0	0	0
TAAR2	NC_000006	132938289	132945414	0	0	0	0
TAAR1	NC_000006	132966123	132967142	0	0.0442975	0	0
VNN1	NC_000006	133001997	133035194	0.0342988	0.0587713	0.3458161	0.0557685
LOC100130820	NC_000006	133020061	133020808	0	0	0	0
VNN3	NC_000006	133043926	133055903	0.0458989	0	1.5516527	0.0159921
VNN2	NC_000006	133065009	133084598	0	0.1302741	0.1127272	0.0294328
C6orf192	NC_000006	133090507	133119747	0.5436895	0.5776023	1.5639026	0.4041221
RPS12	NC_000006	133135708	133138703	185.3165	231.30668	368.1234	273.91914
SNORD101	NC_000006	133136446	133136518	0	0	0	0
SNORD100	NC_000006	133137941	133138016	0	0	0	0

SNORA33	NC_000006	133138358	133138490	0	0	0	0
LOC100129706	NC_000006	133189357	133189998	0	0	0	0
RPL23AP46	NC_000006	133318709	133319179	0	0	0	0
LOC285735	NC_000006	133409219	133427717	0	0	0	0
EYA4	NC_000006	133562495	133853258	1.956963	0.101378	4.5481117	4.9420541
MGC34034	NC_000006	134142285	134175130	0	0	0	0
TCF21	NC_000006	134210259	134216675	0.0360724	0.0370862	0.1283641	0.0335156
LOC100288120	NC_000006	134214440	134219784	0.0136189	0.0140017	0.0121158	0
TBPL1	NC_000006	134274301	134308629	2.9987421	4.3847426	6.4915594	6.1296193
SLC2A12	NC_000006	134308719	134373789	0.392465	0.1936777	0.4259603	0.4594552
LOC100288153	NC_000006	134436029	134467785	0	0	0	0
SGK1	NC_000006	134490384	134639196	7.1935441	22.494839	4.3879992	7.9555169
LOC442261	NC_000006	134714220	134715038	0	0	0	0
FAM8A6P	NC_000006	134921949	134926561	0	0	0	0
LOC645175	NC_000006	135218997	135220402	0	0	0	0
LOC100288216	NC_000006	135238174	135250299	0	0	0	0
ALDH8A1	NC_000006	135238528	135271244	0.0345777	0.0177747	0	0.0602378
HBS1L	NC_000006	135281516	135376036	2.1613884	2.943481	4.4698492	6.9663149
MYB	NC_000006	135502453	135540311	0.1792342	0.7248017	0.1063012	0.2497951
AHI1	NC_000006	135605110	135818903	1.5275326	1.4325697	2.0284597	2.6585177
C6orf217	NC_000006	135818939	136011976	0	0	0	0
GAPDHL19	NC_000006	135940302	135941308	0	0	0	0
PDE7B	NC_000006	136172834	136516709	1.4929509	2.9356211	0.2903105	0.4889065
FAM54A	NC_000006	136552168	136571449	0.6127751	1.1591949	1.6136196	1.5030615
BCLAF1	NC_000006	136578001	136610989	4.8770859	6.9177947	5.7814657	10.220942
MAP7	NC_000006	136663872	136871792	0.1101459	0.0905933	0.0685922	0.0997803
RPLP1P8	NC_000006	136685138	136685636	0	0	0	0
MAP3K5	NC_000006	136878187	137113656	2.8255456	1.5046638	5.3886154	4.503832
PEX7	NC_000006	137143702	137235072	1.0379137	1.707335	2.2160564	2.6037346
RPL7AP37	NC_000006	137221458	137222318	0	0	0	0
SLC35D3	NC_000006	137243402	137246777	0.01863	0.0191536	0.0331476	0.0389464
RPL35AP3	NC_000006	137295067	137295396	0	0	0	0
IL20RA	NC_000006	137321108	137366298	0.2625913	0.4540429	0.1274228	0.0998095
IL22RA2	NC_000006	137464957	137494785	0	0.0311932	0.0134959	0
IFNGR1	NC_000006	137518621	137540567	8.8103767	10.038388	16.210736	7.5262083
OLIG3	NC_000006	137813336	137815531	0	0	0	0.0139458
LOC391040	NC_000006	137865034	137948084	0	0	0	0
LOC442263	NC_000006	138026560	138031224	0	0	0	0
TNFAIP3	NC_000006	138188581	138204449	9.3905631	5.8314318	6.0251889	2.8261677
RPSAP42	NC_000006	138316407	138317284	0	0	0	0
PERP	NC_000006	138409642	138428660	4.5358008	10.103773	10.805906	11.36865
KIAA1244	NC_000006	138483224	138659936	0.0794814	0.12768	0.6363795	0.5953972
PBOV1	NC_000006	138537123	138539627	0	0.0180373	0	0
LOC202451	NC_000006	138661655	138665799	0.0248365	0.0638364	0.41981	0.3894093
LOC100288356	NC_000006	138723721	138725476	0	0	0	0
HEBP2	NC_000006	138725336	138734582	3.0510083	6.2030255	8.1122874	9.0059119
NHSL1	NC_000006	138743180	138820579	0.6707261	1.0827003	0.8811033	1.0658209
LOC100129577	NC_000006	138971428	138972787	0	0	0	0
FLJ46906	NC_000006	139012958	139018732	0.3606009	0.1853678	0.4411007	0.2512811
CCDC28A	NC_000006	139094657	139114456	1.4101037	1.8423713	4.8871957	4.2580045
ECT2L	NC_000006	139134309	139225207	0.0309422	0.0212079	0.0825811	0.086247
CCRL1P	NC_000006	139143873	139145696	0	0	0	0
REPS1	NC_000006	139225620	139309398	6.2500301	7.9525841	8.3898465	9.9783115
C6orf115	NC_000006	139349819	139364439	8.7896464	5.5610341	8.3747448	8.2995232
HECA	NC_000006	139456249	139501946	2.8694184	2.9500638	6.0722602	5.0832612
TXLNB	NC_000006	139561198	139613208	0.0375225	0	0.0417263	0.0196104
LOC100129844	NC_000006	139634620	139660723	0	0	0	0
CITED2	NC_000006	139693396	139695785	105.22527	60.915654	26.760562	31.275459
LOC645440	NC_000006	139935575	139936341	0	0	0	0
LOC100129554	NC_000006	139963857	139982138	0	0	0	0
RPS3AP24	NC_000006	141082677	141083399	0	0	0	0
RPS18P10	NC_000006	141243554	141244091	0	0	0	0
LOC729076	NC_000006	141807342	141809069	0	0	0.2715109	0
RPS3AP23	NC_000006	141956857	141957640	0	0	0	0
LOC645503	NC_000006	142365318	142384190	0	0	0	0
NMBR	NC_000006	142396745	142409936	0.0973742	0.0667406	0.0866268	0.0226181

VTA1	NC_000006	142468410	142542085	10.079373	14.680427	14.245784	16.615224
GPR126	NC_000006	142623056	142767403	0.536647	3.1740675	0.2527497	0.3123641
LOC153910	NC_000006	142847592	142959026	0	0	0	0
HIVEP2	NC_000006	143072604	143266338	2.0880382	1.6727709	2.9994636	4.1572233
AIG1	NC_000006	143382023	143661441	6.6318992	4.8282624	7.1420102	4.6213715
RPL31P27	NC_000006	143648371	143648832	0	0	0	0
LOC100131471	NC_000006	143663383	143664765	0	0	0	0
LOC100288558	NC_000006	143707718	143708547	0	0	0	0
ADAT2	NC_000006	143743969	143771841	0.3514172	0.3974232	0.4939561	0.5485345
PEX3	NC_000006	143771918	143811753	3.5646547	4.0231796	3.9323074	5.1556662
FUCA2	NC_000006	143816242	143832863	43.969179	45.398766	40.625688	54.330694
LOC285740	NC_000006	143875467	143890476	0	0	0	0
PHACTR2	NC_000006	143929317	144152322	5.2552663	8.430291	3.8193714	6.1838697
LTV1	NC_000006	144164508	144184943	0.8479538	0.772153	1.0345552	2.1609616
LOC100128319	NC_000006	144167253	144178467	0	0	0.1714806	0.2686393
FAM164B	NC_000006	144185573	144259483	0	0	0	0.1543593
PLAGL1	NC_000006	144261437	144385735	6.7116064	4.1576745	3.2292335	11.645602
HYMAI	NC_000006	144324023	144329867	0	0	0	0
LOC100131041	NC_000006	144357343	144385804	0	0	0	0
SF3B5	NC_000006	144416018	144416754	14.072975	16.430328	30.23828	29.502936
MRPL42P3	NC_000006	144457574	144457912	0	0	0	0
STX11	NC_000006	144471654	144513076	0.0399239	0	0.0568279	0.1391028
LOC285741	NC_000006	144521377	144522102	0	0	0	0
LOC100288629	NC_000006	144578170	144578760	0	0	0	0
UTRN	NC_000006	144612873	145174170	2.3404175	2.4207341	2.1041164	4.4615602
LOC645749	NC_000006	145810766	145813228	0	0	0	0
EPM2A	NC_000006	145946440	146056991	0.1775686	0.1825592	0.1354028	0.2297971
LOC100289222	NC_000006	146110406	146113109	0.0325061	0.0501295	0.0578366	0.0792804
FBXO30	NC_000006	146119271	146135921	2.9112713	2.3678235	2.971344	4.2658065
SHPRH	NC_000006	146205943	146285233	0.5981793	1.0024356	1.8731922	2.6677449
GRM1	NC_000006	146348782	146758731	0.0443346	0.013023	0.0507102	0.0264807
RAB32	NC_000006	146864828	146876086	56.186517	66.779791	92.332655	120.69206
C6orf103	NC_000006	146920136	147136598	0.0332312	0.0512477	0.0369542	0.0405244
LOC729176	NC_000006	147122805	147124960	0	0	0	0
STXBP5	NC_000006	147525508	147708707	4.0853478	4.0807643	2.8261104	4.9700564
LOC442266	NC_000006	147728025	147729546	0	0	0	0
SAMD5	NC_000006	147830063	147891157	0.0433059	0.1113074	0.1219993	0.0754431
SASH1	NC_000006	148663729	148873184	2.8960568	2.2330881	1.9880978	4.6797388
LOC100289261	NC_000006	148888514	148888790	0	0	0	0
RPSAP40	NC_000006	148968760	148969807	0	0	0	0
UST	NC_000006	149068271	149398126	1.2010448	1.4200204	2.0123092	2.4061906
LOC729200	NC_000006	149433145	149433808	0	0	0	0
MAP3K7IP2	NC_000006	149691044	149732749	6.4636111	6.7341716	20.394967	15.93759
SUMO4	NC_000006	149721495	149722182	0.0638782	0.0656735	0	0.0445129
ZC3H12D	NC_000006	149770878	149806030	0.0921733	0.0947638	0.0819999	0.038538
LOC729496	NC_000006	149812395	149813448	0	0	0	0
PPIL4	NC_000006	149825631	149867171	2.1021043	2.7838983	2.630805	3.066212
RNU7-3P	NC_000006	149838093	149838152	0	0	0	0
C6orf72	NC_000006	149887528	149912067	21.597643	27.134487	35.00801	25.737048
RPS18P9	NC_000006	149915175	149915717	0	0	0	0
KATNA1	NC_000006	149916172	149959728	3.6984705	4.7233142	6.4777032	6.7853938
LATS1	NC_000006	149982051	150039392	4.3994444	5.5018275	7.7537338	8.0442635
LOC645967	NC_000006	150038827	150040038	0	0	0	0
NUP43	NC_000006	150045457	150067688	5.0481077	7.6792996	6.5433556	7.3697082
PCMT1	NC_000006	150070965	150132556	19.211891	24.131723	47.484348	35.681143
LRP11	NC_000006	150139932	150185480	2.8480418	4.8086246	3.9752832	5.22676
LOC100288743	NC_000006	150160499	150240644	0.2883742	0.6522538	0.4361277	0.7435174
LOC442267	NC_000006	150201098	150202708	0	0	0	0
RAET1E	NC_000006	150209601	150212097	0	0.1531641	0.1325341	0.1384175
LOC100240714	NC_000006	150226774	150228142	0	0	0	0
RAET1F	NC_000006	150231063	150231725	0	0	0	0
RAET1G	NC_000006	150238014	150244214	0.7543057	1.0612181	1.5893319	1.438567
LOC100288775	NC_000006	150244665	150247453	0.0787885	0.0972034	0.1121479	0.1098059
ULBP2	NC_000006	150263136	150270371	4.6912864	4.5886776	2.492506	2.8377389
ULBP1	NC_000006	150285143	150294846	0.8392406	1.5099482	0.5101847	0.3996244
RAET1J	NC_000006	150298971	150299802	0	0	0	0

RAET1K	NC_000006	150319155	150326280	0	0	0	0
RAET1L	NC_000006	150341266	150346668	0.2191932	0.0563384	0.1462502	0.1527425
RAET1M	NC_000006	150353905	150354702	0	0	0	0
PHBP1	NC_000006	150363622	150365439	0	0	0	0
ULBP3	NC_000006	150385743	150390202	1.0164897	2.7663307	1.1702673	0.999996
PPP1R14C	NC_000006	150464188	150571528	1.2323792	0.2452288	0.4774465	0.3185763
RNU4P1	NC_000006	150647700	150648199	0	0	0	0
IYD	NC_000006	150690028	150725764	0.0059381	0.0122101	0	0.0124138
PLEKHG1	NC_000006	150920999	151164799	0.0667999	0.0249736	0.0702319	0.0634756
LOC644850	NC_000006	151148761	151149756	0	0	0	0
MTHFD1L	NC_000006	151186691	151423023	2.5162134	3.3152913	4.2813899	3.2599579
RPL32P16	NC_000006	151417295	151421069	0	0	0	0
RPS12P11	NC_000006	151546663	151547164	0	0	0	0
AKAP12	NC_000006	151561134	151679694	0.3500257	0.478104	0.1690417	0.8258159
ZBTB2	NC_000006	151685250	151712677	2.6488565	2.9575655	4.5736297	4.9519813
RMND1	NC_000006	151725989	151773316	4.9904586	4.5869074	6.7515419	6.1217705
C6orf211	NC_000006	151773422	151791232	4.2376211	5.4107651	5.9892849	8.6206889
C6orf97	NC_000006	151815175	151942328	0.0998256	0.0342104	0.1924166	0.0695625
ESR1	NC_000006	152011631	152424409	0.1576562	0.0713183	0.0280511	0.0615222
SYNE1	NC_000006	152442822	152958534	0.3987617	0.2974285	0.2364996	1.1605286
NANOGP11	NC_000006	152867649	152868249	0	0	0	0
MYCT1	NC_000006	153019030	153045717	0.2886583	0.1483856	0.0385198	0.0201149
VIP	NC_000006	153071933	153080900	0	0.0285249	0.0246828	0.0193339
LOC442271	NC_000006	153218371	153220443	0	0	0	0
FBXO5	NC_000006	153291658	153304740	3.9223574	6.3971632	9.2311494	12.225104
MTRF1L	NC_000006	153308400	153323925	1.5412479	1.8408916	2.1071148	3.7590103
RGS17	NC_000006	153332026	153452389	0.7165473	1.1971146	2.7888889	2.0804944
LOC100289365	NC_000006	153552455	153668623	0	0.03728	0.0322587	0
RPL27AP6	NC_000006	153603384	153603889	0	0	0	0
LOC100288838	NC_000006	153986842	153988453	0	0	0	0
LOC729635	NC_000006	154259559	154267408	0	0	0	0
OPRM1	NC_000006	154331636	154568001	0.0266798	0	0	0.0278873
IPCEF1	NC_000006	154475618	154677900	0.0063208	0.0064984	0.0056231	0.0088091
RPL17P24	NC_000006	154538028	154538658	0	0	0	0
CNKSR3	NC_000006	154726433	154831753	1.4703869	1.329747	2.5435219	4.2218311
LOC100128473	NC_000006	154834572	154846574	0	0	0	0
LOC100129996	NC_000006	154864092	154871376	0	0	0	0
RPS4P8	NC_000006	154897390	154898275	0	0	0	0
RPL31P29	NC_000006	154926759	154927062	0	0	0	0
LOC646274	NC_000006	155027783	155028932	0	0	0	0
RBM16	NC_000006	155054512	155155194	0.764088	0.7494449	1.6251586	2.8947976
TIAM2	NC_000006	155411423	155578857	2.5304409	1.0690315	2.0958986	3.6989016
TFB1M	NC_000006	155578790	155635626	1.9152537	1.5471359	2.8904815	3.3842278
CLDN20	NC_000006	155585147	155597679	0	0	0	0
NOX3	NC_000006	155716504	155777037	0.0215222	0.0442541	0.0382934	0.029995
TRNAV37P	NC_000006	156869046	156869120	0	0	0	0
ARID1B	NC_000006	157099086	157530402	2.6271087	4.1170259	7.1817648	10.550463
C6orf35	NC_000006	157710054	157744793	0.6254949	0.8607305	1.3611808	1.3679604
LOC641708	NC_000006	157787017	157802004	0	0	0	0
ZDHHC14	NC_000006	157802557	158094977	1.1353687	1.8806152	1.8938541	1.3405939
SNX9	NC_000006	158244294	158366109	34.206374	34.726672	30.32854	55.416446
SYNJ2	NC_000006	158402919	158519568	7.9573824	12.821411	6.9456495	9.7401475
SERAC1	NC_000006	158530547	158589269	1.9308035	1.8927402	2.4866651	3.1993806
GTF2H5	NC_000006	158591501	158613387	15.073558	16.302252	22.291708	20.189229
LOC153932	NC_000006	158658168	158663816	0	0	0	0
TULP4	NC_000006	158733692	158932860	0.9364138	1.4055072	1.5747289	2.3127661
LOC727863	NC_000006	158936611	158937176	0	0	0	0
TMEM181	NC_000006	158957468	159056467	4.2181182	5.132004	4.8319578	6.5596367
DYNLT1	NC_000006	159057506	159065740	39.774731	44.663305	77.745087	50.630194
SYTL3	NC_000006	159071046	159185901	0.907144	0.7342055	0.5494607	0.833879
EZR	NC_000006	159186773	159240456	8.4064318	8.1294054	8.2708697	10.211427
OSTCL	NC_000006	159262149	159278664	0	0.302483	0.1308705	0.4869228
LOC100130967	NC_000006	159316251	159331385	0	0	0.1461591	0.0572428
LOC442272	NC_000006	159337840	159343182	0	0	0	0
RSPH3	NC_000006	159398266	159421198	2.483065	1.3905778	2.2269629	2.1382552
TAGAP	NC_000006	159456024	159466184	0.0862939	0.0380225	0.0219341	0.0859043

FNDC1	NC_000006	159590429	159693140	0.1475898	1.0897539	0.0358091	0.0420736
RPL21P69	NC_000006	159947046	159947599	0	0	0	0
SOD2	NC_000006	160100148	160114353	187.12642	97.235902	1441.7447	341.66742
LOC100132803	NC_000006	160131654	160135017	0	0	0	0
WTAP	NC_000006	160148152	160177351	25.508414	21.224797	115.4026	32.92477
LOC100129518	NC_000006	160182119	160183352	0	0	0	0
ACAT2	NC_000006	160182989	160200087	9.1523397	13.138436	8.7723745	16.546517
TCP1	NC_000006	160199530	160210735	39.809609	68.539518	65.316683	89.277825
SNORA20	NC_000006	160201282	160201413	0	0	0	0
SNORA29	NC_000006	160206626	160206765	0	0	0	0
MRPL18	NC_000006	160211492	160219461	20.197294	32.002983	29.053053	25.076666
PNLDC1	NC_000006	160221301	160241735	0.0457794	0	0.0407266	0
MAS1	NC_000006	160327974	160329108	0.0387209	0	0	0.0539645
IGF2R	NC_000006	160390131	160527583	3.514505	3.2206407	7.762744	9.5738537
LOC729603	NC_000006	160514114	160517244	0	0	0	0
LOC100289162	NC_000006	160542847	160581794	0.0237301	0.0487942	0.021111	0.0661444
SLC22A1	NC_000006	160542863	160579750	0	0.0711176	0.0820516	0.1285409
SLC22A2	NC_000006	160637790	160679959	0	0.017987	0.0155643	0
SLC22A3	NC_000006	160769425	160876014	0.0859585	0.0321361	0.0903749	0.0054454
LOC100289195	NC_000006	160874457	160885356	0	0	0	0.0340276
LPAL2	NC_000006	160887587	160932156	0.1930633	0.072178	0.1717545	0.0978431
LPA	NC_000006	160952515	161087407	0.0067727	0.0069631	0	0.009439
PLG	NC_000006	161123274	161174347	0.0319624	0.0164303	0.0284346	0.0222726
LOC100128831	NC_000006	161195020	161326977	0	0	0	0
MAP3K4	NC_000006	161412822	161538417	1.3222936	2.8754572	2.3170512	4.4619398
AGPAT4	NC_000006	161551057	161695107	0.2342716	0.3784877	0.893205	1.4575872
C6orf59	NC_000006	161581146	161583014	0	0	0	0
PARK2	NC_000006	161768590	163148834	0.1618521	0.0221868	0.3551706	0.3458739
LOC100129958	NC_000006	162989411	162990760	0	0	0	0
PACRG	NC_000006	163148164	163736524	0.0485616	0.124816	0	0.0845991
TRNAE33P	NC_000006	163210014	163210082	0	0	0	0
RPL34P15	NC_000006	163378281	163378580	0	0	0	0
RPL37AP4	NC_000006	163784230	163784506	0	0	0	0
QKI	NC_000006	163835675	163994896	4.1679834	4.435981	6.1281783	5.8779206
LOC100289273	NC_000006	164522035	164531530	0.0600386	0	0	0.0209186
LOC728316	NC_000006	165676257	165678521	0	0	0	0
C6orf118	NC_000006	165693153	165723111	0.0238979	0.0245695	0.0850409	0.016653
PDE10A	NC_000006	165740778	166075584	0.0475573	0.1955756	0.0094018	0.0073644
C6orf176	NC_000006	166337536	166401527	0	0	0	0
LOC441177	NC_000006	166401039	166403103	0	0	0	0
GAPDHL18	NC_000006	166477708	166478989	0	0	0	0
LOC729681	NC_000006	166511208	166511878	0	0	0	0
T	NC_000006	166571086	166582131	0.0349073	0.0179442	0.0155272	0.0121624
GNG5P	NC_000006	166653416	166654027	0	0	0	0
PRR18	NC_000006	166719168	166721871	0.2275426	0.1670984	0.0578366	0.0339773
SFT2D1	NC_000006	166733516	166755991	10.112545	5.1002972	13.409729	15.777795
LOC100289337	NC_000006	166739130	166749042	0	0	0	0
LOC100289495	NC_000006	166756119	166761643	0.2959477	0.0760663	0.3291041	0
BRP44L	NC_000006	166778407	166796486	4.9338971	6.2467697	7.2342689	9.2638665
LOC100130465	NC_000006	166796424	166798303	0.0265068	0	0.0235812	0.018471
RPS6KA2	NC_000006	166822854	167275771	25.709679	6.54648	24.887315	35.61856
LOC100127984	NC_000006	166822854	166826348	0.0502984	0.038784	0.0111867	0.0262874
FAM103A2P	NC_000006	166999006	167000150	0	0	0	0
RNASET2	NC_000006	167343004	167370077	3.5861757	1.6266025	2.5022442	1.6169936
LOC100289406	NC_000006	167369066	167370211	0.321259	0.0660576	0	0
FGFR1OP	NC_000006	167412816	167454066	1.3056239	1.4142285	5.6001818	2.989378
CCR6	NC_000006	167525295	167552629	0	0.0490192	0.0106042	0.0083062
GPR31	NC_000006	167570360	167571319	0.3662353	0.3765283	0.325813	0.0957027
TCP10L2	NC_000006	167584081	167596396	0.0201136	0	0.0178936	0.014016
UNC93A	NC_000006	167704803	167729502	0.0208187	0.0214038	0	0.0435219
LOC100289559	NC_000006	167704803	167709717	0	0	0	0
TTLL2	NC_000006	167738574	167756177	0	0.0314101	0.0543588	0.0212895
TCP10	NC_000006	167786577	167797998	0	0.0354936	0	0.0240572
LOC729995	NC_000006	167871479	167875742	0	0	0	0
LOC401286	NC_000006	168069829	168070320	0	0	0	0
LOC441178	NC_000006	168084341	168084736	0	0	0	0

C6orf123	NC_000006	168185217	168197539	0	0	0	0
LOC441179	NC_000006	168196616	168198928	0	0	0	0
C6orf124	NC_000006	168224570	168227476	0.038034	0.0195515	0.0338361	0.0927625
MLLT4	NC_000006	168227671	168372703	0.158164	0.1186608	0.6655067	1.0574683
HGC6.3	NC_000006	168376604	168377619	0.0432561	0.0444719	0.0384819	0.0904278
KIF25	NC_000006	168418553	168445769	0	0.0299228	0	0
FRMD1	NC_000006	168456464	168479839	0.0139873	0	0.0248871	0.0097469
DACT2	NC_000006	168707584	168720402	3.2415929	1.0597059	1.6611814	1.7071652
SMOC2	NC_000006	168842031	169068671	0.0745644	0	0	0.0519594
THBS2	NC_000006	169615875	169654137	12.785765	5.4058296	0.4576751	0.2477826
LOC100289471	NC_000006	169646160	169649099	0.0541234	0	0.0481497	0.0754307
LOC100130617	NC_000006	169696675	169721964	0	0	0	0
LOC100289536	NC_000006	169768139	169788530	0	0	0	0.0324416
DKFZp686L13185	NC_000006	169822767	169846566	0.0237494	0.0366253	0.0316922	0.0413738
WDR27	NC_000006	169857307	170102159	0.4691531	0.2607236	0.4512125	0.6803565
LOC100289601	NC_000006	170102216	170103729	0.3983223	1.5015632	0.2362391	0.5551337
C6orf120	NC_000006	170102257	170106402	5.2046748	3.7053441	4.5359211	3.5529586
PHF10	NC_000006	170104001	170124106	0.8926164	1.2415989	1.2845676	1.1342548
LOC729088	NC_000006	170124224	170126191	0	0	0	0
TCTE3	NC_000006	170140215	170151638	0.2353319	0	0.1046789	0.1639886
C6orf70	NC_000006	170151721	170181617	2.1995265	2.6962184	3.2361796	2.9622717
C6orf122	NC_000006	170188886	170198921	0	0	0	0
C6orf208	NC_000006	170190169	170202969	0	0	0	0
RPL12P23	NC_000006	170515014	170515459	0	0	0	0
LOC154449	NC_000006	170563422	170571657	0	0	0	0
DLL1	NC_000006	170591294	170599697	0.1194967	0.1774575	0.1063076	0.0277567
LOC100286936	NC_000006	170599226	170600170	0	0.1912525	0	0
FAM120B	NC_000006	170615844	170714237	3.2428612	4.1012412	4.9852716	9.4644962
PSMB1	NC_000006	170844204	170862417	32.877303	44.83849	65.446852	75.376609
TBP	NC_000006	170863471	170881947	1.4760511	1.2727719	2.0120632	2.7539165
PDCD2	NC_000006	170886450	170893748	6.1705262	9.5504024	9.710994	12.443913
OR4F7P	NC_000006	170948694	170949616	0	0	0	0
LOC100289674	NC_000006	170960229	170960510	0	0	0	0
RPL23AP47	NC_000006	171048925	171055059	0	0	0	0
LOC100132858	NC_000007	19828	36378	0.0761008	0	0.0338507	0
LOC100286915	NC_000007	161771	164889	0	0.028973	0.0250706	0.0098188
FAM20C	NC_000007	192969	300711	2.7317876	2.5457751	7.191337	7.5142831
LOC100288496	NC_000007	302775	304070	0	0	0	0.0665035
LOC100288524	NC_000007	326261	331454	0.0247456	0	0	0.0344875
LOC100286949	NC_000007	469653	470360	0	0.0638184	0	0.0432555
PDGFA	NC_000007	536895	559481	1.3611592	0.9651136	1.210925	1.0575341
PRKAR1B	NC_000007	589387	752133	2.1621347	0.8891607	1.1136023	1.5225211
HEATR2	NC_000007	766338	826116	4.8144812	5.9556329	7.3064578	7.0866004
UNC84A	NC_000007	856252	914539	5.2632613	5.3773659	7.091799	12.026836
C7orf20	NC_000007	916191	936071	3.5992086	3.1810154	2.9023576	5.0308109
ADAP1	NC_000007	937537	994289	0.4920991	0.2140471	0.1683788	0.2242131
LOC100286985	NC_000007	937548	938895	0	0	0	0
COX19	NC_000007	1004486	1015235	0.7008714	0.7482837	0.6075271	1.1082812
CYP2W1	NC_000007	1022835	1029276	0.0381495	0.0392217	0	0.0797523
C7orf50	NC_000007	1036623	1177893	5.8121791	7.4082608	13.274425	13.453156
GPR146	NC_000007	1097141	1098897	0	0	0	0
GPER	NC_000007	1126443	1133451	0.0427789	0	0	0.0099367
ZFAND2A	NC_000007	1192543	1199798	3.0722105	3.8009734	3.5206339	3.7011109
UNCX	NC_000007	1272654	1276613	0.055073	0.0283104	0.0244972	0.0191885
LOC100288559	NC_000007	1430371	1430589	0	0	0	0
MICALL2	NC_000007	1473995	1499109	0.9076111	0.9185396	1.1480731	1.0178646
INTS1	NC_000007	1509913	1544018	5.9236217	7.5154497	9.2782436	10.881624
MAFK	NC_000007	1570368	1582679	3.6601662	4.3969519	2.334183	3.8303952
TMEM184A	NC_000007	1581871	1596066	0.0770284	0.0863927	0.0498376	0.1512701
PSMG3	NC_000007	1606968	1609668	3.9811562	3.8052547	4.4548536	3.5544799
KIAA1908	NC_000007	1609904	1629257	0	0	0	0
TFAMP1	NC_000007	1654106	1656328	0	0	0	0
LOC401296	NC_000007	1732446	1733531	0	0	0	0
ELFN1	NC_000007	1784107	1787590	0.3153576	0.5965662	0.011222	0.0087901
MAD1L1	NC_000007	1855428	2272583	2.1990167	3.7955378	1.6849937	2.0468783
LOC100128374	NC_000007	1878222	1887418	0	0	0	0

FTSJ2	NC_000007	2273926	2281833	2.5803459	4.487296	1.6850294	3.6918186
NUDT1	NC_000007	2281857	2290780	5.3493075	7.2250309	6.4851571	7.4917662
LOC100287081	NC_000007	2290783	2292284	0.4681569	0.2105751	0.0260303	0.2446728
SNX8	NC_000007	2294638	2354099	9.2073605	10.615378	4.344174	7.563976
EIF3B	NC_000007	2394474	2420380	20.563326	30.664352	22.426587	42.531243
LOC100287125	NC_000007	2418867	2425618	0	0.0296674	0.0256714	0.1206496
LOC645357	NC_000007	2421617	2431910	0	0	0	0
LOC402634	NC_000007	2432729	2433770	0	0	0	0
CHST12	NC_000007	2443259	2474240	5.7323781	7.1887591	3.2316669	5.2821696
LOC100288594	NC_000007	2480699	2486315	0.5761004	0.3384524	0.2196492	0.05735
GRIFIN	NC_000007	2514772	2516489	0	0	0	0
LFNG	NC_000007	2559479	2568811	0.6072011	0.961708	0.2189931	0.1829716
C7orf27	NC_000007	2577511	2595146	4.5575944	5.1542547	4.4165769	5.8187269
IQCE	NC_000007	2598632	2654368	0.3274927	0.4687349	0.4627269	0.8412445
TTYH3	NC_000007	2671603	2704436	8.558148	12.17197	8.8703311	18.69121
AMZ1	NC_000007	2719163	2755069	0.3384462	0.0409363	0.0531337	0.1040483
GNA12	NC_000007	2767739	2883959	8.497059	8.7152663	10.545467	26.533182
LOC100131264	NC_000007	2875255	2875659	0	0	0	0
CARD11	NC_000007	2945769	3083579	0.0201044	0.0103347	0.0178854	0.0210143
LOC100129603	NC_000007	3180554	3208356	0	0	0	0.064338
SDK1	NC_000007	3341080	4308632	0.033106	0.0255273	0.0257705	0.0317207
RPL21P72	NC_000007	3377717	3378258	0	0	0	0
LOC730351	NC_000007	4305252	4308632	0	0	0	0
LOC100288658	NC_000007	4721601	4723482	0	0.0518753	0	0.0703212
FOXK1	NC_000007	4721930	4811074	2.246304	2.7414467	4.8561895	4.4797539
RNU13P2	NC_000007	4729060	4729264	0	0	0	0
KIAA0415	NC_000007	4815264	4831399	13.595059	10.428142	4.5726565	6.8667547
RADIL	NC_000007	4838740	4923335	0.107775	0.1477386	0.0213066	0
PAPOLB	NC_000007	4897369	4901625	0	0	0	0
RPL22P16	NC_000007	4905061	4905433	0	0	0	0
MMD2	NC_000007	4945620	4998844	0.0181905	0.0187017	0.0647311	0.0380276
RNF216L	NC_000007	5013616	5037800	0	0	0	0
LOC100130307	NC_000007	5071239	5077462	0	0	0	0
RBAK	NC_000007	5085553	5109119	0.8184528	1.1311369	1.4502684	1.9727826
LOC389458	NC_000007	5111738	5112854	0	0	0	0
OR10AH1P	NC_000007	5156620	5157818	0	0	0	0
LOC645700	NC_000007	5161203	5184177	0	0	0	0
WIPI2	NC_000007	5229835	5273486	9.2240453	10.490531	9.4311057	9.7574533
SLC29A4	NC_000007	5322561	5343704	0.1520174	0.5157566	0.3110495	0.0529659
TNRC18	NC_000007	5346421	5463177	3.1967641	2.6882671	2.403842	2.0885866
LOC100133305	NC_000007	5415620	5463724	0.1223698	0.0179727	0.0622077	0.0365452
LOC100129484	NC_000007	5467214	5469243	0	0	0	0
FBXL18	NC_000007	5515428	5553399	1.4793608	1.7790038	1.0167553	1.4811887
ACTB	NC_000007	5566779	5570232	636.88555	948.57715	625.0432	863.88523
LOC100288712	NC_000007	5569807	5600688	0	0	0	0
LOC100288744	NC_000007	5632246	5635063	0.1822066	0.149862	0.0972576	0.0761813
FSCN1	NC_000007	5632454	5646286	29.135288	42.515027	12.136155	12.759447
LOC100287342	NC_000007	5643492	5646286	0.2537101	0.1159292	0.1003145	0.0785757
RNF216	NC_000007	5659678	5821292	3.9648535	4.4283646	3.2361275	5.186343
ZNF815	NC_000007	5862791	5894066	0	0	0	0
OCM	NC_000007	5920429	5925994	0	0	0	0.0440013
C7orf28A	NC_000007	5938341	5965605	3.0085044	4.7014493	5.2458399	4.2264345
RSPH10B	NC_000007	5965777	6010314	0.0141495	0.0872828	0.0125878	0.0788793
PMS2	NC_000007	6012870	6048737	3.068318	3.7281086	3.2121766	3.4339603
JTV1	NC_000007	6048882	6063465	2.6678992	3.5212659	2.8545392	3.5423362
EIF2AK1	NC_000007	6061878	6098860	4.2127308	5.1204482	10.928054	11.001636
USP42	NC_000007	6144550	6201195	0.8583639	1.3237325	1.2447077	1.0706745
CYTH3	NC_000007	6201410	6312242	3.5205789	3.6296356	2.0121817	2.3230776
C7orf70	NC_000007	6369040	6388590	4.1685315	5.8571077	6.8694458	5.9064241
RAC1	NC_000007	6414126	6443598	33.685092	44.750033	34.564987	31.735956
DAGLB	NC_000007	6448747	6487643	3.1262144	3.2451309	2.781167	4.6831859
KDELR2	NC_000007	6500712	6523849	63.417606	79.260534	74.777512	61.945777
RPL31P34	NC_000007	6530372	6530732	0	0	0	0
GRID2IP	NC_000007	6536409	6591067	0.0189759	0	0.0084408	0.0330579
ZDHHC4	NC_000007	6617065	6628610	10.321587	9.6063599	11.28465	10.656246
C7orf26	NC_000007	6629915	6648355	2.3203196	3.9207761	2.616042	4.1943116

ZNF853	NC_000007	6655527	6663921	0.1351119	0.0883968	0.0437088	0.0342369
ZNF316	NC_000007	6681537	6694242	0	0	0	0
LOC100133111	NC_000007	6703506	6716133	0	0	0	0
ZNF12	NC_000007	6728064	6746566	2.2915926	2.3827708	3.0734409	2.6191136
LOC100130530	NC_000007	6755261	6761679	0	0	0	0
LOC100288876	NC_000007	6769391	6771581	0	0	0	0
PMS2CL	NC_000007	6774936	6791232	0	0	0	0
RSPH10B2	NC_000007	6793740	6838396	0	0	0.0251755	0.0591595
C7orf28B	NC_000007	6838572	6865861	9.2627435	9.5998729	6.2024467	7.8253938
OR7E39P	NC_000007	6874454	6875425	0	0	0	0
OR7E136P	NC_000007	6906287	6907253	0	0	0	0
OR7E59P	NC_000007	6919253	6920211	0	0	0	0
LOC100130527	NC_000007	6928031	6928357	0	0	0	0
LOC647415	NC_000007	6963443	6971462	0	0	0	0
LOC641924	NC_000007	6970611	6979244	0	0	0	0
LOC100128349	NC_000007	6982902	6983075	0	0	0	0
LOC100131257	NC_000007	7117926	7141265	0.0343883	0.1060643	0	0.0239631
C1GALT1	NC_000007	7222246	7283981	12.517763	4.9193487	6.9197446	9.2443518
COL28A1	NC_000007	7398244	7575460	0.0250061	0.0385634	0.0444923	0.069701
MIOS	NC_000007	7606616	7647112	3.6215987	4.0495199	3.1278053	4.946033
RPA3	NC_000007	7676575	7758238	4.4940789	6.5479083	6.9659402	8.895432
LOC729852	NC_000007	7680366	7918854	0	0	0	0
RPL23AP51	NC_000007	7781737	7782193	0	0	0	0
LOC100132073	NC_000007	7948927	7950092	0	0	0	0
GLCCI1	NC_000007	8008423	8128710	0.5371965	0.7332185	0.5603023	0.709955
ICA1	NC_000007	8152814	8302185	0.2817194	0.0181023	0.0939845	0.0368087
NXPH1	NC_000007	8473585	8792593	0	0.0773424	0.013385	0.0419375
RPL9P19	NC_000007	8978401	8979084	0	0	0	0
GAPDHL13	NC_000007	9654538	9655816	0	0	0	0
PER4	NC_000007	9673900	9675447	0	0	0	0
LOC340268	NC_000007	9765870	10162566	0	0	0	0
LOC401308	NC_000007	10490763	10493236	0	0	0	0
LOC100287551	NC_000007	10490940	10493131	0	0	0	0
LOC100128638	NC_000007	10513527	10516720	0	0	0	0
NDUFA4	NC_000007	10972815	10979813	18.423013	34.862042	36.057172	25.303666
PHF14	NC_000007	11013516	11209245	1.0267382	1.7954976	0.8536586	1.7519035
RPL23AP52	NC_000007	11046026	11046479	0	0	0	0
THSD7A	NC_000007	11414170	11871824	0.0542822	0.0558078	0	0.037826
TMEM106B	NC_000007	12250848	12276890	2.928083	4.5779928	4.4535452	4.7107964
VWDE	NC_000007	12370509	12443852	0	0.3965586	0.1960831	0.1919883
LOC389465	NC_000007	12510968	12512452	0	0	0	0
PS9	NC_000007	12530721	12531630	0	0	0	0
TAS2R2	NC_000007	12530721	12531630	0	0	0	0
SCIN	NC_000007	12610203	12693228	0.1066381	0.4659495	0.0355756	0.0185774
LOC100287584	NC_000007	12723721	12726376	0	0.0340236	0.0441614	0.0230609
ARL4A	NC_000007	12726481	12730559	0.5116361	0.9306432	1.505548	1.2981292
RPL26P21	NC_000007	13911409	13912181	0	0	0	0
ETV1	NC_000007	13930854	14028764	3.3400085	3.9548317	4.4062537	4.0034146
RPL6P21	NC_000007	14110131	14111024	0	0	0	0
DGKB	NC_000007	14184674	14881075	0.0232838	0.0837838	0.0155355	0.044619
LOC100128637	NC_000007	14195183	14237020	0	0	0	0
LOC100128217	NC_000007	15024815	15212070	0	0	0	0
TMEM195	NC_000007	15239943	15601640	0.1775686	0	0	0
MEOX2	NC_000007	15650837	15726308	3.6886116	0.0952834	0.0989394	0.3874932
RPL36AP26	NC_000007	15741003	15741406	0	0	0	0
LOC100289069	NC_000007	16128696	16131043	0	0	0	0
LOC729920	NC_000007	16131158	16460947	0.4053197	0.327416	0.2318037	0.6859327
RPL36AP29	NC_000007	16248573	16248893	0	0	0	0
SOSTDC1	NC_000007	16501106	16505474	0.0235143	0	0.041838	0.0491571
LOC100129335	NC_000007	16552444	16621114	0	0	0	0
LOC317727	NC_000007	16622868	16626546	0	0	0	0
ANKMY2	NC_000007	16639412	16685398	5.3616843	4.9701742	5.7864397	5.6962274
BZW2	NC_000007	16685759	16746147	5.3277759	6.6703945	6.7198941	10.956314
TSPAN13	NC_000007	16793351	16824161	0.2310633	0.4513589	1.0278014	0.6118535
AGR2	NC_000007	16832264	16844738	0.0894165	0.0919296	0.1193212	0.0934635
LOC100287613	NC_000007	16889582	16893171	0	0	0.048448	0.0758981

AGR3	NC_000007	16899030	16921613	0	0.0602445	0.0521301	0.0408332
RAD17P1	NC_000007	16903507	16905994	0	0	0	0
LOC100131425	NC_000007	16928245	16928515	0	0	0	0
LOC100131512	NC_000007	17325899	17338978	0.6547223	0.3365617	0.4368443	0.3421774
AHR	NC_000007	17338307	17385774	22.049706	9.8464689	18.432478	13.331883
SNX13	NC_000007	17830385	17980131	3.5732513	3.9786358	6.3515901	6.4220432
PRPS1L1	NC_000007	18066397	18067486	0	0.0414527	0.0358693	0.0561924
HDAC9	NC_000007	18535369	19036993	5.7696203	2.7866138	5.3342078	4.3061572
NPM1P13	NC_000007	18988214	18989478	0	0	0	0
TWIST1	NC_000007	19155091	19157295	14.798894	5.0444854	19.032488	7.2610006
FERD3L	NC_000007	19184405	19185044	0.3433456	0.0705991	0	0.0478514
TWISTNB	NC_000007	19735085	19748660	1.8405863	1.9735813	3.094052	2.9979647
TMEM196	NC_000007	19758938	19812404	0.0261286	0.0134315	0	0.0364148
RPL21P75	NC_000007	20042348	20042915	0	0	0	0
MACC1	NC_000007	20174278	20257013	0.0239918	0.0246661	0.0298813	0.0702175
ITGB8	NC_000007	20370725	20455382	2.9192001	0.7464502	2.6148195	6.1480045
LOC100130234	NC_000007	20593777	20622836	0	0	0	0
LOC402641	NC_000007	20628648	20629544	0	0	0	0
ABCB5	NC_000007	20687216	20795296	0.110502	0.0324593	0	0.0440013
SP8	NC_000007	20821894	20826508	0.0117855	0.0242335	0.0419389	0.0164252
RPL23P8	NC_000007	20866917	20867439	0	0	0	0
RPS26P30	NC_000007	20883249	20883586	0	0	0	0
ASSP11	NC_000007	21259597	21261152	0	0	0	0
RNU1P8	NC_000007	21420981	21421112	0	0	0	0
SP4	NC_000007	21467689	21554162	0.2798287	0.3421218	1.0428709	1.2068658
DNAH11	NC_000007	21582833	21941457	0.0774391	0.0764309	0.1074715	0.2741302
CDCA7L	NC_000007	21940517	21985542	4.3028316	3.5695193	7.6426369	12.293376
RAPGEF5	NC_000007	22157908	22396533	0.0730037	0.0477626	0.1476048	0.2173617
MGC87042	NC_000007	22526422	22544077	3.605509	1.5695639	2.4851372	4.5269591
EEF1AL4	NC_000007	22549936	22551681	0	0	0	0
LOC541472	NC_000007	22765802	22765957	0	0	0	0
IL6	NC_000007	22766818	22771620	8.0824334	1.1984987	3.007505	0.812331
RPS26P32	NC_000007	22813189	22813638	0	0	0	0
TOMM7	NC_000007	22852251	22862421	23.330543	26.589409	34.592496	27.936274
SNORD93	NC_000007	22896232	22896305	0	0	0	0
RPL12P10	NC_000007	22921440	22922051	0	0	0	0
FAM126A	NC_000007	22980878	23053770	5.0728734	3.9666781	8.2822513	10.380893
KLHL7	NC_000007	23145353	23215040	3.9856317	3.5922717	3.6639194	3.4624258
NUPL2	NC_000007	23221446	23240630	3.1793379	5.2550534	4.9171118	6.1692854
LOC730020	NC_000007	23242018	23247664	0	0	0	0
GPNMB	NC_000007	23286316	23314729	73.374163	34.385778	53.161947	32.20816
C7orf30	NC_000007	23338940	23349180	1.5716586	3.7104251	2.6928125	3.8128988
IGF2BP3	NC_000007	23349828	23509995	2.250775	0.7496164	8.2632268	4.9630123
LOC389473	NC_000007	23520202	23522188	0	0	0	0
RPS2P32	NC_000007	23530007	23531047	0	0	0	0
TRA2A	NC_000007	23544399	23571656	2.5304251	3.3623716	1.0406196	2.0959993
LOC442517	NC_000007	23602011	23603985	0	0	0	0
CLK2P	NC_000007	23624335	23626146	0	0	0	0
CCDC126	NC_000007	23636998	23684327	0.5153654	0.6394739	0.5059127	0.6068011
C7orf46	NC_000007	23719749	23742269	0.2687052	0.2148667	0.1328042	0.2288544
STK31	NC_000007	23749838	23872127	0.0363209	0.0373417	0.0538534	0.0674928
NPY	NC_000007	24323809	24331477	0	0	0	0
MPP6	NC_000007	24613085	24727498	2.1702831	3.5535186	3.1285203	2.7586196
DFNA5	NC_000007	24737974	24797639	5.4849587	4.035491	9.7133968	6.8081828
OSBPL3	NC_000007	24836159	25019760	2.8417063	3.1100613	4.2289679	5.543687
CYCS	NC_000007	25158275	25164955	1.4681327	2.0426048	2.8251327	2.6632746
C7orf31	NC_000007	25174316	25219817	0.2922576	0.2629126	0.368334	0.2545709
RPL7AP41	NC_000007	25251783	25252604	0	0	0	0
NPVF	NC_000007	25264189	25268105	0	0.0379692	0	0.0257352
LOC100131016	NC_000007	25305541	25305908	0	0	0	0
UBA52P1	NC_000007	25729016	25729690	0	0	0	0
NFE2L3	NC_000007	26191860	26225907	3.9349716	0.777383	13.343692	3.0646385
HNRNPA2B1	NC_000007	26229556	26240413	23.873553	30.305345	40.713429	69.249286
CBX3	NC_000007	26241099	26252976	14.520641	17.59209	16.617476	19.049498
RPL23P7	NC_000007	26315781	26316286	0	0	0	0
SNX10	NC_000007	26331515	26413949	0.3409483	0.6134287	3.1393313	0.9265867

LOC100129036	NC_000007	26411661	26416414	0.0779915	0	0	0.0543476
LOC100289338	NC_000007	26437325	26519642	0.0447538	0	0.0398142	0.0311862
LOC441204	NC_000007	26526288	26535987	0	0	0	0
KIAA0087	NC_000007	26572740	26578444	0	0	0	0
LOC285941	NC_000007	26677490	26686924	0	0	0.0589114	0.0307633
SKAP2	NC_000007	26706687	26904341	4.5818605	3.9482852	7.3744842	8.7995432
RPL7AP38	NC_000007	26961723	26962585	0	0	0	0
LOC442661	NC_000007	27022387	27034836	0	0	0	0
LOC402643	NC_000007	27063289	27066099	0	0	0	0
LOC442290	NC_000007	27087754	27088508	0	0	0	0
HOXA1	NC_000007	27132612	27135625	0.1724136	0.1240815	0.8436117	0.949143
HOXA2	NC_000007	27139973	27142394	0.2224601	0	0.1099482	0.103346
HOXA3	NC_000007	27145809	27166639	0.0866097	0.1272055	0.110072	0.2414123
LOC285943	NC_000007	27154967	27162506	0	0	0	0
HOXA4	NC_000007	27168126	27170399	0.3560621	0.522956	1.2444248	2.0558367
LOC100133311	NC_000007	27170641	27192345	0.2398921	0.1973074	0.2987805	0.2674662
HOXA5	NC_000007	27180996	27183287	0.0329942	0	0.0880576	0.0919666
HOXA6	NC_000007	27185116	27187368	0	0.1570686	0.0453042	0
HOXA7	NC_000007	27193335	27196296	0.4573404	0.3358528	0.8137254	1.5175856
LOC100289444	NC_000007	27199952	27209863	1.3904525	0.8338933	1.3695192	2.4223068
HOXA9	NC_000007	27202057	27205149	0.2350173	0.0878627	0.1520566	0.1637694
HOXA10	NC_000007	27210210	27219876	1.5602106	1.4621082	1.8302034	2.0012487
HOXA11	NC_000007	27220776	27224835	2.1203821	1.12405	0.7515929	0.5771745
HOXA11AS	NC_000007	27225027	27228912	0	0	0	0
HOXA13	NC_000007	27236499	27239725	0.2447396	0	0.1710713	0.1096356
LOC100129463	NC_000007	27274968	27286750	0.0730644	0.1126768	0.1300002	0.0509142
EVX1	NC_000007	27282164	27286192	0.047307	0	0	0.0164827
RPL35P4	NC_000007	27308934	27309372	0	0	0	0
LOC100129606	NC_000007	27368127	27373527	0	0	0	0
LOC401316	NC_000007	27495991	27498476	0	0	0	0
LOC442291	NC_000007	27500671	27506646	0	0	0	0
LOC442292	NC_000007	27531301	27532382	0	0	0	0
HIBADH	NC_000007	27565061	27702602	8.3097263	7.4011235	9.5866124	10.326994
TAX1BP1	NC_000007	27779738	27869386	15.405973	16.243081	13.423573	17.194464
JAZF1	NC_000007	27870192	28220437	2.939038	2.2309305	1.9548783	1.9906171
LOC100128081	NC_000007	28219947	28280478	0.1259262	0.1294653	0.1120274	0.0438752
LOC402644	NC_000007	28318842	28319318	0	0	0.0819655	0.0642031
CREB5	NC_000007	28338940	28865511	1.1658413	0.2561127	0.4255035	0.7325529
RPS2P30	NC_000007	28654714	28655481	0	0	0	0
KIAA0644	NC_000007	28992974	28998029	0.1390767	0.4736393	0.1159936	0.0121143
CPVL	NC_000007	29035247	29186153	0.1482234	3.7589339	3.0987953	0.6369424
CHN2	NC_000007	29234121	29553944	0.034281	0.4229336	0.0609946	0.0875907
NANOGP4	NC_000007	29251737	29253503	0	0	0	0
LOC646745	NC_000007	29591137	29591757	0	0	0	0
PRR15	NC_000007	29603427	29606911	0.2619084	3.5274289	0.0699003	0
LOC646762	NC_000007	29685538	29724754	0	0	0	0
LOC100289602	NC_000007	29724170	29727859	0.3344315	0.3236054	0.0700046	0.2193366
DPY19L2P3	NC_000007	29771461	29782302	0	0	0	0
WIPF3	NC_000007	29915437	29945791	0.0278418	0	0	0.0388025
SCRN1	NC_000007	29959719	30029905	21.427929	25.70864	18.696692	32.505837
FKBP14	NC_000007	30052884	30066268	3.2076146	4.4439394	1.7747894	2.1261598
PLEKHA8	NC_000007	30068288	30130360	0.7756874	0.8202736	0.9661022	0.8802915
C7orf41	NC_000007	30174552	30202381	1.1036192	0.9481209	1.2171757	3.0077065
RPS27P16	NC_000007	30207444	30207620	0	0	0	0
ZNRF2	NC_000007	30323923	30407308	1.7528486	2.324464	2.5650715	2.5579739
DKFZP586I1420	NC_000007	30409666	30412425	0	0	0	0
NOD1	NC_000007	30464143	30518393	1.1045708	0.8240745	0.7478627	0.9399985
GGCT	NC_000007	30536237	30544452	1.8484664	4.0335397	5.7387843	7.2290485
LOC100287795	NC_000007	30536237	30544417	0.9812179	0.9557006	0.4134878	0.3238824
LOC100287825	NC_000007	30554738	30565824	0	0	0.1902558	0.5215916
GARS	NC_000007	30634181	30673649	42.046472	44.805504	38.756351	39.478252
CRHR2	NC_000007	30692863	30722140	0	0	0.0461873	0.0180891
INMT	NC_000007	30791751	30797218	0.2232618	0.10594	0.3055691	0.0957401
C7orf67	NC_000007	30811033	30932002	0.4663369	0.4959759	0.6866751	2.0170063
AQP1	NC_000007	30951468	30965131	0.1117057	0.3117228	0.0709832	0.0667209
LOC100287857	NC_000007	30951468	30965132	0.0599294	0.225917	0.0888581	0.0556816

GHRHR	NC_000007	31003636	31019143	0	0.0264385	0	0
ADCYAP1R1	NC_000007	31092142	31146311	0.0267488	0	0	0
LOC100289642	NC_000007	31158749	31159184	0	0	0	0
NEUROD6	NC_000007	31377079	31380538	0.0203747	0.0209473	0.0181259	0
LOC100289675	NC_000007	31426617	31435482	0	0	0	0
CCDC129	NC_000007	31556978	31693303	0.0176216	0	0.023515	0.0306986
C7orf16	NC_000007	31726799	31748069	0.0488857	0.0251298	0	0.0510982
PDE1C	NC_000007	31829247	32110991	1.4029502	0.7882776	1.1900521	3.9787333
LOC100130673	NC_000007	32486042	32497793	0	0	0	0
SLC25A5P5	NC_000007	32511041	32512613	0	0	0	0
LSM5	NC_000007	32524945	32534870	0.6108769	0.8074873	1.211124	1.3986739
AVL9	NC_000007	32535176	32623779	4.46393	3.8212907	3.2899779	5.2191144
DPY19L1P1	NC_000007	32660015	32763222	0	0	0	0
LOC441208	NC_000007	32767742	32769595	0	0	0	0
LOC401321	NC_000007	32797898	32802536	0.2178938	0.1558384	0.2612685	0.4951209
LOC100133317	NC_000007	32861140	32878316	0	0	0	0
KBTBD2	NC_000007	32907778	32931468	4.1579055	4.9811351	4.8265997	5.5223837
RP9P	NC_000007	32956427	32982782	0	0	0	0
FKBP9	NC_000007	32997005	33046543	12.840373	20.263598	15.85736	13.487641
NT5C3	NC_000007	33053742	33102409	4.2912415	4.9189562	3.2252201	4.0214487
RPS29P14	NC_000007	33075824	33075994	0	0	0	0
RP9	NC_000007	33134409	33149002	4.4693117	7.6582037	3.6272496	3.0597559
BBS9	NC_000007	33169152	33645680	1.8370995	1.6413975	1.4300428	2.179327
FLJ20712	NC_000007	33765593	33768068	0	0	0	0
LOC100288021	NC_000007	33769904	33771676	0	0	0.0677601	0.1592281
BMPER	NC_000007	33945112	34194112	1.7713762	2.5921634	0.1840427	0.4775283
NPSR1	NC_000007	34697897	34917944	0.0481889	0.0247716	0	0
RPL7P31	NC_000007	34750715	34751094	0	0	0	0
AAA1	NC_000007	34758474	34873941	0	0	0	0
DPY19L1	NC_000007	34968493	35077653	3.2520788	5.767501	6.3407974	10.216686
DPY19L2P1	NC_000007	35129456	35147346	0	0	0	0
LOC347701	NC_000007	35208787	35209317	0	0	0	0
TBX20	NC_000007	35242042	35293242	0.0323387	0.7979409	0	0.0450697
HERPUD2	NC_000007	35672269	35734772	3.759227	3.0267136	4.6068241	3.8820268
LOC442293	NC_000007	35807725	35808249	0	0	0	0
SEPT7	NC_000007	35840627	35944917	15.938419	21.38287	20.39334	22.346572
LOC646913	NC_000007	35985900	36014302	0	0	0	0
LOC100286896	NC_000007	36010757	36011553	0	0	0	0
LOC100129326	NC_000007	36076632	36122843	0	0	0	0
LOC100131181	NC_000007	36147007	36152144	0	0	0	0
EEPD1	NC_000007	36192836	36341152	1.4158701	1.6388262	0.7841202	1.457083
LOC100286994	NC_000007	36345240	36345546	0	0	0.210202	0
KIAA0895	NC_000007	36363759	36429734	0.2414738	0.2780517	0.9624016	0.6999972
ANLN	NC_000007	36429432	36493400	15.966936	27.214238	21.00777	39.78024
AOAH	NC_000007	36552607	36764153	0.0369468	0.0189926	0.0164345	0.038619
NPM1P18	NC_000007	36858468	36859623	0	0	0	0
ELMO1	NC_000007	36893961	37488511	0.0327483	0.0112229	0.0097113	0.0456407
RPS17P13	NC_000007	37156413	37156790	0	0	0	0
RPS10P14	NC_000007	37415096	37415676	0	0	0	0
LOC442668	NC_000007	37624717	37625937	0	0	0	0
GPR141	NC_000007	37779996	37780913	0.0478739	0.0984388	0.0425899	0
TXNDC3	NC_000007	37888199	37940003	0	0.019543	0	0.0132461
SFRP4	NC_000007	37945534	37956525	2.4087296	0.2582777	0.0788787	0.0102975
EPDR1	NC_000007	37960163	37991543	14.3394	8.3447606	8.0481715	6.9875155
LOC100288050	NC_000007	37960303	37991623	1.2603317	0.0239954	1.4534411	1.4149572
STARD3NL	NC_000007	38217933	38270270	13.45354	11.456521	19.36744	18.011292
TRG@	NC_000007	38279625	38407656	0	0	0	0
TRGC2	NC_000007	38279625	38289173	0	0	0	0
TRGJ2	NC_000007	38292981	38293030	0	0	0	0
TRGJP2	NC_000007	38295938	38295997	0	0	0	0
TARP	NC_000007	38299244	38313248	0	0	0.0380697	0
TRGC1	NC_000007	38299689	38305279	0	0	0	0
TRGJ1	NC_000007	38309092	38309141	0	0	0	0
TRGJP	NC_000007	38313188	38313249	0	0	0	0
TRGJP1	NC_000007	38315860	38315919	0	0	0	0
TRGV11	NC_000007	38331217	38331679	0	0	0	0

TRGVB	NC_000007	38335364	38335834	0	0	0	0
TRGV10	NC_000007	38339411	38339881	0	0	0	0
TRGV9	NC_000007	38356618	38357093	0	0	0	0
TRGVA	NC_000007	38362047	38362464	0	0	0	0
TRGV8	NC_000007	38369944	38370422	0	0	0	0
TRGV7	NC_000007	38374642	38375115	0	0	0	0
TRGV6	NC_000007	38380301	38380772	0	0	0	0
TRGV5P	NC_000007	38384631	38385100	0	0	0	0
TRGV5	NC_000007	38388956	38389425	0	0	0	0
TRGV4	NC_000007	38393316	38393784	0	0	0	0
TRGV3	NC_000007	38398113	38398580	0	0	0	0
TRGV2	NC_000007	38402465	38402929	0	0	0	0
TRGV1	NC_000007	38407187	38407656	0	0	0	0
AMPH	NC_000007	38423305	38671020	0.3358929	4.4893322	1.4821456	1.5541821
KRT8P20	NC_000007	38694744	38696462	0	0	0	0
FAM183B	NC_000007	38724946	38726689	0.0266838	0	0.0237387	0
VPS41	NC_000007	38763543	38948800	7.5162791	5.7425574	6.6467459	8.9484217
POU6F2	NC_000007	39046408	39504390	0.0395396	0	0.0175877	0
C7orf36	NC_000007	39606009	39612471	4.1586528	5.5951409	3.7910023	5.116072
LOC100101126	NC_000007	39607217	39609830	0	0	0	0
LOC646999	NC_000007	39649086	39651687	0	0	0	0
RALA	NC_000007	39663162	39747719	7.1360863	13.525933	7.2433974	7.7328913
LOC349114	NC_000007	39773167	39834222	0	0	0	0
LOC100288083	NC_000007	39833821	39837997	0	0	0	0
LOC100131851	NC_000007	39871433	39874549	0.9487075	0.3426979	0.0912429	0.035735
LOC100129321	NC_000007	39872813	39874549	0	0	0	0
LOC647009	NC_000007	39892296	39894859	0	0	0	0
LOC100128795	NC_000007	39899640	39989216	0	0	0	0
LOC100128370	NC_000007	39987120	39989990	0.2449222	0.2518058	0.1770353	0.1280037
CDC2L5	NC_000007	39989959	40135154	2.4515229	3.1233152	2.3475929	3.3769093
LOC100288154	NC_000007	40155282	40155545	0	0	0	0
C7orf11	NC_000007	40172342	40174258	3.6623526	4.3848871	7.4648306	4.5549661
C7orf10	NC_000007	40174575	40900357	0.7652677	1.0115687	1.0455195	0.6856316
INHBA	NC_000007	41728601	41742706	3.0309125	7.2916662	0.161783	0.7885026
LOC285954	NC_000007	41733514	41818976	0	0	0	0
GLI3	NC_000007	42000547	42276618	1.0164533	1.3255265	1.4801391	2.0429012
LOC100131183	NC_000007	42152964	42153222	0	0	0	0
TCP1P1	NC_000007	42834504	42840607	0	0	0	0
C7orf25	NC_000007	42948872	42951689	4.5513511	5.2982181	5.5272175	4.5475847
PSMA2	NC_000007	42956460	42971805	29.918373	39.635645	44.298129	52.350576
LOC100132780	NC_000007	42958346	42959690	0	0	0	0
MRPL32	NC_000007	42971939	42977456	7.7584588	11.611377	10.222157	9.9915804
LOC100288217	NC_000007	43152163	43351583	0.2212159	0.0758111	0	0.1541521
HECW1	NC_000007	43152198	43602938	0.0706873	0.0924942	0.080036	0.1343393
RNU7-35P	NC_000007	43234021	43234081	0	0	0	0
RPL18AP10	NC_000007	43313758	43314181	0	0	0	0
STK17A	NC_000007	43622692	43666978	6.2799151	7.5592877	7.9924848	32.657193
C7orf44	NC_000007	43678859	43769083	8.2563189	12.073511	25.821729	23.835232
BLVRA	NC_000007	43798272	43846941	4.95993	3.5528114	6.5102329	7.1108644
MRPS24	NC_000007	43906157	43909145	24.815596	31.225886	44.546508	32.516933
URG4	NC_000007	43915501	43965996	0.8825855	0.7561589	0.734841	1.4192786
TUBG1P	NC_000007	43958236	43959851	0	0	0	0
UBE2D4	NC_000007	43966047	43993163	3.7409439	4.4924842	5.1738553	11.391228
POLR2J4	NC_000007	43980494	44058748	0	0	0	0
SPDYE1	NC_000007	44040489	44049723	0.0497527	0.0170503	0	0
LOC100287164	NC_000007	44059982	44067028	0.1127601	0	0.0250786	0.0196439
RASA4P	NC_000007	44068486	44080222	0	0	0	0
FLJ35390	NC_000007	44078698	44082082	0	0	0	0
DBNL	NC_000007	44084239	44101315	21.260799	22.225019	12.638843	18.681452
PGAM2	NC_000007	44102326	44105163	1.317393	0.8668279	0.8907119	0.4773662
POLM	NC_000007	44111846	44122129	2.1965732	2.4479371	2.9834083	3.0730038
AEBP1	NC_000007	44143960	44154159	15.50734	10.949861	0.9771996	0.5703236
POLD2	NC_000007	44154286	44163147	34.564356	54.449181	51.03609	78.10638
MYL7	NC_000007	44178463	44180916	0	0.0756841	0	0
GCK	NC_000007	44183870	44229022	0.0429042	0.1029235	0.0127229	0
YKT6	NC_000007	44240578	44253893	17.064498	26.806205	14.969025	24.048169

CAMK2B	NC_000007	44256749	44365230	0.1244992	0.0886142	0.0511191	0.0667354
NUDCD3	NC_000007	44421965	44530385	5.2466162	5.7800203	5.4070255	7.51141
RPL32P18	NC_000007	44507442	44508580	0	0	0	0
RPL36AP27	NC_000007	44550571	44550952	0	0	0	0
NPC1L1	NC_000007	44552134	44580914	0.0174121	0.0537045	0.0619613	0.0849343
DDX56	NC_000007	44605403	44614137	3.0418897	4.2183297	2.8949505	4.9542057
TMED4	NC_000007	44618762	44621827	20.813057	20.292741	40.206988	30.415119
OGDH	NC_000007	44646171	44748669	16.97511	17.163731	16.740647	16.57354
LOC100288256	NC_000007	44747532	44748669	0.0386188	0	0.0343564	0.0269111
ZMIZ2	NC_000007	44788530	44809480	5.7754675	3.5749698	8.4898874	11.377166
PPIA	NC_000007	44836241	44842716	52.405756	64.003202	88.570759	95.817117
H2AFV	NC_000007	44866488	44887725	8.754144	13.658758	13.473034	19.908775
PURB	NC_000007	44915892	44924960	0.5330583	0.687541	0.7673797	1.5195937
MRPS23P1	NC_000007	44959186	44960089	0	0	0	0
MYO1G	NC_000007	45002261	45018668	0.0271118	0.0557476	0.0241194	0.0472315
C7orf40	NC_000007	45022627	45026259	0	0	0	0
SNORA9	NC_000007	45024977	45025109	0	0	0	0
CCM2	NC_000007	45039787	45116069	8.2712631	13.049499	7.0362054	7.3308468
LOC100128364	NC_000007	45117402	45119749	0	0	0	0
NACAD	NC_000007	45120031	45128493	0.138318	0.3223323	0.0738309	0.0835341
TBRG4	NC_000007	45139699	45151317	12.458845	18.097491	14.180915	17.091054
LOC100287196	NC_000007	45142013	45151280	0.2006769	0.1375446	0.0595092	0.0932264
SNORA5A	NC_000007	45143948	45144081	0	0	0	0
SNORA5C	NC_000007	45144505	45144641	0	0	0	0
SNORA5B	NC_000007	45145567	45145698	0	0	0	0
RAMP3	NC_000007	45197367	45223849	0	0	0.0290472	0
LOC647102	NC_000007	45431072	45431737	0	0	0	0
ADCY1	NC_000007	45614125	45762715	0.049226	0.1012189	0.0406647	0.0294022
LOC100288357	NC_000007	45763383	45767703	0.0915376	0.0313701	0.0180965	0.0354373
SEPT13	NC_000007	45763386	45808617	0	0	0	0
LOC100129904	NC_000007	45847165	45848086	0	0.2141393	0.4323585	0.2902832
LOC100287230	NC_000007	45849818	45850389	0	0	0	0
LOC730234	NC_000007	45850406	45851278	0	0	0	0
LOC730235	NC_000007	45851932	45854865	0	0	0	0
LOC100129050	NC_000007	45856768	45865030	0	0	0	0
IGFBP1	NC_000007	45927959	45933267	0.6658823	0	0.047391	0.0742421
IGFBP3	NC_000007	45951844	45960871	374.95475	133.66112	13.641796	8.2527703
LOC100129619	NC_000007	45960492	45960902	0.1273862	0	0	0.0887678
LOC100287349	NC_000007	45977482	45978924	0.0654966	0	0	0.136922
RNU7-76P	NC_000007	46014880	46015136	0	0	0	0
LOC442304	NC_000007	46037139	46039165	0	0	0	0
LOC222052	NC_000007	46039312	46040377	0	0	0	0
LOC100287431	NC_000007	46514891	46674544	0	0	0	0
EPS15L2	NC_000007	46821588	46823018	0	0	0	0
MRPL42P4	NC_000007	47065720	47066070	0	0	0	0
LOC647145	NC_000007	47092057	47094618	0	0	0	0
TNS3	NC_000007	47314752	47579199	2.4950288	2.914676	5.8336213	6.4445955
C7orf65	NC_000007	47694842	47701246	0.0150973	0.0310432	0.0268619	0.0315612
PKD1L1	NC_000007	47814290	47988037	0.0195304	0.0903568	0.0304058	0.0544382
C7orf69	NC_000007	47834889	47859445	0.0661871	0	0	0.0461218
HUS1	NC_000007	48003783	48019246	1.9892573	2.2138391	2.3352722	2.7009342
SUNC1	NC_000007	48026745	48068716	0.0305833	0.0943286	0	0.1918051
C7orf57	NC_000007	48075117	48100894	0.0827261	0.0850511	0.128792	0.1729405
UPP1	NC_000007	48128355	48148330	11.151044	9.2785267	8.4111078	8.8895684
ABCA13	NC_000007	48211057	48687091	0.1125304	0.0788816	0.0546055	0.7413844
CDC14BL	NC_000007	48885998	48887677	0	0	0	0
GDI2P	NC_000007	48941420	48943601	0	0	0	0
CDC14C	NC_000007	48964157	48967049	0	0	0	0
LOC100130122	NC_000007	49298072	49299457	0	0	0	0
VWC2	NC_000007	49813257	49952138	0.0232407	0.0238939	0	0.0647803
ZPBP	NC_000007	49977039	50132826	0.0373392	0	0.033218	0
LOC100270790	NC_000007	49981806	49984124	0	0	0	0
LOC100130988	NC_000007	50135682	50198852	0.0313022	0.0643638	0	0.0436252
IKZF1 (NC_000007 5034	NC_000007	50344378	50367357	0	0	0	0
IKZF1 (NC_000007 5044	NC_000007	50444231	50472799	0.0149636	0.0153842	0.0266242	0.0052136
FIGNL1	NC_000007	50511831	50518088	1.3313875	1.5351099	1.704707	2.6358899

DDC	NC_000007	50526134	50633154	0	0	0.0367458	0.0287828
GRB10	NC_000007	50657760	50861159	5.4143955	3.3656325	3.6163025	7.0061084
RPL39P23	NC_000007	50911382	50911529	0	0	0	0
COBL	NC_000007	51083909	51384515	0.3393366	0.2722917	0.4344174	0.9573879
LOC642663	NC_000007	51449981	51453802	0	0	0	0
LOC100133177	NC_000007	51453970	51458166	0	0	0	0
LOC100131871	NC_000007	51459797	51498371	0.187813	0.3861829	0.3898618	0.8725037
LOC100133258	NC_000007	52959636	52965501	0	0	0	0
LOC392027	NC_000007	53070113	53071122	0	0	0	0
DKFZp564N2472	NC_000007	53103349	53104618	0.0346049	0	0.0307855	0
LOC653175	NC_000007	53254728	53256733	0	0	0	0
RNU1P7	NC_000007	53433751	53433904	0	0	0	0
FLJ45974	NC_000007	53833838	53879618	0	0	0	0
HPVC1	NC_000007	54268917	54270114	0	0	0	0
SLC25A5P3	NC_000007	54487064	54488293	0	0	0	0
VSTM2A	NC_000007	54610019	54636948	0.0341744	0.0351348	0.0608049	0.0476281
RPL31P35	NC_000007	54724008	54724468	0	0	0	0
SEC61G	NC_000007	54819940	54826939	51.304374	64.3815	54.451331	78.993785
LOC643168	NC_000007	55001074	55007281	0	0	0	0
EGFR	NC_000007	55086725	55275031	7.0560622	9.4066191	9.1987272	11.465104
LOC100130121	NC_000007	55311922	55313392	0	0	0	0
CALM1P2	NC_000007	55327411	55327794	0	0	0	0
LOC100129276	NC_000007	55410003	55417136	0	0	0	0
LANCL2	NC_000007	55433141	55501435	1.3225863	2.6572366	0.7185401	1.6322012
ECOP	NC_000007	55538306	55640200	5.9562991	13.352432	5.7371428	7.6562196
CDC42P2	NC_000007	55705826	55706598	0	0	0	0
LOC442308	NC_000007	55713312	55714643	0	0	0	0
LOC643348	NC_000007	55725458	55746149	0	0	0	0
FKBP9L	NC_000007	55748767	55772260	0	0	0	0
LOC652489	NC_000007	55799717	55800706	0	0	0	0
LOC100288904	NC_000007	55804472	55808193	0	0	0	0
LOC730376	NC_000007	55808581	55812280	0	0	0	0
LOC100288969	NC_000007	55813582	55815074	0	0	0	0
LOC100128326	NC_000007	55814949	55815849	0.0975543	0.1504442	0.2603611	0
PSPHL	NC_000007	55832490	55841389	0	0	0	0
SEPT14	NC_000007	55861237	55930482	0	0.0119375	0.0103296	0.0080911
ZNF713	NC_000007	55980331	56008433	0.1276946	0.4594922	0.2461348	0.1927958
MRPS17	NC_000007	56019611	56023034	5.8497646	5.628649	8.6735214	10.765742
GBAS	NC_000007	56032296	56067871	5.3432501	7.0727818	7.7838619	6.6710728
PSPH	NC_000007	56078744	56119268	2.9049823	4.3732754	3.119683	3.2099731
CCT6A	NC_000007	56119378	56131682	19.63153	33.751866	24.949648	43.327765
SNORA15	NC_000007	56128163	56128295	0	0	0	0
SUMF2	NC_000007	56131917	56148365	30.832231	33.832557	22.594358	22.379719
PHKG1	NC_000007	56148675	56160689	0.3378271	0.4104712	0.355184	0.4280207
CHCHD2	NC_000007	56169266	56174187	113.99578	183.17296	203.97896	171.27386
LOC389493	NC_000007	56182374	56184090	0.0732471	0.1506113	0.2606504	0.1531244
LOC442309	NC_000007	56230806	56231396	0	0	0	0
LOC100130909	NC_000007	56242160	56244177	0	0	0	0
LOC100128459	NC_000007	56296324	56297475	0	0	0	0
LOC441228	NC_000007	56357876	56359677	0	0	0	0
LOC154937	NC_000007	56371015	56373219	0	0	0	0
LOC442676	NC_000007	56416079	56433841	0.086173	0	0.0191655	0.0150122
LOC731631	NC_000007	56434264	56442946	0	0	0	0
LOC100132050	NC_000007	56444056	56445542	0.029555	0.1215424	0.2103433	0.0823803
LOC650226	NC_000007	56489553	56499384	0	0	0	0
LOC728376	NC_000007	56515651	56535232	0	0	0	0
LOC100128867	NC_000007	56558309	56559203	0	0	0	0
DKFZp434L192	NC_000007	56563916	56564977	0	0	0	0
LOC100286929	NC_000007	56569968	56594856	0	0	0	0
LOC100130210	NC_000007	56635599	56637035	0	0	0	0
LOC346296	NC_000007	56665244	56669238	0	0	0	0
LOC100289196	NC_000007	56671103	56671246	0.9155882	0	0	0.2126728
LOC728406	NC_000007	56706692	56721759	0	0	0	0
LOC730382	NC_000007	56752751	56777582	0	0	0	0
LOC728416	NC_000007	56804089	56804283	0	0	0	0
LOC100287093	NC_000007	56871752	56872469	0	0	0	0

LOC100287136	NC_000007	56872524	56875189	0	0	0	0
LOC100289275	NC_000007	56875504	56875910	0	0	0	0
LOC730275	NC_000007	56876817	56880429	0.0121639	0	0	0.0423815
LOC401357	NC_000007	56876817	56888256	0	0	0	0
LOC100287197	NC_000007	56888805	56892129	0	0	0	0
LOC100287231	NC_000007	56892797	56897875	0	0	0	0
LOC100289306	NC_000007	56897122	56898621	0	0	0	0
LOC100287258	NC_000007	56898609	56900562	0	0	0	0.0531682
LOC100130849	NC_000007	56945081	56952060	0	0	0	0
LOC100287294	NC_000007	56959089	57012060	0	0	0	0.0662876
TNRC18C	NC_000007	57063241	57076859	0	0	0	0
LOC402509	NC_000007	57080872	57093741	0.0372127	0.0191293	0	0
PHKG1P4	NC_000007	57128219	57129008	0	0	0	0
LOC100128980	NC_000007	57159201	57159598	0	0	0	0
ZNF479	NC_000007	57187321	57207571	0.0212105	0	0.0566084	0.0295607
LOC100101483	NC_000007	57214508	57218254	0	0	0	0
LOC642006	NC_000007	57233377	57247863	0	0	0	0
LOC100289445	NC_000007	57234907	57235587	0	0	0	0
TRNAQ52P	NC_000007	57253980	57254051	0	0	0	0
LOC100287350	NC_000007	57257285	57257933	0	0	0	0
LOC100289472	NC_000007	57258168	57258848	0	0	0	0
LOC100287389	NC_000007	57261878	57263759	0	0	0	0
LOC100287433	NC_000007	57264561	57265484	0	0	0	0
LOC642007	NC_000007	57268636	57271418	0	0	0	0
LOC442317	NC_000007	57484207	57485943	0	0	0	0
ZNF716	NC_000007	57509889	57529655	0	0.0335936	0	0.0227694
LOC100287575	NC_000007	57545123	57545458	0	0	0	0
LOC100128575	NC_000007	57688119	57701660	0	0	0	0
LOC100287633	NC_000007	57710228	57714704	0	0	0	0.1166662
LOC100287663	NC_000007	57830100	57880261	0	0	0	0
LOC100129985	NC_000007	57881907	57886834	0	0	0	0
LOC100287729	NC_000007	61820462	61822404	0	0	0	0
LOC100128613	NC_000007	62505204	62514856	0	0	0	0.1311558
LOC442318	NC_000007	62573753	62575176	0	0	0	0
LOC100132729	NC_000007	62577834	62644762	0	0	0	0
PHKG1P1	NC_000007	62693341	62694624	0	0	0	0
LOC100132044	NC_000007	62723443	62723840	0	0	0	0
LOC643955	NC_000007	62751665	62764434	0	0	0	0
ARAF3P	NC_000007	62802994	62804211	0	0	0	0
LOC100287919	NC_000007	62805996	62806895	0	0	0	0
LOC100287704	NC_000007	62809239	62812151	0	0	0	0
LOC100287956	NC_000007	62809248	62812147	0	0	0	0
LOC340239	NC_000007	62812228	62814714	0	0	0	0
LOC100129000	NC_000007	62854156	62856642	0	0.0181678	0	0
LOC100287834	NC_000007	62856719	62859628	0	0	0	0
LOC100287992	NC_000007	62856723	62859619	0	0	0	0
LOC100133079	NC_000007	62857832	62859970	0	0	0	0
LOC100288022	NC_000007	62861972	62862871	0	0	0	0
LOC641719	NC_000007	62864967	62865758	0	0	0	0
LOC644017	NC_000007	62904563	62917271	0	0	0	0
LOC100130577	NC_000007	62940973	62941367	0	0	0	0
PHKG1P2	NC_000007	62969580	62971436	0	0	0	0
LOC100132308	NC_000007	63016784	63024085	0	0	0	0
TNRC18B	NC_000007	63026751	63045452	0	0	0	0
LOC402269	NC_000007	63092986	63145929	0	0	0.0423134	0
LOC100132653	NC_000007	63152954	63161735	0	0	0.0868835	0
LOC100288155	NC_000007	63204505	63206437	0	0	0	0.05518
LOC100287894	NC_000007	63206505	63207007	0	0	0	0
LOC100288187	NC_000007	63207212	63210453	0	0	0	0
LOC100288218	NC_000007	63210684	63212276	0	0	0	0
LOC100288258	NC_000007	63212507	63214099	0	0	0	0
LOC100288292	NC_000007	63214800	63227328	0	0	0	0
LOC100287930	NC_000007	63225421	63226032	0	0	0	0
LOC100132838	NC_000007	63227732	63231955	0	0	0	0
LOC100288360	NC_000007	63232010	63232763	0	0	0	0
LOC100288399	NC_000007	63303692	63303886	0	0	0	0

LOC731184	NC_000007	63328771	63337606	0	0	0	0
GABPAP	NC_000007	63353444	63358288	0	0	0	0
LOC441238	NC_000007	63369338	63370830	0	0	0	0
LOC644159	NC_000007	63391465	63392611	0	0	0	0
LOC100129587	NC_000007	63394900	63395417	0	0	0	0
LOC100129126	NC_000007	63411914	63441761	0	0	0	0.1068931
ZNF727	NC_000007	63475729	63539627	0.2032288	0.0348234	0.090399	0.1298164
LOC653435	NC_000007	63560220	63581051	0	0	0	0
RPL6P20	NC_000007	63601871	63602798	0	0	0	0
LOC402273	NC_000007	63609997	63635663	0	0	0	0
LOC100131065	NC_000007	63640366	63641195	0	0	0	0
LOC442320	NC_000007	63642088	63643472	0	0	0	0
ZNF735	NC_000007	63667581	63680668	0.0354707	0	0	0
ZNF679	NC_000007	63688852	63727309	0	0	0	0.0195436
LOC728927	NC_000007	63771206	63810017	1.8470444	1.5465722	1.9142222	2.7466854
LOC649395	NC_000007	63894112	63894973	0	0	0	0
LOC728947	NC_000007	63924569	63931061	0	0	0	0
LOC100127907	NC_000007	63961203	63962107	0	0	0	0
ZNF680	NC_000007	63980255	64023505	1.163125	0.9290561	0.9631119	1.0973083
LOC100133092	NC_000007	64027174	64027851	0	0	0	0
LOC100129293	NC_000007	64029806	64030815	0	0	0	0
LOC641746	NC_000007	64035389	64043589	0	0	0	0
LOC100128885	NC_000007	64075976	64076440	0	0	0	0
ZNF107	NC_000007	64126511	64171404	1.2207842	0.8695426	0.965372	1.3121771
LOC644387	NC_000007	64196311	64197121	0	0	0	0
ZNF138	NC_000007	64254806	64293705	1.4189564	1.3575282	1.3149405	1.5106442
LOC168474	NC_000007	64310071	64324845	0	0	0	0
EEF1DP4	NC_000007	64323329	64324760	0	0	0	0
LOC441239	NC_000007	64348515	64350478	0.0346868	0.0356617	0.0617168	0.0241712
ZNF273	NC_000007	64363620	64391955	0.5487178	0.4910104	0.7683915	0.616038
LOC100288747	NC_000007	64405377	64409591	0	0	0.0463791	0.0726569
ZNF117	NC_000007	64434830	64451414	0.4803835	0.1411099	0.3479952	0.5738578
ERV3 (NC_000007 6445	NC_000007	64450734	64453562	0.8699544	0.2715157	0.4284286	1.4181191
LOC441241	NC_000007	64498542	64499046	0	0	0	0
LOC643180	NC_000007	64525377	64535091	0	0	0	0
LOC100288236	NC_000007	64600906	64602101	0	0	0	0
INTS4L1	NC_000007	64609370	64673211	0	0	0	0
LOC100288808	NC_000007	64721415	64722254	0	0	0	0
LOC100130721	NC_000007	64800087	64800918	0	0	0	0
LOC100128166	NC_000007	64820560	64822012	0	0	0	0
ZNF92	NC_000007	64838768	64865998	1.764139	1.5090153	1.7075366	1.5932018
LOC100288905	NC_000007	64973376	64975020	0	0	0	0
LOC100288268	NC_000007	65019171	65020372	0	0	0	0
LOC402279	NC_000007	65081995	65084067	0	0	0	0
LOC441242	NC_000007	65112081	65113280	0	0	0	0
INTS4L2	NC_000007	65121421	65183702	0	0	0	0
LOC100131973	NC_000007	65186263	65215899	0.1798393	0.0231117	0.15999	0.2349735
CCT6P1	NC_000007	65216092	65228662	0	0	0	0
SNORA22	NC_000007	65220513	65220646	0	0	0	0
LOC100130576	NC_000007	65303246	65305766	0	0	0	0
VKORC1L1	NC_000007	65338257	65419800	3.0295901	2.8387478	2.9340232	1.9507994
GUSB	NC_000007	65425671	65447246	7.0082258	4.5284033	14.681179	4.5971421
LOC644667	NC_000007	65534487	65536329	0	0	0	0
LOC100289005	NC_000007	65535892	65540783	0	0	0	0
ASL	NC_000007	65540776	65558330	8.2752923	10.341647	10.258729	13.513101
CRCP	NC_000007	65579805	65619555	6.1304883	5.0293334	5.0772436	10.663532
TPST1	NC_000007	65670259	65825438	8.9996379	11.036999	6.3288112	6.3011695
LOC100288377	NC_000007	65823407	65825438	0.0648842	0	0.0577228	0.0301426
NCRNA00174	NC_000007	65841031	65865395	0	0	0	0
TCEB1P	NC_000007	65887829	65888973	0	0	0	0
LOC729126	NC_000007	65892404	65915392	0	0	0	0
LOC100289070	NC_000007	65938945	65940707	0.0647249	0	0	0.0451029
LOC100289098	NC_000007	65958659	65959559	0.0487772	0.4011845	0.5207223	0.2039393
LOC346329	NC_000007	65969847	65971210	0	0	0	0
LOC493754	NC_000007	66018553	66043498	0	0	0	0
LOC643353	NC_000007	66021139	66022759	0	0	0	0

RPL35P5	NC_000007	66071690	66072144	0	0	0	0
KCTD7	NC_000007	66093890	66108034	1.5686082	1.491509	1.2986813	3.0075185
LOC100289135	NC_000007	66147056	66148154	0.391521	0.3522091	0.3483079	0.6820686
RABGEF1	NC_000007	66205643	66276451	8.6050869	9.0129621	7.2243271	7.3869457
LOC729156	NC_000007	66274980	66309813	0	0	0	0
LOC644791	NC_000007	66367244	66367988	0	0	0	0
LOC644794	NC_000007	66369107	66371286	0.1007987	0.1450843	0.2331506	0.0280962
RPL31P38	NC_000007	66379557	66379970	0	0	0	0
C7orf42	NC_000007	66386203	66423538	5.1228	4.9362707	5.2677466	8.7293188
SBDS	NC_000007	66452690	66460588	39.211133	46.084255	32.301166	39.15405
TYW1	NC_000007	66461817	66704499	1.253402	0.9359516	1.5023982	1.5721568
LOC442572	NC_000007	66743159	66752518	0	0.0998528	0.0648026	0.0845991
PMS2L4	NC_000007	66757423	66767406	0	0	0	0
STAG3L4	NC_000007	66767616	66786513	2.5524968	2.0951554	2.2524593	2.1085982
LOC100289197	NC_000007	67234613	67235718	0	0	0	0
LOC100289234	NC_000007	68795699	68802025	0	0	0	0
AUTS2	NC_000007	69063905	70257885	0.6323487	1.1783441	1.2423087	1.8819245
WBSCR17	NC_000007	70597789	71178584	0.0270451	0.0417078	0.0360901	0.009423
CALN1	NC_000007	71244476	71877360	0.0308223	0.0226347	0	0.0245465
RPS28P6	NC_000007	71407218	71407601	0	0	0	0
TYW1B	NC_000007	72039492	72298813	0.6786324	0.8232924	0.7486254	0.8890484
SBDSP	NC_000007	72299952	72307978	0	0	0	0
SPDYE7P	NC_000007	72333318	72339655	0	0	0	0
POM121	NC_000007	72349936	72418838	2.6162279	2.9685277	3.4618655	5.459062
LOC100131972	NC_000007	72349962	72414039	0.4836119	0.4972039	0.3495656	0.7582501
NSUN5C	NC_000007	72418832	72425286	1.7067274	1.3676301	1.5406808	2.9208193
TRIM74	NC_000007	72430016	72439997	0.0325542	0	0	0.0226851
LOC100101148	NC_000007	72440213	72442259	0	0	0	0
STAG3L3	NC_000007	72469013	72476455	0.138202	0.0710431	0.1229483	0.3852186
LOC100101440	NC_000007	72476588	72483807	0	0	0	0
SPDYE8P	NC_000007	72490260	72500309	0	0	0	0
LOC729299	NC_000007	72507939	72511870	0	0	0	0
LOC728524	NC_000007	72518322	72525621	0	0	0	0
LOC100093631	NC_000007	72569012	72621336	0	0	0	0
LOC100289276	NC_000007	72601874	72610045	0	0	0	0
NCF1B	NC_000007	72634674	72649979	0	0	0	0
GTF2IRD2P	NC_000007	72656902	72685658	0	0	0	0
LOC100288540	NC_000007	72705857	72717222	0.0481255	0.0494781	0.0941904	0.0335358
NSUN5	NC_000007	72717226	72722823	5.9186042	8.0865746	6.2583817	5.0649533
TRIM50	NC_000007	72726535	72742085	0.0214696	0.022073	0.0381999	0.0299217
FKBP6	NC_000007	72742155	72772641	0.0256258	0	0.0911897	0.0357141
FZD9	NC_000007	72848109	72850450	0.075061	0.0771706	0.0333882	0.0392291
BAZ1B	NC_000007	72854740	72936608	0.9444256	0.9857927	0.96846	2.8434516
BCL7B	NC_000007	72950686	72972024	16.029109	14.988855	17.942194	21.126941
TBL2	NC_000007	72983274	72993013	8.8445636	7.3250303	5.6698541	6.908473
LOC100289307	NC_000007	72988739	72993008	0	0.0689823	0	0
MLXIPL	NC_000007	73007524	73038870	0.0670351	0.1516222	0.0357818	3.8958433
LOC100289339	NC_000007	73011725	73020280	0.1413126	0	0.1885735	0
VPS37D	NC_000007	73082174	73086440	0.2190566	0.3659715	0.6089963	0.3434566
DNAJC30	NC_000007	73095248	73097781	2.2026146	1.5334541	0.5091632	1.0272749
WBSCR22	NC_000007	73097898	73112542	14.685141	13.665224	17.832304	17.652877
STX1A	NC_000007	73113540	73133988	2.269923	1.3613364	1.3088616	1.7428793
WBSCR26	NC_000007	73149399	73150330	0	0	0	0
ABHD11	NC_000007	73150424	73153190	1.4003685	1.5357078	0.8305378	0.7156105
CLDN3	NC_000007	73183327	73184600	0.137985	0.0709316	0.0306888	0.0240384
LOC100128031	NC_000007	73245192	73247022	0.1265608	0.1301178	0.28148	0.1102407
CLDN4	NC_000007	73245193	73247015	0.0482153	0.0743556	0.0857873	0.1343933
WBSCR27	NC_000007	73248920	73256855	2.3130648	2.1839453	1.7638	2.8947253
WBSCR28	NC_000007	73275489	73280223	0	0.2116319	0.0457817	0.1075815
ELN	NC_000007	73442427	73484237	30.479006	88.320079	0.1120916	0.3248625
LIMK1	NC_000007	73498156	73536854	2.8621748	3.078221	2.4876002	2.6194749
EIF4H	NC_000007	73588706	73611429	51.198136	63.320651	54.868657	94.039787
LAT2	NC_000007	73624087	73644164	0.4613461	0.1443559	1.7844621	0.964453
RFC2	NC_000007	73645832	73668738	5.2924671	6.9615513	6.7624479	8.6234161
CLIP2	NC_000007	73703805	73820273	0.8374101	0.633526	0.9558276	0.979908
GTF2IRD1	NC_000007	73868120	74016917	3.7157397	4.8476653	4.8216531	5.9285479

GTF2I	NC_000007	74072030	74175022	11.149596	13.637825	8.5032241	16.235446
LOC100289374	NC_000007	74155555	74159530	0	0	0	0
NCF1	NC_000007	74188309	74203659	0.0314365	0.0646401	0.0559336	0.0438124
LOC100288630	NC_000007	74197786	74200583	0	0	0	0
GTF2IRD2	NC_000007	74210484	74267841	0.8787171	0.7125515	0.7376899	0.2414803
STAG3L2	NC_000007	74299267	74306731	1.0794303	0.4756148	0.2057767	1.1820128
PMS2L5	NC_000007	74306901	74321854	0.5963125	0.3678432	0.2652481	0.2077672
LOC100132585	NC_000007	74320394	74330410	0	0.04089	0.0707648	0
LOC100101268	NC_000007	74320406	74328423	0	0	0	0
GATSL1	NC_000007	74379083	74438803	0.2219608	0.9584358	0.15797	0.4021449
WBSCR16	NC_000007	74456283	74489699	5.414984	5.5291716	4.0610171	7.0702515
GTF2IRD2B	NC_000007	74508347	74565623	0.8949012	0.4411211	0.3707998	0.5894328
NCF1C	NC_000007	74572445	74587759	0	0	0	0
GTF2IP1	NC_000007	74601104	74653445	0	0	0	0
LOC100289407	NC_000007	74612392	74620561	0	0	0	0
LOC643862	NC_000007	74697002	74704300	0	0	0	0
LOC441259	NC_000007	74700807	74715111	0	0	0	0
LOC729425	NC_000007	74710752	74714649	0	0	0	0
LOC653375	NC_000007	74765876	74790133	0.0250989	0.0258043	0.0446574	0.0349799
GATSL2	NC_000007	74807605	74867341	1.0654117	2.3732696	1.1057897	1.9488559
LOC641776	NC_000007	74911266	74918564	0	0	0	0
LOC100288713	NC_000007	74915995	74929250	0.2012896	0.5058701	0.3183517	0.2337777
LOC643909	NC_000007	74939286	74946587	0	0	0	0
LOC729453	NC_000007	74953039	74956948	0	0	0	0
LOC441258	NC_000007	74967309	74974606	0	0	0	0
PMS2L2	NC_000007	74977964	74988215	0	0	0	0
LOC100101441	NC_000007	74981056	74984931	0	0	0	0
STAG3L1	NC_000007	74988447	74996990	1.7328985	1.1877346	1.7129273	2.2138466
LOC541473	NC_000007	75021229	75024708	0	0	0	0
TRIM73	NC_000007	75024903	75034888	0	0	0	0.0453702
NSUN5B	NC_000007	75039646	75046065	0.8851849	0.3935408	1.128019	1.6837848
POM121C	NC_000007	75046065	75115568	3.3656415	3.4679398	4.7613273	9.5797419
SPDYE5	NC_000007	75124299	75133628	0.0164785	0.0169417	0.117278	0.0459316
PMS2L3	NC_000007	75137069	75157394	1.5211578	1.4619159	1.4415205	2.050876
HIP1	NC_000007	75163409	75368279	1.6938191	3.5452649	3.570036	4.6916687
CCL26	NC_000007	75398842	75419064	0.9383964	1.2863602	1.113098	1.5802873
CCL24	NC_000007	75441114	75443033	0	0	0	0
LOC100129130	NC_000007	75459166	75465462	0	0	0	0
RHBDD2	NC_000007	75508317	75518244	10.29671	10.802634	8.4315836	9.6212344
LOC100288746	NC_000007	75510861	75513128	0.1085142	0.055782	0.0482686	0
POR	NC_000007	75544420	75616173	11.325595	14.265588	8.5423253	7.1445794
LOC100131964	NC_000007	75552068	75552773	0	0	0	0
SNORA14A	NC_000007	75573101	75573234	0	0	0	0
TMEM120A	NC_000007	75616155	75623992	2.8681375	3.9316624	4.624729	3.9556267
STYXL1	NC_000007	75625655	75677321	6.3315249	5.5841069	6.626706	6.9208764
MDH2	NC_000007	75677393	75695931	35.939289	35.288721	29.996754	43.099944
LOC645324	NC_000007	75739959	75770855	0	0	0	0
LOC100129416	NC_000007	75779120	75779931	0	0	0	0
FLJ37078	NC_000007	75831216	75916605	0.0851003	0.3624671	0.0648922	0.1016593
HSPB1	NC_000007	75931875	75933614	44.487173	55.358482	56.837487	58.893997
LOC100289506	NC_000007	75947795	75988329	14.765676	15.180667	18.479978	19.363349
YWHAG	NC_000007	75956108	75988342	4.7853959	6.1619211	4.4554739	6.3178625
SRCRB4D	NC_000007	76018646	76039012	0.3758936	0.1449218	0.1114685	0.1855392
ZP3	NC_000007	76026841	76071388	0.1954496	0.1722366	0.6955094	0.1556538
DTX2	NC_000007	76090972	76135312	2.8062014	1.1059435	3.7863198	5.1168207
FDPSL2A	NC_000007	76099337	76104317	0	0	0	0
UPK3B	NC_000007	76139745	76157200	0.1040195	0.0534715	0.069404	0.235576
LOC100288807	NC_000007	76163011	76693255	0.4263507	0.5435334	0.3717076	0.3862276
LOC100133091	NC_000007	76178893	76179075	0	0	0	0
POMZP3	NC_000007	76239303	76256620	0.169903	0.6404864	0.277109	0.2565228
FDPSL2	NC_000007	76597453	76598627	0	0	0	0
FDPSL2B	NC_000007	76597453	76598627	0	0	0	0
LOC100288878	NC_000007	76606134	76607744	0	0	0	0
PMS2L11	NC_000007	76610139	76653078	0	0	0	0
LOC100132581	NC_000007	76652920	76655095	0	0	0	0
LOC100132832	NC_000007	76668797	76682355	0.0188457	0	0	0

LOC100288933	NC_000007	76684290	76685363	0	0	0	0
FAM185B	NC_000007	76713047	76751683	0	0	0	0
RPL7AP43	NC_000007	76744671	76745525	0	0	0	0
CCDC146	NC_000007	76751934	76924521	0.1450437	0.1491201	0.2815306	0.1745793
FGL2	NC_000007	76822688	76829150	0.1750515	0.3917024	0	0.0071755
PION	NC_000007	76940068	77045717	0.3649961	0.0972882	1.1545267	1.0362155
PTPN12	NC_000007	77166773	77269388	6.21167	7.4414884	6.3917464	5.9633521
RSBN1L	NC_000007	77325743	77409120	2.3884909	2.245138	3.2176568	2.5298812
TMEM60	NC_000007	77423045	77427747	3.8763045	3.0018755	3.7171798	3.7886447
PHTF2	NC_000007	77428109	77586821	0.8532343	1.377501	0.9666166	2.1135021
MAGI2	NC_000007	77646374	79082890	0.5749042	0.3415025	0.5512302	0.8012323
RPL13AP17	NC_000007	77976559	77988775	0	0	0	0
LOC100124402	NC_000007	78115833	78116717	0	0	0	0
LOC100130215	NC_000007	79173569	79174942	0	0	0	0
RPL10P11	NC_000007	79725837	79726578	0	0	0	0
GNAI1	NC_000007	79764140	79848725	1.1523497	1.1438836	1.6614698	2.3720897
GNAT3	NC_000007	80087987	80141242	0	0	0	0.0287558
CD36	NC_000007	80231504	80308593	0.3238035	0.6158725	0.0864194	0.0282049
SEMA3C	NC_000007	80371854	80548667	8.2477258	3.1176023	8.4860004	5.1080924
LOC100128317	NC_000007	81102005	81259953	0.0581326	0	0	0.0202545
HGF	NC_000007	81331444	81399452	0.564228	0.021094	0.0091264	0.021446
CACNA2D1	NC_000007	81579418	82073031	1.0118902	1.0285076	0.8490575	1.3381357
LOC442707	NC_000007	82218082	82220079	0	0	0	0
PCLO	NC_000007	82383321	82792197	0.2758711	0.255262	0.383911	0.3158195
SEMA3E	NC_000007	82993222	83278324	0.210441	0.1116674	0.0603917	0.1371828
LOC100130572	NC_000007	83290318	83293944	0	0	0	0
SEMA3A	NC_000007	83587659	83824217	2.213332	1.4451662	2.5079371	1.0606956
RPL7P30	NC_000007	84157433	84158146	0	0	0	0
HMGN2L11	NC_000007	84505800	84507140	0	0	0	0
LOC402562	NC_000007	84608212	84614351	0	0	0	0
SEMA3D	NC_000007	84624869	84751247	0.1333679	1.3711622	0.0187339	0.0342396
LOC100289643	NC_000007	84657138	84659027	0	0	0	0
LOC729630	NC_000007	85010421	85010787	0	0	0	0
GRM3	NC_000007	86273230	86494192	0.0103165	0.0212129	0.0091778	0.0503226
KIAA1324L	NC_000007	86509238	86689014	0.2048869	0.5500181	0.1923993	0.0872504
DMTF1	NC_000007	86781677	86825648	4.3210741	3.5668296	4.4540361	6.5071533
C7orf23	NC_000007	86825478	86849031	5.4777278	4.505344	5.885252	7.2818503
LOC100289677	NC_000007	86830475	86849858	0.2197412	0.451834	0.1466159	0.1914055
TP53TG1	NC_000007	86954663	86974808	0	0	0	0
CROT	NC_000007	86974951	87029112	2.6607213	1.8644958	1.318954	2.4629044
ABCB4	NC_000007	87031361	87105019	0.0661207	0.2039371	0.3627407	0.1382266
ABCB1	NC_000007	87132948	87342564	0.0360823	0.0278223	0.0481497	0.0188577
RUNDC3B	NC_000007	87257729	87461613	0.0642988	0.0991589	0.085803	0.3883184
SLC25A40	NC_000007	87465417	87505668	3.6551005	4.1753639	6.4517435	4.6695359
DBF4	NC_000007	87505544	87538856	1.8672655	2.659888	2.6618767	2.3358622
ADAM22	NC_000007	87563702	87826454	0.048508	0.0997426	0.1726162	0.1267586
SRI	NC_000007	87834432	87856308	8.9784454	12.077664	16.273545	11.065887
STEAP4	NC_000007	87905744	87936209	0.4144198	0.0101445	0.1667835	0.0412549
LOC100133009	NC_000007	88084624	88085374	0	0	0	0
LOC100128464	NC_000007	88142819	88143064	0	0	0	0
LOC645680	NC_000007	88193833	88195721	0	0	0	0
LOC442709	NC_000007	88268266	88269936	0	0	0	0
ZNF804B	NC_000007	88388753	88966346	0	0.0098482	0.0085217	0.006675
C7orf62	NC_000007	88423420	88425031	0	0	0	0
DPY19L2P4	NC_000007	89748714	89754914	0	0	0	0
STEAP1	NC_000007	89783689	89794141	7.497642	6.4351075	13.34022	5.9943593
STEAP2	NC_000007	89841000	89866992	1.9772085	0.9942248	9.8360379	2.897238
C7orf63	NC_000007	89874525	89939688	0.1446617	0.0743637	0.1673036	0.1411285
GTPBP10	NC_000007	89975990	90016650	1.3910929	2.0393446	1.8334146	2.3067391
CLDN12	NC_000007	90032796	90045268	4.7508178	4.0377211	6.8637698	6.1620539
PFTK1	NC_000007	90338712	90839904	4.1259697	4.6433175	2.8496308	3.4007032
FZD1	NC_000007	90893783	90898132	6.758935	9.2132592	3.5322628	3.0202466
LOC100132321	NC_000007	90949457	90950289	0	0	0	0
MTERF	NC_000007	91502021	91510016	1.4019547	1.2115753	1.3014446	1.769815
AKAP9	NC_000007	91570189	91739987	0.2891563	0.3009085	0.2980236	0.9141021
CYP51A1	NC_000007	91741463	91763840	23.412571	26.16919	23.948478	21.708533

LOC100129737	NC_000007	91774202	91794516	0	0.0230999	0.0399771	0
KRIT1	NC_000007	91828283	91875414	1.0224549	1.5678783	2.3202617	2.3125647
ANKIB1	NC_000007	91875548	92030698	8.3112098	7.8537832	7.8889513	5.2275323
LOC645862	NC_000007	92071391	92071808	0	0	0	0
LOC100286960	NC_000007	92076718	92078183	0.1883496	0.1290954	0	0.0437498
GATAD1	NC_000007	92076765	92088742	2.2335258	2.4219674	2.1451762	1.8971164
ERVWE1	NC_000007	92097695	92100738	0.0144377	0.0148434	0	0.0804859
PEX1	NC_000007	92116337	92157845	1.1934823	1.2682698	1.436492	1.6423657
C7orf64	NC_000007	92158087	92166823	0.7166864	0.8684055	0.8425218	0.8204685
MGC16142	NC_000007	92167789	92169079	0	0	0	0
LOC442710	NC_000007	92178032	92187191	0	0	0	0
FAM133B	NC_000007	92190072	92219706	0.8013251	0.9520004	1.0138753	0.7073007
CDK6	NC_000007	92234235	92465941	1.9504316	3.5956184	3.0481608	3.7649697
LOC100128994	NC_000007	92464968	92546501	0.2788593	0.172018	0.0496162	0.0777281
RN7SLP4	NC_000007	92600256	92600640	0	0	0	0
SAMD9	NC_000007	92728831	92747322	11.167068	2.7958717	7.0062197	2.1190122
SAMD9L	NC_000007	92759368	92777680	11.133388	5.1308784	6.6487238	1.5069862
HEPACAM2	NC_000007	92817899	92855782	0	0.082189	0.0177797	0.0278535
CCDC132	NC_000007	92861653	92988338	2.9879151	2.9267621	3.0976658	3.5821927
CALCR	NC_000007	93053799	93203750	0	0.0130212	0.0112673	0.0264768
RPS27P17	NC_000007	93299081	93299423	0	0	0	0
LOC100128313	NC_000007	93407153	93407889	0	0	0	0
LOC402676	NC_000007	93474101	93474624	0	0	0	0
TFPI2	NC_000007	93515745	93520065	1.4649411	9.4614816	1.6708361	0.6020275
GNGT1	NC_000007	93535820	93540485	0	0	0	0
GNG11	NC_000007	93551016	93555826	17.367383	24.296735	12.70466	14.156783
BET1	NC_000007	93621000	93633690	2.8140652	4.0740343	6.4374953	4.5622164
COL1A2	NC_000007	94023873	94060544	17.421726	29.092399	13.576848	9.6725036
CASD1	NC_000007	94139170	94186331	1.8453276	1.7807987	2.0042286	2.926798
SGCE	NC_000007	94214536	94285521	20.371584	12.581456	23.588145	15.532397
PEG10	NC_000007	94285637	94299007	11.674372	18.672802	13.540589	14.363799
RPS3AP25	NC_000007	94324293	94325151	0	0	0	0
LOC645973	NC_000007	94367966	94368773	0	0	0	0
LOC100129871	NC_000007	94409076	94412841	0	0	0	0
ARF1P1	NC_000007	94463140	94465616	0	0	0	0
PPP1R9A	NC_000007	94536949	94925727	0.0725593	0.0139872	0.076654	0.1264054
LOC100233146	NC_000007	94647312	94647797	0	0	0	0
PON1	NC_000007	94927669	94953884	0.0250846	0.0257896	0.0669479	0.0699198
PON3	NC_000007	94989184	95025687	0	0.0758747	0.0328275	0.0514272
PON2	NC_000007	95034174	95064384	13.099443	12.675392	17.539537	11.572279
ASB4	NC_000007	95115284	95167071	0	0	0	0
PDK4	NC_000007	95212809	95225925	0.0712674	0.0122117	0.1268029	0.1903709
LOC100287137	NC_000007	95401629	95434112	0	0	0.0728074	0
DYNC1I1	NC_000007	95401818	95727736	0.2741354	0.3263411	0.2438784	0.1709202
SLC25A13	NC_000007	95749532	95951459	2.2180769	3.5693471	2.7944342	4.0705167
CYCSP18	NC_000007	95757139	95757374	0	0	0	0
RPL21P74	NC_000007	95912625	95913165	0	0	0	0
FLJ42280	NC_000007	96113171	96125607	0	0	0	0
SHFM1	NC_000007	96318079	96339203	6.0892128	5.2623239	9.8921552	9.7163268
LOC402679	NC_000007	96483795	96631702	0	0	0	0
FLJ34048	NC_000007	96597827	96643377	0	0	0	0
DLX6	NC_000007	96635290	96640352	0.0981535	0	0.0654901	0
DLX5	NC_000007	96649702	96654143	0.0308625	0.0951898	0.0274562	0
ACN9	NC_000007	96745905	96811075	0.9614343	1.3838378	1.4635452	0.8486233
RPL7AP40	NC_000007	96830134	96830782	0	0	0	0
TAC1	NC_000007	97361271	97369784	0	0.1901658	0.0329104	0.051557
ASNS	NC_000007	97481440	97501854	9.5060986	7.8329874	11.752588	8.195047
RPS3AP29	NC_000007	97527608	97528456	0	0	0	0
LOC441268	NC_000007	97552966	97557991	0	0	0	0
OR7E7P	NC_000007	97576295	97577301	0	0	0	0
OR7E38P	NC_000007	97595404	97596389	0	0	0	0
MGC72080	NC_000007	97595908	97601638	0	0	0	0
OCM2	NC_000007	97614012	97619416	0	0	0	0
LMTK2	NC_000007	97736197	97835601	0.407945	0.4678039	1.2004251	1.8641706
LOC100289123	NC_000007	97823231	97832913	0	0.0663486	0	0
BHLHA15	NC_000007	97841566	97842271	0.1244992	0	0.3322739	0.043378

TECPR1	NC_000007	97846036	97881468	1.4082748	0.6929018	0.8501416	1.0163899
BRI3	NC_000007	97910987	97920839	116.80239	71.727929	67.409596	66.490796
BAIAP2L1	NC_000007	97922555	98030380	2.4896782	2.1144942	5.2386886	4.103432
FLJ30064	NC_000007	97937197	97938183	0	0	0	0
RPS3AP26	NC_000007	98015062	98015919	0	0	0	0
LOC442713	NC_000007	98083225	98083960	0	0	0	0
RPS26P33	NC_000007	98107863	98108108	0	0	0	0
NPTX2	NC_000007	98246597	98259181	0.0971948	0.1665441	0.0144112	0.0225764
TMEM130	NC_000007	98444111	98467673	0.6739207	1.0761463	1.007735	0.1798525
TRRAP	NC_000007	98476113	98610864	1.8433958	2.6059064	2.9040534	4.3134659
SMURF1	NC_000007	98625064	98741723	2.3900729	2.6698925	2.1535351	2.4235131
KPNA7	NC_000007	98771197	98805089	0	0	0.0928132	0.054525
MYH16	NC_000007	98870924	98895595	0	0	0	0
ARPC1A	NC_000007	98923510	98963885	36.775262	48.303411	54.8544	55.540034
ARPC1B	NC_000007	98972329	98992424	71.040004	58.946504	49.540731	39.610862
PDAP1	NC_000007	98992589	99006292	2.7479171	2.3321689	2.0344516	3.726905
BUD31	NC_000007	99006601	99017239	12.935865	14.512406	14.619416	15.209671
PTCD1	NC_000007	99016745	99036419	1.2988922	1.173976	0.8634734	0.7061924
CPSF4	NC_000007	99036563	99054996	5.3671718	5.5180168	4.7105501	5.8868194
LOC100131859	NC_000007	99039958	99041104	0	0	0	0
ATP5J2	NC_000007	99055784	99063808	15.004548	19.259996	37.280911	40.709434
ZNF789	NC_000007	99070515	99085217	0.9451233	0.7159792	0.8186813	0.7105942
ZNF394	NC_000007	99090854	99097877	2.6225518	2.4434847	2.223731	1.6990026
ZKSCAN5	NC_000007	99102273	99131445	0.9726573	1.2857066	1.0948731	1.4800641
C7orf38	NC_000007	99143923	99149534	0.7179181	0.7580438	1.4327143	1.3250499
ZNF655	NC_000007	99156045	99174076	5.3023556	4.9025326	3.8057873	4.9466317
LOC100289187	NC_000007	99195903	99208667	0.1994625	0.3417806	0.0295746	0.0926623
ZNF498	NC_000007	99214571	99230040	0.8973142	1.1402097	0.7713674	1.5315952
LOC442603	NC_000007	99232688	99236745	0	0	0	0
CYP3A5	NC_000007	99245817	99277621	0.0514918	0	0.0229043	0.0538223
CYP3A	NC_000007	99245817	99464173	0	0	0	0
CYP3A5P1	NC_000007	99282647	99297743	0	0	0	0
CYP3A7	NC_000007	99302660	99332821	0	0.0434456	0.0187969	0.058894
CYP3A5P2	NC_000007	99345872	99350114	0	0	0	0
CYP3A4	NC_000007	99354604	99381808	0.0158773	0	0	0.0221278
CYP3A43	NC_000007	99425636	99464173	0.0202807	0	0.0180423	0.0141324
OR2AE1	NC_000007	99473685	99474656	0.0452142	0.046485	0	0
TRIM4	NC_000007	99488036	99517154	0.3117817	0.4006805	0.5547393	0.3711558
GJC3	NC_000007	99521168	99527243	0.2092773	0.6992669	0.1396342	0.0364582
AZGP1	NC_000007	99564350	99573687	0	0.0371269	0.0321262	0.0503285
AZGP1P1	NC_000007	99578352	99585434	0	0	0	0
LOC100128334	NC_000007	99587218	99598817	0.1970773	0	0	0
ZKSCAN1	NC_000007	99613219	99635403	1.8238436	1.8010857	2.4053471	3.0045157
ZSCAN21	NC_000007	99647417	99662663	0.7512518	1.0676822	0.6683345	0.4465166
ZNF3	NC_000007	99661653	99679371	1.4463675	2.145206	2.0988442	1.7596706
COPS6	NC_000007	99686583	99689823	33.93198	40.8425	32.270571	35.285186
MCM7	NC_000007	99690404	99699427	5.0065989	7.3270381	8.6125995	18.574287
AP4M1	NC_000007	99699130	99704803	2.3056353	2.1769305	2.8255735	3.3280783
TAF6	NC_000007	99704693	99716979	3.4249532	2.2542338	2.306557	3.8141691
CNPY4	NC_000007	99717265	99723131	4.0659589	4.3646552	5.9577243	4.9791469
MBLAC1	NC_000007	99724317	99726121	0.6729756	0.5414789	0.234273	0.4485668
LOC100289264	NC_000007	99737257	99738305	0	0	0	0
C7orf59	NC_000007	99746530	99751833	16.372871	11.738825	13.926911	20.066301
C7orf43	NC_000007	99752043	99756302	1.5558443	1.4737625	0.6842852	1.2303551
GAL3ST4	NC_000007	99756865	99766373	0.5017712	0.480296	0.1385347	0.012057
LOC100287198	NC_000007	99761789	99773497	0.0830779	0.0569419	0.0246361	0.0192973
GPC2	NC_000007	99767229	99774990	0.1214999	0.0713798	0.0154414	0.0120951
STAG3	NC_000007	99775538	99812010	0.1463199	0.268629	0.0557873	0.0873956
GATS	NC_000007	99806559	99869829	0.6102408	0.6385951	0.4265542	0.9795709
PVRIG	NC_000007	99816871	99819111	0.0280103	0.0287976	0	0
SPDYE3	NC_000007	99912064	99918757	0.2078477	0.4036352	0.3903593	0.3218589
PMS2L1	NC_000007	99918263	99933930	0	0	0	0
PILRB	NC_000007	99933737	99965452	1.4576346	1.0704297	1.9987534	2.894471
PILRA	NC_000007	99971068	99997722	0.1335813	0.1030017	0.3862222	0.0232712
ZCWPW1	NC_000007	99998495	100026302	0.8650486	0.5413501	0.3680558	0.4586524
LOC100289298	NC_000007	99998495	100001824	0	0	0	0

MEPCE	NC_000007	100027529	100031741	8.0633086	9.9183077	8.7389535	11.193075
LOC100287232	NC_000007	100032908	100034400	2.0500633	1.1855703	2.2512447	2.0982059
C7orf47	NC_000007	100032912	100034094	2.315552	3.3037326	7.1468668	5.0712164
C7orf61	NC_000007	100054238	100061894	0.0875463	0.1350102	0.3504762	0.2440229
TSC22D4	NC_000007	100064142	100076902	12.135097	9.6889292	9.302694	11.900393
C7orf51	NC_000007	100081550	100092422	0.1840892	0.2523508	0.1746889	0.0256561
AGFG2	NC_000007	100136834	100165843	3.9737021	3.0710814	3.0800202	4.2076584
IRS3L	NC_000007	100162878	100167751	0	0	0	0
LOC100289367	NC_000007	100169851	100170374	0	0	0	0.1954779
LOC100131225	NC_000007	100171072	100172785	0.0512815	0.0263614	0.159675	0.2322774
LRCH4	NC_000007	100171634	100183776	2.5262339	1.5526942	1.7954833	3.1093675
FBXO24	NC_000007	100183956	100198740	0.175481	0.2004587	0.1040752	0.1494559
LOC100129845	NC_000007	100194043	100198464	0	0	0	0
PCOLCE	NC_000007	100199882	100205798	82.742041	70.257401	40.352549	21.380702
MOSPD3	NC_000007	100209725	100213000	2.2932244	1.5502525	1.8444884	4.0716421
TFR2	NC_000007	100218039	100239173	0.6847889	0.7979063	0.2707588	0.5196049
ACTL6B	NC_000007	100240720	100254084	0	0.0293971	0.0254376	0
GNB2	NC_000007	100271363	100276792	38.533936	36.114137	22.156854	29.170933
LOC100287259	NC_000007	100275222	100276791	0.0469031	0.0964427	0.2086316	0.1307359
GIGYF1	NC_000007	100277130	100286870	2.6039796	2.4772698	2.2856848	3.6533075
POP7	NC_000007	100303676	100305123	7.8276894	12.313931	7.6768825	6.2104099
EPO	NC_000007	100318423	100321323	0	0	0.0293967	0.0690787
ZAN	NC_000007	100331249	100395419	0.0201297	0.0103477	0	0
EPHB4	NC_000007	100400187	100425143	6.2422448	7.7780667	6.2182292	6.5247672
SLC12A9	NC_000007	100450358	100464634	11.434012	10.630582	8.8545185	4.648585
TRIP6	NC_000007	100464950	100471076	24.758507	17.227461	18.767724	26.866688
SRRT	NC_000007	100472701	100486285	1.2981721	1.1996921	0.8564352	3.8217571
UFSP1	NC_000007	100486344	100487339	0.485372	0.8619324	0.471055	0.1229915
ACHE	NC_000007	100487615	100493541	0	0.0152801	0	0.0310702
LOC100127899	NC_000007	100490036	100491253	0	0	0.0448367	0
LOC100287322	NC_000007	100491183	100493752	0	0	0.0509083	0
RPS29P15	NC_000007	100525960	100526215	0	0	0	0
MUC3A	NC_000007	100547257	100611619	0.0076233	0	0.0135638	0.0212488
MUC12	NC_000007	100612904	100662230	0.0209902	0.0107901	0.0046684	0.0292536
MUC17	NC_000007	100663364	100702140	0.0030626	0.0062973	0.0054491	0.0106707
TRIM56	NC_000007	100728786	100733909	2.2674165	1.2865869	2.4690879	2.4088923
SERPINE1	NC_000007	100770379	100782542	141.73858	463.32904	76.053136	179.31368
AP1S1	NC_000007	100797686	100804557	12.401737	8.5001928	13.595221	15.843711
VGF	NC_000007	100805790	100808852	0.1377148	0.2123779	0.168458	0.1199564
C7orf52	NC_000007	100813778	100823557	0.0149943	0.0462471	0.0666966	0.0313458
MOGAT3	NC_000007	100839010	100844302	0	0	0	0
RPSAP46	NC_000007	100846881	100847852	0	0	0	0
PLOD3	NC_000007	100849258	100861011	18.999454	17.788024	16.705443	9.2275834
ZNHIT1	NC_000007	100860985	100867471	9.5362858	13.452417	9.3386952	10.276978
CLDN15	NC_000007	100875374	100881116	0.593895	0.969755	0.9323744	1.2415491
FIS1	NC_000007	100882893	100888371	20.576574	24.823357	22.537974	24.201528
AZGP1P2	NC_000007	100930760	100933089	0	0	0	0
RABL5	NC_000007	100956648	100965093	2.6093704	3.3993206	2.2895687	3.0014622
EMID2	NC_000007	101006122	101202304	0.014625	0.0601443	0.0390325	0.0509565
MYL10	NC_000007	101256755	101272398	0	0	0	0
LOC100289497	NC_000007	101442411	101458561	0.0728826	0.224793	0	0.0507875
CUX1	NC_000007	101459292	101927250	1.0641364	1.0693199	2.0431286	2.383797
SH2B2	NC_000007	101928446	101962178	0.5884873	0.5082225	0.3560035	0.3116619
PMS2L12	NC_000007	101977726	101979560	0	0	0	0
SPDYE6	NC_000007	101987648	101998476	0.0134152	0.0275845	0.0596727	0.037393
LOC100287351	NC_000007	102004188	102004764	0.1162652	0	0.3102981	0.5671274
LOC100289561	NC_000007	102016255	102021078	1.0987058	0.7907095	1.7593905	1.6078061
PRKRIP1	NC_000007	102036804	102067129	0.7455684	1.0151246	0.5019403	0.7161251
LOC100131389	NC_000007	102065470	102067129	0.0529497	0	0	0.0184487
ORAI2	NC_000007	102073996	102097268	2.6118366	2.8908315	1.430443	2.2067885
ALKBH4	NC_000007	102096685	102105288	0.2335413	0.240105	0.264428	0.8137045
LRWD1	NC_000007	102105390	102113612	4.2988489	5.5091682	2.7393199	3.9430576
POLR2J	NC_000007	102113547	102119381	32.477608	36.350209	38.257188	44.855886
LOC100286937	NC_000007	102120179	102121475	0	0	0.0301446	0.0472242
LOC100132214	NC_000007	102120532	102213112	2.3377487	2.4034514	0.6162151	0.502789
POLR2J3	NC_000007	102178366	102213068	1.700378	3.3080705	3.0952239	1.9869713

SPDYE2	NC_000007	102196256	102202755	0.0360527	0.0741319	0	0.025123
RASA4	NC_000007	102220093	102257205	0.1646295	0.4271709	0.0767166	0.0163886
UPLP	NC_000007	102277474	102283238	6.5594376	1.0115687	3.2386789	5.0965283
POLR2J2	NC_000007	102277474	102312182	0.6923	2.8997516	0.6387	1.4651342
LOC442609	NC_000007	102290772	102301606	0.0252286	0.0259377	0.0224441	0.0175803
LOC100286974	NC_000007	102313326	102319965	0.1535309	0.078923	0	0.0267466
LOC100133005	NC_000007	102320187	102330843	0	0	0	0
LOC100287390	NC_000007	102329357	102330749	0.2328383	0.1196911	0.0517849	0.0811255
FAM185A	NC_000007	102389399	102449672	0.2140968	0.4602384	0.5887145	0.9629612
LOC100287006	NC_000007	102389399	102392229	0	0	0	0
RPL7AP39	NC_000007	102395577	102396436	0	0	0	0
FBXL13	NC_000007	102453308	102715288	0.0673639	0	0.1078719	0.1314372
LRRC17	NC_000007	102553452	102585396	0.0878087	0.0677074	0.0390585	0.0152971
NF-E4	NC_000007	102613884	102619114	0	0	0	0.0318346
ARMC10	NC_000007	102715328	102740205	4.5803787	4.8809761	5.978403	6.4884204
CRYZP1	NC_000007	102728148	102730222	0	0	0	0
NAPEPLD	NC_000007	102740023	102789569	1.2865695	0.7414021	0.5977998	1.1649217
RPL19P12	NC_000007	102781717	102782850	0	0	0	0
LOC100287434	NC_000007	102792454	102792717	0	0	0	0.34801
DPY19L2P2	NC_000007	102815720	102920752	0	0	0	0
S100A11P	NC_000007	102902398	102902928	0	0	0	0
PMPCB	NC_000007	102937873	102955133	2.1419016	2.1444534	2.3344808	2.2193073
DNAJC2	NC_000007	102952921	102985320	0.3576258	0.3881034	0.5656067	0.7614685
PSMC2	NC_000007	102988089	103008656	23.168518	27.903044	28.395023	36.922332
RPS29P16	NC_000007	102989019	102989306	0	0	0	0
SLC26A5	NC_000007	102993177	103086598	0.0307116	0	0.0409828	0.0321015
RELN	NC_000007	103112231	103629963	5.8795145	1.6283362	0.2061146	1.5006746
LOC100287468	NC_000007	103616167	103629767	0	0	0	0
ORC5L	NC_000007	103766788	103848463	3.2224749	4.0854088	3.9748312	5.276351
LHFPL3	NC_000007	103969104	104549005	0	0.0426527	0.0123026	0.0192731
LOC645579	NC_000007	104308180	104312032	0	0	0	0
RN7SLP5	NC_000007	104552320	104552592	0	0	0	0
LOC100216545	NC_000007	104650989	104654588	0	0	0	0
MLL5	NC_000007	104654637	104754532	1.8506541	1.2640555	3.7941103	5.0245684
SRPK2	NC_000007	104756823	105029341	1.5991486	1.5974519	1.5134542	2.9083755
RPL36P12	NC_000007	104885036	104885462	0	0	0	0
PUS7	NC_000007	105096958	105162685	1.6777023	2.5548594	1.5374186	2.1096357
LOC100131785	NC_000007	105170482	105171118	0	0	0	0
RINT1	NC_000007	105172532	105208124	3.3817692	4.5547784	4.4536541	5.0321121
EFCAB10	NC_000007	105205580	105221976	0	0	0	0
YBX1P2	NC_000007	105222302	105223820	0	0	0	0
ATXN7L1	NC_000007	105245221	105517031	0.5277016	0.2489268	0.7400867	0.6705546
RPL13AP16	NC_000007	105459938	105460573	0	0	0	0
FLJ23834	NC_000007	105603657	105676877	0.0338063	0.0139026	0.0661651	0.0753843
SYPL1	NC_000007	105730951	105753057	13.264093	12.199319	20.231219	14.074182
LOC100129009	NC_000007	105761108	105767146	0	0	0	0
NAMPT	NC_000007	105888731	105925638	12.631999	8.1452401	112.49723	16.174641
FLJ36031	NC_000007	106299149	106301591	8.6529265	6.5287519	4.4810963	2.4068672
PIK3CG	NC_000007	106505924	106547592	0.024511	0.0923996	0.0436113	0.0626276
PRKAR2B	NC_000007	106685178	106802256	0.1440532	0.3949382	0.0854358	0.1171123
HBP1	NC_000007	106809460	106842974	10.889965	9.9821938	17.745237	17.104032
COG5	NC_000007	106842991	107204485	5.3167117	5.03988	5.8038839	7.6222297
RPL37AP6	NC_000007	107090535	107090807	0	0	0	0
GPR22	NC_000007	107110502	107116125	0	0	0	0
DUS4L	NC_000007	107204432	107218968	1.1524203	1.0241571	1.5639026	1.1024956
BCAP29	NC_000007	107220710	107271165	2.4460361	2.0669443	3.5293929	4.5110754
LOC100287065	NC_000007	107283205	107283466	0	0	0	0
LOC286002	NC_000007	107297075	107329563	0.0217243	0	0	0
SLC26A4	NC_000007	107301080	107358254	0.0356578	0.0733199	0.0713749	0.0621194
LOC100128597	NC_000007	107330468	107333679	0.155569	0.0799706	0	0
LOC100128737	NC_000007	107383198	107389492	0.2758834	0.3403646	0.2208902	0.3652685
CBLL1	NC_000007	107384279	107402083	1.1847273	1.4722205	1.4663878	3.1156112
SLC26A3	NC_000007	107405912	107443678	0.0455579	0	0.0135099	0.0105822
LOC100128307	NC_000007	107436058	107450040	0	0	0	0
DLD	NC_000007	107531586	107561643	7.4904782	7.360135	7.7998496	10.447884
LAMB1	NC_000007	107564246	107643804	12.787537	9.9471499	7.12932	10.456253

LAMB4	NC_000007	107663996	107770801	0.0450136	0.0077131	0.0200227	0.0209115
NRCAM	NC_000007	107788082	108096826	0.2207186	0.1231862	0.0897634	0.0791
RNU7-83P	NC_000007	107911445	107911504	0	0	0	0
PNPLA8	NC_000007	108112071	108166638	7.0840068	5.4655277	6.2467499	7.079292
RPL7P32	NC_000007	108150657	108151486	0	0	0	0
THAP5	NC_000007	108202671	108210167	3.2532015	3.0731131	5.4251744	4.3498871
DNAJB9	NC_000007	108210356	108215294	2.9101107	2.2486889	4.3863148	3.2807757
C7orf66	NC_000007	108524038	108524637	0.1041427	0	0	0.0725708
LOC646614	NC_000007	108633888	108635117	0	0	0	0
LOC100287604	NC_000007	109244364	109300859	0	0	0	0
LOC100128056	NC_000007	109479267	109479491	0	0	0	0
EIF3IP1	NC_000007	109599284	109600270	0	0	0	0
RPL3P8	NC_000007	109638436	109639703	0	0	0	0
IMMP2L	NC_000007	110303110	111202347	1.4176849	1.636002	1.9304262	1.8548313
LRRN3	NC_000007	110731062	110765510	0.0353661	0.0606001	0	0
DOCK4	NC_000007	111366164	111846462	0.7170057	0.3306367	0.5393738	0.2828834
RPL7AP42	NC_000007	111611219	111612080	0	0	0	0
ZNF277	NC_000007	111846643	111983989	0.9322848	0.8575934	1.5860196	0.9915759
LOC100287209	NC_000007	112014379	112015484	0	0	0	0
IFRD1	NC_000007	112063226	112116247	9.4872373	10.148336	10.720801	7.8871214
C7orf53	NC_000007	112120908	112130943	0.0973383	0.1250925	0.4762716	0.3052313
NPM1P14	NC_000007	112160451	112162039	0	0	0	0
LOC100128875	NC_000007	112375173	112383013	0	0	0	0
TMEM168	NC_000007	112405787	112430478	1.1568251	1.6925192	1.4942375	1.8447788
C7orf60	NC_000007	112459202	112579932	2.0848096	3.7366284	3.332517	2.6802595
GPR85	NC_000007	112720468	112727833	0.1959986	0.4946085	0.309103	0.3600736
LOC401397	NC_000007	112756773	112758637	0	0	0	0
LOC100287664	NC_000007	113091130	113091456	0	0	0	0
PPP1R3A	NC_000007	113516882	113559082	0	0.0105176	0	0.0071287
RPL36P13	NC_000007	113937122	113937517	0	0	0	0
FOXP2	NC_000007	114055052	114331092	0.5368079	0.4559132	3.3532963	1.7646305
MDFIC	NC_000007	114562209	114659265	7.3072163	14.43672	6.9504897	9.2067411
LOC100287693	NC_000007	115136881	115144135	0	0	0	0
TFEC	NC_000007	115575202	115670798	0.1988308	0.0340698	0.1061312	0.0554213
TES	NC_000007	115850581	115898837	5.2693836	1.665673	0.9375579	2.4223687
CAV2	NC_000007	116139444	116148595	8.9733742	10.936546	6.2774038	6.0117907
CAV1	NC_000007	116164839	116201230	115.36411	80.42445	43.305181	83.9919
LOC100287239	NC_000007	116166414	116201239	1.8433033	1.0173746	0.6559415	2.4067233
MET	NC_000007	116312459	116438440	8.6632524	8.9473423	11.572317	19.009811
CAPZA2	NC_000007	116502563	116559313	24.566987	22.438277	28.576404	32.76966
ST7OT1	NC_000007	116592500	116594388	0	0	0	0
ST7	NC_000007	116593381	116870077	4.1570773	4.0309141	4.5269563	6.2683633
ST7OT4	NC_000007	116593953	116599867	0	0	0	0
TPM3L2	NC_000007	116610961	116613033	0	0	0	0
ST7OT2	NC_000007	116752346	116785614	0	0	0	0
ST7OT3	NC_000007	116822735	116849991	0	0	0	0
WNT2	NC_000007	116916685	116963343	0.0302361	0.5284608	0	0
ASZ1	NC_000007	117003276	117067577	0	0	0	0
LOC100130680	NC_000007	117080036	117085284	0	0	0	0
CFTR	NC_000007	117120017	117308719	0	0.0073685	0.0637599	0.054937
CTTNBP2	NC_000007	117350705	117513561	0.3901602	0.3178732	0.2750583	0.6053159
LOC100127914	NC_000007	117513734	117523777	0	0	0	0
LSM8	NC_000007	117824086	117832880	7.0949326	9.9562508	11.337397	12.278069
ANKRD7	NC_000007	117864717	117882746	0.1279425	0.1973074	0.1422764	0.2228885
LOC100135063	NC_000007	118520241	118521304	0	0	0	0
LOC648442	NC_000007	118590436	118591315	0	0	0	0
LOC100287273	NC_000007	119589609	119590879	0.0691554	0	0.0307613	0
KCND2	NC_000007	119913722	120390387	0.1565754	0.7540451	0.029325	0.0344551
TSPAN12	NC_000007	120427374	120498177	0.4113719	0.5286669	0.3049732	0.4538789
ING3	NC_000007	120590817	120615711	1.8450993	1.5982228	1.8353237	2.9865584
C7orf58	NC_000007	120628751	120937498	2.7668496	1.9904227	2.6915749	3.0477407
WNT16	NC_000007	120965421	120981158	0.0680735	0.167968	0.1332321	0.1233344
FAM3C	NC_000007	120988905	121036422	14.60907	9.0295705	23.363179	34.480095
CYCSP19	NC_000007	121038508	121038824	0	0	0	0
RPL18P4	NC_000007	121080866	121081315	0	0	0	0
LOC100131285	NC_000007	121377856	121485221	0	0	0	0

PTPRZ1	NC_000007	121513159	121702090	0.0215459	0.0443029	0.0239598	0.0375351
AASS	NC_000007	121713598	121784344	0.6063374	0.8080833	0.6393061	1.142369
LOC100130618	NC_000007	121799333	121802835	0	0	0	0
RPL31P37	NC_000007	121874562	121875002	0	0	0	0
FEZF1	NC_000007	121942051	121944599	0.0288374	0	0.0256546	0
LOC100287843	NC_000007	121944699	121945931	0	0	0	0
LOC154860	NC_000007	121945660	121946714	0	0	0.0487501	0
CADPS2	NC_000007	121958478	122526554	0.6580888	0.6143698	0.9555687	1.5865901
RPS26P31	NC_000007	122321347	122321780	0	0	0	0
RNF133	NC_000007	122337766	122339208	0	0	0	0
RNF148	NC_000007	122341720	122343021	0	0	0	0
TAS2R16	NC_000007	122634759	122635754	0	0	0	0.0307479
SLC13A1	NC_000007	122753588	122840025	0	0.0356993	0.0514848	0.0564588
LOC100129401	NC_000007	122870146	122870809	0	0	0	0
IQUB	NC_000007	123092235	123174718	0	0.0435153	0.0627569	0.0393257
NDUFA5	NC_000007	123181083	123197958	0.935672	1.3700773	1.9170419	2.0350726
ASB15	NC_000007	123249112	123277932	0.016241	0.0166975	0	0.0226348
LMOD2	NC_000007	123295861	123304147	0.0613516	0.0420506	0.0545801	0.0142508
WASL	NC_000007	123321997	123389116	4.0946792	4.5673859	5.1723374	8.6500391
HYALP1	NC_000007	123454193	123459484	0	0	0	0
HYAL4	NC_000007	123485223	123517532	0.0547755	0.056315	0.0324866	0.0127233
SPAM1	NC_000007	123565329	123611464	0	0	0.0155643	0.0243829
LOC730130	NC_000007	123670970	123673523	0.0172076	0.0353825	0	0
LOC136157	NC_000007	124116425	124117009	0.2253755	0.0772366	0.1336669	0.7329031
RPS2P31	NC_000007	124120556	124121490	0	0	0	0
GPR37	NC_000007	124386114	124405681	0.8468726	0.1680248	1.3085392	1.480513
LOC154872	NC_000007	124417359	124430864	0	0	0	0
POT1	NC_000007	124462440	124570037	3.2729697	3.2985433	6.0054321	3.7887221
LOC646837	NC_000007	124673487	124675443	0	0	0	0
RPL31P39	NC_000007	124940517	124940958	0	0	0	0
GRM8	NC_000007	126078652	126892428	0	0.0114941	0.009946	0.0311624
LOC646873	NC_000007	126930170	126936257	0	0	0	0
ZNF800	NC_000007	127010353	127032767	2.8937964	2.5684549	3.5189661	3.2786464
GCC1	NC_000007	127220682	127225654	1.1486953	1.1153696	1.2395404	1.6972646
ARF5	NC_000007	127228517	127231752	23.277284	36.220641	32.701236	33.881512
FSCN3	NC_000007	127233689	127241843	0.0233395	0.0479909	0.0415269	0.0325278
PAX4	NC_000007	127250346	127255780	0	0	0.0194515	0
SND1	NC_000007	127292202	127732659	14.707875	17.091897	12.270929	11.995035
LOC100196944	NC_000007	127497590	127498524	0	0	0	0
LRRC4	NC_000007	127667124	127671002	0.0118555	0	0.010547	0.0165227
LEP	NC_000007	127881331	127897682	0.0512964	0.0263691	0.0114087	0.0625545
MGC27345	NC_000007	127937736	127948588	0	0	0	0
RBM28	NC_000007	127950436	127983962	0.4693652	0.2895341	0.3340483	0.4469989
RNU7-27P	NC_000007	127984136	127984193	0	0	0	0
LOC401399	NC_000007	127990379	128001739	0	0.0263614	0.0342161	0.0089337
IMPDH1	NC_000007	128032331	128050036	4.7811813	10.360736	2.9931836	3.5560756
RPL37P16	NC_000007	128073476	128073767	0	0	0	0
RNU7-54P	NC_000007	128083403	128083663	0	0	0	0
C7orf68	NC_000007	128095884	128098472	2.0853623	2.7839631	2.8243284	3.7305093
METTL2B	NC_000007	128116783	128142978	4.5922075	5.9430047	4.8200024	5.8807626
LOC100128729	NC_000007	128173707	128262748	0	0	0	0
RPS10P15	NC_000007	128210249	128210779	0	0	0	0
FLJ45340	NC_000007	128281295	128301052	0	0	0	0
LOC100130600	NC_000007	128294508	128298845	0.0921347	0.0378896	0.0163931	0.0385219
FAM71F2	NC_000007	128312346	128323375	0.1467387	0.1508628	0.0979071	0.2045067
LOC100287978	NC_000007	128332960	128333984	0	0	0	0
FAM71F1	NC_000007	128355443	128371797	0.0767431	0	0.0682728	0.0178259
CALU	NC_000007	128379346	128411531	31.631996	36.58301	41.516755	50.118072
OPN1SW	NC_000007	128412543	128415844	0.5203343	0.5349583	1.3174954	1.8966227
CCDC136	NC_000007	128432099	128462183	0.1265304	0.0867244	0.1500866	0.1102143
FLNC	NC_000007	128470483	128499328	14.25504	18.7745	8.6427098	15.754821
ATP6V1F	NC_000007	128502898	128505902	34.344494	37.598345	31.685437	35.987556
LOC100130705	NC_000007	128506464	128508898	0.0732471	0.3765283	0	0.1531244
KCP	NC_000007	128516919	128550773	0.122101	0.0502131	0.0506914	0.0226893
LOC392787	NC_000007	128552714	128563124	0	0	0	0
IRF5	NC_000007	128577994	128590089	0.2004154	0	0.140089	0.0598532

TNPO3	NC_000007	128594948	128695198	3.8689776	5.3939302	4.8911918	8.1799893
LOC100132905	NC_000007	128637443	128670509	0	0	0	0
LOC286016	NC_000007	128695277	128697293	0	0	0	0
CYCSP20	NC_000007	128757567	128757881	0	0	0	0
LOC407835	NC_000007	128766325	128768061	0	0	0	0
TSPAN33	NC_000007	128784712	128808671	0.0458511	0.1178492	0.0611855	0.0319508
SMO	NC_000007	128828713	128853386	1.2580152	1.6197367	0.0732164	0.1392785
AHCYL2	NC_000007	128864864	129070052	0.9083867	1.0413588	0.8367322	0.9130886
RNU7-16P	NC_000007	129045476	129045537	0	0	0	0
FAM40B	NC_000007	129074274	129128240	4.317721	2.90125	5.0003729	7.043722
LOC100287482	NC_000007	129142320	129152773	0.2547724	0.1309664	0.5666314	0.5621958
LOC100127891	NC_000007	129232744	129252801	0.1959475	0.0575585	0.0747087	0.0585189
NRF1	NC_000007	129251555	129396922	0.4123178	0.8976835	0.906235	1.4703998
LOC100287517	NC_000007	129415053	129416229	0	0	0	0
UBE2H	NC_000007	129472995	129592789	3.0931307	3.7266369	6.5783661	6.028431
ZC3HC1	NC_000007	129658126	129691233	6.3144011	7.0348243	5.0046518	6.5441877
LOC100288107	NC_000007	129690577	129691274	0	0	0	0
KLHDC10	NC_000007	129710349	129773593	3.725426	2.7083766	4.529756	7.116808
LOC100287552	NC_000007	129775030	129775560	1.4897706	2.21237	3.6815034	4.6715916
TMEM209	NC_000007	129804555	129845338	4.7109529	3.9766492	6.363694	5.2524474
C7orf45	NC_000007	129847704	129856684	0.0380175	0	0	0.0264921
CPA2	NC_000007	129906703	129929638	0.1652189	0.0339725	0	0.0690787
CPA4	NC_000007	129932974	129964020	5.7411961	0.3047514	1.4295525	13.589314
CPA5	NC_000007	129984630	130008571	0.0423393	0.0435293	0	0.1180149
CPA1	NC_000007	130020290	130027949	0	0	0.0547202	0.042862
TSGA14	NC_000007	130036375	130080854	1.0220519	1.4992791	2.8053557	3.7434267
MEST	NC_000007	130126046	130146133	40.519424	5.1670367	0.8078585	49.215827
MESTIT1	NC_000007	130126898	130131013	0	0	0	0
COPG2	NC_000007	130146080	130353598	3.9437693	3.2776783	3.844629	4.5089826
TSGA13	NC_000007	130353486	130371406	0.026587	0	0	0.0370537
KLF14	NC_000007	130417478	130418860	0	0.228694	0	0.0221438
LOC347674	NC_000007	130507218	130507984	0	0	0	0
LOC100133252	NC_000007	130524963	130528484	0	0	0	0
FLJ43663	NC_000007	130628919	130793562	0	0	0	0
MKLN1	NC_000007	130794855	131181398	2.0594118	2.2558062	3.544362	4.7451528
RPL27P11	NC_000007	130927314	130927803	0	0	0	0
PODXL	NC_000007	131185021	131241376	5.5008733	11.960312	2.6918778	3.8690521
RPL31P36	NC_000007	131204873	131205305	0	0	0	0
LOC647030	NC_000007	131346642	131347418	0	0	0	0
RPS14P10	NC_000007	131350338	131350544	0	0	0	0
RPS15AP22	NC_000007	131386983	131387440	0	0	0	0
LOC100128596	NC_000007	131438412	131460814	0	0	0	0
LOC100130704	NC_000007	131577362	131578385	0	0	0	0
LOC100132328	NC_000007	131771025	131773157	0	0	0	0
PLXNA4	NC_000007	131808091	132333447	0.0399218	0.1788339	0.0228314	0.0317933
CHCHD3	NC_000007	132469623	132766833	11.434806	14.050763	10.099183	19.046946
LOC729998	NC_000007	132719268	132720699	0	0	0	0
FAM10A7	NC_000007	132852760	132855356	0	0	0	0
EXOC4	NC_000007	132937823	133750514	3.100921	2.9076885	3.7291404	7.2004713
RPS15AP23	NC_000007	132999763	133000165	0	0	0	0
RPS3AP27	NC_000007	133417246	133417973	0	0	0	0
LRGUK	NC_000007	133812105	133948933	0.0651085	0.0669384	0.0579223	0.0567127
SLC35B4	NC_000007	133974089	134001827	1.7473549	1.8892708	2.9139618	4.1291466
AKR1B1	NC_000007	134127107	134143888	138.66642	75.95207	776.58169	352.51469
AKR1B10	NC_000007	134212344	134226166	0.3029013	0.1132416	1.0043861	0.3837704
AKR1B10L	NC_000007	134250233	134264301	0.2310633	0.1425344	0.4111206	0.0966084
BPGM	NC_000007	134331531	134364568	7.2203648	5.621107	2.5990784	5.2641054
LOC100130187	NC_000007	134419649	134419945	0	0	0	0
CALD1	NC_000007	134464171	134655474	55.478133	50.809703	17.841606	20.362613
AGBL3	NC_000007	134671259	134820530	0.0621968	0.0383669	0.0663984	0.2427106
LOC100287674	NC_000007	134671259	134672316	0	0.0427064	0	0.115784
TMEM140	NC_000007	134832824	134850650	10.202268	4.4107607	9.681302	4.9400845
C7orf49	NC_000007	134850532	134855532	8.4988947	10.174888	6.963943	12.448662
WDR91	NC_000007	134868590	134896316	0.2949546	0.7238735	0.2962578	0.3911816
STRA8	NC_000007	134916731	134943244	0	0.0455019	0	0
SLC23A4	NC_000007	134961969	134998313	0	0	0	0

CNOT4	NC_000007	135046549	135194851	1.9262684	1.4530155	2.1234504	3.6329656
LOC647081	NC_000007	135129191	135130492	0	0	0	0
NUP205	NC_000007	135242662	135333501	9.8457503	13.727333	15.995877	17.259785
LOC100288247	NC_000007	135335209	135336168	0	0	0	0
RPL15P11	NC_000007	135344786	135345400	0	0	0	0
PL-5283	NC_000007	135347221	135361160	5.313197	5.3901736	7.9509861	8.7289485
SLC13A4	NC_000007	135365985	135412933	0.3490364	0.124816	0.2160086	0.2960969
FAM180A	NC_000007	135414355	135433447	31.685473	17.249347	7.0114175	18.877585
MTPN	NC_000007	135611503	135662204	18.319307	22.161272	16.176567	21.604574
LUZP6	NC_000007	135611503	135662204	13.227467	14.390285	22.337152	16.91307
MTPN-LUZP6	NC_000007	135611503	135662204	0	0	0	0
LOC392100	NC_000007	136398573	136400158	0	0	0	0
CHRM2	NC_000007	136553399	136701771	0.0443474	0.0151979	0.0263018	0.030903
RPL18P5	NC_000007	136848778	136849395	0	0	0	0
PTN	NC_000007	136912088	137028546	0.7944161	0.4667104	0.5300509	0.2372489
LOC100287705	NC_000007	136936018	136939712	0	0	0	0
DGKI	NC_000007	137074385	137531609	0.386156	1.2855529	0.0245382	0.2178339
RPL6P19	NC_000007	137404799	137407632	0	0	0	0
LOC100288278	NC_000007	137531743	137533814	0.0636316	0.1090333	0.037739	0.1034624
CREB3L2	NC_000007	137559725	137686846	5.629854	10.745831	6.1150586	14.965316
LOC100130880	NC_000007	137638094	137642712	0	0	0	0
LOC100128052	NC_000007	137692110	137709119	0	0	0	0
AKR1D1	NC_000007	137761196	137803050	0.032651	0.0167843	0.0290472	0.0113763
LOC100131932	NC_000007	137804126	137809027	0	0	0	0
RPS17P12	NC_000007	137982806	137983252	0	0	0	0
LOC442727	NC_000007	138088705	138089396	0	0	0	0
IMPDH1P3	NC_000007	138125435	138127681	0	0	0	0
TRIM24	NC_000007	138145079	138270333	0.5267204	0.6430597	2.9286566	3.5939308
RPS3AP28	NC_000007	138175288	138176118	0	0	0	0
SVOPL	NC_000007	138279030	138363790	0	0.0248397	0.021494	0.0168361
RPL21P73	NC_000007	138330855	138331193	0	0	0	0
RPL17P27	NC_000007	138373684	138374300	0	0	0	0
LOC647123	NC_000007	138386404	138387812	0	0	0	0
ATP6V0A4	NC_000007	138391039	138482941	0.0130915	0.0538378	0.0232932	0.0364908
TMEM213	NC_000007	138482739	138490769	0	0	0	0.0090339
KIAA1549	NC_000007	138516126	138604570	0.1332934	0.0144252	0.0873758	0.0317762
ZC3HAV1L	NC_000007	138710452	138720775	2.2312565	2.3712909	1.2712842	1.3801286
ZC3HAV1	NC_000007	138728266	138794465	4.4288916	2.6527807	4.8592476	3.8412447
RPL17P28	NC_000007	138734202	138734752	0	0	0	0
TTC26	NC_000007	138818490	138874550	1.3893444	0.7982191	1.4541168	1.9505385
LOC100132310	NC_000007	138883205	138883930	0	0	0	0
RPL37AP5	NC_000007	138912224	138912605	0	0	0	0
UBN2	NC_000007	138916451	138992982	0.2935238	0.3716576	0.5332486	0.7384936
C7orf55	NC_000007	139025897	139030541	1.3836775	1.6257896	1.6998942	1.1019446
LUC7L2	NC_000007	139044634	139108200	1.2853251	0.9656745	0.8502658	1.5731565
LOC100129148	NC_000007	139102202	139112272	0	0.2902574	0.2511621	0.1967337
KLRG2	NC_000007	139138088	139168457	0.0284639	0	0	0
LOC100288422	NC_000007	139164915	139168379	0.052759	0	0	0
LOC442730	NC_000007	139187200	139188563	0	0	0	0
CLEC2L	NC_000007	139208674	139229731	0	0.0350803	0	0.0237771
HIPK2	NC_000007	139246316	139342363	1.0081609	0.69208	2.541651	5.0586326
LOC653052	NC_000007	139413130	139477693	4.5170492	3.5786128	6.9969041	11.035325
TBXAS1	NC_000007	139478181	139720125	0.1513499	0.0389009	0.2356289	0.3295833
PARP12	NC_000007	139723549	139763521	12.450647	6.7697632	7.6214985	2.3265537
JHDM1D	NC_000007	139784546	139876741	0.2968828	0.2658426	0.4217323	0.3503609
LOC100134229	NC_000007	139877061	139879440	0	0	0	0
LOC100130972	NC_000007	139910580	139993046	0	0	0	0
SLC37A3	NC_000007	140033552	140098311	4.4996831	4.1212284	4.038468	3.616529
RAB19	NC_000007	140107447	140125950	0.1105616	0	0.0491793	0
LOC642355	NC_000007	140135113	140146606	0	0	0	0
MKRN1	NC_000007	140152840	140179369	10.064388	9.1365332	11.837274	12.33455
LOC100287491	NC_000007	140154961	140179490	0.1910793	0	0.0566631	0.2663033
LOC100287532	NC_000007	140191292	140191649	0	0	0	0
DENND2A	NC_000007	140218220	140302342	0.1396391	0.6134084	0.1242268	0.026538
ADCK2	NC_000007	140372953	140394908	7.0086647	4.6062605	8.6662963	13.459919
LOC100134713	NC_000007	140395136	140396877	0	0	0	0

NDUFB2	NC_000007	140396481	140406446	24.3269	26.653643	23.221584	29.325641
BRAF	NC_000007	140433812	140624564	1.849196	2.5911079	3.2238576	2.8889434
LOC650172	NC_000007	140697740	140699594	0	0	0	0
MRPS33	NC_000007	140705961	140714781	5.8095582	7.5766537	8.1832114	6.934642
LOC100129514	NC_000007	140774032	140912564	0	0.2134649	0	0.0964563
LOC100287594	NC_000007	141051777	141087904	0	0	0	0
LOC100130169	NC_000007	141115747	141116169	0	0	0	0
LOC100131199	NC_000007	141130692	141170586	0	0	0	0.0520831
AGK	NC_000007	141251078	141354209	3.5308836	4.726879	3.8496065	5.9365149
KIAA1147	NC_000007	141356528	141401953	0.397558	0.4830462	0.5626706	0.4197489
FLJ40852	NC_000007	141404138	141438030	0	0	0	0
WEE2	NC_000007	141408153	141431071	0	0.029522	0.0510912	0.0600292
SSBP1	NC_000007	141438176	141450257	7.3480324	9.1374077	8.529246	8.6802999
TAS2R3	NC_000007	141463897	141464997	0	0.0410385	0.1420438	0.111262
TAS2R4	NC_000007	141478289	141479188	0	0	0.0434417	0.1020829
PS3	NC_000007	141487614	141488436	0	0	0	0
TAS2R5	NC_000007	141490017	141491166	0	0.0392899	0.1359915	0.0532607
TRNAQ48P	NC_000007	141503357	141503428	0	0	0	0
LOC100288984	NC_000007	141503494	141504450	0	0	0	0
LOC642627	NC_000007	141511710	141512057	0	0	0	0
LOC136242	NC_000007	141536086	141541221	0.110887	0.0380012	0.2301791	0.0257568
OR9A3P	NC_000007	141562560	141563719	0	0	0	0
OR9A1P	NC_000007	141586948	141587736	0	0	0	0
OR9N1P	NC_000007	141611104	141611924	0	0	0	0
OR9A4	NC_000007	141618676	141619620	0	0	0	0
CLEC5A	NC_000007	141627157	141646783	0.0125173	0.0257382	0.0111357	0.0087226
TAS2R38	NC_000007	141672431	141673573	0	0	0	0
MGAM	NC_000007	141695679	141806547	0.0271118	0.1114951	0.0120597	0.0188926
LOC93432	NC_000007	141811549	141843783	0	0	0	0
LOC100124692	NC_000007	141870970	141921088	0	0	0	0
LOC100289017	NC_000007	141940556	141946886	0	0	0	0
MOXD2	NC_000007	141940630	141943268	0	0	0	0
TRYX3	NC_000007	141951963	141957878	0.0962721	0	0	0
LOC730441	NC_000007	141968659	141973463	0.0545941	0	0.0485684	0
TRY3	NC_000007	141987077	141993159	0	0	0	0
TRBV1	NC_000007	141998672	141999502	0	0	0	0
TRBV2	NC_000007	142000718	142001343	0	0	0	0
TRBV3-1	NC_000007	142008412	142009022	0	0	0	0
TRBV4-1	NC_000007	142013036	142013628	0	0	0	0
TRBV5-1	NC_000007	142020894	142021485	0	0	0	0
TRBV6-1	NC_000007	142028136	142028791	0	0	0	0
TRBV7-1	NC_000007	142031940	142032685	0	0	0	0
TRBV4-2	NC_000007	142045336	142046030	0	0	0	0
TRBV7-8	NC_000007	142099280	142099938	0	0	0	0
TRBV6-9	NC_000007	142103433	142104595	0	0	0	0
TRBV5-7	NC_000007	142111253	142118659	0	0	0	0
TRBV7-7	NC_000007	142119645	142120321	0	0	0	0
TRBV6-8	NC_000007	142123778	142124607	0	0	0	0
TRBV5-6	NC_000007	142131229	142132219	0	0	0	0
TRBV7-6	NC_000007	142138715	142139770	0	0	0	0
TRBV6-7	NC_000007	142143283	142144174	0	0	0	0
TRBV5-5	NC_000007	142148787	142155057	0	0	0	0
TRBV7-5	NC_000007	142157259	142157849	0	0	0	0
TRBV6-6	NC_000007	142161675	142162453	0	0	0	0
TRBV5-4	NC_000007	142168330	142169010	0	0	0	0
TRBV7-4	NC_000007	142176154	142176790	0	0	0	0
TRBV6-5	NC_000007	142180148	142190437	0	0	0	0
TRBV11-2	NC_000007	142190520	142198008	0	0	0	0
TRBV10-2	NC_000007	142206345	142206960	0	0	0	0
TRBV12-1	NC_000007	142213427	142222744	0	0	0	0
TRBV11-1	NC_000007	142223654	142224267	0	0	0	0
TRBV9	NC_000007	142237905	142240052	0	0	0	0
TRBV5-3	NC_000007	142242036	142242747	0	0	0	0
TRBV7-3	NC_000007	142246962	142247616	0	0	0	0
TRBV6-4	NC_000007	142250345	142251227	0	0	0	0
TRBV5-2	NC_000007	142251642	142259498	0	0	0	0

TRBV19	NC_000007	142326571	142327137	0	0	0	0
TRBV20-1	NC_000007	142334241	142500429	0	0	0	0
TRBVB	NC_000007	142418284	142423900	0	0	0	0
TRBV28	NC_000007	142428199	142429208	0	0	0	0
PRSS1	NC_000007	142457319	142460927	0	0	0	0
TRY5	NC_000007	142468264	142471794	0	0	0	0
TRY6	NC_000007	142478757	142482400	0	0	0	0
LOC100288415	NC_000007	142486017	142494693	0	0	0	0
TRBV30	NC_000007	142494686	142510975	0	0	0	0
EPHB6	NC_000007	142552792	142568847	0.0652212	0.1005814	0	0.0605983
TRPV6	NC_000007	142568960	142583477	0	0	0.0270197	0.0211644
TRPV5	NC_000007	142605648	142630820	0.0176783	0	0	0.0123189
C7orf34	NC_000007	142636603	142637957	0.0572243	0	0	0.0398761
KEL	NC_000007	142638201	142659503	0	0.0177399	0.0921026	0.0601195
OR9A2	NC_000007	142723287	142724219	0	0.0484281	0	0.0328241
OR9P1P	NC_000007	142744067	142744972	0	0	0	0
OR6V1	NC_000007	142749438	142750379	0	0.0479654	0	0.0325105
OR6W1P	NC_000007	142759381	142760882	0	0	0	0
PIP	NC_000007	142829174	142836834	0	0	0	0
TAS2R39	NC_000007	142880512	142881528	0	0	0	0.0903389
TAS2R40	NC_000007	142919172	142920143	0.0452142	0	0	0.1260283
GSTK1	NC_000007	142960522	142966222	22.475972	13.414974	19.899577	19.184263
TMEM139	NC_000007	142982063	142985141	0.0185985	0.0191212	0.0661829	0.1425619
CASP2	NC_000007	142985402	143004785	5.5694028	4.5873141	4.6989078	9.6839026
HINTP1	NC_000007	143009623	143010216	0	0	0	0
CLCN1	NC_000007	143013219	143049097	0.0284179	0.0438248	0.0252813	0.0297041
FAM131B	NC_000007	143050493	143059840	0.2968138	0.3865307	0.044009	0.0620495
ZYX	NC_000007	143078360	143088204	13.62662	25.883467	21.057752	46.163086
EPHA1	NC_000007	143088205	143105985	0.0261363	0.0268709	0.0348774	0.0728513
TAS2R62P	NC_000007	143134128	143135066	0	0	0	0
TAS2R60	NC_000007	143140546	143141502	0	0	0	0.0320009
TAS2R41	NC_000007	143174966	143175889	0.095126	0	0.0423134	0
OR2R1P	NC_000007	143185355	143186704	0	0	0	0
OR10AC1P	NC_000007	143207826	143209204	0	0	0	0
RPL26P22	NC_000007	143229787	143230303	0	0	0	0
LOC780811	NC_000007	143242134	143243404	0	0	0	0
LOC441294	NC_000007	143268890	143271244	0.0186617	0.0191862	0.0664078	0.0260084
FAM115B	NC_000007	143276019	143305850	0	0	0	0
FAM115C	NC_000007	143318045	143422176	0.5948754	0.1790032	0.1936162	1.324483
CTAGE6	NC_000007	143452182	143454843	0.0165095	0.0169735	0.0440619	0.0230089
LOC780812	NC_000007	143479608	143535687	0	0	0	0
RPL26P24	NC_000007	143493346	143493862	0	0	0	0
FAM115D	NC_000007	143497824	143515065	0	0	0	0
LOC154761	NC_000007	143509061	143533810	0	0	0	0
FAM115A	NC_000007	143550049	143599172	4.3344429	4.1680469	3.9615541	5.1016419
LOC100288478	NC_000007	143557350	143558536	0.0807872	0.1661154	0.0718705	0
OR2F2	NC_000007	143632326	143633279	0	0	0	0.0321015
OR2F1	NC_000007	143657020	143658108	0	0	0	0
OR2Q1P	NC_000007	143677898	143679025	0	0	0	0
OR6B1	NC_000007	143701090	143702025	0	0	0	0
OR2A5	NC_000007	143747495	143748430	0	0	0.0417709	0.0654378
OR2A25	NC_000007	143771313	143772245	0	0	0	0.1312964
OR2A41P	NC_000007	143774386	143775047	0	0	0	0
OR2A12	NC_000007	143792201	143793133	0.0471042	0	0	0.0984723
OR2A2	NC_000007	143806676	143807632	0	0	0.0817086	0
OR2A15P	NC_000007	143815457	143816553	0	0	0	0
OR2A14	NC_000007	143826206	143827138	0	0	0	0.0328241
OR2A13P	NC_000007	143839028	143840234	0	0	0	0
OR2A3P	NC_000007	143854119	143855452	0	0	0	0
OR2AO1P	NC_000007	143873733	143874709	0	0	0	0
CTAGE4	NC_000007	143880548	143883173	0.0334716	0	0.0297773	0.0466487
ARHGEF5L	NC_000007	143883677	143892736	0.1833086	0.1177878	0.22423	0.6865849
OR2A42	NC_000007	143929004	143929918	0.2401543	0.0493808	0.0854592	0.0334698
OR2A20P	NC_000007	143947767	143948696	0	0	0	0
OR2A7	NC_000007	143955789	143956721	0.0471042	0	0.1257156	0.0984723
LOC100289604	NC_000007	143963767	143966877	0	0	0	0

LOC728377	NC_000007	143970400	143991514	0	0	0	0
OR2A9P	NC_000007	143996614	143997598	0	0	0	0
OR2A1	NC_000007	144015218	144016150	0.1413126	0.0484281	0.0419052	0.0656482
ARHGEF5	NC_000007	144052489	144077725	3.2672884	1.1855703	2.8852978	3.0022202
NOBOX	NC_000007	144096039	144107320	0.0298359	0	0	0.0831633
TPK1	NC_000007	144149034	144533146	0.4865118	0.4260837	0.6412065	0.5901473
LOC402715	NC_000007	144707146	144708295	0	0	0	0
LOC643308	NC_000007	144737444	144738056	0	0	0	0
CNTNAP2	NC_000007	145813453	148118086	0.0488807	0.0182744	0	0.0402551
LOC392145	NC_000007	148131639	148143273	0	0	0	0
RPL32P17	NC_000007	148277493	148286036	0	0	0	0
C7orf33	NC_000007	148287657	148312952	0.0324581	0	0	0.0226181
LOC643438	NC_000007	148334292	148334684	0	0	0	0
LOC100132884	NC_000007	148394137	148405726	0	0	0	0
CUL1	NC_000007	148395933	148498202	4.4920598	5.9981705	7.5051731	8.7799589
EZH2	NC_000007	148504475	148581414	4.5175836	4.6950351	6.4507343	9.5695616
RNU7-20P	NC_000007	148518008	148518262	0	0	0	0
RNY5	NC_000007	148638580	148638663	0	0	0	0
RNY4	NC_000007	148660407	148660502	0	0	0	0
RNY3	NC_000007	148680847	148680948	0	0	0	0
RNY1	NC_000007	148684228	148684340	0	0	0	0
PDIA4	NC_000007	148700154	148725782	39.735037	50.418064	56.116662	37.53415
COX6BP1	NC_000007	148750972	148751400	0	0	0	0
ZNF786	NC_000007	148766733	148787869	0.2286169	0.5115624	0.3349853	0.3654744
ZNF425	NC_000007	148799876	148823378	0.292061	0.5433447	0.7918494	0.6687078
ZNF398	NC_000007	148823508	148880134	0.461657	0.5290979	0.6530849	0.7383301
LOC100288541	NC_000007	148844200	148888333	0.7016945	1.0251696	1.1499284	0.8235261
ZNF282	NC_000007	148892577	148923339	3.5550706	4.3714122	3.7195749	3.3661852
ZNF212	NC_000007	148936774	148952697	0.5701392	0.5047515	0.6199254	0.9049514
LOC100289678	NC_000007	148959262	148982085	0.3551372	0.283981	0.3598205	0.5843131
LOC100288576	NC_000007	148959262	148964367	0	0	0.1497991	0.0782245
LOC100128415	NC_000007	148973303	148982565	0	0	0	0
LOC100286898	NC_000007	148986130	148990824	0.0604099	0.1552694	0.1074847	0.2525763
ZNF783	NC_000007	148991180	148994393	0	0	0	0
NPM1P12	NC_000007	149031005	149032261	0	0	0	0
TRNAY17P	NC_000007	149053745	149053820	0	0	0	0
LOC729986	NC_000007	149119766	149121981	0.1388256	0.2446755	0.0882165	0.0552796
ZNF777	NC_000007	149128454	149158053	1.0872609	1.8091011	1.8581525	2.4324448
LOC100288645	NC_000007	149152029	149158232	0.024999	0.0771048	0.1334388	0.1567827
ZNF746	NC_000007	149169885	149194794	1.5355693	1.5787267	2.1285511	1.8912438
ZNF767	NC_000007	149244245	149321818	0.3620735	0.4749392	0.3998615	0.8787252
KRBA1	NC_000007	149412148	149431664	0.7435879	0.8881535	0.7393419	1.4706647
ZNF467	NC_000007	149461452	149470295	0.0192755	0.0198173	0.2915169	0.013432
SSPO	NC_000007	149473131	149531054	0.0084624	0.0058002	0.0200758	0.0176909
ZNF862	NC_000007	149535509	149564568	0.484278	0.3603142	0.793629	0.905825
LOC401431	NC_000007	149564783	149570951	0	0	0	0
ATP6V0E2	NC_000007	149570057	149577787	7.0587919	3.5126077	5.9642784	14.296102
LOC100130217	NC_000007	149730628	149731146	0	0	0	0
LOC100132710	NC_000007	149731182	149734293	0	0	0	0
LOC100286961	NC_000007	149767902	149770808	0	0	0.0134495	0.0105349
LOC100132628	NC_000007	149941590	149941994	0	0	0	0.151234
ARP11	NC_000007	149944591	150020752	0.35694	0.3669718	0.4366226	0.9638287
LRRC61	NC_000007	150020296	150035245	2.843314	0.0230175	2.0913115	1.6381112
C7orf29	NC_000007	150026938	150029810	0	0	0	0
LOC100287053	NC_000007	150034421	150035244	0.3906509	0	0.2606504	0.4083317
RARRES2	NC_000007	150035407	150038763	1.1268777	0	0.8969752	0.8679115
REPIN1	NC_000007	150065879	150071133	6.924699	7.2271869	11.095728	16.852365
ZNF775	NC_000007	150076406	150095719	0.5215834	2.2244136	0.7905442	0.7538432
LOC728743	NC_000007	150102840	150109558	0	0	0	0
LOC100287094	NC_000007	150105085	150109558	0.0312576	0.0803403	0.2363651	0.2940511
GIMAP8	NC_000007	150147962	150176483	0.0111459	0.0114591	0.0198314	0.0233007
LOC389599	NC_000007	150209087	150211135	0	0	0	0
GIMAP7	NC_000007	150211945	150218161	0.0357593	0.0367644	0	0
ATQL3	NC_000007	150238720	150239243	0	0	0	0
GIMAP4	NC_000007	150264458	150271041	0	0.0229941	0.039794	0.0155852
LOC100288724	NC_000007	150311313	150311861	0	0	0.071216	0.4462642

GIMAP6	NC_000007	150322463	150329680	0.0120505	0.0123892	0.0107205	0.0083973
GIMAP2	NC_000007	150382794	150390729	1.8578255	0.0939364	1.3276374	0.4669074
GIMAP1	NC_000007	150413698	150418253	0	0	0.0313282	0
GIMAP5	NC_000007	150434451	150440736	0	0	0.0210089	0
LOC100287165	NC_000007	150439542	150451054	0	0.0707095	0	0
GIMAP3P	NC_000007	150444027	150444853	0	0	0	0
LOC100128542	NC_000007	150446824	150487837	0	0	0.0804477	0.3780849
TMEM176B	NC_000007	150488375	150498448	0	0.0492462	0.0213066	0
TMEM176A	NC_000007	150497854	150502208	0	0	0.0376662	0.0295037
ABP1	NC_000007	150549573	150558379	0.0362012	0	0	0
KCNH2	NC_000007	150642049	150675014	0.0778304	0.1155813	0.0307734	0.1566798
NOS3	NC_000007	150688147	150711676	0.1219655	0.0104494	0.1085039	0.0283301
ATG9B	NC_000007	150709300	150721586	0.0384415	0.0197609	0.0427483	0.0200907
ABCB8	NC_000007	150725510	150744869	2.6558509	3.6953327	2.7467647	4.0480033
ACCN3	NC_000007	150745605	150749843	0.10524	0.2380352	0.337048	0.4106784
CDK5	NC_000007	150750899	150754996	7.8078653	8.1862628	5.8801088	7.1107897
SLC4A2	NC_000007	150756657	150773614	21.666415	18.457343	11.704728	21.530576
LOC100287222	NC_000007	150772157	150773614	0.1466571	0	0.0869801	0.068131
FASTK	NC_000007	150773708	150777951	9.6344396	11.320248	6.6077419	8.6483863
TMUB1	NC_000007	150778172	150780620	4.7236245	4.8801883	4.4082608	5.6150989
AGAP3	NC_000007	150783826	150841523	1.0706154	1.7763856	2.3103964	2.1273432
GBX1	NC_000007	150845676	150864867	0.0995806	0.0682529	0.0295299	0.0693917
ASB10	NC_000007	150872785	150884919	0.0569524	0	0	0.0264578
LOC100288700	NC_000007	150883505	150884919	0	0	0	0
IQCA1L	NC_000007	150887963	150902582	0	0	0	0
ABCF2	NC_000007	150904923	150924317	3.9827366	5.467452	4.2906967	8.2056706
LOC100287283	NC_000007	150929575	150932355	0.2388491	0	0.1416578	0.0554799
CSGLCA-T	NC_000007	150929585	150935905	14.014726	13.885076	8.8043385	9.442028
SMARCD3	NC_000007	150936059	150974231	4.4177129	2.0944389	2.3825079	1.6269467
NUB1	NC_000007	151038858	151075535	3.8474152	2.2395378	6.1660145	3.4892845
LOC401433	NC_000007	151074131	151077504	0	0	0	0
WDR86	NC_000007	151078207	151107124	0.0215327	0	0	0.0300097
LOC100131176	NC_000007	151106322	151109942	0	0	0	0
CRYGN	NC_000007	151127056	151137099	0	0.0624943	0.0540769	0.0423581
RHEB	NC_000007	151163098	151217010	20.480935	17.746734	35.178388	31.451381
ETF1P2	NC_000007	151198915	151201402	0	0	0	0
PRKAG2	NC_000007	151253200	151574316	0.5956839	0.3288952	1.0304328	1.8218113
LOC644090	NC_000007	151478407	151511917	0	0	0	0
GALNTL5	NC_000007	151653511	151717012	0.0261441	0	0	0
GALNT11	NC_000007	151722778	151819427	4.1494106	4.8636065	7.0524999	10.238295
LOC100131012	NC_000007	151826110	151826835	0	0	0	0
LOC731075	NC_000007	151827830	151829646	0.0406928	0.2091824	0.0724029	0.226851
MLL3	NC_000007	151832010	152133090	2.1398113	2.0847282	3.8397325	5.4794958
FABP5L3	NC_000007	152133980	152140101	0	0	0	0
CCT8L1	NC_000007	152142324	152144354	0.0216387	0	0	0.0150787
LOC100128822	NC_000007	152149134	152162634	0	0	0	0
RPS20P19	NC_000007	152287631	152288149	0	0	0	0
RPL36AP28	NC_000007	152299239	152299600	0	0	0	0
XRCC2	NC_000007	152343583	152373250	0.8437832	1.3821151	1.4376912	1.6543214
LOC644333	NC_000007	152446048	152446823	0	0	0	0
ACTR3B	NC_000007	152456851	152552463	0.35974	0.8424372	0.7111881	1.5040868
LOC100288889	NC_000007	152841290	152841673	0	0	0	0
LOC100130228	NC_000007	153421653	153422057	0	0	0.0965372	0.151234
DPP6 (NC_000007 1535	NC_000007	153584419	154264035	0.0296747	0	0	0.0206785
DPP6 (NC_000007 1544	NC_000007	154400205	154685995	0.0225029	0	0.0100096	0.0078405
LOC100132707	NC_000007	154720227	154741200	0	0	0	0
PAXIP1	NC_000007	154735400	154794682	1.2668267	1.3998091	2.7490476	2.8710823
LOC202781	NC_000007	154795141	154797413	0.2410323	0.2891077	0.6611563	0.3079284
LOC100128264	NC_000007	154860253	154875916	0	0	0	0
HTR5A	NC_000007	154862546	154877459	0.0183118	0	0.0162907	0.0127604
LOC100287210	NC_000007	155014613	155015561	0.04631	0	0.0823974	0.096812
LOC100129810	NC_000007	155089434	155094130	0.4512139	0.1855581	0.160565	0.2829814
INSIG1	NC_000007	155089543	155101945	23.994223	21.157307	66.377944	33.065551
LOC100286906	NC_000007	155174771	155188078	0	0	0	0
EN2	NC_000007	155250824	155257526	0.0776699	0.0532352	0.0575811	0.0270617
CNPY1	NC_000007	155293953	155326539	0.2754142	0.6876614	0.6475425	0.1919196

RBM33	NC_000007	155437203	155574179	1.013044	0.9933784	1.6472098	2.5478712
SHH	NC_000007	155595558	155604967	0	0.0286697	0	0.019432
LOC389602	NC_000007	155754953	155757596	0	0	0	0
LOC393076	NC_000007	155897113	156058961	0	0	0	0
C7orf4	NC_000007	156333185	156333795	0	0	0	0
RPL26P23	NC_000007	156426008	156426514	0	0	0	0
C7orf13	NC_000007	156431060	156433348	0	0	0	0
RNF32	NC_000007	156433401	156469824	0.3020497	0.0776347	0.7837427	1.0173213
LMBR1	NC_000007	156473570	156685902	2.1749432	2.0789909	3.7738346	2.8933934
NOM1	NC_000007	156742417	156765876	0.2386526	0.4014981	0.2766489	0.5946578
MNX1	NC_000007	156797547	156803347	0	0.0207644	0	0.0140739
LOC645249	NC_000007	156803208	156809284	0.0312799	0	0.0278274	0
LOC645272	NC_000007	156880703	156881172	0	0	0	0
UBE3C	NC_000007	156931655	157062066	12.214911	15.052413	17.311294	22.769163
RPS27AP12	NC_000007	156985030	156985574	0	0	0	0
LOC777650	NC_000007	156986368	156990953	0	0	0	0
RPL36AP30	NC_000007	157055716	157056095	0	0	0	0
DNAJB6	NC_000007	157129710	157210133	3.3202269	3.4701986	2.365464	4.2721225
LOC393078	NC_000007	157233688	157263477	0	0	0	0
PTPRN2	NC_000007	157331750	158380482	0.0364187	0.1029661	0	0.0317225
LOC100131172	NC_000007	157406721	157408556	0	0	0	0
LOC285872	NC_000007	158414865	158416073	0	0	0	0
NCAPG2	NC_000007	158424003	158497520	6.3262205	8.4115591	8.9490867	14.517999
RPL21P76	NC_000007	158512363	158512894	0	0	0	0
FAM62B	NC_000007	158523689	158622319	6.1086345	5.5521656	7.0555452	11.089116
WDR60	NC_000007	158649269	158738883	0.1514653	0.0958291	0.2073042	0.3166411
LOC154822	NC_000007	158801045	158818929	0	0	0	0
VIPR2	NC_000007	158820866	158937649	0.0222861	0.0114562	0.0297395	0.0232948
LOC100129824	NC_000007	159024125	159026067	0	0	0	0
LOC728836	NC_000008	17102	21265	0.3166299	0.3255288	0.5915338	0.3971526
OR4F21	NC_000008	116086	117024	0	0	0	0
LOC100132317	NC_000008	141884	150543	0	0	0	0
RPL23AP53	NC_000008	158344	182318	0	0	0	0
ZNF596	NC_000008	182200	197339	0.3017041	0.1772477	0.1278116	0.250285
LOC100289630	NC_000008	214245	215571	0.0796164	0.081854	0.0708289	0.0554799
FAM87A	NC_000008	325931	327759	0	0	0	0
LOC100288862	NC_000008	327776	328394	0.4969913	0.0729942	0.5052997	0.3463233
FBXO25	NC_000008	356808	419876	2.285596	1.8872674	3.4422509	4.3642333
C8orf42	NC_000008	441646	495331	0.0233147	0.02397	0	0
LOC389607	NC_000008	607527	609962	0	0	0	0
ERICH1	NC_000008	614200	681226	0.1220784	0.1506113	0.0868835	0.289235
LOC401442	NC_000008	688548	690374	0	0	0	0
LOC100289665	NC_000008	977264	977972	0	0	0	0
LOC100286888	NC_000008	977981	978665	0	0	0	0
DLGAP2	NC_000008	1449569	1656642	0.0043535	0	0.0154919	0.027303
LOC100130321	NC_000008	1678372	1705167	0	0	0	0
CLN8	NC_000008	1711870	1734736	0.8643745	1.3172452	0.4799255	1.0252435
ARHGEF10	NC_000008	1772149	1906807	2.8219308	3.5154319	1.0978926	1.7801977
KBTBD11	NC_000008	1922044	1955109	0.1897423	0.0336336	0.0058207	0.0410338
MYOM2	NC_000008	1993158	2093380	0.1052442	0.0090168	0.023407	0.0427807
LOC100286951	NC_000008	2084136	2104762	0.1636806	0.0420702	0.1456148	0.0285148
CSMD1	NC_000008	2792875	4852328	0.009207	0.0157763	0.0136514	0.0213861
LOC100288956	NC_000008	3567203	3568721	0	0	0	0
LOC780813	NC_000008	4644658	4699427	0	0	0	0
LOC100129861	NC_000008	4860049	4930391	0	0	0	0
LOC648237	NC_000008	4985438	4985832	0	0	0	0
RPL23AP54	NC_000008	5334031	5334491	0	0	0	0
LOC392180	NC_000008	5706219	5707217	0	0	0	0
LOC100287015	NC_000008	6262436	6264116	1.2318823	1.1980447	0.325813	0.5104146
MCPH1	NC_000008	6264121	6506026	1.0013106	1.102583	0.7058201	0.7473202
ANGPT2	NC_000008	6357172	6420784	0	0.0085737	0.0148378	0.0058112
AGPAT5	NC_000008	6565878	6619024	0.1193813	0.1554663	0.1345262	0.9428159
XKR5	NC_000008	6666038	6693037	0.3412559	0.0284471	0.0082052	0.006427
DEFB1	NC_000008	6728097	6735529	0	0	0	0.1322889
LOC392181	NC_000008	6756400	6756990	0	0	0	0
DEFA6	NC_000008	6782219	6783598	0	0.0971686	0	0

DEFA4	NC_000008	6793345	6795786	0	0	0	0
DEFA8P	NC_000008	6807766	6812345	0	0	0	0
DEFA9P	NC_000008	6816811	6817683	0	0	0	0
DEFA10P	NC_000008	6826451	6826635	0	0	0	0
DEFA1	NC_000008	6835171	6837602	0	0	0	0
DEFT1P	NC_000008	6844712	6847248	0	0	0	0
LOC728358	NC_000008	6854288	6856724	0	0	0	0
LOC100287083	NC_000008	6864031	6867111	0	0	0	0
DEFA3	NC_000008	6873395	6875816	0	0	0	0
DEFA11P	NC_000008	6886740	6887053	0	0	0	0
DEFA7P	NC_000008	6896789	6896961	0	0	0	0
DEFA5	NC_000008	6912829	6914259	0	0	0	0
LOC648665	NC_000008	6940493	6949908	0	0	0	0
LOC100129712	NC_000008	6970882	6971250	0	0	0	0
RPS3AP30	NC_000008	6980370	6981218	0	0	0	0
RPS3AP33	NC_000008	7048261	7049109	0	0	0	0
LOC100131970	NC_000008	7079187	7079835	0	0	0	0
LOC441320	NC_000008	7095808	7096178	0	0	0	0
OR7E125P	NC_000008	7104141	7105109	0	0	0	0
LOC729261	NC_000008	7111351	7113374	0.0868542	0.0892953	0.038634	0.0453926
FAM90A15	NC_000008	7112766	7117995	0	0	0.0164691	0
LOC349196	NC_000008	7118141	7121014	0	0	0	0
FAM90A3	NC_000008	7120389	7125617	0	0	0	0
LOC729270	NC_000008	7125763	7130623	0.027123	0.0092951	0.0402156	0.0063001
FAM90A4P	NC_000008	7129733	7133235	0	0	0	0
LOC729273	NC_000008	7134484	7134756	0	0	0	0
FAM90A13	NC_000008	7135632	7140861	0	0	0	0
LOC729278	NC_000008	7141007	7145867	0.0361639	0.0092951	0.0160862	0.0189004
FAM90A5	NC_000008	7143254	7148483	0.0185045	0	0	0
LOC729284	NC_000008	7149728	7150000	0	0.165507	0	0
FAM90A20	NC_000008	7152536	7156106	0	0	0	0.0626596
DEF109P1B	NC_000008	7170368	7177473	0	0	0	0
LOC401447	NC_000008	7189909	7191501	0	0	0	0
USP17L4	NC_000008	7194637	7196229	0	0	0.0245434	0
LOC402329	NC_000008	7199348	7200548	0	0	0	0
LOC100289400	NC_000008	7212030	7212875	0	0	0	0
ZNF705G	NC_000008	7215498	7243080	0	0	0.0659875	0
DEFB108P2	NC_000008	7230666	7235022	0	0	0	0
HSPD1P3	NC_000008	7248860	7278554	0	0	0	0
LOC100289462	NC_000008	7272385	7274349	0	0	0	0
DEFB103A	NC_000008	7286410	7287682	0	0.1369194	0	0.0928027
SPAG11B	NC_000008	7305276	7321192	0.032651	0.0335686	0	0
DEFB104B	NC_000008	7327830	7332604	0	0	0	0
DEFB106B	NC_000008	7340026	7343909	0	0	0	0
DEFB105B	NC_000008	7345243	7347073	0	0	0	0
DEFB107B	NC_000008	7353368	7366833	0	0	0	0
LOC100131608	NC_000008	7397150	7404344	0	0	0	0
LOC645489	NC_000008	7404294	7405872	0.027833	0	0.024761	0.0193951
FAM90A6P	NC_000008	7406020	7409583	0	0	0	0
LOC100132648	NC_000008	7412145	7412417	0	0	0.1432145	0
FAM90A7	NC_000008	7413660	7417234	0	0	0	0
LOC100132106	NC_000008	7419795	7420067	0.1609825	0	0	0
FAM90A21P	NC_000008	7421875	7424882	0	0	0	0
LOC729339	NC_000008	7426966	7428812	0	0	0.0211681	0
FAM90A22	NC_000008	7428960	7432527	0	0	0	0
LOC100132485	NC_000008	7434675	7436461	0	0	0.0218789	0
FAM90A23	NC_000008	7436609	7440174	0	0	0	0
LOC729346	NC_000008	7442319	7443273	0	0	0	0
OR7E157P	NC_000008	7449409	7450577	0	0	0	0
LOC100133084	NC_000008	7468611	7469314	0	0	0	0
LOC100132212	NC_000008	7537783	7538431	0	0	0	0
OR7E154P	NC_000008	7562586	7563754	0	0	0	0
LOC728731	NC_000008	7562593	7564613	0	0	0	0
FAM90A14	NC_000008	7571306	7576534	0	0	0	0
LOC729372	NC_000008	7576680	7578526	0.0475888	0.0489263	0	0.0331618
FAM90A18	NC_000008	7578952	7584185	0	0	0	0

LOC100132048	NC_000008	7584328	7585895	0.0280282	0	0	0
FAM90A16	NC_000008	7586600	7591833	0	0	0	0
LOC729379	NC_000008	7591976	7593543	0	0	0	0
FAM90A8	NC_000008	7594248	7599480	0	0	0	0
LOC729383	NC_000008	7599623	7601190	0	0	0	0
FAM90A17	NC_000008	7601895	7607128	0	0	0	0
LOC729387	NC_000008	7607271	7608838	0	0	0	0
FAM90A19	NC_000008	7609543	7614776	0	0	0	0
LOC729394	NC_000008	7614919	7616486	0	0	0	0
FAM90A9	NC_000008	7617191	7622424	0	0	0	0
LOC100132221	NC_000008	7622567	7624414	0.047563	0.0488998	0.0211567	0
FAM90A10	NC_000008	7624840	7630072	0	0	0	0
LOC100133099	NC_000008	7630215	7632070	0.047358	0.1217225	0.0631965	0.0495014
LOC100133251	NC_000008	7633768	7638935	0	0.0507109	0	0
DEFB107A	NC_000008	7669242	7673238	0	0	0	0
DEFB105A	NC_000008	7679530	7681360	0	0	0	0
DEFB106A	NC_000008	7682694	7686575	0	0	0	0
DEFB104A	NC_000008	7693993	7698764	0	0	0	0
SPAG11A	NC_000008	7705402	7721319	0	0	0	0
DEFB103B	NC_000008	7738726	7740105	0	0	0	0
HSPD1P2	NC_000008	7748319	7750279	0	0	0	0
DEFB4	NC_000008	7752199	7754237	0	0	0	0
LOC100132396	NC_000008	7783859	7812375	0	0	0	0.0251918
DEFB108P1	NC_000008	7791934	7796292	0	0	0	0
FAM66E	NC_000008	7812537	7819131	0	0	0	0
LOC392187	NC_000008	7824866	7826066	0	0	0	0
USP17L8	NC_000008	7829183	7830775	0	0	0	0
USP17L3	NC_000008	7833915	7835507	0.082765	0	0.0245434	0.0384493
LOC100286963	NC_000008	7847937	7855043	0	0	0	0
FAM90A11	NC_000008	7869312	7872873	0	0	0	0
LOC729456	NC_000008	7875434	7875706	0	0	0	0
FAM90A24P	NC_000008	7876953	7880519	0	0	0	0
LOC729459	NC_000008	7879567	7884453	0.0179858	0.0647194	0.0160006	0.0125332
FAM90A12	NC_000008	7884599	7889826	0	0	0	0
LOC729462	NC_000008	7890311	7891265	0	0	0	0
OR7E96P	NC_000008	7897501	7898469	0	0	0	0
LOC100132046	NC_000008	7922740	7923386	0	0	0	0
RPS3AP31	NC_000008	7953444	7954292	0	0	0	0
LRLE1	NC_000008	8046158	8046331	0	0	0	0.176005
FLJ10661	NC_000008	8086092	8102387	0	0	0	0
PRAGMIN	NC_000008	8175258	8239257	0.3324375	0.60544	0.1520977	0.2647493
CLDN23	NC_000008	8559666	8561617	50.815143	68.168606	53.578887	110.21505
MFHAS1	NC_000008	8641999	8751131	0.8279496	1.2811279	1.5698547	2.0688548
MRPS18CP2	NC_000008	8791228	8791520	0	0	0	0
LOC645960	NC_000008	8812999	8852958	0.1290697	0.1326972	0.057412	0.3147932
ERI1	NC_000008	8860314	8890849	3.5710914	5.2379458	3.9309362	4.101013
LOC100128472	NC_000008	8923497	8923885	0	0	0	0
RNU7-55P	NC_000008	8929966	8930024	0	0	0	0
PPP1R3B	NC_000008	8993765	9008220	0.4819925	1.2510327	0.6467055	0.9305294
LOC100287634	NC_000008	9090523	9090961	0	0	0	0
TNKS	NC_000008	9413445	9639856	2.9668147	3.530321	3.0914733	3.6051789
LOC157627	NC_000008	9757574	9760839	0	0	0	0
MSRA	NC_000008	9911830	10286401	1.827462	2.0621228	1.3481919	2.3294786
LOC346702	NC_000008	10353836	10405089	0	0	0	0
UNQ9391	NC_000008	10383081	10396328	0	0	0	0
RP1L1	NC_000008	10463859	10512617	0.0055087	0.0056635	0.0049007	0.0422253
C8orf74	NC_000008	10530147	10558103	0.0424211	0	0	0.0591214
SOX7	NC_000008	10581278	10588022	0.027331	0.0280991	0.0364716	0.0095227
PINX1	NC_000008	10622884	10697299	1.2592617	2.2009107	0.560137	1.0530044
XKR6	NC_000008	10753654	11058875	0.1519083	0.0600683	0.020791	0.1709977
RPL17P29	NC_000008	11032519	11033125	0	0	0	0
RPL19P13	NC_000008	11113499	11114187	0	0	0	0
MTMR9	NC_000008	11142000	11185655	1.0978157	1.348303	0.5595925	0.7360557
AMAC1L2	NC_000008	11188495	11189695	0	0	0	0
TDH	NC_000008	11197146	11225961	0	0	0	0
C8orf12	NC_000008	11225911	11296166	0	0	0	0

FAM167A	NC_000008	11278973	11324276	0.1610612	6.4910433	1.8149371	2.0501384
BLK	NC_000008	11351521	11422108	0.0509053	0	0.0301912	0.0591214
GATA4	NC_000008	11561717	11617509	0	0.0397741	0	0.0179723
C8orf49	NC_000008	11618765	11620732	0	0	0	0
NEIL2	NC_000008	11627172	11644854	1.2940046	2.2829854	1.2222431	2.2821156
SUB1P1	NC_000008	11647461	11648594	0	0	0	0
FDFT1	NC_000008	11660190	11696818	33.931541	41.72921	32.822648	26.131484
CTSB	NC_000008	11700033	11725646	332.05698	125.78878	425.93809	248.24572
OR7E158P	NC_000008	11777405	11778304	0	0	0	0
OR7E161P	NC_000008	11786077	11787072	0	0	0	0
TRNAE35P	NC_000008	11793380	11793451	0	0	0	0
DEFB137	NC_000008	11831446	11832108	0	0	0	0
DEFB136	NC_000008	11839830	11842099	0	0	0	0
DEFB134	NC_000008	11851489	11853760	0	0	0	0
OR7E160P	NC_000008	11855115	11892149	0	0	0	0
LOC100128174	NC_000008	11901012	11907134	0	0.2121286	0.1835566	0
LOC100133267	NC_000008	11921898	11929256	0	0	0	0
ZNF705D	NC_000008	11946847	11973025	0.0119327	0	0.0106157	0.0083152
DEFB108P3	NC_000008	11952685	11957044	0	0	0	0
FAM66D	NC_000008	11973291	12037657	0	0	0	0
LOC392196	NC_000008	11985367	11986804	0	0	0	0
USP17L7	NC_000008	11989926	11991518	0	0	0.0245434	0.0192247
DUB3	NC_000008	11994677	11996269	0.0551767	0	0	0
LOC100287392	NC_000008	12008397	12015542	0	0	0	0
LOC100287352	NC_000008	12028014	12028792	0.2256649	0.6960216	0.1003789	0.3538176
FAM90A2P	NC_000008	12029708	12032663	0	0	0	0
FAM86B1	NC_000008	12039613	12051624	2.343425	1.1675777	1.0423879	0.8793033
LOC100287665	NC_000008	12063546	12083526	0.258519	0.0885949	0.1533238	0.7806342
LOC100287066	NC_000008	12147577	12153698	0	0	0	0
DEFB130	NC_000008	12168471	12175825	0	0	0	0
ZNF705CP	NC_000008	12192994	12219362	0	0	0	0
DEFB108P4	NC_000008	12198933	12203287	0	0	0	0
FAM66A	NC_000008	12219528	12268510	0	0	0	0
DEFB109P1	NC_000008	12250793	12257874	0	0	0	0
LOC100288947	NC_000008	12269405	12270550	0.3067939	0.8279681	0.1705827	0.2672328
LOC100289516	NC_000008	12270515	12270796	1.0909136	0.8011241	0.4159315	0.3257966
FAM90A25P	NC_000008	12272031	12278402	0	0	0	0
FAM86B2	NC_000008	12283123	12293852	0.7625088	0.5701376	0.1541702	0.1449127
LOC646344	NC_000008	12305800	12326036	0.172346	0	0.1533238	0.1200976
LOC100127885	NC_000008	12334357	12334530	0	0	0	0
RPS3AP34	NC_000008	12427815	12428663	0	0	0	0
LOC729732	NC_000008	12452479	12523119	0	0	0	0
RPS3AP35	NC_000008	12485941	12486789	0	0	0	0
OR7E8P	NC_000008	12541649	12542674	0	0	0	0
OR7E15P	NC_000008	12553879	12554534	0	0	0	0
OR7E10P	NC_000008	12560561	12561547	0	0	0	0
LONRF1	NC_000008	12579406	12612992	0.7725275	0.9581302	1.2763435	0.8288541
LOC340357	NC_000008	12623571	12668910	0	0	0	0
C8orf79	NC_000008	12803183	12887286	0.0790278	0.130901	0.0039059	0.0122377
DLC1	NC_000008	12940872	13372395	5.2393702	5.1048961	2.730345	3.7197445
C8orf48	NC_000008	13424352	13425797	2.1882937	2.062313	1.1896908	1.4613531
SGCZ	NC_000008	13947373	15095792	0.0403565	0.0207454	0	0.042183
LOC100131565	NC_000008	14166535	14167837	0	0	0	0
TUSC3	NC_000008	15397730	15621995	32.73271	32.456886	26.163588	23.2147
LOC137012	NC_000008	15663434	15664812	0	0	0	0
RPL32P19	NC_000008	15800577	15801041	0	0	0	0
MSR1	NC_000008	15965387	16050300	0.0720463	0.0411506	0.0142432	0.0055783
LOC646440	NC_000008	16133207	16133810	0	0	0	0
MRPL49P2	NC_000008	16232128	16232595	0	0	0	0
FGF20	NC_000008	16850334	16859674	0.0432561	0.0444719	0.0384819	0.0602852
EFHA2	NC_000008	16884748	16980143	0.6182118	0.6469364	0.2848102	0.3000176
RPL9P20	NC_000008	17007712	17008418	0	0	0	0
ZDHHC2	NC_000008	17013836	17080241	7.0216392	7.1401488	7.3965734	9.3584498
CNOT7	NC_000008	17086740	17104387	9.9253872	14.418074	8.5553381	10.76172
LOC100289616	NC_000008	17102249	17104373	0.0627832	0	0.1117073	0.0437498
VPS37A	NC_000008	17104401	17155533	5.4348195	6.346371	4.4427114	5.0495983

LOC100289654	NC_000008	17104440	17132442	0.2520931	0.2591782	0.3737817	0.4098932
MTMR7	NC_000008	17155539	17270836	0.0684908	0.0821516	0.192949	0.3340896
LOC646479	NC_000008	17289331	17329037	0	0	0	0
SLC7A2	NC_000008	17396286	17428077	0.0114092	0.0175948	0.0659746	0.0119256
PDGFRL	NC_000008	17434701	17500623	5.9104812	7.8514433	7.9913133	1.3049216
MTUS1	NC_000008	17501303	17658426	0.088964	0.3780528	0.0738685	0.0413291
FGL1	NC_000008	17721900	17753047	0.0282444	0.0290382	0	0.0196818
PCM1	NC_000008	17780366	17887457	1.5861994	1.9137226	1.4333845	2.8626921
ASAH1	NC_000008	17913925	17942507	16.647507	10.418725	12.526533	10.256543
LOC100286907	NC_000008	17940957	17942453	0	0	0	0
MRPS18CP3	NC_000008	17944382	17944610	0	0	0	0
LOC100133073	NC_000008	17945901	17951417	0	0	0	0
NAT1	NC_000008	18067615	18080820	1.5431261	0.9201676	0.9335093	1.2903741
TRNAK41P	NC_000008	18120203	18120272	0	0	0	0
AACP	NC_000008	18227219	18229587	0	0	0	0
RPL10AP11	NC_000008	18240753	18241458	0	0	0	0
LOC100286975	NC_000008	18244442	18245297	0	0	0	0
NAT2	NC_000008	18248755	18258723	0.03337	0	0	0
PSD3	NC_000008	18384813	18871196	3.370058	1.5012747	1.7981971	4.5129492
NSAP11	NC_000008	18590990	18591289	0	0	0	0
RPL35P6	NC_000008	18632057	18632476	0	0	0	0
LOC100128993	NC_000008	19041184	19042214	0.2707283	0.3711162	0	0.0628848
LOC442382	NC_000008	19095947	19098383	0	0	0	0
SH2D4A	NC_000008	19171207	19253316	6.1312393	4.992292	1.7224802	7.3563956
CSGALNACT1	NC_000008	19261672	19540261	1.0056361	0.4175364	2.4860718	3.0186899
INTS10	NC_000008	19674918	19709589	1.2472498	1.5850701	1.386985	1.7744805
LPL	NC_000008	19796582	19824770	0.0234578	0.0120586	0.0313031	0.0326927
RPL30P9	NC_000008	19970847	19971168	0	0	0	0
SLC18A1	NC_000008	20002366	20040717	0	0	0	0.0103567
ATP6V1B2	NC_000008	20054704	20079207	36.801591	26.243185	19.754289	28.019148
LZTS1	NC_000008	20103676	20112803	1.2236914	0.4221201	0.2506713	0.2243992
LOC724059	NC_000008	20790128	20792327	0	0	0	0
GFRA2	NC_000008	21549530	21646346	0.1319767	0	0.011741	0
OR6R2P	NC_000008	21654598	21655408	0	0	0	0
DOK2	NC_000008	21766384	21771205	0.0248295	0.0255273	0.044178	0
XPO7	NC_000008	21777180	21864096	5.0974993	7.7701621	4.9127831	6.900773
NPM2	NC_000008	21882354	21894408	0.0799059	0	0.0355432	0.0278408
FGF17	NC_000008	21900428	21906320	0.0359348	0.0369447	0	0
EPB49	NC_000008	21911081	21940036	0.1472975	0.0963692	0.607549	0.4199024
FAM160B2	NC_000008	21946714	21961891	6.6887984	7.4216038	2.828602	4.1030116
NUDT18	NC_000008	21964383	21966932	2.9510992	2.2904029	1.3899069	1.4919296
HR	NC_000008	21971932	21988565	0.1593482	0.4750974	0.2055528	0.0555201
REEP4	NC_000008	21995533	21999448	6.900962	8.1618188	3.1850437	4.7727083
LGI3	NC_000008	22004343	22014344	0.0269126	0.0138345	0.0239422	0
SFTPC	NC_000008	22019184	22021992	0.0446628	0.0459181	0	0.0311228
BMP1	NC_000008	22022675	22069839	2.6558923	3.2088786	1.6728998	2.1749472
PHYHIP	NC_000008	22077216	22089851	0.2502297	0.2031019	0.0585819	0.2202568
POLR3D	NC_000008	22102619	22108680	4.1464895	6.5585031	1.7836111	3.5086045
PIWIL2	NC_000008	22132810	22213584	0.0428868	0.0330691	0.0763065	0.097127
SLC39A14	NC_000008	22224762	22291642	19.387951	25.29316	17.808491	11.326931
RPL21P77	NC_000008	22247378	22247799	0	0	0	0
PPP3CC	NC_000008	22298596	22398638	3.6740641	2.2705227	2.6791388	2.2244658
LOC652964	NC_000008	22350013	22350882	0	0	0	0
SORBS3	NC_000008	22409251	22433008	14.711881	13.749077	4.168184	11.091374
PDLIM2	NC_000008	22436643	22455538	3.7245625	4.3409077	1.4210805	1.6948563
C8orf58	NC_000008	22457122	22461663	6.376131	5.6974202	1.0469163	2.6241376
LOC100287037	NC_000008	22460646	22462841	0.2109853	0.173532	0.0563095	0.0441069
KIAA1967	NC_000008	22462257	22477984	7.0786929	11.464776	5.0896806	6.7271123
BIN3	NC_000008	22477931	22526661	2.9723442	2.9103641	1.1332628	2.9589254
LOC100288890	NC_000008	22497869	22499722	0	0	0	0
EGR3	NC_000008	22545174	22550815	0.3244909	0.3440361	0.0811901	0.0635957
PEBP4	NC_000008	22570765	22785421	0	0.0505407	0	0
RHOBTB2	NC_000008	22857462	22877708	4.3022004	4.8413966	1.4554737	2.5093459
TNFRSF10B	NC_000008	22877646	22926700	4.7397226	3.1652311	4.8754307	3.1627522
TNFRSF10C	NC_000008	22960434	22974950	0.5703448	0.3257635	0.1127543	0.0441599
TNFRSF10D	NC_000008	22993101	23021540	1.8151179	1.6360609	3.0415362	3.6299361

TNFRSF10A	NC_000008	23048970	23082639	3.9087953	1.6920642	2.0818481	1.3798219
RPL23AP55	NC_000008	23064431	23064882	0	0	0	0
LOC100288918	NC_000008	23069430	23082680	0.0620738	0.5105469	0.0552226	0
CHMP7	NC_000008	23101150	23119512	1.4950132	1.5502809	1.1694873	1.9219132
R3HCC1	NC_000008	23145612	23153792	1.0000862	1.7707781	0.7414203	1.3163664
LOXL2	NC_000008	23154410	23261722	29.471846	34.47458	14.458916	17.394288
ENTPD4	NC_000008	23289572	23315244	2.1606656	3.9592145	2.1314058	4.5066711
SLC25A37	NC_000008	23386363	23430063	3.9117461	2.5438376	3.2493955	2.7258605
LOC646721	NC_000008	23490226	23509871	0	0	0	0
NKX3-1	NC_000008	23536206	23540450	0.4554221	0.5370779	0.1906617	0.2520182
NKX2-6	NC_000008	23559964	23563922	0.0612946	0.3781038	0	0
LOC100288985	NC_000008	23699429	23709041	0.1448698	0.0947809	0.152313	0.0734189
STC1	NC_000008	23699434	23712320	4.4775733	3.2631809	16.306619	18.578724
LOC100132107	NC_000008	23744141	23746542	0.0954359	0	0	0.0997553
ADAM28	NC_000008	24151580	24212726	0.0358468	0.0122848	0.0637807	0.099918
ADAMDEC1	NC_000008	24241798	24263526	0.1081581	0.018533	0.0481102	0.0502459
ADAM7	NC_000008	24298509	24366281	0.0169032	0.0347565	0	0.0117788
NEFM	NC_000008	24771274	24776606	0.2665732	0.0498301	0.1185755	0.2701947
LOC100289018	NC_000008	24771518	24773186	0.0463589	0	0.0824843	0.1615236
LOC100129717	NC_000008	24799795	24814069	0	0	0	0
NEFL	NC_000008	24808468	24814131	0.3673577	0.7301859	0.0544686	0
DOCK5	NC_000008	25042287	25270619	5.8054713	5.4124393	2.1631744	4.4184139
GNRH1	NC_000008	25276774	25282556	0.2861745	0.3362486	0.2545888	0.6409858
KCTD9	NC_000008	25285363	25315920	5.0425922	5.2519952	2.3074357	4.6607065
CDCA2	NC_000008	25316513	25365425	1.7839202	2.5506759	1.1140704	1.9757986
EBF2	NC_000008	25701573	25902392	0.6122522	0.8851777	0.238296	0.3866441
LOC100129404	NC_000008	26112480	26114150	0	0	0	0
PPP2R2A	NC_000008	26149034	26228646	6.5466685	6.8262694	5.9895551	7.4132164
SDAD1P1	NC_000008	26236646	26239154	0	0	0	0
LOC100287240	NC_000008	26240499	26248944	0.1003384	0.2063169	0.0892638	0
BNIP3L	NC_000008	26240523	26270644	9.944857	8.5461177	6.6354154	9.4849623
LOC650531	NC_000008	26290512	26301638	0	0	0	0
LOC100289047	NC_000008	26304765	26306770	0.0219084	0	0.1169419	0.1373998
PNMA2	NC_000008	26362196	26371483	6.2122449	4.8111092	1.8556418	1.8137309
PSME2P5	NC_000008	26405185	26405965	0	0	0	0
DPYSL2	NC_000008	26435421	26515693	48.827066	50.139643	25.060334	29.653517
ADRA1A	NC_000008	26605667	26722922	0	0	0.0109825	0.06882
RPLP1P9	NC_000008	26629313	26629646	0	0	0	0
LOC100132229	NC_000008	26868549	26869888	0	0	0	0
STMN4	NC_000008	27093814	27115903	0	0.0343862	0	0
TRIM35	NC_000008	27142404	27168834	3.3998277	2.7792014	1.5261647	1.977902
PTK2B	NC_000008	27168999	27316903	0.4448577	0.4097187	0.1401642	0.1033315
CHRNA2	NC_000008	27317278	27336813	0.0108061	0.0111098	0.0096134	0.0301204
EPHX2	NC_000008	27348645	27402486	1.2608099	0.5349583	0.071216	0.0278915
GULOP	NC_000008	27435128	27446590	0	0	0	0
CLU	NC_000008	27454451	27472327	1.3531207	3.217035	2.068986	1.1197037
SCARA3	NC_000008	27491577	27534286	16.985212	31.706202	6.6420969	8.3435211
CCDC25	NC_000008	27590833	27630170	1.7627587	2.3336482	1.8152441	2.7259507
ESCO2	NC_000008	27632058	27662425	2.5905775	5.1384518	2.2697307	2.6804746
PBK	NC_000008	27667138	27695349	8.91291	13.01399	7.4441428	8.4738741
LOC100287332	NC_000008	27672964	27695597	0.2803715	0.4323771	0.0623566	0.1465305
LOC100130612	NC_000008	27696033	27697935	0	0	0	0
SCARA5	NC_000008	27727736	27850198	0	0.1116252	0	0.016813
C8orf80	NC_000008	27879481	27941388	0	0.0116242	0.0301756	0.0078788
ELP3	NC_000008	27950584	28048670	1.2693123	1.8241746	0.6920998	0.9130398
LOC100131127	NC_000008	28082897	28085124	0	0	0	0
LOC100130891	NC_000008	28095947	28096523	0	0	0	0
LOC100129848	NC_000008	28107606	28197099	0	0	0	0.0257785
RPL5P22	NC_000008	28157125	28158148	0	0	0	0
PNOC	NC_000008	28174649	28200868	0.036746	0	0.0326903	0.0256061
ZNF395	NC_000008	28203102	28243977	6.9372677	1.6748533	5.3003988	9.0305764
FBXO16	NC_000008	28285929	28347784	0.3741723	0.0699433	0.4841804	0.1659243
FZD3	NC_000008	28351773	28421982	0.1340907	0.0229766	0.2485225	0.1245863
EXTL3	NC_000008	28559153	28611202	3.9732847	7.6501385	3.1293256	3.4237145
LOC100130764	NC_000008	28615186	28617781	0	0	0	0.0117969
INTS9	NC_000008	28625178	28747698	2.0533582	2.0292437	1.2319769	2.2516662

RPL36AP32	NC_000008	28667620	28667931	0	0	0	0
HMBOX1	NC_000008	28747911	28910242	0.5621576	0.8219834	0.866859	1.4363573
KIF13B	NC_000008	28924795	29120610	0.4462004	0.262874	0.517376	0.6812516
DUSP4	NC_000008	29193611	29208185	0.2911167	1.7530348	0.0462474	0.0434704
LOC100132051	NC_000008	29209936	29210681	0	0	0	0
RPL17P33	NC_000008	29489572	29490190	0	0	0	0
C8orf75	NC_000008	29578775	29605625	0	0	0	0
LOC286135	NC_000008	29779029	29811125	0	0	0	0
LOC100289207	NC_000008	29885229	29886416	0	0	0	0
MAP2K1P1	NC_000008	29886429	29886618	0	0	0	0
TMEM66	NC_000008	29920631	29940649	15.271844	21.917492	30.910215	28.502669
LOC100289246	NC_000008	29924353	29940979	0.4304813	0.7242219	0.5570446	0.1090824
LEPROTL1	NC_000008	29952922	29995222	2.4718262	2.3690058	2.6338366	2.8707782
RPS15AP24	NC_000008	29974856	29975325	0	0	0	0
MBOAT4	NC_000008	29989187	30002200	0	0	0.0235103	0.0552463
DCTN6	NC_000008	30013813	30041060	3.4101441	3.4183369	7.3947869	4.3961998
LOC392209	NC_000008	30094898	30098513	0	0	0	0
LOC642319	NC_000008	30107018	30135321	0	0	0	0
LOC100128441	NC_000008	30189477	30189793	0	0	0	0
TUBBP1	NC_000008	30209396	30211038	0	0	0	0
LOC100128750	NC_000008	30240646	30248015	0	0	0	0
RBPMS	NC_000008	30241944	30429734	1.7381373	1.5000263	2.9345748	2.5815429
GTF2E2	NC_000008	30436031	30515738	5.9513231	7.0304903	16.239744	13.458198
GSR	NC_000008	30536435	30585443	8.1407202	8.2896162	11.165795	14.757346
UBXN8	NC_000008	30601690	30624522	4.3917733	3.8567373	4.531085	5.376887
PPP2CB	NC_000008	30643126	30670352	11.645166	14.220157	24.431017	22.820975
TEX15	NC_000008	30689060	30706533	0.2694611	0.0804293	0.7732905	1.3113699
LOC100129398	NC_000008	30840972	30852564	0	0	0	0
PURG	NC_000008	30853321	30891231	0.0699813	0.0959308	0.1245145	0.065021
WRN	NC_000008	30890778	31031277	0.6883642	0.920024	1.3336448	1.4230495
LOC642513	NC_000008	31075712	31078507	0	0	0	0
NRG1	NC_000008	31497268	32622073	2.8374241	3.1354124	1.2967475	1.8342384
LOC100287675	NC_000008	32871599	32872426	0	0	0	0
TRNAQ43P	NC_000008	32872646	32872717	0	0	0	0
FUT10	NC_000008	33228342	33330664	0.4705643	0.9930418	0.8482707	0.9750947
MAK16	NC_000008	33342685	33358778	0.6271292	1.0366645	0.7876398	0.9854116
C8orf41	NC_000008	33356233	33370703	1.4953055	1.8553998	2.8593057	2.467237
SNORD13	NC_000008	33370993	33371096	0	0	0	0
LOC100289349	NC_000008	33380684	33397915	0.1842693	0	0.8196555	0.1284062
RNF122	NC_000008	33405273	33424643	0.6116992	0.8465841	0.8162768	0.9508795
DUSP26	NC_000008	33448851	33457439	0	0.0822015	0.0237099	0
VENTXP5	NC_000008	33578599	33580707	0	0	0	0
RPL6P22	NC_000008	33716866	33717766	0	0	0	0
CYCSP3	NC_000008	33827079	33827396	0	0	0	0
RPL10AP3	NC_000008	34180478	34181193	0	0	0	0
RPL21P80	NC_000008	34731638	34732198	0	0	0	0
UNC5D	NC_000008	35092975	35652181	0.0120571	0.006198	0.0107264	0.0126028
LSM12P	NC_000008	35381017	35383392	0	0	0	0
LOC100287706	NC_000008	36135269	36152356	0	0	0	0
RPL23P10	NC_000008	36438715	36439106	0	0	0	0
KCNU1	NC_000008	36641842	36793643	0.0117446	0	0.0208966	0.0572887
MRPS7P1	NC_000008	36652781	36653485	0	0	0	0
FKSG2	NC_000008	36745997	36746828	0	0	0	0
RPL26P25	NC_000008	36845448	36846016	0	0	0	0
LOC642879	NC_000008	36941297	36953922	0	0	0	0
ZNF703	NC_000008	37553301	37556396	2.2236916	2.6602923	1.9243052	2.479291
ERLIN2	NC_000008	37594097	37615319	2.3790662	2.6743152	2.2567321	3.5703225
LOC728024	NC_000008	37604853	37605521	0	0	0	0
PROSC	NC_000008	37620101	37637286	2.6344989	2.7437177	5.9049651	6.5326716
GPR124	NC_000008	37654424	37701492	10.629919	12.992393	13.927571	3.5683931
BRF2	NC_000008	37701400	37707411	3.3327779	2.6954709	3.7951125	3.6229634
RAB11FIP1	NC_000008	37716470	37757003	0.2380668	0.0910726	1.3692521	1.4313215
GOT1L1	NC_000008	37791799	37797647	0	0	0.0283727	0.0222241
ADRB3	NC_000008	37820513	37824184	0.0996182	0.0341393	0.0590821	0.034709
EIF4EBP1	NC_000008	37888020	37917883	35.81346	42.185202	52.388007	49.662928
ASH2L	NC_000008	37963011	37997228	6.6689505	7.4654912	9.5142365	13.295834

STAR	NC_000008	38000218	38008600	0.0815366	0.1005938	0.0145074	0.0681815
LSM1	NC_000008	38020857	38034026	3.3402565	3.4831939	3.0989384	1.8288472
BAG4	NC_000008	38034106	38068537	5.7201182	9.7324468	7.4360631	6.4281367
DDHD2	NC_000008	38089107	38118986	4.5408747	4.941508	8.8353411	8.6045731
PPAPDC1B	NC_000008	38120648	38126738	2.9098144	3.1291397	3.4961674	2.4355402
WHSC1L1	NC_000008	38132560	38239790	2.0251206	2.639382	6.5412062	6.2005283
LETM2	NC_000008	38244020	38266061	1.0239318	1.412847	1.0068043	1.1078282
FGFR1	NC_000008	38268656	38326352	16.069559	11.612586	19.435848	16.104276
RPS20P22	NC_000008	38291866	38292369	0	0	0	0
C8orf86	NC_000008	38368352	38386180	0.0195325	0.0602445	0.0347534	0
RNF5P1	NC_000008	38457691	38458775	0	0	0	0
LOC100288608	NC_000008	38568511	38602817	0.0581326	0.0298832	0.2585818	0.0405091
TACC1	NC_000008	38644722	38710546	1.9489987	1.789499	2.3803312	3.9017084
PLEKHA2	NC_000008	38758753	38831430	6.653393	4.3092839	10.264757	12.155487
HTRA4	NC_000008	38831668	38846181	0.0208285	0.0856557	0.0370593	0.1161133
TM2D2	NC_000008	38846327	38854041	3.645253	4.268907	1.922127	3.9027585
LOC100289384	NC_000008	38852196	38854189	0	0.0380652	0.0988144	0
ADAM9	NC_000008	38854505	38962780	10.796748	13.249683	22.415047	26.171981
ADAM32	NC_000008	38965050	39142436	0.0966958	0.1491201	0.1003605	0.1684537
RPL3P10	NC_000008	39015001	39016238	0	0	0	0
ADAM5P	NC_000008	39172182	39274745	0	0	0	0
ADAM3A	NC_000008	39308564	39380470	0	0	0	0
ADAM18	NC_000008	39442142	39587487	0.0195499	0	0.0695686	0.0136232
ADAM2	NC_000008	39601254	39695779	0.0498845	0.0512865	0.0147929	0
IDO1	NC_000008	39771328	39785950	3.1032148	1.293418	12.659454	1.3052588
IDO2	NC_000008	39792474	39873910	0.0383158	0.0393927	0.0170434	0.01335
C8orf4	NC_000008	40010989	40012821	0.0719284	0.02465	0.1279789	0.0167075
ZMAT4	NC_000008	40388109	40755343	0.0356	0.2013031	0	0
SFRP1	NC_000008	41119478	41166980	0.1677037	5.7303304	0.0877611	0.0893655
RPS29P2	NC_000008	41128923	41129178	0	0	0	0
GOLGA7	NC_000008	41348081	41368499	8.3550167	12.581072	18.13166	13.761347
KRT18P37	NC_000008	41368742	41370109	0	0	0	0
GINS4	NC_000008	41386725	41402565	2.5744213	4.8700673	2.4327827	6.960562
AGPAT6	NC_000008	41435707	41482520	2.5909259	3.4156074	3.1352406	4.8728016
NKX6-3	NC_000008	41503829	41504878	0	0	0	0
ANK1	NC_000008	41510744	41754280	0.7102936	0.2872342	0.2948852	0.6236507
MYST3	NC_000008	41786997	41909505	1.2969107	1.6545349	4.6698116	5.7423708
RPL17P30	NC_000008	41958836	41959358	0	0	0	0
LOC100129729	NC_000008	41986292	41997888	0	0	0	0
AP3M2	NC_000008	42010464	42028701	2.2872241	2.9178382	4.3571946	3.8218513
PLAT	NC_000008	42032236	42065194	10.429568	15.706679	3.0804889	1.8145216
LOC100289453	NC_000008	42037601	42038282	0	0	0.0672936	0
IKBKB	NC_000008	42128829	42189965	2.4802245	1.6038031	2.6757272	4.6375263
POLB	NC_000008	42196030	42229313	2.897302	2.7634011	4.3786789	3.6000651
RPL5P23	NC_000008	42199227	42200201	0	0	0	0
DKK4	NC_000008	42231586	42234674	0	0	0	0.0374387
VDAC3	NC_000008	42249346	42263415	9.2619442	13.621235	15.688137	14.981186
SLC20A2	NC_000008	42273993	42397068	15.723273	13.532775	10.13799	11.559719
C8orf40	NC_000008	42396298	42408140	5.3492403	4.5443908	11.54643	6.4741895
CHRNB3	NC_000008	42552562	42592209	0.0225029	0.0231354	0	0.0156809
LOC100288666	NC_000008	42580687	42587385	0	0	0	0
CHRNA6	NC_000008	42607763	42623619	0.1261672	0.151332	0.1870697	0.2637549
THAP1	NC_000008	42691817	42698474	2.3728395	3.0024965	2.5078734	2.6992855
RNF170	NC_000008	42710391	42751782	2.5879834	2.294565	2.9148914	3.9377207
HOOK3	NC_000008	42752033	42885682	0.9221824	1.3423601	1.8438672	3.1558046
FNTA	NC_000008	42911442	42940931	6.6415274	10.348973	14.701977	11.570202
SGK196	NC_000008	42948657	42978323	1.1701387	3.1054846	2.3482748	2.3324211
HGSNAT	NC_000008	42995592	43057970	3.7761427	4.0642531	6.7112239	7.4007186
LOC100131789	NC_000008	43075045	43075952	0	0	0	0
LOC643654	NC_000008	43101400	43102251	0	0	0	0
LOC347028	NC_000008	43102346	43127103	0	0	0	0
LOC100128173	NC_000008	43129795	43132481	0	0	0	0
LOC100288815	NC_000008	43139742	43146878	0	0	0	0
POTEA	NC_000008	43147585	43218328	0	0	0	0.0464953
LOC100130474	NC_000008	43280190	43285314	0	0	0	0
LOC100130767	NC_000008	43349412	43369416	0	0	0	0

ASNSL1	NC_000008	47505079	47526158	0	0	0	0
LOC100130861	NC_000008	47696132	47697483	0	0	0	0
TRNAL41P	NC_000008	47740038	47740112	0	0	0	0
BEYLA	NC_000008	47752508	47767407	0	0	0	0
LOC728587	NC_000008	47782532	47815011	0	0	0	0
MAPK6PS4	NC_000008	47882951	47887020	0	0	0	0
LOC100287980	NC_000008	48001124	48037134	0.0552808	0.0568345	0.0491793	0
RPL10AP2	NC_000008	48068710	48069425	0	0	0	0
LOC100288013	NC_000008	48076416	48077155	0	0	0	0
LOC100287846	NC_000008	48100118	48101379	0.0368076	0.037842	0	0.051298
LOC100287875	NC_000008	48102395	48104885	0.0176428	0.0362773	0.0156955	0
ATP6V1GP2	NC_000008	48105036	48106072	0	0	0	0
LOC100128541	NC_000008	48105623	48107847	0	0	0	0
LOC392217	NC_000008	48114054	48114520	0	0	0	0
LOC100129954	NC_000008	48171254	48173385	0.1030681	0.2331226	0.0916922	0.1436439
KIAA0146	NC_000008	48173542	48648475	2.8252435	2.3012664	4.8811247	5.763564
CEBPD	NC_000008	48649476	48650726	21.88629	8.6321607	29.909169	13.219372
PRKDC	NC_000008	48685669	48872743	4.8834984	9.0346734	10.382216	27.684364
MCM4	NC_000008	48873494	48890068	30.463974	37.407713	39.341026	61.145738
UBE2V2	NC_000008	48920995	48974454	2.8549048	4.5657768	4.2586587	6.5710861
LOC100287157	NC_000008	49106313	49107363	0	0	0	0
LOC100130299	NC_000008	49206584	49207357	0	0	0	0
RPL29P19	NC_000008	49297097	49297752	0	0	0	0
LOC100289527	NC_000008	49340703	49343601	0.4851133	0.4208182	0.094406	0.1690231
EFCAB1	NC_000008	49627474	49647870	0.1167594	0.132045	0.3531661	0.1708614
LOC644334	NC_000008	49820194	49826830	0	0	0	0
SNAI2	NC_000008	49830236	49833988	23.15597	45.635021	4.6522567	7.7400335
C8orf22	NC_000008	49984903	49988642	0	0.0357181	0	0
C8orf62	NC_000008	50651756	50653949	0	0	0	0
LOC100127998	NC_000008	50818333	50824134	0	0.4634195	0	0
SNTG1	NC_000008	50824597	51705427	0	0.5740612	0.0620925	0.0486367
CYCSP22	NC_000008	51675046	51675358	0	0	0	0
LOC100128686	NC_000008	51772075	51772622	0	0	0	0
PXDNL	NC_000008	52232137	52722005	0.0274276	0.0469975	0.0488008	0.0891925
BTF3P1	NC_000008	52633816	52635606	0	0	0	0
PCMTD1	NC_000008	52730140	52811735	1.9422893	1.3168598	3.362428	3.6433802
LOC100287313	NC_000008	52853613	52859534	0.2751063	0.0707095	0.3671133	0.191705
ST18	NC_000008	53023392	53322439	0.0208285	0.0356899	0.0679421	0.2128744
RPL34P17	NC_000008	53225713	53226054	0	0	0	0
FAM150A	NC_000008	53446597	53478021	0	0	0.0333882	0.0261528
RB1CC1	NC_000008	53535018	53627026	4.5022712	5.7131555	8.737869	14.175547
NPBWR1	NC_000008	53852468	53853454	0	0	0	0.0310282
LOC100133156	NC_000008	53887590	53888676	0	0	0	0
LOC100131925	NC_000008	54091225	54108067	0	0	0	0
OPRK1	NC_000008	54138276	54164194	0	0	0.0078842	0.0432293
RPL21P79	NC_000008	54445845	54446298	0	0	0	0
MAPK6PS1	NC_000008	54448877	54454126	0	0	0	0
LOC100129667	NC_000008	54625257	54625459	0	0	0	0
ATP6V1H	NC_000008	54628115	54755850	10.803134	9.5741041	9.8691212	14.233148
RGS20	NC_000008	54764368	54871863	0.5333986	0.8813407	1.4744206	1.5796968
RPS27AP13	NC_000008	54799568	54800039	0	0	0	0
TCEA1	NC_000008	54879116	54935008	15.809969	16.498369	26.919174	17.788235
LOC100131254	NC_000008	54884509	54886342	0	0	0	0
LYPLA1	NC_000008	54958938	55014577	3.6879635	5.2310232	6.6681551	7.1862574
TDGF5	NC_000008	54985551	54986497	0	0	0	0
MRPL15	NC_000008	55047781	55061074	1.9097002	2.0925418	1.0953578	2.1011923
LOC392221	NC_000008	55104145	55104596	0	0	0	0
RNU105C	NC_000008	55243246	55243455	0	0	0	0
LOC100287492	NC_000008	55370449	55374387	0.13657	0.0280817	0	0
SOX17	NC_000008	55370495	55373456	0.0374028	0	0.0166373	0
LOC100129098	NC_000008	55378154	55382986	0.0914445	0	0.0162703	0.0254889
LOC100287567	NC_000008	55392123	55392507	0	0	0	0
SEC11B	NC_000008	55435050	55435941	0	0	0	0
RP1	NC_000008	55528627	55543394	0.0061899	0.0063639	0.0110134	0.0301935
LOC100287651	NC_000008	55568647	55733589	0.0283446	0.0145706	0	0.0592548
LOC100131013	NC_000008	55608953	55609683	0	0	0	0

XKR4	NC_000008	56015017	56438714	0.0449254	0.011547	0	0.0078264
TMEM68	NC_000008	56651320	56685885	0.3603869	0.546023	0.9787047	0.7269609
TGS1	NC_000008	56685791	56738005	2.2497876	3.4022172	2.4250657	3.5834094
LYN	NC_000008	56792386	56923940	1.3218582	0.277647	3.4646614	2.4068064
PSMC6P1	NC_000008	56962240	56963775	0	0	0	0
RPS20	NC_000008	56985613	56987069	166.96967	245.78734	303.43598	386.23651
SNORD54	NC_000008	56986398	56986460	0	0	0	0
NPM1P21	NC_000008	57013747	57014826	0	0	0	0
MOS	NC_000008	57025501	57026541	0	0.0434038	0.0375577	0.0294187
PLAG1	NC_000008	57073468	57123859	1.3235976	1.8472364	1.7689277	2.0366504
CHCHD7	NC_000008	57124315	57131178	0.5328596	0.8869721	0.5643413	1.3614986
SDR16C5	NC_000008	57212570	57233241	0.0289419	0	0	0.0302518
SDR16C6	NC_000008	57285627	57307924	0	0	0	0.0630142
PENK	NC_000008	57353513	57359282	2.8594323	11.759188	0.331804	0.3681916
LOC389662	NC_000008	57389386	57453025	0	0	0	0
RPL37P6	NC_000008	57500850	57501439	0	0	0	0
IMPAD1	NC_000008	57870488	57906427	7.1574746	11.353682	12.4212	14.327205
LOC286177	NC_000008	58173173	58179170	0.0524755	0.0134876	0.0350127	0.0274253
C8orf71	NC_000008	58192102	58197290	0	0	0	0
RPL30P10	NC_000008	58305105	58305441	0	0	0	0
FAM110B	NC_000008	58907113	59062277	1.5393955	0.9723534	1.0293544	2.1454242
RPL26P26	NC_000008	59025700	59026224	0	0	0	0
UBXN2B	NC_000008	59323823	59364060	0.5203027	0.4368561	1.5660627	2.7011288
CYP7A1	NC_000008	59402737	59412720	0.0305727	0.015716	0.0135992	0.0319564
LOC100287720	NC_000008	59416156	59416786	0	0	0	0
SDCBP	NC_000008	59465728	59495419	73.213252	39.84563	193.61143	105.51239
NSMAF	NC_000008	59496063	59572404	3.6826829	2.9620628	10.345404	10.75878
RPS26P7	NC_000008	59500890	59501347	0	0	0	0
LOC100289309	NC_000008	59571858	59573985	0.1858713	0	0.2388479	0.1439139
TOX	NC_000008	59717977	60031767	2.2766695	0.8968867	0.1230376	0.4892864
LOC100287748	NC_000008	60470013	60536490	0	0	0	0
LOC100289376	NC_000008	60672607	60672828	0	0	0	0
CA8	NC_000008	61101423	61193954	0.0578774	0.0793387	0.0686524	0.0672188
LOC392225	NC_000008	61197205	61300554	0	0	0	0
RAB2A	NC_000008	61429559	61533629	31.965831	30.699524	38.382366	38.734602
CHD7	NC_000008	61591339	61779465	0.0841598	0.0216313	0.2882528	0.4134535
LOC442389	NC_000008	61818598	61819159	0	0	0	0
NASPP1	NC_000008	61849507	61852325	0	0	0	0
LOC100130298	NC_000008	61878325	61880340	0	0	0.0433935	0.1019696
NPM1P6	NC_000008	62114814	62116115	0	0	0	0
RLBP1L1	NC_000008	62200525	62414204	0.1006832	0.0646956	0.1231596	0.3157204
ASPH	NC_000008	62413115	62627199	7.1247133	7.6058359	35.942691	23.218764
LOC100289411	NC_000008	62415515	62430223	0.0630534	0	0	0
LOC728638	NC_000008	62490703	62492474	0	0	0	0
LOC100287835	NC_000008	62596594	62627235	0.2321209	0.4772895	0.3441687	0.3235022
LOC645551	NC_000008	62822326	62823979	0	0	0	0
LOC100287895	NC_000008	63023071	63023719	0	0	0	0
LOC392226	NC_000008	63055710	63057347	0	0	0	0
NKAIN3	NC_000008	63161501	63903628	0	0.0288527	0	0.0586683
GGH	NC_000008	63927638	63951610	21.252929	27.6911	64.080203	47.301386
TTPA	NC_000008	63973431	63998612	0.2111204	0	0.8764867	1.8144443
LOC100128540	NC_000008	64048966	64050643	0	0	0	0
YTHDF3	NC_000008	64081121	64125346	11.918454	12.314778	14.311757	19.824485
IFITM8P	NC_000008	64321623	64322021	0	0	0	0
LOC100130155	NC_000008	65285706	65291386	0	0.0329806	0	0.0223539
LOC401463	NC_000008	65486865	65489820	0	0	0	0
BHLHE22	NC_000008	65492814	65496186	0.0521177	0.0669781	0.034774	0.0181588
CYP7B1	NC_000008	65508529	65711348	0.09175	0.0565972	0.1632466	0.089509
RPL31P41	NC_000008	66067642	66068013	0	0	0	0
ARMC1	NC_000008	66515064	66546432	5.8896271	8.0166851	8.826102	9.6071249
MTFR1	NC_000008	66556974	66622786	3.1689427	4.4068821	4.1990934	4.8116507
PDE7A	NC_000008	66629905	66753755	0.9787636	1.608238	1.3372005	2.0583036
DNAJC5B	NC_000008	66933791	67012755	0	0.0327416	0.0849947	0.0443839
TRIM55	NC_000008	67039278	67087720	0.1078295	0	0.013704	0.0536714
CRH	NC_000008	67088619	67090698	0	0	0.0611377	0.0239444
RRS1	NC_000008	67341263	67342968	2.8852298	2.3836496	1.9709207	2.0464456

ADHFE1	NC_000008	67344718	67381044	0.5566003	0.4768697	0.3920073	0.3070568
LOC100131770	NC_000008	67392885	67393724	0	0	0	0
C8orf46	NC_000008	67405491	67430759	0.5961232	0.1043195	0.7447155	0.4154024
MYBL1	NC_000008	67474410	67525480	3.5663465	2.7608208	4.363433	6.8888267
VCPIP1	NC_000008	67542488	67579452	0.5267768	0.3868442	0.5547111	1.2960137
C8orf44	NC_000008	67588454	67593235	0.7014302	0.3730055	0.9037412	0.8427319
SGK3	NC_000008	67624891	67774257	1.5448962	0.9822479	1.4105505	2.0185223
PTTG3	NC_000008	67679632	67680240	0	0	0	0
C8orf45	NC_000008	67782984	67834283	0.203827	0.1692564	0.1813301	0.131109
SNHG6	NC_000008	67834165	67837777	0	0	0	0
SNORD87	NC_000008	67834709	67834784	0	0	0	0
LOC100129654	NC_000008	67860073	67874000	0.306473	0.3150865	0	0
LOC100288001	NC_000008	67896382	67898821	0	0	0	0
LRRC67	NC_000008	67900366	67940786	0	0.1250463	0	0
COPS5	NC_000008	67955314	67974562	18.818102	18.649525	15.770226	21.663505
CSPP1	NC_000008	67976603	68108498	0.6093002	0.7072536	0.7169053	1.0272209
ARFGEF1	NC_000008	68109897	68255912	3.0651637	4.1349203	3.3719753	4.3822621
RPL17P31	NC_000008	68318704	68319336	0	0	0	0
CPA6	NC_000008	68334405	68658620	0.0461641	0.0237308	0	0.0482535
LOC100132812	NC_000008	68481651	68482061	0	0	0	0
PREX2	NC_000008	68864603	69143897	0.0243525	0.025037	0.0072216	0.0113132
RPL31P40	NC_000008	69205772	69206051	0	0	0	0
C8orf34	NC_000008	69350148	69731257	0.0361021	0.0247445	0.0214116	0.0670863
RPS15AP25	NC_000008	69437718	69438181	0	0	0	0
LOC100129096	NC_000008	70015349	70042702	0	0	0	0
TRNAE28P	NC_000008	70072340	70072414	0	0	0	0
SULF1	NC_000008	70378859	70573147	10.974331	9.0396712	0.0776387	0.0202713
LOC100288066	NC_000008	70580989	70588883	0	0.0616417	0	0
SLCO5A1	NC_000008	70584575	70747208	0.0714218	0.0489528	0.0635388	0.0414746
LOC100101127	NC_000008	70706403	70712477	0	0	0	0
LOC100288097	NC_000008	70771499	70772775	0	0	0	0
LOC100288139	NC_000008	70846744	70850618	0	0	0	0
LOC100129960	NC_000008	70855183	70856883	0	0	0	0
LOC100288171	NC_000008	70899645	70899989	0	0	0	0
PRDM14	NC_000008	70964021	70983562	0	0.0204542	0.0176992	0
H2AFZP2	NC_000008	71015653	71016474	0	0	0	0
NCOA2	NC_000008	71024267	71316020	1.720241	1.7539115	2.3939877	3.2728878
BTF3L2	NC_000008	71185498	71186394	0	0	0	0
RPS18P11	NC_000008	71336323	71336854	0	0	0	0
RPL13P11	NC_000008	71418490	71419053	0	0	0	0
TRAM1	NC_000008	71485673	71520604	52.110722	37.358524	44.018055	42.538823
LACTB2	NC_000008	71549501	71581447	4.6976217	4.305952	3.7763264	3.8650844
XKR9	NC_000008	71581600	71648177	0.0692316	0.0711774	0.1354988	0.1254327
EYA1	NC_000008	72109668	72274467	0	0	0.0085759	0.0604571
TRAPPC2P2	NC_000008	72360946	72361475	0	0	0	0
MSC	NC_000008	72753777	72756731	3.5021915	5.0496513	1.1778664	0.8035682
LOC100132891	NC_000008	72755708	72965237	2.1493344	2.0643639	2.2894971	1.9312986
RPS20P20	NC_000008	72808230	72808567	0	0	0	0
TRPA1	NC_000008	72933486	72987819	0.1185501	0.121882	0.1958645	0.1888239
LOC392232	NC_000008	73071589	73163800	0	0	0	0
LOC100129527	NC_000008	73341856	73342161	0	0	0.3833095	0.4003252
KCNB2	NC_000008	73449626	73850584	0.0245384	0.012614	0.010915	0.0427483
LOC100271703	NC_000008	73530746	73531877	0	0	0	0
LOC100288310	NC_000008	73856383	73860870	0	0	0	0
TERF1	NC_000008	73921097	73959987	2.09348	2.579728	1.4397414	2.0278636
C8orf84	NC_000008	73976778	74005507	0.0231428	0	0.0308828	0.0645074
LOC646197	NC_000008	74128134	74128862	0	0	0	0
LOC100130301	NC_000008	74153659	74198142	0	0	0	0
LOC100131252	NC_000008	74189594	74190281	0	0	0	0
RPL7	NC_000008	74202874	74205869	38.172479	55.979914	35.267219	75.592591
RDH10	NC_000008	74207265	74237516	0.8214623	0.8189572	0.8747402	0.7892563
LOC100127988	NC_000008	74255472	74275023	0	0	0	0.0923363
LOC100128126	NC_000008	74332309	74353753	0	0	0	0
STAU2	NC_000008	74461840	74659057	1.4787134	1.2184539	2.176386	2.0911594
VENTXP6	NC_000008	74563136	74564492	0	0	0	0
UBE2W	NC_000008	74702840	74791110	2.1761937	1.98006	3.6687243	2.8736888

LOC100128120	NC_000008	74741237	74744210	0	0	0	0
LOC100132965	NC_000008	74817589	74827245	0	0	0	0
TCEB1	NC_000008	74858634	74884346	31.937952	46.494065	41.544894	36.703098
TMEM70	NC_000008	74888430	74895016	4.1497107	5.2831864	4.7819919	5.738419
RPS20P21	NC_000008	74894224	74894749	0	0	0	0
LOC100287016	NC_000008	74896666	74897118	0	0.1994852	0.2589243	0.1352092
LY96	NC_000008	74903587	74941314	6.4565015	2.4892358	5.7438828	8.1291413
RPS3AP32	NC_000008	74968093	74968925	0	0	0	0
JPH1	NC_000008	75146939	75233562	0	0.2479766	0.1251694	0.0210095
GDAP1	NC_000008	75262618	75279345	1.6682068	2.978234	0.2406621	0.3534546
PCBP2P2	NC_000008	75515112	75516763	0	0	0	0
LOC100289136	NC_000008	75716491	75717040	0	0	0	0
PI15	NC_000008	75736772	75767264	0.0065283	0.0201352	0.0174232	0.0181966
CRISPLD1	NC_000008	75896843	75946793	0.7125607	0.2693336	0.820359	0.2409683
LOC100289164	NC_000008	76214444	76214887	0	0	0	0
HNF4G	NC_000008	76452203	76479069	0.1285976	0.1542472	0.5910873	0.2090945
MRPL9P1	NC_000008	77515060	77516081	0	0	0	0
LOC100192378	NC_000008	77523114	77595510	0	0	0	0
ZFHX4	NC_000008	77593515	77779521	1.189079	1.5036074	1.1483478	3.3498384
PXMP3	NC_000008	77892494	77912524	3.3119243	4.3336444	4.7409928	3.9094101
LOC100289199	NC_000008	77925911	77929261	0	0	0	0
PKIA	NC_000008	79428336	79515485	2.0726501	2.4826045	2.8642905	3.9963783
FAM164A	NC_000008	79578282	79632000	1.363549	1.9410531	2.6360531	2.4668011
IL7	NC_000008	79645005	79717758	0.7613746	0.5653361	0.7714149	0.5895068
RPL3P9	NC_000008	80483330	80484586	0	0	0	0
STMN2	NC_000008	80523380	80577237	1.1002339	6.7869366	0.2175108	0.0851874
HEY1	NC_000008	80676245	80680098	0.0571994	0.0392047	0.0508862	0.0664314
LOC100287048	NC_000008	80679245	80680092	0	0.0794084	0	0
RNU7-85P	NC_000008	80712371	80712429	0	0	0	0
MRPS28	NC_000008	80831095	80942506	8.6404586	9.7141119	7.4102883	14.034598
TPD52	NC_000008	80947103	81083836	0.1761452	0.2615828	1.5844482	2.8504117
LOC100133047	NC_000008	81095256	81143472	0	0	0	0
RPL13AP18	NC_000008	81177740	81181009	0	0	0	0
RPS5P5	NC_000008	81213032	81213771	0	0	0	0
LOC100216346	NC_000008	81263351	81264031	0	0	0	0
ZBTB10	NC_000008	81398448	81434610	0.5003181	0.7082038	1.5223603	0.8741303
RPSAP47	NC_000008	81471029	81472062	0	0	0	0
LOC100287085	NC_000008	81480279	81480741	0	0	0	0
MCART5P	NC_000008	81506985	81508103	0	0	0	0
ZNF704	NC_000008	81550769	81787016	0.3881089	0.1680071	0.7268894	0.8113493
CKS1A	NC_000008	81556687	81557451	0	0	0	0
PAG1	NC_000008	81880045	82024303	0.891727	0.4373673	0.8042221	1.2228288
UBE2HP	NC_000008	82167120	82168005	0	0	0	0
FABP5	NC_000008	82192785	82197008	2.7301174	2.4645492	2.6065044	1.2064346
PMP2	NC_000008	82352561	82359719	0	0	0.0219095	0.042904
FABP9	NC_000008	82370618	82373758	0.1101459	0	0	0
FABP4	NC_000008	82390732	82395473	0.4195535	0.0539181	0.1399674	0.0365452
FTHL11	NC_000008	82433755	82434668	0	0	0	0
FABP12	NC_000008	82437281	82443550	0.1038965	0	0	0.0723992
IMPA1P	NC_000008	82516901	82539209	0	0	0	0
RPS26P34	NC_000008	82539504	82539852	0	0	0	0
LOC100127992	NC_000008	82546639	82547489	0	0	0	0
IMPA1	NC_000008	82569151	82598589	2.6635292	2.2108087	3.2282394	2.6308277
SLC10A5	NC_000008	82605891	82607207	0.1001099	0.0686156	0.0296868	0.1395211
ZFAND1	NC_000008	82613958	82633530	4.828668	4.4099807	5.7566968	6.6271348
CHMP4C	NC_000008	82644688	82671750	0	0.0733894	0.5715399	0.3647793
SNX16	NC_000008	82711818	82754521	1.5768463	1.0851338	1.1199823	2.4368754
LOC100128836	NC_000008	82720007	82720968	0	0	0	0
HNRNPA1P4	NC_000008	83203143	83204625	0	0	0	0
LOC100130153	NC_000008	84113070	84114666	0	0	0	0
RALYL	NC_000008	85095453	85834079	0.0409328	0	0.036415	0.0475394
ACTBP6	NC_000008	85860744	85862510	0	0	0	0
IGJP1	NC_000008	85891209	85892805	0	0	0	0
LRRCC1	NC_000008	86019377	86058315	1.5063366	1.2413023	1.1661754	1.2179439
E2F5	NC_000008	86089619	86126753	0.8287651	0.2191005	1.2639299	0.8910256
C8orf59	NC_000008	86126288	86132643	2.8204325	2.510189	5.0931695	6.6881918

CA13	NC_000008	86157716	86196302	0.8322233	0.4688291	0.760653	0.9930246
CA1	NC_000008	86240458	86290396	0.0311911	0.0320677	0	0.0434704
CA3	NC_000008	86351056	86361269	0.0752109	0.1030996	0.0892129	0.1222899
CA2	NC_000008	86376131	86393721	0	0	0.633634	0.1654405
REXO1L8	NC_000008	86554954	86556751	0	0	0	0
REXO1L3P	NC_000008	86566828	86568889	0	0	0	0
REXO1L1	NC_000008	86568695	86575726	0	0.0064254	0	0.0043551
LOC100289448	NC_000008	86726458	86728150	0	0	0	0
LOC100288470	NC_000008	86747403	86749604	0	0	0	0
LOC100288500	NC_000008	86756684	86758885	0	0	0	0
REXO1L6P	NC_000008	86775084	86777113	0	0	0	0
LOC100288527	NC_000008	86787245	86789446	0	0	0	0
LOC100288562	NC_000008	86802520	86804721	0	0	0	0
REXO1L4P	NC_000008	86814712	86816773	0	0	0	0
REXO1L5P	NC_000008	86826907	86828965	0	0	0	0
REXO1L7P	NC_000008	86838856	86840931	0	0	0	0
REXO1L2P	NC_000008	86839094	86840171	0	0	0	0
RPL36AP31	NC_000008	87028987	87029414	0	0	0	0
RPL32P4	NC_000008	87055281	87055772	0	0	0	0
PSKH2	NC_000008	87060691	87081851	0	0	0.033763	0.0528927
ATP6V0D2	NC_000008	87111139	87166454	0.0927178	0.0190647	0.0329937	0
SLC7A13	NC_000008	87226288	87242604	0	0	0.0209638	0
WWP1	NC_000008	87354994	87480181	4.0546202	4.333559	4.8062976	6.1279576
FAM82B	NC_000008	87484578	87521009	4.9341357	4.7631328	3.9174127	5.1174731
SLC2A3P4	NC_000008	87515645	87519176	0	0	0	0
CPNE3	NC_000008	87526656	87573726	6.0989014	6.1774182	11.727662	21.514229
CNGB3	NC_000008	87586161	87755901	0.0404401	0.0623649	0.0179883	0.0070451
CNBD1	NC_000008	87878676	88394955	0	0	0	0
LOC100128412	NC_000008	88587866	88588734	0	0	0	0
LOC642461	NC_000008	88610688	88614042	0	0	0	0
SOX5P	NC_000008	88800263	88802112	0	0	0	0
WDR21C	NC_000008	88882971	88886296	0.0132135	0.0135849	0.0235103	0.0276232
MMP16	NC_000008	89049460	89339717	0.4179228	0.5387908	0.4308109	0.7396197
LOC100129100	NC_000008	89455825	89498323	0	0	0	0
LOC100289570	NC_000008	90424742	90427384	0	0	0	0
RIPK2	NC_000008	90769975	90803292	7.2935363	6.8343173	8.5606275	3.8977118
OSGIN2	NC_000008	90914096	90940096	2.5174609	2.9393288	4.6267767	3.311349
NBN	NC_000008	90945564	90996899	8.8733392	11.449851	6.6502243	7.6611901
DECR1	NC_000008	91013580	91064227	14.052192	11.666058	14.001366	9.9390093
CALB1	NC_000008	91070838	91095107	0.017364	0	0.0463424	0.0483996
TMEM64	NC_000008	91634223	91658133	0.7843013	1.8564668	1.5820762	1.4235262
LOC100289606	NC_000008	91713089	91713995	0	0	0	0
NECAB1	NC_000008	91803921	91971630	1.2910862	4.2985766	1.5136995	1.4418717
LOC100127983	NC_000008	91970706	91997468	3.1815805	7.1961982	6.151451	7.1241271
TMEM55A	NC_000008	92006499	92053203	4.0368077	4.525852	10.741268	7.8789691
OTUD6B	NC_000008	92082424	92099323	0.239863	0.2877051	0.355648	0.8543022
LRRC69	NC_000008	92114847	92231464	0.0664372	0.0683045	0.0295522	0.0462961
CPP	NC_000008	92169506	92172150	0	0	0	0
SLC26A7	NC_000008	92261516	92410378	0.038123	0.0156778	0.0135661	0.0796969
LOC100289644	NC_000008	92921961	92925070	0	0	0	0
MRPS16P1	NC_000008	92929735	92930150	0	0	0	0
RUNX1T1	NC_000008	92971152	93107443	0.4033896	0.5566071	0.0755509	0.0295893
RPS26P10	NC_000008	93156235	93156687	0	0	0	0
LOC100289679	NC_000008	93364139	93364374	0	0	0	0
C8orf83	NC_000008	93895863	93978339	0	0	0	0
LOC100286900	NC_000008	94005353	94137059	0	0	0	0
LOC389676	NC_000008	94146324	94179079	0.0747419	0	0	0
LOC100133058	NC_000008	94231268	94242673	0	0	0	0
LOC100288659	NC_000008	94658048	94659325	0	0	0	0
FAM92A1	NC_000008	94712773	94740674	7.6380617	10.470307	10.349356	10.91796
RBM12B	NC_000008	94743728	94753224	2.3506793	2.7885524	3.1176938	5.0041435
C8orf39	NC_000008	94752339	94753047	0	0	0	0
TMEM67	NC_000008	94767072	94831462	1.1665271	0.8739177	0.965372	1.1027477
LOC157667	NC_000008	94861789	94862308	0	0	0	0
LOC100288714	NC_000008	94869987	94871826	0	0.0736686	0	0.0832198
PPM2C	NC_000008	94929162	94938296	6.9391945	7.3386711	4.2924453	9.3501057

LOC100131775	NC_000008	94951583	94955105	0	0	0	0
RPL34P18	NC_000008	94969566	94969919	0	0	0	0
CDH17	NC_000008	95139394	95229531	0.0231063	0.0118779	0.030834	0.0241521
RPL6P23	NC_000008	95214111	95215300	0	0	0	0
GEM	NC_000008	95261481	95274557	10.1419	6.9924245	9.680963	6.4678851
RPS4P10	NC_000008	95365149	95365943	0	0	0	0
RAD54B	NC_000008	95384188	95487310	1.3873825	2.4961564	2.0185195	2.8802089
KIAA1429	NC_000008	95500600	95565688	1.1439887	1.455097	1.2525834	3.7048284
LOC100286997	NC_000008	95577264	95606768	0	0	0	0
LOC100288748	NC_000008	95649513	95651697	0.0201136	0.0206789	0.0178936	0.014016
ESRP1	NC_000008	95653364	95719694	0.0115471	0	0	0.0080465
LOC100288776	NC_000008	95677385	95681009	0	0	0	0
RPS15AP26	NC_000008	95700466	95700838	0	0	0	0
DPY19L4	NC_000008	95732103	95806076	3.0384485	4.0733192	3.5623063	3.1685991
INTS8	NC_000008	95835534	95892721	1.6286774	1.7682209	2.3878146	2.3969665
CCNE2	NC_000008	95892452	95907482	2.4012785	3.6948656	4.5879065	8.1980789
LOC100128548	NC_000008	95902665	95904335	0	0	0	0
TP53INP1	NC_000008	95938200	95961615	1.1430273	0.9353252	1.3350726	1.9127003
C8orf38	NC_000008	96037221	96070938	1.2514572	2.1191545	1.8555517	2.7187915
LOC100130098	NC_000008	96084842	96085849	0	0	0	0.0303818
PLEKHF2	NC_000008	96146032	96168911	3.0977399	2.9180947	2.4435979	2.233064
C8orf37	NC_000008	96258235	96281437	1.3063583	0.9020644	0.8672929	0.7336927
LOC643228	NC_000008	97080748	97081959	0	0	0	0
LOC100287097	NC_000008	97138927	97142619	0	0	0	0
GDF6	NC_000008	97154558	97173020	1.614958	0.5982131	0	0.0248243
UQCRB	NC_000008	97239304	97247862	1.0939818	1.0633795	0.9997793	2.1206637
MTERFD1	NC_000008	97251645	97273796	3.9072041	6.073729	5.4224931	5.1186674
PTDSS1	NC_000008	97274167	97346774	3.7208567	7.6689081	4.4968446	6.8367839
SDC2	NC_000008	97505882	97624037	16.885705	11.525981	3.6236768	3.9807948
PGCP	NC_000008	97657499	98155722	16.434228	6.219867	10.991035	5.0233846
TSPYL5	NC_000008	98285714	98290176	0.1083196	1.3161197	0	0.0137239
RPS2P33	NC_000008	98636394	98637330	0	0	0	0
MTDH	NC_000008	98656407	98742488	11.458526	15.481518	13.299394	13.604844
LAPTM4B	NC_000008	98787809	98864830	6.7748615	6.8441346	8.1234888	8.4567358
LOC100127982	NC_000008	98865245	98865983	0	0	0	0
RPS23P1	NC_000008	98877152	98877856	0	0	0	0
LOC100287139	NC_000008	98880805	98944866	0.1472303	0.0756841	0.0654901	0.2308408
MATN2	NC_000008	98881311	99048948	0.256632	0.208877	0.8276127	1.4530052
RPL30	NC_000008	99053942	99057773	83.022996	120.07154	123.71863	108.0664
SNORA72	NC_000008	99054314	99054445	0	0	0	0
C8orf47	NC_000008	99076750	99105838	0.0567074	0	0.0756727	0.019758
HRSP12	NC_000008	99114567	99129418	3.8253654	3.217809	5.0273823	4.9072505
POP1	NC_000008	99130068	99170638	2.2502796	4.7246062	1.760717	4.0335666
NIPAL2	NC_000008	99204387	99306621	0.8770157	0.6612205	0.2947488	0.5839777
LOC100131849	NC_000008	99425110	99426012	0	0	0	0
KCNS2	NC_000008	99439250	99443025	0.0832353	0.2139366	0.0370242	0.0096669
STK3	NC_000008	99466861	99837909	1.0108404	0.9752964	1.3281551	1.8639346
RPL19P14	NC_000008	99794240	99794938	0	0	0	0
LOC643494	NC_000008	99916825	99917898	0	0	0	0
MRP63P7	NC_000008	99918483	99918755	0	0	0	0
OSR2	NC_000008	99956631	99964328	1.4313092	0.6754593	0.7514748	0.7030805
LOC100287169	NC_000008	100015633	100017377	0	0	0	0
VPS13B	NC_000008	100025494	100889808	1.1320997	1.1331261	1.2682596	2.4731144
LETM1P3	NC_000008	100708158	100710748	0	0	0	0
COX6C	NC_000008	100890372	100905895	31.539319	34.126758	40.753463	39.632195
RGS22	NC_000008	100973276	101118344	0.0945349	0.0431964	0.0280336	0.0219586
FBXO43	NC_000008	101145659	101158076	0.1890247	0.3886744	0.6366117	0.3951597
POLR2K	NC_000008	101162839	101166230	9.3689845	11.214418	13.609657	14.949735
SPAG1	NC_000008	101170263	101254130	0.9155882	1.1364728	0.9337325	1.4705544
RNF19A	NC_000008	101269288	101322327	2.6004871	2.5448464	2.7076114	3.7182079
ANKRD46	NC_000008	101533000	101572012	0.6167618	0.6666137	0.7315845	0.5620255
GAPDHL7	NC_000008	101562768	101563961	0	0	0	0
SNX31	NC_000008	101585110	101661893	0.0178869	0.0367793	0.0159127	0.0498574
PABPC1	NC_000008	101715144	101734315	64.800592	73.588911	81.01125	251.20967
RPS20P23	NC_000008	101788740	101789096	0	0	0	0
RPS26P6	NC_000008	101907964	101908430	0	0	0	0

YWHAZ	NC_000008	101930804	101965623	35.714709	51.977619	40.907341	76.323135
ZNF706	NC_000008	102209266	102217960	2.9196378	3.7916141	4.86669	4.7971837
RNU7-67P	NC_000008	102319596	102319655	0	0	0	0
DUXAP2	NC_000008	102378566	102379275	0	0	0	0
NACAP1	NC_000008	102381121	102381823	0	0	0	0
LOC100288906	NC_000008	102503396	102504518	0	0.0402346	0	0.0545412
GRHL2	NC_000008	102504668	102681954	0.0084015	0	0.0074742	0.0175635
LOC100131593	NC_000008	102540645	102541834	0	0	0	0
NCALD	NC_000008	102698770	103137135	0.6660307	1.2083816	0.017427	0.0341262
LOC643831	NC_000008	102883723	102884887	0	0	0	0
LOC100288934	NC_000008	103166778	103166960	0	0	0	0
RRM2B	NC_000008	103216731	103251346	4.6444277	2.4653824	5.2182958	2.609016
UBR5	NC_000008	103265569	103424495	2.0023252	2.7896927	2.8592748	3.980517
RPS12P15	NC_000008	103515983	103516366	0	0	0	0
ODF1	NC_000008	103563848	103573245	0	0	0	0
POU5F1P2	NC_000008	103632785	103633475	0	0	0	0
LOC100129512	NC_000008	103633728	103640638	0	0	0	0
KLF10	NC_000008	103661005	103667983	2.9786707	3.1019864	3.5979355	3.5876647
LOC100288971	NC_000008	103700547	103701052	0	0	0	0
FLJ45248	NC_000008	103819056	103822203	0	0	0	0
AZIN1	NC_000008	103838536	103876397	14.393349	16.502126	22.749963	24.581607
RPL5P24	NC_000008	103935541	103936557	0	0	0	0
LOC643972	NC_000008	104032372	104033650	0	0	0	0
ATP6V1C1	NC_000008	104033248	104085285	8.2672603	7.1133757	8.3144017	13.911125
LOC100287262	NC_000008	104097880	104098824	0	0	0	0
FLJ10489	NC_000008	104133266	104145194	0.0799059	0	0.142173	0.1670448
C8orf56	NC_000008	104145191	104153570	0	0	0	0
LOC100131102	NC_000008	104148391	104225293	0.2195216	0.1805531	0.0390585	0.0611886
BAALC	NC_000008	104152921	104242533	3.7022027	5.3921926	2.0996587	0.5267178
FZD6	NC_000008	104311100	104345094	6.3569232	6.4633689	17.01796	9.8629403
CTHRC1	NC_000008	104383786	104395217	14.877557	52.072019	5.544163	3.1126925
SLC25A32	NC_000008	104410866	104427468	6.4091171	8.0834062	8.5849152	6.602983
WDSOF1	NC_000008	104426942	104455200	3.9860497	6.5861969	4.667826	8.0494821
LOC100289041	NC_000008	104512855	104709520	0	0.0699433	0.1210451	0.0948139
RIMS2	NC_000008	104512976	105265451	0.2017296	0.1481423	0.2179209	0.1255118
TARBP2P	NC_000008	104671415	104672838	0	0	0	0
RPS6P9	NC_000008	104780468	104781291	0	0	0	0
TM7SF4	NC_000008	105352054	105368916	0.0225145	0	0	0
DPYS	NC_000008	105391652	105479277	0.041402	0.0212828	0.1841619	0.0144253
LRP12	NC_000008	105501464	105601220	16.886723	8.2521242	8.17702	7.4998182
RPL23P9	NC_000008	105993365	105997113	0	0	0	0
RPL29P18	NC_000008	106023295	106023905	0	0	0	0
LOC100128132	NC_000008	106207092	106209197	0	0	0	0
RPL17P32	NC_000008	106300069	106300593	0	0	0	0
ZFPM2	NC_000008	106331147	106816767	0.1961975	0.9682158	0.7679879	0.4853496
RPL12P24	NC_000008	106749508	106749984	0	0	0	0
LOC100128685	NC_000008	107221971	107229626	0	0	0	0
OXR1	NC_000008	107282473	107764922	3.919972	3.407303	4.5414363	4.2463849
RNU7-84P	NC_000008	107741923	107742184	0	0	0	0
ABRA	NC_000008	107771711	107782472	0.015958	0.0164065	0.0425899	0.0556007
ANGPT1	NC_000008	108261710	108510254	4.3461944	1.760257	0.7390503	1.0730709
LOC100129815	NC_000008	108659508	108660264	0	0	0	0
RSPO2	NC_000008	108911544	109095913	0.1256063	0.057394	0.1489904	0.252857
LOC728381	NC_000008	108995202	108996994	0	0	0	0
LOC644233	NC_000008	109143280	109153960	0	0	0	0
EIF3E	NC_000008	109213972	109260959	48.931751	57.797601	84.78053	90.595214
RPS17P14	NC_000008	109391388	109391774	0	0	0	0
TTC35	NC_000008	109455853	109499136	0.4614919	0.4379651	0.6316246	0.7915962
LOC100130095	NC_000008	109659551	109665750	0	0	0	0
TMEM74	NC_000008	109795346	109799770	0.0421162	0.0649498	0.3746772	0.513594
TRHR	NC_000008	110099726	110131813	0.0654966	0	0	0.0228203
NUDCD1	NC_000008	110253148	110346350	4.2204083	4.58254	4.3484309	4.4339302
ENY2	NC_000008	110346636	110355911	3.7846456	6.5909005	5.9780109	10.495345
PKHD1L1	NC_000008	110374706	110543500	0.0094061	0.0161174	0.0139465	0.0393271
MAPK6PS5	NC_000008	110482367	110484483	0	0	0	0
EBAG9	NC_000008	110551929	110577392	1.7938054	1.7228902	1.2178619	1.2828896

GOLSYN	NC_000008	110586405	110704020	0.0968236	0.1493173	0.6546409	0.5667525
LOC100132813	NC_000008	110656344	110660654	0	0	0	0
LOC644335	NC_000008	110790155	110794517	0	0	0	0
RPL18P6	NC_000008	110911397	110915382	0	0	0	0
KCNV1	NC_000008	110979233	110986959	0.030009	0.0308524	0	0.0104557
RPSAP48	NC_000008	111117305	111118193	0	0	0	0
LOC100132280	NC_000008	111549948	111568749	0	0	0	0
LOC100129370	NC_000008	111821525	111822123	0	0	0	0
CSMD3	NC_000008	113235157	114449242	0.0131631	0.0169163	0.0087827	0.0229314
LOC100289099	NC_000008	113551118	113551546	0	0	0	0
RPL18P7	NC_000008	114388865	114389519	0	0	0	0
TRPS1	NC_000008	116420724	116681228	1.5793208	0.7100895	3.4596845	2.3114272
EIF3H	NC_000008	117657055	117768062	37.950591	46.033913	80.801636	112.31524
UTP23	NC_000008	117778742	117786923	1.6898589	1.3091319	1.9691706	1.9321951
RAD21	NC_000008	117858173	117887105	20.450577	33.95382	54.496793	53.221955
C8orf81	NC_000008	117886632	117889107	0	0	0	0
C8orf85	NC_000008	117950464	117956239	0.1230354	0.4300772	0.0218911	0.0171472
SLC30A8	NC_000008	118147337	118188953	0.0081795	0.0084093	0	0.0227991
MED30	NC_000008	118532965	118552501	1.6729265	2.8820688	3.620145	3.2767359
RPS10P16	NC_000008	118535456	118535965	0	0	0	0
EXT1	NC_000008	118811602	119124058	37.832332	38.156664	27.250875	47.499068
SAMD12	NC_000008	119201694	119634184	0.6271104	0.3461666	0.5279407	1.5456149
RPS26P35	NC_000008	119774006	119774459	0	0	0	0
TNFRSF11B	NC_000008	119935796	119964383	5.5450454	24.979911	0.4666376	3.3810074
LOC100287667	NC_000008	119994759	120003574	0	0	0	0
COLEC10	NC_000008	120079446	120119207	0	0.0267992	0	0.0544927
MAL2	NC_000008	120220610	120257913	0.0935732	0	0.0277484	0.0434704
NOV	NC_000008	120428552	120436678	11.185594	17.388922	3.2618884	3.9443807
LOC392264	NC_000008	120492145	120498595	0	0	0	0
ENPP2	NC_000008	120569319	120651106	40.407193	47.596959	48.373174	20.928371
CYCSP23	NC_000008	120630396	120630714	0	0	0	0
TAF2	NC_000008	120743014	120845074	3.9220733	5.1483876	6.7680846	7.6684207
DSCC1	NC_000008	120846181	120868170	1.3558291	2.4095159	2.4640599	3.4417558
DEPDC6	NC_000008	120885900	121063157	0.267264	1.2880103	0.1188828	0.2444403
COL14A1	NC_000008	121137352	121384273	1.911976	2.1685794	0.7082234	0.09957
MRPL13	NC_000008	121408083	121457647	8.6312744	17.422068	28.706771	24.582673
MTBP	NC_000008	121457666	121535875	1.1349821	1.8020186	2.492326	2.1324286
SNTB1	NC_000008	121547985	121824309	5.5741375	4.7771798	4.2280383	2.8501143
LOC100133147	NC_000008	122125593	122126160	0	0	0	0
RPL35AP19	NC_000008	122392333	122392779	0	0	0	0
HAS2	NC_000008	122625271	122653630	19.739801	26.571979	1.9339865	3.1606745
HAS2AS	NC_000008	122651586	122656933	0	0	0	0
MRPS36P3	NC_000008	123103709	123103841	0	0	0	0
LOC100131552	NC_000008	123712508	123730574	0	0	0.0681142	0.0533534
LOC392265	NC_000008	123770570	123782442	0	0	0	0
ZHX2	NC_000008	123793901	123986755	0.5646022	0.5493738	0.8790001	1.2646199
DERL1	NC_000008	124025568	124054648	7.9665729	7.7187716	11.465816	10.250276
WDR67	NC_000008	124084920	124164393	0.5517039	0.8637055	1.2604921	1.3368349
FAM83A	NC_000008	124194752	124222318	0.0733285	0.0125649	0.1848328	0.0766474
LOC100131726	NC_000008	124213412	124214983	0	0	0	0
C8orf76	NC_000008	124232231	124253617	4.5451904	5.0242819	5.0468086	6.2154691
UBA52P5	NC_000008	124249092	124249456	0	0	0	0
ZHX1	NC_000008	124260696	124286547	4.8548994	4.6399705	6.4785797	6.8150278
TRNAF14P	NC_000008	124270725	124270797	0	0	0	0
LOC392266	NC_000008	124300573	124303727	0	0	0	0
ATAD2	NC_000008	124332090	124408705	1.4294034	1.9404911	2.8945547	9.1186745
IMPDH1P6	NC_000008	124412813	124415055	0	0	0	0
WDYHV1	NC_000008	124428965	124454260	3.0646393	3.7876294	6.6129414	4.6346255
FBXO32	NC_000008	124515358	124553446	13.727235	8.5057483	88.180481	37.803732
LOC100289165	NC_000008	124657767	124659221	0	0	0	0
KLHL38	NC_000008	124657915	124665190	0.2396305	0.0246365	0.2345001	0.083492
ANXA13	NC_000008	124693034	124749647	0.0551767	0	0.0490867	0.0384493
FAM91A1	NC_000008	124780882	124827692	5.0665066	3.8854672	7.2160922	8.2628521
FER1L6	NC_000008	124953961	125132302	0.1939002	0.076673	0.1990373	0.0935428
LOC392268	NC_000008	125154123	125155753	0	0	0	0
LOC442396	NC_000008	125164165	125299683	0	0	0	0

TMEM65	NC_000008	125323159	125384940	2.9081138	2.9583746	5.7098604	7.7148811
TRMT12	NC_000008	125463048	125465267	1.4055516	2.1574057	3.1172383	2.9383329
RNF139	NC_000008	125487008	125500859	4.0737039	4.3382614	7.496061	6.5466225
TATDN1	NC_000008	125500749	125551311	4.2459945	4.0053016	8.9176718	7.4426995
NDUFB9	NC_000008	125551343	125562227	15.647771	26.267958	38.336278	32.754564
MTSS1	NC_000008	125563025	125740730	2.2632024	0.5681745	1.2096053	0.709079
LOC100130448	NC_000008	125932738	125934324	0	0	0	0
LOC157381	NC_000008	125954250	125963337	0	0	0	0
ZNF572	NC_000008	125985539	125991631	0.4561172	0.2068837	0.7160726	0.5048057
SQLE	NC_000008	126010720	126034525	14.24752	14.481666	17.240077	16.557311
KIAA0196	NC_000008	126036502	126104061	2.0676556	2.0606928	3.1251775	7.851025
NSMCE2	NC_000008	126104083	126379367	3.7711354	4.8464045	9.1937345	10.536777
TRIB1	NC_000008	126442563	126450645	3.0000444	2.5371357	3.9172898	3.2622703
LOC650095	NC_000008	126964098	126965093	0	0	0	0
LOC100130158	NC_000008	127084831	127086482	0	0	0	0
LOC100287876	NC_000008	127552113	127595251	0	0	0	0
FAM84B	NC_000008	127564687	127570466	0.0167998	0.0259079	0.0298911	0.046827
SRRM1L	NC_000008	128099755	128100775	0	0	0	0
POU5F1B	NC_000008	128427857	128429455	0.0274848	0.0282573	0.0244513	0
LOC727677	NC_000008	128455595	128494384	0	0	0	0
MYC	NC_000008	128748316	128753674	7.3588603	8.4084415	18.910692	25.328163
PVT1	NC_000008	128806779	129113499	0	0	0	0
LOC100287906	NC_000008	130364905	130365381	0	0	0	0
LOC100130376	NC_000008	130713357	130742691	0	0	0	0
GSDMC	NC_000008	130760442	130799134	0.5505674	0.1664827	0.6914824	0.5529178
FAM49B	NC_000008	130853716	130952000	6.9644739	7.9626487	19.619999	20.249236
RPL15P12	NC_000008	130876724	130877415	0	0	0	0
ASAP1	NC_000008	131064353	131414217	8.9976767	11.022895	13.576083	29.70745
DDEF1IT1	NC_000008	131307601	131308779	0	0	0	0
ADCY8	NC_000008	131792547	132052835	0.0642988	0.033053	2.8314989	2.4717958
EFR3A	NC_000008	132916359	133025774	7.6965388	8.0578062	7.2159689	12.627922
OC90	NC_000008	133036467	133071627	0.0246623	0	0	0.1031141
HHLA1	NC_000008	133073733	133117512	0.010706	0.0220138	0.0190488	0.0447623
KCNQ3	NC_000008	133141256	133493004	0.8656255	0.5106293	2.9793431	3.0654544
HPYR1	NC_000008	133572745	133573726	0	0	0	0
LRRC6	NC_000008	133584447	133687813	0.972306	0.6569015	0.4448522	0.3871666
TMEM71	NC_000008	133722191	133772914	0.9862156	0.1478653	4.277153	0.5297431
PHF20L1	NC_000008	133787604	133861052	1.1913987	1.2565611	1.9690408	2.9057491
TG	NC_000008	133879205	134147143	0.0935843	0.042762	0.0693793	0.0434755
RPL21P78	NC_000008	134014822	134015360	0	0	0	0
SLA	NC_000008	134048973	134115310	0	0	0	0
WISP1	NC_000008	134203312	134241571	2.2146893	0.0968908	0.0419202	0.1422886
NDRG1	NC_000008	134249414	134309547	28.911315	27.177595	58.01683	119.11827
RPL32P20	NC_000008	134338240	134338647	0	0	0	0
FAM10A6	NC_000008	134420107	134421768	0	0	0	0
ST3GAL1	NC_000008	134467091	134584183	0.6619659	0.810203	1.9910538	1.9242141
LOC100129104	NC_000008	134580983	134586104	0	0	0	0
ZFAT	NC_000008	135490031	135725281	0.4213581	0.4094635	0.5853851	1.1221882
ZFATAS	NC_000008	135610314	135612932	0	0	0	0
RPL23AP56	NC_000008	135991070	135991525	0	0	0	0
LOC286094	NC_000008	136246374	136311962	0	0	0	0
KHDRBS3	NC_000008	136469716	136659849	1.0330192	1.1313167	2.1976148	2.8168003
MAPRE1P	NC_000008	136636495	136638273	0	0	0	0
LOC100129367	NC_000008	138708967	138710710	0	0	0	0
FLJ45872	NC_000008	138843911	139095788	0	0	0	0
FAM135B	NC_000008	139142266	139509065	0.0378755	0.00649	0.0280793	0.0219943
COL22A1	NC_000008	139600478	139926236	0.0692534	0.0711998	0.1663464	0.0965171
LOC100288076	NC_000008	139883433	139926249	0	0	0	0
LOC100288108	NC_000008	140618287	140716793	0	0.0217332	0.0188059	0.0147306
KCNK9	NC_000008	140624804	140715299	0	0.0346764	0	0.0235034
TRAPPC9	NC_000008	140742588	141468678	1.1301535	0.7577717	2.8413929	3.6363619
CHRAC1	NC_000008	141521401	141527252	5.4320014	5.7834754	13.574675	17.002932
EIF2C2	NC_000008	141541264	141595410	6.2527452	6.8228646	13.286575	17.838524
PTK2	NC_000008	141668501	142011332	4.5684434	6.7810005	10.164849	11.375341
DENND3	NC_000008	142138720	142205901	1.227063	0.6556739	1.163447	1.9126485
LOC100289278	NC_000008	142178113	142186004	0	0.037066	0.0320735	0.0502459

SLC45A4	NC_000008	142220870	142238673	0.1431963	0.1070697	0.2779448	0.2993546
LOC731779	NC_000008	142350648	142354720	0	0	0	0
GPR20	NC_000008	142366585	142377365	0	0.0591406	0.1023496	0
PTP4A3	NC_000008	142432007	142441620	0.5279067	0.2035288	1.5850364	0.8966744
FLJ43860	NC_000008	142443929	142517330	0	0.0108431	0.0093827	0.0293975
LOC100131146	NC_000008	143049452	143050202	0	0	0	0
NCRNA00051	NC_000008	143279717	143290364	0	0	0	0
TSNARE1	NC_000008	143293441	143436128	0.5523548	0.4691173	0.9827803	1.6902255
BAI1	NC_000008	143545377	143626368	0.0397506	0.0490413	0.0070726	0.0221598
ARC	NC_000008	143692410	143695833	2.3146367	1.4431668	0.4915426	0.4786763
JRK	NC_000008	143738874	143751401	1.7303592	1.2933613	2.8171859	5.4612911
PSCA	NC_000008	143761875	143764143	1.5080276	0.5315694	1.9548783	2.9123659
LOC100289310	NC_000008	143781341	143783411	0	0.4493828	0.3535042	0.3045874
LY6K	NC_000008	143781531	143785611	5.4460345	19.948407	4.3041124	7.3782128
LOC100288181	NC_000008	143796843	143821552	1.0419338	0.4120067	0.7843281	1.7872269
C8orf55	NC_000008	143808621	143818350	3.2029409	1.2972266	2.7630788	1.961399
SLURP1	NC_000008	143822362	143823829	0	0	0	0
LYPD2	NC_000008	143831628	143833952	2.715902	2.8768458	1.3911119	1.2616991
LYNX1	NC_000008	143845756	143859640	0.4197682	0.0616523	0.1066964	0.0835746
LY6D	NC_000008	143866298	143868008	0	0	0	0
LOC100288207	NC_000008	143877674	143887558	0	0	0	0.0534466
GML	NC_000008	143916217	143928262	0	0	0	0
LOC100288248	NC_000008	143939709	143940213	0	0	0	0
CYP11B1	NC_000008	143953773	143961236	0.0124323	0	0.0110601	0.0173267
CYP11B2	NC_000008	143991975	143999259	0	0	0.0133166	0.0208616
LOC100133669	NC_000008	144063448	144099807	0	0	0	0
LOC100128627	NC_000008	144077327	144077974	0	0	0	0
LY6E	NC_000008	144099902	144103827	111.02908	49.486583	166.66335	40.833171
C8orf31	NC_000008	144120679	144135720	1.3360262	1.2289885	0.6881172	0.1469994
RPS26P36	NC_000008	144147332	144147580	0	0	0	0
LOC100288318	NC_000008	144153619	144155050	0	0	0	0
LY6H	NC_000008	144239331	144242053	0.0759693	0	0	0.0264692
GPIHBP1	NC_000008	144295068	144299044	0.0192671	0	0.1371243	0.0537043
ZFP41	NC_000008	144329109	144344875	0.4683159	0.6419706	1.0783282	1.16459
GLI4	NC_000008	144349607	144359101	1.8652637	1.0429527	2.2125205	2.2803335
ZNF696	NC_000008	144373559	144382122	0.2063297	0.2024864	0.3337394	0.5685797
TOP1MT	NC_000008	144391526	144417050	0.4832005	0.3311872	0.4503385	1.0261739
C8orf51	NC_000008	144448793	144450805	0	0	0	0
RHPN1	NC_000008	144451025	144466390	0.0591497	0	0.1999606	0.0989229
MAFA	NC_000008	144511515	144512576	0	0.1276367	0.0736301	0.057674
ZC3H3	NC_000008	144519825	144623620	2.5481123	2.9506402	5.1183569	4.1773941
LOC100289342	NC_000008	144638999	144640441	1.4923516	1.0019881	1.6798677	0.7852533
GSDMD	NC_000008	144640492	144645232	3.4464269	1.8104397	3.4912537	2.6645572
C8orf73	NC_000008	144648362	144654928	0.1216654	0.0833899	0.2525527	0.3485452
NAPRT1	NC_000008	144656955	144660513	2.4113189	1.9459518	5.2591416	4.3543337
EEF1D	NC_000008	144661898	144679845	5.9710637	8.5356735	8.7909311	11.277334
TIGD5	NC_000008	144680221	144682484	3.2029409	3.7719359	4.0410028	4.0174862
PYCRL	NC_000008	144686083	144691764	2.0833248	2.1418768	1.7504177	2.3734857
TSTA3	NC_000008	144694788	144699732	12.168288	18.599059	22.773324	17.905857
ZNF623	NC_000008	144718373	144735900	1.1198348	1.3685357	2.3778087	2.7017621
ZNF707	NC_000008	144766622	144777555	0.4806237	0.6324886	0.3933701	0.6698355
BREA2	NC_000008	144779285	144780583	0	0	0	0
LOC100130274	NC_000008	144788864	144789892	0	0	0	0.0656482
MAPK15	NC_000008	144798507	144804633	0	0	0.0414169	0.0486624
FAM83H	NC_000008	144806113	144815914	0.2674494	0.0404362	0.1959427	0.4001461
LOC100127925	NC_000008	144840323	144842682	0	0	0	0
LOC642574	NC_000008	144853300	144872778	0.0504283	0	0	0.0878511
SCRIB	NC_000008	144873090	144897549	2.4088145	2.6583565	4.0311403	3.439283
PUF60	NC_000008	144898547	144911537	27.962916	38.464419	35.309356	38.653991
NRBP2	NC_000008	144915916	144924200	6.0553135	2.9897926	5.0509889	7.6320951
EPPK1	NC_000008	144935822	144947434	0.0302008	0.0496794	0.021494	0.0168361
PLEC1	NC_000008	144989321	145049543	4.8651577	5.4732471	4.3041871	7.2239833
PARP10	NC_000008	145051320	145060635	12.404944	6.0878148	6.9570995	3.11646
GRINA	NC_000008	145064226	145067583	62.594966	42.956374	106.42564	114.57875
SPATC1	NC_000008	145086582	145102015	0	0.0443845	0.0192031	0.0601668
LOC392275	NC_000008	145104023	145106107	0	0.0323895	0.0840808	0.0878133

OPLAH	NC_000008	145106167	145115584	0.7344803	0.6086066	2.0480142	1.3291915
LOC100288424	NC_000008	145115690	145118837	0	0.1132416	0.0979889	0.0767541
EXOSC4	NC_000008	145133522	145135551	5.9416045	10.551208	9.3484682	6.9462014
GPAA1	NC_000008	145137524	145141119	30.383175	27.630295	36.733175	35.588272
CYC1	NC_000008	145149960	145152428	31.308575	38.910004	67.047478	46.823161
SHARPIN	NC_000008	145153536	145159140	16.456386	16.04807	17.352774	21.197947
MAF1	NC_000008	145159305	145162515	19.857173	23.037171	39.295807	63.837774
KIAA1875	NC_000008	145162629	145173218	0	0	0	0
C8orf30A	NC_000008	145192672	145195620	2.2293906	4.0580519	3.0562754	6.3159832
LOC648630	NC_000008	145195767	145198567	0	0	0	0
HEATR7A	NC_000008	145202919	145316843	3.8111063	2.1062357	5.1125009	6.9122476
LOC727967	NC_000008	145316948	145332431	0.2067179	0.2125278	0.1287314	0.2592887
SCXB	NC_000008	145321517	145323045	0	0	0	0
C8orf30B	NC_000008	145437880	145440957	1.7863725	1.4264689	2.8852584	3.7707032
LOC100131992	NC_000008	145440975	145443775	0	0	0	0
LOC377711	NC_000008	145448755	145485896	0.056972	0.0117146	0.0405471	0.0635206
BOP1	NC_000008	145486056	145515120	5.7967958	13.4046	8.0897196	14.273542
SCXA	NC_000008	145490603	145492131	0.0725218	0	0.0645174	0
HSF1	NC_000008	145515270	145538385	15.622861	16.355888	31.903032	42.066515
DGAT1	NC_000008	145538247	145550567	1.5420432	1.902459	3.5456902	3.8684057
SCRT1	NC_000008	145554454	145559943	0.2227106	0.2035288	0.3522303	0.1465718
C8ORFK29	NC_000008	145576886	145578505	0	0	0	0
FBXL6	NC_000008	145579091	145582132	3.4251917	2.8223443	4.6379347	6.8620787
GPR172A	NC_000008	145582225	145584946	19.184832	20.283571	21.70742	18.709936
ADCK5	NC_000008	145597731	145618457	1.1406411	1.172699	1.7907282	2.0416586
CPSF1	NC_000008	145618446	145634733	2.2101247	2.2018614	3.3755798	4.9746687
SLC39A4	NC_000008	145637798	145642273	0.3199297	0.2250514	0.2995982	0.6688192
VPS28	NC_000008	145649000	145653927	12.82858	12.848764	18.591592	16.062201
NFKBIL2	NC_000008	145654163	145669812	3.2121631	3.1223082	6.2694657	6.8236163
LOC100287098	NC_000008	145660557	145663650	0.0732471	0.0753057	0.0651626	0
CYHR1	NC_000008	145675315	145691031	1.4044252	1.7080242	2.2093324	2.6137367
KIFC2	NC_000008	145691738	145699499	1.7996043	0.7929353	2.9010201	5.9684569
FOXH1	NC_000008	145699115	145701718	0	0.0206978	0	0.0982016
PPP1R16A	NC_000008	145722109	145727504	1.4494799	1.0928263	4.1779721	5.9391255
GPT	NC_000008	145729465	145732555	0.3029305	0.2635299	0.4353387	0.6008062
MFSD3	NC_000008	145734552	145736589	3.1000827	2.0935601	3.6772261	3.9604787
RECQL4	NC_000008	145736671	145743200	5.0282696	6.1916235	8.751191	13.91088
LRRC14	NC_000008	145743391	145750557	2.4603744	3.0727769	3.4448165	4.6026494
LOC100287263	NC_000008	145745046	145745934	0.0622496	0.5119932	0.3322739	0.5639142
LRRC24	NC_000008	145747772	145752408	0.3259139	0.2319741	0.2007291	0.4891595
C8orf82	NC_000008	145751603	145754458	1.75156	2.4322328	1.7606047	5.0248894
KIAA1688	NC_000008	145754563	145838891	0.2391276	0.4038936	0.471055	0.7081929
ZNF251	NC_000008	145946294	145980970	1.1692247	1.4725552	2.5354258	2.464656
ZNF34	NC_000008	145998499	146012725	0.3403322	0.3965502	0.6257225	0.7114711
RPL8	NC_000008	146015154	146017805	85.462408	145.00396	178.8463	152.46478
ZNF517	NC_000008	146024261	146034529	0.8846111	0.4450613	1.8418553	1.2984424
LOC100129596	NC_000008	146045697	146046149	0.2910479	0	0.0863081	0
LOC100287297	NC_000008	146052754	146064326	0.0511919	0.0526306	0	0.0178363
ZNF7	NC_000008	146052903	146068606	3.5533444	3.1112516	5.9575589	7.4827557
COMMD5	NC_000008	146075551	146078932	4.408263	3.1227672	3.9934517	9.8149457
LOC100287170	NC_000008	146075551	146078907	1.1456454	2.4538415	4.5014576	0.9313866
ZNF250	NC_000008	146102336	146126846	0.4350626	0.5253884	1.4437347	1.4580984
ZNF16	NC_000008	146155744	146176274	1.6799783	0.9499568	2.3763429	2.8213286
ZNF252	NC_000008	146198975	146228285	0	0	0	0
TMED10P	NC_000008	146220251	146224283	0	0	0	0
C8orf77	NC_000008	146228197	146231432	0	0	0	0
C8orf33	NC_000008	146277824	146281416	4.7693152	6.3018501	8.3364316	8.3437288
LOC100287596	NC_000009	11987	14924	0.023253	0	0.0827462	0.0324073
LOC100287171	NC_000009	13534	29739	4.7278926	4.5415558	5.3862735	5.6253791
FAM138C	NC_000009	34394	35864	0	0	0	0
LOC100287382	NC_000009	47489	93335	0.0378538	0	0.0336758	0.052756
FOXD4	NC_000009	116234	118417	0.0201228	0.0620651	0.0179018	0.0140224
CBWD1	NC_000009	121038	179075	0.5792189	0.5210606	0.5582299	1.0258746
LOC642313	NC_000009	168877	170976	0	0	0	0
C9orf66	NC_000009	213108	215893	0.0157747	0	0	0.0879393
DOCK8	NC_000009	273048	465255	0.0121521	0.0249874	0.0054054	0.0211702

RPL12P25	NC_000009	477781	478396	0	0	0	0
LOC100133062	NC_000009	499448	501483	0	0	0	0
LOC100289539	NC_000009	503029	503991	0	0	0	0
LOC645586	NC_000009	503057	518363	0	0	0	0
KANK1	NC_000009	504703	746103	1.1364522	1.8694278	0.3755215	0.9899065
LOC642350	NC_000009	706912	707998	0	0	0	0
DMRT1	NC_000009	841690	969090	0	0	0	0.0137826
DMRT3	NC_000009	976964	991732	0	0	0	0.0140224
DMRT2	NC_000009	1050620	1057554	0.0825784	0	0	0.0230176
RPS27AP14	NC_000009	1164414	1164867	0	0	0	0
SMARCA2	NC_000009	2015342	2193624	0.6259779	0.5807837	0.8828702	2.4416917
FLJ35024	NC_000009	2535652	2622373	0	0	0	0
VLDLR	NC_000009	2621793	2654485	0.5801747	0.3230936	0.7096918	0.320062
KCNV2	NC_000009	2717526	2729757	0.0233519	0	0.1038724	0.1464527
KIAA0020	NC_000009	2804152	2844130	3.1843678	3.96524	5.1907209	7.3736768
LOC392281	NC_000009	2875442	2876426	0	0	0	0
LOC138234	NC_000009	2900240	2900846	0	0	0	0
CARM1L	NC_000009	2943562	3053404	0	0	0	0
RFX3	NC_000009	3224645	3525983	0.4001356	0.4913724	1.325006	1.2469918
GLIS3	NC_000009	3824128	4300035	0.4979328	0.5642835	0.9413216	2.4525137
C9orf70	NC_000009	3898646	3901248	0	0	0	0
LOC100287493	NC_000009	4294224	4300092	0	0.0666422	0	0
SLC1A1	NC_000009	4490444	4587469	8.7140771	5.2564248	5.1681898	9.0015091
C9orf68	NC_000009	4598316	4666464	0.0791249	0	0.0502798	0.0866445
RPS6P11	NC_000009	4633032	4633770	0	0	0	0
PPAPDC2	NC_000009	4662298	4665258	0.0296847	0.0457785	0.1584501	0.0103427
CDC37L1	NC_000009	4679566	4706594	0.5694117	0.3193173	0.6447184	0.613219
AK3	NC_000009	4711158	4741227	9.1655851	7.3880506	16.27832	7.7547478
RPS5P6	NC_000009	4781417	4782149	0	0	0	0
RCL1	NC_000009	4792834	4861064	2.3503895	2.2081331	3.2265856	4.9841319
LOC100128701	NC_000009	4944346	4945962	0	0	0	0
LOC100287533	NC_000009	4944670	4986126	0	0	0	0
JAK2	NC_000009	4985245	5128183	2.1454388	0.9147822	2.4190925	1.6572782
LOC100129107	NC_000009	5110861	5113421	0	0	0	0
IGHEP2	NC_000009	5113452	5114909	0	0	0	0
INSL6	NC_000009	5163863	5185618	0	0	0	0.086511
INSL4	NC_000009	5231419	5233967	0	0	0.0635733	0
RLN2	NC_000009	5299868	5304580	0.1977423	0	0.0439793	0.0344487
LOC645930	NC_000009	5311445	5311716	0	0	0	0
RLN1	NC_000009	5334969	5339873	0	0	0	0.09501
C9orf46	NC_000009	5357971	5437860	4.6539066	4.5380709	8.8780498	6.8872543
CD274	NC_000009	5450559	5468477	1.1985881	1.4963334	1.2186254	0.5767023
PDCD1LG2	NC_000009	5510545	5571282	5.4889851	6.2412143	5.3197267	3.6729589
KIAA1432	NC_000009	5629327	5776557	2.0336778	2.291234	4.109753	5.3878778
ERMP1	NC_000009	5784572	5833081	0.4443371	0.9221102	4.1359529	3.3199409
LOC100128705	NC_000009	5855723	5856426	0	0	0	0
MLANA	NC_000009	5890909	5909822	0.0865123	0.2371832	0.1282729	0.301426
KIAA2026	NC_000009	5919008	6008003	0.3660319	0.2759675	0.7001091	1.0245136
RANBP6	NC_000009	6011019	6015640	1.5381881	1.8564485	2.2353608	3.2289274
LOC100135064	NC_000009	6196040	6196776	0	0	0	0
IL33	NC_000009	6241678	6257982	0	1.6747252	0.0739364	0.1158278
LOC645969	NC_000009	6278596	6280392	0.0244564	0.0251438	0.0435143	0.2556334
TPD52L3	NC_000009	6328375	6331900	0.532127	0.9118041	1.1045882	1.1884849
UHRF2	NC_000009	6413151	6507051	4.6970355	4.7666555	7.2774811	10.047599
GLDC	NC_000009	6532464	6645692	0.126918	0.0237245	0.0615871	0.0964816
RPL23AP57	NC_000009	6639141	6639611	0	0	0	0
LOC100287623	NC_000009	6662366	6663797	0	0	0	0
RNF2P	NC_000009	6669691	6670633	0	0	0	0
RPL35AP20	NC_000009	6675215	6675662	0	0	0	0
LOC100287652	NC_000009	6715892	6735076	0	0	0	0
SNRPEL1	NC_000009	6748553	6749012	0	0	0	0
KDM4C	NC_000009	6757654	7175648	1.6703537	1.1157593	2.5102367	4.5638105
LOC100287684	NC_000009	6758646	6835560	0.026587	0.0546684	0.0236525	0.0555805
RPL4P5	NC_000009	7476951	7478376	0	0	0	0
LOC392285	NC_000009	7499155	7598374	0	0	0	0
C9orf123	NC_000009	7796490	7799799	2.7920469	2.2709792	3.6472196	3.2139498

PTPRD	NC_000009	8314246	10612509	0.0653343	0.7388762	0.0193744	0.0303517
RPL18AP11	NC_000009	8713276	8713893	0	0	0	0
RPS26P3	NC_000009	9090873	9091312	0	0	0	0
RN7SLP2	NC_000009	9441998	9442310	0	0	0	0
LOC646087	NC_000009	10829629	10830308	0	0	0	0
LOC646114	NC_000009	11011223	11040285	0	0	0	0
LOC100049717	NC_000009	12287323	12289233	0	0	0	0
TYRP1	NC_000009	12693386	12710266	0.0916862	0.031421	0.0543777	0.0851874
C9orf150	NC_000009	12775012	12823059	7.6460121	6.7523151	12.543197	9.0622316
TDPX2	NC_000009	12972559	12973504	0	0	0	0
LOC100130801	NC_000009	13021693	13066017	0	0	0	0
MPDZ	NC_000009	13105703	13250365	0.2599788	0.3887789	0.2523102	1.3175532
LOC100128272	NC_000009	13322922	13323454	0	0	0	0
FLJ41200	NC_000009	13406379	13431400	0.0183118	0.0188264	0.048872	0.0638018
LOC347193	NC_000009	13986174	13987913	0	0	0	0
RPL3P11	NC_000009	14040656	14041886	0	0	0	0
LOC138864	NC_000009	14068931	14069365	0.1010304	0	0	0
NFIB	NC_000009	14081847	14313945	1.4381882	0.9802013	5.5874202	5.2962009
RPL7AP47	NC_000009	14204217	14205093	0	0	0	0
ZDHHC21	NC_000009	14617028	14693472	2.290685	3.3986267	2.7944265	3.8424473
CER1	NC_000009	14719731	14722715	0	0	0	0.0253938
FREM1	NC_000009	14737150	14910234	0.0059599	0.1776944	0.0159063	0.0083062
LDHAL4	NC_000009	14920687	14922431	0	0	0	0
LOC389705	NC_000009	14993325	15019722	0	0	0	0
PSIP1P	NC_000009	15054976	15056598	0	0	0	0
TTC39B	NC_000009	15171561	15307244	4.0002303	2.3261636	3.8807715	1.1351891
RPL7P33	NC_000009	15361372	15362202	0	0	0	0
SNAPC3	NC_000009	15422782	15461627	2.2719603	2.1314303	2.5012336	2.6023725
PSIP1	NC_000009	15464064	15511003	5.8405906	4.969833	19.022299	15.038796
LOC100131433	NC_000009	15524574	15524705	0	0	0	0
FTHL12	NC_000009	15526970	15527884	0	0	0	0
C9orf93	NC_000009	15553097	15971897	0.2823011	0.1658487	0.2242347	0.8501056
HMGN2L	NC_000009	15588353	15588613	0	0	0	0
BNC2	NC_000009	16409501	16870786	3.0565883	2.7125422	3.8413978	5.3687122
LOC100130943	NC_000009	16726812	16727522	0	0	0	0
LOC100287970	NC_000009	17096255	17134976	0	0	0	0
RPL31P42	NC_000009	17108299	17108632	0	0	0	0
CNTLN	NC_000009	17135038	17503917	0.4340919	0.475718	0.6747545	1.5590001
SH3GL2	NC_000009	17578953	17797122	0.0322201	0.0165628	0.1146556	0.0561306
LOC100289413	NC_000009	17578988	17747136	0	0	0.1053843	0.0825468
ADAMTSL1	NC_000009	18474104	18910948	12.603085	8.2038327	20.262099	22.445444
FAM154A	NC_000009	18927893	19033184	0.0236281	0	0.0210202	0.049395
PSMC3P	NC_000009	19026890	19028193	0	0	0	0
RRAGA	NC_000009	19049372	19051023	39.21289	26.010542	30.743183	32.441608
HAUS6	NC_000009	19053141	19102902	5.1033068	5.6684761	11.275884	11.249955
SCARNA8	NC_000009	19063654	19063784	0	0	0	0
ADFP	NC_000009	19115759	19127573	88.051836	50.865557	107.59729	84.883501
RPS6P10	NC_000009	19200321	19201053	0	0	0	0
LOC100288002	NC_000009	19228902	19231241	0	0.1427918	0.1765127	0.2350442
DENND4C	NC_000009	19290749	19374139	5.0625259	4.098224	8.5844292	6.8157034
RPS6	NC_000009	19376254	19380235	185.97153	261.01726	382.43928	504.99649
LOC100288035	NC_000009	19383215	19408981	0	0	0	0
ACER2	NC_000009	19408925	19452500	0.3698308	0.2851687	0.4935177	0.3758313
LOC392288	NC_000009	19460924	19490506	0	0.0398443	0.0344776	0.0270061
SLC24A2	NC_000009	19515978	19786926	0.910229	0.4679056	1.3026654	0.2895644
LOC646505	NC_000009	19895560	19895980	0	0	0	0
LOC100288098	NC_000009	19957366	19958012	0	0	0	0
SMNP	NC_000009	20331406	20332375	0	0	0	0
MLLT3	NC_000009	20344968	20622514	0.9967904	0.6569265	2.2737753	2.7962233
RPL7AP48	NC_000009	20532524	20533396	0	0	0	0
KIAA1797	NC_000009	20658309	20995954	5.2204298	6.1974281	7.5821694	13.521173
PTPLAD2	NC_000009	21006365	21031635	4.655533	1.9464601	6.1849256	6.2720443
IFNNP1	NC_000009	21058770	21059322	0	0	0	0
IFNB1	NC_000009	21077104	21077943	0.7847899	0.2151591	0.3723578	0
LOC100288140	NC_000009	21096510	21106829	0	0	0	0
IFNW1	NC_000009	21140631	21142144	0	0.0596875	0	0

IFNA21	NC_000009	21165636	21166659	0	0	0	0
LOC100130866	NC_000009	21178588	21179187	0	0	0	0
IFNA4	NC_000009	21186617	21187598	0	0	0	0
LOC100130671	NC_000009	21190145	21191243	0	0	0	0
IFNA7	NC_000009	21201468	21202204	0	0	0	0.0415534
IFNA10	NC_000009	21206180	21207142	0	0	0.0405998	0
IFNWP18	NC_000009	21208931	21209356	0	0	0	0
IFNA16	NC_000009	21216372	21217310	0	0	0	0
IFNA17	NC_000009	21227242	21228221	0	0.0461055	0	0.0312499
LOC392292	NC_000009	21230820	21231852	0	0	0	0
IFNA14	NC_000009	21239201	21239978	0	0	0	0
IFNAP22	NC_000009	21278048	21278617	0	0	0	0
IFNA5	NC_000009	21304686	21305255	0	0	0	0.0537279
KLHL9	NC_000009	21331018	21335429	5.8969522	4.7313535	8.7641642	7.3508037
IFNA6	NC_000009	21350317	21350886	0	0	0	0
IFNA13	NC_000009	21367371	21368075	0	0	0	0.0434395
IFNA2	NC_000009	21384254	21385396	0	0	0	0.0267934
LOC100288238	NC_000009	21394887	21402975	0.0229735	0	0	0.0320176
IFNWP12	NC_000009	21403144	21403653	0	0	0	0
IFNA8	NC_000009	21409146	21410184	0.0422986	0	0	0
LOC646581	NC_000009	21420295	21421164	0	0	0	0
IFNA1	NC_000009	21440440	21441315	0.0501692	0	0.0446319	0.0699198
LOC554202	NC_000009	21454267	21559697	0	0	0	0
IFNWP19	NC_000009	21455337	21456166	0	0	0	0
IFNE	NC_000009	21480838	21482312	0	0.0306328	0.0265068	0.0415253
LOC402359	NC_000009	21695173	21700072	0	0	0	0
MTAP	NC_000009	21802635	21865970	2.3036659	2.7264263	5.3141551	7.248676
C9orf53	NC_000009	21967138	21967753	0	0	0	0
CDKN2A	NC_000009	21967751	21994490	51.326223	25.739621	54.434663	40.119105
CDKN2BAS	NC_000009	21994550	22121096	0	0	0	0
CDKN2B	NC_000009	22002902	22009312	0.8935258	0.3062128	0.9813646	0.0614957
UBA52P6	NC_000009	22011949	22012537	0	0	0	0
LOC729983	NC_000009	22154358	22155868	0.0581711	0	0.0258753	0.020268
DMRTA1	NC_000009	22446840	22452472	0.2290959	0.6084646	0.883177	1.3968776
LOC646609	NC_000009	22734319	22748233	0	0	0	0
LOC402360	NC_000009	23681895	23682796	0.0525068	0.0539826	0.0467115	0.1463555
ELAVL2	NC_000009	23690102	23826063	0.0230821	0	0	0
TUSC1	NC_000009	25676387	25678856	2.9180202	3.1097888	3.4823743	4.5875324
C9orf82	NC_000009	26840683	26892725	1.635509	1.7647165	2.4922177	2.3357958
PLAA	NC_000009	26903368	26947468	2.608151	2.4169563	4.2459609	4.654932
IFT74	NC_000009	26947037	27062931	1.2674328	0.5700862	3.2275928	2.605433
LRRC19	NC_000009	26993134	27005691	0	0	0	0
TEK	NC_000009	27109147	27230172	1.55144	5.5351804	0.16431	0.0836569
RPL36AP34	NC_000009	27210325	27210624	0	0	0	0
NCRNA00032	NC_000009	27245682	27282791	0	0	0	0
C9orf11	NC_000009	27284667	27297137	0	0	0	0
MOBKL2B	NC_000009	27325207	27529850	0.0269414	0.2977603	0.005992	0.0281608
LOC100288294	NC_000009	27455144	27529926	0	0.0918362	0	0.0311228
IFNK	NC_000009	27524312	27526496	0	0	0	0
C9orf72	NC_000009	27546544	27573842	0.7433529	1.1463675	1.8945563	1.4069944
LOC392298	NC_000009	27608380	27656933	0	0	0	0
LINGO2	NC_000009	27948528	28719303	0.0157408	0.0485495	0.0140034	0.0219376
LOC646700	NC_000009	28147886	28149234	0	0	0	0
LOC100289571	NC_000009	29214320	29216365	0	0.0883351	0	0.0149682
LOC100288404	NC_000009	29636433	29636858	0	0	0	0
LOC286239	NC_000009	29824741	29826503	0	0	0	0
LOC100288436	NC_000009	30558583	30559468	0.0992059	0.0509971	0.0441282	0.207392
LOC100131855	NC_000009	30670982	30672779	0	0	0	0
RBMXP2	NC_000009	30688946	30690958	0	0	0	0
LOC442405	NC_000009	30773462	30774736	0	0	0	0
KRT18P36	NC_000009	30799856	30800783	0	0	0	0
RPS26P2	NC_000009	30831807	30832247	0	0	0	0
LOC100129251	NC_000009	30934585	30936003	0	0	0	0
LOC100130670	NC_000009	30987881	30990574	0	0	0	0
LOC138412	NC_000009	31253793	31255041	0	0	0	0
LOC100288563	NC_000009	31644643	31645578	0	0	0	0

SLC25A5P8	NC_000009	32333193	32334430	0	0	0	0
ACO1	NC_000009	32384601	32450834	8.6782283	13.033184	17.841706	28.338796
DDX58	NC_000009	32455300	32526322	25.273005	8.0657503	18.110163	5.6987
TOPORS	NC_000009	32540542	32552601	3.6446942	3.3876659	6.7487601	5.4634547
LOC100129250	NC_000009	32551771	32553006	0	0	0	0
NDUFB6	NC_000009	32553523	32573182	0.7647604	0.9959219	2.4039106	1.7408574
TAF1L	NC_000009	32629452	32635667	0.0282807	0.0436133	0.0188695	0.0246339
LOC100288596	NC_000009	32670632	32709391	0	0	0	0
RPS11P4	NC_000009	32727064	32727462	0	0	0	0
TMEM215	NC_000009	32783497	32789199	0.3020778	0.0335749	0.0217895	0.0512027
LOC100288631	NC_000009	32783497	32784585	0	0	0.0504485	0
ASSP12	NC_000009	32945892	32947458	0	0	0	0
LOC100131180	NC_000009	32954489	32958868	0	0	0.0089264	0.0839038
APTX	NC_000009	32972608	33001626	1.9322067	2.3760237	2.578417	5.3065522
LOC646808	NC_000009	33019485	33020130	0	0	0	0
DNAJA1	NC_000009	33025209	33039062	13.401639	16.598584	22.090887	23.516243
SMU1	NC_000009	33041850	33076714	3.7372421	3.9824603	5.8059141	9.1948055
B4GALT1	NC_000009	33110639	33167356	18.818509	24.318764	26.117807	18.860666
SPINK4	NC_000009	33240196	33248565	0	0	0	0
BAG1	NC_000009	33252469	33264761	2.5745158	2.9500389	1.7051583	2.8372468
CHMP5	NC_000009	33264877	33281516	21.641394	13.117762	39.303478	25.82651
NFX1	NC_000009	33290510	33371155	4.4070225	3.496148	5.7181207	5.7806849
AQP7	NC_000009	33384948	33402517	0.0349906	0	0.0311286	0.0243829
AQP3	NC_000009	33441158	33447590	0.5782662	0.3220308	0.3429611	0.1678996
NOL6	NC_000009	33461351	33473941	8.0687976	8.6214363	6.4612091	10.052634
SUGT1P	NC_000009	33499507	33511047	0	0	0	0
ANKRD18B	NC_000009	33523927	33572358	0.0899525	0.2378074	0.2286407	0.4119136
bA255A11.4	NC_000009	33572428	33574633	0	0	0	0
LOC100288689	NC_000009	33602220	33604263	0	0	0	0
TRBV20OR9-2	NC_000009	33617843	33618506	0	0	0	0
ANXA2P2	NC_000009	33624223	33625532	0	0	0	0
TRBV21OR9-2	NC_000009	33629141	33629586	0	0	0	0
TRBV22OR9-2	NC_000009	33633851	33634300	0	0	0	0
TRBV23OR9-2	NC_000009	33638035	33638506	0	0	0	0
TRBV24OR9-2	NC_000009	33649129	33649611	0	0	0	0
TRBV25OR9-2	NC_000009	33662201	33662662	0	0	0	0
PTENP1	NC_000009	33673502	33677418	0	0	0	0
TRBVAOR9-2	NC_000009	33682268	33682721	0	0	0	0
TRBV26OR9-2	NC_000009	33695578	33696059	0	0	0	0
PRSS3	NC_000009	33750515	33799229	0.1293864	4.9661835	0	0.0601077
TRBV29OR9-2	NC_000009	33786219	33786826	0	0	0	0
UBE2R2	NC_000009	33817182	33920401	8.4208638	9.0529006	15.991668	11.358071
UBAP2	NC_000009	33921691	34048947	2.9852791	3.0375396	3.8787267	10.501388
SNORD121B	NC_000009	33934286	33934378	0	0	0	0
SNORD121A	NC_000009	33952762	33952852	0	0	0	0
RNU7-36P	NC_000009	34018264	34018318	0	0	0	0
WDR40A	NC_000009	34086382	34126755	6.1580445	6.67735	14.049016	14.424033
IMPDH1P1	NC_000009	34132457	34134697	0	0	0	0
UBAP1	NC_000009	34179011	34252521	9.8180511	7.2029116	15.067215	15.250853
RPL35AP2	NC_000009	34191517	34191890	0	0	0	0
RPS8P9	NC_000009	34223929	34224649	0	0	0	0
KIF24	NC_000009	34252378	34329198	0.317596	0.4788991	0.7094949	1.2688644
SERPINHP1	NC_000009	34318264	34320329	0	0	0	0
NUDT2	NC_000009	34329504	34343696	9.0384099	8.9088028	18.073403	12.4233
KIAA1161	NC_000009	34368907	34376894	0.7425808	0.8817323	0.8094927	1.0567842
C9orf24	NC_000009	34379017	34397849	0	0	0.1179414	0.1154784
C9orf25	NC_000009	34398182	34458568	1.8831463	1.1566146	2.7631557	6.5016088
DNAI1	NC_000009	34458811	34520982	0	0	0.0465263	0.0242958
ENHO	NC_000009	34521040	34523037	0.8561344	1.2574231	0.7616409	2.3863542
LOC100288715	NC_000009	34551430	34559353	0	0.0319092	0	0.0216277
CNTFR	NC_000009	34551431	34589722	0.0431288	0.0443409	0.0383686	0.0150269
LOC415056	NC_000009	34568010	34583069	0	0	0	0
C9orf23	NC_000009	34610492	34612101	3.8551081	3.5462504	3.8989262	2.7712263
DCTN3	NC_000009	34613548	34620496	15.362856	18.875317	26.318924	17.698793
ARID3C	NC_000009	34621455	34628011	0	0.0729353	0.1577787	0.0988697
LOC100288749	NC_000009	34628803	34638138	1.1204138	1.6990572	2.8158221	2.4398405

SIGMAR1	NC_000009	34634719	34637768	11.093213	10.040756	12.206184	15.219973
GALT	NC_000009	34646635	34650574	5.0038436	3.7568222	5.6815938	4.8632766
IL11RA	NC_000009	34652182	34661884	5.2093334	2.0651379	2.6902895	1.8150355
CCL27	NC_000009	34661893	34662689	0	0	0	0.0686656
LOC730098	NC_000009	34664772	34666038	0	0	0	0
CCL19	NC_000009	34689567	34691274	0	0	0.0571602	0
CCL21	NC_000009	34709002	34710147	0.1001099	0.2573087	0.4007723	0.0348803
C9orf144B	NC_000009	34723050	34729535	0	0	0.0090967	0.0142508
LOC100128556	NC_000009	34809395	34893045	0	0.0491124	0.0424974	0.0998637
C9orf144	NC_000009	34830264	34838583	0	0	0	0
LOC100129969	NC_000009	34889061	34895775	0.0447083	0	0	0.062309
GLULP	NC_000009	34917072	34918387	0	0	0	0
LOC158383	NC_000009	34921184	34923016	0	0	0	0
KIAA1045	NC_000009	34958192	34982541	0.0075164	0.0077276	0.0133735	0.0104754
DNAJB5	NC_000009	34989725	34998428	2.4398846	2.0722043	4.3681418	3.5060205
LOC100128275	NC_000009	35010696	35011269	0	0	0	0
C9orf131	NC_000009	35041102	35045988	0.0127645	0.0918629	0.0567783	0.0800534
VCP	NC_000009	35056065	35072739	60.16546	84.453972	75.935801	96.088631
FANCG	NC_000009	35073835	35080013	4.9610889	5.3581229	10.640006	10.545853
PIGO	NC_000009	35088688	35096579	2.4935167	2.784597	4.4174799	4.9655892
STOML2	NC_000009	35099893	35103154	19.402126	36.522954	40.663925	29.73476
KIAA1539	NC_000009	35104118	35115893	5.9362044	5.237574	4.1964032	6.8066523
LOC730110	NC_000009	35147380	35149181	0	0	0	0.0184933
UNC13B	NC_000009	35161989	35405332	1.0762832	1.0426939	0.810185	1.4807633
LOC158381	NC_000009	35406752	35483026	0	0	0	0
RUSC2	NC_000009	35490007	35561895	9.2382988	7.5812123	8.8186956	13.392773
RPL36AP33	NC_000009	35507531	35507928	0	0	0	0
FAM166B	NC_000009	35561946	35563896	0.0988712	0.050825	0.1319378	0.3444868
RPS29P17	NC_000009	35592753	35593041	0	0	0	0
TESK1	NC_000009	35605281	35610038	6.4629753	6.1597404	6.6820164	7.7292519
CD72	NC_000009	35609976	35618424	0.4826356	0.0875647	0.5303933	0.0791341
LOC100131778	NC_000009	35616588	35617542	0	0	0	0
LOC100287027	NC_000009	35646267	35647473	0.0462127	0	0.0411121	0.0644056
SIT1	NC_000009	35649295	35650947	0.1064981	0	0.0631625	0.0247374
RMRP	NC_000009	35657750	35658014	0	0	0	0
CCDC107	NC_000009	35658311	35661184	6.3193536	6.4477404	6.1329515	8.7404337
C9orf100	NC_000009	35659341	35665278	0.3737293	0.3121893	1.1221199	1.1312405
CA9	NC_000009	35673915	35681156	2.8244365	0.0871145	12.387596	7.3413108
TPM2	NC_000009	35681990	35690053	6.1147794	9.8748985	5.8124716	11.343268
TLN1	NC_000009	35697334	35732392	6.5187619	6.1930297	14.115732	15.777223
CREB3	NC_000009	35732317	35737005	13.462262	15.451698	13.222623	8.835054
GBA2	NC_000009	35736863	35749225	4.9169442	3.1031525	5.825115	6.6745442
RGP1	NC_000009	35749203	35752871	4.4422981	4.2533758	4.6458527	7.0418316
MSMP	NC_000009	35752991	35754272	3.793151	3.2946231	4.4799294	11.074175
NPR2	NC_000009	35792406	35809728	1.6400506	1.7256635	2.5989052	2.5892754
SPAG8	NC_000009	35807782	35812259	0.0753829	0.2906308	0.2514852	0.1707219
HINT2	NC_000009	35812957	35815042	7.6842	4.9202784	7.0759398	7.6562196
C9orf128	NC_000009	35818429	35828744	0.0255216	0.0787167	0.0681142	0.0889224
C9orf127	NC_000009	35829222	35854844	0.7038542	0.4714599	1.4420844	2.0436403
LOC100287099	NC_000009	35858363	35865515	0	0	0	0
OR13E1P	NC_000009	35859022	35859977	0	0	0	0
LOC92973	NC_000009	35860271	35865515	0	0	0	0
OR13J1	NC_000009	35869460	35870398	0	0	0	0
LOC100128136	NC_000009	35874807	35875191	0	0	0	0
HRCT1	NC_000009	35906189	35907138	1.4341002	1.2365984	0.3292427	1.0960483
LOC158376	NC_000009	35909480	35911617	0	0	0	0
LOC392310	NC_000009	35943090	35943931	0	0	0	0
OR2S2	NC_000009	35957105	35958151	0	0	0	0.0292501
bA327L3.4	NC_000009	35970941	35972454	0	0	0	0
OR13C6P	NC_000009	35991336	35992282	0	0	0	0
OR13C7P	NC_000009	36002909	36003864	0	0	0	0
OR2S1P	NC_000009	36013081	36014227	0	0	0	0
OR2AM1P	NC_000009	36021608	36022212	0	0	0	0
RECK	NC_000009	36036910	36124452	5.9168744	6.3289534	2.1179325	2.866744
GLIPR2	NC_000009	36136742	36163903	3.4309029	7.2706165	2.2009251	2.6672756
CCIN	NC_000009	36169389	36171331	0.7916563	1.2557405	0.2817117	0.3940412

CLTA	NC_000009	36190892	36212056	54.585618	50.746183	65.62104	103.77576
GNE	NC_000009	36214438	36277041	4.1460596	4.0142792	7.162038	5.4136303
LOC646993	NC_000009	36303494	36304921	0	0.2882514	0.0623566	0.195374
RNF38	NC_000009	36336397	36401195	2.6944169	1.7465716	6.7271432	5.0711098
MRPS21P4	NC_000009	36410487	36410631	0	0	0	0
MELK	NC_000009	36572905	36677679	8.9193828	9.9666545	15.244684	17.001265
PAX5	NC_000009	36838531	37034476	0.0120406	0	0.0107117	0.0251711
LOC100130458	NC_000009	37036063	37038870	0.0156511	0.0321819	0.0696182	0.0327189
RPL32P21	NC_000009	37046832	37047239	0	0	0	0
LOC100287249	NC_000009	37078810	37079773	0.0911789	0	0.0405576	0.0317685
ZCCHC7	NC_000009	37120469	37358146	0.4744969	0.4541891	1.0189239	1.5506267
GRHPR	NC_000009	37422707	37436986	11.067126	11.524511	25.674596	19.91237
ZBTB5	NC_000009	37438099	37465407	0.8411597	0.767632	2.564464	2.818806
LOC647013	NC_000009	37477127	37478422	0	0.0697275	0.0603357	0.0236303
POLR1E	NC_000009	37485945	37503693	4.9068414	7.94487	7.0645547	12.43826
RPL21P83	NC_000009	37490386	37490930	0	0	0	0
FBXO10	NC_000009	37510889	37576250	1.3544701	1.9851068	1.5126271	2.1889257
TOMM5	NC_000009	37588410	37592636	15.456986	25.755037	42.416764	42.273812
RAB1C	NC_000009	37636685	37637290	0	0	0	0
FRMPD1	NC_000009	37651052	37746901	0.0089398	0.0735287	0	0.0186889
RG9MTD3	NC_000009	37753802	37778969	0.5558215	0.4335084	0.5626776	0.6945023
EXOSC3	NC_000009	37780308	37785067	4.7472722	7.2470923	8.5105994	14.836275
WDR32	NC_000009	37800790	37867666	2.1710371	2.7287007	6.4215837	3.934997
MRPS10P5	NC_000009	37835818	37836396	0	0	0	0
MCART1	NC_000009	37877572	37904350	2.9737051	4.0871026	5.6251483	7.5689692
LOC780814	NC_000009	37878058	37912432	0	0	0	0
LOC100233198	NC_000009	37885679	37887570	0	0	0	0
SHB	NC_000009	37915895	38069210	1.5985157	2.2137691	2.0519566	3.2145695
ALDH1B1	NC_000009	38392702	38398658	2.4843463	2.2106259	3.5801738	2.895443
IGFBPL1	NC_000009	38408991	38424444	0.0402088	0.1240167	0	0.0280191
LOC100130871	NC_000009	38452779	38453503	0	0	0	0
LOC340501	NC_000009	38453817	38479347	0	0	0	0
LOC340502	NC_000009	38486674	38505122	0	0	0	0
C9orf51	NC_000009	38527094	38527909	0	0	0	0
LOC100289137	NC_000009	38541591	38544705	0	0	0	0
ANKRD18A	NC_000009	38567501	38620950	0.0620884	0.0531945	0.1012652	0.1802736
C9orf122	NC_000009	38621085	38623281	0	0	0	0
LOC100131630	NC_000009	38621637	38623273	0	0.0276013	0.0238837	0.0187079
LOC647051	NC_000009	38643042	38643821	0	0	0	0
VN2R3P	NC_000009	39026653	39039601	0	0	0	0
CNTNAP3	NC_000009	39072764	39288300	0.1596895	0.1036911	0.0224312	0.0234269
LOC100133330	NC_000009	39154067	39155662	0	0	0	0
FAM75A1	NC_000009	39355699	39361956	0.0104069	0	0.0092582	0.0072519
FAM74A5	NC_000009	39370916	39372036	0	0	0	0
LOC653501	NC_000009	39443814	39464526	0	0	0	0
LOC100133160	NC_000009	39485392	39485897	0	0	0	0
LOC647069	NC_000009	39631025	39632395	0	0	0	0
LOC100289279	NC_000009	39654226	39817684	0	0	0	0
FAM75A2	NC_000009	39884975	39891210	0.0103995	0	0.0092517	0.0144936
FAM74A1	NC_000009	39900200	39907240	0	0	0	0
VN2R4P	NC_000009	40063114	40076067	0	0	0	0
LOC642373	NC_000009	40291722	40341109	0	0	0	0
VN2R5P	NC_000009	40371834	40384803	0	0	0	0
LOC642389	NC_000009	40479774	40481179	0	0	0	0
LOC642406	NC_000009	40500161	40633719	0.3615994	0.2859709	0.0494906	0.0387657
FAM75A3	NC_000009	40700291	40706537	0	0	0.0092582	0
FAM74A3	NC_000009	40715524	40722679	0	0	0	0
ZNF658	NC_000009	40771402	40792112	0.6332913	0.7184442	0.6605303	0.7304319
LOC642490	NC_000009	40812917	40813628	0	0	0	0
LOC642545	NC_000009	41016328	41030100	0	0	0	0
LOC100133302	NC_000009	41054827	41056158	0	0	0	0
FAM74A2	NC_000009	41306133	41312131	0	0	0	0
FAM75A4	NC_000009	41321107	41327391	0	0	0.0182401	0
FAM75A5	NC_000009	41500679	41506925	0	0	0	0
FAM74A6	NC_000009	41515908	41523018	0	0	0	0
ZNF658B	NC_000009	41588833	41592207	0.0651085	0.0133877	0.0695068	0.0272221

LOC100101119	NC_000009	41630410	41631121	0	0	0	0
LOC389727	NC_000009	41776064	41777434	0	0	0	0
LOC392322	NC_000009	41799252	41845883	0	0	0	0
LOC100133203	NC_000009	41879737	41880662	0	0	0	0
LOC100288841	NC_000009	41884765	41886211	0	0	0	0
LOC100132952	NC_000009	41942388	41944916	0.0173777	0.0178661	0.0154597	0.0363284
LOC100288879	NC_000009	41950682	41955141	0	0	0	0
MGC21881	NC_000009	41952399	41955076	0	0	0	0
KGFLP2	NC_000009	41958802	42019584	0	0	0	0
LOC100132167	NC_000009	42128814	42134324	0.4088208	0.1401036	0.1212328	0
RPL7AP49	NC_000009	42128839	42129412	0	0	0	0
LOC100288972	NC_000009	42250839	42253384	0.2416635	0.7453664	0.0767823	0.3007156
LOC642840	NC_000009	42332161	42338939	0	0	0	0
LOC100289006	NC_000009	42366102	42366815	1.3541472	0.7593849	0.4928265	0.5575958
ANKRD20A2	NC_000009	42368303	42411410	0	0.0257382	0.0111357	0
FAM95B1	NC_000009	42468589	42474238	0	0	0	0
LOC642888	NC_000009	42491255	42492280	0	0	0	0
LOC100132541	NC_000009	42493517	42494809	0	0	0	0
LOC100289166	NC_000009	42502970	42503700	0	0	0	0
LOC728601	NC_000009	42664885	42666950	0	0	0	0
CBWD7	NC_000009	42668222	42715361	0	0.1359308	0.1411464	0.036853
FOXD4L2	NC_000009	42717234	42720342	0.0141358	0	0.0125756	0
LOC100133034	NC_000009	42743177	42752205	0	0	0	0
LOC100132790	NC_000009	42796892	42798049	0	0	0	0
LOC728034	NC_000009	42808132	42810595	0.3380633	0.6082381	0.8270639	0.176682
AQP7P3	NC_000009	42858152	42893138	0	0	0	0
LOC100289631	NC_000009	42998218	42999418	0	0	0	0
LOC100132441	NC_000009	43007105	43008397	0	0	0	0
LOC392334	NC_000009	43009627	43010747	0	0	0	0
LOC100132599	NC_000009	43023230	43023796	0	0	0	0
LOC100132340	NC_000009	43029021	43032891	0	0	0	0
ANKRD20A3	NC_000009	43089972	43133544	0.0110868	0.0113984	0.0098632	0.0077258
LOC100289528	NC_000009	43134817	43135808	0	0	0	0
LOC389747	NC_000009	43162902	43169678	0	0	0	0
LOC100132253	NC_000009	43318596	43319527	0	0	0	0
LOC442416	NC_000009	43354534	43401315	0	0	0	0
LOC100133300	NC_000009	43425619	43427188	0	0	0	0
FAM74A7	NC_000009	43525578	43615527	0	0	0	0
PTS-P1	NC_000009	43617657	43618342	0	0	0	0
FAM75A6	NC_000009	43624502	43630730	0.0104291	0	0.009278	0
LOC389722	NC_000009	43684887	43921535	0.2827552	0.2491732	0.0287482	0.0281479
LOC100132949	NC_000009	43838439	43840031	0	0	0	0
LOC647507	NC_000009	43937229	43998733	0	0	0	0
LOC644587	NC_000009	44017308	44022018	0	0	0	0
LOC100289385	NC_000009	44049449	44049676	0	0	0	0
LOC100289454	NC_000009	44050738	44074818	0.1125435	0	0	0.1568496
LOC653436	NC_000009	44074927	44118705	0	0	0	0
LOC100289484	NC_000009	44120111	44120528	0	0	0	0
LOC643157	NC_000009	44147907	44154668	0	0	0	0
LOC441416	NC_000009	44170442	44178505	0	0	0	0
LOC728832	NC_000009	44243403	44247136	0	0	0	0
LOC728195	NC_000009	44302233	44306442	0	0	0	0
LOC653453	NC_000009	44341480	44345689	0	0	0	0
LOC100289546	NC_000009	44400710	44404453	0.3051961	0.1568868	0.0522136	0.0654378
LOC643198	NC_000009	44471970	44473415	0	0	0	0
LOC100132772	NC_000009	44477528	44478458	0	0	0	0
LOC643860	NC_000009	44492278	44560257	0	0	0	0
LOC100132268	NC_000009	44584983	44586314	0	0	0	0
LOC100289617	NC_000009	44751833	44753014	0	0.0382262	0.0661549	0
LOC100129770	NC_000009	44873610	44873997	0	0	0	0
LOC100287211	NC_000009	44877290	44877652	0	0	0	0
FAM27A	NC_000009	44990236	44991492	0	0	0	0
LOC100287241	NC_000009	44997492	44998761	0.1164192	0	0.0517849	0.1216883
LOC100132439	NC_000009	44998147	44998488	0	0	0	0
LOC648761	NC_000009	45352098	45352823	0	0	0	0
LOC643395	NC_000009	45355446	45356974	0.0287431	0.0295509	0.0511414	0.0200294

LOC100289027	NC_000009	45359303	45365866	0	0.0289637	0	0
LOC728297	NC_000009	45362027	45362866	0.1046386	0	0	0
LOC441420	NC_000009	45369843	45378019	0	0	0	0
LOC100289056	NC_000009	45433234	45439139	0	0	0	0
LOC100289224	NC_000009	45440169	45442708	0.6574932	0.1245212	0.5541387	0.9163349
RPL7AP45	NC_000009	45558047	45565124	0	0	0	0
LOC100132429	NC_000009	45727113	45728283	0.1419975	0.8759302	0.2526499	0.1484243
LOC100132144	NC_000009	45727440	45727852	0	0.1185916	0	0.0803803
LOC100289124	NC_000009	45733588	45733927	0	0	0	0
LOC100132161	NC_000009	45944298	45949828	0.2044104	0.3502589	0.2424655	0.4273239
LOC100287368	NC_000009	46113631	46114039	0	0	0	0.1620364
LOC100133312	NC_000009	46117314	46117656	0	0	0	0
LOC100287333	NC_000009	46386660	46387002	0	0	0	0
LOC100287410	NC_000009	46390277	46390685	0	0	0	0.0816663
LOC643630	NC_000009	46572714	46578224	0.0681368	0.4203107	0.1818491	0.4273239
KGFLP1	NC_000009	46687566	46748386	0	0	0	0
LOC728433	NC_000009	46783425	46787634	0	0	0	0
LOC100132771	NC_000009	46842609	46846357	0.1055039	0.0361564	0.0730016	0.0408441
LOC100133122	NC_000009	46862127	46913973	0	0	0	0
LOC100129331	NC_000009	46918077	46921551	0	0	0	0
LOC442415	NC_000009	46952853	46999505	0	0	0	0
LOC441410	NC_000009	47021237	47022839	0	0	0	0
VN2R6P	NC_000009	47253859	47264888	0	0	0	0
FLJ37512	NC_000009	47299530	47314534	0.0501692	0	0	0
FAM74A4	NC_000009	65487273	65494386	0	0	0	0
FAM75A7	NC_000009	65503366	65509610	0	0	0.0092582	0
LOC643827	NC_000009	65572847	65665647	0.0877614	0.1388123	0.0600577	0.0376343
VN2R7P	NC_000009	65696613	65707642	0	0	0	0
RBPJP1	NC_000009	65972985	65975258	0	0	0	0
LOC100132871	NC_000009	65978712	66019804	0	0	0	0
LOC100132619	NC_000009	66040922	66060135	0	0	0	0
LOC643880	NC_000009	66081965	66083335	0	0	0	0
LOC100131760	NC_000009	66342521	66343811	0	0	0	0
LOC100133295	NC_000009	66345046	66346168	0	0	0	0
LOC100132939	NC_000009	66364745	66369455	0	0	0	0
LOC100132428	NC_000009	66396886	66397113	0	0	0	0.1343196
LOC100287759	NC_000009	66454657	66457040	0.0777845	0.7997062	0	0.1084066
LOC100288109	NC_000009	66456417	66465795	0.4955822	1.1005429	0.0705414	0.3039005
LOC100132947	NC_000009	66486672	66489956	0	0	0	0
LOC100132249	NC_000009	66492599	66494126	0.0287619	0.0591406	0.0255874	0.0601274
LOC442421	NC_000009	66494269	66503030	0	0	0	0
LOC392335	NC_000009	66504408	66515122	0	0	0	0
LOC389740	NC_000009	66516575	66520775	0	0	0	0
LOC100113421	NC_000009	66544359	66548570	0	0	0	0
FLJ20444	NC_000009	66553225	66553724	0	0	0	0
LOC100287847	NC_000009	66563377	66565905	0.0521331	0.0178661	0.0154597	0.0484379
LOC728566	NC_000009	66571620	66666709	0	0	0	0
LOC100132785	NC_000009	66699434	66708473	0	0	0	0.0729164
LOC100132004	NC_000009	66713941	66721306	0	0	0	0
LOC100132427	NC_000009	66948892	66950033	0	0	0	0
LOC653458	NC_000009	66958310	66962510	0	0	0	0
LOC100132938	NC_000009	67032331	67048428	0	0	0	0
AQP7P1	NC_000009	67270215	67289492	0	0	0	0
LOC728611	NC_000009	67327775	67334984	0	0	0	0
LOC100132245	NC_000009	67348384	67349525	0	0	0	0
FAM27E3	NC_000009	67785913	67786254	0	0	0	0
FAM27B	NC_000009	67792167	67794917	0	0	0	0
LOC100132859	NC_000009	67793360	67793778	0	0	0	0
LOC100287110	NC_000009	67924497	67925488	0	0	0	0
ANKRD20A1	NC_000009	67926761	67969840	0	0	0	0
RPL7AP46	NC_000009	68175421	68182483	0	0	0	0
LOC100287168	NC_000009	68298800	68301327	0.1390767	0.196605	0.1855897	0.1090284
LOC441442	NC_000009	68357260	68367209	0	0	0	0
LOC401525	NC_000009	68370182	68371085	0	0	0	0
LOC728683	NC_000009	68371590	68372429	0	0.0537898	0	0.0364582
LOC100130039	NC_000009	68377440	68378113	0	0	0	0

LOC728701	NC_000009	68408651	68410515	1.0132836	1.5505296	0.6918068	2.4631269
LOC642236	NC_000009	68427859	68439246	0	0	0	0
LOC100287354	NC_000009	68454270	68455368	0.0705429	0.4351531	0.1255139	0.1966284
LOC100287409	NC_000009	68726461	68750959	2.7135107	1.8598495	3.2186858	6.8220068
PGM5P1	NC_000009	68773919	68809958	0	0	0	0
PGM5P2	NC_000009	69080240	69147854	0	0	0	0
LOC440896	NC_000009	69174214	69181041	0	0	0	0
FOXD4L6	NC_000009	69199480	69202204	0.0161278	0	0	0
CBWD6	NC_000009	69204538	69262593	0.2300955	0.2102776	0.2729324	0.5166501
LOC100288651	NC_000009	69252417	69254500	0	0	0	0
LOC100288676	NC_000009	69379717	69380708	0	0	0	0
ANKRD20A4	NC_000009	69381981	69425109	0.0249919	0.0128471	0	0
CCDC29	NC_000009	69425668	69448861	0	0	0	0
LOC644576	NC_000009	69451450	69452569	0	0	0	0
LOC100288827	NC_000009	69476438	69480319	0	0	0	0
LOC100132955	NC_000009	69479773	69482111	0	0	0	0
LOC392343	NC_000009	69498582	69499702	0	0	0	0
LOC100132672	NC_000009	69500882	69502210	0	0	0	0
LOC100288927	NC_000009	69509884	69511163	0	0	0	0
AQP7P2	NC_000009	69633349	69651317	0	0	0	0
LOC100133920	NC_000009	69651361	69664948	0	0	0	0
LOC644632	NC_000009	69719831	69722298	0	0	0	0
LOC100132351	NC_000009	69732340	69733826	0.0486691	0	0	0
LOC100288993	NC_000009	69777287	69790637	0	0	0	0.0250408
LOC100289028	NC_000009	69823281	69826241	0	0	0	0
LOC100288765	NC_000009	69830375	69849574	0	0.0251298	0.0326175	0.0596146
LOC100289057	NC_000009	69882431	69891468	0	0	0	0.1458328
LOC100132154	NC_000009	69894100	69901464	0	0	0	0
LOC644684	NC_000009	70085199	70094234	0.2099438	0.8633771	0.2490291	0.3901258
LOC100132263	NC_000009	70097843	70099000	0	0	0	0
LOC644704	NC_000009	70143948	70152857	0	0	0	0
FOXD4L5	NC_000009	70175707	70178815	0	0	0.0125756	0
LOC653510	NC_000009	70181099	70217473	0.3249407	0.1670366	0.1445381	0.0566079
LOC644716	NC_000009	70334349	70337920	0	0	0	0
LOC100132670	NC_000009	70348573	70349730	0.0804913	0	0	0.0560895
LOC100132426	NC_000009	70394456	70403788	0.0457318	0.0470171	0	0
FOXD4L4	NC_000009	70426623	70429731	0	0.0435993	0	0.0098504
CBWD5	NC_000009	70432004	70490171	0.1118276	0.0287426	0.0248712	0.4480739
LOC727836	NC_000009	70480016	70482081	0	0	0	0
LOC644777	NC_000009	70727365	70734146	0	0	0	0
CBWD3	NC_000009	70856839	70914932	0.1273124	0.1308905	0.1585649	0.1774327
FOXD4L3	NC_000009	70917783	70920000	0	0	0	0.0138074
LOC100289174	NC_000009	70938770	70939517	0	0	0	0
LOC572558	NC_000009	70970105	70972757	0	0	0	0
PGM5	NC_000009	70971815	71145977	0.1711585	0.2842575	0.3279604	0.8073665
C9orf71	NC_000009	71151496	71155783	0.0352998	0.0362919	0	0.0245983
LOC347097	NC_000009	71192735	71226900	0	0	0	0
PIP5K1B	NC_000009	71320616	71624091	0.0331185	0.0170246	0	0
FAM122A	NC_000009	71394964	71398609	0	0	0	0
PRKACG	NC_000009	71627445	71629039	0.0275538	0.0283282	0.0245126	0.0576017
FXN	NC_000009	71650344	71688952	1.3939527	2.050172	1.2228754	1.5514806
LOC100131414	NC_000009	71735743	71736641	0	0	0.0661549	0
TJP2	NC_000009	71789089	71870120	0.8783918	1.3055382	0.8748749	1.9826665
LOC100289287	NC_000009	71869184	71879804	0.0687766	0.0707095	0	0.0958525
C9orf61	NC_000009	71939488	72007370	0.0354136	0.0182044	0.0315049	0.0493552
APBA1	NC_000009	72042449	72287275	0.7676256	1.0911545	0.7304071	0.8465565
LOC100130054	NC_000009	72287665	72288629	0.0455422	0	0	0
PTAR1	NC_000009	72324438	72374876	24.053251	16.471195	26.743186	12.608272
C9orf135	NC_000009	72435731	72521148	0	0	0	0
MAMDC2	NC_000009	72658497	72841888	0.8381504	0.1623505	1.5885412	0.2624022
RNU2P3	NC_000009	72803490	72803666	0	0	0	0
RPL24P8	NC_000009	72832073	72832622	0	0	0	0
SMC5	NC_000009	72873878	72969789	0.8030775	0.7650501	0.7996485	1.4940217
KLF9	NC_000009	72999513	73029573	3.5020192	2.7068397	5.1274265	2.5109107
TRPM3	NC_000009	73149949	73736514	0.0785374	0.0269149	0.0232897	0.0456067
RPL35AP21	NC_000009	74204456	74204893	0	0	0	0

TMEM2	NC_000009	74298282	74383800	3.5101991	2.8953951	2.1997251	3.6244682
FAM108B1	NC_000009	74477368	74526148	3.5589979	2.28689	2.4106168	2.865211
C9orf85	NC_000009	74526423	74588373	0.5005493	0.583233	0.8015446	1.0696617
HSPBL2	NC_000009	74622658	74623052	0	0	0	0
C9orf57	NC_000009	74666297	74675521	0	0	0.0261173	0.040915
LOC392350	NC_000009	74722479	74723338	0	0	0	0
GDA	NC_000009	74764293	74867140	0.0242808	0.0499264	0.0144006	0.0056399
LOC100289320	NC_000009	74890369	74890964	0	0	0	0
ZFAND5	NC_000009	74966341	74980163	13.773142	14.083238	8.9881759	8.2981521
LOC100289351	NC_000009	75013516	75085992	0	0	0	0
TMC1	NC_000009	75136717	75451267	0.0411886	0.0282308	0.0122142	0
RPS20P24	NC_000009	75270716	75271226	0	0	0	0
RPS27AP15	NC_000009	75356068	75356520	0	0	0	0
ALDH1A1	NC_000009	75515587	75567969	0.0838707	0.0646709	0.1865342	0.1899444
LOC100133307	NC_000009	75657553	75671444	0	0	0	0
LOC100132782	NC_000009	75735640	75754257	0	0	0	0
ANXA1	NC_000009	75766781	75785307	193.29054	144.56105	462.18409	318.55127
LOC138971	NC_000009	76089154	76090991	0	0	0	0
LOC100130911	NC_000009	77109925	77112545	0.0351867	0.0723513	0.0313031	0.0245195
RORB	NC_000009	77112252	77302117	0	0	0.3146031	0.1359595
TRPM6	NC_000009	77337411	77503010	0.0104328	0.005363	0.013922	0.01454
RNY4P1	NC_000009	77462422	77462515	0	0	0	0
LOC100133316	NC_000009	77550092	77550699	0	0	0	0
C9orf40	NC_000009	77561499	77567802	3.5725659	4.0810815	3.0605406	3.5432655
C9orf41	NC_000009	77597873	77643310	0.9619312	2.2457778	1.3192977	2.1366194
C9orf95	NC_000009	77676116	77703133	5.562601	2.8026586	6.9477652	4.8260496
LOC100289547	NC_000009	77701400	77703166	0	0	0	0
OSTF1	NC_000009	77703398	77762114	12.183668	10.28929	9.7371698	13.92464
LOC100129601	NC_000009	78016805	78017908	0	0	0	0
PCSK5	NC_000009	78505560	78808357	0.2478529	0.2279958	0.0696306	0.0181804
LOC100133206	NC_000009	78901410	78902997	0	0	0	0
LOC100289618	NC_000009	78906657	78974803	0.0373815	0.0128107	0	0.2083916
RFK	NC_000009	79000433	79009444	2.6138402	2.1698715	3.5674543	3.7673012
RPSAP9	NC_000009	79013515	79014954	0	0	0	0
GCNT1	NC_000009	79056582	79122332	1.5567315	0.8069665	1.2161618	1.8869921
LOC392352	NC_000009	79164198	79188979	4.0912768	4.8168492	3.4635982	3.1268644
LOC100008589	NC_000009	79186647	79186950	0	0	0	0
PRUNE2	NC_000009	79226292	79521003	3.2162353	0.8006276	2.5754376	4.0103138
PCA3	NC_000009	79379354	79402465	0	0	0	0
LOC100128790	NC_000009	79568871	79585944	0	0	0	0
LOC100128367	NC_000009	79607066	79631197	0	0	0	0
FOXB2	NC_000009	79634571	79635869	0	0	0	0.0235757
LOC645225	NC_000009	79655332	79655589	0	0	0	0
LOC100286938	NC_000009	79791517	79797827	0	0.2362531	0.0511079	0
LOC100130426	NC_000009	79791679	79792806	0	0	0	0
VPS13A	NC_000009	79792361	80032399	0.9733712	1.0975725	1.8560248	3.7876923
GNA14	NC_000009	80037995	80263232	0.3881418	0	0.0313911	0.0860595
GNAQ	NC_000009	80335189	80646192	11.356498	10.863996	9.7068576	12.20199
SYNGR2P2	NC_000009	80764658	80766126	0	0	0	0
LOC158452	NC_000009	80793179	80794428	0	0	0	0
RPL21P84	NC_000009	80795096	80795612	0	0	0	0
LOC100286976	NC_000009	80833972	80834273	0	0	0	0
CEP78	NC_000009	80850991	80881983	0.9428893	1.5444508	1.5639026	2.9177157
PSAT1	NC_000009	80912059	80945009	12.29265	10.944791	13.777067	11.589305
KRT18P24	NC_000009	81651269	81652664	0	0	0	0
CHCHD9	NC_000009	82006182	82006955	0	0	0	0
TLE4	NC_000009	82186878	82341656	1.3146511	1.0470137	1.1448413	1.4451175
LOC347119	NC_000009	82437273	82481421	0	0	0	0
LOC100128222	NC_000009	82972203	82974730	0	0	0	0
LOC100287067	NC_000009	83178428	83179546	0	0	0	0
RPS19P6	NC_000009	83397247	83397685	0	0	0	0
RPS20P25	NC_000009	84025194	84025548	0	0	0	0
LOC100287111	NC_000009	84103223	84103673	0	0	0	0
TLE1	NC_000009	84198598	84303596	2.8770026	1.6447919	4.80796	3.1477391
FLJ43950	NC_000009	84528352	84534842	0	0	0	0
FLJ43859	NC_000009	84543343	84549913	0	0.009178	0.0079418	0

FLJ44082	NC_000009	84558415	84565009	0	0	0.0079354	0.0124315
LOC100130636	NC_000009	84581478	84586206	0	0	0	0
FLJ46321	NC_000009	84603687	84610171	0	0.0093489	0	0.0063366
FAM75B	NC_000009	84675660	84677004	0	0	0	0
LOC401533	NC_000009	84701072	84703773	0	0	0	0
RPS2P34	NC_000009	84745649	84746581	0	0	0	0
LOC442427	NC_000009	85046804	85048559	0	0	0	0
RPS6P12	NC_000009	85358970	85359707	0	0	0	0
RASEF	NC_000009	85597315	85678043	1.0346378	0.0490946	1.4443867	1.5306893
FRMD3	NC_000009	85862300	86153348	0	0.3394562	0.0309194	0.0968759
C9orf103	NC_000009	86237966	86259038	0.8985698	0.6718721	0.6540485	0.5692357
UBQLN1	NC_000009	86274878	86323168	25.917647	33.779146	30.711475	30.74244
GKAP1	NC_000009	86354336	86432752	0.3988895	0.0723706	1.3985782	1.2099525
KIF27	NC_000009	86451613	86536342	0.1511222	0.3495814	0.4201329	0.4804677
C9orf64	NC_000009	86553226	86571663	1.6875837	2.3011757	2.2440802	2.6242822
HNRNPK	NC_000009	86582998	86595569	18.615875	21.97125	24.456506	35.107292
RMI1	NC_000009	86595637	86618989	3.5701653	4.1874778	5.3009857	5.4574654
SLC28A3	NC_000009	86893092	86983413	0.4177967	0.2249966	0.8141639	0.4713653
NTRK2	NC_000009	87283466	87638505	0.0338404	0.0187339	0.0162106	0.036279
STK33P	NC_000009	88107494	88109009	0	0	0	0
AGTPBP1	NC_000009	88161454	88356944	2.1282918	2.5664254	4.9369635	4.7056557
LOC100129901	NC_000009	88396062	88397435	0	0	0	0
LOC389765	NC_000009	88419923	88465390	0	0	0	0
LOC548599	NC_000009	88513746	88515114	0	0	0	0
LOC100130049	NC_000009	88549491	88551514	0	0	0	0
MAK10	NC_000009	88556057	88637217	3.7712446	5.1186315	4.0318905	4.4260268
GOLM1	NC_000009	88641056	88715083	4.4575285	2.4105289	5.5541827	4.4645449
LOC100287212	NC_000009	88747566	88747781	0	0	0	0
C9orf153	NC_000009	88842226	88874519	0.4298116	0	0.2867792	0.1497549
ISCA1	NC_000009	88879461	88897490	3.1140963	6.0650375	6.7308683	7.3658639
ZCCHC6	NC_000009	88902648	88969369	1.0117355	0.8641416	0.7477487	1.4208815
RPS6P13	NC_000009	88989850	88990662	0	0	0	0
GAS1	NC_000009	89559277	89562104	6.837773	14.011967	14.170794	5.4470699
CDC20P	NC_000009	89626505	89628125	0	0	0	0
LOC494127	NC_000009	89698401	89700287	0	0	0	0
C9orf170	NC_000009	89763559	89774641	0.0128918	0.0132542	0.0573446	0.0539012
LOC100287242	NC_000009	89920231	90038808	0	0	0	0
DAPK1	NC_000009	90112756	90323549	7.0755321	0.3576322	3.3250708	11.599082
RPS29P18	NC_000009	90140187	90140479	0	0	0	0
CTSL1	NC_000009	90340974	90346384	141.7933	94.663708	125.52737	77.008883
LOC649166	NC_000009	90347526	90356868	0	0	0	0
CTSL3	NC_000009	90387830	90401799	0.1337846	0	0.0595092	0
ELF2P3	NC_000009	90431389	90434340	0	0	0	0
LOC729537	NC_000009	90439852	90440863	0	0.0968562	0.0419052	0
CTSLL3	NC_000009	90459068	90462235	0	0	0	0
LOC392364	NC_000009	90473043	90475030	0	0	0	0
C9orf79	NC_000009	90497772	90503814	0.0494133	0.0406417	0.0439595	0.0344332
FAM75C1	NC_000009	90532877	90538572	0	0	0	0
LOC645937	NC_000009	90558608	90559949	0.2315096	0.2776855	0.7208506	0.6721878
CCRK	NC_000009	90581356	90589667	2.1362456	1.4266467	1.413165	3.0917513
RPS10P3	NC_000009	90631177	90631765	0	0	0	0
LOC100287411	NC_000009	90681350	90682940	0	0	0	0
LOC645961	NC_000009	90744217	90749894	0	0	0	0
LOC100129202	NC_000009	90759763	90760765	0	0	0	0
LOC389768	NC_000009	90795373	90797472	0	0	0	0
LOC100129340	NC_000009	90802580	90843430	0	0	0	0
RPSAP49	NC_000009	90939296	90940325	0	0	0	0
SPIN1	NC_000009	91003297	91093623	5.6788674	8.3890682	7.1298097	13.593799
RPL21P85	NC_000009	91099767	91100236	0	0	0	0
NXNL2	NC_000009	91150020	91190701	0.1813545	0.0621505	0.161338	0.1685
LOC286238	NC_000009	91262094	91267075	0.0644402	0	0	0
LOC100128911	NC_000009	91585802	91586256	0	0	0	0
C9orf47	NC_000009	91605778	91611057	0	0	0	0.0125102
S1PR3	NC_000009	91606362	91619925	2.0270939	3.383921	2.909543	1.9804962
LOC100131540	NC_000009	91620686	91625315	0.1328888	0.9173304	0.1942212	0.2910356
SHC3	NC_000009	91628046	91793682	2.007602	4.9161343	1.039138	2.9378517

CKS2	NC_000009	91926113	91931618	37.925962	51.448637	68.309124	94.272668
SECISBP2	NC_000009	91933412	91974561	2.4591742	2.6064841	2.9771437	3.7806311
SEMA4D	NC_000009	91975706	92094611	0.0646964	0.0931205	0.0460445	0.135249
PA2G4P6	NC_000009	92064357	92066679	0	0	0	0
LOC100287518	NC_000009	92087152	92090096	0	0	0	0
GADD45G	NC_000009	92219927	92221470	0	0	0.1100307	0.0861863
LOC100129066	NC_000009	92254698	92334674	0	0	0	0
LOC100287553	NC_000009	92277897	92288460	0	0	0	0
IL6RL1	NC_000009	92843463	92845018	0	0	0	0
OR7E31P	NC_000009	92978508	92979164	0	0	0	0
OR7E116P	NC_000009	92994280	92995505	0	0	0	0
LOC340515	NC_000009	93225396	93268035	0	0	0	0
DIRAS2	NC_000009	93372114	93405108	0.0535041	0.0330047	0.0666382	0.0671108
OR7E109P	NC_000009	93504784	93505730	0	0	0	0
OR7E108P	NC_000009	93513500	93514522	0	0	0	0
SYK	NC_000009	93564012	93660832	0	0.0089242	0	0.0120975
LOC100128909	NC_000009	93868624	93925498	0	0	0.0721357	0.0565035
AUH	NC_000009	93976097	94124206	0.4981538	0.4267954	1.0833079	0.8871186
NFIL3	NC_000009	94171327	94186144	3.3936045	3.2939458	2.4752416	2.8054445
LOC100132701	NC_000009	94183334	94189397	0.0602031	0	0.0535583	0.0419519
LOC780815	NC_000009	94373447	94374912	0	0	0	0
ROR2	NC_000009	94484884	94712444	0.891641	1.0602803	0.009557	0.0299437
RPL21P82	NC_000009	94768306	94768859	0	0	0	0
LOC100289607	NC_000009	94789732	94790106	0	0	0	0
SPTLC1	NC_000009	94793427	94877690	10.187208	12.317741	9.7892778	10.401078
LOC100128076	NC_000009	94895116	94900911	0	0	0	0
LOC392368	NC_000009	94933523	94938359	0	0	0	0
LOC138652	NC_000009	94939445	94952858	0	0	0	0
IARS	NC_000009	94972625	95056038	9.1430762	12.707832	23.854983	30.404026
SNORA84	NC_000009	95054743	95054875	0	0	0	0
NOL8	NC_000009	95059640	95087876	1.8723025	1.9876931	1.6928096	2.6164807
CENPP	NC_000009	95087741	95377446	0.2440141	0.2112608	0.2970592	0.7159527
OGN	NC_000009	95146249	95166937	0	0	0	0
OMD	NC_000009	95176527	95186836	0	0	0	0.0120476
ASPN	NC_000009	95218487	95244788	0.0355856	0.5122005	0	0.0123987
ECM2	NC_000009	95257599	95298252	0.8058797	0.0714249	0.2348573	0.0193645
IPPK	NC_000009	95375466	95432547	1.3980351	1.5913264	1.3414525	1.7396545
LOC100128361	NC_000009	95380332	95383120	0	0	0	0.0109806
RPL21P86	NC_000009	95439179	95439729	0	0	0	0
BICD2	NC_000009	95473645	95527083	0.417837	0.5633841	0.4509383	0.9307748
LOC100287676	NC_000009	95567667	95569952	0	0	0.0342061	0.0267934
LOC100287707	NC_000009	95570039	95571580	0	0	0	0
ANKRD19	NC_000009	95571893	95600739	0	0	0	0
EEF1DP2	NC_000009	95599039	95600023	0	0	0	0
ZNF484	NC_000009	95608351	95640290	1.0423717	1.0559079	1.0500567	1.3672774
LOC642943	NC_000009	95644841	95650973	0	0	0	0
LOC642952	NC_000009	95655950	95656787	0	0	0	0
LOC100287734	NC_000009	95657810	95658671	0	0	0	0
LOC642959	NC_000009	95674709	95683842	0	0	0	0
FGD3	NC_000009	95709601	95798518	0.0116759	0.0360123	0	0.0162725
SUSD3	NC_000009	95820989	95847415	0.2215877	0.6454772	0.0328551	0.0257352
C9orf89	NC_000009	95858450	95875565	14.018807	15.160877	18.55624	18.185634
NINJ1	NC_000009	95883771	95896570	6.4498576	6.5958596	14.192325	18.38449
WNK2	NC_000009	95947212	96082854	0.0128616	0.0066116	0.0171631	0.0403313
C9orf129	NC_000009	96080481	96108696	0.1523336	0.1174612	0.4404405	0.1326901
FAM120AOS	NC_000009	96208782	96215874	4.4822428	5.4252765	3.690584	3.4446066
FAM120A	NC_000009	96214173	96328397	15.488715	18.357751	18.3854	21.147662
PHF2	NC_000009	96338909	96441869	3.1550049	3.5055359	2.0027543	3.4638346
BARX1	NC_000009	96713909	96717608	1.5420432	0	1.8730952	1.6325003
PTPDC1	NC_000009	96793076	96872136	0.7567849	0.6843129	0.5759185	0.5337116
CYCSP24	NC_000009	96800312	96800627	0	0	0	0
ZNF169	NC_000009	97021578	97065291	0.2101012	0.1905937	0.6376993	0.6803615
FAM22F	NC_000009	97080478	97090926	0	0.0176429	0	0.0597909
LOC100286931	NC_000009	97094093	97094919	0.1062835	0.1639059	0.1418291	0.2221878
LOC100287797	NC_000009	97094914	97101169	0	0	0	0
LOC728026	NC_000009	97109174	97110846	0.0788073	0.0810222	0.1402184	0.1098322

HIATL1	NC_000009	97136833	97223202	27.490965	29.893931	19.738462	18.22821
FBP2	NC_000009	97321002	97356075	0.2020608	0	0.0898795	0.070402
FBP1	NC_000009	97365415	97402531	0.4877717	0	0.0216968	0.0679798
C9orf3	NC_000009	97488994	97849441	3.1480648	3.1243842	1.871692	4.0072979
FANCC	NC_000009	97861336	98079991	2.0863925	2.3713414	1.6177129	2.4675969
RPS26P37	NC_000009	97885803	97886150	0	0	0	0
LOC643342	NC_000009	98054620	98057092	0	0	0	0
MT1P1	NC_000009	98175301	98175899	0	0	0	0
PTCH1	NC_000009	98205264	98279247	0.0390694	0.1305443	0.0999271	0.1020943
LOC100130840	NC_000009	98268517	98269918	0	0	0	0
PSMA7P	NC_000009	98415043	98415648	0	0	0	0
LOC100287056	NC_000009	98534605	98537016	0	0.0187328	0.0162096	0.0380906
C9orf130	NC_000009	98568370	98638259	0	0	0	0
LOC100287100	NC_000009	98637284	98637838	0.996764	0.7452932	0.9673624	0.8840171
C9orf102	NC_000009	98637900	98731122	1.2105388	0.8264664	0.9675533	2.4713427
NCRNA00092	NC_000009	98782014	98784037	0	0	0	0
LOC100128771	NC_000009	98903012	98910257	0	0	0	0
HSD17B3	NC_000009	98997589	99064434	0	0	0.0344776	0
SLC35D2	NC_000009	99082988	99145992	1.3784501	1.4449796	2.7892482	2.4484835
ZNF367	NC_000009	99148223	99180669	4.7816246	4.6354462	7.9799567	7.5239307
HABP4	NC_000009	99212414	99253618	1.1444852	0.890893	1.2217989	1.3671821
CDC14B	NC_000009	99262395	99382112	1.6146747	1.3060151	2.2806346	3.2955206
C9orf21	NC_000009	99403533	99417599	12.484885	10.401404	10.819653	10.974956
LOC100287858	NC_000009	99449338	99483602	0.4155861	0	0.5545754	0.4343954
LOC441455	NC_000009	99488103	99489749	0	0	0	0
ZNF510	NC_000009	99518147	99540328	1.2364171	1.4903334	1.5778606	1.1170891
ZNF782	NC_000009	99579273	99616389	0.5040848	0.4500611	0.460249	0.9521167
LOC100132781	NC_000009	99619552	99632670	0.0927178	0	0	0
LOC441454	NC_000009	99671357	99673454	0.1047384	0.2153642	0.204992	0.1021802
LOC100287885	NC_000009	99680997	99687263	0	0	0	0
LOC100288067	NC_000009	99687295	99688122	0.1061551	0.1091387	0.0944386	0.0739731
FAM22G	NC_000009	99690592	99704572	0.2517493	0.2773122	0.3119487	0.5200215
HIATL2	NC_000009	99708327	99775862	0	0	0	0
LOC100128237	NC_000009	99717160	99717743	0	0	0	0
CTSL2	NC_000009	99794937	99801539	0.2350173	0.0302028	0.4442905	0.1637694
TCEA1P	NC_000009	99829898	99833028	0	0	0	0
LOC340508	NC_000009	99837953	99844227	0	0	0	0
LOC100288099	NC_000009	99883186	99893292	0.0168513	0.0346498	0.0449742	0.0117427
LOC100287922	NC_000009	99914279	99983517	0	0	0	0
ZNF322B	NC_000009	99959537	99961910	0.1851231	0.1332282	0.1811597	0.0516005
LOC100287958	NC_000009	99983795	99999969	0	0	0	0
KIAA1529	NC_000009	100000779	100139569	0.1682497	0.1729784	0.0935498	0.1954052
LOC100288141	NC_000009	100069649	100070286	0	0.0977996	0	0
LOC286359	NC_000009	100153118	100158973	0	0	0	0
TDRD7	NC_000009	100174302	100258407	8.2081846	3.7212685	7.4891989	4.841074
TMOD1	NC_000009	100286437	100363530	0.4124271	0.1865681	0.6751081	0.8736827
C9orf97	NC_000009	100362362	100395962	1.6872627	1.8265196	1.8012429	2.8148883
NCBP1	NC_000009	100395705	100436030	2.0663265	2.5190523	1.6784125	3.5741356
XPA	NC_000009	100437191	100459691	1.4659383	1.2303173	1.3307544	1.8137266
KRT18P13	NC_000009	100461154	100463083	0	0	0	0
FOXE1	NC_000009	100615537	100618997	0.0380944	0.966071	0.0112966	0.0088486
C9orf156	NC_000009	100666772	100684852	2.9637021	3.4313033	2.8266162	6.2328884
HEMGN	NC_000009	100689073	100707134	0.019611	0	0	0.0409972
LOC100287994	NC_000009	100745192	100757035	0	0	0	0.2232676
ANP32B	NC_000009	100745489	100778225	2.0927729	3.0178153	2.3453704	2.8030192
NANS	NC_000009	100818959	100845365	6.0606029	7.7977807	3.6259839	9.0145811
TRIM14	NC_000009	100831563	100881488	5.0095027	3.4149706	5.6530471	1.6604986
CORO2A	NC_000009	100883257	100954956	0.2289368	0.3216738	0.2104575	0.1116726
TBC1D2	NC_000009	100961280	101018003	43.299012	21.726719	21.900295	27.365734
LOC100131625	NC_000009	100975331	100997849	0	0	0	0
GABBR2	NC_000009	101050364	101471175	0.0160278	0.123587	0.0213882	0.027922
LOC100130564	NC_000009	101364031	101370518	0	0	0	0
ANKS6	NC_000009	101494291	101558794	1.4512079	1.8586708	2.0295504	2.5410036
LOC100288086	NC_000009	101494291	101498864	0.1457653	0	0.3241921	0
GALNT12	NC_000009	101569981	101612359	3.4437648	2.1508027	0.3722214	0.0224276
LOC100288125	NC_000009	101640379	101654225	0	0	0	0

COL15A1	NC_000009	101706138	101833068	0.5667513	4.2672737	0.0148293	0.0348472
TGFBR1	NC_000009	101867412	101916474	4.7579476	5.5197021	5.6215959	5.5905183
ALG2	NC_000009	101978707	101984246	4.1463526	5.013282	4.37948	3.9498518
SEC61B	NC_000009	101984570	101992901	44.104631	42.369489	39.445409	55.255563
KRT8P11	NC_000009	102067366	102069133	0	0	0	0
NR4A3	NC_000009	102584137	102629173	0.1989452	0.1406189	0.1050861	0.0996424
LOC441461	NC_000009	102648599	102669342	0	0	0	0
STX17	NC_000009	102668915	102736818	2.2394003	1.9425985	2.8468552	2.8860446
ERP44	NC_000009	102741461	102861330	5.0257542	4.8696221	6.6100779	5.9523879
INVS	NC_000009	102861511	103063426	1.1893207	2.0728468	1.5316572	2.9361997
RPS2P35	NC_000009	102879001	102879956	0	0	0	0
NANOGP5	NC_000009	102937281	102939278	0	0	0	0
TEX10	NC_000009	103064369	103115174	9.9697378	12.730244	14.946027	17.631585
C9orf30	NC_000009	103189642	103213513	12.085764	24.656115	15.03234	17.79411
TMEFF1	NC_000009	103235717	103339914	0.423134	0.711861	0.547537	0.6701286
MURC	NC_000009	103340336	103350180	0	0.1030017	0.0594188	0.1512627
LOC644160	NC_000009	103493522	103494637	0	0	0	0
LOC392374	NC_000009	103737870	103739019	0	0	0	0
PRG-3	NC_000009	103791031	104087417	0	0.0337694	0.0292209	0
LOC347275	NC_000009	104096374	104101629	0	0	0	0
LOC100288173	NC_000009	104118748	104119126	0	0	0	0
LOC100129070	NC_000009	104122340	104124906	0	0	0	0
BAAT	NC_000009	104122699	104147287	0.0373709	0.0512283	0.0443283	0.0954857
MRPL50	NC_000009	104152249	104160919	4.4766074	7.0363966	5.6674238	5.429092
ZNF189	NC_000009	104161163	104172942	2.5272894	2.5436177	1.7868439	1.6035424
ALDOB	NC_000009	104182842	104198062	0.0181155	0	0.0161161	0.0378708
C9orf125	NC_000009	104237608	104249475	5.9326957	6.22921	4.7164129	3.1812344
RNF20	NC_000009	104296133	104325626	1.7512366	2.1674271	2.1037269	3.9874524
GRIN3A	NC_000009	104331634	104500862	0.0622096	0.0465149	0.0201249	0.0630547
PPP3R2	NC_000009	104353897	104357283	0	0	0	0
LOC100131629	NC_000009	104817534	104818032	0	0	0	0
LOC100127962	NC_000009	105277486	105280590	0	0	0	0
LOC100131879	NC_000009	105373377	105542494	0	0	0	0
CYLC2	NC_000009	105757663	105780770	0	0.0215057	0	0.043729
SMC2	NC_000009	106856541	106903700	3.0956364	4.5507426	4.6356317	8.1357246
LOC347281	NC_000009	106987575	107040107	0	0	0	0
LOC100128906	NC_000009	107090601	107091677	0	0	0	0
OR13F1	NC_000009	107266544	107267503	0	0	0.0407266	0
OR13C4	NC_000009	107288534	107289490	0.0459229	0	0	0
OR13C3	NC_000009	107298051	107299094	0	0	0	0
OR13C8	NC_000009	107331449	107332411	0	0	0	0
OR13D2P	NC_000009	107352198	107353331	0	0	0	0
OR13C5	NC_000009	107360738	107361694	0.0459229	0	0	0.0320009
OR13C2	NC_000009	107366952	107367908	0.0459229	0	0	0
OR13C9	NC_000009	107379529	107380485	0	0	0	0.0320009
OR13I1P	NC_000009	107391935	107393399	0	0	0	0
OR13C1P	NC_000009	107419044	107419997	0	0	0	0
OR13D1	NC_000009	107456703	107457743	0	0	0	0.0294187
OR13D3P	NC_000009	107484516	107485649	0	0	0	0
NIPSNAP3A	NC_000009	107509969	107522403	8.4236354	6.5022042	7.2065935	7.3702248
NIPSNAP3B	NC_000009	107526451	107536291	0.1505076	0.1547377	0.4240033	0.1922795
LOC286367	NC_000009	107536633	107540045	0	0	0	0
ABCA1	NC_000009	107543283	107690436	2.4143669	3.3371145	5.2720882	7.8268153
SLC44A1	NC_000009	108006929	108153682	5.6726132	8.0514036	11.686211	10.759181
FSD1L	NC_000009	108210315	108311385	0.2909837	0.2991618	0.6471678	1.0476398
GARNL2P	NC_000009	108282828	108289987	0	0	0	0
FKTN	NC_000009	108320411	108403399	3.6250218	3.3875431	4.4152562	6.0420059
TAL2	NC_000009	108424738	108425393	0	0	0.0595999	0.0933685
TMEM38B	NC_000009	108456825	108537444	7.3035356	7.7264488	10.697214	6.1957846
DEPDC1P2	NC_000009	108520329	108523986	0	0	0	0
LOC100129319	NC_000009	108611991	108612884	0	0	0	0
LOC644620	NC_000009	109376976	109460799	0.1010304	0	0	0.070402
LOC100288437	NC_000009	109477943	109485211	0	0	0	0
LOC100288311	NC_000009	109621022	109625013	0.0678213	0	0	0.0472606
ZNF462	NC_000009	109625378	109773807	0.4338737	0.620143	0.8802365	0.9475793
RPL7AP44	NC_000009	109741021	109741857	0	0	0	0

LOC100128086	NC_000009	109879110	109880203	0	0	0	0
LOC644661	NC_000009	109931857	109975989	0	0	0	0
RAD23B	NC_000009	110045544	110094470	3.2774953	4.3432955	3.9476235	5.2277335
LOC100128786	NC_000009	110218971	110225106	0	0	0	0
KLF4	NC_000009	110247133	110252047	5.8138131	7.7642123	3.0126321	1.8063771
LOC100288528	NC_000009	110290888	110297375	0	0	0	0
LOC100288564	NC_000009	110305426	110306678	0	0	0	0
RPL36AP6	NC_000009	110596738	110597137	0	0	0	0
RPS15AP27	NC_000009	110725942	110726356	0	0	0	0
RPL31P43	NC_000009	110802466	110802911	0	0	0	0
LOC100128657	NC_000009	110859536	110860131	0	0	0	0
RPL36P14	NC_000009	111389046	111389479	0	0	0	0
RPL36AP35	NC_000009	111583977	111584298	0	0	0	0
LOC100288416	NC_000009	111598613	111598877	0	0	0	0
ACTL7B	NC_000009	111616868	111618275	0.0624265	0	0	0
ACTL7A	NC_000009	111624603	111626035	0	0.0315306	0	0
IKBKAP	NC_000009	111629800	111696608	3.5509366	4.988354	3.4659602	5.4397478
LOC100288597	NC_000009	111693291	111696642	0.0803441	0.2478066	0.0714764	0.1679609
C9orf6	NC_000009	111696673	111703238	2.7582572	3.3084081	5.0303353	4.8852447
CTNNAL1	NC_000009	111704851	111775764	7.5493895	7.8539656	12.664733	14.016049
C9orf5	NC_000009	111777415	111882225	5.2153147	5.5709434	8.7269169	7.9386486
C9orf4	NC_000009	111899581	111929571	0.739247	0.2660083	0.0657655	0.0257568
EPB41L4B	NC_000009	111934254	112083021	0.7231915	0.2655417	1.0110119	0.9958985
PTPN3	NC_000009	112137974	112260593	0.6086125	0.1129115	0.854903	1.0554805
LOC402375	NC_000009	112295110	112297579	0	0	0	0
PALM2	NC_000009	112403072	112713756	0.103264	0.130295	0.0250545	0.0261667
PALM2-AKAP2	NC_000009	112542577	112934792	4.1499195	5.4984456	2.7663127	11.738383
RPL21P87	NC_000009	112787121	112787678	0	0	0	0
AKAP2	NC_000009	112810976	112934792	0.5998681	0.1038699	0.1404367	0.7480215
C9orf152	NC_000009	112961841	112970413	0.0990941	0.0339597	0.0587712	0
TXN	NC_000009	113006310	113018778	79.245237	90.722579	153.31171	115.38586
TXNDC8	NC_000009	113065867	113100125	0	0.0918362	0	0.0622457
SVEP1	NC_000009	113127528	113342160	1.3173077	0.2598851	0.4402594	0.210881
MUSK	NC_000009	113431087	113563278	0.3557252	0.0166238	0.1150775	0.3380229
RPS21P5	NC_000009	113489729	113489959	0	0	0	0
LPAR1	NC_000009	113636054	113800365	31.934605	14.561949	24.843245	19.585228
LOC100288449	NC_000009	113734353	113801539	2.6354693	1.6110775	1.2673441	1.489054
OR2K2	NC_000009	114089763	114090713	0	0.0475115	0	0.0322028
KIAA0368	NC_000009	114122973	114247025	10.625827	10.282571	15.552272	23.468991
ZNF483	NC_000009	114287447	114340124	0.3717982	0.078187	0.3232446	0.3474078
PTGR1	NC_000009	114325252	114361711	5.2016023	6.075385	10.167885	7.964441
C9orf29	NC_000009	114365359	114375833	0	0	0	0
DNAJC25	NC_000009	114393632	114416631	1.6277123	2.1914348	2.2927585	1.8769216
DNAJC25-GNG10	NC_000009	114393666	114432509	0.7284237	0.6552841	0.4050162	1.2055373
GNG10	NC_000009	114423861	114432526	0.8471948	0.6895459	1.695798	1.500496
C9orf84	NC_000009	114448901	114545779	0.0615645	0.0271263	0.1330115	0.1716023
UGCG	NC_000009	114659206	114695439	56.700468	20.563002	92.955239	54.309115
SUSD1	NC_000009	114803061	114937556	2.405711	1.7795866	1.6573401	1.4924006
RPL29P20	NC_000009	114935694	114936293	0	0	0	0
ROD1	NC_000009	114980715	115095912	5.9023144	5.9244627	11.291247	12.406676
EPF5	NC_000009	115043268	115043688	0	0	0	0
RPL32P22	NC_000009	115124895	115125396	0	0	0	0
HSDL2	NC_000009	115142351	115234684	7.9818751	6.1652118	14.360134	18.537115
C9orf147	NC_000009	115241310	115254860	0	0	0	0
KIAA1958	NC_000009	115249248	115422706	0.8681537	0.724147	1.7632521	2.579658
C9orf80	NC_000009	115448786	115480387	3.4028158	4.5657768	5.0539504	5.1242414
SNX30	NC_000009	115513134	115637267	2.0107789	3.5108323	2.051003	3.374132
SLC46A2	NC_000009	115641200	115653193	0.0878262	0.0541767	0.0156265	0.0612008
LOC100129193	NC_000009	115721414	115724541	0	0	0	0
LOC169834	NC_000009	115759400	115774472	0.9421437	1.4434378	0.8874605	1.1456974
ZFP37	NC_000009	115804174	115818996	0.3034217	0.722409	0.809797	0.6788218
C9orf110	NC_000009	115867003	115873957	0	0	0	0
C9orf109	NC_000009	115875176	115882126	0	0	0	0
SLC31A2	NC_000009	115913238	115926422	8.4229982	6.214628	5.1792153	6.4564287
FKBP15	NC_000009	115927800	115983641	2.5744582	2.4071416	2.9214971	5.389018
SLC31A1	NC_000009	115983808	116026772	5.9879236	5.900888	8.3546703	6.7108095

CDC26	NC_000009	116029288	116037869	1.7135778	1.502659	3.0040561	5.2329207
PRPF4	NC_000009	116037974	116055056	1.7032953	3.3206083	1.7583183	2.6873751
RNF183	NC_000009	116059373	116061320	0	0.0373417	0.0969361	0.0253098
WDR31	NC_000009	116077930	116102567	0.411735	0.2944743	0.4777707	0.3867093
BSPRY	NC_000009	116111812	116133513	0.0187173	0	0.0333029	0.013043
HDHD3	NC_000009	116135698	116138341	2.0684525	1.8640451	1.4993837	2.1175831
ALAD	NC_000009	116148592	116163618	2.554327	1.9589411	1.0317925	1.6260146
POLE3	NC_000009	116169515	116173029	2.1702831	2.071902	2.9995487	7.5481953
C9orf43	NC_000009	116173022	116191882	0.1287546	0.1323732	0.0763624	0.0448607
RGS3	NC_000009	116207011	116360018	9.4790304	1.8891561	3.0552019	4.7288415
LOC100288542	NC_000009	116521274	116521699	0	0	0	0
LOC100132609	NC_000009	116612967	116633114	0	0	0	0
ZNF618	NC_000009	116638562	116818875	1.3599694	2.3104867	1.4844461	1.4752904
AMBP	NC_000009	116822407	116840752	0.0306473	0	0.0272647	0.0427125
KIF12	NC_000009	116853918	116861337	0.0877648	0.0225579	0	0.0152895
COL27A1	NC_000009	116918231	117072975	0.3148718	0.4046516	0.0630267	0.0383977
ORM1	NC_000009	117085303	117088759	0	0	0	0
ORM2	NC_000009	117092069	117095536	0.0520714	0.0535348	0	0.0362854
AKNA	NC_000009	117096429	117156685	0.3986099	0.330297	0.7780347	1.1027775
DFNB31	NC_000009	117164360	117267730	0.2960901	0.2500525	0.3292625	0.4052859
LOC100131877	NC_000009	117253842	117254022	0	0	0	0
LOC100288716	NC_000009	117258477	117267753	0	0.0697813	0	0.0709457
ATP6V1G1	NC_000009	117349994	117361152	12.821644	13.181998	13.420828	9.0106718
C9orf91	NC_000009	117373706	117408703	0.5044563	1.0061501	1.3283838	1.0545757
LOC100129633	NC_000009	117416580	117508476	0.0983182	0.0336938	0.0291555	0.068512
TNFSF15	NC_000009	117551600	117568408	0.7236984	0	0.3706855	0.0458456
LOC645266	NC_000009	117607874	117610871	0	0	0	0
TNFSF8	NC_000009	117665124	117692770	0	0.0228314	0.0197562	0
TNC	NC_000009	117782805	117880486	9.993755	30.19362	18.249647	17.466538
DEC1	NC_000009	117904097	118164923	0.1053914	0.1083535	0.0937592	0.1224016
C9orf27	NC_000009	118650544	118687377	0	0	0	0
PAPPA	NC_000009	118916071	119164600	2.896497	19.131896	2.7799545	6.3176025
PAPPAS	NC_000009	119158786	119164600	0	0	0	0
ASTN2	NC_000009	119187507	120177317	0.2057418	0.156344	0.1432437	0.2368706
TRIM32	NC_000009	119449581	119463579	0.0472689	0.1700908	0.0525646	0.0658777
RPL10P3	NC_000009	119942907	119943374	0	0	0	0
SNORA70C	NC_000009	119943351	119943478	0	0	0	0
RPL35AP22	NC_000009	120447536	120447863	0	0	0	0
TLR4	NC_000009	120466460	120479768	6.8025301	3.213113	3.6656129	3.8409762
LOC100129489	NC_000009	120631980	120659499	0	0	0	0.0476281
LOC389787	NC_000009	120845142	120845660	0.508072	0	0.150665	0.7080897
LOC442434	NC_000009	121497699	121526555	0	0.0475115	0.0411121	0
DBC1	NC_000009	121928908	122131739	0.1100081	0.2968872	0.0244666	0.0191645
CDK5RAP2	NC_000009	123151147	123342437	0.8747321	1.1531558	1.2174844	2.6299053
MEGF9	NC_000009	123363091	123476612	1.6959537	0.9902032	1.0927698	1.1963983
LOC392387	NC_000009	123482951	123484250	0	0	0	0
FBXW2	NC_000009	123519254	123555740	2.8896943	3.6792058	3.9625336	6.4110212
LOC100288725	NC_000009	123550065	123555641	0.7134453	0.4400981	0.5077606	0.3977257
LOC100288842	NC_000009	123558882	123562196	0.9766274	1.3722367	0.8688348	0.8166634
PSMD5	NC_000009	123578331	123605206	7.9410445	10.618833	8.1611984	10.298712
LOC253039	NC_000009	123605320	123616651	0	0	0	0
PHF19	NC_000009	123617931	123639606	9.7502002	11.749469	8.3394413	16.384343
LOC100288880	NC_000009	123620393	123622824	0	0	0	0.0963046
TRAF1	NC_000009	123664672	123689173	2.0250365	0.6296631	2.9615373	1.7966045
C5	NC_000009	123714613	123812554	0.4503387	0.4133889	0.5794882	0.5379668
CEP110	NC_000009	123850574	123939886	0.4139922	0.4134667	0.4735272	0.9025499
RAB14	NC_000009	123940415	123964365	7.393537	8.2434077	8.0112821	7.8545127
LOC645482	NC_000009	123998802	124002356	0	0	0	0
GSN	NC_000009	124030380	124095120	4.1665564	5.683269	3.3152191	1.9643617
LOC100128064	NC_000009	124042136	124049119	0	0	0	0
C9orf31	NC_000009	124043705	124044328	0	0	0	0
STOM	NC_000009	124101353	124132545	45.592359	23.047357	19.854034	25.524053
GGTA1	NC_000009	124217319	124262306	0	0	0	0
LOC100132863	NC_000009	124282095	124282709	0	0	0	0
DAB2IP	NC_000009	124329399	124547809	3.6861749	3.0936941	1.3251143	1.5307197
TTLL11	NC_000009	124584204	124855885	0.1739048	0.1660215	0.1657613	0.1731197

NDUFA8	NC_000009	124906337	124922098	7.0296342	10.332223	9.7743914	11.393616
MORN5	NC_000009	124922190	124962367	0.1246758	0	0	0
LHX6	NC_000009	124964858	124991019	0.0130798	0.0268949	0.0465447	0.0091145
RBM18	NC_000009	125001834	125027143	2.1916817	2.3416431	2.4977584	2.4855263
MRRF	NC_000009	125027147	125085743	3.6644442	5.1866726	5.9171073	11.385948
PTGS1	NC_000009	125133229	125157981	0.1380663	2.7236019	0.0307069	0.0420919
OR1J1	NC_000009	125239237	125240205	0	0	0	0
OR1J2	NC_000009	125273081	125274022	0	0	0	0
OR1J4	NC_000009	125281420	125282361	0	0	0.0415048	0
OR1N1	NC_000009	125288637	125289572	1.2207842	0.5792744	1.2113562	2.5520732
OR1N2	NC_000009	125315449	125316441	0	0	0.0393732	0
OR1L8	NC_000009	125329827	125330756	0	0	0.0420404	0.03293
OR1H1P	NC_000009	125370043	125371176	0	0	0	0
OR1Q1	NC_000009	125377017	125377961	0	0	0.0413731	0
OR1B1	NC_000009	125390858	125391814	0	0	0	0
OR1L1	NC_000009	125423845	125424927	0	0	0.0361012	0
OR1L3	NC_000009	125437409	125438383	0.0450751	0	0.0401001	0.0314101
LOC100129793	NC_000009	125448219	125450710	0	0	0	0
OR1L4	NC_000009	125486269	125487204	0.0469532	0	0	0
OR1L6	NC_000009	125512019	125513062	0.042096	0.0432791	0	0.0586683
LOC729012	NC_000009	125524141	125524933	0.1108404	0.0569778	0.2465168	0.077238
OR5C1	NC_000009	125551212	125552174	0	0	0	0
OR1K1	NC_000009	125562402	125563352	0	0	0	0
PDCL	NC_000009	125580623	125590840	4.4541044	5.3699559	5.003006	6.1071121
LOC100133030	NC_000009	125599833	125601106	0	0	0	0
RC3H2	NC_000009	125611732	125667562	3.1615927	2.9706096	4.0326932	3.1223077
SNORD90	NC_000009	125642492	125642598	0	0	0	0
ZBTB6	NC_000009	125670335	125675609	0.9419007	0.8473263	1.1997792	1.1411608
ZBTB26	NC_000009	125680378	125693779	0.8973723	0.9462493	1.0849063	1.1384117
RABGAP1	NC_000009	125703288	125867147	2.0747715	2.0698138	1.6111419	2.0032677
RPS25P8	NC_000009	125791611	125792096	0	0	0	0
GPR21	NC_000009	125796846	125797895	0	0	0	0
C9orf45	NC_000009	125871773	125877756	0	0	0	0
STRBP	NC_000009	125886987	125967377	0.0821718	0.0281604	0.1462047	0.4771717
CRB2	NC_000009	126118448	126141032	0.0155817	0.0320393	0.0277239	0.0217159
DENND1A	NC_000009	126141933	126692417	1.753668	1.8619302	2.1068798	2.8380691
LHX2	NC_000009	126773889	126795442	0.072762	0.074807	0.0647311	0.1394345
NEK6	NC_000009	127020243	127114721	9.9134951	10.799375	8.4784768	12.759439
PSMB7	NC_000009	127115744	127177721	69.89734	96.841255	100.32658	127.04345
GPR144	NC_000009	127213423	127239379	0.0151965	0	0	0.021179
NR5A1	NC_000009	127243515	127269699	0	0	0.0378975	0.009895
NR6A1	NC_000009	127284703	127533576	0.0231063	0.4751146	0.4933447	0.5152451
LOC100288920	NC_000009	127534006	127536095	0.0420557	0.0216189	0.0374139	0
OLFML2A	NC_000009	127539437	127577161	1.807104	1.6161596	1.4761692	1.3762938
WDR38	NC_000009	127615755	127620160	0.2106145	0.1443559	0.1561404	0.1712254
RPL35	NC_000009	127620158	127624240	147.13104	198.21762	256.25904	274.22581
ARPC5L	NC_000009	127631484	127639696	8.7038937	13.136248	16.05861	15.984693
GOLGA1	NC_000009	127640573	127703386	2.9976979	3.2207751	2.0261541	3.2494238
C9orf126	NC_000009	127704887	127905797	0.3466746	0.5049254	0.4497666	0.8279034
PPP6C	NC_000009	127908852	127952218	5.7082186	6.0971625	6.3005502	5.9208098
NDUFB3P2	NC_000009	127940692	127941195	0	0	0	0
RABEPK	NC_000009	127963057	127996438	7.7591784	12.328479	7.503022	10.514613
HSPA5	NC_000009	127997127	128003661	9.221924	13.40118	14.347278	12.99916
GAPVD1	NC_000009	128024111	128127290	5.51187	6.8014498	10.675355	11.513781
RPS10P17	NC_000009	128025815	128026380	0	0	0	0
RNU5B-5P	NC_000009	128188068	128188155	0	0	0	0
MAPKAP1	NC_000009	128199673	128469513	8.03278	10.43635	11.179808	21.237561
LOC51145	NC_000009	128506656	128508825	0	0	0	0
PBX3	NC_000009	128509617	128729656	3.0855763	3.3905741	2.8079733	2.7123483
FAM125B	NC_000009	129089128	129269319	0.3513776	0.2408354	0.5788802	0.6121348
LMX1B	NC_000009	129376748	129463311	0.0535022	0.0314319	0.0815949	0.1438038
ZBTB43	NC_000009	129567285	129600489	0.4340384	0.4386738	0.7264529	0.9688822
ZBTB34	NC_000009	129622944	129648157	0.4084159	0.4405451	1.1376653	1.059087
RALGPS1	NC_000009	129677053	129985443	0.1942157	0.2210678	0.6540944	0.4156786
ANGPTL2	NC_000009	129849628	129885044	6.6931238	6.1222751	12.324709	5.0927149
GARNL3	NC_000009	130026756	130155828	0.0470286	0.0725255	0.1778113	0.1556642

SLC2A8	NC_000009	130159465	130170161	0.3121323	0.4064795	0.9996537	1.2180349
ZNF79	NC_000009	130186653	130207651	1.4326365	1.6245231	0.8996564	1.3947092
RPL12	NC_000009	130209955	130213684	150.99805	172.06928	301.31769	181.46818
SNORA65	NC_000009	130210781	130210916	0	0	0	0
LRSAM1	NC_000009	130213792	130265778	1.5859636	1.3745025	2.1338665	2.6487369
FAM129B	NC_000009	130267618	130341268	71.934186	59.592149	43.681821	50.736511
STXBP1	NC_000009	130374568	130454995	0.7805477	1.1165011	1.4491762	0.9695907
C9orf117	NC_000009	130469271	130476303	0.4849108	0.3935836	0.3178664	0.7825172
PTRH1	NC_000009	130476227	130477936	2.9119074	2.7719878	1.8709265	1.540638
TTC16	NC_000009	130478358	130493879	0.0152386	0	0.0135567	0.0318567
TOR2A	NC_000009	130493803	130497604	0.7531981	1.0084777	1.3712976	1.4158971
SH2D3C	NC_000009	130500596	130541048	0.0455776	0.0351439	0.1013678	0.0238202
CDK9	NC_000009	130548305	130553052	2.6134264	2.3761498	2.7045646	2.601628
FPGS	NC_000009	130565154	130576556	16.272233	14.388105	11.980328	11.040136
ENG	NC_000009	130577291	130617047	24.779322	16.625682	13.664575	25.642107
AK1	NC_000009	130628759	130640022	5.4304964	3.4495713	5.5730422	7.2575285
ST6GALNAC6	NC_000009	130647601	130661871	7.7340091	8.8746382	7.1249525	12.732695
ST6GALNAC4	NC_000009	130670165	130679305	5.9775833	5.4775857	4.9478883	7.7875208
PIP5KL1	NC_000009	130683808	130693076	0.7976553	0.1851779	0.2517993	0.3227446
DPM2	NC_000009	130697382	130700135	11.928806	9.7318096	13.748595	23.052793
FAM102A	NC_000009	130702861	130742495	8.2013371	8.3868668	10.253504	13.015002
LOC100289007	NC_000009	130766148	130829599	0.4061759	0.2923141	0.542018	0.7359028
NAIF1	NC_000009	130823512	130829599	0.2845383	0.3457235	0.2876513	0.117164
SLC25A25	NC_000009	130830479	130871524	3.6013916	1.9554404	3.4141536	3.3879507
LOC100289019	NC_000009	130877120	130880727	0.2794166	0.2051926	0.1065329	0.1668931
PTGES2	NC_000009	130882972	130890712	6.0398338	8.6394213	6.2909869	8.9796378
LOC100289048	NC_000009	130890808	130892913	0	0	0.0185648	0.0290835
LOC389791	NC_000009	130890808	130892913	0	0	0	0
LCN2	NC_000009	130911732	130915734	0	0	0	0.0745131
C9orf16	NC_000009	130922539	130926207	6.1638474	8.0480954	8.5543061	8.8910937
CIZ1	NC_000009	130928344	130966662	3.6095413	4.2633868	10.344309	21.61022
DNM1	NC_000009	130965663	131017527	0.4188119	0.4028032	0.8533437	0.3859883
LOC100289042	NC_000009	131010551	131016955	0	0	0.1006372	0
GOLGA2	NC_000009	131018108	131038268	0.7015211	0.784876	0.7617601	1.1286634
C9orf119	NC_000009	131038425	131051268	7.3023056	8.9814102	8.3296547	8.0854674
TRUB2	NC_000009	131071396	131084697	14.170938	15.385592	11.112512	20.877697
COQ4	NC_000009	131084791	131096351	2.2365512	3.1904311	3.1089031	2.5715547
SLC27A4	NC_000009	131102840	131123502	4.1294641	7.2802293	3.4482249	5.5681597
TMSL4	NC_000009	131104334	131105073	0	0	0	0
URM1	NC_000009	131133598	131153015	12.65485	10.209459	6.5691159	13.20099
CERCAM	NC_000009	131182759	131199630	18.206891	9.3256928	11.922649	8.2797644
LOC100129352	NC_000009	131207280	131213435	0	0	0	0
ODF2	NC_000009	131218432	131263239	0.983582	1.2985057	0.8253047	2.897369
GLE1	NC_000009	131266971	131304580	6.6777347	5.8901396	9.6161579	18.213148
SPTAN1	NC_000009	131314866	131395941	20.871922	22.657683	11.433866	16.267158
WDR34	NC_000009	131395940	131419129	7.9774018	6.4370193	8.0001619	18.10877
SET	NC_000009	131445934	131458675	12.625715	16.349984	15.772857	22.510034
PKN3	NC_000009	131464802	131483199	5.5439582	0.4281519	5.5456719	5.1600698
ZDHHC12	NC_000009	131483148	131486408	4.3871933	3.2161797	3.9369077	7.6030514
ZER1	NC_000009	131492065	131534198	6.7999248	7.2335621	8.5129589	8.8408342
LOC100289100	NC_000009	131515597	131516345	0.2419169	0.248716	0.1434773	0
TBC1D13	NC_000009	131549510	131572711	3.9654244	4.1700587	2.6609325	4.9265079
LOC100129035	NC_000009	131580738	131581447	0.2647484	0.2721892	0.7065825	0.2767308
ENDOG	NC_000009	131580779	131584955	1.7272231	3.8277642	1.6731709	3.503807
C9orf114	NC_000009	131584345	131592085	6.4170101	6.2078489	2.6963838	6.9797002
CCBL1	NC_000009	131595392	131644354	0.9360403	0.9388759	1.5639026	2.0522646
LRRC8A	NC_000009	131644391	131680318	7.2155191	8.385918	7.0922311	7.8250004
PHYHD1	NC_000009	131683174	131704320	2.4267157	1.8183306	1.2806877	1.0031546
DOLK	NC_000009	131707809	131710012	7.9760856	9.9633091	3.9204002	3.9740087
NUP188	NC_000009	131709977	131769374	9.1310969	10.618775	7.0511693	19.422493
SH3GLB2	NC_000009	131770313	131790579	5.6707396	2.8467365	11.212961	9.462223
FAM73B	NC_000009	131799253	131834351	2.3156442	2.2673578	1.0354806	1.6990105
DOLPP1	NC_000009	131843383	131852717	2.7265677	4.2774728	1.8686337	3.2792264
CRAT	NC_000009	131857073	131873083	12.196886	6.5817727	8.4349253	10.909518
PPP2R4	NC_000009	131873244	131911223	21.123113	22.701066	14.654828	24.546496
IER5L	NC_000009	131937831	131940540	2.7731172	3.267877	1.7168304	0.9831603

C9orf106	NC_000009	132083295	132084882	0.0521331	0.3751884	0.0463791	0
LOC100289167	NC_000009	132142415	132144182	0	0	0	0
LOC100128077	NC_000009	132252760	132253362	0	0	0	0
C9orf50	NC_000009	132374504	132383055	0.1627712	0.111564	0.1448058	0.0378085
METTL11A	NC_000009	132388435	132397879	10.26336	13.257405	10.301155	11.98099
ASB6	NC_000009	132396883	132404444	2.8264671	2.1720908	1.659403	3.4948703
LOC100131746	NC_000009	132402755	132403562	0	0	0	0
PRRX2	NC_000009	132427920	132484953	4.7602204	6.4104598	4.0558878	3.0367919
PTGES	NC_000009	132500615	132515344	3.1233494	7.4842121	1.4877641	0.3256143
TOR1B	NC_000009	132565432	132573560	2.8092586	4.2752081	2.8380681	2.2451608
TOR1A	NC_000009	132575221	132586441	12.687809	14.653496	8.9111261	9.6993323
C9orf78	NC_000009	132589564	132597572	3.8194833	4.4984575	2.6452148	4.3629502
USP20	NC_000009	132597696	132644107	2.1931207	1.6567574	2.0952698	2.6977003
FNBP1	NC_000009	132649466	132805473	1.142249	1.1160508	2.1548704	3.7653076
GPR107	NC_000009	132815985	132902448	7.9601064	8.2157443	8.8491523	8.3203788
FLJ46836	NC_000009	132903812	132904381	0	0	0	0
FREQ	NC_000009	132934857	132999583	8.6054322	8.4594872	10.09045	13.350246
LOC100289200	NC_000009	133028161	133072949	0.0185201	0.0190406	0.049428	0.0258111
HMCN2	NC_000009	133223389	133309104	0.0231794	0.0079436	0.0343685	0.0511491
ASS1	NC_000009	133320094	133376661	14.673192	6.8215201	34.781601	13.174664
LOC100289236	NC_000009	133452735	133454881	0.1432872	0.1683592	0.0728413	0.1569044
FUBP3	NC_000009	133454960	133513739	2.0602909	2.6477445	1.6347414	3.637735
RPL19P15	NC_000009	133527158	133527851	0	0	0	0
PRDM12	NC_000009	133539981	133558384	0.0176357	0	0	0.0491571
EXOSC2	NC_000009	133569158	133580452	5.2681791	6.8990153	5.8093922	8.8496686
ABL1	NC_000009	133589268	133763062	8.6599316	9.2746074	11.762709	11.391243
RPL37P17	NC_000009	133600173	133600437	0	0	0	0
QRFP	NC_000009	133768815	133769225	0	0	0.4756395	0.5215916
FIBCD1	NC_000009	133777825	133814455	0.0529019	0.081583	0.0235315	0.0184321
LAMC3	NC_000009	133884504	133968446	0.0720699	0.1037336	0.0128231	0.0351548
AIF1L	NC_000009	133971912	133998539	0.2594347	0.306735	0.1961802	0.0271177
NUP214	NC_000009	134000981	134109091	3.5619306	3.668884	5.212219	8.5272726
FAM78A	NC_000009	134133465	134151906	0.0334971	0.1033157	0.0794666	0.054465
PPAPDC3	NC_000009	134165081	134184649	2.7061202	3.5585973	1.9035569	1.2133007
BAT2L	NC_000009	134305477	134375578	4.6919962	4.974994	6.6199368	6.5474451
SNORD62A	NC_000009	134361052	134361137	0	0	0	0
SNORD62B	NC_000009	134365873	134365958	0	0	0	0
POMT1	NC_000009	134378289	134399193	1.0772898	1.2810415	1.2586044	1.6822881
UCK1	NC_000009	134399183	134406662	5.2641067	5.2669041	6.423556	9.5289892
LOC642515	NC_000009	134421947	134433589	0.05069	0.1042293	0	0.0353228
RAPGEF1	NC_000009	134452157	134612925	6.3051918	6.4322598	7.1649946	12.214996
MED27	NC_000009	134735499	134955253	10.955303	8.2596826	6.9776725	11.152412
NTNG2	NC_000009	135037334	135118220	2.8294074	0.0730886	0.075893	0.1089853
LOC100289277	NC_000009	135135414	135140400	0.0474091	0.7798645	0.1265293	0.2642924
SETX	NC_000009	135136827	135230372	2.4375789	2.0426048	1.8988015	2.7577959
TTF1	NC_000009	135250935	135282221	0.7285011	0.5761352	0.8225819	0.9957723
C9orf171	NC_000009	135285611	135448675	0.0736974	0	0	0.0342369
RNU5D	NC_000009	135297017	135297077	0	0	0	0
BARHL1	NC_000009	135457993	135465640	0	0.0710803	0.0205021	0
DDX31	NC_000009	135469676	135545788	0.8822149	1.5395295	1.1875911	1.6420629
GTF3C4	NC_000009	135545728	135565471	5.5702622	6.8254285	7.8073773	9.2603421
C9orf98	NC_000009	135600965	135754198	0.0834724	0.0214546	0.0556945	0.0581669
C9orf9	NC_000009	135754290	135765418	0.5501169	0.7541041	1.6857087	1.8315296
TSC1	NC_000009	135766736	135820020	2.6053937	2.3507274	2.7892536	4.9645331
RPL39P24	NC_000009	135837840	135838158	0	0	0	0
RNU7-21P	NC_000009	135839120	135839180	0	0	0	0
GFI1B	NC_000009	135854098	135867084	0	0	0.02183	0.0683973
EEF1AL3	NC_000009	135894822	135896562	0	0	0	0
GTF3C5	NC_000009	135906062	135933890	8.0298695	11.457815	7.4959692	12.382939
CEL	NC_000009	135937365	135947248	0.055304	0.1895277	0.1311999	0.051384
CELP	NC_000009	135957926	135962478	0	0	0	0
RALGDS	NC_000009	135973107	136024588	1.4986222	1.8558928	1.7675211	2.67404
LOC100289412	NC_000009	135982481	135985488	0	0	0	0
GBGT1	NC_000009	136028340	136039301	0.4509824	0.3013772	0.1003016	0.0942787
OBP2B	NC_000009	136080666	136084628	0	0	0.0567454	0
LCN1L1	NC_000009	136100308	136104002	0	0	0	0

ABO	NC_000009	136130563	136150630	0	0	0.024761	0
LCN1L2	NC_000009	136184440	136185304	0	0	0	0
SURF6	NC_000009	136197552	136203047	0.6069674	0.7800328	0.388107	0.4361765
RPL21P81	NC_000009	136205827	136206299	0	0	0	0
MED22	NC_000009	136207744	136214972	4.923379	3.8399492	4.4907325	7.8474772
RPL7A	NC_000009	136215069	136218280	77.279756	161.84796	135.74323	116.37454
SNORD24	NC_000009	136216251	136216325	0	0	0	0
SNORD36B	NC_000009	136216949	136217019	0	0	0	0
SNORD36A	NC_000009	136217311	136217382	0	0	0	0
SNORD36C	NC_000009	136217701	136217768	0	0	0	0
SURF1	NC_000009	136218666	136223361	9.4931571	9.9778197	6.8618678	5.9655018
SURF2	NC_000009	136223421	136228040	1.4579982	2.8373463	1.4823722	1.1611328
SURF4	NC_000009	136228340	136242970	63.235718	66.991356	91.218874	89.677565
C9orf96	NC_000009	136243284	136271220	0.0948864	0.0812944	0.1266204	0.2534625
REXO4	NC_000009	136271186	136283164	1.973946	2.5463527	1.3584747	3.7762032
ADAMTS13	NC_000009	136279459	136324508	0.3295672	0.1739936	0.1188617	0.0806898
C9orf7	NC_000009	136325087	136335909	1.0049868	1.5744489	1.1069365	1.9897834
SLC2A6	NC_000009	136336216	136344276	4.5743446	5.8564504	2.6720253	2.0689234
LOC389827	NC_000009	136379758	136389966	0	0	0	0
ADAMTSL2	NC_000009	136397286	136440641	0.0319005	0.0109323	0.0189197	0.0148197
FAM163B	NC_000009	136444144	136445345	0	0	0	0
DBH	NC_000009	136501485	136524466	0.0318465	0.0163708	0.0141658	0.011096
SARDH	NC_000009	136528682	136605077	0.0504717	0.1426981	0.0224505	0.0439634
VAV2	NC_000009	136627016	136857446	2.9534371	2.8687874	3.8686521	4.8484656
LOC100133001	NC_000009	136869885	136877844	0	0	0	0.0341414
NCRNA00094	NC_000009	136890589	136893220	0	0	0	0
BRD3	NC_000009	136895427	136933141	1.0027099	0.8790545	1.1340645	1.3595409
ARF4P	NC_000009	136943753	136944869	0	0	0	0
WDR5	NC_000009	137001210	137025094	5.6885553	7.3815171	4.9869971	7.2064197
RNU6ATAC	NC_000009	137029562	137029686	0	0	0	0
RXRA	NC_000009	137218316	137332431	11.006955	7.9287788	8.6946415	14.436175
COL5A1	NC_000009	137533652	137736689	2.4945139	6.2268392	1.0794801	1.2229629
FCN2	NC_000009	137772658	137779366	0	0	0.0369892	0
FCN1	NC_000009	137801432	137809809	0	0.0351622	0	0.0238326
OLFM1	NC_000009	137967089	138013030	1.0163029	3.2051976	0	0.0287108
C9orf62	NC_000009	138235245	138236253	0	0	0	0
LOC100128299	NC_000009	138339292	138371170	0	0	0	0
KIAA0649	NC_000009	138371648	138380739	0.801975	1.1818043	1.5458283	1.0928586
C9orf116	NC_000009	138387026	138391761	0.7913117	0.7509707	0.649821	0.933168
MRPS2	NC_000009	138392483	138396519	12.147817	14.470697	13.326944	17.49993
LCN1	NC_000009	138413286	138418378	0	0	0	0
OBP2A	NC_000009	138437985	138441815	0	0	0	0
PAEP	NC_000009	138453604	138458622	0.0523193	0	0	0
LOC138159	NC_000009	138479751	138483838	0	0	0	0
GLT6D1	NC_000009	138515502	138531386	0.1439981	0	0	0.0200687
LCN9	NC_000009	138555168	138557949	0	0	0	0
SOHLH1	NC_000009	138585253	138591374	0.0442358	0.0227395	0.0393534	0
KCNT1	NC_000009	138594038	138684992	0.0091122	0.0187366	0.016213	0.0190493
CAMSAP1	NC_000009	138700333	138799005	1.6701595	1.8828885	2.5822531	2.7169496
UBAC1	NC_000009	138824815	138853226	3.5073843	4.9128177	5.2144048	4.1828302
NACC2	NC_000009	138903202	138987131	3.2327305	1.8247143	2.0488628	3.2244463
LOC100288840	NC_000009	138940153	138987680	0.172346	0.1012513	0	0.1200976
LOC402382	NC_000009	138988123	138994444	0.0777157	0	0	0
C9orf69	NC_000009	139006427	139010731	10.828721	12.530652	9.8121834	16.351209
LOC100129999	NC_000009	139015441	139015815	0	0	0	0
LHX3	NC_000009	139088096	139096955	0	0.0175197	0.01516	0.0356241
QSOX2	NC_000009	139098179	139137687	2.7067454	4.7676967	3.4782161	3.9616288
ESP33	NC_000009	139141805	139143051	0	0	0	0
LOC26102	NC_000009	139217006	139221779	0	0	0	0
GPSM1	NC_000009	139221932	139254057	4.2409563	3.2824703	2.3355876	1.8160486
DNLZ	NC_000009	139256352	139258241	3.4600127	3.3076247	1.1340454	2.5379733
CARD9	NC_000009	139258408	139268133	0.20701	0.1276968	0.0552486	0.201954
LOC100289446	NC_000009	139264624	139265583	0	0	0	0
SNAPC4	NC_000009	139270029	139292889	1.2948189	1.0688547	1.1434987	1.356715
SDCCAG3	NC_000009	139296374	139305054	1.1638245	1.2155266	1.3969286	2.3943789
PMPCA	NC_000009	139305116	139318213	4.2891784	5.5827569	4.1541163	4.0489623

INPP5E	NC_000009	139323071	139334256	3.5148171	2.9042649	3.1268788	3.2747574
SEC16A	NC_000009	139334548	139377507	8.9237949	9.2145757	7.1129723	7.4851782
C9orf163	NC_000009	139377947	139380519	0.1708054	0.0702424	0.0607813	0.059512
NOTCH1	NC_000009	139388896	139440238	1.2766028	1.327065	2.3008465	1.8088282
LOC100289474	NC_000009	139399037	139400323	0	0	0	0.0280962
LOC401561	NC_000009	139510761	139511298	0	0	0	0
EGFL7	NC_000009	139557377	139567130	1.1459774	1.7672778	0.7646199	0.0798563
AGPAT2	NC_000009	139567595	139581911	5.5361145	6.217096	6.2889495	6.6076934
FAM69B	NC_000009	139607030	139618489	0.2134705	0.3840726	0.4985118	0.818151
SNHG7	NC_000009	139619046	139622636	0	0	0	0
SNORA43	NC_000009	139620556	139620689	0	0	0	0
SNORA17	NC_000009	139621199	139621331	0	0	0	0
LCN10	NC_000009	139632619	139637411	0.0217889	0.0448026	0.0581521	0
LCN6	NC_000009	139638469	139642980	0	0	0	0
LCN8	NC_000009	139648840	139652731	0	0	0	0
LCN15	NC_000009	139654086	139658965	0	0	0	0
TMEM141	NC_000009	139685777	139687769	16.493392	18.747426	16.2223	17.489732
KIAA1984	NC_000009	139690802	139702193	0.5416155	0.2916769	0.4818362	1.0963131
LOC100131193	NC_000009	139698379	139703300	0	0	0	0
C9orf86	NC_000009	139702381	139735639	2.6596913	3.3710319	3.0797314	7.3056466
LOC389813	NC_000009	139738867	139741797	0.269897	0.1849883	0.3468225	0.6896083
PHPT1	NC_000009	139743256	139745490	6.9207048	6.7179159	6.25061	7.0992923
MAMDC4	NC_000009	139746819	139755251	0.7925091	0.2962846	0.865274	2.0834958
EDF1	NC_000009	139756571	139760738	10.110966	9.9226294	13.543601	11.649464
TRAF2	NC_000009	139780965	139821067	1.215959	0.9326394	1.511017	2.1250465
FBXW5	NC_000009	139834887	139839173	24.363625	18.632132	27.943505	26.484969
C8G	NC_000009	139839698	139841426	0.150336	0	0.3566483	0.03492
LCN12	NC_000009	139846768	139849949	0.3827132	0	0.1702361	0.0888966
PTGDS	NC_000009	139871956	139876194	0.7577281	0	0.1925988	0.0377154
LCNL1	NC_000009	139877445	139880210	0.0478739	0.0246097	0.0638849	0.0333604
C9orf142	NC_000009	139886870	139888428	7.414911	7.1781513	4.1890249	5.4310129
CLIC3	NC_000009	139889060	139891024	2.1622746	1.8895888	0.1923619	0.2260139
ABCA2	NC_000009	139901686	139923374	0.6781486	0.4269723	2.0390596	2.2822128
C9orf139	NC_000009	139921916	139931234	0	0.0118436	0	0.016055
FUT7	NC_000009	139924626	139927292	0	0	0.0323923	0
NPDC1	NC_000009	139933909	139940676	7.8232455	4.9382383	5.4245314	9.8208052
ENTPD2	NC_000009	139942553	139948505	0	0	0	0.0437498
C9orf140	NC_000009	139956579	139965028	1.0319178	2.0039599	1.6320403	2.70854
UAP1L1	NC_000009	139971953	139978990	2.7949247	2.0544013	1.9868302	3.9862397
LOC100289341	NC_000009	139979398	139981280	0.4201106	0.7678539	0.4567958	0.3578053
MAN1B1	NC_000009	139981417	140003639	10.395664	11.200719	14.287576	8.8813268
DPP7	NC_000009	140004992	140009195	12.638174	6.84597	14.072222	12.291618
GRIN1	NC_000009	140033609	140063208	0.030129	0.0206505	0.0178691	0.0209951
LRRC26	NC_000009	140063210	140064491	0.0734306	0	0.032663	0.0767541
ANAPC2	NC_000009	140069236	140083057	4.4924859	5.3383353	3.4753392	5.013406
SSNA1	NC_000009	140083054	140084822	19.241946	26.457019	15.040721	26.556749
C9orf75	NC_000009	140086069	140094980	1.9787119	2.623207	2.5323858	3.2898763
TMEM203	NC_000009	140098534	140100090	7.8751167	9.4023264	9.4918945	4.9369585
NDOR1	NC_000009	140100119	140113813	2.1266727	1.9362992	1.4991275	3.0141361
RNF208	NC_000009	140114707	140115775	0.3288923	0.1690679	0.4388875	0.2578334
C9orf169	NC_000009	140119644	140120763	0.356337	0.1221173	0.1056691	0
LOC643596	NC_000009	140123075	140123538	0	0	0	0
SLC34A3	NC_000009	140125385	140131006	0.1862213	0.1063639	0.1104451	0.1874404
TUBB2C	NC_000009	140135711	140138159	108.44799	165.39795	113.43455	154.06758
FAM166A	NC_000009	140138037	140142222	0.1954993	0.0803975	0.0695686	0.2724633
LOC441476	NC_000009	140145730	140147934	0.1123996	0.077039	0.0666625	0.2088653
COBRA1	NC_000009	140149759	140168000	15.294533	18.50653	12.021816	16.959451
C9orf167	NC_000009	140172280	140177093	1.4689013	1.5323936	2.3445087	2.5213404
NRARP	NC_000009	140194083	140196703	0	0	0.014917	0.0233689
EXD3	NC_000009	140201348	140317714	0.4881442	0.1568324	0.7328249	0.3082685
NOXA1	NC_000009	140317847	140328858	0.2960383	0.027669	0.8619182	0.3375675
ENTPD8	NC_000009	140328816	140335901	0	0.0406691	0.0703827	0.0275651
NELF	NC_000009	140342022	140353786	5.3234087	5.6959071	3.7822529	4.909716
PNPLA7	NC_000009	140354405	140444986	0.0823001	0.1128175	0.0650813	0.0127444
MRPL41	NC_000009	140446309	140447007	12.224011	16.831565	12.557827	18.963087
WDR85	NC_000009	140449361	140473387	1.7131958	1.2476197	1.2065843	2.2549992

LOC100289409	NC_000009	140475648	140476299	0.2022158	0.4157982	0.1798968	0.140912
ZMYND19	NC_000009	140476531	140484937	6.2874843	8.3440881	6.1642877	8.1591829
ARRDC1	NC_000009	140500096	140509812	2.1451577	1.9509729	2.0796577	2.7021951
C9orf37	NC_000009	140509789	140513249	1.7731275	2.1159374	4.7604385	4.6996391
LOC100130014	NC_000009	140535433	140588877	0	0	0	0
EHMT1	NC_000009	140605417	140730579	1.1851209	1.9799468	1.3911119	1.1642042
FLJ40292	NC_000009	140657474	140659224	0	0	0	0
CACNA1B	NC_000009	140772241	141019076	0.0269346	0.0830747	0.0359426	0.03441
LOC100128449	NC_000009	141031487	141039036	0	0	0	0
LOC643224	NC_000009	141044565	141071885	0	0	0	0
TUBBP5	NC_000009	141069494	141071716	0	0	0	0
FAM157B	NC_000009	141106637	141134172	0	0	0.0611855	0
TUBB8	NC_000010	92997	95178	0.03292	0	0.0292866	0.1376399
IL9RP2	NC_000010	125916	132183	0	0	0	0
ZMYND11	NC_000010	180643	300577	3.2270386	3.1207444	4.0909798	7.477024
DIP2C	NC_000010	320130	735608	1.1910995	0.6809555	0.9903132	1.5436552
C10orf108	NC_000010	695888	711109	0	0	0	0
LARP5	NC_000010	855484	931702	3.935081	2.8039345	4.214773	5.8806282
LOC100288619	NC_000010	1018440	1018871	0	0.3137736	0	0
GTPBP4	NC_000010	1034349	1063708	4.2778374	3.8056739	6.8036288	6.3147842
IDI2	NC_000010	1064847	1071799	0.5174185	0.1329901	0.3452324	0.6084413
C10orf110	NC_000010	1068577	1090141	0	0	0	0
IDI1	NC_000010	1085963	1095061	15.238952	8.9647929	26.766373	16.695359
LOC100130837	NC_000010	1094557	1102508	0.3092224	0.7153045	0.3782526	0.4040221
WDR37	NC_000010	1102776	1178237	1.0103042	1.0484979	1.0598993	1.4080404
C10orf139	NC_000010	1205708	1210612	0	0	0	0
LOC100288652	NC_000010	1206525	1221907	0	0	0	0
ADARB2	NC_000010	1228073	1779718	0	0.0247309	0.0106999	0.0167624
LOC100288677	NC_000010	1402026	1403834	0	0	0	0.0872504
NCRNA00168	NC_000010	1568825	1599184	0	0	0	0
LOC100129465	NC_000010	1814626	1815571	0	0	0	0
LOC727878	NC_000010	2540865	2544348	0	0	0	0
PFKP	NC_000010	3109752	3178996	51.743331	52.351258	41.877438	61.917042
PITRM1	NC_000010	3179920	3215003	4.4380593	4.0136876	3.8916558	9.5082449
LOC100287172	NC_000010	3810198	3824481	36.026233	28.183066	35.350016	25.498826
KLF6	NC_000010	3821234	3827455	11.880874	9.415565	12.893878	9.5247588
LOC100130652	NC_000010	3869773	3870997	0	0	0.1646213	0.0644734
LOC727894	NC_000010	3974896	3977913	0	0	0	0.0981567
LOC100128356	NC_000010	4482199	4482522	0	0	0	0
LOC100216001	NC_000010	4692377	4720262	0	0	0	0
LOC338588	NC_000010	4698348	4704611	0	0	0	0
AKR1CL2	NC_000010	4868402	4890254	0.1637822	0.7016056	0.0242842	0.0380433
tAKR	NC_000010	4913859	4958465	0	0	0	0
AKR1C1	NC_000010	5005454	5020158	98.604662	38.433742	256.55828	147.57238
AKR1C2	NC_000010	5031965	5060207	1.6584238	2.9838096	6.6637989	3.832925
LOC100134257	NC_000010	5042103	5046053	0	0.0478131	0.0413731	0.0324073
AKR1C3	NC_000010	5136568	5149878	17.521844	12.218714	47.051237	48.989797
AKR1CL1	NC_000010	5203694	5227144	0.267977	0	0.0794666	0
AKR1C4	NC_000010	5238798	5260912	0.1472303	0.1135261	0.2947053	0.4616816
LOC648947	NC_000010	5315979	5330457	0	0	0	0
RPL26P28	NC_000010	5346343	5346864	0	0	0	0
UCN3	NC_000010	5406976	5416169	0	0.0636386	0	0
TUBAL3	NC_000010	5435061	5446793	0	0	0	0.0170327
NET1	NC_000010	5454518	5500426	3.4809296	4.9069617	8.555918	8.1938637
CALML5	NC_000010	5540658	5541533	0	0	0	0
LOC100132159	NC_000010	5557803	5558553	0	0	0	0.0407788
CALML3	NC_000010	5566924	5568225	0.0337544	0	0	0
ASB13	NC_000010	5680820	5708558	1.2086972	1.4746325	3.1971973	2.1898978
LOC100288895	NC_000010	5722756	5727847	0.1294626	0.0798607	0.0844606	0.0601431
C10orf18	NC_000010	5726801	5806943	4.2718786	5.243766	7.4224347	7.6763961
GDI2	NC_000010	5807186	5855512	22.739384	19.18234	29.388013	42.496463
LOC100118954	NC_000010	5817797	5820737	0	0	0	0
ANKRD16	NC_000010	5903689	5931860	0.6997065	0.4110696	0.7262247	1.1260854
RPL12P28	NC_000010	5904156	5904557	0	0	0	0
FBXO18	NC_000010	5932214	5979556	2.7308538	2.2365665	3.7265189	5.6282688
IL15RA	NC_000010	5994334	6020142	2.6635292	1.5712062	6.1375215	3.5751845

IL2RA	NC_000010	6053506	6104272	0.0190582	0	0.0169547	0.026561
RPL32P23	NC_000010	6113474	6113884	0	0	0	0
RBM17	NC_000010	6130949	6159422	2.4964282	2.1103078	4.0074758	4.9018361
PFKFB3	NC_000010	6186843	6277508	1.8108473	2.0353753	3.7979061	5.838603
LOC100128930	NC_000010	6335031	6336222	0	0	0	0
LOC399715	NC_000010	6349587	6370299	0	0	0.0584418	0.0915542
DKFZp667F0711	NC_000010	6392283	6393331	0.0418954	0.0430728	0	0.0291944
PRKCQ	NC_000010	6469105	6557111	0.0549525	0	0.0244436	0.0382931
LOC439949	NC_000010	6622398	6626265	0	0	0.0344776	0
SFMBT2	NC_000010	7204249	7453450	0.0302188	0.0207121	0.0268835	0.0210577
LOC100128916	NC_000010	7453561	7455437	0.0234141	0	0	0
ITIH5	NC_000010	7601635	7708934	0.0119036	0	0.0105898	0.0290323
ITIH2	NC_000010	7745236	7791483	0.0413436	0	0.0122601	0.0192066
KIN	NC_000010	7797367	7829944	0.5072724	0.3555882	0.6153867	0.3856228
ATP5C1	NC_000010	7830093	7849764	39.584035	41.719866	80.953757	84.938687
TAF3	NC_000010	7860673	8056714	0.2047767	0.1295581	0.3783635	0.7025062
FLJ45983	NC_000010	8092413	8095447	0	0	0	0
GATA3	NC_000010	8096667	8117164	0	0.0147177	0.0254707	0.0099755
LOC389935	NC_000010	8203223	8204160	0	0	0	0
LOC100287325	NC_000010	8555645	8575656	0	0	0	0
KRT8P16	NC_000010	8578598	8584663	0	0	0	0
LOC338591	NC_000010	8645909	8662648	0	0	0	0
LOC644495	NC_000010	9763922	9766392	0	0	0	0
TCEB1P3	NC_000010	10216117	10216740	0	0	0	0
LOC100287395	NC_000010	10818176	10818624	0	0	0	0
SFTA1P	NC_000010	10826402	10836877	0	0	0	0
LOC254312	NC_000010	10976903	10994126	0	0	0	0
CUGBP2	NC_000010	11047259	11378674	5.9058382	1.1119371	6.7751015	13.712865
USP6NL	NC_000010	11502509	11653679	2.3168905	1.4385454	3.2736229	3.0833814
LOC439951	NC_000010	11653306	11654226	0	0	0	0
ECHDC3	NC_000010	11784356	11806065	0.876839	1.9941888	2.6711154	2.6107061
C10orf47	NC_000010	11865397	11914276	0.3428305	0.8269389	0.0117304	0.0183768
UPF2	NC_000010	11962021	12085023	0.6576833	0.7959948	1.1479679	1.9028149
DHTKD1	NC_000010	12110934	12165224	2.3851812	1.5478533	3.0549676	1.679771
SEC61A2	NC_000010	12171640	12211957	1.3610477	1.1754121	1.6830479	2.086551
NUDT5	NC_000010	12209573	12238143	14.600741	9.0066582	33.707037	25.28333
CDC123	NC_000010	12237961	12292589	20.696551	18.563723	36.698167	46.332988
CAMK1D	NC_000010	12391583	12871735	0.5870636	0.1508908	0.3336712	0.3749985
LOC283070	NC_000010	12875133	12877545	0	0	0	0
CCDC3	NC_000010	12938626	13043704	0.0160571	0	0.5856778	1.3986517
RPL5P25	NC_000010	13099890	13100725	0	0	0	0
OPTN	NC_000010	13142082	13180276	23.177591	12.36043	28.217203	32.957716
LOC441548	NC_000010	13198669	13200136	0	0	0	0
RPL36AP36	NC_000010	13201421	13201817	0	0	0	0
MCM10	NC_000010	13203581	13253104	0.6218631	1.9979395	1.2620483	1.9567963
RNU6-2	NC_000010	13259331	13259375	0	0	0	0
UCMA	NC_000010	13263771	13276331	0	0	0	0
PHYH	NC_000010	13319796	13342130	2.8814793	2.6358928	2.1799365	2.7510216
LOC100287471	NC_000010	13350145	13350447	2.610786	2.0876819	3.6129764	3.5375272
SEPHS1	NC_000010	13360080	13390280	6.6260411	6.1345157	8.7668772	10.424237
BEND7	NC_000010	13480484	13544976	0.2130651	0.1168284	0.3917339	0.2969445
PRPF18	NC_000010	13628939	13672868	3.5055481	2.6235524	5.2282962	6.8254861
RPL6P24	NC_000010	13633425	13634350	0	0	0	0
FRMD4A	NC_000010	13685706	14372866	1.6212531	1.7398664	1.471043	3.6188128
LOC100127937	NC_000010	13771383	13798200	0	0	0	0
FAM107B	NC_000010	14560559	14816896	3.9477936	3.4857473	2.0246031	2.1846016
LOC100289125	NC_000010	14607109	14609081	0	0.0229009	0	0
RPSAP7	NC_000010	14765098	14766130	0	0	0	0
ARMETL1	NC_000010	14861251	14879983	0.0991313	0.1019174	0.4115533	0.0921049
HSPA14	NC_000010	14880261	14913740	4.1368767	4.6034031	8.8110239	10.191335
SUV39H2	NC_000010	14920899	14946314	0.5552255	0.7464702	1.7098061	2.4107047
DCLRE1C	NC_000010	14948870	14996094	1.1768027	1.2428735	1.8178274	2.1768414
MEIG1	NC_000010	15001438	15014850	0.1406343	0.1445869	0.2502244	0.0979996
OR7E110P	NC_000010	15028875	15029470	0	0	0	0
OR7E26P	NC_000010	15041198	15041851	0	0	0	0
OR7E115P	NC_000010	15049787	15050725	0	0	0	0

LOC100289151	NC_000010	15057357	15077424	0	0	0	0
OLAH	NC_000010	15085895	15115851	0.0479261	0.0739096	0.0426364	0.0333968
ACBD7	NC_000010	15117474	15130775	0.2086563	0.0804452	0.4176595	0.3362375
LOC100240710	NC_000010	15135060	15135612	0	0	0	0
C10orf111	NC_000010	15137384	15139318	0	0.0527228	0.0228107	0.0178675
RPP38	NC_000010	15139182	15146256	2.4681072	1.5825105	3.2817401	3.0883784
NMT2	NC_000010	15147771	15210695	0.6850444	0.5869147	0.7422599	1.4810593
LOC100192204	NC_000010	15196589	15197361	0	0	0	0
LOC100287503	NC_000010	15213032	15233308	0	0	0	0
FAM171A1	NC_000010	15253642	15413058	0.6449892	0.5373532	2.2457357	5.3159581
ITGA8	NC_000010	15559088	15761770	0.0673846	0.0692784	0.0239789	0.07513
C10orf97	NC_000010	15820175	15902519	5.958455	3.2912541	3.1607295	5.6736617
PTER	NC_000010	16478967	16555736	0.7831684	0.6184712	1.8680397	1.3524933
C1QL3	NC_000010	16555742	16564004	0.1410292	0	0.250927	0.1842652
RSU1	NC_000010	16632615	16859453	16.760018	17.219027	17.711041	20.846122
LOC100287543	NC_000010	16824033	16859603	0.064158	0.0659612	0	0.0894157
CUBN	NC_000010	16865965	17171816	0.0368292	0.0302914	0.0556992	0.0333632
LOC100289189	NC_000010	17184058	17186785	0.0161101	0.0331257	0.1003237	0.0449045
TRDMT1	NC_000010	17184982	17243681	0.3500889	0.2978716	0.7195542	1.3417575
LOC100287578	NC_000010	17271173	17281729	15.337638	15.701316	11.924612	27.336301
VIM	NC_000010	17271298	17279592	771.17607	839.84373	778.60675	938.77605
ST8SIA6	NC_000010	17362676	17496254	0.062873	0.0646401	0.1118671	0.0219062
LOC100128098	NC_000010	17428950	17451551	0	0	0	0
LOC100131947	NC_000010	17619284	17620217	0	0	0	0
PTPLA	NC_000010	17631958	17659373	2.959024	1.1753907	2.6324298	1.8745144
STAM	NC_000010	17686124	17757907	2.3411398	1.7366513	4.1391219	5.4311146
FAM23A	NC_000010	17794251	17842867	0.0080432	0	0.0071555	0.0112097
MRC1L1	NC_000010	17851362	17953178	0.0424949	0.0174757	0.0226828	0.0177673
LOC100289299	NC_000010	18041224	18089855	0.0161012	0	0.007162	0.01122
MRC1	NC_000010	18098352	18200091	0.016998	0.0087378	0.0151219	0.0355346
SLC39A12	NC_000010	18240768	18332221	0.0156511	0.0321819	0.0696182	0.0872504
LOC100129213	NC_000010	18290715	18318269	0	0	0	0
CACNB2	NC_000010	18429606	18830688	0.2798312	0.1741317	0.262048	0.5131514
NSUN6	NC_000010	18834264	18940550	1.0144687	1.0243558	1.3376331	2.1838846
ARL5B	NC_000010	18948313	18966940	3.318465	2.4549355	7.2334309	4.5054154
LOC645120	NC_000010	19024676	19027565	0	0	0	0
LOC100287636	NC_000010	19148690	19229887	0	0	0	0
LOC100128493	NC_000010	19340384	19342419	0	0	0	0
LOC100130846	NC_000010	19353216	19417352	0	0.0398443	0	0
C10orf112	NC_000010	19422755	20023407	0.1311888	0.0952065	0.0686524	0.0376425
TRNAQ38P	NC_000010	20036608	20036682	0	0	0	0
PLXDC2	NC_000010	20105372	20569115	0.2202494	0.1045106	0.0452169	0.0354181
LOC100133024	NC_000010	20639169	20640038	0	0	0	0
NEBL	NC_000010	21068902	21463116	0.0221357	0.031861	0.0472621	0.0370201
LOC100129337	NC_000010	21317928	21319745	0	0	0	0
LOC100129615	NC_000010	21399611	21402484	0	0	0	0
C10orf113	NC_000010	21414692	21435427	0	0	0	0
LOC100128511	NC_000010	21462202	21463850	0	0	0	0
LOC100289386	NC_000010	21536681	21537001	0	0	0	0
C10orf114	NC_000010	21783421	21786213	0.1546695	0.0908666	0.1572552	0.2771482
C10orf140	NC_000010	21802407	21814611	0.0532867	0.0479363	0.0829594	0.0928308
MLLT10	NC_000010	21823102	22032554	1.1843109	0.9617244	3.0004576	3.1934554
LOC100125387	NC_000010	21912854	21915285	0	0	0	0
DNAJC1	NC_000010	22045477	22292650	5.0022378	4.9915666	8.1336399	8.3628912
LOC645220	NC_000010	22453382	22456165	0	0	0	0
LOC340900	NC_000010	22497812	22498912	0	0	0	0
PSME2P6	NC_000010	22514626	22515000	0	0	0	0
LOC100130992	NC_000010	22541001	22543553	0.1205004	0.1238871	0.0765718	0.1199564
LOC100287942	NC_000010	22542182	22606590	0.1792891	0.046082	0.0996878	0.0780849
RPL31P45	NC_000010	22555919	22556134	0	0	0	0
COMMD3	NC_000010	22605299	22609237	9.75595	10.794343	18.763525	19.359067
BMI1	NC_000010	22610140	22620188	1.7837247	1.2413798	10.863826	10.297532
SPAG6	NC_000010	22634399	22706539	0.0338845	0.0174184	0.0301446	0.0708363
LOC100289455	NC_000010	22725955	22727388	0.7355353	0.630173	0.2999116	0.6834004
PIP4K2A	NC_000010	22823766	23003503	1.4790822	1.5795919	2.7744685	5.4330595
TRNAP22P	NC_000010	22852579	22852651	0	0	0	0

ARMC3	NC_000010	23216954	23326514	0.0469532	0.0482729	0.0278473	0.0327189
MSRB2	NC_000010	23384427	23410942	1.4803104	1.1726228	4.4473211	2.5873034
YWHAZP3	NC_000010	23425765	23428707	0	0	0	0
LOC729385	NC_000010	23461997	23463910	0	0	0	0
PTF1A	NC_000010	23481460	23483181	0.0329694	0.1016881	0.117322	0.0918976
C10orf115	NC_000010	23492745	23512728	0	0	0	0
C10orf67	NC_000010	23605520	23633772	0.0895988	0.0307057	0.199274	0.2705562
OTUD1	NC_000010	23728198	23731310	0.6494117	0.5225193	2.3988548	1.4363097
KIAA1217	NC_000010	23983675	24836772	1.0682044	0.3337747	1.8214164	2.0214682
PRINS	NC_000010	24536051	24544975	0	0	0	0
ARHGAP21	NC_000010	24872538	25012597	11.313588	10.553511	19.600607	23.399168
PRTFDC1	NC_000010	25137554	25241533	3.9475826	3.9881507	6.8816587	7.2348492
ENKUR	NC_000010	25270917	25305030	0.0132454	0.0136177	0.0353504	0.0553795
THNSL1	NC_000010	25305508	25315593	0.4429795	0.4434445	0.7881737	1.4865657
LOC100128811	NC_000010	25447001	25465205	0	0	0	0
GPR158	NC_000010	25464290	25891158	0.2704304	0.4204369	0.1114894	0.7308053
MYO3A	NC_000010	26223002	26501465	0.030393	0.0312472	0.0202788	0.0635371
GAD2	NC_000010	26505236	26593491	0.0149078	0.0153268	0	0.0415534
APBB1IP	NC_000010	26727266	26856732	2.9998793	0.5997039	3.2618371	3.7511702
C10orf50	NC_000010	26878794	26883253	0	0	0	0
LOC731789	NC_000010	26932037	26942383	0	0	0	0
PDSS1	NC_000010	26986595	27035727	2.5516577	2.065208	3.3567397	5.4099538
ABI1	NC_000010	27035525	27149959	7.5004343	8.0068733	11.373256	13.0999
C10orf51	NC_000010	27220135	27230930	0	0	0	0
LOC100289517	NC_000010	27232291	27233347	0	0.2218498	0	0
LOC100289548	NC_000010	27255728	27260827	0	0	0.1068239	0
ANKRD26	NC_000010	27293045	27389427	0.3630478	0.2532777	0.3921868	0.6957119
YME1L1	NC_000010	27399383	27443321	15.671632	15.6184	25.631381	28.389042
MASTL	NC_000010	27443717	27475848	7.7951359	6.3224656	15.483021	8.7380085
ACBD5	NC_000010	27484143	27531068	3.5668757	2.8447985	4.9617176	5.370275
RNU7-12P	NC_000010	27521087	27521344	0	0	0	0
LOC387646	NC_000010	27534782	27541235	0	0	0	0
LOC100288039	NC_000010	27551925	27602834	0.2753649	0.0566208	0.1469833	0.1535082
LOC729549	NC_000010	27608635	27633558	0	0.0382262	0	0.0518187
LOC100131328	NC_000010	27638641	27639219	0	0	0	0
LOC100289619	NC_000010	27667062	27667291	0	0	0	0
PTCHD3	NC_000010	27687117	27703297	0.0173846	0.0178732	0.0154658	0.0363428
RAB18	NC_000010	27793249	27829099	9.0259272	6.8827763	13.466939	11.898698
MKX	NC_000010	27961803	28034778	1.1352387	0.1489972	1.7620227	1.1277092
ARMC4	NC_000010	28101097	28287977	0.2214637	0.1897399	1.3791415	1.457511
MRPS21P5	NC_000010	28293595	28293864	0	0	0	0
MPP7	NC_000010	28339922	28571067	0.4473317	0.0086774	0.5406231	0.6469631
LOC100288110	NC_000010	28525545	28592173	0	0	0.0196076	0.030717
RPSAP10	NC_000010	28725052	28726084	0	0	0	0
WAC	NC_000010	28821427	28909925	14.113822	11.735262	25.043483	27.328606
LOC100131803	NC_000010	28919690	28948399	0	0	0	0
LOC729590	NC_000010	28966420	28970414	0.7626591	1.4701758	1.0177241	0
BAMBI	NC_000010	28966424	28971868	1.7079649	1.4414658	0.4082112	0.1954024
LOC729601	NC_000010	29161750	29164821	0	0	0	0
RPL21P93	NC_000010	29187879	29188453	0	0	0	0
LYZL1	NC_000010	29577990	29600158	0	0	0	0.0377154
LOC387647	NC_000010	29698501	29711299	0	0	0	0
SVIL	NC_000010	29746277	30024730	2.9463364	2.2890386	2.0081703	3.855431
CKS1BP2	NC_000010	29986864	29987582	0	0	0	0
KIAA1462	NC_000010	30303384	30336800	5.4071368	0.7822156	0.0465017	0.024283
MTPAP	NC_000010	30598730	30638267	1.2668352	1.2863602	1.6557332	2.7464304
DNM1P17	NC_000010	30646083	30647913	0	0	0	0
LOC729668	NC_000010	30653256	30663377	0	0	0	0
LOC401825	NC_000010	30669061	30670050	0	0	0	0
CCND3P	NC_000010	30692123	30692750	0	0	0	0
MAP3K8	NC_000010	30722866	30750761	4.0740124	1.0215885	4.6977695	3.8775686
LOC645940	NC_000010	30806540	30807790	0	0	0	0
LYZL2	NC_000010	30900708	30918647	0	0	0	0.039567
LOC645954	NC_000010	30951270	31006198	0	0	0	0
ZNF438	NC_000010	31133563	31320866	1.2373329	1.2956659	1.7734558	2.6265863
LOC100129214	NC_000010	31133567	31137208	0.0120671	0	0	0.0084088

LOC220930	NC_000010	31605457	31608024	0	0	0	0
ZEB1	NC_000010	31607424	31818127	5.208905	7.9076825	7.5268403	6.2884216
LOC646034	NC_000010	31892078	31895444	0.0391579	0.0670974	0.1161199	0.1455296
GLUDP5	NC_000010	31906539	31907129	0	0	0	0
ARHGAP12	NC_000010	32095225	32217770	4.2979095	3.5739508	7.000451	6.0339525
RPL34P19	NC_000010	32273183	32273483	0	0	0	0
KIF5B	NC_000010	32297938	32345371	3.1045109	2.9155532	3.2465121	8.7365929
RPS4P11	NC_000010	32391381	32392269	0	0	0	0
LOC100288279	NC_000010	32398789	32399140	0	0	0	0
LOC439953	NC_000010	32477194	32477745	0	0	0	0
RPS24P13	NC_000010	32520708	32521211	0	0	0	0
EPC1	NC_000010	32557859	32636113	2.0669096	1.2563871	2.2548545	3.185492
CCDC7	NC_000010	32735041	32863492	0.2848497	0.3137736	0.4525181	0.7372656
LOC554049	NC_000010	32799585	32800718	0	0	0	0
C10orf68	NC_000010	32856651	33171792	0.0969803	0.0830883	0.1150351	0.1689493
ITGB1	NC_000010	33189246	33247293	119.59601	112.96709	154.1901	192.93091
LOC100288319	NC_000010	33231309	33233932	0	0	0	0
RPL7AP53	NC_000010	33346483	33347360	0	0	0	0
NRP1	NC_000010	33466419	33623833	33.888689	39.511589	59.092819	35.064993
RPL23P11	NC_000010	34153241	34153651	0	0	0	0
PARD3	NC_000010	34400097	35103923	3.4113587	2.519438	5.3781962	5.0928623
TRNAL44P	NC_000010	34591416	34591483	0	0	0	0
RPL37P18	NC_000010	34952805	34953029	0	0	0	0
RPS12P16	NC_000010	34964541	34965056	0	0	0	0
LOC646213	NC_000010	35164137	35165155	0	0	0	0
LOC646218	NC_000010	35253997	35255187	0	0	0	0
CUL2	NC_000010	35298798	35379294	8.7318398	8.9772491	15.657973	15.773561
CREM	NC_000010	35415801	35501886	1.0434347	1.0940034	1.9576355	1.8573622
RNU7-77P	NC_000010	35424372	35424428	0	0	0	0
CCNY	NC_000010	35535953	35860848	9.4792322	9.385873	11.829989	16.208952
GJD4	NC_000010	35894338	35897863	0	0.0285971	0.0247453	0.0193828
FZD8	NC_000010	35927177	35930362	3.7520148	3.7723744	2.4052489	1.7782808
RPL7P37	NC_000010	35986202	35987033	0	0	0	0
LOC100288345	NC_000010	36721781	36723406	0	0	0	0
NAMPTL	NC_000010	36810664	36813162	0	0	0	0
LOC389948	NC_000010	37232976	37233585	0	0	0	0
LOC646348	NC_000010	37283951	37284518	0	0	0	0
ANKRD30A	NC_000010	37414785	37521495	0.0698383	0.0410292	0.0266272	0.0278092
LOC646360	NC_000010	37626327	37627802	0	0	0	0
LOC219752	NC_000010	37812245	37845238	0	0	0	0
LOC100288485	NC_000010	37889718	37891802	0	0	0	0
TLK2P2	NC_000010	38107534	38109910	0	0	0	0
ZNF248	NC_000010	38117899	38146486	0.3150088	0.4626603	1.0569074	2.3644438
BA775A3.1	NC_000010	38172447	38173125	0	0	0	0
BA393J16.4	NC_000010	38183420	38185963	0	0	0	0
ZNF25	NC_000010	38238795	38265453	1.8468609	1.3787226	3.1499912	2.3935933
ZNF33A	NC_000010	38299578	38348995	1.0841665	0.9891482	2.1142451	3.9075363
LOC340947	NC_000010	38368984	38379498	0.2310633	0.1900459	0.1644482	0.5152451
LOC100128512	NC_000010	38380692	38380979	0	0	0	0
ZNF37A	NC_000010	38383275	38412280	0.4757928	0.6114563	1.1473075	2.3950225
LOC100129055	NC_000010	38464599	38503273	0	0	0	0
LOC100131082	NC_000010	38638927	38641218	9.3955644	7.5502805	11.190228	14.363763
HSD17B7P2	NC_000010	38645308	38667433	0	0	0	0
CDC10L	NC_000010	38671997	38691780	0	0	0	0
LOC399744	NC_000010	38717074	38741081	0	0	0	0
bA291L22.4	NC_000010	38741834	38744392	0	0	0	0
LOC100287112	NC_000010	38741842	38742248	0	0	0	0
ABCD1P2	NC_000010	38896985	38897774	0	0	0	0
LOC100129604	NC_000010	38897886	38931216	0	0	0	0
FKSG74	NC_000010	38989728	38991001	0	0	0	0
LOC100131485	NC_000010	39033340	39035964	0	0	0	0
LOC100288750	NC_000010	42606447	42611048	0.0213237	0	0.0569106	0.0297185
LOC100288777	NC_000010	42644462	42645990	0	0	0	0
LOC642424	NC_000010	42672462	42681264	0	0	0	0
LOC100128631	NC_000010	42725384	42743303	0	0	0	0
LOC653097	NC_000010	42746439	42772290	0	0	0	0

LOC100132766	NC_000010	42775177	42777243	0	0	0	0
LOC441666	NC_000010	42827314	42863493	0	0	0	0
CCNYL2	NC_000010	42905175	42950162	0	0.0509396	0.0220392	0.0345264
LOC84856	NC_000010	42970939	42990785	0	0	0	0
ZNF37B	NC_000010	43008958	43048280	0	0	0	0
LOC100128934	NC_000010	43050888	43051175	0	0.1568868	0.1357554	0
EIF3LP2	NC_000010	43062124	43063114	0	0	0	0
ZNF33B	NC_000010	43084555	43133992	0.9294188	0.9858748	1.9883455	3.5261273
LOC728064	NC_000010	43139894	43215776	0	0	0	0
LOC100129622	NC_000010	43230155	43234243	0	0	0	0
DUXAP3	NC_000010	43240749	43244181	0	0	0	0
LOC283028	NC_000010	43248353	43250788	0	0	0	0
BMS1	NC_000010	43277954	43330385	0.4914688	0.8365217	0.4955208	1.3014051
RET	NC_000010	43572517	43625799	0.6318546	0.1482812	0.1527487	0.1005036
LOC100288973	NC_000010	43600180	43602245	0	0.0647327	0	0.0877504
LOC100289008	NC_000010	43631781	43633868	0.189432	0.2596747	0.2246987	0.2493405
CSGALNACT2	NC_000010	43633934	43680750	1.2155392	1.1283717	0.9973869	1.817427
RASGEF1A	NC_000010	43689984	43701575	0.8470275	0.2141393	0.3582399	0.0774089
FXYD4	NC_000010	43867092	43871783	0	0	0.0507102	0
HNRNPF	NC_000010	43881065	43904696	41.365344	54.370291	61.56503	69.903625
ZNF487	NC_000010	43932574	43978007	0	0	0	0
LOC100130881	NC_000010	43991440	44020837	0	0	0	0
ZNF239	NC_000010	44051792	44070066	0.9809091	1.1345374	1.1375537	1.208395
LOC100129906	NC_000010	44068748	44069940	0	0	0	0
LOC100289101	NC_000010	44093979	44094411	0	0	0	0
LOC399748	NC_000010	44100291	44101699	0	0	0	0
ZNF485	NC_000010	44101855	44113352	0.235246	0.5276894	1.2366627	1.2816251
RPL21P88	NC_000010	44126395	44126862	0	0	0	0
ZNF32	NC_000010	44139307	44144326	4.5700663	6.9241179	6.6945793	8.2368711
LOC100132615	NC_000010	44218917	44224140	0	0	0	0
LOC100131995	NC_000010	44227782	44228057	0	0	0	0
HNRNPA3P1	NC_000010	44282860	44285865	0	0	0	0
LOC100288632	NC_000010	44320716	44321748	0	0	0	0
LOC100130539	NC_000010	44788209	44790179	0.1488509	0.0382586	0.3972657	0.414901
CXCL12	NC_000010	44865605	44880542	34.978862	26.861373	4.1690123	34.885324
LOC100289168	NC_000010	44876246	44886606	0.2622657	0.0385195	0.0666625	0.1305408
RPL9P21	NC_000010	44909863	44910650	0	0	0	0
LOC100129178	NC_000010	45301271	45303447	0	0	0	0
TMEM72	NC_000010	45406764	45430642	0.0810106	0.0416437	0.1081039	0.1129028
RASSF4	NC_000010	45455219	45490172	0.8631116	0.7062736	0.9558924	0.98196
C10orf10	NC_000010	45471709	45474330	3.8188027	0.5044749	2.0877341	2.9435563
C10orf25	NC_000010	45493146	45496336	0.1856163	0.4110251	0.3048543	0.3183873
ZNF22	NC_000010	45496273	45500777	3.5892385	5.3301654	5.9249487	5.1273049
DUXAP4	NC_000010	45550961	45552773	0	0	0	0
LOC100289237	NC_000010	45566223	45566660	0	0	0	0
RPS19P7	NC_000010	45568537	45571354	0	0	0	0
LOC100133308	NC_000010	45594924	45650044	0	0	0	0
LOC100289280	NC_000010	45727466	45744344	0	0	0	0
OR6D1P	NC_000010	45753656	45754668	0	0	0	0
OR13A1	NC_000010	45798102	45811056	0	0.0434874	0.018815	0.0589507
ALOX5	NC_000010	45869629	45941565	0	0.0176912	0.0153084	0.0239819
MARCH8	NC_000010	45952817	46090354	4.0432373	3.5982418	6.5826083	6.2363389
ANUBL1	NC_000010	46111039	46168251	0.3919707	0.7571273	1.0355571	0.8442534
LOC100289311	NC_000010	46159203	46168426	0	0.0600045	0.1038448	0
CTGLF10P	NC_000010	46174040	46196080	0	0	0	0
LOC100288690	NC_000010	46216994	46217973	0	0.1026895	0.0888581	0.208806
LOC648708	NC_000010	46221118	46221486	0	0	0	0
FAM21C	NC_000010	46222667	46288412	3.1391594	2.5525688	3.0719516	5.8730827
LOC643479	NC_000010	46310876	46312766	0	0	0	0
AGAP4	NC_000010	46321050	46342921	0.0542348	0.0185863	0.0321658	0.1133788
LOC100133130	NC_000010	46349814	46411606	0.3343887	0.245562	0.1699894	0.2663033
PTPN20A	NC_000010	46550123	46641003	0.4123257	0.0146177	0	0.0099078
FRMPD2L2	NC_000010	46657139	46676732	0	0	0	0.0298343
CTGLF8P	NC_000010	46717127	46733199	0	0	0	0
BMS1P1	NC_000010	46753698	46761170	0	0	0	0
LOC439962	NC_000010	46761948	46762753	0.0545263	0	0	0.0379961

LOC441554	NC_000010	46773063	46775211	0	0	0	0
CTSLL5	NC_000010	46775417	46791512	0	0	0	0
FAM35B	NC_000010	46897648	46939149	0	0	0	0
RHEBP1	NC_000010	46913771	46915114	0	0	0	0
SYT15	NC_000010	46955444	46970601	0.1393247	1.2085911	0.4880417	0.4065518
GPRIN2	NC_000010	46993546	47000568	0.0241076	0	0	0.0167992
PPYR1	NC_000010	47083534	47088320	0	0.2078991	0	0
LOC728643	NC_000010	47133295	47133836	0	0	0	0
LOC100132646	NC_000010	47151363	47151848	0	0.1859399	0	0
ANXA8L1	NC_000010	47157989	47174040	0.0224914	0	0.080036	0.0156729
FAM25B	NC_000010	47177221	47181688	0	0	0	0
AGAP10	NC_000010	47191740	47215264	0.0319314	0.0328288	0.0568141	0.0890042
BMS1P2	NC_000010	47230095	47241942	0	0	0	0
GLUDP2	NC_000010	47248344	47248741	0	0	0	0
LOC728657	NC_000010	47253863	47256011	0	0	0	0
LOC100133189	NC_000010	47270681	47273487	0.0756424	0	0	0.0527106
FAM35B2	NC_000010	47379726	47421236	0	0	0	0
LOC642862	NC_000010	47395842	47397188	0	0	0	0
LOC340844	NC_000010	47591487	47595656	0	0	0	0
LOC100288618	NC_000010	47655725	47657233	0.0873722	0.0299426	0.0259096	0
ANTXRL	NC_000010	47658234	47701446	0	0	0	0
CTGLF11P	NC_000010	47708227	47730335	0	0	0	0
LOC100288518	NC_000010	47740330	47744769	0	0	0	0
ANXA8L2	NC_000010	47746920	47763041	0.0432988	0.0667735	0	0
LOC728449	NC_000010	47768351	47770843	0	0.1930915	0	0.0163594
FAM21B	NC_000010	47894023	47949417	0.1320682	0.459563	0.379588	0.8778282
SLC9A3P4	NC_000010	47965760	47967028	0	0	0	0
ASAH2C	NC_000010	47999324	48055018	2.0405624	2.2231611	2.0682242	2.4901726
CTSLL2	NC_000010	48152062	48159662	0	0	0	0
LOC728725	NC_000010	48173342	48175490	0	0	0	0
LOC642826	NC_000010	48185856	48186665	0	0	0	0
BMS1P6	NC_000010	48187408	48197199	0	0	0	0
AGAP9	NC_000010	48214086	48237612	0.053219	0.0547147	0.1325662	0.0519191
FAM25G	NC_000010	48247662	48252126	0	0	0	0
ANXA8	NC_000010	48255225	48271369	0	0.022127	0.0191467	0
LOC653110	NC_000010	48276679	48279171	0	0	0	0
ZNF488	NC_000010	48355089	48373866	0.012571	0.1550918	0.0447341	0.0788398
RBP3	NC_000010	48381487	48390991	0.0102779	0.0211335	0.0091435	0.0286482
GDF2	NC_000010	48413092	48416853	3.2461762	2.5672387	2.2416476	1.9773294
GDF10	NC_000010	48425789	48439166	0.0164354	0.0168973	0.0292428	0
PTPN20B	NC_000010	48737042	48827924	0.4691982	0	0.0252977	0.039631
FRMPD2L1	NC_000010	48844040	48863632	0	0	0	0
CTGLF9P	NC_000010	48903537	48922780	0	0	0	0
BMS1P5	NC_000010	48927373	48952629	0	0	0	0
LOC728813	NC_000010	48962798	48964946	0	0	0	0
LOC644021	NC_000010	48980297	48981273	0.170342	0	0	0
LOC338598	NC_000010	49087556	49094511	0.0299171	0.0307579	0.0532302	0.0416949
FAM25C	NC_000010	49203372	49207825	0	0	0	0
CTGLF12P	NC_000010	49217898	49239743	0	0	0	0
BMS1P7	NC_000010	49258300	49268060	0	0	0	0
PTPN20C	NC_000010	49299174	49348566	0	0	0	0
FRMPD2	NC_000010	49364605	49482717	0	0.0178767	0.0154689	0.0605833
RPS6P14	NC_000010	49500754	49501460	0	0	0	0
MAPK8	NC_000010	49609687	49643183	3.8074694	6.5848208	5.7504143	5.9028476
ARHGAP22	NC_000010	49654079	49813138	0.9112584	1.0190509	0.7537908	1.7378978
WDFY4	NC_000010	49893518	50191001	0	0.0090249	0.0156187	0.0275266
RPL13AP19	NC_000010	49953247	49960153	0	0	0	0
LRRC18	NC_000010	50117528	50122280	0	0	0	0
C10orf72	NC_000010	50222333	50323559	0.9289071	1.5785821	1.3367936	2.3417009
FAM170B	NC_000010	50339199	50342077	0	0	0.0274369	0.0214911
C10orf128	NC_000010	50363890	50396407	0.1144485	0	0	0.0797523
C10orf71	NC_000010	50507187	50535537	0.0249422	0	0.0147929	0.0405551
DRGX	NC_000010	50574161	50599907	0.0544588	0	0	0.037949
ERCC6	NC_000010	50664491	50747147	0.7778448	0.5804319	0.66967	0.6207155
PGBD3	NC_000010	50723244	50732326	0	0.0416053	0	0.0140999
CHAT	NC_000010	50817141	50873150	0	0	0.014332	0.0561306

SLC18A3	NC_000010	50818347	50820766	0	0	0	0
C10orf53	NC_000010	50887684	50916956	0	0.0379055	0.0328	0.077076
OGDHL	NC_000010	50942687	50970425	0.081775	0.0360314	0.0519638	0.0325623
MAPK6PS6	NC_000010	50979109	50982464	0	0	0	0
RPL21P89	NC_000010	51023104	51023644	0	0	0	0
PARG	NC_000010	51026325	51371331	0.5063398	0.5949378	0.6618915	1.3681465
RPL35AP24	NC_000010	51117591	51118033	0	0	0	0
FAM21D	NC_000010	51187942	51192032	0.0293184	0.0602847	0.0260824	0
AGAP8	NC_000010	51224688	51246535	0	0	0	0.0126549
LOC100287519	NC_000010	51252705	51371319	0.1131229	0.1744533	0.1509559	0.157657
LOC728407	NC_000010	51287081	51371070	0	0	0	0
RPL35AP25	NC_000010	51339370	51339837	0	0	0	0
TIMM23	NC_000010	51371419	51387764	0.6209326	0.3546578	0.8592872	0.7692277
AGAP7	NC_000010	51464162	51486327	0.0544813	0	0.0646241	0.0506196
RPL23AP61	NC_000010	51532102	51532572	0	0	0	0
MSMB	NC_000010	51549553	51562595	0	0	0.0683524	0
NCOA4	NC_000010	51565108	51590734	6.158351	7.0951888	17.60444	20.954259
LOC100287932	NC_000010	51591918	51623392	21.217475	25.638501	28.944539	28.493879
LOC100287554	NC_000010	51623436	51632950	0	0	0	0
RPL35AP23	NC_000010	51654908	51655375	0	0	0	0
AGAP6	NC_000010	51748078	51770259	0.0634174	0.0162999	0.3244026	0.6407803
LOC100287995	NC_000010	51768933	51770949	0.0648682	0.0333457	0	0.0226014
SLC9A3P3	NC_000010	51785152	51786927	0	0	0	0
FAM21A	NC_000010	51827684	51893269	1.4298226	0.7446751	2.0586475	2.6547679
SLC9A3P	NC_000010	51910338	51912326	0	0	0	0
ASAH2	NC_000010	51947000	52008370	0.0181754	0.0186863	0.0485081	0.0886576
LOC728532	NC_000010	52024648	52027113	0	0	0	0
SGMS1	NC_000010	52065345	52383737	2.997801	3.2024474	2.927369	3.5170057
LOC644451	NC_000010	52389281	52391008	0	0	0	0
LOC644459	NC_000010	52407498	52420234	0	0	0	0
LOC729023	NC_000010	52436869	52445373	0	0	0	0
LOC644496	NC_000010	52461764	52468666	0	0	0	0
LOC653895	NC_000010	52486702	52487729	0	0	0	0
LOC729047	NC_000010	52497327	52500077	0	0	0	0
ASAH2B	NC_000010	52499708	52514569	1.1821518	1.9303036	1.1135383	0.7268563
A1CF	NC_000010	52566325	52645435	0	0.0198173	0	0.0537279
LOC100287708	NC_000010	52722710	52723187	0	0	0	0
LOC100288126	NC_000010	52724024	52724980	0	0.0472136	0	0
PRKG1	NC_000010	52750945	54055274	1.6744152	1.6364638	2.5286525	2.7441389
CSTF2T	NC_000010	53455246	53459355	0	0.0109935	0	0.0149026
LOC100288158	NC_000010	54073969	54074819	0.0720463	0	0.1281887	0.1506142
DKK1	NC_000010	54074041	54077417	56.511826	140.35642	27.249162	89.041197
RPL31P44	NC_000010	54148825	54149285	0	0	0	0
LOC399774	NC_000010	54170895	54173258	0	0	0	0
MBL2	NC_000010	54525140	54531460	0.0123139	0	0.0219095	0.0257424
PCDH15	NC_000010	55562531	56561051	0.0388048	0.0326417	0.0251068	0.039332
LOC389970	NC_000010	57426899	57428180	0	0	0	0
ZWINT	NC_000010	58117199	58121034	16.382647	27.949754	24.480864	59.975788
LOC100128586	NC_000010	59714560	59716752	0	0	0	0
MRPS35P3	NC_000010	59742233	59742771	0	0	0	0
IPMK	NC_000010	59955618	60027694	2.0099024	2.1419906	4.9716927	5.4656782
CISD1	NC_000010	60028895	60049019	2.3721455	3.7017727	6.8208765	6.2282885
UBE2D1	NC_000010	60094739	60130509	3.3653305	4.4314163	7.403613	5.6721295
TFAM	NC_000010	60145176	60155897	3.0872725	3.057348	5.6546066	5.1252379
BICC1	NC_000010	60272904	60588845	13.132335	13.25515	9.0254079	12.096778
RPLP1P10	NC_000010	60848456	60848784	0	0	0	0
LOC644871	NC_000010	60896382	60898175	0	0	0	0
PHYHIPL	NC_000010	60936348	61007534	0.1413126	0.012107	0.0523815	0.0902663
FAM13C	NC_000010	61005889	61122661	0.3340256	0.1717067	0.1167408	0.3075789
MRPL50P4	NC_000010	61311157	61311636	0	0	0	0
SLC16A9	NC_000010	61410522	61469649	0.4960297	0.9066145	0.451088	0.168986
CCDC6	NC_000010	61548521	61666818	7.0288886	6.3031391	11.185112	12.550533
C10orf40	NC_000010	61717975	61720671	0	0	0	0
ANK3	NC_000010	61788159	62149488	0.3663052	0.3047299	0.1492558	0.1422419
ARL4P	NC_000010	62443989	62445154	0	0	0	0
CDC2	NC_000010	62538220	62554610	13.707819	20.711873	33.974168	35.32466

RHOBTB1	NC_000010	62629196	62761198	1.0935796	0.9034673	0.8425825	1.8711045
TMEM26	NC_000010	63166401	63213208	0.2898622	0.0525898	0.1213503	0.0594081
C10orf107	NC_000010	63422719	63526091	0.1404097	0.4330677	0.4059651	0.1223038
ARID5B	NC_000010	63661443	63856703	7.7101134	3.9844694	6.0414515	9.8683336
RTKN2	NC_000010	63952953	64028466	0.483093	0.4286334	0.7594618	1.2819931
ZNF365	NC_000010	64133916	64431771	0.1900207	0.3724074	0.1796132	0.1282761
ATQL4	NC_000010	64500842	64501291	0	0	0	0
ADO	NC_000010	64564516	64568239	7.0336053	6.3213084	6.7507343	6.2499752
EGR2	NC_000010	64571757	64578927	0.586535	0.258437	0.4721028	0.2822121
NRBF2	NC_000010	64893007	64914786	3.4305582	4.2180707	9.8156336	7.0424298
JMJD1C	NC_000010	64926985	65225722	2.5273894	2.491571	4.8667076	7.6932538
LOC728737	NC_000010	64981915	64982900	0	0	0	0
LOC84989	NC_000010	65224989	65226323	0	0	0	0
REEP3	NC_000010	65281123	65381972	10.096215	8.6073002	22.787029	18.876887
LOC100287799	NC_000010	65382024	65384888	2.4299022	2.3131434	5.3642394	5.9995495
MRPL35P2	NC_000010	65394217	65394587	0	0	0	0
RPL7AP50	NC_000010	65662145	65663025	0	0	0	0
LOC645084	NC_000010	65928714	65929451	0	0	0	0
RPL17P35	NC_000010	66380137	66380679	0	0	0	0
ANXA2P3	NC_000010	66585285	66586634	0	0	0	0
LOC100129561	NC_000010	66929225	67013949	0	0	0	0
CTNNA3	NC_000010	67679725	69455949	0.2872433	0.3355868	0.1393853	0.1000813
LRRTM3	NC_000010	68685792	68860867	0	0	0	0
RPL7AP51	NC_000010	69093881	69094646	0	0	0	0
LOC340888	NC_000010	69510042	69510990	0	0	0	0
RPL21P92	NC_000010	69553487	69554037	0	0	0	0
DNAJC12	NC_000010	69556427	69597937	0.3451432	0.2365623	0.5117482	0.1603397
LOC100288295	NC_000010	69570600	69583144	0	0	0.0873689	0
LOC100129853	NC_000010	69593521	69593845	0	0	0	0
RPL12P8	NC_000010	69634090	69634649	0	0	0	0
SIRT1	NC_000010	69644427	69678147	1.2329553	1.5085578	1.8311403	2.2721913
HERC4	NC_000010	69681656	69835103	20.188749	10.643157	14.316443	48.489956
RPS3AP38	NC_000010	69720442	69721251	0	0	0	0
POU5F1P5	NC_000010	69769700	69770634	0	0	0	0
MYPN	NC_000010	69869250	69971774	1.2672236	0.0235453	2.220758	3.5854035
ATOH7	NC_000010	69990381	69991855	0	0.0918984	0.0265068	0.0415253
LOC100128041	NC_000010	70013732	70021293	0	0	0	0
PBLD	NC_000010	70042417	70092684	0.8623339	0.3641269	0.6575624	2.7040888
HNRNPH3	NC_000010	70091768	70102953	4.3729584	5.4137658	6.7107762	9.8273863
RUFY2	NC_000010	70103275	70167051	0.6715717	0.6904463	0.9700513	0.9560839
DNA2	NC_000010	70173821	70231879	0.9553963	1.9235687	1.7795767	2.1983891
RPL26P29	NC_000010	70184317	70184754	0	0	0	0
SLC25A16	NC_000010	70242097	70287584	3.335434	5.5257719	3.136415	3.9380005
RPL26P27	NC_000010	70259245	70259766	0	0	0	0
TMEM14D	NC_000010	70303776	70304699	0.047563	0.0488998	0.4654472	0.3314381
TET1	NC_000010	70320117	70454239	0.4539728	0.744885	0.3916284	0.1405984
RPS3AP37	NC_000010	70434061	70434907	0	0	0	0
CCAR1	NC_000010	70480971	70551309	0.9682736	1.2531426	1.2566351	2.1988313
SNORD98	NC_000010	70514929	70514995	0	0	0	0
STOX1	NC_000010	70587381	70655209	0.1343983	0.150737	0.1847814	0.2128501
DDX50	NC_000010	70661034	70706603	2.2684076	2.6526111	4.2825545	4.7421502
DDX21	NC_000010	70715892	70744279	5.6403582	8.6820021	8.181022	7.3372872
KIAA1279	NC_000010	70748493	70776738	12.899362	14.04832	15.001835	21.575517
LOC389976	NC_000010	70782513	70783628	0	0	0	0
SRGN	NC_000010	70847828	70864567	100.51318	68.027323	51.880661	18.316315
VPS26A	NC_000010	70883908	70932617	8.8319356	6.5383827	12.400671	15.221766
RPS12P17	NC_000010	70921270	70921737	0	0	0	0
SUPV3L1	NC_000010	70939993	70968849	7.4076041	7.451426	16.719978	9.8905731
HKDC1	NC_000010	70980059	71027315	0.297833	0.1347296	0.3709446	0.3237653
HK1	NC_000010	71029756	71161638	1.834165	2.1490823	1.7866922	4.9482492
RPS15AP28	NC_000010	71060149	71060603	0	0	0	0
TACR2	NC_000010	71163958	71176674	0.3638099	0.4114383	0.4369347	0.4056275
LOC100130962	NC_000010	71192725	71197342	0	0	0	0
TSPAN15	NC_000010	71211226	71267423	0.9312162	0.8776058	3.0375978	2.4694575
LOC645269	NC_000010	71283064	71283406	0	0	0	0
NEUROG3	NC_000010	71331791	71333122	0.0376592	0.0387176	0.0335026	0.0262424

LOC100287959	NC_000010	71349501	71350111	0	0	0	0
LOC100287996	NC_000010	71350824	71351018	0	0	0	0.1570507
LOC100288026	NC_000010	71355240	71356192	0	0	0	0
C10orf35	NC_000010	71390003	71393355	0.2450645	0.2519521	0.2906882	0.0853853
RPL5P26	NC_000010	71538646	71539652	0	0	0	0
COL13A1	NC_000010	71561644	71718904	38.094956	70.969817	23.562437	66.684922
H2AFY2	NC_000010	71812357	71872040	3.0455177	3.172584	0.1076574	0.0281091
AIFM2	NC_000010	71872030	71892690	2.4415684	1.1435305	2.3168928	7.2875868
LOC100288438	NC_000010	71888883	71899882	0.9380626	0.7715415	0.3755369	1.4707786
TYSND1	NC_000010	71897733	71906496	2.115341	1.7826171	3.0336091	2.6742398
SAR1A	NC_000010	71909960	71930285	15.550693	12.899038	17.207002	13.767239
CALM2P2	NC_000010	71923387	71924517	0	0	0	0
RPS25P9	NC_000010	71958729	71958995	0	0	0	0
PPA1	NC_000010	71962586	71993190	45.365917	36.889367	78.945853	82.889456
NPFFR1	NC_000010	72014713	72026147	0.0341744	0	0	0.0952562
LRRC20	NC_000010	72058729	72142382	1.7112105	1.0053167	2.1868539	5.6215011
C6orf182P	NC_000010	72149122	72150376	0	0	0	0
LOC100288471	NC_000010	72163581	72165086	0.0291821	0	0	0.0203352
EIF4EBP2	NC_000010	72163861	72188374	7.6563644	6.3416354	11.743287	21.267841
NODAL	NC_000010	72191692	72201465	0.0632046	0	0.1311999	0.0440434
KIAA1274	NC_000010	72238564	72328206	0.1058066	0	0.0085571	0.0469193
PRF1	NC_000010	72357104	72362531	0.0173434	0	0	0.0241712
ADAMTS14	NC_000010	72432559	72522197	1.0926586	1.2948745	0.1558263	0.5579784
C10orf27	NC_000010	72530995	72545157	0.0555252	0.0285429	0	0.1354227
RPS26P40	NC_000010	72553923	72554279	0	0	0	0
SGPL1	NC_000010	72575704	72640946	2.0518801	3.1955752	3.3803878	3.5639881
PCBD1	NC_000010	72643267	72648541	3.7570059	2.5151794	7.9671978	8.7064763
UNC5B	NC_000010	72972298	73060749	3.1743321	5.4332182	9.2776536	2.1078348
SLC29A3	NC_000010	73079010	73123147	0.1163164	0.2391711	0.3449278	0.1756169
CDH23	NC_000010	73156704	73575704	0.0348703	0.0478005	0.0275748	0.0431983
C10orf105	NC_000010	73471455	73479578	0.0090802	0	0	0
C10orf54	NC_000010	73507313	73533337	5.9285005	1.5143159	4.2340682	2.2580555
PSAP	NC_000010	73576055	73611082	190.01284	138.66245	316.78557	184.41997
RNU7-38P	NC_000010	73604393	73604648	0	0	0	0
CHST3	NC_000010	73724120	73773322	0.781526	0.7775718	1.0148658	1.6733226
SPOCK2	NC_000010	73818792	73848790	0.0144187	0.007412	0.0384819	0.0251188
LOC100288529	NC_000010	73829228	73846582	0	0	0	0
ASCC1	NC_000010	73856278	73975694	1.9687792	3.0470502	5.310941	6.8448668
RPL15P14	NC_000010	73949705	73950334	0	0	0	0
LOC100288565	NC_000010	73974092	73975711	0	0	0	0
C10orf104	NC_000010	73975806	73995618	2.2008504	2.1637122	4.7235244	4.3421189
DDIT4	NC_000010	74033677	74035797	7.2494515	9.4905776	37.245341	12.987605
LOC100288598	NC_000010	74034213	74046803	0.9572882	0.5368325	1.6645498	3.4263478
DNAJB12	NC_000010	74092588	74114907	3.9916284	4.2385347	4.2505152	7.326089
CBARA1	NC_000010	74127098	74385899	21.058905	20.737784	23.265067	34.059916
LOC100129990	NC_000010	74396255	74396535	0	0	0	0
CCDC109A	NC_000010	74451889	74647452	2.7719129	2.2982402	2.8504499	5.4208838
OIT3	NC_000010	74653339	74692787	0	0.0382748	0.0165597	0
PLA2G12B	NC_000010	74694938	74714510	0	0	0	0.0280448
LOC729046	NC_000010	74765552	74766353	0	0	0	0
P4HA1	NC_000010	74766975	74856732	23.234986	16.529782	27.776778	17.755319
NUDT13	NC_000010	74870210	74891581	0.4177589	0.64425	0.5017273	0.9606663
ECD	NC_000010	74894282	74927853	7.9580916	10.406109	11.448089	15.207559
FAM149B1	NC_000010	74927877	75001939	3.5921926	4.1836483	3.3954463	4.5467971
DNAJC9	NC_000010	75002582	75007025	15.75041	30.059382	25.439265	30.028151
MRPS16	NC_000010	75008601	75012451	12.947543	16.707909	19.860495	24.602218
TTC18	NC_000010	75013520	75118612	0.2735614	0.1834238	0.1904618	0.256934
ANXA7	NC_000010	75135189	75173841	43.848531	43.584352	45.767568	45.381512
RPL26P6	NC_000010	75181974	75182517	0	0	0	0
ZMYND17	NC_000010	75183337	75193319	0.2176553	0.130534	0.4034004	0.4676519
PPP3CB	NC_000010	75196563	75255782	5.0645105	4.9056265	5.3743638	13.66939
USP54	NC_000010	75257296	75335433	0.8200627	1.143698	1.3322227	2.2261797
RPS26P41	NC_000010	75297951	75298211	0	0	0	0
MYOZ1	NC_000010	75391412	75401515	0.0285193	0	0	0
SYNPO2L	NC_000010	75404644	75415832	0.0174398	0.0358598	0	0.0243055
LOC100288087	NC_000010	75404645	75408345	0	0.0514617	0	0.0348803

AGAP5	NC_000010	75434033	75457554	0.0543465	0.1676219	0.1772767	0.2019778
BMS1P4	NC_000010	75458909	75490280	0	0	0	0
GLUDP3	NC_000010	75490834	75497169	0	0	0	0
SEC24C	NC_000010	75504132	75531919	9.1292022	9.2261913	16.553892	20.227293
FUT11	NC_000010	75532049	75535977	2.8643303	4.5491071	2.9475305	5.168693
CHCHD1	NC_000010	75541808	75543406	7.2379197	10.385758	12.970756	16.908997
KIAA0913	NC_000010	75545605	75561551	8.9026238	7.9293579	7.0221506	9.9248749
NDST2	NC_000010	75561669	75571589	1.8806676	3.2903829	2.6123749	3.6556724
LOC100288127	NC_000010	75566373	75567728	0	0	0.1490377	0.1167403
CAMK2G	NC_000010	75572259	75634343	1.7655764	3.0638694	3.9717844	4.7959124
C10orf55	NC_000010	75669734	75671988	0.1430207	0.1260346	0.3817057	0.2705126
PLAU	NC_000010	75670862	75677259	24.36669	35.109065	22.243914	39.899971
VCL	NC_000010	75757872	75879918	33.237351	36.294864	30.309515	43.39919
AP3M1	NC_000010	75880015	75910826	4.4843427	4.2663178	7.8001616	11.893157
ADK	NC_000010	75910965	76469060	3.8422519	4.4592902	6.7834893	9.3848207
RPSAP6	NC_000010	76131213	76132250	0	0	0	0
MRPL35P3	NC_000010	76287342	76287711	0	0	0	0
LOC645646	NC_000010	76504140	76508496	0	0	0	0
MYST4	NC_000010	76586379	76792639	0.5756961	0.7081375	0.7545144	1.3861787
DUPD1	NC_000010	76797594	76818272	0.0662869	0	0.0589707	0.0461914
PPIAP13	NC_000010	76848751	76849602	0	0	0	0
DUSP13	NC_000010	76854190	76868970	0.0239761	0.02465	0.0426596	0.0167075
LOC100288189	NC_000010	76854194	76863846	0	0.036089	0.0312281	0.0244608
SAMD8	NC_000010	76871467	76936449	5.0293386	6.0424909	6.0870461	5.4810993
RPS26P42	NC_000010	76885374	76885629	0	0	0	0
VDAC2	NC_000010	76970563	76991206	26.228585	33.699663	31.657843	52.101007
COMTD1	NC_000010	76993729	76995770	0.9916524	0.7414712	1.7243029	1.193585
RPL39P25	NC_000010	77029150	77029303	0	0	0	0
HMGA1L5	NC_000010	77034894	77036294	0	0	0	0
SPA17P1	NC_000010	77141975	77142668	0	0	0	0
LOC100288678	NC_000010	77155424	77158600	0.0691662	0.1706644	0.0615322	0.0674769
ZNF503	NC_000010	77157602	77161513	2.3893916	3.7629169	0.946107	0.2887324
C10orf41	NC_000010	77161286	77168740	0	0	0	0
C10orf11	NC_000010	77542519	78317130	0.7357405	0.1512837	0.2181784	0.0341796
LOC100288222	NC_000010	78596222	78630006	0	0	0	0
KCNMA1	NC_000010	78629360	79397577	2.9132062	3.6568228	3.2266957	6.2112788
LOC399783	NC_000010	79488527	79494532	0	0	0	0
LOC100129156	NC_000010	79498001	79498810	0	0	0	0
IMPDH1P5	NC_000010	79539353	79541637	0	0	0	0
DLG5	NC_000010	79550549	79686348	2.7618223	3.842311	3.7639052	7.0675946
LOC100131132	NC_000010	79626666	79628009	0	0	0	0
LOC100128292	NC_000010	79686570	79689583	0	0	0	0
LOC100288330	NC_000010	79713253	79713640	0	0	0	0
POLR3A	NC_000010	79735894	79789259	1.4416963	2.2313786	1.5823048	2.6753771
RPS24	NC_000010	79793518	79816571	36.687865	65.775432	84.759824	94.709505
LOC401646	NC_000010	79827828	79830782	0	0	0	0
LOC100132987	NC_000010	80008497	80310751	1.4056819	0.4250555	0.3310236	0.8642957
LOC283050	NC_000010	80703083	80827205	0	0	0	0
ZMIZ1	NC_000010	80828792	81076285	2.9841751	4.1206306	4.7300033	9.1003113
PPIF	NC_000010	81107220	81115090	26.975948	21.903994	35.988945	39.056808
ZCCHC24	NC_000010	81142081	81205383	5.7997768	6.4299104	13.877932	11.981759
TPRX1P1	NC_000010	81258844	81259768	0	0	0	0
TPRX1P2	NC_000010	81258844	81259768	0	0	0	0
LOC729815	NC_000010	81265728	81270678	0	0	0.0196273	0.0461218
EIF5AP1	NC_000010	81272278	81273226	0	0	0	0
EIF5AL1	NC_000010	81272357	81276196	0.7668051	0.4588939	0.6923527	1.8662035
RPS12P18	NC_000010	81303835	81304112	0	0	0	0
RPS12P19	NC_000010	81303835	81304112	0	0	0	0
SFTPA2	NC_000010	81315608	81320151	0	0	0	0
SFTPA2B	NC_000010	81315622	81320163	0	0	0.0178773	0
LOC100288405	NC_000010	81355045	81355491	0	0	0	0
SFTPA1B	NC_000010	81370712	81373969	0.0454011	0.0466771	0	0.0316373
SFTPA1	NC_000010	81370716	81375202	0	0.0344648	0.0596454	0.1167997
LOC650623	NC_000010	81387007	81445192	0	0	0	0
FAM22B	NC_000010	81461963	81472511	0.2549201	0.7443205	0.7075662	0.6750263
LOC642361	NC_000010	81585280	81587969	0	0	0	0

LOC100288907	NC_000010	81586123	81587969	0.1903551	0.3180207	0.1693452	0.0663235
FAM22E	NC_000010	81600091	81614135	0.3033676	0.3472025	0.4990312	0.3310582
LOC642413	NC_000010	81629851	81633659	0	0	0	0
LOC100132402	NC_000010	81651904	81652931	0	0	0	0
LOC100288974	NC_000010	81664660	81686377	0.528587	0.2329041	0.0671779	0.3157204
MBL1P1	NC_000010	81679934	81682875	0	0	0	0
SFTPD	NC_000010	81697496	81708861	0.1370171	0.070434	0	0.0238697
LOC100130879	NC_000010	81741595	81743175	0	0	0	0
LOC642521	NC_000010	81784493	81788175	0	0	0	0.0646094
LOC642538	NC_000010	81791724	81792152	0	0	0.0911365	0
LOC727879	NC_000010	81800377	81800802	0	0	0	0
C10orf57	NC_000010	81838426	81852307	2.2664854	2.6408766	3.6293909	2.511962
RPL22P18	NC_000010	81885697	81886070	0	0	0	0
PLAC9	NC_000010	81892258	81904784	16.325495	10.070595	4.5313672	3.3218664
ANXA11	NC_000010	81914880	81965328	29.524197	30.337427	36.791812	46.812314
RPS12P2	NC_000010	81975607	81975875	0	0	0	0
EIF5AL3	NC_000010	82006775	82007553	0	0	0	0
LOC100130698	NC_000010	82009472	82013395	0	0	0	0
MAT1A	NC_000010	82031576	82049434	0.051643	0.0530945	0.0229715	0
DYDC1	NC_000010	82095861	82116500	0	0	0	0
DYDC2	NC_000010	82116558	82127829	0	0	0	0.0332518
C10orf58	NC_000010	82168242	82192753	0.3017041	0.1809404	2.7735115	2.1023944
TSPAN14	NC_000010	82214038	82282391	2.9855494	4.1466037	4.8214958	7.1361071
SH2D4B	NC_000010	82297658	82406316	0.0107165	0	0.028601	0.0373383
LOC642666	NC_000010	82409512	82413488	0	0	0	0.0745131
RPS7P9	NC_000010	82476224	82476890	0	0	0	0
LOC647532	NC_000010	82535800	82537833	0	0	0	0
LOC389990	NC_000010	82896296	82897091	0	0	0	0
NRG3	NC_000010	83635070	84746935	0.0357109	0.0489528	0.0847185	0.0746544
LOC728027	NC_000010	85071387	85074439	0	0	0	0
LOC100288691	NC_000010	85672483	85841753	0	0	0.151541	0.1780516
GHITM	NC_000010	85899185	85913311	20.098824	18.191439	30.891707	48.29452
LOC642934	NC_000010	85926984	85931832	0	0	0.0421083	0.0659664
C10orf99	NC_000010	85933554	85945050	0	0	0	0
PCDH21	NC_000010	85954517	85977122	0.0330128	0.1103069	0.0954495	0.0402581
LRIT2	NC_000010	85980241	85985284	0	0.0145941	0.0126284	0.0197835
LRIT1	NC_000010	85991276	86001217	0.0381992	0	0	0.0133094
RGR	NC_000010	86004809	86018944	0	0.0306328	0.0265068	0.0415253
LOC100288717	NC_000010	86053083	86053813	0	0	0	0
KIAA1128	NC_000010	86088410	86278276	3.4295194	4.1321975	4.5943205	6.3994665
RPL12P29	NC_000010	86148876	86149466	0	0	0	0
LOC100131699	NC_000010	86320148	86321071	0.0987601	0.2538393	0.4392985	0.1032299
RPS3AP5	NC_000010	86320175	86321062	0	0	0	0
GRID1	NC_000010	87359312	88126250	0.0450828	0.0231749	0.0534759	0.0157078
WAPAL	NC_000010	88195013	88281541	1.1715809	1.6490293	1.5447943	2.8476958
LOC100289138	NC_000010	88281703	88282410	0.2886583	0	0.5135969	0.1508615
RPL7AP8	NC_000010	88390523	88391385	0	0	0	0
OPN4	NC_000010	88414314	88426217	0.0913305	0	0.0975002	0.0254571
LDB3	NC_000010	88428321	88495825	0.0617347	0.0634697	0.0854325	0.0477991
BMPR1A	NC_000010	88516396	88684945	4.8129148	5.1730997	8.8769639	9.7396599
MMRN2	NC_000010	88695297	88717425	0.1051393	0.1945697	0.888581	0.3296937
SNCG	NC_000010	88718288	88723017	0.4256487	0.3282087	0.3786689	0.1483045
C10orf116	NC_000010	88728188	88730666	20.993129	10.018344	22.57419	14.400984
AGAP11	NC_000010	88730498	88769960	0.1671565	0.1002484	0.4461212	0.3397372
BMS1P3	NC_000010	88752010	88760192	0	0	0	0
FAM25A	NC_000010	88780046	88784481	0	0	0	0.1720499
GLUD1	NC_000010	88810243	88854623	25.148396	33.631081	47.845951	51.968574
FAM35A	NC_000010	88854953	88951222	3.0598505	4.0086332	5.8118003	5.9198502
FAM22A	NC_000010	88985205	88994735	0.4408181	0.1922698	0.1901401	0.2234034
LOC728190	NC_000010	88998424	89102315	0	0	0	0
LOC439994	NC_000010	89086611	89103402	0	0	0	0
FAM22D	NC_000010	89117477	89130452	0.0872146	0.05706	0.3244611	0.6077461
CTSLL1	NC_000010	89146118	89149969	0	0	0	0
MINPP1	NC_000010	89264223	89313168	3.7356769	5.7451322	5.3283651	4.1521612
RPS26P38	NC_000010	89402363	89402783	0	0	0	0
PAPSS2	NC_000010	89419476	89507462	16.859353	19.10164	10.194959	23.194892

ATAD1	NC_000010	89512875	89577917	6.6776977	8.5482112	12.680292	8.4183087
CFLP1	NC_000010	89577970	89604948	0	0	0	0
KILLIN	NC_000010	89618918	89623194	0.0924793	0.1056427	0.1919684	0.1360469
PTEN	NC_000010	89623195	89728532	8.0035547	9.9882529	12.287605	15.041862
RPL11P3	NC_000010	89705237	89705837	0	0	0	0
LOC100128990	NC_000010	89807887	89810066	0	0	0	0
RNLS	NC_000010	90033621	90343008	1.3412987	1.2257743	1.1932565	0.2769394
LIPJ	NC_000010	90346519	90366733	0	0.030447	0	0
RPL7P34	NC_000010	90378339	90378692	0	0	0	0
LIPF	NC_000010	90424094	90438572	0	0	0.0261173	0
LOC100288843	NC_000010	90459438	90460182	0	0	0	0
LIPK	NC_000010	90484301	90512513	0.0366235	0.0376528	0	0.0255207
LIPN	NC_000010	90521163	90537999	0.2202919	0.1132416	0.032663	0.0511694
LOC100130253	NC_000010	90545325	90552454	0	0	0	0
LIPM	NC_000010	90562487	90580303	0.0592294	0.030447	0.0263461	0
ANKRD22	NC_000010	90579656	90611732	0.0220513	0	0.0882785	0.0307324
LOC100132487	NC_000010	90636063	90637302	0	0	0	0
STAMBPL1	NC_000010	90640026	90683244	2.1787055	1.8326768	2.7213629	4.6773099
LOC100132116	NC_000010	90692594	90694684	0	0	0	0
ACTA2	NC_000010	90694831	90751147	28.752733	3.3930269	7.1393718	2.3990315
FAS	NC_000010	90750288	90775542	8.743884	4.4360052	12.824118	5.9905861
LOC100289238	NC_000010	90898819	90899207	0	0	0	0
CH25H	NC_000010	90965694	90967071	3.0617056	0.852517	1.3051437	0.0222241
LIPA	NC_000010	90973326	91011660	28.830172	20.797629	31.622382	19.467602
LOC100128465	NC_000010	90994415	91123237	0	0	0	0
IFIT2	NC_000010	91061706	91069033	18.333769	3.7103394	22.451735	1.3406599
IFIT3	NC_000010	91087602	91100725	85.149699	30.727403	56.275477	6.4029693
IFIT1L	NC_000010	91137813	91144962	0.2674335	0.0916499	0.0594791	0.0621194
IFIT1	NC_000010	91152322	91163745	211.00347	77.328928	91.829908	5.8254739
IFIT5	NC_000010	91174325	91180759	12.561307	7.6836425	9.8373895	5.0332906
SLC16A12	NC_000010	91190056	91295313	0.0571127	0.0097863	0.1016181	0.192359
PANK1	NC_000010	91342749	91405215	0.4196124	0.5202245	1.218711	0.9976091
FLJ37201	NC_000010	91451057	91457685	0	0	0	0
KIF20B	NC_000010	91461367	91534700	2.4098397	3.981295	5.82436	8.1294249
LOC643529	NC_000010	91589681	91597461	0	0	0	0
LOC100288975	NC_000010	91597350	91600285	0	0	0	0
LOC119358	NC_000010	91738474	91738827	0.1241475	0	0	0
HTR7	NC_000010	92500575	92617671	0.1835547	0.0539181	0.8981243	0.8039944
RPP30	NC_000010	92631709	92668312	1.7369943	2.1055434	3.387466	2.9863749
ANKRD1	NC_000010	92671857	92681032	1.1354406	0.3433389	0.0594188	0.3257966
DDX18P6	NC_000010	92812766	92816782	0	0	0	0
NUDT9P1	NC_000010	92911752	92912840	0	0	0	0
PCGF5	NC_000010	92980369	93044021	4.828324	3.9877031	8.2561858	7.1287601
LOC100188947	NC_000010	93066719	93299337	0	0	0	0
HECTD2	NC_000010	93170096	93274520	0.4443019	0.371625	0.6632383	1.2699144
RPS27P1	NC_000010	93304615	93304845	0	0	0	0
PPP1R3C	NC_000010	93388199	93392858	6.1708719	12.44257	7.2690145	9.1958016
LOC441572	NC_000010	93426338	93427609	0	0	0	0
LOC100128043	NC_000010	93525656	93526962	0	0	0	0
TNKS2	NC_000010	93558151	93625232	8.3147197	10.860148	11.12355	10.34334
LOC728475	NC_000010	93656501	93669104	0	0	0	0.0763713
FGFBP3	NC_000010	93666345	93669258	0.034281	0	0.0152487	0
BTAF1	NC_000010	93683736	93790082	3.2461762	4.839245	4.9094102	7.0602525
CPEB3	NC_000010	93808399	94050844	0.1128807	0.0618951	0.5757518	0.3303711
LOC100289102	NC_000010	93874712	93898129	0	0	0	0
LOC100128302	NC_000010	93878412	93879028	0.1940319	0.0997426	0.0863081	0.0676046
NHP2P1	NC_000010	93976056	93976812	0	0	0	0
MARCH5	NC_000010	94050920	94113721	3.7612343	4.2697509	4.9494371	5.3121605
LOC643768	NC_000010	94149510	94180660	0	0	0	0
IDE	NC_000010	94213600	94333852	5.7625458	8.0645783	7.6583939	10.940383
KIF11	NC_000010	94352825	94415152	5.2187443	9.2626864	8.2229618	12.990545
RPL11P4	NC_000010	94356609	94357047	0	0	0	0
LOC283014	NC_000010	94428213	94429604	0	0	0	0
HHEX	NC_000010	94449681	94455408	0.4497261	0.7192355	0.6223603	0.487491
EXOC6	NC_000010	94594470	94819251	0.2708492	0.2905685	0.6704834	0.8452204
CYP26C1	NC_000010	94821021	94828454	0.0280103	0.0575952	0.0498376	0.0195187

LOC100132649	NC_000010	94831468	94833223	0	0	0	0
CYP26A1	NC_000010	94833232	94837641	0.0177282	0	0	0.0370612
LOC389997	NC_000010	94866626	94881857	0	0	0	0
LOC387703	NC_000010	94966614	94969384	0	0	0	0
RPL17P34	NC_000010	95043716	95044350	0	0	0	0
MYOF	NC_000010	95066186	95242074	14.848504	17.405151	15.963554	36.729267
CEP55	NC_000010	95256369	95288849	4.1335809	7.2245846	9.8552931	14.86309
GPR120	NC_000010	95326422	95347366	0	0	0	0.0514704
RBP4	NC_000010	95351593	95360993	12.678284	0.8268222	0.0841713	0
PDE6C	NC_000010	95372345	95425430	0	0.0304265	0	0.0928027
C10orf4	NC_000010	95428329	95462329	0.7874058	0.6212718	0.9774391	1.3653592
LGI1	NC_000010	95517566	95557916	0.0185749	0.019097	0.0330495	0.0647187
LOC100129731	NC_000010	95641043	95641695	0	0	0	0
TMEM20	NC_000010	95653730	95662491	0.749845	1.090569	0.5043797	0.994066
PIPSL	NC_000010	95717897	95721672	0	0	0	0
PLCE1	NC_000010	95753746	96088149	1.5012346	1.2042123	2.6661878	4.537019
NOC3L	NC_000010	96092989	96122683	4.8476076	4.4841502	5.7576741	5.9627601
TBC1D12	NC_000010	96162186	96296089	1.544189	1.3282301	2.0606301	1.9443522
HELLS	NC_000010	96305574	96361856	1.7301337	2.7993589	2.5358537	4.9905013
LOC100130970	NC_000010	96404671	96407745	0	0	0	0
CYP2C18	NC_000010	96443251	96495947	0.0172685	0.0177538	0.030725	0.0240667
CYP2C19	NC_000010	96522463	96612671	0.0596717	0.0306744	0.0265428	0.0207908
RPL7AP52	NC_000010	96642321	96643106	0	0	0	0
CYP2C9	NC_000010	96698415	96749148	0	0.0242791	0	0.0164561
CYP2C8	NC_000010	96796529	96829254	0.0456842	0	0	0.0477519
C10orf129	NC_000010	96953957	96988686	0	0	0.0456481	0
PDLIM1	NC_000010	96997329	97050781	7.0522989	13.69194	11.228433	6.5594608
LOC100289239	NC_000010	97031395	97055446	0.4092014	0	0.0728074	0.1140591
SORBS1	NC_000010	97071530	97321171	0.0289896	0.0238435	0.010316	0.036362
LOC100289463	NC_000010	97081825	97096743	0	0	0.0698171	0
RPS3AP36	NC_000010	97354318	97355182	0	0	0	0
ALDH18A1	NC_000010	97365686	97416567	6.8276491	8.9244721	14.744851	17.907993
TCTN3	NC_000010	97423153	97453900	10.172756	11.761871	12.061863	13.260718
ENTPD1	NC_000010	97515526	97637023	0.4569061	0.6380153	0.5490464	0.9622993
C10orf131	NC_000010	97667722	97698415	0.0373709	0.0768425	0	0.0520831
RPL21P90	NC_000010	97759491	97760043	0	0	0	0
CC2D2B	NC_000010	97759883	97792441	0	0	0.0432256	0.0507875
CCNJ	NC_000010	97803159	97820627	1.3163239	0.9385924	2.5498412	2.5964571
LOC728558	NC_000010	97848666	97849969	0.3487955	0.0717197	0.0620596	0.4374983
ZNF518A	NC_000010	97889472	97923517	0.5298159	0.5338123	0.7824227	1.2109657
LOC399804	NC_000010	97949022	97949975	0	0	0	0
BLNK	NC_000010	97951455	98031333	0.0241076	0	0.1072341	0.0167992
DNTT	NC_000010	98064085	98098321	0.0215433	0	0	0.0300244
OPALIN	NC_000010	98102973	98119092	0.0484411	0.0249013	0.0215473	0.0421947
TLL2	NC_000010	98124366	98273668	0.0195239	0.1338173	0.0405276	0.049885
TM9SF3	NC_000010	98277867	98346809	9.9491925	12.907441	17.243682	23.058438
PIK3AP1	NC_000010	98353069	98480279	0.0365398	0.1314836	0.0243801	0.0254624
RPS2P36	NC_000010	98380634	98381464	0	0	0	0
RPL13AP5	NC_000010	98510023	98510680	0	0	0	0
LCOR	NC_000010	98592737	98718665	0.6364039	0.7477601	1.2940857	1.235385
C10orf12	NC_000010	98741041	98745585	10.404466	9.6232196	4.1377182	5.2355402
SLIT1	NC_000010	98757795	98945683	0.0554132	0.0284853	0.0197189	0.0810897
ARHGAP19	NC_000010	98981930	99052413	0.5309506	1.2406206	1.0305783	1.2164742
RPL12P27	NC_000010	99069287	99069803	0	0	0	0
FRAT1	NC_000010	99079022	99081672	0.0994679	0.0511317	0.25072	0.1039698
FRAT2	NC_000010	99092254	99094458	0.7573845	1.1270236	1.595819	1.5277717
RRP12	NC_000010	99116458	99161127	2.0454893	2.9809203	1.6342182	1.7229089
RPL34P20	NC_000010	99152955	99153303	0	0	0	0
PGAM1	NC_000010	99186027	99193198	73.45998	53.85231	92.356633	108.21977
EXOSC1	NC_000010	99195666	99205768	7.3756598	9.19384	10.199365	7.0570372
ZDHHC16	NC_000010	99205930	99217127	10.215355	11.16457	12.288371	20.875675
MMS19	NC_000010	99218081	99258366	8.7753387	9.3651978	11.731391	17.090443
UBTD1	NC_000010	99258768	99330960	6.0686383	7.7482134	5.976378	8.241377
ANKRD2	NC_000010	99332256	99343641	0.0605765	0.0934185	0.0538905	0.0633181
NPL2	NC_000010	99344102	99372559	0.2828527	0.0545254	0.2359065	0.1601462
C10orf62	NC_000010	99349484	99350691	0	0	0	0

MORN4	NC_000010	99374310	99393913	0.5975867	0.5792744	1.6860256	1.3920399
PI4K2A	NC_000010	99400443	99436187	13.294734	13.398471	23.636043	29.819923
AVPI1	NC_000010	99437181	99447015	24.026296	8.1148351	18.284853	16.148463
MARVELD1	NC_000010	99473481	99477905	0	0	0	0
LOC100270710	NC_000010	99477217	99493298	0	0	0	0
ZFYVE27	NC_000010	99496880	99520652	2.8856357	2.6107284	3.5683267	4.3836004
LOC100289281	NC_000010	99526508	99531680	0	0	0	0.0507314
SFRP5	NC_000010	99526508	99531756	0	0	0	0
GOLGA7B	NC_000010	99609996	99627782	0	0.0312256	0.0135099	0.0317466
CRTAC1	NC_000010	99624758	99790585	0.0456368	0.0312796	0	0.021201
LOC100128300	NC_000010	99628991	99629761	0	0	0	0
C10orf28	NC_000010	99894381	100004654	2.4900956	2.6673085	3.3750791	4.851286
LOXL4	NC_000010	100007443	100028007	7.7873815	0.1729745	2.5444957	0.2261066
PYROXD2	NC_000010	100143324	100174941	0.9978374	1.6726333	1.6017265	0.9825356
HPS1	NC_000010	100175956	100206704	6.0439404	4.6141046	6.4597616	12.48008
HPSE2	NC_000010	100218877	100995619	0.0383325	0	0.0170508	0.3205395
LOC641380	NC_000010	100770917	100773979	0	0	0	0
RPL7P36	NC_000010	100807380	100808126	0	0	0	0
CNNM1	NC_000010	101088856	101154087	0.0442506	0.1516476	0.0459277	0.0256963
GOT1	NC_000010	101156627	101190530	9.1326368	10.982519	17.058082	11.144173
NKX2-3	NC_000010	101292690	101296281	0.2305344	0.2154669	0.2237343	0.0438124
SLC25A28	NC_000010	101370275	101380221	16.364004	12.116706	15.060734	8.4292269
LOC100289312	NC_000010	101380818	101381675	0.0512217	0.1053226	0.1367048	0.10708
ENTPD7	NC_000010	101419263	101471002	1.0942108	0.8080725	0.9917208	1.4748626
COX15	NC_000010	101468505	101492423	1.7147184	1.7452816	2.5119258	2.4096828
CUTC	NC_000010	101491958	101515894	2.3773166	3.2698515	8.1167461	8.3278178
ABCC2	NC_000010	101542463	101611662	0.087009	0.1789087	0.1857734	0.2182727
NANOGP6	NC_000010	101548231	101549171	0	0	0	0
DNMBP	NC_000010	101635334	101769676	2.0738072	3.099299	3.6348518	5.0866009
NCRNA00093	NC_000010	101686966	101718755	0	0	0	0
CPN1	NC_000010	101802065	101841642	0	0	0	0.0348803
LOC644566	NC_000010	101874169	101877873	0	0	0	0
LOC644573	NC_000010	101902826	101903756	0	0	0	0
ERLIN1	NC_000010	101909847	101945814	3.8840296	5.5596513	6.88846	10.974132
CHUK	NC_000010	101948124	101989344	1.1224009	1.294984	1.5643464	2.2160454
LOC100289414	NC_000010	101989352	102008889	0	0	0	0
CWF19L1	NC_000010	101992055	102027437	1.9195012	3.1575184	3.7271132	6.3855139
SNORA12	NC_000010	101996913	101997059	0	0	0	0
BLOC1S2	NC_000010	102033713	102046439	4.7878901	4.9224543	6.8262638	5.3032615
PKD2L1	NC_000010	102047903	102090243	0.0432136	0.0148094	0.0128147	0.0401506
SCD	NC_000010	102106772	102124588	52.700906	38.035698	204.94046	113.95277
C10orf75	NC_000010	102133333	102148111	0	0	0	0
WNT8B	NC_000010	102222812	102243399	0	0	0.0391759	0.0460294
SEC31B	NC_000010	102246403	102279595	0.2954023	0.0783754	0.3984357	0.869874
NDUFB8	NC_000010	102283497	102289636	8.712769	10.274943	9.6889011	13.526732
HIF1AN	NC_000010	102295641	102313681	3.0074528	3.2750122	2.3417813	6.2339704
PAX2	NC_000010	102505468	102589695	0.0208979	0	0.0092957	0.0291249
FAM178A	NC_000010	102672326	102724891	2.1735914	2.61427	2.8872864	5.4153748
LOC100289594	NC_000010	102672555	102673643	0	0	0.0359023	0
LOC100289632	NC_000010	102727881	102729911	0.151471	0.0889875	0.3272568	0.2563382
SEMA4G	NC_000010	102732286	102745372	0.0099882	0.010269	0.0533149	0
MRPL43	NC_000010	102737579	102747272	5.3958328	5.2847891	5.8699833	6.4308055
C10orf2	NC_000010	102747312	102754158	0.390434	0.3637753	0.6512643	0.8077078
LZTS2	NC_000010	102756965	102767590	20.308439	19.710106	18.309173	18.002161
PDZD7	NC_000010	102777089	102790879	0.1957121	0.1564987	0.3869131	0.2121466
SFXN3	NC_000010	102790996	102800998	9.9559101	8.2178226	10.830911	15.252973
KAZALD1	NC_000010	102820999	102825351	3.7235375	3.4867064	3.5458413	2.6556179
TD1	NC_000010	102849078	102890903	0.0233519	0.0480164	0	0.0162725
TLX1	NC_000010	102891061	102897546	0.2294402	0	0.018556	0.0581393
LOC100129417	NC_000010	102896232	102899468	0	0	0	0
LOC728940	NC_000010	102899466	102903827	0	0	0	0
RNY5P7	NC_000010	102925966	102926040	0	0	0	0
LBX1	NC_000010	102986733	102988717	0.034201	0	0	0.0238326
FLJ41350	NC_000010	102989351	102998525	0.0313245	0	0.0278671	0.0436563
BTRC	NC_000010	103113825	103317070	1.0913464	1.365615	2.1589572	2.2614679
POLL	NC_000010	103338639	103347973	2.2162911	1.8734993	3.4467783	4.5302397

RP11-529I10.4	NC_000010	103348089	103369410	0.6050445	0.904799	1.0275956	1.6864764
FBXW4	NC_000010	103370421	103454743	2.8212699	3.6971952	3.2522387	4.208844
LOC100289476	NC_000010	103433262	103455305	0	0	0	0
FGF8	NC_000010	103529887	103535827	0	0.040927	0.0708289	0.0277399
NPM3	NC_000010	103541082	103543170	3.8586247	5.873327	4.7255895	6.9141686
MGEA5	NC_000010	103544200	103578222	3.4807135	3.5872251	8.1096258	9.5548207
KCNIP2	NC_000010	103585731	103603677	0.0477698	0.0491124	0.2266526	0.2330154
LOC100289509	NC_000010	103587961	103599623	0	0.0406691	0.0351913	0
C10orf76	NC_000010	103605356	103815932	4.4366174	4.4841855	4.6428955	7.9978651
HPS6	NC_000010	103825147	103827793	5.6284286	7.0327018	7.0307674	8.1103286
LDB1	NC_000010	103867317	103880210	10.226637	9.0603455	18.649232	21.023813
PPRC1	NC_000010	103892787	103910090	3.6472587	3.5466177	5.9254272	9.3458087
NOLC1	NC_000010	103911933	103923627	4.8570835	6.9680704	10.539257	13.974157
ELOVL3	NC_000010	103986143	103989346	0.5355459	0.5161851	1.8461912	0.9562986
PITX3	NC_000010	103989946	104001231	0.094989	0.0325529	0.2816827	0.1985763
GBF1	NC_000010	104005309	104142649	10.76649	14.612324	11.871532	22.627635
NFKB2	NC_000010	104154229	104162281	7.8629005	7.0717152	16.921258	8.2233469
PSD	NC_000010	104162376	104178901	0.2206342	0.0864134	0.4673388	0.4246337
FBXL15	NC_000010	104179571	104182893	0.8558927	1.3964388	1.2911135	1.0113211
LOC100286939	NC_000010	104181242	104182896	0.3838274	0.3551534	0.1707317	0.0802399
CUEDC2	NC_000010	104183002	104192423	23.554194	30.525241	53.056312	44.93868
C10orf95	NC_000010	104209594	104211300	0.1196684	0.0307579	0.0532302	0.1667795
TMEM180	NC_000010	104221170	104236802	0.7753746	0.8768832	1.2140387	1.1022363
ACTR1A	NC_000010	104238986	104262512	31.481191	44.601506	36.403059	50.131849
SUFU	NC_000010	104263764	104393209	1.4804937	2.1032698	2.8497001	2.4322331
RPL23AP58	NC_000010	104332514	104332976	0	0	0	0
TRIM8	NC_000010	104404252	104418076	19.587714	12.003899	43.329336	24.140529
LOC100289540	NC_000010	104416007	104416838	0.2787414	0.0955252	0.3306348	0.064746
ARL3	NC_000010	104433488	104474190	2.4983491	3.2457327	7.3042739	7.0429307
SFXN2	NC_000010	104474298	104498946	0.411693	0.6878035	2.029655	1.2909785
C10orf26	NC_000010	104503727	104576021	3.1491567	2.9190369	4.0822981	5.085569
CYP17A1	NC_000010	104590288	104597290	0	0	0.0209078	0
LOC649704	NC_000010	104597774	104605369	0	0	0	0
LOC100289608	NC_000010	104605579	104605707	0	0	0.3030819	0
C10orf32	NC_000010	104613967	104624718	3.6277596	1.7999138	3.4521465	4.6408953
AS3MT	NC_000010	104629210	104661656	1.4429699	1.8131969	2.2504476	3.2027639
RPL22P17	NC_000010	104647599	104647971	0	0	0	0
CNNM2	NC_000010	104678114	104838241	0.3958386	0.4904434	0.6410917	0.8133628
NT5C2	NC_000010	104845940	104953056	4.7547052	3.53983	6.789747	8.6373468
RPS15AP29	NC_000010	104884774	104885173	0	0	0	0
LOC729081	NC_000010	104934937	104936052	0	0	0	0
LOC401648	NC_000010	104975466	104976557	0	0	0	0
LOC729020	NC_000010	105005644	105007773	0	0.0636386	0	0.1006451
INA	NC_000010	105036920	105050108	2.7340126	3.3702259	2.4685557	6.4453473
PCGF6	NC_000010	105062553	105110891	2.9758668	4.9843573	6.6448329	6.1524419
TAF5	NC_000010	105127724	105148822	0.9010194	2.5854639	2.6080995	2.858197
USMG5	NC_000010	105148814	105156223	20.027042	17.485078	45.036436	51.115303
PDCD11	NC_000010	105156412	105206019	0.513734	0.5422572	0.5423447	1.5751574
LOC100286901	NC_000010	105200559	105202975	0	0	0	0
CALHM2	NC_000010	105206543	105212162	11.680443	16.552565	12.979028	17.410141
CALHM1	NC_000010	105213144	105218648	0.0287619	0.0295703	0.0383811	0.0601274
CALHM3	NC_000010	105232561	105238997	0.026603	0.0273507	0	0.0185381
NEURL	NC_000010	105253735	105352309	0.0509249	0.1047124	0.0362434	0.0567785
SH3PXD2A	NC_000010	105353784	105615164	1.5239451	3.6558101	1.397459	2.4451979
OBFC1	NC_000010	105637316	105678045	0.6846786	1.3799651	1.5076957	1.2471029
SLK	NC_000010	105727470	105787342	0.9329004	1.6603248	1.3251048	2.58394
COL17A1	NC_000010	105791046	105845638	0.3290242	0.5074072	0.1881701	0.2893259
C10orf78	NC_000010	105881816	105886143	2.2707065	2.925544	3.7333189	3.7855474
C10orf79	NC_000010	105889646	105992120	0.0737249	0.1852814	0.1894756	0.273997
LOC751602	NC_000010	105916167	105917303	0	0	0	0
GSTO1	NC_000010	106014678	106027209	6.1336201	5.4382071	6.5579784	12.234266
GSTO2	NC_000010	106028631	106059176	0.6471627	0.2117027	0.4187156	0.1844872
ITPRIP	NC_000010	106071899	106093663	4.8036908	4.3174791	2.553347	2.5965181
CCDC147	NC_000010	106113522	106214848	0	0.0818293	0.047205	0.1663893
SORCS3	NC_000010	106400859	107024993	0.0152678	0.0156969	0.0135826	0.0319175
YWHAZP5	NC_000010	107445976	107449482	0	0	0	0

RPL23AP59	NC_000010	108309864	108310242	0	0	0	0
SORCS1	NC_000010	108333421	108924292	0.0059126	0.0182363	0.03156	0.0082402
LOC100128304	NC_000010	109673555	109675175	0.0271118	0.0836213	0.0964776	1.7003325
LOC100129859	NC_000010	110597370	110609323	0	0	0	0
RNU5B-6P	NC_000010	110944688	110944765	0	0	0	0
LOC645318	NC_000010	111287877	111288781	0	0	0	0
LOC100130409	NC_000010	111294413	111294893	0	0	0	0
LOC100132843	NC_000010	111421572	111440802	0	0	0	0
RPL21P91	NC_000010	111567369	111567925	0	0	0	0
XPNPEP1	NC_000010	111624524	111683202	14.197268	14.8566	16.234339	13.737085
RNU4P5	NC_000010	111629564	111629766	0	0	0	0
ADD3	NC_000010	111765726	111895323	21.575277	20.307147	13.145163	9.0392359
MXI1	NC_000010	111967363	112047123	2.8305641	2.0500422	9.2977783	10.780596
SMNDC1	NC_000010	112052798	112064707	3.0757264	4.4982983	7.977522	9.4183953
LOC645360	NC_000010	112210387	112211061	0	0	0	0
DUSP5	NC_000010	112257625	112271302	10.88275	4.2716903	32.308075	18.29255
RPL7P35	NC_000010	112309804	112310631	0	0	0	0
SMC3	NC_000010	112327449	112364392	0.9298386	1.7031681	1.692453	5.474048
RBM20	NC_000010	112404155	112599227	0.0243043	0.0124937	0.0108109	0.0423405
LOC282997	NC_000010	112628648	112630662	0	0	0	0
PDCD4	NC_000010	112631596	112659764	1.5992106	1.2732942	2.0110376	2.7315213
NCRNA00081	NC_000010	112658488	112679124	0	0	0	0
SHOC2	NC_000010	112679301	112773425	11.969686	12.111487	16.780156	17.209521
RPL13AP6	NC_000010	112696361	112697013	0	0	0	0
ADRA2A	NC_000010	112836790	112840665	0.1814168	0.781034	0.3026127	0.0237035
LOC724065	NC_000010	112864590	112865393	0	0	0	0
RPS6P15	NC_000010	113257934	113258699	0	0	0	0
GPAM	NC_000010	113909622	113943525	1.2347722	3.8722551	2.3135744	3.0187449
TECTB	NC_000010	114043493	114063070	0.0443922	0	0	0.0618684
GUCY2G	NC_000010	114067936	114116353	0	0	0	0
ACSL5	NC_000010	114133916	114188138	1.6551017	4.1514664	1.7334925	0.8343316
ZDHHC6	NC_000010	114190058	114206672	2.1265273	3.602179	7.8915824	8.2560156
VTI1A	NC_000010	114206756	114578503	1.9516978	2.5725007	3.2944886	5.8236787
LOC143188	NC_000010	114610673	114615127	0	0	0	0
TCF7L2	NC_000010	114710009	114926073	1.0321176	1.3007343	1.40692	2.0764596
RPS15AP30	NC_000010	114747241	114747623	0	0	0	0
HABP2	NC_000010	115312778	115349361	0.0145572	0.0149663	0.0129505	0.0304321
NRAP	NC_000010	115348583	115423805	0.007979	0.0082032	0.0070983	0.0222403
CASP7	NC_000010	115438935	115490664	2.7318364	3.5299533	3.4661904	2.9543021
C10orf81	NC_000010	115511213	115542191	0	0	0	0.0282778
DCLRE1A	NC_000010	115594484	115613859	1.2787086	2.103435	3.001447	3.3174667
NHLRC2	NC_000010	115614420	115668452	3.5847969	4.9967527	6.8995704	8.2146732
LOC100286977	NC_000010	115670143	115676956	0.870709	0.9283352	2.3926746	4.0000208
LOC100132839	NC_000010	115800691	115804503	0.0345777	0.0118498	0	0.0160634
ADRB1	NC_000010	115803806	115806667	0	0	0.0273218	0
LOC100287173	NC_000010	115875425	115877315	0	0	0	0
C10orf118	NC_000010	115881974	115934364	0.5671191	0.8277985	0.5045249	0.6488942
TDRD1	NC_000010	115939029	115992063	0.0389785	0.0300555	0.0433454	0.0407426
VWA2	NC_000010	115999018	116049751	0.0298359	0.0306744	0.0265428	0.0415816
AURKAPS2	NC_000010	116005759	116006956	0	0	0	0
AFAP1L2	NC_000010	116054583	116164515	0.0110534	0.011364	0.0196668	0.053917
ABLIM1	NC_000010	116190869	116444414	1.6302608	1.4420814	3.6163953	5.4182761
PPIAP19	NC_000010	116449902	116450393	0	0	0	0
LOC100130319	NC_000010	116528300	116571095	0	0	0	0
FAM160B1	NC_000010	116581503	116659586	7.8131778	6.533514	7.8411351	8.7222124
RPL15P13	NC_000010	116643057	116661103	0	0	0	0
TRUB1	NC_000010	116697952	116737439	3.7611031	3.0457745	4.9502193	4.6583563
ATRNL1	NC_000010	116853124	117708496	0.3422446	0.201804	0.3044703	0.2069249
GFRA1	NC_000010	117816436	118033126	1.3961265	0.6259132	0.0529391	0.0765542
C10orf96	NC_000010	118083940	118139541	0.0258671	0	0.0230121	0.0180252
LOC100131273	NC_000010	118145676	118168994	0	0	0	0
PNLIPRP3	NC_000010	118187424	118237469	0.3997024	0.2152522	0.4571824	0.1193694
PNLIP	NC_000010	118305428	118327367	0.0293379	0	0	0.0408877
PNLIPRP1	NC_000010	118350490	118368686	0.118779	0.0305293	0.0792518	0.041385
PNLIPRP2	NC_000010	118380465	118404654	0	0	0	0.0206785
C10orf82	NC_000010	118423207	118429481	0	0	0	0

HSPA12A	NC_000010	118430703	118502085	0.9984743	0.8291257	1.0044289	0.7332416
RPL5P27	NC_000010	118509375	118510843	0	0	0	0
C10orf134	NC_000010	118608703	118641531	0.0505733	0.1559841	0.2099601	0.2114491
KIAA1598	NC_000010	118644306	118765088	0.7467744	0.2979377	1.0510631	2.8659853
VAX1	NC_000010	118888032	118897812	0.007593	0.0078064	0.0135099	0.0052911
LOC100287068	NC_000010	118934609	118936905	0	0.0196706	0.0170211	0
RPL12P26	NC_000010	118955637	118956234	0	0	0	0
KCNK18	NC_000010	118957000	118969810	0	0.0391198	0.0677014	0.0795451
SLC18A2	NC_000010	119000716	119037095	0.1160196	0.1192804	0.0206429	0.1616942
PDZD8	NC_000010	119042606	119134937	3.5277705	6.6179494	7.0410263	7.582391
EMX2OS	NC_000010	119243804	119304579	0	0	0	0
EMX2	NC_000010	119301956	119309057	0.5614933	1.5758023	0.0405016	0.0105749
RAB11FIP2	NC_000010	119764427	119806114	1.6967309	1.8189655	2.5351169	2.9306104
LOC100129413	NC_000010	119799524	119805321	0	0	0.0451995	0.0354045
CASC2	NC_000010	119806332	119969665	0	0	0	0
C10orf84	NC_000010	120068572	120101839	5.1638378	5.9787148	7.8873614	7.4513894
PRLHR	NC_000010	120352916	120355160	0.0665882	0	0.0197462	0.0464013
LOC100287264	NC_000010	120353677	120355211	0.0733693	0.2640099	0.4895355	0.0511267
LOC390007	NC_000010	120380919	120403710	0	0	0	0
C10orf46	NC_000010	120440494	120514758	3.8666619	4.5719703	7.825314	8.905751
LOC100287147	NC_000010	120489799	120514542	0.1727978	0.1776543	0	0.1605498
RPL17P36	NC_000010	120631520	120632149	0	0	0	0
LDHAL5	NC_000010	120691610	120692797	0	0	0	0
NANOS1	NC_000010	120789228	120793854	0.2374553	0.0781213	0.1689975	0.3507929
EIF3A	NC_000010	120794541	120840334	1.7314195	2.7595518	2.5868512	8.9074811
SNORA19	NC_000010	120819523	120819650	0	0	0	0
FAM45A	NC_000010	120863611	120897223	12.064615	10.747106	16.484766	14.035233
SFXN4	NC_000010	120900425	120925204	2.3113984	1.4648797	4.2252412	4.7878952
PRDX3	NC_000010	120927215	120938345	10.275765	10.42257	19.880535	23.868541
GRK5	NC_000010	120967197	121215131	0.8074929	0.5828977	1.0240566	2.0352734
RGS10	NC_000010	121259339	121302222	9.5206128	16.329114	26.292511	19.274179
TIAL1	NC_000010	121332977	121356541	5.3054421	5.9931319	6.7327535	10.050366
LOC100133264	NC_000010	121355308	121356881	0	0.1468583	0.0847185	0
LOC100128880	NC_000010	121380580	121381419	0	0	0	0
RPS8P4	NC_000010	121397945	121398646	0	0	0	0
BAG3	NC_000010	121410882	121437331	2.6324495	3.1282169	3.3455715	4.8242224
LOC93202	NC_000010	121442309	121443518	0	0	0	0
INPP5F	NC_000010	121485609	121588659	0.695045	4.8463138	1.268372	1.626869
LOC100128743	NC_000010	121485616	121586927	0	0	0	0
C10orf119	NC_000010	121588972	121632394	13.150273	19.07057	27.693725	27.909728
SEC23IP	NC_000010	121652223	121701245	8.64878	9.9886946	9.3067502	12.068064
LOC651144	NC_000010	121756521	121757260	0	0	0	0
RPL21P16	NC_000010	122114157	122114738	0	0	0	0
PPAPDC1A	NC_000010	122216466	122349367	0.7512518	4.158893	0.4626931	1.2483514
C10orf85	NC_000010	122357461	122359633	0	0	0	0
BRWD2	NC_000010	122610695	122669036	7.7123379	7.6899778	10.850319	12.22013
RPL19P16	NC_000010	122892530	122893268	0	0	0	0
LOC100129699	NC_000010	122942781	122947809	0	0	0	0.1479463
FGFR2	NC_000010	123237844	123357972	0.0157662	0.0081046	0	0.0274663
LOC729426	NC_000010	123383126	123408584	0	0	0	0.0541076
RPS15AP5	NC_000010	123473091	123473551	0	0	0	0
ATE1	NC_000010	123502625	123687546	3.8722565	5.1986036	6.5720031	8.0950277
LOC100130887	NC_000010	123687827	123711429	0.0488314	0.2008151	0.2172087	0.1361106
NSMCE4A	NC_000010	123716603	123734710	6.2462866	7.2122194	9.9168752	8.5490732
TACC2	NC_000010	123748689	124014057	0.1175319	0.1611136	0.2091193	0.5884733
LOC100130255	NC_000010	123921860	123923892	0	0.022225	0	0
BTBD16	NC_000010	124030821	124097676	0	0.0488998	0.0211567	0.0331438
PLEKHA1	NC_000010	124134220	124191866	3.0217304	3.7018014	6.1489059	5.6877079
ARMS2	NC_000010	124214179	124216868	0	0	0	0.0379021
HTRA1	NC_000010	124221041	124274424	58.832586	55.626735	56.082876	26.854551
DMBT1	NC_000010	124320181	124403252	0.0343078	0.0117573	0.1678662	0.07969
C10orf120	NC_000010	124457225	124459338	0.9913135	1.0616401	2.2414958	2.4465363
FLJ46361	NC_000010	124516210	124557161	0	0	0	0
CUZD1	NC_000010	124591674	124605691	0.0965471	0.2779295	0.360742	0.7535119
FAM24B	NC_000010	124608610	124639157	0.3415666	0.8779158	1.0128903	2.3801719
LOC399815	NC_000010	124639149	124658230	0	0	0	0

FAM24A	NC_000010	124670217	124672627	0	0	0	0
C10orf88	NC_000010	124690419	124713919	2.4913964	2.7663307	3.6222558	4.3452208
PSTK	NC_000010	124739556	124749906	1.8301023	1.855037	3.5543241	6.0172048
IKZF5	NC_000010	124753197	124768311	2.4121165	2.8886855	5.8952904	7.1113258
ACADSB	NC_000010	124768429	124817806	0.8802962	0.7149032	1.2108992	2.8867079
HMX3	NC_000010	124895567	124897247	0.3273611	0.0420702	0.0728074	0.0570296
HMX2	NC_000010	124907638	124910188	0.1619714	0.0277539	0.3602356	0.4326611
BUB3	NC_000010	124913760	124924886	36.793129	38.235882	60.895449	72.828265
RPS26P39	NC_000010	124930960	124932166	0	0	0	0
GPR26	NC_000010	125425871	125456915	0.0042643	0.0131526	0.0037937	0.0178293
CPXM2	NC_000010	125505152	125651500	0.1416162	0.1577294	0.052494	0.0164473
LOC100287396	NC_000010	125531607	125537357	0	0	0	0.0255847
LOC100287436	NC_000010	125618355	125651350	0.0481889	0	0.0428701	0
LOC100124332	NC_000010	125751207	125752187	0	0	0	0
GALNAC4S-6ST	NC_000010	125767184	125806241	0.9484554	0.5503107	0.2506254	0.0392627
OAT	NC_000010	126085872	126107519	30.610603	37.375815	47.064688	48.738916
OATL3	NC_000010	126132353	126132812	0	0	0	0
NKX1-2	NC_000010	126135998	126138544	0	0	0.037922	0
LHPP	NC_000010	126150412	126302709	2.833612	2.2689743	2.7632501	1.2151223
RPS10P18	NC_000010	126178762	126179147	0	0	0	0
FAM53B	NC_000010	126307863	126432930	0.9111219	1.3381844	0.6198395	1.1897819
METTL10	NC_000010	126447406	126480439	0.6475505	0.8409474	0.9853981	1.4012158
FAM175B	NC_000010	126490354	126525238	4.6431432	5.5440684	5.5424165	6.9010926
LOC100287472	NC_000010	126556080	126556889	0	0	0	0
ZRANB1	NC_000010	126630692	126676005	5.4423954	6.4364862	10.688145	9.1155799
CTBP2	NC_000010	126676418	126849624	0.2978177	0.4054921	0.5513759	0.9086502
MRPS21P6	NC_000010	126855188	126855665	0	0	0	0
LOC642622	NC_000010	126915917	126947022	0	0	0	0
RPS27P18	NC_000010	127162248	127162504	0	0	0	0
LOC100169752	NC_000010	127262940	127267014	0	0	0	0
C10orf122	NC_000010	127344263	127371713	0.0953324	0	0.0424052	0
ALDOAP2	NC_000010	127355249	127356685	0	0	0	0
LOC100287412	NC_000010	127389006	127408115	0.1549291	0.5840393	1.2404633	0.6477648
C10orf137	NC_000010	127408084	127452712	2.7545627	3.4733692	4.2265331	4.889012
MMP21	NC_000010	127455022	127464390	0	0	0.0407479	0.0319175
UROS	NC_000010	127477147	127511837	7.9846861	9.8776122	11.781246	11.738783
BCCIP	NC_000010	127512104	127542264	1.1205301	1.5404955	2.4689539	3.27767
DHX32	NC_000010	127524909	127569884	1.2883846	2.2812467	1.6046558	2.2345188
FANK1	NC_000010	127585108	127698161	0.3051961	0.522956	0.1810072	0.2599334
ADAM12	NC_000010	127702898	128077127	9.4125347	21.145961	5.5245952	27.143682
SAR1P2	NC_000010	127961297	127961891	0	0	0	0
C10orf90	NC_000010	128113571	128210010	0.9693415	0.3663915	0.4819032	2.3939655
LOC728152	NC_000010	128213578	128216249	0	0	0	0.1142719
LOC728065	NC_000010	128496358	128585313	0.4840114	0.0995229	0.172236	0.0337278
DOCK1	NC_000010	128768965	129250780	3.4870097	3.3741294	3.2963922	3.6701902
C10orf141	NC_000010	128933690	128994422	0	0.0102526	0	0.0208474
NPS	NC_000010	129347613	129350935	0	0	0	0.0951083
LOC387720	NC_000010	129532826	129535254	0	0	0	0
FOXI2	NC_000010	129535538	129539450	0	0	0.0123026	0.0289096
CLRN3	NC_000010	129676114	129691211	0.0384499	0	0	0
PTPRE	NC_000010	129705325	129884180	1.3040546	0.1308005	1.4925975	2.5100543
MKI67	NC_000010	129894923	129924655	2.3145237	4.01464	5.6377679	10.729814
MGMT	NC_000010	131265454	131565783	6.5661785	4.9290984	8.5921923	9.2721964
LOC100129103	NC_000010	131566059	131586940	0.0971232	0.0832107	0.0144006	0.1466385
EBF3	NC_000010	131633547	131762091	1.5620215	4.7866846	0.3855068	0.4705037
LOC387723	NC_000010	131877695	131909079	0	0	0	0
CTAGEP	NC_000010	131904029	131907098	0	0	0	0
GLRX3	NC_000010	131934663	131977870	13.63667	19.8313	25.520276	34.80995
TCERG1L	NC_000010	132890655	133109984	0.1511403	0.1035921	0.1792781	0.245748
FLJ46300	NC_000010	133607899	133608375	0	0	0	0
LOC100289353	NC_000010	133664246	133665973	0.050866	0.0784434	0	0.0354455
PPP2R2D	NC_000010	133747957	133770054	2.6364471	4.8697922	7.1755065	8.0649146
LOC100289387	NC_000010	133769199	133770533	0	0.135381	0.0878597	0.1376399
BNIP3	NC_000010	133781204	133795435	35.038258	22.208791	43.35395	76.182878
JAKMIP3	NC_000010	133918313	133980537	0.0173366	0	0.0616924	0.0724849
DPYSL4	NC_000010	134000414	134019280	0.7111887	0.3655884	0.0431382	0.3491619

STK32C	NC_000010	134020996	134121477	1.1614445	0.9595343	1.9927027	3.2373633
LRRC27	NC_000010	134145614	134195010	0.2948556	0.2475664	0.3628646	0.7088617
PWWP2B	NC_000010	134210702	134231363	2.0655837	1.156778	2.9730285	3.0777008
C10orf91	NC_000010	134258714	134261825	0	0	0.1059555	0
INPP5A	NC_000010	134351353	134596984	1.2864356	2.8604877	1.3174469	2.5642342
NKX6-2	NC_000010	134598320	134599537	0.0431288	0	0	0.0601077
C10orf92	NC_000010	134621896	134671237	0.0159986	0.0328965	0.0142328	0.0445939
C10orf93	NC_000010	134742689	134756064	0	0.1157065	0	0.0196062
LOC100289424	NC_000010	134777759	134778163	0	0	0	0
LOC100128127	NC_000010	134898760	134901995	0	0.0139627	0	0.0283914
GPR123	NC_000010	134901409	134945179	0	0.021099	0.0456427	0.0143007
RPL5P28	NC_000010	134957011	134958854	0	0	0	0
KNDC1	NC_000010	134973971	135039916	0.1881734	0.0800533	0.2482202	0.1085187
UTF1	NC_000010	135043778	135045062	0.0379846	0.1171566	0.0337922	0.0529384
VENTX	NC_000010	135051408	135055433	0.0182282	0.037481	0	0
ADAM8	NC_000010	135075921	135090372	0.2557478	0.0830323	1.8081876	0.150076
TUBGCP2	NC_000010	135093138	135122644	9.4410367	11.415944	16.027801	25.942087
ZNF511	NC_000010	135122423	135126666	11.353293	20.58355	14.73399	19.565894
CALY	NC_000010	135138928	135150475	0	0	0	0
PRAP1	NC_000010	135160844	135166187	0.3393686	0	0.1509559	0.0394143
C10orf125	NC_000010	135168658	135171529	5.1501834	8.4130553	0.712716	0.1993807
ECHS1	NC_000010	135175987	135186908	12.103125	16.591046	11.002626	20.623977
PAOX	NC_000010	135192741	135205198	1.0898214	1.2665967	1.0749196	2.2122554
MTG1	NC_000010	135207621	135234174	8.0598988	6.7467161	8.5995265	9.0128979
SPRN	NC_000010	135234170	135238121	0.6371316	0.6407983	0.4312685	0.9362159
OR6L2P	NC_000010	135243898	135244822	0	0	0	0
LOC619207	NC_000010	135267432	135281949	0	0	0	0
OR7M1P	NC_000010	135294625	135295374	0	0	0	0
CYP2E1	NC_000010	135340867	135352620	0.237273	0.4065693	0.1641769	0.29394
SYCE1	NC_000010	135367404	135382876	0.2628769	0.0245695	0	0
OR6L1P	NC_000010	135388560	135389639	0	0	0	0
FRG2B	NC_000010	135438603	135440299	0	0.1015357	0	0.03441
LOC644425	NC_000010	135450912	135451396	0	0	0	0
LOC644435	NC_000010	135455490	135456498	0	0	0	0
LOC440013	NC_000010	135480349	135481965	0	0	0	0
LOC399839	NC_000010	135483659	135485275	0	0	0	0
LOC440014	NC_000010	135486968	135488584	0	0	0	0
LOC100289581	NC_000010	135490278	135491883	0	0.0281341	0.0243447	0
LOC728022	NC_000010	135493577	135495193	0	0	0	0
LOC440017	NC_000010	135496887	135498492	0	0	0	0
RPL23AP60	NC_000010	135515566	135516575	0	0	0	0
LOC100287555	NC_000011	75771	76052	0	0	0	0
OR4F2P	NC_000011	86652	87586	0	0	0	0
LOC100132561	NC_000011	89189	125169	0	0	0	0
LOC100288633	NC_000011	125788	126191	0	0	0	0
LOC100288692	NC_000011	126287	128107	0	0	0	0
LOC100133161	NC_000011	128856	131344	0	0.0345175	0.0597365	0.0701869
LOC653486	NC_000011	193080	194500	0	0	0	0
SCGB1C1	NC_000011	193080	194573	0	0	0	0
ODF3	NC_000011	196761	200258	0.171138	0.5982226	0.1217993	0.1192558
BET1L	NC_000011	202924	207422	9.6043835	9.4157563	8.8882152	13.292192
RIC8A	NC_000011	208530	215113	31.440389	38.711908	33.931825	46.934723
SIRT3	NC_000011	215030	236362	1.3478265	1.1054519	1.6840785	1.5090826
PSMD13	NC_000011	236808	252984	0.8827821	1.4963016	1.9315301	2.7931485
COX8B	NC_000011	253316	257071	0	0	0	0
NLRP6	NC_000011	278570	285304	0	0.0168658	0.0583764	0.0114315
ATHL1	NC_000011	289138	295688	0.8068831	0.4147803	1.5313612	1.7524028
LOC100288718	NC_000011	290414	291646	0	0	0	0.0789301
IFITM5	NC_000011	298203	299526	0	0	0	0
MRPS24P1	NC_000011	303710	304368	0	0	0	0
IFITM2	NC_000011	308107	309410	80.792503	18.692311	178.5634	69.430375
IFITM1	NC_000011	313991	315272	203.25291	61.693934	520.81011	25.883788
IFITM3	NC_000011	319673	320914	61.84332	28.949844	161.71643	51.026182
B4GALNT4	NC_000011	369804	381792	0.6338687	0.2751553	0.1127814	0.0196313
PKP3	NC_000011	394217	404908	0.4850107	0.7881761	0.821202	1.5263378
SIGIRR	NC_000011	405716	417397	2.3556443	1.687956	2.2438235	1.2435657

ANO9	NC_000011	417930	442011	0.045859	0.1100118	0.0951941	0.0745649
PTDSS2	NC_000011	450280	491387	2.5703874	3.640544	5.2130088	9.2438283
RNH1	NC_000011	494512	507273	66.232124	56.278917	68.327396	72.934283
LOC100128703	NC_000011	528484	531297	0.0751252	0	0.0668334	0.4449769
HRAS	NC_000011	532242	535550	7.8351791	8.8243111	7.6674318	12.309513
LRRC56	NC_000011	537522	554916	0.4911302	0.1303054	0.3241687	0.2097594
C11orf35	NC_000011	554857	560779	0.1471246	0.1512596	0.3365644	0.5419036
RASSF7	NC_000011	560971	564025	1.8509785	1.620122	3.315616	6.7280609
LOC100288751	NC_000011	573808	575885	0.1057465	0.0434874	0	0.0736884
LOC143666	NC_000011	573808	575885	0	0	0	0
PHRF1	NC_000011	576483	612222	0.8036885	0.7935524	0.9202759	1.1200843
IRF7	NC_000011	612555	615999	15.211268	6.0230447	17.866381	1.9767227
MUPCDH	NC_000011	616575	625007	0.0385624	0.1057231	0.0457415	0.1343589
SCT	NC_000011	626313	627173	0.1710048	0	0	0
DRD4	NC_000011	637305	640703	0	0.1661154	0.0574964	0.0450366
DEAF1	NC_000011	644225	695740	1.8446607	1.5138769	3.512447	5.0064234
TMEM80	NC_000011	695616	704129	1.8954875	1.3919717	3.0353008	4.4531554
EPS8L2	NC_000011	706120	727727	1.1193437	0.8631022	1.0704841	2.2522594
TALDO1	NC_000011	747432	765024	49.428502	52.562509	64.952915	71.326647
PDDC1	NC_000011	767222	777487	5.3789493	3.7385304	4.3192818	9.9462417
NS3BP	NC_000011	778754	781182	0	0	0	0
CEND1	NC_000011	787110	790126	0.4272003	0.8509632	0.9738762	1.0977326
SLC25A22	NC_000011	790475	796221	6.0321102	5.5617913	4.78427	6.3607227
LRDD	NC_000011	799179	805245	3.9731267	1.9170503	4.0067349	6.5467674
RPLP2	NC_000011	809936	812876	56.161026	88.533806	116.18242	188.59459
SNORA52	NC_000011	811681	811814	0	0	0	0
PNPLA2	NC_000011	818902	825217	9.7568468	5.1681787	9.3592628	16.716572
EFCAB4A	NC_000011	827585	831991	0.6036845	0.2730865	0.2577861	0.3870177
CD151	NC_000011	832952	838835	204.22167	170.49515	188.06712	181.75242
POLR2L	NC_000011	839721	842529	40.336048	42.965495	24.324399	23.353218
TSPAN4	NC_000011	842824	867116	48.169248	28.360881	56.658337	34.362152
CHID1	NC_000011	867859	915058	3.8916695	4.5574037	7.5388547	8.1436341
AP2A2	NC_000011	925841	1012240	2.5325454	2.8386453	3.1423737	3.0651416
MUC6	NC_000011	1012824	1036706	0.0164744	0.0056458	0.0244268	0.0191334
MUC2	NC_000011	1074875	1104417	0.0101933	0.0052399	0.0136023	0.0248607
MUC5AC	NC_000011	1142474	1288726	0.0211019	0.0168739	0.0208587	0.0277754
MUC5B	NC_000011	1244296	1283406	0	0.0025208	0	0.0017086
LOC100287643	NC_000011	1294844	1298366	0.1163091	0.0170826	0.1921619	0.0347352
TOLLIP	NC_000011	1295601	1330839	7.7555703	7.6601161	6.5958157	8.370007
LOC100287677	NC_000011	1299918	1307248	0	0	0	0
LOC255512	NC_000011	1331005	1331937	0.0942084	0.0968562	0.1257156	0.0984723
BRSK2	NC_000011	1411129	1483332	0.1629569	0.1804243	0.1895775	0.1659648
HCCA2	NC_000011	1490693	1502115	1.7504274	2.2495293	2.8641887	2.5266614
DUSP8	NC_000011	1575281	1593150	0.216155	0.0707095	0.5506699	0.855826
LOC338651	NC_000011	1593971	1620414	0	0	0	0
KRTAP5-1	NC_000011	1605572	1606513	0	0	0	0.0325105
KRTAP5-2	NC_000011	1618407	1619524	0.0786194	0	0	0
KRTAP5-3	NC_000011	1628795	1629693	0	0	0	0
LOC100131343	NC_000011	1639803	1650266	0	0	0	0
KRTAP5-4	NC_000011	1642190	1643368	0	0	0	0.0259753
KRTAP5-5	NC_000011	1651033	1652160	0	0.0400562	0	0
FAM99A	NC_000011	1686829	1689086	0	0	0	0
FAM99B	NC_000011	1704500	1706859	0	0	0	0
KRTAP5-6	NC_000011	1718425	1718985	0	0	0	0
LOC100287770	NC_000011	1772028	1785203	1.1590054	0.5474824	2.8703131	3.0341112
CTSD	NC_000011	1773985	1785222	191.73453	56.223497	331.33343	217.62443
RPL36AP39	NC_000011	1813755	1814150	0	0	0	0
LOC390029	NC_000011	1824119	1825325	0	0	0	0
SYT8	NC_000011	1855674	1858750	0	0.0635044	0.0274755	0.064564
TNNI2	NC_000011	1860903	1862908	0	0	0.0558537	0
LSP1	NC_000011	1874200	1913493	0.3437484	0.4240914	0.0917424	0.095815
TNNT3	NC_000011	1940799	1959936	0.2409065	0.3184421	0.3674008	0.1678733
MRPL23	NC_000011	1968502	1977839	15.458879	25.149252	34.091813	38.590649
LOC100133545	NC_000011	2004439	2011150	0	0	0	0
H19	NC_000011	2016406	2019065	0	0	0	0
IGF2	NC_000011	2150347	2170833	0.0069704	0	0	0.0048572

INS-IGF2	NC_000011	2150350	2182439	0.0530776	0	0	0.0739731
IGF2AS	NC_000011	2161737	2169894	0.0212208	0	0	0
INS	NC_000011	2181009	2182439	0	0	0	0
TH	NC_000011	2185159	2193035	0.0460191	0.0236562	0.0614098	0.0641359
ASCL2	NC_000011	2289728	2292182	0	0.0244367	0	0
C11orf21	NC_000011	2317507	2323143	0.0194289	0	0.0172845	0
TSPAN32	NC_000011	2323243	2339430	0.1596956	0	0.1420696	0.2893339
RPL26P30	NC_000011	2356541	2356994	0	0	0	0
CD81	NC_000011	2398547	2418649	192.6148	101.142	116.74423	99.77123
TSSC4	NC_000011	2423523	2425106	4.6293783	6.9488518	4.7499149	6.043252
TRPM5	NC_000011	2425745	2444275	0.0223712	0.023	0.019902	0.0467674
KCNQ1	NC_000011	2466221	2870340	0.0250631	0.0515351	0.0334453	0.0261975
KCNQ1OT1	NC_000011	2661768	2721228	0	0	0	0
KCNQ1DN	NC_000011	2891263	2893335	0	0	0	0
CDKN1C	NC_000011	2904448	2906995	0.1821688	0.3745774	1.5598511	0.1428103
SLC22A18AS	NC_000011	2909327	2925175	0.6818084	0.1752427	1.9207595	0.2573519
SLC22A18	NC_000011	2920951	2946476	3.4076783	1.306843	4.6195278	1.6961471
PHLDA2	NC_000011	2949503	2950650	8.5030275	4.9603517	6.0346243	5.4592174
NAP1L4	NC_000011	2965660	3013607	7.2083478	8.0030966	10.20139	24.750448
SNORA54	NC_000011	2985001	2985123	0	0	0	0
CARS	NC_000011	3022159	3078671	1.9393526	1.7687453	2.1844547	4.1959353
LOC100287800	NC_000011	3105525	3106838	0	0	0	0
OSBPL5	NC_000011	3108346	3186582	2.2952125	1.0810539	1.9714749	1.1424254
MRGPRG	NC_000011	3239174	3240043	0	0	0	0
C11orf36	NC_000011	3239562	3244361	0	0	0	0
MRGPRE	NC_000011	3249041	3253616	0.0339107	0	0.0301679	0
LOC100128558	NC_000011	3359924	3367618	0	0	0	0
ZNF195	NC_000011	3380011	3400448	1.8821904	2.1041751	1.6907055	2.3812275
LOC650368	NC_000011	3402191	3430378	0	0	0	0
OR7E12P	NC_000011	3412037	3412978	0	0	0	0
LOC387745	NC_000011	3429470	3443771	0	0	0	0
LOC728199	NC_000011	3443872	3468523	0	0	0	0
LOC100287886	NC_000011	3486554	3486727	0	0	0	0
RPS3AP39	NC_000011	3570925	3571773	0	0	0	0
RPS24P14	NC_000011	3580742	3581254	0	0	0	0
LOC100132021	NC_000011	3599634	3602749	0	0	0	0
OR7E117P	NC_000011	3620774	3621999	0	0	0	0
TRPC2	NC_000011	3647714	3658789	0	0	0	0
ART5	NC_000011	3659736	3663546	0.0844076	0.0578533	0.0250305	0.0196062
ART1	NC_000011	3666361	3685646	0	0.0344386	0.0298	0
CHRNA10	NC_000011	3686817	3692614	0.0225955	0.0232305	0.0402032	0.2046907
NUP98	NC_000011	3696240	3818892	6.8897739	8.8483422	10.977453	17.016497
RNU7-50P	NC_000011	3802626	3802681	0	0	0	0
FRAG1	NC_000011	3818954	3847582	1.9178894	1.9508154	1.9603236	2.8719709
RHOG	NC_000011	3848208	3862213	7.7300908	5.9947652	11.649237	20.966084
STIM1	NC_000011	3876933	4114440	6.8458241	4.9122123	5.6835738	7.2504665
LOC728325	NC_000011	4009584	4010833	0	0	0	0
RPS29P20	NC_000011	4040178	4040348	0	0	0	0
RRM1	NC_000011	4115924	4160106	26.098997	43.087776	46.461288	62.862097
OR55B1P	NC_000011	4167446	4168625	0	0	0	0
LOC643244	NC_000011	4231577	4233321	0	0	0	0
LOC196120	NC_000011	4254508	4255102	0	0	0	0
LOC390031	NC_000011	4263286	4263880	0	0	0	0
LOC643263	NC_000011	4299927	4300522	0	0	0	0
LOC441584	NC_000011	4308729	4309323	0	0	0	0
LOC143506	NC_000011	4351094	4351679	0	0	0	0
LOC390033	NC_000011	4359890	4360474	0	0	0	0
OR52B4	NC_000011	4388581	4389525	0	0	0.0413731	0.0324073
OR52B3P	NC_000011	4399512	4400454	0	0	0	0
TRIM21	NC_000011	4406127	4414926	10.844665	6.2774244	11.167838	5.3978531
OR51R1P	NC_000011	4439290	4440238	0	0	0	0
OR52P2P	NC_000011	4452463	4453559	0	0	0	0
OR52K2	NC_000011	4470570	4471514	0	0	0	0
OR52K3P	NC_000011	4495939	4497085	0	0	0	0
OR52K1	NC_000011	4510131	4511075	0	0	0	0.0324073
OR52M2P	NC_000011	4536175	4537154	0	0	0	0

OR52M1	NC_000011	4566421	4567374	0	0	0	0.0321015
C11orf40	NC_000011	4592653	4599050	0	0	0	0.046827
OR52I2	NC_000011	4608043	4609095	0.0417362	0	0	0.0290835
OR52I1	NC_000011	4615269	4616243	0	0	0	0
TRIM68	NC_000011	4619902	4629437	1.3051293	2.286554	1.966726	3.3780169
OR51D1	NC_000011	4661021	4661995	0.0450751	0.046342	0	0
OR51E1	NC_000011	4665156	4676718	0	0.0145471	0.0377633	0.0492995
OR51A9P	NC_000011	4681962	4683060	0	0	0	0
OR51E2	NC_000011	4701401	4719076	0.0315607	0.0162238	0.0701931	0.0329891
OR51C1P	NC_000011	4711891	4713032	0	0	0	0
OR51F5P	NC_000011	4730799	4731740	0	0	0	0
OR51C4P	NC_000011	4739842	4740789	0	0	0	0
LOC643447	NC_000011	4743965	4753243	0	0	0	0
OR51F3P	NC_000011	4757308	4758408	0	0	0	0
OR51F4P	NC_000011	4773176	4774274	0	0	0	0
OR51F1	NC_000011	4790209	4791147	0	0	0	0
OR51N1P	NC_000011	4807984	4808932	0	0	0	0
OR52Y1P	NC_000011	4814899	4815592	0	0	0	0
OR52R1	NC_000011	4824663	4825847	0.0370871	0.0381295	0	0
OR51F2	NC_000011	4842616	4843644	0	0	0.0379957	0.0297618
OR51A8P	NC_000011	4853363	4854299	0	0	0	0
OR51S1	NC_000011	4869467	4870438	0.0452142	0	0	0.0315071
OR51H1P	NC_000011	4880750	4881894	0	0	0	0
OR51H2P	NC_000011	4897787	4898733	0	0	0	0
OR51T1	NC_000011	4903049	4904113	0	0	0	0
OR51A6P	NC_000011	4910708	4911926	0	0	0	0
OR51A7	NC_000011	4928600	4929538	0	0	0.0416375	0
OR51G2	NC_000011	4935949	4936893	0	0	0.0413731	0.0648146
OR51G1	NC_000011	4944604	4945569	0	0	0.0404737	0
OR51A3P	NC_000011	4958568	4959524	0	0	0	0
OR51A4	NC_000011	4967389	4968330	0	0	0.0415048	0
OR51A2	NC_000011	4976002	4976943	0	0	0	0.0325105
OR51A5P	NC_000011	4994161	4995126	0	0	0	0
MMP26	NC_000011	5009424	5013659	0.0443474	0.0911875	0.0394526	0
OR51L1	NC_000011	5020213	5021160	0	0	0.0412422	0
OR51P1P	NC_000011	5036260	5037411	0	0	0	0
OR52J2P	NC_000011	5058146	5059304	0	0	0	0
OR52J3	NC_000011	5067756	5068691	0.0469532	0.0482729	0.0417709	0.0654378
OR52E2	NC_000011	5079880	5080857	0	0	0	0
OR52E1	NC_000011	5090702	5091826	0	0	0	0
OR52S1P	NC_000011	5097342	5098296	0	0	0	0
OR52E3P	NC_000011	5113906	5114880	0	0	0	0
OR52J1P	NC_000011	5125454	5126323	0	0	0	0
OR52A4	NC_000011	5141894	5142808	0	0	0	0
OR52A5	NC_000011	5152922	5153872	0	0	0.0411121	0
OR52A1	NC_000011	5172661	5173599	0	0	0	0.0326144
OR51A1P	NC_000011	5191287	5192553	0	0	0	0
OR52Z1	NC_000011	5198850	5199940	0	0	0	0
OR51V1	NC_000011	5220965	5221930	0	0.0467737	0	0
HBB	NC_000011	5246696	5248301	0	0	0.1873685	0
HBD	NC_000011	5254059	5255858	0	0.116753	0	0
HBBP1	NC_000011	5263184	5264822	0	0	0	0
HBG1	NC_000011	5269502	5271087	0	0	0	0
HBG2	NC_000011	5274421	5276011	0.0753829	0	0.0670627	0
LOC100288908	NC_000011	5275316	5276394	0	0	0	0.0317685
HBE1	NC_000011	5289580	5291373	0	0.0553718	0	0
OR51AB1P	NC_000011	5312697	5313581	0	0	0	0
OR51B4	NC_000011	5322244	5323176	0	0.0484281	0.0419052	0
OR51B3P	NC_000011	5335975	5336923	0	0	0	0
OR51B2	NC_000011	5344528	5345582	0.0416571	0	0.0370593	0.0290283
OR51B8P	NC_000011	5351818	5352575	0	0	0	0
OR51B5	NC_000011	5363816	5364754	0.0468032	0	0	0
OR51B6	NC_000011	5372738	5373676	0	0	0	0
LOC643745	NC_000011	5375687	5376787	0	0	0	0
LOC100288935	NC_000011	5383845	5384079	0	0	0	0
OR51M1	NC_000011	5410629	5411609	0	0	0	0

OR51J1	NC_000011	5423727	5424901	0	0	0	0
OR51Q1	NC_000011	5443431	5444384	0	0	0.1229483	0.0642031
OR51K1P	NC_000011	5451886	5452832	0	0	0	0
OR51I1	NC_000011	5461800	5462744	0.0930121	0	0	0.0324073
OR51I2	NC_000011	5474719	5475657	0	0.0481186	0	0
OR51A10P	NC_000011	5489734	5490589	0	0	0	0
OR52D1	NC_000011	5509937	5510893	0	0	0.0408543	0.0320009
UBQLN3	NC_000011	5528530	5531153	0.0374825	0	0.0333455	0.0130596
UBQLNL	NC_000011	5535623	5537956	0.0564887	0.0193588	0.1340105	0.0393636
LOC643810	NC_000011	5539653	5544336	0	0	0	0
OR52V1P	NC_000011	5548453	5549356	0	0	0	0
OR52H1	NC_000011	5565791	5566753	0	0	0	0
OR52H2P	NC_000011	5572895	5573826	0	0	0	0
OR52B5P	NC_000011	5582197	5583141	0	0	0	0
OR52T1P	NC_000011	5587781	5588946	0	0	0	0
LOC728732	NC_000011	5592477	5593422	0	0	0	0
OR52B6	NC_000011	5602107	5603114	0	0	0.0387873	0.0303818
TRIM6	NC_000011	5617347	5634188	0.2783135	0.2080986	0.1463064	0.1674936
TRIM6-TRIM34	NC_000011	5617884	5665628	0.3935284	0.6525621	0.587254	0.8315247
TRIM34	NC_000011	5641174	5665628	3.1568948	0.6564175	2.4929037	0.6550115
TRIMP1	NC_000011	5664412	5687621	0	0	0	0
TRIM5	NC_000011	5684788	5706293	4.9086794	2.7944196	4.6676478	4.3251751
TRIM22	NC_000011	5710919	5732093	19.143726	5.1396309	35.291145	5.6044859
OR52U1P	NC_000011	5740520	5741496	0	0	0	0
OR52P1P	NC_000011	5747632	5748791	0	0	0	0
OR56B1	NC_000011	5757747	5758721	0	0	0.0401001	0
OR52N4	NC_000011	5775923	5776959	0	0	0.1508103	0
OR56B2P	NC_000011	5786292	5787376	0	0	0	0
OR52N5	NC_000011	5798890	5799864	0	0	0	0
OR52N1	NC_000011	5809084	5810046	0	0	0.1217993	0
OR52N3P	NC_000011	5821594	5822551	0	0	0	0
OR52N2	NC_000011	5841566	5842531	0	0	0	0
OR52E6	NC_000011	5862186	5863127	0	0	0	0
OR52E8	NC_000011	5877979	5878932	0	0	0	0.0963046
OR52E7P	NC_000011	5895190	5896118	0	0	0	0
OR52E4	NC_000011	5905523	5906461	0	0.0481186	0	0.0652287
OR52E5	NC_000011	5922007	5923155	0	0	0	0
OR52Q1P	NC_000011	5924765	5925808	0	0	0	0
OR56A3	NC_000011	5968577	5969524	0	0.0953236	0	0
OR56A5	NC_000011	5988783	5989724	0	0	0	0.0325105
OR52L1	NC_000011	6007122	6008215	0	0	0	0
LOC644030	NC_000011	6015104	6020082	0	0	0	0
OR56A4	NC_000011	6023281	6024378	0	0	0	0
OR56A1	NC_000011	6047978	6048934	0	0	0	0
OR56A7P	NC_000011	6066940	6068077	0	0	0	0
OR52L2P	NC_000011	6078449	6079560	0	0	0	0
OR52X1P	NC_000011	6087943	6089122	0	0	0	0
OR56B4	NC_000011	6129009	6129968	0.0457794	0.047066	0	0.0319009
OR56B3P	NC_000011	6149840	6150783	0	0	0	0
OR52B1P	NC_000011	6172825	6173779	0	0	0	0
OR52B2	NC_000011	6190585	6191556	0	0	0.0402238	0
OR52W1	NC_000011	6220454	6221416	0	0.1876777	0.0405998	0.0636031
C11orf42	NC_000011	6226798	6232362	0.0385511	0.1189037	0.1714806	0.1880475
FAM160A2	NC_000011	6232564	6255941	3.3980592	2.8983626	4.7696343	6.2003978
CNGA4	NC_000011	6260330	6265707	0.0230942	0.0474865	0.0410905	0
CCKBR	NC_000011	6280966	6293356	0.0414606	0.0213129	0	0.0144457
LOC100130103	NC_000011	6318462	6320025	0	0	0	0
LOC100288128	NC_000011	6337084	6341734	3.232034	2.1006653	4.1642378	5.4622564
PRKCDBP	NC_000011	6340176	6341740	42.803967	38.211999	21.754383	23.035106
LOC100289043	NC_000011	6411613	6412766	0	0.13173	0	0.0446427
SMPD1	NC_000011	6411655	6416226	19.907722	16.597984	23.733896	13.002399
APBB1	NC_000011	6416355	6440644	6.6951751	7.466678	3.2737541	3.3776609
HPX	NC_000011	6452441	6462193	0.0316402	0	0.028148	0
TRIM3	NC_000011	6469843	6495205	2.2719369	2.499444	1.6349657	2.3193026
ARFIP2	NC_000011	6496910	6502595	12.950846	14.724637	17.154132	23.122019
FXC1	NC_000011	6502677	6505911	6.0772889	5.5326615	6.3970331	8.2650534

LOC644169	NC_000011	6509411	6510607	0	0	0	0
DNHD1	NC_000011	6518526	6593255	0.2242114	0.1555962	0.5560077	0.4257524
RRP8	NC_000011	6621152	6624811	1.3914315	1.9973547	3.6668565	4.9577909
ILK	NC_000011	6624964	6632101	26.869448	29.949238	25.954378	24.949639
TAF10	NC_000011	6632073	6633445	20.820619	35.162411	30.323702	24.596359
TPP1	NC_000011	6633997	6640692	14.952102	5.3074191	28.00904	19.252152
DCHS1	NC_000011	6642558	6677080	1.5853397	4.0075275	0.0690641	0.048403
MRPL17	NC_000011	6701616	6704632	9.9933004	12.546701	14.987951	10.898625
RPL21P94	NC_000011	6707077	6707642	0	0	0	0
GVIN1	NC_000011	6734370	6743110	0	0	0	0
LOC100128135	NC_000011	6770182	6771605	0	0	0	0
OR2AG2	NC_000011	6789238	6790188	0	0	0	0
OR2AG1	NC_000011	6806269	6807219	0.0924253	0	0	0.1288113
OR6A2	NC_000011	6815756	6817139	0	0.0652939	0	0.110639
LOC100131597	NC_000011	6864688	6865897	0	0	0	0
OR10A5	NC_000011	6866914	6867867	0	0.0473621	0.0409828	0.1284062
OR10A2	NC_000011	6890986	6891897	0	0	0	0
OR10A4	NC_000011	6897879	6898826	0.0463589	0	0.0412422	0
OR2D2	NC_000011	6912805	6913731	0	0	0.0421764	0.0991096
OR2D3	NC_000011	6942233	6943225	0.044258	0	0	0.0308408
LOC100288223	NC_000011	6945781	6948086	0	0.216707	0.1875183	0.073441
ZNF215	NC_000011	6947654	6979278	0.3964712	0.6175971	0.4916588	0.6530182
ZNF214	NC_000011	7020549	7041586	0.1647235	0.3895121	0.6447874	0.9871587
LOC100289103	NC_000011	7041677	7060083	0	0	0.0637807	0
NLRP14	NC_000011	7041700	7092757	0.0689745	0.0354565	0.0306808	0.0160214
RBMXL2	NC_000011	7110165	7112379	0.7738063	0.5507683	0.1765127	0.0138261
SYT9	NC_000011	7273181	7490273	0.0222241	0.0571219	0.4151954	0.1780966
OLFML1	NC_000011	7506755	7532564	5.9604586	0.0834874	0.1011393	0.0452696
PPFIBP2	NC_000011	7535001	7674991	0.6294187	0.190326	0.7136596	0.2322021
CYB5R2	NC_000011	7686326	7694821	4.7925742	6.234505	1.5952271	0.8633126
OVCH2	NC_000011	7712444	7727941	32.332416	51.740993	21.0135	50.029668
OR10AB1P	NC_000011	7749847	7750984	0	0	0	0
OR5P4P	NC_000011	7767437	7768556	0	0	0	0
OR5P1P	NC_000011	7794440	7795361	0	0	0	0
RPL7AP55	NC_000011	7805221	7806013	0	0	0	0
OR5P2	NC_000011	7817521	7818489	0	0	0	0
OR5P3	NC_000011	7846584	7847519	0	0	0	0
OR5E1P	NC_000011	7870147	7871279	0	0	0	0
OR10A6	NC_000011	7949265	7950209	0	0	0	0
OR10A3	NC_000011	7960123	7961067	0	0	0	0.0324073
NLRP10	NC_000011	7981156	7985059	1.5229585	0.2013122	0.1548418	0.3486991
EIF3F	NC_000011	8008867	8017718	37.999825	44.212103	33.463283	109.52783
TUB	NC_000011	8060180	8127654	1.4112428	0.9513016	0.4560001	0.0649422
RIC3	NC_000011	8127597	8190590	0.0151311	0.0388909	0.0471136	0.0105439
LMO1	NC_000011	8245857	8285406	0	0	0	0.0240384
STK33	NC_000011	8413418	8615503	0.0487051	0.0834566	0.2455333	0.0339397
TRIM66	NC_000011	8633584	8680383	0.1667266	0.1575142	0.4209212	0.9200338
RPL27A	NC_000011	8703995	8711419	7.0349598	13.520756	22.806245	23.78936
SNORA3	NC_000011	8705774	8705903	0	0	0	0
SNORA45	NC_000011	8706986	8707116	0	0	0	0
ST5	NC_000011	8714899	8932498	1.1769346	1.2580288	1.8447736	3.1308324
C11orf17	NC_000011	8932701	8941626	1.0862288	2.142967	2.6639624	3.3754876
C11orf16	NC_000011	8941623	8954553	0.091321	0	0	0.0636361
ASCL3	NC_000011	8959119	8964580	0.1352253	0	0.1203002	0.0471152
TMEM9B	NC_000011	8968840	8985989	2.2312565	2.4743905	3.3008783	2.7427872
NRIP3	NC_000011	9002123	9025596	1.3960977	1.3522992	2.5456015	3.6984626
LOC100288260	NC_000011	9025715	9091046	0.1695534	0.0697275	0.0301679	0.0472606
SCUBE2	NC_000011	9041935	9113155	0.1058427	0.0846358	0.0209246	0.1065356
FLJ46111	NC_000011	9115937	9117745	0	0	0	0
DENND5A	NC_000011	9160372	9286871	26.085688	21.941024	21.434758	22.93936
TMEM41B	NC_000011	9302201	9336296	4.1649401	6.2283582	8.9022149	5.8422845
IPO7	NC_000011	9406169	9469674	4.4785747	6.0711548	7.1161105	12.315237
SNORA23	NC_000011	9450313	9450501	0	0	0	0
LOC100288296	NC_000011	9481103	9482278	0.1868547	0.2689488	0.5651859	0.5729144
ZNF143	NC_000011	9482513	9550071	0.1491793	0.2300581	0.2256139	0.8316328
WEE1	NC_000011	9595228	9611314	5.8843729	5.435849	10.209023	9.562709

RPL23AP65	NC_000011	9627681	9628229	0	0	0	0
RPL21P97	NC_000011	9681942	9682503	0	0	0	0
SWAP70	NC_000011	9685628	9774508	4.0612227	3.7652835	3.7339466	5.5526543
LOC283104	NC_000011	9779833	9781601	0.2484355	0.2809595	0.2431166	0.8309747
SBF2	NC_000011	9800214	10315754	5.2638627	4.6039815	8.3566513	8.8675881
RNU7-28P	NC_000011	9865364	9865422	0	0	0	0
GLULL2	NC_000011	9978132	9981461	0	0	0	0
LOC653503	NC_000011	10292762	10293835	0	0	0	0
ADM	NC_000011	10326642	10328923	61.600317	42.813534	71.314607	90.923552
AMPD3	NC_000011	10472224	10529126	1.2580925	1.5468979	1.3763553	2.0140152
RNF141	NC_000011	10533225	10562774	7.0592654	5.3879326	8.2139352	7.8361895
LOC100129827	NC_000011	10562336	10621479	0	0.1263871	0.2187276	0.385488
LYVE1	NC_000011	10579413	10590365	0	0	0	0.01225
MRVI1	NC_000011	10594638	10715535	1.193672	0.4135936	0.1760094	0.2481608
CTR9	NC_000011	10772811	10801290	0.4385644	1.0380963	0.7258773	2.7362678
EIF4G2	NC_000011	10818593	10830582	119.16927	187.56137	141.92486	220.49756
SNORD97	NC_000011	10823014	10823155	0	0	0	0
ZBED5	NC_000011	10874251	10879620	2.5653838	2.1429559	3.8227463	3.7093979
LOC100288365	NC_000011	11157029	11158908	0	0	0	0
GALNTL4	NC_000011	11292421	11643561	0.7863501	1.077934	0.7306503	0.0730613
CSNK2A1P	NC_000011	11373321	11374904	0	0	0	0
USP47	NC_000011	11862970	11980872	0.5368499	0.6042271	0.9350839	2.5714344
DKK3	NC_000011	11984543	12030917	32.703952	31.386597	94.527191	111.14065
MICAL2	NC_000011	12132138	12285332	4.7368678	27.588944	7.1668861	19.969679
MICALCL	NC_000011	12308447	12380691	0.0721883	0.0890606	0.0513766	0.0905466
PARVA	NC_000011	12399146	12551410	22.554244	23.449982	18.138954	29.38253
TEAD1	NC_000011	12695969	12966299	7.9079789	9.0021856	14.107625	14.524979
RASSF10	NC_000011	13030696	13032647	0	0.0244499	0	0.0331438
ARNTL	NC_000011	13299325	13408813	2.1154015	2.8525273	2.9592507	3.8668315
BTBD10	NC_000011	13409555	13484838	1.2266253	2.179901	2.2604254	3.162617
PTH	NC_000011	13513601	13517567	0	0	0	0.0376227
LOC644992	NC_000011	13631942	13632253	0	0.1448186	0	0
FAR1	NC_000011	13690206	13753893	4.7572399	7.1179918	9.7169495	11.988696
RPL39P26	NC_000011	13707046	13707202	0	0	0	0
LOC100130384	NC_000011	13777494	13779147	0	0	0	0
SPON1	NC_000011	13983914	14289656	0.0244882	0.0587451	0.0944035	0.7280803
RRAS2	NC_000011	14299466	14380729	14.051824	10.584923	25.487846	36.966393
LOC401676	NC_000011	14402953	14463225	0	0	0	0
COPB1	NC_000011	14479049	14521441	32.74623	32.83387	41.458717	38.509026
RNU7-49P	NC_000011	14500341	14500597	0	0	0	0
PSMA1	NC_000011	14526422	14665180	35.316688	39.428394	55.595712	62.619464
PDE3B	NC_000011	14665269	14893605	0.0216458	0.0296722	0.2952697	0.2111714
MORF4LP3	NC_000011	14695128	14695837	0	0	0	0
CYP2R1	NC_000011	14899555	14913751	0.2171624	0.1116329	1.400654	1.1349553
CALCP	NC_000011	14928946	14929646	0	0	0	0
CALCA	NC_000011	14988216	14993832	0	0	0	0.086756
OR7E41P	NC_000011	15009332	15009974	0	0	0	0
LOC440031	NC_000011	15092872	15096190	0	0	0	0
CALCB	NC_000011	15095146	15100177	0.0426268	0	0.037922	0
INSC	NC_000011	15133970	15268754	0.0582096	0.0598456	0.0906235	0.0304221
SOX6	NC_000011	15991795	16497918	0.3540228	0.0423224	0.0732438	0.0458972
AKR1B1P3	NC_000011	16505059	16506256	0	0	0	0
C11orf58	NC_000011	16760148	16779901	5.8720201	6.937609	7.340414	9.5476675
LOC100289201	NC_000011	16800698	16808009	0.0218648	0.0449586	0	0
PLEKHA7	NC_000011	16809210	17035959	0.0183079	0.4046837	0.1791599	0.1275771
RPL36AP37	NC_000011	16996176	16996591	0	0	0	0
OR7E14P	NC_000011	17073802	17074453	0	0	0	0
RPS13	NC_000011	17095939	17099220	100.52431	191.58104	239.24163	216.80561
SNORD14A	NC_000011	17096200	17096291	0	0	0	0
SNORD14B	NC_000011	17097325	17097415	0	0	0	0
PIK3C2A	NC_000011	17108122	17191354	2.539349	3.8860996	4.3577986	6.2136363
RPL34P24	NC_000011	17159523	17159812	0	0	0	0
RPL29P21	NC_000011	17214991	17215974	0	0	0	0
RPS2P40	NC_000011	17249798	17250745	0	0	0	0
NUCB2	NC_000011	17298286	17353070	20.20201	15.836614	52.679846	28.64908
DKFZp686O24166	NC_000011	17373317	17398888	0	0	0	0

KCNJ11	NC_000011	17406795	17410206	0.025761	0.026485	0.0114588	0.0089756
ABCC8	NC_000011	17414432	17498449	0.0441425	0.0453831	0.0471244	0.0246082
USH1C	NC_000011	17515442	17565963	0.0397123	0.0544378	0.0353291	0.0184487
OTOG	NC_000011	17568920	17667491	0.0300125	0.0205706	0.0400499	0.0906268
MYOD1	NC_000011	17741110	17743678	0	0	0	0
KCNC1	NC_000011	17757495	17804602	0.0787979	0.06481	0.0280403	0.0988372
SERGEF	NC_000011	17809595	18034637	0.7942452	0.6048648	1.6486925	1.6808835
TPH1	NC_000011	18042538	18062309	0.06584	0.0676905	0	0.02294
SAAL1	NC_000011	18101890	18127638	0.7176393	0.4721975	0.56182	0.8001275
LOC100289300	NC_000011	18122973	18128393	0	0	0	0
SAA3P	NC_000011	18134019	18137679	0	0	0	0
MRGPRX3	NC_000011	18142502	18160027	0	0	0	0
LOC645297	NC_000011	18162247	18175879	0	0	0	0
MRGPRX4	NC_000011	18194384	18195827	0	0	0.0270759	0
GLTPP1	NC_000011	18210451	18212785	0	0	0	0
LOC645319	NC_000011	18218219	18218895	0	0	0	0
LOC494141	NC_000011	18230685	18235125	0	0	0	0
SAA4	NC_000011	18252902	18258355	0	0.0710431	0.0614742	0
SAA2	NC_000011	18260588	18270182	0.0298561	0.0613905	0.0265608	0.1872445
FAM10A5	NC_000011	18283432	18285048	0	0	0	0
SAA1	NC_000011	18287772	18291526	0	0	0.5460554	0
HPS5	NC_000011	18300217	18343721	3.2330769	3.7313342	2.2272794	2.6671257
GTF2H1	NC_000011	18343816	18388590	3.0757511	3.3164488	3.7373491	6.1946767
LDHA	NC_000011	18415936	18429765	334.20958	493.70233	422.6716	845.55455
LDHC	NC_000011	18433853	18472793	0.0342276	0.246327	0.0608996	0.0477023
LOC100288566	NC_000011	18471633	18477605	0	0	0	0
LDHAL6A	NC_000011	18477374	18501147	0.2211874	0.2099113	0.1362285	0.1897011
TSG101	NC_000011	18501858	18548489	15.527993	16.141137	25.997078	23.198245
UEVLD	NC_000011	18553244	18610281	4.0235757	4.8403172	6.1626177	6.6915142
LOC100129966	NC_000011	18616663	18617922	0	0	0	0
SPTY2D1	NC_000011	18627948	18656020	0.9044108	1.481423	3.1638072	2.6704638
LOC100132715	NC_000011	18686597	18687098	0	0	0	0
TMEM86A	NC_000011	18720351	18726332	0.0613459	0.1135261	0.010915	0.025649
IGSF22	NC_000011	18725852	18747777	0.078041	0.0343862	0.0198364	0.0233066
PTPN5	NC_000011	18749475	18813389	0	0	0	0.0195374
LOC441592	NC_000011	18885096	18886056	0	0	0	0
LOC645397	NC_000011	18892531	18895090	0	0	0	0
LOC390098	NC_000011	18908474	18909467	0	0	0	0
LOC390099	NC_000011	18931978	18932943	0	0	0	0
MRGPRX1	NC_000011	18955360	18956549	0.0738626	0	0	0
LOC645415	NC_000011	18971994	18979430	0	0	0	0
LOC390101	NC_000011	18993283	18998617	0	0	0	0
MRGPRX2	NC_000011	19076002	19082228	0.0215856	0	0.0192031	0.0300834
ZDHHC13	NC_000011	19138692	19197967	5.2542874	7.7409526	7.4532746	8.5180947
CSRP3	NC_000011	19203578	19223589	0.0655455	0	0	0
E2F8	NC_000011	19245614	19262507	1.5252413	2.4190199	3.8708378	5.0176354
LOC390102	NC_000011	19295719	19296815	0	0	0	0
NAV2	NC_000011	19372271	20143147	2.768545	3.1051148	10.609736	27.748568
LOC100126784	NC_000011	19732480	19736146	0	0	0	0
LOC100289464	NC_000011	20135929	20140149	0	0	0.044178	0
LOC100289498	NC_000011	20153276	20155074	0	0.0251158	0.0434659	0.0680931
DBX1	NC_000011	20177760	20181870	0	0	0.0340275	0.053307
HTATIP2	NC_000011	20385231	20405329	2.7147968	1.7596043	6.8254479	6.8816871
PRMT3	NC_000011	20409076	20530877	1.5126817	2.2169813	4.4666571	5.0013532
LOC645490	NC_000011	20557855	20618167	0.0601619	0.0309264	0.0267608	0.1048079
SLC6A5	NC_000011	20620946	20676610	0.0614017	0.1104729	0.0273123	0.0106968
NELL1	NC_000011	20691117	21597232	0.0270451	0.0139026	0.02406	0.0282691
ANO5	NC_000011	22214722	22304913	0.0395871	0.0406997	0.0293481	0.0781599
SLC17A6	NC_000011	22359667	22401049	0.011197	0	0.0199223	0.0234076
FANCF	NC_000011	22644079	22647387	1.1422025	1.1606495	1.1461057	0.9717776
GAS2	NC_000011	22688160	22834547	0.0173161	0.0534083	0.0154049	0.0603327
SVIP	NC_000011	22843598	22851382	2.0566127	3.4481214	2.6459116	3.0205963
LOC100131557	NC_000011	23391686	23424594	0	0	0	0
LOC645598	NC_000011	23425088	23427654	0	0	0	0
LOC100129382	NC_000011	23499614	23501979	0	0	0	0
RPS2P38	NC_000011	23541841	23542785	0	0	0	0

LOC100288844	NC_000011	23810802	23815418	0	0	0	0
LUZP2	NC_000011	24518556	25104186	0.0250798	0.0601643	0.0148745	0.0233022
LOC100130747	NC_000011	25161810	25162747	0	0	0	0
RPL36AP40	NC_000011	25609812	25610513	0	0	0	0
LOC100289562	NC_000011	25652266	25652592	0	0	0	0
LOC645705	NC_000011	26067815	26069232	0	0	0	0
ANO3	NC_000011	26353678	26684836	0	0.0456706	0.0065865	0.0154775
MUC15	NC_000011	26580579	26593815	0	0	0	0.0180571
SLC5A12	NC_000011	26688566	26743574	0.021085	0.0289035	0.0187578	0.0195905
FIBIN	NC_000011	27015628	27018634	12.218398	17.670662	6.1890393	5.1737407
LOC100288909	NC_000011	27015673	27016664	0	0.1647026	0.0475062	0.0744225
BBOX1	NC_000011	27062509	27149354	0	0.0479145	0.0414608	0
CCDC34	NC_000011	27360061	27384795	0.1791977	0.4452323	0.4384029	0.9781646
LGR4	NC_000011	27387508	27494334	3.8696556	13.485269	5.8493594	6.7296521
RPL37AP7	NC_000011	27505366	27505738	0	0	0	0
LIN7C	NC_000011	27515965	27528326	2.4908829	3.1010043	3.3682569	4.3046511
BDNFOS	NC_000011	27528375	27699352	0	0	0	0
RPS25P1	NC_000011	27603166	27603647	0	0	0	0
BDNF	NC_000011	27676442	27743605	1.9847222	1.1284157	1.2900982	2.9642092
CBX3P1	NC_000011	27827864	27830062	0	0	0	0
HSP90AA2	NC_000011	27909718	27912639	0	0	0	0
KIF18A	NC_000011	28042163	28129746	1.1040994	1.4352221	1.3661003	2.8210616
METT5D1	NC_000011	28129798	28355054	1.9406488	1.9031052	3.913298	4.0222893
RPS15AP31	NC_000011	28308743	28309190	0	0	0	0
OR2BH1P	NC_000011	29008182	29009045	0	0	0	0
LOC401677	NC_000011	29297501	29298222	0	0	0	0
RPL7AP58	NC_000011	29747285	29748168	0	0	0	0
KCNA4	NC_000011	30031765	30038488	0.2554423	0.2626215	0.0757495	0.728962
FSHB	NC_000011	30252563	30256824	0	0	0	0.0474559
C11orf46	NC_000011	30344649	30359165	1.6884066	1.7103322	2.5402373	2.3357958
RPL12P30	NC_000011	30389607	30390235	0	0	0	0
MPPED2	NC_000011	30406040	30607930	0.0060963	0.0313382	0.0216938	0.0084963
DCDC5	NC_000011	30886486	30953451	0.0251348	0.0646031	0.0111803	0.0525448
LOC100288976	NC_000011	31027307	31263299	0.0295153	0.0303448	0	0.1234045
DCDC1	NC_000011	31284171	31391321	0.025761	0	0.2520945	0.0718051
CYCSP25	NC_000011	31302215	31302534	0	0	0	0
DNAJC24	NC_000011	31391377	31454382	0.5273788	0.5271397	0.899244	0.9085381
IMMP1L	NC_000011	31453949	31531169	0.3316848	0.2841723	1.3770212	1.2712214
ELP4	NC_000011	31531297	31805329	4.0666839	3.6331261	4.4661546	5.3158691
PAX6	NC_000011	31806340	31839509	0.0369365	0.1139237	0.071196	0.1887512
RCN1	NC_000011	32112477	32127272	57.782492	77.275111	115.8009	101.58793
LOC100289009	NC_000011	32112581	32122062	0.4822851	0.4338598	0.2145271	0.6301415
WT1	NC_000011	32409325	32457087	0.0145092	0.0298339	0.051631	0.0202211
LOC100289633	NC_000011	32450057	32452359	0	0	0	0
WIT1	NC_000011	32457285	32461636	0	0	0	0
EIF3M	NC_000011	32605391	32624019	30.431143	39.471812	52.696719	48.008565
CCDC73	NC_000011	32623626	32816187	0.3940564	0.4839069	0.3700392	0.3737532
RPL34P2	NC_000011	32779714	32780266	0	0	0	0
PRRG4	NC_000011	32851489	32876124	0.9077469	0.5235356	5.2195743	4.0267472
QSER1	NC_000011	32914792	33001816	1.8866785	3.0606382	7.1903084	12.308089
DEPDC7	NC_000011	33037410	33055128	1.4859754	1.2556758	1.1770921	1.3759209
TCP11L1	NC_000011	33060963	33095109	1.7526713	3.0032171	3.9110559	4.5799918
LOC100289666	NC_000011	33095971	33101000	0	0	0	0
PIGCP1	NC_000011	33096625	33098223	0	0	0	0
LOC283267	NC_000011	33097696	33101000	0	0	0	0
CSTF3	NC_000011	33106130	33183037	0.2486854	0.5255536	0.4178929	0.7605676
RPL29P23	NC_000011	33211461	33212100	0	0	0	0
RPS24P15	NC_000011	33258516	33259024	0	0	0	0
HIPK3	NC_000011	33279168	33375939	6.7919152	9.4120179	12.216444	11.054141
C11orf41	NC_000011	33563877	33695648	0.8910466	2.4687838	0.2586351	0.1789082
C11orf91	NC_000011	33719975	33722286	0.2020608	1.3503086	0	0.4224121
CD59	NC_000011	33724556	33758025	33.216813	37.337819	31.955486	77.627244
FBXO3	NC_000011	33762490	33796071	2.1384471	3.5079779	4.8259965	4.7882189
LOC646120	NC_000011	33808372	33809367	0	0	0	0
LMO2	NC_000011	33880122	33913836	0.6364039	0.7290661	0.1132325	0.0760237
LOC100129531	NC_000011	34072262	34074667	0	0	0.0523394	0

CAPRIN1	NC_000011	34073230	34124157	8.7114991	11.30079	14.508439	23.514336
NAT10	NC_000011	34127111	34168458	3.6868227	6.0266898	4.5280031	7.4421258
ABTB2	NC_000011	34172535	34378802	1.3770234	1.5899679	1.2344616	0.8340832
LOC100216355	NC_000011	34356512	34357655	0	0	0	0
CAT	NC_000011	34460478	34493607	8.391162	8.6466929	22.412074	23.269034
ELF5	NC_000011	34500342	34535330	0.0171807	0.0353271	0.0305689	0.0239444
EHF	NC_000011	34642668	34683081	0.0486019	0.012492	1.7295025	0.1862724
NDUFB8P3	NC_000011	34695870	34731711	0	0	0	0
APIP	NC_000011	34903842	34937939	4.6426647	7.0341118	15.744611	17.197608
PDHX	NC_000011	34937677	35017675	6.1480458	6.1242563	11.462339	9.0808709
CD44	NC_000011	35160417	35253949	99.872587	139.72802	106.22915	132.6989
SLC1A2	NC_000011	35272752	35441105	0.0329447	0.1053753	0.0227955	0.0357112
PAMR1	NC_000011	35453375	35547176	6.8146576	21.634838	1.8805003	1.549931
FJX1	NC_000011	35639735	35642421	8.6359011	7.0120873	4.9617677	6.2229935
TRIM44	NC_000011	35684353	35830930	4.2661299	4.6256469	6.5988974	9.1796961
KRT18P14	NC_000011	35881735	35883140	0	0	0	0
LDLRAD3	NC_000011	35965612	36252841	2.7236358	2.9371494	5.1094158	8.066236
RPL12P31	NC_000011	36018698	36019316	0	0	0	0
COMMD9	NC_000011	36293842	36310999	10.521567	10.391879	9.9124292	23.777015
FLJ14213	NC_000011	36397555	36486754	0.7939757	1.0577285	0.4775275	0.6311998
TRAF6	NC_000011	36510723	36531822	0.5292911	0.5441668	1.1999641	0.6900711
RAG1	NC_000011	36589563	36601312	0.0333852	0.0274588	0.0297004	0.0372226
RAG2	NC_000011	36613493	36619812	0	0.0748689	0	0.1141773
C11orf74	NC_000011	36616093	36680817	4.3948232	3.030594	7.1996737	10.345233
LOC100129825	NC_000011	37369832	37565530	0	0	0	0.0488435
RPL7AP56	NC_000011	37747195	37748040	0	0	0	0
RPL18P8	NC_000011	39182990	39183523	0	0	0	0
LOC100289139	NC_000011	39788569	39788763	0	0	0	0
LOC646388	NC_000011	40105260	40117687	0	0	0	0
LRRC4C	NC_000011	40135753	40315664	0.0542035	0.0222908	0.0192884	0.0075542
RPL9P23	NC_000011	41557675	41560536	0	0	0	0
LOC100128134	NC_000011	43085113	43087744	0	0	0	0
LOC399881	NC_000011	43283054	43330828	0	0	0	0
API5	NC_000011	43333505	43366080	8.5610409	11.607626	17.328274	20.985519
TTC17	NC_000011	43380491	43515502	4.6241402	4.6504706	9.7743914	7.6825598
LOC120449	NC_000011	43543526	43544817	0	0	0	0
LOC100131381	NC_000011	43591956	43606151	0	0.1517919	0	0
LOC100286987	NC_000011	43665773	43667921	0.2045055	0.0630759	0.2001271	0.4417735
HSD17B12	NC_000011	43702143	43878169	9.5149217	9.0366804	11.583326	8.1104786
RPL23AP63	NC_000011	43740220	43740733	0	0	0	0
ALKBH3	NC_000011	43902357	43941825	4.4256346	6.7962287	6.8775832	9.9155119
LOC729799	NC_000011	43918853	43921438	0	0	0	0
LOC387763	NC_000011	43964106	43965433	2.2834548	1.0207094	5.5054554	1.2914105
ACCSL	NC_000011	44069531	44081527	0.0482948	0.0496521	0	0
ACCS	NC_000011	44087729	44105569	1.9480599	0.4606464	1.3344471	2.6063726
EXT2	NC_000011	44117099	44266980	21.248802	28.87347	47.470453	27.551878
ALX4	NC_000011	44286158	44331716	0.0554202	0	0.0246517	0.0579285
LOC646535	NC_000011	44561329	44562243	0	0	0	0
CD82	NC_000011	44587141	44641339	11.429103	3.820171	27.812846	13.303519
RPL34P22	NC_000011	44650907	44651224	0	0	0	0
TSPAN18	NC_000011	44881052	44952887	0.0429882	0.0147321	0.0382435	0.0798823
TP53I11	NC_000011	44953899	44972608	20.395931	25.019318	25.657777	34.478173
LOC221122	NC_000011	44995453	44999579	0	0	0	0
PRDM11	NC_000011	45115564	45246903	0.4731977	0.4662262	0.1052424	0.096175
LOC100287017	NC_000011	45254409	45256675	0.0969304	0	0	0.013509
SYT13	NC_000011	45261853	45307884	0.0596544	0.0175231	0.0151629	0.0475081
FLJ41423	NC_000011	45393018	45393521	0	0	0	0
CHST1	NC_000011	45670427	45687172	0.1293546	0.1662377	0.0143847	0.0676046
DKFZp779M0652	NC_000011	45792983	45793909	0	0	0	0
SLC35C1	NC_000011	45825623	45834567	2.2119344	2.6186618	1.2721119	1.0664993
LOC100289343	NC_000011	45832435	45847808	1.0319336	0.4368561	0.7560303	1.2689867
CRY2	NC_000011	45868669	45904799	2.4851679	2.730865	3.3207868	2.4679389
LOC100289377	NC_000011	45905907	45922123	0	0	0.0377754	0.0295893
MAPK8IP1	NC_000011	45907202	45928016	2.4063458	3.3776445	2.9611599	6.2555079
LOC100289415	NC_000011	45923543	45924987	0.0694285	0.0356899	0.0926483	0.0483805
LOC143678	NC_000011	45928085	45928833	0.1191009	0.2448965	0.1059555	0.1659885

PEX16	NC_000011	45931220	45939674	3.777521	3.2443009	6.8236317	6.6450208
GYLTL1B	NC_000011	45943196	45950647	0	0.0357464	0.0309316	0.0121143
LOC100289449	NC_000011	45949451	45950648	0	0	0	0
PHF21A	NC_000011	45950870	46142985	1.4545656	1.1323544	3.1791401	3.816639
LOC401679	NC_000011	46156329	46165145	0	0	0	0
CREB3L1	NC_000011	46299228	46342972	16.88165	22.54941	21.839599	25.79782
DGKZ	NC_000011	46354739	46402104	5.4669044	4.9453562	5.9688529	8.0520177
MDK	NC_000011	46402618	46405375	45.19136	25.185424	62.09422	17.580005
CHRM4	NC_000011	46406640	46408107	0	0.0615578	0.0266332	0
AMBRA1	NC_000011	46417964	46612914	1.6623858	1.7990604	2.2339242	2.7619092
RPS10P19	NC_000011	46450143	46450702	0	0	0	0
HARBI1	NC_000011	46624856	46638777	1.3282646	1.6624642	1.823868	1.2475312
KIAA0652	NC_000011	46638826	46696366	9.8934711	11.932169	14.373861	18.127178
LOC100289510	NC_000011	46638826	46651644	0.0655944	0.0674379	0	0.0457088
ARHGAP1	NC_000011	46698630	46722120	24.062511	18.648619	16.008268	38.44127
ZNF408	NC_000011	46722368	46727461	2.5745994	2.75802	2.2423839	2.6973981
F2	NC_000011	46740743	46761056	0	0	0.0194515	0.060945
CKAP5	NC_000011	46765084	46867843	2.814839	5.1133202	6.508478	9.949199
SNORD67	NC_000011	46783939	46784049	0	0	0	0
LRP4	NC_000011	46878418	46940076	0.1707262	0.8493121	0.2939667	0.2532885
LOC100289541	NC_000011	46958035	47120939	0	0	0	0
C11orf49	NC_000011	46958251	47185932	2.6682164	2.8624768	5.7106871	6.009105
ARFGAP2	NC_000011	47185852	47198419	11.854248	11.794798	13.716706	22.253487
PACSIN3	NC_000011	47199085	47207958	0.2396305	1.0593709	0.7248186	0.2170793
RPS20P26	NC_000011	47212692	47213207	0	0	0	0
DDB2	NC_000011	47236493	47260769	11.581596	10.391204	17.835091	10.838025
ACP2	NC_000011	47260853	47270457	4.3339952	5.6870112	4.3798744	3.6559111
NR1H3	NC_000011	47270449	47290401	2.3651437	0.9924965	4.4228987	1.446315
MADD	NC_000011	47290927	47351582	2.2275695	2.8257243	3.1036589	4.9576768
MYBPC3	NC_000011	47352957	47374253	0.0416867	0.0214292	0.0185428	0.029049
SPI1	NC_000011	47376409	47400127	0	0.0320905	0	0.0217506
SLC39A13	NC_000011	47430051	47438047	12.278859	8.8478763	9.9786085	8.7696804
LOC100287086	NC_000011	47436755	47438055	0.0337803	0.0694595	0.0601039	0.1647764
PSMC3	NC_000011	47440320	47448024	2.9206103	4.461146	6.2358143	9.2843777
RAPSN	NC_000011	47459315	47470730	0.0528224	0.1900744	0.0469923	0.0552131
LOC100289572	NC_000011	47469090	47471095	0.0820695	0.0843761	0.109517	0.0571893
CUGBP1	NC_000011	47489945	47574688	5.4093011	10.198979	11.822518	18.507965
PTPMT1	NC_000011	47586982	47595013	4.3787837	5.0295393	7.8052439	6.013206
KBTBD4	NC_000011	47593749	47600567	3.6462833	3.6946938	4.2263424	6.7919555
NDUFS3	NC_000011	47600632	47606114	11.08604	18.622889	19.240581	16.864376
LOC100287127	NC_000011	47603913	47606114	0	0	0.433214	0.5090007
FAM180B	NC_000011	47608198	47610746	0.0306259	0.0314867	0.0544914	0.0426828
C1QTNF4	NC_000011	47611216	47615961	0.0310808	0	0	0.0216583
MTCH2	NC_000011	47638858	47664206	11.823212	13.015158	12.973013	27.62998
AGBL2	NC_000011	47681143	47736302	0.1036821	0.0399735	0.1844769	0.1806245
FNBP4	NC_000011	47738069	47788993	2.6398865	2.9228563	4.0980182	6.017729
NUP160	NC_000011	47799670	47870057	6.4660969	11.180437	9.4348389	8.6873842
LOC100132562	NC_000011	47862752	47863751	0	0	0	0
LOC100287189	NC_000011	47926683	47927159	0	0.0947241	0	0.1926093
PTPRJ	NC_000011	48002110	48192394	1.5336986	2.3252861	4.1321226	4.6368535
LOC100287223	NC_000011	48158649	48161323	0	0.1906473	0.0824843	0.1292189
OR4B1	NC_000011	48238362	48239291	0	0	0	0
OR4B2P	NC_000011	48248881	48249983	0	0	0	0
OR4X2	NC_000011	48266656	48267567	0	0.0495432	0	0.0671598
OR4X1	NC_000011	48285413	48286330	0	0	0	0
OR4S1	NC_000011	48327775	48328704	0	0	0	0
OR4C3	NC_000011	48346493	48347482	0	0	0.0394925	0.0928027
OR4C4P	NC_000011	48366865	48374014	0	0	0	0
OR4C45	NC_000011	48366900	48373999	0	0	0	0
OR4C17P	NC_000011	48373686	48374008	0	0	0	0
OR4C5	NC_000011	48386949	48387940	0	0	0	0
OR4C2P	NC_000011	48441595	48442631	0	0	0	0
OR4C10P	NC_000011	48453754	48454699	0	0	0	0
OR4C9P	NC_000011	48485605	48486529	0	0	0	0
OR4R1P	NC_000011	48507638	48508683	0	0	0	0
OR4A47	NC_000011	48510345	48511274	0	0.0485843	0.0840808	0

OR4A48P	NC_000011	48513173	48514146	0	0	0	0
OR4A46P	NC_000011	48517770	48518910	0	0	0	0
OR4A40P	NC_000011	48533772	48534915	0	0	0	0
OR4A43P	NC_000011	48547516	48548626	0	0	0	0
OR4A45P	NC_000011	48600888	48602003	0	0	0	0
OR4A41P	NC_000011	48611191	48612342	0	0	0	0
OR4A42P	NC_000011	48631515	48632648	0	0	0	0
OR4A44P	NC_000011	48648932	48650112	0	0	0	0
LOC100129384	NC_000011	48903272	48923685	0	0	0	0
LOC100130105	NC_000011	48967246	48973447	0	0	0	0
LOC120824	NC_000011	48997050	49003247	0	0	0	0.0225349
LOC340970	NC_000011	49007237	49016153	0	0	0.0233418	0.0548505
LOC100129108	NC_000011	49031731	49037446	0	0	0	0
LOC283116	NC_000011	49049053	49060003	0	0	0	0
LOC646754	NC_000011	49075266	49080664	0	0.0326706	0	0
UBTFL7	NC_000011	49103468	49104649	0	0	0	0
LOC100129672	NC_000011	49119638	49123487	0	0	0	0
LOC646770	NC_000011	49138562	49146122	0	0	0	0
LOC100128427	NC_000011	49155359	49156371	0	0	0	0
FOLH1	NC_000011	49168187	49230222	0	0	0.0148378	0.0116223
LOC729960	NC_000011	49327267	49401452	0	0	0	0
LOC100286965	NC_000011	49424935	49428269	0	0	0	0
TYRL	NC_000011	49426247	49437309	0	0	0	0
LOC653698	NC_000011	49454018	49456312	0	0	0	0
LOC440040	NC_000011	49580080	49831971	0	0	0	0
TRIM49B	NC_000011	49854689	49860872	0	0	0	0
LOC387770	NC_000011	49865511	49909504	0	0	0	0
LOC100287484	NC_000011	49872175	49911852	0.0572243	0.0588326	0.0169694	0.0265841
LOC100287028	NC_000011	49913421	49918666	0	0	0	0
OR4A1P	NC_000011	49919833	49920738	0	0	0	0
OR4A49P	NC_000011	49936564	49937195	0	0	0	0
OR4A18P	NC_000011	49938942	49940029	0	0	0	0
OR4A19P	NC_000011	49941863	49942965	0	0	0	0
OR4R3P	NC_000011	49944602	49945439	0	0	0	0
OR4C13	NC_000011	49973975	49974904	0	0	0	0
OR4C12	NC_000011	50003108	50004037	0	0	0	0
LOC646797	NC_000011	50128878	50149703	0	0	0	0
PHKG1P3	NC_000011	50205648	50206933	0	0	0	0
LOC646801	NC_000011	50227332	50228446	0	0	0	0
LOC441601	NC_000011	50238999	50257623	0	0	0	0
LOC646813	NC_000011	50368318	50379802	0	0	0	0
OR4A6P	NC_000011	51393463	51394432	0	0	0	0
OR4A5	NC_000011	51411448	51412395	0	0	0.0412422	0
OR4A7P	NC_000011	51425920	51426829	0	0	0	0
OR4A8P	NC_000011	51435359	51436503	0	0	0	0
OR4A2P	NC_000011	51451069	51451981	0	0	0	0
OR4A3P	NC_000011	51455829	51456699	0	0	0	0
OR4A4P	NC_000011	51458684	51459613	0	0	0	0
OR4R2P	NC_000011	51461226	51462282	0	0	0	0
OR4C7P	NC_000011	51483227	51484351	0	0	0	0
OR4C46	NC_000011	51515282	51516211	0	0	0	0
OR4C50P	NC_000011	51526874	51527800	0	0	0	0
TRIM48	NC_000011	55029658	55038595	0	0	0	0.0211937
LOC440041	NC_000011	55059539	55065745	0	0	0	0
OR4A11P	NC_000011	55085936	55087053	0	0	0	0
OR4A12P	NC_000011	55093132	55094276	0	0	0	0
OR4A16	NC_000011	55110677	55111663	0	0	0	0
OR4A15	NC_000011	55135360	55136394	0	0	0	0
OR4A9P	NC_000011	55155905	55157048	0	0	0	0
OR4X7P	NC_000011	55178902	55179293	0	0	0	0
OR4A10P	NC_000011	55199333	55199943	0	0	0	0
OR4A17P	NC_000011	55211811	55212914	0	0	0	0
OR4A13P	NC_000011	55234147	55235288	0	0	0	0
OR4A50P	NC_000011	55243319	55244004	0	0	0	0
OR4A14P	NC_000011	55244927	55246085	0	0	0	0
OR4A21P	NC_000011	55258532	55259721	0	0	0	0

OR4C1P	NC_000011	55277114	55278238	0	0	0	0
OR4C14P	NC_000011	55304378	55305500	0	0	0	0
OR4C15	NC_000011	55321783	55322895	0	0	0	0
OR4C16	NC_000011	55339604	55340536	0	0	0	0
OR4C11	NC_000011	55370917	55371849	0	0	0	0
OR4P4	NC_000011	55405834	55406772	0	0	0	0
OR4S2	NC_000011	55418380	55419315	0	0.0482729	0.0417709	0
OR4C6	NC_000011	55432643	55433572	0	0	0	0.03293
OR4V1P	NC_000011	55441013	55442152	0	0	0	0
OR4P1P	NC_000011	55450716	55451851	0	0	0	0
OR5D2P	NC_000011	55482473	55483109	0	0	0	0
OR5D3P	NC_000011	55493890	55494538	0	0	0	0
OR5D17P	NC_000011	55522453	55523430	0	0	0	0
OR5D13	NC_000011	55540914	55541858	0	0	0	0
OR5D15P	NC_000011	55554441	55555382	0	0	0	0
OR5D14	NC_000011	55563032	55563976	0	0	0	0.0324073
OR5L1	NC_000011	55578943	55579878	0.0469532	0	0	0.0981567
OR5D18	NC_000011	55587106	55588047	0	0	0.0415048	0.0325105
OR5L2	NC_000011	55594695	55595630	0.0939065	0	0	0.0327189
OR5D16	NC_000011	55606228	55607214	0	0	0	0
OR9M1P	NC_000011	55623068	55623997	0	0	0	0
LOC100129915	NC_000011	55631411	55648722	0	0	0	0
SPRYD5	NC_000011	55650773	55659286	0	0	0	0.0375996
OR5W1P	NC_000011	55670720	55671722	0	0	0	0
OR5W2	NC_000011	55681126	55682058	0	0	0	0.0328241
OR5I1	NC_000011	55702932	55703876	0.0465061	0.0478131	0	0.0648146
OR10AF1P	NC_000011	55715595	55716399	0	0	0	0
OR10AK1P	NC_000011	55724691	55725105	0	0	0	0
OR10AG1	NC_000011	55735034	55735939	0.048508	0	0	0
OR7E5P	NC_000011	55746704	55747355	0	0	0	0
OR5F1	NC_000011	55761157	55762101	0	0	0	0
OR5F2P	NC_000011	55782393	55783533	0	0	0	0
OR5AS1	NC_000011	55797895	55798869	0	0	0	0.0314101
OR5AQ1P	NC_000011	55821841	55822776	0	0	0	0
OR5J1P	NC_000011	55838587	55839522	0	0	0	0
OR5BE1P	NC_000011	55850277	55851212	0	0	0	0
OR8I2	NC_000011	55860784	55861716	0	0	0	0
OR8I4P	NC_000011	55864620	55864960	0	0	0	0
OR8H2	NC_000011	55872519	55873457	0	0	0	0
OR5BN2P	NC_000011	55884015	55884832	0	0	0	0
OR8H3	NC_000011	55889849	55890787	0	0	0	0
OR5BN1P	NC_000011	55900042	55901070	0	0	0	0
OR8J3	NC_000011	55904247	55905194	0	0	0	0
OR8K4P	NC_000011	55909991	55910862	0	0	0	0
OR8K5	NC_000011	55926870	55927793	0	0	0	0
OR5J7P	NC_000011	55932876	55933497	0	0	0	0
OR5J2	NC_000011	55944094	55945032	0	0	0	0.0652287
OR8V1P	NC_000011	55955807	55956727	0	0	0	0
LOC642890	NC_000011	55972597	55973100	0	0	0	0
OR8J2	NC_000011	55978232	55979396	0	0	0	0
OR5T2	NC_000011	55999582	56000661	0	0	0	0
OR5T3	NC_000011	56019676	56020698	0	0	0	0
OR5T1	NC_000011	56043115	56044095	0	0	0	0.031218
OR8H1	NC_000011	56057603	56058538	0	0.0965457	0	0
OR8I1P	NC_000011	56064016	56064946	0	0	0	0
OR8K3	NC_000011	56085783	56086721	0	0	0	0.0652287
FAM8A2P	NC_000011	56095320	56100703	0	0	0	0
OR8K2P	NC_000011	56102518	56103644	0	0	0	0
OR8K1	NC_000011	56113515	56114474	0.0457794	0	0.0407266	0.0638018
RPL5P29	NC_000011	56124749	56125762	0	0	0	0
OR8J1	NC_000011	56127723	56128673	0.0462127	0	0.0411121	0
OR8U1	NC_000011	56143100	56144029	0.1417685	0	0	0
OR8L1P	NC_000011	56149011	56150165	0	0	0	0
OR5AL2P	NC_000011	56161105	56162224	0	0	0	0
OR5AL1	NC_000011	56180072	56181256	0	0	0	0
OR5R1	NC_000011	56184734	56185708	0	0	0.0401001	0

OR5M4P	NC_000011	56216044	56217168	0	0	0	0
OR5M9	NC_000011	56229945	56230877	0	0	0	0.0328241
OR5M3	NC_000011	56237050	56237973	0	0.0488998	0	0.0331438
OR5M2P	NC_000011	56246967	56247902	0	0	0	0
OR5M8	NC_000011	56257911	56258846	0	0	0	0
LOC100128210	NC_000011	56259265	56263901	0	0	0	0
OR5M7P	NC_000011	56267775	56268746	0	0	0	0
LOC100129607	NC_000011	56268807	56277684	0	0	0	0
OR5M6P	NC_000011	56279710	56280646	0	0	0	0
OR5M5P	NC_000011	56294043	56294978	0	0	0	0
OR5M11	NC_000011	56309816	56310733	0	0	0	0.0333604
OR5M10	NC_000011	56344250	56345197	0	0	0	0
OR5M13P	NC_000011	56364983	56365470	0	0	0	0
OR5M1	NC_000011	56380031	56380978	0	0	0	0
OR5AM1P	NC_000011	56387222	56388286	0	0	0	0
OR5M12P	NC_000011	56396308	56397475	0	0	0	0
OR5AP1P	NC_000011	56400639	56401578	0	0	0	0
OR5AP2	NC_000011	56408965	56409915	0	0	0	0.0644056
OR5AR1	NC_000011	56431162	56432094	0.0471042	0	0	0
OR2AH1P	NC_000011	56436496	56437420	0	0	0	0
LOC642975	NC_000011	56457694	56459162	0	0	0	0
OR9G1	NC_000011	56467864	56468781	0.0478739	0	0	0.0667209
OR9G9	NC_000011	56467864	56468781	0	0	0	0
OR9G3P	NC_000011	56507555	56508668	0	0	0	0
OR9G4	NC_000011	56510304	56511287	0	0	0	0
OR9G2P	NC_000011	56518473	56519460	0	0	0	0
OR5G1P	NC_000011	56542760	56543682	0	0	0	0
OR5G4P	NC_000011	56557597	56558577	0	0	0	0
OR5G5P	NC_000011	56569335	56570270	0	0	0	0
OR5G3	NC_000011	56586955	56588122	0	0	0	0
LOC643181	NC_000011	56658083	56695366	0	0	0	0
OR5AK3P	NC_000011	56738425	56739551	0	0	0	0
OR5AK2	NC_000011	56756389	56757318	0	0	0.0420404	0
OR5AK1P	NC_000011	56785466	56786593	0	0	0	0
OR5BQ1P	NC_000011	56796762	56797297	0	0	0	0
OR5AK4P	NC_000011	56805009	56805936	0	0	0	0
OR5AO1P	NC_000011	56812454	56812984	0	0	0	0
OR5BP1P	NC_000011	56823075	56823708	0	0	0	0
LRRC55	NC_000011	56949221	56957578	0.0115745	0.0118998	0.010297	0.0161311
APLNR	NC_000011	57001067	57004913	0.011424	0.0234902	0	0.0238822
TNKS1BP1	NC_000011	57067103	57092413	1.8274007	2.0495564	2.9491119	4.5148016
SSRP1	NC_000011	57093459	57103351	5.3006026	9.2803085	9.8472052	22.411892
P2RX3	NC_000011	57105949	57137549	0	0.033494	0	0.0454038
PRG3	NC_000011	57144242	57148623	0	0	0.0449914	0
PRG2 (NC_000011 5715	NC_000011	57154834	57158130	0.3076889	0.421782	0.1368643	0.035735
SLC43A3	NC_000011	57174427	57195053	21.08643	26.658527	21.197475	5.7408082
RTN4RL2	NC_000011	57228339	57244384	0.1739835	0.2504227	0.9286833	0.7031841
SLC43A1	NC_000011	57252007	57283159	0.7565148	0.6873376	1.659064	0.8214039
TIMM10	NC_000011	57295936	57298232	3.1298444	6.1004296	8.0051396	12.268126
SMTNL1	NC_000011	57308979	57318108	0.3410144	0.1095621	0.2085709	0.0594081
UBE2L6	NC_000011	57319129	57335453	68.258676	44.826349	81.172388	21.824626
RPS4P13	NC_000011	57343702	57344578	0	0	0	0
SERPING1	NC_000011	57365027	57382326	20.67761	6.1292663	17.153244	3.258623
YPEL4	NC_000011	57412560	57417417	0.4083459	0.1574335	0.8400755	0.6758103
CLP1	NC_000011	57425216	57429337	2.928295	4.4302252	4.1512042	5.2589851
ZDHHC5	NC_000011	57435474	57468659	2.6793001	2.6456071	3.0695019	7.8039712
MED19	NC_000011	57471186	57479673	5.1055372	4.7718444	7.3033736	7.6410587
TMX2	NC_000011	57480042	57508445	3.6887005	4.1174323	9.047758	10.153212
C11orf31	NC_000011	57508722	57510883	6.8265801	6.7391009	7.4327675	6.3900442
LOC643376	NC_000011	57510986	57519253	0.201862	0.1334156	0.2308911	0.472234
CTNND1	NC_000011	57529234	57586652	6.678747	7.7203136	6.8035921	8.232546
OR5BA1P	NC_000011	57633774	57634752	0	0	0	0
LOC100287801	NC_000011	57637795	57638043	0	0	0	0
OR5AZ1P	NC_000011	57684774	57685702	0	0	0	0
OR5BD1P	NC_000011	57712969	57713995	0	0	0	0
CYCSP26	NC_000011	57772858	57773182	0	0	0	0

OR6Q1	NC_000011	57798425	57799378	0	0	0	0.0321015
VN2R9P	NC_000011	57808240	57834431	0	0	0	0
OR9L1P	NC_000011	57844721	57846177	0	0	0	0
OR9I3P	NC_000011	57876192	57877133	0	0	0	0
OR9I1	NC_000011	57885972	57886916	0	0	0	0
OR9I2P	NC_000011	57912321	57912806	0	0	0	0
OR5BL1P	NC_000011	57938461	57939293	0	0	0	0
OR9Q1	NC_000011	57946903	57949059	0	0	0	0
OR9Q2	NC_000011	57957963	57958907	0	0	0	0.0324073
OR1S2	NC_000011	57970676	57971653	0.0449368	0	0	0
OR1S1	NC_000011	57982217	57983194	0	0	0	0
OR10Q1	NC_000011	57995388	57996347	0	0	0	0
LOC100133309	NC_000011	58009512	58010028	0	0	0	0
OR10W1	NC_000011	58034264	58035732	0	0	0.0266151	0
OR10Q2P	NC_000011	58059301	58060243	0	0	0	0
OR5BC1P	NC_000011	58085112	58085945	0	0	0	0
OR5B19P	NC_000011	58111286	58112143	0	0	0	0
OR5B10P	NC_000011	58116458	58117111	0	0	0	0
OR5B17	NC_000011	58125598	58126542	0	0	0	0.0324073
OR5B1P	NC_000011	58133394	58134041	0	0	0	0
OR5B15P	NC_000011	58155044	58155959	0	0	0	0
OR5B3	NC_000011	58169938	58170882	0	0	0	0
OR5B2	NC_000011	58189805	58190734	0	0	0	0.03293
OR5B12	NC_000011	58206680	58207624	0	0	0	0.0648146
OR5B21	NC_000011	58274649	58275578	0.0472562	0.2429215	0	0.1317199
LPXN	NC_000011	58294344	58345639	11.197414	16.990911	15.604478	29.211654
ZFP91	NC_000011	58346587	58388515	3.6560346	3.9146823	6.632422	11.581922
ZFP91-CNTF	NC_000011	58346587	58393206	0	0	0	0
CNTF	NC_000011	58390146	58393206	0.2320392	0.1669925	0.2270714	0.2263719
GLYAT	NC_000011	58476230	58499447	0.0354994	0	0.0157906	0.0123687
LOC100101405	NC_000011	58562676	58564428	0	0	0	0
GLYATL2	NC_000011	58601538	58611997	0.1910793	0.5238655	0.4249735	0.9320615
LOC100129933	NC_000011	58652420	58660933	0	0	0	0
LOC100287413	NC_000011	58672903	58676071	0.1105616	0.1136689	0.0245897	0.2503921
GLYATL1	NC_000011	58710722	58724516	0.0212516	0.131093	0.0945299	0.1480894
LOC100129032	NC_000011	58746304	58761161	0	0	0	0
LOC100287485	NC_000011	58763236	58767745	0	0	0	0
LOC643637	NC_000011	58808291	58818825	0.0601207	0.0309052	0.1337126	0.0628417
LOC100287520	NC_000011	58853499	58862259	0	0	0.0985653	0.0257352
FAM111B	NC_000011	58874658	58894888	2.9249455	4.0095352	10.836646	15.824144
FAM111A	NC_000011	58910318	58922512	6.9902439	6.4072861	12.090292	14.683161
DTX4	NC_000011	58939812	58976060	0.0455029	0.1481423	0.0404807	0.0211388
MPEG1	NC_000011	58975983	58980494	0	0	0.0086652	0.0203623
LOC643709	NC_000011	59054812	59057481	0	0	0	0
OR5AN2P	NC_000011	59077115	59078061	0	0	0	0
OR5BR1P	NC_000011	59100229	59101558	0	0	0	0
OR5AN1	NC_000011	59131932	59132867	0	0	0	0
OR5BB1P	NC_000011	59158826	59159764	0	0	0	0
OR5A2	NC_000011	59189452	59190426	0	0	0	0
OR5A1	NC_000011	59210642	59211589	0.0463589	0	0	0
OR4D6	NC_000011	59224434	59225378	0	0	0.0413731	0.0648146
OR4D10	NC_000011	59244903	59245838	0	0	0	0.0327189
OR4D8P	NC_000011	59259025	59260183	0	0	0	0
OR4D11	NC_000011	59271049	59271984	0	0	0	0
OR4D9	NC_000011	59282386	59283330	0.1395182	0.1912525	0.0827462	0.3888873
OR4D7P	NC_000011	59299070	59300283	0	0	0	0
RNU7-58P	NC_000011	59326273	59326328	0	0	0	0
OSBP	NC_000011	59341871	59383617	1.0461806	1.6267091	2.1460202	3.9222498
PATL1	NC_000011	59404189	59436511	5.895235	4.8903101	14.739688	14.993584
OR10V1	NC_000011	59480389	59481318	0.0472562	0.0485843	0	0
OR10Y1P	NC_000011	59495892	59497065	0	0	0	0
OR10V3P	NC_000011	59508698	59509622	0	0	0	0
OR10V2P	NC_000011	59516184	59517147	0	0	0	0
STX3	NC_000011	59522889	59570179	4.8250438	4.3309719	7.1231459	12.168757
FABP5L7	NC_000011	59548580	59549224	0	0	0	0
MRPL16	NC_000011	59573608	59578345	6.4695367	8.1039604	11.577113	8.4205461

GIF	NC_000011	59596746	59612974	0.0580942	0.0895904	0	0.121447
TCN1	NC_000011	59620281	59634041	0	0.0288343	0	0.0390873
LOC390203	NC_000011	59665658	59666610	0	0	0	0
PLAC1L	NC_000011	59807748	59815517	0.0269126	0.027669	0	0.0562613
MS4A3	NC_000011	59824101	59838588	0.0265548	0	0	0
LOC441609	NC_000011	59844608	59848035	0	0	0	0
MS4A2	NC_000011	59856137	59866017	0.0425649	0.0109403	0.0378669	0.0148304
MS4A6A	NC_000011	59939080	59950674	0.0425237	0	0.0189151	0
MS4A4E	NC_000011	59974148	60008328	0	0	0	0.0836745
MS4A4A	NC_000011	60048139	60076445	0	0	0	0.0370986
MS4A6E	NC_000011	60102355	60108441	0	0	0	0
MS4A7	NC_000011	60145958	60163427	0.0146056	0	0.0129935	0.0101778
MS4A14	NC_000011	60163487	60185229	0	0	0.0116605	0.0091336
MS4A5	NC_000011	60197062	60215265	0.0608701	0	0	0
MS4A1	NC_000011	60223282	60238225	0.0978258	0.1382909	0.1305428	0.0170422
MS4A12	NC_000011	60260251	60274903	0.0376592	0	0	0.0262424
MS4A13	NC_000011	60282886	60310194	0	0.2434451	0.084262	0
C11orf64	NC_000011	60383224	60454622	0	0	0	0
MS4A8B	NC_000011	60467047	60483284	0	0	0	0.0452696
LOC728588	NC_000011	60489669	60511467	0.0373709	0	0	0
MS4A15	NC_000011	60524340	60544204	0	0.0230645	0.0199579	0.0156329
MS4A10	NC_000011	60552821	60568778	0.0382824	0	0.0170286	0.0133384
CCDC86	NC_000011	60609429	60618561	5.9760871	9.8663508	4.5403625	7.1888672
GPR44	NC_000011	60618398	60623444	0.0453075	0.0621078	0.0134356	0.0631441
ZP1	NC_000011	60635015	60643164	0.0222861	0	0.0198264	0.1552986
PRPF19	NC_000011	60658019	60674061	8.4479728	12.46838	13.711705	28.741075
TMEM109	NC_000011	60681371	60690915	8.8338154	11.125561	11.951936	23.225148
TMEM132A	NC_000011	60691913	60704631	2.8716174	3.2603932	10.140931	3.3147951
SLC15A3	NC_000011	60704560	60719257	9.4516804	2.9109155	9.6123338	1.2621338
CD6	NC_000011	60739115	60787849	0.0133622	0.0412132	0	0.2886504
TRNAA45P	NC_000011	60763508	60763577	0	0	0	0
CD5	NC_000011	60869930	60895324	0.027833	0.0572304	0.0742829	0.0290927
VPS37C	NC_000011	60897728	60928916	4.620115	2.584786	6.9713341	6.6892379
PGA3	NC_000011	60970984	60980350	0.0944446	0.2265643	0	0.0219376
PGA4	NC_000011	60989821	60999179	0.0315041	0.1619477	0	0.0219533
PGA5	NC_000011	61008669	61018916	0.0969446	0.1661154	0	0.0450366
VWCE	NC_000011	61025758	61062788	0.3852928	0.0766687	0.1547981	0.1645568
DDB1	NC_000011	61066919	61100666	7.1018235	11.196901	14.312341	23.028666
DAK	NC_000011	61100654	61116231	1.2331619	1.26782	1.3275288	1.4947771
CYBASC3	NC_000011	61116277	61129729	12.208667	8.7343379	10.028744	21.03067
TMEM138	NC_000011	61129856	61136678	10.627757	8.7100534	10.968852	16.867403
TMEM216	NC_000011	61159865	61166323	2.1715597	3.1894166	0.8586132	0.9607805
CPSF7	NC_000011	61170120	61197464	7.1866501	7.7724567	10.451619	17.950552
C11orf79	NC_000011	61197597	61214229	4.144002	3.4382738	24.027702	17.908841
C11orf66	NC_000011	61248592	61258399	0.0287056	0.088537	0.2298355	0.0800127
LOC390205	NC_000011	61276272	61278490	0.1188325	0.2443447	0.2466723	0.069006
SYT7	NC_000011	61282785	61348298	0.2036436	0.7377696	0.34508	0.2500266
RPLP0P2	NC_000011	61382508	61406921	0	0	0	0
DAGLA	NC_000011	61447910	61514473	1.9712524	1.2097086	2.1819052	2.7792396
C11orf9	NC_000011	61520121	61555989	0.0514186	0.1510393	0.2025781	0.1842714
DKFZP434K028	NC_000011	61521501	61525136	0	0	0	0
C11orf10	NC_000011	61556602	61560085	33.576021	28.803045	65.733377	48.880584
FEN1	NC_000011	61560150	61564710	6.389983	11.511823	16.897658	24.892075
FADS1	NC_000011	61567097	61584529	22.779502	15.885748	74.886876	49.02752
LOC100131326	NC_000011	61584002	61593982	0.6140472	0.1803729	1.404703	0.611275
FADS2	NC_000011	61595713	61634825	8.2900126	5.7106999	21.765015	16.737191
FADS3	NC_000011	61640998	61659006	18.849073	15.492903	25.141998	26.665507
RAB3IL1	NC_000011	61664773	61684997	3.8291148	4.2334711	3.6974931	3.6739127
BEST1	NC_000011	61717356	61731935	1.1312119	1.0426939	1.5153199	1.1869405
FTH1	NC_000011	61731757	61735132	490.47514	284.40911	1014.628	817.40267
LOC399900	NC_000011	61735266	61736962	0	0	0	0
RPS2P37	NC_000011	61779193	61780120	0	0	0	0
INCENP	NC_000011	61891445	61920635	0.5300076	1.2205839	1.5465511	1.8466521
LOC283236	NC_000011	61936758	61937293	0	0	0	0
SCGB1D1	NC_000011	61957710	61961009	0	0	0	0
SCGB2A1	NC_000011	61976140	61981411	0	0	0	0

SCGB1D2	NC_000011	62009724	62012280	0.1003384	0	0	0
SCGB2A2	NC_000011	62037630	62040628	0	0	0	0
SCGB1D4	NC_000011	62063754	62066536	0	0	0	0.0705642
LOC100129067	NC_000011	62098420	62099299	0	0	0	0
LOC100287007	NC_000011	62104290	62105562	0.076035	0	0	0
ASRGL1	NC_000011	62104774	62160887	0.1271755	0.1307498	0.0646508	0.0379804
SCGB1A1	NC_000011	62186523	62190667	0	0.2126278	0	0.1441171
AHNAK	NC_000011	62201016	62314332	14.647129	21.668705	31.005265	74.80305
EEF1G	NC_000011	62327073	62341460	0.9715474	2.7321563	3.4064199	7.2281085
TUT1	NC_000011	62342520	62359016	1.6581201	1.9198808	2.8070047	3.3204996
MTA2	NC_000011	62360675	62369303	13.270643	18.811652	22.206395	26.691683
EML3	NC_000011	62369691	62380237	4.2530547	3.6507633	6.7865206	6.614221
ROM1	NC_000011	62380213	62382592	0.5850404	0.4571271	1.2491235	1.8753253
B3GAT3	NC_000011	62382768	62389448	21.425525	16.293598	37.850348	23.191655
GANAB	NC_000011	62392298	62414104	28.858181	28.759356	56.767712	47.338583
INTS5	NC_000011	62414320	62420774	3.9867802	5.5017841	4.5584072	7.3928549
C11orf48	NC_000011	62430289	62439241	1.6647057	3.2860656	6.0719704	5.127347
METTL12	NC_000011	62432779	62434923	0.0954014	0.0653884	0.3394868	0.1994384
SNORA57	NC_000011	62432894	62433042	0	0	0	0
C11orf83	NC_000011	62439126	62439852	0.618022	2.0473729	2.5657777	3.5410016
UBXN1	NC_000011	62443972	62446527	6.887155	7.6415684	9.6758134	18.389182
LRRN4CL	NC_000011	62453874	62457200	2.6398068	1.8155717	2.4456224	2.067877
BSCL2	NC_000011	62457747	62477046	8.566035	8.8067844	20.658205	14.491381
GNG3	NC_000011	62475114	62476678	0	0	0	0
HNRNPUL2	NC_000011	62482220	62494821	2.9416487	4.6272159	5.5087266	5.4628715
TTC9C	NC_000011	62495952	62506108	4.2829269	4.5223071	8.3412717	9.0073171
ZBTB3	NC_000011	62518435	62521656	0.7832066	0.698869	0.7493481	1.3283824
POLR2G	NC_000011	62529011	62534187	8.0335477	11.984128	12.705541	17.123588
TAF6L	NC_000011	62538875	62554814	3.400756	3.2497983	3.0835878	4.0104007
TMEM179B	NC_000011	62554874	62557872	12.935865	9.010688	18.030613	9.219762
TMEM223	NC_000011	62557787	62559486	3.0487034	4.4845852	5.6330537	3.8567083
NXF1	NC_000011	62559598	62572964	5.9028611	4.3145954	6.7315317	8.8765623
LOC100287678	NC_000011	62559874	62572968	0.2271226	0.5253884	0.2020546	0.3561032
STX5	NC_000011	62574369	62599560	10.533857	14.78409	19.439815	12.990821
WDR74	NC_000011	62600383	62607628	4.3723186	4.3299383	6.3494218	6.8777159
RNU2-2	NC_000011	62609096	62609233	0	0	0	0
SNHG1	NC_000011	62619460	62623360	0	0	0	0
SNORD22	NC_000011	62620383	62620507	0	0	0	0
SNORD31	NC_000011	62620798	62620865	0	0	0	0
SNORD30	NC_000011	62621135	62621204	0	0	0	0
SNORD29	NC_000011	62621376	62621440	0	0	0	0
SNORD28	NC_000011	62622093	62622167	0	0	0	0
SNORD27	NC_000011	62622484	62622555	0	0	0	0
SNORD26	NC_000011	62622764	62622838	0	0	0	0
SNORD25	NC_000011	62623037	62623103	0	0	0	0
SLC3A2	NC_000011	62623518	62656353	27.414924	21.681095	23.893791	13.072885
CHRM1	NC_000011	62676151	62689012	0.0155679	0.0160055	0.041549	0.0542417
SLC22A6	NC_000011	62744069	62752469	0	0	0	0.0278915
SLC22A8	NC_000011	62760296	62783311	0	0	0.0179511	0.070305
LOC100127954	NC_000011	62800010	62803206	0	0	0	0
LOC644436	NC_000011	62814257	62815596	0	0	0	0
SLC22A24	NC_000011	62847412	62911693	0	0	0.0186356	0.0145972
RPL29P22	NC_000011	62883239	62883855	0	0	0	0
SLC22A25	NC_000011	62931296	62997124	0	0	0	0.0186283
CCND2P	NC_000011	63010557	63011509	0	0	0	0
LOC100287736	NC_000011	63033309	63033728	0.1046386	0	0	0
SLC22A10	NC_000011	63057430	63079246	0.0341478	0	0	0.0475911
SLC22A9	NC_000011	63137261	63177731	0.0188862	0.019417	0.0168017	0.0526427
HRASLS5	NC_000011	63230809	63258666	0	0.0387508	0	0
LGALS12	NC_000011	63273524	63284246	0.057274	0	0.1188892	0
TMSL5	NC_000011	63287971	63288408	0	0	0	0
RARRES3	NC_000011	63304273	63313930	7.295981	2.185341	8.9438876	1.8815285
HRASLS2	NC_000011	63320242	63330855	0	0	0.2634607	0.1238203
PLA2G16	NC_000011	63341934	63381941	5.1432058	4.3378599	5.5892806	4.3351264
ATL3	NC_000011	63396437	63439083	17.026044	20.929368	26.463645	40.507374
LOC729101	NC_000011	63399694	63400195	0	0	0	0

RTN3	NC_000011	63448922	63527363	12.991931	11.259529	30.831762	30.212526
LOC65998	NC_000011	63527364	63536113	0.8660526	1.5298572	1.1486924	1.5032635
C11orf84	NC_000011	63580923	63595190	4.4374043	6.2210701	6.1806453	14.102124
ATP5LP1	NC_000011	63602064	63602516	0	0	0	0
MARK2	NC_000011	63606895	63677057	6.0896316	7.4043031	7.8294081	8.6807627
TRNAR34P	NC_000011	63661767	63661835	0	0	0	0
LOC100287771	NC_000011	63674585	63677068	0.0383827	0	0	0.0267466
RCOR2	NC_000011	63678702	63684316	0.1862931	0.1044703	0.3163965	0.2006254
NAT11	NC_000011	63706442	63724458	1.9821335	2.2267806	2.5341015	5.5798016
COX8A	NC_000011	63742079	63744015	84.342465	63.185467	91.304769	125.03649
OTUB1	NC_000011	63753325	63765892	7.375793	9.4345464	11.010038	19.784686
MACROD1	NC_000011	63766030	63933585	3.3564282	1.1143082	9.4244729	5.1894185
FLRT1	NC_000011	63871362	63886645	0	0.0139154	0	0
STIP1	NC_000011	63953587	63972020	5.0916852	13.548862	15.507576	25.824523
FERMT3	NC_000011	63974152	63991363	1.1193711	0.6196783	0.720057	0.3600103
TRPT1	NC_000011	63991271	63993681	6.9323104	3.8997579	7.835028	6.3498012
NUDT22	NC_000011	63993738	63997488	7.1543633	5.9813448	10.071645	10.354386
DNAJC4	NC_000011	63997753	64001753	2.8386796	2.3207039	5.537554	3.6225537
VEGFB	NC_000011	64002266	64006259	18.436244	9.3761621	17.835204	27.611697
FKBP2	NC_000011	64008413	64011607	3.7132731	3.914284	5.352394	4.651051
PPP1R14B	NC_000011	64011951	64014413	25.603073	53.789764	48.287672	64.445666
PLCB3	NC_000011	64019122	64035028	3.9737268	5.5976198	6.1521945	8.0013177
BAD	NC_000011	64037300	64052176	5.5294344	4.9834634	9.5507942	8.1065854
GPR137	NC_000011	64053331	64056963	6.1704567	3.0782531	7.3192125	11.321045
KCNK4	NC_000011	64058793	64067503	0.0485616	0.0499264	0	0.0676793
C11orf20	NC_000011	64067863	64072239	0.0590702	0	0.105101	0.0823249
ESRRA	NC_000011	64073044	64084210	5.9560638	6.8354899	11.565556	11.761829
HSPC152	NC_000011	64084163	64085033	51.863436	79.904095	166.44967	109.52465
PRDX5	NC_000011	64085569	64089283	45.737979	41.912205	74.568434	56.434306
CCDC88B	NC_000011	64107695	64125006	0.026825	0.0827366	0.0715927	0.1495416
RPS6KA4	NC_000011	64126625	64139687	7.0048186	12.214066	4.6363705	8.5812139
SLC22A11	NC_000011	64323098	64338999	0.0174953	0.017987	0	0.0243829
LOC100287113	NC_000011	64323098	64329616	0	0	0	0
SLC22A12	NC_000011	64358282	64369820	0	0.0140934	0	0
NRXN2	NC_000011	64373646	64490660	2.0019802	0.0428801	3.0796754	1.1334859
RASGRP2	NC_000011	64494383	64512928	0.034042	0.0524981	0	0.0237218
PYGM	NC_000011	64513861	64528187	0.0850768	0.0749725	0.0432495	0.0762234
SF1	NC_000011	64532078	64546241	8.0971824	9.2459365	9.8801802	13.897975
LOC644613	NC_000011	64545906	64546877	0	0.046485	0.1206715	0.2205495
MAP4K2	NC_000011	64556609	64570713	1.3831423	1.6513731	2.5932734	3.4304009
MEN1	NC_000011	64570986	64578766	1.3861894	1.7225707	2.0803422	3.4690276
CDC42BPG	NC_000011	64591661	64612041	0.0177928	0.0640251	0.0316579	0.068193
EHD1	NC_000011	64620208	64646191	3.6867189	1.9474478	2.6012265	4.1459193
LOC100287827	NC_000011	64621668	64627515	0.1369104	0.2111374	0.1217993	0.4293207
LOC100127953	NC_000011	64621953	64646240	0	0.0564793	0.1466159	0.3062488
RPS16P6	NC_000011	64649007	64649417	0	0	0	0
ATG2A	NC_000011	64662019	64684663	2.8605771	2.8979353	5.4062517	6.3544556
PPP2R5B	NC_000011	64692180	64701947	1.2973648	1.4672101	2.178499	2.7686698
GPHA2	NC_000011	64701943	64703360	0.058833	0	0.0523394	0.1639886
C11orf85	NC_000011	64705929	64739358	0.1563994	0	0.0463791	0.0726569
BATF2	NC_000011	64755417	64764517	6.9208197	1.055687	5.0242199	0.5867383
ARL2	NC_000011	64781586	64789656	32.399463	32.01646	62.5641	49.663657
LOC100287860	NC_000011	64793728	64794387	0	0	0	0
SNX15	NC_000011	64794910	64808044	2.4863327	2.9349093	4.8539146	6.689667
SAC3D1	NC_000011	64808376	64812300	2.5802408	4.8153338	3.3932795	4.8663655
NAALADL1	NC_000011	64812295	64826009	0.4416086	1.3788757	0.0727532	0.1025768
CDCA5	NC_000011	64844927	64851615	7.2678365	12.190398	16.573968	18.581788
ZFPL1	NC_000011	64851694	64855874	7.45154	7.6609664	9.4499888	7.0927748
LOC100130348	NC_000011	64855613	64856536	0.0887843	0	0.15797	0.1237369
C11orf2	NC_000011	64863683	64879180	3.9921103	5.2383944	7.7416295	16.471548
LOC100287251	NC_000011	64875726	64876967	0.1993117	0.153685	0	0
LOC100131416	NC_000011	64876377	64879174	0.1140525	0.039086	0.0676428	0.0529842
TM7SF2	NC_000011	64879341	64883707	1.2469549	0.0854667	1.1339773	0.7337613
ZNHIT2	NC_000011	64883875	64885170	3.5606206	5.2992879	5.1285387	5.2931888
FAU	NC_000011	64888102	64889660	16.872982	23.156493	29.253357	33.796741
MRPL49	NC_000011	64889728	64894841	15.000043	12.674712	16.638457	12.622317

SYVN1	NC_000011	64894751	64902003	2.2182982	1.8659812	4.2929153	5.9222151
LOC100287960	NC_000011	64913162	64927835	0.3515859	0.1204891	0.4170407	0.3266654
SPDYC	NC_000011	64937707	64940688	0	0	0.0443283	0.0347221
LOC440043	NC_000011	64941588	64943941	0	0	0	0
LOC728975	NC_000011	64947342	64949305	0	0	0	0
CAPN1	NC_000011	64949343	64979476	25.796019	29.646442	21.453603	21.07179
SLC22A20	NC_000011	64981309	65009557	0	0	0.0254045	0.0397984
POLA2	NC_000011	65029432	65065088	8.9954596	12.349279	16.368258	27.163043
CDC42EP2	NC_000011	65082331	65089900	13.599327	9.9216455	4.9143331	6.8948489
DPF2	NC_000011	65101346	65120451	9.1946487	11.403698	13.21105	11.110894
TIGD3	NC_000011	65122282	65125084	0.0669943	0.1377543	0.1191999	0.1867371
SLC25A45	NC_000011	65142663	65150142	1.5572208	0.6720191	1.2827285	1.6879854
FRMD8	NC_000011	65154041	65180996	7.4379337	7.9239548	4.9705594	6.2359827
NCRNA00084	NC_000011	65192517	65207155	0	0	0	0
MALAT1	NC_000011	65265233	65273940	0	0	0	0
LOC100288027	NC_000011	65281871	65282053	0	0	0	0
SCYL1	NC_000011	65292548	65306182	3.9319483	4.0253993	5.977544	5.2717797
LTBP3	NC_000011	65306030	65325430	2.9146808	1.2944489	3.8630123	1.8099973
SSSCA1	NC_000011	65337943	65339239	6.2495192	9.6032001	9.3260248	15.827536
FAM89B	NC_000011	65339820	65341669	10.971987	13.604605	15.150977	24.701548
EHBP1L1	NC_000011	65343509	65360116	2.0616642	1.168837	3.6229404	3.5472832
KCNK7	NC_000011	65360326	65363467	0.0562357	0.0578163	0.2001155	0.1567492
MAP3K11	NC_000011	65365226	65381720	5.7564435	5.5641469	6.9813173	11.845391
PCNXL3	NC_000011	65383783	65404910	10.080115	9.1454068	12.802279	16.072697
SIPA1	NC_000011	65405578	65418391	4.0438065	1.3045397	7.8596024	5.9911054
RELA	NC_000011	65421067	65430443	4.6028016	4.5744251	9.7213109	13.720611
KAT5	NC_000011	65479489	65487075	11.186477	12.516232	11.286592	15.008043
RNASEH2C	NC_000011	65485144	65488409	1.4833515	3.098754	1.9654072	3.4198697
LOC100288054	NC_000011	65487531	65488418	1.2765047	0.8749206	0.7029991	0.5082967
KRT8P26	NC_000011	65494338	65495486	0	0	0	0
DKFZp761E198	NC_000011	65543373	65547822	2.5480098	2.2642469	2.8466542	2.6151581
OVOL1	NC_000011	65554529	65564685	0.0293673	0.0150964	0.013063	0.0204643
SNX32	NC_000011	65601410	65621172	0.1031044	0.0530011	0.183449	0.1616562
CFL1	NC_000011	65622282	65625804	63.899334	71.827265	82.073858	153.87786
MUS81	NC_000011	65627872	65633914	8.972764	9.0743332	10.882156	11.356726
EFEMP2	NC_000011	65633912	65640340	36.970929	34.400743	21.764246	21.894571
CTSW	NC_000011	65647284	65651212	0.1356427	0	0.1206715	0.2126728
FIBP	NC_000011	65651211	65656010	16.700328	15.206552	16.501869	22.620169
CCDC85B	NC_000011	65657875	65659106	15.981175	19.877763	18.057236	33.50839
FOSL1	NC_000011	65659692	65667997	30.194648	27.548873	82.297094	127.65874
C11orf68	NC_000011	65684281	65686531	15.494569	15.119061	40.927131	31.410132
DRAP1	NC_000011	65686728	65689031	31.315339	47.515744	37.593813	46.433267
TSGA10IP	NC_000011	65713115	65727434	0.0254625	0.0523562	0.0226521	0.0177433
SART1	NC_000011	65729160	65747607	0.2929068	0.6650154	0.3474374	0.5868138
EIF1AD	NC_000011	65764017	65769578	2.8048766	3.4572631	4.742441	6.176809
BANF1	NC_000011	65769550	65771617	11.063357	14.708139	29.900135	41.285101
CST6	NC_000011	65779462	65780976	0.2886583	0	0	0
CATSPER1	NC_000011	65784223	65793988	0.2349275	0.0345043	0.1343559	0.2455603
GAL3ST3	NC_000011	65808236	65816651	0.0264748	0	0	0.0368974
LOC100287343	NC_000011	65818200	65819790	0	0	0	0
SF3B2	NC_000011	65819816	65836382	1.9286197	3.0913316	2.5128359	10.825588
PACS1	NC_000011	65837824	66012213	5.5229061	7.8023923	13.563928	22.64112
KLC2	NC_000011	66024765	66035332	1.1965104	1.4789306	3.4205885	5.4991751
RAB1B	NC_000011	66036056	66044963	50.489887	51.608459	90.434369	141.40705
CNIH2	NC_000011	66045696	66051683	0.1982627	0.3057523	0.9112966	1.2203899
YIF1A	NC_000011	66052051	66056638	8.1545336	9.6735205	41.015823	28.849127
TMEM151A	NC_000011	66059373	66064135	0.2084501	0.0535772	0.1545358	0.0968376
CD248	NC_000011	66081958	66084515	57.14301	44.017607	25.433287	8.5840648
RIN1	NC_000011	66099542	66104000	8.4981864	9.5619122	13.372416	17.046784
BRMS1	NC_000011	66104804	66112582	2.3549157	2.7040867	3.5642179	11.913227
LOC100128074	NC_000011	66112726	66128025	1.1504773	1.3010927	2.4905081	1.5232269
B3GNT1	NC_000011	66112843	66115161	0.6369309	0.327416	1.4165785	0.6953475
SLC29A2	NC_000011	66129992	66139291	0.9436201	0.3952425	0.1710033	0.7184365
NPAS4	NC_000011	66188475	66194177	0.0798575	0.1368365	0.0947246	0.1205704
MRPL11	NC_000011	66202550	66206310	6.438442	9.9424924	22.632992	21.815231
PELI3	NC_000011	66234336	66244808	0.8488543	1.1197053	4.6164764	3.8169491

DPP3	NC_000011	66247880	66277130	10.02615	8.6600119	12.644505	38.295345
BBS1	NC_000011	66278119	66301084	1.9561528	1.2871236	4.5014408	3.7985754
ZDHHC24	NC_000011	66306735	66313671	2.9365561	3.4307822	2.8796233	6.0226602
ACTN3	NC_000011	66314391	66330797	0.0922959	0.015815	0.0684242	0.0643155
CTSF	NC_000011	66330935	66336047	7.5501928	6.5957896	36.787431	18.825024
CCDC87	NC_000011	66357640	66360554	0.1055361	0.1395028	0.2682509	0.0525298
CCS	NC_000011	66360690	66373490	3.2364977	3.2836775	4.2431467	5.1931722
RBM14	NC_000011	66384053	66397405	3.6359756	5.1983859	4.9025387	8.6812905
RBM4	NC_000011	66406138	66413940	12.377148	17.232356	14.625914	23.17357
RBM4B	NC_000011	66432469	66445275	3.3158852	2.9370522	4.1525563	5.2256031
SPTBN2	NC_000011	66452720	66488870	0.0446912	0.0689209	0.2882495	0.0973207
C11orf80	NC_000011	66512207	66610987	1.6459303	1.847793	6.0085368	4.1922993
RCE1	NC_000011	66610883	66614003	2.6590025	3.1023274	6.6978834	8.3484542
PC	NC_000011	66615997	66725847	0.808291	0.6925067	2.8025607	3.8272071
LRFN4	NC_000011	66624876	66627946	0.2259996	0.0536195	0.1237265	0.2301712
RNU7-23P	NC_000011	66687234	66687293	0	0	0	0
C11orf86	NC_000011	66742754	66744479	0	0	0	0.0516876
SYT12	NC_000011	66790891	66818330	0.1024434	0.0921573	0.1139206	0.21416
RHOD	NC_000011	66824289	66839488	2.2497771	2.3927662	3.4853081	3.4598274
KDM2A	NC_000011	66887498	67025141	10.919826	8.1926748	21.601248	26.556356
ADRBK1	NC_000011	67033905	67054029	5.2191715	4.499973	13.293626	15.801512
ANKRD13D	NC_000011	67056819	67069954	1.0496874	1.1199131	3.2595988	3.7401271
SSH3	NC_000011	67071001	67080071	1.3709201	0.9558339	3.4765853	3.3490813
LOC100130987	NC_000011	67085310	67159158	0	0	0	0
POLD4	NC_000011	67119019	67121017	19.580421	21.410436	31.516556	41.667182
CLCF1	NC_000011	67131639	67141206	4.771795	1.6925379	2.8017799	6.6337277
RAD9A	NC_000011	67159423	67165883	0.7248766	0.4897353	1.7319374	3.0451693
PPP1CA	NC_000011	67165652	67169376	17.514964	21.73493	31.839934	64.387635
TBC1D10C	NC_000011	67171412	67177560	0.0773283	0.0265005	0	0.0359236
ATPGD1	NC_000011	67183149	67193078	0.1226359	0.0573103	0.0396728	0.0310755
RPS6KB2	NC_000011	67195935	67202879	11.694699	14.576114	19.349982	25.34771
PTPRCAP	NC_000011	67202981	67205153	0.2422725	0.0996326	0.4310647	1.1142458
CORO1B	NC_000011	67205518	67211263	3.5158585	1.9927039	10.686668	14.181674
GPR152	NC_000011	67218772	67220200	0	0.0632378	0	0.064293
CABP4	NC_000011	67222818	67226691	0	0	0	0
TMEM134	NC_000011	67231819	67236731	2.0019802	4.0184804	8.3538183	8.0049845
AIP	NC_000011	67250505	67258579	13.223677	15.31765	32.026392	38.026511
PITPNM1	NC_000011	67259239	67272843	4.1181968	4.1377133	10.741191	10.210708
CDK2AP2	NC_000011	67273968	67276102	6.8569447	8.6563266	19.860882	29.980378
CABP2	NC_000011	67286418	67290899	0	0	0	0
GSTP1	NC_000011	67351066	67354124	50.095479	52.635362	95.703331	126.07987
NDUFV1	NC_000011	67374323	67380012	9.8620802	12.383189	18.553964	23.677481
LOC100287422	NC_000011	67377386	67379399	0.0292793	0	0.0260477	0
LOC390213	NC_000011	67380308	67384027	0.0339107	0.1045912	0.2111751	0.307194
NUDT8	NC_000011	67395409	67397401	0.4889173	0.781913	1.5948327	1.1356568
TBX10	NC_000011	67398774	67407031	0.0283903	0.0291882	0.0252568	0.0791341
ACY3	NC_000011	67410026	67418130	0	0	0	0.0706728
ALDH3B2	NC_000011	67429633	67448685	0	0.0336061	0.0436194	0.0113889
RPL37P2	NC_000011	67450164	67450529	0	0	0	0
LOC729196	NC_000011	67477977	67483550	0.0770121	0.0527843	0.0228374	0.0715535
OR7E145P	NC_000011	67489930	67490955	0	0	0	0
OR7E11P	NC_000011	67503149	67503812	0	0	0	0
LOC100131501	NC_000011	67511793	67512119	0	0	0	0
LOC645015	NC_000011	67552741	67560816	0	0	0	0
LOC645332	NC_000011	67559238	67572807	0	0	0	0
RPS3AP40	NC_000011	67693078	67693926	0	0	0	0
LOC100287494	NC_000011	67703029	67703397	0	0	0	0
LOC100132261	NC_000011	67722755	67733631	0	0	0	0.0559359
OR7E1P	NC_000011	67741606	67742828	0	0	0	0
UNC93B1	NC_000011	67758575	67771593	4.3093947	1.0344364	8.7146194	3.1881623
LOC100288224	NC_000011	67776148	67783243	0.1178237	0	0.262048	0.5747296
ALDH3B1	NC_000011	67777790	67796743	8.2588329	2.3113357	12.722334	14.784799
NDUFS8	NC_000011	67798094	67804114	24.077432	32.607165	82.085191	54.38423
TCIRG1	NC_000011	67806483	67818366	8.8333035	5.2813712	12.85559	10.749129
CHKA	NC_000011	67820326	67888858	0.9381662	1.0643127	3.050675	3.956324
SUV420H1	NC_000011	67923507	67980784	0.3923276	0.3800836	0.932972	1.7086842

C11orf24	NC_000011	68028803	68039469	9.0530849	12.488847	18.432772	22.043502
LRP5	NC_000011	68080108	68216743	2.1597025	2.8019348	3.0039861	1.8394038
SAPS3	NC_000011	68228199	68382802	7.0735557	8.8762875	15.897679	26.86317
GAL	NC_000011	68451983	68458643	0.1694662	0.3484581	0	0.0393636
MTL5	NC_000011	68474908	68518988	0.1377957	0	1.0802972	0.7021577
CPT1A	NC_000011	68522088	68609399	5.5285834	6.7795697	9.8082498	17.872422
MRPL21	NC_000011	68658746	68671303	20.083536	32.437731	60.217971	35.333075
IGHMBP2	NC_000011	68671319	68708069	1.2337516	1.428408	3.0455362	3.5395977
MRGPRD	NC_000011	68747490	68748455	0	0	0	0
MRGPRF	NC_000011	68771862	68780850	9.8902763	8.664768	5.4092954	4.5320529
TPCN2	NC_000011	68816350	68858072	1.2416731	1.3574798	2.1859164	1.9376664
MYEOV	NC_000011	69061622	69064754	0.0768662	0.0790265	0.0341911	0.1071268
IFITM9P	NC_000011	69070818	69071273	0	0	0	0
CCND1	NC_000011	69455873	69469242	50.045037	56.213251	13.409307	32.202891
ORAOV1	NC_000011	69480331	69490165	1.3038439	0.8221663	0.3557136	0.6178278
FGF19	NC_000011	69513006	69519106	0	0	0.0182955	0.0286616
LOC100129779	NC_000011	69552059	69552784	0	0	0	0
FGF4	NC_000011	69587797	69590171	0	0.0370356	0	0
FGF3	NC_000011	69624736	69634192	0.2839033	0.1459412	0.2020546	0.1582681
LOC100127946	NC_000011	69830650	69866574	0.0579028	0.0595302	0	0
ANO1	NC_000011	69924408	70035651	0.0274276	0.0093995	0.0081335	0.0063709
FADD	NC_000011	70049269	70053508	13.234276	16.410377	7.4059609	9.51966
PPFIA1	NC_000011	70116823	70230502	1.9333973	2.163124	1.5104235	2.4567832
CTTN	NC_000011	70244612	70282690	20.159125	25.821054	14.287711	15.671798
SHANK2	NC_000011	70313961	70507872	0.1181757	0.1889954	0.019469	0.0243999
C11orf76	NC_000011	70709441	70710014	0	0	0	0
LOC100128069	NC_000011	71093647	71101539	0.1101459	0	0	0
DHCR7	NC_000011	71145457	71159477	11.741516	11.325521	12.968949	9.7103273
NADSYN1	NC_000011	71164217	71212584	3.5223614	3.3613628	3.1817994	4.5062501
LOC645474	NC_000011	71217107	71233133	0	0	0	0
KRTAP5-7	NC_000011	71238313	71239210	0	0	0	0
KRTAP5-8	NC_000011	71249071	71250253	0	0.1145817	0	0
KRTAP5-9	NC_000011	71259466	71260653	0.0739869	0	0.0329104	0
KRTAP5-10	NC_000011	71276609	71277666	0.041539	0	0.0369542	0
KRTAP5-11	NC_000011	71292901	71293921	0	0	0	0
OR7E87P	NC_000011	71304704	71305351	0	0	0	0
UNC93B6	NC_000011	71314210	71319090	0	0	0	0
OR7E4P	NC_000011	71331146	71332068	0	0	0	0
LOC645544	NC_000011	71340197	71353366	0	0	0	0
RPS3AP41	NC_000011	71380542	71381387	0	0	0	0
LOC100129925	NC_000011	71468504	71468677	0	0	0	0
FAM86C	NC_000011	71498557	71512280	1.4034563	0.762676	0.3924026	0.7125314
LOC100288439	NC_000011	71505658	71512282	0	0.058226	0.0503835	0
LOC285407	NC_000011	71511277	71515701	0	0	0	0
LOC100287624	NC_000011	71524486	71524928	0	0	0	0
LOC100131539	NC_000011	71525091	71554465	0.0115928	0	0.0206265	0
DEFB108B	NC_000011	71544246	71548608	0	0	0.1761152	0
LOC729523	NC_000011	71567323	71574704	0	0.1906473	0	0
LOC100133315	NC_000011	71576555	71639641	0.2718447	0.4192274	0.6449083	0.4420086
LOC100129216	NC_000011	71589499	71595607	0	0	0	0
OR7E128P	NC_000011	71604353	71605584	0	0	0	0
OR7E126P	NC_000011	71613950	71615368	0	0	0	0
RNF121	NC_000011	71639768	71708643	1.7538819	3.5023205	1.5603019	1.8215104
IL18BP	NC_000011	71709958	71713574	0.6740526	0.4157982	0.7024541	0.6039086
NUMA1	NC_000011	71713911	71791573	2.1356075	3.0386498	1.7529123	3.1767661
LRTOMT	NC_000011	71791382	71821828	0.329077	0.2029954	0.3293505	0.493025
C11orf59	NC_000011	71808338	71814322	36.376626	38.583579	18.633578	27.843405
C11orf51	NC_000011	71820633	71823822	4.9329648	10.604268	3.2802946	7.847191
FOLR3	NC_000011	71846771	71850934	1.6643117	0.9090152	1.3418099	1.5946682
FOLR1P	NC_000011	71869877	71870500	0	0	0	0
LOC100288543	NC_000011	71883493	71889027	0	0	0	0
FOLR1	NC_000011	71900602	71907366	0	0	0.0242691	0.0190099
FOLR2	NC_000011	71927819	71932994	0	0	0.069138	0
INPPL1	NC_000011	71935882	71950191	4.7413417	5.5641281	3.0330032	3.8362236
PHOX2A	NC_000011	71950121	71955220	0.0776014	0.0797823	0.0230121	0.0180252
LOC220077	NC_000011	71960891	71962332	0	0	0	0

LOC401703	NC_000011	71991195	71992226	0	0	0	0
CLPB	NC_000011	72003469	72145568	2.8067067	3.6541134	2.6976818	3.1941866
ART2P	NC_000011	72231995	72232684	0	0	0	0
PDE2A	NC_000011	72287185	72385773	0.4278558	0.3538171	0.1985908	0.3629615
ARAP1	NC_000011	72396114	72463434	5.9625125	5.2898032	3.0953971	4.5129116
RPS12P20	NC_000011	72419231	72419610	0	0	0	0
STARD10	NC_000011	72465774	72504750	1.2821919	4.4092455	3.0286847	2.3107303
ATG16L2	NC_000011	72525451	72540680	0.8400361	0.484484	0.6744102	0.7138666
FCHSD2	NC_000011	72547790	72853143	2.1533655	1.9824343	2.0898476	2.1348746
RPL15P16	NC_000011	72651543	72652157	0	0	0	0
P2RY2	NC_000011	72929344	72947397	0.4609954	0.0631936	1.203002	1.1136319
OR8R1P	NC_000011	72960260	72961013	0	0	0	0
P2RY6	NC_000011	72975570	73009664	0.4130185	0.2516305	1.4969482	0.9380401
LOC100287837	NC_000011	73018280	73020498	0.3934928	0.455121	0.0875155	0.3256143
ARHGEF17	NC_000011	73019663	73080425	2.7168816	2.417338	2.3119257	2.9476396
RELT	NC_000011	73087405	73108519	0.7600161	0.5085148	0.4722187	1.0508125
FAM168A	NC_000011	73117028	73309228	4.0292097	7.568923	3.6066232	7.5045684
PLEKHB1	NC_000011	73357223	73373864	0.2279473	0.1093651	0.0540769	0.0105895
RAB6A	NC_000011	73386683	73472201	6.4534114	6.3261906	5.3405895	5.2900686
LOC268276	NC_000011	73402374	73403574	0	0	0	0
MRPL48	NC_000011	73498917	73575656	1.7489602	3.7345465	4.1890249	6.3437248
CHCHD8	NC_000011	73583712	73587890	10.112935	11.005504	7.5132409	6.8971574
PAAF1	NC_000011	73588033	73638779	0.471369	0.4846169	0.3946757	0.2318603
DNAJB13	NC_000011	73661364	73681332	0	0.0240978	0	0
UCP2	NC_000011	73685716	73693889	2.4564018	4.8038246	1.0213823	0.3162958
LOC100287870	NC_000011	73692193	73694291	0	0	0.154231	0.1208082
LOC100037267	NC_000011	73700358	73703225	0	0	0	0
UCP3	NC_000011	73711326	73720282	0.0630534	0.0518604	0	0.0263629
C2CD3	NC_000011	73745480	73882064	0.5806595	0.8055621	0.8962193	1.2675053
PPME1	NC_000011	73882368	73965748	8.5280711	12.249067	6.5507947	11.098865
P4HA3	NC_000011	73977702	74022699	0.6585456	0.5774873	0.6892475	0.32393
PGM2L1	NC_000011	74041361	74109502	0.7289379	1.3606577	0.4369214	0.558388
LOC100129920	NC_000011	74065488	74066770	0	0	0	0
KCNE3	NC_000011	74165886	74178600	0.0858923	0.0294354	0.0636768	0.0698287
CYCSP27	NC_000011	74193295	74193592	0	0	0	0
LOC387787	NC_000011	74202923	74204755	0.5035735	0.1411981	0.2036332	0.1595046
LOC100287896	NC_000011	74204485	74209287	0.3717426	0.5095872	0.4593229	0.5036987
POLD3	NC_000011	74303629	74353765	3.5747979	2.9112338	3.7957695	4.2856973
CHRDL2	NC_000011	74407474	74442186	0	0.0269269	0.0699003	0
RPL36AP38	NC_000011	74449525	74449841	0	0	0	0
RPS12P22	NC_000011	74456716	74457239	0	0	0	0
RNF169	NC_000011	74459913	74553458	4.0111258	2.795445	5.3726043	6.2009213
XRRA1	NC_000011	74551955	74660232	0.7645463	0.7589294	0.8130668	1.6289177
RPL31P46	NC_000011	74630620	74631051	0	0	0	0
SPCS2	NC_000011	74660292	74690076	2.9699137	2.4694031	2.0934812	2.0921723
NEU3	NC_000011	74699950	74718743	0.4477986	0.6248069	0.4552846	0.5683656
OR2AT2P	NC_000011	74782165	74783136	0	0	0	0
LOC100129795	NC_000011	74790302	74791162	0	0	0	0
OR2AT4	NC_000011	74799796	74800758	0	0	0	0
OR2AT1P	NC_000011	74842148	74842917	0	0	0	0
SLCO2B1	NC_000011	74862040	74917445	0.049469	0.0406874	0.0704143	0.0344719
LOC441617	NC_000011	74951422	74953243	0	0.078037	0	0.0528927
ARRB1	NC_000011	74976482	75062873	3.2901353	3.4646075	5.3927677	6.3917623
RPS3	NC_000011	75110562	75116733	169.83562	186.15991	173.82378	225.40784
SNORD15A	NC_000011	75111435	75111582	0	0	0	0
SNORD15B	NC_000011	75115465	75115610	0	0	0	0
KLHL35	NC_000011	75133438	75141233	2.8770386	0.2112784	0.3917592	0.2659475
GDPD5	NC_000011	75145685	75236599	0.983089	0.8541286	0.4064964	0.2412167
SERPINH1	NC_000011	75273170	75283846	75.772813	152.48373	96.661813	45.006343
MAP6	NC_000011	75297963	75379479	0.1335096	0.1291877	0.0349335	0.0437811
MOGAT2	NC_000011	75428934	75442331	0	0.0899172	0	0.060945
DGAT2	NC_000011	75479778	75512579	3.6205313	3.6854325	6.3142887	4.8210453
UVRAG	NC_000011	75526212	75855282	1.6333838	1.4343937	1.4001258	1.2449138
WNT11	NC_000011	75897370	75917574	0.2508721	1.3834046	0.4463656	0.1748177
PRKRIR	NC_000011	76061004	76091880	0.6888438	0.8073523	0.6005582	1.5840454
C11orf30	NC_000011	76156069	76262589	0.8851849	1.2052187	1.5536866	2.2506035

LRRC32	NC_000011	76368568	76381791	8.362619	5.2439576	0.0263816	0.5028376
GUCY2E	NC_000011	76391210	76432833	0	0	0	0
TSKU	NC_000011	76494285	76509198	7.346328	8.3530939	4.0396009	2.1245303
ACER3	NC_000011	76571917	76734850	0.7513374	0.7415557	0.5258164	0.6492167
LOC100288668	NC_000011	76745424	76751582	0	0	0	0
B3GNT6	NC_000011	76745435	76753017	0.0175093	0.0180014	0	0
CAPN5	NC_000011	76777992	76837201	1.8607719	1.9130691	1.0145965	0.9690094
OMP	NC_000011	76813886	76814377	0	0	0	0
MYO7A	NC_000011	76839310	76926286	0.0227593	0	0.0101237	0.0277543
GDPD4	NC_000011	76927603	76998463	0	0	0.0154353	0.0241807
LOC387791	NC_000011	77023780	77025083	0	0	0	0
PAK1	NC_000011	77033060	77185108	2.0843513	2.9091929	3.0118274	4.2253353
DKFZp434E1119	NC_000011	77183485	77184975	0	0	0	0
RNU7-59P	NC_000011	77277882	77278139	0	0	0	0
AQP11	NC_000011	77300680	77320694	0.3039297	0.2499773	0.189269	0.1906113
LOC100288759	NC_000011	77301025	77314676	0.558664	0.1276367	0	0.1297664
CLNS1A	NC_000011	77327196	77348851	9.8907528	12.241971	12.757254	11.308677
RSF1	NC_000011	77377274	77531880	0.3509695	0.4576426	0.3122322	0.5547553
FTHL16	NC_000011	77445360	77446277	0	0	0	0
RPS20P27	NC_000011	77524322	77524842	0	0	0	0
C11orf67	NC_000011	77532208	77583398	1.395182	2.5998386	0.9308944	1.5798548
RPL21P95	NC_000011	77579742	77580281	0	0	0	0
INTS4	NC_000011	77589766	77705717	3.4890625	3.7162595	2.0610339	3.1607131
KCTD14	NC_000011	77726761	77734320	1.0783229	0.4596755	1.3102715	1.0813093
THRSP	NC_000011	77774907	77779397	0	0.0384867	0	0.1304296
NDUFC2	NC_000011	77779396	77791265	12.427109	18.204963	8.9677669	7.4332229
ALG8	NC_000011	77811988	77850699	9.4552994	8.1138295	7.2452746	7.3794887
LOC100289388	NC_000011	77850839	77885516	0.251612	0.4311393	0.1865342	0.4383332
KCTD21	NC_000011	77882298	77899664	0.2341824	0.1203821	0.0925934	0.1994515
USP35	NC_000011	77899958	77925753	1.4031601	1.356471	1.183081	0.9266999
GAB2	NC_000011	77926342	78128766	0.4446459	0.435023	0.5614533	0.8046027
LOC729944	NC_000011	78095143	78096785	0	0	0	0
NARS2	NC_000011	78147238	78285719	1.5082329	1.5936948	1.0622313	1.2845516
ODZ4	NC_000011	78364328	79151695	0.1978773	0.2968204	0.0057717	0.0293861
LOC646112	NC_000011	79698556	79700678	0	0	0	0
LOC729790	NC_000011	80364466	80365127	0	0	0	0
LOC100288816	NC_000011	80653260	80668682	0	0	0	0.0887678
LOC100288855	NC_000011	81259734	81266942	0	0	0	0
RPS28P7	NC_000011	82400572	82400946	0	0	0	0
FAM181B	NC_000011	82443046	82444906	0.307	0.1456746	0.8823739	0.5759649
RBMXP3	NC_000011	82516229	82518245	0	0	0	0
PRCP	NC_000011	82535409	82611557	18.149066	14.173578	17.262479	9.731659
LOC100129629	NC_000011	82539313	82548284	0	0	0	0
C11orf82	NC_000011	82612737	82645701	2.5888159	4.5880481	3.1804471	3.7711982
RPL7AP54	NC_000011	82668435	82669315	0	0	0	0
RAB30	NC_000011	82692478	82782884	3.1548356	2.0871234	1.9036268	2.1984151
LOC100127960	NC_000011	82843080	82848743	0	0	0	0
PCF11	NC_000011	82868137	82896836	0.7358151	0.9016813	0.7074987	1.2223019
ANKRD42	NC_000011	82905291	82960013	0.2058465	0.3174478	0.1678662	0.1314885
RPL32P24	NC_000011	82924047	82924446	0	0	0	0
CCDC90B	NC_000011	82972506	82997377	7.0920272	5.1958042	5.6137547	4.5139592
CYCSP28	NC_000011	83039969	83040294	0	0	0	0
DLG2	NC_000011	83166055	85338314	0.0397947	0.0409131	0.044253	0.0485284
RPL9P22	NC_000011	84351008	84351702	0	0	0	0
TMEM126B	NC_000011	85339662	85347538	7.4555036	8.6332572	10.751831	8.996058
TMEM126A	NC_000011	85359045	85367587	7.5063096	5.7879564	4.7390989	3.5011913
CREBZF	NC_000011	85368870	85376173	0.7581431	0.630984	0.4228789	1.0482228
CCDC89	NC_000011	85394893	85397320	0.0181006	0.0186093	0	0.0882925
SYTL2	NC_000011	85405264	85469044	0.8534838	0.1080943	0.3796414	0.8576345
CCDC83	NC_000011	85566144	85631051	0.0187413	0	0.0333455	0.0783579
LOC100131457	NC_000011	85634842	85646783	0	0	0	0
PICALM	NC_000011	85668485	85780108	41.894364	37.355345	32.475308	30.395567
LOC100130431	NC_000011	85742493	85780311	1.5212849	1.853678	0.9523766	0.7852533
FNTAL1	NC_000011	85906173	85907820	0	0	0	0
EED	NC_000011	85955815	85989781	2.0657863	2.5937227	1.9678891	2.0764789
LOC100289518	NC_000011	85995413	85996462	0.1255664	0.1721272	0.0744716	0.3499986

LOC100130043	NC_000011	85999887	86013247	0	0	0	0
C11orf73	NC_000011	86013253	86056985	6.8415453	7.5504366	6.7397738	7.2185289
CCDC81	NC_000011	86085778	86134150	0.2451296	0.1620122	0.109037	0.0610057
LOC100129472	NC_000011	86143141	86145175	0	0	0	0
ME3	NC_000011	86152150	86383427	3.4546935	1.9990938	3.3253084	4.2227603
PRSS23	NC_000011	86511491	86522275	31.68522	32.979372	12.840766	19.271304
LOC100131078	NC_000011	86532283	86534089	0	0	0	0
OR7E13P	NC_000011	86543648	86544301	0	0	0	0
OR7E2P	NC_000011	86568058	86568894	0	0	0	0
FZD4	NC_000011	86656721	86666433	3.9049221	5.018338	0.7307956	1.2858882
LOC100131842	NC_000011	86735626	86736821	0	0	0	0
TMEM135	NC_000011	86749065	87034572	2.8467996	2.0977559	2.7391122	2.2391835
LOC100130923	NC_000011	87040395	87041092	0	0	0	0
LOC100289080	NC_000011	87525897	87599828	0	0	0	0
RAB38	NC_000011	87846431	87908599	0.0624265	0.6097192	0.0277682	0
CTSC	NC_000011	88026760	88070941	0.5015737	2.0863909	1.318126	2.6073129
GAPDHL15	NC_000011	88141346	88142390	0	0	0	0
GRM5	NC_000011	88237744	88799134	0.0218485	0.0280782	0.0097185	0.0380622
TYR	NC_000011	88911040	89028927	0.0212824	0	0	0
NOX4	NC_000011	89057521	89231363	0.02819	0.2801622	0.0083595	0.0458358
FOLH1B	NC_000011	89392465	89431886	0.0222635	0.0228893	0.0198063	0.0155141
FOLH2	NC_000011	89431610	89431764	0	0	0	0
LOC100289112	NC_000011	89443467	89451040	0	0.033395	0	0
LOC100130203	NC_000011	89464173	89468035	0	0	0	0
UBTFL2	NC_000011	89485953	89487143	0	0	0	0
LOC727828	NC_000011	89510149	89515728	0	0	0	0
TRIM49	NC_000011	89530823	89541743	0	0	0	0.0141585
LOC642414	NC_000011	89553250	89560102	0	0	0	0
LOC642425	NC_000011	89575226	89584143	0.0262534	0.0269913	0	0
LOC399937	NC_000011	89586010	89596232	0	0	0	0
LOC642446	NC_000011	89603606	89609185	0.0286681	0	0	0
LOC440059	NC_000011	89628668	89643804	0	0	0	0
LOC399939	NC_000011	89644577	89653576	0	0	0	0
LOC729384	NC_000011	89657232	89666231	0	0	0	0.0185493
LOC100128800	NC_000011	89667004	89682216	0	0	0	0
TRIM64	NC_000011	89701672	89707240	0	0.0294738	0	0.0199771
LOC399940	NC_000011	89714607	89724838	0	0	0	0
TRIM53	NC_000011	89726704	89735615	0	0	0	0.0182944
LOC642579	NC_000011	89750767	89757677	0	0	0	0
LOC653111	NC_000011	89764274	89775193	0	0	0.0180756	0.0141585
LOC440061	NC_000011	89790587	89796165	0	0	0	0
UBTFL1	NC_000011	89819118	89820299	0	0	0	0.0259094
NAALAD2	NC_000011	89867818	89925779	0.1237591	0.1555123	0.2568989	0.2203918
CHORDC1	NC_000011	89933597	89956532	1.0386584	1.7753006	1.0857226	2.696075
LOC399942	NC_000011	90015731	90017341	0	0	0	0
LOC100130038	NC_000011	90848101	90849444	0	0	0	0
RPL7AP57	NC_000011	91894247	91895113	0	0	0	0
NDUFB11P	NC_000011	92069111	92069679	0	0	0	0
FAT3	NC_000011	92085262	92629636	0.4799051	0.7377172	0.1190497	0.9727032
RPS3AP42	NC_000011	92231267	92232124	0	0	0	0
LOC642791	NC_000011	92647769	92649134	0	0	0	0
LOC100128354	NC_000011	92670609	92670836	0	0	0.1714806	0
MTNR1B	NC_000011	92702789	92715948	0.026443	0	0.0470488	0.0921326
RPL26P31	NC_000011	92785544	92786040	0	0	0	0
SLC36A4	NC_000011	92880850	92931095	2.42473	2.7311671	3.5211601	5.0565215
LOC100128230	NC_000011	92884996	92931273	0	0	0	0
CCDC67	NC_000011	93063883	93171636	0.1648471	0.1355841	0.175983	0.0918976
C11orf75	NC_000011	93211638	93276546	3.7374009	1.1110673	3.9257802	4.6439365
LOC729466	NC_000011	93268635	93283415	0	0	0	0
LOC642897	NC_000011	93342920	93343561	0	0	0	0
KIAA1731	NC_000011	93394816	93463522	1.4642126	1.6626413	1.1956739	2.3870958
SCARNA9	NC_000011	93454680	93455032	0	0	0	0
SNORA25	NC_000011	93463679	93463812	0	0	0	0
SNORA32	NC_000011	93464145	93464265	0	0	0	0
SNORD6	NC_000011	93464669	93464739	0	0	0	0
SNORA1	NC_000011	93465170	93465299	0	0	0	0

SNORA8	NC_000011	93465527	93465665	0	0	0	0
SNORD5	NC_000011	93466394	93466466	0	0	0	0
SNORA18	NC_000011	93466632	93466763	0	0	0	0
SNORA40	NC_000011	93468276	93468402	0	0	0	0
TAF1D	NC_000011	93469095	93474662	2.7662144	2.7728602	3.5990678	4.1202629
C11orf54	NC_000011	93474793	93496247	1.7652846	0.6427764	1.0633229	1.4351407
MED17	NC_000011	93517405	93546496	3.6948185	4.6901844	1.8000309	2.3907898
C11orf90	NC_000011	93553735	93583668	0.1457653	0.149862	0.0648384	0
HPRTP4	NC_000011	93723942	93724588	0	0	0	0
HPRTP3	NC_000011	93731815	93732386	0	0	0	0
HEPHL1	NC_000011	93754378	93847374	0.0295401	0.0379629	0.0065699	0.0257309
LOC100129932	NC_000011	93861921	93913179	0.3329411	0.8557462	0.6664358	0.3770108
PANX1	NC_000011	93862094	93915138	8.7293346	6.9186574	6.4527221	5.8838697
LOC729494	NC_000011	93921250	93922202	0	0	0	0
FOLR4	NC_000011	94038803	94040858	0	0.1234519	0.053412	0.0418373
GPR83	NC_000011	94110477	94134585	0.0410539	0.0316558	0	0.042912
MRE11A	NC_000011	94150466	94227040	0.6448197	0.5854557	0.8567492	1.6339493
ANKRD49	NC_000011	94227153	94232744	1.2688466	0.735268	1.1082775	1.8487459
LOC643037	NC_000011	94245605	94265289	0	0	0	0
FUT4	NC_000011	94277017	94283064	0.9737207	0.9936165	0.4913054	0.6127001
PIWIL4	NC_000011	94300474	94354587	0.1373812	0.2118634	0.061109	0.0765861
AMOTL1	NC_000011	94501508	94609918	1.8216125	1.2200874	1.6292463	3.4337707
LOC728162	NC_000011	94646212	94646931	0	0	0	0
CWC15	NC_000011	94695787	94706776	12.696156	16.208649	15.539731	15.516605
KDM4D	NC_000011	94706845	94732678	1.0344237	1.2002316	0.8676662	0.3192237
LOC390245	NC_000011	94758422	94760760	0	0.0193174	0.0167155	0.0130932
LOC643118	NC_000011	94770754	94771620	0	0	0	0
LOC100129053	NC_000011	94782586	94784502	0	0	0	0
SFRS2B	NC_000011	94800056	94804388	2.4545285	2.1064037	1.5429688	1.9436514
ENDOD1	NC_000011	94823017	94865815	8.7896464	3.5855216	5.8099823	6.2237656
SESN3	NC_000011	94906133	94964246	0.7298901	2.3421693	2.6760286	2.2964806
LOC100129203	NC_000011	94927211	94964841	0	0	0	0.064746
FAM76B	NC_000011	95502106	95522954	0.6562758	0.5489252	0.7619622	0.7053566
CEP57	NC_000011	95523642	95565854	4.1888593	5.4225805	7.3168655	6.7688933
MTMR2	NC_000011	95566044	95657371	5.4419088	5.4816364	4.0983458	6.3372843
RPL32P25	NC_000011	95696383	95696788	0	0	0	0
MAML2	NC_000011	95711440	96076344	2.9228651	2.1809232	2.7875383	3.7632266
CCDC82	NC_000011	96085929	96123083	2.350345	1.2082009	1.9047532	2.1762734
LOC100287213	NC_000011	96121080	96123092	0.0218322	0	0.0194225	0
JRKL	NC_000011	96123158	96126727	1.6495975	1.4048621	1.6975134	1.1066132
LOC100131233	NC_000011	96513671	96515157	0	0	0	0
LOC100289416	NC_000011	98000882	98001703	0	0	0	0
LOC643381	NC_000011	98435804	98437722	0	0	0	0
CNTN5	NC_000011	98891871	100227473	0.0105543	0	0	0.022064
LOC440063	NC_000011	100537196	100537693	0	0	0	0
LOC100128386	NC_000011	100554893	100558680	0.0236408	0.0243052	0.042063	0.0823692
FLJ32810	NC_000011	100558407	100861656	1.6092156	1.2360779	1.2012299	0.9795836
TMEM133	NC_000011	100862811	100864666	0.5446171	0.1217225	0.252786	0.4290123
PGR	NC_000011	100900355	101000544	0.0438235	0.0138631	0.0119959	0.0211417
TRPC6	NC_000011	101322295	101454659	0.2191694	0.0685785	0.0423868	0.0199208
LOC100127938	NC_000011	101464751	101465665	0	0	0	0
LOC100129471	NC_000011	101636663	101643045	0	0	0	0
ANGPTL5	NC_000011	101761405	101787253	0.5196581	0	0.0165108	0.0517312
KIAA1377	NC_000011	101785746	101871793	0.2932794	0.0898151	0.1276791	0.4304789
C11orf70	NC_000011	101918199	101954150	1.8122982	1.325762	1.3022187	0.6314408
YAP1	NC_000011	101981210	102104154	27.071457	19.643862	19.207224	18.807546
RPS6P17	NC_000011	102165750	102166570	0	0	0	0
BIRC3	NC_000011	102188194	102208465	1.2438972	0.2073822	1.4879357	0.5446766
BIRC2	NC_000011	102217966	102249401	9.3109666	7.4239359	6.8940404	8.4191824
TMEM123	NC_000011	102267056	102323775	22.79432	22.327397	34.044287	22.751128
LOC727869	NC_000011	102323656	102332770	0	0.1260346	0	0
MMP7	NC_000011	102391239	102401478	0.0785491	0.0403784	0.0349397	0
MMP20	NC_000011	102447566	102496063	0	0.0230999	0	0.0313138
MMP27	NC_000011	102562415	102576468	0.0266838	0.0548675	0	0
MMP8	NC_000011	102582526	102595685	0.0287619	0.0147851	0	0.0100212
LOC100128088	NC_000011	102622073	102638486	0	0	0	0

MMP10	NC_000011	102641234	102651344	0.0756424	0.0518456	0.112156	0
MMP1	NC_000011	102660651	102668894	28.912724	55.328484	0	0.031044
LOC100289609	NC_000011	102667995	102668893	0.3192366	0.2188058	0.0946672	0
LOC120321	NC_000011	102677680	102679057	0	0	0	0
LOC100289645	NC_000011	102702424	102702920	0	0	0	0
LOC100288077	NC_000011	102703553	102707497	0	0	0.0741889	0.2324469
MMP3	NC_000011	102706528	102714342	1.0337932	18.538048	0.1711053	0.619869
MMP12	NC_000011	102733464	102745712	0.0240944	0	0	0.0503699
LOC100288111	NC_000011	102751224	102751793	0	0	0	0
MMP13	NC_000011	102813724	102826462	0.0161872	0.0832107	0.0288012	0.0225598
RPL21P96	NC_000011	102915932	102916468	0	0	0	0
DCUN1D5	NC_000011	102932821	102962869	4.4335286	8.139524	4.8832828	8.753468
DYNC2H1	NC_000011	102980160	103350591	0.5806723	0.8014867	0.4537932	1.0305912
LOC100190922	NC_000011	103497972	103499959	0	0	0	0
PDGFD	NC_000011	103777914	104035027	0.1761011	0.0452626	0.0195831	0.0383482
DDI1	NC_000011	103907308	103909922	0.0168062	0	0	0
CASP12	NC_000011	104757417	104769397	0	0	0	0
LOC643733	NC_000011	104774180	104780632	0	0	0	0
CASP4	NC_000011	104813594	104839325	19.091757	9.9734872	23.491626	23.454596
LOC100289680	NC_000011	104846072	104846803	0	0	0	0
CASP5	NC_000011	104864967	104893895	0.0590702	0.0910956	0.0525505	0.0205812
CASP1	NC_000011	104896235	104905857	5.3437423	1.3984546	4.177706	2.7307371
CARD16	NC_000011	104912053	104916051	0.4058544	0	0.4126392	0.0808044
LOC440067	NC_000011	104934074	104941595	0	0	0	0
CARD17	NC_000011	104963196	104972158	0.0943095	0	0.0839004	0
CARD18	NC_000011	105008669	105009805	0	0	0	0.0722285
OR2AL1P	NC_000011	105064827	105065763	0	0	0	0
GRIA4	NC_000011	105480800	105852819	0.0231611	0	0.0051512	0.0080698
KIAA1826	NC_000011	105878629	105892954	2.0000908	1.3155921	1.7219383	2.2179995
KBTBD3	NC_000011	105921825	105948465	0.1350336	0.1708661	0.2494999	0.2678139
AASDHPPT	NC_000011	105948292	105969419	4.4547316	5.2590258	4.0997098	4.5600133
LOC643855	NC_000011	106180831	106182948	0	0	0	0
GUCY1A2	NC_000011	106557910	106889171	0.9521621	0.6577137	0.0794128	0.0622035
ASSP13	NC_000011	107047012	107048256	0	0	0	0
CWF19L2	NC_000011	107197071	107328572	0.2143816	0.3030594	0.2026398	0.3641373
ALKBH8	NC_000011	107373453	107436461	0.7023937	1.0887567	0.2980144	0.7680692
ELMOD1	NC_000011	107461817	107537505	0.0146935	0.0755323	0	0.020478
LOC643923	NC_000011	107462471	107463949	0	0	0	0
LOC100131459	NC_000011	107463479	107514591	0	0	0	0
SLN	NC_000011	107578101	107582787	0.0602031	0	0	0
LOC203930	NC_000011	107606687	107609489	0	0	0	0
SLC35F2	NC_000011	107661717	107729914	0.4575053	1.6961593	1.0853583	0.9660845
RPLP2P3	NC_000011	107779073	107779528	0	0	0	0
RAB39	NC_000011	107799277	107834208	0.0246623	0	0.1535819	0.0171857
CUL5	NC_000011	107879408	107978488	2.3050975	2.6316307	2.6567001	3.2029777
ACAT1	NC_000011	107992258	108018895	2.2405787	1.9442811	2.8161951	4.2256029
NPAT	NC_000011	108028117	108093365	3.5599696	4.6035632	3.2921493	3.5328463
ATM	NC_000011	108093559	108239826	1.944845	1.8743246	1.5867376	2.2060002
C11orf65	NC_000011	108253727	108338258	0.1701771	0.3936598	0.1513943	0.1778793
KDELC2	NC_000011	108342833	108369159	22.336019	23.865137	11.191463	17.951535
EXPH5	NC_000011	108376158	108464374	0.6167156	0.1531929	0.4344992	0.4326363
DDX10	NC_000011	108535816	108811650	2.4224519	2.4480828	1.6530446	1.5346009
RPS2P39	NC_000011	108561016	108561683	0	0	0	0
CYCSP29	NC_000011	108693060	108693377	0	0	0	0
SKCG-1	NC_000011	108829514	108830144	0	0	0	0
C11orf87	NC_000011	109292875	109296323	2.9887805	0.5217929	0.0334453	0.1178888
TFAMP2	NC_000011	109776655	109778599	0	0	0	0
RPSAP50	NC_000011	109853124	109853737	0	0	0	0
LOC390250	NC_000011	109920253	109920885	0	0	0	0
ZC3H12C	NC_000011	109964087	110042566	2.4801035	1.3390335	2.375065	1.3005228
RDX	NC_000011	110100166	110167437	10.590643	10.224128	12.565209	14.555343
FDX1	NC_000011	110300594	110335605	1.0376097	0.6456777	0.7166065	0.8942959
ARHGAP20	NC_000011	110447766	110583451	1.8178619	1.452819	0.8907347	0.6036896
LOC120364	NC_000011	110658734	110659699	0	0	0	0.0317028
RPS17P15	NC_000011	110976292	110976619	0	0	0	0
C11orf53	NC_000011	111126707	111156973	0	0.0859	0	0.0291111

C11orf92	NC_000011	111164170	111170539	0.0080743	0.0332048	0.0502816	0.0562647
LOC120376	NC_000011	111169976	111179353	0	0.0319543	0	0.0433167
POU2AF1	NC_000011	111222980	111250157	0	0.0298044	0	0.0101006
LOC644277	NC_000011	111299223	111327330	0	0	0	0
BTG4	NC_000011	111338255	111383064	0.0879844	0.2261432	0.0391367	0.0613111
LOC728196	NC_000011	111381327	111384617	0	0	0	0
C11orf88	NC_000011	111385510	111407756	0.0552808	0.0568345	0.1967173	0.0385219
LAYN	NC_000011	111411233	111431788	18.315309	12.400285	18.186235	25.021682
SIK2	NC_000011	111473170	111597635	2.746281	2.7121176	4.3151159	5.3853641
PPP2R1B	NC_000011	111597632	111637169	2.7892956	2.2987642	3.585773	3.7102116
ALG9	NC_000011	111652919	111742305	1.6023136	1.5253211	1.4665253	1.8425471
GNG5P3	NC_000011	111734929	111735421	0	0	0	0
FDXACB1	NC_000011	111744780	111750153	0.2459249	0.3123277	0.2445207	0.2721763
C11orf1	NC_000011	111749948	111754798	1.4292108	2.9846759	2.1058648	0.7780711
RPL37AP8	NC_000011	111759867	111760551	0	0	0	0
CRYAB	NC_000011	111779350	111782473	15.137018	7.846611	8.2042649	17.639221
HSPB2	NC_000011	111783460	111784817	10.555937	6.3933708	4.4164908	3.7871431
C11orf52	NC_000011	111789601	111797595	1.0794303	0.0396346	0.2743689	0.1343196
DIXDC1	NC_000011	111807927	111893308	1.3565969	2.2513823	1.3784005	1.1036857
DLAT	NC_000011	111895538	111934978	5.9078951	6.8789168	4.7492415	5.9166528
PPIHP1	NC_000011	111900410	111901118	0	0	0	0
PIH1D2	NC_000011	111934734	111944895	0.3018423	0.3723907	0.4564963	0.5679064
C11orf57	NC_000011	111944968	111955874	3.1601375	2.971162	2.9994657	3.0289241
TIMM8B	NC_000011	111955869	111957459	19.426392	21.718592	10.577119	16.052171
SDHD	NC_000011	111957571	111966518	7.9847337	8.6394637	9.3089442	6.1698473
IL18	NC_000011	112013976	112034840	0.1535309	0.0394615	0	0.1337331
TEX12	NC_000011	112038095	112043279	0.0384163	0	0	0.02677
BCO2	NC_000011	112046208	112089652	0.1608754	0.0751804	0.1040867	0.2955479
MRPS36P4	NC_000011	112079320	112079621	0	0	0	0
RPS12P21	NC_000011	112089049	112089443	0	0	0	0
PTS	NC_000011	112097088	112104696	1.4101037	0.8215164	1.4635452	1.8669712
RPS6P16	NC_000011	112106087	112106766	0	0	0	0
C11orf34	NC_000011	112118876	112131583	0.0570386	0.0293208	0.0253716	0.0397468
LOC100132686	NC_000011	112131195	112131624	0	0	0	0
LOC399950	NC_000011	112138013	112139094	0	0	0	0
LOC100287058	NC_000011	112183598	112183985	0	0	0	0
RPL23AP62	NC_000011	112332206	112332643	0	0	0	0
NCAM1	NC_000011	112831995	113149158	0.0521765	0.03755	0.0464176	0.025451
LOC100288346	NC_000011	113140254	113144554	0	0	0	0
TTC12	NC_000011	113185251	113237114	0.4746029	0.6636007	0.6755519	0.8863356
ANKK1	NC_000011	113258513	113271140	0.017282	0.0177678	0.0153746	0.0361284
DRD2	NC_000011	113280317	113346001	0.0325663	0.0334816	0	0.0567338
TMPRSS5	NC_000011	113558268	113577068	0.0795443	0.0817799	0.0353824	0.0831445
LOC100132520	NC_000011	113577499	113583109	0	0	0	0
ZW10	NC_000011	113603909	113644425	4.5411141	6.0160979	3.6738697	5.0014971
RPS29P19	NC_000011	113621838	113622008	0	0	0	0
LOC100288385	NC_000011	113640382	113640721	0	0	0	0
LOC644672	NC_000011	113650518	113651207	0.1910793	0	0.0566631	0
LOC390251	NC_000011	113659989	113660982	0	0	0	0
LOC100127952	NC_000011	113660773	113662175	0	0	0	0
USP28	NC_000011	113668597	113746256	3.4251536	3.0922147	1.8654063	2.426453
LOC100288453	NC_000011	113689853	113692658	0	0	0	0
HTR3B	NC_000011	113775589	113817283	0.0241872	0.0497341	0	0
HTR3A	NC_000011	113845910	113861035	0.0197876	0	0	0.0137888
ZBTB16	NC_000011	113930431	114121398	0.036231	0	0.0322321	0.0504944
NNMT	NC_000011	114166535	114183238	54.69856	44.982969	47.868502	54.437759
C11orf71	NC_000011	114262170	114271139	0.5434094	0.5586819	0.60429	0.284002
RBM7	NC_000011	114271384	114279635	5.2957963	8.8151238	7.1179188	4.5529944
REXO2	NC_000011	114310108	114321001	30.245166	34.675634	26.199747	25.613055
FAM55A	NC_000011	114392437	114430580	0	0	0.0207085	0.0324416
FAM55D	NC_000011	114441313	114466484	0	0.0208892	0.0361512	0.0424756
LOC100132172	NC_000011	114453975	114455419	0	0	0	0
FAM55B	NC_000011	114549200	114577652	0.0247178	0	0	0.0172243
CADM1	NC_000011	115044345	115375241	0.1836703	0.0209814	0.0090777	0.028442
LOC441623	NC_000011	115409584	115410315	0	0	0	0
LOC100287201	NC_000011	115529892	115532135	0	0	0	0.0136475

LOC283143	NC_000011	115624966	115630912	0	0	0	0
LOC100288599	NC_000011	115628497	115630910	0	0.0697275	0	0
RPL15P15	NC_000011	115820908	115821519	0	0	0	0
BUD13	NC_000011	116618889	116643704	2.9258741	2.6784506	2.7455655	3.2119115
ZNF259	NC_000011	116649276	116658739	10.62865	9.2501453	5.1455739	5.4256674
APOA5	NC_000011	116660086	116662584	0.0235395	0	0	0
APOA4	NC_000011	116691418	116694011	0.0301015	0.0618951	0.0267792	0.0419519
LOC100288634	NC_000011	116695684	116708338	0	0	0.0299369	0.0468987
APOC3	NC_000011	116700624	116703787	0	0	0	0.0574576
APOA1	NC_000011	116706469	116708338	0.0979894	0.1007434	0.043587	0.0341414
KIAA0999	NC_000011	116714118	116968993	3.8886473	2.389829	2.9569587	4.6121501
RPS27P19	NC_000011	116906456	116906801	0	0	0	0
LOC100132676	NC_000011	116988447	116997100	0	0	0	0
LOC653303	NC_000011	117006239	117009569	0	0	0	0
PAFAH1B2	NC_000011	117015040	117038814	42.841223	42.641598	28.986692	32.707679
SIDT2	NC_000011	117049939	117068161	2.4783259	1.7870445	1.5862498	1.1018392
TAGLN	NC_000011	117070040	117075508	244.63669	143.90783	2.6366374	3.3044185
PCSK7	NC_000011	117075788	117102811	10.091442	7.833173	4.5112576	4.5884577
RNF214	NC_000011	117103404	117156404	1.2699014	0.9750624	1.172641	1.4225894
BACE1	NC_000011	117156414	117186972	5.068922	5.6450214	3.2229527	4.0885656
CEP164	NC_000011	117198571	117283982	1.1635193	1.043682	0.6391215	1.2080176
DSCAML1	NC_000011	117298489	117667976	0.0318512	0.0196478	0.0056671	0.0488293
FXYD2	NC_000011	117690790	117698807	0	0	0	0.0324416
LOC100287298	NC_000011	117690857	117694520	0	0	0	0
FXYD6	NC_000011	117707693	117747367	0	0	0	0.0548505
TMPRSS13	NC_000011	117771360	117800115	0.0129603	0	0	0.0180624
IL10RA	NC_000011	117857106	117872198	0.0961668	0.0247174	0.0106941	0.0083766
TMPRSS4	NC_000011	117947727	117990558	0	0.0127817	0.0221203	0.0433167
SCN4B	NC_000011	118004092	118023630	0.0455989	0.0187522	0.0486793	0.0254201
SCN2B	NC_000011	118033519	118047336	0.0267923	0.036727	0.007945	0.0124466
AMICA1	NC_000011	118064442	118095809	0.0364413	0.0187328	0	0.0253938
MPZL3	NC_000011	118100336	118123011	0.0447083	0.229824	0	0
MPZL2	NC_000011	118124134	118135009	0	0.0307057	0	0.010406
CD3E	NC_000011	118175295	118186890	0	0.0298635	0	0.0202412
CD3D	NC_000011	118209789	118213459	0	0	0	0
CD3G	NC_000011	118215059	118224497	0	0	0	0.0233599
LOC100132158	NC_000011	118225905	118230908	0	0	0	0
UBE4A	NC_000011	118230302	118269926	6.2598592	6.4357933	3.2634525	5.4676563
ATP5L	NC_000011	118272104	118280562	11.726098	12.325816	12.711092	13.824683
MGC13053	NC_000011	118303363	118305915	0	0	0	0
MLL	NC_000011	118307205	118395936	1.020824	1.1460214	1.4509576	2.072729
TTC36	NC_000011	118398210	118401740	0	0	0.2303244	0
TMEM25	NC_000011	118401803	118417313	1.5914808	0.4503329	1.6496315	1.3124948
C11orf60	NC_000011	118415258	118436750	1.0630688	0.9439083	0.7522896	1.582594
RPL5P30	NC_000011	118431332	118432353	0	0	0	0
ARCN1	NC_000011	118443102	118473748	11.282527	10.463288	8.2946031	10.309969
PHLDB1	NC_000011	118477213	118528741	7.0798754	5.9165034	1.0373923	1.9734157
LOC100287397	NC_000011	118498906	118515020	0	0.0225354	0.0585001	0.0152743
TREH	NC_000011	118528942	118550381	0	0.0975357	0.0421992	0.0495816
LOC768086	NC_000011	118558685	118561309	0	0	0	0
DDX6	NC_000011	118618472	118661972	7.9046121	7.17623	5.5441064	14.847681
LOC100288660	NC_000011	118692606	118692796	0	0	0	0
LOC649925	NC_000011	118704497	118705316	0	0	0	0
CXCR5	NC_000011	118754541	118766971	0.0434844	0.0298044	0.02579	0.0303017
BCL9L	NC_000011	118766849	118781613	4.8604246	5.1896698	3.3793632	3.5729025
UPK2	NC_000011	118827026	118829269	0.0959568	0	0.0426829	0.1002998
FOXR1	NC_000011	118842417	118851995	0.0762328	0.0391877	0.0678188	0.0531221
CCDC84	NC_000011	118868852	118886455	2.4131309	1.8043289	1.3336108	1.6051309
RPL23AP64	NC_000011	118873663	118874303	0	0	0	0
RPS25	NC_000011	118886422	118889057	35.372967	33.887551	48.951424	53.967011
TRAPPC4	NC_000011	118889241	118894384	14.283175	15.061134	9.2530905	11.86714
SLC37A4	NC_000011	118895064	118901590	3.6252521	3.3348097	5.5449514	5.5547295
HYOU1	NC_000011	118914896	118927925	7.9966529	9.0347206	14.507902	11.815584
VPS11	NC_000011	118938493	118952688	10.448276	7.600684	8.7490207	6.5869152
HMBS	NC_000011	118955587	118964259	5.1332413	6.2935032	3.0525894	4.1126476
H2AFX	NC_000011	118964584	118966177	24.565793	30.443522	17.78277	24.438422

DPAGT1	NC_000011	118967213	118972785	4.0624416	2.9109755	2.8474418	5.4043903
C2CD2L	NC_000011	118978093	118987827	0.3154179	0.5269595	0.5027498	0.6135965
HINFP	NC_000011	118992288	119005765	3.0190524	3.7914768	2.7028317	2.5698267
ABCG4	NC_000011	119019750	119033375	0.0558285	0.0114795	0.0298	0.0077807
NLRX1	NC_000011	119039440	119054725	2.9463158	2.0757332	1.4306534	1.292396
PDZD3	NC_000011	119056195	119060920	0.0199402	0	0.0532181	0.0833708
CCDC153	NC_000011	119060963	119066584	0.2291955	0.2945463	0.2548733	0.1597125
CBL	NC_000011	119076990	119178859	1.7458726	2.4227673	2.792932	2.692327
MCAM	NC_000011	119179234	119187840	4.7219289	0.8271871	0.7040378	0.2573519
LOC100287473	NC_000011	119204630	119205903	0	0.1392401	0.3614567	0.1415634
RNF26	NC_000011	119205237	119208022	9.2755062	8.4982421	6.7922548	11.212267
LOC100287504	NC_000011	119209644	119211624	3.2053631	1.0323097	1.7521756	0.5382228
C1QTNF5	NC_000011	119209652	119217383	0.1570782	0.3806618	0.1097966	0.0312738
MFRP	NC_000011	119209652	119217383	0.1458583	0.1845633	0.1996302	0.0234554
MFRP-C1QTNF5	NC_000011	119209652	119217383	0	0	0	0
USP2	NC_000011	119225925	119252436	0.2269272	0.0555488	0.3845347	0.7755993
LOC100130353	NC_000011	119274236	119274680	0	0	0	0
THY1	NC_000011	119288655	119294246	40.923009	42.544405	14.679937	8.2151843
DUXAP5	NC_000011	119314947	119316264	0	0	0	0
KRT8P7	NC_000011	119473573	119479069	0	0	0	0
PVRL1	NC_000011	119508808	119599435	0.7978825	1.9140499	0.7993451	0.5810412
LOC390255	NC_000011	119692127	119707183	0	0	0	0
TRIM29	NC_000011	119981994	120008863	0.2765871	0.2394616	0.051802	0.0507202
LOC729173	NC_000011	120039691	120041489	0	0	0	0
OAF	NC_000011	120081747	120100650	37.386681	49.13877	24.483223	15.361781
POU2F3	NC_000011	120110951	120190653	0.133401	0.0457168	0.0791182	0.0103288
TMEM136	NC_000011	120196016	120201347	3.323399	2.3458651	2.8683316	2.7306607
ARHGEF12	NC_000011	120207946	120360645	5.9855817	4.6367558	5.5636153	7.0010773
LOC100287579	NC_000011	120490631	120490979	0	0	0	0
GRIK4	NC_000011	120531028	120856969	0	0.0157379	0	0.0533349
TBCEL	NC_000011	120894803	120960356	1.2941944	1.3752929	1.0062229	1.2807732
TECTA	NC_000011	120973375	121061515	0.0679472	0.0419141	0.0362686	0.0946966
RPS4P12	NC_000011	121120973	121121682	0	0	0	0
SC5DL	NC_000011	121163388	121184119	2.5237842	2.0580954	2.7532728	2.5117792
LOC283155	NC_000011	121231064	121237365	0	0	0	0
LOC645470	NC_000011	121297227	121298186	0	0	0	0
SORL1	NC_000011	121322961	121504471	0.136785	0.2564419	0.1682155	0.0953173
LOC399959	NC_000011	121959811	122073770	0	0	0	0
BLID	NC_000011	121986062	121986923	0	0	0	0.0355277
GLULL3	NC_000011	122484151	122485271	0	0	0	0
LOC100288752	NC_000011	122525884	122647867	0.2001286	0.2057532	0	0.2789151
UBASH3B	NC_000011	122526398	122685187	3.2405693	3.1418272	1.942701	3.9484487
CRTAM	NC_000011	122709255	122743347	0.0181454	0.0746216	0	0.0126445
C11orf63	NC_000011	122753473	122830430	0.77806	0.5999456	0.5732147	0.1948471
LOC100128516	NC_000011	122831035	122831796	0	0	0	0
BSX	NC_000011	122848357	122852379	0	0	0	0
LOC341056	NC_000011	122888347	122889372	0	0	0	0
RPL34P23	NC_000011	122906577	122906922	0	0	0	0
RPS26P43	NC_000011	122920442	122920674	0	0	0	0
RPL31P47	NC_000011	122921834	122922192	0	0	0	0
HSPA8	NC_000011	122928200	122932844	196.47445	238.34694	246.17114	381.20592
LOC85391	NC_000011	122928627	122928958	0	0	0	0
LOC85390	NC_000011	122929539	122929766	0	0	0	0
LOC85389	NC_000011	122929970	122930180	0	0	0	0
ASAM	NC_000011	122943020	123066007	36.498815	28.532387	13.195243	13.232543
LOC100128242	NC_000011	123296128	123306418	0	0	0	0
GRAMD1B	NC_000011	123396528	123493518	2.5627183	1.1946354	0.2548918	0.45477
SF3A3P2	NC_000011	123467446	123470177	0	0	0	0
SCN3B	NC_000011	123499895	123525315	0.007245	0.0223459	0.0064454	0.0201944
ZNF202	NC_000011	123594997	123612363	0.2601128	0.2228528	0.1542691	0.3247521
OR6X1	NC_000011	123624288	123625226	0	0	0	0.0326144
OR6M1	NC_000011	123676116	123677057	0	0	0	0.0325105
OR6M2P	NC_000011	123711666	123712611	0	0	0	0
OR6M3P	NC_000011	123732389	123733231	0	0	0	0
TMEM225	NC_000011	123753633	123756340	0	0	0	0.0278915
OR8D4	NC_000011	123777139	123778083	0.0465061	0	0	0.0324073

OR4D5	NC_000011	123810324	123811280	0	0	0	0.0320009
OR6T1	NC_000011	123813574	123814545	0	0	0	0.0315071
OR10S1	NC_000011	123847403	123848398	0	0	0	0.0307479
OR10G6	NC_000011	123864873	123865631	0	0	0	0
OR10G5P	NC_000011	123880219	123881123	0	0	0	0
OR10G4	NC_000011	123886282	123887217	0	0	0	0
OR10G9	NC_000011	123893720	123894655	0	0	0	0
OR10G8	NC_000011	123900330	123901265	0	0	0.0417709	0
OR10G7	NC_000011	123908773	123909708	0	0	0	0.0654378
OR10D5P	NC_000011	123925500	123926433	0	0	0	0
OR10D4P	NC_000011	123964143	123965439	0	0	0	0
OR10N1P	NC_000011	123975759	123976681	0	0	0	0
VWA5A	NC_000011	123986111	124017619	1.2782577	0.5791316	5.6184079	4.740553
OR10D1P	NC_000011	124029028	124030160	0	0	0	0
OR10D3P	NC_000011	124055877	124057012	0	0	0	0
OR8F1P	NC_000011	124077790	124078952	0	0	0	0
OR8G3P	NC_000011	124085927	124086491	0	0	0	0
OR8G2	NC_000011	124095398	124096312	0	0	0	0
OR8G7P	NC_000011	124109022	124110418	0	0	0	0
OR8G1	NC_000011	124120423	124121287	0	0	0	0
LOC100288845	NC_000011	124120797	124121268	0	0	0.0828338	0
OR8G5	NC_000011	124134723	124135763	0	0	0	0
OR8D1	NC_000011	124179736	124180662	0	0	0	0.1321462
OR8D2	NC_000011	124189158	124190093	0	0	0.0417709	0.0981567
OR8B7P	NC_000011	124194685	124195303	0	0	0	0
OR8B6P	NC_000011	124208708	124209608	0	0	0	0
OR8B5P	NC_000011	124227505	124228307	0	0	0	0
OR8B1P	NC_000011	124235315	124236287	0	0	0	0
OR8C1P	NC_000011	124246837	124247511	0	0	0	0
OR8B2	NC_000011	124252298	124253239	0	0	0	0.0325105
OR8B3	NC_000011	124266306	124267247	0	0	0	0
OR8X1P	NC_000011	124274129	124274997	0	0	0	0
OR8B4	NC_000011	124293838	124294767	0	0	0	0
OR8B8	NC_000011	124310046	124310981	0	0	0	0
OR8A2P	NC_000011	124330360	124331522	0	0	0	0
OR8B9P	NC_000011	124350974	124351459	0	0	0	0
OR8B10P	NC_000011	124386206	124387138	0	0	0	0
OR8A3P	NC_000011	124401402	124401980	0	0	0	0
OR8B12	NC_000011	124412618	124413550	0.0471042	0	0.0419052	0.0328241
OR8A1	NC_000011	124439965	124440945	0	0	0	0
OR8Q1P	NC_000011	124471398	124472254	0	0	0	0
PANX3	NC_000011	124481453	124490252	0.0274676	0	0	0
LOC100129778	NC_000011	124485943	124487312	0	0	0	0
TBRG1	NC_000011	124492742	124505822	2.417919	1.5668014	1.689027	1.9044142
SIAE	NC_000011	124505693	124543717	2.8483532	2.3872879	2.7956343	1.833825
SPA17	NC_000011	124543740	124564690	2.5714391	1.1055513	1.3725741	2.573793
NRGN	NC_000011	124609829	124617102	0.5773167	1.2983736	0.1925988	0.653733
VSIG2	NC_000011	124617370	124622109	0	0.0806127	0.0697548	0.0273192
ESAM	NC_000011	124623019	124632223	0.0470286	0	0.1045949	0.0327714
C11orf61	NC_000011	124636394	124670299	0.4542963	0.0609214	0.4041546	0.6056156
LOC100287696	NC_000011	124677705	124678485	0	0	0	0
ROBO3	NC_000011	124735282	124751362	1.0580664	0.7021277	1.3948136	1.2534148
ROBO4	NC_000011	124754114	124767831	0.2061684	0.5534584	0.0509481	0.2713698
HEPACAM	NC_000011	124789145	124806308	0	0	0	0.0259094
HEPN1	NC_000011	124789146	124790573	0.0307761	0	0	0
CCDC15	NC_000011	124824017	124911385	0.6886314	1.3927583	0.9842182	1.6047971
SLC37A2	NC_000011	124933013	124960412	0.1361599	0.5599468	0.0559069	0.160569
TMEM218	NC_000011	124966398	124973205	1.7128113	1.3099751	1.7839193	2.6491102
LOC399965	NC_000011	124983057	124984761	0	0	0	0
LOC729492	NC_000011	125002743	125009516	0.0702048	0.072178	0.0624562	0
PKNOX2	NC_000011	125034559	125303285	0.0235647	0.1332486	0.0104819	0.0328417
FEZ1	NC_000011	125315648	125366123	2.226551	1.3980583	2.3796206	0.7809813
MGC39545	NC_000011	125365225	125366707	0	0	0	0
EI24	NC_000011	125439298	125454575	11.176574	12.529851	10.375294	18.001089
STT3A	NC_000011	125462739	125491280	16.11801	15.861744	13.334739	17.808973
CHEK1	NC_000011	125495036	125546150	2.9887515	3.3520916	3.1423082	4.5534981

ACRV1	NC_000011	125542229	125550942	0.0995806	0.1023793	0.0295299	0.1156529
PATE1	NC_000011	125616188	125619743	0	0	0.0259268	0
PATE2	NC_000011	125646025	125648714	0	0.0275509	0.02384	0.0560211
PATE3	NC_000011	125658006	125661495	0	0	0	0
PATE4	NC_000011	125703211	125709967	0	0	0	0.0771019
LOC100288936	NC_000011	125743344	125744423	0.0406928	0.0418365	0	0.0850691
HYLS1	NC_000011	125753509	125770541	0.9075886	2.3686295	1.350869	1.4473235
PUS3	NC_000011	125763380	125773116	0.5481612	0.0980117	0.2968362	0.3653727
DDX25	NC_000011	125774272	125793005	0.0241209	0.2975855	0	0.0168084
LOC338667	NC_000011	125815951	125830390	0.0070486	0.0507272	0.0313533	0.0687648
CDON	NC_000011	125826713	125933187	0.2184577	0.1740631	0.4518546	0.2892371
LOC729561	NC_000011	125937438	125938513	0	0	0	0
RPUSD4	NC_000011	126071989	126081587	2.8342411	2.9858458	2.1167472	3.7427698
FAM118B	NC_000011	126081619	126132541	4.2894678	4.6679199	2.3208601	3.4785107
SRPR	NC_000011	126132833	126138750	15.857763	15.107146	12.859994	17.828128
FOXRED1	NC_000011	126139055	126148027	5.7216048	5.0486836	5.3906946	6.6241408
LOC100289010	NC_000011	126146807	126159827	0.1076283	0.2213065	0.0319164	0.1999992
RPL35AP26	NC_000011	126148097	126148513	0	0	0	0
TIRAP	NC_000011	126152982	126164828	2.1753048	1.3273193	2.139746	2.452455
DCPS	NC_000011	126173647	126215644	3.5954943	3.2688465	2.3262919	2.9403196
FLJ39051	NC_000011	126224393	126225476	0.3150411	0.0809738	0.2802693	0.2195332
ST3GAL4	NC_000011	126226096	126284533	4.4629013	6.8176906	4.3740822	3.9181571
KIRREL3	NC_000011	126294403	126870491	0.810268	0.7062736	0.1410333	0.613725
PRR10	NC_000011	126873343	126874067	0	0	0	0
LOC100132514	NC_000011	127527463	127527774	0	0	0	0
LOC387820	NC_000011	127810145	127811708	0	0	0	0
ETS1	NC_000011	128328656	128457453	11.524415	9.8423328	11.825938	11.905069
FLI1	NC_000011	128563813	128683162	1.4469008	1.2718123	0.1277377	0.1462359
KCNJ1	NC_000011	128707915	128737268	0.0309931	0	0.0275723	0.0215972
KCNJ5	NC_000011	128761313	128787964	0.0754606	0.0155163	0.0134264	0.0210336
C11orf45	NC_000011	128769460	128775592	0.012127	0.074807	0.0647311	0.0507034
TP53AIP1	NC_000011	128805265	128812822	0.0495843	0.0169926	0	0.0345523
RICS	NC_000011	128834955	129062093	0.5469338	0.9732209	1.0629627	1.1169901
LOC100188891	NC_000011	129165098	129166510	0	0	0	0
ZNF123P	NC_000011	129165153	129165374	0	0	0	0
BARX2	NC_000011	129245881	129322174	0	0	0	0.0519947
RPS27P20	NC_000011	129407732	129408068	0	0	0	0
TMEM45B	NC_000011	129685741	129729898	0.0796524	0	0.0531458	0.22202
NFRKB	NC_000011	129733670	129765490	0.8830012	1.2795912	0.5910271	1.4357243
PRDM10	NC_000011	129769601	129872730	0.2005548	0.302414	0.3092597	0.9316969
NCRNA00167	NC_000011	129872519	129875381	0	0	0	0
LOC253665	NC_000011	129902710	129903172	0	0	0	0
LOC100130501	NC_000011	129910260	129910966	0	0	0	0
APLP2	NC_000011	129939716	130014706	58.486196	36.471492	50.410424	69.122889
RPL34P21	NC_000011	129952390	129952676	0	0	0	0
ST14	NC_000011	130029682	130080257	0.0532706	0	0.047391	0.0278408
ZBTB44	NC_000011	130096572	130184607	2.8226225	2.2925426	2.7412658	4.6091392
LOC100130783	NC_000011	130155488	130155806	0	0	0	0
DDX18P5	NC_000011	130164896	130167279	0	0	0	0
ADAMTS8	NC_000011	130274818	130298539	0.0599894	0.0123351	0.0320209	0.0167212
ADAMTS15	NC_000011	130318869	130343716	0.8318277	3.0248965	0.1644482	0.4830422
BCL2L7P2	NC_000011	130415438	130417549	0	0	0	0
C11orf44	NC_000011	130542853	130584262	0	0	0	0
SNX19	NC_000011	130745766	130786382	3.1308957	2.4680634	2.283586	3.2600027
NTM	NC_000011	131240371	132206716	1.2487678	1.8340922	0.0373425	0.1828133
OPCML	NC_000011	132284875	133402403	0.0537182	0.317561	0.017921	0.037433
LOC100128095	NC_000011	132526971	133402878	0.1315028	0.5948742	0.0233977	0.0549818
SPATA19	NC_000011	133710517	133715392	0	0	0	0
LOC283172	NC_000011	133734641	133735539	0	0	0	0
LOC283174	NC_000011	133766330	133771635	0	0	0	0
LOC646543	NC_000011	133778894	133782257	0.0937063	0	0	0.0652982
IGSF9B	NC_000011	133785185	133826880	0.0300645	0	0.0053492	0.0209501
LOC100128239	NC_000011	133902167	133911236	0	0	0	0
LOC100287907	NC_000011	133902781	133910227	0	0	0.0238837	0.0561238
JAM3	NC_000011	133938820	134021649	0.323766	0.8383278	0.2773634	0.4679381
PTP4AP2	NC_000011	133993421	133995015	0	0	0	0

LOC100289344	NC_000011	134019173	134021747	0	0	0.0151835	0.0356795
NCAPD3	NC_000011	134022337	134094426	4.0772668	5.6912545	6.5011474	9.9168874
LOC100287943	NC_000011	134031371	134043585	0	0	0	0
VPS26B	NC_000011	134094561	134117686	1.8087171	2.6600204	1.4705544	2.3872214
THYN1	NC_000011	134118173	134123260	5.3230258	6.1567088	6.2745525	6.6767335
ACAD8	NC_000011	134123434	134135749	3.0907332	2.4395757	1.40141	2.1398508
LOC729860	NC_000011	134144782	134146451	0	0	0	0
GLB1L3	NC_000011	134146275	134189458	0.0298561	0.0613905	0	0.0104025
GLB1L2	NC_000011	134201806	134246218	0.0986967	0.072479	0.0250867	0.0393005
B3GAT1	NC_000011	134248398	134281812	0.0120209	0	0.0106941	0.0251298
LOC100288778	NC_000012	73727	91265	0	0	0	0
LOC100288406	NC_000012	91213	94659	0.0254994	0.0262161	0	0.1510365
FAM138D	NC_000012	147946	149412	0	0	0	0
LOC100288881	NC_000012	175937	178665	0.0161042	0.0331135	0.0143267	0
IQSEC3	NC_000012	186542	280494	0.0488495	0.0334816	0.0434578	0.0680805
SLC6A12	NC_000012	299250	323371	0.0806601	0	0.0820085	0.0562072
SLC6A13	NC_000012	329791	372002	0.0402272	0.0620367	0.0178936	0
KDM5A	NC_000012	389223	498620	1.193874	1.6531017	1.7522919	2.2185039
CCDC77	NC_000012	498516	551808	0.947009	1.424377	1.232525	1.808652
B4GALNT3	NC_000012	569543	671058	0.0766985	0.0262847	0.0113722	0.0445388
NINJ2	NC_000012	673462	772755	1.200093	1.2763673	0.8467458	1.6725451
LOC100049716	NC_000012	740030	755046	0	0	0	0
LOC100132369	NC_000012	860983	863405	0	0.1048339	0.0907136	0.0236851
WNK1	NC_000012	862225	1020618	1.5507131	1.8877517	4.195522	11.666903
HSN2	NC_000012	976870	978799	0	0	0	0
RAD52	NC_000012	1021255	1058863	0.4439215	0.9296996	0.8337304	1.0884263
LOC100130219	NC_000012	1099143	1137339	0	0	0	0
ERC1	NC_000012	1100404	1602697	1.7862631	1.5198341	1.4411576	3.6397893
FBXL14	NC_000012	1675159	1703331	4.2678292	3.1463572	5.2228866	4.4682437
WNT5B	NC_000012	1726222	1756378	11.486254	9.0914244	2.283934	4.5056074
LOC100132548	NC_000012	1800074	1807210	0	0	0	0
ADIPOR2	NC_000012	1800247	1897845	2.826431	3.1223839	6.9024706	7.4998126
RPS4P14	NC_000012	1866332	1867198	0	0	0	0
CACNA2D4	NC_000012	1901123	2027870	0.0658031	0.0676525	0.0292701	0.0458542
LRTM2	NC_000012	1929695	1945918	0	0	0	0.0083039
LOC100288635	NC_000012	2038368	2039637	0	0	0	0.0964563
DCP1B	NC_000012	2055214	2113677	3.6133345	2.9805495	5.2516329	4.406352
CACNA1C	NC_000012	2162416	2807115	0.4912528	0.6507498	0.0196104	0.0855811
RPS6P18	NC_000012	2769103	2769706	0	0	0	0
LOC100132450	NC_000012	2861760	2863863	0	0	0	0
LOC283440	NC_000012	2870366	2880835	0	0	0	0
RPL23AP14	NC_000012	2891060	2891586	0	0	0	0
LOC100288472	NC_000012	2896094	2902469	0	0	0	0.0713867
LOC728147	NC_000012	2901735	2904038	0	0	0	0
FKBP4	NC_000012	2904108	2914589	3.134966	8.7517944	10.52908	17.443826
ITFG2	NC_000012	2921863	2934106	1.2236167	1.3418737	0.7438516	1.421108
NRIP2	NC_000012	2934514	2944221	0.0474774	0.0650823	0.0985535	0.1654207
FOXM1	NC_000012	2966849	2986303	11.320366	18.055915	11.300124	33.192436
C12orf32	NC_000012	2986365	2998626	0	0	0	0
TULP3	NC_000012	3000033	3050304	8.6399079	10.699306	8.4589282	7.1892815
TEAD4	NC_000012	3068496	3149842	3.5313509	4.1224877	2.7970265	6.175779
RPL13AP24	NC_000012	3180712	3181311	0	0	0	0
TSPAN9	NC_000012	3186557	3395730	1.018499	2.2303742	0.5255293	0.5535899
RPS27P3	NC_000012	3320772	3321117	0	0	0	0
LOC100128253	NC_000012	3407072	3409384	0.0741117	0	0	0.051644
RPS26P44	NC_000012	3578116	3578462	0	0	0	0
PRMT8	NC_000012	3600425	3703139	0.0562717	0.0385688	0.0500609	0.0392124
LOC100128816	NC_000012	3720148	3721156	0.0871125	0.1343411	0.232493	0.3338688
EFCAB4B	NC_000012	3724494	3862366	0.3481508	0.5249722	0.3716695	0.517558
PARP11	NC_000012	3918027	3982608	1.262093	0.7946324	0.8877898	1.3635298
HIN1L	NC_000012	3948627	3955439	0	0	0	0
LOC399988	NC_000012	4206617	4208689	0	0	0	0
RPL18P9	NC_000012	4218662	4229121	0	0	0	0
CCND2	NC_000012	4382902	4414522	0.0134769	0.2009075	0.0119894	0.0516519
C12orf5	NC_000012	4430359	4469194	0.7957573	0.6918348	0.4608657	0.5172996
RPS15P7	NC_000012	4433513	4434005	0	0	0	0

FGF23	NC_000012	4477393	4488894	0	0	0.0129548	0
FGF6	NC_000012	4543308	4554780	0	0	0	0
C12orf4	NC_000012	4596901	4647637	2.8654385	3.7150008	3.8800674	4.8114498
RAD51AP1	NC_000012	4647950	4669213	5.5007923	7.3480279	10.562718	11.593993
DYRK4	NC_000012	4699244	4723041	2.1438162	3.4040612	4.0898808	3.0707873
AKAP3	NC_000012	4724676	4754343	0.5966482	0.2543437	0.3107091	0.2129545
NDUFA9	NC_000012	4758283	4796399	13.298756	15.595217	16.144306	15.396905
GALNT8	NC_000012	4829752	4881892	0.0411693	0.0211632	0.0183127	0.0430326
KCNA6	NC_000012	4918342	4960278	0.0294066	0.1738405	0.026161	0.0409834
KCNA1	NC_000012	5019073	5027422	0.0440418	0.1414988	0.0195904	0.1227604
LOC390282	NC_000012	5141715	5142095	0	0.1185916	0	0
KCNA5	NC_000012	5153085	5155949	0.0153397	0	0	0
NTF3	NC_000012	5541280	5604465	1.6358683	0.2587453	0.4757757	0.2630627
ANO2	NC_000012	5671817	6055398	0.1182358	0.1215588	0.0841486	0.057674
VWF	NC_000012	6058040	6233836	0.064681	0.0358071	0.0354105	0.0416052
CD9	NC_000012	6309555	6347427	27.597062	22.096188	39.192848	23.907805
ATP5J2P5	NC_000012	6379191	6379607	0	0	0	0
PLEKHG6	NC_000012	6419602	6437672	0.2700729	0.1388317	0.1856588	0.3763952
TNFRSF1A	NC_000012	6437923	6451261	34.077826	33.006024	31.948601	39.851248
LOC100288159	NC_000012	6437932	6439213	0.1169873	0.0801835	0.0346917	0.0271738
SCNN1A	NC_000012	6456013	6484715	0.1682229	0.0432377	0.2743689	0.2539862
LTBR	NC_000012	6493357	6500734	36.168122	36.991632	28.186617	28.00861
RPL31P10	NC_000012	6518762	6519206	0	0	0	0
SRP14P1	NC_000012	6547667	6547998	0	0	0	0
LOC678655	NC_000012	6548167	6560683	0	0	0	0
CD27	NC_000012	6554051	6560884	0.865647	0.3080686	0.4146712	0.34801
TAPBPL	NC_000012	6561177	6571488	5.8984426	4.2996307	4.6237495	3.116393
LOC100288190	NC_000012	6562672	6566798	0.2006769	0.1031585	0	0.2097594
VAMP1	NC_000012	6571403	6579843	0.7140363	0.8636522	0.8096023	0.9756253
PKP2P1	NC_000012	6586805	6591355	0	0	0	0
MRPL51	NC_000012	6601316	6602471	11.360326	19.021078	14.899811	17.596865
NCAPD2	NC_000012	6603298	6641132	16.679479	26.465101	22.884197	39.934688
SCARNA10	NC_000012	6619388	6619717	0	0	0	0
GAPDH	NC_000012	6643657	6647536	1929.8306	1742.9411	1658.8709	2540.1349
LOC100133042	NC_000012	6646269	6647160	1.7376566	1.9353681	0.450878	1.2613212
IFFO1	NC_000012	6648694	6665229	6.0198995	4.4207782	2.6645439	4.9284976
NOP2	NC_000012	6666037	6677480	3.4623928	5.9604341	4.0831096	6.5648786
CHD4	NC_000012	6679248	6716551	7.9346277	8.9991261	12.781781	19.632541
SCARNA11	NC_000012	6690639	6690775	0	0	0	0
LPAR5	NC_000012	6728001	6745297	0.0150508	0.0154738	0	0.0419519
ACRBP	NC_000012	6747242	6756580	0.1165117	0	0.1036521	0.064952
ING4	NC_000012	6759704	6772308	5.1900191	5.1486611	7.6953082	7.5927702
ZNF384	NC_000012	6775643	6798676	7.8802944	8.3232582	8.9775112	11.701611
C12orf53	NC_000012	6802957	6809812	0.1822738	0.1665748	0.1081039	0.0846771
COPS7A	NC_000012	6833221	6841031	27.56864	17.192347	12.437164	19.091498
MLF2	NC_000012	6857158	6862636	47.644812	62.594116	55.990187	58.805931
PTMS	NC_000012	6875541	6880118	17.770372	29.192407	35.255805	67.774187
LAG3	NC_000012	6881670	6887621	0.0441247	0	0.2551548	0.5380877
CD4	NC_000012	6898651	6929976	0.2549366	0	0.0629996	0.0394778
GPR162	NC_000012	6930963	6936583	0.48909	0.2693764	0.2330936	0.0243441
LEPREL2	NC_000012	6937572	6949018	35.981886	39.409111	17.806665	5.5628808
GNB3	NC_000012	6949375	6956557	1.1198457	0.916356	0.4066309	0.6210974
CDCA3	NC_000012	6957972	6960456	16.001664	24.715707	16.474444	30.258427
USP5	NC_000012	6961285	6975796	4.7102148	6.1736018	4.3128621	9.606864
TPI1	NC_000012	6976706	6979992	200.39673	226.25033	218.08109	273.4149
SPSB2	NC_000012	6980099	6982452	1.9976469	2.3580563	1.3822372	1.0053622
RPL13P5	NC_000012	6993145	6993778	0	0	0	0
LRRC23	NC_000012	7013897	7023406	0.8301333	0.6275472	1.0208809	1.3270781
ENO2	NC_000012	7023614	7032859	23.379806	7.9625723	17.394625	38.928859
ATN1	NC_000012	7033626	7051484	15.037277	12.729879	10.93564	13.146729
RNU7	NC_000012	7052979	7053041	0	0	0	0
C12orf57	NC_000012	7053203	7055165	19.505569	26.793841	34.921398	21.488174
PTPN6	NC_000012	7055740	7070479	0	0	0.0612815	0.0240007
PHB2	NC_000012	7074515	7079916	38.709773	55.203283	66.463152	63.541847
SCARNA12	NC_000012	7076500	7076769	0	0	0	0
EMG1	NC_000012	7079944	7085143	1.7177097	1.8521318	2.4226328	2.3355484

LPCAT3	NC_000012	7085347	7125842	6.9452365	6.0978905	12.544582	12.78592
LOC100288261	NC_000012	7092592	7126115	0.2025264	0	0.2402308	0.3763426
RPL37P20	NC_000012	7139412	7139702	0	0	0	0
C1S	NC_000012	7168017	7178336	82.747518	43.668601	171.93627	40.284853
C1R (NC_000012 71875	NC_000012	7187513	7189412	97.287111	44.913074	133.90081	41.173447
C1R (NC_000012 72412	NC_000012	7241205	7245043	157.3811	66.252529	175.61857	64.094691
C1RL	NC_000012	7247150	7261793	4.8610612	2.3558555	11.524237	4.1996141
LOC283314	NC_000012	7260904	7274447	0	0	0	0
RBP5	NC_000012	7276280	7281466	0.5142878	0.3364721	0.2079658	0.5538542
CLSTN3	NC_000012	7282967	7311541	2.4312254	0.7318518	2.8935901	2.4802107
PEX5	NC_000012	7341759	7371170	2.6894606	4.4513087	4.2323976	5.6933594
ACSM4	NC_000012	7456928	7480969	0	0	0.0672936	0.0175702
CD163L1	NC_000012	7507556	7596749	0.1342516	0.0985891	0.3753639	0.2739734
CD163	NC_000012	7623409	7656414	0.0209627	0.0431037	0.018649	0.0438229
LOC643739	NC_000012	7718894	7720174	0	0	0	0
LOC100131676	NC_000012	7734535	7759932	0	0	0	0
LOC100270648	NC_000012	7756850	7757869	0	0	0	0
APOBEC1	NC_000012	7801996	7818502	0	0.050654	0	0
GDF3	NC_000012	7842381	7848360	0	0	0	0.0250203
DPPA3	NC_000012	7864089	7870155	0	0	0	0
CLEC4C	NC_000012	7882011	7902069	0.0334461	0	0	0
LOC360030	NC_000012	7917812	7926717	0.0484545	0	0	0
NANOG	NC_000012	7941995	7948655	0	0	0.0186356	0
SLC2A14	NC_000012	7966397	8025495	0.5005493	0.4116939	0.8906051	0.2371858
RPS20P29	NC_000012	8013045	8013360	0	0	0	0
LOC100130582	NC_000012	8023448	8023971	0	0	0	0
NANOGP1	NC_000012	8028871	8051665	0	0	0	0
SLC2A3	NC_000012	8071824	8088892	7.6334664	5.5303199	7.595134	7.8545269
RPS20P28	NC_000012	8147366	8147674	0	0	0	0
HSPD1P12	NC_000012	8167556	8169307	0	0	0	0
FOXJ2	NC_000012	8185359	8208118	3.7679075	3.3710345	3.6373146	6.374131
C3AR1	NC_000012	8210919	8218955	0.4945581	0.2080054	1.139929	0.1879788
NECAP1	NC_000012	8234807	8250372	0.7799455	0.7147066	0.8899523	1.5123397
CLEC4A	NC_000012	8276228	8291203	0.0686155	0	0.0305211	0.071721
POU5F1P3	NC_000012	8286108	8287469	0	0	0	0
LOC100287495	NC_000012	8293380	8295303	0	0	0	0
ZNF705A	NC_000012	8325150	8332642	0	0.0262847	0.0113722	0
FAM66C	NC_000012	8332805	8353596	0	0	0	0
LOC100287535	NC_000012	8347713	8371931	0.227711	0.4682218	0.135052	0.1057854
LOC100287458	NC_000012	8372156	8373711	0.9038197	0.987298	0.3769046	0.393636
FAM90A1	NC_000012	8373856	8380214	0	0	0	0.0243441
LOC643856	NC_000012	8380303	8385198	0	0	0	0
LOC653113	NC_000012	8383645	8395542	0	0	0	0
LOC100287597	NC_000012	8407389	8407904	0	0	0	0
LOC100130149	NC_000012	8426688	8426861	0	0	0	0
LOC389634	NC_000012	8509559	8543348	0	0	0	0
RPS3AP43	NC_000012	8511606	8512454	0	0	0	0
LOC100288174	NC_000012	8521588	8521956	0	0	0	0
LOC728650	NC_000012	8548037	8550389	0.0186775	0	0.016616	0
OR7E140P	NC_000012	8567276	8568471	0	0	0	0
OR7E148P	NC_000012	8580158	8581310	0	0	0	0
OR7E149P	NC_000012	8589899	8591079	0	0	0	0
CLEC6A	NC_000012	8608591	8630926	0.0544925	0	0	0
RPL15P17	NC_000012	8613180	8613828	0	0	0	0
LOC100287625	NC_000012	8652207	8665320	0	0	0	0
CLEC4D	NC_000012	8666136	8674962	0	0	0	0.0158186
CLEC4E	NC_000012	8685901	8693558	0	0	0	0.042574
AICDA	NC_000012	8754762	8765442	0.0471885	0	0.0279868	0.0767266
LOC399994	NC_000012	8786899	8790861	0	0	0	0
MFAP5	NC_000012	8798540	8815433	10.567705	17.292607	0.0949629	0.1700201
LOC100128534	NC_000012	8831273	8832265	0	0	0	0
RIMKLB	NC_000012	8850518	8929787	0.3316848	0.5657613	0.1676568	0.6145988
RPSAP51	NC_000012	8895054	8895671	0	0	0	0
A2ML1	NC_000012	8975150	9029379	0.0254036	0	0.030133	0.023603
PHC1	NC_000012	9067316	9094063	0.7128116	0.8200888	0.7624742	1.0170842
M6PR	NC_000012	9092964	9102252	37.769691	32.792029	40.977563	57.655639

KLRG1	NC_000012	9142221	9163343	0.0490768	0.0504561	0.2401302	0.2222911
LOC643996	NC_000012	9188943	9189837	0	0	0	0
C12orf33	NC_000012	9208185	9217660	0	0	0	0
LOC144571	NC_000012	9217773	9220651	0	0	0	0
A2M	NC_000012	9220304	9268558	0.1227868	0.0776848	0.0840266	0.052654
LOC100130074	NC_000012	9280122	9281240	0	0	0	0
LOC100129641	NC_000012	9287518	9288193	0	0	0	0
PZP	NC_000012	9301436	9360966	0.0190665	0.0294035	0.0169621	0.0199294
A2MP	NC_000012	9384202	9386908	0	0	0	0
PTMAP4	NC_000012	9391909	9393092	0	0	0	0
LOC642846	NC_000012	9436253	9466684	0	0	0	0
LOC100289634	NC_000012	9465764	9467366	0.1919137	0.0281868	0.1219512	0.1719425
LOC731039	NC_000012	9487153	9488193	0.0955396	0	0	0
LOC728715	NC_000012	9549659	9555084	0	0.1358899	0.17638	0.1842098
LOC100287838	NC_000012	9559072	9567234	0	0	0	0
LOC100286889	NC_000012	9569627	9571183	0.1693573	0.0290195	0.1004433	0.255699
DDX12	NC_000012	9570287	9600768	0.5303124	0.3944122	0.6323868	1.8005384
LOC100286917	NC_000012	9570287	9571496	0.1927554	0	0.0685922	0.1074557
OVOS	NC_000012	9620233	9726188	0.0196461	0.0201982	0.0349554	0.0342254
KLRB1	NC_000012	9747870	9760497	0	0.0610587	0	0.3724647
LOC644138	NC_000012	9794266	9795807	0	0	0	0
LOC374443	NC_000012	9800643	9811007	0	0	0	0
CLEC2D	NC_000012	9822309	9852039	0.5525973	0.3701441	0.4990545	0.4784226
NPM1P16	NC_000012	9847883	9849144	0	0	0	0
NPM1P7	NC_000012	9847883	9849159	0	0	0	0
CLECL1	NC_000012	9875091	9885860	0	0	0	0.0482282
CD69	NC_000012	9905082	9913497	0	0	0	0
KLRF1	NC_000012	9980077	9997604	0	0	0.0315557	0.0247174
CLEC2B	NC_000012	10004968	10022458	0.3019015	4.0571946	0.7673713	0.4808617
CLEC2A	NC_000012	10065826	10084980	0	0	0	0.0668665
CLEC12A	NC_000012	10124008	10138194	0	0	0	0.0393889
CLEC1B	NC_000012	10145660	10151899	0	0.046485	0	0.0315071
CLEC12B	NC_000012	10163231	10171399	0	0.0387286	0	0.0262499
CLEC9A	NC_000012	10183276	10218565	0	0.0270074	0	0.0732215
CLEC1A	NC_000012	10223080	10251605	0.0494078	0	0.0219773	0.0172147
LOC390294	NC_000012	10264136	10265073	0	0	0	0
CLEC7A	NC_000012	10269376	10282868	0	0.0165386	0.014311	0.0112097
OLR1	NC_000012	10310899	10324790	0.0174675	0.0538753	0	0
C12orf59	NC_000012	10331557	10344403	0.0315154	0	0.0420555	0
GABARAPL1	NC_000012	10365489	10375727	0.5640415	1.1114634	0.7108648	0.9334856
KLRD1	NC_000012	10457050	10469850	0.0916953	0.0134675	0.0116535	0.0182563
KLRK1	NC_000012	10524952	10542640	0.1394297	0.0573393	0.1488486	0.019432
KLRC4	NC_000012	10559983	10562356	0	0	0	0
KLRC3	NC_000012	10564914	10573194	0.0383827	0.078923	0	0
KLRC2	NC_000012	10583198	10588592	0	0	0	0.0250818
KLRC1	NC_000012	10598627	10606984	0	0	0	0.0212231
LOC255308	NC_000012	10658382	10661253	0	0	0	0
KLRA1	NC_000012	10741077	10752434	0.3184654	0.204635	0.2124868	0.4854487
MAGOHB	NC_000012	10756794	10766183	3.5104055	2.2950856	2.8652345	3.8830303
STYK1	NC_000012	10771538	10826891	0.220108	0.2262942	0.326357	0.245408
CSDA	NC_000012	10851676	10875953	13.269071	12.520426	9.0910753	21.145749
LOC100286954	NC_000012	10922906	10923208	0	0	0	0
TAS2R7	NC_000012	10954131	10955226	0	0	0	0.0558848
TAS2R8	NC_000012	10958650	10959579	0	0.0485843	0	0.06586
TAS2R9	NC_000012	10961693	10962767	0	0	0.0363698	0
TAS2R10	NC_000012	10977945	10978868	0	0	0.0423134	0.0331438
PRR4	NC_000012	10998448	11324213	0.7523866	1.092046	1.4568076	1.9429681
LOC440082	NC_000012	11005926	11009863	0	0	0	0
PRH1	NC_000012	11033560	11324210	0.1461288	0.0375589	0.1625003	0.0763713
LOC100287971	NC_000012	11034548	11039004	0	0	0	0
PS10	NC_000012	11047533	11048480	0	0	0	0
TAS2R12	NC_000012	11047533	11048480	0	0	0	0
TAS2R13	NC_000012	11060525	11062161	0	0	0	0
PRH2	NC_000012	11081834	11087444	0.0101661	0	0.0090441	0
TAS2R14	NC_000012	11090853	11091806	0	0	0	0
TAS2R15	NC_000012	11117049	11117951	0	0	0	0

PS8	NC_000012	11117181	11117951	0	0	0	0
TAS2R50	NC_000012	11138512	11139511	0	0	0	0
TAS2R20	NC_000012	11148561	11150474	0	0	0	0
TAS2R19	NC_000012	11174271	11175170	0	0	0	0
TAS2R31	NC_000012	11182986	11184006	0	0	0	0
TAS2R63P	NC_000012	11200971	11201855	0	0	0	0
TAS2R46	NC_000012	11213964	11214893	0	0	0	0
TAS2R64P	NC_000012	11229912	11230841	0	0	0	0
TAS2R43	NC_000012	11243886	11244912	0	0	0	0
TAS2R30	NC_000012	11285884	11286843	0	0	0.0407266	0.0319009
TAS2R18	NC_000012	11311375	11312293	0	0	0	0
LOC100129361	NC_000012	11323789	11325932	1.9883295	2.2971039	1.5682792	2.0426108
PS5	NC_000012	11332139	11333061	0	0	0	0
TAS2R42	NC_000012	11338599	11339543	0	0	0	0.0648146
PRB3	NC_000012	11418857	11422641	0.1208476	0	0	0.0280705
PRB4	NC_000012	11460015	11463366	0	0	0	0
PRB1	NC_000012	11504757	11508524	0.0374665	0	0.0666625	0
PRB2	NC_000012	11544474	11548498	0	0	0	0
LOC100288003	NC_000012	11629096	11634597	0	0	0	0
LOC100130073	NC_000012	11652887	11653959	0	0	0	0
LOC100129640	NC_000012	11661749	11667075	0	0	0	0
RNU7-60P	NC_000012	11699313	11699572	0	0	0	0
ETV6	NC_000012	11802788	12048336	1.6364094	1.3353585	4.0736151	4.1317251
BCL2L14	NC_000012	12223878	12363947	0.0968023	0.0663486	0.014353	0.1011835
LRP6	NC_000012	12268959	12419811	0.5839428	1.0752619	1.205686	1.4849346
RPL21P100	NC_000012	12308157	12308738	0	0	0	0
MORF4LP2	NC_000012	12352491	12353513	0	0	0	0
MANSC1	NC_000012	12482218	12503169	4.8023536	3.9931348	6.3148441	4.6397291
RPL23AP66	NC_000012	12491710	12492188	0	0	0	0
LOH12CR2	NC_000012	12508342	12510001	0	0	0	0
LOH12CR1	NC_000012	12510020	12619838	1.096071	1.213559	1.106358	1.6597656
LOC100288143	NC_000012	12605942	12620485	0.0825707	0.1910055	0.2571	0.2013848
DUSP16	NC_000012	12628831	12715317	0.8380482	0.8109192	2.1050849	2.1298289
RPL19P17	NC_000012	12721058	12721732	0	0	0	0
CREBL2	NC_000012	12764831	12798041	3.1542354	3.471937	3.411127	4.7718594
GPR19	NC_000012	12813995	12849121	0.3025696	0.4925328	0.2018807	0.2811234
CDKN1B	NC_000012	12870302	12875305	0.9835079	0.7489996	0.9559703	1.0787877
APOLD1	NC_000012	12878851	12944400	0.5372867	0.6647372	0.5184924	0.3807486
DDX47	NC_000012	12966280	12982915	8.4171627	13.154661	10.694271	13.837658
RPL37AP9	NC_000012	12993738	12994013	0	0	0	0
RPS6P1	NC_000012	13003891	13004685	0	0	0	0
RPL13AP20	NC_000012	13028411	13029070	0	0	0	0
GPRC5A	NC_000012	13043956	13066600	14.366802	9.9844066	5.6500165	26.371125
GPRC5D	NC_000012	13093709	13103318	0.0423393	0.0435293	0.0376662	0.0295037
HEBP1	NC_000012	13127798	13153221	6.5903835	6.6614114	6.7523176	11.635906
HTR7P	NC_000012	13153376	13157764	0	0	0	0
RPS26P46	NC_000012	13174199	13174537	0	0	0	0
KIAA1467	NC_000012	13197315	13236383	1.2593953	1.4002922	1.0291017	1.1311248
GSG1	NC_000012	13237042	13256619	0.1865375	0.2109582	0.0331898	0.1169881
EMP1	NC_000012	13349602	13369708	9.4667375	11.166939	11.32212	16.26264
C12orf36	NC_000012	13524023	13529645	0.0639247	0	0.0142173	0.033409
GRIN2B	NC_000012	13714410	14133022	0.0221923	0.0228161	0	0.0412387
LOC100288200	NC_000012	14341212	14345225	0	0	0	0
MRPS18CP4	NC_000012	14390195	14390258	0	0	0	0
LOC644693	NC_000012	14402553	14408165	0	0	0	0
RPL30P11	NC_000012	14509799	14510071	0	0	0	0
ATF7IP	NC_000012	14518611	14651697	4.2430174	3.7224686	8.7321531	11.090924
PLBD1	NC_000012	14656597	14720791	2.7751989	3.999152	0.3035525	0.0792569
GUCY2C	NC_000012	14765566	14849519	0.0914635	0.1057884	0.1017106	0.1593386
HIST4H4	NC_000012	14923654	14924065	21.120752	26.868771	34.637406	23.042991
H2AFJ	NC_000012	14927270	14930936	0.6232092	0.6037597	0.6823682	0.3758166
WBP11	NC_000012	14939412	14956401	7.8396808	9.8849253	7.3545533	19.046063
C12orf60	NC_000012	14956506	14976792	0.1559831	0.2806422	0.4163006	0.5434761
C12orf69	NC_000012	14957584	14967116	0.0208879	0	0	0.0145556
ART4	NC_000012	14982245	14996413	0.0961668	0.0329565	0.0285176	0.0670129
MGP	NC_000012	15034827	15038828	0.3989249	0.0683561	0	0.0463311

ERP27	NC_000012	15066976	15091463	0	0.0882488	0.0254541	0.0398761
ARHGDIB	NC_000012	15094950	15114562	0.0361416	1.3005091	0.2250682	0.2014795
PDE6H	NC_000012	15125978	15134799	0	0.0623219	0	0.0422412
RERG	NC_000012	15260717	15374304	0.1189397	0.142663	0.458519	0.1519502
LOC728996	NC_000012	15407674	15408757	0	0	0	0
LOC100288269	NC_000012	15408057	15475537	0	0	0	0
PTPRO	NC_000012	15475487	15750335	0.2402668	0.3881736	0.1343559	0.4113932
RPS2P42	NC_000012	15592438	15593384	0	0	0	0
EPS8	NC_000012	15773075	15942510	9.9911708	6.7520517	6.7812534	12.694095
MRPS7P2	NC_000012	15956050	15957098	0	0	0	0
STRAP	NC_000012	16035288	16056412	10.255356	14.737324	13.490403	18.034472
DERA	NC_000012	16064186	16190315	13.306831	11.846228	11.520628	15.419022
MGST1	NC_000012	16500076	16517344	33.655093	41.299215	58.612052	48.00584
SUPT16HP	NC_000012	16552209	16555438	0	0	0	0
LOC121520	NC_000012	16666274	16667804	0	0	0	0
LMO3	NC_000012	16701307	16761024	0.011321	0.1513097	0.1107865	0.3471135
LOC728622	NC_000012	17142060	17142918	0.0866829	0.1782383	0.0771155	1.0872738
LOC387845	NC_000012	17143604	17145016	0	0	0	0
RPL7P40	NC_000012	17274650	17275394	0	0	0	0
LOC390297	NC_000012	17536163	17537581	0	0	0	0
LOC390298	NC_000012	17552622	17553839	0	0	0	0
RERGL	NC_000012	18233803	18243114	0	0.0398443	0.0689551	0
PIK3C2G	NC_000012	18414474	18801352	0.0090727	0	0	0.0189667
PLCZ1	NC_000012	18836116	18890918	0.0635396	0.0217751	0	0.0590359
LOC643668	NC_000012	18845697	18847253	0	0	0	0
CAPZA3	NC_000012	18891045	18892122	0.0815366	0	0	0.056818
RPL7P6	NC_000012	19242065	19242885	0	0	0	0
LOC100128109	NC_000012	19260785	19261117	0	0	0	0
PLEKHA5	NC_000012	19282648	19529334	1.3064939	2.1053198	2.3658025	2.1243063
AEBP2	NC_000012	19592608	19675173	0.9084705	1.0297984	0.9325391	1.0388651
RPL34P25	NC_000012	19661838	19662179	0	0	0	0
LOC400013	NC_000012	20084163	20095952	0	0	0	0
PDE3A	NC_000012	20522197	20833881	1.6198184	0.5039856	0.0094805	0.0668341
LOC728688	NC_000012	20704503	20707294	0	0	0	0
SLCO1C1	NC_000012	20848399	20906320	0	0	0	0.009101
SLCO1B3	NC_000012	20963638	21069658	0.0162051	0.0333211	0.0144165	0.0225847
LST-3TM12	NC_000012	21168630	21243040	0	0.0469926	0.0203315	0.0159256
SLCO1B1	NC_000012	21284137	21392730	0.0316402	0	0.014074	0.0440963
SLCO1A2	NC_000012	21417534	21548371	0.0111671	0.011481	0.0099346	0.0350176
IAPP	NC_000012	21525802	21532914	0.022241	0	0	0.0619937
PYROXD1	NC_000012	21590538	21624184	0.5112737	0.7665628	0.8338792	1.3211896
RECQL	NC_000012	21621844	21654603	11.083002	12.948829	15.881123	15.105788
GOLT1B	NC_000012	21654699	21671337	7.6963132	11.088714	15.103705	10.716835
C12orf39	NC_000012	21679256	21684210	0	0.1550031	0.9388781	0.0525298
GYS2	NC_000012	21689123	21757781	0.014032	0.0288527	0	0.0684464
LOC100287252	NC_000012	21733274	21757831	0	0	0	0
LDHB	NC_000012	21788276	21810776	89.144418	118.4894	123.20384	163.44237
KCNJ8	NC_000012	21917889	21927747	1.2551364	7.30601	0.7717705	0.5787986
ABCC9	NC_000012	21950323	22089628	0.5037195	3.988184	2.1805567	1.6863043
CMAS	NC_000012	22199159	22218602	1.7417737	1.6869162	2.8071199	3.9402486
ST8SIA1	NC_000012	22346325	22487648	0.2533561	0.0790733	0.6198296	0.1923119
KIAA0528	NC_000012	22601517	22697452	2.9710919	4.1206279	3.3261314	6.2111255
LOC100288378	NC_000012	22708461	22727992	0	0	0	0
ETNK1	NC_000012	22778076	22843608	2.6527745	2.1769173	2.499119	3.1102737
RPS27P22	NC_000012	22875163	22875403	0	0	0	0
SOX5	NC_000012	23685231	24715380	0.1957813	0.0287548	0.0663514	0.0454761
LOC100129937	NC_000012	24715603	24737486	0	0	0	0
C12orf67	NC_000012	24719898	24737102	0.0257007	0.026423	0.0228641	0.0179093
RPL21P102	NC_000012	24795587	24796132	0	0	0	0
BCAT1	NC_000012	24964278	25102308	13.933509	14.516404	12.556434	14.980889
DAD1L	NC_000012	25070636	25071266	0	0	0	0
BRI3P2	NC_000012	25120061	25120438	0	0	0	0
LOC196415	NC_000012	25146358	25150373	0.1159584	0	0	0.0202011
LOC645177	NC_000012	25157276	25177900	0.0291821	0	0	0.0203352
LRMP	NC_000012	25205241	25261269	0.0156455	0.0160852	0	0.0218048
CASC1	NC_000012	25261223	25348094	0	0.0502038	0	0.0793978

LYRM5	NC_000012	25348150	25357949	1.5875573	0.5971375	2.0668317	1.5110072
KRAS	NC_000012	25358180	25403854	4.9047556	5.7343997	4.7456554	4.0900963
RPL39P27	NC_000012	25562208	25562379	0	0	0	0
IFLTD1	NC_000012	25629016	25801488	0.0371812	0.0382262	0.0165387	0
LOC645233	NC_000012	25956127	25959193	0	0	0	0
RASSF8	NC_000012	26147970	26232825	13.064611	8.8313013	5.5802889	7.113324
BHLHE41	NC_000012	26272959	26278003	6.6968734	5.4865559	5.3888444	7.2835359
LOC729204	NC_000012	26278088	26288111	0.0878965	0.1355502	0.3127805	0.2143741
SSPN	NC_000012	26348269	26387710	0.5576007	0.6114903	0.7771561	0.4662701
ITPR2	NC_000012	26488285	26986131	0.4301108	0.7298083	0.7092811	1.2451713
LOC100288514	NC_000012	27034045	27091264	0.1300244	0.0850683	0.0630945	0.2388708
C12orf11	NC_000012	27058112	27091254	1.0484961	1.685268	1.2480742	2.4594576
FGFR1OP2	NC_000012	27091316	27118862	0.6687774	0.8643781	0.7649524	0.8654857
TM7SF3	NC_000012	27124503	27167339	8.7471745	7.2983774	10.408625	7.2580821
MED21	NC_000012	27175483	27182682	2.9332248	3.660036	4.5721626	3.6686962
C12orf71	NC_000012	27233990	27235455	0.0454951	0	0	0.0634055
LOC729222	NC_000012	27274914	27309829	0	0	0	0
STK38L	NC_000012	27397078	27478892	2.4655148	4.9632611	1.8636148	1.3696493
ARNTL2	NC_000012	27485988	27573466	6.7857892	6.8828602	7.6574507	6.6486135
C12orf70	NC_000012	27619743	27655118	0	0.0710431	0.1536854	0.1926093
PPFIBP1	NC_000012	27677045	27848496	4.6720253	3.1341931	1.6587611	2.2083111
REP15	NC_000012	27849428	27850566	0.1929246	0.1190081	0.1716311	0.3226502
MRPS35	NC_000012	27863729	27909228	10.368484	14.948075	13.018546	12.315635
LOC100287284	NC_000012	27915599	27930361	0	0.037188	0	0.0252057
KLHDC5	NC_000012	27933187	27955973	1.5761307	1.4178746	0.9972327	1.7989571
PTHLH	NC_000012	28111017	28124916	0.2164938	0.4109138	0.2370447	0.1392567
LOC100129646	NC_000012	28317888	28318598	0	0	0	0
LOC729291	NC_000012	28340722	28393117	0	0	0	0
CCDC91	NC_000012	28410133	28703099	2.7667623	2.6897186	3.0809217	4.1314076
RPL29P27	NC_000012	28717480	28718104	0	0	0	0
FAR2	NC_000012	29376598	29487006	4.992687	3.3860592	7.3529551	9.3555714
ERGIC2	NC_000012	29493579	29534143	10.486241	12.916808	19.119744	21.749351
OVCH1	NC_000012	29580489	29650619	0.0128504	0.026423	0	0.0626825
TMTC1	NC_000012	29653757	29937692	3.9758178	2.4381929	2.9430658	3.6106874
RPL21P99	NC_000012	29811274	29811773	0	0	0	0
IPO8	NC_000012	30781922	30848751	2.9406788	2.8918779	3.5184776	5.6367357
CAPRIN2	NC_000012	30862486	30907448	1.9205916	2.5286586	1.4732416	2.1645678
LOC100287314	NC_000012	30947977	30949140	0.4908308	1.0092512	0.1679449	0.9734712
TSPAN11	NC_000012	31079838	31149537	0.0790152	0.0487415	0.0702941	0.0825914
DDX11	NC_000012	31226779	31257725	1.5220167	1.3922057	1.6228427	5.0144631
OVOS2	NC_000012	31267272	31359088	0.0095602	0	0.01701	0.0066619
LOC100288647	NC_000012	31372450	31397622	0	0	0	0
RPL13AP22	NC_000012	31404847	31406741	0	0	0	0
FAM60A	NC_000012	31433518	31479159	0.8906545	1.0158396	5.5835979	2.4728765
FLJ13224	NC_000012	31477250	31478879	0	0	0	0
DENND5B	NC_000012	31535157	31743952	0.8236491	1.3856692	1.5154418	1.4837951
MRPL30P2	NC_000012	31630410	31630791	0	0	0	0
RPL31P50	NC_000012	31750040	31750342	0	0	0	0
AK3L2	NC_000012	31767388	31769519	0	0	0	0
C12orf72	NC_000012	31800094	31822016	0.6275682	0.5882761	0.328413	0.4501767
AMN1	NC_000012	31824071	31882108	0.7706562	0.9636271	2.2050286	1.3207886
LOC144383	NC_000012	31907570	31908217	0	0	0	0
LOC440093	NC_000012	31944119	31945175	0.0415783	0	0.0739784	0.0869202
RPLP2P4	NC_000012	32050754	32051063	0	0	0	0
RPL12P32	NC_000012	32101180	32101805	0	0	0	0
LOC100132543	NC_000012	32106094	32108555	0	0	0	0
C12orf35	NC_000012	32112353	32146041	0.8182977	1.1096405	1.8513294	2.2464798
BICD1	NC_000012	32260185	32531141	0.4040935	0.5539342	0.3485991	0.4522481
FGD4	NC_000012	32655041	32798984	5.5259802	0.8225068	0.1602552	0.941452
DNM1L	NC_000012	32832137	32898584	8.6714855	12.353918	9.240318	11.467729
YARS2	NC_000012	32899459	32908874	2.5614619	3.7739234	2.8349772	3.9633849
PKP2	NC_000012	32943680	33049780	0.0396019	0.0101787	0.0176155	0.0137981
RPL35AP27	NC_000012	33015965	33016405	0	0	0	0
ASSP3	NC_000012	33138670	33140234	0	0	0	0
ASSP14	NC_000012	33138672	33140235	0	0	0	0
SYT10	NC_000012	33528348	33592754	0.0133744	0	0.0237964	0

LOC390301	NC_000012	33607281	33608393	0	0	0	0
ALG10	NC_000012	34175216	34181237	1.2223453	2.513399	1.9873763	2.4672217
LOC260338	NC_000012	34317708	34319889	0	0	0	0
LOC100131369	NC_000012	34360418	34362612	0	0	0	0
LOC100132086	NC_000012	34372100	34403200	0	0	0	0
LOC100130629	NC_000012	37935623	37940447	0	0	0	0
LOC100130830	NC_000012	37935811	37938386	0	0	0	0.1607605
LOC100128817	NC_000012	38471981	38482615	0	0	0	0
LOC727847	NC_000012	38555427	38555948	0	0	0	0.0586683
LOC121014	NC_000012	38595369	38597559	0	0	0	0
ALG10B	NC_000012	38710557	38723528	0.5365925	0.7804161	1.0905714	1.2798366
CPNE8	NC_000012	39046002	39299420	3.5681748	4.3864846	7.4670589	7.034608
LOC100287679	NC_000012	39529669	39564685	0	0	0	0
KIF21A	NC_000012	39687030	39836918	0.1805711	0.242768	0.5560653	0.8904832
LOC100130906	NC_000012	39763882	39837158	0	0.0738291	0.0638849	0.0500407
LOC390306	NC_000012	39860246	39861147	0	0	0	0
ABCD2	NC_000012	39945022	40013843	0.0329139	0.0169195	0.0292811	0.0344035
C12orf40	NC_000012	40019972	40115716	0	0.0162006	0	0.0109806
SLC2A13	NC_000012	40148823	40499661	1.4998754	1.7936578	1.697224	1.5393342
RPL30P13	NC_000012	40462042	40462460	0	0	0	0
LRRK2	NC_000012	40618813	40763087	0.3430106	0.3330592	1.1273661	1.4042627
LOC100287737	NC_000012	40787250	40872906	0.0205847	0.0070544	0.0183127	0.0286884
MUC19	NC_000012	40936368	40964554	0.0398262	0.0204728	0.0531458	0.0693812
CNTN1	NC_000012	41086358	41464094	0.0384723	0.1054763	0.0684521	0.0178727
PDZRN4	NC_000012	41831541	41968392	0.0271034	0	0	0.0094434
LOC100287802	NC_000012	42093193	42094065	0	0	0	0
LOC100129078	NC_000012	42305058	42326571	0	0	0	0
GLT8D3	NC_000012	42475647	42538673	0.9565445	0.6877965	1.2007531	2.0937292
YAF2	NC_000012	42550906	42632050	2.6180099	2.5702472	3.9326653	3.1327695
LOC727884	NC_000012	42680713	42682362	0	0	0	0
ZCRB1	NC_000012	42705888	42719932	6.6494342	8.6005282	10.516482	10.97779
PPHLN1	NC_000012	42719947	42842422	2.0895779	2.4340895	3.8799111	4.08777
LOC100132797	NC_000012	42824621	42827158	0	0	0	0
LOC100288182	NC_000012	42850556	42859914	0.0244746	0.0251625	0	0.0227398
PRICKLE1	NC_000012	42852140	42983572	0.1557149	0.0376685	0.0651898	0.0382971
LOC390308	NC_000012	43030434	43031065	0	0	0	0
RPS27P21	NC_000012	43072619	43072960	0	0	0	0
MRPS36P5	NC_000012	43447886	43448187	0	0	0	0
ADAMTS20	NC_000012	43748012	43945724	0.0153317	0.0157626	0.0204592	0.0053419
RPL21P101	NC_000012	43954569	43955139	0	0	0	0
LOC400026	NC_000012	43963572	43975627	0	0	0	0
LOC401717	NC_000012	44040729	44054904	0	0	0	0
PUS7L	NC_000012	44122410	44152596	1.898196	1.7666618	2.3997142	5.4162663
IRAK4	NC_000012	44152747	44183346	3.2194282	1.6237297	3.4405137	3.9930157
TWF1	NC_000012	44187526	44200178	16.381361	21.144942	22.611104	30.644993
TMEM117	NC_000012	44229962	44783541	0.9451233	1.1727246	0.7683244	0.7380857
ZNF75B	NC_000012	44401376	44404318	0	0	0	0
NELL2	NC_000012	44902058	45307711	0.0103798	0	0.1477471	0.0433985
LOC100288347	NC_000012	45269000	45270157	0	0	0	0
DBX2	NC_000012	45408539	45444882	0.0322911	0.0331987	0	0
RACGAP1P	NC_000012	45456401	45459194	0	0	0	0
LOC100287961	NC_000012	45465846	45466094	0	0	0	0
LOC100129945	NC_000012	45466204	45466462	0	0	0	0
PLEKHA9	NC_000012	45566847	45609789	0.3584684	0.4422519	0.3826842	0.6661202
ANO6	NC_000012	45609770	45834187	6.136636	11.037751	12.60607	14.832981
LOC100128248	NC_000012	45838579	45839188	0	0	0	0
LOC400027	NC_000012	46119503	46121704	0.9779579	0.5335007	0.6391973	0.8205576
ARID2	NC_000012	46123620	46301819	1.1680076	2.12948	2.7432027	3.5631355
SFRS2IP	NC_000012	46312914	46384401	3.8185667	5.1809825	9.3780627	10.716288
LOC100288055	NC_000012	46383845	46386160	0.3403071	0.1999266	0.2162476	0.1016312
RPL13AP21	NC_000012	46397809	46398468	0	0	0	0
SLC38A1	NC_000012	46576838	46663208	8.0056517	13.171172	22.863346	22.79816
SLC38A2	NC_000012	46751971	46766645	109.85258	139.48201	104.42028	142.7029
LOC100288798	NC_000012	46781530	47268854	0.2811188	0.6262092	0.5418639	0.9794737
OR7A19P	NC_000012	46986256	46987188	0	0	0	0
SLC38A4	NC_000012	47158544	47219780	0.0772377	0.3289778	0.0196322	0

AMIGO2	NC_000012	47469490	47473734	0.3781928	0.7890799	0.2078079	0.1782769
LOC100289175	NC_000012	47472345	47473000	0.0755124	0.0776347	0.0671779	0.0526201
LOC643058	NC_000012	47529105	47529731	0	0	0	0
LOC100233209	NC_000012	47602203	47610226	0	0	0	0
FAM113B	NC_000012	47610052	47630443	0.1058994	0.1524259	0.2072642	0.0442769
LOC100288986	NC_000012	47628527	47629980	0.0326753	0.0671872	0.0290688	0.0227694
LOC728148	NC_000012	47801702	47813962	0	0	0	0.0819943
LOC100288129	NC_000012	47856996	47857410	0	0	0	0
LOC100127978	NC_000012	47875372	47879517	0	0	0	0
RPAP3	NC_000012	48057074	48099774	4.7101294	6.6634512	5.2811149	6.3474061
P11	NC_000012	48103528	48119350	0.1514803	0.0584016	0.0673806	0.2506991
RAPGEF3	NC_000012	48128453	48152889	1.0318049	0.3148192	0.971221	1.0112426
SLC48A1	NC_000012	48166967	48176536	2.4914621	0.8637565	4.9484065	4.0981215
HDAC7	NC_000012	48176505	48213763	15.847999	19.179248	14.982843	20.87346
VDR	NC_000012	48235320	48298814	3.0740753	5.8951329	0.6632257	1.5520881
TMEM106C	NC_000012	48357330	48362661	52.555222	53.036437	110.34968	100.26369
COL2A1	NC_000012	48366748	48398285	0.025918	0.0621749	0.0768578	0.054182
LOC728114	NC_000012	48413554	48419165	0	0	0	0
SENP1	NC_000012	48436757	48499641	4.175361	5.7746032	7.3959424	7.225294
RPL37P19	NC_000012	48448751	48449036	0	0	0	0
PFKM	NC_000012	48513028	48539887	12.781095	12.33263	12.91604	17.349536
ASB8	NC_000012	48541572	48551377	3.0610709	3.4756463	4.2344474	5.5202134
LOC390311	NC_000012	48562630	48563442	0	0	0	0
LOC100130826	NC_000012	48564511	48567151	0	0	0	0
C12orf68	NC_000012	48577366	48579709	0.074997	0.2313143	0.0667194	0.0391957
DKFZP779L1853	NC_000012	48592150	48596357	0	0	0	0
OR10AD1	NC_000012	48596122	48597075	0	0	0	0.0642031
H1FNT	NC_000012	48722763	48724062	0	0	0	0
ZNF641	NC_000012	48735912	48744577	1.058083	0.8741415	0.4874589	0.4213225
OR5BK1P	NC_000012	48749442	48750328	0	0	0	0
OR5BT1P	NC_000012	48779034	48780188	0	0	0	0
OR5BJ1P	NC_000012	48788166	48789269	0	0	0	0
OR8S21P	NC_000012	48810834	48811221	0	0	0	0
OR8T1P	NC_000012	48835716	48836830	0	0	0	0
ANP32D	NC_000012	48866448	48866843	0	0	0	0
C12orf54	NC_000012	48877023	48890167	0	0	0	0
RPS10P20	NC_000012	48881696	48882270	0	0	0	0
OR8S1	NC_000012	48919415	48921886	0	0	0.0724029	0.0850691
OR5BS1P	NC_000012	48953665	48954597	0	0	0	0
LALBA	NC_000012	48961467	48963829	0	0	0	0
OR11M1P	NC_000012	49021523	49022695	0	0	0	0
C12orf41	NC_000012	49046994	49076035	6.4256108	8.6694588	7.2560627	6.094184
SNORA34	NC_000012	49048165	49048301	0	0	0	0
SNORA2A	NC_000012	49050431	49050565	0	0	0	0
SNORA2B	NC_000012	49061240	49061376	0	0	0	0
LOC100288297	NC_000012	49075007	49081832	0.1916347	0.1313471	0.4546229	0.6231807
LOC144438	NC_000012	49082245	49086229	0	0	0	0
CCNT1	NC_000012	49086752	49110781	5.322391	5.9998209	10.566087	7.25075
LOC255411	NC_000012	49121224	49159573	0	0	0	0
ADCY6	NC_000012	49159975	49182820	3.4381716	1.8955712	5.7087816	6.87208
LOC390314	NC_000012	49194354	49204388	0	0	0	0
CACNB3	NC_000012	49212512	49222724	4.0071405	3.2489177	2.1012406	1.5777828
DDX23	NC_000012	49223539	49245957	0.5515389	0.6776819	0.6941104	1.3779789
RND1	NC_000012	49250928	49259653	0.0531739	0.0820025	0.2601774	0.1482148
RPL32P27	NC_000012	49297198	49297596	0	0	0	0
CCDC65	NC_000012	49297893	49315359	0.0484278	0.1493666	0.301579	0.0674928
FKBP11	NC_000012	49315742	49319330	11.734868	9.158341	3.5676163	3.6649069
ARF3	NC_000012	49329992	49351252	26.565259	25.996105	33.29428	43.084364
WNT10B	NC_000012	49359123	49365543	0.0193519	0	0.0688641	0.067426
WNT1	NC_000012	49372236	49376396	0	0.0197826	0	0.0134084
DDN	NC_000012	49388933	49393088	0.0469532	0.0724093	0.0417709	0.0572581
PRKAG1	NC_000012	49396055	49412592	4.1903541	5.8393253	8.9378697	11.99665
MLL2	NC_000012	49412758	49449107	2.1590511	2.6385487	4.5965159	5.0434297
RHEBL1	NC_000012	49458468	49463775	1.344211	1.4203788	3.5875421	2.2376717
DHH	NC_000012	49483204	49488602	0.0454011	0.2333853	0.1615602	0.2056423
LMBR1L	NC_000012	49490922	49504680	4.1418163	5.6647847	4.0014566	3.2649124

LOC100288366	NC_000012	49521564	49542129	0.9654339	0.8199471	1.6804111	7.6635321
TUBA1B	NC_000012	49521565	49525304	362.71707	591.01849	506.37258	700.45892
LOC100289071	NC_000012	49524691	49582179	0.1494838	0.6915827	0.5984321	1.4062444
TUBA1A	NC_000012	49578582	49582861	63.759229	123.75262	60.852334	82.793789
LOC100289104	NC_000012	49580320	49582859	0.045448	0.140176	0.1617273	0.1583499
TUBA1C	NC_000012	49658865	49667113	401.75985	579.96519	508.49493	693.48686
PRPH	NC_000012	49688909	49692481	0.0719284	0.02465	0.0426596	0.1002451
TROAP	NC_000012	49716971	49725514	3.5904411	4.5943852	6.539109	8.1394312
C1QL4	NC_000012	49726200	49730971	0.0638473	0.0218806	0.3029351	0.1038131
DNAJC22	NC_000012	49741041	49745697	0.2867748	0.3931128	0.2551228	0.1665301
SPATS2	NC_000012	49760688	49921207	2.8828002	7.3083796	6.1180068	5.2520537
KCNH3	NC_000012	49932940	49952077	0.0570312	0.0938145	0.0304419	0.0635866
MCRS1	NC_000012	49952085	49961911	11.489005	17.889875	12.860519	21.06432
FAM186B	NC_000012	49981288	49999354	0.1222482	0.2042365	0.0815665	0.2449138
LOC100288407	NC_000012	49999323	50013398	0	0	0	0
PRPF40B	NC_000012	50017403	50038449	0.3795799	0.4024433	0.6542643	1.033228
FMNL3	NC_000012	50031724	50101197	2.1165205	3.794889	2.9972937	4.3890551
LOC100288440	NC_000012	50102785	50103165	0	0	0	0
TMBIM6	NC_000012	50135293	50158717	79.449917	74.776107	127.13699	131.92606
KIAA1602	NC_000012	50184929	50222208	3.004624	3.7622098	2.1463766	1.8062428
TRNAQ47P	NC_000012	50211190	50211264	0	0	0	0
BCDIN3D	NC_000012	50231556	50236875	1.3048058	1.46343	1.4773709	0.9918989
RPL35AP28	NC_000012	50256889	50257324	0	0	0	0
FAIM2	NC_000012	50260680	50297720	0.1588603	0.1537177	0.02494	0.0195353
LOC283332	NC_000012	50302665	50305646	0	0	0	0
AQP2	NC_000012	50344524	50352664	0.0105164	0.010812	0	0
LOC100131403	NC_000012	50344737	50356705	0	0	0	0
AQP5	NC_000012	50355279	50359461	0.0724024	0.0248124	0.0214704	0
AQP6	NC_000012	50366620	50370922	0.0165592	0.0510739	0.1031209	0.1269305
RACGAP1	NC_000012	50382945	50419307	11.689035	18.627213	17.734629	20.35461
ACCN2	NC_000012	50451487	50477394	0.5386913	0.9576666	0.1996811	0.1876908
SMARCD1	NC_000012	50478983	50494494	5.4727019	5.6001592	6.909631	8.9847266
GPD1	NC_000012	50497801	50505096	0.0305514	0.015705	0.0815382	0.117092
C12orf62	NC_000012	50505900	50514234	10.091275	16.614862	17.499576	17.172353
LASS5	NC_000012	50523581	50561097	8.5046501	7.7437328	9.859911	7.0296941
LIMA1	NC_000012	50569563	50677353	54.788278	34.814376	47.536954	61.821895
LOC100288501	NC_000012	50654851	50655624	0	0	0	0
LOC100288530	NC_000012	50686375	50691145	0	0	0	0
LOC100129228	NC_000012	50691347	50691718	0	0	0	0
FAM186A	NC_000012	50721095	50790405	0.0060493	0.018658	0.0215265	0.021077
RPL26P33	NC_000012	50759382	50759913	0	0	0	0
LARP4	NC_000012	50794650	50873788	1.7842521	2.231968	3.0297897	3.5172753
DIP2B	NC_000012	50898768	51142450	1.2808779	2.8411365	2.7455778	3.8478763
ATF1	NC_000012	51157819	51214907	4.1803976	4.4460912	7.2296032	6.6422963
TMPRSS12	NC_000012	51236701	51281667	0.0299988	0	0.0266878	0
METTL7A	NC_000012	51318534	51326300	2.4761393	1.0262897	2.2835746	3.6225888
LOC100288567	NC_000012	51342890	51346946	0	0	0	0
HIGD1C	NC_000012	51347782	51364289	0	0	0.1329849	0
SLC11A2	NC_000012	51379775	51420199	5.847099	4.8720815	13.098248	7.8474412
RNU7-39P	NC_000012	51404683	51404941	0	0	0	0
LETMD1	NC_000012	51442084	51454207	1.8552255	1.3139638	3.9244273	1.7524555
CSRNP2	NC_000012	51454990	51477333	4.120454	5.2169692	4.5230346	3.0357622
TFCP2	NC_000012	51488620	51566664	2.2974624	3.4287573	2.4065112	3.4730574
RPL35AP29	NC_000012	51500605	51500927	0	0	0	0
LOC494150	NC_000012	51517793	51519268	0	0	0	0
POU6F1	NC_000012	51580719	51611477	0.3460491	0.2019262	1.2979826	0.3584525
DAZAP2	NC_000012	51632508	51640501	22.806999	21.463316	39.956244	37.415942
SMAGP	NC_000012	51639133	51664202	1.5252413	2.9903462	2.3982365	2.9166551
BIN2	NC_000012	51674822	51717938	0	0	0.0353664	0.0277023
CELA1	NC_000012	51722227	51740463	0	0.0474616	0	0.2573519
GALNT6	NC_000012	51745833	51785200	0.8847985	0.3598678	0.717942	0.1219575
LOC100288040	NC_000012	51818555	51846046	0.2742479	0.7048893	1.0369089	0.9555344
SLC4A8	NC_000012	51818594	51902980	0.8761668	1.3398328	0.8318631	1.5699804
SCN8A	NC_000012	51985020	52202307	2.6192293	1.9288271	3.0616943	2.8948256
FLJ33996	NC_000012	52203159	52205359	0	0	0	0
FIGNL2	NC_000012	52211676	52216208	0.0290855	0.029903	0.0603757	0.1148517

LOC728503	NC_000012	52238457	52246513	0	0	0.0239422	0.0562613
ANKRD33	NC_000012	52281793	52285505	0.08865	0	0	0.0308874
ACVRL1	NC_000012	52301202	52317145	1.5168327	2.9342536	0.0887774	0.0208616
ACVR1B	NC_000012	52345486	52390860	1.1902646	1.3007343	1.4217297	0.8874255
GRASP	NC_000012	52400748	52409671	0.3184654	0.2806422	0.121421	0.0317028
NR4A1	NC_000012	52445191	52453287	1.6789212	1.1338157	1.0543164	0.7570195
C12orf44	NC_000012	52463758	52471279	11.743995	14.023519	11.291225	9.931241
OR7E47P	NC_000012	52501102	52502047	0	0	0	0
KRT80	NC_000012	52562780	52585784	0.5417348	0.1624467	1.94785	3.4683029
KRT7	NC_000012	52626954	52642709	1.7042205	28.08698	0.9418332	1.0976014
KRT121P	NC_000012	52643405	52653128	0	0	0	0
KRT122P	NC_000012	52657565	52662281	0	0	0	0
LOC100288636	NC_000012	52664359	52671517	0	0	0	0
KRT81	NC_000012	52679697	52685299	0.0230095	0.1182812	0.0818797	0.0320679
KRT86	NC_000012	52695649	52702947	0.0630534	0	0.2243763	0.1757525
KRT83	NC_000012	52708085	52715182	0.0234391	0	0	0.0326665
KRT123P	NC_000012	52734781	52748247	0	0	0	0
KRT85	NC_000012	52753790	52761309	0.0176428	0.0181387	0.0313911	0.0491768
LOC100129509	NC_000012	52766148	52796191	0	0	0	0.0172729
KRT84	NC_000012	52771596	52779417	0	0.0217961	0	0.0295464
KRT82	NC_000012	52787735	52800176	0	0	0.0145832	0
LOC100128683	NC_000012	52799432	52822278	0.0507485	0.0521748	0.1805892	0.1060908
KRT124P	NC_000012	52807645	52813024	0	0	0	0
KRT75	NC_000012	52817854	52828110	1.034076	0.0212628	5.0228873	0.4179395
KRT6B	NC_000012	52840435	52845910	0	0	0.017133	0.0268404
KRT6C	NC_000012	52862300	52867569	0.0383995	0	0	0.0133792
KRT6A	NC_000012	52880958	52887181	0.0179381	0	0	0.0125
LOC100129218	NC_000012	52890445	52919549	0.0238979	0	0.0212602	0.016653
KRT5	NC_000012	52908359	52914243	0	0	0.0169768	0
KRT71	NC_000012	52937693	52946903	3.3201838	0.7069374	2.0798437	13.854438
KRT74	NC_000012	52959603	52967609	0.0157634	0	0	0.0659072
KRT72	NC_000012	52979373	52995292	0.0221402	0	0	0.0154282
KRT73	NC_000012	53001354	53012343	0.0192418	0.0395652	0.034236	0.0402253
KRT2	NC_000012	53038342	53045959	0	0	0	0.0380591
KRT1	NC_000012	53068520	53074191	0.1075844	0.2580855	0.0957101	0.1249485
KRT77	NC_000012	53083410	53097247	0	0.0136712	0	0.0185325
KRT126P	NC_000012	53107922	53115587	0	0	0	0
LOC400036	NC_000012	53124209	53126649	0	0	0	0
LOC100128678	NC_000012	53140906	53143911	0	0	0	0
LOC643898	NC_000012	53157166	53157677	0	0	0	0
KRT76	NC_000012	53161939	53171105	0	0	0.0312156	0.0122255
KRT3	NC_000012	53183469	53189892	0.0190252	0	0.0169254	0.0530301
KRT4	NC_000012	53200333	53208335	0	0.0175401	0	0
LOC100288809	NC_000012	53200508	53208335	0.0518257	0	0.0230528	0.0541714
KRT79	NC_000012	53215231	53228077	0	0.02187	0.0189243	0.0296465
KRT78	NC_000012	53232746	53242778	0.0494356	0.0762375	0	0.0344487
RPL7P41	NC_000012	53279507	53280193	0	0	0	0
KRT8	NC_000012	53290971	53298868	0.0244701	1.106943	0.2394617	0.0682069
LOC100129362	NC_000012	53340879	53344388	0	2.1563666	0	0.0339899
KRT18	NC_000012	53342655	53346685	0.0286308	6.5641033	0	0.0399021
LOC100288147	NC_000012	53345526	53346695	0	0.2284875	0.049428	0
EIF4B	NC_000012	53400062	53435993	10.103293	15.28922	39.049033	79.451335
TENC1	NC_000012	53440810	53458163	7.1331361	4.1881538	10.834537	14.205232
SPRYD3	NC_000012	53458398	53473138	11.397789	10.680033	8.9674712	12.843845
IGFBP6	NC_000012	53491436	53496128	71.42725	73.715364	10.846944	6.5624739
SOAT2	NC_000012	53497274	53518323	0.1060015	0	0	0
LOC100127976	NC_000012	53525134	53532440	0	0	0	0
HIGD1D	NC_000012	53535744	53537088	0	0	0	0
LOC100129802	NC_000012	53536301	53541609	0	0	0	0.0567127
LOC728698	NC_000012	53547042	53548414	0	0	0	0
CSAD	NC_000012	53551447	53574430	0.9458247	0.4862036	0.9574914	0.8409057
ZNF740	NC_000012	53574535	53584654	1.5902321	1.4862961	3.4724812	3.3675853
ITGB7	NC_000012	53585107	53601000	0.1427406	0.0326116	0.126986	0.2210385
RARG	NC_000012	53604353	53626036	10.394395	7.6600195	11.24026	13.143101
MFSD5	NC_000012	53645880	53648188	12.510264	10.705386	14.839303	15.034031
ESPL1	NC_000012	53662083	53687427	1.4930322	2.1558138	3.837146	5.4101023

PFDN5	NC_000012	53689235	53693234	47.146793	66.09799	70.682935	49.614977
C12orf10	NC_000012	53693470	53700965	7.4151338	12.495163	11.616643	11.468576
AAAS	NC_000012	53701240	53715412	8.3494463	10.03529	8.4282177	9.6525882
SP7	NC_000012	53720359	53729537	0	0	0.0131465	0.0308926
SP1	NC_000012	53773979	53810230	4.8844049	4.7870236	9.5129626	15.260748
AMHR2	NC_000012	53817641	53825312	0.0236918	0	0.0421537	0.0990562
PRR13	NC_000012	53835433	53840427	19.050363	13.120201	22.378858	21.783386
PCBP2	NC_000012	53845886	53874946	29.645307	40.780478	43.560927	82.970145
MAP3K12	NC_000012	53874280	53893271	6.6889104	4.7950281	6.6828479	6.1814187
TARBP2	NC_000012	53894705	53900215	5.4382621	5.3410963	4.0513574	6.1927571
NPFF	NC_000012	53900474	53901422	0.0742369	0.0763233	0.0660432	0.4655809
ATF7	NC_000012	53905843	54020199	2.3147856	1.4050485	5.3867499	6.1149511
RPL31P51	NC_000012	54026519	54026962	0	0	0	0
ATP5G2	NC_000012	54058944	54070512	24.040058	27.348772	54.651531	62.130192
CALCOCO1	NC_000012	54104902	54121307	9.8977305	7.1498358	10.563787	12.034793
HOXC13	NC_000012	54332576	54340328	0.055027	0.8486031	0.0326357	0.038345
HOXC12	NC_000012	54348714	54350350	0	2.6077582	0	0
HOTAIR	NC_000012	54356096	54362515	0	0	0	0
HOXC11	NC_000012	54366910	54370203	2.2006478	2.5286735	0.6333921	0.4510291
HOXC10	NC_000012	54378946	54384063	6.9734184	5.7862418	6.3433805	7.9687183
HOXC9	NC_000012	54393877	54397121	2.6790751	3.3111053	1.2931101	1.5093974
HOXC8	NC_000012	54402890	54406547	3.8766563	3.7488412	2.3902442	2.6612885
HOXC4	NC_000012	54410642	54449814	1.7090979	1.2937128	0.9356682	2.1725341
HOXC6	NC_000012	54410642	54424607	0.7462166	1.4132431	1.3102401	0.8210423
HOXC5	NC_000012	54410642	54429145	0	0.028012	0.024239	0
FLJ12825	NC_000012	54452038	54516018	0	0	0	0
LOC100240735	NC_000012	54472623	54475607	0	0	0	0
LOC100240734	NC_000012	54495993	54496483	0	0	0	0
LOC400043	NC_000012	54519855	54526627	0	0	0	0
SMUG1	NC_000012	54575252	54582757	6.270321	6.9357961	7.3463579	10.904017
LOC100288249	NC_000012	54576044	54581693	0.3474169	0.1190603	0.1545358	0.080698
CBX5	NC_000012	54624730	54673915	1.8626526	3.8300053	4.4572806	8.6471155
LOC100132010	NC_000012	54625143	54632284	0	0	0	0
HNRNPA1	NC_000012	54674488	54679030	34.747641	49.24404	54.208521	85.797386
LOC100288977	NC_000012	54685468	54689084	0.0397362	0.040853	0.2121025	0.0276898
NFE2	NC_000012	54685895	54694791	0.2356474	0.1938162	0.9643367	0.3284169
COPZ1	NC_000012	54718911	54745633	27.429998	28.891653	67.333551	64.187937
GPR84	NC_000012	54756229	54758258	0	0.0301826	0.0261173	0.0613725
ZNF385A	NC_000012	54762920	54785083	0.3560003	0.2013031	1.3776785	1.7117186
ITGA5	NC_000012	54789045	54813050	103.39726	135.57147	93.548975	72.642211
GTSF1	NC_000012	54849736	54867386	0.0541234	0	0	0
NCKAP1L	NC_000012	54891495	54936899	0.0225781	0.0464253	0.0602583	0.0314666
LOC100289011	NC_000012	54943146	54963114	0.0565614	0	0	0.0394143
PDE1B	NC_000012	54943404	54973023	0.0138463	0.1281193	0	0.0771894
PPP1R1A	NC_000012	54973024	54982443	9.800887	8.5735136	4.860548	4.5920619
GLYCAM1	NC_000012	55001971	55004246	0	0	0	0
LACRT	NC_000012	55024623	55028663	0	0	0	0
DCD	NC_000012	55038375	55042149	0.0850063	0.0873954	0	0
VDAC5P	NC_000012	55196413	55196685	0	0	0	0
MUCL1	NC_000012	55248299	55252177	0	0	0	0
KIAA0748	NC_000012	55343865	55378456	0.0211799	0	0.0188422	0
NEUROD4	NC_000012	55413729	55423801	0.044437	0	0.0395324	0.0154827
OR9K1P	NC_000012	55509217	55510355	0	0	0	0
LOC644383	NC_000012	55515081	55520219	0	0	0	0
OR9K2	NC_000012	55523553	55524560	0.0435994	0	0.0775745	0
OR9R1P	NC_000012	55552531	55553684	0	0	0	0
OR10U1P	NC_000012	55587545	55588700	0	0	0	0
OR10A7	NC_000012	55614809	55615759	0	0	0	0.0322028
OR6C74	NC_000012	55641072	55642010	0.0468032	0	0.0832749	0.0652287
OR6C69P	NC_000012	55656323	55657218	0	0	0	0
OR6C72P	NC_000012	55677534	55678467	0	0	0	0
OR6C6	NC_000012	55688072	55689016	0	0.0478131	0	0
OR6C5P	NC_000012	55705619	55706541	0	0	0	0
OR6C1	NC_000012	55714384	55715322	0	0	0.0416375	0
OR6C3	NC_000012	55725485	55726420	0	0	0	0
OR6C7P	NC_000012	55736622	55737560	0	0	0	0

OR6C75	NC_000012	55758895	55759833	0.0468032	0.1443559	0.1249124	0.0652287
OR6C71P	NC_000012	55770485	55771620	0	0	0	0
OR6C66P	NC_000012	55782229	55783161	0	0	0	0
OR6C73P	NC_000012	55790060	55791057	0	0	0	0
OR6C65	NC_000012	55794313	55795251	0	0.0481186	0.0832749	0.0326144
PHC1B	NC_000012	55803470	55808727	0	0	0	0
LOC100288280	NC_000012	55807977	55808683	0.1967831	0.5395033	0.3501275	0.5027965
OR6C76	NC_000012	55820038	55820976	0	0	0.0416375	0
OR6C2	NC_000012	55845998	55846936	0	0	0	0
OR6C70	NC_000012	55862984	55863922	0	0	0	0
OR6C68	NC_000012	55886147	55887100	0.0460673	0	0	0
OR6C64P	NC_000012	55916377	55917255	0	0	0	0
OR6C4	NC_000012	55945011	55945940	0	0	0	0
OR2AP1	NC_000012	55968199	55969125	0	0	0	0
OR6U2P	NC_000012	56005407	56006338	0	0	0	0
OR10P1	NC_000012	56030676	56031617	0	0	0.0830097	0
OR10AE3P	NC_000012	56039879	56040547	0	0	0	0
PSMB3P	NC_000012	56048318	56049018	0	0	0	0
METTL7B	NC_000012	56075330	56078395	1.4591046	0.3600271	1.4019047	1.4641376
ITGA7	NC_000012	56078352	56106089	2.727478	0.2046813	6.5265925	3.9468982
BLOC1S1	NC_000012	56109820	56113487	18.439818	21.32783	30.006698	22.326179
RDH5	NC_000012	56114151	56118526	12.425155	1.2166062	10.702849	8.3605689
CD63	NC_000012	56119230	56122910	196.06011	150.12263	249.57856	140.41672
GDF11	NC_000012	56137064	56146665	0.5158243	0.9121532	0.2845128	0.3666359
SARNP	NC_000012	56146247	56211540	11.672272	15.935651	19.380621	15.279509
ORMDL2	NC_000012	56211806	56214959	5.6664887	8.467654	6.1547892	4.8898794
DNAJC14	NC_000012	56214744	56223420	5.5826132	6.1465709	6.6454121	6.7503485
LOC440104	NC_000012	56225380	56227719	0.9390648	0.5020378	0.4678341	0.471152
MMP19	NC_000012	56229214	56236735	2.0528984	2.6937861	3.4483716	1.6281819
LOC100128111	NC_000012	56263811	56266386	0	0	0	0
GSTPP	NC_000012	56294084	56294495	0	0	0	0
WIBG	NC_000012	56295197	56321697	3.2031235	4.2846329	5.7910971	6.3601824
DGKA	NC_000012	56324946	56347807	3.3806332	1.922698	1.6125346	0.6014706
SILV	NC_000012	56347889	56359826	0.1653743	0.2550333	0.2942432	0.1152394
CDK2	NC_000012	56360556	56366568	7.2256796	9.0051525	12.566466	17.001949
RAB5B	NC_000012	56367862	56388490	12.560447	12.557038	20.806168	25.012795
LOC100131294	NC_000012	56373743	56391573	0.2429757	0.0624511	0.5674146	0.698425
SUOX	NC_000012	56391043	56399309	2.0171203	2.440858	2.3026592	3.1221951
IKZF4	NC_000012	56414689	56432219	0.7981792	0.721144	0.6455294	1.2191522
RPS26	NC_000012	56435686	56438007	2.2554078	2.9813095	4.5288969	5.1191146
ERBB3	NC_000012	56473892	56497128	0.0443399	0.0455861	0.098615	0.0926934
PA2G4	NC_000012	56498103	56507694	11.748537	20.957115	26.814439	41.87888
RPL41	NC_000012	56510374	56511616	16.254825	18.64589	47.466051	33.351751
ZC3H10	NC_000012	56512030	56516278	1.9477293	2.769374	2.4885296	2.541244
FAM62A	NC_000012	56522054	56538455	17.857259	15.522999	26.686383	35.40864
MYL6B	NC_000012	56546335	56551771	15.677735	21.109852	31.359037	31.012535
MYL6	NC_000012	56552100	56555366	282.86574	334.14144	308.52854	413.43586
SMARCC2	NC_000012	56555636	56583351	5.1599139	4.2312216	6.3728826	7.6872164
RNF41	NC_000012	56598285	56615735	7.6076521	7.0078236	8.9482665	7.4093882
OBFC2B	NC_000012	56618125	56623638	6.9012213	9.4817417	15.65577	21.334676
SLC39A5	NC_000012	56623820	56631630	0.1041921	0	0.0556153	0.2758998
ANKRD52	NC_000012	56631591	56652143	4.4527032	4.677025	4.1509546	9.8993079
COQ10A	NC_000012	56660642	56664750	3.1408986	1.4018119	6.0216749	5.2596744
CS	NC_000012	56665483	56694175	17.691526	24.80836	24.238914	48.4791
CNPY2	NC_000012	56704217	56710120	9.9237943	14.687409	14.487766	10.360277
PAN2	NC_000012	56710718	56727825	1.7172247	1.1302856	2.8128908	2.8744459
IL23A	NC_000012	56732663	56734194	0.6748289	0.1734488	1.2757363	0.2057333
STAT2	NC_000012	56735381	56753909	38.467115	14.220623	39.892591	21.221532
RNU7-40P	NC_000012	56746174	56746233	0	0	0	0
APOF	NC_000012	56754355	56756583	0.0780608	0.1070063	0.0231484	0.0362639
LOC100128676	NC_000012	56772672	56776628	0	0	0	0
TIMELESS	NC_000012	56810157	56843200	3.4385343	5.2851741	4.9613882	8.0227883
MIP	NC_000012	56843884	56848429	0	0	0.0195098	0
SPRYD4	NC_000012	56862301	56864769	3.1704467	2.9593305	1.8927156	3.4447538
GLS2	NC_000012	56864736	56882181	0.318344	0.2411619	0.1937737	0.361941
LOC100288386	NC_000012	56874080	56882194	0.0765649	0	0	0

HSPD1P4	NC_000012	56904725	56906974	0	0	0	0
RBMS2	NC_000012	56915609	56989980	1.61582	1.3790362	1.6816653	3.0603225
BAZ2A	NC_000012	56989380	57030163	2.0217958	2.2455149	3.9473925	4.2814499
ATP5B	NC_000012	57031959	57039852	69.129639	85.022267	111.20425	123.44667
SNORD59B	NC_000012	57037464	57037538	0	0	0	0
SNORD59A	NC_000012	57038811	57038885	0	0	0	0
PTGES3	NC_000012	57057125	57082078	19.313367	24.478273	31.016858	67.227682
NACA	NC_000012	57106211	57119326	1.3708159	1.5002682	2.1411776	2.795285
PRIM1	NC_000012	57125364	57146146	8.7475619	13.381951	16.473393	10.452677
HSD17B6	NC_000012	57157108	57181574	0.2615966	0.5378976	0.439589	0.3848364
LOC100128944	NC_000012	57265059	57266188	0	0	0	0
SDR9C7	NC_000012	57317617	57328045	0	0	0	0
RDH16	NC_000012	57345215	57351418	0.076476	0.0524169	0.0680352	0.0532916
LOC390332	NC_000012	57375021	57379744	0	0	0	0
GPR182	NC_000012	57388355	57390469	0.055985	0.0575585	0.0747087	0.0975315
HBCBP	NC_000012	57390745	57391335	0	0	0	0
ZBTB39	NC_000012	57392618	57400230	0.7692721	0.9666465	1.2673441	0.9976662
TAC3	NC_000012	57403784	57410325	0	0	0.0958274	0
MYO1A	NC_000012	57422301	57443893	0.0486557	0.0875405	0.0108214	0.0169526
TMEM194A	NC_000012	57449426	57472574	5.9463515	6.9098348	15.835315	13.668608
NAB2	NC_000012	57482677	57489259	9.7590688	9.1333472	6.3456027	5.3206631
STAT6	NC_000012	57489191	57505161	35.255782	21.075746	30.214882	30.840384
LRP1	NC_000012	57522282	57607142	11.298868	12.683486	28.712644	16.104515
NXPH4	NC_000012	57610578	57620232	0.4141463	0.5259709	0.520145	0.4753307
SHMT2	NC_000012	57623552	57628714	28.52964	27.390415	36.970061	28.110568
NDUFA4L2	NC_000012	57628686	57634545	1.4797384	0.228199	0.5265665	0.4640133
STAC3	NC_000012	57637242	57644969	0.0534975	0	0.1427787	0.0559188
R3HDM2	NC_000012	57647548	57704246	1.680959	1.4439587	5.4897941	9.6868324
INHBC	NC_000012	57828543	57844609	0	0.0454105	0.0589411	0
INHBE	NC_000012	57849096	57851791	0.0901687	0.222487	0.6256894	0.1633662
GLI1	NC_000012	57853934	57866051	0.0731252	0.3633718	0.1517931	0.1358841
ARHGAP9	NC_000012	57866038	57873633	0.0147725	0	0.0394261	0.0308822
MARS	NC_000012	57881851	57910433	22.846791	23.375742	28.704188	33.320306
DDIT3	NC_000012	57910371	57914300	24.651533	17.011972	59.69782	15.026697
MBD6	NC_000012	57916659	57923931	1.5461306	1.3391707	2.9016988	8.5897249
DCTN2	NC_000012	57924094	57940978	31.609214	42.364334	45.647707	51.766836
KIF5A	NC_000012	57943847	57978554	0.0451098	0.1971049	0.1404583	0.0471515
PIP4K2C	NC_000012	57985057	57997031	6.0271002	4.403991	5.134564	6.221333
DTX3	NC_000012	57998604	58003583	4.4403093	3.7188902	1.6957052	1.3584224
GEFT	NC_000012	58003963	58011026	0.282222	0.3707521	0.237124	0.043703
SLC26A10	NC_000012	58013693	58019934	0.4743271	0.7502433	0.7628002	0.5085077
B4GALNT1	NC_000012	58019551	58026985	2.5259283	5.268895	5.9490648	3.3677192
LOC100289202	NC_000012	58019958	58022624	0.2839441	0.2919243	0.1403358	0.0439697
RPL13AP23	NC_000012	58068448	58069033	0	0	0	0
OS9	NC_000012	58087886	58115340	21.739055	19.75003	25.267036	19.204092
AGAP2	NC_000012	58118994	58135940	1.233545	1.6617976	0.2724569	0.3379052
LOC100130776	NC_000012	58120023	58122139	0	0	0	0
TSPAN31	NC_000012	58138784	58142026	1.3891682	1.6619182	2.0896975	2.2352641
CDK4	NC_000012	58142003	58146164	81.531127	107.24404	150.91715	160.99815
MARCH9	NC_000012	58148900	58153689	0.4105643	1.046082	0.0952825	0.1119512
CYP27B1	NC_000012	58156117	58160976	0.3710948	0.0908392	0.1100454	0.0492559
LOC100289240	NC_000012	58157908	58160294	0	0.0423859	0	0
METTL1	NC_000012	58162351	58165914	8.1036908	10.336524	5.3546016	5.8344259
FAM119B	NC_000012	58166383	58176324	1.8260181	1.7818806	3.0286847	1.9517968
TSFM	NC_000012	58176536	58191367	2.8605358	4.12176	3.8943335	4.4850044
AVIL	NC_000012	58191443	58209852	0.3242216	0.2833338	0.2884365	0.3501923
CTDSP2	NC_000012	58213710	58240747	20.048098	27.074038	33.04601	46.486369
LOC100289282	NC_000012	58273046	58273873	0	0	0.0654901	0
LOC100289313	NC_000012	58289528	58299022	0.1601028	0.1234519	0.3204718	0.1952406
XRCC6BP1	NC_000012	58335445	58351052	0.9850466	0.8958778	1.9885831	1.1880341
LOC338805	NC_000012	58368929	58536027	0	0	0	0
RPL21P103	NC_000012	58638127	58638687	0	0	0	0
LOC100127973	NC_000012	58639313	58788761	0	0	0	0
LRIG3	NC_000012	59265937	59314262	6.2480107	3.2608827	6.5964756	6.6749373
RPS6P22	NC_000012	59413925	59414706	0	0	0	0
LOC644915	NC_000012	59447099	59447965	0	0	0	0

SLC16A7	NC_000012	60083126	60175408	4.7162745	5.8313512	12.046158	12.912438
LOC100288586	NC_000012	60811803	60812015	0	0	0	0
LOC100289417	NC_000012	60812485	60813026	0	0	0	0
PGBD3P1	NC_000012	61624813	61626725	0	0	0	0
FAM19A2	NC_000012	62102040	62586551	0.0325061	0.0445596	0.0289183	0.0453031
RPL21P104	NC_000012	62169663	62170212	0	0	0	0
KRT8P19	NC_000012	62273025	62274753	0	0	0	0
RPS3P6	NC_000012	62415269	62415956	0	0	0	0
LOC100133321	NC_000012	62626856	62628967	0	0	0	0
USP15	NC_000012	62654187	62799898	5.7758899	4.879925	9.8697823	8.3751836
MON2	NC_000012	62860597	62991363	1.003539	1.0839837	2.1132801	2.9801645
C12orf61	NC_000012	62995531	62997214	0.5219505	0.456127	0.8358149	1.1093335
PPM1H	NC_000012	63037762	63328665	0.5173715	0.4007559	0.3341672	0.839579
RPL32P26	NC_000012	63052603	63053005	0	0	0	0
LOC100240709	NC_000012	63148933	63150213	0	0	0	0
RPL14P1	NC_000012	63359082	63359804	0	0	0	0
LOC121498	NC_000012	63397333	63398474	0	0	0	0
LOC100129941	NC_000012	63449055	63449545	0	0	0	0
AVPR1A	NC_000012	63540216	63546590	0.0105797	0.0217542	0	0
LOC100289542	NC_000012	63640764	63666116	0	0	0	0
DPY19L2	NC_000012	63952693	64062354	0.1624904	0.378663	0.5396755	0.8907409
RPS27P24	NC_000012	64154083	64154423	0	0	0	0
TMEM5	NC_000012	64173637	64202887	1.787006	3.425344	1.8053321	2.0050747
PABPCP5	NC_000012	64215723	64217806	0	0	0	0
SRGAP1	NC_000012	64238541	64541613	3.199535	4.6862785	5.9050011	7.494513
RPL36AP41	NC_000012	64265519	64265824	0	0	0	0
C12orf66	NC_000012	64586419	64616076	0.9803836	1.0426939	0.8120264	0.88341
LOC100289573	NC_000012	64615038	64616332	0.1469841	0	0.0980708	0.0256061
C12orf56	NC_000012	64660763	64784345	0.5254981	0.8530535	0.0492103	0.0578192
ATP6V1EL1	NC_000012	64731729	64732685	0	0	0	0
RPS11P6	NC_000012	64789790	64790780	0	0	0	0
XPOT	NC_000012	64798233	64842464	24.621463	33.245078	50.185991	51.344968
LOC100128392	NC_000012	64845371	64846681	0.0670454	0.206789	0.298227	0.1401596
TBK1	NC_000012	64845937	64895891	3.3160135	3.9092279	4.6675833	5.8435801
RASSF3	NC_000012	65004293	65089192	13.542761	8.2120917	15.06002	13.308193
LOC100289610	NC_000012	65089997	65100371	7.0027402	4.3859346	6.5878682	4.3188931
GNS	NC_000012	65107222	65153226	42.868381	32.327984	95.403245	66.642554
LOC100288653	NC_000012	65146486	65153207	0	0	0.0984825	0.1542815
LOC100289646	NC_000012	65174589	65212660	0.1612779	0	0.107608	0.0280962
TBC1D30	NC_000012	65218352	65271724	0.0462613	0.0760984	0.0329243	0.0773681
RPL7P39	NC_000012	65261668	65262500	0	0	0	0
WIF1	NC_000012	65444406	65515116	0.0218866	0.0225017	0.0389418	0.0457543
LOC100288706	NC_000012	65514188	65515296	0	0	0	0.0495548
LOC100131528	NC_000012	65527139	65529055	0	0	0	0
LEMD3	NC_000012	65563371	65642107	0.9356601	1.0762488	2.1180595	2.4337224
MSRB3	NC_000012	65672488	65860687	12.673491	11.742286	16.620059	19.590915
LOC729298	NC_000012	66039855	66040238	0	0	0	0
RPSAP52	NC_000012	66151800	66220754	0	0	0	0
HMGA2	NC_000012	66218240	66360071	4.9774305	7.3818836	14.58206	20.053348
RPL21P18	NC_000012	66430975	66431524	0	0	0	0
LLPH	NC_000012	66516849	66524533	0.6861269	1.2994405	0.9637855	1.8621537
TMBIM4	NC_000012	66530717	66563807	8.3249955	8.1857595	15.931827	9.4269311
IRAK3	NC_000012	66582978	66648402	1.3009604	1.8898618	3.5049112	1.5672127
RBMS1P	NC_000012	66626796	66629006	0	0	0	0
LOC390340	NC_000012	66669713	66670429	0	0	0	0
HELB	NC_000012	66696356	66731958	0.274024	0.3085565	0.7313381	0.4364591
GRIP1	NC_000012	66741223	67072925	0.0174121	0.0179015	0.3175515	0.2730031
GGTA1P	NC_000012	67659619	67660742	0	0	0	0
CAND1	NC_000012	67663061	67708388	14.036952	20.037324	31.301876	32.392496
LOC645328	NC_000012	67744021	67745911	0	0	0	0
DYRK2	NC_000012	68042512	68056444	2.9113295	2.9197912	3.5866414	4.7685108
RPL39P28	NC_000012	68395174	68395328	0	0	0	0
LOC341333	NC_000012	68409942	68432198	0	0	0	0
LOC100286966	NC_000012	68536359	68537475	0	0	0	0
IFNG	NC_000012	68548550	68553521	0	0.0373417	0	0
IL26	NC_000012	68595129	68619571	1.6370402	0	0.5974795	1.3747558

IL22	NC_000012	68642025	68647281	0	0	0	0.0534
MDM1	NC_000012	68688346	68726161	0.7249597	0.5640371	1.3857586	1.5155423
LOC100286998	NC_000012	68726304	68729486	0.1104574	0.0851714	0.0736995	0.0577283
LOC100288856	NC_000012	68863614	68881821	0	0	0.0564994	0
PSMC6P2	NC_000012	68867505	68868701	0	0	0	0
RPSAP12	NC_000012	68946705	68947738	0	0	0	0
RAP1B	NC_000012	69004652	69054374	14.246152	14.818097	14.78916	11.904022
RPL10P12	NC_000012	69020590	69020967	0	0	0	0
LOC246723	NC_000012	69036223	69036818	0	0	0	0
RPL7P42	NC_000012	69079560	69080088	0	0	0	0
NUP107	NC_000012	69080731	69136473	5.4302233	9.4734726	12.327497	14.17528
SLC35E3	NC_000012	69139936	69159853	2.4155611	1.73264	0.8495849	1.7745988
LOC100130075	NC_000012	69198784	69199257	0	0	0	0
MDM2	NC_000012	69201971	69239212	4.1559869	2.206292	3.4869096	4.0083409
CPM	NC_000012	69244955	69357020	1.0583638	3.7815156	6.7361496	4.060858
RNU7-4P	NC_000012	69305602	69305861	0	0	0	0
CPSF6	NC_000012	69633317	69668138	5.4470299	8.0791966	7.9660699	13.229429
LOC729409	NC_000012	69678177	69679270	0	0	0	0
LYZ	NC_000012	69742134	69748013	0	0.0298044	0.02579	0.0606033
YEATS4	NC_000012	69753532	69784576	2.8558418	2.9361055	4.4319984	5.2405026
RPS26P45	NC_000012	69816457	69816775	0	0	0	0
FRS2	NC_000012	69864129	69973562	3.9156764	3.9996282	4.172289	4.2366258
CCT2	NC_000012	69979208	69995357	40.147182	67.220138	54.304398	113.44057
LRRC10	NC_000012	70002344	70004942	0.0169097	0.0173849	0	0.03535
BEST3	NC_000012	70047389	70093196	0.082278	0.0241687	0.0313701	0.0573346
RAB3IP	NC_000012	70132631	70216984	0.127132	0.1802829	1.3845023	0.8370291
C12orf28	NC_000012	70219298	70352504	0.0706109	0.0290382	0.0502539	0.0688863
LOC100125409	NC_000012	70507543	70508088	0	0	0	0
CNOT2	NC_000012	70636777	70748773	7.2353796	8.4030107	9.8816713	11.297592
KCNMB4	NC_000012	70760062	70824978	0.3502925	1.0527095	0.359573	0.1126605
PTPRB	NC_000012	70910630	71031219	0.635257	0.3879083	0.558292	1.250214
PTPRR	NC_000012	71031862	71314584	0.197965	0.1556397	0.5283455	0.4219644
FAHD2P	NC_000012	71065569	71066829	0	0	0	0
TSPAN8	NC_000012	71518877	71551779	0.0388922	0.1599412	0	0.081305
LGR5	NC_000012	71833813	71978622	0.0457794	0.1882642	0.0271511	0.0744355
ZFC3H1	NC_000012	72003379	72057749	0.7490478	0.706977	1.305437	2.2675587
THAP2	NC_000012	72057784	72074419	0.1860636	0.2008576	0.6538331	0.376004
LOC100289049	NC_000012	72057805	72059220	0.0777157	0.1198499	0.034569	0.2166216
LOC100289020	NC_000012	72079867	72083418	0	0	0.050579	0.0396182
TMEM19	NC_000012	72079878	72097840	1.7828323	2.3954283	2.1399183	4.4763988
RAB21	NC_000012	72148658	72181150	5.8553031	7.8602264	7.1587092	6.9363542
LOC100287059	NC_000012	72228807	72229184	0	0	0	0
TBC1D15	NC_000012	72233539	72318095	7.7902006	10.021988	14.421034	12.946405
MRS2P2	NC_000012	72242072	72244763	0	0	0	0
TPH2	NC_000012	72332626	72426221	0.0372443	0.0191455	0.0497003	0.0519066
LOC283392	NC_000012	72647287	72667289	0	0	0	0
TRHDE	NC_000012	72666529	73059422	0.0079157	0.3987728	0.0211262	0.0386121
RPL31P48	NC_000012	74042420	74042866	0	0	0	0
LOC100128674	NC_000012	74422463	74565610	0	0	0	0
VENTXP3	NC_000012	74686093	74688112	0	0	0	0
LOC552889	NC_000012	74931551	74935232	2.2678338	3.0801287	3.78021	3.6264114
LOC387869	NC_000012	75057545	75057921	0	0	0	0
LOC100128673	NC_000012	75121809	75122627	0	0	0	0
KCNC2	NC_000012	75433896	75603511	0.0240549	0	0.0213999	0.0167624
LOC100128681	NC_000012	75645379	75652531	0	0	0	0
CAPS2	NC_000012	75669759	75723836	0.061754	0.0423264	0.2563775	0.2868841
GLIPR1L1	NC_000012	75728463	75764170	0.0913685	0	0.2438518	0.1910076
GLIPR1L2	NC_000012	75784889	75817820	0	0.0522654	0.0226128	0.0885624
GLIPR1	NC_000012	75874513	75895716	29.516294	9.9572329	10.227211	18.694736
KRR1	NC_000012	75891419	75905418	5.315157	5.7471887	9.3172632	12.589315
LOC100129649	NC_000012	75993827	75995336	0	0	0	0
RPL10P13	NC_000012	76082505	76082876	0	0	0	0
LOC100289143	NC_000012	76332785	76371830	0	0	0.1810072	0.1417818
LOC100131830	NC_000012	76407814	76425158	32.06364	30.164694	74.230308	51.976026
PHLDA1	NC_000012	76419227	76425556	17.97916	3.4921046	39.302542	23.715765
LOC100289208	NC_000012	76425609	76427712	0.3133191	0.04295	0.6318048	0.6258887

NAP1L1	NC_000012	76438672	76478738	6.088438	7.3086908	14.92192	24.780755
LOC100289249	NC_000012	76449904	76452402	0	0	0	0
LOC641695	NC_000012	76531205	76532395	0	0	0	0
BBS10	NC_000012	76738266	76742222	0.8340719	1.3241019	2.5424875	2.2308382
OSBPL8	NC_000012	76745577	76953589	7.5492406	8.3977988	9.2048259	10.686146
RPL7AP59	NC_000012	76993432	76994309	0	0	0	0
RPL21P98	NC_000012	77054145	77054708	0	0	0	0
RPL7P43	NC_000012	77115298	77116135	0	0	0	0
RWDD1L1	NC_000012	77150973	77151872	0	0	0	0
ZDHHC17	NC_000012	77157854	77247474	2.4594798	2.9737137	3.2861295	3.8257027
CSRP2	NC_000012	77252496	77272799	2.1461956	3.0590317	4.8166812	3.8068661
LOC727803	NC_000012	77377731	77378449	0	0	0	0
E2F7	NC_000012	77415026	77459360	0.4210334	0.6296241	0.7899874	2.3578116
NAV3	NC_000012	78225069	78606790	4.4092354	4.2229209	5.3209231	4.9744243
MRPL11P3	NC_000012	78867094	78867203	0	0	0	0
LOC642550	NC_000012	79187301	79187894	0	0	0	0
SYT1	NC_000012	79257773	79845788	0.7920087	0.6276651	0.7853275	0.2989476
LOC338756	NC_000012	79939855	79952951	0	0	0	0.122255
PAWR	NC_000012	79985745	80084790	3.4014264	2.7790919	2.9458443	3.8614659
PPP1R12A	NC_000012	80167343	80329235	1.7892786	2.379274	1.532593	3.5189758
LOC100289425	NC_000012	80382776	80382967	0	0	0	0
RPL7P38	NC_000012	80422657	80423480	0	0	0	0
RPL26P32	NC_000012	80496639	80497137	0	0	0	0
C12orf64	NC_000012	80613536	80771828	0.0650796	0.0133817	0.0868449	0.0952351
PTPRQ	NC_000012	80838126	81073968	0.1096542	0.0118669	0.0205371	0.0361949
MYF6	NC_000012	81101446	81103251	0	0	0.0302145	0
MYF5	NC_000012	81110708	81113447	0.0578266	0	0	0
LIN7A	NC_000012	81191171	81331694	3.573027	0.8081584	0.349653	0.1493897
ACSS3	NC_000012	81471809	81649582	1.6228823	0.6405824	0.7863342	0.1211667
PPFIA2	NC_000012	81653356	82153109	0.1518448	0.0111509	0.1254364	0.1511593
CCDC59	NC_000012	82746089	82752199	1.3094445	1.7108551	2.0628759	2.5663306
C12orf26	NC_000012	82752324	82872945	0.3115318	0.2745321	0.4751097	0.5582256
TMTC2	NC_000012	83080934	83528065	0.2088449	0.5227832	0.6785528	0.7213298
RPL6P25	NC_000012	83545090	83546011	0	0	0	0
LOC100128335	NC_000012	84842391	84843657	0	0	0	0
LOC100287299	NC_000012	85062822	85065807	0	0	0	0
SLC6A15	NC_000012	85253267	85306606	1.9224899	3.1520623	11.338843	8.5769969
LOC100289485	NC_000012	85285711	85306655	0.0593094	0.0609763	0.1055265	0.2066456
TSPAN19	NC_000012	85408094	85430055	0	0	0.2299857	0.0900732
LRRIQ1	NC_000012	85430099	85638883	0.0531969	0.0390657	0.0743685	0.1482789
ALX1	NC_000012	85674036	85695562	0.0679262	0.279341	2.2358732	1.9643469
LOC441643	NC_000012	85961656	85962020	0	0	0	0
LOC100129589	NC_000012	86175666	86188881	0	0	0	0
RASSF9	NC_000012	86198331	86230318	0.7096318	1.8113613	1.1973085	1.671068
NTS	NC_000012	86268075	86276767	0	0.1464616	0.0316836	0.0248176
MGAT4C	NC_000012	86373037	87232681	0	0.0233627	0.0202159	0.047505
RPL23AP68	NC_000012	87563813	87574610	0	0	0	0
CYCSP30	NC_000012	88140324	88144091	0	0	0	0
MKRNP6	NC_000012	88176662	88178488	0	0	0	0
RPS4P15	NC_000012	88338663	88344595	0	0	0	0
C12orf50	NC_000012	88373816	88423176	0.0515522	0	0.0458622	0
C12orf29	NC_000012	88429268	88443937	2.2019991	3.6473727	5.1558724	4.7205362
CEP290	NC_000012	88442790	88535993	0.2762302	0.2215151	0.4226764	1.047136
TMTC3	NC_000012	88536073	88593664	4.4051955	6.4735903	6.1227341	8.28228
KITLG	NC_000012	88886566	88974238	7.6414129	5.9939711	5.7549315	7.5787417
LOC100287355	NC_000012	89420862	89422315	0	0	0	0
MRPS6P4	NC_000012	89713076	89713383	0	0	0	0
DUSP6	NC_000012	89741837	89746296	14.477981	6.5051281	12.422489	1.8353207
LOC100287398	NC_000012	89744664	89746274	0	0.2284875	0	0.0387167
WDR51B	NC_000012	89813501	89919777	1.3165239	1.8553938	2.8819814	2.8243745
CENPC2	NC_000012	89893713	89896850	0	0	0	0
GALNT4	NC_000012	89913189	89918535	0	0.0084502	0	0
ATP2B1	NC_000012	89981826	90049844	13.990155	9.5997622	14.696128	13.499153
LOC338758	NC_000012	90101527	90105546	0.8350912	0.5766458	0.3770043	0.625352
LOC643153	NC_000012	90103300	90105729	0.0361714	0.018594	0.0804477	0
MRPL2P1	NC_000012	90146688	90147644	0	0	0	0

LOC100287505	NC_000012	90313195	90313672	0	0	0	0
C12orf12	NC_000012	91345992	91348953	0	0.0152544	0.0131997	0.0206785
EPYC	NC_000012	91357456	91398803	0.1427818	0.2348715	0.0762136	0.0596976
KERA	NC_000012	91444268	91452131	0.0173503	0.0356758	0.0154353	0.0362711
LUM	NC_000012	91497232	91505542	73.295515	24.278605	62.378725	22.534469
DCN	NC_000012	91539035	91576806	104.51937	82.046863	57.080264	28.598681
BTG1	NC_000012	92534054	92539673	9.1277097	4.5762676	17.243029	17.327923
RPL21P106	NC_000012	92562142	92562693	0	0	0	0
CLLU1OS	NC_000012	92813870	92821924	0.0640645	0	0	0.0892854
CLLU1	NC_000012	92817735	92824730	0	0	0	0
LOC100132126	NC_000012	92924301	92925209	0	0	0	0
C12orf74	NC_000012	93096840	93102065	0	0	0.0283933	0
PLEKHG7	NC_000012	93130265	93165868	0.0403565	0.0138302	0	0.074992
EEA1	NC_000012	93166285	93323107	2.712786	2.5462594	4.6410955	6.0231861
LOC100287544	NC_000012	93177490	93253051	0	0	0	0
LOC728170	NC_000012	93277550	93278480	0	0	0	0
LOC100127919	NC_000012	93429041	93429523	0	0	0	0
LOC100287580	NC_000012	93477044	93492820	0.0681368	0.2101554	1.0304785	0.4748043
RPL41P5	NC_000012	93477069	93477492	0	0	0	0
LOC643339	NC_000012	93517835	93534554	0	0	0	0
NUDT4	NC_000012	93771701	93797024	6.510511	9.6037025	5.8975343	5.3957816
UBE2N	NC_000012	93802088	93836026	13.208579	13.491282	18.59892	23.208666
MRPL42	NC_000012	93861270	93896432	4.9716168	7.6780805	14.091973	11.668049
SOCS2	NC_000012	93963598	93969978	1.3522533	0.6337943	0.3715153	1.1640225
LOC642969	NC_000012	94033679	94035398	0	0	0	0
CRADD	NC_000012	94071151	94244532	2.3286279	1.5960495	4.9652922	4.6877442
LOC100287069	NC_000012	94243740	94250926	0.1648059	0	0.0977439	0.3445299
LOC441644	NC_000012	94533943	94534926	0.0548667	0.0564087	0	0
PLXNC1	NC_000012	94542499	94699225	0.3004274	0.0352995	0.1450886	0.0598142
CCDC41	NC_000012	94702056	94853764	0.2725472	0.4203107	0.351575	0.8926321
RBMS2P	NC_000012	94817244	94818826	0	0	0	0
LOC144486	NC_000012	94853779	94856346	0	0	0	0
LOC400061	NC_000012	94914866	94915647	0	0	0	0
LOC283398	NC_000012	94940745	94944333	0	0	0.0442113	0.0692609
TMCC3	NC_000012	94960900	95044324	0.0974462	0.0231196	0.6201729	0.365639
KRT19P2	NC_000012	95228200	95228834	0	0	0	0
NDUFA12	NC_000012	95365109	95397511	17.65254	25.980456	23.197889	20.875959
NR2C1	NC_000012	95414057	95467404	1.7849985	2.6418325	2.4866785	2.966123
FGD6	NC_000012	95470525	95611240	0.8801002	1.5713005	2.4330742	2.1036469
LOC100287148	NC_000012	95494281	95494693	0	0	0	0
RPL14P4	NC_000012	95519511	95520199	0	0	0	0
VEZT	NC_000012	95611522	95696566	2.1001128	2.4463613	2.6139319	3.020867
RPL29P26	NC_000012	95861038	95861652	0	0	0	0
METAP2	NC_000012	95867822	95909615	6.3803908	7.9257821	8.1183764	10.560604
USP44	NC_000012	95910879	95945263	0.0742787	0.0381832	0	0.0345069
LOC100132594	NC_000012	96043031	96067775	0	0	0	0
NTN4	NC_000012	96051583	96184536	9.2516195	8.4617325	3.5366263	7.1415691
SNRPF	NC_000012	96252709	96260238	7.2600484	8.8885381	7.8885378	5.2908049
CCDC38	NC_000012	96260827	96336428	0.0225029	0.0231354	0.0800769	0.0313619
AMDHD1	NC_000012	96337071	96362370	0.5966754	0.5499852	0.0915205	0.05735
HAL	NC_000012	96367142	96390071	0.0280014	0	0.0124554	0.0195125
LTA4H	NC_000012	96394611	96429365	7.1682553	9.9578954	13.589251	18.910119
LOC100132930	NC_000012	96453802	96454153	0	0	0	0
ELK3	NC_000012	96588207	96661606	4.4745062	6.2597182	6.2708787	8.2292792
PCTK2	NC_000012	96672040	96794223	4.4046112	8.0728573	6.0179871	4.6986945
C12orf55	NC_000012	96883388	97022570	0.1061551	0.0970121	0.3147952	0.2383579
C12orf63	NC_000012	97041753	97159048	0.0476921	0.0612906	0.0530352	0.0332337
LOC387876	NC_000012	97174967	97296229	0	0	0	0
LOC390352	NC_000012	97294462	97296476	0	0	0	0
NEDD1	NC_000012	97301001	97347469	3.2494666	4.0574651	7.7851668	7.6450099
TRNAQ46P	NC_000012	97490691	97490764	0	0	0	0
RMST	NC_000012	97858799	97927544	0	0	0	0
LOC643711	NC_000012	98125958	98127147	0	0	0	0
LOC100131529	NC_000012	98711456	98712163	0	0	0	0
LOC121456	NC_000012	98848381	98877977	0	0	0	0
LOC100128191	NC_000012	98906751	98910004	0	0	0	0

TMPO	NC_000012	98909409	98942735	13.675434	19.802224	25.693696	27.750766
PPIAP8	NC_000012	98984291	98984983	0	0	0	0
SLC25A3	NC_000012	98987403	98995778	41.337659	59.712184	71.323787	85.92105
SNORA53	NC_000012	98993413	98993662	0	0	0	0
IKIP	NC_000012	99007182	99038589	5.5348123	7.7862043	7.7607503	7.5391641
APAF1	NC_000012	99039078	99129211	1.2467583	1.7844648	2.2889828	2.1804947
ANKS1B	NC_000012	99129071	100378432	0.2389975	0.0702042	0.413088	0.5710046
FAM71C	NC_000012	100041535	100043827	0	0	0	0
RPS4P1	NC_000012	100402765	100403672	0	0	0	0
UHRF1BP1L	NC_000012	100430863	100536642	2.4572857	3.5485217	4.8395552	7.4914637
DNM1P19	NC_000012	100547374	100550137	0	0	0	0
GOLGA2L1	NC_000012	100550190	100567335	0.0775785	0.0398794	0.034508	0.1621794
LOC100287305	NC_000012	100557018	100558358	0	0	0	0.0228373
ACTR6	NC_000012	100594574	100618201	2.479131	3.4756463	4.9011193	4.2403678
DEPDC4	NC_000012	100646041	100660857	0.2283025	0.3911983	0.1354028	0.3181806
LOC100287334	NC_000012	100661162	100676922	2.0556431	2.5506759	1.387333	1.0372943
SCYL2	NC_000012	100661549	100733917	8.4040748	10.56297	11.101778	11.052951
SLC17A8	NC_000012	100750857	100815837	0.0551698	0.0680644	0.0392644	0.0384445
NR1H4	NC_000012	100867679	100957643	0.0615234	0	0	0.0714533
GAS2L3	NC_000012	100967489	101018690	2.4488942	1.9948094	7.0720843	5.9070704
PIGAP1	NC_000012	100972422	100976450	0	0	0	0
ANO4	NC_000012	101188374	101522419	0.0640023	0.9979829	0.2657116	0.133798
LOC728467	NC_000012	101460383	101463780	0	0	0	0
SLC5A8	NC_000012	101549994	101604016	0.0401232	0.0550011	0	0.0279594
RPS6P19	NC_000012	101621878	101622695	0	0	0	0
UTP20	NC_000012	101673905	101780397	0.6828804	1.3389532	0.6986357	2.3283065
ARL1	NC_000012	101786914	101801572	14.631093	15.70614	21.534827	19.089093
RPS27P23	NC_000012	101818187	101818529	0	0	0	0
SPIC	NC_000012	101870657	101880775	0	0	0	0.0306555
MYBPC1	NC_000012	101988747	102079658	0	0	0.0195978	0.038377
CHPT1	NC_000012	102091417	102122846	7.0676161	5.2766832	10.154888	12.058424
SYCP3	NC_000012	102122426	102133247	0.0783391	0.0402704	0.2090779	0.1637694
GNPTAB	NC_000012	102139277	102224632	10.268937	6.7674516	13.215882	15.080639
LOC100287369	NC_000012	102266973	102267427	0	0	0	0
DRAM	NC_000012	102271105	102317401	43.191573	10.737913	85.654898	30.815041
RPL9P24	NC_000012	102329994	102330452	0	0	0	0
LOC100129880	NC_000012	102357770	102358249	0	0	0	0
CCDC53	NC_000012	102406717	102455899	1.2515378	1.5836461	2.2696287	3.6897444
NUP37	NC_000012	102467970	102512361	5.2388621	7.9295374	11.295572	10.799833
C12orf48	NC_000012	102513956	102591298	1.7364306	3.0887368	3.1508543	4.1966359
PMCH	NC_000012	102590237	102591614	0.1161116	0.2387498	0.1549441	0.485467
IGF1	NC_000012	102789645	102874378	0.0168729	0.0115647	0.0200141	0.0195962
PAH	NC_000012	103232104	103311381	0	0	0.0291773	0.0114272
ASCL1	NC_000012	103351452	103354294	0.035385	0	0.0314795	0.0369866
C12orf42	NC_000012	103695733	103889749	0.0300604	0.0309052	0	0.0209472
LOC644203	NC_000012	103897474	103898714	0	0	0	0
STAB2	NC_000012	103981069	104160502	0.03204	0.0164703	0.0095012	0.0632592
NT5DC3	NC_000012	104166081	104234975	1.8702671	1.534507	2.7044199	2.6532505
LOC253724	NC_000012	104237527	104323989	0	0	0	0
HSP90B1	NC_000012	104324189	104341703	44.501537	48.450259	100.48637	85.80474
C12orf73	NC_000012	104343980	104350993	0.6485867	0.7735057	1.1078413	1.3378046
TDG	NC_000012	104359593	104382656	1.6121577	1.5878261	3.5072107	3.7573063
GLT8D2	NC_000012	104382765	104443915	5.994005	8.1363824	1.1456399	0.6363187
LOC643576	NC_000012	104424519	104426242	0	0	0	0
HCFC2	NC_000012	104458236	104500302	0.6822593	0.8827038	1.2002741	1.8108902
NFYB	NC_000012	104510858	104532040	1.0728316	1.6998925	1.92007	2.5506073
TXNRD1	NC_000012	104609559	104744062	28.842	23.941973	48.103029	67.457209
RPL18AP3	NC_000012	104659048	104659663	0	0	0	0
LOC100130902	NC_000012	104680460	104683185	0	0	0	0
EID3	NC_000012	104697549	104698983	0	0	0	0
CHST11	NC_000012	104850778	105151883	4.4427087	1.805148	6.2953707	4.5789013
SLC41A2	NC_000012	105197275	105322472	0.6324379	0.5992155	1.003916	1.356689
KRT18P20	NC_000012	105370462	105371855	0	0	0	0
C12orf45	NC_000012	105380098	105388505	4.3100356	8.8623393	8.3600904	4.0865995
ALDH1L2	NC_000012	105418202	105478236	4.2469658	5.2395928	4.1700338	2.2798764
KIAA1033	NC_000012	105501492	105562908	7.0220315	6.6359232	9.8215131	10.097562

APPL2	NC_000012	105567075	105630008	8.8984965	15.373548	8.6426198	12.031879
C12orf75	NC_000012	105724414	105765296	7.8180237	19.713988	6.9551306	6.9724284
FAM10A3	NC_000012	106409591	106412186	0	0	0	0
NUAK1	NC_000012	106457125	106533811	1.9329233	4.5441744	2.6768162	2.4424474
LOC341378	NC_000012	106565157	106566215	0.0414997	0	0	0.0578374
CKAP4	NC_000012	106631659	106641713	121.56695	140.44656	147.72338	128.06767
LOC100130905	NC_000012	106632225	106634225	0.6116992	0.3386336	0.8790673	0.3606784
TCP11L2	NC_000012	106696581	106740793	2.8717862	2.0362056	5.8144194	5.0788442
POLR3B	NC_000012	106751489	106903975	1.489594	2.6452478	1.4363884	2.7075325
LOC100287944	NC_000012	106889736	107168690	0.389612	0.7210117	0.2772877	0.380096
RFX4	NC_000012	106977033	107156581	0.0099724	0.0102526	0	0.0069491
RIC8B	NC_000012	107168399	107283094	0.7223401	0.9056605	1.2225336	1.7985747
LOC100130631	NC_000012	107295061	107296116	0	0	0	0
RPL30P12	NC_000012	107304535	107304890	0	0	0	0
LOC100287486	NC_000012	107347807	107349465	0.1854356	0.1634119	0.2121025	0.2953575
C12orf23	NC_000012	107349544	107367813	19.958681	18.467659	23.82526	21.752845
MTERFD3	NC_000012	107371069	107380929	0.5199509	0.6872968	1.8061974	1.7253453
CRY1	NC_000012	107385142	107487598	4.599861	3.689007	3.8041523	5.3955433
BTBD11	NC_000012	107712197	108053419	0.022305	0.0535077	0.0330719	0.025905
PWP1	NC_000012	108079590	108106257	6.0242045	8.9976661	6.2665823	7.139745
RPL17P38	NC_000012	108113341	108113940	0	0	0	0
PRDM4	NC_000012	108126643	108154914	8.1766593	9.0539466	9.3098335	13.633813
ASCL4	NC_000012	108168162	108170421	0.0194461	0	0.0172998	0.0135508
LOC390358	NC_000012	108274835	108277484	0	0	0	0
LOC644746	NC_000012	108281507	108283544	0	0	0	0
LOC728739	NC_000012	108296927	108297725	0	0	0	0
LOC100287556	NC_000012	108304180	108306397	0	0	0	0
WSCD2	NC_000012	108523511	108644314	0.0284393	0	0	0.0198177
LOC196469	NC_000012	108674859	108676141	0	0	0	0
CMKLR1	NC_000012	108681821	108733094	2.2075884	0.024145	0.0208929	0.0381856
FICD	NC_000012	108909051	108913380	1.4906729	1.3683647	0.8051588	1.1129574
SART3	NC_000012	108915991	108955165	0.5732378	0.7134221	0.8320535	1.3595484
ISCU	NC_000012	108956294	108963160	13.675281	16.678954	37.09769	21.748102
TMEM119	NC_000012	108983622	108991894	10.901635	12.479385	1.5922741	1.2472183
SELPLG	NC_000012	109015671	109027670	1.1233327	1.1371363	1.3375888	1.5294375
CORO1C	NC_000012	109038885	109125295	62.725589	68.598234	63.867898	117.43417
SSH1	NC_000012	109180970	109251351	10.738531	10.883101	9.5338583	15.33147
DAO	NC_000012	109273857	109294710	0	0	0	0.038377
SVOP	NC_000012	109304658	109372453	0.042285	0.0289823	0.0125393	0.265193
LOC100131733	NC_000012	109489846	109491770	0.1797474	0.0923996	0.1199312	0.0313138
USP30	NC_000012	109490380	109525831	1.3349583	2.0466769	2.4273202	2.0318664
ALKBH2	NC_000012	109525993	109531293	3.0840864	5.4355972	6.3039517	7.4195612
UNG	NC_000012	109535415	109548798	4.926236	4.4864366	6.4357423	8.9604123
ACACB	NC_000012	109577202	109706031	0.3705905	0.3761213	0.7016428	0.7217539
FOXN4	NC_000012	109715783	109747025	0	0.0404386	0.0233279	0.0274089
MYO1H	NC_000012	109826488	109886178	0.0230881	0.0118685	0.0616195	0.0563105
KCTD10	NC_000012	109886460	109915155	0.9741674	1.2746955	2.5211528	7.089235
LOC100287749	NC_000012	109888546	109894121	0.1746287	0.1196911	0.0517849	0.8518178
UBE3B	NC_000012	109915439	109974507	2.8301741	3.2014766	4.2986852	7.8566442
MMAB	NC_000012	109991520	110011358	1.8369278	1.2663142	3.4950711	3.4109855
MVK	NC_000012	110011500	110035071	3.8372552	6.4298618	5.7524156	8.9082497
MGC14436	NC_000012	110171130	110211292	0	0	0	0
C12orf34	NC_000012	110172572	110208299	0.2092007	0.6286962	0.1431621	0.2579173
TRPV4	NC_000012	110220894	110271212	0.339421	0.5862536	0.7850917	0.7947142
GLTP	NC_000012	110288748	110318293	13.654544	15.169809	15.848122	30.091933
TCHP	NC_000012	110338079	110355874	0.4248772	0.4095172	0.4842901	0.767935
GIT2	NC_000012	110367607	110434194	1.6175581	2.0012611	1.5060977	3.9069168
ANKRD13A	NC_000012	110437235	110477237	16.073138	23.134826	15.629027	32.105211
C12orf76	NC_000012	110478983	110505500	0.4742615	0.3018418	1.1251098	0.6767059
IFT81	NC_000012	110562140	110656600	1.0978205	1.5655812	2.3418633	2.8132443
ATP2A2	NC_000012	110719032	110788898	21.649063	24.387626	20.928835	22.094846
LOC100287423	NC_000012	110777306	110778799	0	0.0806846	0.0698171	0.0546873
ANAPC7	NC_000012	110810705	110841535	8.6352728	9.373944	10.600547	17.816889
HMGA1L3	NC_000012	110866403	110868198	0	0	0	0
ARPC3	NC_000012	110872706	110888158	46.881428	62.612746	88.234891	71.954607
GPN3	NC_000012	110890291	110906064	3.4941706	5.512437	6.9137573	5.7513479

RPL31P49	NC_000012	110898765	110899206	0	0	0	0
C12orf24	NC_000012	110906232	110928192	2.5715928	3.5647654	4.8839826	4.892732
VPS29	NC_000012	110929330	110939916	5.4685811	6.2788924	12.428836	10.236108
RAD9B	NC_000012	110940005	110969891	0.7693855	0.4074896	0.6844667	0.6011249
PPTC7	NC_000012	110972237	111021064	2.0754635	2.1579053	4.9341378	4.5594136
TCTN1	NC_000012	111051880	111086935	3.3111681	3.1610697	5.5088546	5.7983502
HVCN1	NC_000012	111086491	111127617	0.0733285	0.6031155	0.0652351	0.0851637
PPP1CC	NC_000012	111157615	111180757	13.398635	16.959698	44.010349	40.084919
RPL29P25	NC_000012	111279361	111279808	0	0	0	0
CCDC63	NC_000012	111284811	111345338	0.0225955	0	0.0603047	0.0944726
MYL2	NC_000012	111348623	111358404	0	0	0	0
LOC100131138	NC_000012	111375004	111375244	0	0	0	0
CUX2	NC_000012	111471829	111788356	0.025697	0.0198144	0.0057152	0.0089533
FAM109A	NC_000012	111798483	111806925	1.3016515	1.4533515	0.5354125	0.7314859
LOC642580	NC_000012	111819226	111823484	0	0	0	0
LOC100287812	NC_000012	111843745	111888757	0.2700352	0.0520546	0.1201154	0.1293678
SH2B3	NC_000012	111843752	111889427	11.779687	4.2625851	7.9193182	12.916153
ATXN2	NC_000012	111890018	112037480	2.0562763	2.6329758	5.1803027	4.9369753
LOC100101246	NC_000012	112019098	112019749	0	0	0	0
BRAP	NC_000012	112079950	112123790	1.0479459	1.1559589	1.8645635	2.0538294
PCNPP	NC_000012	112104728	112108183	0	0	0	0
ACAD10	NC_000012	112123857	112194911	1.2765474	1.3018408	1.3371386	1.4921468
ALDH2	NC_000012	112204346	112247784	3.4426115	5.4596611	9.3997064	3.3304555
C12orf47	NC_000012	112277573	112280706	0	0	0	0
MAPKAPK5	NC_000012	112280279	112331228	4.4937282	5.7031887	5.4897267	5.6185564
RPS2P41	NC_000012	112317484	112318057	0	0	0	0
ADAM1	NC_000012	112336967	112339606	0	0	0	0
TMEM116	NC_000012	112369103	112450935	4.1233481	2.1854443	5.3542704	2.9090065
LOC100128618	NC_000012	112430807	112431865	0	0	0	0
LOC724085	NC_000012	112437040	112438873	0	0	0	0
ERP29	NC_000012	112451152	112461024	44.127369	34.317285	65.950581	40.673666
C12orf30	NC_000012	112464493	112546597	1.6572763	1.6022483	2.0289344	2.5957776
TRAFD1	NC_000012	112563349	112591408	9.8569013	8.3288687	10.232518	9.6142273
C12orf51	NC_000012	112598111	112744038	0.6893718	0.7376097	1.6122994	1.7650224
LOC100287839	NC_000012	112600587	112617139	0.0220624	0.0680473	0	0.0461218
RPL7AP60	NC_000012	112739616	112740489	0	0	0	0
LOC100287871	NC_000012	112744963	112819932	0.9880834	0.3091728	1.5287416	2.0356713
RPL6	NC_000012	112842994	112847443	94.741498	146.22628	144.315	159.05966
PTPN11	NC_000012	112856536	112947717	1.3569882	1.9416709	2.0223952	6.5363619
RPH3A	NC_000012	113229549	113336686	0.0765315	0.0688472	0.0085106	0.0533302
OAS1	NC_000012	113344739	113357712	30.027977	7.3374755	41.987129	1.8168606
OAS3	NC_000012	113376249	113411054	13.541918	5.5567334	14.708048	3.220996
LOC100287897	NC_000012	113388378	113405409	0.0389612	0	0.034661	0.0542994
OAS2	NC_000012	113416274	113449528	37.942662	14.326266	78.340899	3.3310574
RPS15AP32	NC_000012	113491867	113492243	0	0	0	0
DTX1	NC_000012	113495662	113535833	0	0	0.011891	0.0279424
RASAL1	NC_000012	113537318	113574021	0.2672908	0.2169498	0.1626979	0.3529115
LOC387885	NC_000012	113587663	113597081	0.1161116	0.1790624	0.0774721	0.0404556
DDX54	NC_000012	113594978	113623284	2.7285823	4.5585628	3.0521268	5.6622121
C12orf52	NC_000012	113623555	113630119	6.0165748	8.0693738	8.1939687	8.9890488
IQCD	NC_000012	113633246	113658880	0.3906509	0.2342843	1.4770191	1.2703653
TPCN1	NC_000012	113659260	113736390	5.0684222	3.1755064	14.995926	14.734287
SLC24A6	NC_000012	113736571	113772925	6.7783059	7.7127581	13.847054	10.414105
PLBD2	NC_000012	113796378	113827201	14.820549	10.592059	18.30034	13.04658
SDS	NC_000012	113830250	113841692	0.2464387	0.1689099	0.1461591	0.0954046
SDSL	NC_000012	113860216	113876081	3.8065398	2.6197964	3.1345221	2.1921889
LHX5	NC_000012	113900694	113909877	0	0	0.0187608	0.0440857
LOC100287933	NC_000012	113904962	113909717	0.0221961	0.0456398	0	0
LOC100129739	NC_000012	114227344	114227612	0	0	0	0
RBM19	NC_000012	114259862	114404116	0.9248902	0.8306576	0.8606382	1.5630985
TRNAE42P	NC_000012	114386545	114386617	0	0	0	0
TBX5	NC_000012	114791735	114846247	0.0101007	0.051923	0.1437741	0.0915016
LOC100287972	NC_000012	114846558	114847613	0.9538014	0	1.4359707	0.1022533
LOC100288004	NC_000012	114848517	114850637	0	0	0	0
LOC652191	NC_000012	114986659	114987666	0	0	0	0
OSTF1P	NC_000012	115025856	115026604	0	0	0	0

LOC100288068	NC_000012	115107393	115121369	0	0.1973074	0.0284553	0.1114442
TBX3	NC_000012	115108059	115121969	0.0091884	0.7746266	0.1389627	0.2945315
UBA52P7	NC_000012	116030409	116030788	0	0	0	0
MED13L	NC_000012	116396381	116714991	3.3600855	4.1290441	5.373935	6.1981253
LOC100287569	NC_000012	116972085	116973326	0.035385	0	0.1259181	0.172604
MAP1LC3B2	NC_000012	116997186	117014425	1.1620927	0.6516837	1.1748067	1.5459674
C12orf49	NC_000012	117153596	117175843	5.8122526	8.010895	7.5377289	10.296581
RNFT2	NC_000012	117176096	117291436	0.5611058	0.2182773	1.1332628	1.2152729
RPL21P105	NC_000012	117193158	117193722	0	0	0	0
HRK	NC_000012	117299027	117319232	0.2522137	0.2593022	0.1121881	0.0439381
RPL36P15	NC_000012	117346117	117346436	0	0	0	0
FBXW8	NC_000012	117348761	117468953	1.6746494	1.6286499	2.9554699	3.1917999
TESC	NC_000012	117476728	117537251	0	0	0.0767371	0.0601077
FBXO21	NC_000012	117581585	117628300	2.8447473	4.4510935	4.0270585	5.7805995
NOS1	NC_000012	117650979	117799582	0.0185019	0.0190219	0.0219464	0.0214881
KSR2	NC_000012	117890817	118406028	0.0206718	0.0212528	0.0183902	0.0342117
RFC5	NC_000012	118454508	118470039	3.0628427	4.1741554	5.5763141	5.5839527
WSB2	NC_000012	118470492	118498951	28.577252	30.004853	23.899114	36.843183
LOC100128895	NC_000012	118478052	118478405	0	0.1276367	0	0
FLJ20674	NC_000012	118501398	118541810	1.0716938	1.589502	1.6411131	1.6099027
PEBP1	NC_000012	118573870	118583390	57.772323	53.547331	89.7592	90.12875
TAOK3	NC_000012	118587606	118810750	2.9071595	2.7415112	4.9585416	5.2531726
RPS2P5	NC_000012	118683866	118684816	0	0	0	0
LOC100287626	NC_000012	118813621	118818000	0	0.1282405	0.0369892	0.1158936
SUDS3	NC_000012	118814358	118855840	1.0317796	1.1467869	1.7779138	3.6661762
RPL17P37	NC_000012	119175737	119176341	0	0	0	0
KIAA1853	NC_000012	119419396	119600857	0.0157295	0.0215621	0	0.0401901
HSPB8	NC_000012	119616595	119632551	2.2171685	2.6180193	3.2223268	7.9851081
LOC100287653	NC_000012	119632650	119633907	0	0	0	0
LOC144742	NC_000012	119721630	119741185	0	0	0	0
CCDC60	NC_000012	119772517	119978852	0	0	0.0478746	0.0374999
LOC387890	NC_000012	120031264	120079363	0	0	0.0497108	0
PRKAB1	NC_000012	120105761	120119429	4.5394259	3.8013524	4.1361023	6.1096696
CIT	NC_000012	120123598	120315092	3.8997906	4.2464811	8.9218675	9.4631445
RPL35AP30	NC_000012	120397228	120397561	0	0	0	0
CCDC64	NC_000012	120427648	120532299	0.1426428	0.0439955	0.0761394	0.0993992
RAB35	NC_000012	120532903	120554599	14.912362	15.95534	14.4136	17.41083
LOC100130711	NC_000012	120532903	120535175	0.2706886	0.0993916	0.0860043	0.3368332
GCN1L1	NC_000012	120565014	120632513	3.8381185	5.6401062	5.8141165	7.5466936
RPLP0	NC_000012	120634503	120639014	267.47923	421.09215	426.02118	641.06302
PXN	NC_000012	120648250	120703563	20.257897	27.226182	15.296947	44.892771
RPS20P31	NC_000012	120685263	120685594	0	0	0	0
NME2P1	NC_000012	120719940	120720632	0	0	0	0
RNU4-1B	NC_000012	120729563	120729706	0	0	0	0
RNU4-2	NC_000012	120729566	120729706	0	0	0	0
RNU4-1	NC_000012	120730897	120731040	0	0	0	0
SIRT4	NC_000012	120740163	120751045	0.0375305	0.1929266	0.3004937	0.0523055
PLA2G1B	NC_000012	120759914	120765592	0.5444914	0.1599412	0.2767969	0
MSI1	NC_000012	120779133	120806958	0.0297954	0.0918984	0.0397602	0.0311439
RPS27P25	NC_000012	120809159	120809498	0	0	0	0
COX6A1	NC_000012	120875904	120878532	70.33321	80.719983	107.30427	75.109191
TRIAP1	NC_000012	120881764	120884215	4.9822869	8.7155801	8.0378244	6.8659837
GATC	NC_000012	120884284	120897762	4.2772002	4.8371525	3.9953717	4.9923768
RPL31P52	NC_000012	120888248	120888603	0	0	0	0
SFRS9	NC_000012	120899471	120907558	2.6452074	4.2735806	4.0341426	3.7129044
DYNLL1	NC_000012	120907660	120936298	24.296583	30.994712	32.938904	42.015839
LOC100287721	NC_000012	120933885	120934699	0.0709988	0.2189826	0	0.1484243
COQ5	NC_000012	120941082	120966964	3.0871521	3.3835152	5.5964706	6.0681502
RPL29P24	NC_000012	120960342	120961254	0	0	0	0
LOC100131988	NC_000012	120972112	120973690	0.0623379	0.0640899	0	0.0868791
RNF10	NC_000012	120972132	121015397	7.4256175	8.057139	11.098139	19.854028
POP5	NC_000012	121016848	121019201	5.8158709	4.7383343	6.6870992	6.6525953
RPL11P5	NC_000012	121031767	121032369	0	0	0	0
CABP1	NC_000012	121078422	121105127	0.0462857	0.0713798	0.0617655	0.0322537
MLEC	NC_000012	121124949	121139667	10.161601	12.95936	14.988734	31.112059
UNC119B	NC_000012	121147831	121161443	3.2794461	3.9632915	6.06252	11.018845

ACADS	NC_000012	121163571	121177811	4.5163285	2.6398232	5.5882801	3.5784939
SPPL3	NC_000012	121201035	121342151	4.1584868	3.598208	5.5607469	9.2153616
LOC390363	NC_000012	121342256	121352929	0	0	0	0
RPL12P33	NC_000012	121354458	121355084	0	0	0	0
C12orf27	NC_000012	121407641	121410095	0	0	0	0
LOC100287253	NC_000012	121416498	121426921	0	0.0655782	0	0
HNF1A	NC_000012	121416549	121440315	0.0949792	0.1534478	0.0603544	0.2458311
C12orf43	NC_000012	121440848	121454300	0.3662353	0.4000614	1.3236155	2.7434787
OASL	NC_000012	121458095	121476780	49.622866	7.2988572	45.520737	2.8269118
LOC100287285	NC_000012	121495653	121517937	0	0	0	0
LOC100287315	NC_000012	121533550	121545339	0	0	0	0
P2RX7	NC_000012	121570678	121623876	1.1143767	0.1861757	3.9035604	0.3203236
P2RX4	NC_000012	121647664	121671909	4.3558159	1.7601029	11.625164	4.3792972
CAMKK2	NC_000012	121675495	121736111	2.4627995	2.2482562	3.4060842	2.4707027
ANAPC5	NC_000012	121746048	121792012	2.3968838	3.6219491	5.5097228	5.929927
RNF34	NC_000012	121837902	121862155	0.8683747	2.1775133	2.4871704	2.8632416
KDM2B	NC_000012	121866899	122018889	2.5756924	3.6756966	3.6183716	4.8273295
ORAI1	NC_000012	122064455	122079946	6.0810722	5.9197505	7.631343	6.4893626
MORN3	NC_000012	122089293	122107560	0.0759037	0	0.033763	0.0528927
TMEM120B	NC_000012	122150658	122219974	0.3511177	0.6016432	0.9631225	0.6565387
RHOF	NC_000012	122215660	122231594	0.3790197	0.1298907	1.1560676	0.8426558
LOC100287344	NC_000012	122231123	122231889	0.3433456	0.0882488	0.0763624	0.1196284
LOC338799	NC_000012	122233173	122241390	0	0	0	0
SETD1B	NC_000012	122242638	122270562	0.9277662	1.0310306	2.5142669	2.2907932
HPD	NC_000012	122277433	122296769	0	0	0	0.064564
PSMD9	NC_000012	122326646	122355771	9.078508	10.953552	12.047991	9.1495582
WDR66	NC_000012	122356480	122441833	0.1053353	0.1684601	0.312364	0.3017631
BCL7A	NC_000012	122459861	122499950	1.964299	1.6423692	2.0633072	2.4242634
MLXIP (NC_000012 1225	NC_000012	122516760	122517172	1.170534	3.2820873	3.6920219	3.8559169
MLXIP (NC_000012 122(	NC_000012	122611777	122628989	1.6426783	2.5468158	2.5554475	5.6622052
LRRC43	NC_000012	122652266	122688018	0.1051896	0	0	0.0879604
IL31	NC_000012	122656577	122658746	0	0	0	0.0338771
LOC100288201	NC_000012	122659110	122684943	0.0563439	0	0.0250625	0.0196313
B3GNT4	NC_000012	122688228	122692084	0.1753254	0.1201686	0.0259957	0.142536
DIABLO	NC_000012	122692209	122712068	3.721107	4.2887988	3.9724799	3.1389135
VPS33A	NC_000012	122716094	122751068	3.2585512	4.4263417	4.2356948	5.5531858
CLIP1	NC_000012	122755981	122907116	4.5368328	4.2954969	7.0482006	7.1145551
RPL21P1	NC_000012	122849289	122850239	0	0	0	0
ZCCHC8	NC_000012	122957435	122985518	1.4270899	2.2402385	4.0271585	3.1865271
RSRC2	NC_000012	122989273	123011547	1.3261047	0.825706	1.5951408	2.6941563
KNTC1	NC_000012	123011809	123110947	3.8679492	5.3649208	7.8812609	7.1186903
NIACR1	NC_000012	123185840	123187904	0	0	0	0.0444913
NIACR2	NC_000012	123199303	123201439	0	0.0634301	0.0365911	0.0143308
GPR81	NC_000012	123212153	123215129	0.0590504	0.091065	0	0.0102872
DENR	NC_000012	123237371	123255953	2.7853502	3.4835945	4.2022539	4.6822748
CCDC62	NC_000012	123259073	123311927	0.0549525	0.1412423	0.0366654	0.0478663
HIP1R	NC_000012	123320039	123347507	2.83949	1.636421	2.193939	1.7184986
LOC100288544	NC_000012	123346250	123347508	0	0.0717767	0	0.0243248
VPS37B	NC_000012	123349875	123380712	2.6218661	3.1064616	3.0293858	3.2307074
ABCB9	NC_000012	123413539	123459752	0.6229916	1.2248174	0.7292508	0.9596455
LOC100288239	NC_000012	123433190	123444660	0	0	0.056296	0
OGFOD2	NC_000012	123459354	123464588	4.0356309	2.6936259	3.6465999	4.6231787
ARL6IP4	NC_000012	123464880	123467460	4.2257915	6.1444461	5.6122192	10.558852
PITPNM2	NC_000012	123468027	123594975	1.2070105	1.6903529	2.5074448	3.7099021
MPHOSPH9	NC_000012	123640946	123706441	1.7525742	2.127161	3.6488174	5.9621405
C12orf65	NC_000012	123717841	123742484	0.8484215	0.4485942	0.9704305	1.1486441
CDK2AP1	NC_000012	123745538	123756687	67.214943	75.79061	45.851882	27.913202
SBNO1	NC_000012	123780455	123834988	4.3327761	4.8654058	6.782899	8.6107279
LOC728046	NC_000012	123848415	123850660	0.0782693	0.1810555	0.243707	0.368153
SETD8	NC_000012	123868704	123893900	5.23992	6.2905281	5.3579725	9.4512983
RILPL2	NC_000012	123899936	123921264	1.1944119	1.1650076	1.0080905	0.4695103
SNRNP35	NC_000012	123942651	123950941	0.4844263	0.6363859	0.6703808	0.3938288
RILPL1	NC_000012	123955909	124018265	1.5241193	1.4980777	2.1902981	0.9103432
TMED2	NC_000012	124069076	124082688	50.046411	68.816489	71.390757	67.803999
DDX55	NC_000012	124086672	124105482	0.8045443	0.7065292	0.5666314	0.9927974
EIF2B1	NC_000012	124105570	124118247	6.4917957	8.009098	9.7852882	11.761729

GTF2H3	NC_000012	124118381	124145334	4.8766476	7.11526	9.5860477	6.7354383
TCTN2	NC_000012	124155660	124192948	2.1143479	2.2203527	4.6084072	10.229341
ATP6V0A2	NC_000012	124196865	124244441	2.4316582	3.233591	2.7312335	3.1795964
RPL27P12	NC_000012	124205765	124206189	0	0	0	0
DNAH10	NC_000012	124247042	124420267	0.0963777	0.0924806	0.0771662	0.0693985
CCDC92	NC_000012	124420955	124457163	7.9660766	5.921974	6.2582272	11.614566
ZNF664	NC_000012	124457670	124499969	5.9882477	6.0238639	10.914865	13.861535
FAM101A	NC_000012	124773710	124800568	5.5855993	0.2313861	0	0.0285148
NCOR2	NC_000012	124808961	125020149	3.9902492	7.3282997	3.908859	5.0865701
SCARB1	NC_000012	125262174	125348519	2.6125316	2.4223049	5.1759359	6.5560917
LOC100288270	NC_000012	125262174	125298973	0.0276578	0.2559161	0.1230257	0.0963653
LOC100288312	NC_000012	125348439	125399894	1.4511799	0.7202592	1.1685865	3.9316311
UBC	NC_000012	125396192	125399577	0.2048869	0.8250272	0.4101143	1.1540844
RPL22P19	NC_000012	125420026	125420411	0	0	0	0
DHX37	NC_000012	125431372	125473667	1.8935708	2.4334872	1.2892141	3.2045377
BRI3BP	NC_000012	125478194	125510349	2.7640397	4.2270635	3.5655013	7.8247527
LOC100131779	NC_000012	125540903	125541127	0	0	0	0
AACS	NC_000012	125549925	125627871	4.367033	4.9387458	3.0609356	2.6373728
TMEM132B	NC_000012	125811162	126143589	0.0231978	0.0238498	0.0154781	0.0404129
FAM32E	NC_000012	126942083	126943441	0	0	0	0
PGBD3P3	NC_000012	126994564	126995921	0	0	0	0
LOC100288339	NC_000012	127008772	127009174	0	0	0	0
LOC100288701	NC_000012	127219986	127221519	0.7735347	0.235637	0.7136453	1.1579159
LOC121296	NC_000012	127349422	127350021	0	0	0	0.0510415
LOC100288726	NC_000012	127396244	127396872	0	0	0	0
LOC100288417	NC_000012	128310489	128316318	0.0779224	0	0	0.0542994
TMEM132C	NC_000012	128899291	129192463	0.0181192	0.0186285	0.0241791	0.0315655
LOC100132385	NC_000012	128899582	128923748	0	0	0	0
SLC15A4	NC_000012	129277739	129308541	8.2097629	6.8042412	17.270778	13.703782
MGC16384	NC_000012	129297731	129299295	0	0	0	0
LOC100128276	NC_000012	129328682	129337512	0	0	0	0
GLT1D1	NC_000012	129338081	129469509	0.096909	0.1660544	0.2155323	0.7315756
NLRP9P	NC_000012	129497875	129501711	0	0	0	0
TMEM132D	NC_000012	129556271	130388212	0	0.0156425	0.0067678	0.0106023
TRNAI29P	NC_000012	129716198	129716272	0	0	0	0
LOC100288515	NC_000012	130476683	130492097	0	0	0	0
LOC100190940	NC_000012	130517999	130526887	0	0	0	0
FZD10	NC_000012	130647032	130650285	0.0135059	0.0416565	0.0240305	0.0282344
RPS20P30	NC_000012	130701352	130701702	0	0	0	0
PIWIL1	NC_000012	130822614	130856873	0	0.0265317	0.011479	0.0269743
RIMBP2	NC_000012	130880681	131002410	0	0.007147	0.0061844	0.0096884
STX2	NC_000012	131274145	131323811	2.6061289	2.9277067	5.6887693	6.5820898
RAN	NC_000012	131356617	131360826	132.46022	185.58523	212.24393	270.50546
GPR133	NC_000012	131438452	131626010	0.0244791	0.7466251	0.0217773	0.0227441
LOC116437	NC_000012	131649556	131697476	0	0	0	0
RPS6P20	NC_000012	131777130	131777721	0	0	0	0
LOC100128002	NC_000012	131780941	131781585	0	0	0	0
LOC338797	NC_000012	131850570	131851069	0	0	0	0
LOC100130825	NC_000012	132148319	132148963	0	0	0	0
RPS6P21	NC_000012	132152025	132152826	0	0	0	0
SFRS8	NC_000012	132195635	132284282	0.9209491	1.2810086	1.5181181	2.5387033
MMP17	NC_000012	132312941	132336316	0.3832695	0.8256103	0.0487096	0.0381539
ULK1	NC_000012	132379279	132407696	2.2855442	2.7486353	4.756833	4.5605269
PUS1	NC_000012	132413789	132428404	0.6259817	0.6879595	0.5184844	0.6467926
LOC100288577	NC_000012	132423775	132426574	0.0558427	0.0574122	0	0.0389134
EP400	NC_000012	132434508	132565005	0.4228203	0.9246664	1.7373154	2.4619755
SNORA49	NC_000012	132515769	132515905	0	0	0	0
EP400NL	NC_000012	132568858	132610742	0	0	0	0
LOC100288611	NC_000012	132610714	132617112	0	0	0	0
DDX51	NC_000012	132621139	132628880	0.9501313	1.2354088	0.8949845	1.1943573
NOC4L	NC_000012	132628993	132636986	1.8042473	1.7442122	1.4853242	1.3135671
GALNT9 (NC_000012 13	NC_000012	132680917	132690573	0.1017909	0.6017477	0.067917	0
GALNT9 (NC_000012 13	NC_000012	132824459	132905905	0.0734306	0.3019776	0.032663	0.0511694
LOC100130238	NC_000012	132851977	132857486	0	0	0	0
LOC100129528	NC_000012	133049334	133050840	0	0	0	0
FBRSL1	NC_000012	133067157	133161774	0.5701288	0.4271958	0.8682617	1.1177946

LOC645277	NC_000012	133179736	133182837	0	0.061726	0	0.0836745
P2RX2	NC_000012	133195403	133198972	0	0	0.0204914	0.0160508
POLE	NC_000012	133200348	133263945	0.6222262	0.6915827	1.2766552	2.7070205
PXMP2	NC_000012	133264192	133281577	3.6279105	2.2190385	6.1689994	9.6322762
LOC100129532	NC_000012	133272503	133277936	0	0	0	0
PGAM5	NC_000012	133287436	133295752	4.6582835	7.654877	7.473036	9.1528741
ANKLE2	NC_000012	133302254	133338451	5.8262461	8.1082821	4.6043567	8.1542688
GOLGA3	NC_000012	133345495	133405288	2.3038146	2.7348363	3.807491	5.0710456
RPS11P5	NC_000012	133402267	133402848	0	0	0	0
LOC100128843	NC_000012	133413289	133414204	0.1919137	0.1479806	0.0426829	0.0668665
CHFR	NC_000012	133416938	133464184	4.7197432	5.1979631	4.3358677	3.747595
LOC647503	NC_000012	133476572	133476957	0	0	0	0
LOC100289635	NC_000012	133524324	133533302	0	0	0.1910305	0.2493883
LOC100129967	NC_000012	133548969	133559255	0	0	0	0
ZNF26	NC_000012	133563056	133589153	0.6752425	1.0191745	0.8435565	1.0912428
ZNF84	NC_000012	133613878	133639885	0.9867587	1.0915416	1.7834757	1.4927289
ZNF140	NC_000012	133657037	133684260	1.3756212	1.2374978	1.6444689	1.6675431
LOC440122	NC_000012	133696555	133698610	0	0	0	0
RPL23AP67	NC_000012	133702065	133702538	0	0	0	0
ZNF10	NC_000012	133707214	133736051	0.4988449	0.5333795	0.5946735	0.9316081
ZNF268	NC_000012	133757995	133781758	2.3379169	2.2615382	2.4999492	2.0866007
LOC647589	NC_000012	133794898	133812675	0	0	0	0
LOC729501	NC_000013	19040601	19059500	0.0252503	0.05192	0.0898536	0.0615841
LOC729524	NC_000013	19238296	19241874	0	0	0	0
LOC283523	NC_000013	19254635	19257440	0	0	0	0
LOC729535	NC_000013	19267434	19271807	0	0.1292801	0.0559336	0.0438124
LOC654337	NC_000013	19283849	19284042	0	0	0	0
LOC100101111	NC_000013	19297860	19298842	0	0	0	0
LOC100131550	NC_000013	19299878	19302053	0	0	0	0
LOC100132598	NC_000013	19307890	19308456	0	0	0	0.0540121
LOC645626	NC_000013	19385283	19411480	0	0	0	0
LOC400094	NC_000013	19464957	19469871	0	0	0	0
LOC348021	NC_000013	19582399	19586774	0	0	0	0
DKFZp686A1627	NC_000013	19620293	19692423	0	0	0	0
LOC441654	NC_000013	19725607	19745541	0	0	0	0
TUBA3C	NC_000013	19747916	19755936	0.0590305	0	0.0525152	0.0205674
LOC645739	NC_000013	19759450	19761466	0	0	0	0
LOC642395	NC_000013	19775777	19801572	0	0	0	0
LOC100101938	NC_000013	19836940	19919113	0	0	0	0
MRPL3P1	NC_000013	19878205	19879888	0	0	0	0
LOC100287114	NC_000013	19919189	19920889	0.0416966	0.085737	0.0370945	0
LOC645771	NC_000013	19923276	19982293	0	0	0	0
TPTE2	NC_000013	19997017	20110903	0.0407494	0.062842	0.0362518	0.1135832
CYCSP32	NC_000013	20032541	20032858	0	0	0	0
MRPS31P2	NC_000013	20126710	20142962	0	0	0	0
ESRRAP1	NC_000013	20133953	20135412	0	0	0	0
MPHOSPH8	NC_000013	20207880	20246657	0.2581993	0.1816278	0.157164	0.5303009
PSPC1	NC_000013	20248896	20357083	0.7657969	0.9841496	0.8137441	1.9566718
ZMYM5	NC_000013	20397624	20437776	1.1015546	0.8070789	1.8022502	1.129353
LOC400099	NC_000013	20437124	20439188	0	0	0	0
ZMYM2	NC_000013	20532810	20660939	4.6837679	4.4256696	4.3916376	4.1737183
GJA3	NC_000013	20712394	20735183	0.0675347	0.0347164	0.0150202	0.0588261
LOC390378	NC_000013	20753203	20753601	0	0	0	0
GJB2	NC_000013	20761602	20767114	0	0	0.0335026	0.0393636
GJB6	NC_000013	20796101	20806534	0.0350464	0.0360314	0.0467674	0.0366326
CRYL1	NC_000013	20977806	21100012	1.2297307	1.053577	4.8969636	2.8564177
IFT88	NC_000013	21141208	21265576	0.5702144	0.5719418	0.631005	0.881919
LOC727991	NC_000013	21181218	21266663	0	0	0	0
IL17D	NC_000013	21277482	21297237	0.921	0.2670701	0.420178	0.2632983
N6AMT2	NC_000013	21303073	21348057	3.4271557	1.1399482	2.6901995	3.090584
XPO4	NC_000013	21351468	21476913	2.7164764	2.32202	2.6262806	2.7573234
LOC100130563	NC_000013	21495608	21497247	0	0	0	0.0320009
LOC440125	NC_000013	21522085	21523798	0	0	0	0
LOC728031	NC_000013	21535373	21536406	0	0	0	0
RPSAP54	NC_000013	21535373	21536407	0	0	0	0
LATS2	NC_000013	21547175	21635722	6.4063091	2.4128246	4.1615666	3.7901257

RPS12P23	NC_000013	21668434	21668934	0	0	0	0
SAP18	NC_000013	21714653	21723224	5.7871857	4.2021938	3.9760236	4.5651166
C13orf3	NC_000013	21727734	21750710	1.2934902	1.2359726	1.9223872	3.0646087
MRP63	NC_000013	21750372	21753223	3.9186347	2.8728348	2.3535028	2.4887034
ESRRAP2	NC_000013	21834308	21836496	0	0	0	0
LOC650794	NC_000013	21861602	21876010	0.0968734	0	0.1149083	0.360028
GRK6PS	NC_000013	21893222	21895824	0	0	0	0
ZDHHC20	NC_000013	21950508	22033423	8.837908	7.1946117	7.7014422	7.6930031
LOC100130907	NC_000013	22057970	22058313	0	0	0	0
EFHA1	NC_000013	22066839	22178307	7.1292756	3.9782611	4.8819799	5.4909067
FNTAL2	NC_000013	22105108	22105979	0	0	0	0
RPS7P10	NC_000013	22202537	22203198	0	0	0	0
FGF9	NC_000013	22245215	22278640	0.0291048	0.0099743	0.0172616	0.0405628
FTHL7	NC_000013	23270001	23270929	0	0	0	0
LOC646164	NC_000013	23276879	23280344	0	0	0	0
LOC401730	NC_000013	23410938	23412581	0	0	0	0
LOC100129167	NC_000013	23424240	23424706	0	0	0	0
LOC646201	NC_000013	23471480	23472653	0	0	0	0
LOC646208	NC_000013	23489600	23491485	0	0	0	0
LOC100130029	NC_000013	23708417	23708706	0	0	0	0
SGCG	NC_000013	23755060	23899304	0	0.0546023	0	0.0370089
SACS	NC_000013	23902965	24007841	1.8940539	1.8346096	2.7775047	5.4869818
TNFRSF19	NC_000013	24144723	24250232	4.4813045	0.7476681	1.8359769	2.4036968
MIPEP	NC_000013	24304328	24463559	4.6126424	1.731309	3.2242057	4.6811038
PCOTH	NC_000013	24463028	24466242	0.2428079	0.0998528	0.0432017	0
RP11-45B20.2	NC_000013	24465428	24471125	0.0438605	0	0	0.122255
FLJ46358	NC_000013	24515423	24522113	0	0.0660576	0	0
LOC100128337	NC_000013	24695163	24697953	0	0	0	0
SPATA13	NC_000013	24734887	24878048	0.4516518	0.1160864	0.334835	0.4983209
C1QTNF9	NC_000013	24883716	24896669	0	0	0.0642348	0.0335431
LOC100287275	NC_000013	24895171	24895776	0	0	0.0880576	0.1379499
LOC724034	NC_000013	24897096	24898002	0	0	0	0
LOC100288620	NC_000013	24897159	24897598	0	0	0	0
YDD19	NC_000013	24902348	24903372	0	0	0	0
CYCSP33	NC_000013	24974850	24975167	0	0	0	0
PARP4	NC_000013	24995069	25086948	12.865445	6.8407125	10.887839	15.136048
LOC374491	NC_000013	25154346	25171814	0	0	0	0
ATP12A	NC_000013	25254695	25285918	0	0	0	0.0257713
RPL26P34	NC_000013	25278476	25278926	0	0	0	0
IRX1P1	NC_000013	25314992	25320841	0	0	0	0
RNF17	NC_000013	25338301	25454059	0	0.0176912	0	0.0179864
CENPJ	NC_000013	25456412	25497085	0.9685093	0.8734468	1.8139215	2.1253298
LOC646405	NC_000013	25502893	25542607	0	0	0	0
RPL34P27	NC_000013	25562671	25563094	0	0	0	0
LOC100133284	NC_000013	25591595	25591978	0.1144485	0	0	0.3190091
PABPC3	NC_000013	25670276	25672705	0.0904285	0.4090678	0.4183279	0.4537019
LOC100288679	NC_000013	25691059	25716015	0	0	0.1589332	0.1244914
FAM123A	NC_000013	25742660	25745857	0.0824545	0.0423859	0.0122256	0.038305
LOC100287306	NC_000013	25763947	25764423	0	0	0	0
RPL23AP69	NC_000013	25780190	25780619	0	0	0	0
MTMR6	NC_000013	25820339	25861704	5.8221296	3.7508461	3.4480215	3.199877
LOC646482	NC_000013	25874262	25875879	0	0	0	0
NUPL1	NC_000013	25875666	25916561	9.2382784	6.0284996	8.7262756	9.1867779
LOC246717	NC_000013	25939901	25940337	0	0	0	0
ATP8A2	NC_000013	25946209	26595420	0.0263373	0.0090258	0.0078101	0.1468232
SHISA2	NC_000013	26618735	26625198	0	0.0469194	0	0.0636031
LOC100129595	NC_000013	26689950	26693129	0	0	0	0
RNF6	NC_000013	26786905	26796508	2.4205369	1.5804166	1.4389864	1.798642
LOC100128764	NC_000013	26808350	26811266	0	0	0	0
CDK8	NC_000013	26828756	26978569	2.8321642	2.6052612	3.5583426	2.6314197
RPS3AP44	NC_000013	27150995	27151858	0	0	0	0
WASF3	NC_000013	27187097	27263082	4.256961	3.8403019	2.5357895	3.2650093
GPR12	NC_000013	27331923	27334922	0.0194118	0.0798293	0	0.0270538
RPS20P32	NC_000013	27591654	27592165	0	0	0	0
USP12	NC_000013	27642433	27746029	3.9036041	2.5539279	5.2839039	5.4664041
RPL21	NC_000013	27825692	27830705	4.9432106	2.4616616	5.4283088	3.6060929

SNORD102	NC_000013	27829201	27829272	0	0	0	0
SNORA27	NC_000013	27829538	27829663	0	0	0	0
RASL11A	NC_000013	27844464	27847827	0.9399168	0.4392424	0.2533867	0.6351239
GTF3A	NC_000013	27998681	28009846	11.331382	7.4983851	9.3288351	10.011979
LOC100287370	NC_000013	28008286	28008752	0	0	0	0
MTIF3	NC_000013	28009780	28024711	5.2912796	1.6185099	3.5401776	6.4297008
LNX2	NC_000013	28120050	28194720	0.9056775	0.4608632	1.0905649	1.1729762
POLR1D	NC_000013	28196003	28241548	4.1506663	2.9942969	5.6474261	5.8697683
LOC100287414	NC_000013	28239852	28246236	0	0.2819557	0.1219893	0
LOC100128903	NC_000013	28270530	28271246	0	0	0	0
GSX1	NC_000013	28366780	28368089	0.0518869	0	0	0
PDX1	NC_000013	28494168	28500451	0	0.0175606	0	0
ATP5EP2	NC_000013	28519343	28519710	0	0	0	0
LOC100132234	NC_000013	28527669	28529602	0	0.0934507	0	0.047505
LOC100287444	NC_000013	28535055	28537111	0	0	0.0190071	0.0148881
CDX2	NC_000013	28536278	28543317	0.0210279	0.0216189	0.018707	0.0293061
PRHOXNB	NC_000013	28552243	28562774	0	0	0	0
FLT3	NC_000013	28577411	28674729	0.0228778	0	0.0101764	0.0239132
LOC646630	NC_000013	28681732	28682013	0	0	0	0
LOC100288730	NC_000013	28710879	28752056	0.2815071	0.2213204	0.2946312	0.5654179
PAN3	NC_000013	28748417	28869472	1.6761064	1.0283694	1.9240139	2.7441197
FLT1	NC_000013	28876389	29069265	0.008424	0.4157185	0.0074943	0.029351
LOC100287487	NC_000013	29069605	29071404	0.0976627	0.1004076	0	0.0170138
LOC341784	NC_000013	29172954	29174341	0	0	0	0
POMP	NC_000013	29233241	29253094	27.749985	21.032509	27.041048	25.183454
SLC46A3	NC_000013	29274217	29293150	0.893417	0.2701549	1.0636408	0.7232781
CYP51P2	NC_000013	29295001	29298296	0	0	0	0
KIAA0774	NC_000013	29598748	30080084	0.0305366	0.0439527	0.0217329	0.046814
GAPDHL14	NC_000013	29880844	29882111	0	0	0	0
SLC7A1	NC_000013	30083551	30169825	5.8114848	4.159605	3.9454305	7.5112905
UBL3	NC_000013	30338545	30424820	8.8988596	4.8032056	4.1562529	4.3689426
LOC100288766	NC_000013	30440096	30473040	0	0	0	0
KATNAL1	NC_000013	30776767	30881584	1.0465241	0.9035492	1.1984914	1.9943843
PRDX2P1	NC_000013	30870205	30871128	0	0	0	0
LOC100188949	NC_000013	30914407	30948036	0	0	0	0
UBE2L5P	NC_000013	31002122	31002478	0	0	0	0
HMGB1	NC_000013	31032877	31040081	2.2692057	2.8733902	2.6346376	3.3858894
LOC646755	NC_000013	31115780	31117034	0	0	0	0
LOC646758	NC_000013	31127808	31128947	0	0	0	0
USPL1	NC_000013	31191830	31233686	1.3058389	0.9721839	1.6023592	1.9454328
ALOX5AP	NC_000013	31309669	31338556	0.0503416	0.1035129	0.0447853	0
C13orf33	NC_000013	31480312	31499709	17.26669	9.8117931	5.7092261	10.020358
C13orf26	NC_000013	31506834	31549153	0	0	0.0391759	0
HSPH1	NC_000013	31710762	31736117	7.7760094	4.7918057	6.9710491	16.781899
B3GALTL	NC_000013	31774112	31906413	4.5877158	2.197532	2.625056	5.3257499
RXFP2	NC_000013	32313679	32377009	0.015679	0	0.0278969	0.043703
EEF1DP3	NC_000013	32420920	32533721	0	0	0	0
FRY	NC_000013	32605437	32870776	1.0744086	0.3077716	0.3356327	0.3800605
LOC646799	NC_000013	32877908	32886091	0	0	0.0708931	0
BRCA2	NC_000013	32889617	32973809	2.6131172	1.7659067	2.9496583	2.9694243
IFIT1P	NC_000013	32958821	32959533	0	0	0	0
N4BP2L1	NC_000013	32974860	33002315	0.3667054	0.0725022	1.191999	0.5110699
N4BP2L2	NC_000013	33006930	33112936	3.2963419	1.7175787	3.1003179	2.847807
LOC100288799	NC_000013	33051408	33052909	0	0	0	0
CG030	NC_000013	33078643	33083532	0	0	0	0
PDS5B	NC_000013	33160592	33352157	1.1944693	1.0707541	0.9998172	1.5827022
LOC122038	NC_000013	33528195	33528596	0	0	0	0
KL	NC_000013	33590571	33640282	0.0087791	0.0361034	0	0.0244705
STARD13	NC_000013	33677272	33859901	5.0305523	2.095771	1.9917628	4.5455794
RFC3	NC_000013	34392206	34540695	3.3287142	4.7470171	4.9673696	6.306694
LOC100130499	NC_000013	34656540	34657447	0	0	0	0
LOC100129452	NC_000013	35148339	35148886	0	0	0	0
NBEA	NC_000013	35516456	36246873	0.2321209	0.2302712	0.2173697	0.3036381
MAB21L1	NC_000013	36048319	36050832	0	0	0.0155519	0
DCLK1	NC_000013	36343122	36705464	0.7832358	0.2069042	0.0725821	0.109916
TRNAQ50P	NC_000013	36639747	36639818	0	0	0	0

SOHLH2	NC_000013	36742354	36788676	0.0833932	0.0214343	0.0556417	0.0871676
C13orf38	NC_000013	36801182	36871992	0.74851	0.1099353	0.4122209	0.2235393
SPG20	NC_000013	36875775	36944317	6.019971	2.2026385	3.4350781	6.0398277
CCNA1	NC_000013	37005967	37017019	0.057274	0.0588837	0.0169842	0.0798216
LOC651868	NC_000013	37064187	37064578	0	0	0	0.0911455
C13orf36	NC_000013	37248049	37271976	0	0	0.0121913	0.0477469
LOC646877	NC_000013	37314178	37315060	0	0	0	0
LOC100130120	NC_000013	37315800	37316563	0	0	0	0
LOC646879	NC_000013	37368621	37369187	0	0	0.0753325	0.0590075
RFXAP	NC_000013	37393339	37403740	0.5911621	0.4638296	0.3736758	0.8238905
SMAD9	NC_000013	37422207	37494409	1.3261047	1.2289578	1.4289803	2.2776684
LOC100130119	NC_000013	37496580	37497827	0	0	0	0
RPL29P28	NC_000013	37500575	37500968	0	0	0	0
LOC390394	NC_000013	37512452	37516487	0	0	0	0
ALG5	NC_000013	37523907	37573504	6.9506264	4.9500762	4.5087802	6.1300209
EXOSC8	NC_000013	37574678	37583751	7.6309828	7.8774747	9.9475734	7.9220982
FAM48A	NC_000013	37583449	37633850	4.5978843	2.7885466	3.2387752	4.8817875
CSNK1A1L	NC_000013	37677397	37679801	0.0182737	0.0375746	0	0.0127338
RPS12P24	NC_000013	37794995	37795389	0	0	0	0
POSTN	NC_000013	38136719	38172981	19.555663	12.418752	0.0925386	0.0815455
TRPC4	NC_000013	38210773	38443939	1.3499022	0.6284565	0.0679761	0.1331131
LOC646910	NC_000013	38567181	38568857	0	0	0	0
UFM1	NC_000013	38923942	38937143	14.328374	6.1669003	11.799919	10.761683
LOC100128902	NC_000013	39191556	39247153	0	0	0	0
FREM2	NC_000013	39261173	39461268	0.0108763	0.0195684	0.0241895	0.0360003
LOC646929	NC_000013	39483465	39510241	0	0	0	0
STOML3	NC_000013	39540062	39564996	0	0.0226143	0.0391367	0.0306555
C13orf23	NC_000013	39584002	39612252	0.6919652	0.4771673	0.8032718	1.2231134
LOC100287644	NC_000013	39611524	39612232	0	0	0	0
NHLRC3	NC_000013	39612455	39624246	4.8790091	2.1388593	3.4371486	2.200435
LOC100288994	NC_000013	39743766	39774820	0	0	0	0
LHFP	NC_000013	39917029	40177356	12.463539	5.8415983	5.4352501	6.2725654
COG6	NC_000013	40229764	40365802	1.9314425	1.4193057	1.0027906	0.9752303
LOC100289029	NC_000013	40391884	40392187	0	0	0	0
LOC646982	NC_000013	40921269	41055143	0	0	0	0
LOC100289058	NC_000013	41110607	41120625	0	0.0564087	0	0.0382333
FOXO1	NC_000013	41129801	41240734	0.5820958	0.1023674	0.8244694	0.5283832
LOC100133016	NC_000013	41193549	41195487	0	0	0	0
MRPS31	NC_000013	41303432	41345347	0.1312867	0.1012324	0.1751945	0.3430718
SLC25A15	NC_000013	41363597	41384246	1.924938	1.6166794	1.5677617	1.908151
SUGT1L1	NC_000013	41371127	41495886	0	0	0	0
CYCSP34	NC_000013	41437730	41438042	0	0	0	0
ELF1	NC_000013	41506055	41593508	3.7025122	0.9304953	2.059968	2.3589101
LOC100128628	NC_000013	41566872	41567467	0	0	0	0
WBP4	NC_000013	41635697	41658139	1.1490965	0.5035441	0.8211662	0.8663703
KBTBD6	NC_000013	41701709	41706936	0.5043791	0.4494141	0.560887	0.8669629
CALM2P3	NC_000013	41744724	41745360	0	0	0	0
KBTBD7	NC_000013	41765711	41768702	1.2191522	0.8305772	0.9800526	0.5527217
MTRF1	NC_000013	41790516	41837713	0.9501228	0.8729084	1.0790496	1.0987767
RAC1P3	NC_000013	41861642	41862596	0	0	0	0
LOC100289088	NC_000013	41861934	41885491	0	0	0	0
NARG1L	NC_000013	41885341	41951166	0.8709253	0.5903755	0.7237136	1.033723
TUBBP2	NC_000013	41957824	41959291	0	0	0	0
OR7E36P	NC_000013	42005400	42006394	0	0	0	0
OR7E155P	NC_000013	42013973	42014998	0	0	0	0
OR7E37P	NC_000013	42017060	42017723	0	0	0	0
C13orf15	NC_000013	42031542	42045013	0.674296	0.4077924	0.7057322	0.2211183
KIAA0564	NC_000013	42140961	42535221	0.9602932	0.5423958	0.4271517	0.7352614
RPS28P8	NC_000013	42577568	42577772	0	0	0	0
DGKH	NC_000013	42622889	42803888	1.0449085	0.9360027	1.0400247	1.3265013
MAPK6PS3	NC_000013	42641853	42646014	0	0	0	0
AKAP11	NC_000013	42846289	42897402	2.5831051	1.6079989	2.1521596	2.8312343
FABP3P2	NC_000013	42943250	42943893	0	0	0	0
TNFSF11	NC_000013	43136872	43182149	0.0483479	0.0165689	0	0.044921
C13orf30	NC_000013	43355686	43365685	0	0	0.0118406	0.0278239
EPSTI1	NC_000013	43462122	43566377	30.286003	7.4108822	15.062381	1.9663781

DNAJC15	NC_000013	43597362	43683306	3.9578739	3.059971	1.3520772	2.4270437
ENOX1	NC_000013	43787661	44361106	0.4790766	1.0575147	0.4638057	0.3338396
LOC100287709	NC_000013	44359764	44361042	0	0	0	0
CCDC122	NC_000013	44410488	44453826	0.8094	0.3148669	0.7589871	0.4115837
C13orf31	NC_000013	44453420	44468068	0.3766991	0.1986084	0.2921576	0.3365371
LOC400126	NC_000013	44542382	44545943	0	0	0	0
LOC121838	NC_000013	44596471	44604599	0	0	0	0
LOC100287738	NC_000013	44732690	44735359	0	0	0.0159127	0.0124643
SERP2	NC_000013	44947978	44971850	0.9529591	0.9221102	0.8976482	1.1718703
TSC22D1	NC_000013	45007655	45150701	8.6278215	4.9386509	9.5698111	6.1902613
LOC641467	NC_000013	45150035	45151221	0	0	0	0
LOC100287772	NC_000013	45151626	45153283	0.1325339	0.1362588	0.141487	0.0923549
LOC400129	NC_000013	45168994	45170285	0	0	0	0
NUFIP1	NC_000013	45513384	45563613	1.5124141	1.0884444	0.975477	1.2559098
KIAA1704	NC_000013	45563687	45602405	4.353809	2.9841156	3.2557915	3.517574
GTF2F2	NC_000013	45694631	45858240	1.8739961	1.3673106	1.8822762	3.2225628
KCTD4	NC_000013	45766988	45775175	0	0.021323	0.018451	0.028905
TPT1	NC_000013	45911304	45915297	341.03616	208.74841	365.17787	302.07434
SNORA31	NC_000013	45911615	45911744	0	0	0	0
LOC100287803	NC_000013	45914130	45915357	0.8996567	0.7862004	0.4001798	0.4074958
LOC100190939	NC_000013	45915480	45965618	0	0	0	0
LOC100287828	NC_000013	45963311	45972636	0	0	0	0
LOC728913	NC_000013	45963429	45965561	0	0	0	0
SLC25A30	NC_000013	45967451	45992516	1.7359013	0.9477314	1.0543882	1.0928518
LOC647085	NC_000013	46010291	46011787	0	0	0	0
COG3	NC_000013	46039071	46110760	2.9122322	1.8145945	2.5471863	2.2548438
FLJ32682	NC_000013	46115432	46189874	0.1270705	0.1119787	0.0322987	0.0126497
LOC652170	NC_000013	46245980	46246526	0	0	0	0
AKR1B1P4	NC_000013	46261525	46262634	0	0	0	0
SPERT	NC_000013	46276446	46288693	0	0.0560241	0	0
SIAH3	NC_000013	46354405	46425846	0.03292	0.0225635	0.0195244	0.02294
ZC3H13	NC_000013	46536314	46626894	0.2125753	0.1057499	0.1830125	0.4061655
CPB2	NC_000013	46627322	46679211	0.0248857	0.0255852	0	0.0346828
LCP1	NC_000013	46700058	46756459	0.2551085	0.5841654	1.4751852	0.4040221
LRRC63	NC_000013	46786083	46841111	0.0925713	0.0237933	0.041177	0.0161268
LOC390403	NC_000013	46844212	46844757	0	0	0	0
C13orf18	NC_000013	46916136	46961635	0.5855345	0.1930915	0.1670836	0.3387367
PPP1R2P4	NC_000013	47011529	47012332	0	0	0	0
LOC731932	NC_000013	47032199	47035198	0	0	0	0.0226851
OR7E101P	NC_000013	47034570	47035443	0	0	0	0
COX17P	NC_000013	47064992	47065396	0	0	0	0
FKBP1P3	NC_000013	47079214	47082644	0	0	0	0
LRCH1	NC_000013	47127303	47317730	0.6650825	0.4667034	0.4320173	0.5443769
ESD	NC_000013	47345391	47371367	45.570482	26.950838	44.411161	49.893887
HTR2A	NC_000013	47407513	47470175	0.0146056	0	0.0649677	0.0203555
GNG5P5	NC_000013	47881251	47881675	0	0	0	0
LOC730174	NC_000013	48337595	48340753	0	0	0	0
RPL27AP8	NC_000013	48340775	48341231	0	0	0	0
SUCLA2	NC_000013	48516791	48575462	4.3866997	2.2758738	1.8789958	3.3964745
NUDT15	NC_000013	48611703	48621282	1.4997171	1.6312504	1.4888786	1.832646
MED4	NC_000013	48650092	48669240	2.7820839	1.7429799	2.3009942	2.014396
LOC100129174	NC_000013	48708494	48712005	0	0	0	0
ITM2B	NC_000013	48807274	48836232	83.979137	32.028741	51.779529	24.648504
RB1	NC_000013	48877883	49056026	6.2441117	3.6737552	5.5631281	6.4047838
P2RY5	NC_000013	48985181	48989946	0	0	0.0179511	0
RCBTB2	NC_000013	49063099	49107316	1.0381472	0.5550087	0.4186826	1.0031456
LOC100129597	NC_000013	49144759	49147453	0	0	0	0
CYSLTR2	NC_000013	49280951	49283498	0.0344963	0	0	0.0120192
PSME2P2	NC_000013	49345233	49346006	0	0	0	0
FNDC3A	NC_000013	49550048	49783915	6.4131354	3.1515069	5.4663054	4.324924
RAD17P2	NC_000013	49617829	49621014	0	0	0	0
LOC100289330	NC_000013	49618196	49784150	0	0	0	0
COX7CP1	NC_000013	49761495	49761885	0	0	0	0
LOC387924	NC_000013	49784350	49786423	0	0	0	0
MLNR	NC_000013	49794474	49796513	0.0354707	0	0	0
CDADC1	NC_000013	49822100	49867618	0.2365634	0.189165	0.2572208	0.2106376

CAB39L	NC_000013	49882786	50018221	0.8040654	0.4529664	0.7349166	1.0131539
SETDB2	NC_000013	50018510	50066298	1.7190182	0.6028107	0.6520213	0.612869
PHF11	NC_000013	50069801	50103117	7.2553863	2.5181076	5.4267914	3.1397746
RCBTB1	NC_000013	50106082	50159719	2.5826261	1.4900552	2.2781869	2.559034
LOC100131941	NC_000013	50194448	50199689	0	0	0	0
ARL11	NC_000013	50202624	50207731	0.0133419	0.0274338	0.035608	0.0185943
EBPL	NC_000013	50234862	50265611	5.2410911	2.9166529	5.0903833	5.1599904
KPNA3	NC_000013	50273443	50367057	9.5381612	8.5135467	8.0659484	11.164322
LOC220429	NC_000013	50464545	50467516	0	0	0	0
C13orf1	NC_000013	50486842	50510626	0.5076755	0.3526647	0.3173702	0.4493816
DLEU2	NC_000013	50556688	50699677	0	0	0	0
TRIM13	NC_000013	50571143	50592603	0.3264658	0.0305128	0.0739284	0.190268
KCNRG	NC_000013	50589390	50595058	0.0540901	0.0834155	0.1443602	0.0565382
DLEU1	NC_000013	50656414	50679433	0	0	0	0
RPL18P10	NC_000013	50673470	50674011	0	0	0	0
FLJ31945	NC_000013	50694973	50702600	0.1825472	0.1055687	0.172549	0.2782634
FAM10A4	NC_000013	50746154	50747752	0	0	0	0
RPL34P26	NC_000013	50935546	50935899	0	0	0	0
DLEU7	NC_000013	51286759	51417885	0.0744885	0.038291	0	0
RNASEH2B	NC_000013	51483892	51544596	4.2046626	2.3460613	5.2443369	7.3911966
GUCY1B2	NC_000013	51568647	51640293	0	0	0	0
LOC647166	NC_000013	51658287	51723129	0.0300809	0	0	0.0209616
RPL5P31	NC_000013	51735831	51736488	0	0	0	0
FAM124A	NC_000013	51796507	51855616	0.1879915	0.064425	0.2787374	0.6549998
LOC100129303	NC_000013	51825477	51854856	0.0773056	0	0.0687732	0
SERPINE3	NC_000013	51915168	51936239	0.0609969	0.0313556	0.0271322	0.0637576
INTS6	NC_000013	51935701	52027275	3.3915191	1.9867452	3.1126976	3.0094401
RPS4P16	NC_000013	52034631	52035516	0	0	0	0
LOC100289499	NC_000013	52070060	52070387	0	0	0	0
WDFY2	NC_000013	52158484	52336171	3.3344812	1.8979639	3.9928992	4.5748374
DHRS12	NC_000013	52342131	52378293	0.5735228	0.3032443	0.4664885	0.6394456
FLJ37307	NC_000013	52387483	52419286	0	0	0	0
CCDC70	NC_000013	52436117	52440372	0	0.0391537	0	0.0796141
CTAGE3	NC_000013	52481952	52484815	0	0	0	0
ATP7B	NC_000013	52506805	52585630	0.1654179	0.2516992	0.0529777	0.0553295
FABP5L2	NC_000013	52540833	52541476	0	0	0	0
ALG11	NC_000013	52586550	52603780	0.2597413	0.1602248	0.1694536	0.1206654
UTP14C	NC_000013	52598827	52607736	0.3656391	0.3350551	0.1767841	0.3711099
NEK5	NC_000013	52638898	52703214	0	0.0310326	0.0268527	0.0631007
NEK3	NC_000013	52706779	52733996	0.7344633	0.2985301	0.6230082	0.4879984
THSD1P	NC_000013	52741847	52768602	0	0	0	0
LOC100289529	NC_000013	52784705	52785467	0	0	0	0
LOC644623	NC_000013	52793663	52908484	0	0	0	0
THSD1	NC_000013	52951302	52980629	1.4308116	0.6882776	1.3079233	0.9238688
VPS36	NC_000013	52986737	53024763	0.8445851	0.4290533	0.5834138	0.8724247
CKAP2	NC_000013	53029495	53050763	14.186715	11.646578	11.825548	14.541899
LOC220115	NC_000013	53063128	53161225	0	0	0	0
LOC100289595	NC_000013	53167365	53170008	0	0	0	0
HNRNPA1L2	NC_000013	53191605	53217919	1.6910313	0.9552516	2.0664675	2.5768925
SUGT1	NC_000013	53226831	53262433	5.5858055	4.3007717	4.400118	8.5221526
LECT1	NC_000013	53277399	53313947	0	0	0	0
LOC121981	NC_000013	53394632	53395153	0	0	0	0
PCDH8	NC_000013	53418109	53422774	0	0.0112902	0.019539	0.0076524
OLFM4	NC_000013	53602972	53626192	0.04677	0	0	0
LOC100133285	NC_000013	53774116	53776547	0	0	0	0
RPL13AP25	NC_000013	54941090	55001672	0	0	0	0
LOC100287887	NC_000013	55014792	55015467	1.3652557	1.2031083	0.1735099	0.634243
LOC100130462	NC_000013	56573334	56748481	0	0	0	0
PRR20	NC_000013	57715052	57718073	0	0	0	0.0165809
LOC729233	NC_000013	57721622	57724643	0	0	0.0211681	0.0165809
LOC729240	NC_000013	57728195	57731216	0	0.0244631	0	0
LOC729246	NC_000013	57734766	57737787	0.0237944	0.0244631	0	0.0331618
LOC729250	NC_000013	57741331	57744352	0	0.0244631	0.0423363	0.0165809
SLC25A5P4	NC_000013	57888799	57890551	0	0	0	0
RPL31P53	NC_000013	57965299	57965652	0	0	0	0
PCDH17	NC_000013	58205789	58303065	0.0054874	0.0056416	0.0146451	0.019119

LOC100129744	NC_000013	58206893	58207709	0	0	0	0
TRNAE39P	NC_000013	58458550	58458621	0	0	0	0
LOC341689	NC_000013	59102749	59105166	0	0	0	0
LOC100129308	NC_000013	59325048	59326489	0	0	0	0
LOC100289667	NC_000013	59645596	59645934	0	0	0	0
DIAPH3	NC_000013	60239717	60738119	1.3213539	1.2407549	1.0031045	2.6149926
LOC440142	NC_000013	60843337	60850370	0	0	0	0
TDRD3	NC_000013	61013820	61148011	1.9270845	1.0640022	2.1271108	2.872248
LOC390407	NC_000013	61211020	61212231	0	0	0	0
PCDH20	NC_000013	61983818	61989655	0.009198	0.0094566	0	0.0064096
LOC100129024	NC_000013	62371387	62384754	0	0	0	0
RPL32P28	NC_000013	62902017	62902529	0	0	0	0
LOC647259	NC_000013	62980191	62984640	0	0	0	0
OR7E156P	NC_000013	64311568	64316701	0	0	0	0
LOC647262	NC_000013	64320874	64322306	0	0.0315306	0	0.0213712
LOC100128626	NC_000013	64385256	64385855	0	0	0	0
LOC647264	NC_000013	64405811	64407126	0	0	0	0
OR7E104P	NC_000013	64411061	64411806	0	0	0	0
NFYAP	NC_000013	64549812	64550655	0	0	0	0
LGMN2P	NC_000013	65531701	65533650	0	0	0	0
STARP1	NC_000013	65854187	65885295	0	0	0	0
LOC387933	NC_000013	66361676	66362895	0	0	0	0
LOC100130023	NC_000013	66843170	66844506	0	0	0	0
PCDH9	NC_000013	66876966	67804468	0.4305191	0.9070058	0.0753446	0.2803305
LOC730236	NC_000013	67804715	67806186	0.3284175	0.6752954	0.6374603	0.2704643
RPSAP53	NC_000013	67840887	67841912	0	0	0	0
LOC390411	NC_000013	68405808	68406975	0	0	0	0
OR7E111P	NC_000013	68476376	68477356	0	0	0	0
OR7E33P	NC_000013	68485042	68486249	0	0	0	0
LOC100131269	NC_000013	68863835	68865463	0	0	0	0
RPL37P21	NC_000013	68905464	68905745	0	0	0	0
RPL12P34	NC_000013	69228704	69229180	0	0	0	0
LOC730239	NC_000013	69559190	69560258	0	0	0	0
LOC100128625	NC_000013	69982262	69982867	0	0	0	0
KLHL1	NC_000013	70274725	70682625	0.0317698	0.0653254	0.0188422	0.0073795
ATXN8OS	NC_000013	70681345	70713885	0	0	0	0
LOC100288130	NC_000013	71498351	71498941	0	0	0.0661549	0
DACH1	NC_000013	72012098	72441330	0.1091777	0.0518059	0.0373567	0.0351136
LOC100287087	NC_000013	72249190	72249648	0	0	0	0
RPL21P109	NC_000013	72278272	72278825	0	0	0	0
RPL35AP31	NC_000013	72420052	72420484	0	0	0	0
RPS10P21	NC_000013	72487840	72488314	0	0	0	0
RPL21P110	NC_000013	73277791	73278308	0	0	0	0
C13orf37	NC_000013	73282495	73301938	2.2775825	1.4049565	4.0524011	3.7201863
C13orf34	NC_000013	73302042	73330328	3.0429356	2.57446	2.3686949	2.8051638
DIS3	NC_000013	73329540	73356344	2.3307607	1.2655102	2.6666649	2.211885
PIBF1	NC_000013	73356230	73590592	1.1713883	0.798037	1.2053199	1.2391569
PSMD10P3	NC_000013	73610523	73610983	0	0	0	0
KLF5	NC_000013	73633142	73651676	5.1819557	1.0385439	2.9060579	2.349431
FABP5L1	NC_000013	73674481	73675183	0	0	0	0
LOC647281	NC_000013	74185158	74196664	0	0	0	0
KLF12	NC_000013	74260149	74708066	0.8661536	0.2402271	1.4550932	2.8157258
RPL21P108	NC_000013	74951730	74952088	0	0	0	0
LOC100288208	NC_000013	74988682	74992947	0	0	0	0.070402
LOC122145	NC_000013	75402529	75404524	0	0	0	0
LOC100131189	NC_000013	75702152	75711110	0	0	0	0
LOC647288	NC_000013	75811889	75814511	0	0	0	0
TBC1D4	NC_000013	75858809	76056250	1.9684498	0.5538748	1.9908237	2.5219922
COMMD6	NC_000013	76099350	76111991	5.7423648	3.6209695	5.5172523	4.3394136
UCHL3	NC_000013	76123927	76180069	12.861627	6.0494365	6.9022954	9.5430604
LMO7	NC_000013	76194570	76434006	1.5337985	0.4566125	0.119452	1.2847334
FLJ35379	NC_000013	76445174	76457948	0	0	0	0
LOC100132423	NC_000013	77050895	77055113	0	0	0	0
KCTD12	NC_000013	77454304	77460540	3.0510797	1.0721731	0.6770141	0.1325752
BTF3L1	NC_000013	77502585	77503224	0	0	0	0
IRG1	NC_000013	77511985	77532120	0.0272801	0	0.0485383	0.0950493

RPL7P44	NC_000013	77555247	77556072	0	0	0	0
DHX9P	NC_000013	77558877	77563104	0	0	0	0
CLN5	NC_000013	77566059	77576652	5.179613	1.9041577	1.8850612	1.5531188
FBXL3	NC_000013	77579389	77601331	6.7488832	4.8633586	5.331987	5.712502
MYCBP2	NC_000013	77618792	77901177	1.2556638	0.6991392	1.1542206	2.1137089
SCEL	NC_000013	78109859	78218644	0.0181454	0.0186554	0	0.1011557
LOC100129307	NC_000013	78235511	78236536	0	0	0	0
SLAIN1	NC_000013	78272470	78338377	0	0.0340365	0.4417804	0.0346044
EDNRB	NC_000013	78469616	78492966	0.0204126	0.083945	0.0453989	0.0426728
RPL31P54	NC_000013	78996958	78997309	0	0	0	0
POU4F1	NC_000013	79173227	79177695	0.4605526	0.2959353	0.1024301	0.056163
RNF219	NC_000013	79188421	79233314	3.3825464	2.1862936	2.1352094	3.0676873
RPL21P111	NC_000013	79335269	79335821	0	0	0	0
LOC647298	NC_000013	79423366	79425343	0	0	0	0
LOC390415	NC_000013	79483065	79484682	0	0	0	0
LOC100128339	NC_000013	79740356	79741027	0	0	0	0
RBM26	NC_000013	79894089	79979923	3.6138447	2.4686296	2.7294934	4.0985281
NDFIP2	NC_000013	80055287	80128221	6.5188766	2.9310932	3.6487106	4.7168819
LOC729479	NC_000013	80585271	80600798	0	0	0	0
SPRY2	NC_000013	80910111	80915086	7.5571806	2.5256473	1.7039204	2.2051073
LOC100129459	NC_000013	81228890	81231137	0	0	0	0
LOC100129023	NC_000013	81455698	81463020	0	0	0	0
LOC100287316	NC_000013	81807668	81847305	0	0	0	0
PTMAP5	NC_000013	82264031	82265215	0	0	0	0
LOC647317	NC_000013	83397265	83398278	0	0	0	0
SLITRK1	NC_000013	84451343	84456528	0.0508464	0.0261377	0.0226172	0.0472424
VENTXP2	NC_000013	84481336	84482016	0	0	0	0
LOC100287345	NC_000013	84481436	84481992	0	0	0	0
LOC100131800	NC_000013	84594549	84594877	0	0	0	0
LOC100287383	NC_000013	85071741	85098047	0	0	0	0
SLITRK6	NC_000013	86366922	86373483	0.0105114	0.0324205	0.037405	0.0146495
LOC100130242	NC_000013	86543052	86543741	0	0	0	0
LOC100130117	NC_000013	86927755	86929582	0	0	0	0
LOC100287388	NC_000013	88031721	88032939	0	0	0	0
LOC100289603	NC_000013	88323637	88328080	0.0345234	0	0	0
SLITRK5	NC_000013	88324870	88331871	0.0098827	0.0101604	0	0.0413198
LOC100287432	NC_000013	88459889	88463481	0.0244633	0.0251508	0.0326448	0.0340939
CXXC6P1	NC_000013	88542198	88545652	0	0	0	0
RPL29P29	NC_000013	88888817	88889224	0	0	0	0
LOC100131026	NC_000013	89630570	89631075	0	0	0	0
LOC100132195	NC_000013	89901114	89902925	0	0	0	0
SP3P	NC_000013	90012120	90015754	0	0	0	0
LOC100129260	NC_000013	90485555	90486306	0	0	0	0
LOC121727	NC_000013	90643797	90645149	0	0	0	0
LOC100128011	NC_000013	90815579	90824164	0	0	0	0
KRT18P27	NC_000013	90882585	90883991	0	0	0	0
LOC144776	NC_000013	91543208	91578851	0	0	0	0
LOC390419	NC_000013	91924335	91924831	0	0	0	0
MIRHG1	NC_000013	92002443	92002655	0	0	0	0
GPC5	NC_000013	92050935	93519490	0.0152439	0	0.9357378	2.7087561
GPC6	NC_000013	93879078	95060274	3.8422993	0.4007538	6.7263445	4.9324033
DCT	NC_000013	95091835	95131936	0.0182889	0.0376058	0.0325406	0.0509777
TGDS	NC_000013	95226308	95248511	1.8485061	1.4728554	3.3917447	1.7228507
LOC100289676	NC_000013	95253857	95264638	0.1056448	0.1086139	0.0469923	0.1840437
GPR180	NC_000013	95254104	95281946	3.6418091	2.2925795	7.0279918	4.5991977
LOC100129538	NC_000013	95293724	95356156	0	0.0524778	0	0
SOX21	NC_000013	95361876	95364389	0.0174814	0	0.0155519	0
LOC100129259	NC_000013	95409705	95411526	0	0	0	0
RPL21P112	NC_000013	95412768	95413307	0	0	0	0
LOC100286897	NC_000013	95617579	95620345	0	0	0.0551447	0
ABCC4	NC_000013	95672083	95953687	0.9349192	1.2141413	1.3632868	3.2819367
CLDN10	NC_000013	96085858	96231906	0.0312022	0.0160395	0.0416375	0.0652287
DZIP1	NC_000013	96230456	96296957	2.4826525	1.5507658	1.770042	1.9467738
LOC100289274	NC_000013	96326410	96329208	0.1413126	0.0484281	0.0838104	0.0218827
DNAJC3	NC_000013	96329402	96447242	4.9137604	2.8659603	4.7076804	5.6903779
UGCGL2	NC_000013	96453834	96705637	3.1378601	1.9543317	4.6120887	4.259089

HS6ST3	NC_000013	96743093	97491812	0.0056344	0.0173782	0.0451126	0.0353364
HSP90AB6P	NC_000013	97535897	97541419	0	0	0	0
LOC100128017	NC_000013	97598849	97601181	0	0	0	0
OXGR1	NC_000013	97637973	97646604	0.0192334	0	0	0
LOC100286928	NC_000013	97874572	97876907	0	0	0.0334739	0
MBNL2	NC_000013	97874574	98046374	1.1865275	2.0171162	3.6737083	4.5833151
LOC727934	NC_000013	98084326	98086084	0.0999391	0.025687	0.1333629	0.3830286
RAP2A	NC_000013	98086475	98120252	4.0438827	3.2347915	5.1226962	8.0110971
LOC121906	NC_000013	98127124	98128673	0	0	0	0.074786
RPL7AP61	NC_000013	98553906	98554507	0	0	0	0
IPO5	NC_000013	98605929	98676550	6.8170283	4.3738015	5.4718352	14.210311
FARP1	NC_000013	98795434	99102023	2.9554037	1.3381844	2.1864666	4.5190369
RNF113B	NC_000013	98828039	98829521	0	0	0	0
STK24	NC_000013	99102455	99229396	10.219277	5.3081574	8.3627768	11.432378
LOC100287024	NC_000013	99109725	99111809	0	0	0	0.0146882
LOC100130167	NC_000013	99262540	99263121	0	0	0	0
CYCSP35	NC_000013	99281938	99282250	0	0	0	0
LOC643193	NC_000013	99293068	99293493	0	0	0	0
SLC15A1	NC_000013	99336055	99404929	0.0421633	0.0144494	0	0.0195874
DOCK9	NC_000013	99445741	99738660	0.6987174	0.6173363	1.0877971	2.2518852
LOC100129122	NC_000013	99739204	99740879	0.0524442	0.0269591	0.3265906	0.1644534
RPS6P23	NC_000013	99825808	99826745	0	0	0	0
LOC390421	NC_000013	99842833	99843245	0	0	0	0
LOC100289373	NC_000013	99849810	99853015	0.4717163	0.4849739	0.3497099	0.2191405
UBAC2	NC_000013	99852679	100038753	6.7030677	4.6397936	7.8407682	8.8054618
LOC728023	NC_000013	99867626	99868012	0	0	0	0
GPR18	NC_000013	99906967	99910682	0	0	0	0
GPR183	NC_000013	99946789	99959749	0	0	0	0
FKSG29	NC_000013	100003674	100004281	0	0	0	0
LOC100133168	NC_000013	100060033	100061300	0	0	0	0
TM9SF2	NC_000013	100153728	100215276	13.686682	9.5132906	14.745461	13.652363
LOC100129537	NC_000013	100234962	100235558	0	0	0	0
CLYBL	NC_000013	100425078	100544824	0.3158903	0.040596	0.9835866	0.4952811
ZIC5	NC_000013	100615275	100624178	0.0095602	0.0098289	0	0.0133239
ZIC2	NC_000013	100634319	100639019	0.0327727	0.0505407	0.0145778	0
PCCA	NC_000013	100741337	101182689	0.8282145	0.8333747	1.1443955	1.338457
RPL15P18	NC_000013	100802694	100803397	0	0	0	0
A2LD1	NC_000013	101183810	101185997	0.7163526	0.3314186	0.6372871	0.4243056
RPS26P47	NC_000013	101192070	101192526	0	0	0	0
TMTC4	NC_000013	101256090	101327103	0.292676	0.1925771	0.458256	0.6771084
ARF4P3	NC_000013	101407646	101409416	0	0	0	0
NALCN	NC_000013	101706130	102068813	0.1396593	0.0978985	0.4743891	1.0660979
ITGBL1	NC_000013	102104966	102368794	1.9031311	1.4131136	0.7368026	2.2594136
FGF14	NC_000013	102373205	103054124	0.0726657	0.1045912	0.0905036	0.4962365
LOC100131188	NC_000013	102654571	102655649	0	0	0	0
LOC100126007	NC_000013	102668922	102671150	0	0	0	0
RPL39P29	NC_000013	102915195	102915603	0	0	0	0
LOC283481	NC_000013	103046928	103048054	0	0	0	0
TPP2	NC_000013	103249286	103331524	7.1365734	4.718793	5.9173348	12.104335
C13orf39	NC_000013	103338097	103346854	0	0.1249311	0.0360346	0.0282257
LOC643677	NC_000013	103381717	103411120	0.0168432	0.0303041	0.0131112	0.0366783
C13orf27	NC_000013	103418460	103426149	0.6860956	0.627003	1.0851016	0.5312208
KDELC1	NC_000013	103436631	103451404	2.8367155	5.4468838	4.8616812	3.2994055
BIVM	NC_000013	103451399	103493881	3.3727448	2.3077859	4.2067643	5.5501141
ERCC5	NC_000013	103498174	103528345	0.3111569	0.319902	0.6013542	1.4131108
LOC121952	NC_000013	103532449	103548383	0	0	0	0
SLC10A2	NC_000013	103696348	103719196	0.0116296	0.0119564	0.010346	0
RPL7P45	NC_000013	105466662	105467486	0	0	0	0
DAOA	NC_000013	106118364	106143383	0	0	0	0.0631441
LOC728192	NC_000013	107029119	107030140	0	0	0	0
RPL35P7	NC_000013	107085687	107094264	0	0	0	0
EFNB2	NC_000013	107142093	107187337	1.9523509	0.0104543	0.7417863	0.3117757
ARGLU1	NC_000013	107195662	107220514	5.4966663	2.8384776	5.2472461	8.2727398
LOC390424	NC_000013	107315785	107316300	0.085171	0.0875647	0	0
LOC122335	NC_000013	107515839	107516777	0	0	0	0
FAM155A	NC_000013	107820879	108519083	1.2747764	0.0389288	0.1347418	0.3430127

LIG4	NC_000013	108859790	108870716	0.6454077	0.3110376	0.6100584	0.6605641
ABHD13	NC_000013	108870763	108886603	0.6631193	0.3323562	0.4793176	0.8895193
TNFSF13B	NC_000013	108921977	108959365	3.3216687	0.2626942	4.6111747	0.1780516
MYO16	NC_000013	109248500	109860355	0.159835	0.0328654	0.0625652	0.0356414
IRS2	NC_000013	110406184	110438914	1.3753448	0.8458168	1.1900231	1.0765533
LOC728767	NC_000013	110434126	110460599	0.5053993	0.420105	0.5452805	0.247277
COL4A1	NC_000013	110801310	110959496	8.5716545	3.7837295	2.5558268	3.0146658
COL4A2	NC_000013	110959631	111165374	28.120133	12.702422	19.024486	8.2233469
LOC100129836	NC_000013	111109000	111115441	0	0	0	0
RAB20	NC_000013	111175413	111214071	1.0193427	0.8383931	0.1036383	0.2638326
CARKD	NC_000013	111268008	111292343	3.4339715	2.019844	3.2899529	3.4973565
CARS2	NC_000013	111293759	111358463	11.684554	2.623236	9.3766272	18.893373
LOC100289537	NC_000013	111294579	111298625	0	0	0.0554575	0.0868791
LOC100131435	NC_000013	111362831	111365875	0.0432988	0.0890313	0.0385198	0.1106317
ING1	NC_000013	111365083	111373421	0.2674552	0.2199776	0.4092491	0.2012833
RPL21P107	NC_000013	111415003	111415525	0	0	0	0
LOC283486	NC_000013	111462023	111465435	0.0386301	0.0529545	0.0458219	0.008973
LOC100129390	NC_000013	111465811	111471491	0	0	0	0.0460524
C13orf29	NC_000013	111520519	111522655	0	0	0	0
ANKRD10	NC_000013	111530887	111567416	2.3251169	1.3582185	4.3406917	5.3394076
PARP1P1	NC_000013	111589050	111592578	0	0	0	0
ARHGEF7	NC_000013	111767624	111958081	3.2886091	1.9709844	3.0146984	3.8193897
C13orf16	NC_000013	111973015	111996594	0.1239724	0.0637284	0	0.0431945
LOC100287321	NC_000013	112111256	112112975	0.0783391	0.0805408	0	0.1091796
SOX1	NC_000013	112721913	112726020	0.1069821	0.0329966	0.0190348	0.0521846
C13orf28	NC_000013	113030669	113089001	0	0	0.051648	0.0809112
TUBGCP3	NC_000013	113139328	113242481	3.4119193	3.995653	6.3621489	7.6286651
C13orf35	NC_000013	113301358	113338811	0	0.0201622	0.0174465	0.0136657
ATP11A	NC_000013	113344643	113541482	0.3424538	0.2755397	0.4194544	0.6225272
LOC100289410	NC_000013	113621834	113623676	0	0.0446476	0	0
MCF2L	NC_000013	113622757	113752862	0.0387005	0.2307712	0.0275432	0.026968
F7	NC_000013	113760105	113774995	0.0421903	0.231339	0.0250224	0
F10	NC_000013	113777113	113803843	0.0858364	0.2941628	0.0254541	0.1395665
LOC100289447	NC_000013	113786069	113807115	0	0.1699689	0.0245126	0.0960028
PROZ	NC_000013	113812968	113826694	0	0.0304265	0.0263283	0.1237369
PCID2	NC_000013	113831925	113863029	7.0345403	3.1686433	9.2092767	11.377765
CUL4A	NC_000013	113863086	113919392	3.190886	2.2801099	3.4930111	5.8505818
LOC100289475	NC_000013	113922321	113932049	0	0	0	0
LAMP1	NC_000013	113951469	113977741	62.323437	25.793674	82.181852	46.938052
GRTP1	NC_000013	113978505	114018463	0.1250305	0.3856336	0.2502689	0.1524709
LOC100289508	NC_000013	114002381	114016183	0	0	0	0
DKFZp451A211	NC_000013	114054764	114062504	0	0	0	0
ADPRHL1	NC_000013	114076260	114107839	0.0404494	0	0.0359849	0.0704668
DCUN1D2	NC_000013	114110134	114145023	1.0591683	0.4325912	1.9103466	2.2243226
TMCO3	NC_000013	114145308	114204544	8.5304572	4.14352	12.897382	17.495677
TFDP1	NC_000013	114239003	114295788	3.6637341	1.87483	5.383105	6.7464839
ATP4B	NC_000013	114303119	114312501	0	0.0607304	0	0.0205812
GRK1 (NC_000013 1143	NC_000013	114321594	114325992	0.0394508	0.1216788	0.0350966	0.1099637
GRK1 (NC_000013 1144	NC_000013	114426046	114438636	0.0435994	0	0	0
LOC100130386	NC_000013	114451484	114453515	0.0216281	0.0222359	0.0384819	0
FAM70B	NC_000013	114462216	114514899	5.5841109	0.262949	5.0436239	5.6734741
GAS6	NC_000013	114523522	114567046	47.461388	9.874825	29.771449	35.936957
LOC100128430	NC_000013	114545293	114546167	0.0502266	0	0	0
FLJ44054	NC_000013	114586610	114626485	0	0	0	0
RASA3	NC_000013	114747194	114898095	1.4649411	1.0112476	1.2846343	1.9833255
LOC100286952	NC_000013	114964051	114965825	0	0	0	0
CDC16	NC_000013	115000362	115038150	10.023953	4.5124287	7.877486	13.182187
UPF3A	NC_000013	115047078	115071283	0.5589008	0.2873044	0.8121156	0.6101608
LOC100128145	NC_000013	115072065	115072671	0	0	0	0
ZNF828	NC_000013	115080015	115092797	5.2219191	2.8464823	7.7841777	9.7849824
LOC100132257	NC_000014	19110324	19118312	0	0	0	0
LOC644734	NC_000014	19170910	19190616	0	0	0	0
LOC100129777	NC_000014	19190626	19195750	0	0	0	0
OR11H12	NC_000014	19377594	19378574	0	0	0	0
LOC100128514	NC_000014	19399050	19403260	0	0	0	0
USP10P2	NC_000014	19410589	19413794	0	0	0	0

LOC100132698	NC_000014	19421519	19422698	0	0	0	0
RPL22P20	NC_000014	19468509	19468885	0	0	0	0
LOC100158258	NC_000014	19474524	19476597	0	0	0	0
NF1L4	NC_000014	19489122	19495427	0	0	0	0
MED15P	NC_000014	19497920	19502484	0	0	0	0
POTEG	NC_000014	19553365	19584942	0	0	0	0
LOC100287445	NC_000014	19619479	19643375	0.2592816	0.1332844	0	0
LOC100287820	NC_000014	19683435	19688209	0	0.056775	0.0245639	0.0064136
DUXAP10	NC_000014	19691736	19694003	0	0	0	0
LOC642661	NC_000014	19719057	19720591	0	0	0	0
LOC642669	NC_000014	19742756	19758827	0	0	0	0
OR11H13P	NC_000014	19806453	19807382	0	0	0	0
LOC727980	NC_000014	19854816	19856379	0	0	0	0
DUXAP9	NC_000014	19881382	19883648	0	0	0	0
FLJ39632	NC_000014	19887179	19891953	0	0	0	0
LOC100288480	NC_000014	19932014	19955914	0	0.2673574	0.3470198	0.8154553
P704P	NC_000014	19983954	20020272	0.0527432	0.0542255	0.0762479	0.1653909
LOC642850	NC_000014	20021205	20074901	0	0	0	0
NF1L7	NC_000014	20078032	20087766	0	0	0	0
NEK2P	NC_000014	20097140	20099190	0	0	0	0
LOC400174	NC_000014	20136988	20138162	0	0	0	0
USP10P1	NC_000014	20145907	20149106	0	0	0	0
LOC254398	NC_000014	20156420	20159406	0	0	0	0
OR11H8P	NC_000014	20180895	20182275	0	0	0	0
OR11H2	NC_000014	20180998	20182142	0	0	0	0
OR11K2P	NC_000014	20201405	20202552	0	0	0	0
OR4Q3	NC_000014	20215587	20216528	0	0	0	0.0325105
OR4H12P	NC_000014	20228069	20228947	0	0	0	0
OR4M1	NC_000014	20248482	20249423	0	0	0	0
OR4N1P	NC_000014	20264401	20265549	0	0	0	0
OR4N2	NC_000014	20295608	20296531	0	0.0977996	0	0
OR4K6P	NC_000014	20315426	20316488	0	0	0	0
OR4K3	NC_000014	20336156	20337502	0	0	0	0
OR4K2	NC_000014	20344427	20345371	0	0	0	0.0324073
OR4K4P	NC_000014	20373871	20374803	0	0	0	0
OR4K5	NC_000014	20388766	20389737	0	0.046485	0	0
OR4K1	NC_000014	20403826	20404761	0	0	0	0
OR4K16P	NC_000014	20424706	20425713	0	0	0	0
OR4K15	NC_000014	20443678	20444724	0	0	0	0.0292501
OR4Q2	NC_000014	20470222	20471352	0	0	0	0
OR4K14	NC_000014	20482420	20483352	0	0	0.0419052	0.0328241
OR4K13	NC_000014	20502003	20502917	0	0	0	0
OR4U1P	NC_000014	20512075	20513014	0	0	0	0
OR4L1	NC_000014	20528204	20529142	0.0468032	0	0	0.0652287
OR4T1P	NC_000014	20561414	20562534	0	0	0	0
OR4K17	NC_000014	20585566	20586597	0	0	0.0757705	0.0296753
OR4N5	NC_000014	20611895	20612821	0	0	0	0.0330365
OR11G1P	NC_000014	20647287	20648437	0	0	0	0
OR11P1P	NC_000014	20652084	20652457	0	0	0	0
OR11G2	NC_000014	20665495	20666532	0	0	0	0
OR11H5P	NC_000014	20677356	20678454	0	0	0	0
OR11H6	NC_000014	20691869	20692861	0	0	0.0393732	0.0308408
OR11H7	NC_000014	20697461	20698602	0	0	0	0
OR11H4	NC_000014	20710951	20711925	0.0450751	0.046342	0	0
TTC5	NC_000014	20757304	20774136	2.64404	2.718351	0.7840704	1.8258735
CCNB1IP1	NC_000014	20779529	20801457	3.6720363	4.8265718	4.0317426	4.5832049
SNORD126	NC_000014	20794600	20794698	0	0	0	0
RPPH1	NC_000014	20811230	20811570	0	0	0	0
PARP2	NC_000014	20811773	20826063	3.7264002	7.1594262	3.6673392	6.6702835
TEP1	NC_000014	20835926	20881580	1.145364	0.7464855	0.5686092	1.6247754
KLHL33	NC_000014	20897008	20898594	0.0322674	0	0.057412	0.0899409
OSGEP	NC_000014	20915216	20923198	6.0794581	8.0027486	5.5095471	9.3438543
APEX1	NC_000014	20923290	20925926	2.6403007	4.0863542	2.1215733	5.5196002
LOC100288648	NC_000014	20923385	20925355	0.1147473	0.1179723	0.1020824	0.3198421
TMEM55B	NC_000014	20926012	20929637	4.2563632	4.0860136	2.0985858	2.6443886
NP	NC_000014	20937542	20945248	20.245485	24.097814	15.195921	23.193241

GAFA1	NC_000014	20947799	20948350	0.1592327	0.081854	0.0708289	0
RNASE10	NC_000014	20978631	20979281	0	0	0	0
LOC100131302	NC_000014	20992445	20993278	0	0	0	0
LOC100133314	NC_000014	20996269	20997119	0	0	0	0
RNASE9	NC_000014	21024252	21029090	0	0.0362337	0	0.0245588
RNASE11	NC_000014	21051051	21058417	0.0248717	0	0	0
RNASE12	NC_000014	21058439	21058882	0	0	0	0
RPS26P9	NC_000014	21084803	21085150	0	0	0	0
OR6S1	NC_000014	21108855	21109850	0.0441247	0	0.0392546	0
TRNAL40P	NC_000014	21145178	21145259	0	0	0	0
ANG	NC_000014	21152336	21162345	0	0.0682529	0	0
RNASE4	NC_000014	21152372	21168758	4.0396156	1.6708073	4.9775559	3.623863
RANBP20P	NC_000014	21188964	21191300	0	0	0	0
LOC100128220	NC_000014	21202737	21203173	0	0	0	0
FAM12A	NC_000014	21214099	21216539	0	0.0514032	0.0444796	0.0348406
FAM12B	NC_000014	21236586	21239107	0	0.0504279	0	0
RNASE6	NC_000014	21249210	21250626	0.0414215	0	0	0.0577283
LOC100287557	NC_000014	21260999	21270492	0	0	0	0
RNASE1	NC_000014	21269515	21271036	0	0	0.0403068	0.031572
RNASE3	NC_000014	21359562	21360507	0	0	0	0
LOC643332	NC_000014	21387500	21388335	0	0	0	0
RNASE2	NC_000014	21423630	21424594	0.0597935	0	0	0
LOC100130571	NC_000014	21448911	21457294	0	0	0	0
METT11D1	NC_000014	21457965	21465194	3.5926326	5.0597315	3.7652751	7.3389966
SLC39A2	NC_000014	21467419	21470030	0	0	0.0554969	0.0869407
NDRG2	NC_000014	21484922	21493935	0.0999278	0.82189	0.3022549	0.0835604
TPPP2	NC_000014	21498456	21500327	0	0	0	0.0449705
RNASE13	NC_000014	21500979	21502944	0	0.0281166	0	0
RNASE7	NC_000014	21510385	21512392	0	0	0	0
RNASE8	NC_000014	21526052	21526516	0	0.1943372	0	0
FLJ10357	NC_000014	21538527	21558036	7.2871289	10.488708	2.3710689	2.4520102
ZNF219	NC_000014	21558205	21572863	0.7581639	0.6885337	1.2140705	2.9321692
C14orf176	NC_000014	21569511	21571883	0.0418156	0.0859817	0	0
LOC554207	NC_000014	21584645	21584773	0	0	0	0
OR5AU1	NC_000014	21623096	21624184	0	0.0414907	0	0
LOC401744	NC_000014	21630671	21631949	0	0	0	0
LOC100129923	NC_000014	21652949	21653148	0	0	0	0
HNRNPC	NC_000014	21677295	21737638	43.55632	56.993332	61.519755	97.93934
RPGRIP1	NC_000014	21756136	21819460	0	0.0687026	0.0297245	0.038805
MRPS17P6	NC_000014	21759398	21759608	0	0	0	0
SUPT16H	NC_000014	21819631	21852425	2.3550397	4.0032263	2.6293196	7.8850562
LOC728353	NC_000014	21852451	21853084	0	0	0	0
CHD8	NC_000014	21853358	21905404	1.6057666	2.2296245	1.6446596	4.0794634
SNORD9	NC_000014	21860310	21860412	0	0	0	0
SNORD8	NC_000014	21865452	21865560	0	0	0	0
UBA52P2	NC_000014	21866947	21867486	0	0	0	0
EIF4EBP1P	NC_000014	21887658	21888665	0	0	0	0
RAB2B	NC_000014	21927188	21945121	2.7670402	2.3739434	2.7841949	3.5505178
TOX4	NC_000014	21945335	21967319	5.3720982	6.1622809	4.4161928	9.1048765
METTL3	NC_000014	21966282	21979457	7.3734118	7.4650838	7.4795343	17.857985
SALL2	NC_000014	21989231	22005337	0.1252341	0.0275901	0.0477479	0.0187003
LOC100288451	NC_000014	22031950	22034351	0	0	0	0
OR10G3	NC_000014	22037934	22038875	0	0	0	0
LOC100288580	NC_000014	22053413	22054404	0	0	0	0
OR10G1P	NC_000014	22069935	22071076	0	0	0	0
LOC100288613	NC_000014	22080078	22080535	0	0	0	0
TRA@	NC_000014	22090057	23021075	0	0	0	0
TRAV1-1	NC_000014	22090057	22090759	0	0	0	0
OR10G2	NC_000014	22102066	22102998	0	0	0	0
TRAV1-2	NC_000014	22111193	22111806	0	0	0	0
LOC100288702	NC_000014	22117944	22118553	0	0	0	0
OR4E2	NC_000014	22133297	22134238	0.0466542	0	0.1245145	0
OR4E1	NC_000014	22138104	22139250	0	0	0	0
TRAV2	NC_000014	22180554	22181066	0	0	0	0
TRAV3	NC_000014	22192136	22192568	0	0	0	0
TRAV4	NC_000014	22204501	22205264	0	0	0	0

TRAV5	NC_000014	22217474	22217989	0	0	0	0
RPL4P1	NC_000014	22218496	22219914	0	0	0	0
TRAV6	NC_000014	22236793	22237320	0	0	0	0
TRAV7	NC_000014	22251210	22251720	0	0	0	0
TRAV8-1	NC_000014	22265591	22266058	0	0	0	0
TRAV9-1	NC_000014	22279552	22280188	0	0	0	0
TRAV10	NC_000014	22293672	22294239	0	0	0	0
TRAV11	NC_000014	22297692	22298223	0	0	0	0
TRAV12-1	NC_000014	22309425	22309956	0	0	0	0
TRAV8-2	NC_000014	22314944	22315403	0	0	0	0
TRAV8-3	NC_000014	22320735	22321183	0	0	0	0
TRAV13-1	NC_000014	22337043	22337546	0	0	0	0
TRAV12-2	NC_000014	22356139	22356682	0	0	0	0
TRAV8-4	NC_000014	22362741	22363217	0	0	0	0
TRAV8-5	NC_000014	22371257	22372778	0	0	0	0
TRAV13-2	NC_000014	22386433	22386931	0	0	0	0
TRAV14DV4	NC_000014	22392314	22392827	0	0	0	0
TRAV9-2	NC_000014	22409369	22409848	0	0	0	0
TRAV15	NC_000014	22418334	22418877	0	0	0	0
TRAV12-3	NC_000014	22433736	22434290	0	0	0	0
TRAV8-6	NC_000014	22446919	22447360	0	0	0	0
TRAV16	NC_000014	22458735	22459177	0	0	0	0
TRAV17	NC_000014	22465925	22466407	0	0	0	0
TRAV18	NC_000014	22471450	22471912	0	0	0	0
TRAV19	NC_000014	22475868	22476420	0	0	0	0
TRAV20	NC_000014	22508909	22509399	0	0	0	0
TRAV21	NC_000014	22520780	22521315	0	0	0	0
TRAV22	NC_000014	22538953	22539471	0	0	0	0
TRAV23DV6	NC_000014	22554729	22555238	0	0	0	0
TRDV1	NC_000014	22564327	22564896	0	0	0	0
TRAV24	NC_000014	22573620	22574123	0	0	0	0
TRAV25	NC_000014	22580376	22580984	0	0	0	0
TRAV26-1	NC_000014	22591485	22592243	0	0	0	0
TRAV8-7	NC_000014	22600515	22600996	0	0	0	0
TRAV27	NC_000014	22616046	22616587	0	0	0	0
TRAV28	NC_000014	22623012	22623571	0	0	0	0
TRAV29DV5	NC_000014	22631245	22631766	0	0	0	0
TRAV30	NC_000014	22636325	22636884	0	0	0	0
TRAV31	NC_000014	22645170	22645761	0	0	0	0
TRAV32	NC_000014	22653458	22653957	0	0	0	0
TRAV33	NC_000014	22658052	22658608	0	0	0	0
TRAV26-2	NC_000014	22670477	22671262	0	0	0	0
TRAV34	NC_000014	22675430	22676025	0	0	0	0
TRAV35	NC_000014	22689792	22690371	0	0	0	0
TRAV36DV7	NC_000014	22694657	22695149	0	0	0	0
TRAV37	NC_000014	22733632	22735056	0	0	0	0
TRAV38-1	NC_000014	22739854	22740446	0	0	0	0
TRAV38-2DV8	NC_000014	22749034	22749631	0	0	0	0
TRAV39	NC_000014	22771939	22772438	0	0	0	0
TRAV40	NC_000014	22782922	22783351	0	0	0	0
TRAV41	NC_000014	22788620	22789123	0	0	0	0
TRD@	NC_000014	22891537	22935569	0	0	0	0
TRDV2	NC_000014	22891537	22892033	0	0	0	0
TRDD1	NC_000014	22907539	22907546	0	0	0	0
TRDD2	NC_000014	22907999	22908007	0	0	0	0
TRDD3	NC_000014	22918105	22918117	0	0	0	0
TRDJ1	NC_000014	22919081	22919131	0	0	0	0
TRDJ4	NC_000014	22924241	22924288	0	0	0	0
TRDJ2	NC_000014	22925681	22925734	0	0	0	0
TRDJ3	NC_000014	22928090	22928148	0	0	0	0
TRDC	NC_000014	22931906	22935569	0	0	0	0
TRDV3	NC_000014	22938033	22938606	0	0	0	0
TRAJ61	NC_000014	22944306	22944365	0	0	0	0
TRAJ60	NC_000014	22945296	22945352	0	0	0	0
TRAJ59	NC_000014	22945543	22945596	0	0	0	0
TRAJ58	NC_000014	22946696	22946758	0	0	0	0

TRAJ57	NC_000014	22947861	22947923	0	0	0	0
TRAJ56	NC_000014	22948510	22948571	0	0	0	0
TRAJ55	NC_000014	22950686	22950742	0	0	0	0
TRAJ54	NC_000014	22951276	22951335	0	0	0	0
TRAJ53	NC_000014	22951993	22952058	0	0	0	0
TRAJ52	NC_000014	22955216	22955284	0	0	0	0
TRAJ51	NC_000014	22956171	22956233	0	0	0	0
TRAJ50	NC_000014	22957581	22957640	0	0	0	0
TRAJ49	NC_000014	22958476	22958531	0	0	0	0
TRAJ48	NC_000014	22959479	22959541	0	0	0	0
TRAJ47	NC_000014	22961838	22961894	0	0	0	0
TRAJ46	NC_000014	22962389	22962451	0	0	0	0
TRAJ45	NC_000014	22962911	22962976	0	0	0	0
TRAJ44	NC_000014	22963806	22963868	0	0	0	0
TRAJ43	NC_000014	22964896	22964949	0	0	0	0
TRAJ42	NC_000014	22965872	22965937	0	0	0	0
TRAJ41	NC_000014	22966642	22966703	0	0	0	0
TRAJ40	NC_000014	22968672	22968732	0	0	0	0
TRAJ39	NC_000014	22970585	22970647	0	0	0	0
TRAJ38	NC_000014	22971215	22971276	0	0	0	0
TRAJ37	NC_000014	22972735	22972797	0	0	0	0
TRAJ36	NC_000014	22974096	22974155	0	0	0	0
TRAJ35	NC_000014	22975629	22975687	0	0	0	0
TRAJ34	NC_000014	22976651	22976708	0	0	0	0
TRAJ33	NC_000014	22977587	22977643	0	0	0	0
TRAJ32	NC_000014	22978325	22978390	0	0	0	0
TRAJ31	NC_000014	22979951	22980007	0	0	0	0
TRAJ30	NC_000014	22981834	22981890	0	0	0	0
TRAJ29	NC_000014	22982921	22982980	0	0	0	0
TRAJ28	NC_000014	22984601	22984666	0	0	0	0
TRAJ27	NC_000014	22985251	22985309	0	0	0	0
TRAJ26	NC_000014	22987424	22987483	0	0	0	0
TRAJ25	NC_000014	22987790	22987849	0	0	0	0
TRAJ24	NC_000014	22988947	22989009	0	0	0	0
TRAJ23	NC_000014	22989394	22989456	0	0	0	0
TRAJ22	NC_000014	22991016	22991078	0	0	0	0
TRAJ21	NC_000014	22992573	22992627	0	0	0	0
TRAJ20	NC_000014	22993296	22993352	0	0	0	0
TRAJ19	NC_000014	22994233	22994292	0	0	0	0
TRAJ18	NC_000014	22994620	22994685	0	0	0	0
TRAJ17	NC_000014	22995812	22995874	0	0	0	0
TRAJ16	NC_000014	22997487	22997546	0	0	0	0
TRAJ15	NC_000014	22998580	22998641	0	0	0	0
TRAJ14	NC_000014	22999278	22999329	0	0	0	0
TRAJ13	NC_000014	23000026	23000088	0	0	0	0
TRAJ12	NC_000014	23000889	23000948	0	0	0	0
TRAJ11	NC_000014	23001452	23001511	0	0	0	0
TRAJ10	NC_000014	23002445	23002508	0	0	0	0
TRAJ9	NC_000014	23004502	23004562	0	0	0	0
TRAJ8	NC_000014	23005092	23005151	0	0	0	0
TRAJ7	NC_000014	23006567	23006625	0	0	0	0
TRAJ6	NC_000014	23008016	23008077	0	0	0	0
TRAJ5	NC_000014	23009190	23009249	0	0	0	0
TRAJ4	NC_000014	23011142	23011204	0	0	0	0
TRAJ3	NC_000014	23012122	23012183	0	0	0	0
TRAJ2	NC_000014	23013015	23013080	0	0	0	0
TRAJ1	NC_000014	23013981	23014042	0	0	0	0
TRAC	NC_000014	23016447	23021083	0	0	0	0
DAD1	NC_000014	23033807	23058143	34.048845	30.856957	33.656441	39.544319
ABHD4	NC_000014	23067147	23081265	5.2198378	5.708704	3.1945002	6.9330135
OR6J1	NC_000014	23102660	23103649	0	0	0	0
RPL26P2	NC_000014	23163261	23163790	0	0	0	0
OR6E1P	NC_000014	23171252	23172377	0	0	0	0
OXA1L	NC_000014	23235731	23240998	13.447801	17.873597	7.7103402	15.58858
SLC7A7	NC_000014	23242431	23289014	0.3282168	0.0337441	0.1167963	0.125793
MRPL52	NC_000014	23299092	23304246	10.167885	11.287168	4.0269591	7.1795449

MMP14	NC_000014	23305793	23316803	182.15287	170.67015	68.282605	49.702671
LOC100288791	NC_000014	23311138	23315194	0.163579	0.0560588	0.0970163	0.1899806
LRP10	NC_000014	23340960	23347293	36.880297	26.190256	19.596873	24.974532
REM2	NC_000014	23352432	23356889	0.0945632	0.0729157	0.042063	0.0494215
RBM23	NC_000014	23369854	23388396	7.2403193	9.1269084	3.5734334	7.5386125
PRMT5	NC_000014	23389733	23398661	13.415776	20.707535	6.1732998	13.155059
HAUS4	NC_000014	23415437	23426320	10.389263	9.550963	5.1836672	10.725411
JUB	NC_000014	23440409	23451851	6.9343218	5.2065138	2.1908474	5.038557
LOC100288817	NC_000014	23447821	23450982	0.0932094	0.2874872	0.0829217	0
C14orf93	NC_000014	23456110	23479360	1.0446711	0.8518182	0.9934627	1.4559368
PSMB5	NC_000014	23495060	23504429	42.829879	49.471785	18.473196	41.156129
PSMB11	NC_000014	23511376	23513269	0	0.0715682	0	0.0161694
CDH24	NC_000014	23516270	23526747	2.7707301	1.818527	1.9367215	2.7151243
LOC100288857	NC_000014	23516410	23517348	0	0.0524778	0	0.1422759
ACIN1	NC_000014	23527773	23564823	3.6490026	4.2365158	2.1615301	6.3631278
C14orf119	NC_000014	23564788	23569665	7.5980922	8.9253128	4.9560294	6.3622106
LOC100128908	NC_000014	23569631	23574171	0	0	0	0
CEBPE	NC_000014	23586515	23588474	0	0	0	0.1019979
SLC7A8	NC_000014	23594504	23652849	3.1254765	1.5341004	0.9597246	1.0468242
RPL39P2	NC_000014	23698977	23699341	0	0	0	0
HMGN2L1	NC_000014	23732310	23733000	0	0	0	0
HOMEZ	NC_000014	23742844	23755309	1.1395291	1.485581	1.0346589	1.7846093
H3F3AP2	NC_000014	23763755	23765009	0	0	0	0
PPP1R3E	NC_000014	23765112	23772057	0	0	0	0
BCL2L2	NC_000014	23776012	23780968	6.979433	5.337321	2.2817883	4.3565694
PABPN1	NC_000014	23789397	23795372	6.9774952	7.7897358	3.9732266	10.609333
SLC22A17	NC_000014	23815527	23822080	2.9724323	2.7934966	0.1297845	0.0635371
EFS	NC_000014	23825611	23834842	3.5942268	1.651991	1.2790095	2.0626121
IL25	NC_000014	23842018	23845612	0	0	0	0.022403
CMTM5	NC_000014	23846017	23848981	0.0366235	0	0.0325813	0
MYH6	NC_000014	23851199	23877482	0	0.0076105	0.0197562	0.0206332
MYH7	NC_000014	23881947	23904870	0.0145765	0.0074931	0.0129677	0.020315
LOC100288922	NC_000014	23884659	23886804	0	0	0.0892638	0
NGDN	NC_000014	23938898	23947402	2.2140167	3.7273461	1.2802729	1.9285188
ZFHX2	NC_000014	23990061	24004534	0.080936	0.1076844	0.0381192	0.0530818
THTPA	NC_000014	24025198	24028787	0.4394823	0.1232275	0.2843459	0.3758508
AP1G2	NC_000014	24028777	24037279	2.9645191	1.3196821	0.1223498	0.2023201
JPH4	NC_000014	24037247	24047994	0.0100201	0.0206035	0.0356567	0.0349121
DHRS2	NC_000014	24105573	24114848	1.4328114	2.1158795	0.1622306	0.1815345
LOC100288948	NC_000014	24218313	24220359	0	0	0	0
BRD7P	NC_000014	24218406	24220538	0	0	0	0
LOC728667	NC_000014	24296480	24297315	0	0	0	0
C14orf165	NC_000014	24391456	24403777	0	0	0	0
C14orf167	NC_000014	24407940	24424298	0	0	0	0
DHRS4	NC_000014	24422965	24438488	2.028909	3.8890252	0.5200772	0.9824883
DHRS4L2	NC_000014	24458027	24475617	0.6397123	0.9865372	0.6544716	1.1144424
DHRS4L1	NC_000014	24505710	24520580	0	0.108527	0.0313031	0.0735586
LRRC16B	NC_000014	24521206	24538937	0.0764317	0.0491124	0.0679958	0.239673
CPNE6	NC_000014	24540756	24547296	0	0.0224793	0	0.1218901
NRL	NC_000014	24549316	24553832	0.289426	0.3662282	0.0396125	0.0775706
PCK2	NC_000014	24563483	24573339	2.6847752	2.5492581	1.1561926	1.2273784
WDR23	NC_000014	24584021	24594451	1.4309684	1.037239	0.5861429	2.6040831
LOC100288988	NC_000014	24600484	24602042	0.0739247	0.0380012	0	0
FITM1	NC_000014	24600675	24602058	0	0	0	0.0305333
PSME1	NC_000014	24605378	24608176	34.294646	22.437228	20.918868	18.087001
FAM158A	NC_000014	24608174	24610797	4.1053438	4.2748368	1.5919967	1.687119
PSME2	NC_000014	24612574	24615855	80.686621	78.594048	41.691493	38.23492
RNF31	NC_000014	24616659	24629870	8.6388678	8.4750644	3.4743619	5.4515039
IRF9	NC_000014	24630422	24635774	51.098331	41.979096	16.333369	9.8919452
REC8	NC_000014	24641234	24649463	1.5460124	0.833499	0.3857761	0.4204187
IPO4	NC_000014	24649425	24658124	4.1506663	5.7734347	1.6290652	6.380183
TM9SF1	NC_000014	24658353	24664882	10.595651	9.6740281	6.0496413	10.31482
TSSK4	NC_000014	24674962	24677454	0	0.1078363	0.0311039	0.1949077
CHMP4A	NC_000014	24678787	24683185	4.8474462	5.6441725	1.4028362	3.3168473
MDP-1	NC_000014	24683158	24685241	2.3807973	4.2338766	1.6600723	2.8248424
NEDD8	NC_000014	24686057	24701576	43.666963	53.352561	34.405858	60.906758

GMPR2	NC_000014	24701648	24708448	6.3583853	7.9875671	3.662173	9.2655744
TINF2	NC_000014	24708849	24711880	12.121564	11.508393	4.7548669	7.3645875
TGM1	NC_000014	24718320	24732416	0.1899095	0.2765999	0.0985535	0.1985048
RABGGTA	NC_000014	24734744	24740803	10.137418	6.7884917	2.2805474	5.1830881
DHRS1	NC_000014	24759804	24769039	2.9553594	1.9383025	1.7678899	2.3789761
C14orf21	NC_000014	24769098	24774374	4.4442032	5.2754436	2.8267903	6.5378954
CIDEB	NC_000014	24774393	24780576	3.2343703	3.7887955	1.2904638	4.3574202
LTB4R2	NC_000014	24778161	24780958	0.015707	0	0	0
LTB4R	NC_000014	24780705	24787061	0.3069149	0.1832172	0.1849626	0.1931734
ADCY4	NC_000014	24787570	24803864	2.6433019	0.2745043	0.0950123	0.0651197
LOC100289021	NC_000014	24792143	24795721	0	0	0.0438805	0
RIPK3	NC_000014	24805227	24809242	6.5659253	0.6821024	1.282221	1.2275459
NFATC4	NC_000014	24836145	24848810	9.4616918	7.4396547	2.2354577	3.394206
KIAA1305	NC_000014	24867992	24888494	1.8850139	1.0983874	0.1293798	0.0662623
CBLN3	NC_000014	24895738	24898731	1.6297938	1.5022614	0.5333001	1.4620573
KIAA0323	NC_000014	24899141	24910548	2.0941025	2.160134	0.6023608	2.3299423
SDR39U1	NC_000014	24908972	24912007	5.2123817	4.881716	2.3820613	5.1746342
CMA1	NC_000014	24974712	24977471	0	0.0576319	0	0.0390623
CTSG	NC_000014	25042724	25045466	0	0	0	0
GZMH	NC_000014	25075693	25078864	0.0487231	0	0	0
GZMB	NC_000014	25100160	25103432	0	0	0.0843529	0.0330365
STXBP6	NC_000014	25281293	25519095	0.2230875	0.4128433	0.0992324	0.0932737
HMGN2L6	NC_000014	25735459	25736652	0	0	0	0
OR7K1P	NC_000014	25826870	25828073	0	0	0	0
LOC401767	NC_000014	25894117	25901442	0	0	0	0
LOC100289051	NC_000014	26677104	26677711	0	0	0	0
NOVA1	NC_000014	26915089	27066960	0.0204839	0.0315894	0	0.014274
UNGP2	NC_000014	27394640	27395266	0	0	0	0
RPS27AP4	NC_000014	27727793	27728499	0	0	0	0
LOC728755	NC_000014	27791032	28142421	0	0	0	0
BNIP3P	NC_000014	28733635	28735166	0	0	0	0
RPL26P3	NC_000014	28959010	28959697	0	0	0	0
BTF3P2	NC_000014	29142198	29143047	0	0	0	0
FOXG1	NC_000014	29236287	29238871	0	0.1573117	0.0151248	0.0118471
C14orf23	NC_000014	29241910	29264000	0	0	0	0
LOC100128215	NC_000014	29485135	29490614	0	0	0	0
PRKD1	NC_000014	30045685	30396899	4.8977372	2.7141973	0.9351959	2.1892751
RPS6P24	NC_000014	30524367	30525194	0	0	0	0
SYF2P	NC_000014	31010956	31011394	0	0	0	0
G2E3	NC_000014	31028329	31089046	1.7481647	2.776905	1.6322761	2.9302428
SCFD1	NC_000014	31091521	31205018	16.266553	14.774721	11.406479	17.286863
UBE2CP1	NC_000014	31152068	31152856	0	0	0	0
RPL12P5	NC_000014	31260304	31260695	0	0	0	0
RPL27P1	NC_000014	31290597	31291091	0	0	0	0
COCH	NC_000014	31343741	31359822	1.433426	0.2978781	4.7888413	6.195109
STRN3	NC_000014	31363005	31495607	2.5606975	2.8360023	1.9817335	2.6620868
LOC100289177	NC_000014	31405753	31433830	0	0	0	0
AP4S1	NC_000014	31494683	31562638	0.437959	0.4502679	0.1016401	0.2255732
HECTD1	NC_000014	31569324	31676689	3.1707778	3.5616432	1.9390406	5.7702447
RPL21P5	NC_000014	31580759	31581489	0	0	0	0
NARSP	NC_000014	31755124	31758779	0	0	0	0
HEATR5A	NC_000014	31760994	31858181	0.519225	0.76448	0.5303491	1.4651342
ATP5GP4	NC_000014	31789327	31789894	0	0	0	0
C14orf126	NC_000014	31915242	31926680	1.3441831	1.9718232	0.8895753	1.4735581
RPL9P4	NC_000014	31931589	31932610	0	0	0	0
GPR33	NC_000014	31952220	31953210	0	0	0	0
NUBPL	NC_000014	32030591	32330430	0.2729704	0.4283487	0.4345594	0.7108095
LOC100288600	NC_000014	32544625	32545999	0.1917741	0.1314426	0.1706076	0.1336358
C14orf128	NC_000014	32544625	32545905	0	0	0	0
ARHGAP5	NC_000014	32546495	32628934	3.2444082	4.6434837	3.9447669	4.8150319
LOC644295	NC_000014	32740751	32741509	0	0	0	0
AKAP6	NC_000014	32798479	33302268	0.1184703	0.0652499	0.1505635	0.1061419
MTCO1P2	NC_000014	32953299	32954328	0	0	0	0
NPAS3	NC_000014	33408459	34273382	0.0820208	0.084326	0.0265338	0.0571554
EGLN3	NC_000014	34393421	34420284	0.2906202	0.1825927	0.157999	0.0787561
LOC100128921	NC_000014	34673037	34697555	0	0	0	0

C14orf147	NC_000014	34902144	34931468	2.4280791	2.3358428	4.3510314	3.1664239
RPL23AP71	NC_000014	34955184	34955709	0	0	0	0
EAPP	NC_000014	34985135	35008943	2.7712164	3.4189221	3.0744377	3.9757817
RNU1G1	NC_000014	35015920	35016082	0	0	0	0
RNU1F1	NC_000014	35025433	35025595	0	0	0	0
SNX6	NC_000014	35030618	35099366	9.3091622	8.343772	6.058249	5.5868388
RPS19P3	NC_000014	35037858	35038375	0	0	0	0
RPL23AP8	NC_000014	35163361	35163899	0	0	0	0
CFL2	NC_000014	35179592	35183906	26.730833	27.327079	20.109758	38.524683
RPL12P6	NC_000014	35209496	35210110	0	0	0	0
BAZ1A	NC_000014	35221937	35344853	0.7889643	0.8411804	0.5719059	1.1708315
RNU7-41P	NC_000014	35314406	35314665	0	0	0	0
C14orf19	NC_000014	35409128	35409702	0	0	0	0
SRP54	NC_000014	35452231	35498768	13.211055	15.432581	12.098595	15.98935
FAM177A1	NC_000014	35514113	35552589	2.0422771	2.3957836	1.1879511	1.7880477
PPP2R3C	NC_000014	35554678	35591519	7.4764713	5.0012162	3.8799111	5.9613313
KIAA0391	NC_000014	35591776	35743271	7.0862526	7.718038	5.1361413	9.5710075
RPL23AP70	NC_000014	35613227	35613686	0	0	0	0
CDC10P	NC_000014	35626803	35628306	0	0	0	0
MRP63P8	NC_000014	35688226	35688500	0	0	0	0
DPRXP3	NC_000014	35691333	35691971	0	0	0	0
RPL7AP3	NC_000014	35704321	35704859	0	0	0	0
RPL9P3	NC_000014	35731639	35732338	0	0	0	0
PSMA6	NC_000014	35761574	35786680	38.897207	47.269473	49.006379	51.382409
RPLP0P3	NC_000014	35857261	35858352	0	0	0	0
LOC100289251	NC_000014	35868405	35874407	0.4622075	0.7523967	0.3769266	0.69785
NFKBIA	NC_000014	35870716	35873960	20.109051	12.97545	21.482453	15.830346
DNAJC8P1	NC_000014	35898956	35900529	0	0	0	0
RPS3AP4	NC_000014	35937453	35938606	0	0	0	0
RPS3AP45	NC_000014	35937453	35938603	0	0	0	0
KRT18P6	NC_000014	35980971	35982341	0	0	0	0
INSM2	NC_000014	36003248	36006260	0	0.0149962	0	0.0304927
GARNL1	NC_000014	36007558	36278432	0.7153324	0.7354369	0.7855309	1.666766
NUTF2P2	NC_000014	36288964	36289815	0	0	0	0
BRMS1L	NC_000014	36295597	36341169	1.3266107	2.0808146	1.0288833	1.7659082
LOC644584	NC_000014	36350209	36351249	0	0	0	0
LOC100289323	NC_000014	36416997	36417887	0	0	0	0
LOC644616	NC_000014	36523400	36524543	0	0	0	0
MBIP	NC_000014	36767764	36789882	3.4401225	4.2770696	4.91092	3.6422792
STELLAR	NC_000014	36840370	36841598	0	0	0	0
SFTA3	NC_000014	36942493	36982990	0.029555	0.0303856	0	0.0205951
NKX2-1	NC_000014	36985602	36989416	0.0176004	0.0542852	0.0313156	0
PHKBP2	NC_000014	37003032	37003558	0	0	0	0
RPL29P3	NC_000014	37046599	37047240	0	0	0	0
NKX2-8	NC_000014	37049216	37051786	0	0	0.0216847	0.067942
PAX9	NC_000014	37126782	37147012	0.014186	0.0145847	0.0126203	0.0197707
SLC25A21	NC_000014	37149078	37641865	0.1542043	0.2491315	0.1959778	0.0921049
LOC729069	NC_000014	37257849	37276368	0	0	0	0
LOC100129794	NC_000014	37641231	37643088	0.1182676	0.0972732	0.1472998	0.3955851
MIPOL1	NC_000014	37667118	38016868	0.4527155	0.3759316	0.5266709	0.8857433
FOXA1	NC_000014	38059189	38064489	0.2259549	0.3629772	0.1884523	0.4133178
C14orf25	NC_000014	38080458	38311920	0.0325965	0.0837816	0	0.0283932
SSTR1	NC_000014	38677204	38682268	3.6768621	2.8219883	0.4100187	4.7318327
CLEC14A	NC_000014	38723319	38725574	0.194806	0.0200281	0	0.0135749
KRT8P1	NC_000014	39196168	39197965	0	0	0	0
LOC283547	NC_000014	39307103	39417441	0	0	0	0
SEC23A	NC_000014	39501123	39572437	36.705527	48.171318	19.879601	22.994571
LOC100289389	NC_000014	39511996	39560305	0	0	0	0
PPIAP4	NC_000014	39577399	39578132	0	0	0	0
SIP1	NC_000014	39583488	39606177	1.4552395	3.4909913	1.8124699	1.3971615
TRAPPC6B	NC_000014	39617015	39639634	3.5279243	3.7349455	2.3101516	2.3395908
RPL7AP2	NC_000014	39626326	39627120	0	0	0	0
PNN	NC_000014	39644387	39652422	0.1706254	0.213011	0.1951626	0.4840871
LOC100130635	NC_000014	39680323	39681459	0	0	0	0
YTHDF2P	NC_000014	39684268	39686363	0	0	0	0
MIA2	NC_000014	39703125	39722575	0.0192671	0.0594258	0.0857027	0.0805565

CTAGE5	NC_000014	39734476	39820397	1.6237031	1.869658	1.8200591	1.433184
LOC100288846	NC_000014	39734896	39736265	0	0	0	0.0256275
LOC729153	NC_000014	39839079	39844043	0	0	0	0
COILP1	NC_000014	39853724	39856332	0	0	0	0
FBXO33	NC_000014	39866877	39901704	3.7337157	4.2025612	2.5191469	4.1374219
LOC100289486	NC_000014	41657037	41767230	0	0	0	0
LRFN5	NC_000014	42076764	42373752	0.0117761	0.024214	0.0209526	0.008206
OR10V7P	NC_000014	42575081	42575380	0	0	0	0
YWHAQP	NC_000014	43276221	43277325	0	0	0	0
TUBBP3	NC_000014	43744070	43745151	0	0	0	0
HNRPUP	NC_000014	43769362	43770212	0	0	0	0
LOC390472	NC_000014	44009976	44010854	0	0	0	0
ARHGAP16P	NC_000014	44064658	44065438	0	0	0	0
EIF4BP	NC_000014	44546640	44547183	0	0	0	0
LOC645086	NC_000014	44588621	44590072	0	0	0	0
YWHAZP1	NC_000014	44759862	44761034	0	0	0	0
FSCB	NC_000014	44973352	44976499	0.0279214	0.0861183	0.0124198	0.0389134
LOC100132537	NC_000014	45329519	45335467	0	0	0	0
LOC401770	NC_000014	45356042	45357006	0	0	0	0
C14orf28	NC_000014	45366507	45376460	0.3478353	0.4664494	0.3767143	0.2634625
KLHL28	NC_000014	45393527	45431179	0.6641543	0.6207459	0.6565001	0.4254105
FAM179B	NC_000014	45431416	45543634	1.5419197	1.8747999	0.977126	1.3295966
PRPF39	NC_000014	45553302	45584804	2.942141	2.7097438	2.8218954	3.2568298
SNORD127	NC_000014	45580078	45580178	0	0	0	0
FKBP3	NC_000014	45584802	45603732	2.0554852	2.1132549	2.0027706	2.8419523
FANCM	NC_000014	45605136	45670093	0.5549019	1.4579381	0.6746634	0.8764696
C14orf106	NC_000014	45672393	45722605	2.1619917	3.2442761	3.6175684	4.9171617
LOC644589	NC_000014	45759239	45759686	0	0	0	0
LOC100289583	NC_000014	47104466	47120990	0.3333621	0.1713656	0.2471401	0.3871666
RPL10L	NC_000014	47120216	47121028	0.2162275	0.1111523	0.0480905	0.075338
MDGA2	NC_000014	47308826	48143988	0.031887	0.0081958	0.0283675	0.0111101
RPA2P	NC_000014	47465582	47467008	0	0	0	0
RPL13AP2	NC_000014	47669752	47670324	0	0	0	0
RPS15AP3	NC_000014	47913274	47913727	0	0	0	0
RPL18P1	NC_000014	48958999	48959769	0	0	0	0
ATP5GP2	NC_000014	49840630	49841252	0	0	0	0
RPS29	NC_000014	50044039	50053094	55.982307	58.53676	57.585196	147.1398
RPL32P29	NC_000014	50044073	50044560	0	0	0	0
RN7SL1	NC_000014	50053298	50053596	0	0	0	0
LOC100288910	NC_000014	50064193	50065882	0.1300244	0.1604144	0.069404	0.0543637
PPIL5	NC_000014	50065415	50081390	4.5585272	5.9554054	4.234636	5.001771
RHOQP	NC_000014	50066691	50067331	0	0	0	0
RPL36AL	NC_000014	50085406	50087349	7.8148863	7.3433827	4.7096494	3.2205895
RPL36AP42	NC_000014	50085460	50085837	0	0	0	0
MGAT2	NC_000014	50087489	50090199	10.456145	10.666683	5.5379805	5.6482623
C14orf104	NC_000014	50091892	50101948	0.8751082	1.1436906	0.7125442	0.6304818
LOC100288937	NC_000014	50091892	50101082	0.1342614	0.1610406	0.2189782	0.1091518
POLE2	NC_000014	50110278	50154935	2.5744822	2.9409315	2.6373506	3.6967309
STK16P	NC_000014	50143386	50145229	0	0	0	0
KLHDC1	NC_000014	50159885	50219868	0.5018831	0.1719962	0.1785957	0.1282351
KLHDC2	NC_000014	50234787	50249856	8.0304078	9.6571391	6.7976941	6.1724561
SDCCAG1	NC_000014	50250532	50319539	0.5865452	0.8223138	0.5977061	1.3376554
RN7SL3	NC_000014	50320334	50320632	0	0	0	0
RN7SL2	NC_000014	50329269	50329567	0	0	0	0
ARF6	NC_000014	50359736	50363772	18.64369	16.437628	8.0497907	11.164591
C14orf182	NC_000014	50448430	50474238	0.0327239	0.1009309	0.0291121	0.102615
PDLIM1P	NC_000014	50517007	50518406	0	0	0	0
RPS15AP2	NC_000014	50519853	50520414	0	0	0	0
LOC196913	NC_000014	50550369	50559361	0	0.046342	0	0.1256405
C14orf138	NC_000014	50575350	50583297	0.936117	1.1650429	0.9204584	0.4978259
SOS2	NC_000014	50583846	50698099	2.3491997	2.381207	2.6639034	2.749495
L2HGDH	NC_000014	50709152	50778947	0.5480009	0.4299651	0.5709078	0.6180246
ATP5S	NC_000014	50779047	50792668	0.8447071	1.3026715	0.7723491	1.0791468
CDKL1	NC_000014	50796720	50862617	3.3605275	1.2668244	1.1294114	1.7172829
MAP4K5	NC_000014	50885243	50999376	4.4716802	3.3453984	2.5751292	4.8688201
ATL1	NC_000014	50999800	51099786	3.3264601	2.018079	2.0928462	1.848143

SNRPGP	NC_000014	51038493	51038884	0	0	0	0
SAV1	NC_000014	51100360	51135023	4.8718594	3.48826	3.0442182	3.1726202
ZNF405P	NC_000014	51170613	51172330	0	0	0	0
NIN	NC_000014	51186481	51297839	0.2429997	0.3886233	0.2127479	0.9864253
ABHD12B	NC_000014	51338878	51371688	0	0.1143884	0.0742359	0.0969142
PYGL	NC_000014	51371937	51411234	23.853175	25.385728	25.365064	6.6444082
MRP63P9	NC_000014	51384375	51384569	0	0	0	0
TRIM9	NC_000014	51441979	51562422	0.1907751	0.0326895	0.2262918	0.1949782
TMX1	NC_000014	51706886	51724373	7.1747844	6.6938373	5.5445378	5.4847187
SETP2	NC_000014	51902698	51906035	0	0	0	0
FRMD6	NC_000014	51955855	52197445	36.738174	82.639787	7.5324199	9.8946467
OR7E105P	NC_000014	52223608	52224803	0	0	0	0
OR7E106P	NC_000014	52229812	52230758	0	0	0	0
OR7E159P	NC_000014	52238506	52238814	0	0	0	0
GNG2	NC_000014	52327050	52436518	0.751836	1.9441277	0.2938905	0.1190703
C14orf166	NC_000014	52456228	52471420	23.124046	36.456256	28.607068	24.068648
NID2	NC_000014	52471520	52535946	4.3634689	3.7697606	3.2000187	1.4444552
COX5AP2	NC_000014	52584319	52584895	0	0	0	0
PTGDR	NC_000014	52734431	52743442	0.0448146	0.0614322	0.0398684	0
LOC100131689	NC_000014	52754705	52755211	0	0	0	0
PTGER2	NC_000014	52781016	52795324	5.5289711	1.9494452	3.2791507	2.2845171
TXNDC16	NC_000014	52897309	53019213	1.0233991	1.3003132	2.1644522	2.6238362
GPR137C	NC_000014	53019866	53104431	0.587785	0.8251084	0.5128539	0.7404163
ERO1L	NC_000014	53108605	53162419	16.304661	14.073742	16.902905	12.277708
PSMC6	NC_000014	53173896	53194716	13.242405	21.187697	11.609007	19.186207
STYX	NC_000014	53196883	53241707	1.8518782	2.0246622	2.6359715	2.3354224
GNPNAT1	NC_000014	53241911	53258386	4.2627995	6.6946894	2.6575993	4.1165824
LOC100288978	NC_000014	53265619	53265788	0	0	0	0
FERMT2	NC_000014	53323989	53417815	36.080719	36.47762	11.703136	14.21329
LOC100289655	NC_000014	53338077	53338961	0	0	0	0
NDUFB3P3	NC_000014	53478647	53479116	0	0	0	0
DDHD1	NC_000014	53512741	53620000	1.5359585	2.5597136	1.1460391	1.3120015
RPS3AP46	NC_000014	54079516	54080099	0	0	0	0
BMP4	NC_000014	54416454	54423554	3.6235042	1.6992793	0.2073641	0.634942
LOC100286880	NC_000014	54416963	54418960	0.1227604	0.3155265	0.2184222	0.0427722
ATP5C2	NC_000014	54457649	54458729	0	0	0	0
CDKN3	NC_000014	54863673	54886936	11.417682	18.923396	20.227408	22.497111
CNIH	NC_000014	54893647	54908148	14.114084	19.686373	17.144681	13.321714
GMFB	NC_000014	54941209	54955744	13.498766	16.605585	13.165217	14.388125
CGRRF1	NC_000014	54976587	55005334	2.1592102	4.1665722	2.0391021	2.060177
LOC645602	NC_000014	55029399	55034628	1.1388578	0.8406214	0.4676121	1.1191816
LOC644925	NC_000014	55032836	55034393	0	0	0	0
SAMD4A	NC_000014	55034637	55255845	5.0000982	2.6862673	2.4749528	3.1567988
GCH1	NC_000014	55308723	55369542	2.9288808	1.5596458	1.2426772	0.3453934
FDPSL3	NC_000014	55351474	55352705	0	0	0	0
LOC100289044	NC_000014	55369024	55370518	0	0.030223	0	0
WDHD1	NC_000014	55406942	55493807	2.7200743	3.8464113	1.9078026	2.6005917
RPSAP13	NC_000014	55471473	55472481	0	0	0	0
SOCS4	NC_000014	55493844	55516207	1.7797373	2.0037128	0.9867773	1.1877894
MAPK1IP1L	NC_000014	55518362	55536912	20.632367	17.985355	20.192451	34.700936
LGALS3	NC_000014	55595872	55612148	153.30202	129.00435	106.99418	45.548384
DLGAP5	NC_000014	55614848	55658396	3.7471806	7.0527969	4.4622935	6.9802928
UBE2L1	NC_000014	55695236	55695941	0	0	0	0
UBE2L7P	NC_000014	55695911	55696654	0	0	0	0
COX5AP1	NC_000014	55707157	55707875	0	0	0	0
FBXO34	NC_000014	55738021	55820329	4.3165626	4.2493016	4.7212976	4.7121752
RPL34P28	NC_000014	55749014	55749361	0	0	0	0
KIAA0831	NC_000014	55833110	55878576	1.0472691	1.0100043	0.9234347	0.6974877
HMGN1L1	NC_000014	55861033	55862045	0	0	0	0
RPL21P6	NC_000014	55880258	55880820	0	0	0	0
TBPL2	NC_000014	55880928	55907263	0	0	0	0.0472242
RPL7AP4	NC_000014	56010094	56010949	0	0	0	0
ABI1P	NC_000014	56013277	56017081	0	0	0	0
C14orf33	NC_000014	56042875	56046810	0	0	0	0
KTN1	NC_000014	56046925	56151302	1.0197636	1.609423	0.9151679	4.0205672
RPL13AP3	NC_000014	56232963	56234435	0	0	0	0

C14orf34	NC_000014	56247853	56263392	0	0	0	0
LOC100286940	NC_000014	56584642	56645185	0.3086252	0	0.3294739	0.3440998
PELI2	NC_000014	56585093	56768031	0.4783691	0.3886915	0.9060531	0.962404
C14orf101	NC_000014	57046511	57116233	1.4326464	2.1087	1.0727991	1.2281553
RPL36AP1	NC_000014	57150095	57150495	0	0	0	0
OTX2	NC_000014	57267425	57277184	0.0195499	0.0602981	0.0173922	0
RPL3P3	NC_000014	57445066	57446394	0	0	0	0
LOC440180	NC_000014	57507406	57509222	0	0	0	0
EXOC5	NC_000014	57669194	57735617	5.3186087	7.2465449	7.2673421	8.5350971
MUDENG	NC_000014	57735606	57756797	1.71817	1.343093	2.0338314	2.3352088
NAT12	NC_000014	57857271	57879466	2.2096876	2.7766336	2.4375912	2.5184257
C14orf105	NC_000014	57936595	57960576	0.0349906	0.017987	0.0311286	0.0487657
SLC35F4	NC_000014	58030640	58063615	0.0494078	0.0761946	0	0.0344293
LOC401777	NC_000014	58096559	58097880	0	0	0	0
C14orf37	NC_000014	58470808	58618847	0.4135298	1.5979853	0.5201169	0.7949352
UBA52P3	NC_000014	58607855	58608575	0	0	0	0
ACTR10	NC_000014	58666833	58702354	8.7644508	10.453981	8.7417161	10.521815
PSMA3	NC_000014	58711593	58738726	17.067274	18.765491	24.673221	25.867616
HMGB1L14	NC_000014	58751427	58752551	0	0	0	0
ARID4A	NC_000014	58765222	58840451	0.5405968	0.6027585	0.359004	0.7852533
TOMM20L	NC_000014	58862644	58875419	0.1440926	0.0740712	0.2563775	0.0502047
TIMM9	NC_000014	58875370	58894232	6.5229287	10.688096	12.585209	10.198823
KIAA0586	NC_000014	58894710	59015549	1.4148957	2.0365261	1.1468433	2.5580072
HSBP1P1	NC_000014	58930506	58931226	0	0	0	0
HNRPCP	NC_000014	58987652	58989464	0	0	0	0
RPL9P5	NC_000014	59030776	59031456	0	0	0	0
DACT1	NC_000014	59104757	59115039	0.2162135	2.374995	0.1316075	0.2458237
RPL31P4	NC_000014	59261348	59261789	0	0	0	0
LOC440181	NC_000014	59294247	59295073	0	0	0	0
AKR1B1P5	NC_000014	59602004	59603171	0	0	0	0
PPIAP5	NC_000014	59648288	59648862	0	0	0	0
DAAM1	NC_000014	59655399	59836471	1.3733822	2.5479362	1.4055281	1.7341625
GPR135	NC_000014	59930240	59932059	0.1448843	0.3972167	0.1503753	0.6225937
C14orf149	NC_000014	59939406	59951073	3.4051128	5.0385546	3.3123933	5.9431335
C14orf100	NC_000014	59951161	59972081	5.3500585	4.662988	7.4440184	8.1528741
C14orf38	NC_000014	59977148	60043493	0	0	0.0157208	0.788095
RTN1	NC_000014	60062694	60337557	0.1421123	0.0974043	0.2949965	0.1402918
LOC100286978	NC_000014	60194140	60212818	0.204094	0.1398867	0.0605225	0.3318485
LRRC9	NC_000014	60386867	60530282	0	0	0	0
C14orf135	NC_000014	60558629	60601532	6.572116	6.9751589	8.3723678	10.249832
DHRS7	NC_000014	60611496	60632211	10.987058	10.192233	19.689219	9.1302618
PSMA3P	NC_000014	60646042	60646799	0	0	0	0
RPL17P2	NC_000014	60679281	60679748	0	0	0	0
PPM1A	NC_000014	60712470	60765805	2.2522747	2.8459804	4.1649268	3.772105
RBM8B	NC_000014	60864446	60867232	0	0	0	0
LOC404718	NC_000014	60865541	60866731	0	0	0	0
C14orf39	NC_000014	60902674	60952764	0.015906	0.016353	0.0141504	0.0110839
LOC390483	NC_000014	60960578	60963603	0	0	0	0
LOC100289241	NC_000014	60975669	60977914	0	0.0396694	0.0343262	0
SIX6	NC_000014	60975938	60978525	0.0297954	0	0.0265068	0
RPL37P5	NC_000014	61038728	61039069	0	0	0	0
RPS15AP4	NC_000014	61068824	61069269	0	0	0	0
LOC100287070	NC_000014	61107448	61110663	0.0392746	0.1615135	0.1048192	0.1094723
SIX1	NC_000014	61111417	61116155	7.0003138	5.4482405	3.6522103	2.5986127
SIX4	NC_000014	61176256	61190792	1.4425486	2.2246373	2.9378219	1.054501
MNAT1	NC_000014	61201470	61435398	6.6306735	8.9576424	9.3469399	10.357102
MAD2L1P	NC_000014	61246569	61247985	0	0	0	0
SRMP2	NC_000014	61385700	61386820	0	0	0	0
TRMT5	NC_000014	61438159	61447782	0.8060084	0.6663463	1.0053449	1.7081375
SLC38A6	NC_000014	61447880	61519176	3.3048143	2.354544	3.3784836	6.3229465
TMEM30B	NC_000014	61744088	61748530	1.2957953	0.0101696	0.5719878	0.0827141
PRKCH	NC_000014	61788515	62017698	0.6142223	0.1159871	0.3568517	0.3581346
HIF1A	NC_000014	62162119	62214977	96.081944	87.127491	105.15598	94.658715
SNAPC1	NC_000014	62229075	62263146	7.1647532	6.821741	7.5368801	6.975923
LOC122867	NC_000014	62298579	62299087	0	0	0	0
MOCS3P	NC_000014	62330934	62332767	0	0	0	0

SYT16	NC_000014	62462541	62568427	0.0560385	0.0288068	0.274194	0.5076486
FLJ43390	NC_000014	62584075	62595132	0	0	0	0
LOC100129782	NC_000014	63070759	63072440	0	0	0	0
KCNH5	NC_000014	63173945	63568584	0	0.5878663	0.0305211	0.031876
PARP1P2	NC_000014	63589719	63590653	0	0	0	0
RHOJ	NC_000014	63671145	63758559	0.9227975	2.3718321	0.0205237	0.0321521
GPHB5	NC_000014	63779642	63784563	0	0	0	0.0781247
PPP2R5E	NC_000014	63841355	64010079	4.9092499	6.8996639	5.0643311	4.1943164
RPL31P5	NC_000014	64034630	64034998	0	0	0	0
GCATP	NC_000014	64060565	64062012	0	0	0	0
WDR89	NC_000014	64063757	64108591	0.7506686	1.3049865	1.2992048	1.7785255
HSPEP2	NC_000014	64068189	64068652	0	0	0	0
SGPP1	NC_000014	64150934	64194756	6.9727476	9.7767651	7.2901777	6.626598
LOC100287149	NC_000014	64165349	64194759	0.3235453	0.221759	0.0959449	0.1878827
EIF2S2P	NC_000014	64201393	64202937	0	0	0	0
RPS28P1	NC_000014	64275153	64276034	0	0	0	0
SYNE2	NC_000014	64319683	64693167	0.2723452	0.3286951	0.7584601	2.1481009
ESR2	NC_000014	64693751	64805268	0.0258215	0.1194626	0.0459431	0.0809706
LOC441687	NC_000014	64812857	64814387	0.100568	0	0	0
MTHFD1	NC_000014	64854759	64926725	9.4575371	12.737316	12.745262	14.957162
AKAP5	NC_000014	64932217	64941221	0.1287215	0.1114436	0.0843789	0.1652337
ZBTB25	NC_000014	64953555	64970554	2.1179871	2.1775133	2.082559	1.9031313
ZBTB1	NC_000014	64971292	65000408	2.806856	2.6465931	2.6970974	3.495818
HSPA2	NC_000014	65007186	65009955	0.5870341	0.163117	0.3387515	0.1990064
LOC100287214	NC_000014	65007459	65011893	1.4745962	0.8121642	1.3821189	1.2660974
C14orf50	NC_000014	65016620	65056096	0.2189592	0.3537491	0.0278274	0.0871883
NUP50P1	NC_000014	65063610	65068669	0	0	0	0
PLEKHG3	NC_000014	65171193	65211060	0.7191529	0.3286066	0.7108648	0.9605075
SPTB	NC_000014	65213001	65289866	0.0551474	0.026168	0.0301912	0.0739017
CHURC1	NC_000014	65381140	65401913	2.820694	2.3787771	3.3072771	3.478247
GPX2	NC_000014	65405872	65409531	0	0.0454561	0.1180007	0.0308097
LOC100289314	NC_000014	65410249	65410640	0.3363385	0.1152638	0.6981708	0.6249975
RAB15	NC_000014	65412532	65438875	1.4385885	2.2651661	0.2421259	0.3070616
FNTB	NC_000014	65453507	65529370	1.917571	2.2279152	1.5949699	2.9658006
MAX	NC_000014	65472892	65569227	6.7143132	5.6479252	9.7065137	12.717831
RPL21P7	NC_000014	65733321	65734450	0	0	0	0
RPL36AP2	NC_000014	65735430	65736396	0	0	0	0
PTBP1P	NC_000014	65746368	65747019	0	0	0	0
LOC645431	NC_000014	65877310	65879335	0	0	0	0
FUT8	NC_000014	65877840	66209962	2.1566519	6.3229179	2.7815361	3.8469852
RPL21P8	NC_000014	65934492	65935102	0	0	0	0
NCOA4P	NC_000014	66344751	66347785	0	0	0	0
FTHL13	NC_000014	66367492	66368168	0	0	0	0
YBX1P1	NC_000014	66479149	66480644	0	0	0	0
C14orf53	NC_000014	66953109	66965271	0	0	0	0
GPHN	NC_000014	66974125	67648525	0.7355353	1.1448142	1.9267048	2.8119077
FAM71D	NC_000014	67656146	67695267	0.6765121	0.5152041	0.6241345	0.5238007
SF3B4P	NC_000014	67665761	67667267	0	0	0	0
MPP5	NC_000014	67708021	67802536	2.0919916	2.4291245	2.7369026	4.8421265
ATP6V1D	NC_000014	67804589	67826591	9.9798561	11.370299	12.860733	17.725364
EIF2S1	NC_000014	67827034	67853233	11.427595	16.977677	14.568889	16.102877
PLEK2	NC_000014	67853700	67878828	2.4984269	8.2320445	3.293836	2.1605222
C14orf83	NC_000014	67936983	67982021	0.5833836	0.1221773	0.1153321	0.0752824
PLEKHH1	NC_000014	68000008	68056255	0.0268797	0.0069088	0.0478258	0.046827
PIGH	NC_000014	68056023	68067017	1.1002339	1.6653131	1.1419317	0.8305774
ARG2	NC_000014	68086579	68118437	4.3482349	3.0335146	6.0195646	5.7817791
VTI1B	NC_000014	68117866	68141602	15.287777	17.41479	10.427976	15.508015
COX7AP1	NC_000014	68118946	68119346	0	0	0	0
RDH11	NC_000014	68143517	68162510	18.363105	19.160718	18.071967	17.172829
RDH12	NC_000014	68168603	68201168	0.0234016	0.0481186	0.1040936	0.0652287
RPL21P9	NC_000014	68181222	68181942	0	0	0	0
ZFYVE26	NC_000014	68213237	68283306	4.4561463	3.1990316	3.8067087	4.3048925
RAD51L1	NC_000014	68286509	69062614	0.2601187	0.4132999	0.3681503	0.4037178
RPL7AP5	NC_000014	68805386	68806266	0	0	0	0
PPIAP6	NC_000014	68888169	68889011	0	0	0	0
RPL12P7	NC_000014	69159788	69160343	0	0	0	0

LOC100289418	NC_000014	69245526	69256963	2.3382372	1.2566121	3.8293867	5.962038
ZFP36L1	NC_000014	69254375	69259785	13.088487	12.155561	25.396599	22.426491
C14orf181	NC_000014	69261464	69263190	0.1272387	0.0261629	0.2943071	0.3369269
MAGOHP	NC_000014	69328440	69329510	0	0	0	0
BLZF2P	NC_000014	69333276	69336928	0	0	0	0
ACTN1	NC_000014	69340840	69446083	88.325396	111.35545	48.657957	78.755457
LOC100036574	NC_000014	69353699	69354839	0	0	0	0
BANF1P1	NC_000014	69403264	69403961	0	0	0	0
RPS29P1	NC_000014	69489759	69490162	0	0	0	0
WDR22	NC_000014	69517637	69619914	1.2262915	1.6986241	1.58742	1.5760171
DDX18P1	NC_000014	69550062	69552423	0	0	0	0
EXD2	NC_000014	69676478	69709072	2.9340218	2.8249601	3.3559549	3.1479476
GALNTL1	NC_000014	69726915	69821183	0.211653	0.1398867	0.443832	0.7163714
ERH	NC_000014	69846840	69865021	27.543087	36.891317	58.377888	47.791633
SLC39A9	NC_000014	69865407	69929107	6.9734558	6.5503802	12.255541	12.604907
FLJ44817	NC_000014	69951409	70019312	0	0.0445376	0.0192694	0.0150936
RPL24P3	NC_000014	70027717	70028230	0	0	0	0
C14orf162	NC_000014	70036531	70037918	0	0	0	0
LOC100130174	NC_000014	70038216	70040390	0	0.0239827	0	0.0162552
KIAA0247	NC_000014	70078310	70181861	3.4144834	3.0412711	3.1318651	3.6229691
LOC100289511	NC_000014	70233000	70234694	0.1296408	0.1599412	0.3229297	0.0722711
SFRS5	NC_000014	70233829	70238722	23.451479	15.955132	19.347493	19.6066
SLC10A1	NC_000014	70242552	70263954	0.1113318	0.1144608	0.0495219	0.1163707
SMOC1	NC_000014	70346143	70499083	1.4134345	0.0123149	5.0936594	9.0647636
RPL7AP6	NC_000014	70351999	70352879	0	0	0	0
SLC8A3	NC_000014	70510934	70655787	0.9467217	1.5766329	0.807427	0.2617045
LOC646548	NC_000014	70696845	70699311	0	0	0	0
LOC100287307	NC_000014	70701212	70701812	0	0	0	0
ADAM21P	NC_000014	70712470	70714518	0	0	0	0
COX16	NC_000014	70791798	70826444	2.5142596	2.5585464	3.7887892	4.7197711
SYNJ2BP	NC_000014	70833213	70883807	1.4192913	1.6319784	1.5672254	2.3250616
ADAM21	NC_000014	70924217	70926622	0.2922576	0.2441331	0.2762505	0.0763713
C14orf55	NC_000014	70935008	70939016	0	0	0	0
ADAM20	NC_000014	70989078	71001732	0.0787039	0.0161832	0.0700171	0.109688
MED6	NC_000014	71050957	71067384	8.5345897	8.5391251	7.5344267	6.8586967
TTC9	NC_000014	71108504	71142077	0.0758164	0.0606256	0.0074943	0.0528319
MAP3K9	NC_000014	71194852	71275888	0.3294941	0.3064922	0.4885451	0.634146
LOC100287335	NC_000014	71325579	71349928	0	0	0	0
PCNX	NC_000014	71374122	71582099	2.8235183	3.1756737	5.4535036	4.5016366
PTTG4P	NC_000014	71552015	71552694	0	0	0	0
C14orf56	NC_000014	71676734	71758137	0	0	0	0
LOC729941	NC_000014	71797053	71803746	0	0	0	0
SNORD56B	NC_000014	71865054	71865124	0	0	0	0
LOC145474	NC_000014	71954578	71956431	0	0	0	0
SIPA1L1	NC_000014	71996042	72206122	3.5629747	4.4228134	2.1999296	3.2271596
LOC100289611	NC_000014	72175948	72190577	0	0.2763511	0	0
RGS6	NC_000014	72399786	73033238	0.0536328	0.0708945	0.0613456	0.0587297
DPF3	NC_000014	73136660	73360809	0.4257424	0.3780205	0.3615363	0.5933486
WDR21A	NC_000014	73393085	73426357	0.420725	0.4145266	0.3742886	0.7329448
RPL36P3	NC_000014	73396277	73396672	0	0	0	0
LOC100287446	NC_000014	73425423	73428241	0.1079809	0	0.0480314	0.0376227
RPS12P1	NC_000014	73430356	73430862	0	0	0	0
ZFYVE1	NC_000014	73436159	73493839	3.5001276	3.588391	4.3820761	4.9191639
RBM25	NC_000014	73525221	73588076	0.9192054	0.9870415	0.7904923	2.1778324
PSEN1	NC_000014	73603143	73690399	5.3401792	5.3863957	6.0155039	6.4769858
LOC100287521	NC_000014	73603208	73604906	0	0.0265941	0	0
PAPLN	NC_000014	73704205	73741348	0.1205299	0.0851933	0.5026255	0.3989528
LOC100287558	NC_000014	73711597	73731006	0	0	0.0276113	0.0216277
NUMB	NC_000014	73741918	73925286	2.2553017	1.6987174	4.1307801	6.2275068
HEATR4	NC_000014	73945192	74025651	0.0380065	0.0260498	0.0563528	0.1500787
C14orf169	NC_000014	73957644	73960105	0.0178506	0.0183523	0.0635216	0.0870732
ACOT1	NC_000014	74003928	74010498	0.2193299	0.1127471	0.0487805	0.0764189
LOC100287586	NC_000014	74004017	74010128	0	0	0	0
NT5CP2	NC_000014	74005679	74006536	0	0	0	0
LOC100287615	NC_000014	74034657	74040193	1.404442	1.3772718	0.8649904	0.8732758
ACOT2	NC_000014	74035772	74042359	9.1527727	6.4438128	4.5801902	3.5703027

NT5CP1	NC_000014	74037602	74038457	0	0	0	0
TRNAK40P	NC_000014	74055529	74055601	0	0	0	0
ACOT4	NC_000014	74058410	74062470	2.7467645	2.3809881	4.0007926	2.3456555
LOC100287645	NC_000014	74058410	74060496	0	0	0	0.0757105
ACOT6	NC_000014	74083548	74086592	0	0.1350102	0.0778836	0.1220115
NDUFB8P1	NC_000014	74098158	74098827	0	0	0	0
DNAL1	NC_000014	74111578	74168661	0.5726154	0.7091265	0.7467554	0.8433858
PNMA1	NC_000014	74178486	74181128	11.007843	15.522712	7.1005795	7.1029324
C14orf43	NC_000014	74181825	74253896	1.8186585	1.5167768	1.6370197	1.4504947
PTGR2	NC_000014	74318671	74352162	1.1428618	0.6943076	2.0180383	1.6169163
ZNF410	NC_000014	74353586	74398803	15.799571	17.695795	21.361847	18.152763
FAM161B	NC_000014	74400705	74416835	1.5641298	1.4119813	1.0351352	0.8506911
COQ6	NC_000014	74416643	74429815	4.750515	5.9639145	3.8863957	3.2438084
ENTPD5	NC_000014	74433172	74486026	2.3288076	2.1965679	1.2164532	2.0843379
RPL41P4	NC_000014	74470056	74470484	0	0	0	0
C14orf45	NC_000014	74486059	74532795	0.4103344	0.6400739	0.667151	0.5028554
ALDH6A1	NC_000014	74526869	74551196	3.0600693	1.9248999	6.4476059	5.3169166
LIN52	NC_000014	74551656	74667117	1.246585	0.9046732	2.7181289	3.4065493
VSX2	NC_000014	74706175	74729441	0	0.0150863	0	0.0204507
ABCD4	NC_000014	74751980	74769767	1.809715	1.302404	0.9907524	1.4259921
LOC100287680	NC_000014	74772126	74773271	0	0	0	0
C14orf115	NC_000014	74815166	74826711	0	0.013227	0	0.0448256
RPS2P2	NC_000014	74844378	74845326	0	0	0	0
TMEM90A	NC_000014	74872596	74892805	0.0337156	0	0	0.0352415
NPC2	NC_000014	74946643	74960084	55.168014	52.134695	52.304436	83.407556
RAP1AP	NC_000014	74949550	74950869	0	0	0	0
ISCA2	NC_000014	74960450	74962271	6.8345507	5.4083808	6.4487031	5.974882
LTBP2	NC_000014	74964886	75079034	3.0826443	13.215907	1.1929465	1.3533089
KIAA0317	NC_000014	75127955	75179807	3.0913081	3.8420413	2.5490608	4.9438509
FCF1	NC_000014	75179850	75203394	5.8013855	7.1460673	5.6155076	8.0725603
YLPM1	NC_000014	75230069	75304013	0.7669471	1.5547932	0.7447613	0.9070414
PROX2	NC_000014	75321835	75330537	0.1235195	0.126991	0	0.051644
LOC100131950	NC_000014	75340387	75341318	0	0	0	0
DLST	NC_000014	75348619	75370446	18.169852	19.547885	25.087489	28.621687
RPS6KL1	NC_000014	75372346	75389145	0.2476367	0.2096678	0.1684681	0.1827139
PGF	NC_000014	75408537	75422291	1.396391	1.4356367	0.7001875	1.3269011
EIF2B2	NC_000014	75469612	75476292	6.3966237	7.4865286	12.041745	12.815089
MLH3	NC_000014	75480467	75518235	0.6066815	0.7152894	0.82196	0.9851468
ACYP1	NC_000014	75519924	75530736	2.4203374	2.2919117	4.1364091	3.8613977
FAM164C	NC_000014	75536299	75544799	0.0846787	0.0870586	0.150665	0.1622705
NEK9	NC_000014	75548818	75593778	3.1793687	3.6273884	3.3786278	4.9116935
TMED10	NC_000014	75598171	75643349	33.512648	35.544858	29.993041	36.374433
LOC730011	NC_000014	75705319	75735981	0	0	0	0
LOC100287415	NC_000014	75745477	75747889	0	0.0815094	0.0235103	0.0368309
FOS	NC_000014	75745531	75748913	1.518365	0.7371572	0.4502599	0.3086
LOC646701	NC_000014	75758809	75761016	0	0	0	0
JDP2	NC_000014	75894509	75939404	0.5244174	0.3911525	0.4390929	0.4370888
BATF	NC_000014	75988784	76013335	0	0	0.041549	0.032545
FLVCR2	NC_000014	76044940	76114512	0.5051521	0.3956948	0.502897	0.7794509
RPS24P2	NC_000014	76062072	76062586	0	0	0	0
C14orf1	NC_000014	76117236	76127532	8.0662659	7.9093088	7.6867193	8.6813829
TTLL5	NC_000014	76127621	76421422	3.0212064	4.0321652	3.8897237	7.1135499
RPS2P43	NC_000014	76304871	76305481	0	0	0	0
TGFB3	NC_000014	76424442	76448092	0.2899506	0.2555141	0.2210984	0.3752341
C14orf179	NC_000014	76452096	76550092	3.8833567	2.8726516	4.1709687	3.696106
C14orf118	NC_000014	76618259	76669134	2.5163202	2.4433172	3.3423366	3.8357203
ESRRB	NC_000014	76837726	76967208	0.0601207	0.0412069	0.0178283	0.0418945
CYCSP1	NC_000014	77100057	77100371	0	0	0	0
RPSAP3	NC_000014	77101043	77102088	0	0	0	0
LOC100130048	NC_000014	77156480	77158357	0	0	0	0
VASH1	NC_000014	77228235	77249363	0.5320433	0.809255	0.6483842	1.0005143
ANGEL1	NC_000014	77253586	77279283	0.8766127	0.9232318	0.960558	1.5345962
C14orf166B	NC_000014	77292725	77336645	0	0	0.0450952	0.1236298
RPL22P2	NC_000014	77352663	77353118	0	0	0	0
RPLP1P1	NC_000014	77434898	77435526	0	0	0	0
C14orf4	NC_000014	77490886	77495034	10.56071	6.9915025	9.4799111	5.5285693

KIAA1737	NC_000014	77564578	77583630	0.8187228	0.7066401	1.5466378	1.9299026
ZDHHC22	NC_000014	77597613	77608134	0.0257912	0.013258	0	0.0359447
TMEM63C	NC_000014	77648102	77725838	0.0244066	0	0.0072376	0.0396842
NGB	NC_000014	77731834	77737655	0.0937063	0.024085	0.0416818	0.0326491
POMT2	NC_000014	77741299	77787225	0.5951135	1.0104618	0.8984258	1.8598613
GSTZ1	NC_000014	77787230	77797940	0.5869973	0.4198225	0.8173707	1.2982672
TMED8	NC_000014	77808114	77843396	2.3816526	3.9731826	3.7578437	6.5758941
C14orf174	NC_000014	77843762	77857587	0.2170283	0.1338767	0.0386149	0.0302468
C14orf148	NC_000014	77860424	77889379	0.0713445	0.1955991	0.1692535	0.1988628
C14orf133	NC_000014	77893018	77923904	3.215336	4.3857848	3.2852717	3.7490796
AHSA1	NC_000014	77924373	77935815	12.776379	20.429352	22.257686	31.652999
ISM2	NC_000014	77940737	77965210	0	0.0755323	0.0522869	0.010239
SPTLC2	NC_000014	77973270	78083110	4.3194903	4.4346441	2.9509636	3.3444365
ALKBH1	NC_000014	78138749	78174356	1.2750938	1.9052189	1.7847245	2.2154167
RPL21P10	NC_000014	78149537	78150370	0	0	0	0
C14orf156	NC_000014	78174425	78183941	24.022081	39.948046	26.294836	28.464937
SNW1	NC_000014	78183942	78227497	2.2521661	3.4200883	2.6837069	3.3259741
C14orf178	NC_000014	78226893	78236085	0.1479738	0	0.0438805	0.0343714
ADCK1	NC_000014	78266426	78400297	1.2000189	1.0719428	0.5425356	1.3982711
FRDAP	NC_000014	78525843	78526727	0	0	0	0
RPS26P48	NC_000014	78697620	78697962	0	0	0	0
TRNASUP6P	NC_000014	78700838	78700910	0	0	0	0
NRXN3	NC_000014	78870093	80330762	0.0824876	0.0484605	0.0366916	0.0739037
LOC730007	NC_000014	80359423	80440512	0	0	0	0
DIO2	NC_000014	80663869	80678525	0.1162823	0.0797003	0.2126429	0.9273445
C14orf145	NC_000014	80962821	81405884	0.4240013	0.7299091	0.6403685	1.3673661
HMGN2L2	NC_000014	81288773	81289950	0	0	0	0
TSHR	NC_000014	81421869	81612646	0.0091597	0.0094171	0.0081487	0.0319142
RPL17P3	NC_000014	81467478	81468161	0	0	0	0
NMNATP	NC_000014	81498718	81499984	0	0	0	0
GTF2A1	NC_000014	81646394	81687575	6.2300241	6.9016405	7.8195131	13.32687
SNORA79	NC_000014	81669039	81669178	0	0	0	0
LOC246720	NC_000014	81712354	81713015	0	0	0	0
UNGP3	NC_000014	81724683	81726575	0	0	0	0
STON2	NC_000014	81736909	81864927	1.3700888	0.1393116	1.1786865	1.4898022
RPS24P3	NC_000014	81799181	81799737	0	0	0	0
LOC731308	NC_000014	81879314	81879583	0	0	0	0
SEL1L	NC_000014	81939239	82000205	9.0448253	10.940441	11.457685	12.461286
EEF1A1P2	NC_000014	82127676	82129733	0	0	0	0
RPL9P6	NC_000014	82243264	82243977	0	0	0	0
EIF3LP1	NC_000014	82382563	82384410	0	0	0	0
ENSAP2	NC_000014	83158851	83159542	0	0	0	0
RNU7-51P	NC_000014	83514924	83514982	0	0	0	0
LOC100287804	NC_000014	84637839	84641861	0	0	0	0
RNU3P3	NC_000014	85738249	85738393	0	0	0	0
FLRT2	NC_000014	85996488	86094270	1.9773337	1.1203016	0.4520265	1.0963357
LOC730121	NC_000014	88100723	88121632	0	0.0823013	0	0.055783
GALC	NC_000014	88399358	88459907	1.8531763	1.3943523	0.8565544	0.9811504
FAM35A2	NC_000014	88418870	88421747	0	0	0	0
GPR65	NC_000014	88471496	88478415	0	0	0	0.03492
KCNK10	NC_000014	88646452	88793256	0.0107479	0.0221	0.0095616	0.0187239
LOC100287861	NC_000014	88729846	88793285	0.0764317	0	0	0
SPATA7	NC_000014	88852012	88904804	1.9886078	1.7718981	0.6290207	0.6466792
PTPN21	NC_000014	88932122	89021123	0.9899843	0.9451074	0.2956694	0.3350751
ZC3H14	NC_000014	89029314	89079853	5.4047805	7.308543	1.9185599	3.2900717
EML5	NC_000014	89081201	89259157	0.049652	0.1148568	0.0276074	0.0605491
TTC8	NC_000014	89290978	89344335	5.4262002	6.7477709	1.2068245	2.0018077
LOC390501	NC_000014	89576449	89577567	0	0	0	0
FOXN3	NC_000014	89622516	90085494	5.3333124	6.3542649	1.5654487	2.501612
CAP2P1	NC_000014	89755862	89758075	0	0	0	0
LOC400236	NC_000014	89882773	89886137	0.027314	0.0280817	0	0.0190335
PRO1768	NC_000014	90042560	90043821	0	0	0	0
CHORDC2P	NC_000014	90201022	90204786	0	0	0	0
C14orf143	NC_000014	90263469	90421089	1.3279322	1.495278	0.5625549	0.6609686
RAB42P	NC_000014	90358366	90359254	0	0	0	0
TDP1	NC_000014	90422246	90511108	2.2754089	3.1955168	1.3460331	2.3865664

KCNK13	NC_000014	90528108	90652195	0.0349211	0.0179513	0	0
GLRXP2	NC_000014	90573955	90575647	0	0	0	0
PSMC1	NC_000014	90722894	90738966	22.611448	35.468686	10.181144	12.609108
C14orf102	NC_000014	90744398	90798279	0.427018	0.3203655	0.0718705	0.3056054
RPL21P11	NC_000014	90832979	90833566	0	0	0	0
CALM1	NC_000014	90863373	90874610	33.361321	29.910745	12.768831	16.3877
TTC7B	NC_000014	91006932	91282761	2.2908368	3.4341491	1.1157721	1.9173992
RPS18P2	NC_000014	91232839	91233371	0	0	0	0
LOC283588	NC_000014	91314206	91316995	0	0	0	0
RPS6KA5	NC_000014	91337159	91526993	0.1626959	0.1149971	0.153785	0.226746
RNU7-30P	NC_000014	91557321	91557581	0	0	0	0
C14orf159	NC_000014	91580357	91691703	4.0327497	3.6069704	1.099619	1.5312439
SNORA11B	NC_000014	91592770	91592896	0	0	0	0
GPR68	NC_000014	91698876	91720224	2.9986	2.3924403	0.8614247	0.3156082
CCDC88C	NC_000014	91737667	91884133	0.1115139	0.1749891	0.0626564	0.0777074
SMEK1	NC_000014	91923955	91976644	0.9367497	1.5316407	0.411659	0.8257864
PP8961	NC_000014	92038788	92041383	0.0169292	0.03481	0	0.0117969
CATSPERB	NC_000014	92047118	92198413	0.0487501	0.0626503	0.0216847	0.2123189
NANOGP7	NC_000014	92070529	92071810	0	0	0	0
LOC100128939	NC_000014	92175444	92176991	0	0	0	0
TC2N	NC_000014	92246266	92333880	0.0414215	0.1788598	0.0884394	0.0230913
FBLN5	NC_000014	92335755	92414046	2.5871538	11.016994	0.0445472	0.1046806
LOC100287962	NC_000014	92409256	92414083	0	0	0	0
TRIP11	NC_000014	92435462	92506817	1.8103075	1.6316401	0.7461982	1.2951342
PTMAP7	NC_000014	92492766	92493911	0	0	0	0
ATXN3	NC_000014	92524896	92572965	1.5172075	1.9579692	0.7228786	1.0041669
NDUFB1	NC_000014	92582467	92588153	13.498385	12.694384	9.0296759	6.781223
CPSF2	NC_000014	92588329	92630543	5.1001221	6.489344	2.2181916	3.0375797
SLC24A4	NC_000014	92788925	92962596	0.0097209	0.0099941	0	0.0203218
RIN3	NC_000014	92980125	93155336	2.9915398	6.6912287	0.8329437	2.1323636
LOC100287697	NC_000014	93151449	93155339	0.0392395	0	0	0
LGMN	NC_000014	93170154	93215012	2.1172441	3.275691	0.7314792	0.3151297
GOLGA5	NC_000014	93260650	93306304	5.1229865	5.6576934	2.4883957	1.959738
CHGA	NC_000014	93389445	93401638	0	0	0.0382747	0.0599606
ITPK1	NC_000014	93403259	93582263	5.7833581	6.4987962	1.6940971	2.7645326
C14orf85	NC_000014	93533797	93538497	0	0	0	0
CYB5P3	NC_000014	93604329	93605122	0	0	0	0
MOAP1	NC_000014	93648541	93651249	1.3637225	0.9347001	0.5338104	0.4561422
C14orf109	NC_000014	93651296	93653431	0.6453485	0.8293576	0.200942	0.1573966
C14orf142	NC_000014	93669237	93673459	1.9059182	2.4205394	1.0638793	1.015621
UBR7	NC_000014	93673401	93695561	5.0801015	5.7855303	2.3814164	3.0757525
RPL18AP1	NC_000014	93689895	93690520	0	0	0	0
BTBD7	NC_000014	93703896	93799385	0.7749059	0.9484341	0.4759989	0.5239143
RPL36AP4	NC_000014	93710306	93710769	0	0	0	0
KIAA1409	NC_000014	93799565	94173689	0.0259404	0.0266695	0.0276928	0.0650747
COX8C	NC_000014	93813537	93814700	0	0	0	0
PRIMA1	NC_000014	94184644	94254766	0.0120406	0.012379	0.032135	0.0503423
C14orf86	NC_000014	94371076	94392718	0	0	0	0
FAM181A	NC_000014	94385258	94395944	0	0	0	0
ASB2	NC_000014	94400513	94423767	0	0.0823913	0.0142588	0.0111688
C14orf48	NC_000014	94463616	94478041	0	0	0	0
OTUB2	NC_000014	94492724	94515276	0.5673668	0.4549839	0.3331319	0.4032711
DDX24	NC_000014	94517268	94547558	23.390358	24.492224	8.1431158	9.3495219
IFI27L1	NC_000014	94547639	94569060	9.465773	6.3193569	6.5071473	1.5847839
IFI27	NC_000014	94577079	94583033	425.66424	238.66815	177.85794	3.8515951
IFI27L2	NC_000014	94594118	94595957	45.736275	28.25172	11.637177	7.8693477
PPP4R4	NC_000014	94640649	94746072	0.2542124	0.0627257	0.1628311	0.0850297
SERPINA10	NC_000014	94749650	94759596	0.0522364	0	0.046471	0.0242669
SERPINA6	NC_000014	94770585	94789688	0.1178237	0.0605676	0	0.0205261
LOC100287997	NC_000014	94780655	94815794	0.0788309	0	0	0.0274663
SERPINA2	NC_000014	94830651	94833143	0	0	0	0
SERPINA1	NC_000014	94843084	94857029	0.1191978	0	0.0530208	0.0498371
SERPINA11	NC_000014	94908801	94919122	0	0	0	0.0208191
SERPINA9	NC_000014	94929058	94942670	0.0238719	0	0.0212371	0.0665397
SERPINA12	NC_000014	94953620	94984181	0	0	0.0379404	0.0148592
SERPINA4	NC_000014	95027783	95036243	0	0	0.0235386	0.0184376

SERPINA5	NC_000014	95047731	95059457	0.0190088	0	0	0
SERPINA3	NC_000014	95078714	95090390	0	0.0284172	0.0491793	0.0577828
LOC390503	NC_000014	95098200	95100865	0	0	0	0
SERPINA13	NC_000014	95107062	95113331	0	0	0	0
LOC100288028	NC_000014	95141212	95142170	0	0	0	0
RPSAP4	NC_000014	95192549	95193582	0	0	0	0
GSC	NC_000014	95234560	95236499	0.1478494	0.3800118	0.0657655	0.0257568
RPL15P2	NC_000014	95265762	95267836	0	0	0	0
DICER1	NC_000014	95552565	95623759	0.5021932	0.5770494	0.3378895	0.829289
FLJ45244	NC_000014	95624025	95646270	0	0	0	0
CLMN	NC_000014	95648276	95786245	0.0551507	0.0531569	0.2115868	0.048039
C14orf139	NC_000014	95873603	95876427	0	0	0	0
C14orf49	NC_000014	95883831	95942173	3.623213	2.5661413	1.1341279	1.561635
LOC100287786	NC_000014	95883831	95899700	0.3866413	0	0	0.1796181
SNHG10	NC_000014	95999249	96001209	0	0	0	0
SCARNA13	NC_000014	95999692	95999966	0	0	0	0
GLRX5	NC_000014	96001323	96011055	5.8547602	9.2990605	3.1718777	4.7074962
TCL6	NC_000014	96117515	96146170	0.0176051	0.0361998	0.0052207	0.0531611
TCL1B	NC_000014	96152763	96158967	0.0252867	0	0	0.0176208
TCL1A	NC_000014	96176304	96180533	0	0	0	0.0219376
C14orf132	NC_000014	96505662	96560138	0	0	0	0
BDKRB2	NC_000014	96671135	96710666	4.0419389	2.1423629	1.4998113	0.7515755
CKS1BP	NC_000014	96689374	96690167	0	0	0	0
BDKRB1	NC_000014	96722559	96731100	22.059353	15.914092	4.1554434	4.4428644
LOC100130815	NC_000014	96742336	96742719	0	0	0	0
ATG2B	NC_000014	96747595	96829678	1.3225254	1.6609449	0.7503182	0.8939959
C14orf129	NC_000014	96846022	96853568	3.6906334	4.6884321	1.3963416	1.3597919
AK7	NC_000014	96858448	96955112	0.0829838	0	0.0295299	0.0925223
PBPP1	NC_000014	96889282	96890624	0	0	0	0
LOC100288056	NC_000014	96889822	96940625	0	0	0	0
RPL23AP10	NC_000014	96922866	96923390	0	0	0	0
LOC730202	NC_000014	96967157	96968750	0	0	0	0
PAPOLA	NC_000014	96968720	97033448	4.8928836	6.9643966	3.6591645	6.2350052
VRK1	NC_000014	97263684	97347951	5.6515929	9.2860779	4.24724	4.1001011
C14orf64	NC_000014	98391947	98444461	0	0	0	0
C14orf177	NC_000014	99177950	99184097	0.0342543	0.070434	0	0.0477395
RPL3P4	NC_000014	99439160	99439638	0	0	0	0
BCL11B	NC_000014	99635624	99737822	0.0281143	0.0289044	0.0100045	0.0274276
SETD3	NC_000014	99864083	99947226	3.2628233	4.107582	1.5520549	1.2621162
CCNK	NC_000014	99947739	99977852	4.9243606	6.0683771	2.5354906	2.8909133
CCDC85C	NC_000014	99977603	100070727	1.5421792	2.3742997	0.6687469	0.7398357
HHIPL1	NC_000014	100111480	100143011	5.5709026	4.1538646	1.1313764	1.3489063
CYP46A1	NC_000014	100150755	100193638	0.1904353	0.0652625	0.0941203	0.0147448
EML1	NC_000014	100259745	100408397	3.479316	3.4072402	1.2104509	1.9910912
LOC341965	NC_000014	100430866	100431715	0	0	0	0
RPS2P3	NC_000014	100440627	100441372	0	0	0	0
EVL	NC_000014	100531751	100610573	0.4294615	1.0057109	0.3396097	0.3491436
DEGS2	NC_000014	100612753	100626012	0.0625598	0.032159	0	0.0217971
YY1	NC_000014	100705102	100744804	9.1050156	12.812423	6.018491	4.9741797
SLC25A29	NC_000014	100757453	100772860	4.3577361	3.8892042	1.3362459	1.5506267
C14orf68	NC_000014	100789679	100796715	0	0	0	0
WARS	NC_000014	100800125	100842680	50.863267	30.355695	13.186207	7.2228487
NDUFB3P4	NC_000014	100832925	100833380	0	0	0	0
WDR25	NC_000014	100842950	100996640	1.030722	1.1047838	0.331666	0.5348656
LOC100288006	NC_000014	101003484	101004444	0.0457318	0	0	0
BEGAIN	NC_000014	101003487	101036131	1.3482061	0.1690363	0.1755222	0.5957702
LOC100130467	NC_000014	101005276	101006746	0.0794724	0.1634119	0.2121025	0.4984157
C14orf70	NC_000014	101123605	101139081	0	0	0	0
LOC100288131	NC_000014	101192796	101195123	0	0	0.0409828	0
DLK1	NC_000014	101193253	101201460	0.1147473	0	0	0.0599704
MEG3	NC_000014	101292445	101327368	0	0	0	0
RTL1	NC_000014	101347049	101351125	0.3449457	0.2548978	0.0958979	0.2478835
LOC100288160	NC_000014	101359010	101359615	0.6462975	0.1107436	0	0.075061
MEG8	NC_000014	101361107	101373305	0	0	0	0
SNORD112	NC_000014	101364257	101364333	0	0	0	0
SNORD113-1	NC_000014	101391158	101391227	0	0	0	0

SNORD113-2	NC_000014	101393679	101393749	0	0	0	0
SNORD113-3	NC_000014	101396256	101396326	0	0	0	0
SNORD113-4	NC_000014	101402828	101402901	0	0	0	0
SNORD113-5	NC_000014	101404524	101404600	0	0	0	0
SNORD113-6	NC_000014	101405893	101405966	0	0	0	0
SNORD113-7	NC_000014	101407463	101407538	0	0	0	0
SNORD113-8	NC_000014	101409788	101409860	0	0	0	0
SNORD113-9	NC_000014	101411986	101412056	0	0	0	0
SNORD114-1	NC_000014	101416170	101416240	0	0	0	0
SNORD114-2	NC_000014	101418193	101418269	0	0	0	0
SNORD114-3	NC_000014	101419686	101419759	0	0	0	0
SNORD114-4	NC_000014	101420681	101420784	0	0	0	0
SNORD114-5	NC_000014	101421707	101421775	0	0	0	0
SNORD114-6	NC_000014	101423503	101423573	0	0	0	0
SNORD114-7	NC_000014	101429391	101429466	0	0	0	0
SNORD114-8	NC_000014	101431118	101431188	0	0	0	0
SNORD114-9	NC_000014	101432366	101432436	0	0	0	0
SNORD114-10	NC_000014	101433389	101433459	0	0	0	0
SNORD114-11	NC_000014	101434448	101434521	0	0	0	0
SNORD114-12	NC_000014	101435285	101435358	0	0	0	0
SNORD114-13	NC_000014	101436216	101436288	0	0	0	0
SNORD114-14	NC_000014	101438440	101438513	0	0	0	0
SNORD114-15	NC_000014	101439007	101439077	0	0	0	0
SNORD114-16	NC_000014	101439932	101440000	0	0	0	0
SNORD114-17	NC_000014	101441143	101441216	0	0	0	0
SNORD114-18	NC_000014	101442162	101442232	0	0	0	0
SNORD114-19	NC_000014	101442814	101442887	0	0	0	0
SNORD114-20	NC_000014	101447341	101447411	0	0	0	0
SNORD114-21	NC_000014	101448312	101448382	0	0	0	0
SNORD114-22	NC_000014	101449263	101449333	0	0	0	0
SNORD114-23	NC_000014	101450213	101450283	0	0	0	0
SNORD114-24	NC_000014	101451114	101451184	0	0	0	0
SNORD114-25	NC_000014	101452394	101452464	0	0	0	0
SNORD114-26	NC_000014	101453383	101453453	0	0	0	0
SNORD114-27	NC_000014	101454498	101454566	0	0	0	0
SNORD114-28	NC_000014	101455467	101455537	0	0	0	0
SNORD114-29	NC_000014	101456428	101456496	0	0	0	0
SNORD114-30	NC_000014	101458256	101458326	0	0	0	0
SNORD114-31	NC_000014	101459573	101459646	0	0	0	0
LOC100130814	NC_000014	101540364	101540920	0.0789017	0	0	0
LOC100128373	NC_000014	101683767	101684432	0	0	0	0
DIO3OS	NC_000014	102018560	102022013	0	0	0	0
DIO3	NC_000014	102027688	102029789	0.0209078	0.0429909	0	0.0145694
RPL26P4	NC_000014	102144274	102144760	0	0	0	0
C14orf72	NC_000014	102196774	102198862	0	0	0	0
PPP2R5C	NC_000014	102276172	102394078	5.7246001	7.6429635	3.2095017	3.3866046
NPM1P20	NC_000014	102308999	102310568	0	0	0	0
LOC100288225	NC_000014	102430633	102437389	0	0	0.0526921	0
DYNC1H1	NC_000014	102430865	102517135	4.1772273	5.8952681	1.6876843	3.6198862
HSP90AA1	NC_000014	102547075	102606086	23.743264	40.414413	9.8751583	26.122104
WDR20	NC_000014	102606212	102690010	1.6685795	2.0198336	0.7781206	0.909557
RAGE	NC_000014	102695178	102771531	3.8176334	1.6587495	0.8086363	1.3459745
ZNF839	NC_000014	102786096	102809044	0.9004157	0.8984949	0.459417	0.4336756
CINP	NC_000014	102814619	102829253	4.9828945	4.9383289	2.7555996	4.7235512
TECPR2	NC_000014	102829378	102968817	1.879046	2.8452892	0.4905934	0.3949531
ANKRD9	NC_000014	102973198	102976128	2.1352462	3.0844758	1.0820359	0.9793934
RCOR1	NC_000014	103059233	103196913	2.5800375	3.1110152	1.1405523	1.8866568
RPL23AP11	NC_000014	103128530	103129065	0	0	0	0
TRAF3	NC_000014	103243816	103372628	2.3112627	3.2387012	1.4926223	1.4674172
AMN	NC_000014	103388993	103397179	0	0.0290195	0.0502217	0.0590075
LOC100288262	NC_000014	103394982	103397250	0.1036515	0.1065646	0	0
CDC42BPB	NC_000014	103398716	103523742	1.9806545	2.5150588	0.8168421	1.0328641
RPL13P6	NC_000014	103507806	103508431	0	0	0	0
C14orf73	NC_000014	103566481	103576896	0.1362208	0.105037	0.2423716	0.1186551
TNFAIP2	NC_000014	103592664	103603776	2.1368841	2.5107902	1.6294554	0.4694592
RPL21P12	NC_000014	103614894	103615775	0	0	0	0

LOC100288298	NC_000014	103650541	103651369	0	0	0	0
LOC100128932	NC_000014	103668116	103687427	0	0	0	0
RPL21P13	NC_000014	103707789	103708342	0	0	0	0
LOC441698	NC_000014	103744859	103746292	0	0	0	0
RPL17P4	NC_000014	103749235	103749835	0	0	0	0
EIF5	NC_000014	103800493	103811362	8.1996207	9.24037	4.2905687	5.8145952
SNORA28	NC_000014	103804186	103804311	0	0	0	0
LOC100130405	NC_000014	103830576	103831157	0	0	0	0
RPSAP5	NC_000014	103840295	103841332	0	0	0	0
MARK3	NC_000014	103851701	103970168	9.6681133	13.12519	4.4056713	3.6590313
RPL10AP1	NC_000014	103878424	103879129	0	0	0	0
CKB	NC_000014	103985996	103989170	37.713787	25.070315	10.300337	9.1810432
TRMT61A	NC_000014	103995509	104003410	4.4341474	4.8515347	0.6393616	1.3228889
BAG5	NC_000014	104022889	104029151	1.4109121	1.666424	0.6948354	0.5501125
C14orf153	NC_000014	104029299	104057236	3.2382908	5.0854193	2.3426881	1.8598104
KLC1	NC_000014	104095525	104167888	10.799186	14.650983	2.8722721	4.160251
XRCC3	NC_000014	104163954	104181823	2.5313116	2.7565471	1.0370707	1.2417059
LOC100288101	NC_000014	104177607	104179153	0	0.0292071	0.0505463	0
ZFYVE21	NC_000014	104182145	104199999	9.7556812	9.7031601	3.5620342	2.8344067
PPP1R13B	NC_000014	104200088	104313927	0.2835942	0.3917899	0.1655674	0.2346734
C14orf2	NC_000014	104378625	104387903	16.271821	19.133037	10.188291	12.269902
TDRD9	NC_000014	104394817	104519003	0	0.028459	0.0082086	0.0385785
ASPG	NC_000014	104552048	104578925	0.0238331	0	0.0212026	0.0332157
KIF26A	NC_000014	104605060	104647235	0.0455287	0.0133738	0.0057862	0.0226616
LOC100288441	NC_000014	104705559	104710782	0.1241475	0	0.1104451	0
LOC122631	NC_000014	104917143	104918171	0	0	0	0
C14orf180	NC_000014	105046056	105056183	0	0	0.0423363	0
LOC100288144	NC_000014	105057201	105058846	0	0.0549009	0.0475062	0.3349015
TMEM179	NC_000014	105060420	105071097	0.0270784	0	0	0.0566079
LOC100128366	NC_000014	105131255	105131889	0	0	0	0
INF2	NC_000014	105155943	105185947	8.5190522	5.2953796	4.1006789	4.811552
C14orf151	NC_000014	105155958	105171146	0.0260203	0	0.0231484	0
ADSSL1	NC_000014	105190534	105213647	0.7613746	0.4131302	0.094075	0.044213
SIVA1	NC_000014	105219470	105225996	8.2082301	14.650909	2.9411917	2.2243751
AKT1	NC_000014	105235686	105262080	5.2348309	6.146834	1.5764177	1.7909286
ZBTB42	NC_000014	105266933	105271049	1.5225398	0.6584329	0.3332484	0.3536555
MGC23270	NC_000014	105287538	105290055	0	0	0	0
RPS26P49	NC_000014	105297173	105297507	0	0	0	0
RPS2P4	NC_000014	105302281	105303244	0	0	0	0
KIAA0284	NC_000014	105331654	105363087	2.8417357	2.7525288	0.8075833	1.2834854
LOC100288473	NC_000014	105349466	105357512	0.0176712	0.0181678	0.0314415	0.024628
PLD4	NC_000014	105391187	105399573	0.022724	0.0467253	0.0606477	0
AHNAK2	NC_000014	105403591	105444694	2.2649523	2.6443526	1.630755	3.420784
C14orf79	NC_000014	105452616	105461855	1.7672305	1.362674	0.7860886	0.5347201
CDCA4	NC_000014	105475910	105487425	6.0556471	10.480781	1.9604568	1.9351228
GPR132	NC_000014	105515737	105531754	0.0241739	0	0.0537645	0.0084227
JAG2	NC_000014	105608076	105635161	0.1731268	0.1690929	0.1463175	0.0784171
NUDT14	NC_000014	105639276	105647660	10.771127	6.0070419	1.4915834	2.8323587
BRF1	NC_000014	105675623	105767329	1.4135683	1.9377291	0.4366492	0.5882822
BTBD6	NC_000014	105715008	105717430	1.7516413	5.4949666	0.1997832	0.3129778
PACS2	NC_000014	105781081	105864484	3.7548947	2.968461	0.9755868	1.6686012
RPS20P33	NC_000014	105840958	105841308	0	0	0	0
LOC647310	NC_000014	105864926	105878187	0.2371307	0	0.0703194	0
MTA1	NC_000014	105886186	105937062	8.7124087	11.530105	2.5286299	3.4338616
CRIP2	NC_000014	105941131	105946500	4.5187237	5.6198261	2.8853096	2.0822886
CRIP1	NC_000014	105953257	105955126	13.642264	33.416891	7.0049805	12.377555
C14orf80	NC_000014	105956520	105965585	0.5059451	0.6620279	0.5728581	0.4967929
TMEM121	NC_000014	105992953	105996539	0.6075792	1.1303287	0.3346072	0.2217733
ATP5GP3	NC_000014	106002984	106003730	0	0	0	0
ELK2P2	NC_000014	106010329	106017197	0	0	0	0
IGHA2	NC_000014	106053244	106054731	0	0	0	0
IGHE	NC_000014	106066279	106068064	0	0	0	0
IGHG4	NC_000014	106090707	106092402	0	0	0	0
LOC100288531	NC_000014	106096137	106097297	0.0667906	0.0686678	0	0.0930847
IGHG2	NC_000014	106109434	106111126	0	0	0	0
IGHGP	NC_000014	106134477	106136377	0	0	0	0

ELK2P1	NC_000014	106136378	106140094	0	0	0	0
IGHA1	NC_000014	106173475	106175001	0	0	0	0
IGHEP1	NC_000014	106187565	106188864	0	0	0	0
IGHG1	NC_000014	106207704	106209407	0	0	0	0
IGHG3	NC_000014	106232251	106237742	0	0	0	0
ATP6V1GP1	NC_000014	106250833	106252309	0	0	0	0
IGHD	NC_000014	106304737	106312010	0	0	0	0
IGHM	NC_000014	106318037	106322322	0	0	0	0
LOC100288273	NC_000014	106320322	106321728	0.074615	0	0	0.0519947
IGHJ6	NC_000014	106329406	106329470	0	0	0	0
IGHJ3P	NC_000014	106329625	106329675	0	0	0	0
IGHJ5	NC_000014	106330022	106330074	0	0	0	0
IGHJ4	NC_000014	106330423	106330472	0	0	0	0
IGHJ3	NC_000014	106330795	106330846	0	0	0	0
IGHJ2P	NC_000014	106331003	106331062	0	0	0	0
IGHJ2	NC_000014	106331407	106331461	0	0	0	0
IGHJ1	NC_000014	106331615	106331668	0	0	0	0
IGHD7-27	NC_000014	106331761	106331771	0	0	0	0
IGHJ1P	NC_000014	106331834	106331887	0	0	0	0
IGHD1-26	NC_000014	106346892	106346911	0	0	0	0
IGHD6-25	NC_000014	106347397	106347414	0	0	0	0
IGHD5-24	NC_000014	106349761	106349780	0	0	0	0
IGHD4-23	NC_000014	106350728	106350746	0	0	0	0
IGHD3-22	NC_000014	106351889	106351919	0	0	0	0
IGHD2-21	NC_000014	106354409	106354436	0	0	0	0
IGHD1-20	NC_000014	106357049	106357065	0	0	0	0
IGHD6-19	NC_000014	106357557	106357577	0	0	0	0
IGHD5-18	NC_000014	106359400	106359419	0	0	0	0
IGHD4-17	NC_000014	106360366	106360381	0	0	0	0
IGHD3-16	NC_000014	106361492	106361528	0	0	0	0
IGHD2-15	NC_000014	106363815	106363845	0	0	0	0
IGHD1-14	NC_000014	106366496	106366512	0	0	0	0
IGHD6-13	NC_000014	106367000	106367020	0	0	0	0
IGHD5-12	NC_000014	106368507	106368529	0	0	0	0
IGHD4-11	NC_000014	106369474	106369489	0	0	0	0
IGHD3-10	NC_000014	106370355	106370385	0	0	0	0
IGHD3-9	NC_000014	106370539	106370569	0	0	0	0
IGHD2-8	NC_000014	106373069	106373099	0	0	0	0
IGHD1-7	NC_000014	106375766	106375782	0	0	0	0
IGHD6-6	NC_000014	106376269	106376286	0	0	0	0
IGHD5-5	NC_000014	106378116	106378135	0	0	0	0
IGHD4-4	NC_000014	106379081	106379096	0	0	0	0
IGHD3-3	NC_000014	106380218	106380248	0	0	0	0
IGHD2-2	NC_000014	106382685	106382715	0	0	0	0
KIAA0125	NC_000014	106383838	106398502	0	0	0	0
IGHD1-1	NC_000014	106385361	106385377	0	0	0	0
IGHV6-1	NC_000014	106405609	106406056	0	0	0	0
IGHVII-1-1	NC_000014	106410796	106411247	0	0	0	0
ADAM6	NC_000014	106435819	106438358	0	0	0	0
RPS8P1	NC_000014	106444554	106445256	0	0	0	0
IGHV1-2	NC_000014	106452669	106453106	0	0	0	0
IGHVIII-2-1	NC_000014	106467677	106467978	0	0	0	0
IGHV1-3	NC_000014	106471244	106471681	0	0	0	0
IGHV4-4	NC_000014	106478108	106478539	0	0	0	0
IGHV2-5	NC_000014	106494134	106494577	0	0	0	0
IGHVIII-5-1	NC_000014	106495898	106495996	0	0	0	0
IGHVIII-5-2	NC_000014	106501831	106502091	0	0	0	0
IGHV3-6	NC_000014	106511777	106512231	0	0	0	0
IGHV3-7	NC_000014	106518398	106518853	0	0	0	0
IGHV1-8	NC_000014	106539077	106539515	0	0	0	0
IGHV3-9	NC_000014	106552284	106552729	0	0	0	0
IGHV2-10	NC_000014	106559540	106559980	0	0	0	0
IGHV3-11	NC_000014	106573231	106573680	0	0	0	0
IGHVIII-11-1	NC_000014	106576885	106577071	0	0	0	0
IGHV1-12	NC_000014	106579014	106579303	0	0	0	0
IGHV3-13	NC_000014	106586135	106586587	0	0	0	0

IGHVIII-13-1	NC_000014	106598994	106599292	0	0	0	0
IGHV1-14	NC_000014	106602571	106602820	0	0	0	0
IGHV3-15	NC_000014	106610311	106610772	0	0	0	0
IGHVII-15-1	NC_000014	106620221	106620491	0	0	0	0
IGHV3-16	NC_000014	106621892	106622345	0	0	0	0
IGHVIII-16-1	NC_000014	106627436	106627740	0	0	0	0
IGHV1-17	NC_000014	106631031	106631467	0	0	0	0
LOC192128	NC_000014	106635565	106637149	0	0	0	0
IGHV1-18	NC_000014	106641561	106641997	0	0	0	0
IGHV3-19	NC_000014	106653369	106653661	0	0	0	0
IGHV3-20	NC_000014	106667579	106668033	0	0	0	0
IGHVII-20-1	NC_000014	106669465	106669965	0	0	0	0
IGHV3-21	NC_000014	106691671	106692124	0	0	0	0
IGHV3-22	NC_000014	106714360	106714821	0	0	0	0
IGHVII-22-1	NC_000014	106719953	106720221	0	0	0	0
IGHVIII-22-2	NC_000014	106721279	106721466	0	0	0	0
IGHV3-23	NC_000014	106725199	106725654	0	0	0	0
IGHV1-24	NC_000014	106733142	106733579	0	0	0	0
IGHV3-25	NC_000014	106745620	106746073	0	0	0	0
IGHVIII-25-1	NC_000014	106750160	106750401	0	0	0	0
IGHV2-26	NC_000014	106757649	106758092	0	0	0	0
IGHVIII-26-1	NC_000014	106765667	106765973	0	0	0	0
IGHVII-26-2	NC_000014	106770911	106771223	0	0	0	0
IGHV7-27	NC_000014	106774086	106774499	0	0	0	0
IGHV4-28	NC_000014	106780511	106780945	0	0	0	0
IGHVII-28-1	NC_000014	106785686	106785938	0	0	0	0
IGHV3-29	NC_000014	106787358	106787815	0	0	0	0
LOC192132	NC_000014	106788488	106789003	0	0	0	0
IGHV3-30	NC_000014	106791003	106791456	0	0	0	0
IGHVII-30-1	NC_000014	106798602	106798875	0	0	0	0
IGHV3-30-2	NC_000014	106800309	106800757	0	0	0	0
IGHV4-31	NC_000014	106805207	106805644	0	0	0	0
IGHVII-31-1	NC_000014	106810541	106810643	0	0	0	0
IGHV3-32	NC_000014	106812071	106812528	0	0	0	0
GOLGA4P1	NC_000014	106813213	106813726	0	0	0	0
IGHV3-33	NC_000014	106815720	106816173	0	0	0	0
IGHVII-33-1	NC_000014	106823321	106823594	0	0	0	0
IGHV3-33-2	NC_000014	106825029	106825477	0	0	0	0
IGHV4-34	NC_000014	106829592	106830024	0	0	0	0
IGHV7-34-1	NC_000014	106833231	106833664	0	0	0	0
LOC100288568	NC_000014	106842211	106929519	0	0	0	0.0376227
IGHV3-35	NC_000014	106845321	106845774	0	0	0	0
IGHV3-36	NC_000014	106848680	106849142	0	0	0	0
IGHV3-37	NC_000014	106852577	106853026	0	0	0	0
IGHV3-38	NC_000014	106866407	106866856	0	0	0	0
IGHVIII-38-1	NC_000014	106873935	106874227	0	0	0	0
IGHV4-39	NC_000014	106877617	106878055	0	0	0	0
IGHV7-40	NC_000014	106881264	106881466	0	0	0	0
IGHVII-40-1	NC_000014	106896845	106896921	0	0	0	0
IGHV3-41	NC_000014	106899040	106899494	0	0	0	0
IGHV3-42	NC_000014	106919179	106919613	0	0	0	0
IGHV3-43	NC_000014	106926187	106926644	0	0	0	0
IGHVII-43-1	NC_000014	106928715	106928923	0	0	0	0
IGHVIII-44	NC_000014	106934223	106934399	0	0	0	0
IGHVIV-44-1	NC_000014	106945349	106945777	0	0	0	0
IGHVII-44-2	NC_000014	106950159	106950395	0	0	0	0
IGHV1-45	NC_000014	106962929	106963366	0	0	0	0
IGHV1-46	NC_000014	106967047	106967484	0	0	0	0
IGHVII-46-1	NC_000014	106971770	106972058	0	0	0	0
IGHV3-47	NC_000014	106974506	106974961	0	0	0	0
IGHVIII-47-1	NC_000014	106987135	106987438	0	0	0	0
IGHV3-48	NC_000014	106993812	106994267	0	0	0	0
IGHV3-49	NC_000014	107012936	107013397	0	0	0	0
IGHVII-49-1	NC_000014	107020316	107020586	0	0	0	0
IGHV3-50	NC_000014	107022088	107022543	0	0	0	0
IGHV5-51	NC_000014	107034727	107035162	0	0	0	0

IGHVIII-51-1	NC_000014	107039488	107039794	0	0	0	0
IGHVII-51-2	NC_000014	107040698	107040960	0	0	0	0
IGHV3-52	NC_000014	107042360	107042810	0	0	0	0
IGHV3-53	NC_000014	107048670	107049120	0	0	0	0
IGHVII-53-1	NC_000014	107055667	107055936	0	0	0	0
IGHV3-54	NC_000014	107057348	107057795	0	0	0	0
IGHV4-55	NC_000014	107062126	107062561	0	0	0	0
IGHV7-56	NC_000014	107065772	107066206	0	0	0	0
IGHV3-57	NC_000014	107074922	107075227	0	0	0	0
IGHV1-58	NC_000014	107078371	107078808	0	0	0	0
IGHV4-59	NC_000014	107083254	107083685	0	0	0	0
IGHV3-60	NC_000014	107087202	107087658	0	0	0	0
IGHVII-60-1	NC_000014	107093710	107093978	0	0	0	0
IGHV4-61	NC_000014	107095124	107095561	0	0	0	0
IGHV3-62	NC_000014	107099133	107099588	0	0	0	0
IGHVII-62-1	NC_000014	107106520	107106792	0	0	0	0
IGHV3-63	NC_000014	107108228	107108695	0	0	0	0
LOC192130	NC_000014	107109482	107110398	0	0	0	0
IGHV3-64	NC_000014	107113739	107114194	0	0	0	0
IGHV3-65	NC_000014	107122054	107122548	0	0	0	0
IGHVII-65-1	NC_000014	107127788	107128060	0	0	0	0
IGHV3-66	NC_000014	107131031	107131481	0	0	0	0
IGHV1-67	NC_000014	107136620	107137059	0	0	0	0
SLC20A1P1	NC_000014	107138547	107140471	0	0	0	0
LOC192127	NC_000014	107138647	107140366	0	0	0	0
IGHVII-67-1	NC_000014	107142410	107142559	0	0	0	0
IGHVIII-67-2	NC_000014	107143119	107143217	0	0	0	0
IGHVIII-67-3	NC_000014	107148631	107148905	0	0	0	0
IGHVIII-67-4	NC_000014	107151109	107151414	0	0	0	0
IGHV1-68	NC_000014	107159824	107160303	0	0	0	0
IGHV1-69	NC_000014	107169929	107170367	0	0	0	0
IGHV2-70	NC_000014	107178819	107179262	0	0	0	0
IGHV3-71	NC_000014	107183396	107183857	0	0	0	0
IGHV3-72	NC_000014	107198930	107199391	0	0	0	0
IGHV3-73	NC_000014	107210930	107211391	0	0	0	0
C14orf99	NC_000014	107217264	107218407	0	0	0	0
IGHV3-74	NC_000014	107218674	107219129	0	0	0	0
IGHVII-74-1	NC_000014	107229473	107229641	0	0	0	0
IGHV3-75	NC_000014	107231908	107232377	0	0	0	0
IGHV3-76	NC_000014	107236089	107236538	0	0	0	0
IGHVIII-76-1	NC_000014	107239990	107240296	0	0	0	0
IGHV5-78	NC_000014	107259327	107259761	0	0	0	0
IGHVII-78-1	NC_000014	107273836	107274110	0	0	0	0
IGHV3-79	NC_000014	107275853	107276304	0	0	0	0
IGHV4-80	NC_000014	107280994	107281394	0	0	0	0
IGHV7-81	NC_000014	107282790	107283226	0	0	0	0
IGHVIII-82	NC_000014	107287760	107288051	0	0	0	0
LOC729722	NC_000015	20059051	20093187	0	0	0	0.0992167
LOC100287545	NC_000015	20103448	20105116	0	0	0	0
IGHV1OR15-9	NC_000015	20169909	20170354	0	0	0	0
IGHV1OR15-2	NC_000015	20172658	20177892	0	0	0	0
LOC100287128	NC_000015	20178025	20178471	0	0	0	0
LOC646057	NC_000015	20192722	20193370	0	0	0	0
LOC100287637	NC_000015	20209536	20225048	0	0	0	0
LOC646071	NC_000015	20251372	20253571	0	0	0	0
LOC646079	NC_000015	20270807	20279587	0.0397003	0.0408161	0.0353185	0.110659
LOC100130655	NC_000015	20347047	20351683	0.0094777	0	0.0084317	0.0396268
LOC642311	NC_000015	20359029	20366079	0	0	0	0
LOC646090	NC_000015	20433870	20482347	0	0	0	0
LOC646096	NC_000015	20485906	20496686	0	0	0	0
LOC100287760	NC_000015	20563202	20563354	0	0	0	0
HERC2P3	NC_000015	20636291	20671623	0	0	0	0
LOC646139	NC_000015	20710600	20739124	0	0	0	0
LOC727832	NC_000015	20737094	20747114	0	0.0110446	0	0
GOLGA8C	NC_000015	20767700	20778481	0	0	0	0
LOC100287821	NC_000015	20787825	20793503	0	0	0	0

LOC646177	NC_000015	20798766	20804529	0	0	0	0
LOC401805	NC_000015	20832122	20834136	0	0	0	0
LOC100287326	NC_000015	20978413	20980200	0	0	0	0
LOC100287357	NC_000015	21008824	21031502	0	0	0	0
LOC100287399	NC_000015	21040701	21071662	0	0.026485	0	0
LOC100287908	NC_000015	21105987	21106959	0	0	0	0
NF1P1	NC_000015	21121930	21141131	0	0	0	0
OR11J2P	NC_000015	21165890	21167035	0	0	0	0
OR11J5P	NC_000015	21174579	21175538	0	0	0	0
LOC100287945	NC_000015	21228445	21231772	0	0	0	0
LOC283804	NC_000015	21258156	21263544	0	0	0	0
LOC100133063	NC_000015	21279846	21286776	0.082765	0	0.1104451	0
LOC100132293	NC_000015	21353663	21358299	0	0.0292323	0.0084317	0.0330223
LOC727914	NC_000015	21365634	21372686	0	0	0	0
BCL8	NC_000015	21901262	21968119	0	0	0	0
LOC646214	NC_000015	21932514	21940739	0	0	0	0
LOC100133065	NC_000015	21985081	21986868	0	0.0420702	0	0
CXADRP2	NC_000015	22014403	22016878	0	0	0	0
POTEB	NC_000015	22051853	22083137	0	0.022357	0	0
LOC100288042	NC_000015	22117163	22122407	0	0	0	0
LOC100288078	NC_000015	22130322	22152194	0	0	0	0
LOC100288112	NC_000015	22172092	22179001	0	0	0	0
LOC100288148	NC_000015	22185801	22186769	0	0	0	0
LOC100287507	NC_000015	22195587	22208058	0	0	0	0
OR11J1P	NC_000015	22268137	22269296	0	0	0	0
LOC727924	NC_000015	22278032	22371088	0	0	0	0
OR11H3P	NC_000015	22297401	22298547	0	0	0	0
OR11K1P	NC_000015	22318754	22319882	0	0	0	0
LOC100288919	NC_000015	22318955	22319614	0.0665882	0.1369194	0.1184775	0.7888226
OR4Q1P	NC_000015	22332368	22333348	0	0	0	0
OR4H8P	NC_000015	22344854	22345489	0	0	0	0
OR4H6P	NC_000015	22344855	22345491	0	0	0	0
OR4M2	NC_000015	22368576	22369517	0	0	0	0.0325105
OR4N4	NC_000015	22382473	22383423	0	0	0	0
OR4N3P	NC_000015	22413462	22414385	0	0	0	0
LOC650137	NC_000015	22413702	22414385	0	0	0	0.0447732
RPS8P10	NC_000015	22440169	22440869	0	0	0	0
IGHV1OR15-1	NC_000015	22448240	22448819	0	0	0	0
IGHV1OR15-3	NC_000015	22465806	22466493	0	0	0	0
LOC642131	NC_000015	22471454	22473374	0	0	0	0.1101614
IGHV1OR15-4	NC_000015	22482824	22490023	0	0	0	0
LOC646396	NC_000015	22545483	22570944	0.3313275	0.2189826	0.5263539	0.6761551
ABCB10P1	NC_000015	22692184	22692636	0	0	0	0
GOLGA8D	NC_000015	22704899	22715702	0	0	0	0
LOC283767	NC_000015	22736246	22746002	0.0229975	0	0.0102296	0.0160256
LOC100129539	NC_000015	22746020	22749325	0	0	0	0
LOC729894	NC_000015	22777497	22789974	0	0	0	0
TUBGCP5	NC_000015	22833395	22873891	2.7121973	3.5101336	2.9521879	2.0011416
CYFIP1	NC_000015	22892684	23003603	7.0667931	6.8588263	7.3193213	10.741472
NIPA2	NC_000015	23005347	23034408	6.8230912	8.6498964	4.9898839	4.5877736
NIPA1	NC_000015	23043279	23086843	0.9671412	1.8789273	2.6761272	2.2867567
LOC729900	NC_000015	23096869	23105415	0	0	0	0
LOC100288671	NC_000015	23108596	23114275	0.3497207	0.4793995	0.1037071	0
LOC100132817	NC_000015	23116383	23116940	0	0	0	0
LOC283683	NC_000015	23128188	23157800	0	0	0	0
LOC100133165	NC_000015	23157046	23159115	0	0.0746833	0	0
LOC100288760	NC_000015	23158203	23158662	0	0	0	0
WHAMML1	NC_000015	23187728	23208357	0	0	0	0
GOLGA9P	NC_000015	23255242	23262743	0	0	0	0
HERC2P2	NC_000015	23282265	23378259	0	0	0	0
LOC100132101	NC_000015	23377990	23403848	0	0	0	0
LOC440233	NC_000015	23405591	23414193	0	0	0	0
GOLGA8E	NC_000015	23435096	23448423	0.0167645	0.0258536	0.0372855	0.1226631
LOC100289052	NC_000015	23455218	23465823	0	0	0	0
LOC646507	NC_000015	23466152	23471916	0	0	0	0
LOC441711	NC_000015	23499526	23500241	0	0	0	0

LOC100133069	NC_000015	23566683	23569654	0	0	0	0
LOC440243	NC_000015	23570874	23586988	0.0162952	0	0	0.0227103
LOC653061	NC_000015	23599895	23610649	0.0174884	0	0.0155581	0.0365597
HERC2P6	NC_000015	23678069	23678654	0	0	0	0
LOC283685	NC_000015	23684690	23692328	0.0176712	0	0.0157208	0.012314
MKRN3	NC_000015	23810454	23813167	0.3724426	0.2330758	0.6626706	0.8237348
MAGEL2	NC_000015	23888696	23891175	0.0177211	0.0182191	0.0157651	0.0123487
NDN	NC_000015	23930554	23932450	7.9695265	0	12.427956	6.231525
PWRN2	NC_000015	24409926	24415053	0	0	0	0
PWRN1	NC_000015	24803304	24832927	0	0	0	0
C15orf2	NC_000015	24920541	24928593	0.0109147	0.0056108	0.0242752	0.0266204
SNRPN	NC_000015	25068794	25664609	11.229744	9.7080803	12.03153	8.5632817
RPL5P1	NC_000015	25154011	25155047	0	0	0	0
RPS27P2	NC_000015	25155861	25156404	0	0	0	0
SNURF	NC_000015	25200070	25223729	0.1232769	0	0.1096706	0
PAR-SN	NC_000015	25227141	25228937	0	0	0	0
SNORD107	NC_000015	25227141	25227215	0	0	0	0
PAR5	NC_000015	25230007	25233379	0	0	0	0
SNORD64	NC_000015	25230247	25230313	0	0	0	0
SNORD108	NC_000015	25232072	25232140	0	0	0	0
SNORD109A	NC_000015	25287121	25287187	0	0	0	0
SNORD116@	NC_000015	25296623	25351750	0	0	0	0
SNORD116-1	NC_000015	25296623	25296719	0	0	0	0
SNORD116-2	NC_000015	25299356	25299452	0	0	0	0
SNORD116-3	NC_000015	25302006	25302102	0	0	0	0
SNORD116-4	NC_000015	25304684	25304781	0	0	0	0
SNORD116-5	NC_000015	25307479	25307575	0	0	0	0
SNORD116-6	NC_000015	25310172	25310269	0	0	0	0
SNORD116-7	NC_000015	25312934	25313030	0	0	0	0
SNORD116-8	NC_000015	25315578	25315674	0	0	0	0
SNORD116-9	NC_000015	25318253	25318349	0	0	0	0
SNORD116-10	NC_000015	25319260	25319363	0	0	0	0
SNORD116-11	NC_000015	25321075	25321168	0	0	0	0
SNORD116-12	NC_000015	25322197	25322290	0	0	0	0
SNORD116-13	NC_000015	25324204	25324297	0	0	0	0
SNORD116-14	NC_000015	25325288	25325381	0	0	0	0
SNORD116-15	NC_000015	25326433	25326526	0	0	0	0
SNORD116-16	NC_000015	25327914	25328007	0	0	0	0
SNORD116-17	NC_000015	25328734	25328827	0	0	0	0
SNORD116-18	NC_000015	25330531	25330624	0	0	0	0
SNORD116-19	NC_000015	25331673	25331766	0	0	0	0
SNORD116-20	NC_000015	25332808	25332901	0	0	0	0
SNORD116-21	NC_000015	25333950	25334043	0	0	0	0
SNORD116-22	NC_000015	25335069	25335162	0	0	0	0
SNORD116-23	NC_000015	25336932	25337025	0	0	0	0
C15orf49	NC_000015	25337108	25337524	0	0	0	0
SNORD116-24	NC_000015	25339183	25339276	0	0	0	0
SNORD116-25	NC_000015	25342809	25342902	0	0	0	0
SNORD116-26	NC_000015	25344645	25344742	0	0	0	0
SNORD116-27	NC_000015	25346721	25346814	0	0	0	0
SNORD116-28	NC_000015	25349788	25349880	0	0	0	0
SNORD116-29	NC_000015	25351667	25351751	0	0	0	0
IPW	NC_000015	25361692	25367623	0	0	0	0
PAR1	NC_000015	25380789	25383200	0	0	0	0
LOC100289059	NC_000015	25414482	25415598	0	0	0	0
SNORD115@	NC_000015	25415870	25515005	0	0	0	0
SNORD115-1	NC_000015	25415870	25415951	0	0	0	0
SNORD115-2	NC_000015	25417782	25417863	0	0	0	0
SNORD115-3	NC_000015	25420074	25420155	0	0	0	0
SNORD115-4	NC_000015	25421979	25422060	0	0	0	0
SNORD115-5	NC_000015	25423885	25423966	0	0	0	0
SNORD115-6	NC_000015	25425644	25425725	0	0	0	0
SNORD115-7	NC_000015	25427532	25427613	0	0	0	0
SNORD115-8	NC_000015	25429453	25429534	0	0	0	0
SNORD115-9	NC_000015	25430778	25430859	0	0	0	0
SNORD115-10	NC_000015	25432683	25432763	0	0	0	0

SNORD115-11	NC_000015	25434561	25434642	0	0	0	0
SNORD115-12	NC_000015	25436563	25436644	0	0	0	0
SNORD115-13	NC_000015	25438468	25438549	0	0	0	0
SNORD115-14	NC_000015	25440068	25440148	0	0	0	0
SNORD115-15	NC_000015	25442723	25442803	0	0	0	0
SNORD115-16	NC_000015	25444595	25444676	0	0	0	0
SNORD115-17	NC_000015	25446470	25446551	0	0	0	0
SNORD115-18	NC_000015	25448374	25448455	0	0	0	0
SNORD115-19	NC_000015	25449504	25449585	0	0	0	0
SNORD115-20	NC_000015	25451409	25451490	0	0	0	0
SNORD115-21	NC_000015	25453230	25453310	0	0	0	0
SNORD115-22	NC_000015	25455065	25455146	0	0	0	0
PAR4	NC_000015	25456839	25457180	0	0	0	0
SNORD115-23	NC_000015	25456943	25457024	0	0	0	0
HBII-52-24	NC_000015	25458806	25458876	0	0	0	0
SNORD115-25	NC_000015	25460688	25460769	0	0	0	0
SNORD115-26	NC_000015	25463764	25463845	0	0	0	0
HBII-52-27	NC_000015	25465650	25465725	0	0	0	0
HBII-52-28	NC_000015	25467501	25467574	0	0	0	0
SNORD115-29	NC_000015	25468393	25468474	0	0	0	0
SNORD115-30	NC_000015	25470350	25470431	0	0	0	0
SNORD115-31	NC_000015	25472256	25472337	0	0	0	0
SNORD115-32	NC_000015	25474114	25474195	0	0	0	0
SNORD115-33	NC_000015	25475985	25476066	0	0	0	0
SNORD115-34	NC_000015	25477534	25477615	0	0	0	0
SNORD115-35	NC_000015	25479394	25479475	0	0	0	0
SNORD115-36	NC_000015	25481232	25481313	0	0	0	0
SNORD115-37	NC_000015	25483133	25483214	0	0	0	0
SNORD115-38	NC_000015	25484985	25485066	0	0	0	0
SNORD115-39	NC_000015	25486893	25486974	0	0	0	0
SNORD115-40	NC_000015	25488761	25488842	0	0	0	0
SNORD115-41	NC_000015	25490625	25490706	0	0	0	0
SNORD115-42	NC_000015	25492492	25492573	0	0	0	0
SNORD115-43	NC_000015	25494345	25494426	0	0	0	0
SNORD115-44	NC_000015	25496006	25496087	0	0	0	0
HBII-52-45	NC_000015	25509674	25509726	0	0	0	0
HBII-52-46	NC_000015	25513664	25513696	0	0	0	0
SNORD115-48	NC_000015	25514930	25515005	0	0	0	0
SNORD109B	NC_000015	25523490	25523556	0	0	0	0
UBE3A	NC_000015	25582396	25684128	1.336121	2.1474505	0.9554341	1.1726671
ATP10A	NC_000015	25923859	26108349	4.5249469	5.5756404	1.7102851	2.0007084
LOC100128714	NC_000015	26147513	26260858	0.0759037	0	0	0.0528927
MRPS18CP5	NC_000015	26173293	26173723	0	0	0	0
LOC100289089	NC_000015	26244635	26246597	0.1024434	0	0	0
GABRB3	NC_000015	26788693	27018927	0.01478	0.022793	0.0262973	0.0154489
RPL9P26	NC_000015	27096261	27096757	0	0	0	0
GABRA5	NC_000015	27112058	27194003	0.0373868	0	0.0166302	0.0130263
LOC100129124	NC_000015	27210062	27210664	0	0	0	0
GABRG3	NC_000015	27216517	27778134	0.0262065	0.026943	0	0.0182617
OCA2	NC_000015	28000021	28344458	0.0279925	0.0143896	0.0249029	0
RPL5P32	NC_000015	28140806	28141786	0	0	0	0
HERC2	NC_000015	28356186	28567295	2.0148433	2.7698186	2.0627319	3.5949381
RPL41P2	NC_000015	28560799	28561217	0	0	0	0
HERC2P7	NC_000015	28573730	28580222	0	0	0	0
LOC645202	NC_000015	28594195	28603175	0	0	0	0
LOC728243	NC_000015	28607000	28615298	0	0	0	0
GOLGA8F	NC_000015	28623865	28635226	0.015679	0	0.0557939	0.1529605
LOC728248	NC_000015	28644646	28650180	0	0	0	0
LOC100289226	NC_000015	28699989	28729959	0	0	0	0
LOC100132289	NC_000015	28751770	28757334	0	0	0	0
GOLGA8G	NC_000015	28764757	28778117	0.0836154	0.0257896	0.0892638	0.2214127
LOC100132202	NC_000015	28786615	28807727	0	0	0	0
LOC728278	NC_000015	28806898	28828666	0	0	0	0
LOC440248	NC_000015	28874311	28910200	0	0	0	0
LOC653720	NC_000015	28946732	28957469	0.0545263	0	0	0.101323
WHAMML2	NC_000015	28982729	29003508	0	0	0	0

LOC100289656	NC_000015	29033389	29035455	0	0	0	0
LOC100289368	NC_000015	29033841	29034299	0	0	0.2555396	0.2001626
LOC100289401	NC_000015	29034702	29035459	0	0.2204068	0	0
LOC646278	NC_000015	29041175	29078587	0	0	0	0
LOC100129687	NC_000015	29075763	29076328	0	0	0	0
LOC728310	NC_000015	29087381	29093728	0	0	0	0
LOC100289501	NC_000015	29093806	29105286	0	0	0	0
APBA2	NC_000015	29213840	29410518	0.3019252	0.4097423	0.010744	0.0168315
KIAA0574	NC_000015	29412455	29862927	0.1120414	0.0959919	0.0581438	0.0845811
NDNL2	NC_000015	29560358	29562017	0.1323742	0.3538459	0.0235528	0.0184487
LOC100130736	NC_000015	29905921	29906517	0	0	0.0654901	0.051298
LOC100130111	NC_000015	29928199	29971379	0	0	0	0
TJP1	NC_000015	29992356	30114706	2.760182	3.222431	2.7556554	4.4665733
HMGN2L5	NC_000015	30022816	30024010	0	0	0	0
LOC727808	NC_000015	30114824	30116302	0	0	0	0
LOC283698	NC_000015	30336543	30338383	0	0	0	0
SYNGR2P1	NC_000015	30336926	30338041	0	0	0	0
LOC100128435	NC_000015	30337759	30365267	0	0.0436977	0	0.0888536
LOC653073	NC_000015	30375158	30386368	0.0312354	0.0160566	0.0277879	0.0761813
LOC100289637	NC_000015	30375158	30376663	0	0	0	0
DNM1P28	NC_000015	30390454	30394304	0	0	0	0
LOC100288176	NC_000015	30391057	30396051	0	0	0	0
FAM7A3	NC_000015	30395935	30423951	0	0	0	0
LOC100288381	NC_000015	30427254	30428759	0	0	0.0519224	0
LOC653075	NC_000015	30427254	30439395	0	0	0	0
DNM1P30	NC_000015	30443100	30446313	0	0	0	0
DKFZP434L187	NC_000015	30488239	30506743	0	0	0	0
LOC728424	NC_000015	30515159	30518447	0	0	0	0
LOC727880	NC_000015	30636502	30640439	0	0	0	0
CHRFAM7A	NC_000015	30653443	30685864	0.4920726	0.0948567	0.2872809	0.2464563
DNM1P29	NC_000015	30685549	30690597	0	0	0	0
LOC643699	NC_000015	30693919	30706463	0.2330347	0.1960234	0.2638544	0.0959565
LOC100288482	NC_000015	30705587	30706266	0	0	0	0
LOC100288203	NC_000015	30762982	30766057	0.0825319	0.0424257	0.110134	0.0862673
LOC642397	NC_000015	30770888	30775415	0.1615744	0.3322309	0.2874821	0.0562957
LOC100132816	NC_000015	30799907	30810600	0	0	0.0198666	0
LOC100132024	NC_000015	30814497	30815776	0.0586759	0.060325	0	0
LOC727909	NC_000015	30844183	30856728	0.0105848	0	0.0470828	0.0737593
LOC100288516	NC_000015	30844380	30845059	0	0	0	0
LOC100132726	NC_000015	30861097	30863327	0	0	0	0
LOC100288380	NC_000015	30865111	30871612	0	0	0	0
LOC728498	NC_000015	30896233	30908390	0.3029305	0.0958291	0.1865737	0.138023
LOC100288601	NC_000015	30896233	30897736	0	0	0.0259957	0.0203623
ARHGAP11B	NC_000015	30918879	30931013	0.864272	0.9329906	0.3075521	1.1141794
LOC100288637	NC_000015	31042609	31048274	0	0.1476582	0.1277698	0.3002439
LOC100288693	NC_000015	31061149	31065212	0.010814	0.0444719	0.2020297	0.0979634
LOC390561	NC_000015	31073661	31120007	0.0973845	0.200243	0.0866359	0.2811401
LOC388104	NC_000015	31174022	31175593	0	0	0	0
MTMR15	NC_000015	31196129	31235308	2.0542503	2.3082317	2.5472044	3.0213168
MTMR10	NC_000015	31231144	31283807	4.1901862	2.7960702	0.5989548	1.6050128
TRPM1	NC_000015	31293537	31393924	0.0323863	0.0249724	0.0072029	0.0282101
LOC283710	NC_000015	31514968	31523050	0	0	0.0755509	0
KLF13	NC_000015	31619083	31670102	8.5699052	8.1994585	9.0844344	10.89435
LOC283711	NC_000015	31694237	31712863	0	0	0	0
LOC400347	NC_000015	31745344	31746341	0	0	0	0
OTUD7A	NC_000015	31775329	31947542	0.0433415	0.0742659	0.0771155	0.0402694
DEPDC1P1	NC_000015	32056304	32060522	0	0	0	0
LOC100289030	NC_000015	32201255	32202510	0	0	0	0.0803803
CHRNA7	NC_000015	32322726	32461249	0.1417032	0.0208123	0.2701352	0.3103396
LOC100289152	NC_000015	32474752	32478669	0	0	0	0
LOC644110	NC_000015	32604646	32607933	0	0	0	0
LOC100132297	NC_000015	32634385	32637212	0	0	0	0
DNM1P31	NC_000015	32679619	32680237	0	0	0	0
LOC653125	NC_000015	32684890	32695493	0.0393273	0	0.0699733	0.0822144
LOC100289428	NC_000015	32693988	32695493	0	0	0	0
FAM7A1	NC_000015	32698812	32727250	0	0	0	0

DNM1P32	NC_000015	32726949	32731944	0	0	0	0
LOC728047	NC_000015	32735283	32747835	0.0317545	0.0108823	0.0376662	0.0811353
LOC100289543	NC_000015	32746959	32747638	0	0	0	0
LOC100129979	NC_000015	32773589	32777573	0.0374028	0.038454	0.0332745	0
LOC400352	NC_000015	32781471	32792215	0	0	0	0
LOC644231	NC_000015	32815353	32828785	1.5669768	2.0606029	2.2369254	2.3108324
LOC100288615	NC_000015	32826071	32829127	0.0478739	0	0	0.0333604
LOC728080	NC_000015	32885657	32898204	0.0211646	0	0.1412144	0.0958641
LOC100286908	NC_000015	32885657	32887159	0	0	0	0
ARHGAP11A	NC_000015	32907691	32931868	2.2863242	3.8100931	2.193505	3.7174708
SCG5	NC_000015	32933870	32989298	1.4484546	0.4358528	0.1257156	0.1969446
GREM1	NC_000015	33010205	33026866	235.94268	229.19592	93.100251	162.90302
LOC100131315	NC_000015	33010302	33011060	0	0.078037	0	0
FMN1	NC_000015	33066345	33360085	0.30414	0.7576668	0.3434175	0.7499305
RYR3	NC_000015	33603177	34158303	0.0395447	0.0377521	0.0251286	0.0354295
AVEN	NC_000015	34158428	34331303	0.2296446	0.4721975	0.2042982	0.320051
CHRM5	NC_000015	34261089	34357287	0.0433842	0.0297357	0.0257305	0.0100773
C15orf24	NC_000015	34376223	34394053	8.8396823	8.445095	5.193225	3.7482062
RPL32P2	NC_000015	34378059	34378567	0	0	0	0
PGBD4	NC_000015	34394274	34396591	0.6067055	0.6042647	0.2192702	0.4227766
C15orf29	NC_000015	34432875	34502297	2.9512681	3.0671944	1.7265713	2.3583392
TMEM85	NC_000015	34517245	34522357	23.5041	43.861183	13.808424	29.017922
SLC12A6	NC_000015	34522197	34630265	2.1563509	2.6472735	1.0086653	0.9525279
NOP10	NC_000015	34633917	34635362	37.047949	43.663213	20.827675	20.435666
C15orf55	NC_000015	34638066	34649933	0	0.0238121	0.0309072	0.0726282
LOC100287947	NC_000015	34638080	34640627	0	0	0	0
LPCAT4	NC_000015	34651089	34659395	7.4398736	6.0860243	3.4015702	3.2904088
GOLGA8A	NC_000015	34671269	34699888	0.378212	0.2937915	0.5458256	1.2006311
LOC644390	NC_000015	34780835	34784830	0	0	0	0
LOC145989	NC_000015	34786507	34789279	0	0	0	0
LOC100129838	NC_000015	34805703	34806974	0	0	0	0
DNM1P5	NC_000015	34812808	34815624	0	0	0	0
GOLGA8B	NC_000015	34817855	34875061	0.7174507	0.3328262	0.560427	1.2010952
GJD2	NC_000015	35044679	35046689	0	0	0	0
ACTC1	NC_000015	35080297	35087927	0.4641162	0.5872741	0.2858474	0.2819512
LOC100132824	NC_000015	35141264	35142596	0	0	0	0
AQR	NC_000015	35148552	35261995	2.6299729	3.6555863	1.8702182	3.110716
RPL36AP8	NC_000015	35235252	35235648	0	0	0	0
ZNF770	NC_000015	35270544	35280454	1.3827872	1.4049253	1.0275503	1.3830225
NANOGP8	NC_000015	35375427	35377509	0	0	0	0
LOC441722	NC_000015	35384514	35385413	0	0.0502038	0.0868835	0
LOC390572	NC_000015	35413622	35414285	0	0	0	0
LOC723972	NC_000015	35529527	35530264	0	0	0	0
LOC100131623	NC_000015	35574797	35575180	0	0	0	0
LOC100128974	NC_000015	35590248	35591811	0	0	0	0
ATPBD4	NC_000015	35663170	35838404	0.2602412	0.4347774	0.4340958	0.4306978
LOC100130660	NC_000015	35692892	35693969	0	0	0	0
LOC100288892	NC_000015	36864186	36864413	0	0	0	0
C15orf41	NC_000015	36871812	37102449	2.9686687	4.0950334	1.79164	2.4221306
LOC751603	NC_000015	36905610	36907675	0	0	0	0
CSNK1A1P	NC_000015	37091301	37110707	0	0	0	0
LOC145845	NC_000015	37156644	37178734	0	0	0	0
MEIS2	NC_000015	37183232	37393500	1.4723962	4.8095988	0.7295559	1.2662959
RPS15P8	NC_000015	37782879	37783340	0	0	0	0
TMCO5A	NC_000015	38227457	38243623	0	0.0390185	0.033763	0.0793391
LOC728288	NC_000015	38363826	38365463	0.1341521	0.3310139	0.1909527	0.1495721
SPRED1	NC_000015	38545052	38649450	2.3503672	2.9893912	2.3065139	2.1485959
FAM98B	NC_000015	38746328	38777063	1.2086407	2.0622032	1.4641569	0.609272
RASGRP1	NC_000015	38780302	38857007	0.2448857	0.3326937	0.1089285	0.1584571
C15orf53	NC_000015	38988799	38992239	0	0.0221162	0	0.0749508
LOC100289563	NC_000015	39143191	39145633	0.0179895	0	0	0.0250715
C15orf54	NC_000015	39542885	39547048	0.0285378	0.0440098	0.025388	0.0596589
THBS1	NC_000015	39873280	39889668	169.52328	351.69699	1.2424788	4.2684416
FSIP1	NC_000015	39892232	40075039	0.0937396	0.1284988	0.0694944	0.1850775
GPR176	NC_000015	40092931	40213093	12.532113	11.23167	9.5938984	11.777963
LOC100286890	NC_000015	40099214	40213017	0.1090527	0.0373725	0.0323388	0.0759923

EIF2AK4	NC_000015	40226347	40327797	8.0369771	9.5990137	7.6109739	8.2118233
H3F3AP1	NC_000015	40243057	40244035	0	0	0	0
MGC10997	NC_000015	40305502	40307606	0	0	0	0
SRP14	NC_000015	40327891	40331389	10.147904	10.352858	9.4792499	9.7096639
BMF	NC_000015	40380091	40401075	0.1566946	0.1516222	0.1803998	0.1413061
BUB1B	NC_000015	40453210	40513337	0.7399088	1.0987946	0.8254163	2.537069
PAK6	NC_000015	40509629	40569688	0.1167425	0.1091123	0.0849742	0.0665597
C15orf56	NC_000015	40542868	40545110	0.0206815	0	0	0
LOC100131244	NC_000015	40573645	40574787	0	0.0395305	0	0.0267934
PLCB2	NC_000015	40580098	40600174	0.0187933	0.0289823	0.0585168	0.0851237
RPLP0P10	NC_000015	40604485	40605563	0	0	0	0
C15orf52	NC_000015	40623653	40633168	2.367776	0.5937372	0.777988	1.8396773
LOC644844	NC_000015	40643234	40648635	0	0	0	0.0702405
DISP2	NC_000015	40650434	40663256	0.2969475	0.2155011	0.108777	0.1156345
C15orf23	NC_000015	40674922	40686489	4.5180425	7.0008714	4.9424862	4.5794211
IVD	NC_000015	40697686	40711063	8.1312162	6.8509129	4.1638202	6.0531122
BAHD1	NC_000015	40733411	40760441	2.5731952	1.8967844	2.2891858	2.7810042
CHST14	NC_000015	40763213	40765354	6.7912534	9.9141918	3.4132795	2.2732753
RPL9P27	NC_000015	40772850	40773549	0	0	0	0
MRPL42P5	NC_000015	40824083	40824749	0	0	0	0
C15orf57	NC_000015	40845298	40857252	3.4802935	4.3093996	1.5141832	1.4869883
RPUSD2	NC_000015	40861537	40866661	0.7886306	1.3021861	0.7441081	0.5995082
CASC5	NC_000015	40886447	40954881	3.2024736	5.773598	2.7676091	4.6266082
RAD51	NC_000015	40987359	41024356	2.5351028	3.2430183	2.1003536	2.2655128
FAM82A2	NC_000015	41028086	41047458	2.6942941	2.9305983	0.9726627	1.9591215
GCHFR	NC_000015	41056285	41059911	2.8353698	2.7249457	1.5902236	1.7180857
DNAJC17	NC_000015	41060071	41099655	1.106451	2.2295939	1.2599417	0.7401783
C15orf62	NC_000015	41062178	41064647	0	0	0.015829	0
ZFYVE19	NC_000015	41099274	41106767	6.1484988	6.3411814	3.4745747	3.6108523
PPP1R14D	NC_000015	41107643	41120907	0.1510249	0	0.0895706	0.03508
SPINT1	NC_000015	41136246	41149853	1.0293552	0.6457334	0.3104213	0.6443504
RHOV	NC_000015	41164902	41166439	0.1129775	0.1161527	0.0670053	0.0262424
VPS18	NC_000015	41186628	41196173	9.5907566	8.7118703	3.0414794	3.7032908
LOC100288995	NC_000015	41221436	41230571	0	0.0196621	0	0
DLL4	NC_000015	41221547	41231237	0	0.040068	0.0346712	0.0181052
LOC100128012	NC_000015	41240685	41241508	0	0	0	0
CHAC1	NC_000015	41245636	41248717	4.0849318	4.6921225	1.1278144	0.8048846
INO80	NC_000015	41271078	41408340	1.2844995	1.1827356	0.9229713	1.7950989
CYCSP2	NC_000015	41401063	41401375	0	0	0	0
LOC644949	NC_000015	41408095	41410140	0	0	0	0
FAM92A2	NC_000015	41446228	41456572	0	0	0	0
EXD1	NC_000015	41474926	41522895	0	0.0307475	0	0.0208403
CHP	NC_000015	41523437	41574085	8.204577	9.5262838	6.0643593	13.311867
LOC729082	NC_000015	41576201	41591795	0	0	0	0
OIP5	NC_000015	41601466	41624819	1.2444888	2.7417113	2.1509988	1.6848639
NUSAP1	NC_000015	41624926	41673248	12.603336	18.894877	18.530954	20.538916
NDUFAF1	NC_000015	41679551	41694642	2.6242252	3.1631482	0.7781945	1.3031863
RTF1	NC_000015	41709302	41775761	0.603949	0.6479197	0.4438481	1.378455
ITPKA	NC_000015	41786122	41795747	0.3965999	0.8154929	0.5733428	0.604552
LTK	NC_000015	41795840	41806085	0.0562178	0.0288989	0.0250064	0.0587622
RPAP1	NC_000015	41809374	41836464	2.972526	2.8529759	1.5900123	2.1631442
TCEB1P2	NC_000015	41849175	41849874	0	0	0	0
TYRO3	NC_000015	41851232	41871526	5.2306075	7.8947955	5.1186228	6.5840774
MGA	NC_000015	41952610	42062141	3.0935748	4.2147451	2.8547734	5.3013777
MAPKBP1	NC_000015	42066632	42120053	0.8486555	1.3025284	0.6363466	1.047582
JMJD7	NC_000015	42120283	42129785	0.5254008	0.8896872	0.3024425	0.4307297
JMJD7-PLA2G4B	NC_000015	42120283	42140346	0.5090529	0.5501988	0.1741798	0.3638239
PLA2G4B	NC_000015	42131011	42140346	0.0642753	0.1652044	0.0571811	0.0335922
SPTBN5	NC_000015	42140344	42186275	0.0899733	0.1194818	0.0333512	0.0783713
LOC100289090	NC_000015	42186434	42187530	0	0	0	0
EHD4	NC_000015	42191638	42264742	4.5153338	8.7041958	3.5555579	2.5724473
PLA2G4E	NC_000015	42275952	42302445	0.0174398	0.0358598	0	0.0486109
PLA2G4D	NC_000015	42359881	42386752	0	0.025214	0.0109089	0.0256347
PLA2G4F	NC_000015	42433396	42448810	0.0258595	0.0132931	0	0.0450498
VPS39	NC_000015	42450899	42500502	8.5495318	9.3508696	5.0247492	7.618192
TMEM87A	NC_000015	42502726	42565755	4.4597713	8.2465273	0.6548361	0.4978428

GANC	NC_000015	42566366	42645864	1.9837078	1.931083	0.980423	0.6210453
CAPN3	NC_000015	42640301	42704515	0.2741789	0.2727917	0.0472098	0.0739582
ZFP106	NC_000015	42705021	42749730	1.5545742	2.1511792	0.8447466	2.3481025
SNAP23	NC_000015	42787835	42825256	6.3626829	6.0910438	5.423156	8.1639793
LRRC57	NC_000015	42834720	42841002	2.6569117	3.2180311	2.7522096	3.4492617
HAUS2	NC_000015	42841011	42862192	2.9736893	3.9389484	2.5067623	3.0733532
LOC645094	NC_000015	42864125	42864631	0	0	0	0
STARD9	NC_000015	42867914	43013179	0.0576295	0.0622116	0.1127913	0.3574107
LOC100190923	NC_000015	42870830	42873908	0	0	0	0
CDAN1	NC_000015	43015760	43029417	0.8176415	1.2513796	0.6116744	0.6992573
LOC100289126	NC_000015	43023015	43028963	0	0	0	0
TTBK2	NC_000015	43036536	43213007	1.2721651	1.227679	1.2359024	1.6043934
LOC729154	NC_000015	43211487	43212988	0	0	0	0
FDPSL4	NC_000015	43231451	43232586	0	0	0	0
UBR1	NC_000015	43235095	43398286	1.2875902	1.4987267	0.6459071	0.9407229
RPS3AP47	NC_000015	43407853	43408715	0	0	0	0
LOC100131250	NC_000015	43419892	43424887	0	0	0	0
TMEM62	NC_000015	43425722	43477342	4.7297122	3.2635177	4.5041977	1.6036863
LOC100190987	NC_000015	43432291	43433447	0	0	0	0
LOC100289266	NC_000015	43476848	43478383	0.1309932	0	0	0.0456407
CCNDBP1	NC_000015	43477540	43487396	7.0970785	6.7699881	2.0828892	1.6655039
EPB42	NC_000015	43489425	43513323	0.0172956	0	0.0307734	0.0120523
TGM5	NC_000015	43525375	43559055	1.4682933	1.3234498	1.3599153	0.9390695
ATP5HP1	NC_000015	43560027	43560623	0	0	0	0
TGM7	NC_000015	43568479	43594453	0.0380175	0	0.0169107	0.0264921
LCMT2	NC_000015	43619974	43622891	4.5936294	6.0389057	1.3800717	1.6162547
ADAL	NC_000015	43622925	43641610	1.7195909	1.4201326	0.8275944	1.1000598
ZSCAN29	NC_000015	43650370	43662258	0.3840669	0.6124378	0.5438934	0.7592042
TUBGCP4	NC_000015	43663313	43698258	2.3347943	2.707784	2.3304024	3.9186352
TP53BP1	NC_000015	43699412	43802707	0.8850494	1.450191	0.9349953	1.7104833
MAP1A	NC_000015	43809806	43823818	4.2643686	6.7588213	4.2579247	4.9625715
HISPPD2A	NC_000015	43825657	43882430	1.0785774	1.366591	1.0541342	2.7840798
CKMT1B	NC_000015	43885252	43891604	0	0	0.0219773	0.0172147
STRC	NC_000015	43891761	43910998	0	0	0.0212679	0.016659
CATSPER2	NC_000015	43922772	43941039	0.2131022	0.1853851	0.1020824	0.2056128
HISPPD2B	NC_000015	43956515	43977026	0	0	0	0
CKMT1A	NC_000015	43985084	43991420	0	0	0.0219773	0
STRCP	NC_000015	43991477	44010560	0	0	0	0
CATSPER2P1	NC_000015	44028132	44038496	0	0	0	0
PDIA3	NC_000015	44038590	44064804	36.508403	37.20899	27.780523	30.534628
ELL3	NC_000015	44064798	44069502	0.4575774	0.4918212	0.2960535	0.6522099
LOC100289302	NC_000015	44067956	44069331	0	0	0	0.0471878
SERF2	NC_000015	44084477	44088287	1.4400924	1.2018715	1.4921584	1.345889
SERINC4	NC_000015	44087165	44092255	0.2359637	0.0909733	0.1836798	0.2671969
HYPK	NC_000015	44092621	44094787	4.0397189	4.6556656	3.3330023	3.9274306
MFAP1	NC_000015	44096733	44116951	0.4686675	0.4818395	0.4927468	0.7867758
WDR76	NC_000015	44119208	44159210	0.3308306	0.6086512	0.4492193	1.031345
FRMD5	NC_000015	44165730	44487429	1.1560168	1.6921114	0.4183422	0.9557474
LOC728758	NC_000015	44168004	44170369	0	0	0	0
ACTBP7	NC_000015	44280676	44282463	0	0	0	0
LOC100289332	NC_000015	44580814	44621400	0.0429601	0.2208377	0.3439669	0
CASC4	NC_000015	44580929	44707955	6.8901419	5.6579064	6.1395319	8.05633
LOC100240708	NC_000015	44646806	44648099	0	0	0	0
CTDSPL2	NC_000015	44719579	44819430	3.3626032	4.3932169	3.7453463	3.0656288
LOC645204	NC_000015	44719627	44743244	0	0	0	0
LOC645212	NC_000015	44827761	44829290	0.4896739	0.4405067	0.1089069	0.1706121
EIF3J	NC_000015	44829266	44855001	1.9757806	1.6603752	1.2074698	2.5979666
SPG11	NC_000015	44854894	44955876	4.9546158	4.3338471	4.2019341	3.731768
PATL2	NC_000015	44957930	44969086	0.6307281	0.0694773	0.1402783	0.031394
B2M	NC_000015	45003685	45010357	854.29663	434.30084	1272.394	290.02101
TRIM69	NC_000015	45028560	45060025	0.8553838	0.1663776	0.514171	0.2094284
SORD2	NC_000015	45117945	45139761	0	0	0	0
LOC100129830	NC_000015	45212155	45213235	0	0	0	0
C15orf43	NC_000015	45248900	45271421	0.172346	0.1417518	0.0613295	0.2642146
LOC100187725	NC_000015	45262013	45263513	0	0	0	0
SORD	NC_000015	45315341	45367039	3.9842221	3.0945085	2.569357	4.5464487

DUOX2	NC_000015	45384852	45406359	0	0.0213599	0.012322	0.0482586
DUOXA2	NC_000015	45406524	45410304	0.7324705	0.2823963	0.2443598	0.3190091
DUOXA1	NC_000015	45409685	45422057	0.0233767	0.0480674	0.0415932	0
DUOX1	NC_000015	45422192	45457774	0.1625705	0.1034674	0.1033052	0.1348638
LOC100289438	NC_000015	45437089	45444698	0.0423393	0.0435293	0.0376662	0.0590075
SHF	NC_000015	45459412	45493373	0.0878965	0.0903668	0.0469171	0.0857497
SLC28A2	NC_000015	45544434	45568133	0.0357593	0.0183822	0	0.0622963
LOC645263	NC_000015	45613312	45613584	0	0	0	0
LOC100129116	NC_000015	45622313	45622803	0	0	0	0
GATM	NC_000015	45653322	45670980	0.1359574	0.0174723	0.0302379	0.0473703
SPATA5L1	NC_000015	45694586	45713613	1.418815	1.4226739	0.654483	0.8300087
C15orf48	NC_000015	45722763	45725647	2.3004527	0.8376421	11.810279	0.901714
RNU7-5P	NC_000015	45738562	45738821	0	0	0	0
SLC30A4	NC_000015	45774676	45815002	2.3270932	1.6758015	1.4318446	0.8929577
C15orf21	NC_000015	45803334	45848928	0	0	0	0
LOC100289502	NC_000015	45858972	45859274	0	0	0	0
PLDN	NC_000015	45879417	45901909	7.5384432	8.1171907	7.4405415	8.5323817
SQRDL	NC_000015	45927256	45983479	45.157334	18.700307	30.048069	29.837277
POM121B	NC_000015	47029544	47039476	0	0	0	0
LOC100289530	NC_000015	47119722	47120158	0	0	0	0
SEMA6D	NC_000015	48010686	48066420	0.7635017	0.0481993	0.9950158	0.5600404
RPL7AP62	NC_000015	48022280	48023157	0	0	0	0
SLC24A5	NC_000015	48413169	48434589	0.0543577	0	0.0725372	0.0757573
MYEF2	NC_000015	48431629	48470558	0.4392383	0.4683084	0.1519617	0.0170044
CTXN2	NC_000015	48483867	48495951	0	0	0	0.0227272
SLC12A1	NC_000015	48498498	48596275	0.0279984	0.0383805	0.0249082	0.0260139
LOC100130218	NC_000015	48622542	48666278	0	0	0	0
DUT	NC_000015	48623621	48635570	10.625062	16.677222	9.5393882	10.09012
FBN1	NC_000015	48700505	48937918	11.419887	29.61833	3.9951753	11.316401
LOC645405	NC_000015	49017335	49018374	0	0	0	0
CEP152	NC_000015	49030348	49103184	0.6294332	1.2410587	0.6903631	1.3458844
SHC4	NC_000015	49115934	49255641	0.1350472	0.1487601	0.0600709	0.0470531
EID1	NC_000015	49170290	49172380	0.2101781	0.1296511	0.1121881	0.2636288
KRT8P24	NC_000015	49257861	49265561	0	0	0	0
SECISBP2L	NC_000015	49280967	49338630	1.79579	1.9395737	1.1996303	1.9019138
COPS2	NC_000015	49417471	49447854	7.7561996	11.262854	6.7192993	7.9320522
GALK2	NC_000015	49447976	49622002	0.3663443	0.5515092	0.2095136	0.629091
RPL15P19	NC_000015	49469512	49470069	0	0	0	0
C15orf33	NC_000015	49620590	49913118	0.1761452	0.0905479	0.0587639	0.1687744
LOC735247	NC_000015	49657062	49658969	0	0	0	0
FGF7	NC_000015	49715375	49779523	1.3510508	1.4349404	0.3774663	0.3345706
DTWD1	NC_000015	49913226	49937333	5.5294866	5.2577049	3.2557609	2.6058987
LOC645453	NC_000015	50086441	50088480	0	0	0	0
ATP8B4	NC_000015	50150435	50411419	0.1316047	0.1114264	0.0413221	0.0917074
SLC27A2	NC_000015	50474422	50528570	0.0187572	0.1542754	0.0333739	0.0392124
LOC100288779	NC_000015	50526063	50528589	0	0	0.0640944	0
HDC	NC_000015	50534144	50557926	0	0.0187639	0	0.0254359
GABPB1	NC_000015	50569389	50647605	3.7700689	5.2049507	3.9169436	3.1619436
FLJ10038	NC_000015	50641133	50647076	0	0	0	0
LOC100129387	NC_000015	50646371	50650509	0	0	0	0
LOC645486	NC_000015	50709580	50710890	0	0	0	0
USP8	NC_000015	50716579	50793277	5.101872	5.7551093	3.8235267	4.225385
RPS20P34	NC_000015	50758817	50759335	0	0	0	0
USP50	NC_000015	50792759	50838902	0.0937063	0.0321133	0.0833637	0.0870643
TRPM7	NC_000015	50852490	50978994	2.7653365	2.4128573	1.990755	2.6876532
SPPL2A	NC_000015	50999730	51057910	10.057804	12.048907	10.795596	10.040694
RPL32P30	NC_000015	51090867	51091154	0	0	0	0
AP4E1	NC_000015	51200946	51298097	0.7760987	0.8722926	0.6202248	1.3382916
TNFAIP8L3	NC_000015	51348798	51397473	2.9960282	3.6802771	4.8114755	2.9147184
CYP19A1	NC_000015	51500254	51630795	0.0486799	0.0600577	0.0086614	0.0203533
LOC100288810	NC_000015	51572312	51574294	0	0	0	0
GLDN	NC_000015	51633713	51700210	0.0864442	0.0533242	0.0922838	0.0240951
DMXL2	NC_000015	51739937	51914967	2.0271018	2.3531242	2.6163588	2.4198864
SCG3	NC_000015	51973550	52013223	0.1460515	0.0546023	0.0354359	0.0555134
LYSMD2	NC_000015	52015261	52043650	1.6876121	2.2169989	1.5013465	0.7839969
TMOD2	NC_000015	52043758	52108560	0.7319921	1.052607	1.0768217	0.3067155

TMOD3	NC_000015	52121889	52201850	4.3305896	5.4748394	2.6728652	3.0177273
LEO1	NC_000015	52230222	52263958	0.879783	0.5889829	0.2548258	0.3564348
MAPK6	NC_000015	52311411	52358462	8.1863334	9.346815	5.9502331	4.921726
RPS13P8	NC_000015	52387469	52387986	0	0	0	0
BCL2L10	NC_000015	52401822	52404972	0.1981882	0.0509396	0.0440784	0
GNB5	NC_000015	52413123	52483565	3.5491317	4.9887037	3.0464034	4.3473802
MYO5C	NC_000015	52484519	52587970	0.0822527	0.0260198	0.0337727	0.030863
MYO5A	NC_000015	52599480	52821247	0.9131166	1.4451297	0.8123339	2.3021892
EEF1B1	NC_000015	52797214	52798208	0	0	0	0
ARPP19	NC_000015	52839432	52861213	13.494372	19.545629	15.792023	20.284087
KIAA1370	NC_000015	52873518	52970820	1.0317465	0.7928792	0.5377422	0.8351579
ONECUT1	NC_000015	53049353	53082209	0.0456605	0.0469438	0.0812417	0.0954542
RPSAP55	NC_000015	53177716	53178739	0	0	0	0
LOC645693	NC_000015	53229336	53230715	0	0	0	0
WDR72	NC_000015	53805938	54051859	0.0300645	0.0247275	0.0320954	0.0502803
UNC13C	NC_000015	54305101	54920806	0.0107981	0.0111016	0.0192126	0.0376227
RSL24D1	NC_000015	55473512	55489231	12.023854	17.33104	19.508969	23.3273
RAB27A	NC_000015	55495792	55582001	3.3551566	4.7670562	6.8279825	8.3786938
PIGB	NC_000015	55611133	55647846	2.6004871	1.2545232	2.3490572	2.1745476
CCPG1	NC_000015	55647438	55700574	4.5601704	2.5296769	4.027668	3.4108927
DYX1C1	NC_000015	55709953	55800432	0.1852792	0.1142919	0.1977954	0.1549319
PYGO1	NC_000015	55838221	55881050	1.9183752	2.5819087	3.0409218	2.649295
PRTG	NC_000015	55903738	56035177	0.0917653	0.0981181	0.1763358	0.0511566
NEDD4	NC_000015	56119120	56285835	4.996743	6.1586735	3.0885432	3.7838309
CNOT6LP1	NC_000015	56295503	56299377	0	0	0	0
CD24L2	NC_000015	56363537	56366289	0	0	0	0
RFX7	NC_000015	56382731	56535483	2.0401264	2.5433326	3.1136769	2.9582058
LOC390586	NC_000015	56483782	56485548	0	0	0	0
TEX9	NC_000015	56657644	56738072	0.435131	0.5751777	0.6083072	0.9096499
MNS1	NC_000015	56720929	56757335	0.0868971	0	0	0.0756917
ZNF280D	NC_000015	56922374	57025787	1.5394952	2.0232488	2.0091446	2.6111099
LOC645877	NC_000015	57138538	57138804	0	0	0	0
LOC145783	NC_000015	57178368	57210697	0	0	0	0
TCF12	NC_000015	57210833	57580716	8.4557041	12.14499	4.7660584	5.7740526
LOC283663	NC_000015	57592563	57599967	0	0	0	0
LOC100128711	NC_000015	57617667	57618645	0	0	0	0
CGNL1	NC_000015	57730183	57842921	0.1105307	0.1325767	0.1529596	0.0813012
RPS13P7	NC_000015	57848103	57848606	0	0	0	0
GCOM1	NC_000015	57884114	58009753	3.4776359	4.0504814	2.3482231	2.6744235
LOC100288996	NC_000015	57976597	57977562	0	0	0	0
LOC100288938	NC_000015	57998719	58004359	0.5588331	0.7386932	0.7102192	0.0278155
GRINL1A	NC_000015	57998901	58009753	0.1385707	0.5589021	0.3793118	0.1337007
ALDH1A2	NC_000015	58245627	58357906	0.1284285	0.0924266	0.1142536	0.0536965
AQP9	NC_000015	58430408	58478110	0.0581903	0	0.0905935	0.1013733
RPL28P4	NC_000015	58492805	58493217	0	0	0	0
LIPC	NC_000015	58724175	58861073	0.7671761	0.0281692	0.1950003	0.0572785
ADAM10	NC_000015	58888510	59042177	12.612594	21.665481	8.3531596	9.7637758
LOC100289060	NC_000015	58978589	58979780	0.0737386	0.0758111	0	0.077076
HSP90AB4P	NC_000015	58983302	58985324	0	0	0	0
LOC100289091	NC_000015	59047825	59048446	0	0	0	0
FAM63B	NC_000015	59063393	59149754	2.9018282	3.2898348	1.2556769	1.6861094
RPL21P14	NC_000015	59161818	59162366	0	0	0	0
SLTM	NC_000015	59171244	59225852	0.9325885	0.7735764	0.5185354	1.240651
RNF111	NC_000015	59279865	59389254	1.7696742	2.1539217	2.5274338	2.9529948
RPL21P113	NC_000015	59341598	59341932	0	0	0	0
CCNB2	NC_000015	59397320	59417244	14.552587	18.575067	12.765497	13.561579
MYO1E	NC_000015	59428563	59665071	23.311671	20.833238	9.1130911	13.055356
LDHAL6B	NC_000015	59499042	59500714	0	0	0	0
LOC100132644	NC_000015	59699161	59700353	0	0	0	0
FAM81A	NC_000015	59730372	59815751	0.15207	0.1693726	0.293119	0.5121808
RPL21P114	NC_000015	59817584	59818123	0	0	0	0
RPL21P117	NC_000015	59844771	59845332	0	0	0	0
GCNT3	NC_000015	59908788	59912199	0.4363654	0.3204497	1.20158	0.4054357
GTF2A2	NC_000015	59930261	59949737	3.3614008	5.1124077	3.7812437	3.7361577
BNIP2	NC_000015	59955061	59981642	6.265852	5.5806346	4.6736963	5.2054996
LOC644761	NC_000015	59968551	59969520	0	0	0	0

LOC100130363	NC_000015	59980673	59981625	0	0	0	0
LOC100130107	NC_000015	60057322	60061422	0.4444203	0.3046072	0.4392985	0.7226095
RPS3AP6	NC_000015	60060529	60061387	0	0	0	0
DDX18P2	NC_000015	60163542	60165752	0	0	0	0
LOC100129256	NC_000015	60257141	60258284	0	0	0	0
FOXB1	NC_000015	60296421	60298142	0	0	0	0.0209903
LOC100287115	NC_000015	60296430	60298691	0	0	0	0
ANXA2	NC_000015	60639350	60690185	356.4124	528.31166	186.38232	223.34866
RPL36AP44	NC_000015	60682537	60682930	0	0	0	0
RPS10P22	NC_000015	60696618	60697205	0	0	0	0
NARG2	NC_000015	60711808	60771344	1.8898532	2.3252937	1.8927799	2.2557651
RORA	NC_000015	60780483	61521502	1.3030165	0.7129067	1.2371575	1.1018053
CYCSP38	NC_000015	60850279	60850552	0	0	0	0
VPS13C	NC_000015	62144588	62352647	1.9991149	1.9424082	1.580222	2.7433662
FAM148A	NC_000015	62359176	62363116	0.0255142	0.0524626	0.0226982	0.0444483
LOC100129972	NC_000015	62374644	62375506	0	0	0	0
FAM148B	NC_000015	62455737	62457482	0.0278859	0	0.0248081	0
FLJ38723	NC_000015	62534728	62535331	0	0	0	0
LOC100289289	NC_000015	62535707	62612506	0.011427	0.0234963	0.0203315	0.0238884
LOC100128015	NC_000015	62654226	62654530	0	0	0	0
LOC100289356	NC_000015	62865101	62866778	0.0785725	0	0	0.0365016
MGC15885	NC_000015	62929371	62937380	0	0	0	0
TLN2	NC_000015	62939510	63136830	3.7157947	4.4756778	1.4565102	4.142902
LOC100287243	NC_000015	63245372	63334580	0	0	0	0
TPM1	NC_000015	63334838	63364114	32.521692	59.399314	5.35695	12.24081
LOC100128979	NC_000015	63338020	63341586	0	0.0126671	0	0
LACTB	NC_000015	63414032	63434260	12.191688	6.4059644	6.7071911	10.8837
RPS27L	NC_000015	63445539	63449741	45.286139	29.51093	36.608883	44.213962
RAB8B	NC_000015	63481728	63559975	12.51673	6.5685938	8.1049085	6.9827485
APH1B	NC_000015	63569749	63601325	3.2492134	2.586219	2.0700357	1.6725727
CA12	NC_000015	63615730	63674075	37.491435	17.505016	21.3635	15.532014
USP3	NC_000015	63796810	63883663	2.5652187	3.3548191	2.3156498	2.3921579
LOC100130855	NC_000015	63848841	63895961	0	0	0	0
LOC100289457	NC_000015	63889590	63891556	0	0	0	0
FBXL22	NC_000015	63889596	63894627	0	0	0	0.1033228
HERC1	NC_000015	63900817	64126147	0.8213001	1.3319842	1.0496681	1.120447
DAPK2	NC_000015	64199235	64338521	0.5016921	0.4814061	0.9075158	0.617625
FAM96A	NC_000015	64364761	64386207	2.6159662	2.5239812	6.2298319	9.4510842
SNX1	NC_000015	64388177	64436433	2.9936759	3.3746027	5.1838397	6.9810269
SNX22	NC_000015	64443916	64449680	0.1580872	0.1250232	0.0973653	0.186427
PPIB	NC_000015	64448014	64455354	205.37676	222.44474	253.71582	197.60196
CSNK1G1	NC_000015	64457716	64648442	2.3204322	2.0867503	2.4714374	3.6653811
KIAA0101	NC_000015	64657193	64673702	7.3636665	10.214333	17.495121	19.649606
TRIP4	NC_000015	64680020	64747502	4.6808768	5.0352312	3.971449	4.86253
ZNF609	NC_000015	64791619	64978267	0.566054	0.7811937	2.5178869	4.1612593
GAPDHL6	NC_000015	64820668	64821939	0	0	0	0
RPS26P50	NC_000015	64885156	64885614	0	0	0	0
RPL21P15	NC_000015	64923276	64923732	0	0	0	0
OAZ2	NC_000015	64979773	64995462	12.019245	17.663031	25.670531	28.515022
RBPMS2	NC_000015	65032093	65067770	3.0164869	0.2263697	0.5092869	0.1841175
PIF1	NC_000015	65107831	65117838	1.4219518	1.0539394	2.2946653	2.200659
PLEKHO2	NC_000015	65134082	65160201	10.842375	4.9906956	13.522861	9.2343761
ANKDD1A	NC_000015	65204101	65251042	0.5082353	0.1886875	0.6656508	0.4820492
SPG21	NC_000015	65255363	65282251	11.675588	11.422906	17.925825	19.519874
MTFMT	NC_000015	65293850	65321977	1.3923875	1.0530727	1.6373707	1.6617286
LOC100130437	NC_000015	65318428	65319014	0	0	0	0
OSTbeta	NC_000015	65342313	65345539	0	0	0.086118	0.1349114
RASL12	NC_000015	65345675	65360388	0.1022845	0.0701061	0.0151659	0.0950345
LOC390594	NC_000015	65369154	65370530	0	0	0.0283933	0.0222403
LOC100287308	NC_000015	65385341	65392099	0.0747419	0.153685	0.1329849	0.83333
LOC390595	NC_000015	65394270	65407532	0.0402825	0.0414147	0.1075093	0.2526342
PDCD7	NC_000015	65409717	65426174	2.2505934	3.1855012	3.9358125	3.4588603
CLPX	NC_000015	65442784	65477563	1.2524592	1.2300033	2.4446372	2.7225009
LOC100287371	NC_000015	65479466	65491217	0.0547983	0	0	0.0190928
CILP	NC_000015	65488337	65503840	0.0590173	0.0404507	0.0175011	0.0479799
PARP16	NC_000015	65550437	65579018	1.5435646	1.5156232	2.7926833	4.2541267

LOC100287416	NC_000015	65578853	65594738	0	0	0	0
RNU5A	NC_000015	65588389	65588504	0	0	0	0
RNU5B-1	NC_000015	65597015	65597131	0	0	0	0
IGDCC3	NC_000015	65619465	65670378	0.0098121	0.0403513	0.0174582	0.0410246
IGDCC4	NC_000015	65673825	65715410	0.426945	0.2159885	0.0422025	0.0330569
DPP8	NC_000015	65737998	65810035	2.5563187	2.8489302	3.8660925	4.3749826
PTPLAD1	NC_000015	65822827	65870693	27.755336	34.324174	46.044452	40.80135
C15orf44	NC_000015	65871116	65903469	3.4202965	5.7075631	7.2017808	9.1503352
SLC24A1	NC_000015	65914270	65948598	0.525733	0.4308403	0.5626037	0.918534
DENND4A	NC_000015	65952954	66084631	1.6583109	1.5818191	4.8945188	4.1133538
LOC646358	NC_000015	66062788	66064492	0	0	0	0
RAB11A	NC_000015	66161796	66181792	18.328598	20.996215	29.033169	42.565125
MEGF11	NC_000015	66187634	66546075	0.0148624	0.0382004	0.0264441	0.139816
DIS3L	NC_000015	66585633	66626236	2.06061	2.9777027	2.2099952	3.3265367
TIPIN	NC_000015	66629008	66649054	2.1974116	2.2238706	2.321418	3.8759612
SCARNA14	NC_000015	66639544	66639680	0	0	0	0
RPL9P25	NC_000015	66671576	66672275	0	0	0	0
MAP2K1	NC_000015	66679211	66783882	8.65029	10.133942	9.524929	17.858591
ATP5J2P6	NC_000015	66707138	66707563	0	0	0	0
RPL35AP32	NC_000015	66738789	66739178	0	0	0	0
SNAPC5	NC_000015	66782666	66790146	2.2953854	3.5686254	5.3292223	3.9792835
RPL4	NC_000015	66791653	66797184	38.516083	65.72418	159.85771	264.78116
SNORD18C	NC_000015	66793590	66793656	0	0	0	0
SNORD18B	NC_000015	66794360	66794429	0	0	0	0
SNORD16	NC_000015	66795149	66795248	0	0	0	0
SNORD18A	NC_000015	66795583	66795652	0	0	0	0
ZWILCH	NC_000015	66797431	66841823	9.2922275	17.162027	15.243506	20.888034
LCTL	NC_000015	66840536	66857835	0.227176	0.2972592	0.1102375	0.2014795
SMAD6	NC_000015	66994634	67074326	3.2634826	2.5201303	0.7742092	1.5160831
LETM1P1	NC_000015	67100905	67103007	0	0	0	0
LOC100287447	NC_000015	67293152	67300393	0	0	0	0
SMAD3	NC_000015	67358195	67487530	12.733038	5.8151448	19.838176	34.501562
FLJ11506	NC_000015	67493366	67547074	8.1071053	8.2079476	9.5751944	11.395554
RPS24P16	NC_000015	67524745	67525252	0	0	0	0
IQCH	NC_000015	67547188	67794139	0.3276662	0.2947659	0.4463608	0.420985
C15orf61	NC_000015	67813522	67819641	1.0165358	0.9405951	1.0550615	1.4875616
MAP2K5	NC_000015	67835021	68099452	2.1007404	2.4273655	2.9273558	4.7543699
LBXCOR1	NC_000015	68117941	68126174	0.0158887	0.0163353	0.0565402	0.3764446
RNU6-1	NC_000015	68132278	68132383	0	0	0	0
LOC100286942	NC_000015	68266783	68266965	0	0	0	0
PIAS1	NC_000015	68346572	68480413	7.4687034	8.6655804	14.33065	15.546574
CALML4	NC_000015	68483043	68498448	0.5120182	0.5556535	1.3580816	1.6320054
CLN6	NC_000015	68499330	68522080	2.4502805	2.9423625	5.2141713	11.392127
FEM1B	NC_000015	68570141	68583640	14.632237	16.049907	21.206495	13.379685
ITGA11	NC_000015	68594042	68724492	5.7819694	4.3568743	0.3122185	0.7642463
CORO2B	NC_000015	68937492	69020145	1.3721211	2.1990085	4.1646626	4.8651092
ANP32A	NC_000015	69070874	69113261	0.7303922	1.3553189	1.5055812	2.0606931
C15orf28	NC_000015	69096160	69099440	0	0	0	0
LOC390600	NC_000015	69126067	69127699	0	0	0	0
NOX5	NC_000015	69222864	69349073	0.102126	0.2624907	0.0151424	0.0830264
SPESP1	NC_000015	69222865	69239148	0	0	0	0
TMEM84	NC_000015	69373190	69388164	0	0	0	0
GLCE	NC_000015	69452973	69564544	0.958617	2.1771895	3.5120359	4.0505243
PAQR5	NC_000015	69591294	69699976	1.3630413	1.5471549	6.6587822	8.8887913
KIF23	NC_000015	69706688	69740764	4.7009303	10.115687	5.6517487	10.36912
RPLP1	NC_000015	69745159	69747884	215.79269	272.86539	374.09956	655.50399
LOC145837	NC_000015	69854059	69863779	0	0	0	0
GEMIN8P	NC_000015	70095454	70096481	0	0	0	0
C15orf50	NC_000015	70127573	70135306	0	0	0	0
TLE3	NC_000015	70340543	70390256	0.4863901	0.7712792	0.5060462	1.6544673
UACA	NC_000015	70946893	71055850	0.5023001	0.9395062	0.8344978	4.1117125
RPL29P30	NC_000015	71088859	71089485	0	0	0	0
LARP6	NC_000015	71123889	71146498	17.950686	24.144097	33.24044	23.305232
KRT8P9	NC_000015	71150976	71152701	0	0	0	0
THAP10	NC_000015	71173681	71184753	1.4302679	1.7601029	3.0267839	2.1896486
LRRC49	NC_000015	71184987	71342436	1.6521892	2.6207344	2.5757079	2.8618307

RPL5P3	NC_000015	71355377	71356404	0	0	0	0
LOC196993	NC_000015	71402583	71407839	0.1440139	0.0740307	0.1281187	0.501773
THSD4	NC_000015	71433788	72075722	4.3539746	6.2303193	7.2423484	16.516337
RPL17P39	NC_000015	71633456	71634086	0	0	0	0
NR2E3	NC_000015	72102894	72110600	0	0.0160795	0.0556549	0.1743765
MYO9A	NC_000015	72118351	72410422	1.0923841	1.5390434	2.1550902	3.5241517
SENP8	NC_000015	72410754	72433304	0.824602	1.13037	0.9237785	1.6174362
RPL21P115	NC_000015	72426943	72427396	0	0	0	0
GRAMD2	NC_000015	72452147	72490136	0.04005	0.0137252	0.0118765	0.0465141
PKM2	NC_000015	72491370	72523684	477.22737	385.24377	583.00925	779.16115
PARP6	NC_000015	72533522	72563628	7.4809062	7.6223332	19.697669	11.813786
BRUNOL6	NC_000015	72577068	72612525	0.0646298	0.0930247	0.0919943	0.0900732
HEXA	NC_000015	72635778	72668520	36.283891	23.064486	66.868549	47.200264
C15orf34	NC_000015	72668454	72671129	0	0	0	0
RPL12P35	NC_000015	72671515	72672139	0	0	0	0
TMEM202	NC_000015	72690668	72700708	0.0678213	0	0	0.0236303
LOC100130579	NC_000015	72742999	72743816	0	0	0	0
ARIH1	NC_000015	72766667	72878896	2.1844389	2.4007178	5.1896788	7.465215
LOC646665	NC_000015	72897519	72904665	0	0	0	0
LOC100129119	NC_000015	72900822	72929228	0	0	0	0.068975
LOC646670	NC_000015	72929419	72943117	0.1385287	0.1780276	0.3389072	0.1930646
GOLGA6B	NC_000015	72947038	72959738	0.0138289	0.0142176	0.0123026	0.0192731
HIGD2B	NC_000015	72968123	72978490	0	0	0	0
BBS4	NC_000015	72978526	73030817	3.3331524	2.9735465	2.4945879	2.3595412
ADPGK	NC_000015	73043708	73076126	15.233326	16.50898	24.552477	19.171964
LOC100287559	NC_000015	73078490	73079178	0	0	0	0
LOC729686	NC_000015	73191423	73192693	0	0	0	0
NEO1	NC_000015	73344875	73597546	2.8853323	5.6631747	3.7280754	0.6658528
FKBP1P2	NC_000015	73434433	73435986	0	0	0	0
HCN4	NC_000015	73612200	73661605	0.066883	0.037507	0.0270459	0.0508437
C15orf60	NC_000015	73735499	73852354	0.0474603	0.2439709	0.126666	0.4630111
MRPS15P1	NC_000015	73775531	73776286	0	0	0	0
NPTN	NC_000015	73852357	73925753	21.587026	21.12014	21.639005	30.39905
CD276	NC_000015	73976622	74006859	36.802283	34.794275	30.188241	44.762208
C15orf59	NC_000015	74032141	74043816	0.1307983	0.3025674	0.0290905	0.0455727
LOC100287587	NC_000015	74032143	74060199	0.1971878	0.228071	0.0438559	0.0343521
TBC1D21	NC_000015	74165968	74181555	0.0364111	0.0374345	0.0323923	0.0253727
LOC100287616	NC_000015	74211971	74229525	2.3253033	1.3148609	1.9135052	4.4560008
LOXL1	NC_000015	74218789	74244478	78.440427	70.865793	77.748018	50.302108
STOML1	NC_000015	74275560	74284635	5.3109646	5.4829808	6.2800219	6.8466495
PML	NC_000015	74287014	74340160	4.4980095	3.3584238	7.3894348	5.4106997
LOC100287646	NC_000015	74290469	74318286	0.1647544	0.5081545	0.2564976	0.401826
DNM1P33	NC_000015	74356030	74358099	0	0	0	0
GOLGA6	NC_000015	74362198	74374891	0	0.0142265	0.0123103	0.0385704
LOC283731	NC_000015	74418714	74421619	0	0	0	0
ISLR2	NC_000015	74421715	74429143	0.0350674	0.3425033	0.0701931	0.0977455
ISLR	NC_000015	74466087	74469212	53.530236	91.408769	0.6885106	0.2054499
STRA6	NC_000015	74471807	74501371	0.0404866	0.1456858	0.0090045	0.0211595
CCDC33	NC_000015	74528667	74628482	0.0464078	0.0357841	0.0309643	0.0080847
CYP11A1	NC_000015	74630103	74660081	1.7014554	2.0842426	2.0611567	0.6054342
LOC729739	NC_000015	74646797	74656961	0	0	0	0
SEMA7A	NC_000015	74701630	74726299	23.796614	81.065126	9.7743914	31.24173
UBL7	NC_000015	74738318	74753529	14.710577	26.600667	25.98342	44.605338
ARID3B	NC_000015	74833548	74890472	0.5492234	0.3409264	1.0140845	1.487556
CLK3	NC_000015	74900713	74922542	3.5944573	2.3979559	4.9031843	6.1004847
EDC3	NC_000015	74922899	74988386	3.2942273	5.0635614	4.5352792	6.4576421
CYP1A1	NC_000015	75011883	75017877	0.2527697	0.3291736	0.1199312	0.1996254
CYP1A2	NC_000015	75041184	75048941	0.1545988	0.3901349	0.1875483	0.2742234
CSK	NC_000015	75074425	75095539	9.5435029	11.229889	8.1619736	10.841654
LMAN1L	NC_000015	75105194	75118099	0	0	0	0.0176411
CPLX3	NC_000015	75118951	75124136	0	0.022457	0.0582966	0.0608844
ULK3	NC_000015	75128459	75135552	2.8520484	3.8238704	3.1604484	5.0557199
SCAMP2	NC_000015	75137197	75165670	6.9786116	5.5298044	10.690504	14.351705
MPI	NC_000015	75182410	75190565	6.6009202	7.6028536	7.219031	8.8883046
C15orf17	NC_000015	75192328	75199462	3.1197055	2.851009	2.7313229	3.8561466
COX5A	NC_000015	75212616	75230495	15.467495	17.427884	27.266744	24.102177

RPP25	NC_000015	75247443	75249775	4.7470872	6.158732	4.9102386	4.003681
SCAMP5	NC_000015	75287901	75313836	0.714711	0.2404794	0.3468146	0.2263814
PPCDC	NC_000015	75315927	75343067	1.8419043	1.9954815	1.6386091	2.3048016
LOC100128721	NC_000015	75406602	75438068	0	0	0	0
RPL36AP45	NC_000015	75478992	75479309	0	0	0	0
C15orf39	NC_000015	75494221	75504510	1.22106	1.1839337	2.0312718	2.8293597
LOC730036	NC_000015	75513863	75527175	0.0220292	0.0905933	0.0979889	0.0307016
LOC100128707	NC_000015	75544032	75545320	0	0	0	0
GOLGA6C	NC_000015	75550905	75563868	0	0	0	0.0355586
GOLGA6D	NC_000015	75575182	75588148	0.0127423	0	0.0113359	0
DNM1P34	NC_000015	75590484	75595261	0	0	0	0
LOC646791	NC_000015	75614114	75614850	0	0	0	0
COMMD4	NC_000015	75628374	75632614	42.142791	52.412746	39.435707	55.886264
NEIL1	NC_000015	75639331	75647588	0.2090561	0.1910503	0.0413294	0.2427977
MAN2C1	NC_000015	75648133	75660941	4.5514917	2.3605342	3.7006915	3.548119
SIN3A	NC_000015	75661720	75748124	3.3695111	4.3173384	8.2713917	9.1292013
RPL13P4	NC_000015	75680577	75681217	0	0	0	0
PTPN9	NC_000015	75759462	75871625	1.8525664	2.4324229	4.32873	4.3161014
SNUPN	NC_000015	75890424	75918719	7.9618001	7.1158626	8.5720859	10.103215
IMP3	NC_000015	75931436	75932595	1.6670019	2.1033653	2.3256311	4.1449189
SNX33	NC_000015	75941348	75950968	9.012582	8.6090243	9.0336782	19.712456
CSPG4	NC_000015	75966663	76005189	29.926148	25.028007	24.222569	19.745473
ODF3L1	NC_000015	76016319	76020027	0.1473537	0.1136213	0.0327725	0.051341
DNM1P35	NC_000015	76025203	76032502	0	0	0	0
LOC391532	NC_000015	76039110	76039601	0	0	0	0
LOC441728	NC_000015	76067900	76079321	0	0	0	0
LOC730058	NC_000015	76083899	76109635	0	0	0	0
UBE2Q2	NC_000015	76135622	76193388	5.0456785	7.8003503	8.16141	9.433672
FBXO22	NC_000015	76196200	76226944	2.1145901	2.4397346	3.9923203	4.191376
FBXO22OS	NC_000015	76225024	76225471	0	0	0	0
NRG4	NC_000015	76235844	76304785	0.1982627	0.067945	0	0.3223671
C15orf27	NC_000015	76352299	76497304	0.0549582	0	0.0325949	0.0638284
LOC100287180	NC_000015	76499321	76502614	0.1200771	0.0685844	0.0949546	0.0929717
ETFA	NC_000015	76508628	76603810	18.098239	19.773991	33.240144	48.389568
TYRO3P	NC_000015	76549426	76554087	0	0	0	0
ISL2	NC_000015	76629147	76634816	0.0244428	0	0.021745	0
SCAPER	NC_000015	76640537	77176192	0.1880541	0.2610081	0.4767996	0.7862613
TRNAK42P	NC_000015	76674765	76674838	0	0	0	0
RCN2	NC_000015	77223962	77242601	7.7251975	14.414587	18.992265	22.432412
KRT8P23	NC_000015	77271286	77272868	0	0	0	0
PSTPIP1	NC_000015	77287465	77329671	0.2369177	0.0974305	0.0421537	0.0660375
TSPAN3	NC_000015	77338331	77363513	45.706161	41.024053	63.027418	62.676121
LOC100287849	NC_000015	77348450	77363573	0.0904285	0.1859399	0.0804477	0.1260283
SGK269	NC_000015	77400471	77567966	0.804857	0.6401242	0.4964877	2.2989823
C15orf5	NC_000015	77516235	77517746	0	0	0	0
LOC646853	NC_000015	77570807	77571079	0.1609825	0.165507	0	0
HMG20A	NC_000015	77713243	77777945	1.8552149	2.0236582	4.3374648	4.0202543
LINGO1	NC_000015	77905369	77924709	0.0299783	0.0462313	0	0.010445
DNM1P9	NC_000015	78198543	78203963	0	0	0	0
LOC645752	NC_000015	78206559	78219188	0	0	0	0
LOC440292	NC_000015	78232732	78245144	0.1273862	0	0.0377754	0.118357
LOC400406	NC_000015	78268086	78286567	0	0	0	0
LOC91450	NC_000015	78285575	78286567	0	0	0	0
TBC1D2B	NC_000015	78287327	78369994	3.4553002	3.8577554	4.6205628	8.7023719
SH2D7	NC_000015	78384927	78396393	0	0	0.0413294	0.0161865
CIB2	NC_000015	78396991	78423878	0.4829476	1.2266987	1.4152965	0.1385741
IDH3A	NC_000015	78441719	78462884	7.6517747	10.342167	11.63391	10.664123
ACSBG1	NC_000015	78463187	78526899	0.0776471	0.0798293	0.0276308	0.0541076
DNAJA4	NC_000015	78556487	78574538	0.3127988	0.3617888	0.0347843	0.163478
WDR61	NC_000015	78575577	78591940	8.081161	14.719698	13.360797	12.496802
CRABP1	NC_000015	78632666	78640572	0.0569278	0	0.101289	0.0793391
LOC100129388	NC_000015	78655993	78681503	0	0	0	0
IREB2	NC_000015	78730518	78793798	2.5056083	2.8385991	2.886109	3.828711
LOC123688	NC_000015	78799906	78829715	0.1342956	0.345175	0.6272337	0.7720558
PSMA4	NC_000015	78832747	78841562	18.80408	22.887986	38.648905	42.473192
CHRNA5	NC_000015	78857906	78886460	0.2715088	0.2047024	0.3059529	0.239651

CHRNA3	NC_000015	78887651	78913322	0.0146543	0	0.0130369	0.0612702
LOC100287983	NC_000015	78911191	78912648	0	0	0	0
CHRNB4	NC_000015	78916636	78933587	0.0538581	0.1292009	0.0958274	0.0625508
RPL18P11	NC_000015	78953350	78953983	0	0	0	0
LOC646934	NC_000015	79019519	79030698	0	0	0	0
DNM1P37	NC_000015	79029473	79033636	0	0	0	0
LOC646938	NC_000015	79044413	79045734	0	0	0	0
ADAMTS7	NC_000015	79051545	79103773	3.1380158	2.2879755	0.3560798	0.5466736
LOC390614	NC_000015	79123306	79124381	0	0	0	0
RPL21P116	NC_000015	79155554	79156113	0	0	0	0
MORF4L1	NC_000015	79165172	79190075	31.801963	42.067305	53.002052	65.158645
CTSH	NC_000015	79214092	79237420	2.2196077	3.1339329	29.303428	11.445662
RASGRF1	NC_000015	79252289	79383215	0.0179674	0.0615745	0.0426248	0.0459081
LOC100129540	NC_000015	79271231	79276919	0	0	0	0
LOC729911	NC_000015	79484049	79579362	0.6113355	0.279341	0.302145	0.0473337
LOC646956	NC_000015	79528674	79529550	0	0	0	0
ANKRD34C	NC_000015	79585627	79587836	0.0397722	0.0613349	0.0707648	0.0277148
TMED3	NC_000015	79603491	79615189	4.6861227	5.013144	8.8166701	6.2220574
KIAA1024	NC_000015	79724858	79764642	0.1434086	0.2077552	0.330549	0.4042738
LOC390617	NC_000015	79848984	79853015	0	0	0	0
LOC390618	NC_000015	79960845	79961415	0	0	0	0
RPS12P25	NC_000015	80044008	80044391	0	0	0	0
MTHFS	NC_000015	80137318	80189370	3.1249871	4.538739	4.4128178	3.6293592
ST20	NC_000015	80191182	80216077	0.1696843	0.2442346	0.5736322	0.4729711
C15orf37	NC_000015	80215113	80217194	0.063326	0.0217019	0.0375577	0.1323842
LOC401822	NC_000015	80236834	80237974	0	0	0	0
BCL2A1	NC_000015	80253232	80263643	0.2330235	0.0479145	0.0829217	0.097428
ZFAND6	NC_000015	80352021	80430710	4.4000613	3.2043056	8.970538	10.00145
FAH	NC_000015	80445341	80478682	5.8314171	7.431686	14.320463	12.211855
ARNT2	NC_000015	80696692	80890278	3.6986467	1.4673067	0.9835491	9.9172498
LOC100287909	NC_000015	80907283	80909216	0	0.0233627	0	0.047505
FAM108C1	NC_000015	80987652	81047962	3.2961174	3.3121731	3.4624539	11.445399
KIAA1199	NC_000015	81071712	81243999	82.88711	45.03662	2.7611275	103.22487
MESDC2	NC_000015	81268095	81282205	2.8691162	3.7910184	4.4322246	5.3266934
MESDC1	NC_000015	81293295	81296345	3.644347	3.7023437	4.1775832	5.0589769
LOC647020	NC_000015	81372889	81426607	0	0	0	0.113174
C15orf26	NC_000015	81426644	81441516	0	0.2868787	0.2482385	0.2527768
IL16	NC_000015	81489219	81605102	0.0676928	0.0160605	0.1297076	0.275769
STARD5	NC_000015	81605007	81616524	3.4063219	2.1287013	2.2282047	3.8630166
TMC3	NC_000015	81624760	81666418	0.0665278	0.082077	0.1065329	0.343058
LOC100288241	NC_000015	82302553	82302972	0	0	0	0
MEX3B	NC_000015	82334128	82338361	0.556143	0.5983676	0.5983147	0.5407571
EFTUD1	NC_000015	82422561	82555104	3.1843088	2.3909806	5.49594	7.704006
FAM154B	NC_000015	82555152	82577268	0	0.028862	0.0374617	0.0391247
LOC441730	NC_000015	82585621	82630296	0	0	0	0
LOC647042	NC_000015	82635112	82641706	0.0282081	0.1160036	0.0752841	0.0982827
LOC100134869	NC_000015	82647286	82707816	0	0	0	0
RPL9P9	NC_000015	82664415	82665118	0	0	0	0
LOC440295	NC_000015	82722185	82731587	0.0200906	0.0103276	0.151922	0.2099992
DNM1P36	NC_000015	82731700	82733632	0	0	0	0
LOC440297	NC_000015	82738195	82763331	0	0	0	0
LOC100288036	NC_000015	82763613	82766494	0.1219937	0.078389	0.3934176	0.1487676
LOC80154	NC_000015	82764078	82798323	0	0	0	0
LOC642402	NC_000015	82804060	82811851	0	0	0	0
DNM1P38	NC_000015	82814261	82817391	0	0	0	0
RPS17	NC_000015	82821161	82824865	247.55994	356.62281	560.50593	623.10577
LOC400419	NC_000015	82882946	82924300	0	0	0	0
DNM1P43	NC_000015	82927158	82928975	0	0	0	0
LOC643696	NC_000015	82932417	82939197	0	0	0	0
LOC100288455	NC_000015	82944727	82962390	0.1058994	0	0.188422	0.2213847
LOC727751	NC_000015	82944795	82972087	0.1391501	0.0715305	0.2888475	0.5009875
LOC100288161	NC_000015	82973384	82976263	0.1373382	0.0784434	0.298662	0.4785137
LOC642288	NC_000015	82976544	82991960	0	0	0	0
DNM1P39	NC_000015	83004753	83008034	0	0	0	0
LOC642325	NC_000015	83010366	83018105	0	0	0	0
LOC100133144	NC_000015	83023773	83084733	0	0	0	0

RPL9P8	NC_000015	83040956	83041659	0	0	0	0
LOC728636	NC_000015	83098710	83111378	0.0460131	0.0118266	0.0614018	0.1082151
LOC390627	NC_000015	83124488	83139917	0	0	0	0
LOC100288549	NC_000015	83140199	83141722	0	0	0	0
LOC727849	NC_000015	83144375	83182928	0	0	0	0
LOC100288587	NC_000015	83154059	83182996	0	0	0.188422	0.1475898
LOC728648	NC_000015	83188541	83195234	0	0	0	0
DNM1P42	NC_000015	83198606	83201760	0	0	0	0
CPEB1	NC_000015	83211951	83316728	7.3163102	8.3505141	11.393187	13.162555
AP3B2	NC_000015	83328033	83378635	0.2609612	0.1219525	0.1160791	0.0743924
LOC338963	NC_000015	83379222	83380512	0	0	0	0
LOC283693	NC_000015	83394090	83396195	0	0	0	0
SCARNA15	NC_000015	83424697	83424823	0	0	0	0
FSD2	NC_000015	83428024	83474806	0	0.0362482	0.0156829	0.0491374
WHAMM	NC_000015	83477973	83503613	1.8152755	1.3360968	2.4957845	2.774279
HOMER2	NC_000015	83517735	83621473	0.2437875	0.4101368	2.9968885	3.258623
FAM103A1	NC_000015	83654995	83659423	1.7257159	3.5090077	2.3199254	2.6456047
C15orf40	NC_000015	83673405	83680358	1.7203934	2.6691974	3.0610194	3.5093277
BTBD1	NC_000015	83685175	83736106	7.9336159	9.5959895	11.091087	12.970974
FABP5L8	NC_000015	83713074	83713719	0	0	0	0
TM6SF1	NC_000015	83776324	83806111	0.3223024	0.1656804	0.8397068	0.9464996
HDGFRP3	NC_000015	83806816	83876321	7.4843416	9.2290476	15.81341	11.750143
BNC1	NC_000015	83924655	83953468	0.9147941	5.3393222	1.3394222	1.9588766
SH3GL3	NC_000015	84116091	84287495	0.1037126	0.1599412	1.0841213	0.7949821
ADAMTSL3	NC_000015	84322838	84708594	0.4373256	0.2278879	0.9486596	1.0436504
LOC642677	NC_000015	84721866	84725567	0	0	0	0
LOC727963	NC_000015	84735556	84782428	0.1605904	0	0.3809751	0.4476231
LOC648809	NC_000015	84782399	84811205	0.3702017	0.2174893	0.0940976	0.5896487
LOC100132799	NC_000015	84839930	84842125	0.1212365	0.0623219	0.161783	0.3801709
LOC440300	NC_000015	84842715	84866373	0	0	0	0
LOC100288331	NC_000015	84849293	84870500	0.2860747	0.4595545	2.4336564	3.264328
LOC388152	NC_000015	84867600	84898920	0	0	0	0
LOC643707	NC_000015	84911122	84912390	0	0	0	0
LOC100288367	NC_000015	84944245	84950212	0	0.0269752	0.4201529	0.1371263
KIAA1920	NC_000015	84953013	84966398	0	0	0	0
DNM1P44	NC_000015	84980851	84982344	0	0	0	0
LOC100132084	NC_000015	85043615	85045612	0.2441568	0	0.2172087	0.1134255
DNM1P40	NC_000015	85046982	85049663	0	0	0	0
FLJ40113	NC_000015	85047738	85060078	0	0	0	0
FLJ43276	NC_000015	85070427	85114026	0	0	0	0
ZSCAN2	NC_000015	85144249	85166947	0.2693151	0.348669	0.4525592	0.6255649
SCAND2	NC_000015	85174691	85185694	0	0	0	0
WDR73	NC_000015	85185999	85197521	3.0562382	3.5805715	4.5526006	7.1485565
NMB	NC_000015	85198360	85201802	7.9789508	2.1056343	4.3652622	2.6759602
LOC100288043	NC_000015	85200465	85201818	0	0	0	0.086511
SEC11A	NC_000015	85212775	85259674	16.397171	19.37365	38.673514	44.686193
ZNF592	NC_000015	85291818	85349663	1.5475108	1.7954035	4.4982038	5.7363142
ALPK3	NC_000015	85359911	85416713	0.0644107	0.0248329	0.0250694	0.0420787
SLC28A1	NC_000015	85427913	85489027	0	0.013258	0.0344169	0.0179723
PDE8A	NC_000015	85525205	85682373	4.6550577	5.9852439	9.9290653	12.115508
LOC642907	NC_000015	85724386	85725356	0	0	0	0
LOC728121	NC_000015	85731039	85746393	0	0	0	0
LOC727858	NC_000015	85746675	85749577	0.0908334	0.0778219	0.2693597	0.4219756
LOC642423	NC_000015	85750818	85778016	0.1116004	0.4818951	0.2581353	0.5599267
LOC100133220	NC_000015	85783603	85790418	0	0.2140126	0.1851868	0.1450557
DNM1P45	NC_000015	85794010	85796487	0	0	0	0
LOC642935	NC_000015	85798655	85869082	0	0	0	0
AKAP13	NC_000015	85923871	86292586	0.6386872	0.8620473	1.2179745	2.9442609
RNU7-79P	NC_000015	85933061	85933114	0	0	0	0
FABP5L9	NC_000015	85958412	85959072	0	0	0	0
KLHL25	NC_000015	86302557	86338189	0.3341163	0.5520644	0.2653921	0.6569007
LOC100288896	NC_000015	86302557	86304102	0	0	0.0252895	0.0198091
LOC100288707	NC_000015	86683067	86683406	0	0	0	0
AGBL1	NC_000015	86685242	87572283	0.0125818	0.0258708	0.0111931	0.03507
NCRNA00052	NC_000015	88120160	88122917	0	0	0	0
NTRK3	NC_000015	88419988	88799661	0.0431288	0.0266046	0.0153474	0.07814

LOC100288732	NC_000015	88669543	88679808	0	0	0	0
MRPL46	NC_000015	89002709	89010633	3.9992891	5.1509078	6.568391	5.4206035
MRPS11	NC_000015	89010684	89021495	18.857083	23.80588	20.461633	27.548899
DET1	NC_000015	89055714	89089912	2.0820545	1.6391759	2.3528625	2.3135311
LOC100288767	NC_000015	89110565	89111838	0	0	0	0.0963046
AEN	NC_000015	89164527	89175512	14.202782	9.7733032	9.3352339	11.518728
ISG20	NC_000015	89182039	89198879	14.159054	4.0646157	18.362769	2.0822354
ACAN	NC_000015	89346674	89418585	50.415011	0.781867	0.0230121	0.0648909
HAPLN3	NC_000015	89420519	89438770	7.3363134	5.1078965	5.1014785	5.9858452
MFGE8	NC_000015	89441914	89456663	46.356657	24.420391	34.89099	33.765891
LOC100129501	NC_000015	89491567	89496080	0	0	0	0
KRT18P47	NC_000015	89502951	89504342	0	0	0	0
LOC100129942	NC_000015	89532750	89534051	0	0	0	0
LOC100288828	NC_000015	89581120	89581845	0	0	0	0
ABHD2	NC_000015	89631381	89745591	7.2469109	8.4103562	8.2236312	17.30257
HMGB1L8	NC_000015	89678298	89679906	0	0	0	0
LOC728003	NC_000015	89739034	89745591	0	0	0	0
RLBP1	NC_000015	89753098	89764922	0.0250846	0.0257896	0	0.0873998
FANCI	NC_000015	89787194	89860362	7.3293324	12.711102	16.333875	20.616495
POLG	NC_000015	89859536	89878026	7.8648364	7.1513298	10.495165	12.434472
LOC100289531	NC_000015	89878284	89879394	0.1186721	0.1220074	0.3519133	0.1653909
LOC100288864	NC_000015	89902072	89906668	0.0344557	0.017712	0	0.012005
LOC254559	NC_000015	89921273	89941718	0	0	0	0
RHCG	NC_000015	90014638	90039799	0	0	0.0807384	0.0158105
LOC283761	NC_000015	90048161	90067265	0	0	0	0
C15orf42	NC_000015	90118818	90171254	0.665483	0.7383794	1.5064579	3.7879347
KIF7	NC_000015	90171201	90198682	0.308544	0.4262585	0.4288895	0.9675258
LOC643368	NC_000015	90206633	90207349	0	0	0	0
PLIN	NC_000015	90207598	90222648	0.0740867	0.0152338	0.0922734	0.0826025
PEX11A	NC_000015	90226284	90233958	0.9069283	0.2331044	3.7651998	3.1862513
RPL36AP43	NC_000015	90231113	90231511	0	0	0	0
WDR93	NC_000015	90234028	90286869	0.1094601	0.0937804	0.1622979	0.0889889
MESP1	NC_000015	90293098	90294540	0.4160332	0.6221467	1.3122247	1.5022531
MRPL15P1	NC_000015	90295575	90296764	0	0	0	0
MESP2	NC_000015	90319589	90321985	0.5718171	0.8958295	1.0658568	0.5502611
ANPEP	NC_000015	90328126	90358072	39.758099	59.555437	62.770338	29.592862
AP3S2	NC_000015	90373831	90437617	4.6881076	4.6980383	5.8969197	7.0343291
LOC100129356	NC_000015	90409520	90420525	0	0	0	0
LOC100287910	NC_000015	90436310	90438761	0.1075405	0.0737086	0.1913421	0.1623668
C15orf38	NC_000015	90443832	90456222	7.5302731	6.7630498	7.4499054	13.012934
ZNF710	NC_000015	90610342	90624025	2.1334093	2.4656493	3.1283288	2.0095334
IDH2	NC_000015	90627210	90645708	7.1260549	5.0058933	7.9187799	8.9418283
SEMA4B	NC_000015	90728152	90772892	4.4929319	3.9544073	15.075672	9.369255
RPS12P26	NC_000015	90749714	90750227	0	0	0	0
CIB1	NC_000015	90773477	90777279	17.048495	14.937808	36.016181	53.068494
C15orf58	NC_000015	90777487	90785312	0.3903618	0.568555	0.8971314	1.0880786
TTLL13	NC_000015	90792764	90802312	0	0.1007996	0.0436113	0.0512407
NGRN	NC_000015	90808895	90815443	37.830438	42.770197	53.026073	53.976348
LOC400446	NC_000015	90818278	90820841	0	0	0	0
LOC100128397	NC_000015	90827555	90828856	0	0	0	0
LOC728477	NC_000015	90836712	90869080	0.4165709	0.4996585	0.4014758	0.1693319
GABARAPL3	NC_000015	90890724	90892762	0	0	0	0
ZNF774	NC_000015	90895477	90904715	0.3347526	0.3933267	0.4041642	0.4665379
IQGAP1	NC_000015	90931473	91045475	4.7788369	4.875546	8.3346683	24.155851
CRTC3	NC_000015	91073198	91188577	3.6460629	5.0679507	6.2302058	7.503717
BLM	NC_000015	91260579	91358686	1.1161764	2.5645173	3.1343676	4.3353681
FURIN	NC_000015	91411885	91426687	13.867875	21.770185	19.249471	20.28715
FES	NC_000015	91427665	91439006	3.3047316	3.5908406	0.348339	0.1418829
MAN2A2	NC_000015	91447420	91465815	1.3600187	0.81444	3.6484409	3.277922
UNC45A	NC_000015	91473410	91497323	3.8116702	4.4435369	3.5165589	8.445111
HDDC3	NC_000015	91474155	91475774	0.6728916	0.9079918	0.9727624	0.3516732
LOC100288997	NC_000015	91479568	91497323	0.0529816	0.1089413	0.1178347	0.1476787
RCCD1	NC_000015	91498106	91506355	1.6518309	1.1920449	2.8118596	2.7005675
PRC1	NC_000015	91509268	91537804	1.7082352	2.4964809	3.1398623	8.2440244
VPS33B	NC_000015	91541774	91565833	2.4980048	3.5270105	2.9926898	3.075253
LOC100131297	NC_000015	91574939	91575667	0	0	0	0

LOC100131061	NC_000015	91576082	91576459	0	0	0	0
LOC390638	NC_000015	91576460	91586325	0.3130216	0.4505467	2.0050034	1.0470044
SV2B	NC_000015	91769103	91838650	0.0082749	0.0085075	0.0220849	0.0115326
LOC100128403	NC_000015	91839181	91844539	0	0.0084313	0.0437741	0.0285733
LOC441732	NC_000015	92208820	92268594	0	0	0	0
TRNAY16P	NC_000015	92254302	92254374	0	0	0	0
SLCO3A1	NC_000015	92396938	92715665	1.5254506	1.9133547	3.8609016	2.1419344
DUXAP6	NC_000015	92824668	92825704	0	0	0	0
NPM1P5	NC_000015	92828960	92830476	0	0	0	0
ST8SIA2	NC_000015	92937140	93011958	0.0078116	0	0	0.0108869
C15orf32	NC_000015	93014907	93044347	0.0254625	0.0261781	0	0.0177433
LOC100144604	NC_000015	93111048	93115493	0	0	0	0
FAM174B	NC_000015	93160678	93199031	1.2031455	1.2541401	0.044598	0.0698666
LOC643790	NC_000015	93254924	93256137	0	0	0	0
LOC643797	NC_000015	93257424	93258991	0	0	0	0
RPL31P6	NC_000015	93262661	93262942	0	0	0	0
H2AFVP	NC_000015	93276901	93277506	0	0	0	0
LOC643806	NC_000015	93305987	93306980	0	0	0	0
LOC728619	NC_000015	93338877	93339870	0	0	0	0
CHD2	NC_000015	93443551	93571237	0.6461618	0.5836545	1.2277013	1.1964351
RGMA	NC_000015	93586637	93632433	0.1913169	0.9413209	0.1702008	0.2571119
LOC100124334	NC_000015	93608397	93609915	0	0	0	0
UNQ9370	NC_000015	93749295	93751277	0	0	0	0
LOC100289190	NC_000015	94571730	94577469	0	0	0	0
RPL26P5	NC_000015	94816725	94817231	0	0	0	0
MCTP2	NC_000015	94841495	95022263	0.199992	0.1542096	0.5930613	0.4993818
LOC440311	NC_000015	95398550	95400144	0	0	0	0
LOC400456	NC_000015	95822517	95871330	0.0297148	0	0.0264351	0
LOC145820	NC_000015	95976322	96051076	0	0	0	0
RNU2P1	NC_000015	96289033	96289298	0	0	0	0
NR2F2	NC_000015	96869157	96883492	5.7974263	3.9785058	2.6043714	5.6187379
LOC100288183	NC_000015	96877378	96881065	0.2315502	0.238058	0.2883909	0.2258948
LOC728800	NC_000015	96902893	96904900	0	0	0	0
LOC100289303	NC_000015	96921878	96952879	0	0	0	0
LOC390643	NC_000015	96984796	96989682	0	0	0	0
RPL31P55	NC_000015	96991356	96991760	0	0	0	0
LOC388181	NC_000015	97292581	97301826	0	0	0	0
SPATA8	NC_000015	97326676	97328845	0	0.0440384	0	0.0596976
LOC91948	NC_000015	98285846	98417659	0	0	0	0
ARRDC4	NC_000015	98503933	98517068	1.3939813	1.4553788	1.7880864	1.1219835
FAM169B	NC_000015	98980391	99057611	0	0	0.0237099	0.0464295
IGF1R	NC_000015	99192761	99507759	1.2314262	3.6775318	4.5420228	5.2875724
LOC145814	NC_000015	99511509	99548791	0.0441691	0.0454105	0	0.0307788
SYNM	NC_000015	99645286	99675800	0.0358566	0.1290252	0.1222796	0.8370411
TTC23	NC_000015	99676528	99789815	1.1079386	1.4119813	1.9097025	1.8095057
LRRC28	NC_000015	99791652	99926498	2.1519479	2.5240383	3.7479735	3.5060205
HSP90B2P	NC_000015	99797849	99800481	0	0	0	0
LOC100289465	NC_000015	100038447	100048617	0	0.055782	0	0
RPL7P5	NC_000015	100091519	100092333	0	0	0	0
MEF2A	NC_000015	100106133	100256630	5.6560936	4.6995169	10.508626	9.5505005
LYSMD4	NC_000015	100267610	100273626	1.0054015	1.2215963	1.0435052	1.4118228
C15orf51	NC_000015	100330361	100347132	0	0	0	0
LOC644800	NC_000015	100331805	100332380	0	0	0	0
DNM1P46	NC_000015	100336533	100339772	0	0	0	0
LOC400464	NC_000015	100346795	100348181	0	0	0	0
ADAMTS17	NC_000015	100511643	100882183	0.0208253	0.0642317	0.0061756	0.0386983
FLJ42289	NC_000015	100889029	100890918	0	0	0	0
LASS3	NC_000015	100940600	101084925	0.0112861	0.03481	0.0200809	0.0235939
PRKXP1	NC_000015	101093231	101099997	0	0	0	0
LOC100288282	NC_000015	101098882	101100003	0	0.2859709	0.0618632	0
LINS1	NC_000015	101109435	101142404	0.9720796	0.8416	1.5930324	1.9964996
ASB7	NC_000015	101142755	101191906	0.4110513	0.7307526	0.9903904	0.9905943
LOC440313	NC_000015	101324166	101324690	0	0	0	0
PHF5HP	NC_000015	101386043	101387000	0	0	0	0
LOC145757	NC_000015	101401776	101402385	0	0	0	0
ALDH1A3	NC_000015	101420009	101456831	4.7392687	10.843499	9.5619046	16.705275

LRRK1	NC_000015	101459460	101610317	2.2518358	2.1907822	4.9749359	4.8840882
CHSY1	NC_000015	101715932	101792126	1.4342532	1.4745631	1.73288	2.7484731
SELS	NC_000015	101811214	101817700	7.0702393	8.1866239	7.1675388	7.693048
SNRPA1	NC_000015	101821715	101835460	1.8259889	2.6452983	3.4704166	2.8340303
PCSK6	NC_000015	101844133	102030187	0.0328364	0.0202556	0.040897	0.0732215
LOC100288388	NC_000015	101847456	101849509	0	0	0	0
LOC100289564	NC_000015	102097390	102103188	0	0	0	0
TM2D3	NC_000015	102182049	102192594	6.8717918	6.9364711	11.031794	9.490012
TARSL2	NC_000015	102193955	102264645	0.2401543	0.5349583	0.5103811	0.873934
LOC100128108	NC_000015	102276894	102284972	0.697591	0.224124	1.3963416	1.9748185
LOC100288426	NC_000015	102284970	102286346	0.0521331	0.1071967	0.0927582	0.5812551
LOC441734	NC_000015	102285799	102298299	0	0	0	0
LOC100289638	NC_000015	102298304	102303178	0	0	0	0
LOC100289668	NC_000015	102303181	102303348	0	0	0	0
LOC100286891	NC_000015	102303353	102305050	0	0	0	0
DNM1P47	NC_000015	102304832	102306895	0	0	0	0
LOC441736	NC_000015	102311962	102337089	0	0	0	0
OR4F6	NC_000015	102345923	102346861	0	0	0	0.0326144
OR4F15	NC_000015	102358390	102359328	0	0	0	0
OR4F14P	NC_000015	102368944	102369879	0	0	0	0
OR4F13P	NC_000015	102388848	102389784	0	0	0	0
OR4F28P	NC_000015	102416167	102417101	0	0	0	0
LOC100286955	NC_000015	102427779	102427970	0	0	0	0
OR4F4	NC_000015	102462345	102463262	0	0	0	0
OR4G2P	NC_000015	102466830	102467506	0	0	0	0
OR4G6P	NC_000015	102477653	102478796	0	0	0	0
FAM138E	NC_000015	102495088	102496558	0	0	0	0
WASH3P	NC_000015	102501016	102516808	0	0	0	0
LOC100288486	NC_000015	102516761	102519296	0	0.0303652	0.0525505	0
LOC100287029	NC_000016	62304	62940	0	0	0.2833157	0
LOC100287061	NC_000016	63569	64070	0	0	0.0778836	0
WASH4P	NC_000016	64079	68148	0	0	0	0
LOC100132169	NC_000016	72836	75127	0	0	0	0
IL9RP3	NC_000016	78928	86491	0	0	0	0
POLR3K	NC_000016	97000	103625	2.4845106	3.2354949	5.0591071	6.5789625
SNRNP25	NC_000016	103829	107669	2.1852712	3.9941129	1.7640706	5.7809393
RHBDF1	NC_000016	108058	122629	13.058148	8.7588145	5.7104399	5.138265
MPG	NC_000016	127018	135845	29.793805	31.020955	20.153095	17.437026
C16orf35	NC_000016	135800	188672	2.8377413	4.0210053	3.165477	6.1065331
HBZ	NC_000016	202854	204504	0	0	0	0.2599735
HBZP	NC_000016	213121	215155	0	0	0	0
HBM	NC_000016	215973	216767	0	0	0.0766619	0
HBA2	NC_000016	222846	223709	0.2179251	0.2240499	0	0
HBA1	NC_000016	226679	227520	0	0	0	0
HBQ1	NC_000016	230335	231178	0	0	0	0
LUC7L	NC_000016	238970	279449	0.2955213	0.282125	0.1690096	0.2647684
LOC100129478	NC_000016	280529	281471	0.1398141	0.0479145	0.0414608	0
ITFG3	NC_000016	284801	316119	17.239855	15.505009	22.76718	20.105357
RGS11	NC_000016	318310	325914	0.2219608	0.2091824	0.6582082	0.1804496
ARHGDIG	NC_000016	330606	333003	0.0466542	0.0479654	0.0415048	0.0975315
PDIA2	NC_000016	333118	337209	0.0254625	0.0261781	0	0.0532298
AXIN1	NC_000016	337440	402464	2.1540752	2.8305147	3.5831876	3.916929
MRPL28	NC_000016	417396	420540	14.11622	16.568618	11.846669	15.716498
TMEM8	NC_000016	420776	431950	11.203433	7.4275778	8.7905358	11.266538
LOC100134368	NC_000016	432241	442960	0	0	0	0
RPL23AP5	NC_000016	436746	437290	0	0	0	0
NME4	NC_000016	447192	450754	23.436147	26.29342	23.528426	25.637311
DECR2	NC_000016	451858	462487	4.5530701	3.510776	2.4204431	2.669762
RAB11FIP3	NC_000016	475668	572481	6.2148017	4.6625858	3.7181363	5.611348
C16orf10	NC_000016	576847	577407	0	0	0	0
SOLH	NC_000016	577856	604636	5.7807118	6.5336875	5.8019996	7.2107903
C16orf11	NC_000016	610422	615529	0.0396466	0.0203804	0	0
FLJ36208	NC_000016	617032	619495	0.0645033	0.1105269	0.1912797	0.1348454
PIGQ	NC_000016	620004	634109	6.1816301	5.4585013	5.3387846	5.4395318
RAB40C	NC_000016	640176	679272	3.1968469	5.8295584	4.4605952	6.4251276
WFIKKN1	NC_000016	681012	684116	0.2030462	0.2783372	0.2007062	0.2358179

C16orf13	NC_000016	684429	686347	10.777781	12.533015	17.919718	14.947857
LOC100287175	NC_000016	688081	688548	0	0	0.1670836	0.1308755
LOC100130285	NC_000016	690063	691729	0	0	0	0
C16orf14	NC_000016	691849	698474	2.3181485	1.4399106	3.2652912	3.3317175
WDR90	NC_000016	699363	717829	3.3153512	2.1282923	2.8224195	3.078516
LOC100131582	NC_000016	704611	706429	0	0	0	0
RHOT2	NC_000016	718133	724171	4.5908157	5.4641237	3.0159633	4.5771212
RHBDL1	NC_000016	726075	728267	1.0148405	0.2318584	0.326022	0.1375075
LOC100287267	NC_000016	728518	729737	0.1080694	0.0740712	0.0320472	0.0251024
STUB1	NC_000016	730115	732768	11.439708	14.243207	10.982462	11.317059
JMJD8	NC_000016	731667	734439	1.0115411	1.4161301	2.0678438	2.6545561
WDR24	NC_000016	734702	740400	4.2073663	4.2970629	3.9159331	3.6672445
FBXL16	NC_000016	742502	755808	0.0771924	0.3174478	0.0457817	0.0448256
LOC100287301	NC_000016	757313	758380	0	0	0	0.028675
METRN	NC_000016	765173	767480	16.420218	12.909543	18.188245	21.959047
FAM173A	NC_000016	771158	772590	3.2781898	2.9490332	1.9594351	2.6769999
CCDC78	NC_000016	772582	776473	0.4364815	0.2243744	0.2912295	0.4942563
HAGHL	NC_000016	776958	779715	2.522205	2.8621863	2.2014872	1.8073155
NARFL	NC_000016	779767	790997	4.4305193	4.317573	3.2877074	3.7897007
MSLN	NC_000016	811133	818865	0.2116967	0.0217646	0.0941656	0.1032631
MSLNL	NC_000016	819428	832926	0	0.028579	0.0123648	0.0096853
RPUSD1	NC_000016	834974	838383	7.7820528	10.359121	6.4272583	8.9335011
CHTF18	NC_000016	838622	848074	1.6071683	2.3395936	1.8726342	2.1308572
GNG13	NC_000016	848041	850733	0.2233142	0.3673447	0.0397333	0.0933685
LOC388199	NC_000016	855443	863861	0.0363509	0	0.0323388	0.0253308
LMF1	NC_000016	903634	1020984	1.2816829	0.8495728	0.6751306	0.9401344
LOC100287129	NC_000016	1029761	1031708	0	0.0536302	0.0232033	0.054525
SOX8	NC_000016	1031808	1036979	0.0432419	0.0296382	0.0384692	0.0301327
LOC146336	NC_000016	1114081	1128731	0	0	0	0
SSTR5	NC_000016	1128869	1129963	0	0.0412634	0.0714111	0.2517113
C1QTNF8	NC_000016	1138226	1146244	0.0147775	0.0455784	0.144611	0.2471409
GS85	NC_000016	1146811	1160695	0.0228897	0.047066	0.0407266	0.0638018
CACNA1H	NC_000016	1203241	1271772	0.021735	0.0167594	0.0241701	0.0265052
TPSG1	NC_000016	1271651	1275254	0	0	0.0353824	0.0277148
TPSB2	NC_000016	1278336	1280185	0	0	0	0
TPSAB1	NC_000016	1290678	1292555	0	0	0	0.025649
TPSD1	NC_000016	1306273	1308494	0	0	0	0
PRSS29P	NC_000016	1311004	1314005	0	0	0	0
TPSP1	NC_000016	1323624	1326495	0	0	0	0
LOC650474	NC_000016	1336243	1339173	0	0	0	0
UBE2I	NC_000016	1359180	1375390	28.6162	33.714408	35.022406	33.516498
RPS20P2	NC_000016	1378627	1379810	0	0	0	0
BAIAP3	NC_000016	1384663	1399442	0.0388664	0.0399588	0.0345767	0.0541674
C16orf42	NC_000016	1399241	1401873	6.3878244	7.0176878	6.6894506	9.5384795
GNPTG	NC_000016	1401932	1413352	34.383242	19.034393	35.648728	13.709241
UNKL	NC_000016	1413206	1464700	1.2145693	1.6758935	1.1871443	2.4889674
C16orf91	NC_000016	1469944	1479345	1.8465644	2.2359667	1.5332379	1.3153542
CCDC154	NC_000016	1484389	1494490	0.0802341	0	0.1070677	0.0698879
CLCN7	NC_000016	1494935	1525085	5.7418755	5.1332622	5.5430632	8.3067717
RPS3AP2	NC_000016	1527984	1528591	0	0	0	0
C16orf38	NC_000016	1535940	1538468	0.1854356	0.4130691	0.0549895	0.1722919
TELO2	NC_000016	1543422	1560455	3.269435	4.2085985	2.2235013	3.4079944
IFT140	NC_000016	1560428	1662109	0.5589468	0.3688091	0.3488203	0.5871512
LOC100287360	NC_000016	1583669	1584395	0.0786194	0.080829	0	0
TMEM204	NC_000016	1584231	1605243	0	1.4853504	0	0
CRAMP1L	NC_000016	1664641	1727909	0.4582117	0.4122036	0.5197383	0.7743029
LOC100287158	NC_000016	1675629	1691997	0	0	0	0.1696669
HN1L	NC_000016	1728278	1752073	14.235743	18.009567	20.159185	31.531235
MAPK8IP3	NC_000016	1756221	1820318	0.9299146	0.5463142	1.0288833	2.0039038
NME3	NC_000016	1820321	1821710	4.2474622	4.7678738	5.3209705	3.7450541
MRPS34	NC_000016	1821896	1823140	12.093432	17.974255	16.098998	18.411567
EME2	NC_000016	1823229	1826239	0.6254805	0.6430597	0.2928657	1.2846391
SPSB3	NC_000016	1826713	1832581	5.3512096	3.804302	6.0520196	4.5818309
NUBP2	NC_000016	1832933	1839192	8.554323	9.1870738	7.4687101	8.2656148
IGFALS	NC_000016	1840419	1843709	0.0427512	0.0659291	0.0190163	0.0595815
HAGH	NC_000016	1859104	1877195	1.419446	0.7857983	1.4084837	1.4456464

FAHD1	NC_000016	1877225	1890208	3.2773327	3.237952	2.5458218	3.0636019
C16orf73	NC_000016	1883989	1922102	0.0256258	0.026346	0.0227974	0.0178571
HS3ST6	NC_000016	1961464	1968231	0.2480615	0.0425056	0.1471216	0.2880986
SEPX1	NC_000016	1988234	1993294	3.1506413	3.3714023	1.6588579	2.0386715
RPL3L	NC_000016	1994580	2004679	0.0574862	0.0886528	0.0255707	0.1402055
NDUFB10	NC_000016	2009517	2011976	15.195874	19.706964	15.201493	19.025211
RPS2	NC_000016	2012062	2014827	303.90529	464.54485	391.51522	828.17213
SNORA10	NC_000016	2012335	2012467	0	0	0	0
SNORA64	NC_000016	2012974	2013107	0	0	0	0
SNHG9	NC_000016	2014997	2015505	0	0	0	0
SNORA78	NC_000016	2015185	2015311	0	0	0	0
RNF151	NC_000016	2016875	2018976	0	0	0.0491175	0.0769469
TBL3	NC_000016	2022064	2028751	6.4041756	8.0298953	5.7425492	4.0494731
NOXO1	NC_000016	2028918	2031440	0.2879963	0.029609	0.1024838	0.2809622
LOC100287190	NC_000016	2031438	2031762	0	0	0	0.3460438
GFER	NC_000016	2034150	2037749	3.996953	4.5971485	3.3771984	5.2016508
LOC100287224	NC_000016	2039732	2042913	0.0559138	0	0	0
SYNGR3	NC_000016	2039968	2044276	1.1913025	0.4008385	2.505019	1.1018315
ZNF598	NC_000016	2047768	2059763	5.5984133	7.84876	7.2933174	7.8023947
NPW	NC_000016	2069521	2070756	0.0432561	0	0.0384819	0.0904278
SLC9A3R2	NC_000016	2076888	2089027	1.454838	0.7478632	0.3774937	1.0137891
NTHL1	NC_000016	2089816	2097867	1.729921	3.1336193	3.334469	4.9367189
TSC2	NC_000016	2097990	2138713	1.3499723	1.7489314	1.6799735	2.7786422
PKD1	NC_000016	2138711	2185899	8.0541709	5.5065074	10.386933	8.1056935
LOC100287400	NC_000016	2155539	2156370	0.1436217	0.2953164	0	0
RAB26	NC_000016	2198651	2204141	0.5412344	0.5564458	0.4814971	1.074888
SNORD60	NC_000016	2205024	2205106	0	0	0	0
TRAF7	NC_000016	2205799	2228130	7.7781299	8.6251381	9.958311	14.840581
CASKIN1	NC_000016	2227184	2246465	0.0617359	0.0317355	0.0823829	0.1667026
GBL	NC_000016	2255450	2259418	9.4863866	8.2377056	6.2937545	9.8223695
C16orf79	NC_000016	2259254	2261011	0.5420131	0.2786232	0.8438324	0.5980192
LOC100287254	NC_000016	2260787	2265483	0.361479	0.5965774	0.6939395	0.888254
PGP	NC_000016	2261603	2264822	3.1531735	4.1062722	2.7303466	1.6113217
E4F1	NC_000016	2273567	2285743	1.1273157	1.1238779	1.0180866	1.1307281
LOC100287438	NC_000016	2284601	2299729	0.0810853	0.0416821	0.2524751	0.1977621
DNASE1L2	NC_000016	2286468	2288712	0.1657173	0.1703748	0.1474267	0.0692871
LOC100287474	NC_000016	2287832	2288711	0.0823001	0.0846131	0	0
DCI	NC_000016	2289896	2301603	9.6096717	6.9816918	13.48847	12.083285
RNPS1	NC_000016	2303121	2318114	26.046539	33.189738	29.625086	30.358832
LOC100287286	NC_000016	2303318	2312343	0.1870138	0.6408993	0.3327452	0.8253513
ABCA3	NC_000016	2325879	2390747	0.4246847	0.0818663	3.4416248	2.913132
RPS16P7	NC_000016	2382248	2382734	0	0	0	0
ABCA17P	NC_000016	2390923	2476700	0	0	0	0
CCNF	NC_000016	2479395	2508859	4.6541864	9.7807784	6.520821	10.86551
C16orf59	NC_000016	2510115	2514964	4.1032747	7.4102999	3.6263713	6.282991
LOC729652	NC_000016	2514951	2518208	0	0	0	0
NTN3	NC_000016	2521500	2524146	0.1106451	0.2047586	0.0196866	0.200465
TBC1D24	NC_000016	2525147	2553499	1.7712899	1.7170109	1.2966489	1.9325695
ATP6V0C	NC_000016	2563953	2570219	29.392642	25.21672	26.041197	37.405501
AMDHD2	NC_000016	2570427	2579732	11.846663	7.1592373	5.6294325	8.8391332
CEMP1	NC_000016	2580036	2594077	6.6158629	2.0081512	3.531393	7.2226415
PDPK1	NC_000016	2587970	2653189	1.1661222	1.0615227	1.2265267	1.3712632
LOC652276	NC_000016	2653385	2680495	0	0	0	0
PDPK2	NC_000016	2666022	2693397	0	0	0	0
LOC100287510	NC_000016	2666122	2693832	0.0150405	0	0	0.0104808
FLJ42627	NC_000016	2688983	2696130	0	0	0	0
KCTD5	NC_000016	2732495	2759031	14.863531	13.666924	9.5402505	10.332633
PRSS27	NC_000016	2762423	2770552	0.2404828	0.154526	0.3476528	0.0418945
LOC100128788	NC_000016	2787077	2802601	0	0	0	0
SRRM2	NC_000016	2802330	2821413	4.9196834	7.9323368	11.629363	11.253898
LOC100132779	NC_000016	2811342	2818171	0.1930376	0.0330772	0.1202121	0.0313871
LOC100287346	NC_000016	2816496	2820784	0.1469139	0.1294653	0.0933562	0.248626
TCEB2	NC_000016	2821415	2827297	52.604702	75.123111	75.075225	87.172633
PRSS33	NC_000016	2833954	2836708	0.0518869	0	0.0692401	0.0180784
TESSP1	NC_000016	2848486	2855133	0	0.0472136	0	0
PRSS21	NC_000016	2867164	2871720	0.0377238	0	0.0335601	0

LOC100130566	NC_000016	2872655	2872995	0	0	0	0
ZG16B	NC_000016	2880173	2882285	0	0	0.0472193	0
MGC52282	NC_000016	2889574	2892752	0	0	0	0
PRSS22	NC_000016	2902728	2908171	0.0634174	0.0325999	0	0.0220959
FLYWCH2	NC_000016	2933196	2949383	11.425684	15.266446	9.5935604	11.74898
FLYWCH1	NC_000016	2961980	3001209	1.590375	1.2240708	1.1442436	1.2535792
KREMEN2	NC_000016	3014217	3018381	0.4065318	0.7699216	0.1713136	0.3578369
PAQR4	NC_000016	3019342	3023485	8.4239553	10.037026	7.7846565	11.69479
PKMYT1	NC_000016	3022792	3030474	4.1835928	5.9457393	2.9373346	8.2599998
CLDN9	NC_000016	3062206	3064506	0.0512516	0.026346	0	0.0357141
CLDN6	NC_000016	3064713	3068188	0.064158	0.0329806	0.0856151	0
TNFRSF12A	NC_000016	3070313	3072384	61.689193	101.65172	15.707154	31.425335
HCFC1R1	NC_000016	3072626	3074287	20.216187	32.848333	32.450979	25.418649
THOC6	NC_000016	3074032	3077756	2.3831083	5.3456419	2.2026798	3.1056215
CCDC64B	NC_000016	3077868	3085542	0	0.0748483	0.1079447	0.0507314
LOC100128770	NC_000016	3086125	3086751	0	0	0	0
MMP25	NC_000016	3096682	3110727	0.0741951	0.1144205	0.099009	0.0430851
IL32	NC_000016	3115313	3119668	4.0890643	7.8777432	3.1461578	0.8471257
LOC100132433	NC_000016	3122714	3124525	0	0	0	0
ZSCAN10	NC_000016	3138895	3142861	0	0	0.015874	0.0497359
MGC3771	NC_000016	3160461	3165599	0	0	0	0
ZNF205	NC_000016	3162563	3170518	1.0880387	1.8643637	1.6891667	1.6501755
ZNF213	NC_000016	3185057	3192805	0.2104045	0.3920762	0.3509656	0.3024001
LOC100287385	NC_000016	3185134	3192307	0.085171	0.5545766	0.0252568	0.1187011
TRNAP24P	NC_000016	3202609	3202680	0	0	0	0
TRNAP26P	NC_000016	3220961	3221031	0	0	0	0
TRNAK37P	NC_000016	3246153	3246225	0	0	0	0
OR1F1	NC_000016	3254247	3255185	0	0	0.1249124	0.0326144
OR1F2P	NC_000016	3265562	3266546	0	0	0	0
ZNF200	NC_000016	3272325	3285457	0.8530138	0.7040607	0.7481765	0.8790629
MEFV	NC_000016	3292028	3306627	0.0251205	0.0387397	0.145261	0.0350099
FLJ39639	NC_000016	3313059	3317567	0.0707132	0.1635763	0.1258168	0.1601462
ZNF263	NC_000016	3333487	3341460	0.347734	0.3162563	0.368845	1.239534
TIGD7	NC_000016	3348832	3351400	0.256607	0.2814069	0.2282847	0.3933908
ZNF75A	NC_000016	3355433	3368576	49.058972	27.271714	13.327042	7.8869155
LOC646140	NC_000016	3373177	3373930	0	0	0	0
OR2C1	NC_000016	3405889	3406924	0.0424211	0.0436133	0	0.0886821
LOC100287461	NC_000016	3419950	3420905	0	0	0	0
ZNF434	NC_000016	3432085	3451025	0.7549746	1.3583381	0.9428881	1.3354754
ZNF174	NC_000016	3451190	3459364	1.3256626	1.8804852	1.5376286	2.0814159
ZNF597	NC_000016	3486110	3493490	0.9914999	0.662588	0.2425681	0.5354604
NAT15	NC_000016	3493668	3536963	3.5584381	4.2838243	3.9232851	5.9130388
C16orf90	NC_000016	3543484	3545421	0	0	0	0.0677542
RPL18P12	NC_000016	3546168	3546809	0	0	0	0
CLUAP1	NC_000016	3550963	3587669	1.0126321	1.3454115	1.0671792	1.4438528
NLRC3	NC_000016	3589033	3627392	0.9200223	0.4164694	0.9223141	1.4257481
BTBD12	NC_000016	3631182	3661585	0.3675911	0.5885676	0.5307362	0.9322258
DNASE1	NC_000016	3702940	3708096	0.6099981	0.437541	0.5426712	0.8501419
TRAP1	NC_000016	3708038	3767598	4.6321743	5.726642	7.0838795	13.92525
LOC100131668	NC_000016	3725473	3725974	0	0	0	0
CREBBP	NC_000016	3775055	3930121	2.2196077	3.4562179	3.9109068	4.5169969
LOC100287698	NC_000016	3779321	3781399	0	0.1179723	0	0
ADCY9	NC_000016	4012652	4166186	1.6498365	1.7429973	2.0092859	2.0733736
LOC791082	NC_000016	4180492	4181095	0	0	0	0
SRL	NC_000016	4239375	4292081	0.0104291	0.0214444	0.009278	0.0072674
TFAP4	NC_000016	4307187	4323001	0.4928774	0.8445496	1.5346708	1.0733018
GLIS2	NC_000016	4382225	4389598	4.8040577	2.7195408	2.4482146	3.1327473
Magmas	NC_000016	4390252	4401300	8.6864411	18.391678	12.241899	8.8698278
CORO7	NC_000016	4404543	4466639	1.5007933	1.0543651	1.5687982	1.5948642
VASN	NC_000016	4421849	4433529	1.6132102	6.6986084	0.4598074	0.4147346
DNAJA3	NC_000016	4475858	4506776	3.7123368	4.6166737	4.5284528	2.3270841
NMRAL1	NC_000016	4511695	4524896	11.487203	10.284034	12.343578	17.38108
HMOX2	NC_000016	4524719	4560348	10.613835	11.837252	6.9316764	13.794733
C16orf5	NC_000016	4560676	4588816	9.2480415	6.8220016	7.3290112	16.976592
LOC342346	NC_000016	4606491	4650318	0.0128279	0.0263768	0.022824	0.0268169
FAM100A	NC_000016	4658883	4664927	6.1539957	6.8062692	5.5577162	6.9090072

MGRN1	NC_000016	4674826	4740975	4.1891023	4.3696601	3.5032579	6.9264362
NUDT16L1	NC_000016	4743716	4745845	1.3723363	1.5141429	0.9528729	1.0029473
ANKS3	NC_000016	4746513	4784163	1.4033057	1.5615601	1.0868595	1.4035444
C16orf71	NC_000016	4784289	4799397	0.1461693	0.2003699	0.0866908	0.1923958
ZNF500	NC_000016	4800815	4817166	0.189199	0.2223046	0.2644977	0.2919346
SEPT12	NC_000016	4827674	4838347	0	0.0700518	0.151541	0.0237402
LOC440335	NC_000016	4845407	4846762	0.0430022	0.0884215	0	0.0599313
ROGDI	NC_000016	4846969	4852674	3.4362815	4.1526583	1.9843758	2.9196557
N-PAC	NC_000016	4853204	4897303	2.1550178	2.3487621	1.9800214	4.258926
UBN1	NC_000016	4897912	4932363	2.075171	2.2401687	2.9018851	3.5880409
PPL	NC_000016	4932508	4987136	0.3522622	0.057946	1.1908524	1.2764457
SEC14L5	NC_000016	5008318	5069156	0.1089176	0.0349933	0.3996963	0.2229506
NAGPA	NC_000016	5074845	5083936	2.1744699	2.3176234	2.0587007	2.2659352
C16orf89	NC_000016	5094123	5116111	0.0469532	0.0482729	0.0417709	0.0654378
ALG1	NC_000016	5121810	5137380	4.4573829	5.7197087	2.5939158	5.2390806
FAM86A	NC_000016	5134301	5147789	1.8697436	1.6368039	0.494072	1.2771116
LOC650177	NC_000016	5161084	5161473	0.5634389	0	0	0.0785253
LOC100127948	NC_000016	5179270	5179443	0	0	0	0
LOC100287538	NC_000016	5289505	5292652	0	0	0	0
LOC100130126	NC_000016	5310677	5311045	0	0	0	0
RPS3AP48	NC_000016	5320151	5320999	0	0	0	0
NPM1P3	NC_000016	5415697	5417009	0	0	0	0
A2BP1	NC_000016	6069132	7763340	0	0.0417206	0.0072202	0.0735223
RPS26P51	NC_000016	7176294	7176640	0	0	0	0
LOC100131080	NC_000016	8558054	8558646	0	0	0	0
TMEM114	NC_000016	8619502	8622304	0	0	0	0.0424167
C16orf68	NC_000016	8715527	8740079	4.1428906	7.655277	2.8638344	3.6866892
ABAT	NC_000016	8768444	8878432	0.044467	0.1371503	0.0395591	0.0154932
RNU7-63P	NC_000016	8799213	8799474	0	0	0	0
TMEM186	NC_000016	8889037	8891505	0.4249138	1.0609362	0.9180368	0.9728898
PMM2	NC_000016	8891670	8943194	10.878275	13.167957	7.9103986	5.2865158
LOC100132944	NC_000016	8891725	8943111	1.3366251	1.4841263	1.0464069	3.4276019
CARHSP1	NC_000016	8946802	8962863	11.282061	8.8916911	15.349699	27.787013
USP7	NC_000016	8985951	9057341	3.0776755	5.9109107	4.0961419	5.9642686
LOC100287598	NC_000016	9052792	9060847	0	0	0	0.0393131
C16orf72	NC_000016	9185537	9213555	4.9150448	7.7103619	5.4042878	5.9187061
RPL21P119	NC_000016	9250199	9250778	0	0	0	0
GRIN2A	NC_000016	9847265	10276611	0.1575419	1.2590843	0.0661102	0.3107022
IMPDH1P11	NC_000016	10205166	10215399	0	0	0	0
LOC100287888	NC_000016	10274201	10276258	0	0	0	0
LOC100287628	NC_000016	10479082	10481598	0.1474773	0.1516222	0	0
ATF7IP2	NC_000016	10522725	10577495	0.1832704	0.1256141	0	0.04257
EMP2	NC_000016	10622279	10674539	12.596627	11.180297	12.142054	15.268107
TEKT5	NC_000016	10721361	10788802	0.027314	0	0.0728979	0.0761339
LOC100287656	NC_000016	10812613	10813670	0	0	0	0
LOC100287687	NC_000016	10813857	10815345	0	0	0	0
LOC100287923	NC_000016	10818474	10819153	0	0	0	0
NUBP1	NC_000016	10837698	10863208	3.5315543	5.1344775	4.1890249	6.4879004
FAM18A	NC_000016	10860533	10912621	0	0	0.0234187	0.0366875
LOC729945	NC_000016	10933455	10935410	0	0	0	0
CIITA	NC_000016	10971055	11018840	0.4815986	0.038834	0.9997014	1.2107816
DEXI	NC_000016	11022748	11036257	8.5202768	9.1209665	6.564015	6.2841189
CLEC16A	NC_000016	11038345	11276046	0.7206719	0.8982914	0.9418366	2.4531901
RPL7P46	NC_000016	11120818	11122418	0	0	0	0
LOC100287963	NC_000016	11217318	11220201	0	0	0	0.0358605
SOCS1	NC_000016	11348274	11350039	0.9758242	1.932185	0.7395099	1.1585069
TNP2	NC_000016	11361714	11363160	0.0736151	0	0	0
PRM3	NC_000016	11367141	11367452	0	0	0	0.0981567
PRM2	NC_000016	11369493	11370337	0.0643459	0	0	0.0896775
PRM1	NC_000016	11374693	11375192	0	0	0	0
C16orf75	NC_000016	11439311	11445617	1.6879558	1.7038434	1.7473772	1.6039566
LOC388210	NC_000016	11452531	11484311	5.0674062	5.6518721	2.3770008	1.8404888
LOC400499	NC_000016	11490387	11602817	0	0	0	0
LITAF	NC_000016	11641578	11681322	5.6722602	4.5393407	6.0107091	5.2884577
SNN	NC_000016	11762301	11773008	3.4900856	1.3248646	1.8987517	2.1888276
TXNDC11	NC_000016	11772943	11836648	12.389738	14.428438	11.124906	9.4276783

ZC3H7A	NC_000016	11844442	11876408	1.6573455	1.7156766	1.4642521	2.2540548
BCAR4	NC_000016	11913687	11922689	0	0	0	0
RSL1D1	NC_000016	11928053	11945442	4.2861717	6.0426313	4.9623541	5.6635457
GSPT1	NC_000016	11961985	12010519	10.786368	14.762779	14.031671	14.741226
COX6CP1	NC_000016	11997658	11998050	0	0	0	0
LOC729978	NC_000016	12021116	12036392	0.4025024	0.2069074	0.8056739	0.5609594
TNFRSF17	NC_000016	12058964	12061925	0	0	0	0.0616195
RUNDC2A	NC_000016	12070602	12147141	0.254149	0.4421863	0.4695882	0.3405792
SNX29	NC_000016	12146055	12668146	0.897415	0.8839249	0.5694633	1.1063964
RPS23P6	NC_000016	12175094	12175389	0	0	0	0
CPPED1	NC_000016	12753656	12897744	4.5500693	3.5305279	3.0295363	2.4428114
RPL35AP34	NC_000016	12974993	12975397	0	0	0	0
LOC729993	NC_000016	12995477	13334273	0.013319	0.0365155	0.0078993	0.0309373
ERCC4	NC_000016	14014014	14046205	1.1440451	1.3700041	1.2375209	0.9648128
RPS26P52	NC_000016	14016189	14016536	0	0	0	0
MKL2	NC_000016	14165196	14360630	1.2318128	1.1774688	1.6030274	2.6176929
CGI-148P	NC_000016	14294044	14296076	0	0	0	0
PARN	NC_000016	14529558	14724124	6.58224	8.9544754	7.0751605	8.7357515
BFAR	NC_000016	14726796	14762708	6.2535033	8.4294726	12.671938	14.198807
PLA2G10	NC_000016	14766405	14788526	0	0	0.1176456	0
LOC642778	NC_000016	14801480	14819207	0.1698035	0.0793527	0.0686645	0.096812
LOC100288132	NC_000016	14820928	14827204	0	0	0	0
LOC642799	NC_000016	14840602	14858327	0.04631	0.1110937	0.0411987	0.0430276
ABCC6P2	NC_000016	14916289	14918559	0	0	0	0
NOMO1	NC_000016	14927643	14990014	14.60112	17.203099	20.685715	19.195532
PKD1P3	NC_000016	15005334	15029565	0	0	0	0
LOC100288162	NC_000016	15020169	15021134	0.5891184	0.1817027	0.262048	0.2873648
LOC100287752	NC_000016	15023823	15024914	7.0650955	3.6032333	6.6812296	3.8765669
NPIP	NC_000016	15031300	15045931	10.129629	8.4872633	15.717079	10.199029
PDXDC1	NC_000016	15068833	15131552	7.45071	7.3221669	9.0847497	11.605539
NTAN1	NC_000016	15131714	15149833	22.132203	24.989022	24.365659	21.095914
RRN3	NC_000016	15153879	15188158	3.84811	4.7402768	3.0685222	3.0739333
PKD1P6	NC_000016	15219096	15248481	0	0	0	0
LOC100288226	NC_000016	15219898	15222223	1.559321	1.0547013	1.8982945	1.0151103
LOC100287779	NC_000016	15223776	15224848	1.5390431	2.1097308	2.1805387	2.0257702
LOC100288191	NC_000016	15226660	15228185	0.6172504	0.3384524	0.4026903	0.3727747
LOC100288442	NC_000016	15457488	15459600	0	0	0	0
LOC100288332	NC_000016	15458459	15476592	0.218784	0.1405831	0.158142	0.2096289
MPV17L	NC_000016	15489611	15503543	0.723271	0.5870516	1.015961	0.8090591
C16orf45	NC_000016	15528325	15682116	6.4364831	10.82318	2.3872553	6.3681963
KIAA0430	NC_000016	15688244	15737009	0.4151425	0.4560437	0.6829932	1.1650769
NDE1	NC_000016	15737124	15820210	2.5348418	3.3055522	2.9677054	4.3968302
MYH11	NC_000016	15796992	15950887	0.0886308	0.0781044	0.0337922	0.0926422
LOC100287815	NC_000016	15931771	15950888	0.0983182	0	0	0
C16orf63	NC_000016	15959577	15982447	5.2542553	6.1851057	10.686668	11.909675
RPL15P20	NC_000016	16025554	16026186	0	0	0	0
ABCC1	NC_000016	16043434	16236931	9.3466989	8.0537142	8.2436062	8.6639852
RPL17P40	NC_000016	16187890	16188370	0	0	0	0
ABCC6	NC_000016	16243422	16317328	0.2757104	0.097186	0.6447349	0.1317435
NOMO3	NC_000016	16326389	16388667	2.067553	2.6282813	3.9777129	2.4698627
LOC100288299	NC_000016	16416376	16418897	15.967173	9.2372609	17.128022	10.339474
PKD1P1	NC_000016	16418782	16422385	0	0	0	0
LOC100288368	NC_000016	16418820	16419785	0.4712947	0.1211351	0.2096384	0.2873648
LOC339047	NC_000016	16425734	16444442	13.721394	6.0750792	19.232868	13.243726
PKD1P2	NC_000016	16450274	16471364	0	0	0	0
LOC100288408	NC_000016	16462546	16464073	0.1642925	0.2111374	0.1461591	0.1717283
LOC100133127	NC_000016	16635074	16635704	0	0	0	0
LOC441750	NC_000016	17046357	17047325	0	0	0	0
XYLT1	NC_000016	17196181	17564738	1.8123467	3.4942605	2.4582553	4.6613059
RPL7P47	NC_000016	18026574	18027202	0	0	0	0
LOC100288502	NC_000016	18055454	18056227	0	0	0.0505137	0.0791341
LOC100133137	NC_000016	18264213	18264842	0	0	0	0
PKD1P4	NC_000016	18428257	18446140	0	0	0	0
PKD1P5	NC_000016	18468378	18495871	0	0	0	0
NOMO2	NC_000016	18511182	18573434	8.111771	5.864387	8.0530138	5.4869874
ABCC6P1	NC_000016	18582570	18609611	0	0	0	0

RPS15A	NC_000016	18794277	18801656	59.973554	126.81308	162.2945	136.65576
ARL6IP1	NC_000016	18802991	18812857	60.525878	52.587572	74.844898	70.962063
SMG1	NC_000016	18816175	18937726	3.0037447	3.6703604	5.1959728	6.9218134
LOC730060	NC_000016	18937382	18938515	0	0	0.036235	0.1986785
TMC7	NC_000016	18995327	19074497	0.1850192	0.317032	0.7900715	0.7305963
COQ7	NC_000016	19078927	19091354	1.4943757	1.5363753	1.0877221	2.0116806
ITPRIPL2	NC_000016	19125282	19132952	2.0051989	2.6505711	2.5484008	2.8425125
SYT17	NC_000016	19179638	19278554	0.0919419	0.0236315	0	0.0160172
LOC728276	NC_000016	19297105	19315716	0	0	0	0
TMC5	NC_000016	19422057	19510435	0.0160278	0.0247174	0.1211996	0.5863625
GDE1	NC_000016	19513015	19533450	9.0978975	11.461231	11.554882	12.491864
CP110	NC_000016	19535179	19564730	0.1996558	0.3530595	0.2415623	0.7568569
C16orf62	NC_000016	19567040	19712444	3.8748723	3.1182911	1.3766748	2.0359074
C16orf88	NC_000016	19717674	19729492	0.7888144	1.853678	1.0426018	0.958009
IQCK	NC_000016	19727778	19868861	0.4473978	1.0437826	0.8113434	0.6475111
GPRC5B	NC_000016	19870293	19896151	0.3243982	5.2886021	0.0274851	0.0538223
GPR139	NC_000016	20043043	20085100	0	0	0	0.0247374
LOC100131046	NC_000016	20121485	20170064	0	0	0	0
LOC100129329	NC_000016	20250243	20250569	0	0	0	0
GP2	NC_000016	20321811	20338835	0.0361565	0	0.0804146	0.0377929
UMOD	NC_000016	20344373	20364037	0.0569524	0.0195177	0	0.0264578
PDILT	NC_000016	20370492	20416033	0	0	0.0183816	0
ACSM5	NC_000016	20420856	20452290	0.0543018	0.0558279	0.0322056	0.0630661
ACSM2A	NC_000016	20462859	20498991	0	0	0	0.0103323
LOC100129488	NC_000016	20499024	20499949	0	0.0487942	0	0
ACSM2B	NC_000016	20548080	20587695	0.0179821	0.0184875	0	0.1879596
ACSM1	NC_000016	20634559	20702578	0	0.0660898	0.0190627	0.0149317
LOC646810	NC_000016	20722692	20744300	0	0	0	0
THUMPD1	NC_000016	20744986	20753199	0.7663075	1.0334066	1.035873	2.3509587
LOC730079	NC_000016	20774994	20781570	0	0	0.0542269	0
ACSM3	NC_000016	20775312	20808479	0.1726469	0.2169434	0.2047886	0.1470422
ERI2	NC_000016	20791515	20817795	1.817683	1.6861883	1.6635285	2.0964975
LOC81691	NC_000016	20817767	20860990	2.8971637	3.4180527	3.7604647	3.2323809
DCUN1D3	NC_000016	20869396	20911561	4.2906224	3.7495292	3.3535569	3.4597143
LYRM1	NC_000016	20911557	20936328	3.4174364	4.9188773	3.3037342	3.4768524
DNAH3	NC_000016	20944476	21170762	0.117423	0.0438993	0.0538142	0.0347137
TMEM159	NC_000016	21169912	21191937	5.7614357	4.5728351	9.6565041	5.0907637
ZP2	NC_000016	21208773	21222868	0.0969732	0	0.051762	0.0675748
ANKS4B	NC_000016	21245016	21263750	0.0453698	0	0.0403622	0.021077
CRYM	NC_000016	21269842	21314417	0.6862038	0.0641354	0.4162267	0.4781741
NCRNA00169	NC_000016	21312170	21329912	0	0	0	0
RUNDC2B	NC_000016	21391313	21403424	0.0359936	0.0370052	0	0.0250818
NPIPL3	NC_000016	21413450	21436658	0.2519079	0.737119	1.1032823	1.4448245
LOC100190986	NC_000016	21443324	21445776	0	0	0	0
IMAA	NC_000016	21529214	21531765	0	0	0	0
LOC646828	NC_000016	21544266	21567065	0	0	0	0
METTL9	NC_000016	21610856	21668792	15.020804	13.788362	21.488365	21.403866
IGSF6	NC_000016	21652606	21663972	0	0.0879055	0	0
OTOA	NC_000016	21689835	21772050	0.0238525	0.0858301	0.1060992	0.0581748
LOC730092	NC_000016	21807951	21830495	0	0	0	0
LOC440345	NC_000016	21845884	21871554	0	0	0	0
LOC100271836	NC_000016	21890266	21927347	0	0	0	0
LOC342293	NC_000016	21950237	21956260	0.1550202	0	0	0.0540121
UQCRC2	NC_000016	21964609	21994668	27.408575	33.388213	35.827757	31.777427
C16orf65	NC_000016	21995486	22012428	0	0.0439527	0.0380327	0
C16orf52	NC_000016	22019530	22098855	0	0	0	0
VWA3A	NC_000016	22103863	22168287	0.0286619	0	0.0169989	0.0266303
LOC100288072	NC_000016	22176904	22201714	0	0.0356055	0.1540487	0.0482662
EEF2K	NC_000016	22217592	22300066	0.4209828	0.7315176	0.9125579	1.7229593
POLR3E	NC_000016	22308741	22345346	1.3649566	2.3042149	2.6984516	2.9591523
CDR2	NC_000016	22357257	22385938	2.3747009	2.3076645	2.8795024	2.164823
LOC100288103	NC_000016	22386175	22387593	0	0	0.1653174	0.021582
LOC100129767	NC_000016	22393338	22418919	0	0	0	0
LOC100131998	NC_000016	22432611	22448491	0	0	0	0
LOC641298	NC_000016	22448329	22503541	0	0	0	0
LOC100132247	NC_000016	22524844	22547842	0.5296355	0.0473496	0.4506924	0.5536067

LOC653786	NC_000016	22557019	22588186	0	0	0	0
HS3ST2	NC_000016	22825860	22927659	0	0.1157362	0	0.015689
USP31	NC_000016	23072728	23160591	1.6432317	2.0822039	3.8410489	3.9991545
SCNN1G	NC_000016	23194040	23228200	0	0.0516529	0.0111739	0.0087525
SCNN1B	NC_000016	23313591	23392620	0.0338454	0	0	0
COG7	NC_000016	23399816	23464503	3.4395574	3.8141833	3.9017393	3.8725923
GGA2	NC_000016	23475643	23521808	5.14396	4.791915	4.2821861	8.9110955
LOC100288178	NC_000016	23505925	23521912	0.1209862	0	0	0.021077
EARS2	NC_000016	23533334	23568696	1.120336	1.5281615	1.1249678	2.6280814
LOC100288661	NC_000016	23548381	23549453	0	0	0	0
UBFD1	NC_000016	23568862	23585710	7.3796666	8.283471	9.3640692	11.992829
NDUFAB1	NC_000016	23592335	23607639	14.298	15.37726	23.270965	18.733059
PALB2	NC_000016	23614483	23652678	3.8013379	4.6764487	3.4684878	4.04508
DCTN5	NC_000016	23652807	23681182	9.5223373	9.0285221	10.871547	18.754628
PLK1	NC_000016	23690201	23701688	12.076139	20.268995	10.36473	21.772043
ERN2	NC_000016	23701625	23724821	0.0364111	0.0249563	0.0107974	0.059203
CHP2	NC_000016	23765948	23770256	0.0185749	0.019097	0.0495743	0.0258875
LOC100288205	NC_000016	23846794	23880647	0.0402456	0	0	0.0280448
PRKCB	NC_000016	23847300	24231932	0.0164518	0.0056381	0.0097573	0.0343928
CACNG3	NC_000016	24266876	24373737	0.0325422	0	0.0144752	0
RNU7-24P	NC_000016	24411383	24411437	0	0	0	0
RBBP6	NC_000016	24550908	24584184	1.0779641	1.1144866	2.4028544	2.9751329
LOC100133134	NC_000016	24618167	24618749	0	0	0	0
FLJ45256	NC_000016	24674112	24674720	0	0	0	0
TNRC6A	NC_000016	24741049	24837548	2.0505349	2.5641299	5.6211744	7.9843563
SLC5A11	NC_000016	24857552	24922944	0	0.019227	0.0332745	0.0390956
ARHGAP17	NC_000016	24930710	25026675	7.0468716	6.102356	7.5498747	15.954858
LOC554206	NC_000016	25043065	25044096	0	0	0	0
LCMT1	NC_000016	25123047	25189551	2.6946361	4.6172819	6.5638249	8.2709525
AQP8	NC_000016	25228285	25240253	0.1343983	0	0.0597822	0.046827
ZKSCAN2	NC_000016	25247322	25268855	0.6414	0.6784485	0.4828215	0.9110966
LOC100288780	NC_000016	25269273	25271191	0.2290163	0.1412717	0.1018696	0.1915052
CYCSP39	NC_000016	25465660	25465968	0	0	0	0
HS3ST4	NC_000016	25703347	26093929	0.9367296	0.9630565	0.5208379	0.0271979
HMGN2L3	NC_000016	26043026	26044239	0	0	0	0
EPF8	NC_000016	26073951	26074420	0	0	0	0
LOC100288242	NC_000016	26353992	26369990	0	0	0	0
C16orf82	NC_000016	27078219	27080487	0	0.0199134	0.0172312	0.0404912
LOC124199	NC_000016	27144804	27146500	0	0	0	0
JMJD5	NC_000016	27214807	27233089	0.1644769	0.388929	0.2194848	0.3209194
LOC100288274	NC_000016	27214807	27227217	0.1071908	0	0.0953599	0
NSMCE1	NC_000016	27236315	27280113	10.428394	10.083302	11.633551	9.66039
IL4R	NC_000016	27325251	27376099	3.9828796	3.4747463	3.3306833	4.3693185
IL21R	NC_000016	27413723	27462115	0.1594104	0.3151744	0.1745427	0.0939938
GTF3C1	NC_000016	27471934	27561251	1.539765	2.4985334	2.3215383	4.4383882
KIAA0556	NC_000016	27561468	27791692	1.7252816	1.5759272	1.4640188	3.0888447
LOC100288847	NC_000016	27789906	27791692	0	0.0252845	0	0
GSG1L	NC_000016	27798850	28074830	0.1138102	0.027002	0.0389418	0.0244023
XPO6	NC_000016	28109316	28223190	8.1694814	10.422223	19.433842	25.022091
LOC647001	NC_000016	28250805	28252048	0	0	0	0
SBK1	NC_000016	28303840	28335170	0.0176075	0.0633581	0.0469923	0.0674827
LOC388237	NC_000016	28354364	28356203	0	0	0	0
LOC100288315	NC_000016	28357497	28358955	0	0.0327416	0	0
EIF3CL	NC_000016	28390900	28415162	6.7513006	5.2242846	7.1715155	10.80347
LOC100288341	NC_000016	28391834	28415245	3.9839757	3.6479517	5.4548303	9.3045841
CDC37P2	NC_000016	28425095	28426339	0	0	0	0
NPIPL1	NC_000016	28467677	28482617	0.0742369	0	0	0.0258656
LOC100288419	NC_000016	28486637	28495439	0.7938104	2.003205	0.3851977	0.1508615
CLN3	NC_000016	28488600	28503623	9.5986124	6.9595798	12.007073	15.670241
LOC100288452	NC_000016	28502872	28503623	0.2296946	0.0787167	0.0681142	0
APOB48R	NC_000016	28505993	28510282	0.0353187	0.0242076	0.062841	0.0246115
IL27	NC_000016	28510683	28518155	0	0	0.0374498	0
NUPR1	NC_000016	28548662	28550495	4.8188851	3.5671107	4.0297929	2.6863928
CCDC101	NC_000016	28565249	28603111	1.641854	1.9235332	1.3927022	2.634112
SULT1A2	NC_000016	28603266	28608391	0.1888892	0.4531287	0.3640891	0.4168142
SULT1A1	NC_000016	28616913	28634866	1.1077694	0.372732	1.4155397	0.6175502

LOC728734	NC_000016	28653686	28670019	0.0166597	0	0.0296418	0.0232183
LOC100132542	NC_000016	28670345	28675457	0	0	0	0
CDC37P1	NC_000016	28711497	28712932	0	0	0	0
LOC100288546	NC_000016	28722702	28746119	2.9091696	3.3393902	3.6434107	7.4987652
EIF3C	NC_000016	28722782	28747051	19.447376	30.136862	19.435127	43.484162
LOC100288583	NC_000016	28760376	28764498	0.0847876	0.0581137	0.0502863	0.0590834
LOC728741	NC_000016	28763382	28784144	0.1239724	0.3505059	0.165434	0.2591668
RPS15AP33	NC_000016	28825263	28825648	0	0	0	0
ATXN2L	NC_000016	28834414	28848558	8.4233272	7.7806087	10.931561	13.184056
TUFM	NC_000016	28853732	28857729	8.7429606	11.95582	11.232763	15.556433
SH2B1	NC_000016	28875314	28885526	3.7090933	2.3066043	2.6800102	6.1148892
LOC100289092	NC_000016	28884692	28901633	0.1452034	0.3980916	0.301413	0.3372784
ATP2A1	NC_000016	28889809	28915830	0.0745728	0.0766687	0.132684	0.1039306
RABEP2	NC_000016	28915742	28936532	3.1611886	3.3887551	1.3204002	3.2371034
LOC100288650	NC_000016	28931022	28936148	0	0	0	0.0458456
CD19	NC_000016	28943260	28950663	0.0225376	0	0	0.0157051
NFATC2IP	NC_000016	28962318	28977786	4.1828469	3.9391256	3.6707542	4.5340934
LOC100289031	NC_000016	28985919	28986714	0.2337672	0	0	0.1221737
SPNS1	NC_000016	28986096	28995869	10.14801	9.4940271	7.6499078	7.2237625
LAT	NC_000016	28996147	29002104	0.3811229	0.416324	0.466204	0.6971517
LOC730153	NC_000016	29049949	29063562	0	0.0610587	0.0528345	0.0827699
LOC100289169	NC_000016	29060841	29064063	0.0136358	0	0	0.009502
LOC653390	NC_000016	29086163	29128039	0	0	0	0
LOC728804	NC_000016	29138391	29151010	0.1503973	0.0994013	0.0191139	0.0673732
LOC100129184	NC_000016	29140551	29141569	0	0	0	0
LOC728809	NC_000016	29143654	29145864	0	0.0204357	0	0
LOC100288673	NC_000016	29223380	29230162	0	0	0	0
RUNDC2C	NC_000016	29302541	29385597	0	0	0	0
LOC728888	NC_000016	29392617	29424055	0.5274686	0.5422931	0.6289956	1.0479402
LOC100288704	NC_000016	29442596	29451192	2.3303962	1.614623	2.073185	3.2654769
BOLA2	NC_000016	29454570	29466285	9.4330033	9.6534271	8.6238943	9.087501
LOC606724	NC_000016	29460666	29461236	0	0	0	0
GIYD2	NC_000016	29465875	29469540	2.8060472	5.5260276	5.3794312	2.7264955
SULT1A4	NC_000016	29466401	29476282	1.7714759	1.5534305	2.8042712	2.1784146
LOC388242	NC_000016	29476288	29478899	0	0	0	0
LOC440353	NC_000016	29495130	29516951	0	0	0	0
LOC440354	NC_000016	29538929	29577540	0	0	0	0
LOC100132767	NC_000016	29606400	29607450	0.0836313	0.0429909	0.111601	0.2331104
SLC7A5P1	NC_000016	29624424	29625038	0	0	0	0
LOC647057	NC_000016	29630106	29648664	0	0	0	0
SPN	NC_000016	29674300	29681828	0.0063026	0.0259191	0.011214	0.0043919
QPRT	NC_000016	29690441	29709315	3.9284915	11.332172	0.6788645	0.0393889
C16orf54	NC_000016	29753784	29757340	0	0.0173916	0.0150491	0.0117879
ZG16	NC_000016	29790582	29791839	0.0721646	0	0	0.0502872
KIF22	NC_000016	29802058	29816706	1.2790277	2.2203676	2.0891829	4.9677761
MAZ	NC_000016	29817855	29822504	35.787999	67.750715	32.597718	63.861522
LOC100289283	NC_000016	29821577	29823178	0	0	0	0
PRRT2	NC_000016	29823409	29827202	0.1712046	0.1232115	0.5635411	0.644232
LOC100133019	NC_000016	29824590	29836148	0	0	0	0
C16orf53	NC_000016	29827528	29833816	1.8038882	3.106728	1.6967832	2.8983545
MVP	NC_000016	29831787	29859341	54.881014	45.055855	39.732177	69.916359
CDIPT	NC_000016	29869678	29874578	20.37941	16.781029	16.043772	13.874345
LOC440356	NC_000016	29875004	29879374	0	0	0	0
SEZ6L2	NC_000016	29882480	29910580	0.9774522	0.2671316	0.0770504	0.0258656
ASPHD1	NC_000016	29912147	29917377	0.6244336	0.3974183	0.1851711	0.0828819
KCTD13	NC_000016	29917660	29937545	1.1281926	1.0544551	1.1633465	1.5187367
TMEM219	NC_000016	29973351	29984373	2.4195723	2.2162029	3.4831665	2.9429312
TAOK2	NC_000016	29985222	30003581	7.880884	9.8975146	5.0018975	8.7566763
HIRIP3	NC_000016	30004316	30007387	2.4936497	3.6432009	2.1350206	2.6130442
INO80E	NC_000016	30007531	30017112	19.248213	23.640261	15.606033	20.235679
DOC2A	NC_000016	30016835	30022401	0.1489049	0.04374	0.0567728	0.1185862
C16orf92	NC_000016	30034655	30036023	0	0	0	0
FAM57B	NC_000016	30035748	30042186	0.2061147	0.1177264	0.1629914	0.0797938
ALDOA	NC_000016	30064491	30081735	233.42866	182.5818	179.48125	279.2501
LOC100288794	NC_000016	30076802	30078977	10.458269	15.101404	7.4745346	2.7840798
PPP4C	NC_000016	30087385	30096695	43.221269	46.440384	40.890762	25.190689

TBX6	NC_000016	30097114	30103205	0.2193755	0.1253006	0.1517931	0.1358841
YPEL3	NC_000016	30103635	30107537	4.5208593	3.2957968	3.7293813	5.8042163
LOC100271831	NC_000016	30105065	30125826	0	0	0	0
[truncated: 711,222 more chars]
