# Supplementary material for: Transcriptional Dynamics of Immortalized Human Mesenchymal Stem Cells during Transformation
Source: PLoS One. 2015 May 15;10(5):e0126562. doi: 10.1371/journal.pone.0126562 (PMC4433180; doi:10.1371/journal.pone.0126562)
Supplement: S2 Table — Expression value of U3-A,-B,-C, and-DT is the expression level of each gene divided by the level of GAPDH expression in the same sample. (*) Expression values for U3-A were obtained by dividing the value of each gene by that of GAPDH of U3-A, and then multiplying by 1,000. (**) REV of each gene in U3-A,-B,-C, and-DT is the expression level in U3-B,-C, or—DT divided by the expression level in U3-A. (PDF) [file pone.0126562.s005.pdf]

Supporting Table S2. The relative expression value (REV) of 1,732 genes shown in Fig. 3A.

Gene Symbol	Chromosome Location	Exp.Val.(*)	Relative Expression Value(**)			
		U3-A	U3-A	U3-B	U3-C	U3-DT
		PDL80	PDL80	PDL110	PDL219	PDL270
(a) Oncogenes						
AKT1	NC_000014	2.71259	1	1.300	0.350	0.260
AKT3	NC_000001	2.33283	1	1.473	2.342	1.442
ALK	NC_000002	0.04027	1	3.519	3.387	1.492
ATAD2	NC_000008	0.74069	1	1.503	2.356	4.847
BCL11A	NC_000002	0.00306	1	3.415	4.140	109.590
BMI1	NC_000010	0.92429	1	0.771	7.085	4.386
BRAF	NC_000007	0.95822	1	1.551	2.028	1.187
DDHD2	NC_000008	2.35299	1	1.205	2.264	1.440
ERBB2	NC_000017	3.02042	1	1.252	0.356	0.337
ERBB3	NC_000012	0.02298	1	1.138	2.587	1.588
ERGIC2	NC_000012	5.43376	1	1.364	2.121	1.576
FGFR1OP	NC_000006	0.67655	1	1.199	4.990	1.740
FOS	NC_000014	0.78679	1	0.538	0.345	0.154
JUNB	NC_000019	16.00137	1	0.826	1.095	0.375
JUND	NC_000019	10.08349	1	0.913	2.176	0.847
KIT	NC_000004	0.42678	1	0.669	0.011	0.044
LETMD1	NC_000012	0.96134	1	0.784	2.461	0.718
MAF	NC_000016	2.60576	1	0.068	0.183	0.176
MAFF	NC_000022	5.02992	1	0.592	0.588	0.233
MYB	NC_000006	0.09288	1	4.478	0.690	1.059
MYC	NC_000008	3.81322	1	1.265	2.990	2.615
PIK3CA	NC_000003	2.52148	1	1.875	3.817	1.959
PIM2	NC_000023_X	1.75979	1	0.391	1.666	1.281
RAB23	NC_000006	18.00614	1	0.472	0.797	0.304
RAB3A	NC_000019	0.43999	1	0.824	0.428	0.858
RAP2B	NC_000003	1.70150	1	1.847	2.692	1.497
RASAL3	NC_000019	0.00692	1	2.277	2.070	1.588
RASEF	NC_000009	0.53613	1	0.053	1.624	1.124
RASGEF1A	NC_000010	0.43891	1	0.280	0.492	0.069
RASGEF1B	NC_000004	0.17062	1	0.201	0.122	0.093
RASGRF2	NC_000005	0.00558	1	11.384	5.175	3.177
RASSF9	NC_000012	0.36772	1	2.826	1.963	1.789
RERG	NC_000012	0.06163	1	1.328	4.485	0.971
RET	NC_000010	0.32741	1	0.260	0.281	0.121
RGL2	NC_000006	2.30861	1	0.783	2.231	1.777
RGPD5	NC_000002	0.04385	1	2.656	0.690	0.706
RHEB	NC_000007	10.61282	1	0.959	1.998	1.167
RHEBL1	NC_000012	0.69654	1	1.170	3.105	1.265
RHOB	NC_000002	5.33008	1	3.316	0.316	0.108
ROS1	NC_000006	0.02164	1	2.114	0.887	0.227
RRAS	NC_000019	20.68809	1	0.968	1.056	0.324
RRAS2	NC_000011	7.28138	1	0.834	2.110	1.999
STAC	NC_000003	0.39198	1	4.353	3.105	5.803
USP6	NC_000017	0.01143	1	3.700	4.916	2.250
(b) Suppressor						
CAV1	NC_000007	59.77942	1	0.772	0.437	0.553
CYLD	NC_000016	0.96989	1	0.355	0.845	0.974
DAB2	NC_000005	30.53093	1	0.470	0.711	0.672
DAB2IP	NC_000009	1.91010	1	0.929	0.418	0.315
DAPK1	NC_000009	3.66640	1	0.056	0.547	1.245
DBC1	NC_000009	0.05700	1	2.988	0.259	0.132
DIRAS3	NC_000001	0.23737	1	4.420	1.339	0.218
EAF2	NC_000003	0.02233	1	5.692	6.210	4.235
FHIT	NC_000003	0.37607	1	0.316	1.610	1.353
FOXD3	NC_000001	0.01101	1	4.553	1.035	1.059
HIC1	NC_000017	0.31372	1	5.071	0.517	0.277
INHA	NC_000002	0.11203	1	0.163	0.000	0.151
INHBB	NC_000002	0.11376	1	0.142	0.129	1.588
JMJD5	NC_000016	0.08523	1	2.618	1.552	1.482
LIMD1	NC_000003	1.76850	1	0.723	1.896	2.140
LZTS1	NC_000008	0.63409	1	0.382	0.238	0.139
MTUS1	NC_000008	0.04610	1	4.705	0.966	0.353
MYCT1	NC_000006	0.14958	1	0.569	0.155	0.053
NEURL	NC_000010	0.02639	1	2.277	0.828	0.847
PHLDA2	NC_000011	4.40610	1	0.646	0.826	0.488
PRDM2	NC_000001	0.41825	1	1.087	2.499	2.945
PSD3	NC_000008	1.74630	1	0.493	0.621	1.017
PYHIN1	NC_000001	0.09840	1	1.518	3.565	2.706
RAP1GAP	NC_000001	0.10133	1	0.356	0.517	0.099
RASA4	NC_000007	0.08531	1	2.873	0.542	0.076

RASSF2	NC_000020	0.03761	1	39.337	0.345	0.353
RB1CC1	NC_000008	2.33299	1	1.405	2.258	2.392
RBBP4	NC_000001	3.79457	1	0.759	1.746	2.182
RUNX3	NC_000001	0.01911	1	0.285	0.776	0.794
SERPINE1	NC_000007	73.44615	1	3.619	0.624	0.961
SERPINE2	NC_000002	57.74317	1	0.819	0.264	0.075
SFN	NC_000001	0.05195	1	8.348	2.415	0.353
TGFB1	NC_000019	2.20354	1	2.272	0.301	0.452
TP63	NC_000003	0.08029	1	0.148	3.015	0.276
TP73	NC_000001	0.02191	1	5.692	0.690	0.882
USP7	NC_000016	1.59479	1	2.127	1.548	1.472
WWOX	NC_000016	0.73101	1	0.094	0.950	0.760

miscellaneous

ANKRD9	NC_000014	1.10644	1	1.599	0.590	0.348
ASXL1	NC_000020	1.52512	1	0.851	1.547	2.232
BIRC3	NC_000011	0.64456	1	0.185	1.392	0.333
CACYBP	NC_000001	3.40738	1	1.611	2.053	1.738
CDC42	NC_000001	5.11763	1	1.684	2.025	1.475
CDCA5	NC_000011	3.76605	1	1.857	2.653	1.942
CDK10	NC_000016	2.33080	1	0.323	0.775	0.712
CDK3	NC_000017	0.55120	1	0.330	0.626	1.031
CHEK2	NC_000022	1.82231	1	1.074	1.972	0.992
CUX1	NC_000007	0.55141	1	1.113	2.234	1.702
DNAJC15	NC_000013	2.05089	1	0.856	0.397	0.466
DSCC1	NC_000008	0.70256	1	1.968	2.114	1.929
E2F2	NC_000001	0.32458	1	1.077	1.706	2.018
GAS1	NC_000009	3.54320	1	2.269	2.411	0.605
HIST1H2BD	NC_000006	0.96367	1	2.546	2.206	2.703
HIST1H3A	NC_000006	2.13650	1	2.303	2.540	2.322
HIST1H4C	NC_000006	18.91920	1	3.001	2.527	5.075
HIST2H2AA3	NC_000001	12.62330	1	2.177	1.745	0.501
HIST2H2BE	NC_000001	1.88495	1	1.720	1.935	0.903
HMGN2	NC_000001	4.75355	1	1.579	1.466	2.046
KLHDC9	NC_000001	0.10025	1	0.759	6.555	3.177
LOC728622	NC_000012	0.04492	1	2.277	1.035	9.530
NEK7	NC_000001	13.11630	1	2.522	0.742	0.925
PRIM1	NC_000012	4.53281	1	1.694	2.191	0.908
RCC1	NC_000001	0.04497	1	2.049	1.035	1.588
REV3L	NC_000006	3.07089	1	0.873	2.010	1.328
SMARCA2	NC_000009	0.32437	1	1.027	1.641	2.963
SMARCB1	NC_000022	3.38320	1	2.119	0.695	0.973
SMARCD3	NC_000007	2.28917	1	0.525	0.627	0.280
SUMO1	NC_000002	3.93720	1	1.570	3.117	1.598
TDP1	NC_000014	1.17907	1	1.555	0.688	0.797
TSSK6	NC_000019	0.07896	1	0.455	0.828	2.118
TUBD1	NC_000017	0.19346	1	2.602	3.450	1.639
TUBE1	NC_000006	0.72374	1	1.326	2.155	1.139
UBE2D1	NC_000010	1.74385	1	1.458	2.559	1.281
UGCG	NC_000009	29.38107	1	0.402	1.907	0.728
UHRF1	NC_000019	0.44230	1	2.124	1.540	2.273
UMPS	NC_000003	0.47979	1	2.663	1.904	2.014
USP2	NC_000011	0.11759	1	0.271	1.971	2.597
WEE1	NC_000011	3.04917	1	1.023	2.018	1.235
WTAP	NC_000006	13.21796	1	0.921	5.263	0.981
YPEL5	NC_000002	4.74259	1	1.274	2.157	1.326

(c) DNA synthesis and repair

ATRX	NC_000023_X	0.17715	1	1.950	2.510	3.998
BLM	NC_000015	0.57838	1	2.544	3.267	2.951
BRCA1	NC_000017	0.43127	1	2.062	3.184	3.806
CDC45L	NC_000022	2.93308	1	2.212	1.073	1.300
CHAF1A	NC_000019	0.17030	1	1.685	2.608	2.033
DDB1	NC_000011	3.68003	1	1.746	2.344	2.464
DDIT3	NC_000012	12.77394	1	0.764	2.817	0.463
DDIT4	NC_000010	3.75652	1	1.450	5.977	1.361
DHFR	NC_000005	3.67440	1	1.682	3.033	3.809
DVL3	NC_000003	2.18643	1	1.743	3.184	3.148
ERCC5	NC_000013	0.16124	1	1.138	2.248	3.450
EXO1	NC_000001	0.79039	1	2.775	2.772	1.607
FANCG	NC_000009	2.57074	1	1.196	2.495	1.615
GADD45A	NC_000001	5.46219	1	1.072	0.787	0.280
GGH	NC_000008	11.01285	1	1.443	3.508	1.691
H1FX	NC_000003	7.92438	1	1.208	2.545	0.547
HAT1	NC_000002	3.66416	1	2.242	1.839	1.484
HELB	NC_000012	0.14199	1	1.247	3.105	1.210
HMGB2	NC_000004	6.87464	1	1.684	4.743	1.847
LIG1	NC_000019	0.79037	1	2.181	1.141	1.430

MCM3	NC_000006	2.88415	1	1.586	1.626	2.091
MDC1	NC_000006	0.70780	1	1.163	3.765	3.958
MPG	NC_000016	15.43856	1	1.153	0.787	0.445
NCAPG	NC_000004	2.08005	1	1.796	2.563	2.026
NTHL1	NC_000016	0.89641	1	2.006	2.242	2.168
ORC1L	NC_000001	0.61315	1	2.277	1.352	1.352
PARP1	NC_000001	2.49733	1	1.508	1.944	1.969
PARP2	NC_000014	1.93095	1	2.127	1.145	1.360
POLA1	NC_000023_X	0.78586	1	1.641	2.174	3.137
POLE	NC_000012	0.32243	1	1.231	2.387	3.305
POLE3	NC_000009	1.12460	1	1.057	1.608	2.642
PRKDC	NC_000008	2.53053	1	2.048	2.473	4.307
RAD51C	NC_000017	0.46280	1	2.473	1.999	1.734
RAD54B	NC_000008	0.71891	1	1.992	1.693	1.577
RAD54L	NC_000001	0.72388	1	1.423	2.267	2.181
RAD9A	NC_000011	0.37562	1	0.748	2.780	3.192
RECQL4	NC_000008	2.60555	1	1.363	2.025	2.102
RNASEH2A	NC_000019	4.56659	1	2.083	2.675	1.359
RPA2	NC_000001	12.97538	1	1.196	2.407	1.970
RUVBL1	NC_000003	0.73420	1	1.438	2.742	2.452
SFPQ	NC_000001	3.54973	1	1.994	1.000	0.833
SMC4	NC_000003	3.71167	1	1.964	3.424	3.036
SUV39H1	NC_000023_X	1.55157	1	1.573	2.164	2.135
TDG	NC_000012	0.83539	1	1.091	2.531	1.771
TERT	NC_000005	16.59524	1	11.864	6.096	6.367
TOP1	NC_000020	0.21956	1	1.992	3.134	3.412
TOP2A	NC_000017	9.52810	1	1.990	2.105	1.175
TOPBP1	NC_000003	1.97231	1	1.962	2.582	2.598
TREX1	NC_000003	6.13219	1	0.852	0.838	0.374
USP9X	NC_000023_X	1.77946	1	1.948	1.825	1.979
WRN	NC_000008	0.35670	1	1.480	2.254	1.571
XRCC2	NC_000007	0.43723	1	1.814	1.982	1.490
XRCC3	NC_000014	1.31168	1	1.206	0.477	0.373
XRCC4	NC_000005	0.65486	1	1.882	2.788	2.172

(d) Chromosome instability

ACTL6A	NC_000003	1.74443	1	1.739	5.238	3.296
ARPC1B	NC_000007	36.81153	1	0.919	0.811	0.424
ASF1B	NC_000019	5.76235	1	1.819	3.343	2.166
AURKB	NC_000017	2.30311	1	2.322	1.333	1.300
AURKC	NC_000019	0.10503	1	2.466	1.552	0.529
BUB1	NC_000002	4.25939	1	2.199	1.782	1.507
BUB1B	NC_000015	0.38341	1	1.644	1.298	2.605
CCNA2	NC_000004	5.02994	1	1.964	1.727	1.431
CCNB1	NC_000005	18.57320	1	2.167	1.983	1.346
CDC2	NC_000010	7.10312	1	1.673	2.883	1.958
CDC20	NC_000001	12.80733	1	2.864	1.116	1.005
CDC45L	NC_000022	2.93308	1	2.212	1.073	1.300
CDCA4	NC_000014	3.13792	1	1.916	0.377	0.243
CENPB	NC_000020	15.56430	1	1.345	0.517	0.441
CENPE	NC_000004	0.51565	1	2.300	1.417	2.788
CENPF	NC_000001	0.53305	1	1.932	2.250	3.752
CEP250	NC_000020	0.13640	1	1.184	1.304	3.579
CEP55	NC_000010	2.14194	1	1.935	2.774	2.732
CEP70	NC_000003	0.35917	1	1.491	3.819	3.290
CETN3	NC_000005	0.60295	1	2.408	3.383	3.034
CHD1	NC_000005	0.29273	1	1.865	2.244	2.583
CKAP5	NC_000011	1.45859	1	2.011	2.690	2.685
CKS1B	NC_000001	8.00966	1	1.424	2.553	0.729
CKS2	NC_000009	19.65249	1	1.502	2.095	1.889
ECT2	NC_000003	2.71414	1	1.756	4.713	4.204
ESPL1	NC_000012	0.77366	1	1.599	2.990	2.753
FEN1	NC_000011	3.31116	1	1.995	3.076	2.960
FOXM1	NC_000012	5.86599	1	1.766	1.161	2.228
FRY	NC_000013	0.55674	1	0.317	0.363	0.269
H2AFZ	NC_000004	19.04573	1	1.404	2.194	1.478
HDGF	NC_000001	18.43499	1	1.581	1.956	1.996
KIF20A	NC_000005	3.40514	1	2.114	1.789	1.682
KIF23	NC_000015	2.43593	1	2.383	1.399	1.676
KIF2C	NC_000001	0.90112	1	2.007	2.242	2.995
KIF4A	NC_000023_X	0.51722	1	2.232	1.670	3.019
LATS1	NC_000006	2.27971	1	1.385	2.050	1.389
LATS2	NC_000013	3.31962	1	0.417	0.756	0.449
MAD2L1	NC_000004	4.52928	1	2.098	2.616	1.727
MARCKSL1	NC_000001	1.04585	1	1.764	0.583	0.470
MCM10	NC_000010	0.32224	1	3.557	2.361	2.391
MCM7	NC_000007	2.59432	1	1.620	2.001	2.819

NEK2	NC_000001	0.78049	1	1.840	3.558	1.987
OIP5	NC_000015	0.64487	1	2.439	2.011	1.029
PRC1	NC_000015	0.88517	1	1.618	2.138	3.667
PTTG1	NC_000005	1.47130	1	1.485	4.387	3.591
RAD21	NC_000008	10.59709	1	1.838	3.100	1.977
RAD51AP1	NC_000012	2.85040	1	1.479	2.234	1.601
RASGRF1	NC_000015	0.00931	1	3.795	2.760	1.941
RFC4	NC_000003	3.76201	1	2.264	5.424	3.172
RNASEH2A	NC_000019	4.56659	1	2.083	2.675	1.359
RNF212	NC_000004	0.03477	1	26.467	0.000	0.265
RRM1	NC_000011	13.52399	1	1.828	2.071	1.830
RRM2	NC_000002	6.72994	1	2.058	4.075	2.072
SFN	NC_000001	0.05195	1	8.348	2.415	0.353
SPC24	NC_000019	1.11428	1	1.992	4.172	4.235
SPC25	NC_000002	0.59217	1	2.244	2.070	2.329
STAG2	NC_000023_X	0.67741	1	1.252	2.536	3.689
TACC2	NC_000010	0.06090	1	1.518	2.070	3.804
TERF2	NC_000016	1.65023	1	0.656	0.665	0.308
TINF2	NC_000014	6.28116	1	1.051	0.456	0.462
TMEM194A	NC_000012	3.08128	1	1.287	3.098	1.746
TOP2A	NC_000017	9.52810	1	1.990	2.105	1.175
TRIP13	NC_000005	2.62506	1	2.264	2.505	1.678
TTK	NC_000006	3.26097	1	2.079	2.584	2.092
USP44	NC_000012	0.03849	1	0.569	0.000	0.353
ZWINT	NC_000010	8.48917	1	1.889	1.738	2.781
(e) Cell Cycle						
ARHGAP19	NC_000010	0.27513	1	2.587	2.258	1.741
ARHGAP26	NC_000005	0.22418	1	0.793	2.384	2.034
BCCIP	NC_000010	0.58064	1	1.522	2.563	2.222
CCNA2	NC_000004	5.02994	1	1.964	1.727	1.431
CCNB1	NC_000005	18.57320	1	2.167	1.983	1.346
CCND1	NC_000011	25.93236	1	1.244	0.312	0.489
CCND2	NC_000012	0.00698	1	16.506	1.035	2.912
CCNE2	NC_000008	1.24430	1	1.704	2.223	2.594
CCNF	NC_000016	2.41171	1	2.327	1.630	1.774
CCNG2	NC_000004	0.33282	1	1.096	2.199	1.171
CCNH	NC_000005	3.99934	1	1.289	2.506	2.334
CDC2	NC_000010	7.10312	1	1.673	2.883	1.958
CDC25C	NC_000005	1.09828	1	2.478	4.160	2.590
CDC2L6	NC_000006	1.05723	1	1.154	2.669	2.521
CDC45L	NC_000022	2.93308	1	2.212	1.073	1.300
CDCA7L	NC_000007	2.22964	1	0.919	2.066	2.171
CDK2	NC_000012	3.74421	1	1.380	2.023	1.788
CDK2AP1	NC_000012	34.82946	1	1.249	0.794	0.316
CDK2AP2	NC_000011	3.55313	1	1.398	3.370	3.322
CDK4	NC_000012	42.24783	1	1.456	2.153	1.500
CDK6	NC_000007	1.01068	1	2.041	1.818	1.467
CDKN1A	NC_000006	32.82709	1	0.471	1.183	0.525
CDKN1C	NC_000011	0.09440	1	2.277	9.961	0.596
CDKN2A	NC_000009	26.59624	1	0.555	1.234	0.594
CDKN2AIPNL	NC_000005	1.48439	1	2.536	3.170	2.500
CDKN2B	NC_000009	0.46301	1	0.379	1.278	0.052
CDKN2D	NC_000019	1.90443	1	1.033	2.957	1.406
CDKN3	NC_000014	5.91642	1	1.835	2.061	1.497
CIZ1	NC_000009	1.87039	1	1.308	3.334	4.549
CKS2	NC_000009	19.65249	1	1.502	2.095	1.889
CLK4	NC_000005	0.39060	1	0.980	1.757	2.142
CLSPN	NC_000001	0.26523	1	2.478	1.360	2.481
E2F1	NC_000020	4.62210	1	1.388	1.358	2.018
E2F8	NC_000011	0.79035	1	1.756	2.952	2.499
KIAA0101	NC_000015	3.81571	1	1.536	2.764	2.027
LCMT2	NC_000015	2.38033	1	1.456	0.350	0.267
MYBL2	NC_000020	2.84559	1	2.754	2.513	4.093
PAK2	NC_000003	2.57074	1	1.613	3.398	3.086
PODN	NC_000001	3.33653	1	0.488	0.000	0.001
PRKCA	NC_000017	1.03349	1	1.196	1.555	2.107
RBBP8	NC_000018	0.62138	1	2.030	2.785	3.351
RRM1	NC_000011	13.52399	1	1.828	2.071	1.830
RRM2	NC_000002	6.72994	1	2.058	4.075	2.072
RUNX1T1	NC_000008	0.20903	1	1.528	0.218	0.056
SERTAD1	NC_000019	3.59078	1	1.174	0.142	0.203
SKP2	NC_000005	3.08702	1	1.423	2.163	1.938
TFDP2	NC_000003	1.47669	1	1.797	3.604	1.658
UBE2S	NC_000019	16.73087	1	1.958	1.352	1.000
(f)Apoptosis						
AIM2	NC_000001	0.72125	1	0.630	3.105	0.327

ANP32A	NC_000015	0.37847	1	2.055	2.398	2.144
APAF1	NC_000012	0.64605	1	1.585	2.136	1.329
ARHGDIA	NC_000017	12.09592	1	1.096	1.352	2.697
ATG5	NC_000006	2.49915	1	1.391	2.050	1.205
BAD	NC_000011	2.86524	1	0.998	2.009	1.114
BCL2	NC_000018	0.24424	1	0.930	2.026	2.051
BCL2L1	NC_000020	5.82009	1	0.552	0.972	1.035
BCL2L11	NC_000002	0.13701	1	1.138	2.329	1.423
BCL2L12	NC_000019	1.04351	1	2.317	1.205	1.134
BCL2L2	NC_000014	3.61661	1	0.847	0.380	0.474
CARD9	NC_000009	0.10727	1	0.683	0.310	0.741
CASP10	NC_000002	0.05370	1	3.036	0.621	0.176
CD274	NC_000009	0.62108	1	1.382	1.183	0.366
CST1	NC_000020	0.02996	1	6.830	0.000	0.000
FAS	NC_000010	4.53091	1	0.562	1.706	0.521
GLRX3	NC_000010	7.06625	1	1.610	2.177	1.939
HIP1	NC_000007	0.87770	1	2.318	2.452	2.104
HRK	NC_000012	0.13069	1	1.138	0.517	0.132
IER3	NC_000006	21.43244	1	2.032	1.024	0.677
IFT57	NC_000003	0.24174	1	2.344	2.618	2.616
MRPL3	NC_000003	12.57580	1	2.990	1.977	1.468
NLRP1	NC_000017	0.84013	1	0.951	0.359	0.478
PDCD2L	NC_000019	0.37365	1	2.696	1.961	1.087
PDCD6	NC_000005	8.20635	1	0.963	2.275	1.292
PERP	NC_000006	2.35036	1	2.466	2.771	1.904
PLA2G6	NC_000022	0.42395	1	0.323	0.949	0.803
PLA2R1	NC_000002	0.26417	1	1.222	1.385	0.280
PPP1R3C	NC_000010	3.19762	1	2.233	1.370	1.132
PPP1R3D	NC_000020	0.56506	1	0.741	1.962	1.213
PPPDE1	NC_000001	3.42181	1	1.546	2.181	1.151
PRKCSH	NC_000019	4.94764	1	1.237	2.151	1.300
PTEN	NC_000010	4.14728	1	1.382	1.786	1.428
RRM2B	NC_000008	2.40665	1	0.588	1.307	0.427
SDHC	NC_000001	2.10360	1	1.315	2.117	1.602
SOX4	NC_000006	0.14469	1	1.249	1.068	0.478
STK17A	NC_000007	3.25413	1	1.333	1.481	3.951
SYVN1	NC_000011	1.14948	1	0.931	2.251	2.028
TMBIM4	NC_000012	4.31385	1	1.089	2.226	0.860
TNFRSF10A	NC_000008	2.02546	1	0.479	0.620	0.268
TNFRSF11B	NC_000008	2.87333	1	4.988	0.098	0.463
TNFRSF6B	NC_000020	0.05877	1	1.708	6.210	4.500
TNFRSF8	NC_000001	0.14629	1	1.802	1.509	0.287
TNFSF10	NC_000003	1.86431	1	0.269	3.550	0.162
TP73	NC_000001	0.02191	1	5.692	0.690	0.882
TRADD	NC_000016	3.34763	1	0.350	0.442	0.403
TXN	NC_000009	41.06333	1	1.268	2.251	1.106
XAF1	NC_000017	11.36771	1	0.530	0.336	0.030
**(g) Signal transduction, TF. And Transcription regulation**						
ADRA2A	NC_000010	0.09401	1	4.767	1.941	0.099
AK2	NC_000001	5.25488	1	1.375	3.894	2.834
AKT1S1	NC_000019	0.40116	1	2.118	2.816	6.488
APC	NC_000005	1.39485	1	1.391	1.940	2.550
ATF3	NC_000001	0.33840	1	0.738	1.902	0.429
ATF5	NC_000019	3.55861	1	0.935	0.346	0.336
BCL6	NC_000003	0.82138	1	0.457	3.950	1.592
BHLHE40	NC_000003	10.35482	1	0.679	0.451	0.430
BMP2	NC_000020	0.17351	1	1.660	2.113	0.507
BMP4	NC_000014	1.87763	1	0.519	0.067	0.133
CALR	NC_000019	135.17289	1	1.116	2.283	0.944
CAMK2N1	NC_000001	0.63931	1	5.450	1.411	0.489
CAMK2N2	NC_000003	0.01674	1	1.138	1.035	13.236
CBX7	NC_000022	0.74110	1	0.768	2.006	2.118
CBX8	NC_000017	0.25453	1	2.478	1.339	1.059
CEBPD	NC_000008	11.34105	1	0.437	1.590	0.459
CREBBP	NC_000016	1.15016	1	1.724	2.050	1.546
CTNNB1	NC_000003	17.04922	1	1.507	0.958	0.683
CYTIP	NC_000002	0.14426	1	0.894	0.296	0.643
DCLK1	NC_000013	0.40586	1	0.292	0.108	0.107
DEPDC1	NC_000001	3.42045	1	2.331	1.296	1.067
DGKA	NC_000012	1.75178	1	0.630	0.555	0.135
DHH	NC_000012	0.02353	1	5.692	4.140	3.441
DKK1	NC_000010	29.28332	1	2.750	0.561	1.197
DKK3	NC_000011	16.94655	1	1.063	3.363	2.582
DLGAP4	NC_000020	1.20491	1	1.270	1.165	2.531
DLX4	NC_000017	0.91407	1	0.061	0.501	0.233
DMPK	NC_000019	2.02428	1	0.559	0.547	0.486

DMXL1	NC_000005	1.23313	1	1.270	2.248	1.340
DOK1	NC_000002	3.81962	1	1.972	0.597	0.499
DUSP2	NC_000002	0.54061	1	0.711	0.207	0.265
EFNB1	NC_000023_X	0.51959	1	0.599	0.422	0.529
EFS	NC_000014	1.86246	1	0.509	0.414	0.436
EGR1	NC_000005	5.94018	1	0.550	0.232	0.067
EHF	NC_000011	0.02518	1	0.285	41.398	2.912
EMX2	NC_000010	0.29095	1	3.107	0.084	0.014
EN1	NC_000002	1.14558	1	0.733	0.236	0.370
EPHA3	NC_000003	1.38536	1	0.011	0.059	0.039
EPHA4	NC_000002	0.98296	1	0.253	0.457	0.338
EPHB3	NC_000003	0.17269	1	7.222	2.296	1.059
ERN1	NC_000017	0.28431	1	1.571	2.981	0.942
EYA4	NC_000006	1.01406	1	0.057	2.704	1.919
FERMT1	NC_000020	0.49958	1	3.828	0.925	0.614
FERMT2	NC_000014	18.69632	1	1.119	0.377	0.299
FLI1	NC_000011	0.74976	1	0.973	0.103	0.077
FLT3LG	NC_000019	1.42881	1	0.858	0.685	0.456
FOSB	NC_000019	0.12062	1	0.512	0.207	0.212
FOSL1	NC_000011	15.64627	1	1.010	3.171	3.212
FOXC1	NC_000006	1.97253	1	0.312	1.246	0.499
FOXC2	NC_000016	1.17722	1	0.393	0.107	0.201
FOXF2	NC_000006	0.13537	1	21.717	1.672	1.955
FOXL1	NC_000016	2.58428	1	0.783	0.146	0.105
FOXO1	NC_000013	0.30163	1	0.195	1.648	0.690
FOXO4	NC_000023_X	0.28801	1	0.949	3.080	2.118
FOXO6	NC_000001	0.04616	1	1.518	3.105	2.471
FOXP3	NC_000023_X	0.02868	1	0.759	2.415	0.882
FRAP1	NC_000001	1.41728	1	2.145	1.510	1.399
FZD1	NC_000007	3.50235	1	1.509	0.608	0.339
FZD3	NC_000008	0.06948	1	0.190	2.156	0.706
FZD4	NC_000011	2.02345	1	1.423	0.218	0.250
FZD5	NC_000002	0.07286	1	0.434	2.119	2.622
FZD7	NC_000002	1.78589	1	1.828	0.117	0.068
GATA2	NC_000003	0.09680	1	24.617	8.668	6.353
GBP2	NC_000001	2.69416	1	0.983	7.133	0.954
GLI1	NC_000012	0.03789	1	5.502	2.415	1.412
GLI2	NC_000002	0.25569	1	4.179	1.362	1.101
GREB1	NC_000002	0.09691	1	0.621	0.894	0.481
HES1	NC_000003	0.21942	1	1.464	0.887	0.113
HHIP	NC_000004	0.32902	1	2.589	0.142	0.384
HIPK2	NC_000007	0.52241	1	0.760	2.933	3.812
HLF	NC_000017	0.04067	1	0.342	10.142	20.647
HLTF	NC_000003	2.97485	1	1.811	2.464	0.965
HLX	NC_000001	0.32976	1	3.864	0.659	0.273
HNF1A	NC_000012	0.04922	1	1.789	0.739	1.966
HOPX	NC_000004	0.15614	1	0.963	2.786	1.466
HOXA3	NC_000007	0.04488	1	1.626	1.478	2.118
HOXA4	NC_000007	0.18450	1	1.626	4.066	4.387
HOXA5	NC_000007	0.01710	1	0.000	3.105	2.118
HOXA9	NC_000007	0.12178	1	0.414	0.753	0.529
HOXB5	NC_000017	0.13756	1	3.622	0.753	0.529
HOXB6	NC_000017	0.47557	1	3.936	1.508	0.635
HOXC6	NC_000012	0.38667	1	2.097	2.043	0.836
HSP90AA1	NC_000014	12.30329	1	1.885	0.484	0.836
HSP90AB1	NC_000006	36.57336	1	1.766	2.331	1.706
HSP90B1	NC_000012	23.05982	1	1.206	2.627	1.465
HSPA1A	NC_000006	1.71572	1	1.866	3.365	1.293
HSPB7	NC_000001	5.92951	1	2.283	0.353	0.697
HSPE1	NC_000002	4.89873	1	1.971	2.423	1.970
ID1	NC_000020	8.44208	1	1.342	0.400	0.708
ID2	NC_000002	8.28113	1	3.085	0.486	0.374
IGF2R	NC_000006	1.82115	1	1.015	2.570	2.070
JAG2	NC_000014	0.08971	1	1.081	0.983	0.344
JAK2	NC_000009	1.11172	1	0.472	1.312	0.587
JAK3	NC_000019	0.74570	1	0.032	0.983	0.333
KLF4	NC_000009	3.01260	1	1.479	0.603	0.236
KREMEN1	NC_000022	0.11327	1	2.800	0.587	0.444
KREMEN2	NC_000016	0.21066	1	2.097	0.490	0.669
LDB1	NC_000010	5.29924	1	0.981	2.121	1.562
LEF1	NC_000004	0.08165	1	1.345	3.952	2.214
LIFR	NC_000005	0.54508	1	0.910	2.390	1.561
LMTK2	NC_000007	0.21139	1	1.270	3.423	3.472
LPHN1	NC_000019	0.02898	1	0.797	10.660	7.253
LTBP1	NC_000002	6.76775	1	0.565	0.260	0.093
LTBP2	NC_000014	1.59737	1	4.747	0.450	0.334

LTBP4	NC_000019	0.25090	1	2.998	4.743	1.376
MAP2K6	NC_000017	0.08529	1	1.301	0.444	0.303
MAP3K1	NC_000005	0.07579	1	1.776	3.063	1.906
MAP3K14	NC_000017	0.78510	1	0.458	1.384	1.781
MAP3K5	NC_000006	1.46414	1	0.590	2.219	1.211
MAP3K8	NC_000010	2.11107	1	0.278	1.341	0.723
MAP3K9	NC_000014	0.17074	1	1.030	1.725	1.462
MARCKS	NC_000006	2.43600	1	2.297	0.846	0.368
MARK3	NC_000014	5.00983	1	1.503	0.530	0.288
MAX	NC_000014	3.47923	1	0.931	1.682	1.439
MDS1	NC_000003	0.19613	1	1.391	1.840	3.118
MELK	NC_000009	4.62185	1	1.237	1.988	1.448
MLLT10	NC_000010	0.61369	1	0.899	2.947	2.049
MRVI1	NC_000011	0.61854	1	0.384	0.172	0.158
MSC	NC_000008	1.81477	1	1.596	0.391	0.174
MSX1	NC_000004	0.16434	1	5.204	1.035	0.151
MUC1	NC_000001	1.11451	1	0.778	3.243	2.391
MXI1	NC_000010	1.46674	1	0.802	3.821	2.894
NCOR2	NC_000012	2.06767	1	2.034	1.140	0.968
NDRG1	NC_000008	14.98128	1	1.041	2.334	3.130
NFE2	NC_000012	0.12211	1	0.911	4.761	1.059
NFKB2	NC_000010	4.07440	1	0.996	2.504	0.795
NLE1	NC_000017	0.81543	1	2.265	1.024	1.124
NOP16	NC_000005	2.45967	1	2.591	1.642	1.917
NOTCH1	NC_000009	0.66151	1	1.151	2.097	1.076
NOTCH2	NC_000001	5.51737	1	1.300	2.296	1.282
NOTCH3	NC_000019	1.15685	1	0.555	0.005	0.010
NPAS2	NC_000002	0.71664	1	1.662	3.105	1.479
NR4A1	NC_000012	0.86998	1	0.748	0.731	0.343
NR4A3	NC_000009	0.10309	1	0.783	0.614	0.381
NRG1	NC_000008	1.47030	1	1.224	0.532	0.491
OSMR	NC_000005	7.63662	1	0.715	2.297	1.914
PAX7	NC_000001	0.02164	1	3.605	1.897	1.412
PAX8	NC_000002	0.09547	1	0.201	2.679	2.896
PDE10A	NC_000006	0.02464	1	4.553	0.230	0.118
PDE3B	NC_000011	0.01122	1	1.518	15.869	7.412
PDE6A	NC_000005	0.01211	1	1.897	0.690	1.235
PHF16	NC_000023_X	0.48551	1	1.536	3.349	2.208
PIK3CB	NC_000003	0.84345	1	1.148	3.435	3.501
PIK3CD	NC_000001	0.30302	1	3.210	2.027	2.669
PIK3CG	NC_000007	0.01270	1	4.174	2.070	1.941
PIK3IP1	NC_000022	0.27626	1	3.946	1.345	0.494
PIK3R1	NC_000005	0.90987	1	1.052	0.912	0.386
PINK1	NC_000001	2.26875	1	1.723	1.101	2.228
PITX2	NC_000004	1.89961	1	0.784	0.310	0.115
POU5F1	NC_000006	0.05679	1	0.569	3.364	1.059
PRDM16	NC_000001	0.01306	1	4.781	1.035	0.847
PRDM5	NC_000004	0.91827	1	1.104	0.994	0.487
PRKACB	NC_000001	1.00285	1	1.705	2.078	1.453
PRKCH	NC_000014	0.31828	1	0.209	0.676	0.443
PRKCI	NC_000003	1.95371	1	1.989	4.429	2.737
PRKD1	NC_000014	2.53791	1	0.614	0.222	0.340
PRKD2	NC_000019	1.07475	1	1.120	0.722	0.432
PRKRA	NC_000002	1.34206	1	1.539	3.477	2.535
PRRX2	NC_000009	2.46665	1	1.491	0.991	0.485
PSIP1	NC_000009	3.02648	1	0.942	3.789	1.956
PTCD1	NC_000007	0.67306	1	1.001	0.773	0.413
PTCH1	NC_000009	0.02025	1	3.700	2.975	1.985
PTCH2	NC_000001	0.01257	1	7.969	9.314	1.853
PTPN21	NC_000014	0.51299	1	1.057	0.347	0.257
PTPRF	NC_000001	0.79807	1	2.134	1.818	0.490
PTPRN2	NC_000007	0.01887	1	3.131	0.000	0.662
PURA	NC_000005	1.82080	1	1.345	1.050	0.484
RAB11FIP1	NC_000008	0.12336	1	0.424	6.691	4.568
RABGGTB	NC_000001	3.62299	1	1.449	2.492	1.982
RANBP2	NC_000002	0.57171	1	2.017	1.669	3.380
RBBP6	NC_000016	0.55858	1	1.145	2.593	2.097
RELA	NC_000011	2.38508	1	1.100	2.457	2.265
RELB	NC_000019	3.92259	1	1.135	1.316	0.391
RGS2	NC_000001	0.94888	1	0.955	0.610	0.170
RNF7	NC_000003	2.56358	1	2.245	1.460	1.071
RUNX2	NC_000006	0.17550	1	2.598	4.005	4.155
S100A6	NC_000001	262.84104	1	1.641	2.189	0.649
SCAND1	NC_000020	6.60240	1	1.165	0.457	0.488
SCN1A	NC_000002	0.04498	1	0.427	2.458	0.430
SCN9A	NC_000002	0.09139	1	5.782	0.517	0.251

SFRP1	NC_000008	0.08690	1	37.834	0.609	0.405
SFRP2	NC_000004	0.01144	1	18.214	4.140	1.588
SFRP4	NC_000007	1.24816	1	0.119	0.038	0.003
SGK1	NC_000006	3.72755	1	3.462	0.710	0.840
SH2B3	NC_000012	6.10400	1	0.401	0.782	0.833
SIM2	NC_000021	0.29391	1	0.142	1.132	0.711
SIX1	NC_000014	3.62743	1	0.862	0.607	0.282
SKAP1	NC_000017	0.13005	1	0.885	3.565	1.000
SMAD3	NC_000015	6.59801	1	0.506	1.812	2.059
SMAD5	NC_000005	2.80531	1	1.827	2.430	1.470
SMAD6	NC_000015	1.69107	1	0.855	0.276	0.353
SMAD7	NC_000018	2.87707	1	1.311	0.234	0.231
SMG1	NC_000016	1.55648	1	1.353	2.012	1.751
SMO	NC_000007	0.65188	1	1.426	0.068	0.084
SMURF2	NC_000017	16.08726	1	0.969	0.294	0.291
SNAI1	NC_000020	0.83300	1	1.836	0.501	0.307
SNAI2	NC_000008	11.99897	1	2.182	0.234	0.254
SNAP25	NC_000020	0.01049	1	0.000	10.349	3.177
SOCS1	NC_000016	0.50565	1	2.192	0.882	0.902
SOCS2	NC_000012	0.70071	1	0.519	0.320	0.654
SORBS2	NC_000004	0.57260	1	0.184	0.353	0.382
SOS1	NC_000002	1.56603	1	1.059	2.814	1.513
SOX10	NC_000022	0.01591	1	2.277	0.517	0.000
SOX11	NC_000002	0.01567	1	0.190	0.345	0.353
SOX9	NC_000017	0.41669	1	2.134	0.733	0.368
SP4	NC_000007	0.14500	1	1.354	4.336	3.277
SRPK1	NC_000006	5.58753	1	1.310	2.275	1.831
STAT1	NC_000002	103.35603	1	0.685	1.055	0.152
STIP1	NC_000011	2.63841	1	2.946	3.543	3.853
STMN3	NC_000020	1.35326	1	1.674	0.293	0.087
SUFU	NC_000010	0.76716	1	1.573	2.239	1.248
TASP1	NC_000020	0.24216	1	2.095	1.076	0.762
TBX3	NC_000012	0.00476	1	93.346	17.594	24.353
TBX5	NC_000012	0.00523	1	5.692	16.559	6.882
TCF21	NC_000006	0.01869	1	1.138	4.140	0.706
TCF4	NC_000018	1.77659	1	2.249	0.777	0.541
TFAP2A	NC_000006	0.08034	1	2.201	1.449	0.635
TGFBR2	NC_000003	6.51629	1	2.408	0.972	0.608
TGIF2	NC_000020	0.50815	1	1.483	2.015	0.711
TIFA	NC_000004	0.74848	1	0.981	2.193	1.190
TLE3	NC_000015	0.25204	1	1.756	1.210	2.584
TLR1	NC_000004	0.20768	1	0.701	2.866	0.672
TLX2	NC_000002	0.20797	1	1.423	3.170	1.357
TNFRSF9	NC_000001	0.09670	1	0.427	3.105	0.397
TNIK	NC_000003	0.06693	1	6.261	1.207	1.500
TNK2	NC_000003	0.38136	1	1.799	2.517	1.458
TOM1L1	NC_000017	1.81990	1	0.298	2.823	1.546
TOX	NC_000008	1.17973	1	0.436	0.063	0.163
TRAF3	NC_000014	1.19765	1	1.552	0.751	0.482
TRAIP	NC_000003	1.21517	1	2.108	1.313	1.088
TRIM29	NC_000011	0.14332	1	0.959	0.218	0.139
TRIM33	NC_000001	0.92578	1	1.286	1.954	1.188
TRPS1	NC_000008	0.81837	1	0.498	2.548	1.112
TSPAN15	NC_000010	0.48254	1	1.044	3.795	2.015
TSPAN8	NC_000012	0.02015	1	4.553	0.000	1.588
TTC1	NC_000005	0.27515	1	1.942	0.731	2.741
TTC37	NC_000005	7.25882	1	1.185	2.016	1.701
TTC38	NC_000022	2.52162	1	1.237	0.451	0.731
TXNIP	NC_000001	2.96501	1	0.769	6.006	0.265
VAV3	NC_000001	0.07487	1	0.356	2.975	0.563
VDAC3	NC_000008	4.79936	1	1.628	1.970	1.229
VDR	NC_000012	1.59293	1	2.123	0.251	0.384
WHSC1L1	NC_000008	1.04938	1	1.443	3.758	2.326
WISP1	NC_000008	1.14761	1	0.048	0.022	0.049
WNK1	NC_000012	0.80355	1	1.348	3.147	5.716
WNT11	NC_000011	0.13000	1	6.106	2.070	0.529
WNT2B	NC_000001	0.33121	1	0.154	1.678	2.375
WNT5A	NC_000003	5.98389	1	0.933	0.132	0.051
WNT5B	NC_000012	5.95195	1	0.876	0.231	0.298
WT1	NC_000011	0.00752	1	2.277	4.140	1.059
ZBTB20	NC_000003	1.40556	1	0.927	2.505	1.232
ZEB2	NC_000002	1.34755	1	0.457	0.985	1.078
ZFAT	NC_000008	0.21834	1	1.076	1.616	2.023
(h) Growth-related factors						
AACS	NC_000012	2.26291	1	1.252	0.815	0.459
ABAT	NC_000016	0.02304	1	3.415	1.035	0.265

ABCA5	NC_000017	0.16509	1	1.574	1.979	2.951
ABCA8	NC_000017	0.16321	1	1.916	0.000	0.052
ABCC2	NC_000010	0.04509	1	2.277	2.484	1.906
ABCC3	NC_000017	0.66967	1	0.433	2.684	1.387
ABCC4	NC_000013	0.48446	1	1.438	1.696	2.667
ABCC5	NC_000003	0.42567	1	2.173	3.936	5.130
ABCC6	NC_000016	0.14287	1	0.390	2.720	0.363
ABCD3	NC_000001	1.07958	1	1.948	2.070	1.221
ABCG2	NC_000004	0.59618	1	0.903	0.268	0.685
ACAP2	NC_000003	2.25155	1	1.499	3.975	1.558
ACLY	NC_000017	15.58491	1	2.034	1.037	0.856
ACOT2	NC_000014	4.74279	1	0.780	0.582	0.296
ACOT7	NC_000001	1.26782	1	1.464	2.054	1.931
ACOX2	NC_000003	1.54730	1	0.343	0.511	0.176
ACP5	NC_000019	0.27680	1	0.259	0.612	0.313
ACSL5	NC_000010	0.85764	1	2.777	1.218	0.383
ACY1	NC_000003	4.82822	1	0.956	0.835	0.462
ADA	NC_000020	4.71219	1	1.332	2.263	1.984
ADIPOQ	NC_000003	0.25867	1	0.547	1.393	0.417
ADORA1	NC_000001	1.86460	1	0.362	1.208	0.904
ADORA2A	NC_000022	0.10650	1	6.925	1.466	0.574
ADORA2B	NC_000017	1.03899	1	0.344	1.456	1.471
ADRA1B	NC_000005	0.15155	1	0.911	2.553	1.588
ADRB2	NC_000005	0.08961	1	0.711	2.458	0.860
ADSL	NC_000022	8.17795	1	2.048	0.814	1.028
AGPAT9	NC_000004	0.43138	1	3.866	2.329	1.268
AHR	NC_000007	11.42572	1	0.494	0.972	0.459
AIP	NC_000011	6.85225	1	1.283	2.817	2.185
AKAP1	NC_000017	0.21235	1	0.800	0.951	2.561
AKR1B1	NC_000007	71.85421	1	0.606	6.515	1.931
AKR1B10	NC_000007	0.15696	1	0.414	3.858	0.963
AKR1C1	NC_000010	51.09500	1	0.432	3.027	1.137
AKR1C3	NC_000010	9.07948	1	0.772	3.124	2.124
ALAD	NC_000009	1.32360	1	0.849	0.470	0.484
ALDH1A1	NC_000009	0.04346	1	0.854	2.587	1.721
ALDH1A3	NC_000015	2.45580	1	2.533	2.347	2.678
ALDH2	NC_000012	1.78389	1	1.756	3.176	0.735
ALDH3A1	NC_000017	0.06054	1	1.138	2.760	1.324
ALDH5A1	NC_000006	0.09250	1	1.572	4.238	1.765
ALDOC	NC_000017	0.52415	1	0.569	0.136	0.307
AMACR	NC_000005	1.42894	1	2.649	0.781	0.776
AMH	NC_000019	0.01128	1	11.384	3.105	3.706
ANXA10	NC_000004	0.09569	1	19.542	0.690	0.882
APOBEC3G	NC_000022	2.15337	1	0.516	0.644	0.142
APOD	NC_000003	0.06046	1	1.897	0.690	0.176
APOE	NC_000019	1.11936	1	0.157	0.357	0.055
AQP1	NC_000007	0.05788	1	3.090	0.739	0.454
AREG	NC_000004	0.03679	1	2.846	6.210	2.382
ARF6	NC_000014	9.66079	1	0.976	0.502	0.455
ARL17	NC_000017	0.89409	1	1.272	1.035	1.183
ARL4C	NC_000002	1.97113	1	1.069	0.340	0.374
ARL4D	NC_000017	0.15805	1	0.517	0.847	0.481
ARL5B	NC_000010	1.71956	1	0.819	2.536	1.031
ARNT	NC_000001	1.21324	1	1.417	2.565	1.930
ASPH	NC_000008	3.69189	1	1.182	5.869	2.476
ASS1	NC_000009	7.60336	1	0.515	2.758	0.682
ATIC	NC_000002	2.43918	1	2.039	1.896	2.108
ATP1A2	NC_000001	0.02917	1	0.650	12.863	1.361
ATP1B1	NC_000001	2.01853	1	2.072	2.287	1.626
ATP5C1	NC_000010	20.51167	1	1.167	2.379	1.630
ATP5J	NC_000021	5.44727	1	1.072	2.611	0.910
ATP5J2	NC_000007	7.77506	1	1.421	2.890	2.061
ATP6V1B1	NC_000002	0.01174	1	9.107	4.140	2.647
ATP8B1	NC_000018	4.30568	1	0.785	0.433	0.907
B2M	NC_000015	442.67973	1	0.563	1.733	0.258
BMP6	NC_000006	0.16869	1	2.772	0.450	0.714
BNC1	NC_000015	0.47403	1	6.463	1.703	1.627
BRF1	NC_000014	0.73248	1	1.518	0.359	0.316
BTRC	NC_000010	0.56551	1	1.386	2.301	1.574
BZW1	NC_000002	38.52221	1	0.885	0.642	0.485
CA9	NC_000009	1.46357	1	0.034	5.102	1.975
CANX	NC_000005	8.97039	1	1.586	2.097	1.379
CAPN3	NC_000015	0.14207	1	1.102	0.200	0.205
CAPN5	NC_000011	0.96422	1	1.138	0.634	0.396
CAT	NC_000011	4.34814	1	1.141	3.107	2.107
CCDC28A	NC_000006	0.73069	1	1.447	4.032	2.294

CCL11	NC_000017	0.02462	1	0.000	0.000	3.177
CCL13	NC_000017	0.02676	1	0.000	33.118	3.177
CCL2	NC_000017	66.25181	1	0.234	3.025	0.460
CCL21	NC_000009	0.05187	1	2.846	4.657	0.265
CCL25	NC_000019	0.02556	1	2.277	0.000	0.529
CCL3	NC_000017	0.02857	1	5.692	2.070	0.529
CCL5	NC_000017	7.05411	1	0.066	0.407	0.008
CCR1	NC_000003	0.43402	1	0.067	7.103	0.291
CCR10	NC_000017	1.28145	1	0.195	0.665	0.333
CCR4	NC_000003	0.20615	1	0.683	0.069	0.035
CCR5	NC_000003	0.02488	1	0.569	1.294	0.265
CCR7	NC_000017	0.30392	1	0.393	0.250	0.237
CCR9	NC_000003	0.03581	1	0.285	0.000	1.191
CCRL2	NC_000003	0.26849	1	0.052	1.411	0.818
CDA	NC_000001	0.56933	1	7.637	0.172	0.221
CES2	NC_000016	1.87129	1	0.552	0.664	0.286
CGRRF1	NC_000014	1.11886	1	2.137	1.099	0.725
CLU	NC_000008	0.70116	1	2.632	1.779	0.629
CMPK2	NC_000002	13.25701	1	0.298	0.701	0.040
CNDP1	NC_000018	0.19686	1	0.779	0.654	0.167
CNP	NC_000017	13.39697	1	0.948	0.617	0.315
COMT	NC_000022	9.19666	1	0.786	0.412	0.279
COX5A	NC_000015	8.01495	1	1.248	2.051	1.184
CP	NC_000003	0.01464	1	0.000	2.070	11.824
CPA4	NC_000007	2.97498	1	0.059	0.290	1.798
CPEB2	NC_000004	3.75572	1	0.283	0.836	0.485
CPT1A	NC_000011	2.86480	1	1.358	2.064	2.456
CRABP1	NC_000015	0.02950	1	0.000	2.070	1.059
CRABP2	NC_000001	8.27259	1	4.405	0.206	0.129
CRELD1	NC_000003	0.34540	1	0.931	3.575	1.913
CSF1R	NC_000005	0.11418	1	0.228	0.880	0.715
CSF2	NC_000005	0.49570	1	0.268	30.318	3.052
CSF3	NC_000017	0.02689	1	3.415	0.517	0.529
CSRP2	NC_000012	1.11212	1	1.578	2.611	1.348
CST3	NC_000020	24.27173	1	0.972	0.309	0.126
CTGF	NC_000006	9.13256	1	1.618	0.011	0.139
CTSS	NC_000001	3.03827	1	0.414	1.368	0.226
CUGBP1	NC_000011	2.80299	1	2.088	2.543	2.599
CXCL1	NC_000004	5.94620	1	1.957	2.339	0.272
CXCL10	NC_000004	0.94969	1	0.163	2.746	0.000
CXCL11	NC_000004	0.03059	1	3.984	5.175	0.000
CXCL12	NC_000010	18.12536	1	0.850	0.139	0.758
CXCL16	NC_000017	0.53182	1	0.224	0.314	0.246
CXCL2	NC_000004	1.68200	1	1.650	5.244	0.393
CXCL3	NC_000004	1.04139	1	4.897	13.259	0.350
CXCL5	NC_000004	0.04632	1	4.326	8.280	0.424
CXCL6	NC_000004	0.21963	1	30.665	0.582	0.000
CXCL9	NC_000004	0.08053	1	0.126	0.230	0.000
CYBA	NC_000016	7.67930	1	0.451	0.245	0.014
CYCS	NC_000007	0.76076	1	1.541	2.239	1.378
CYGB	NC_000017	4.00368	1	3.760	0.021	0.015
CYP11A1	NC_000015	0.88166	1	1.356	1.409	0.270
CYP1B1	NC_000002	14.99941	1	0.190	0.549	0.195
CYP27B1	NC_000012	0.19229	1	0.271	0.345	0.101
DBN1	NC_000005	3.32394	1	2.505	1.803	1.583
DDX24	NC_000014	12.12042	1	1.159	0.405	0.304
DGAT1	NC_000008	0.79906	1	1.366	2.675	1.906
DGAT2	NC_000011	1.87609	1	1.127	2.029	1.012
DHODH	NC_000016	0.56216	1	0.982	0.913	0.467
DHRS7	NC_000014	5.69328	1	1.027	2.085	0.631
DLGAP5	NC_000014	1.94172	1	2.084	1.385	1.415
DOCK11	NC_000023_X	0.55578	1	1.370	1.904	2.693
DPP4	NC_000002	5.95604	1	0.408	1.201	0.592
DPYS	NC_000008	0.02145	1	0.569	5.175	0.265
DPYSL2	NC_000008	25.30123	1	1.137	0.597	0.461
DUSP4	NC_000008	0.15085	1	6.668	0.185	0.113
DUSP5	NC_000010	5.63923	1	0.435	3.454	1.277
DUSP6	NC_000012	7.50221	1	0.498	0.998	0.096
EBI3	NC_000019	0.14008	1	0.650	1.331	0.303
EEF1A1	NC_000006	66.22488	1	1.595	2.298	1.310
EEF1A2	NC_000020	0.07422	1	1.708	3.795	0.971
EGFL7	NC_000009	0.59382	1	1.708	0.776	0.053
EHHADH	NC_000003	0.84478	1	1.284	0.984	0.195
EIF2AK1	NC_000007	2.18295	1	1.346	3.018	1.984
EIF2AK2	NC_000002	2.93019	1	0.767	1.557	0.465
EIF2C1	NC_000001	0.59384	1	1.489	2.272	3.212

EIF2C2	NC_000008	3.24005	1	1.208	2.472	2.167
EIF2S3	NC_000023_X	7.62390	1	1.215	1.896	2.138
EIF3H	NC_000008	19.66525	1	1.343	2.477	2.248
EIF4A2	NC_000003	5.27479	1	1.674	5.333	3.103
EIF4B	NC_000012	5.23533	1	1.676	4.496	5.975
ELOVL7	NC_000005	0.12909	1	0.155	19.993	6.714
ENO2	NC_000012	12.11496	1	0.377	0.866	1.265
ENPEP	NC_000004	0.09574	1	0.163	0.345	0.126
ENPP2	NC_000008	20.93821	1	1.304	1.393	0.394
EPHX1	NC_000001	6.12296	1	0.382	0.912	0.726
ETFA	NC_000015	9.37815	1	1.210	2.137	2.031
ETV4	NC_000017	0.42429	1	2.128	3.847	1.439
ETV5	NC_000003	7.58363	1	1.035	2.869	1.557
ETV6	NC_000012	0.84796	1	0.904	2.896	1.918
F2R	NC_000005	1.48832	1	11.919	8.521	9.113
FASN	NC_000017	7.28050	1	1.237	3.143	1.023
FBLN5	NC_000014	1.34061	1	4.715	0.020	0.031
FBXL7	NC_000005	1.11320	1	0.628	2.149	1.750
FBXO4	NC_000005	1.45445	1	1.996	2.169	1.537
FDPS	NC_000001	18.38994	1	1.198	2.108	1.113
FGF12	NC_000003	0.09848	1	0.737	3.653	1.433
FGF13	NC_000023_X	0.04618	1	0.976	6.062	0.605
FGF14	NC_000013	0.03765	1	1.594	1.449	5.188
FGF3	NC_000011	0.14711	1	0.569	0.828	0.424
FGF5	NC_000004	9.09826	1	0.643	0.254	0.535
FGF7	NC_000015	0.70009	1	1.176	0.325	0.188
FH	NC_000001	8.19323	1	1.612	2.321	1.551
FTH1	NC_000011	254.15458	1	0.642	2.407	1.266
GAL	NC_000011	0.08781	1	2.277	0.000	0.176
GAP43	NC_000003	0.15153	1	1.057	1.331	0.189
GBP1	NC_000001	2.00851	1	0.728	1.593	0.256
GCAT	NC_000022	0.68678	1	2.587	1.106	0.926
GCH1	NC_000014	1.51769	1	0.590	0.494	0.090
GDAP1	NC_000008	0.86443	1	1.977	0.168	0.161
GDF11	NC_000012	0.26729	1	1.958	0.642	0.540
GDF15	NC_000019	12.31646	1	0.154	0.520	0.030
GDF2	NC_000010	1.68210	1	0.876	0.803	0.463
GDF9	NC_000005	0.01127	1	0.000	11.384	2.118
GDNF	NC_000005	0.63462	1	3.415	0.517	0.163
GFRA1	NC_000010	0.72345	1	0.496	0.044	0.042
GGCT	NC_000007	0.95784	1	2.416	3.612	2.971
GGT1	NC_000022	0.19905	1	0.259	1.223	0.144
GHRL	NC_000003	0.03592	1	0.000	7.245	3.706
GLO1	NC_000006	13.02751	1	1.462	3.042	2.039
GLUD1	NC_000010	13.03140	1	1.481	2.213	1.570
GMPS	NC_000003	3.92991	1	1.493	3.818	3.827
GNA12	NC_000007	4.40301	1	1.136	1.444	2.372
GNA13	NC_000017	2.16509	1	1.393	2.279	1.279
GOLGA4	NC_000003	0.34003	1	1.257	1.512	3.660
GOT1	NC_000010	4.73235	1	1.332	2.173	0.927
GPX1	NC_000003	39.92513	1	0.923	0.459	0.270
GRN	NC_000017	42.54274	1	0.569	1.353	0.411
GSK3B	NC_000003	7.18487	1	1.158	2.147	1.539
GSTM1	NC_000001	3.90122	1	1.206	3.380	1.515
GSTM3	NC_000001	0.98774	1	1.514	1.492	0.240
GSTM5	NC_000001	0.05802	1	0.000	2.329	0.397
GSTP1	NC_000011	25.95849	1	1.163	2.222	1.912
GUCY1A2	NC_000011	0.49339	1	0.765	0.097	0.050
GUCY1B3	NC_000004	1.02335	1	1.185	1.056	0.283
HBEGF	NC_000005	1.12996	1	0.564	0.557	0.353
HCCS	NC_000023_X	1.05899	1	1.661	1.975	1.413
HDAC10	NC_000022	1.79676	1	0.891	0.342	0.547
HDAC4	NC_000002	0.21819	1	0.821	2.311	1.761
HGF	NC_000007	0.29237	1	0.041	0.019	0.029
HGS	NC_000017	3.27007	1	1.138	1.448	1.957
HINT1	NC_000005	18.84169	1	1.654	2.639	1.470
HMOX1	NC_000022	102.84631	1	0.645	0.112	0.158
HMOX2	NC_000016	5.49988	1	1.235	0.760	0.987
HNMT	NC_000002	0.44696	1	1.430	3.420	1.130
HNRNPH1	NC_000005	9.57909	1	2.042	1.310	1.164
HPS3	NC_000003	1.78562	1	0.750	4.338	2.139
HRG	NC_000003	0.04667	1	0.285	2.070	1.059
HRH2	NC_000005	0.03938	1	4.228	0.148	0.227
HS3ST3A1	NC_000017	0.25233	1	5.814	4.583	2.950
HSD11B1	NC_000001	0.26053	1	1.071	5.236	0.374
HSD11B2	NC_000016	0.02418	1	0.569	1.035	2.382

HSD17B11	NC_000004	3.03966	1	2.140	0.981	0.614
HSD17B6	NC_000012	0.13555	1	2.277	1.955	1.118
HSPB8	NC_000012	1.14889	1	1.307	1.691	2.736
HTRA1	NC_000010	30.48589	1	1.047	1.109	0.347
HUWE1	NC_000023_X	1.73264	1	1.493	2.168	3.630
IARS	NC_000009	4.73776	1	1.539	3.035	2.526
IDI1	NC_000010	7.89653	1	0.651	2.043	0.832
IDO1	NC_000008	1.60802	1	0.462	4.746	0.320
IFI27	NC_000014	220.57085	1	0.621	0.486	0.007
IFI30	NC_000019	55.21155	1	0.737	0.989	0.430
IFI44	NC_000001	13.48443	1	0.336	1.532	0.217
IFI44L	NC_000001	9.63418	1	0.307	1.758	0.077
IFIH1	NC_000002	2.64943	1	0.217	0.499	0.055
IFIT1	NC_000010	109.33785	1	0.406	0.506	0.021
IFIT2	NC_000010	9.50020	1	0.224	1.425	0.056
IFIT3	NC_000010	44.12290	1	0.400	0.769	0.057
IFITM1	NC_000011	105.32167	1	0.336	2.981	0.097
IFITM2	NC_000011	41.86509	1	0.256	2.571	0.653
IGF1R	NC_000015	0.63810	1	3.307	4.291	3.262
IGF2BP1	NC_000017	0.55316	1	4.249	2.517	2.105
IGF2BP2	NC_000003	1.53227	1	1.281	4.043	5.026
IGF2BP3	NC_000007	1.16631	1	0.369	4.271	1.675
IGFBP2	NC_000002	1.65623	1	0.109	0.010	0.046
IGFBP3	NC_000007	194.29419	1	0.395	0.042	0.017
IGJ	NC_000004	0.30709	1	1.917	0.272	0.613
IL11	NC_000019	0.00967	1	17.076	0.000	1.059
IL12A	NC_000003	0.90332	1	0.340	1.761	1.050
IL13RA2	NC_000023_X	0.34831	1	1.409	2.464	0.630
IL15RA	NC_000010	1.38019	1	0.653	2.681	1.020
IL16	NC_000015	0.03508	1	0.263	2.229	3.095
IL17RE	NC_000003	0.77878	1	1.162	0.338	0.078
IL17REL	NC_000022	0.00626	1	0.000	1.035	72.001
IL18R1	NC_000002	0.04526	1	1.789	10.202	3.706
IL1A	NC_000002	0.06222	1	3.273	5.563	2.581
IL1B	NC_000002	1.29220	1	0.496	6.587	0.367
IL1F7	NC_000002	0.02714	1	1.138	2.070	6.353
IL1RN	NC_000002	0.29100	1	11.257	4.255	0.726
IL20	NC_000001	0.01845	1	0.000	7.245	0.000
IL22RA1	NC_000001	0.05697	1	1.138	5.027	1.513
IL23A	NC_000012	0.34968	1	0.285	2.199	0.232
IL24	NC_000001	0.04612	1	0.000	12.937	0.265
IL32	NC_000016	2.11887	1	2.133	0.895	0.157
IL34	NC_000016	1.34710	1	0.036	0.142	0.072
IL6	NC_000007	4.18816	1	0.164	0.433	0.076
IL6R	NC_000001	0.24540	1	0.354	3.565	2.847
IL7R	NC_000005	3.30557	1	0.348	0.553	2.350
IL8	NC_000004	1.44657	1	9.931	3.410	0.323
ILF2	NC_000014	4.41441	1	0.688	0.560	0.429
IMPDH1	NC_000007	2.47751	1	2.399	0.728	0.565
INHBA	NC_000007	1.57056	1	2.664	0.062	0.198
INSIG1	NC_000007	12.43334	1	0.976	3.218	1.047
IRF1	NC_000005	4.27396	1	0.476	2.033	0.516
IRF4	NC_000006	0.00857	1	44.396	1.552	0.794
IRS1	NC_000002	2.98480	1	0.848	0.520	0.356
ISG15	NC_000001	178.29263	1	0.623	0.667	0.015
ISG20	NC_000015	7.33694	1	0.318	1.509	0.112
ISLR	NC_000015	27.73832	1	1.891	0.015	0.003
ISOC1	NC_000005	1.13748	1	2.418	3.318	1.446
KAT2B	NC_000003	1.39263	1	0.548	2.403	1.691
KCNA4	NC_000011	0.13237	1	1.138	0.345	2.168
KCNE1	NC_000021	0.02412	1	4.553	1.552	0.529
KCNE2	NC_000021	0.11344	1	1.992	3.622	1.059
KCNG1	NC_000020	0.20516	1	3.301	3.985	0.212
KCNJ12	NC_000017	0.07402	1	2.879	0.061	0.343
KCNJ8	NC_000012	0.65039	1	6.445	0.715	0.350
KCNK2	NC_000001	1.06338	1	3.147	0.221	0.205
KCNK6	NC_000019	0.47943	1	1.281	0.092	0.047
KCTD11	NC_000017	1.95986	1	0.970	0.362	0.207
KDM1	NC_000001	1.97668	1	2.207	2.345	1.977
LDHC	NC_000011	0.01774	1	7.969	2.070	1.059
LIPE	NC_000019	0.25729	1	0.980	0.481	0.542
LIPH	NC_000003	0.12887	1	0.732	5.175	2.571
LMBRD1	NC_000006	1.01636	1	1.213	3.401	1.361
LMO2	NC_000011	0.32977	1	1.268	0.207	0.091
LMO4	NC_000001	3.11366	1	2.549	1.239	0.757
LOXL1	NC_000015	40.64629	1	1.000	1.153	0.487

LOXL2	NC_000008	15.27173	1	1.295	0.571	0.448
LOXL4	NC_000010	4.03527	1	0.025	0.380	0.022
LPP	NC_000003	0.91293	1	1.573	3.137	3.349
LSM1	NC_000008	1.73086	1	1.155	1.079	0.416
LSM7	NC_000019	2.27731	1	2.211	1.513	2.077
LTF	NC_000003	0.05787	1	0.000	4.485	0.618
LYAR	NC_000004	0.08513	1	7.399	4.657	2.912
MAF1	NC_000008	10.28960	1	1.285	2.302	2.442
MALL	NC_000002	0.87789	1	11.239	0.061	0.220
MAOA	NC_000023_X	0.16774	1	0.114	4.761	0.547
MBNL1	NC_000003	5.62782	1	1.225	2.386	2.462
MBNL2	NC_000013	0.61484	1	1.882	3.602	2.935
MEST	NC_000007	20.99637	1	0.141	0.023	0.923
MFN1	NC_000003	3.01571	1	1.771	2.938	2.564
MGAM	NC_000007	0.01405	1	4.553	0.517	0.529
MGP	NC_000012	0.20672	1	0.190	0.000	0.088
MGST1	NC_000012	17.43941	1	1.359	2.026	1.084
MGST2	NC_000004	0.48332	1	0.603	1.339	0.436
MIB1	NC_000018	1.24533	1	1.876	2.594	2.259
MIPEP	NC_000013	2.39018	1	0.416	0.813	0.771
MLL3	NC_000007	1.10881	1	1.079	2.088	1.946
MLPH	NC_000002	2.10391	1	0.243	0.853	0.803
MRPL13	NC_000008	4.47256	1	2.235	3.869	2.164
MSI1	NC_000012	0.01544	1	3.415	1.552	0.794
MSLN	NC_000016	0.10970	1	0.114	0.517	0.371
MT1X	NC_000016	3.83310	1	1.088	0.350	0.008
MT2A	NC_000016	178.81512	1	1.192	0.669	0.268
MTAP	NC_000009	1.19371	1	1.310	2.684	2.391
MTHFD1L	NC_000006	1.30385	1	1.459	1.979	0.984
MVD	NC_000016	1.21795	1	0.356	0.765	0.678
MX1	NC_000021	17.82051	1	0.396	0.937	0.014
N4BP1	NC_000016	0.67621	1	0.415	0.893	0.713
N4BP2L1	NC_000013	0.19002	1	0.219	3.782	1.059
NAAA	NC_000004	0.45333	1	1.161	0.913	0.260
NAMPT	NC_000007	6.54565	1	0.714	10.360	0.973
NCBP2	NC_000003	1.59517	1	2.022	6.223	5.413
NCL	NC_000002	0.47776	1	2.257	1.035	2.378
NCOA4	NC_000010	3.19114	1	1.276	3.326	2.585
NCOA7	NC_000006	1.95794	1	0.468	1.642	0.529
NDRG4	NC_000016	0.17991	1	0.734	6.710	1.417
NDUFB4	NC_000003	10.71676	1	1.776	3.426	1.964
NDUFC2	NC_000011	6.43948	1	1.622	0.840	0.454
NDUFV2	NC_000018	9.27506	1	1.504	1.957	1.505
NEDD9	NC_000006	0.96247	1	1.090	0.101	0.126
NEGR1	NC_000001	1.60283	1	1.304	0.249	0.515
NETO2	NC_000016	3.16691	1	0.009	1.436	1.461
NGF	NC_000001	1.80188	1	1.468	0.536	0.402
NGFR	NC_000017	0.02003	1	3.795	0.345	0.176
NMB	NC_000015	4.13454	1	0.292	0.636	0.255
NONO	NC_000023_X	2.52641	1	1.700	1.939	3.205
NOX4	NC_000011	0.01461	1	11.004	0.345	1.235
NPC1	NC_000018	14.69623	1	0.535	2.679	2.022
NPM1	NC_000005	15.29437	1	2.224	2.448	1.720
NPR1	NC_000001	0.09795	1	1.644	0.690	0.324
NPTX1	NC_000017	0.86284	1	0.182	1.075	0.442
NPTX2	NC_000007	0.05036	1	1.897	0.172	0.176
NQO1	NC_000016	35.42972	1	0.346	0.786	0.330
NR2F1	NC_000005	1.02511	1	0.356	0.676	0.279
NRN1	NC_000006	0.01107	1	58.057	0.000	0.529
NTNG2	NC_000009	1.46614	1	0.029	0.031	0.029
NUDT5	NC_000010	7.56582	1	0.683	2.686	1.316
NUP107	NC_000012	2.81384	1	1.932	2.641	1.983
NUP37	NC_000012	2.71468	1	1.676	2.508	1.566
NUP62	NC_000019	0.67940	1	2.277	0.629	2.772
NVL	NC_000001	0.50154	1	1.903	2.248	1.588
NXT1	NC_000020	2.30382	1	1.491	0.485	0.398
OLFM1	NC_000009	0.52663	1	3.492	0.000	0.021
OLFML3	NC_000001	15.52940	1	2.695	0.289	0.060
OSGIN1	NC_000016	1.04900	1	0.379	0.433	0.294
P2RX7	NC_000012	0.57745	1	0.185	4.075	0.218
PA2G4	NC_000012	6.08786	1	1.975	2.655	2.708
PAPSS1	NC_000004	4.25540	1	0.949	0.963	0.430
PAQR3	NC_000004	1.45568	1	1.129	2.239	1.369
PARK2	NC_000006	0.08387	1	0.152	2.553	1.624
PARK7	NC_000001	1.82469	1	1.730	2.245	2.750
PCBD1	NC_000010	1.94681	1	0.741	2.467	1.761

PCOLCE	NC_000007	42.87530	1	0.940	0.567	0.196
PCSK1	NC_000005	0.66688	1	1.454	0.119	0.072
PCSK7	NC_000011	5.22919	1	0.859	0.520	0.345
PDE3A	NC_000012	0.83936	1	0.345	0.007	0.031
PDE4A	NC_000019	0.62584	1	0.674	0.340	0.038
PDE4B	NC_000001	0.65091	1	0.856	0.563	0.135
PDE4C	NC_000019	0.12621	1	0.391	0.194	0.199
PDE4D	NC_000005	0.12203	1	1.442	4.876	1.671
PDE5A	NC_000004	1.04667	1	0.231	0.169	0.542
PDE8A	NC_000015	2.41216	1	1.424	2.481	1.977
PDGFB	NC_000022	0.02654	1	2.277	2.070	6.221
PDGFRA	NC_000004	1.41336	1	1.959	0.023	0.034
PDK4	NC_000007	0.03693	1	0.190	2.070	2.029
PDZK1	NC_000001	0.03242	1	0.379	4.830	3.882
PDZRN3	NC_000003	0.33385	1	0.467	0.187	0.139
PELI1	NC_000002	0.32041	1	0.644	1.992	0.519
PERLD1	NC_000017	0.16103	1	1.198	4.902	3.594
PEX11A	NC_000015	0.46995	1	0.285	4.830	2.669
PEX19	NC_000001	4.37586	1	1.019	1.955	0.942
PHGDH	NC_000001	13.13268	1	1.452	1.259	0.413
PLAGL2	NC_000020	0.59188	1	1.053	2.682	2.957
PLEKHB1	NC_000011	0.11812	1	0.531	0.276	0.035
PLEKHB2	NC_000002	3.94353	1	0.949	1.006	0.720
PMAIP1	NC_000018	6.31672	1	0.850	1.866	0.428
PNPLA4	NC_000023_X	0.87921	1	0.864	0.564	0.364
PNRC1	NC_000006	4.31333	1	1.127	2.047	1.827
POLR3F	NC_000020	1.30041	1	1.240	0.673	0.448
POR	NC_000007	5.86870	1	1.395	0.877	0.479
POT1	NC_000007	1.69599	1	1.116	2.135	0.879
PPARG	NC_000003	1.96233	1	1.471	2.675	1.602
PPARGC1A	NC_000004	0.21266	1	0.135	1.228	0.843
PPIA	NC_000007	27.15563	1	1.352	1.966	1.389
PPIH	NC_000001	2.53351	1	2.187	2.151	1.660
PPP2CA	NC_000005	6.77248	1	1.582	3.372	2.471
PPP2R2B	NC_000005	0.01318	1	11.384	8.280	34.412
PPP2R5C	NC_000014	2.96638	1	1.478	0.652	0.449
PPP3CA	NC_000004	1.55847	1	1.042	2.765	2.010
PPP3CB	NC_000010	2.62433	1	1.073	1.235	2.051
PPP3CC	NC_000008	1.90383	1	0.684	0.848	0.460
PPP4C	NC_000016	22.39641	1	1.190	1.101	0.443
PRDM1	NC_000006	0.23116	1	4.343	2.242	0.490
PRDX2	NC_000019	2.13661	1	1.083	2.213	1.717
PRDX3	NC_000010	5.32470	1	1.123	2.251	1.765
PRDX6	NC_000001	13.29568	1	1.462	1.971	1.106
PRLR	NC_000005	0.01932	1	2.163	5.692	2.118
PROSC	NC_000008	1.36515	1	1.153	2.608	1.884
PRSS3	NC_000009	0.06705	1	42.499	0.000	0.353
PSMD2	NC_000003	16.30436	1	3.144	2.517	4.341
PSME2	NC_000014	41.81022	1	1.079	0.601	0.360
PTGER1	NC_000019	0.44873	1	0.041	0.444	0.113
PTGER2	NC_000014	2.86500	1	0.390	0.690	0.314
PTGER4	NC_000005	0.69333	1	0.394	1.642	0.224
PTGFR	NC_000001	0.18869	1	1.998	4.623	4.400
PTH1R	NC_000003	0.11772	1	0.931	0.565	0.096
PTN	NC_000007	0.41165	1	0.650	0.776	0.227
PTP4A3	NC_000008	0.27355	1	0.427	3.493	1.290
PTPN11	NC_000012	0.70316	1	1.584	1.734	3.660
QPCT	NC_000002	0.98955	1	1.523	1.594	0.114
RAE1	NC_000020	0.49324	1	1.453	2.598	2.343
RAGE	NC_000014	1.97822	1	0.481	0.246	0.268
RAI2	NC_000023_X	0.08184	1	8.538	10.479	46.721
RCAN2	NC_000006	0.79617	1	0.942	0.410	0.265
RDH12	NC_000014	0.01213	1	2.277	5.175	2.118
RNF139	NC_000008	2.11091	1	1.179	2.141	1.221
RNF144B	NC_000006	0.43941	1	0.421	2.092	0.719
ROR1	NC_000001	1.55624	1	1.256	0.600	0.447
RPL22	NC_000001	3.48423	1	1.388	2.667	1.919
RPL27	NC_000017	23.55522	1	1.567	2.595	2.243
RPL4	NC_000015	19.95828	1	1.889	4.828	5.223
RPL9	NC_000004	116.53130	1	1.041	0.480	1.355
RPP40	NC_000006	1.13964	1	2.198	0.535	0.712
RPS11	NC_000019	14.54637	1	1.730	2.429	1.839
RPS15	NC_000019	27.19049	1	2.207	2.468	2.394
RPS16	NC_000019	26.71016	1	2.081	1.895	1.021
RPS19	NC_000019	30.90062	1	1.414	0.910	0.448
RPS6	NC_000009	96.36679	1	1.554	2.392	2.063

RPS6KA1	NC_000001	0.10771	1	1.921	3.493	2.283
RRAD	NC_000016	0.15429	1	0.114	2.484	0.741
RSRC1	NC_000003	1.64457	1	1.177	4.026	2.813
RYR2	NC_000001	0.23100	1	0.885	0.929	0.386
S100A2	NC_000001	1.15876	1	1.580	2.070	0.713
SAT1	NC_000023_X	0.44006	1	1.366	4.036	2.224
SC4MOL	NC_000004	11.29606	1	0.810	2.036	0.514
SCAMP1	NC_000005	2.10808	1	1.239	2.304	1.170
SCG2	NC_000002	0.58864	1	0.516	0.776	0.149
SCG5	NC_000015	0.75056	1	0.333	0.101	0.103
SDHB	NC_000001	11.75195	1	1.730	1.955	0.913
SDPR	NC_000002	0.23332	1	0.379	5.206	2.118
SEC24B	NC_000004	1.93616	1	1.455	2.020	1.041
SEC31B	NC_000010	0.15307	1	0.294	1.569	2.237
SELENBP1	NC_000001	5.68994	1	0.521	0.270	0.021
SEMA5A	NC_000005	1.17260	1	2.138	0.512	0.433
SEMA5B	NC_000003	0.06290	1	2.890	1.115	1.629
SEMA6B	NC_000019	0.02453	1	1.708	35.705	22.897
SERBP1	NC_000001	0.89220	1	1.929	1.906	2.519
SERPINB4	NC_000018	0.06624	1	0.228	12.626	4.871
SIAH1	NC_000016	1.59284	1	0.507	0.775	0.457
SIDT2	NC_000011	1.28422	1	0.798	0.745	0.338
SIK1	NC_000021	0.24196	1	0.683	2.132	0.868
SIL1	NC_000005	1.51134	1	0.923	2.360	1.348
SLC12A6	NC_000015	1.11738	1	1.359	0.544	0.336
SLC12A7	NC_000005	1.41688	1	0.642	2.170	0.772
SLC12A8	NC_000003	1.17434	1	0.717	0.486	0.503
SLC15A2	NC_000003	0.00804	1	5.692	4.140	7.412
SLC16A1	NC_000001	3.40141	1	1.862	2.185	1.352
SLC16A2	NC_000023_X	0.88551	1	1.346	2.058	5.534
SLC16A4	NC_000001	3.13367	1	0.023	1.059	0.230
SLC16A6	NC_000017	0.02571	1	0.379	5.175	0.353
SLC16A8	NC_000022	0.09609	1	1.992	0.000	0.066
SLC16A9	NC_000010	0.25703	1	2.024	1.058	0.259
SLC17A9	NC_000020	5.71085	1	0.002	0.838	0.712
SLC19A1	NC_000021	1.08329	1	1.872	2.078	1.616
SLC19A2	NC_000001	0.81377	1	1.182	3.232	1.197
SLC1A2	NC_000011	0.01707	1	3.542	0.805	0.824
SLC1A3	NC_000005	0.45874	1	0.664	3.302	0.983
SLC1A7	NC_000001	0.10208	1	0.474	0.000	0.132
SLC22A18	NC_000011	1.76579	1	0.425	1.577	0.378
SLC22A4	NC_000005	0.73763	1	1.700	4.169	1.260
SLC22A5	NC_000005	0.43047	1	1.047	2.354	1.400
SLC23A3	NC_000002	0.04709	1	2.277	3.933	4.129
SLC25A10	NC_000017	1.52223	1	2.503	2.505	2.744
SLC25A18	NC_000022	0.03274	1	2.277	2.415	0.882
SLC25A23	NC_000019	1.54673	1	0.673	3.638	3.692
SLC25A27	NC_000006	0.01539	1	1.708	7.245	11.383
SLC25A46	NC_000005	4.84638	1	1.302	1.783	1.478
SLC25A6	NC_000023_X	3.94545	1	2.372	3.101	4.384
SLC26A1	NC_000004	0.01113	1	5.123	1.552	2.118
SLC26A2	NC_000005	0.67967	1	2.310	3.032	2.412
SLC27A2	NC_000015	0.00972	1	9.107	2.070	1.588
SLC27A5	NC_000019	0.00963	1	11.384	7.245	1.588
SLC29A1	NC_000006	4.27298	1	1.093	2.243	1.235
SLC29A3	NC_000010	0.06027	1	2.277	3.450	1.147
SLC29A4	NC_000007	0.07877	1	3.757	2.380	0.265
SLC2A14	NC_000012	0.25937	1	0.911	2.070	0.360
SLC2A5	NC_000001	4.70618	1	0.025	0.396	0.067
SLC33A1	NC_000003	2.02980	1	1.268	4.061	2.153
SLC35C1	NC_000011	1.14618	1	1.311	0.669	0.366
SLC35E2	NC_000001	0.20703	1	1.751	2.229	1.140
SLC37A2	NC_000011	0.07056	1	4.553	0.478	0.896
SLC38A3	NC_000003	0.04642	1	2.049	0.621	0.212
SLC38A4	NC_000012	0.04002	1	4.716	0.296	0.000
SLC39A5	NC_000012	0.05399	1	0.000	0.621	2.012
SLC40A1	NC_000002	0.44121	1	0.841	0.414	0.171
SLC43A2	NC_000017	0.69055	1	2.502	1.638	0.273
SLC46A3	NC_000013	0.46295	1	0.335	1.385	0.615
SLC47A1	NC_000017	1.02788	1	0.215	1.441	0.512
SLC4A3	NC_000002	0.11649	1	2.380	2.728	2.431
SLC6A15	NC_000012	0.99620	1	1.815	6.861	3.390
SLC6A17	NC_000001	0.27030	1	3.610	5.161	5.468
SLC6A8	NC_000023_X	1.58926	1	0.437	3.726	3.602
SLC7A14	NC_000003	0.77985	1	3.304	0.012	0.009
SLC7A8	NC_000014	1.61956	1	0.543	0.357	0.254

SLC9A10	NC_000003	0.01105	1	0.569	6.210	3.177
SLCO4A1	NC_000020	0.13126	1	0.711	17.271	9.265
SMS	NC_000023_X	12.66822	1	2.056	1.668	1.903
SNRPE	NC_000001	1.28071	1	1.361	2.772	2.556
SOD2	NC_000006	96.96524	1	0.575	8.963	1.387
SORT1	NC_000001	1.62572	1	0.389	1.684	1.834
SPINK1	NC_000005	0.20656	1	3.415	5.433	0.265
SPINT2	NC_000019	0.45488	1	0.220	0.033	0.017
SPRY2	NC_000013	3.91598	1	0.370	0.262	0.222
SRPX	NC_000023_X	5.53044	1	2.429	3.848	1.042
STARD13	NC_000013	2.60673	1	0.461	0.461	0.687
STC1	NC_000008	2.32019	1	0.807	4.237	3.152
STRA6	NC_000015	0.02098	1	3.984	0.259	0.397
STS	NC_000023_X	1.82842	1	0.747	1.587	2.290
STXBP6	NC_000014	0.11560	1	2.049	0.517	0.318
SULT1A1	NC_000016	0.57402	1	0.373	1.487	0.424
SULT1A2	NC_000016	0.09788	1	2.656	2.242	1.676
SULT1C2	NC_000002	0.15760	1	0.171	1.190	0.079
SUV39H2	NC_000010	0.28771	1	1.489	3.582	3.299
SUV420H1	NC_000011	0.20330	1	1.073	2.766	3.309
SYNPO2	NC_000004	0.13054	1	2.406	0.485	0.563
TAP1	NC_000006	20.66706	1	0.521	1.269	0.162
TBC1D4	NC_000013	1.02001	1	0.312	1.177	0.973
TDO2	NC_000004	0.05381	1	0.569	5.433	0.926
TFAM	NC_000010	1.59976	1	1.097	2.131	1.261
TFRC	NC_000003	9.88340	1	1.433	5.915	3.882
THRB	NC_000003	0.15409	1	2.868	3.901	2.464
TMEM27	NC_000023_X	0.07262	1	0.911	1.656	0.212
TMEM97	NC_000017	1.77361	1	1.873	2.204	2.282
TMPO	NC_000012	7.08634	1	1.603	2.186	1.542
TNFRSF11A	NC_000018	0.14570	1	3.984	3.260	2.965
TRA2A	NC_000007	1.31122	1	1.471	0.478	0.629
TRIP13	NC_000005	2.62506	1	2.264	2.505	1.678
TSPO	NC_000022	40.73389	1	0.642	0.649	0.447
UQCRH	NC_000001	10.84640	1	1.610	2.589	1.995
(i) Markers						
ACTA2	NC_000010	14.89910	1	0.131	0.289	0.063
ACTG2	NC_000002	0.11977	1	0.488	0.000	0.151
AIM1	NC_000006	1.13670	1	1.528	2.586	2.870
ALPL	NC_000001	1.55405	1	1.181	0.017	0.103
BAALC	NC_000008	1.91841	1	1.613	0.660	0.108
BCAS1	NC_000020	0.02621	1	3.131	1.294	0.794
BEX2	NC_000023_X	0.39264	1	0.455	2.415	0.459
BEX4	NC_000023_X	1.12350	1	1.084	2.349	0.277
CALD1	NC_000007	28.74768	1	1.014	0.374	0.279
CD34	NC_000001	0.25925	1	2.383	0.420	0.165
CD44	NC_000011	51.75201	1	1.549	1.237	1.009
CNN1	NC_000019	0.28718	1	4.269	0.052	0.026
DIRC2	NC_000003	0.96224	1	2.002	3.567	1.904
DMBT1	NC_000010	0.01778	1	0.379	5.692	1.765
ENG	NC_000009	12.84016	1	0.743	0.642	0.786
FLCN	NC_000017	5.04571	1	0.923	0.467	0.358
GALR2	NC_000017	0.08772	1	1.138	0.000	0.212
GFAP	NC_000017	0.02482	1	2.277	2.329	1.588
GPC1	NC_000002	7.12355	1	0.715	0.852	1.022
GPC2	NC_000007	0.06296	1	0.650	0.148	0.076
GPC3	NC_000023_X	0.00000				
GPC4	NC_000023_X	0.18395	1	1.176	0.241	0.088
GPC5	NC_000013	0.00790	1	0.000	71.411	135.002
GPC6	NC_000013	1.99100	1	0.115	2.037	0.975
HMGA2	NC_000012	2.57921	1	1.642	3.408	3.061
HTT	NC_000004	1.56834	1	1.240	1.667	2.095
IQGAP1	NC_000015	2.47630	1	1.130	2.029	3.840
IQGAP2	NC_000005	0.01579	1	4.838	7.503	11.383
IQGAP3	NC_000001	0.65475	1	1.191	1.231	2.060
KIAA1199	NC_000015	42.95047	1	0.602	0.039	0.946
LAG3	NC_000012	0.02286	1	0.000	6.727	9.265
LMOD1	NC_000001	0.29851	1	0.000	0.458	0.071
LYN	NC_000008	0.68496	1	0.233	3.049	1.383
MBP	NC_000018	0.45216	1	0.324	1.458	0.699
MEOX2	NC_000007	1.91137	1	0.029	0.031	0.080
MFI2	NC_000003	1.08078	1	1.564	3.058	0.447
MLANA	NC_000009	0.04483	1	3.036	1.725	2.647
MLF1	NC_000003	0.76499	1	2.321	8.903	3.570
MN1	NC_000022	0.24714	1	10.273	0.025	0.071
MSX2	NC_000005	0.51570	1	0.592	0.083	0.095

MYL12A	NC_000018	79.32603	1	0.764	1.288	0.484
MYLK	NC_000003	1.54455	1	1.087	2.590	2.723
MYOCD	NC_000017	0.00808	1	4.553	0.000	0.529
NT5E	NC_000006	61.60584	1	0.684	2.084	0.568
OSR2	NC_000017	3.37785	1	1.063	0.898	0.650
PGA3	NC_000011	0.04894	1	2.656	0.000	0.176
PITX1	NC_000005	3.78437	1	0.333	1.944	1.126
PLP1	NC_000023_X	0.25199	1	0.423	1.212	0.257
PMP22	NC_000017	11.93641	1	1.075	0.404	0.299
POSTN	NC_000013	10.13336	1	0.703	0.006	0.003
PRNP	NC_000020	15.50477	1	2.071	0.578	0.273
PROM2	NC_000002	0.01788	1	1.138	6.210	5.118
PRPF38B	NC_000001	0.19664	1	1.067	1.100	2.647
PRRX1	NC_000001	10.53347	1	2.433	0.695	0.522
PSCA	NC_000008	0.78143	1	0.390	1.508	1.467
PSG2	NC_000019	0.01486	1	1.138	8.280	12.706
PTMA	NC_000002	11.74474	1	1.705	3.148	2.259
PTPRC	NC_000001	0.01109	1	0.759	0.345	0.706
RRAGD	NC_000006	1.06330	1	1.188	2.097	0.506
RTN1	NC_000014	0.07364	1	0.759	2.415	0.750
SILV	NC_000012	0.08569	1	1.708	2.070	0.529
SMTN	NC_000022	2.82763	1	1.169	0.316	0.572
SNCA	NC_000004	0.14759	1	0.228	3.105	3.018
SNCAIP	NC_000005	0.05743	1	1.897	0.460	0.294
SNCG	NC_000010	0.22056	1	0.854	1.035	0.265
SNPH	NC_000020	0.64297	1	2.043	0.895	0.432
SULF1	NC_000008	5.68668	1	0.912	0.008	0.001
SYNGR1	NC_000022	0.21681	1	1.741	0.061	0.021
TAGLN	NC_000011	126.76593	1	0.651	0.013	0.010
TEX14	NC_000017	0.00462	1	4.553	3.105	1.588
THY1	NC_000011	21.20550	1	1.151	0.417	0.153
TPD52	NC_000008	0.09127	1	1.644	10.464	12.294
TPD52L1	NC_000006	0.78287	1	0.244	2.791	1.380
TPD52L3	NC_000009	0.27574	1	1.897	2.415	1.697
UNC119B	NC_000012	1.69934	1	1.338	2.151	2.553
(j) Adhesion, Cell Menbrane and Cytoskeleton						
A1BG	NC_000019	0.25791	1	0.455	1.138	0.344
ACTC1	NC_000015	0.24050	1	1.401	0.716	0.462
ADAM12	NC_000010	4.87739	1	2.488	0.683	2.191
ADAM23	NC_000002	0.67813	1	2.377	1.012	0.093
ADAM8	NC_000010	0.13252	1	0.359	8.225	0.446
ADRA2C	NC_000004	0.40708	1	0.976	0.059	0.091
AFAP1	NC_000004	3.42916	1	1.008	0.343	0.395
AGRN	NC_000001	2.56699	1	0.723	3.564	0.380
AKAP13	NC_000015	0.33096	1	1.494	2.218	3.502
ANK3	NC_000010	0.18981	1	0.921	0.474	0.295
ANO6	NC_000012	3.17988	1	1.992	2.390	1.836
ANXA1	NC_000009	100.15936	1	0.828	2.782	1.252
ANXA2	NC_000015	184.68590	1	1.641	0.608	0.476
ANXA3	NC_000004	0.04222	1	5.692	2.760	2.471
ANXA9	NC_000001	0.08740	1	0.000	2.809	0.681
AP3M2	NC_000008	1.18519	1	1.413	2.216	1.270
APBA2	NC_000015	0.15645	1	1.503	0.041	0.042
AQP3	NC_000009	0.29965	1	0.617	0.690	0.221
ARHGEF2	NC_000001	2.78729	1	0.919	2.070	1.499
ARPC5	NC_000001	12.04148	1	1.401	3.523	1.480
ARRDC3	NC_000005	4.36197	1	0.964	4.029	0.915
ASB5	NC_000004	0.03110	1	2.561	0.259	0.926
ATRNL1	NC_000010	0.17734	1	0.653	1.035	0.459
B4GALNT1	NC_000012	1.30889	1	2.310	2.740	1.013
BAMBI	NC_000010	0.88503	1	0.934	0.278	0.087
BANK1	NC_000004	0.63365	1	0.080	0.742	0.225
BCAM	NC_000019	0.23831	1	1.103	2.975	0.463
BCAN	NC_000001	0.01689	1	1.138	5.865	5.824
BCAR1	NC_000016	11.13525	1	0.431	0.586	0.572
BDKRB1	NC_000014	11.43072	1	0.799	0.219	0.153
BDKRB2	NC_000014	2.09445	1	0.587	0.432	0.141
BGN	NC_000023_X	11.75973	1	1.715	0.246	0.126
BMPR1A	NC_000010	2.49396	1	1.190	2.146	1.537
BSG	NC_000019	25.94724	1	0.936	2.073	0.844
BST2	NC_000019	3.73489	1	0.436	0.228	0.010
C3	NC_000019	2.18758	1	0.623	8.467	0.378
C4B	NC_000006	0.23919	1	1.078	9.115	1.152
C9	NC_000005	0.07611	1	0.126	1.265	0.294
CACNA1A	NC_000019	0.23222	1	1.548	0.465	0.351
CACNA1C	NC_000012	0.25456	1	1.467	0.046	0.132

CACNA1G	NC_000017	0.02980	1	6.209	1.129	0.337
CALCRL	NC_000002	0.15944	1	0.325	0.769	0.318
CAPG	NC_000002	86.19670	1	0.260	2.851	1.594
CASP1	NC_000011	2.76902	1	0.290	0.909	0.388
CD14	NC_000005	0.59998	1	0.025	1.147	0.242
CD177	NC_000019	0.04817	1	0.911	2.484	3.177
CD2	NC_000001	0.02975	1	1.138	4.657	0.794
CD247	NC_000001	0.01355	1	1.138	5.175	1.588
CD248	NC_000011	29.61039	1	0.853	0.518	0.114
CD27	NC_000012	0.44856	1	0.394	0.557	0.305
CD40	NC_000020	0.52141	1	2.277	0.000	0.029
CD58	NC_000001	1.48755	1	0.849	2.117	1.352
CD68	NC_000017	22.74857	1	0.905	0.806	0.279
CD70	NC_000019	6.36051	1	0.067	1.932	0.876
CD74	NC_000005	1.97046	1	0.035	13.431	1.944
CD97	NC_000019	5.77604	1	1.286	2.499	0.948
CDH1	NC_000016	0.01892	1	3.700	0.259	0.662
CDH11	NC_000016	9.93827	1	0.940	0.074	0.087
CDH12	NC_000005	0.01048	1	0.569	2.587	4.765
CDH13	NC_000016	8.31088	1	0.403	0.759	0.335
CDH15	NC_000016	0.15134	1	0.719	0.163	0.223
CDH18	NC_000005	0.80613	1	1.662	0.021	0.069
CDH2	NC_000018	2.44053	1	3.904	2.101	1.990
CDH4	NC_000020	0.11908	1	4.767	0.712	0.165
CDH6	NC_000005	0.24682	1	0.046	0.021	0.011
CDH8	NC_000016	0.18660	1	0.759	0.086	0.132
CDH9	NC_000005	0.02962	1	0.285	0.776	0.132
CEACAM1	NC_000019	0.21490	1	0.517	1.098	0.353
CFB	NC_000006	11.21751	1	0.186	7.557	0.289
CFH	NC_000001	3.24735	1	0.279	0.342	0.034
CFI	NC_000004	1.69665	1	0.198	1.273	0.233
CHI3L1	NC_000001	311.02589	1	0.002	2.198	0.400
CHIC2	NC_000004	4.27122	1	0.782	0.834	0.339
CHRNA10	NC_000011	0.01171	1	1.138	2.070	6.882
CHRNA7	NC_000015	0.07343	1	0.163	2.218	1.664
CHRNB1	NC_000017	0.13083	1	1.789	1.257	0.454
CHRNB2	NC_000001	0.77251	1	0.980	0.694	0.287
CHRNB4	NC_000015	0.02791	1	2.656	2.070	0.882
CHRNG	NC_000002	0.02083	1	3.415	4.140	3.706
CLDN1	NC_000003	0.25237	1	0.479	7.326	1.379
CLDN11	NC_000003	31.68110	1	0.555	0.016	0.002
CLDN4	NC_000007	0.02498	1	1.708	2.070	2.118
CLDN7	NC_000017	0.26618	1	0.379	4.312	0.412
CLEC11A	NC_000019	7.10547	1	0.239	0.344	0.086
CLEC16A	NC_000016	0.37344	1	1.380	1.520	2.586
CMTM4	NC_000016	0.40962	1	1.037	2.063	1.505
CMTM8	NC_000003	0.21411	1	2.484	0.188	0.096
COL12A1	NC_000006	8.13452	1	0.997	0.279	0.419
COL13A1	NC_000010	19.74006	1	2.063	0.720	1.330
COL15A1	NC_000009	0.29368	1	8.337	0.030	0.047
COL16A1	NC_000001	2.09216	1	2.337	0.329	0.223
COL1A1	NC_000017	12.99070	1	3.343	0.235	0.149
COL1A2	NC_000007	9.02760	1	1.849	0.907	0.422
COL3A1	NC_000002	4.72469	1	1.262	0.300	0.153
COL4A1	NC_000013	4.44166	1	0.489	0.347	0.267
COL4A2	NC_000013	14.57130	1	0.500	0.787	0.222
COL4A4	NC_000002	0.09690	1	0.699	3.669	1.648
COL4A5	NC_000023_X	0.01060	1	3.036	87.625	38.295
COL5A1	NC_000009	1.29261	1	2.764	0.503	0.372
COL6A1	NC_000021	53.77149	1	0.820	0.603	0.177
COL6A2	NC_000021	72.27919	1	0.913	0.670	0.106
COL7A1	NC_000003	0.25085	1	2.716	0.594	0.330
COL8A1	NC_000003	8.59346	1	0.279	0.519	0.254
COLEC12	NC_000018	4.95573	1	1.372	0.120	0.020
CRIP1	NC_000014	7.06915	1	2.712	0.597	0.689
CRYBB2	NC_000022	0.35816	1	0.664	0.345	0.529
CRYL1	NC_000013	0.63722	1	0.949	4.633	1.765
CRYZ	NC_000001	0.66195	1	1.920	3.259	2.529
CTNNA1	NC_000005	1.41689	1	1.090	1.464	2.860
CTNNA3	NC_000010	0.14884	1	1.294	0.565	0.265
CTNNAL1	NC_000009	3.91195	1	1.152	1.952	1.411
CXADR	NC_000021	0.01835	1	2.277	44.502	14.030
DES	NC_000002	0.04052	1	0.854	0.259	0.397
DIAPH1	NC_000005	9.41210	1	1.162	1.255	2.200
DMD	NC_000023_X	0.16817	1	1.178	0.366	0.173
DNALI1	NC_000001	0.53301	1	0.312	3.238	1.195

DPT	NC_000001	0.04012	1	2.277	0.690	0.176
DSC3	NC_000018	0.00979	1	0.379	0.690	0.706
DSG1	NC_000018	0.01672	1	0.569	0.517	0.265
DSG2	NC_000018	2.16733	1	1.672	3.738	1.499
DSG4	NC_000018	0.00615	1	2.277	0.000	1.059
DST	NC_000006	0.96542	1	1.190	1.624	4.849
DTNA	NC_000018	0.96089	1	0.235	2.935	2.078
DYNLRB1	NC_000020	12.26756	1	0.824	2.244	1.911
ECE2	NC_000003	0.12008	1	3.465	6.300	4.212
ECM1	NC_000001	19.16170	1	0.988	0.928	0.372
ECM2	NC_000009	0.41759	1	0.098	0.339	0.018
EFEMP1	NC_000002	36.10353	1	0.248	0.441	1.092
ELN	NC_000007	15.79362	1	3.209	0.004	0.008
EMCN	NC_000004	0.06142	1	7.684	0.000	0.265
EML1	NC_000014	1.80291	1	1.084	0.405	0.435
EMP3	NC_000019	16.31752	1	1.458	0.725	0.345
EPB41L3	NC_000018	0.02049	1	9.392	0.000	0.265
EPB41L4B	NC_000009	0.37474	1	0.407	1.626	1.046
EPCAM	NC_000002	0.06628	1	0.000	3.726	1.059
EPDR1	NC_000007	7.43040	1	0.644	0.653	0.370
EPHB2	NC_000001	0.18241	1	1.343	9.288	2.321
EPOR	NC_000019	0.92423	1	1.503	2.925	1.969
ERRFI1	NC_000001	9.58523	1	0.274	1.044	0.523
ESR1	NC_000006	0.08169	1	0.501	0.207	0.296
ESR2	NC_000014	0.01338	1	5.123	2.070	2.382
ESRRA	NC_000011	3.08632	1	1.271	2.259	1.500
EXT2	NC_000011	11.01071	1	1.505	2.599	0.985
EXTL3	NC_000008	2.05888	1	2.132	0.916	0.655
FABP4	NC_000008	0.21740	1	0.142	0.388	0.066
FABP5	NC_000008	1.41469	1	1.000	1.111	0.336
FCGR2A	NC_000001	0.06563	1	2.927	9.906	3.328
FCN3	NC_000001	0.02207	1	6.830	1.035	2.118
FGD4	NC_000012	2.86345	1	0.165	0.034	0.129
FGFBP1	NC_000004	0.01927	1	4.553	0.000	1.059
FHOD3	NC_000018	0.25344	1	2.360	2.484	1.040
FLRT2	NC_000014	1.02462	1	0.627	0.266	0.421
FMOD	NC_000001	1.37279	1	1.164	0.011	0.009
FN1	NC_000002	304.89636	1	0.571	0.358	0.108
FNTA	NC_000008	3.44151	1	1.725	2.575	1.324
FSCN1	NC_000007	15.09733	1	1.616	0.485	0.333
FST	NC_000005	20.68640	1	0.310	0.378	0.503
FUCA1	NC_000001	2.21830	1	0.393	1.529	0.681
FUT10	NC_000008	0.24384	1	2.337	2.097	1.574
FUT4	NC_000011	0.50456	1	1.130	0.587	0.478
FYCO1	NC_000003	0.77919	1	0.876	1.632	2.090
GABBR2	NC_000009	0.00831	1	8.538	1.552	1.324
GABRA2	NC_000004	0.05169	1	1.301	2.070	0.832
GABRE	NC_000023_X	0.31067	1	1.350	1.564	4.223
GALNT3	NC_000002	0.20912	1	0.569	0.103	0.106
GALNTL4	NC_000011	0.40747	1	1.518	1.081	0.071
GART	NC_000021	2.68010	1	1.178	2.580	1.271
GHR	NC_000005	0.12013	1	1.534	2.430	0.737
GJA1	NC_000006	37.42648	1	0.248	0.239	0.261
GPM6A	NC_000004	0.01199	1	1.138	4.140	1.588
GPM6B	NC_000023_X	0.16135	1	0.667	0.321	0.986
GPR126	NC_000006	0.27808	1	6.549	0.548	0.442
GPR137	NC_000011	3.19741	1	0.552	1.380	1.394
GPR39	NC_000002	0.46822	1	2.983	0.571	1.515
GPR56	NC_000016	0.97792	1	0.426	1.099	1.401
GPRC5A	NC_000012	7.44459	1	0.770	0.458	1.395
GRB7	NC_000017	0.21849	1	0.103	5.410	1.444
GRIA1	NC_000005	0.01948	1	3.415	0.207	1.165
GRIA3	NC_000023_X	0.28805	1	1.478	0.587	0.466
GRIK1	NC_000021	0.01236	1	6.261	4.657	6.088
GRIN2A	NC_000016	0.08164	1	8.849	0.488	1.498
GRIN2C	NC_000017	0.03739	1	1.464	1.626	5.143
GRIN2D	NC_000019	0.66625	1	1.688	1.042	0.266
GRINL1A	NC_000015	0.07180	1	4.466	3.184	0.733
GRPR	NC_000023_X	0.04247	1	4.098	0.414	0.847
GSN	NC_000009	2.15903	1	1.510	0.926	0.358
HAPLN1	NC_000005	2.96469	1	2.832	0.002	0.005
HAS2	NC_000008	10.22878	1	1.490	0.114	0.122
HLA-A	NC_000006	209.03318	1	0.315	1.815	0.260
HLA-B	NC_000006	325.09629	1	0.296	1.460	0.187
HLA-DOA	NC_000006	0.13069	1	0.968	0.207	0.053
HLA-DRA	NC_000006	0.18189	1	0.114	13.247	1.429

HLA-E	NC_000006	82.87256	1	0.464	1.600	0.371
HLA-G	NC_000006	2.49667	1	0.270	0.993	0.208
HOOK3	NC_000008	0.47786	1	1.612	2.326	2.600
HTR3D	NC_000003	2.09141	1	0.394	0.300	0.199
HYAL1	NC_000003	0.07014	1	1.518	1.610	0.176
ICAM1	NC_000019	6.95962	1	0.314	0.541	0.064
ICAM2	NC_000017	1.61490	1	0.664	0.901	0.108
ICAM5	NC_000019	0.09862	1	0.438	0.557	0.081
IFNAR2	NC_000021	0.62352	1	1.258	3.514	1.385
IFNGR1	NC_000006	4.56536	1	1.262	2.140	0.649
IFNGR2	NC_000021	9.44351	1	1.067	1.533	0.377
IGDCC4	NC_000015	0.22123	1	0.560	0.115	0.059
IGFBP4	NC_000017	154.91011	1	0.368	0.186	0.118
IGFBP5	NC_000002	1.59875	1	2.643	0.033	0.002
IGFBP6	NC_000012	37.01220	1	1.143	0.177	0.070
IGFBP7	NC_000004	59.12802	1	0.333	0.285	0.121
ITGA1	NC_000005	2.03230	1	0.888	2.836	1.260
ITGA10	NC_000001	0.72240	1	0.784	0.107	0.110
ITGA11	NC_000015	2.99610	1	0.834	0.063	0.100
ITGA2	NC_000005	0.61932	1	7.437	2.718	1.319
ITGA3	NC_000017	18.17047	1	0.281	7.544	1.700
ITGA6	NC_000002	1.96374	1	1.229	4.024	2.653
ITGA7	NC_000012	1.41333	1	0.083	2.784	1.099
ITGA9	NC_000003	0.03449	1	2.656	1.725	1.324
ITGAE	NC_000017	0.25362	1	2.224	1.420	1.625
ITGB2	NC_000021	0.99990	1	0.041	0.645	0.046
ITGB4	NC_000017	0.07868	1	0.867	1.922	3.076
ITGB7	NC_000012	0.07397	1	0.253	1.035	1.176
ITGB8	NC_000007	1.51267	1	0.283	1.042	1.600
ITGBL1	NC_000013	0.98617	1	0.822	0.450	0.902
ITPR1	NC_000003	0.21022	1	0.363	1.899	1.536
JUP	NC_000017	0.62321	1	0.628	0.205	0.061
KIF13A	NC_000006	1.08863	1	1.393	2.351	2.433
KIF20B	NC_000010	1.24873	1	1.829	2.812	2.563
KIF22	NC_000016	0.66277	1	1.922	1.900	2.951
KIF2A	NC_000005	2.97542	1	1.475	2.256	2.195
KIF5B	NC_000010	1.60870	1	1.040	1.217	2.138
KIFC1	NC_000006	0.38713	1	1.411	2.025	2.762
KIFC2	NC_000008	0.93252	1	0.488	1.875	2.520
KLHL20	NC_000001	0.60483	1	1.361	0.979	0.455
KRIT1	NC_000007	0.52982	1	1.698	2.640	1.718
KRT1	NC_000012	0.05575	1	2.656	1.035	0.882
KRT15	NC_000017	0.14852	1	1.044	3.967	4.235
KRT16	NC_000017	0.11930	1	3.795	0.000	0.235
KRT17	NC_000017	0.11575	1	0.285	0.647	0.066
KRT18	NC_000012	0.01484	1	253.857	0.000	1.059
KRT19	NC_000017	6.30092	1	0.011	0.473	0.303
KRT222P	NC_000017	0.01313	1	36.428	3.105	1.059
KRT7	NC_000012	0.88309	1	18.248	0.643	0.489
KRT8	NC_000012	0.01268	1	50.088	11.384	2.118
KRTAP1-1	NC_000017	0.17654	1	0.325	0.000	0.454
KRTAP1-5	NC_000017	5.72714	1	0.127	0.210	0.485
KRTAP2-2	NC_000017	0.41547	1	0.142	0.129	0.232
KRTAP3-2	NC_000017	0.19689	1	0.949	0.000	0.088
L1CAM	NC_000023_X	0.04981	1	3.519	0.565	0.241
LAD1	NC_000001	0.07219	1	1.644	2.185	0.176
LAMA3	NC_000018	0.49794	1	0.365	2.065	2.962
LAMA4	NC_000006	10.83520	1	0.356	1.787	1.126
LAMB3	NC_000001	0.90627	1	1.762	4.521	0.580
LAMP3	NC_000003	0.02045	1	12.902	4.485	0.176
LASS2	NC_000001	4.40007	1	1.077	2.647	0.937
LASS4	NC_000019	0.36347	1	1.963	0.464	0.146
LEPR	NC_000001	0.52592	1	0.803	2.516	1.567
LGALS1	NC_000022	665.52171	1	1.008	0.790	0.493
LGALS3	NC_000014	79.43809	1	0.932	0.812	0.226
LGALS3BP	NC_000017	77.47896	1	0.493	0.421	0.059
LPAR1	NC_000009	16.54789	1	0.505	0.905	0.466
LPCAT2	NC_000016	1.59767	1	2.852	1.353	0.934
LRP1	NC_000012	5.85485	1	1.243	2.956	1.083
LRP8	NC_000001	1.07266	1	1.217	2.771	1.720
LSP1	NC_000011	0.17812	1	1.366	0.310	0.212
LSR	NC_000019	0.16995	1	2.009	0.609	1.277
LTB4R	NC_000014	0.15904	1	0.661	0.701	0.478
LY6E	NC_000008	57.53309	1	0.494	1.746	0.279
LYPLA1	NC_000008	1.91103	1	1.571	2.103	1.480
MASP1	NC_000003	0.17222	1	16.974	1.035	0.293

MCAM	NC_000011	2.44681	1	0.194	0.173	0.041
MGAT4B	NC_000005	8.45762	1	1.060	2.106	2.050
MICB	NC_000006	4.82427	1	1.004	2.013	1.071
MME	NC_000003	32.92889	1	1.328	10.168	1.377
MR1	NC_000001	4.32743	1	0.792	1.194	0.474
MTMR6	NC_000013	3.01691	1	0.713	0.689	0.418
MUC13	NC_000003	0.07960	1	2.618	1.242	1.429
MXRA5	NC_000023_X	0.03255	1	5.529	0.591	0.038
MYEF2	NC_000015	0.22760	1	1.181	0.402	0.029
MYH10	NC_000017	0.15244	1	2.589	0.528	0.488
NEFM	NC_000008	0.13813	1	0.207	0.517	0.770
NES	NC_000001	0.57773	1	2.253	2.305	2.655
NKTR	NC_000003	0.59256	1	0.977	1.324	1.981
NOV	NC_000008	5.79615	1	1.721	0.339	0.268
NR3C2	NC_000004	0.05820	1	3.719	4.278	4.306
NRCAM	NC_000007	0.10561	1	0.883	0.146	0.157
NSD1	NC_000005	0.54409	1	1.624	1.927	2.522
NTSR1	NC_000020	0.03307	1	20.680	0.862	0.088
OBSCN	NC_000001	0.03010	1	1.205	2.039	1.324
OCLN	NC_000005	0.56070	1	0.648	4.076	1.678
OPCML	NC_000011	0.02784	1	6.546	0.388	0.529
OVGP1	NC_000001	0.04063	1	2.561	1.035	2.647
P2RY6	NC_000011	0.21402	1	0.675	4.216	1.726
PCDH10	NC_000004	0.50269	1	1.244	0.120	0.098
PDGFRB	NC_000005	9.95975	1	0.544	0.345	0.159
PDGFRL	NC_000008	3.06270	1	1.471	1.573	0.168
PDLIM3	NC_000004	0.43183	1	0.629	1.226	0.042
PDLIM4	NC_000005	4.20732	1	1.976	3.263	0.697
PDPN	NC_000001	0.22926	1	0.152	0.034	0.018
PER1	NC_000017	0.73025	1	0.618	1.158	0.452
PFDN6	NC_000006	3.37379	1	1.371	2.611	1.733
PLCB4	NC_000020	0.84040	1	0.184	0.538	1.160
PLD1	NC_000003	0.21173	1	0.898	10.389	6.658
PLD3	NC_000019	17.39275	1	0.685	0.806	0.296
PLEK2	NC_000014	1.29464	1	3.648	1.534	0.657
PNN	NC_000014	0.08841	1	1.382	1.331	2.155
POPDC2	NC_000003	0.20778	1	0.304	0.552	0.318
PPAP2A	NC_000005	3.47104	1	1.063	1.146	0.402
PPAP2B	NC_000001	44.60598	1	0.876	0.343	0.366
PPL	NC_000016	0.18254	1	0.182	3.933	2.753
PRELP	NC_000001	0.11347	1	0.157	0.178	0.219
PTK2	NC_000008	2.36728	1	1.644	2.588	1.892
PTPRJ	NC_000011	0.79473	1	1.679	3.134	2.297
PTX3	NC_000003	73.11367	1	0.213	0.122	0.008
PVRL2	NC_000019	5.07060	1	1.050	1.073	0.314
PVRL3	NC_000003	1.76664	1	2.223	2.466	1.427
PVRL4	NC_000001	0.18858	1	0.314	0.286	0.164
RALBP1	NC_000018	0.40274	1	1.212	0.901	3.087
RAPGEF3	NC_000012	0.53466	1	0.338	1.095	0.745
RARA	NC_000017	2.43672	1	0.710	0.262	0.450
RARB	NC_000003	0.30539	1	0.108	0.197	0.189
RARRES1	NC_000003	0.43742	1	0.711	2.070	0.331
RELN	NC_000007	3.04665	1	0.307	0.041	0.194
RHOBTB2	NC_000008	2.22932	1	1.246	0.394	0.443
RILP	NC_000017	0.82603	1	1.051	0.430	0.310
RND3	NC_000002	22.52696	1	0.755	1.968	0.662
S1PR1	NC_000001	0.92586	1	3.892	0.300	0.098
SCN1B	NC_000019	0.77140	1	0.429	0.675	0.046
SCN2A	NC_000002	0.21557	1	1.060	0.274	0.061
SCN3B	NC_000011	0.00375	1	3.415	1.035	2.118
SDC1	NC_000002	1.55962	1	2.819	3.495	1.689
SDC2	NC_000008	8.74984	1	0.756	0.250	0.179
SECTM1	NC_000017	9.42060	1	0.996	0.755	0.344
SELE	NC_000001	0.01175	1	0.000	0.517	3.441
SELL	NC_000001	0.02807	1	0.759	3.795	0.176
SEMA3F	NC_000003	3.61416	1	0.430	2.298	1.097
SH3GLB2	NC_000009	2.93847	1	0.556	2.300	1.268
SLC9A3R1	NC_000017	11.65929	1	0.109	2.181	1.524
SPARC	NC_000005	91.12144	1	2.435	1.149	0.534
SPON1	NC_000011	0.01269	1	2.656	4.485	22.589
SPON2	NC_000004	0.48750	1	1.572	0.148	0.063
SPP1	NC_000004	4.68075	1	0.556	0.034	0.017
SSTR1	NC_000014	1.90528	1	0.850	0.130	0.978
TCF3	NC_000019	3.22128	1	1.679	2.140	1.728
TES	NC_000007	2.73049	1	0.350	0.207	0.349
TGFBR3	NC_000001	1.63577	1	1.842	0.718	0.416

TGM3	NC_000020	0.02552	1	2.277	0.690	0.176
TLR2	NC_000004	0.19407	1	0.236	1.428	0.365
TLR3	NC_000004	1.22066	1	0.300	0.749	0.231
TLR4	NC_000009	3.52494	1	0.523	0.627	0.429
TM4SF1	NC_000003	54.31340	1	0.137	3.162	1.847
TMC5	NC_000016	0.00831	1	1.708	8.797	27.795
TMEFF2	NC_000002	0.46476	1	2.677	1.594	2.103
TMEM106C	NC_000012	27.23308	1	1.117	2.443	1.449
TMSB10	NC_000002	198.05992	1	1.297	1.550	0.593
TMSB15A	NC_000023_X	0.17097	1	9.335	29.806	9.530
TNC	NC_000009	5.17857	1	3.345	2.124	1.328
TNFSF13B	NC_000013	1.72122	1	0.088	1.615	0.041
TNFSF9	NC_000019	2.01060	1	0.325	0.817	0.414
TNXB	NC_000006	1.11846	1	1.300	0.333	0.181
TPBG	NC_000006	9.19167	1	2.252	0.992	0.370
TPM1	NC_000015	16.85210	1	2.022	0.192	0.286
TPM3	NC_000001	0.37687	1	1.624	1.768	2.103
TRAP1	NC_000016	2.40030	1	1.369	1.779	2.284
TRIO	NC_000005	1.52311	1	1.847	2.330	2.916
TRO	NC_000023_X	0.62795	1	1.165	0.089	0.021
TRPC6	NC_000011	0.11357	1	0.346	0.225	0.069
TRPV1	NC_000017	0.05060	1	1.449	1.976	0.578
TUBA1A	NC_000012	33.03878	1	2.149	1.110	0.987
TUBA3C	NC_000013	0.03059	1	0.000	1.035	0.265
TUBA3D	NC_000002	0.01473	1	1.138	2.070	6.353
TUBA4A	NC_000002	10.26177	1	0.908	1.453	2.305
TUBB1	NC_000020	0.12377	1	0.359	1.198	0.836
TUBB2A	NC_000006	2.03708	1	1.468	0.564	0.194
TUBB4	NC_000019	0.29873	1	0.911	0.517	0.141
TUBB6	NC_000018	49.44839	1	1.969	0.932	0.771
VCAM1	NC_000001	0	1	0.058107	0.05763	0
VCAN	NC_000005	7.14906	1	3.245	0.187	0.148
VWA5A	NC_000011	0.66237	1	0.502	5.113	2.818
WISP2	NC_000020	13.73848	1	0.059	0.530	0.289

(k) Angiogenesis

ADAM17	NC_000002	4.01653	1	1.082	2.259	1.250
ADAM9	NC_000008	5.59466	1	1.359	2.415	1.842
ADAMTS1	NC_000021	22.48912	1	0.473	0.397	0.373
ADAMTS10	NC_000019	0.22037	1	2.249	0.858	0.439
ADAMTS3	NC_000004	0.49685	1	0.350	0.685	0.367
AGT	NC_000001	0.33490	1	1.468	1.035	0.418
AGTR1	NC_000003	0.32773	1	1.938	1.678	0.486
AMOT	NC_000023_X	0.00617	1	18.783	0.517	0.794
ANGPT1	NC_000008	2.25211	1	0.448	0.198	0.188
ANGPTL2	NC_000009	3.46825	1	1.013	2.142	0.578
ANGPTL4	NC_000019	3.19518	1	1.757	0.101	0.315
CD36	NC_000007	0.16779	1	2.106	0.310	0.066
CLEC3B	NC_000003	13.39123	1	0.089	0.019	0.024
COL18A1	NC_000021	0.67773	1	2.685	2.544	0.685
EDN1	NC_000006	0.74826	1	1.435	0.240	0.077
EFNB2	NC_000013	1.01167	1	0.006	0.442	0.121
EREG	NC_000004	1.38764	1	0.093	10.203	0.796
F10	NC_000013	0.04448	1	3.795	0.345	1.235
F12	NC_000005	0.01110	1	5.692	7.245	3.177
F2RL1	NC_000005	0.00790	1	202.630	1.035	4.765
F3	NC_000001	14.15207	1	1.874	0.144	0.240
FBLN1	NC_000022	5.47642	1	1.729	0.139	0.038
FBN1	NC_000015	5.91756	1	2.872	0.407	0.753
FBN2	NC_000005	0.66680	1	2.005	5.079	4.197
FGF1	NC_000005	0.98924	1	0.354	0.869	0.645
FGF2	NC_000004	7.95748	1	1.568	1.279	0.317
FIGF	NC_000023_X	0.03236	1	3.795	0.345	0.353
FLT1	NC_000013	0.00437	1	54.642	1.035	2.647
FLT4	NC_000005	0.01426	1	3.131	0.776	9.000
JAG1	NC_000020	1.50249	1	0.320	0.335	0.172
KDR	NC_000004	0.05265	1	1.382	2.957	2.950
KLKB1	NC_000004	0.03040	1	2.656	0.690	0.529
LAMA5	NC_000020	0.57999	1	1.381	1.124	0.282
MDK	NC_000011	23.41728	1	0.617	1.598	0.296
NRP1	NC_000010	17.56045	1	1.291	2.029	0.786
NRP2	NC_000002	0.61071	1	1.661	5.895	1.586
PDGFB	NC_000022	0.02654	1	2.277	2.070	6.221
PDGFC	NC_000004	11.45093	1	0.995	0.678	0.374
PLAT	NC_000008	5.40440	1	1.667	0.344	0.132
PLGLB1	NC_000002	0.01513	1	1.138	1.035	0.794
PLGLB2	NC_000002	0.04530	1	0.379	1.207	2.294

PTGES	NC_000009	1.61846	1	2.653	0.554	0.079
PTGIS	NC_000020	5.09276	1	2.137	0.025	0.006
PTGR1	NC_000009	2.69537	1	1.293	2.274	1.163
PTGS1	NC_000009	0.07154	1	21.843	0.259	0.232
PTGS2	NC_000001	3.44156	1	2.188	0.264	0.031
RAMP2	NC_000017	0.31911	1	8.072	2.823	1.155
SERPINB2	NC_000018	0.43107	1	3.693	0.480	0.077
SERPINB9	NC_000006	0.13735	1	0.273	0.952	0.064
SERPINF1	NC_000017	4.62256	1	0.204	0.354	0.019
SPHK1	NC_000017	8.11678	1	1.964	0.412	0.608
STOM	NC_000009	23.62506	1	0.560	0.507	0.425
TBXAS1	NC_000007	0.07843	1	0.285	1.811	1.654
TEK	NC_000009	0.80393	1	3.950	0.123	0.041
TFPI	NC_000002	3.77169	1	0.171	0.750	0.519
TFPI2	NC_000007	0.75910	1	7.151	1.327	0.312
TGFB1	NC_000019	2.20354	1	2.272	0.301	0.452
TGFB2	NC_000001	0.11302	1	0.919	0.637	0.367
THBD	NC_000020	1.71137	1	0.639	0.130	0.290
THBS1	NC_000015	87.84364	1	2.297	0.009	0.019
THBS2	NC_000006	6.62533	1	0.468	0.042	0.015
THBS3	NC_000001	6.04244	1	0.898	1.246	0.308
THBS4	NC_000005	0.12897	1	1.078	0.654	0.362
TYMP	NC_000022	4.15812	1	0.374	2.009	0.207
VEGFC	NC_000004	16.73977	1	0.438	0.513	0.248
WARS	NC_000014	26.35635	1	0.661	0.302	0.108
**(I) Invasion and Metastasis**						
ADAMTS15	NC_000011	0.43104	1	4.026	0.230	0.441
BAT2D1	NC_000001	1.16234	1	1.529	3.085	2.219
CALB2	NC_000016	0.03122	1	8.538	2.070	1.059
CD82	NC_000011	5.92234	1	0.370	2.831	0.884
CRMP1	NC_000004	0.09740	1	2.656	5.451	11.541
CTSB	NC_000008	172.06540	1	0.419	1.492	0.568
CTSC	NC_000011	0.25991	1	4.606	3.057	3.949
CTSD	NC_000011	99.35307	1	0.325	2.010	0.862
CTSF	NC_000011	3.91236	1	0.967	5.668	1.894
CTSH	NC_000015	1.15016	1	1.563	15.359	3.918
CTSK	NC_000001	47.19199	1	0.874	0.793	0.142
CTSL1	NC_000009	73.47450	1	0.739	1.030	0.413
CTSL2	NC_000009	0.12178	1	0.142	2.199	0.529
CTSZ	NC_000020	16.04745	1	1.457	1.949	0.966
DFFB	NC_000001	0.12785	1	1.808	2.070	1.806
DNAJB4	NC_000001	8.94658	1	0.459	0.166	0.165
HTATIP2	NC_000011	1.40675	1	0.718	2.925	1.926
LGR4	NC_000011	2.00518	1	3.859	1.759	1.321
LIMD2	NC_000017	1.43347	1	1.015	0.780	0.381
LOX	NC_000005	65.58493	1	1.252	0.360	0.256
MASP2	NC_000001	0.13138	1	1.821	2.070	1.482
MFAP3L	NC_000004	0.14814	1	8.246	3.181	2.014
MFAP5	NC_000012	5.47598	1	1.812	0.010	0.012
MMP1	NC_000011	14.98201	1	2.119	0.000	0.001
MMP11	NC_000022	0.12087	1	2.087	3.795	0.794
MMP11	NC_000022	0.12087	1	2.087	3.795	0.794
MMP14	NC_000014	94.38804	1	1.037	0.436	0.207
MMP17	NC_000012	0.19860	1	2.385	0.148	0.076
MMP2	NC_000016	61.86606	1	0.950	0.875	0.293
MMP3	NC_000011	0.53569	1	19.855	0.193	0.456
MMP9	NC_000020	0.00975	1	3.415	3.105	1.059
MT1E	NC_000016	41.63069	1	1.035	0.936	0.328
MTA1	NC_000014	4.51460	1	1.465	0.338	0.299
MTA2	NC_000011	6.87659	1	1.570	1.947	1.528
MTSS1	NC_000008	1.17275	1	0.278	0.622	0.238
MYO1A	NC_000012	0.02521	1	1.992	0.259	0.265
NME1	NC_000017	3.77711	1	1.951	0.932	0.814
NME2	NC_000017	18.98435	1	2.014	1.204	0.783
NUAK1	NC_000012	1.00160	1	2.603	1.611	0.960
PAPPA	NC_000009	1.50091	1	7.314	1.117	1.657
PI16	NC_000006	0.04159	1	179.009	0.517	0.529
PLAUR	NC_000019	5.47420	1	1.721	0.760	0.218
PLXNB1	NC_000003	0.59457	1	0.575	0.787	0.312
PLXNC1	NC_000012	0.15568	1	0.130	0.562	0.151
PRSS23	NC_000011	16.41866	1	1.152	0.471	0.462
PRUNE2	NC_000009	1.66659	1	0.276	0.932	0.947
RAB27B	NC_000018	20.70283	1	0.167	1.107	0.630
RECK	NC_000009	3.06601	1	1.184	0.416	0.368
RGS4	NC_000001	15.98927	1	0.463	0.596	1.105
RNF5	NC_000006	6.32920	1	1.326	3.432	1.970

S100A11	NC_000001	77.40557	1	1.181	2.088	0.702
S100A4	NC_000001	88.66977	1	0.281	1.465	0.589
SEMA3C	NC_000007	4.27381	1	0.419	1.197	0.471
SERPINB1	NC_000006	3.29356	1	0.907	2.015	0.968
SERPINB5	NC_000018	0.00865	1	3.415	0.000	0.529
SERPING1	NC_000011	10.71473	1	0.328	0.965	0.120
SERPINH1	NC_000011	39.26398	1	2.228	1.484	0.451
SLIT2	NC_000004	1.23757	1	4.727	1.343	0.964
SPIRE2	NC_000016	0.30186	1	1.350	2.070	1.465
ST5	NC_000011	0.60986	1	1.184	1.823	2.021
STOML2	NC_000009	10.05380	1	2.084	2.438	1.164
TIMP1	NC_000023_X	93.62831	1	1.352	0.769	0.252
TIMP3	NC_000022	30.46101	1	0.621	0.024	0.007
TIMP4	NC_000003	0.38247	1	0.237	0.517	0.375
TWIST1	NC_000007	7.66850	1	0.377	1.496	0.373
TWIST2	NC_000002	0.37613	1	5.278	0.282	0.241
WASH1	NC_000019	0.07772	1	3.415	0.000	2.118
