# Supplementary material for: Transcriptional Dynamics of Immortalized Human Mesenchymal Stem Cells during Transformation
Source: PLoS One. 2015 May 15;10(5):e0126562. doi: 10.1371/journal.pone.0126562 (PMC4433180; doi:10.1371/journal.pone.0126562)
Supplement: S3 Table — Of the 1,732 genes listed in S1 Table, gene symbols and function of 180 genes (Fig 3B) are shown, in general, according to the NCBI gene database. (PDF) [file pone.0126562.s006.pdf]

Supportig Table S3. List of genes shown in Fig. 3B

Gene Symbol	Function
(a) Oncogenes	
ATAD2	cofactor for MYC
BMI1	polycomb group ring finger protein
MYBL2	activate cyclin D1
MYC	transcription factor
PIK3CA	PI3-kinase catalytic subunit, suppress Ras-senescence
RHEB	Ras homolog, small GTPase protein
RHEBL1	Ras homolog
RRAS2	Ras-like small GTPase
STAC	Src homology 3
(b) Suppressor	
DAB2IP	Ras GTPase-activating protein (GAP)
DBC1	inhibit SIRT1 function
HIC1	hypermethylated in cancer
PYHIN1	pyrin and HIN domain family
RAP1GAP	GTPase activator for Ras-related protein (RAP1A)
RASA4	GAP1 family of GTPase-activating protein
RASSF2	Ras association domain-containing protein
RB1CC1	enhancse RB1 gene expression
SFN	stratifin, stabilize p53
TP63	p53 family
(c) DNA synthesis and repair	
BRCA1	DNA excision repair protein
ERCC5	DNA excision repair protein ERCC-5
MDC1	mediator of DNA-damage checkpoint
PRKDC	DNA-dependent protein kinase catalytic subunit
RAD9A	DNA repair exonuclease, sensor of damaged DNA
RBBP5	retinoblastoma protein binding protein
TERT	telomerase catalytic subunit
TREX1	3'->5' DNA exonuclease
XRCC3	maintain chromosome stability and repair DNA damage in HR
XRCC5	double-strand-break rejining
(d) Chromosome instability	
CENPB	centromere-associated protein
CETN3	centrin
ESPL1	separase, sister chromatid separation
FRY	depletion of Fry causes mitotic chromosome misalignment
MAD2L1	mitotic spindle assembly check point
MARCKSL1	spindle formation
PTTG1	securine
RAD21	involved in DNA double-strand break repair
RFC4	elongation of primed DNA templates by DNA polymerase delta
STAG2	a component of cohesin
(e) Cell Cycle	
CCNB1	G2/mitotic-specific cyclin B1
CCND2	G1/S specific cyclin D2
CCNE2	G1/S specific cyclin E
CDK2	cyclin-dependent protein kinase for late G1 phase
CDK4	cyclin-dependent protein kinase for early G1 phase
CDKN1C	G1 cyclin-dependent kinase inhibitor, p57
CDKN2AIPNL	CDKN2A(p16)-interacting protein
CDKN2B	cyclin D-dependent kinase (CDK4 or CDK6) inhibitor, p14 or p15
CIZ1	inhibitor of CDKN1A(p21)
E2F1	transcription activation inDNA binding, bind to pRB
(f)Apoptosis	
BCL2	suppress apoptosis
BCL2L11	BCL2-like 11, anti-apoptotic regulator
BCL2L12	BCL2-like 12
CASP10	apoptosis-related cystein protease
HRK	harakiri, BCL2 interacting protein
SOX4	trancription factor, leading to cell death
SYVN1	synovial apoptosis inhibitor 1
TNFRSF11B	Fas-like protein
TNFRSF6B	tumor necrosis factor receptor, suppress cell death
XAF1	inhibitory effect of inhibitor of apoptosis

(g) Signal transduction, TF. And Transcription regulation

APC	antagonist of the Wnt signaling pathway
CTNNB1	beta-catenin, cadherin-asociated protein
DHH	desert hedgehog, activates stromal hedgehog effectors
DKK3	antagonist of canonical Wnt signaling pathway
FZD1	Wnt protein receptor; frizzled 1
GLI1	transcriptional activator
HHIP	hedgehog interacting protein, negative regulators
PTCH1	receptor for hedghog protein (Dhh, Shh, Ihh)
SMO	smoothened, frizzled family receptor
WNT5A	ligand for frizzled-5 receptor,

(h)Growth-related factors

CSF2	colony stimulating factor 2
EIF4B	translation initiation factor 4B
F2R	coagulation factor II (thrombin) receptor
FGF14	fibroblast growth factor 14
IGF1R	insulin-like growth factor 1 receptor (overexpressed in some tumour)
IGF2BP2	binding protein to insulin-like growth factor 2 mRNA
NCBP2	nuclear cap binding protein
PDGFB	platelet-derived growth factor beta
RAI2	retinoic acid induced protein 2
TFRC	transferrin receptor (CD71)

(i) Markers

ACTA2	actin-alpha 2, human aorta smooth muscle actin
CD44	mesenchymal cell-specific glycoprotein
GPC5	glypican 5
HMGA2	transcritional regulation in adipogenesis and mesenchymal differentiation
LMOD1	smooth muscle leiomodin 1
MSX2	malignant melanoma
POSTN	periostin, osteoblast specific factor
TAGLN	transgelin, smooth muscle protein 22, actin cross-linking/gelling protein
THY1	CD90, marker for mesenchymal stroma cel antigenl
TPD52	tumor protein D52, prostate and colon associated protein

(j) Adhesion, Cell Menbrane and Cytoskeleton

CDH1	E-cadhelin, expression in epitherial cells
CDH2	N-cadherin, expression in neuronal cells
COL15A1	collagen, type XV, alpha 1
COL4A5	collagen, type IV, alpha 5
ELN	elastin
FN1	fibronectin
ICAM1	intercellular adhesion molecule 1, induced by IL-1 and TNF-alpha
ITGA6	integrin, alpha 6
MCAM	melanoma cell adhesion molecule
VCAN	a major component of extracellar matrix

(k)Angiogenesis

ANGPT1	angiopoietin 1, important roles in vascular development
F2RL1	thrombin receptor-like 1
FBN2	fibrillin
FGF2	fibroblast growth factor 2 (basic)
FLT1	vascular endothelial growth factor receptor, important role in angiogenesis and vasculogenesis
KDR	type III receptor tyrosine kinase, receptors of the VEGF
NRP1	neuropilin 1, tyrosine kinase receptor for VEGF and semaphorin
PDGFB	platelet-derived growth factor beta
THBS1	thrombospondin-1, adhesive glycoprotein mediating cell-to-cell or matrix interaction
VEGFC	vascular endothelial growth factor C

(l) Invasion and Metastasis

CTSF	cathepsin F, lysosomal protease, implication in tulour invasion and metastasis
CTSH	cathepsin H, degradation of lysosomal protein
MMP1	matrix metallopeptidase1, collagenase
MMP14	matrix metallopeptidase 14
MMP2	matrix metallopeptidase 2, gelatinase A
MMP3	progelatinase
MTA1	metastasis associated 1, expressed in metastatic cells
MTSS1	metastasis suppressor 1
TIMP1	TIMP metallopeptidase 1
TIMP4	TIMP metallopeptidase 4
